# Supplementary figures and images for: Periodic fasting and refeeding re-shapes lipid saturation, storage, and distribution in brown adipose tissue
Source: PLoS Biol. 2026 Jan 12;24(1):e3003593. doi: 10.1371/journal.pbio.3003593 (PMC12795461; doi:10.1371/journal.pbio.3003593)

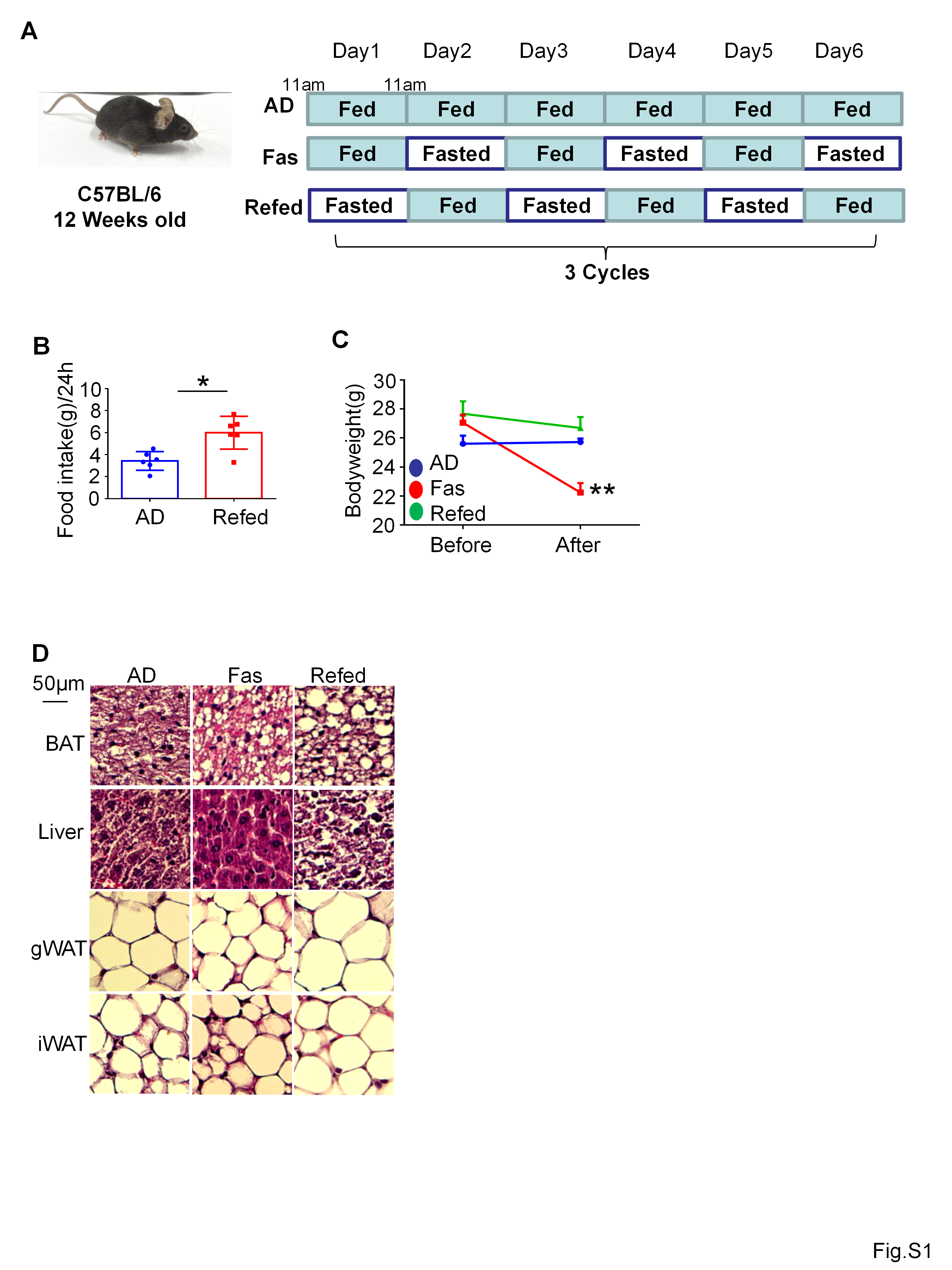

Supplement: S1 Fig — A. Schematic diagram of Alternate Day Fasting (ADF). AD, ad libitum; Fas, ADF ended with fasting; Refed, ADF ended with refeeding. B. The food intake was notably decreased upon refeeding following with 24-h fasting. C. Body weight significantly decreased after fasting and was regained after refeeding. D. The representative images of H&E staining for gWAT, iWAT and BAT and Liver. The raw data for S1B and S1C are presented in S1 Table. Data in S1B and S1C Fig are presented as mean ±SEM. T Test was used to analyze the data. *P < 0.05, **P < 0.01. (TIF) [file pbio.3003593.s001.tif]

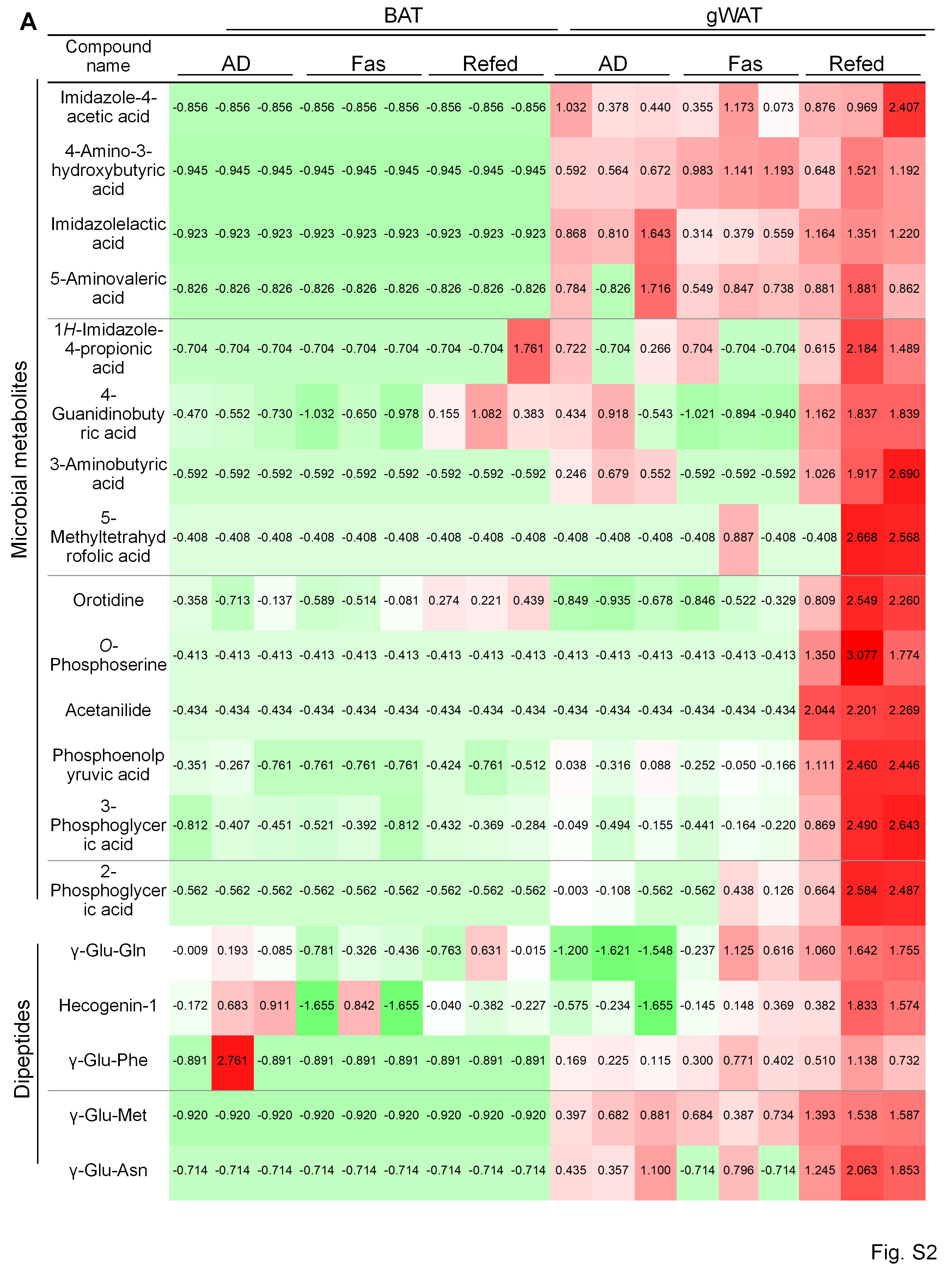

Supplement: S2 Fig — The basal levels of microbial metabolites (A) and dipeptides (B) were significantly higher in gWAT compared to BAT and were further increased by refeeding compared to the AD and Fas groups. The raw data of metabolomics and lipidomics for S2A and S2B are presented in S2 Table. (TIF) [file pbio.3003593.s002.tif]

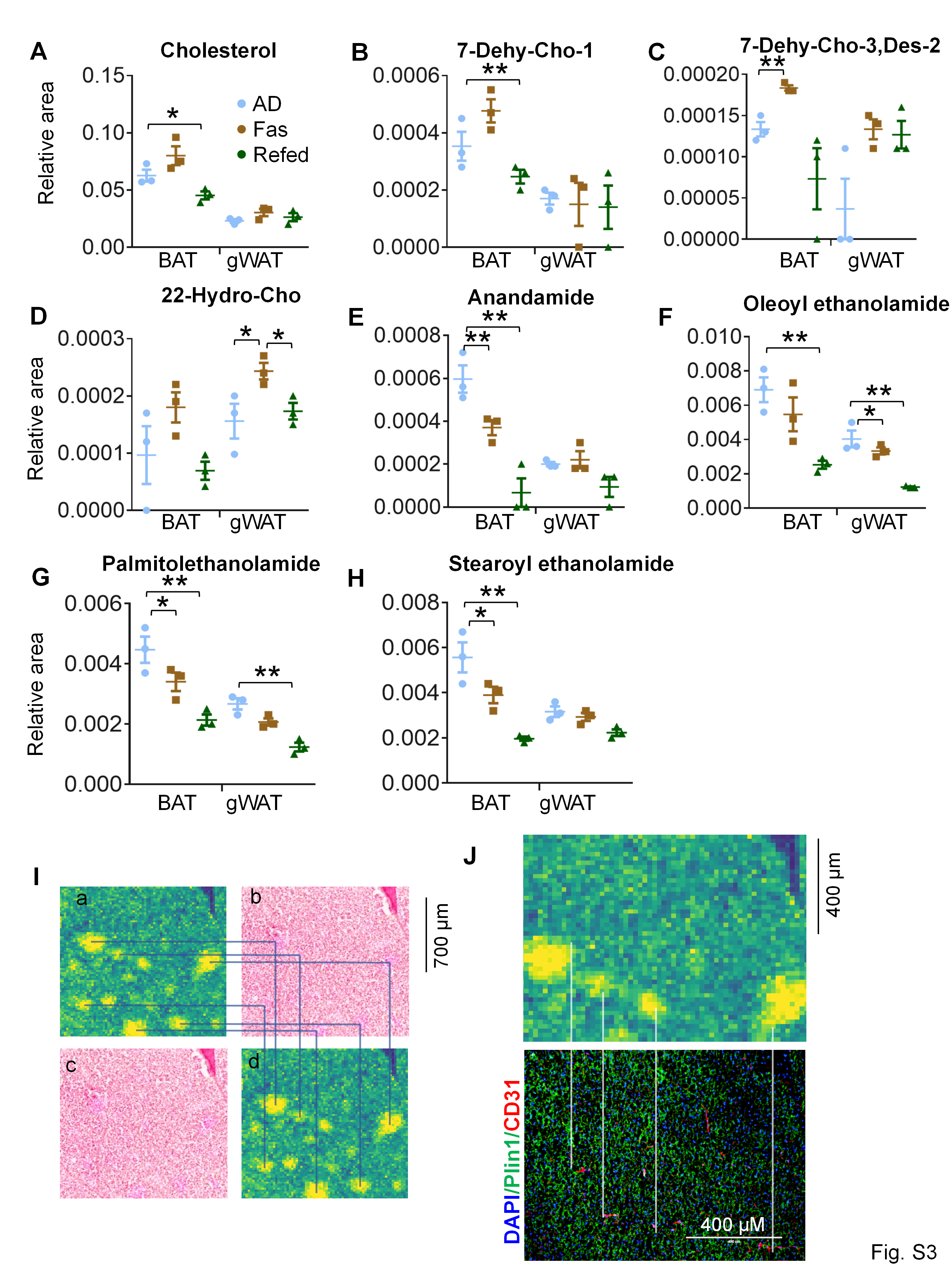

Supplement: S3 Fig — BAT displayed higher basal levels of cholesterol (A), 7-dehydrocholesterol-1 (B), and 7-dehydrocholesterol-3 (C), but not 22-hydroxycholesterol (D) compared with gWAT. Moreover, the levels of bioactive lipids including anandamide (E), oleoyl ethanolamide (F), palmitolethanolamide (G), and stearoyl ethanolamide (H) were higher in BAT than those in gWAT under both AD and fasting conditions. I. Alignment of representative lipid (LPC16:0) images (A, D) with H&E staining (B, C) in a representative BAT section. J. The enriched LPC16:0 “sparkles” were correlated with CD31 expression.The raw data of metabolomics and lipidomics for S3A–S3J are presented in S2 Table. S3A–S3H Fig was analyzed by T Test. The data are presented as mean ± SEM. *P < 0.05, **P < 0.01. (TIF) [file pbio.3003593.s003.tif]

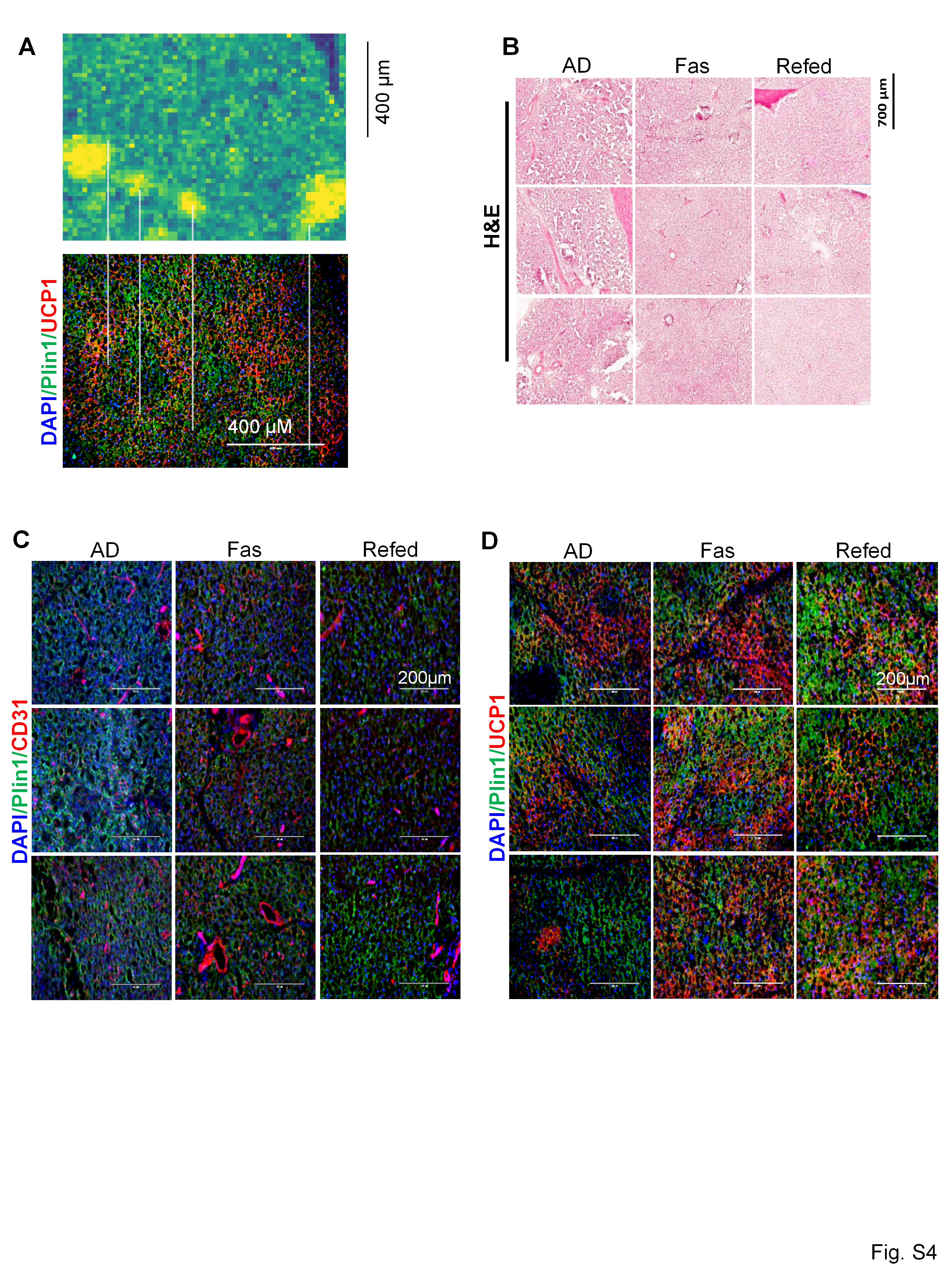

Supplement: S4 Fig — A. The enriched LPC16:0 “sparkles” were not correlated with UCP1-high adipocytes. B. Representative H&E staining images of all BAT tissue samples. C. Representative CD31 staining images of all BAT tissue samples. D. Representative UCP1 staining images of all BAT tissue samples. (TIF)s [file pbio.3003593.s004.tif]

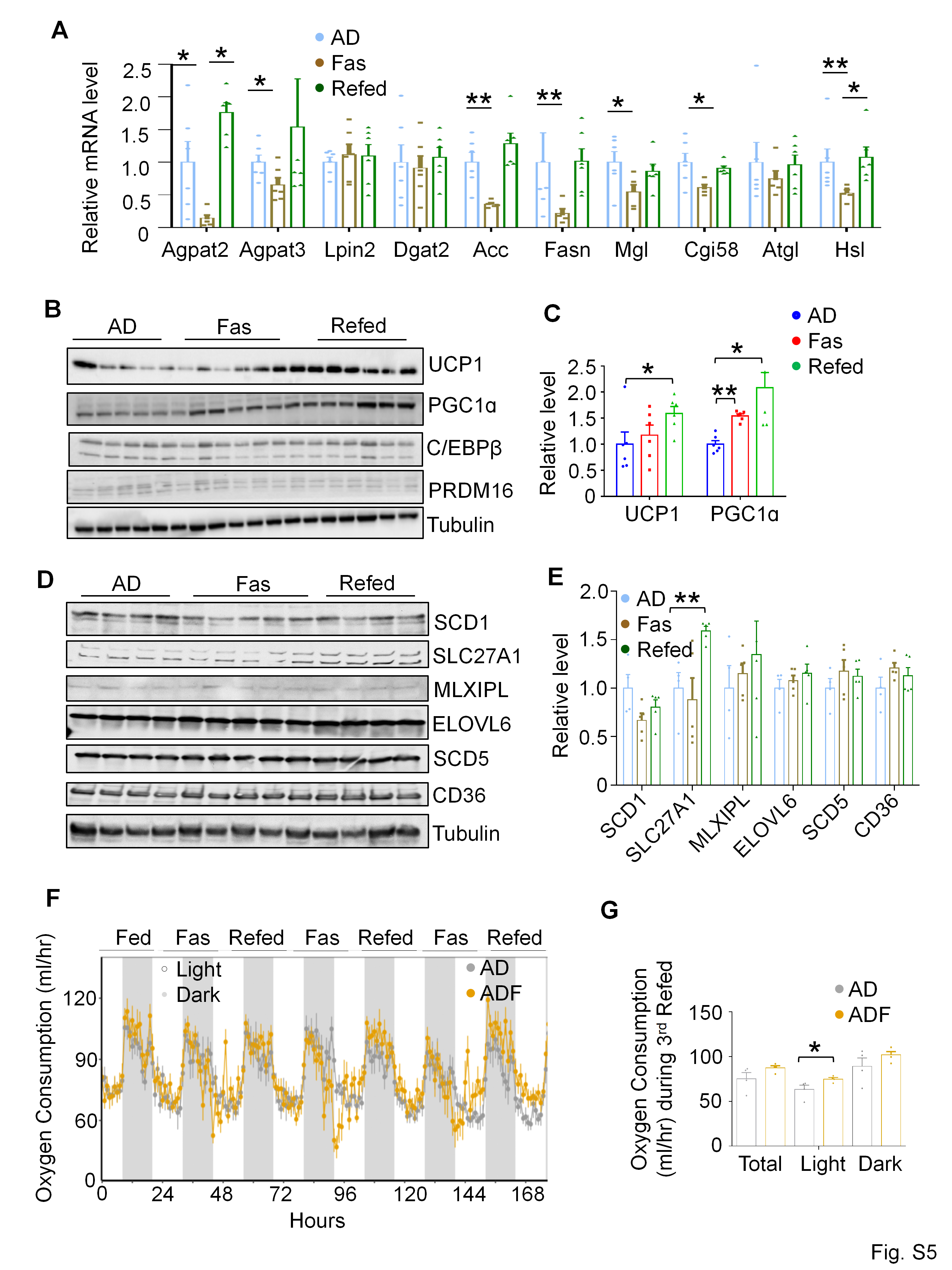

Supplement: S5 Fig — The mRNA expression of genes encoding the enzymes for TG synthesis, De novo lipogenesis, and Lipolysis pathway during periodic fasting and refeeding. B, C. The protein level of thermogenesis markers in BAT during periodic fasting and refeeding. D. The western blot of fatty acids desaturases, elongase, and transport genes. E. Quantification of protein levels from the Western blots shown in S4D Fig. F, G. Oxygen consumption during ADF for 3 cycles, using the CaIR website as described in the methods. The raw data for S5A, S5C, S5E, and S5G are presented in S1 Table. The uncropped western blot images for panel S5B and S5D are provided in S3 Raw Images. Data are presented as the mean ± SEM. *P < 0.05; **P < 0.01. (TIF) [file pbio.3003593.s005.tif]

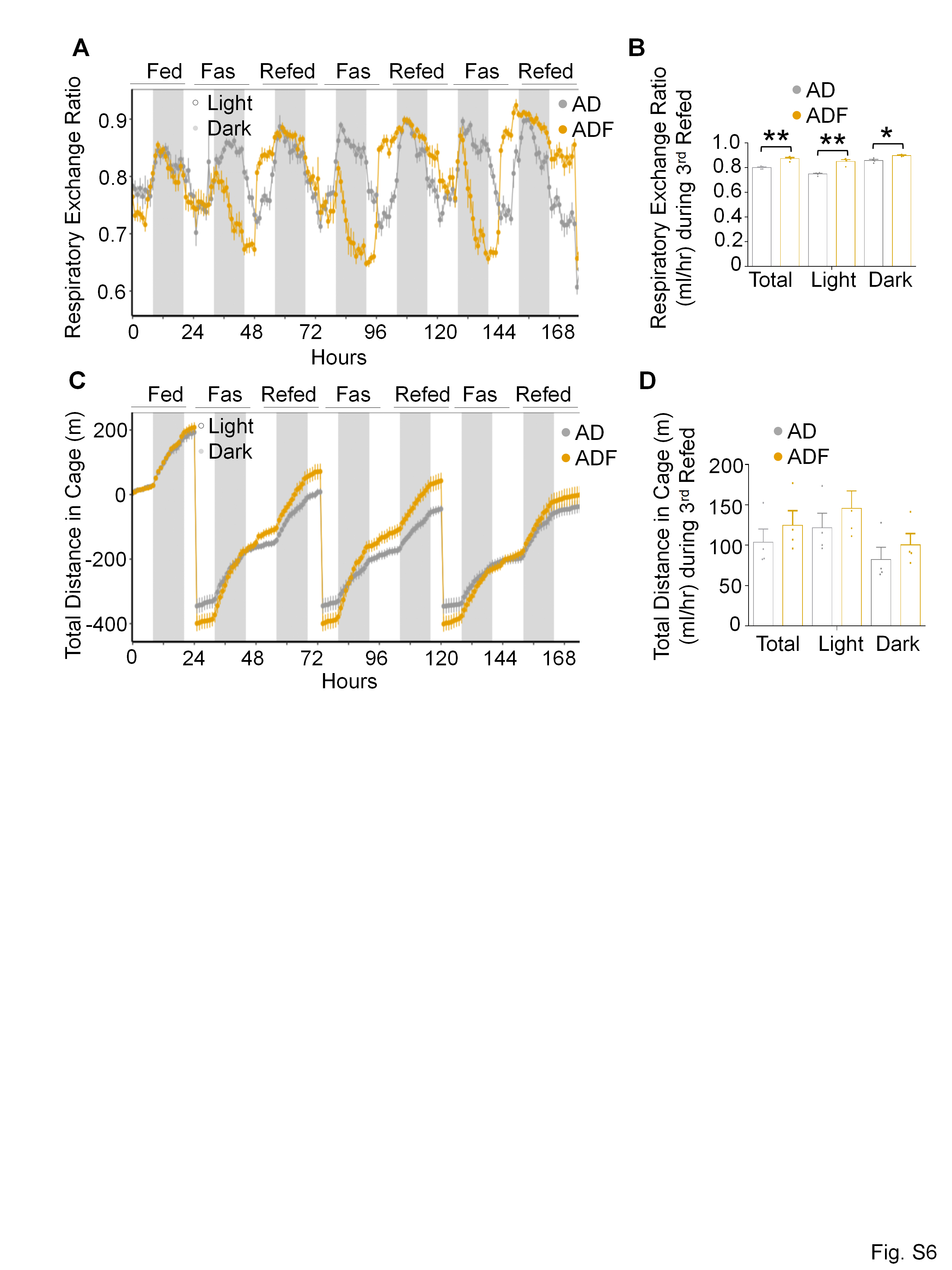

Supplement: S6 Fig — A, B. ADF mice exhibited significantly lower Respiratory Exchange Ratio (RER) during fasting phase but gradually higher RER during refeeding than ad libitum controls. C, D. Refed mice showed a slight increase in motor activities. The raw data for S6B and S6D are presented in S1 Table. Data are presented as the mean ± SEM. *P < 0.05; **P < 0.01. (TIF) [file pbio.3003593.s006.tif]

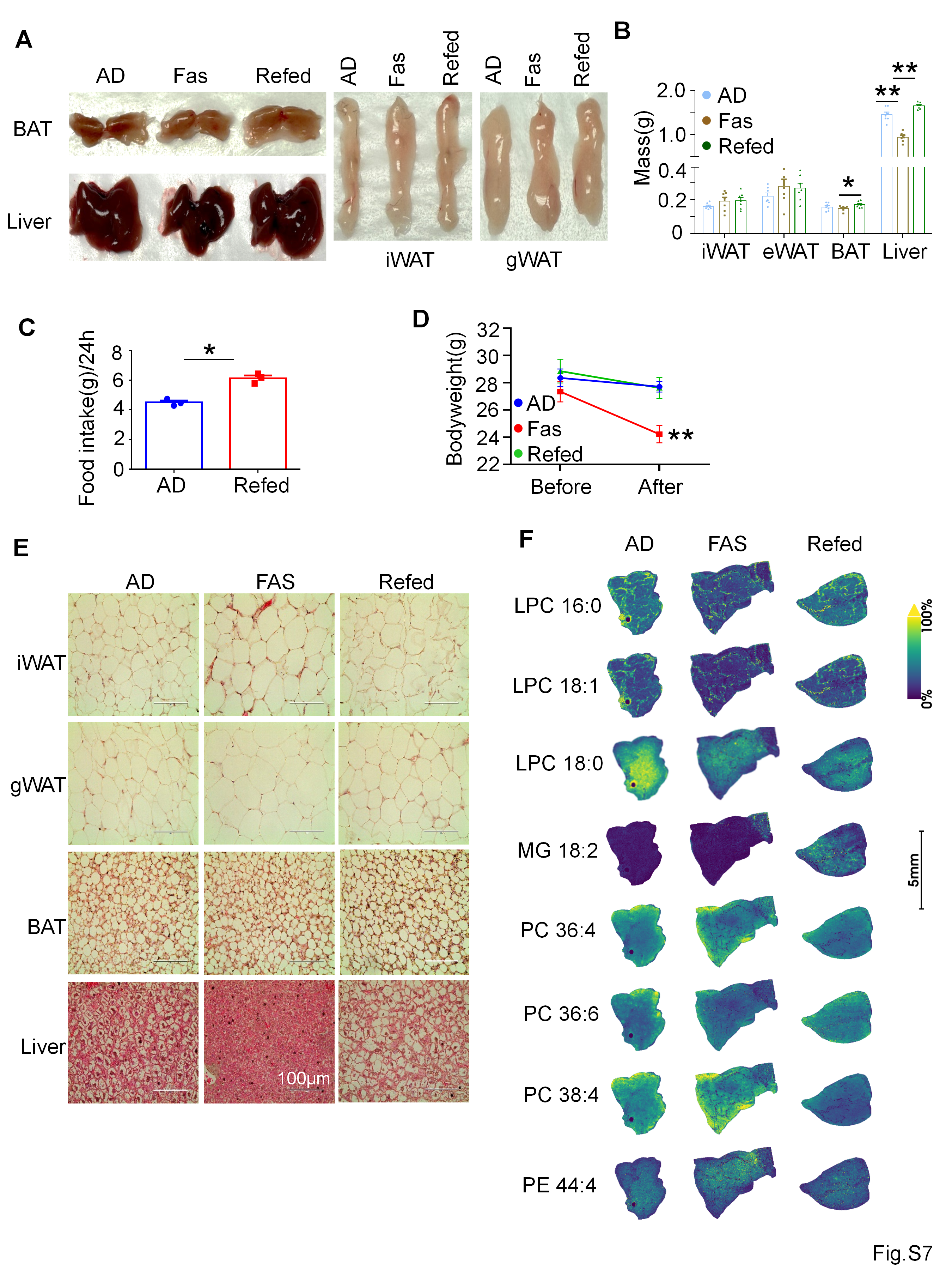

Supplement: S7 Fig — A. The Representative images of gWAT, iWAT, BAT, and liver in three groups ad libitum (AD), fasting of ADF (Fas), and refeeding of ADF (Fed) under thermoneutral conditions (30 ℃). B. The mass of various fat tissues and liver after periodic fasting and refeeding. The food intake (C) and body weight (D) during alternative-day fasting under thermoneutral conditions. E. H&E staining of fat depots. F. The MSI showed a slight decrease for PC 36:4, PC 36:6, PC 38:4, and PE 44:4 in BAT upon refeeding. The raw data for S5B–S5D are provided in S1 Table, and the raw data of MALDI for S7F is presented in S2 Raw Images. Data are presented as the mean ± SEM. *P < 0.05; **P < 0.01. (TIF) [file pbio.3003593.s007.tif]

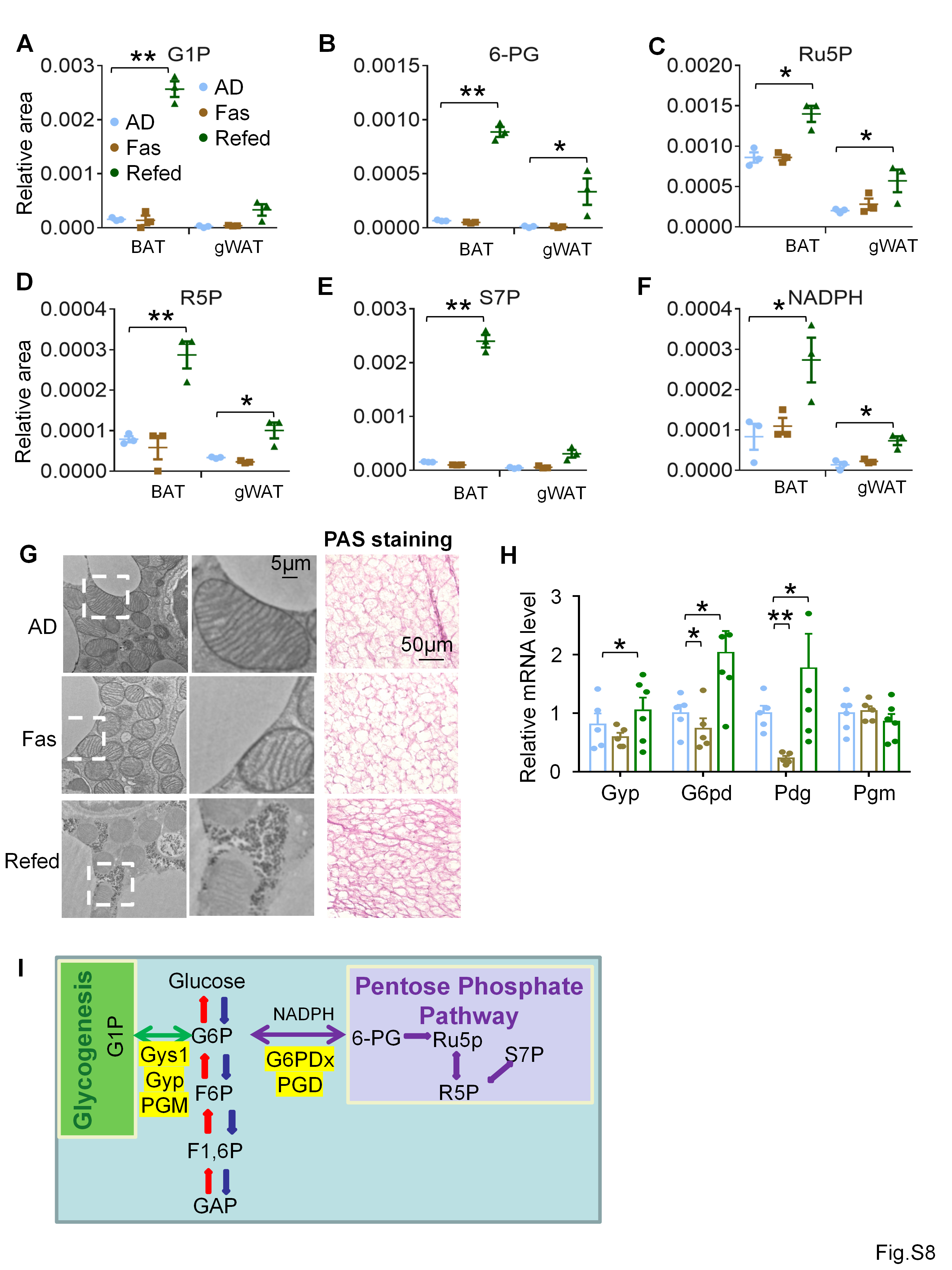

Supplement: S8 Fig — The alterations of intermediate metabolites within glycogenesis and PPP pathways in BAT and gWAT during periodic fasting and refeeding; refeeding activated glycogenesis and PPP pathways in BAT as indicated by Glucose 1-Phosphate (G1P) (A), 6-Phosphogluconic acid (6-PG) (B), Ribulose 5-Phosphate (Ru5P) (C), Ribose 5-Phosphate (R5P) (D), Sedoheptulose 7-Phosphate (S7P) (E), and Nicotinamide Adenine Dinucleotide Phosphate (NADPH) (F), albert to a lesser extent in gWAT. G. Feast BAT exhibited significant glycogen accumulation indicated by TEM and PAS staining. H. The mRNA levels of the enzymes involved in glycogenesis and PPP, including Glycogen Phosphorylase (GYP), Glucose-6-Phosphate Dehydrogenase (G6PD), and 6-Phosphogluconate Dehydrogenase (PGD) but not Phosphoglucomutase (PGM), were induced by refeeding in BAT. I. The summary of glyconeogenesis and PPP pathways. The raw data for S8H is presented in S1 Table, and the raw data for metabolomics and lipidomic (S8A–S8F) are provided in S2 Table. All data in this Figure were analyzed by T Test and are presented as mean ± SEM. *P < 0.05, **P < 0.01. (TIF) [file pbio.3003593.s008.tif]

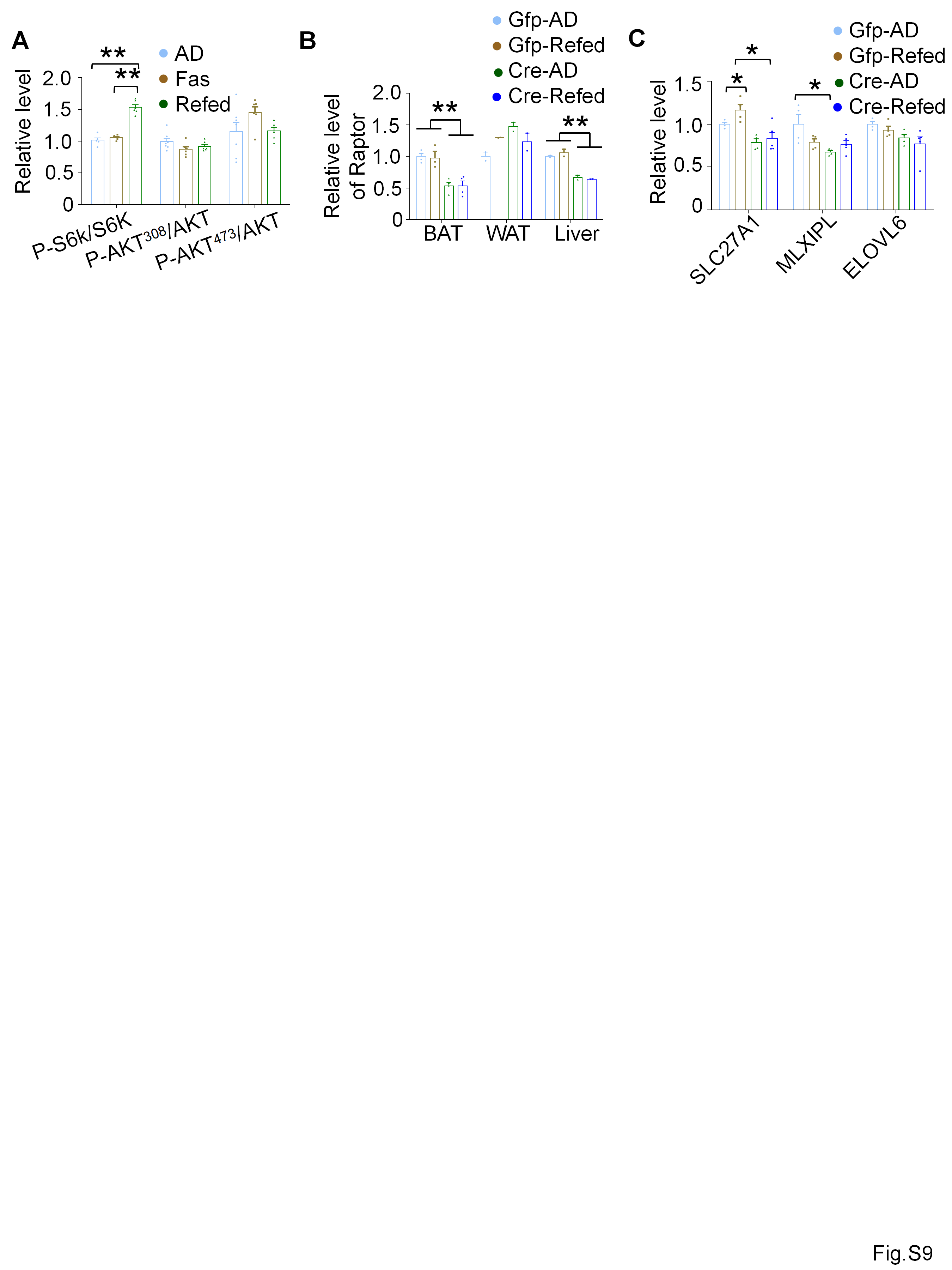

Supplement: S9 Fig — A. Quantification of phosphorylation protein levels from the Western blots shown in Fig 7A. B. Quantification of Raptor protein levels from the Western blots shown in Fig 7B. C. Quantification of protein levels from the Western blots shown in Fig 7G. The raw data for S9A–S9C are presented S1 Table. All data in this Figure were analyzed by T Test and are presented as mean ± SEM. *P < 0.05, **P < 0.01. (TIF) [file pbio.3003593.s009.tif]

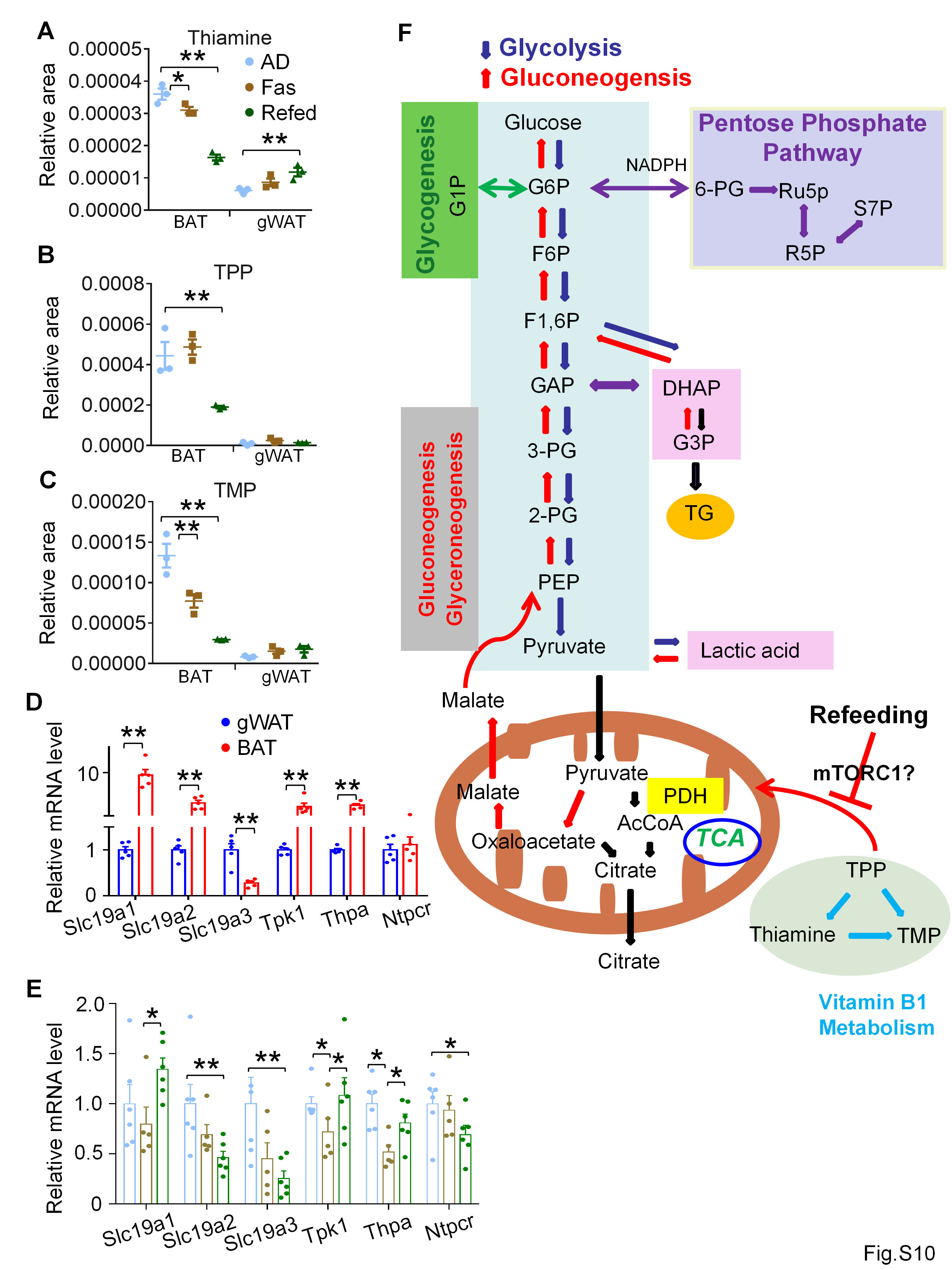

Supplement: S10 Fig — The intermediate metabolites within thiamine metabolism were altered during periodic fasting and refeeding. Refeeding notably reduced thiamine metabolites including thiamine (A), thiamine phosphate (B), and thiamine diphosphate (C) in BAT despite a slight increase in thiamine of gWAT. D. The mRNA levels of thiamine transporters and enzymes were highly expressed in BAT compared to gWAT. E. The mRNA levels of thiamine transporters and enzymes involved in thiamine metabolism in BAT were differentially altered during periodic fasting and refeeding. F. The summary of the glycolysis and thiamine pathways. The levels of Acetyl-CoA (AcCoA). The raw data for S10D and S10E are presented in S1 Table, and all metabolomics and lipidomic data for S10A–S10C are provided in S2 Table. Data in S10D and S10E Fig are presented as the mean ± SEM. *P < 0.05; **P < 0.01. (TIF) [file pbio.3003593.s010.tif]

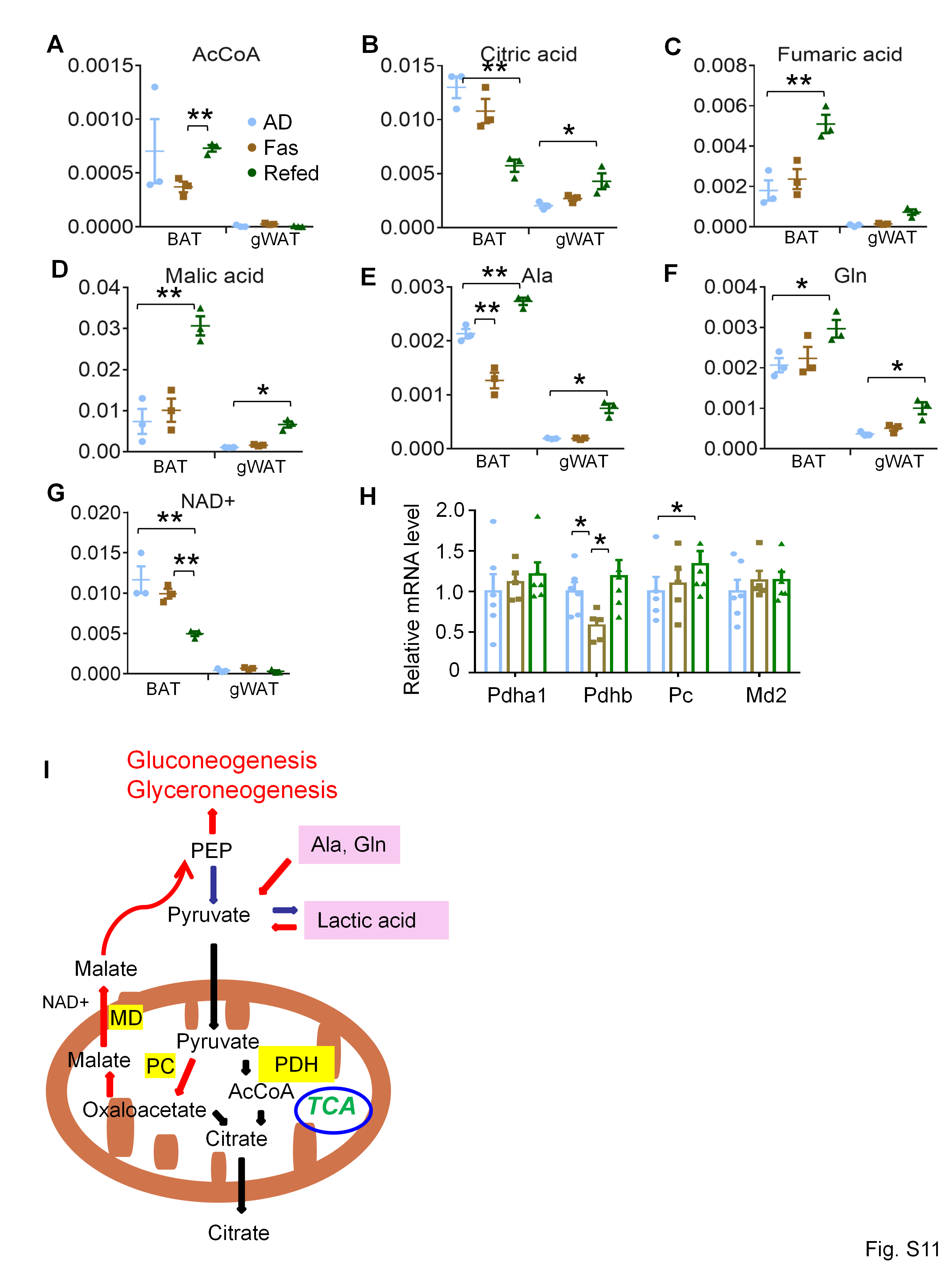

Supplement: S11 Fig — (A), Citric acid (B), Fumaric acid (C), Malic acid (D), Alanine (Ala) (E), Glutamine (Gln) (F), and NAD+ (G) in BAT. H. Expression levels of mRNA of glyceroneogenesis pathway enzymes increased by refeeding of ADF in BAT. I. The summary of pyruvate destinations. The raw data for S11H is presented in S1 Table, and all metabolomics and lipidomic data for S11A–S11G are provided in S2 Table. Data in S11A–S11H are presented as the mean ± SEM. *P < 0.05; **P < 0.01. (TIF) [file pbio.3003593.s011.tif]

Fig 7A

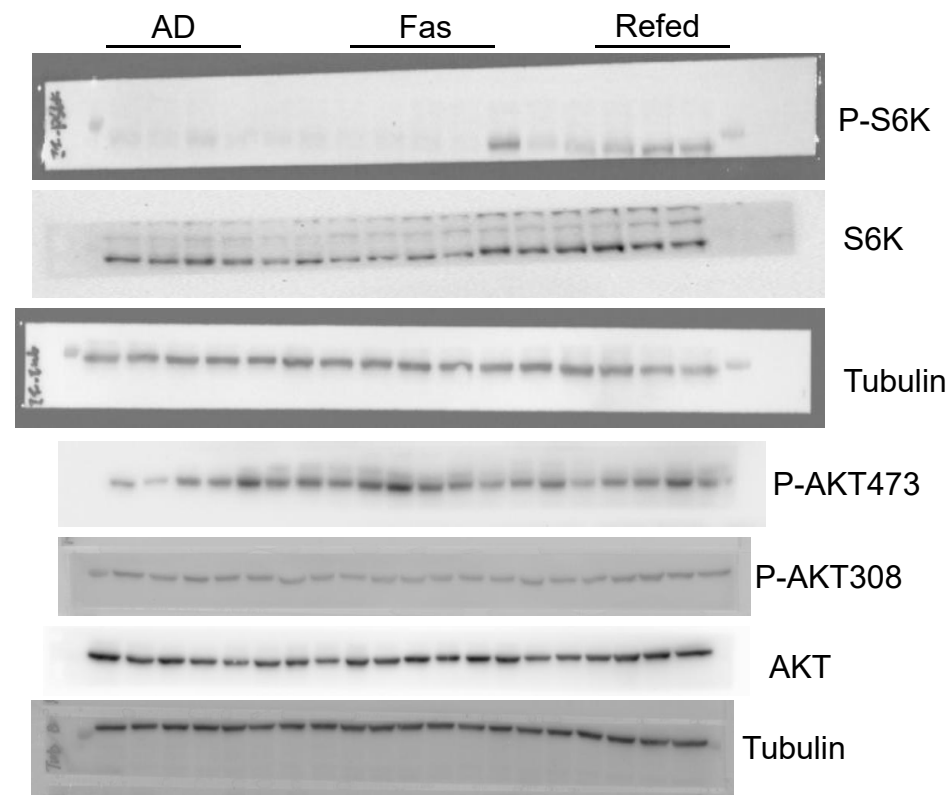

Fig 7B

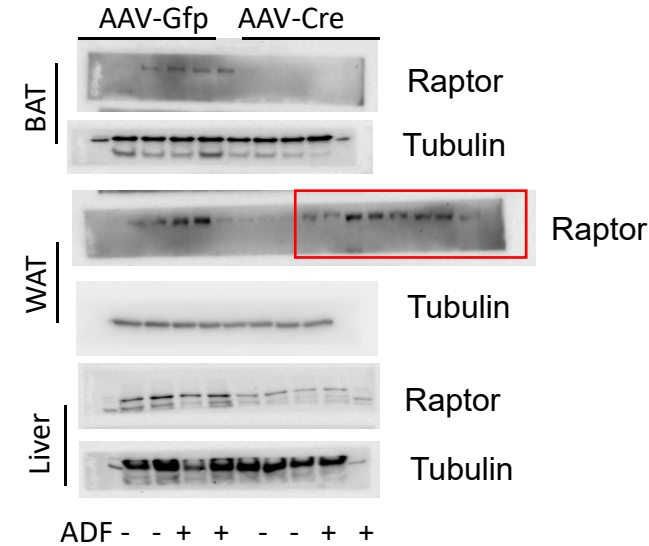

Fig 7G

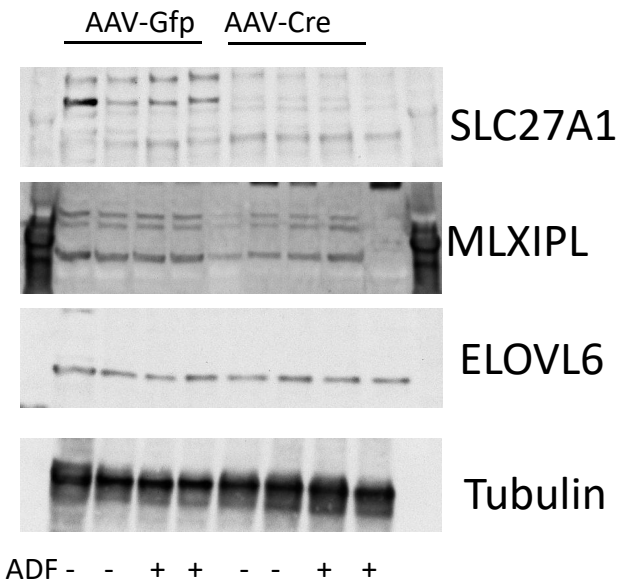

Fig S4D

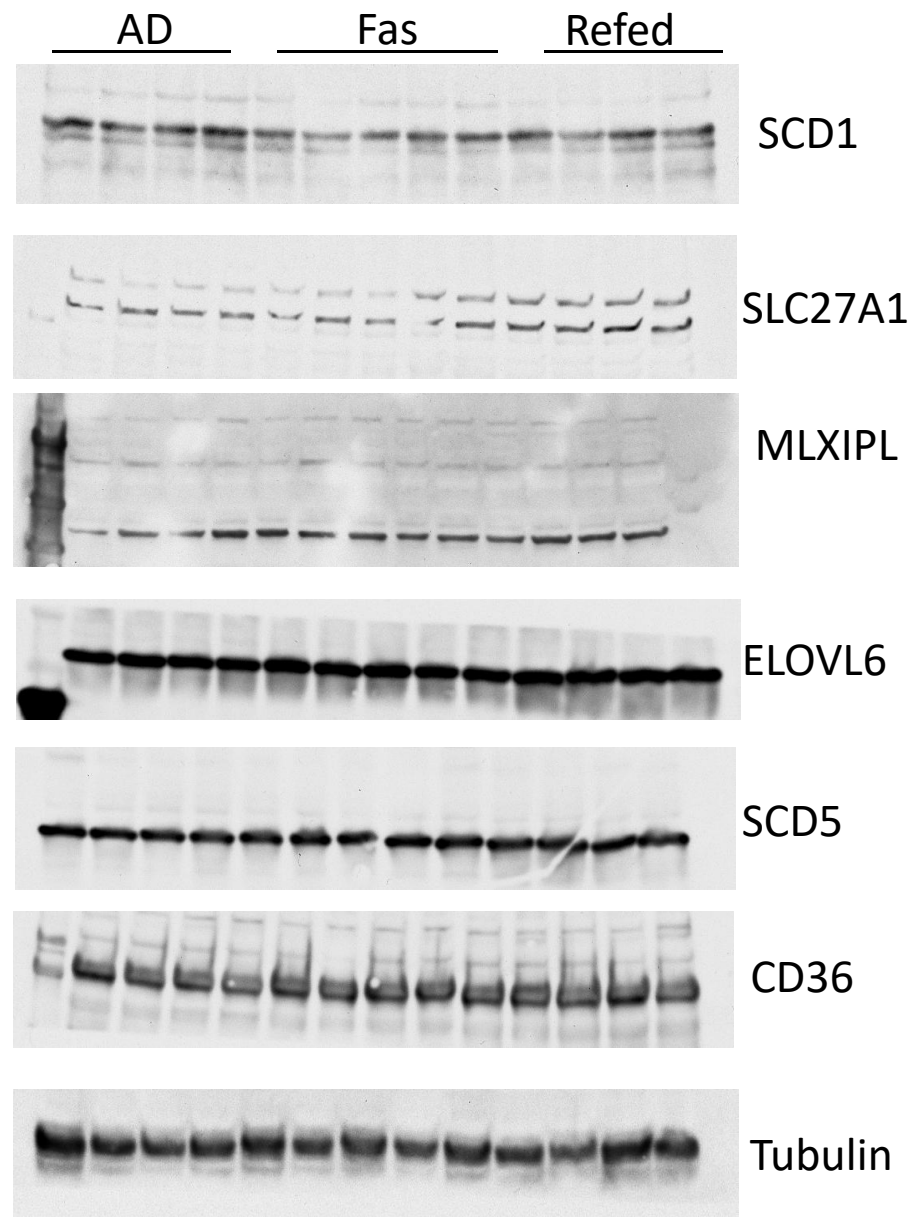

Fig S4B

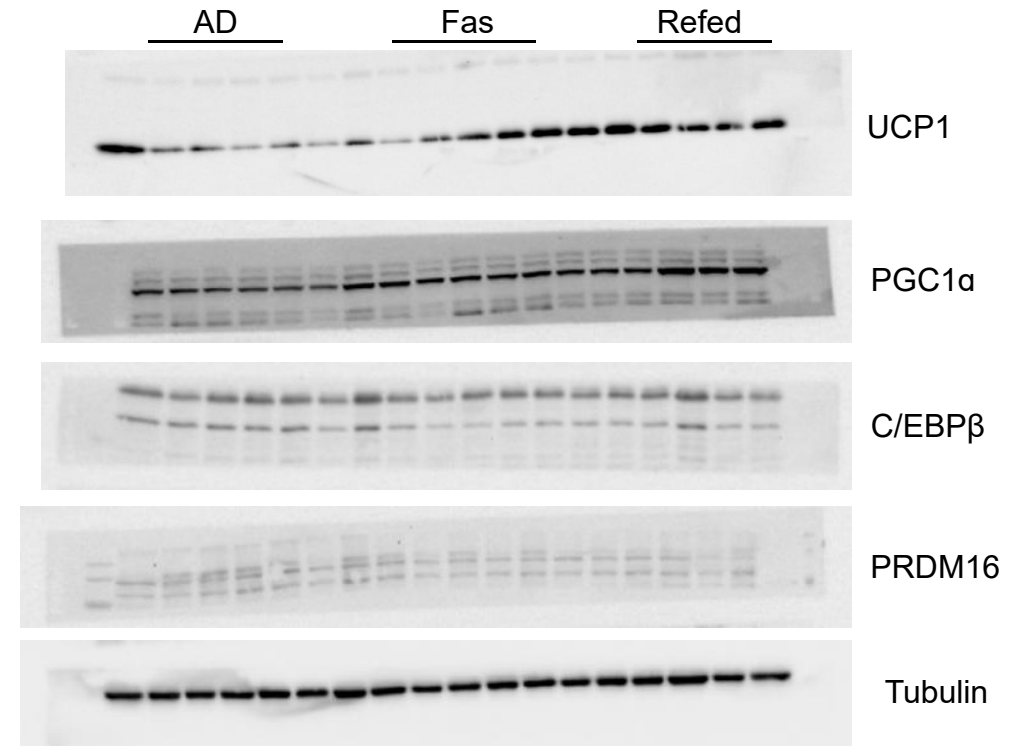

Supplement: S3 Raw Images — (PDF) [file pbio.3003593.s018.pdf]
