# Supplementary material for: Periodic fasting and refeeding re-shapes lipid saturation, storage, and distribution in brown adipose tissue
Source: PLoS Biol. 2026 Jan 12;24(1):e3003593. doi: 10.1371/journal.pbio.3003593 (PMC12795461; doi:10.1371/journal.pbio.3003593)
Supplement: S1 Raw Images — The results showed lipid and metabolite profile of BAT under AD, Refed, and FAS conditions at room temperature (22 ℃) (n = 3/group). (PDF) [file pbio.3003593.s016.pdf]

Brown fat

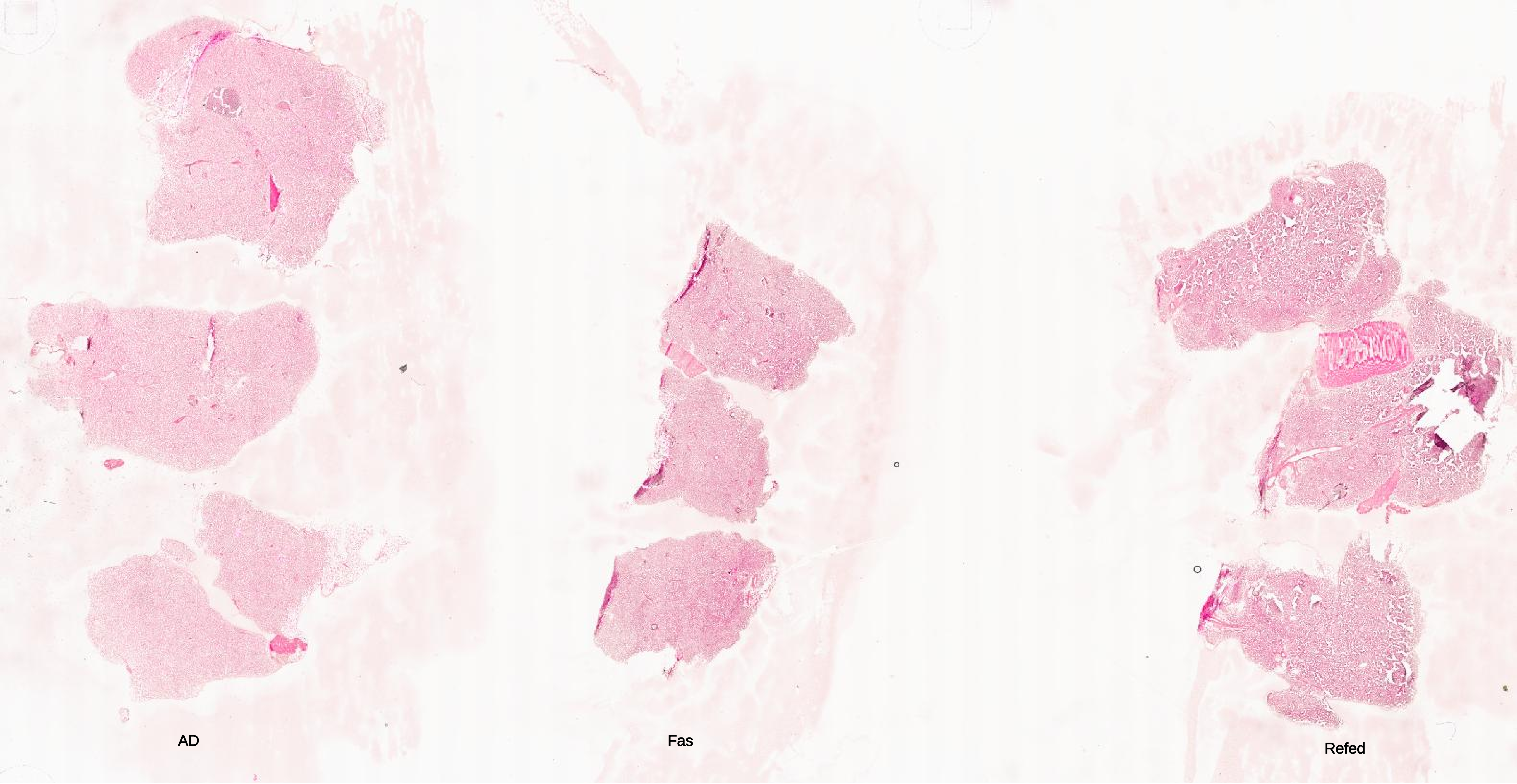

AD
Fas
Refed

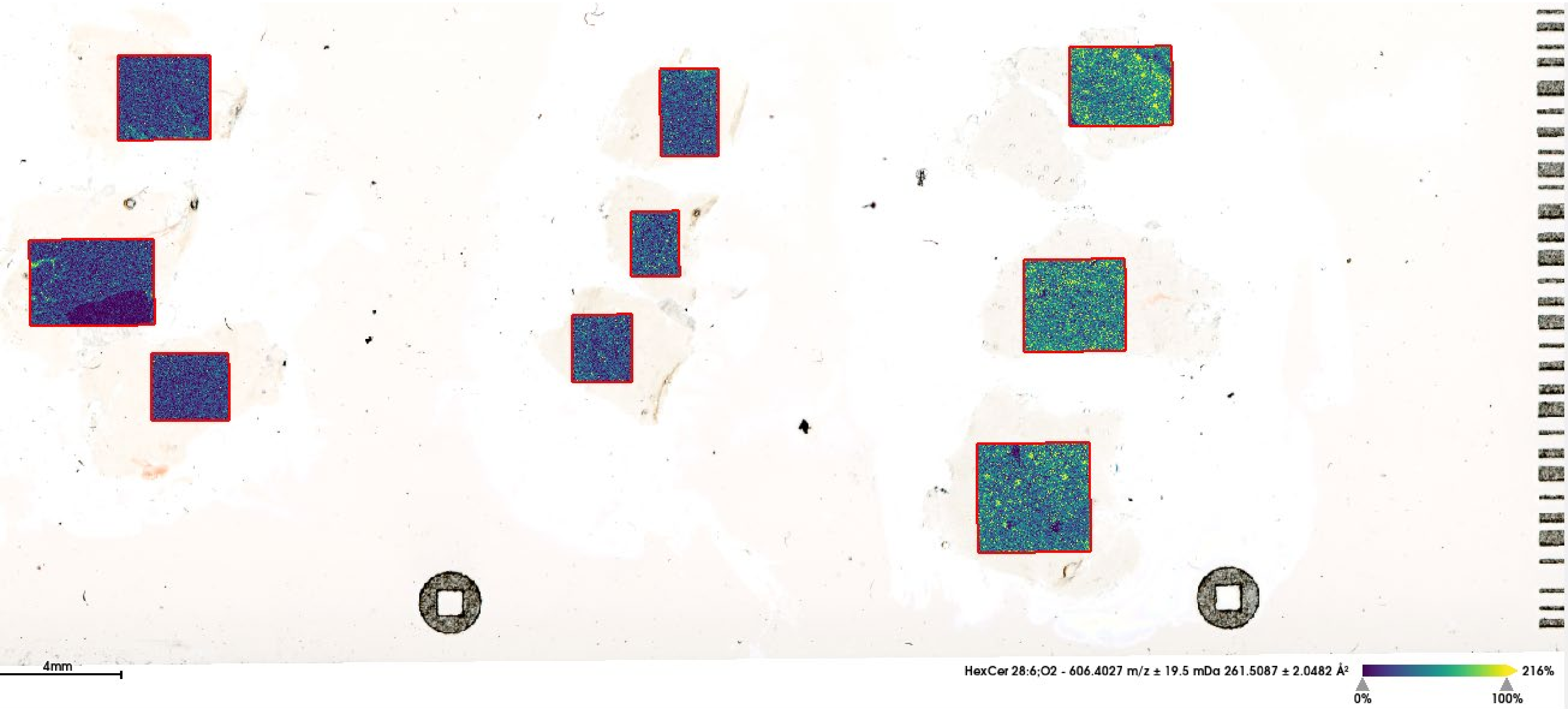

4mm
HexCer 28:6;O2 - 606.4027 m/z ± 19.5 mDa 261.5087 ± 2.0482 Å²
216%
0%
100%

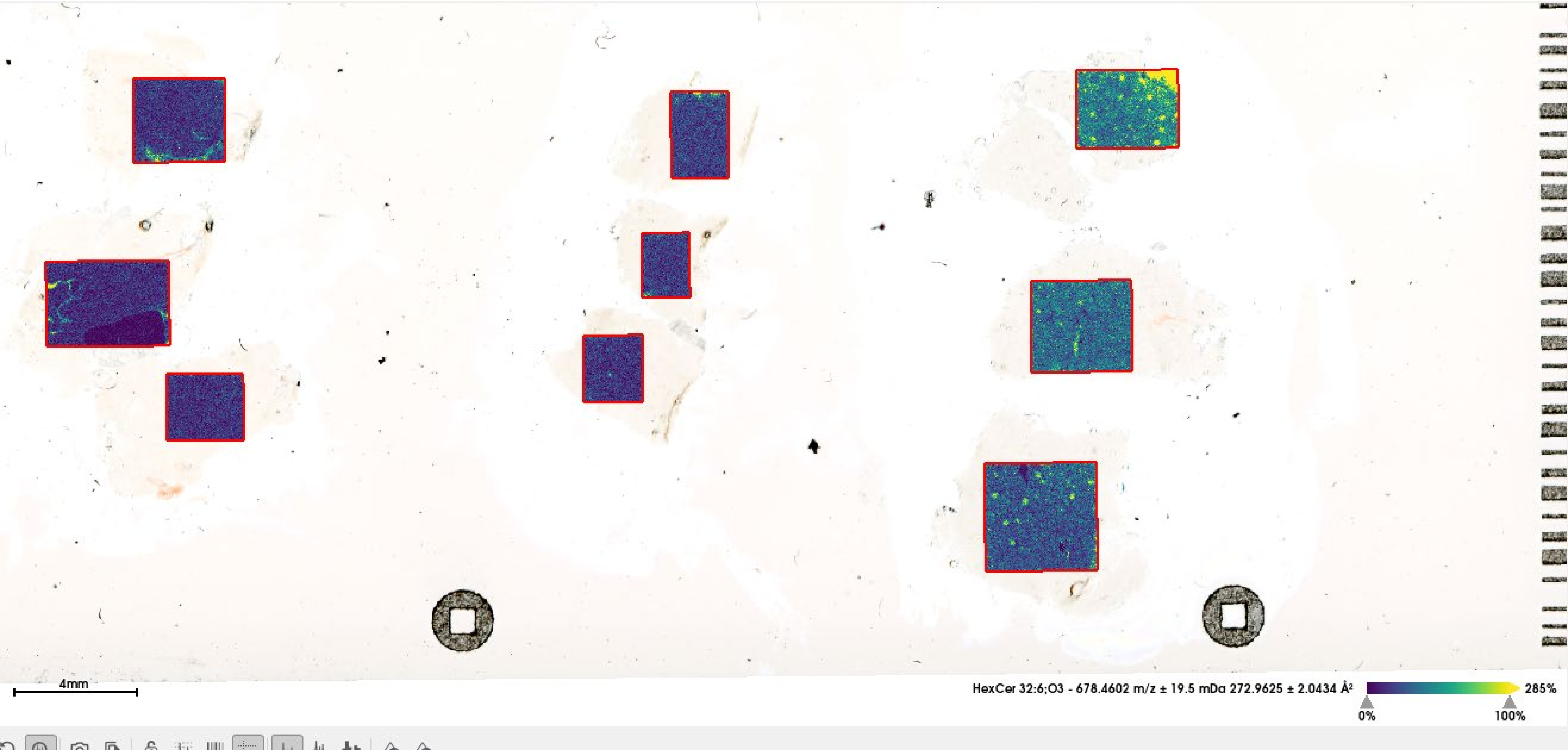
4mm
HexCer 32:6;O3 - 678.4602 m/z ± 19.5 mDa 272.9625 ± 2.0434 Å²
285%
0%
100%

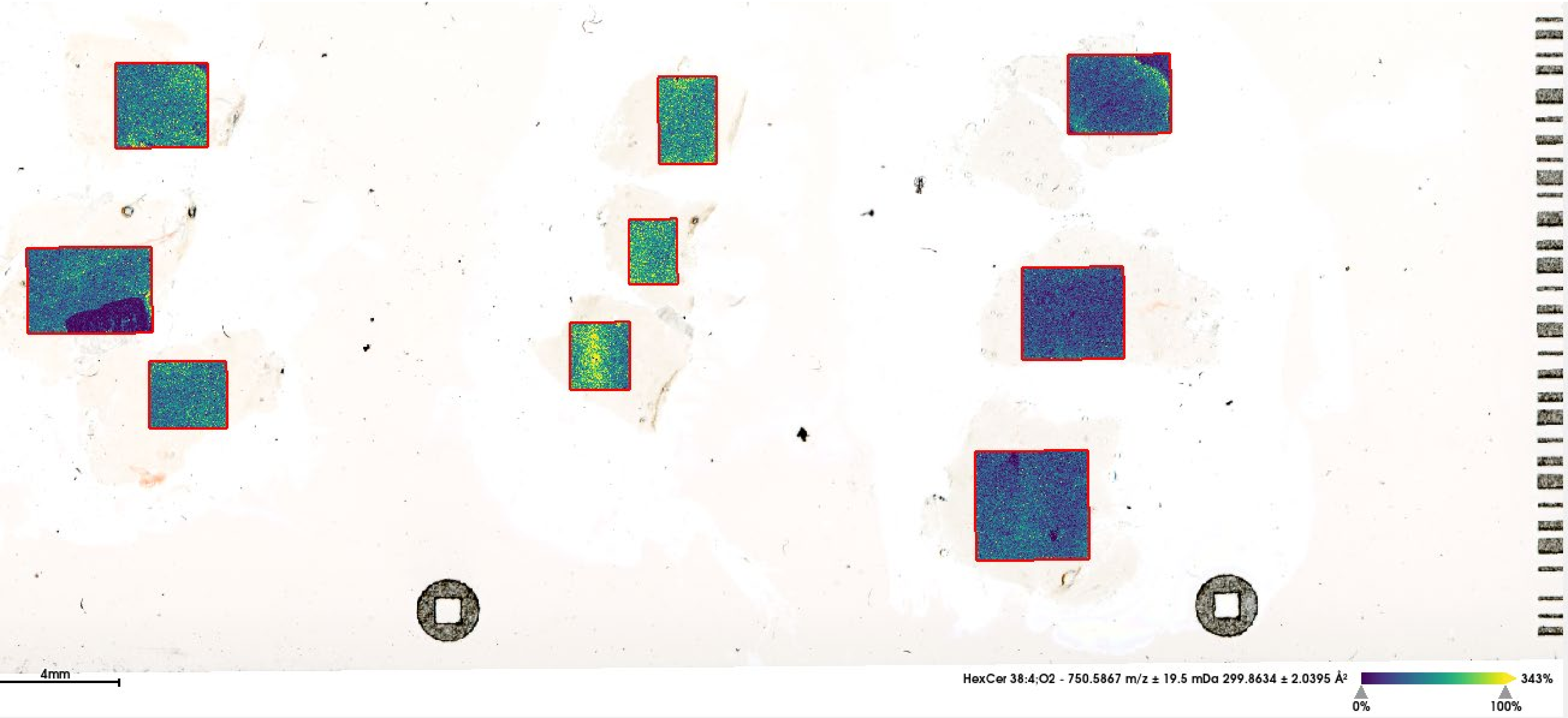
4mm
HexCer 38:4;O2 - 750.5867 m/z ± 19.5 mDa 299.8634 ± 2.0395 Å²
343%
0%
100%

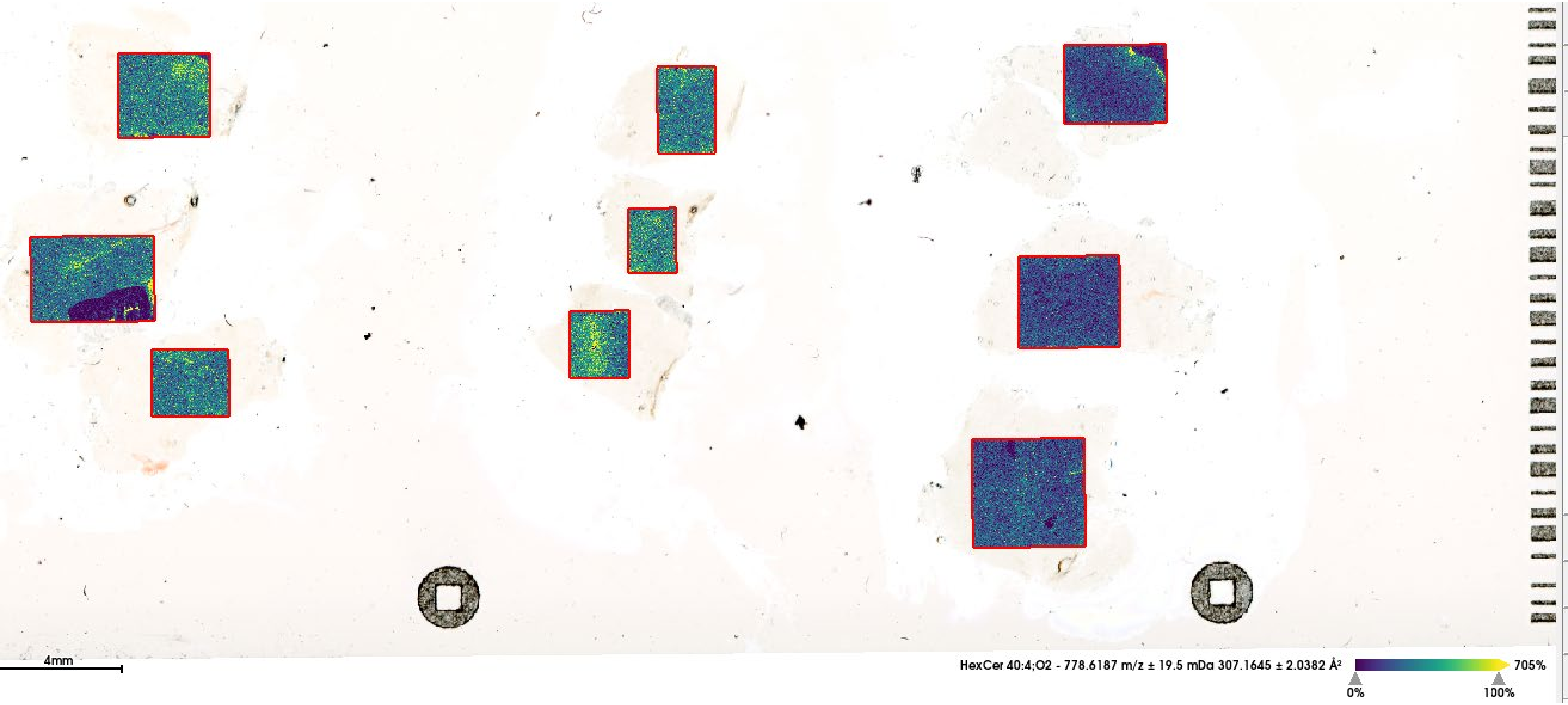
4mm
HexCer 40:4;O2 - 778.6187 m/z ± 19.5 mDa 307.1645 ± 2.0382 Å²
705%
0%
100%

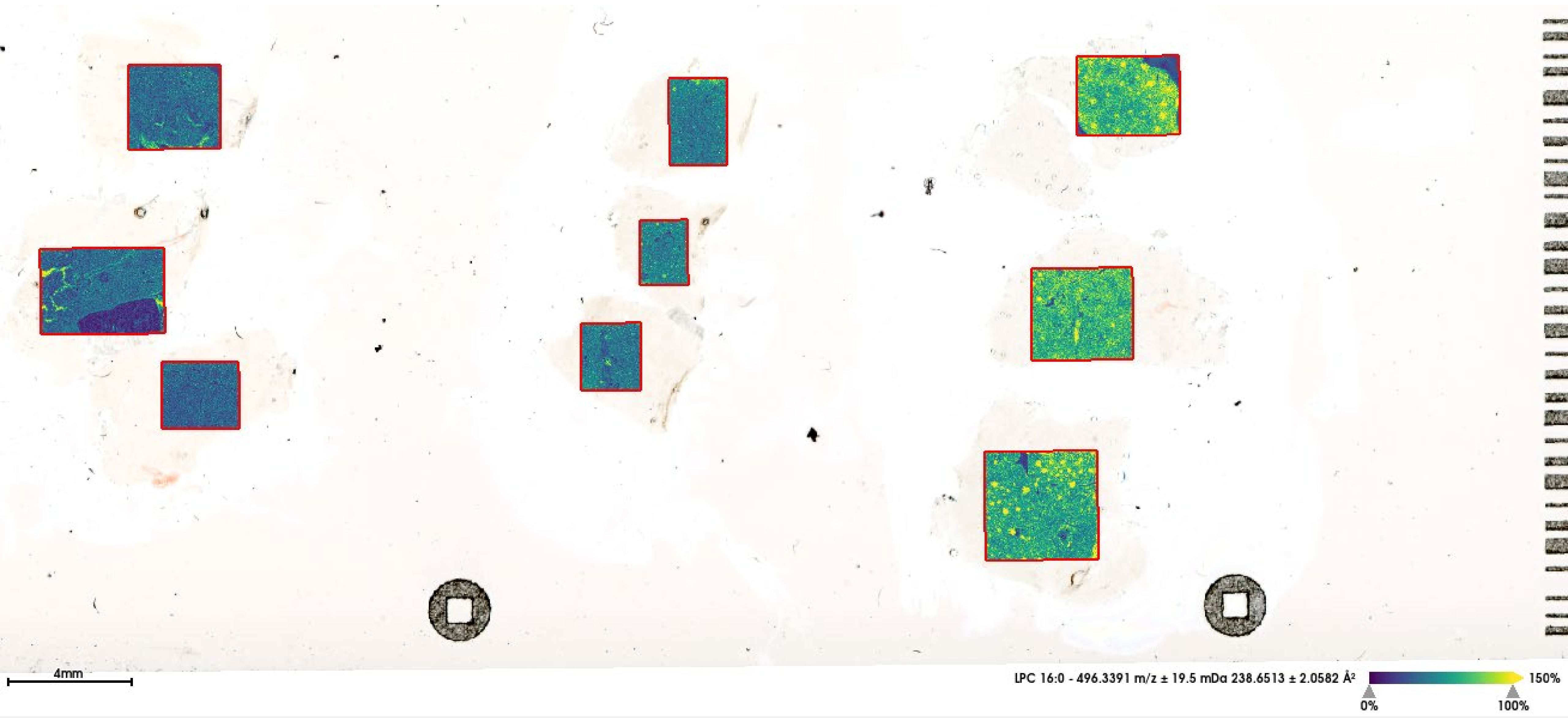
4mm
LPC 16:0 - 496.3391 m/z ± 19.5 mDa 238.6513 ± 2.0582 Å²
150%
0%
100%

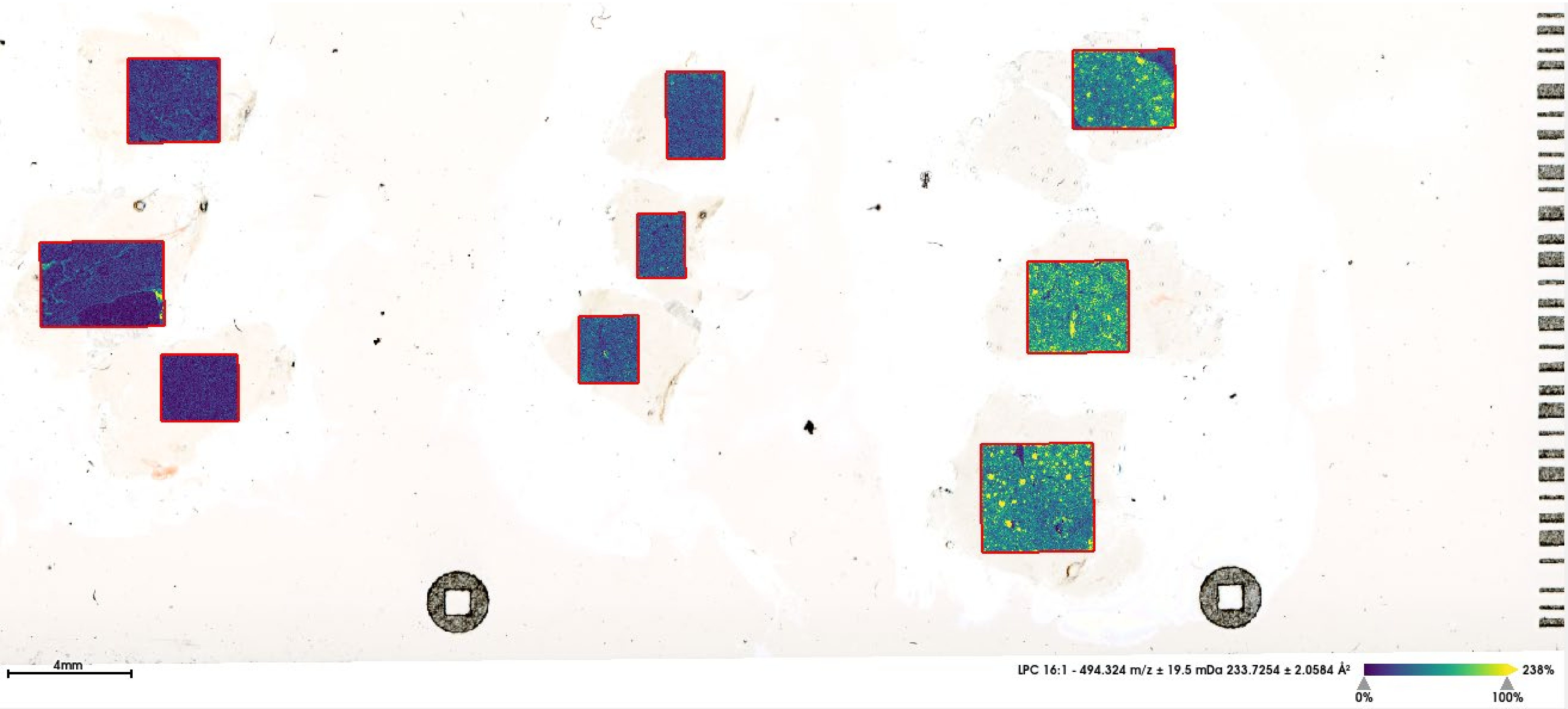
4mm
LPC 16:1 - 494.324 m/z ± 19.5 mDa 233.7254 ± 2.0584 Å²
238%
0%
100%

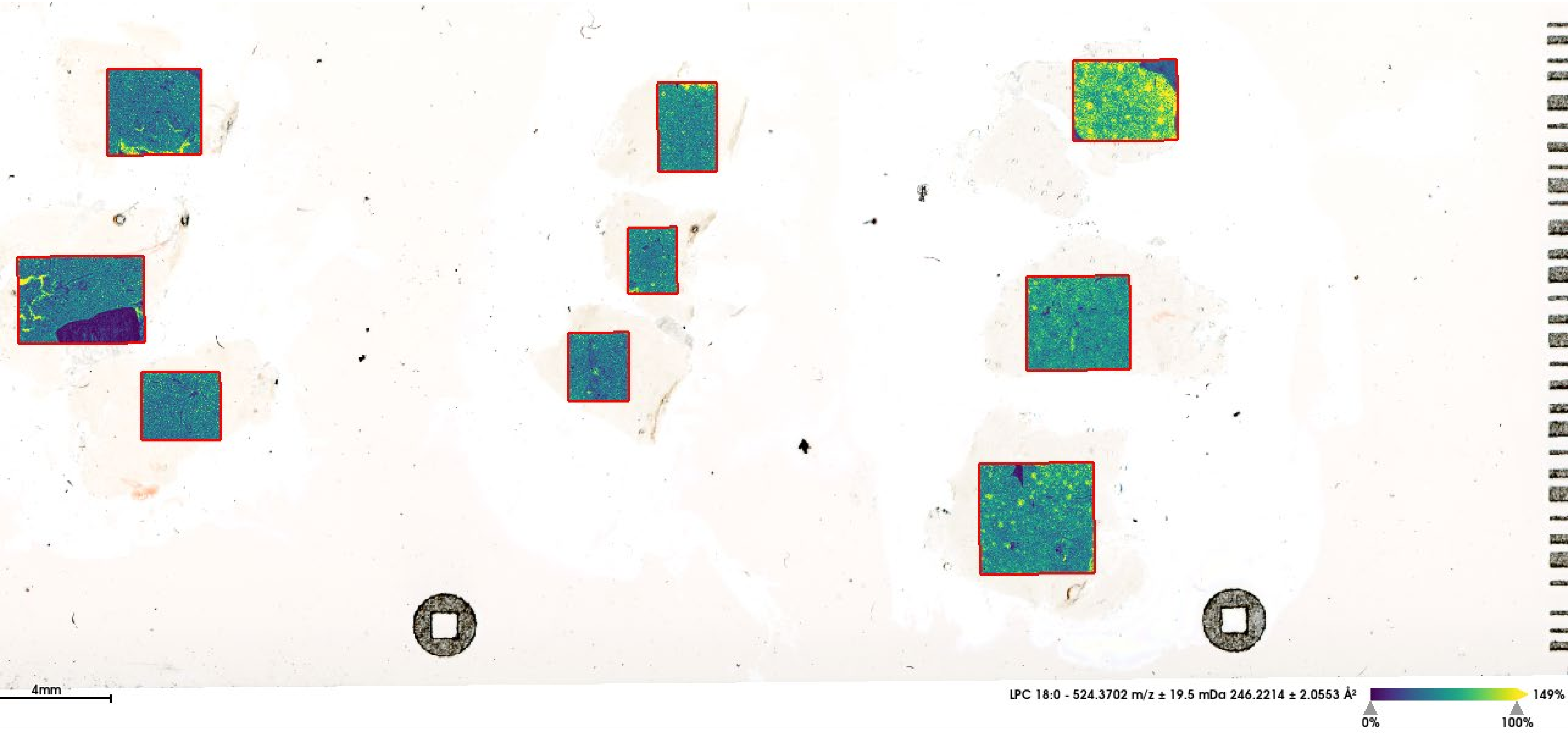

4mm
LPC 18:0 - 524.3702 m/z ± 19.5 mDa 246.2214 ± 2.0553 Å²
149%
0%
100%

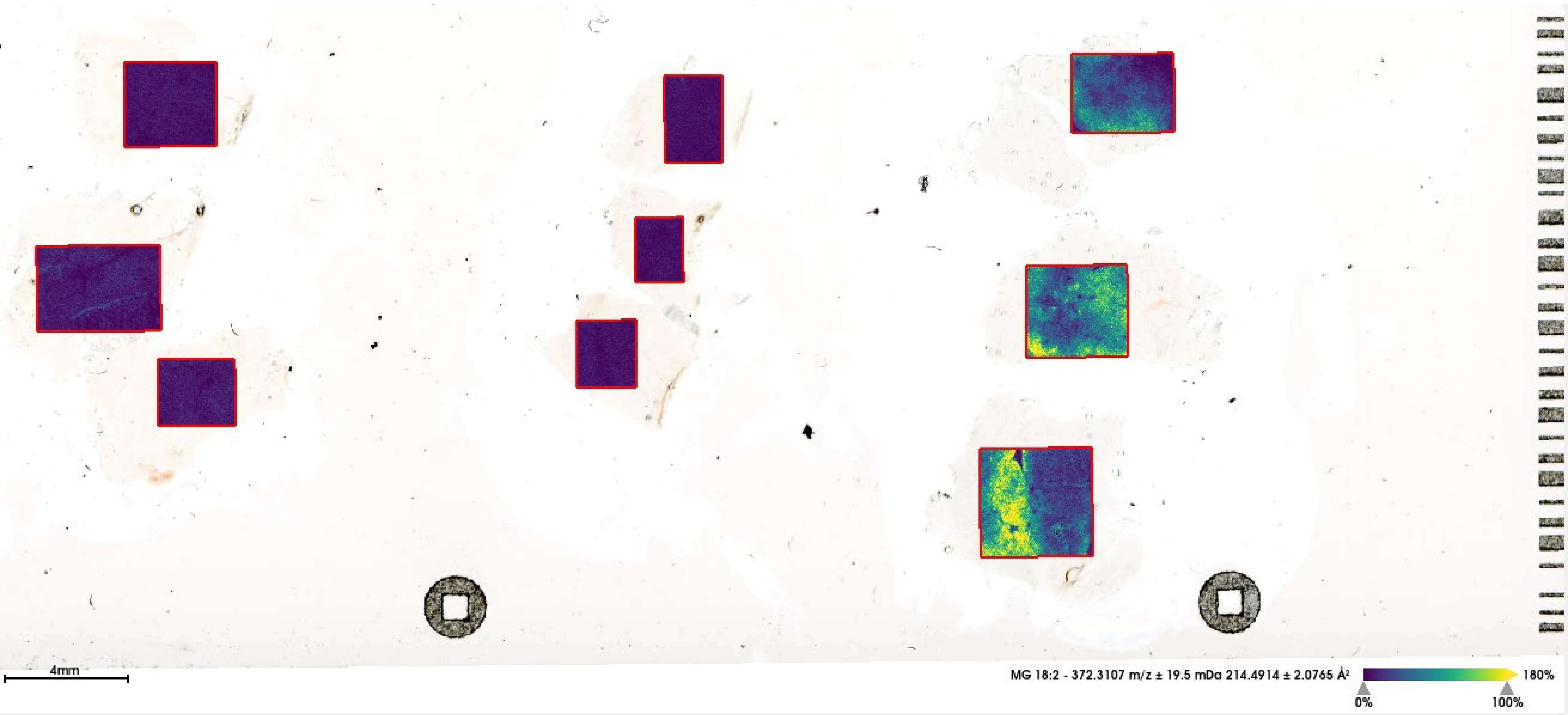
4mm
MG 18:2 - 372.3107 m/z ± 19.5 mDa 214.4914 ± 2.0765 Å²
180%
0%
100%

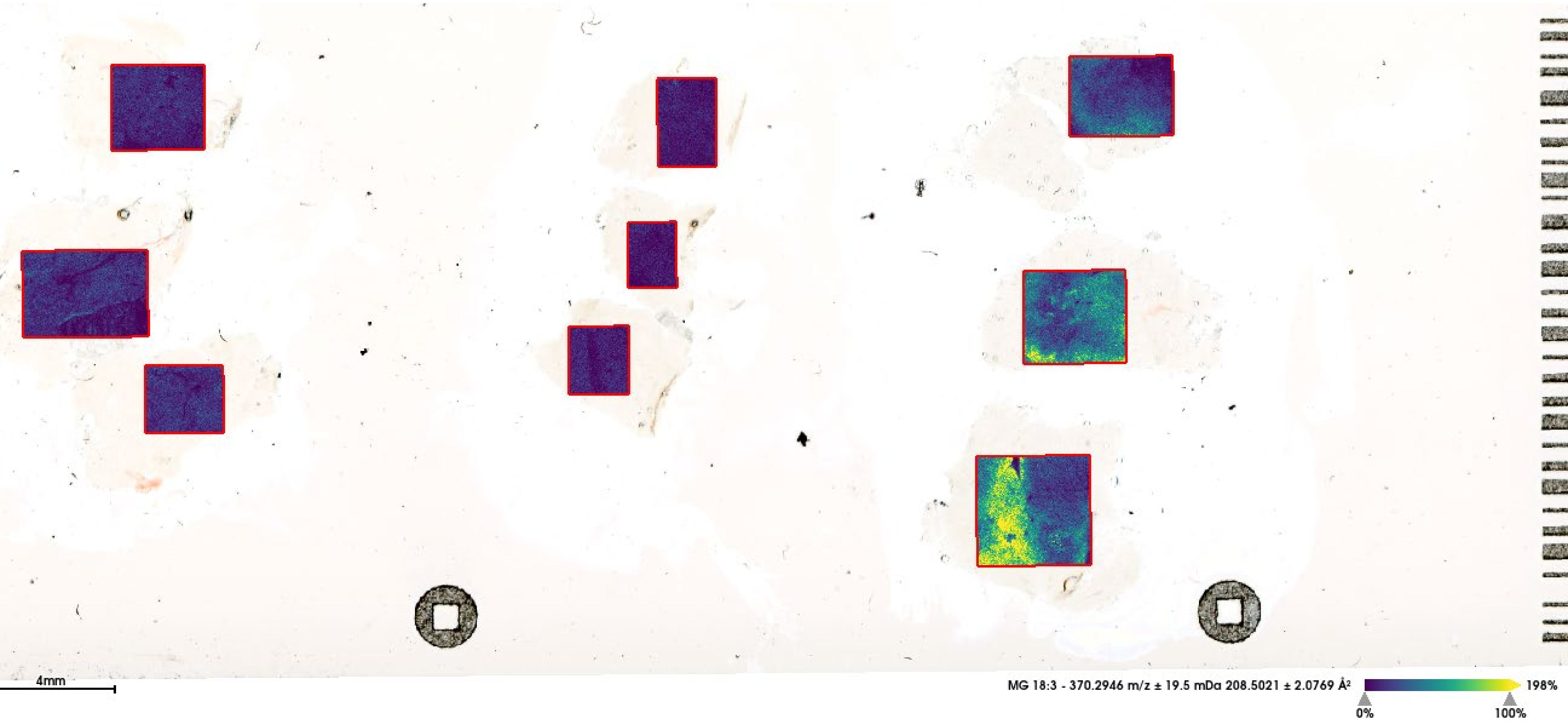
4mm
MG 18:3 - 370.2946 m/z ± 19.5 mDa 208.5021 ± 2.0769 Å²
198%
0%
100%

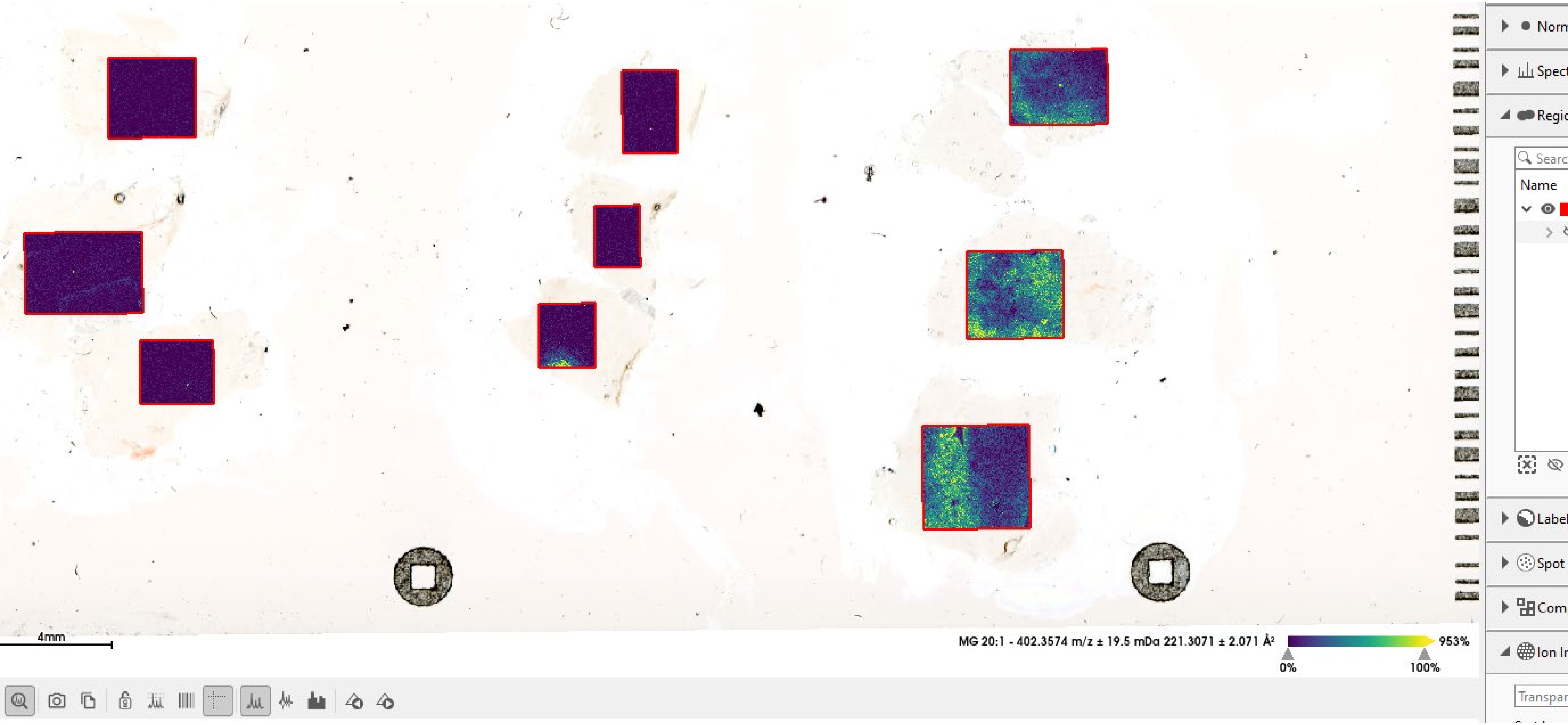

4mm
MG 20:1 - 402.3574 m/z ± 19.5 mDa 221.3071 ± 2.071 Å²
953%
0%
100%
Norm
Spect
Regio
Searc
Name
Label
Spot
Com
Ion Ir
Transpar

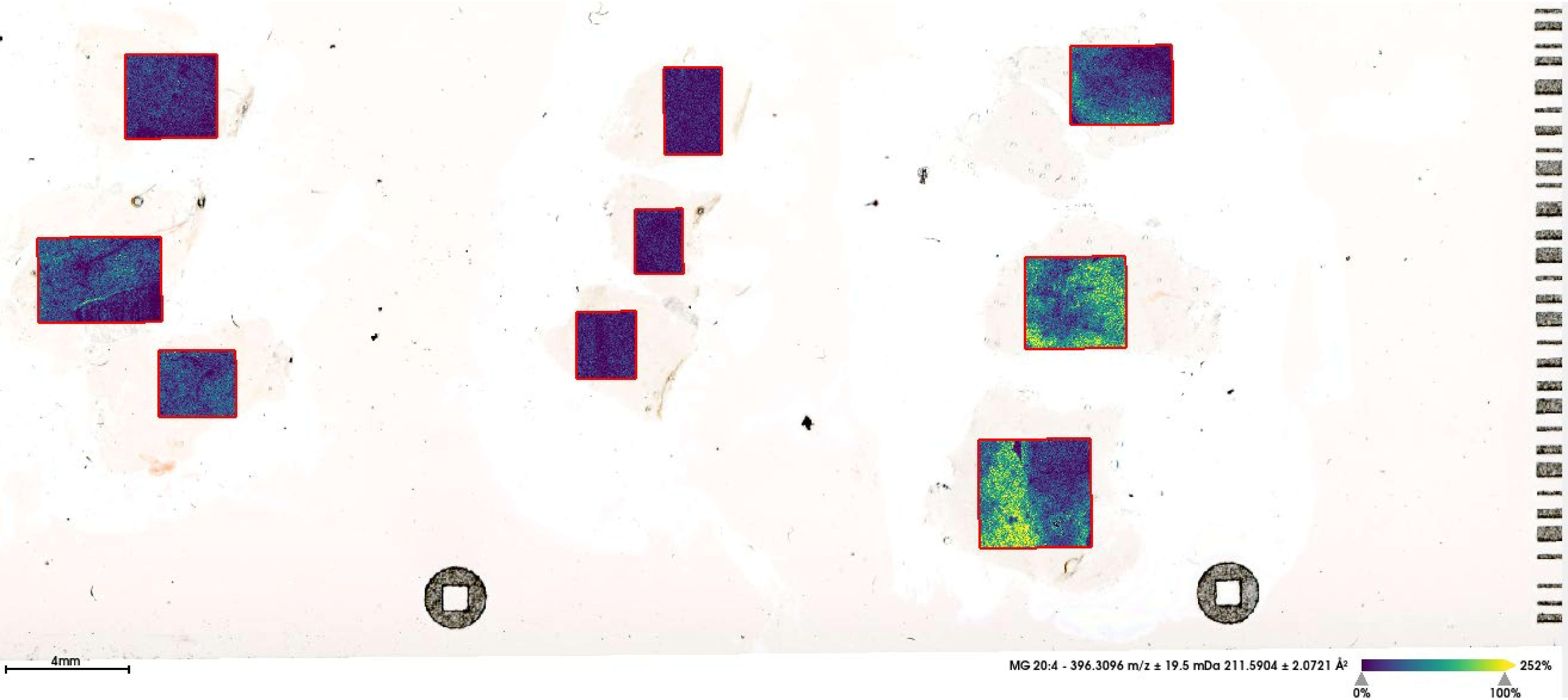
4mm
MG 20:4 - 396.3096 m/z ± 19.5 mDa 211.5904 ± 2.0721 Å²
252%
0%
100%

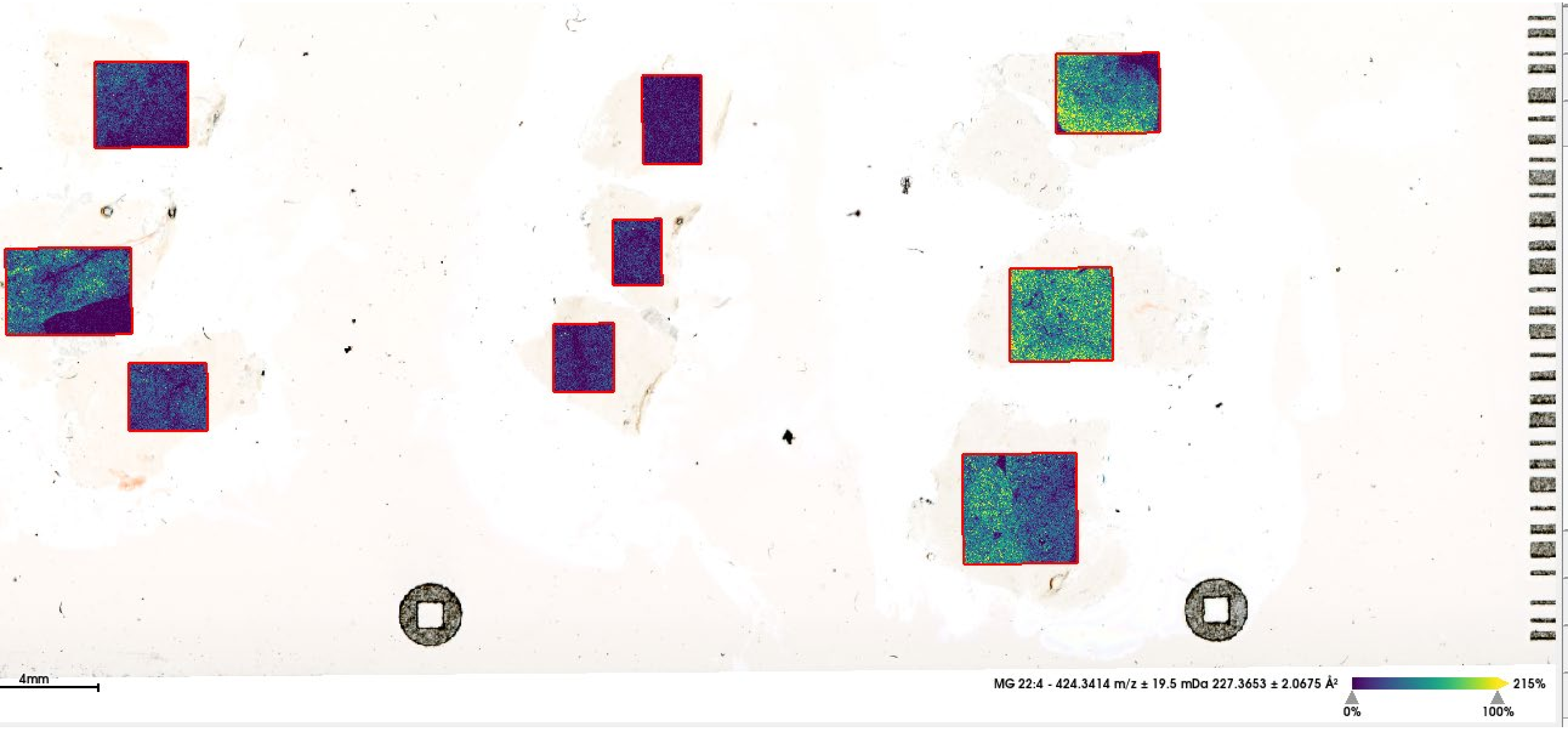

4mm
MG 22:4 - 424.3414 m/z ± 19.5 mDa 227.3653 ± 2.0675 Å²
215%
0%
100%

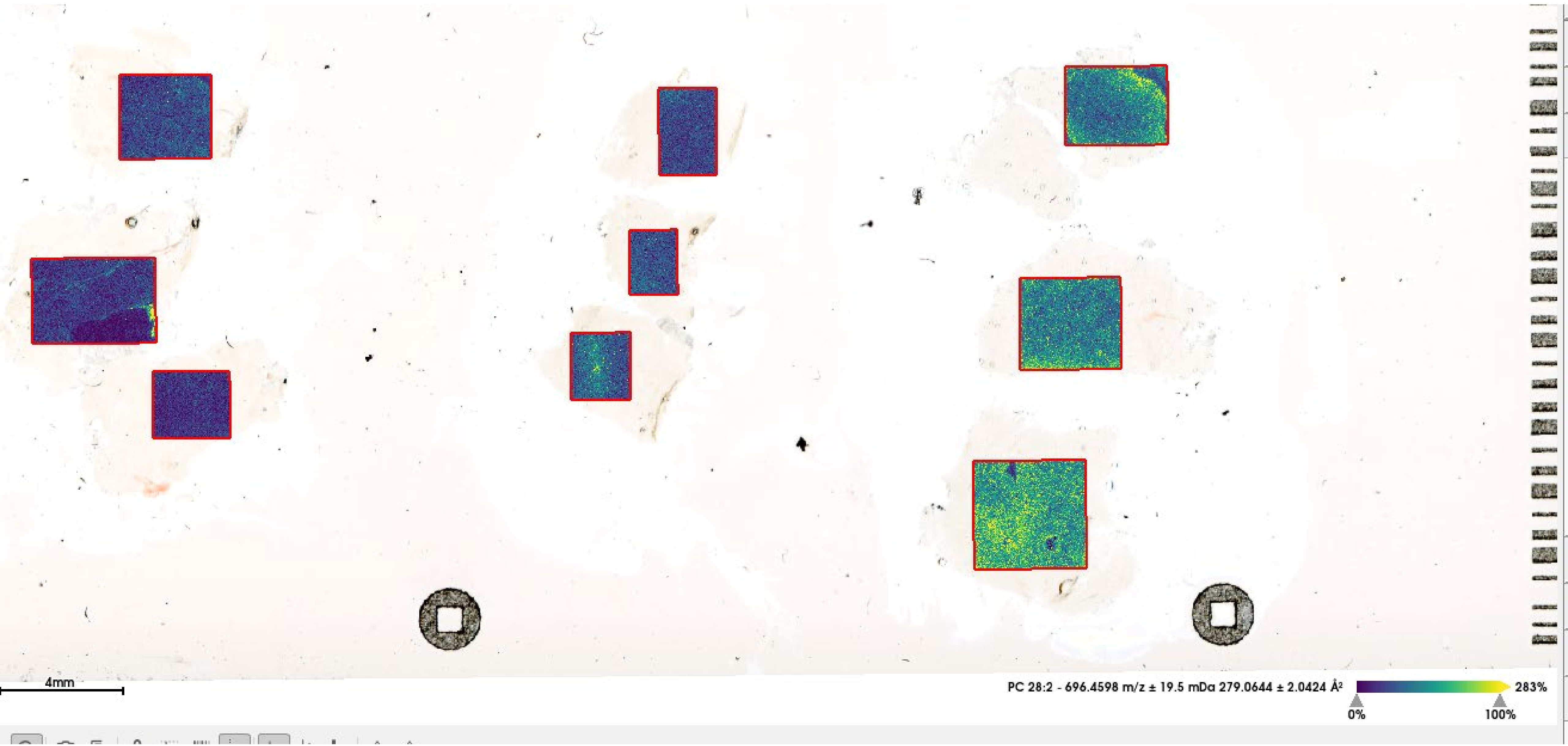
4mm
PC 28:2 - 696.4598 m/z ± 19.5 mDa 279.0644 ± 2.0424 Å²
283%
0%
100%

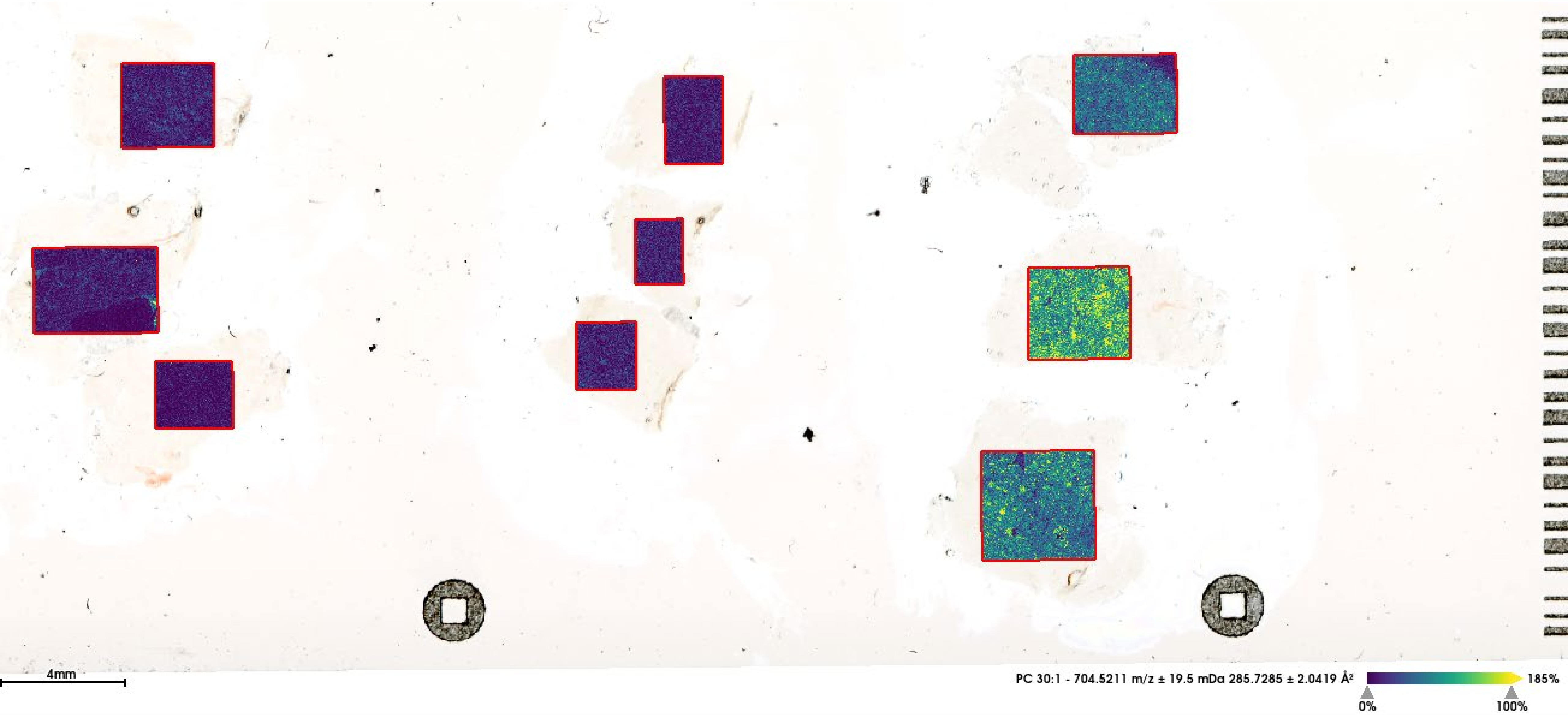

4mm
PC 30:1 - 704.5211 m/z ± 19.5 mDa 285.7285 ± 2.0419 Å²
185%
0%
100%

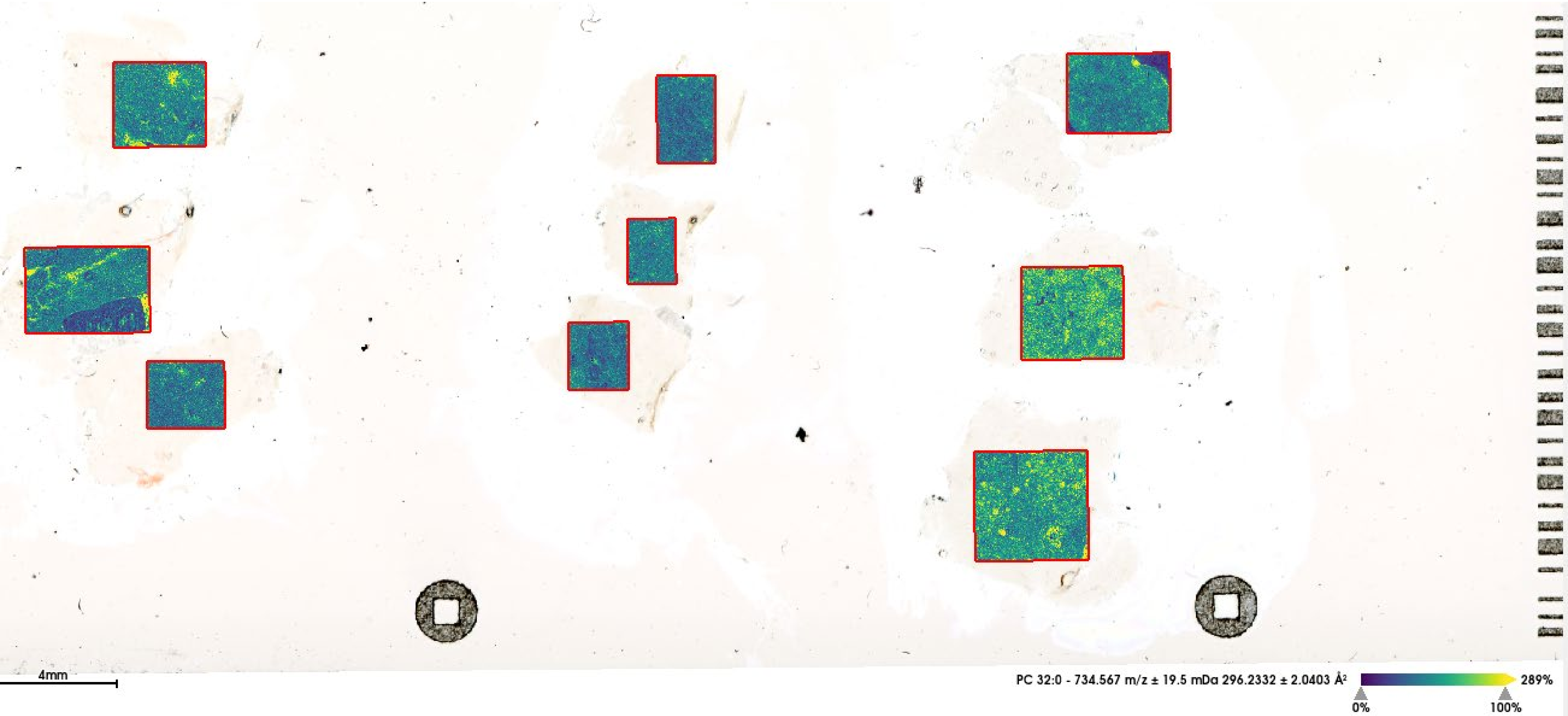

4mm
PC 32:0 - 734.567 m/z ± 19.5 mDa 296.2332 ± 2.0403 Å²
289%
0%
100%

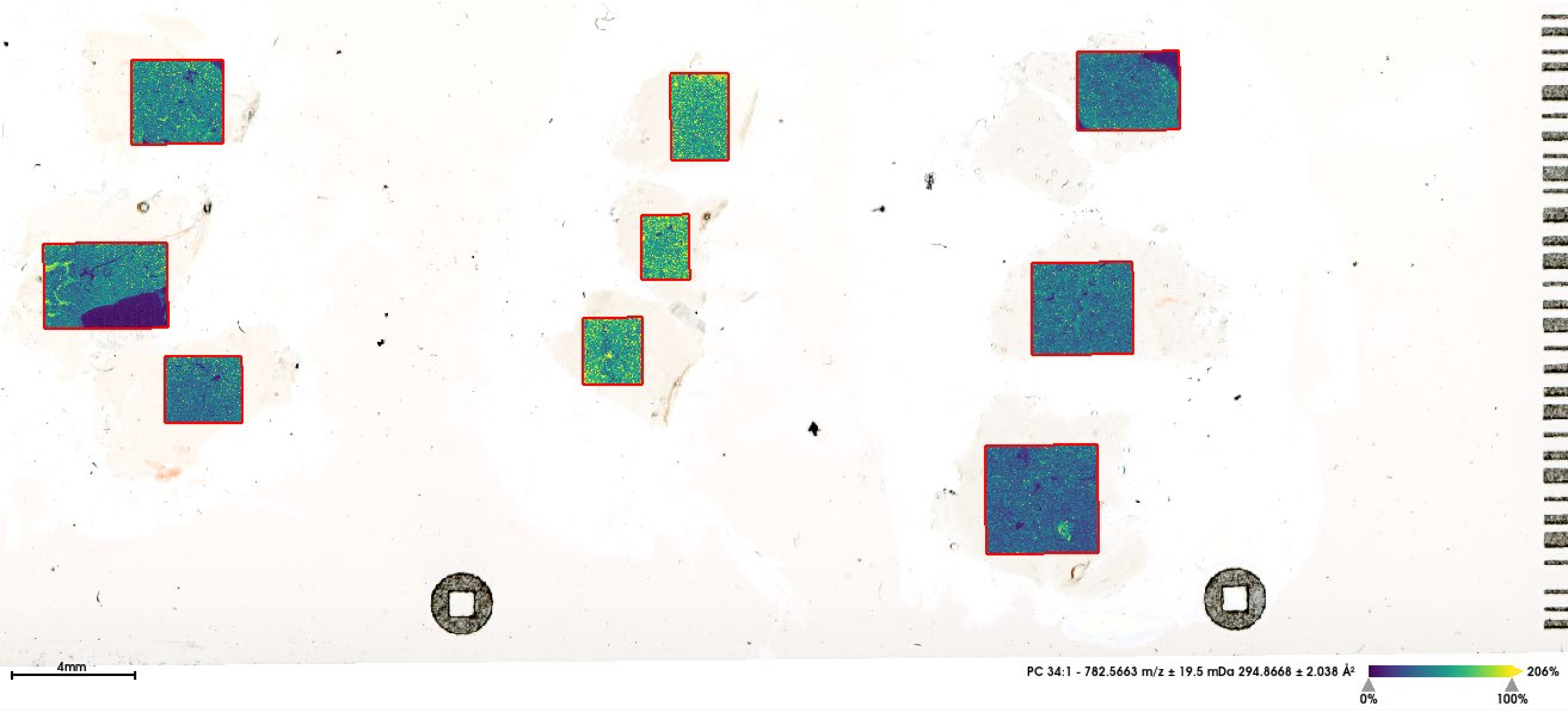

4mm
PC 34:1 - 782.5663 m/z ± 19.5 mDa 294.8668 ± 2.038 Å²
206%
0%
100%

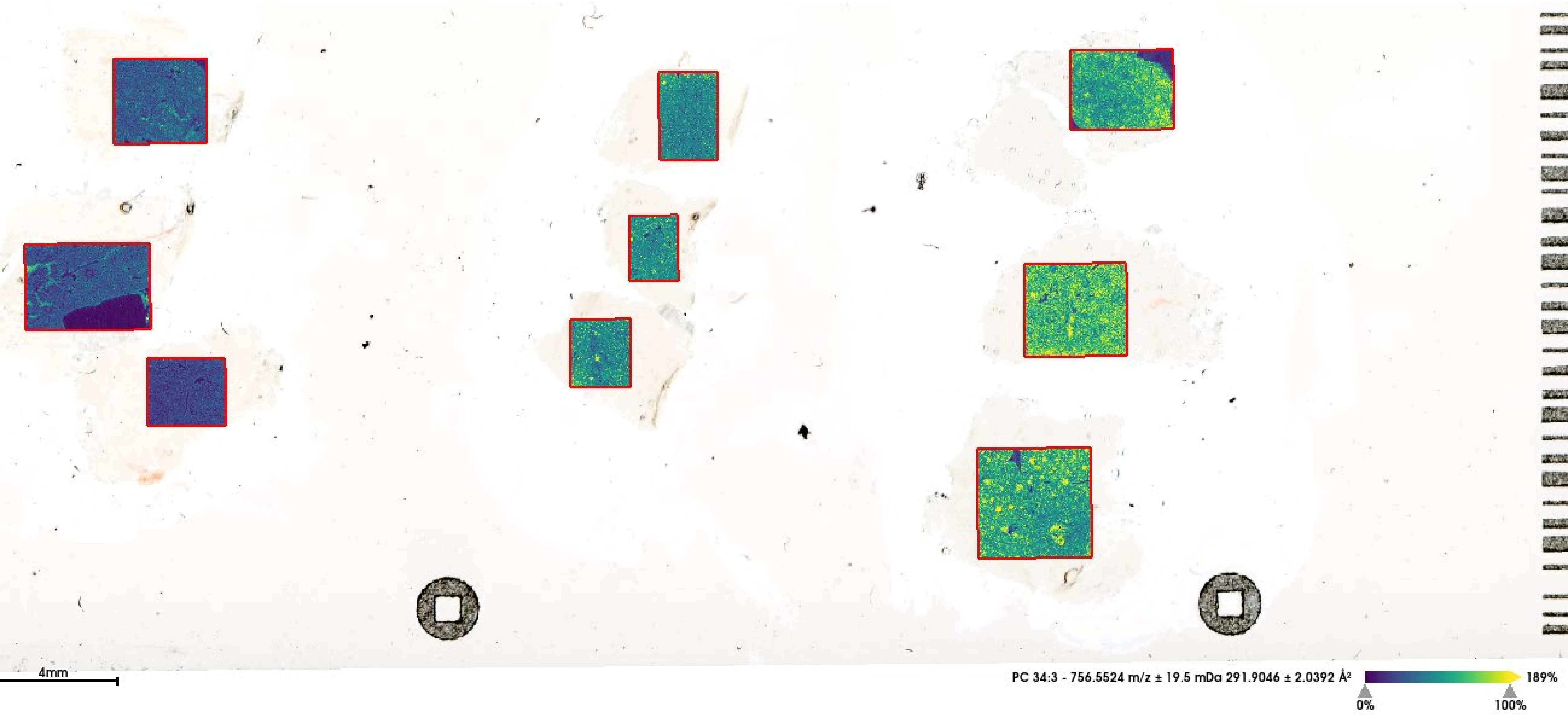
4mm
PC 34:3 - 756.5524 m/z ± 19.5 mDa 291.9046 ± 2.0392 Å²
189%
0%
100%

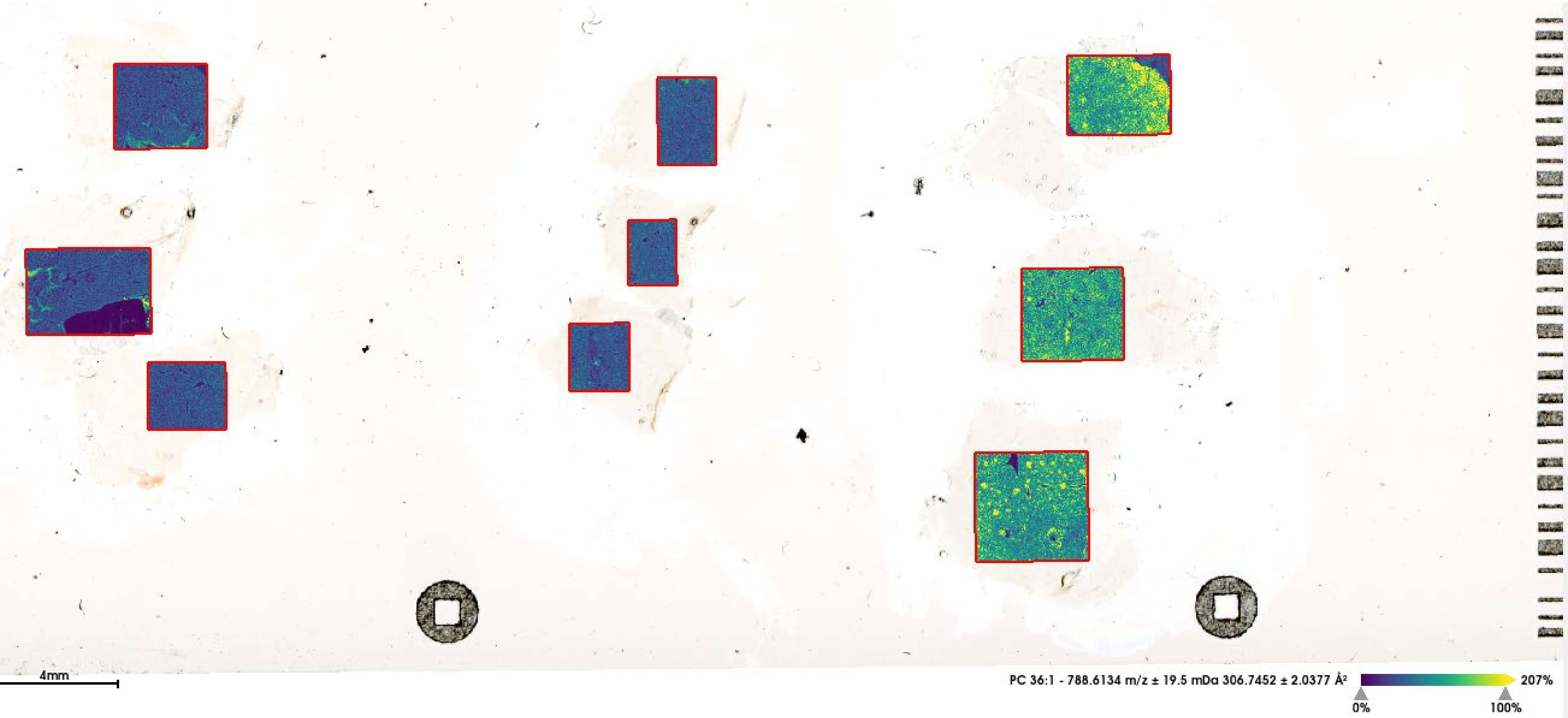
4mm
PC 36:1 - 788.6134 m/z ± 19.5 mDa 306.7452 ± 2.0377 Å²
207%
0%
100%

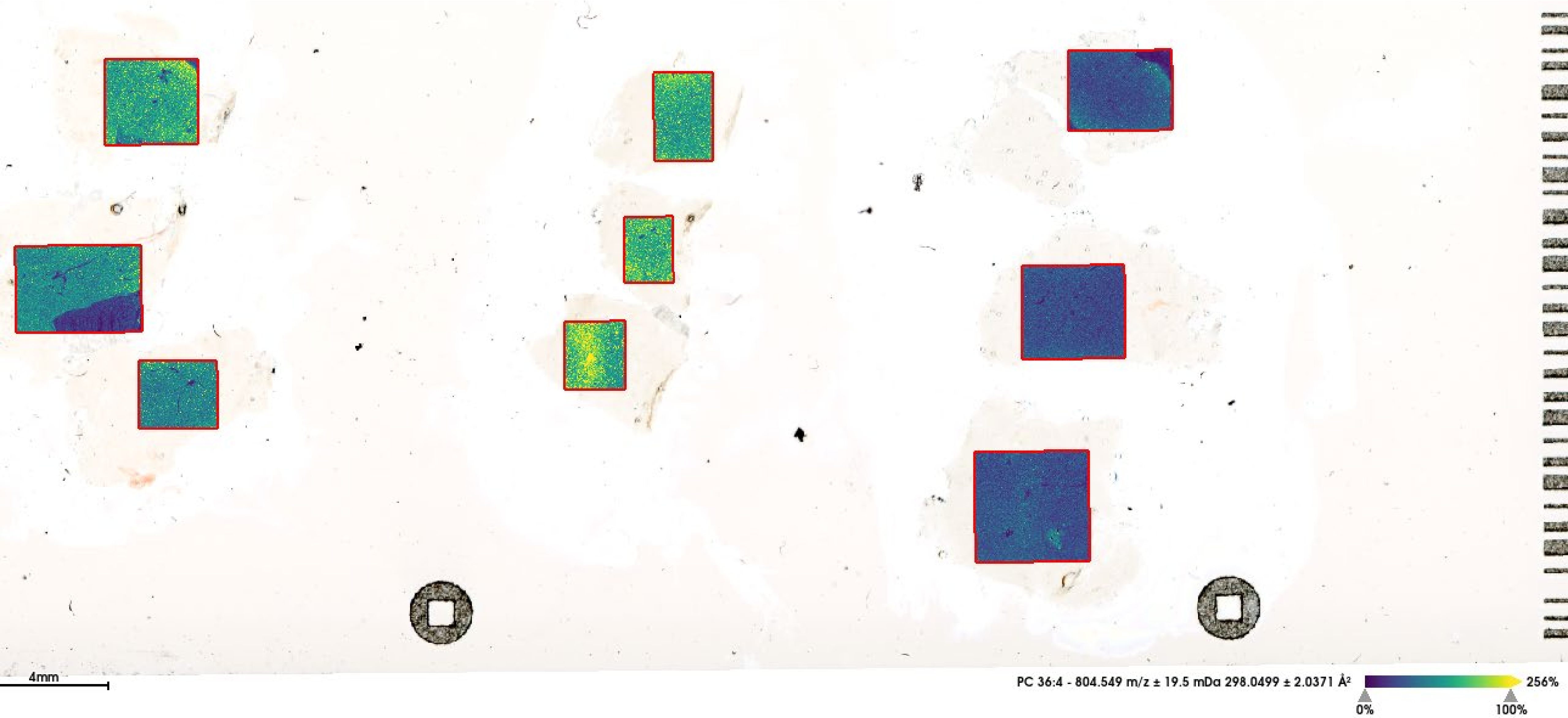

4mm
PC 36:4 - 804.549 m/z ± 19.5 mDa 298.0499 ± 2.0371 Å²
256%
0%
100%

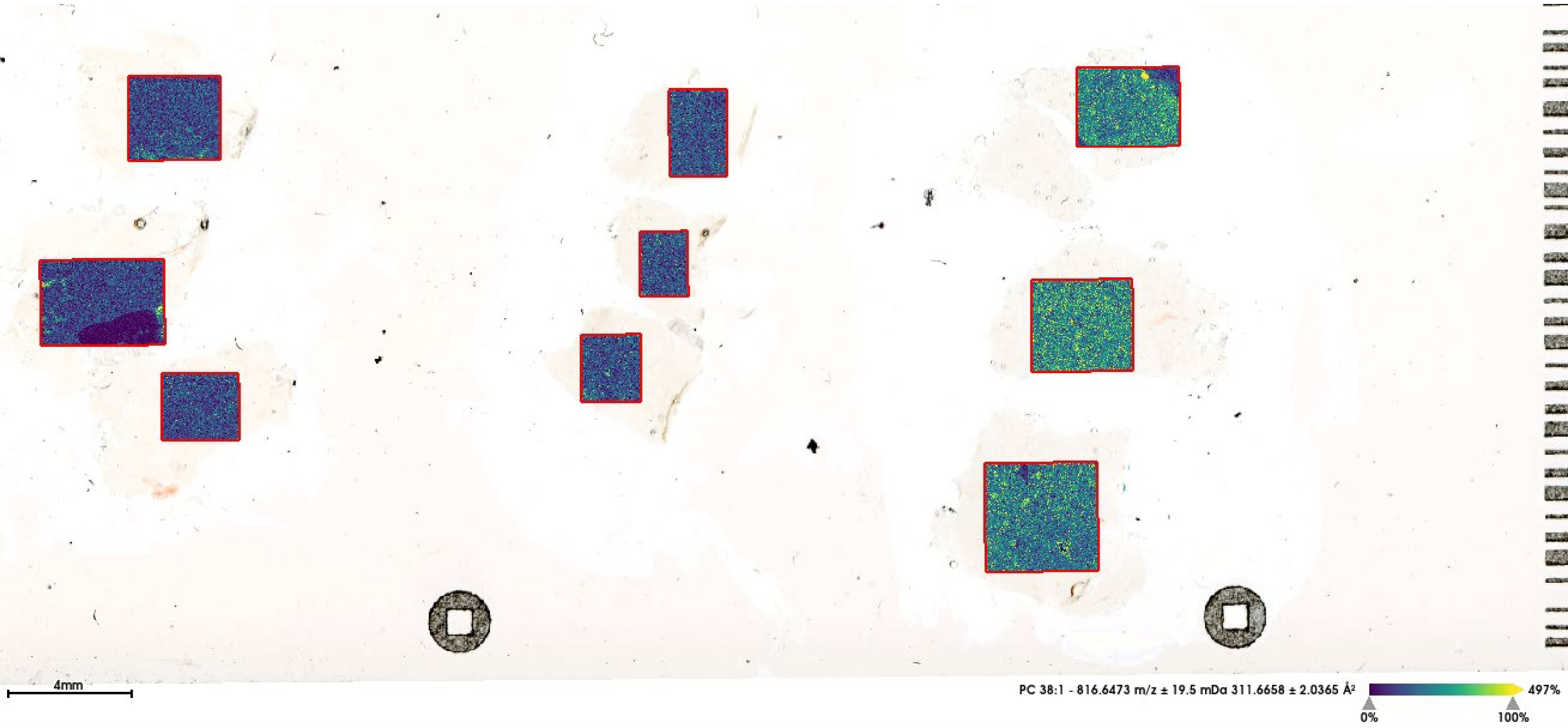

4mm
PC 38:1 - 816.6473 m/z ± 19.5 mDa 311.6658 ± 2.0365 Å²
497%
0%
100%

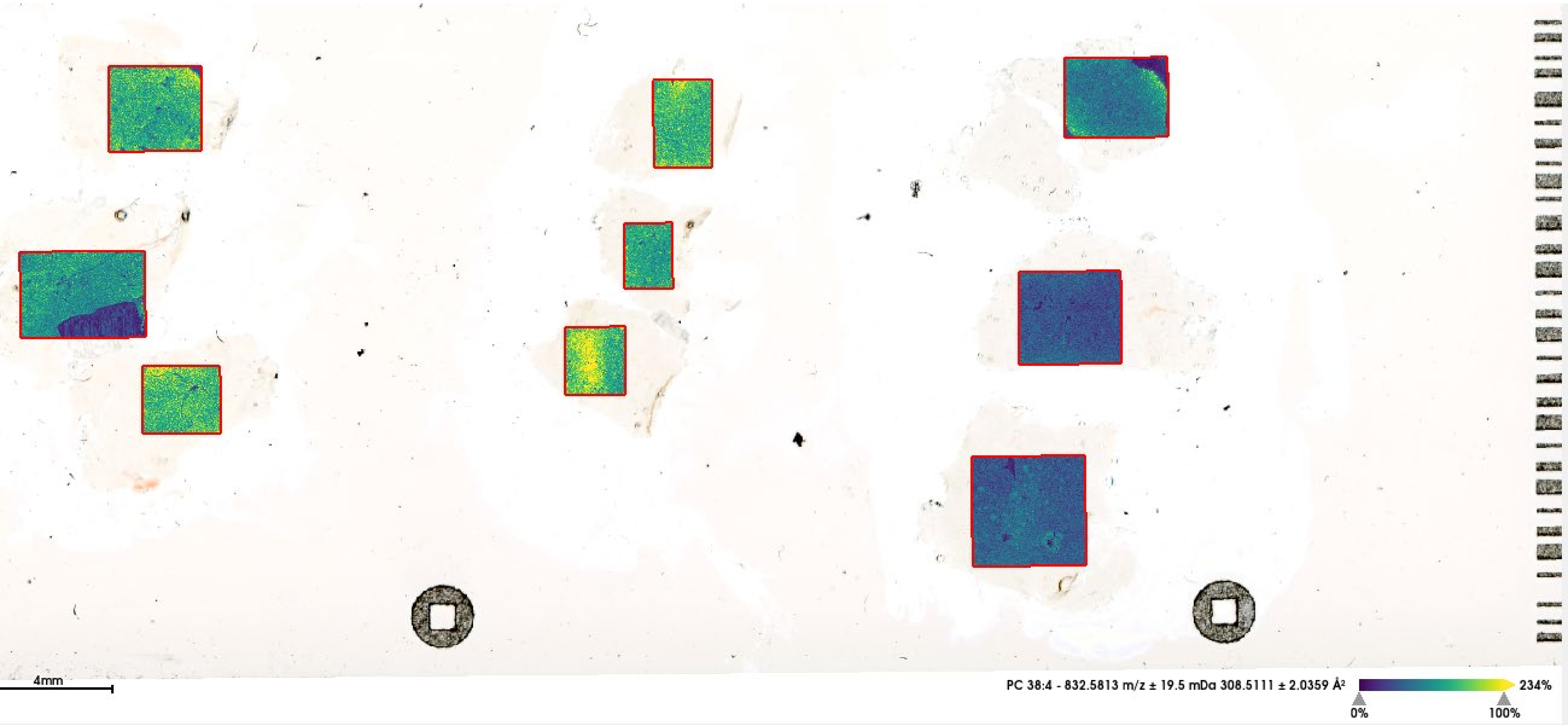
4mm
PC 38:4 - 832.5813 m/z ± 19.5 mDa 308.5111 ± 2.0359 Å²
234%
0%
100%

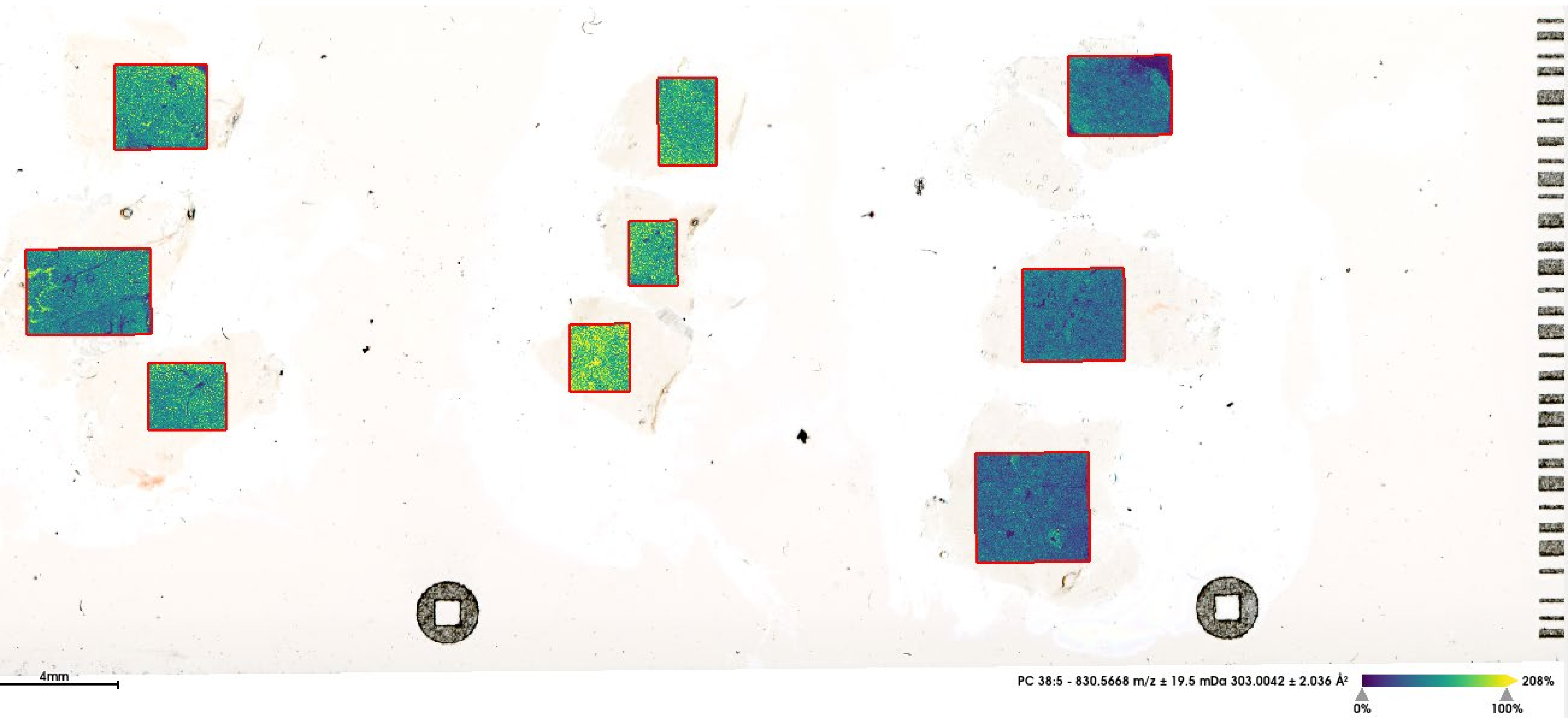

4mm
PC 38:5 - 830.5668 m/z ± 19.5 mDa 303.0042 ± 2.036 Å²
208%
0%
100%

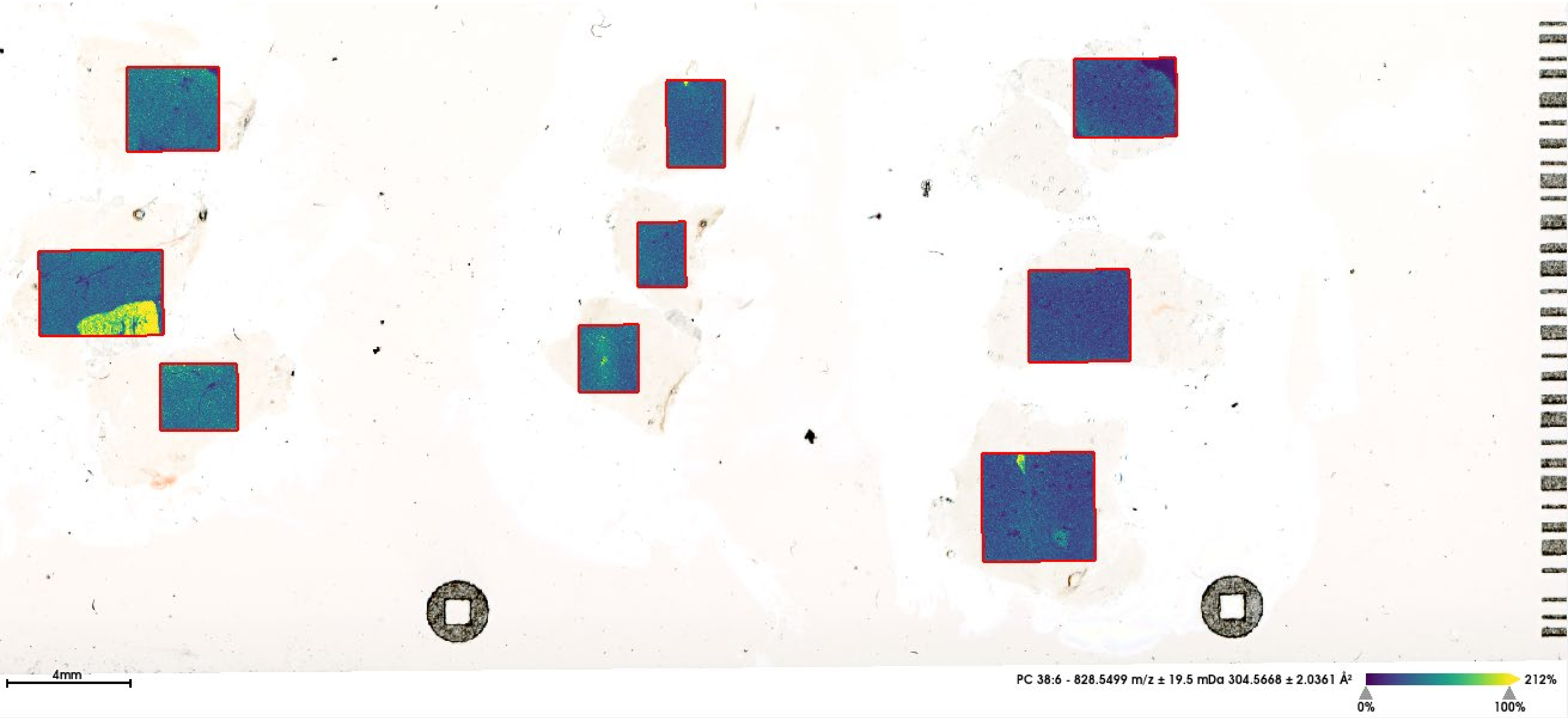
4mm
PC 38:6 - 828.5499 m/z ± 19.5 mDa 304.5668 ± 2.0361 Å²
212%
0%
100%

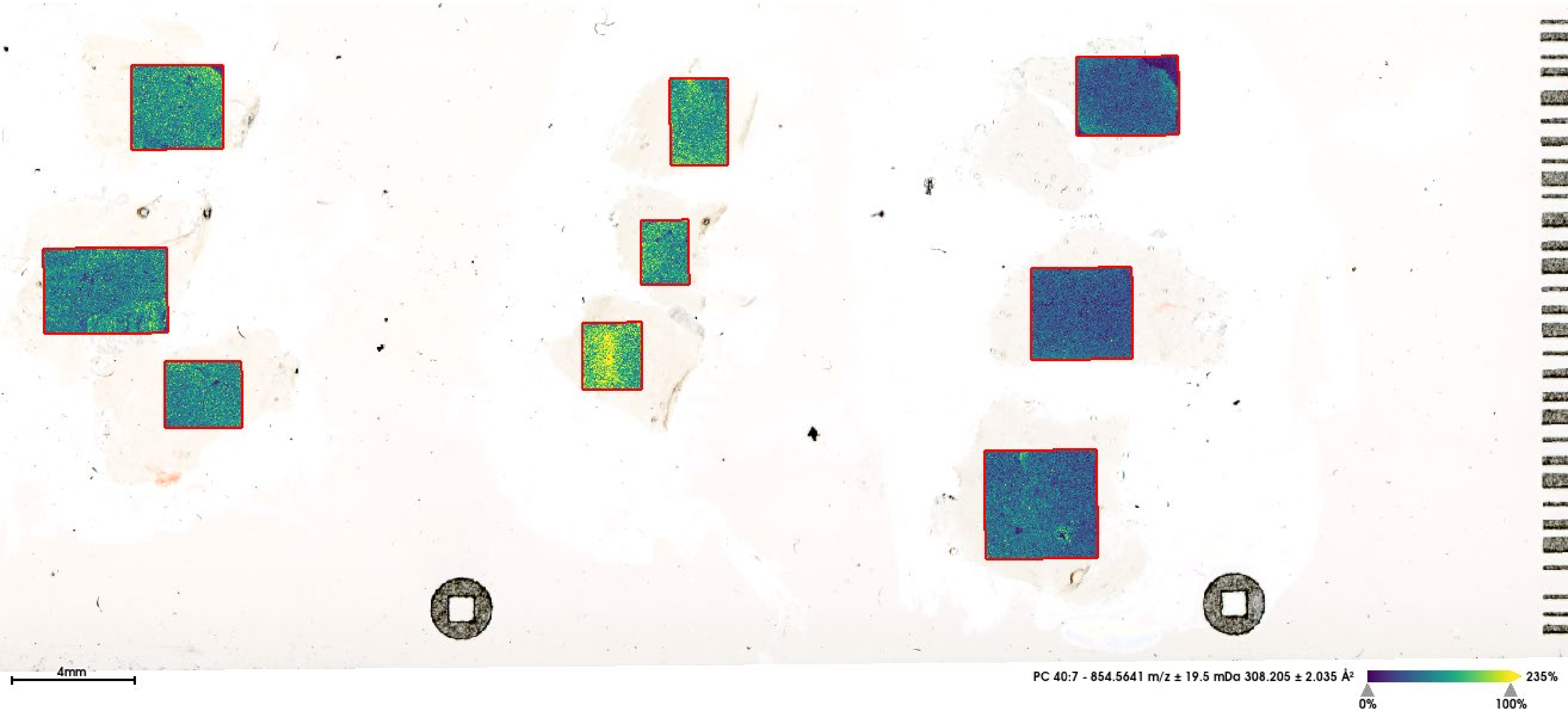
4mm
PC 40:7 - 854.5641 m/z ± 19.5 mDa 308.205 ± 2.035 Å²
235%
0%
100%

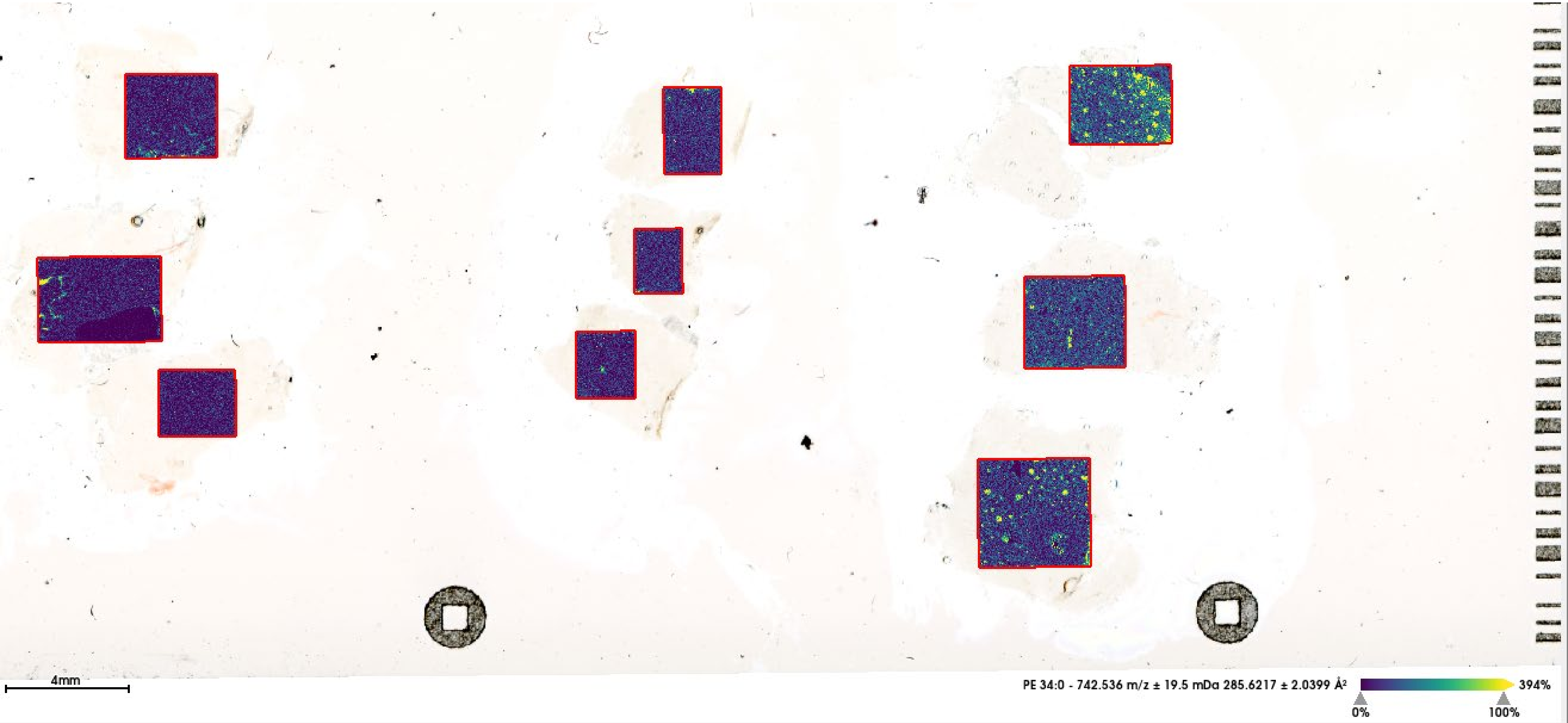
4mm
PE 34:0 - 742.536 m/z ± 19.5 mDa 285.6217 ± 2.0399 Å²
394%
0%
100%

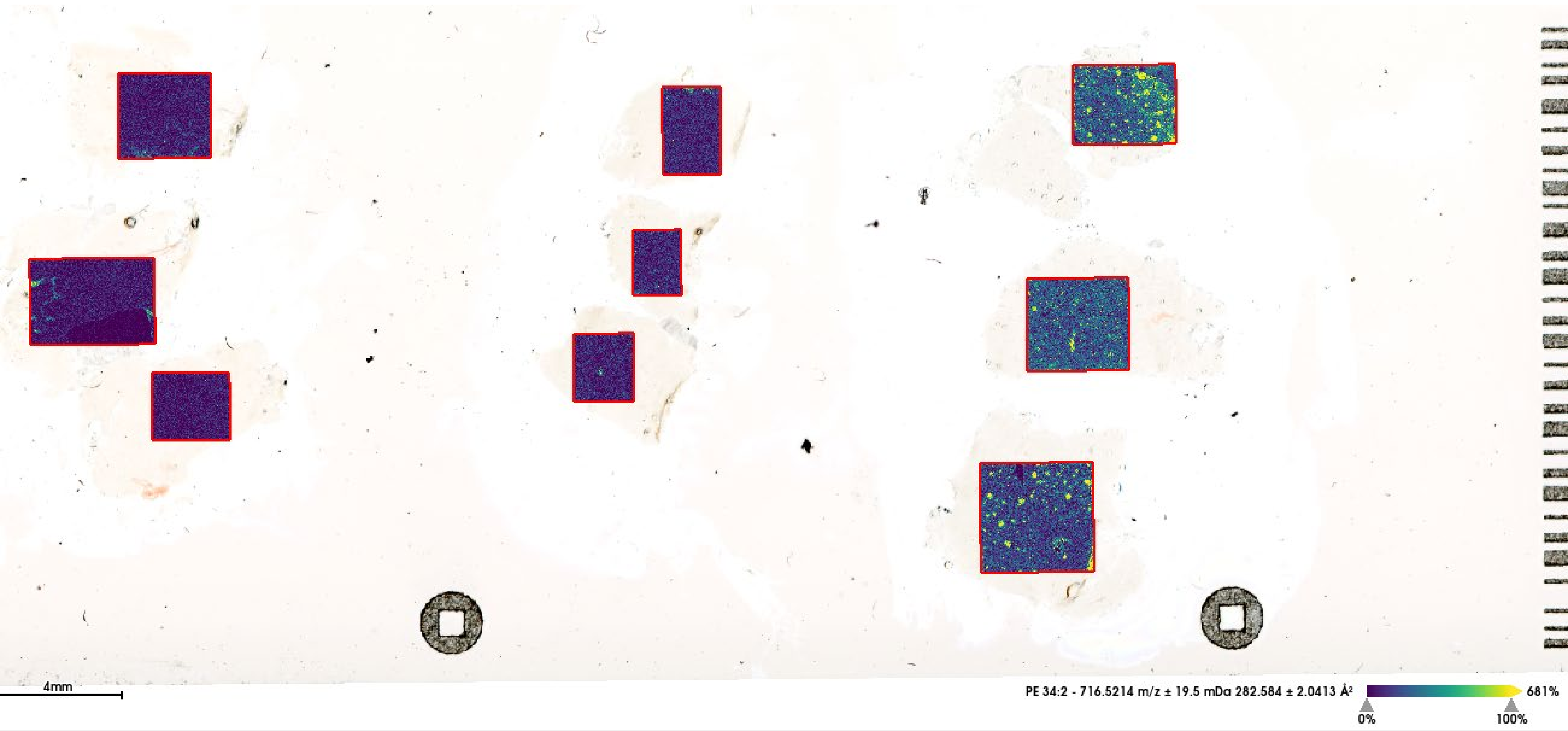

4mm
PE 34:2 - 716.5214 m/z ± 19.5 mDa 282.584 ± 2.0413 Å²
681%
0%
100%

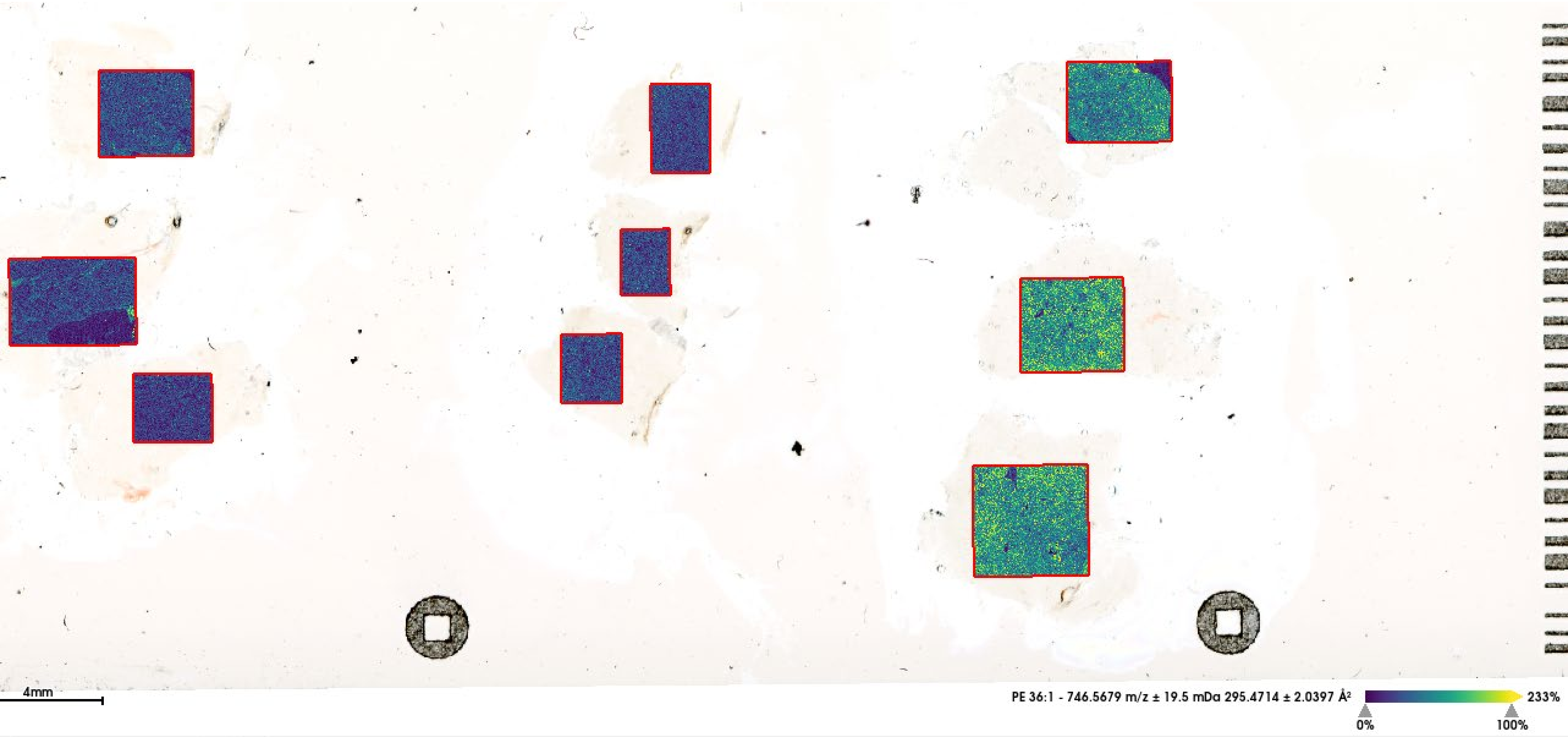
4mm
PE 36:1 - 746.5679 m/z ± 19.5 mDa 295.4714 ± 2.0397 Å²
233%
0%
100%

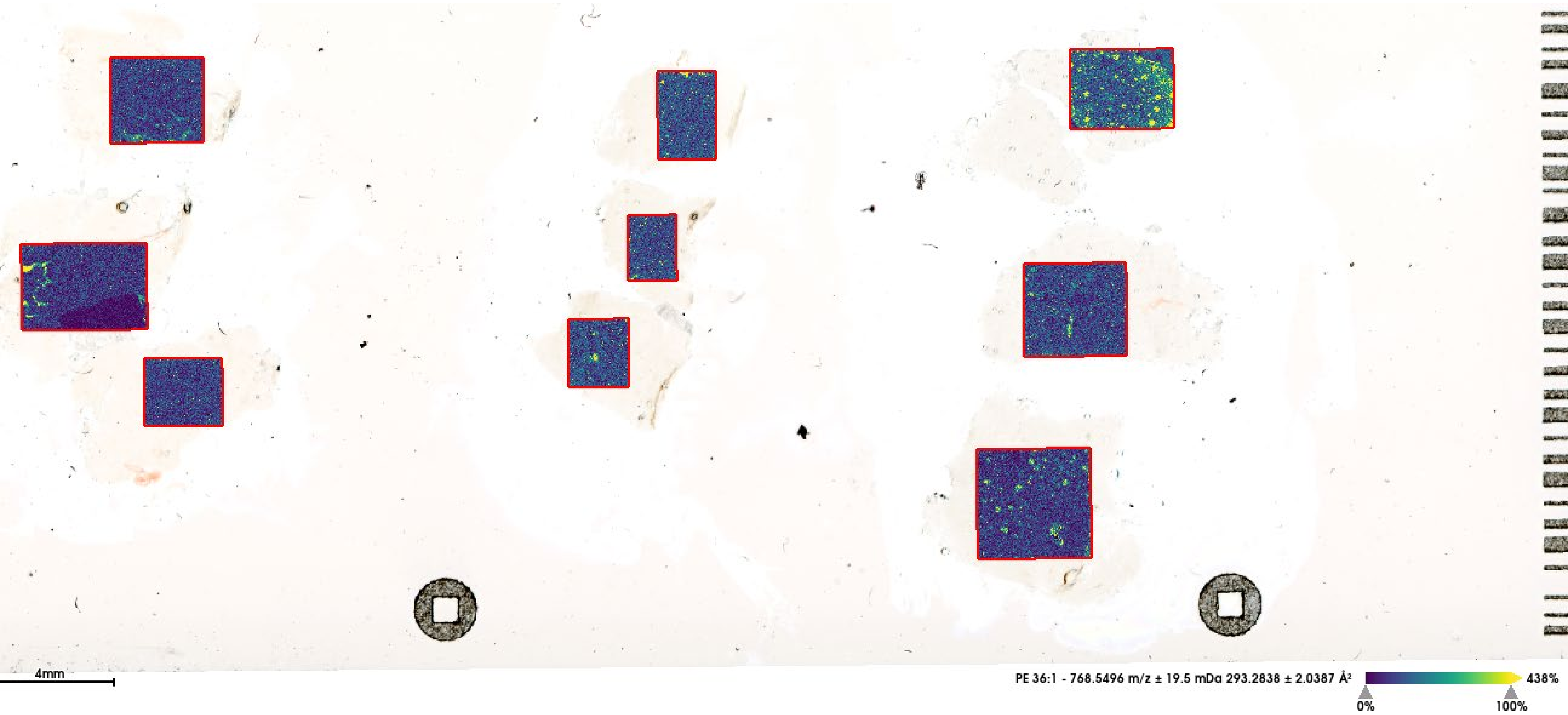
4mm
PE 36:1 - 768.5496 m/z ± 19.5 mDa 293.2838 ± 2.0387 Å²
438%
0%
100%

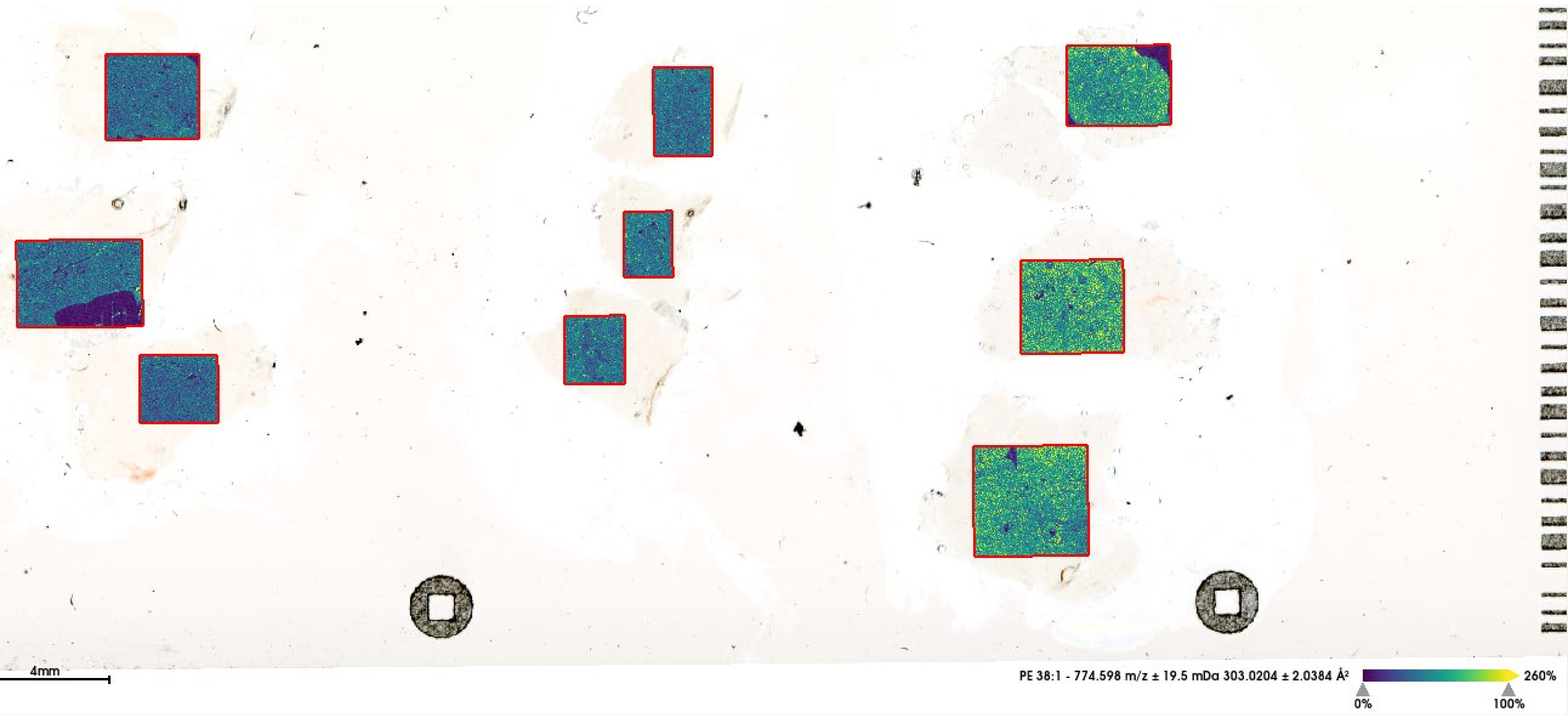
4mm
PE 38:1 - 774.598 m/z ± 19.5 mDa 303.0204 ± 2.0384 Å²
260%
0%
100%

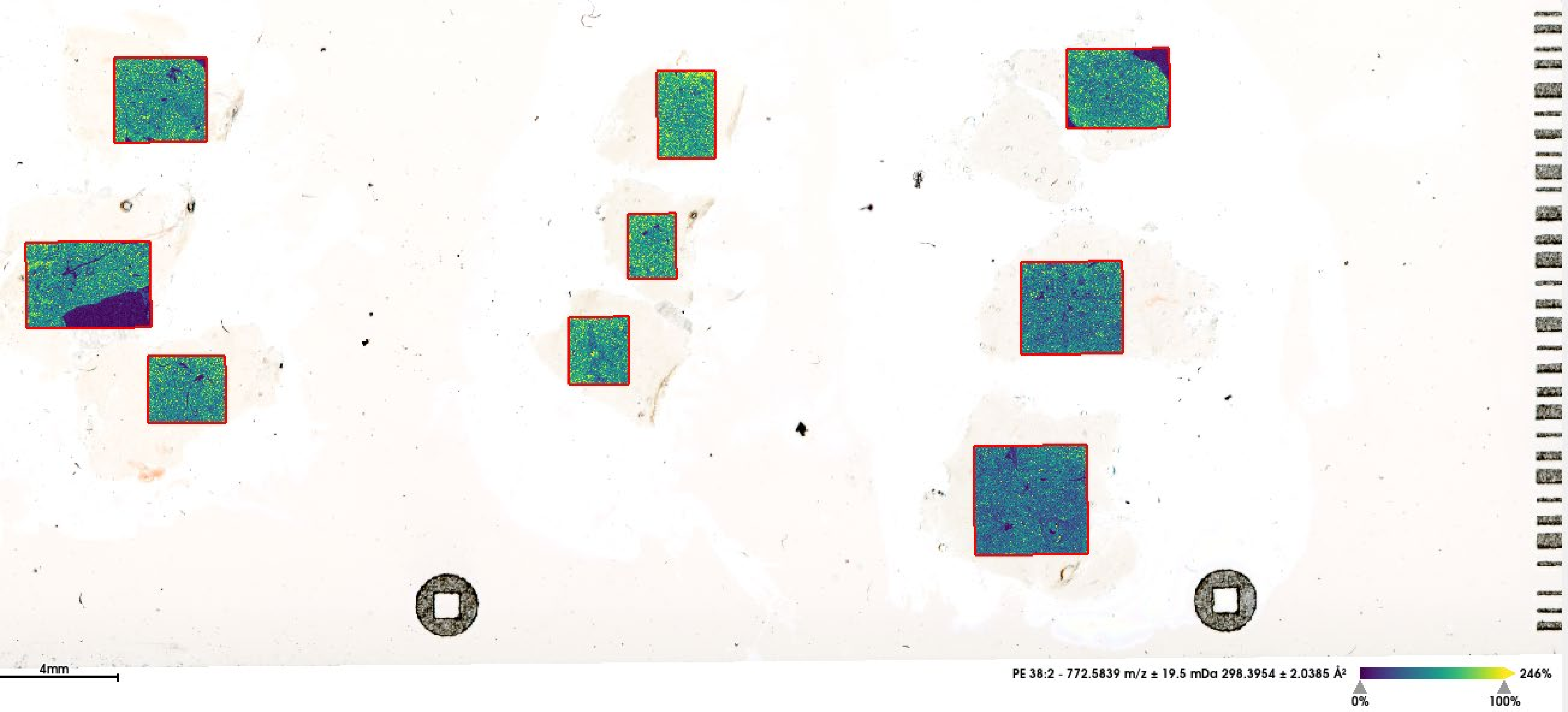
4mm
PE 38:2 - 772.5839 m/z ± 19.5 mDa 298.3954 ± 2.0385 Å²
246%
0%
100%

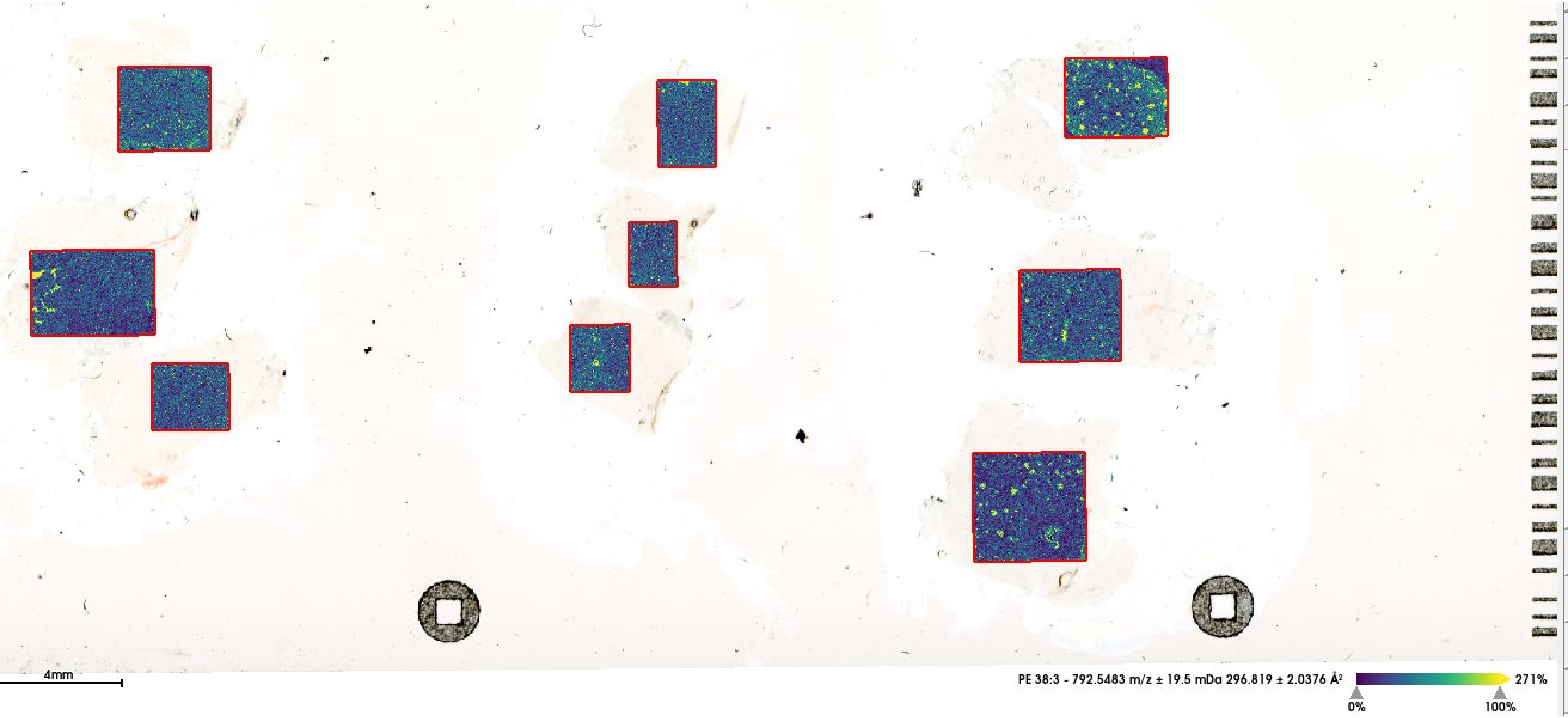

4mm
PE 38:3 - 792.5483 m/z ± 19.5 mDa 296.819 ± 2.0376 Å²
271%
0%
100%

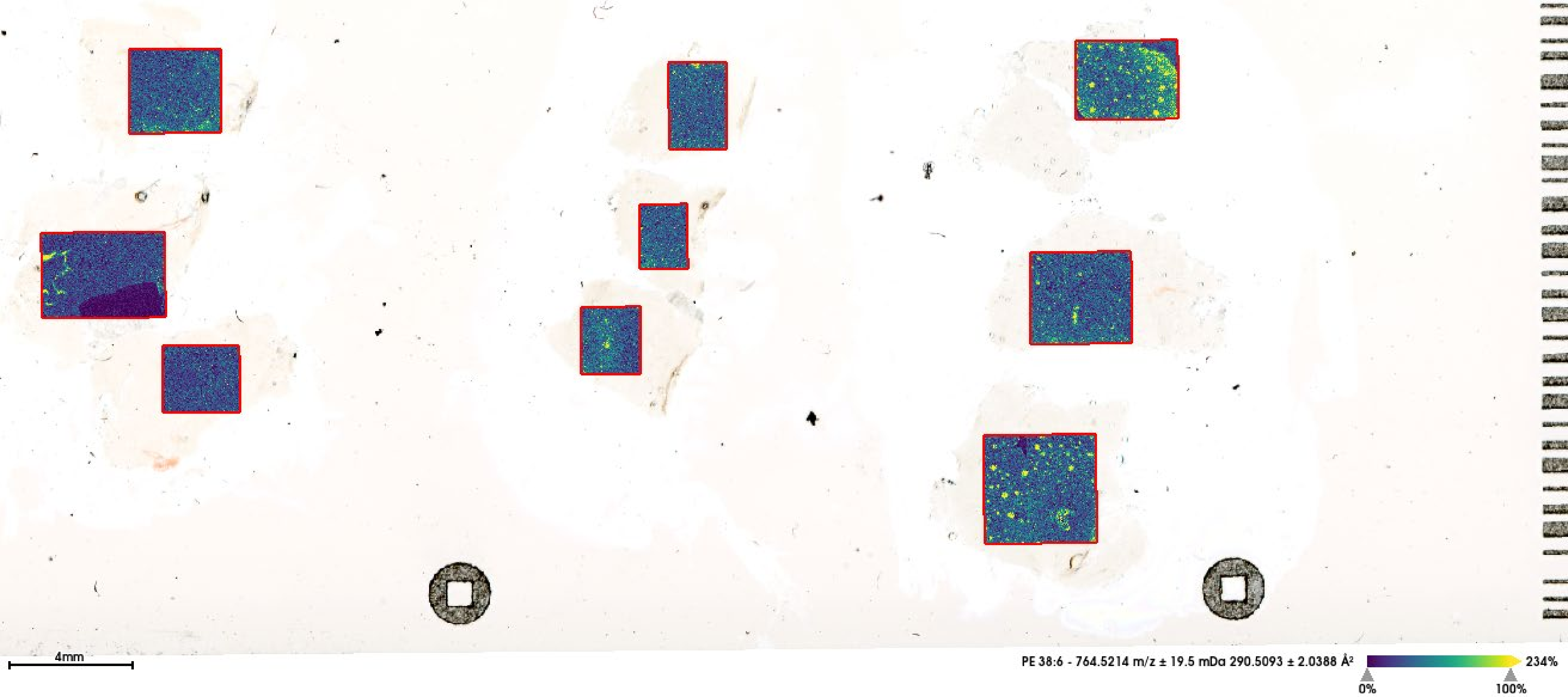
4mm
PE 38:6 - 764.5214 m/z ± 19.5 mDa 290.5093 ± 2.0388 Å²
234%
0%
100%

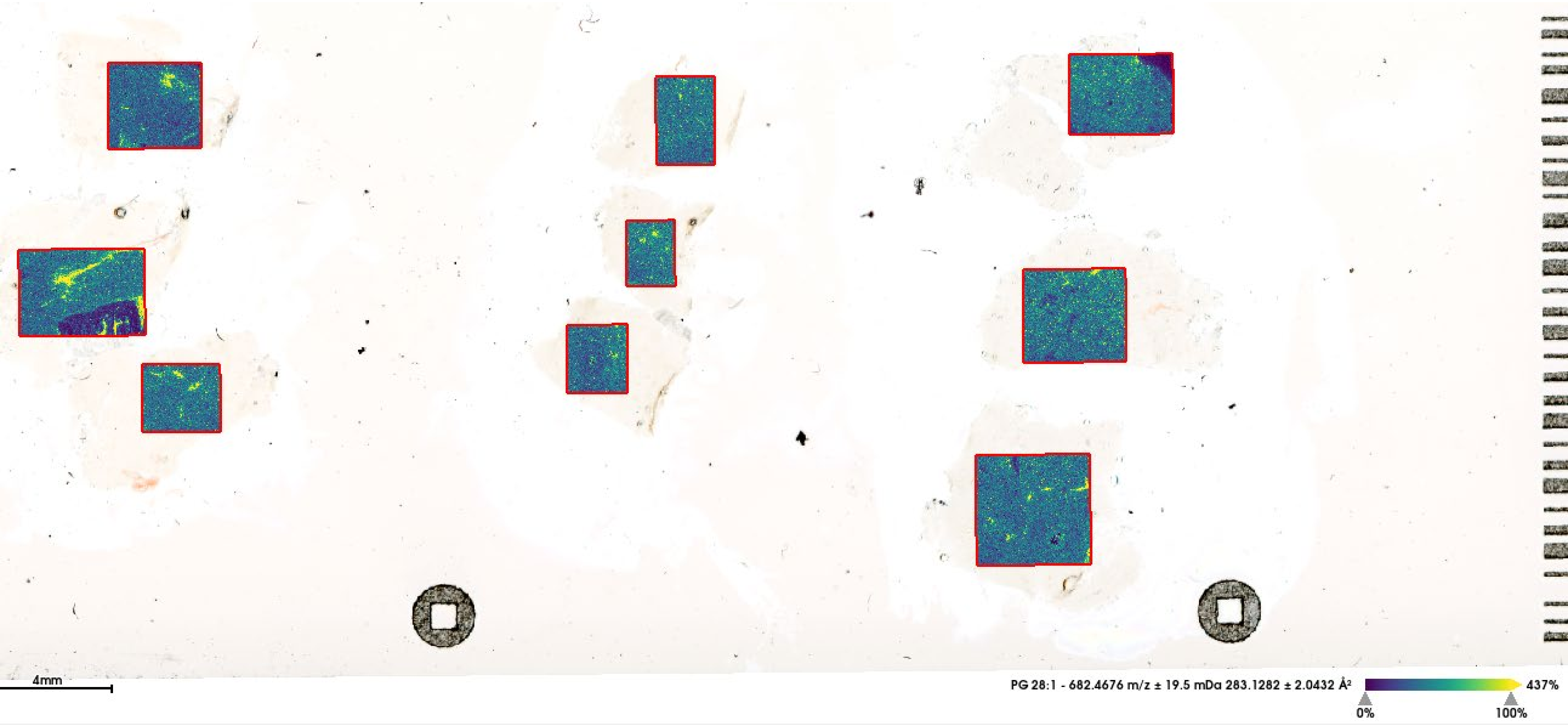
4mm
PG 28:1 - 682.4676 m/z ± 19.5 mDa 283.1282 ± 2.0432 Å²
437%
0%
100%

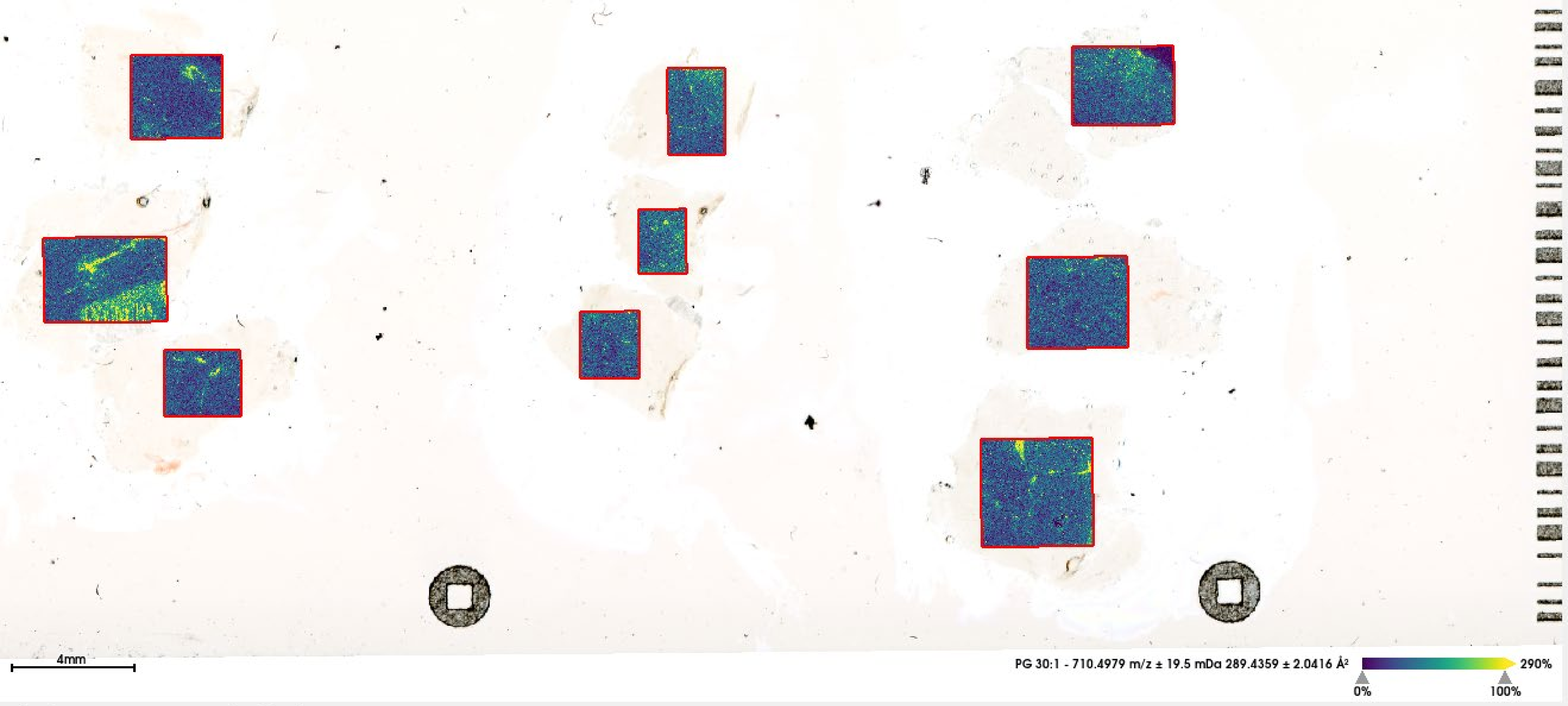
4mm
PG 30:1 - 710.4979 m/z ± 19.5 mDa 289.4359 ± 2.0416 Å²
290%
0%
100%

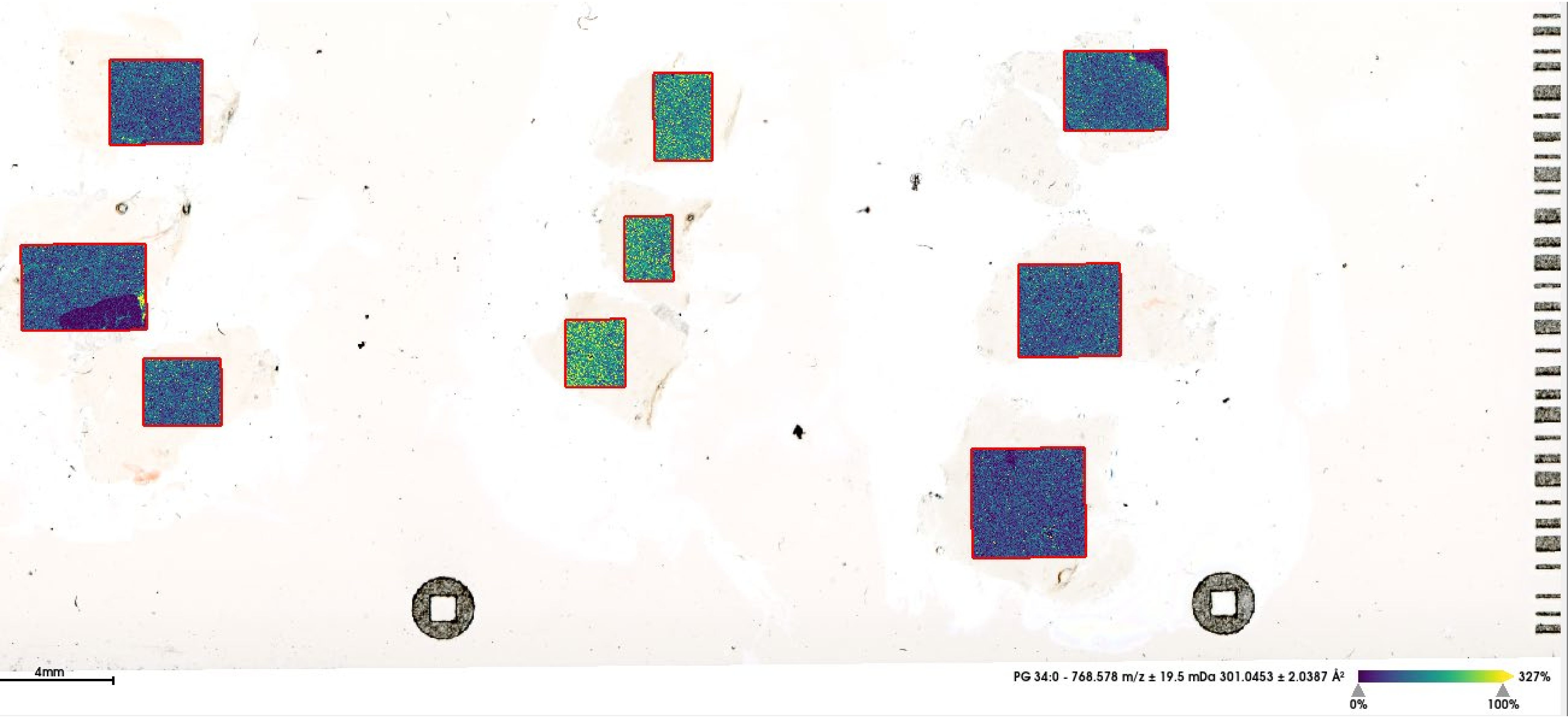

4mm
PG 34:0 - 768.578 m/z ± 19.5 mDa 301.0453 ± 2.0387 Å²
327%
0%
100%

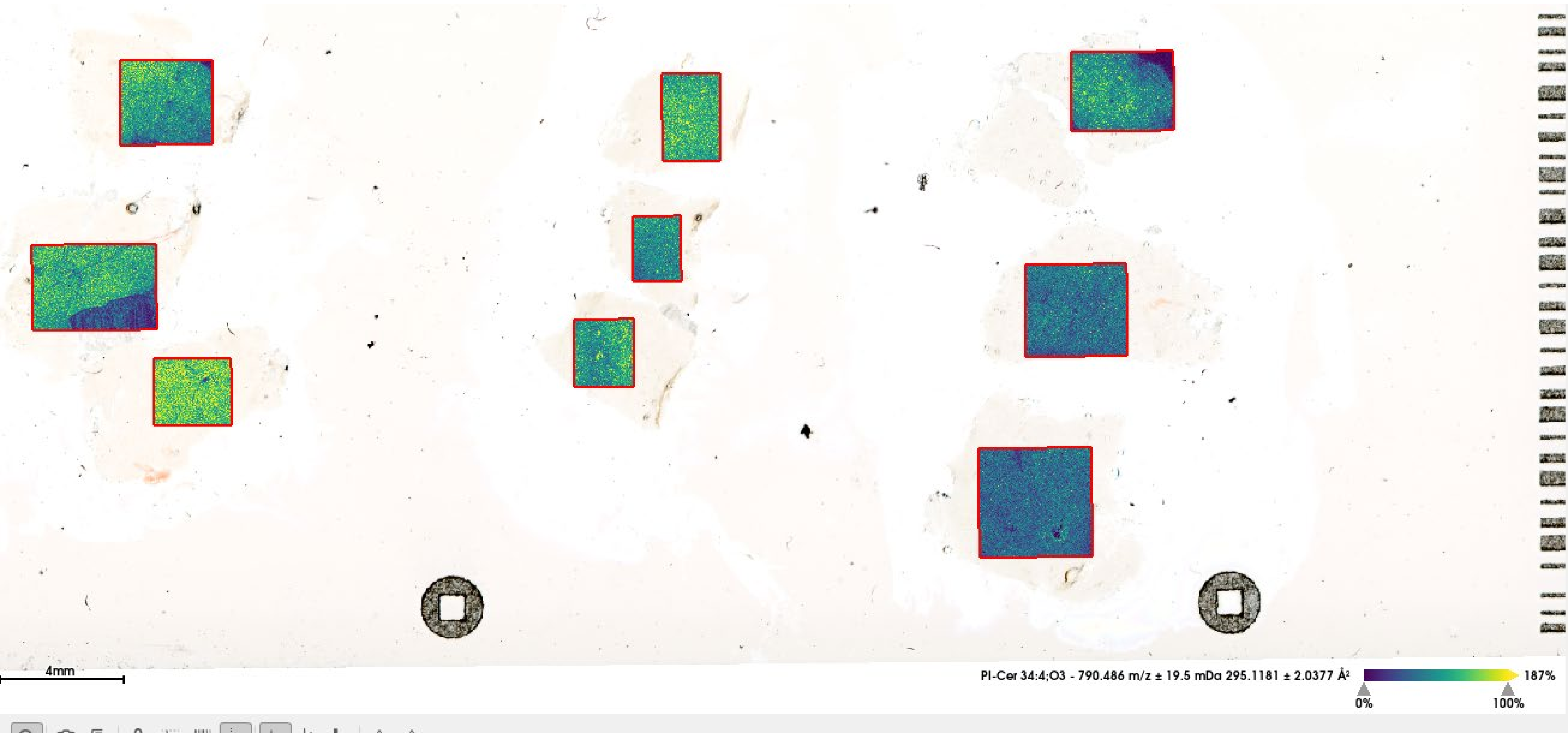
4mm
PI-Cer 34:4;O3 - 790.486 m/z ± 19.5 mDa 295.1181 ± 2.0377 Å²
187%
0%
100%

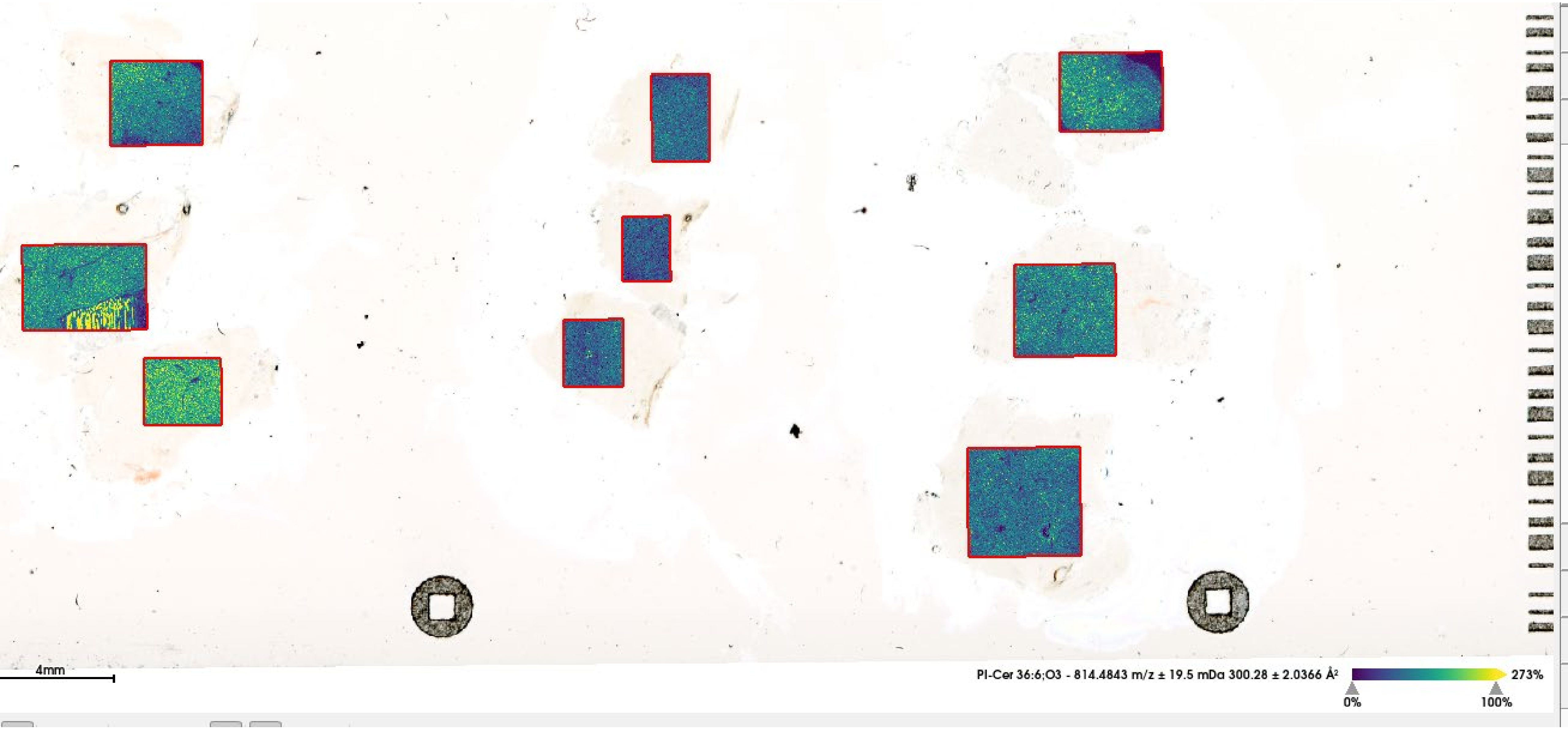
4mm
PI-Cer 36:6;O3 - 814.4843 m/z ± 19.5 mDa 300.28 ± 2.0366 Å²
273%
0%
100%

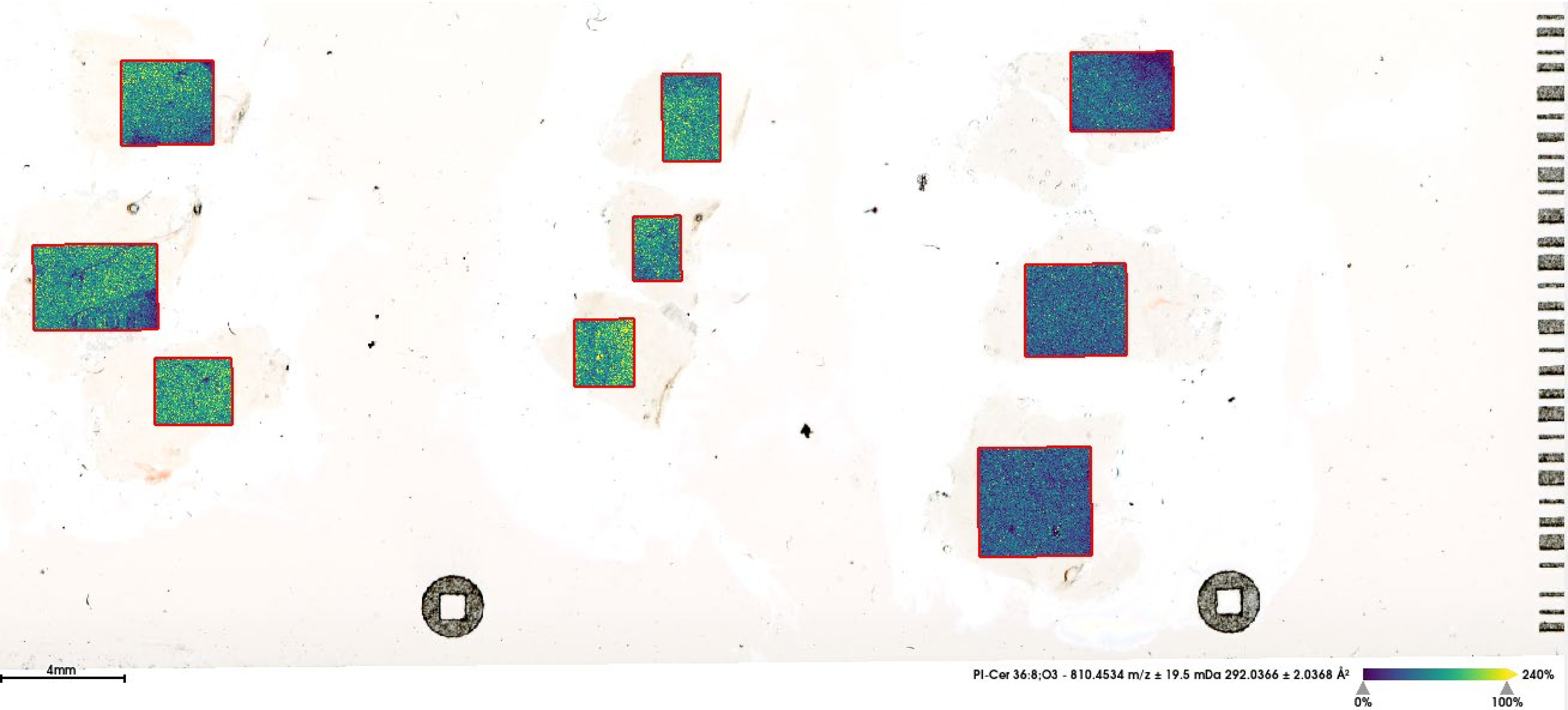
4mm
PI-Cer 36:8;O3 - 810.4534 m/z ± 19.5 mDa 292.0366 ± 2.0368 Å²
240%
0%
100%

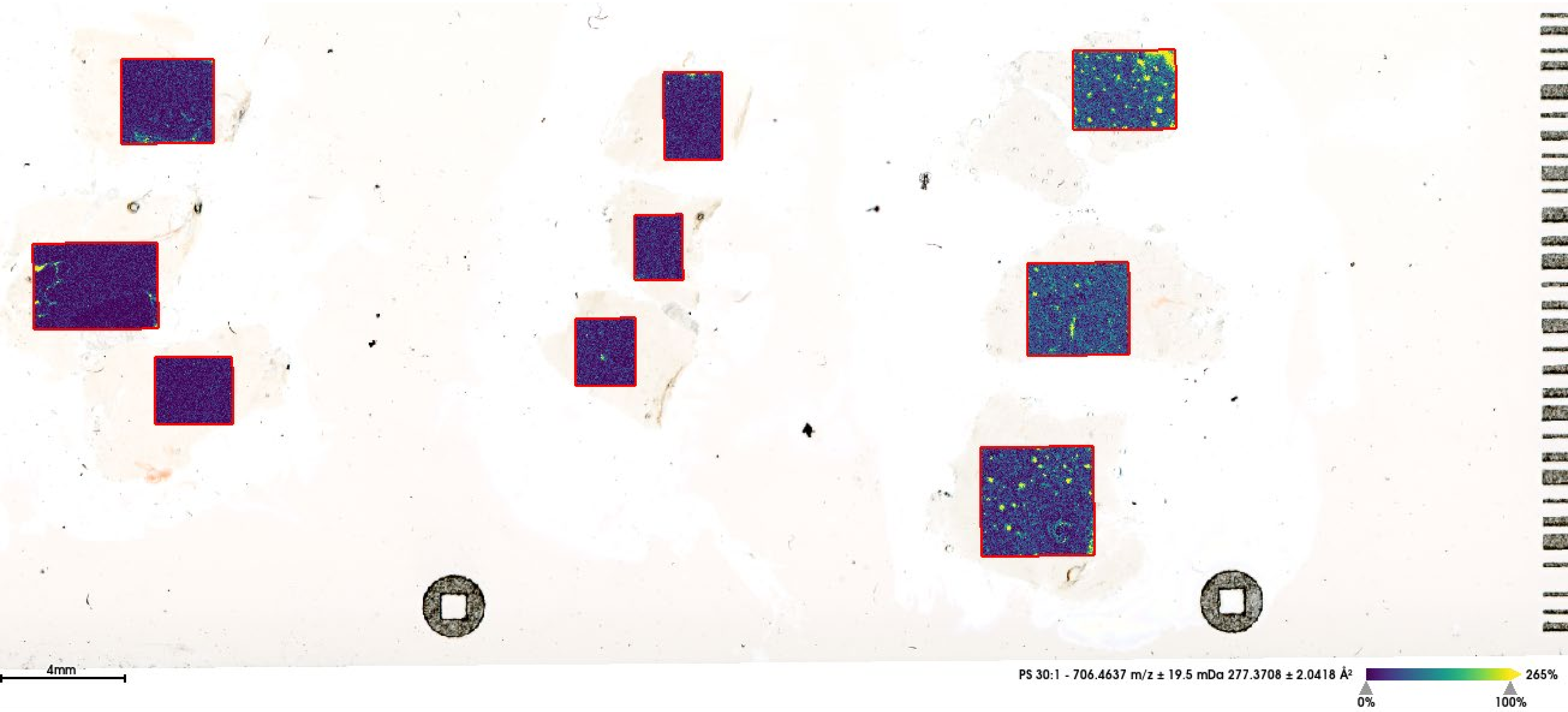
4mm
PS 30:1 - 706.4637 m/z ± 19.5 mDa 277.3708 ± 2.0418 Å²
265%
0%
100%

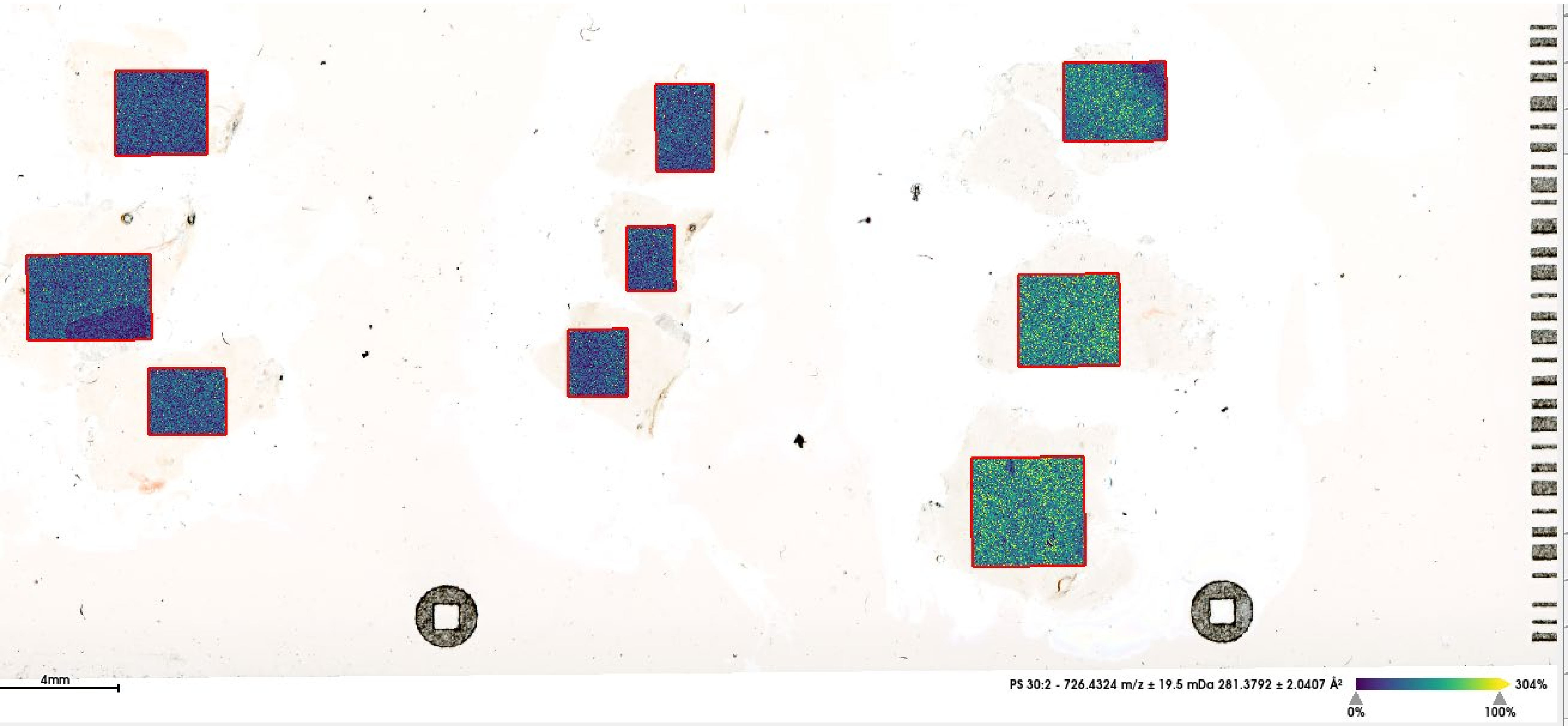
4mm
PS 30:2 - 726.4324 m/z ± 19.5 mDa 281.3792 ± 2.0407 Å²
304%
0%
100%

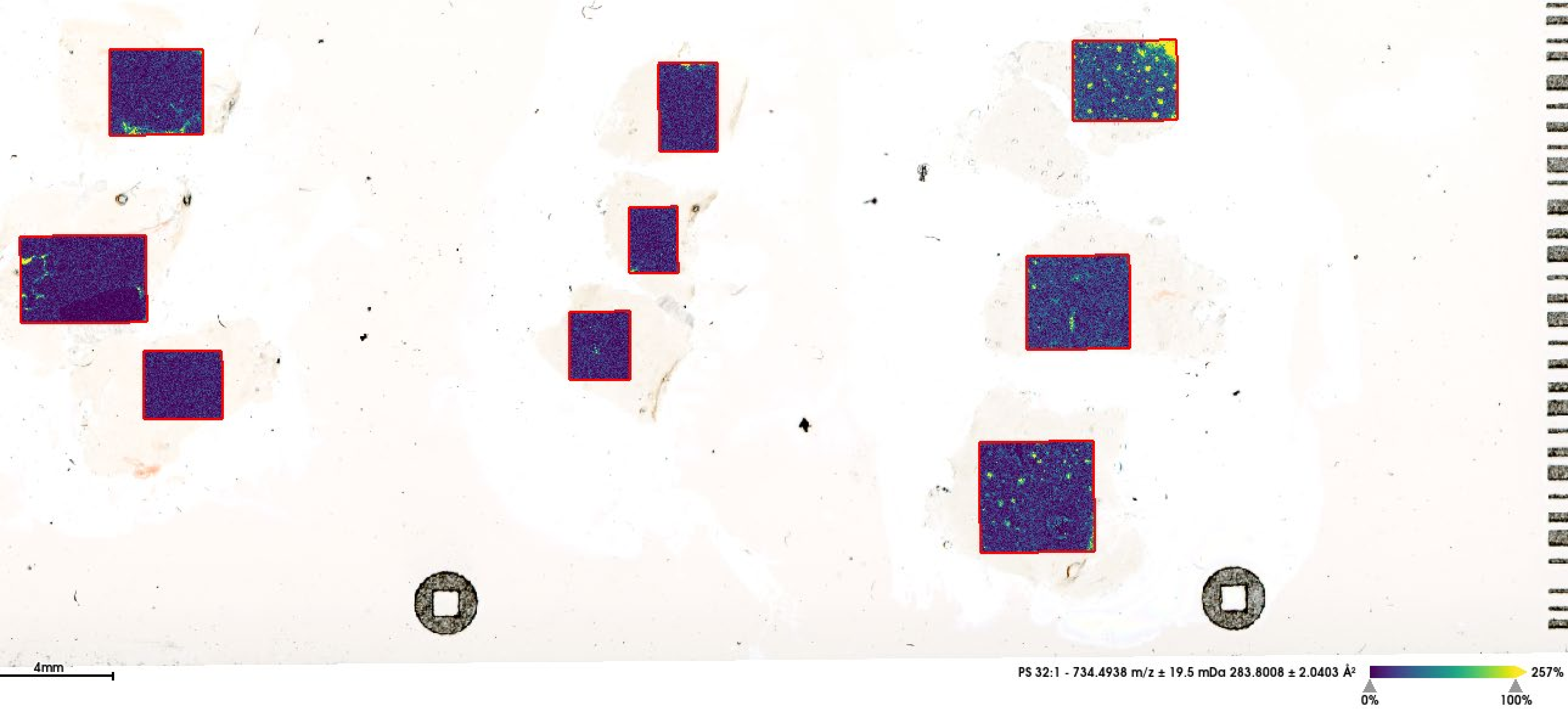

4mm
PS 32:1 - 734.4938 m/z ± 19.5 mDa 283.8008 ± 2.0403 Å²
257%
0%
100%

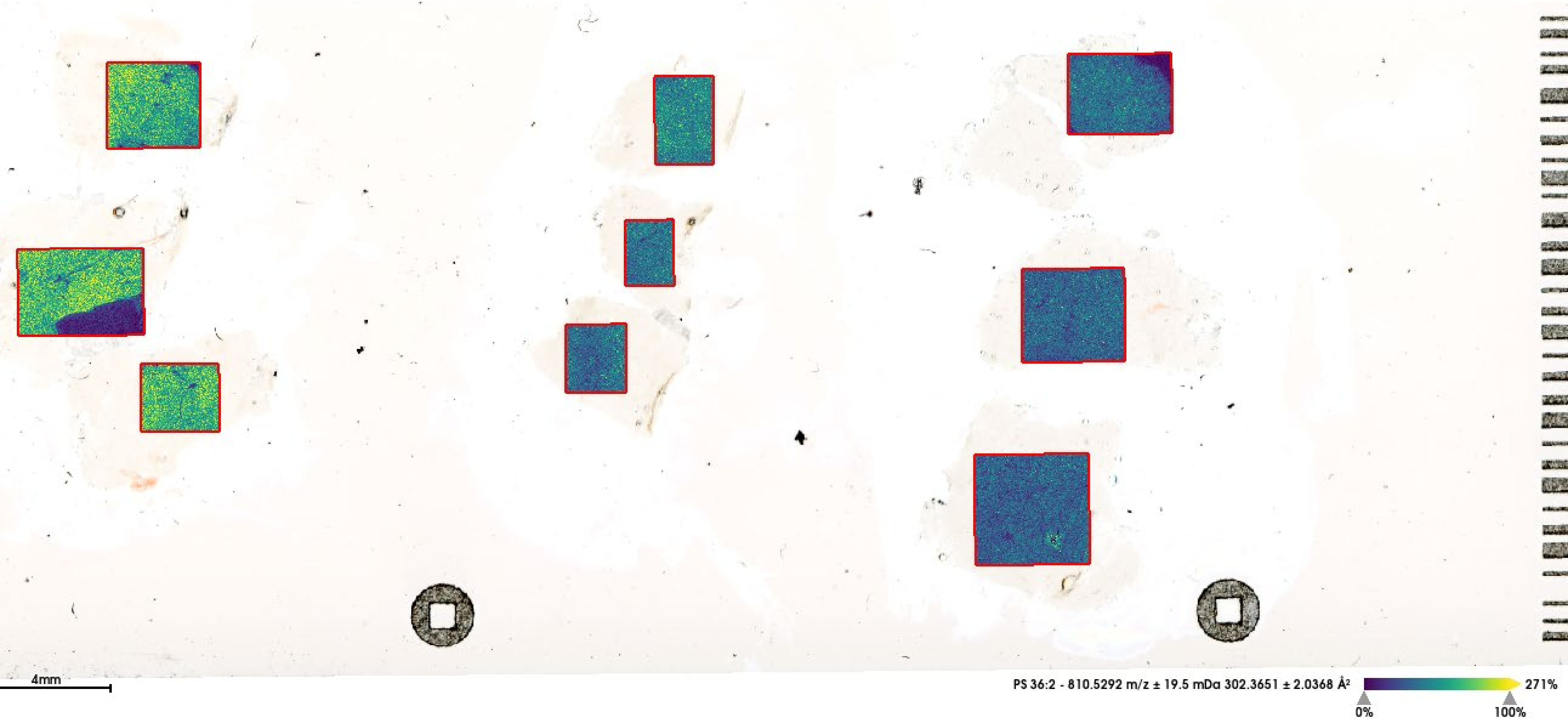

4mm
PS 36:2 - 810.5292 m/z ± 19.5 mDa 302.3651 ± 2.0368 Å²
271%
0%
100%

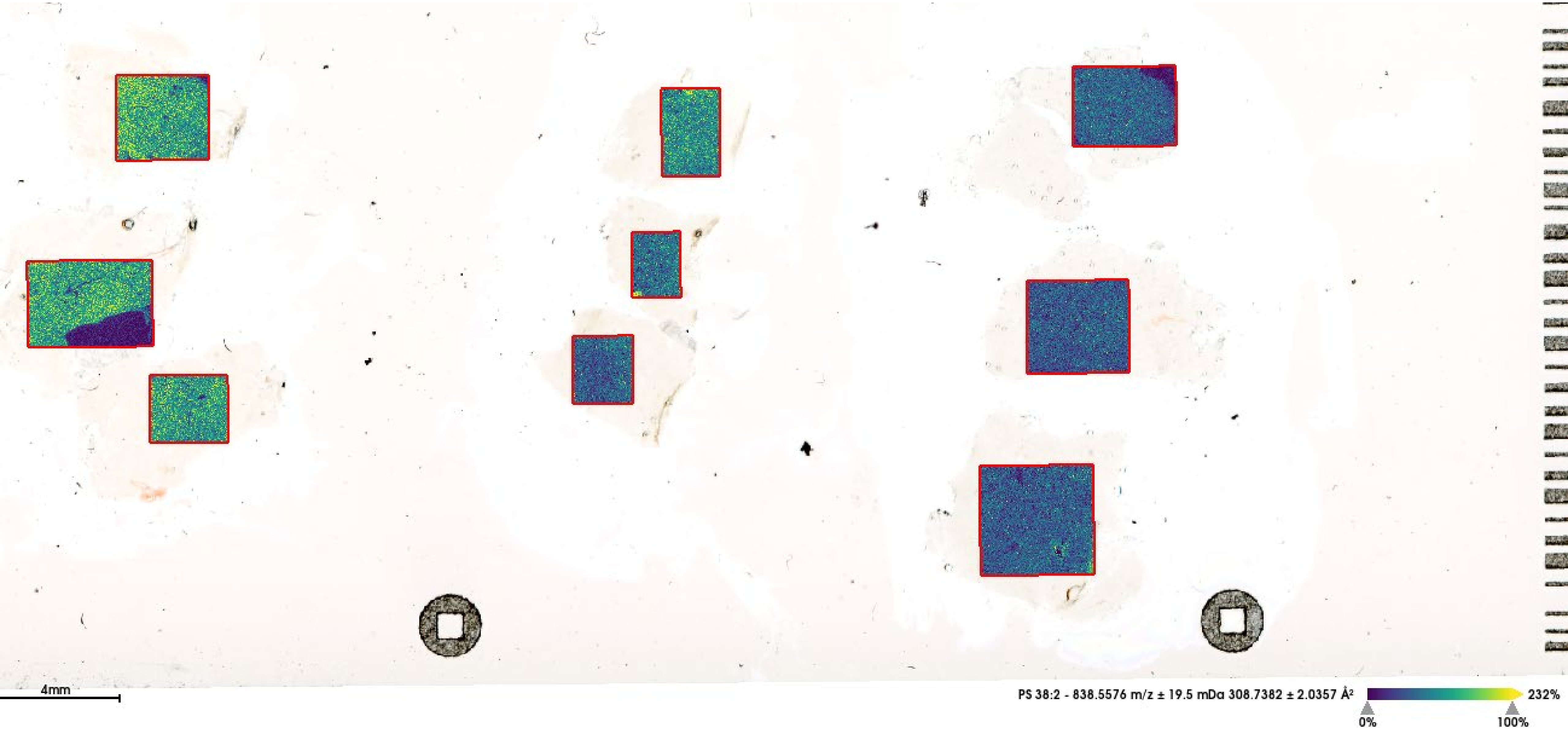

4mm
PS 38:2 - 838.5576 m/z ± 19.5 mDa 308.7382 ± 2.0357 Å²
232%
0%
100%

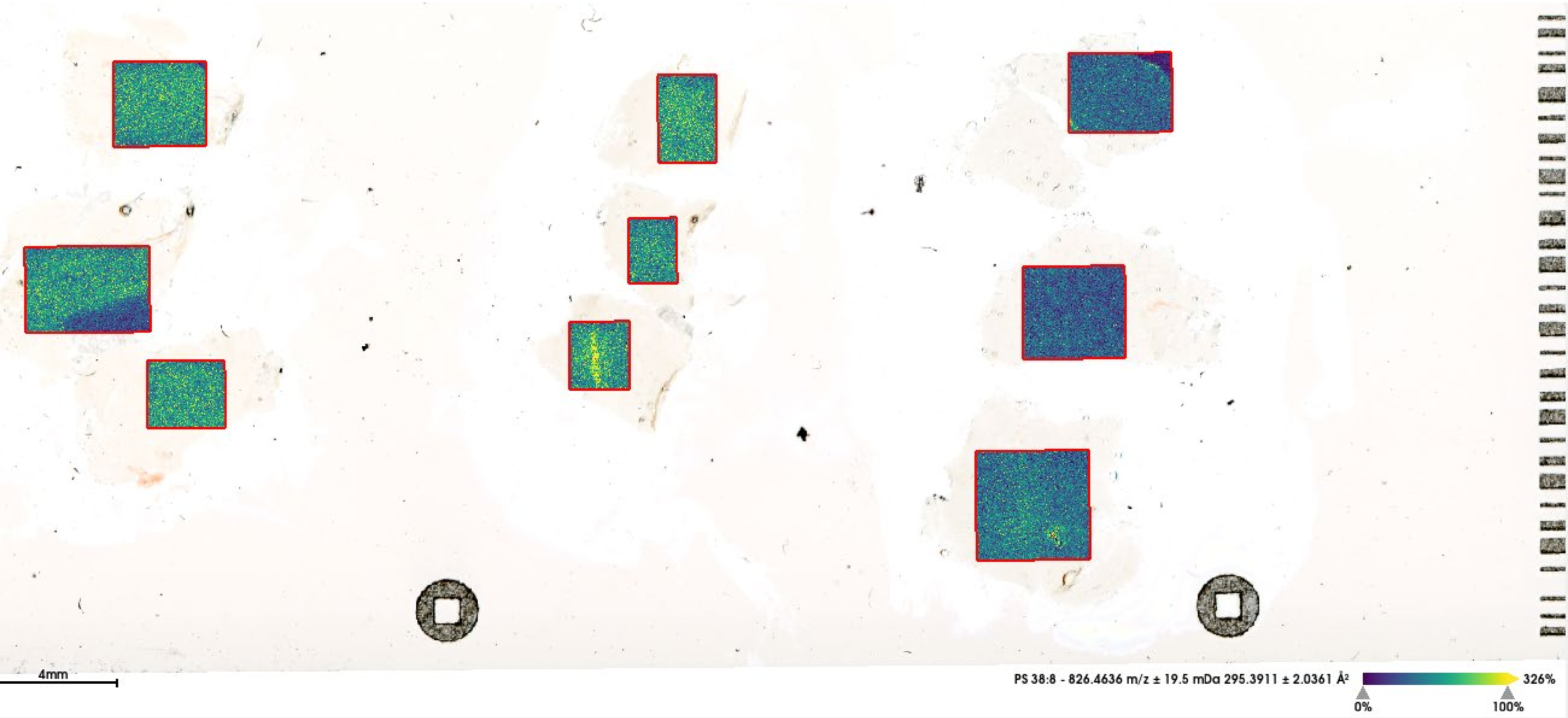

4mm
PS 38:8 - 826.4636 m/z ± 19.5 mDa 295.3911 ± 2.0361 Å²
326%
0%
100%

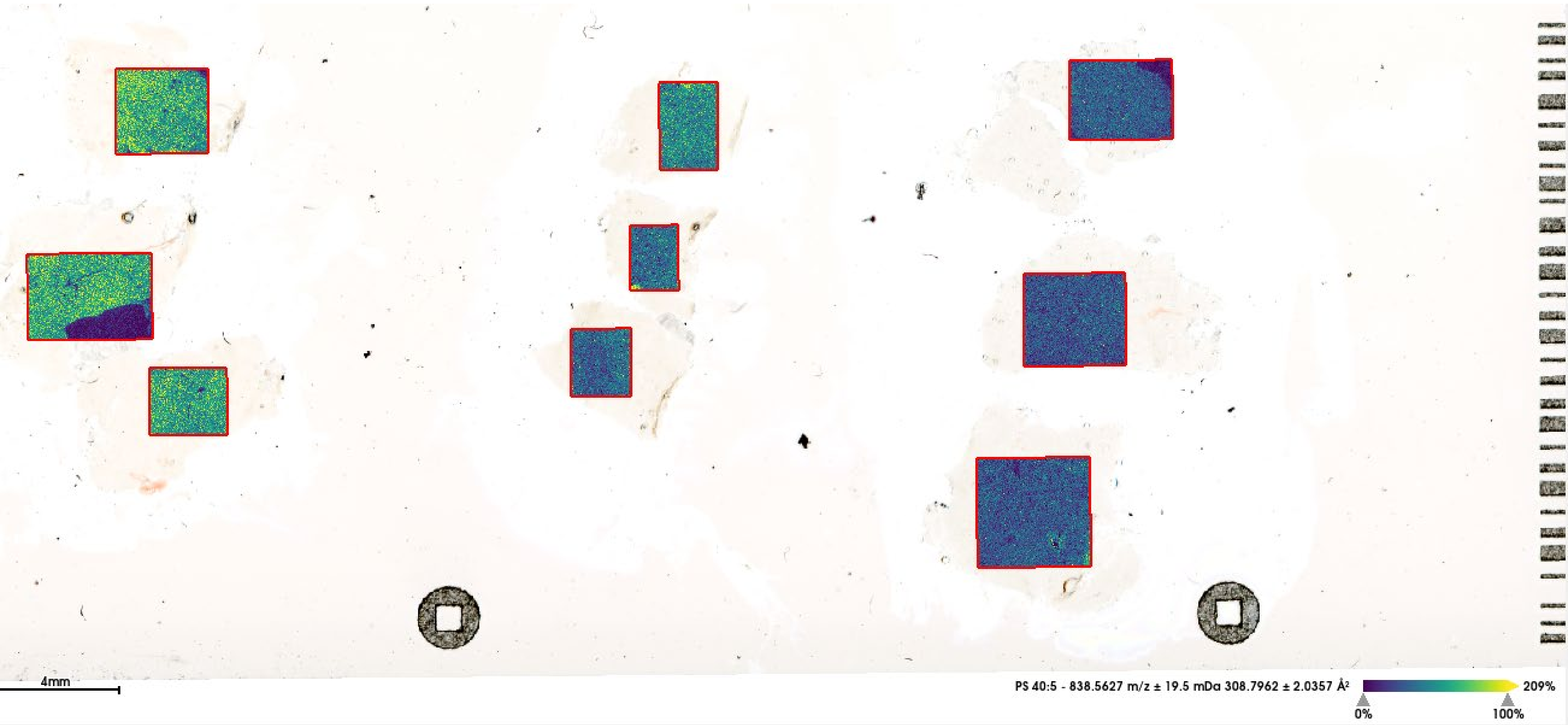

4mm
PS 40:5 - 838.5627 m/z ± 19.5 mDa 308.7962 ± 2.0357 Å²
209%
0%
100%

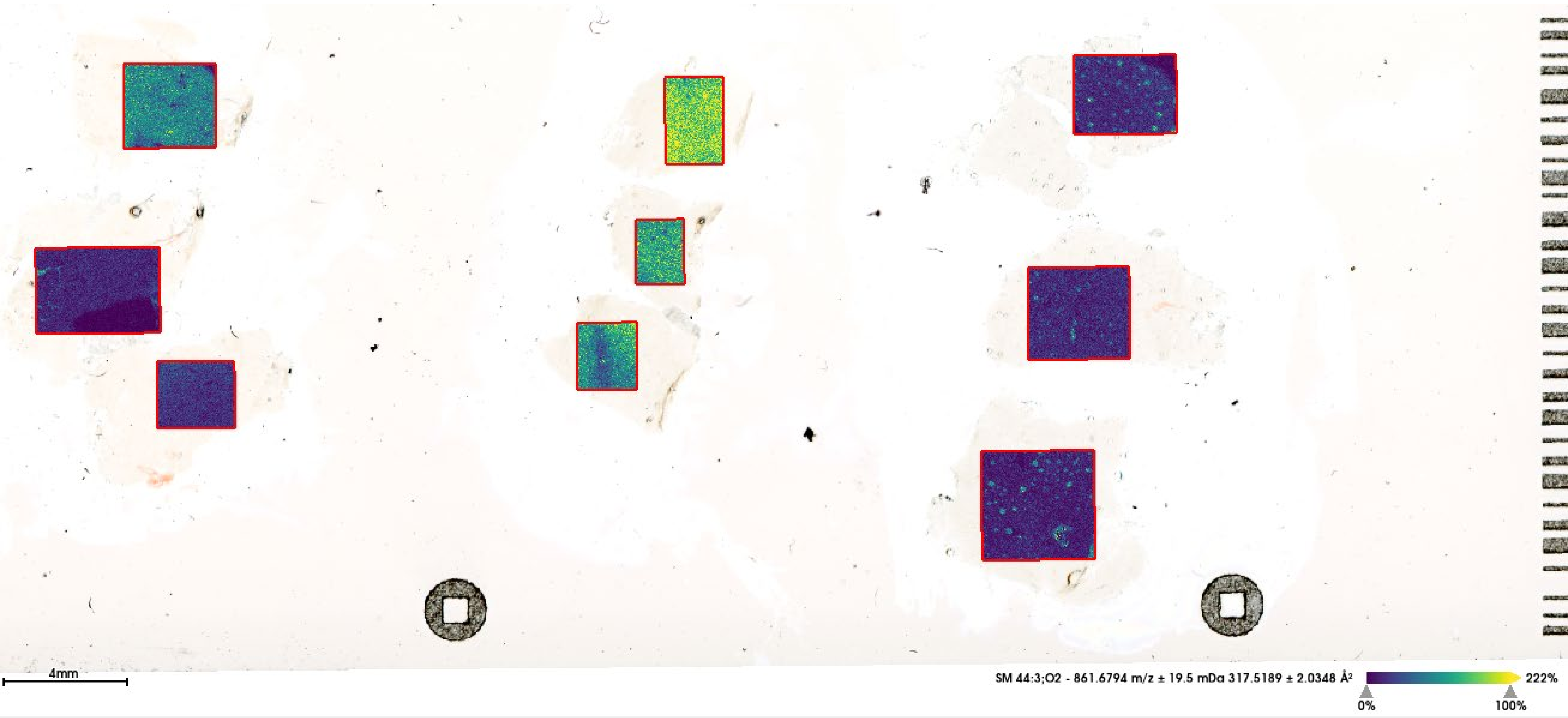
4mm
SM 44:3;O2 - 861.6794 m/z ± 19.5 mDa 317.5189 ± 2.0348 Å²
222%
0%
100%

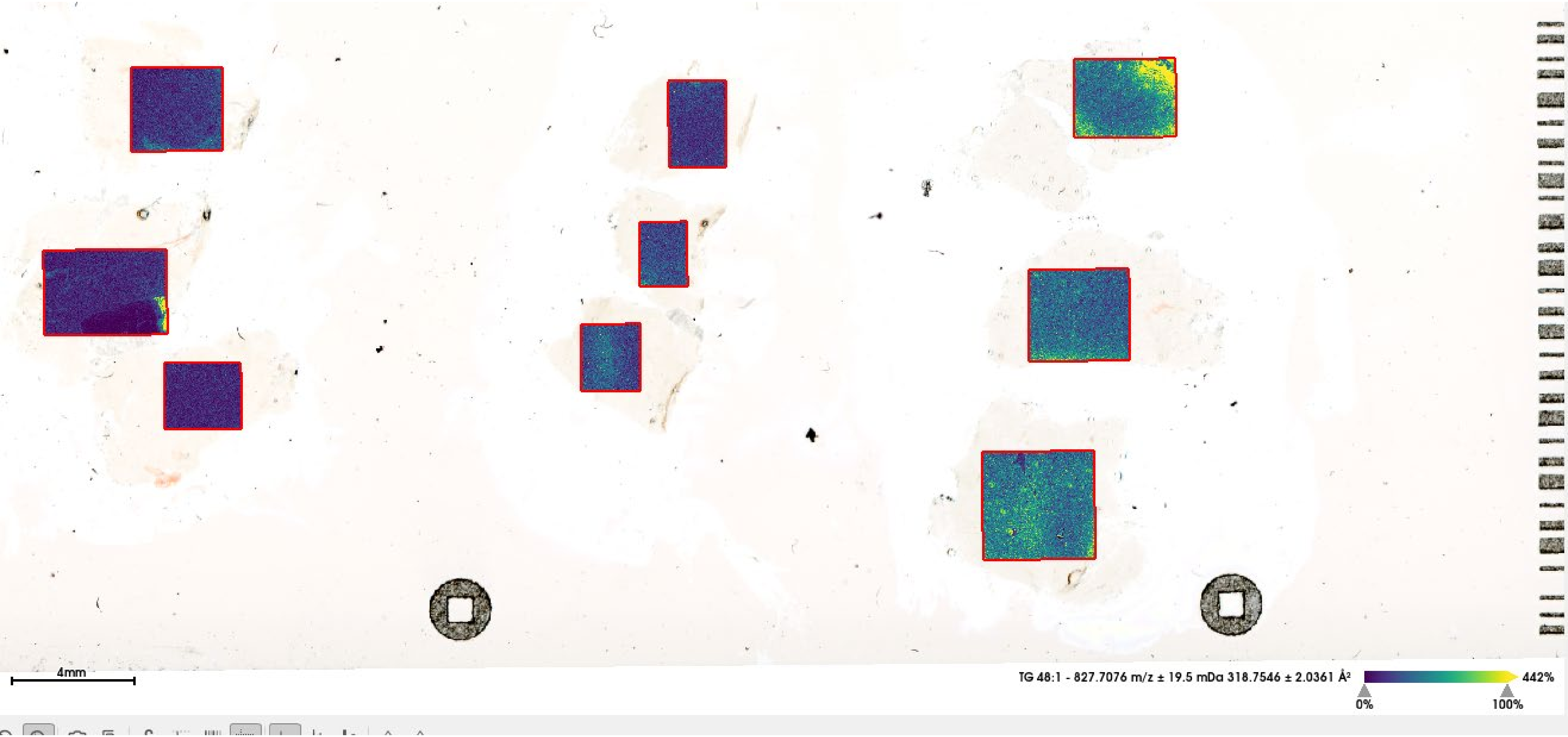
4mm
TG 48:1 - 827.7076 m/z ± 19.5 mDa 318.7546 ± 2.0361 Å²
442%
0%
100%

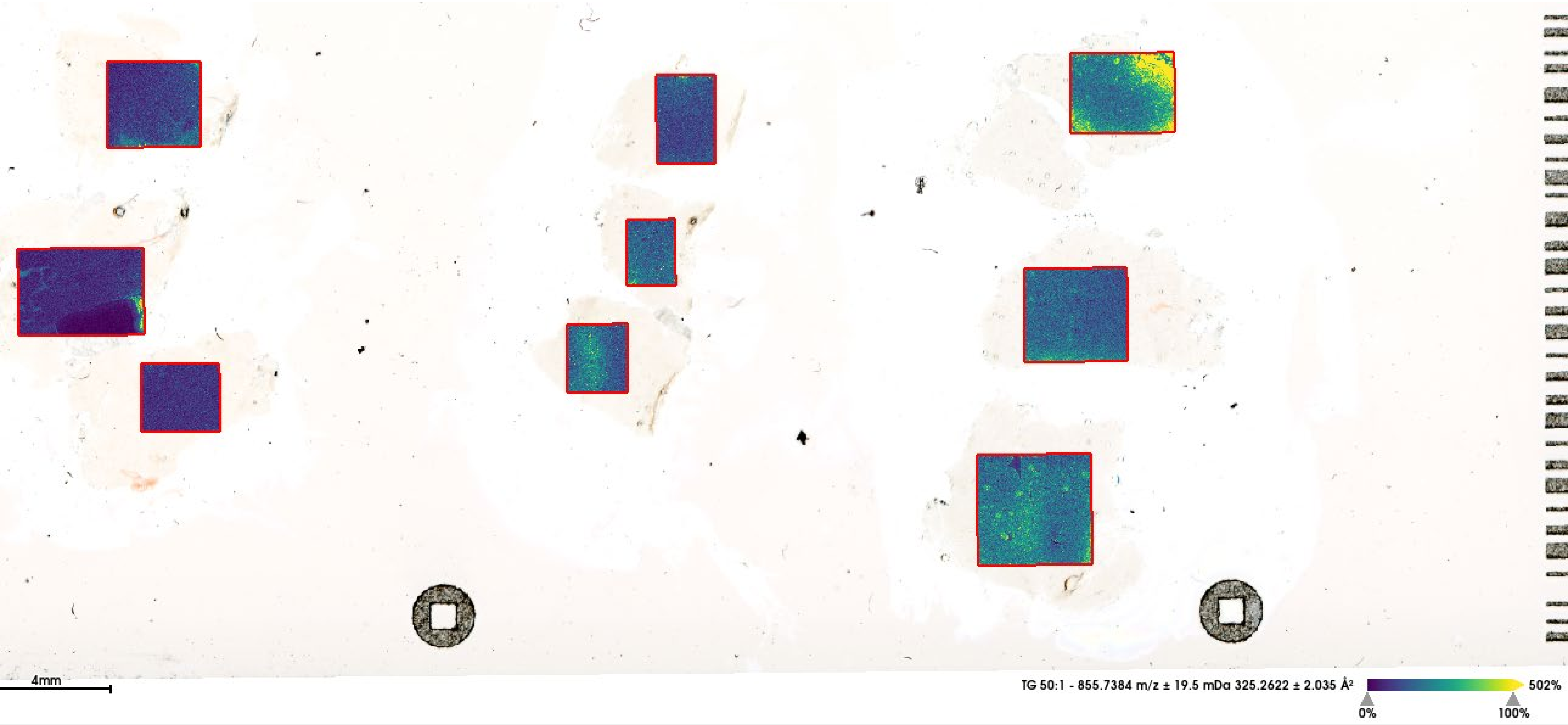

4mm
TG 50:1 - 855.7384 m/z ± 19.5 mDa 325.2622 ± 2.035 Å²
502%
0%
100%

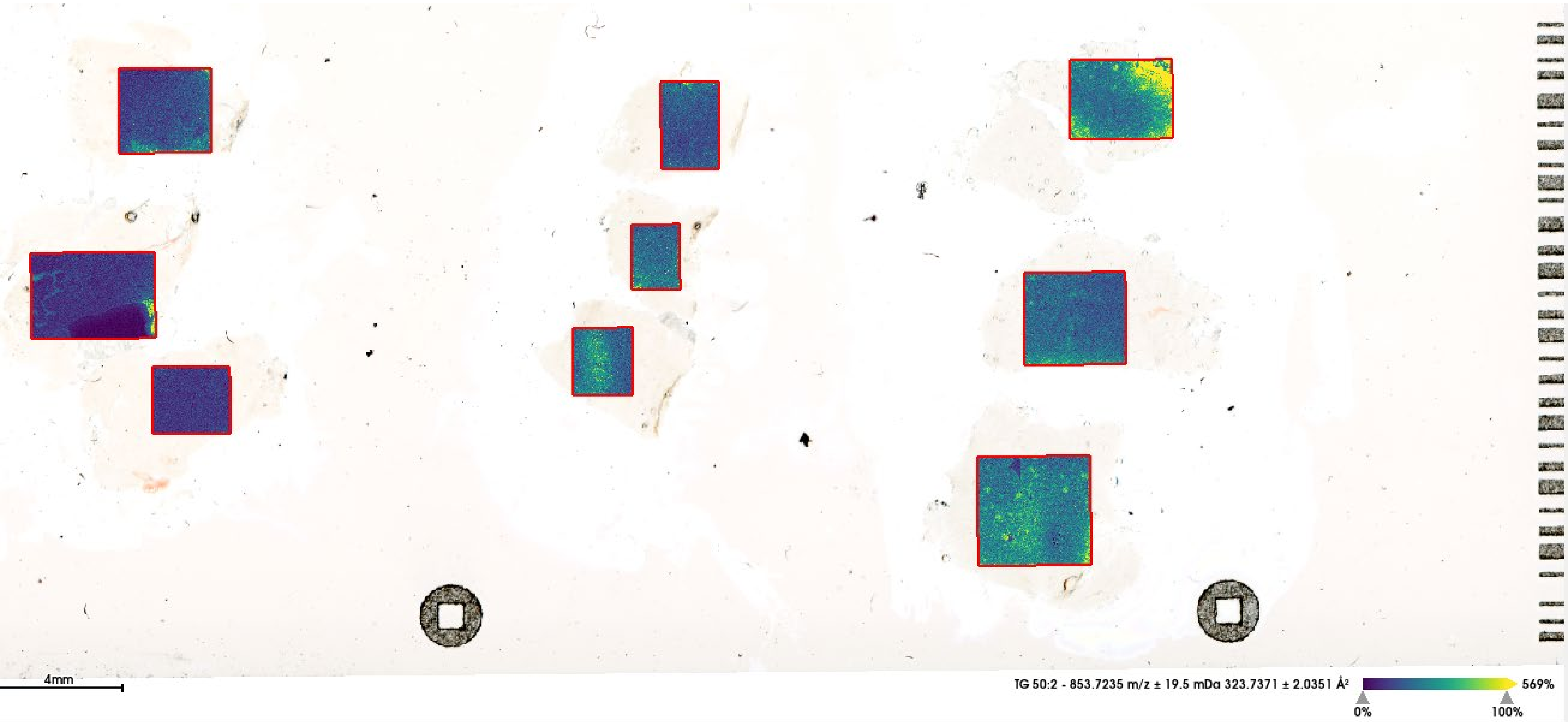
4mm
TG 50:2 - 853.7235 m/z ± 19.5 mDa 323.7371 ± 2.0351 Å²
569%
0%
100%

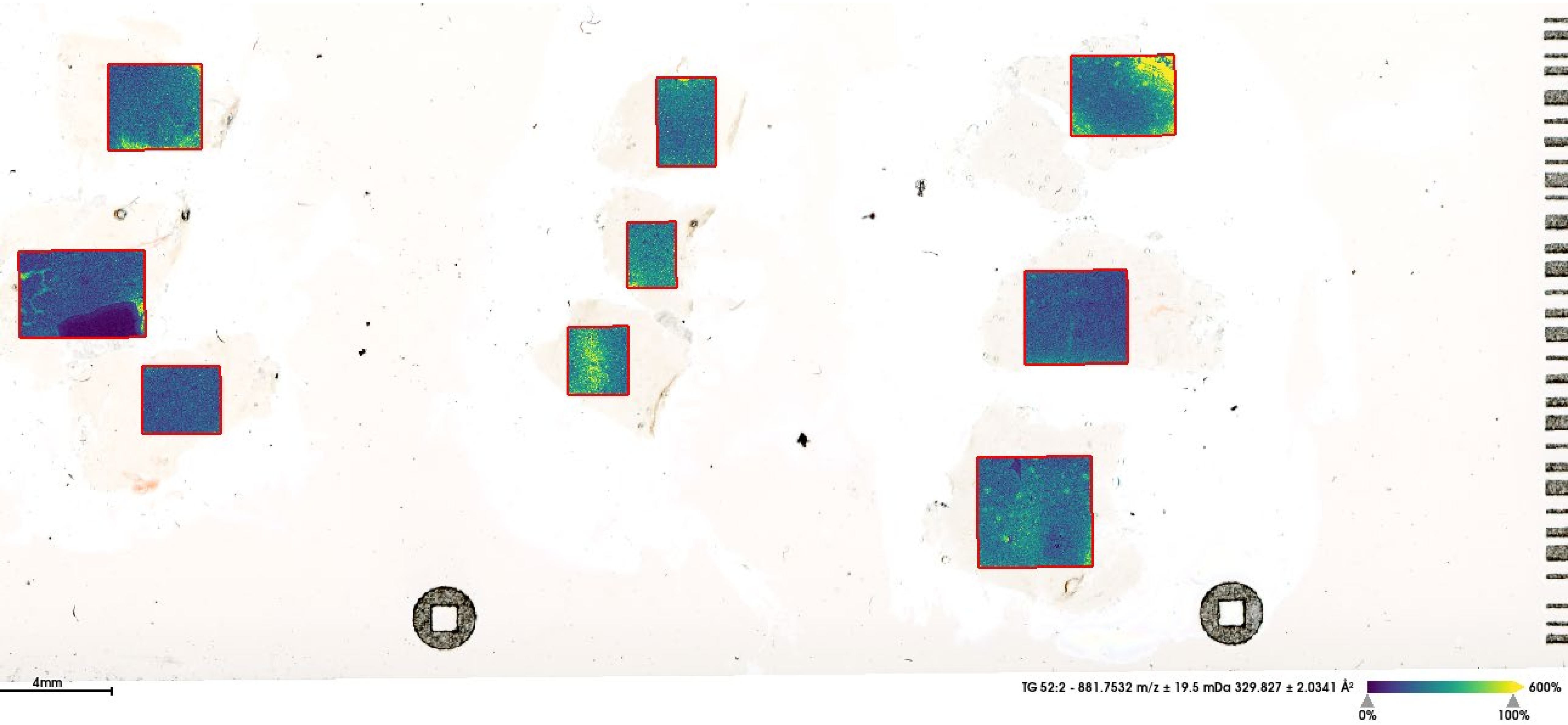
4mm
TG 52:2 - 881.7532 m/z ± 19.5 mDa 329.827 ± 2.0341 Å²
600%
0%
100%

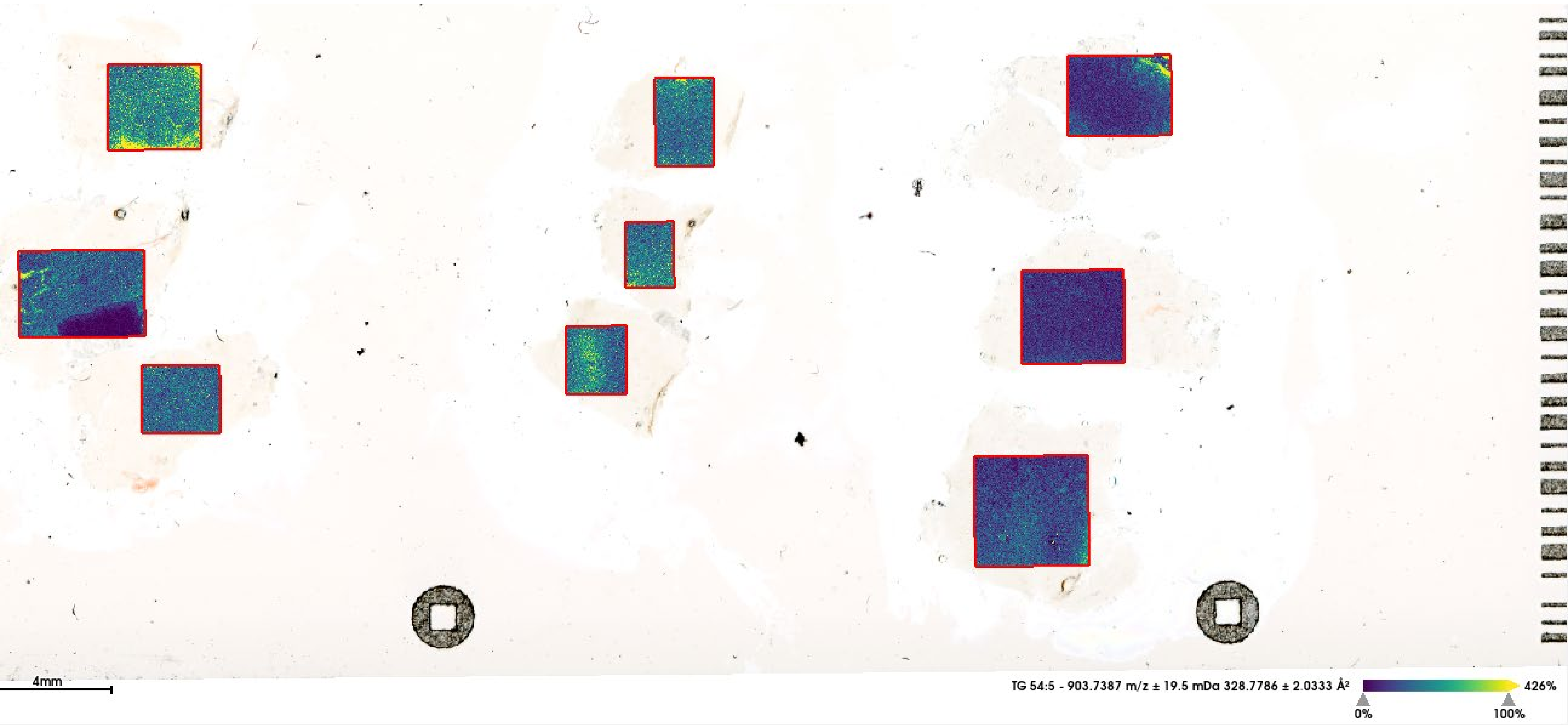
4mm
TG 54:5 - 903.7387 m/z ± 19.5 mDa 328.7786 ± 2.0333 Å²
426%
0%
100%

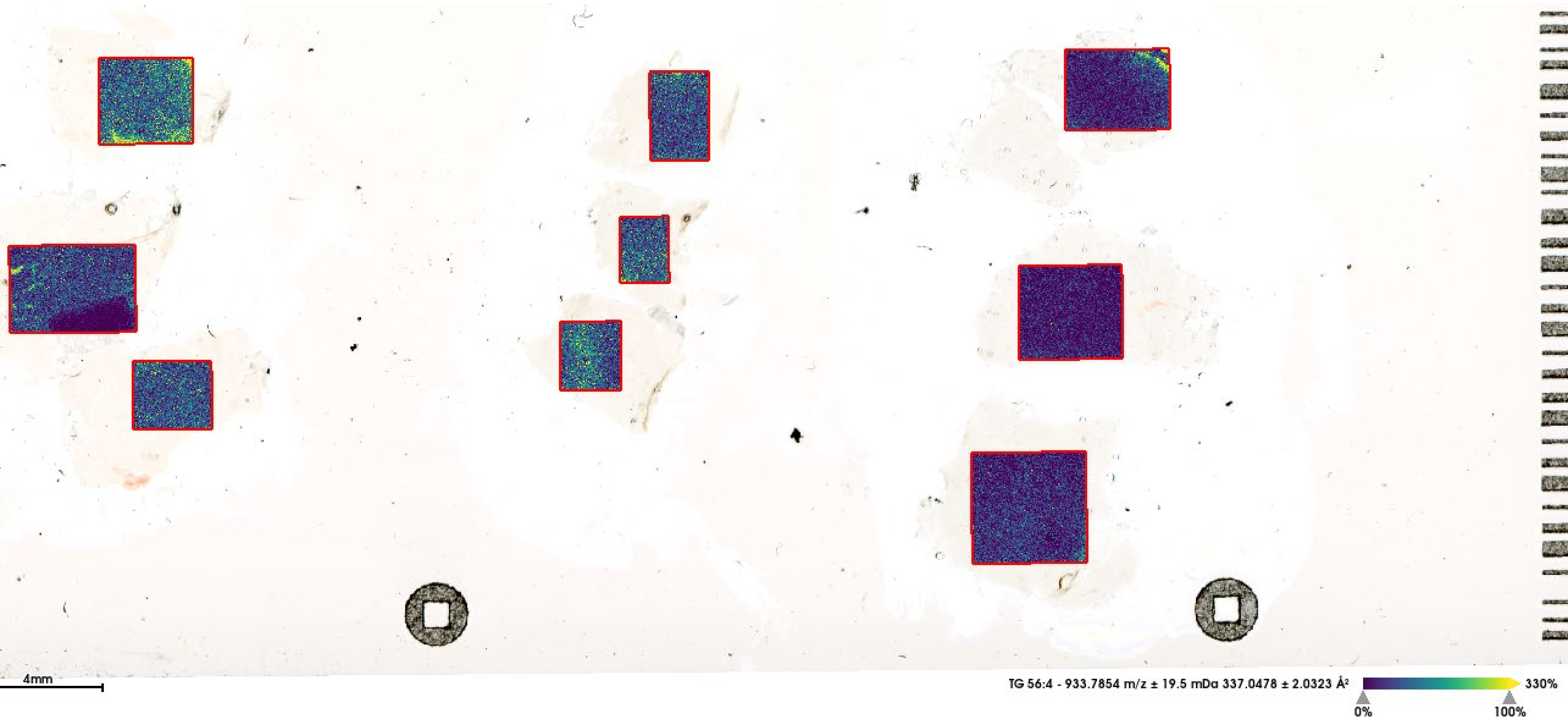

4mm
TG 56:4 - 933.7854 m/z ± 19.5 mDa 337.0478 ± 2.0323 Å²
330%
0%
100%
