# Supplementary material for: Periodic fasting and refeeding re-shapes lipid saturation, storage, and distribution in brown adipose tissue
Source: PLoS Biol. 2026 Jan 12;24(1):e3003593. doi: 10.1371/journal.pbio.3003593 (PMC12795461; doi:10.1371/journal.pbio.3003593)
Supplement: S2 Raw Images — (PDF) [file pbio.3003593.s017.pdf]

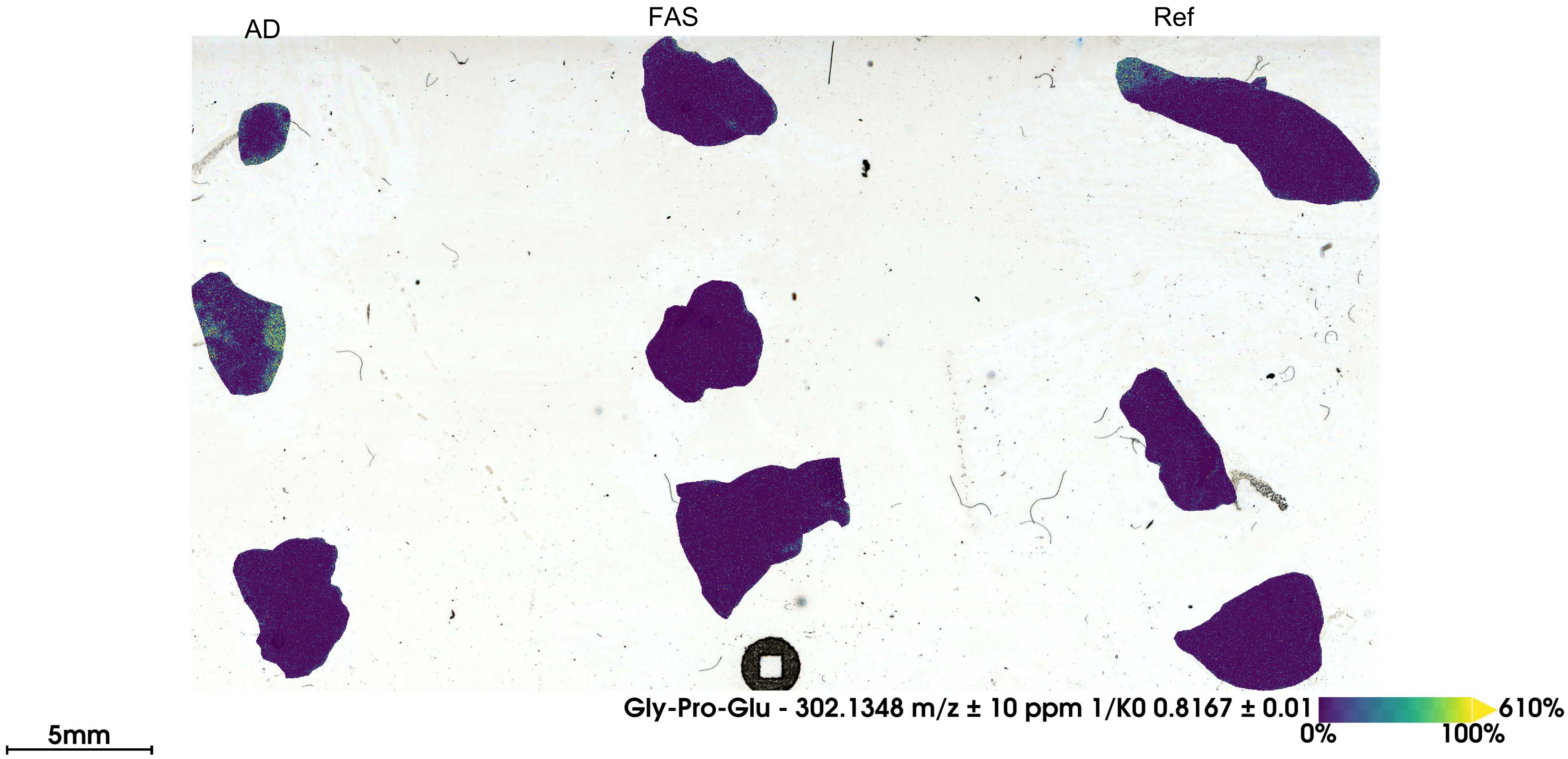

AD
FAS
Ref
Gly-Pro-Glu - 302.1348 m/z ± 10 ppm 1/K0 0.8167 ± 0.01
610%
0%
100%
5mm

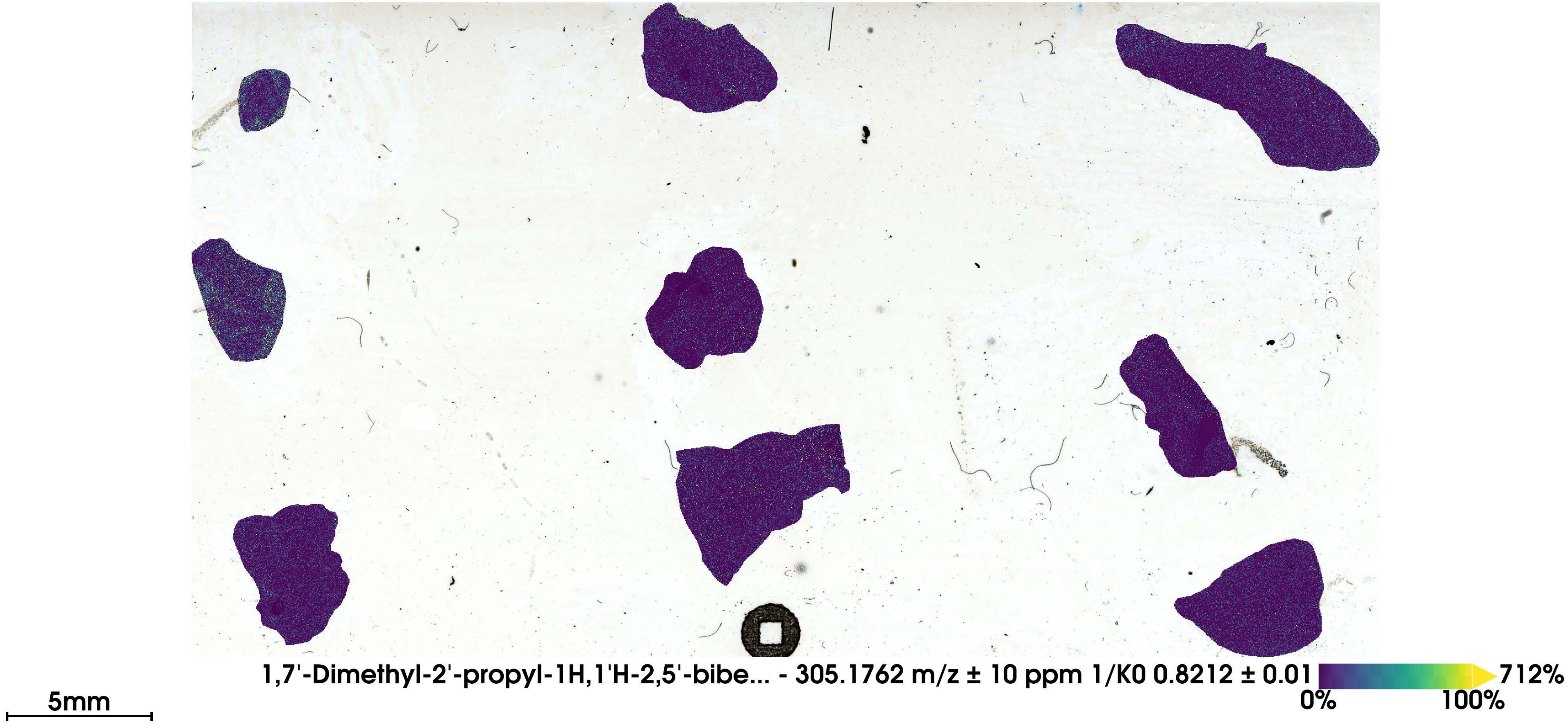
1,7'-Dimethyl-2'-propyl-1H,1'H-2,5'-bibe... - 305.1762 m/z ± 10 ppm 1/K0 0.8212 ± 0.01
712%
0%
100%
5mm

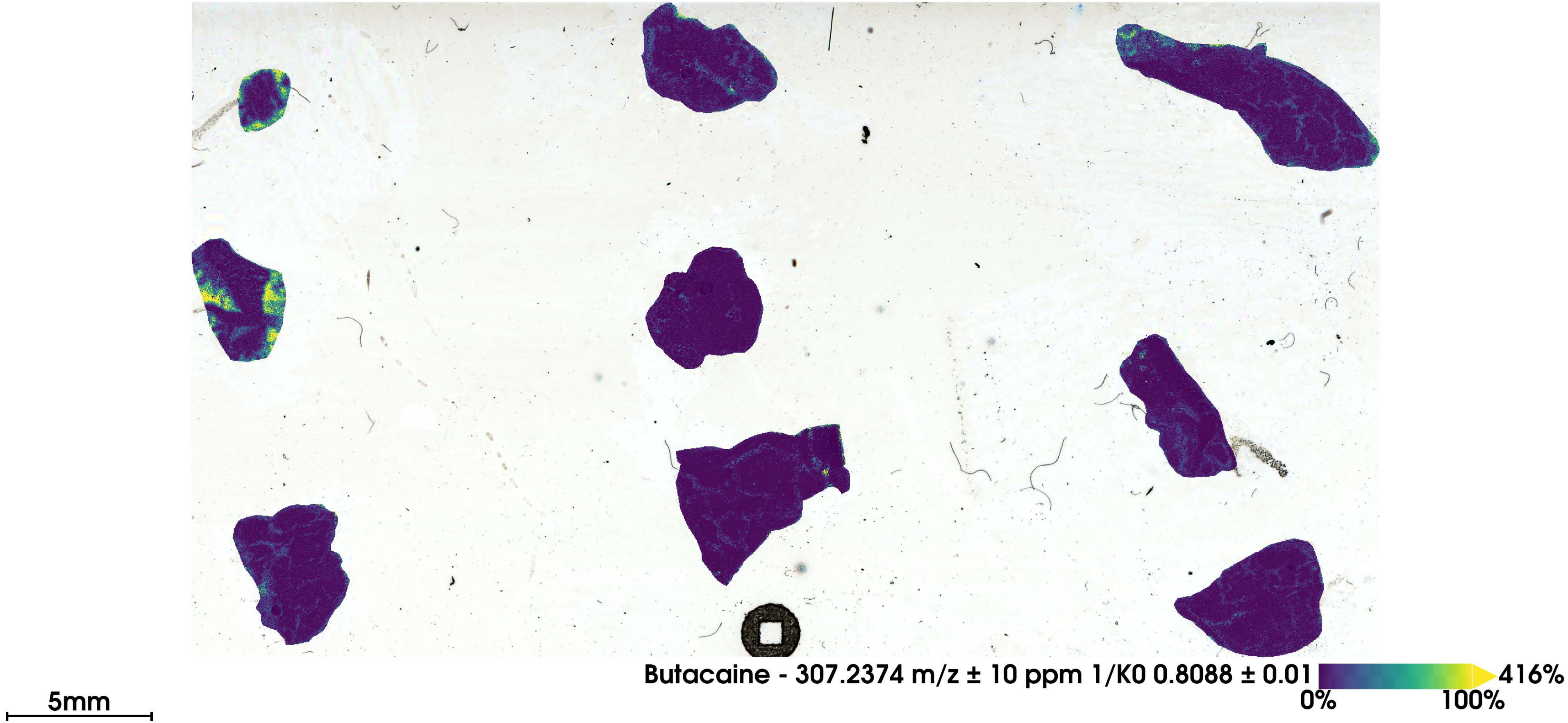
Butacaine - 307.2374 m/z ± 10 ppm 1/K0 0.8088 ± 0.01
416%
0%
100%
5mm

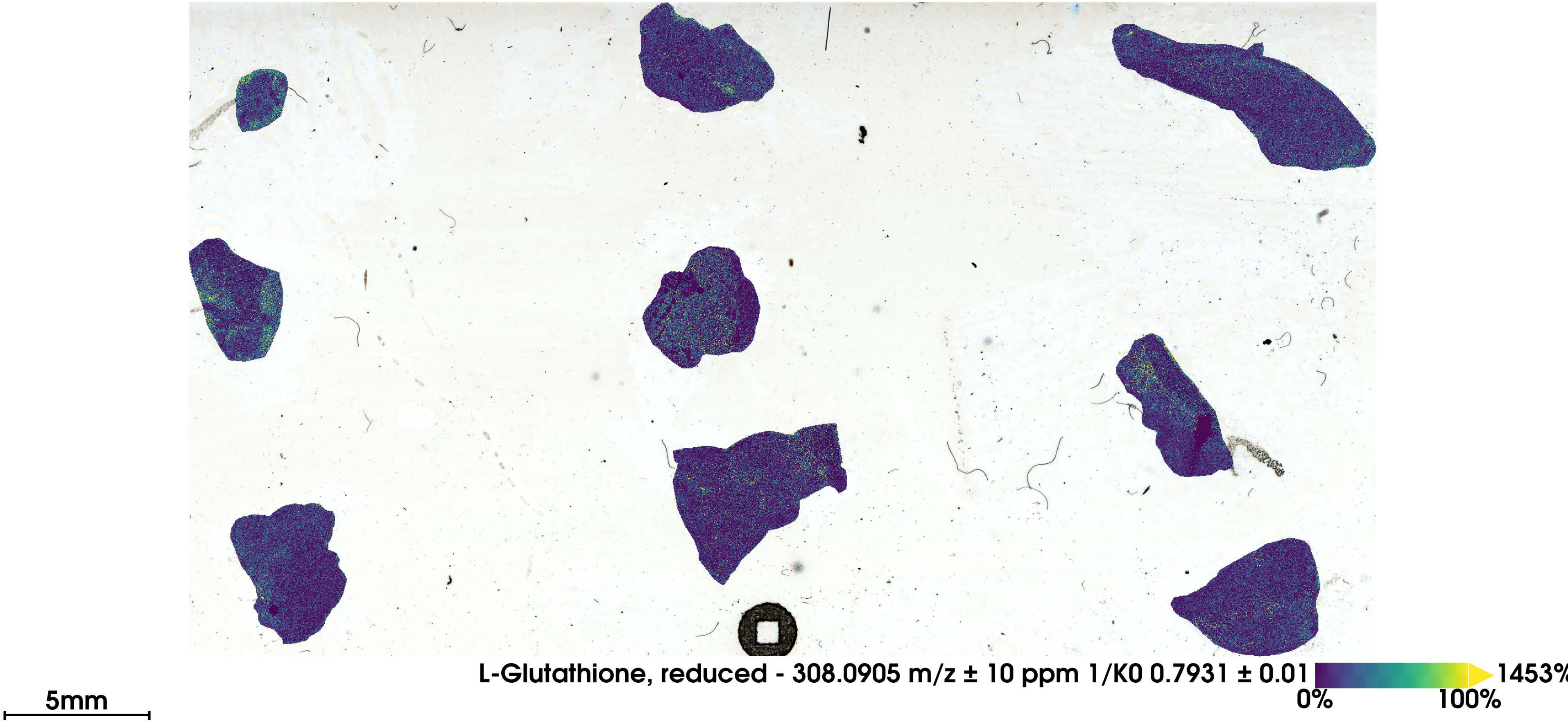
L-Glutathione, reduced - 308.0905 m/z ± 10 ppm 1/K0 0.7931 ± 0.01
1453%
0%
100%
5mm

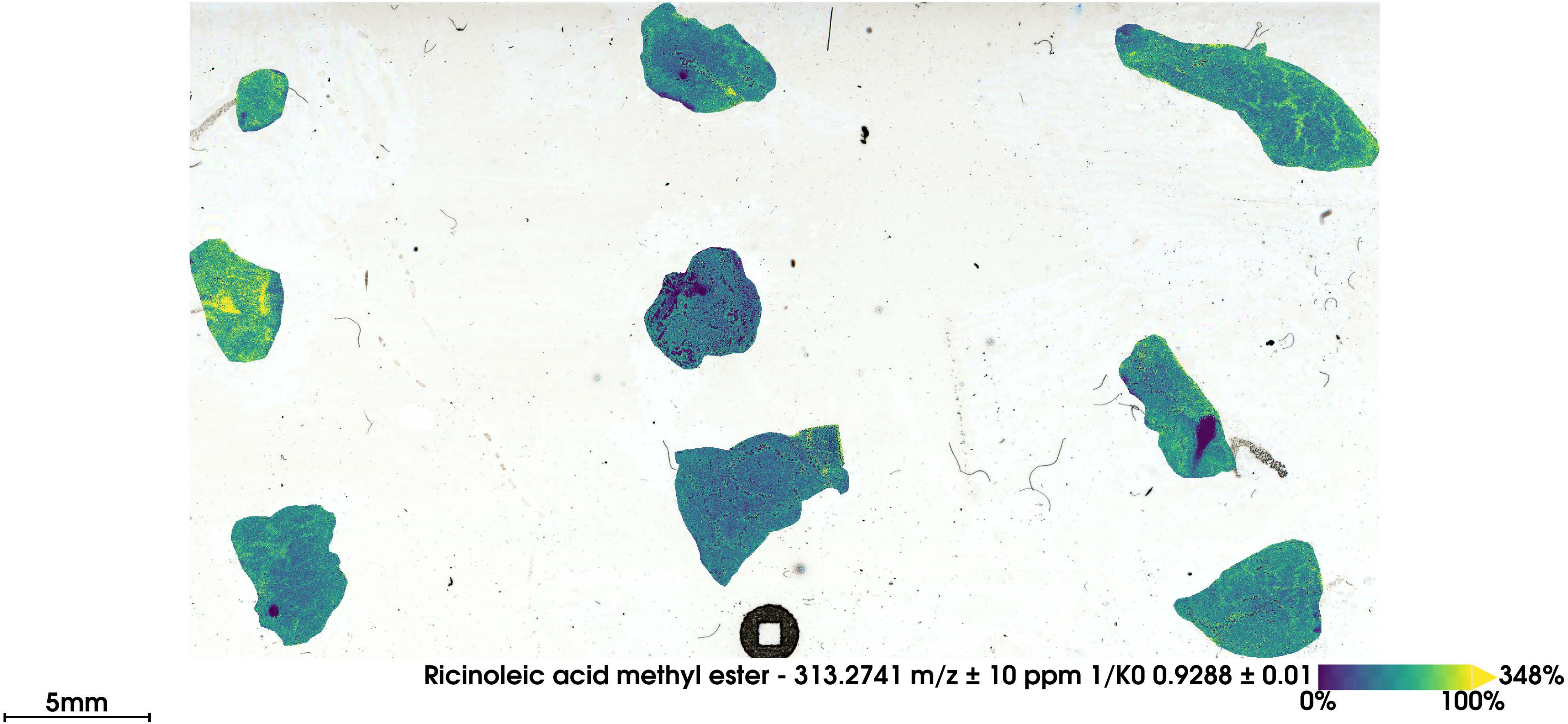

Ricinoleic acid methyl ester - 313.2741 m/z ± 10 ppm 1/K0 0.9288 ± 0.01
348%
0%
100%
5mm

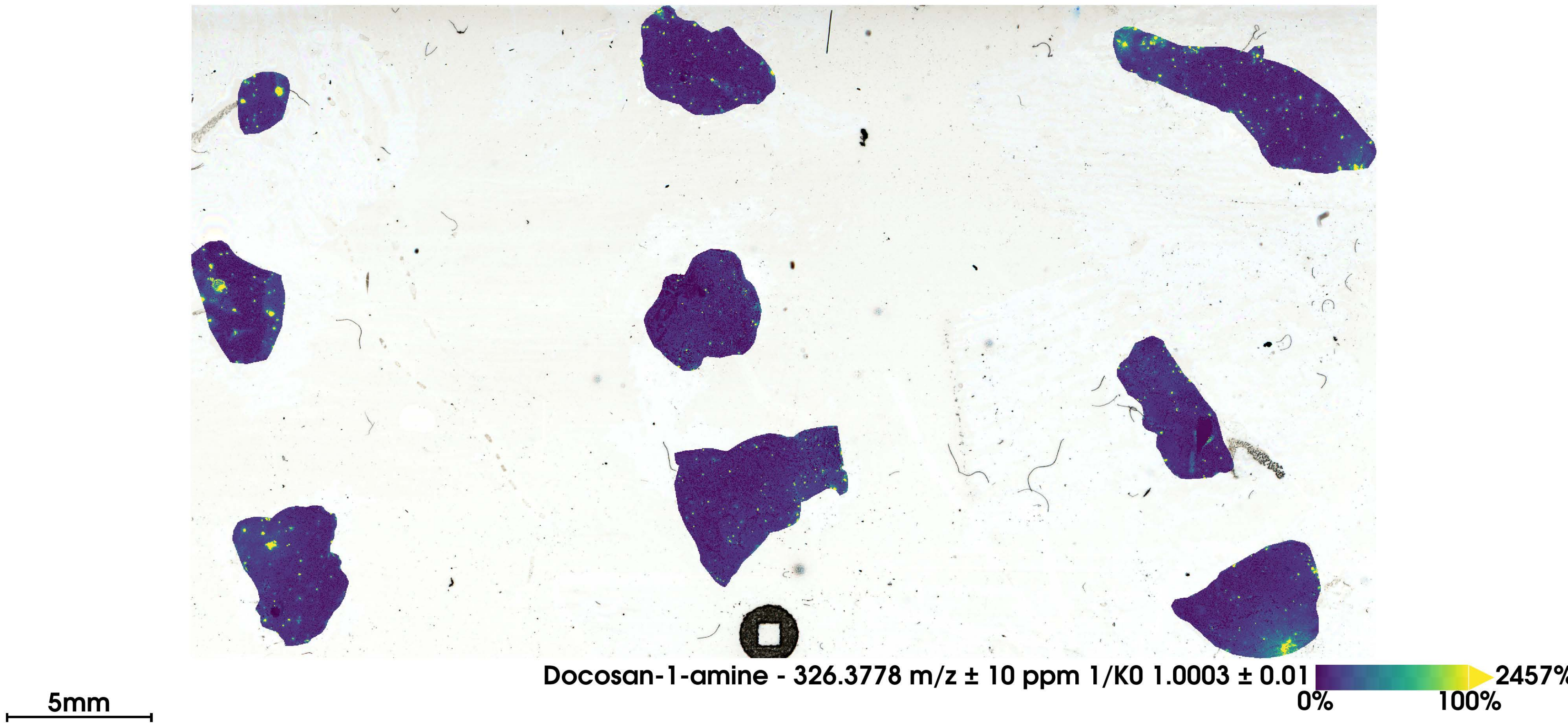

Docosan-1-amine - 326.3778 m/z ± 10 ppm 1/K0 1.0003 ± 0.01
2457%
0%
100%
5mm

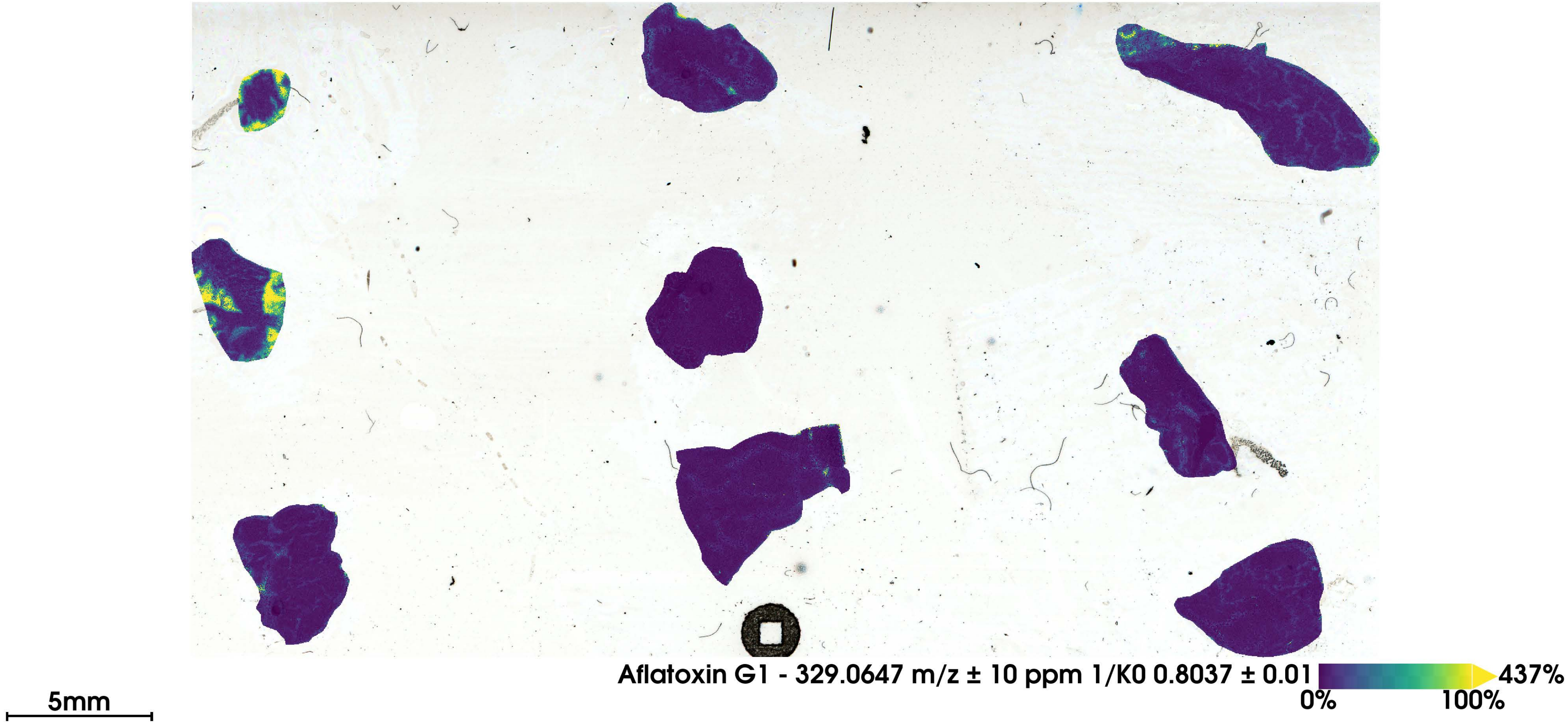
Aflatoxin G1 - 329.0647 m/z ± 10 ppm 1/K0 0.8037 ± 0.01
437%
0%
100%
5mm

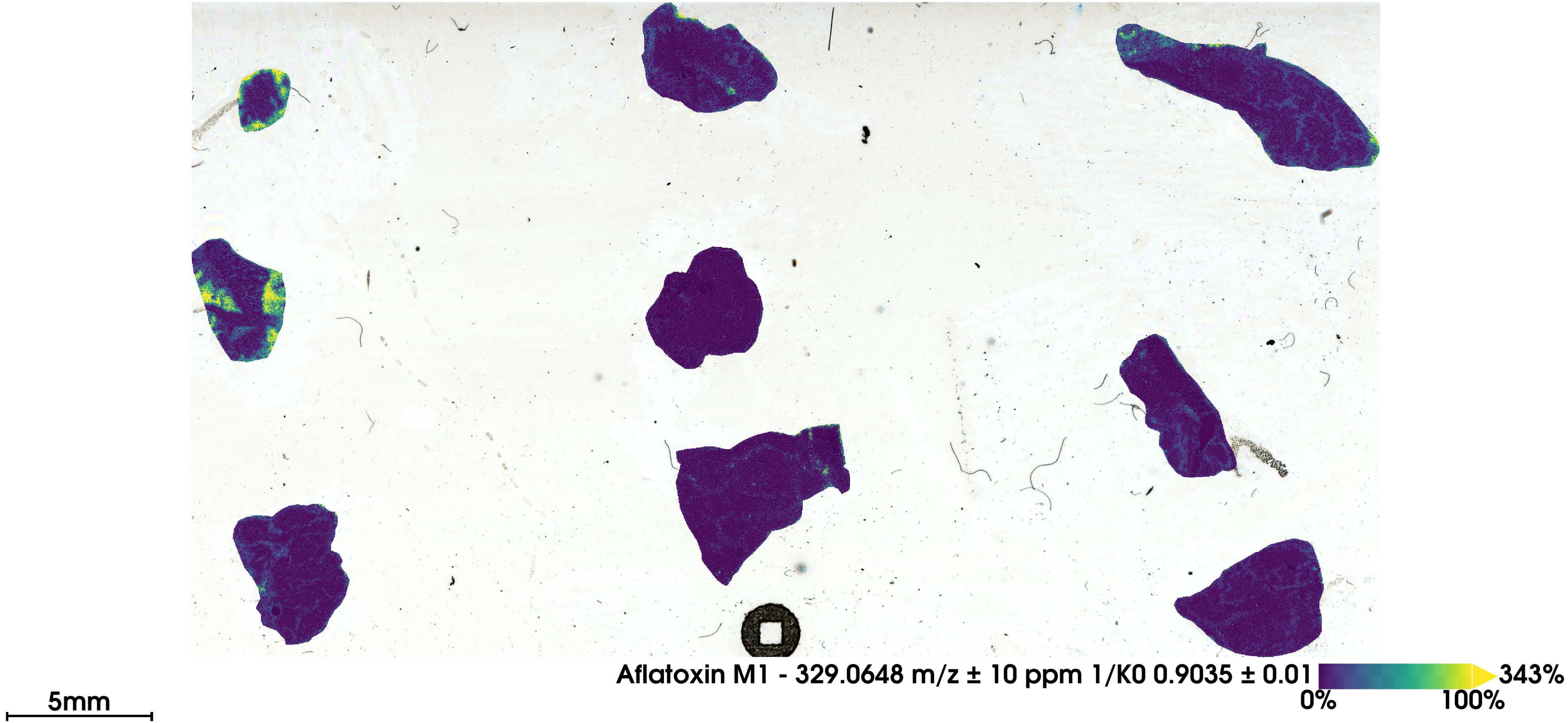

Aflatoxin M1 - 329.0648 m/z ± 10 ppm 1/K0 0.9035 ± 0.01
343%
0%
100%
5mm

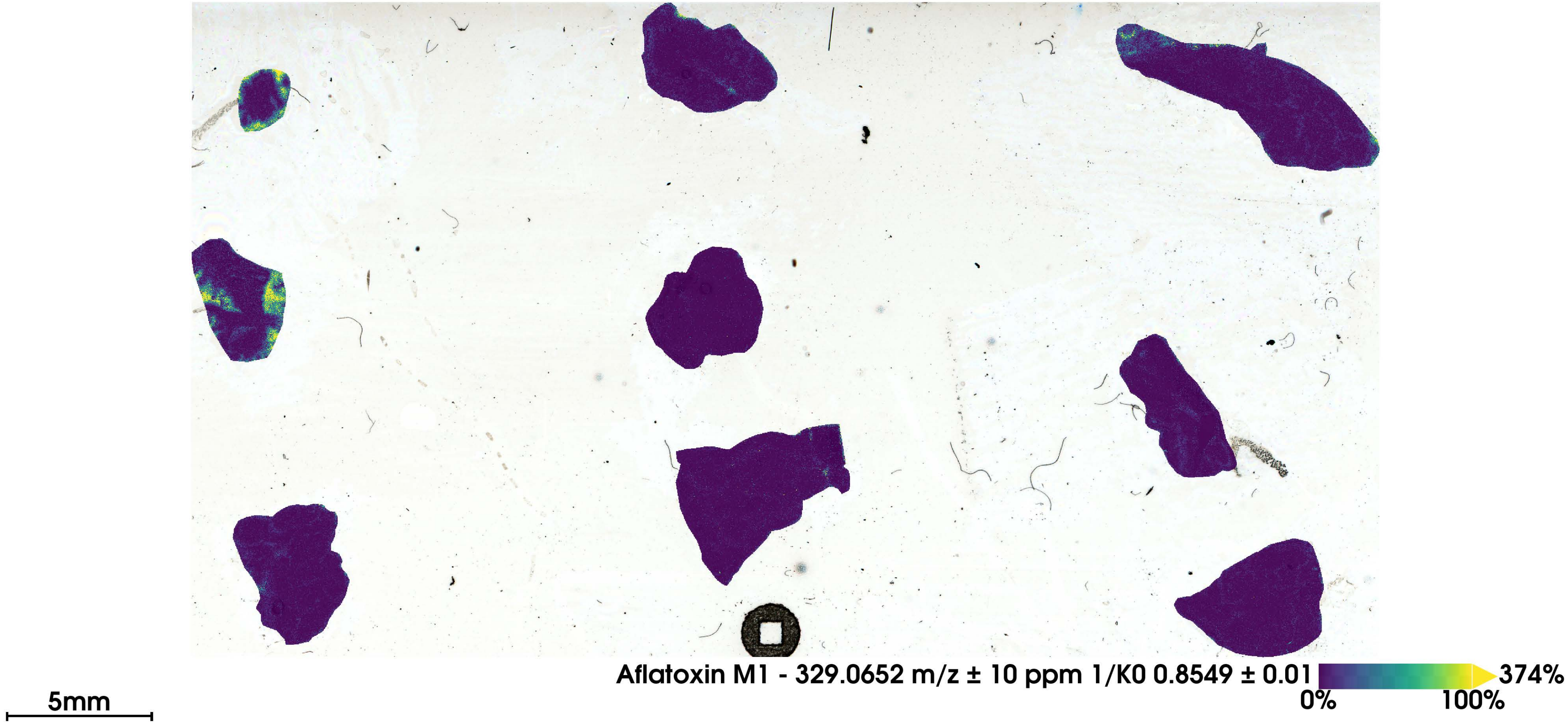
Aflatoxin M1 - 329.0652 m/z ± 10 ppm 1/K0 0.8549 ± 0.01
374%
0%
100%
5mm

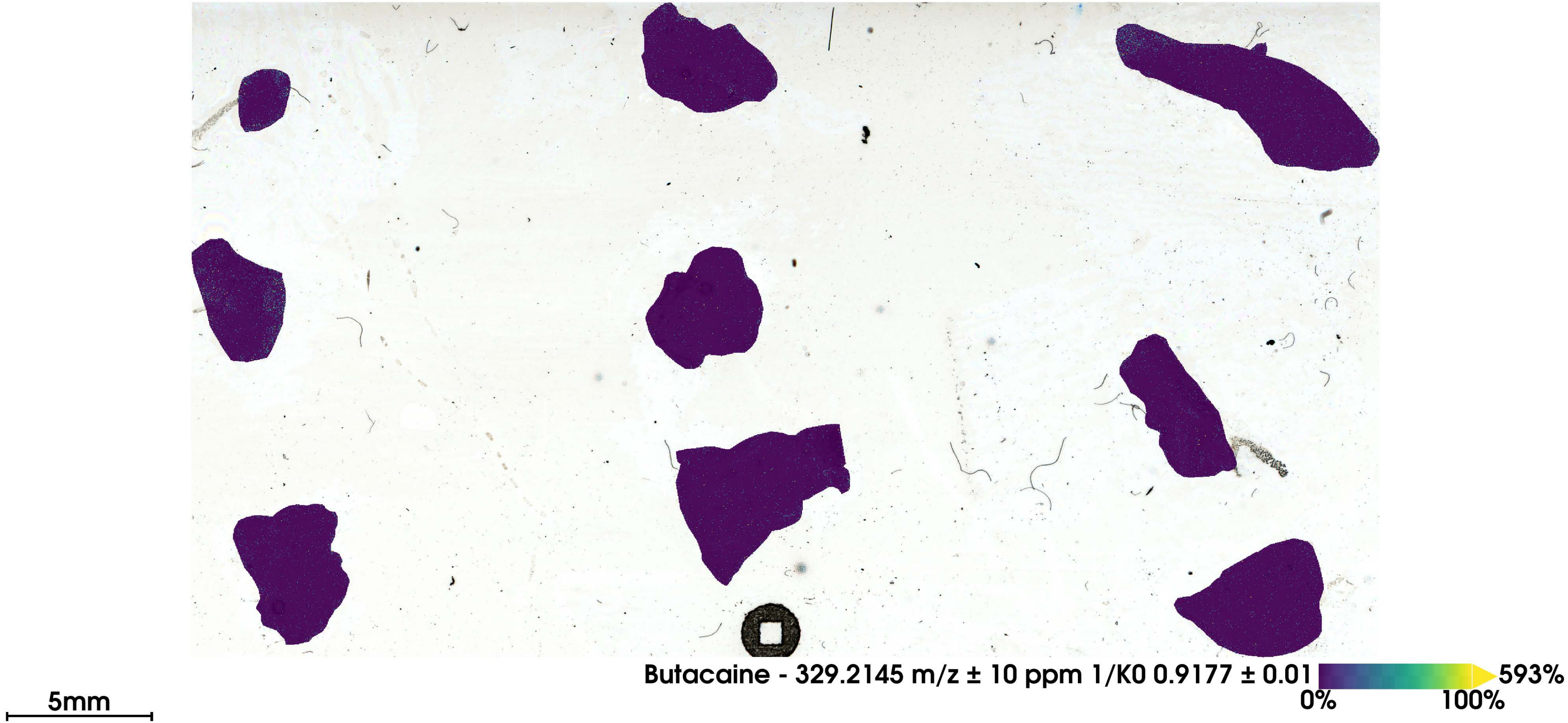
5mm
Butacaine - 329.2145 m/z ± 10 ppm 1/K0 0.9177 ± 0.01
593%
0%
100%

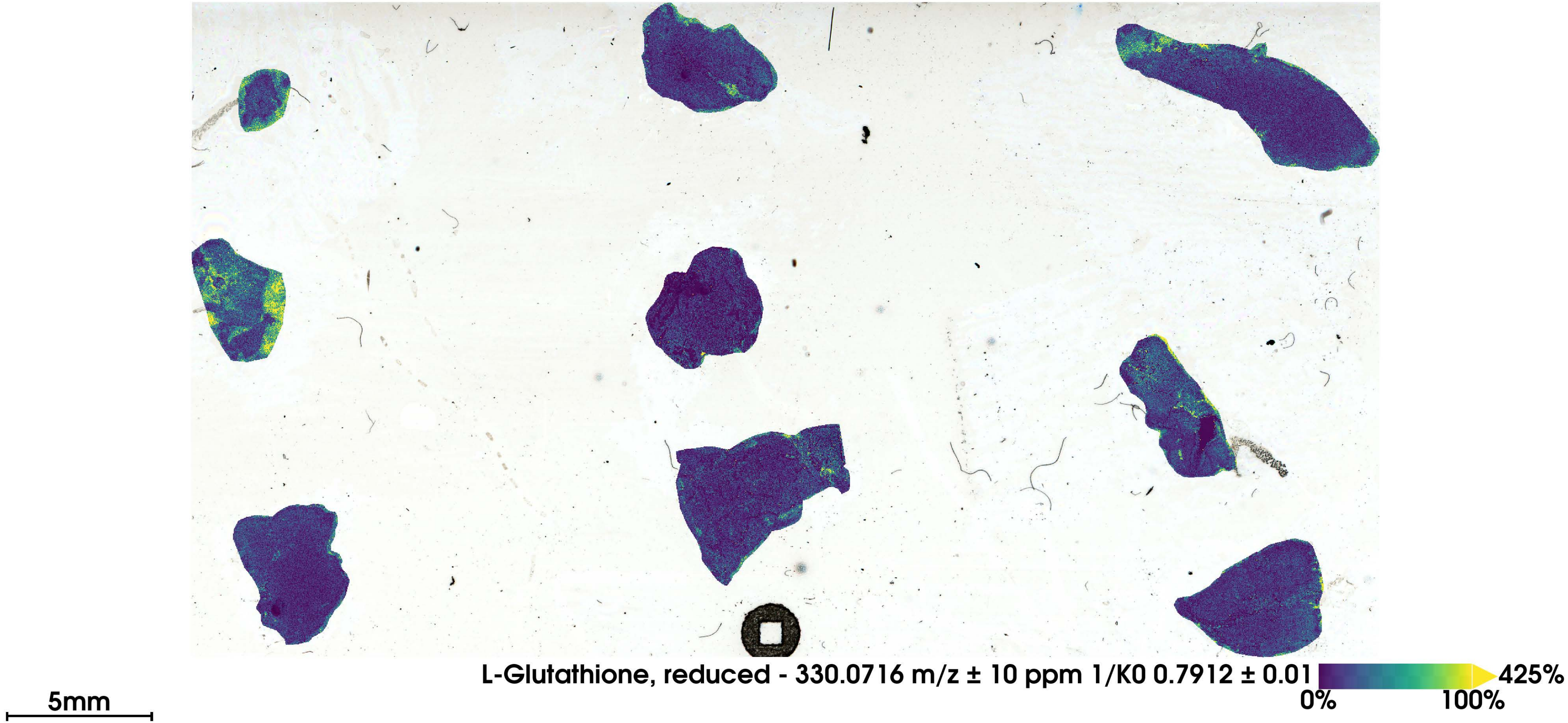
L-Glutathione, reduced - 330.0716 m/z ± 10 ppm 1/K0 0.7912 ± 0.01
425%
0%
100%
5mm

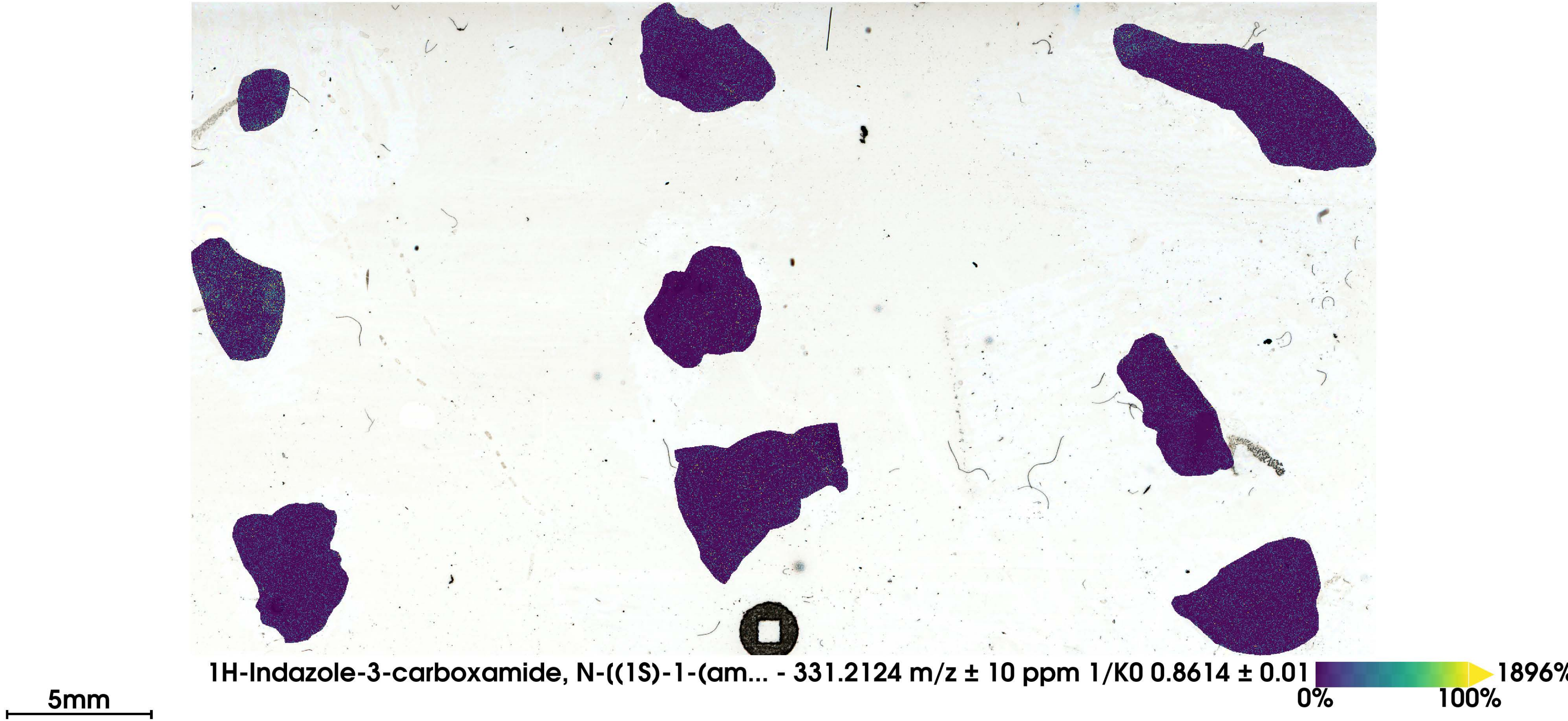
1H-Indazole-3-carboxamide, N-((1S)-1-(am... - 331.2124 m/z ± 10 ppm 1/K0 0.8614 ± 0.01
1896%
0%
100%
5mm

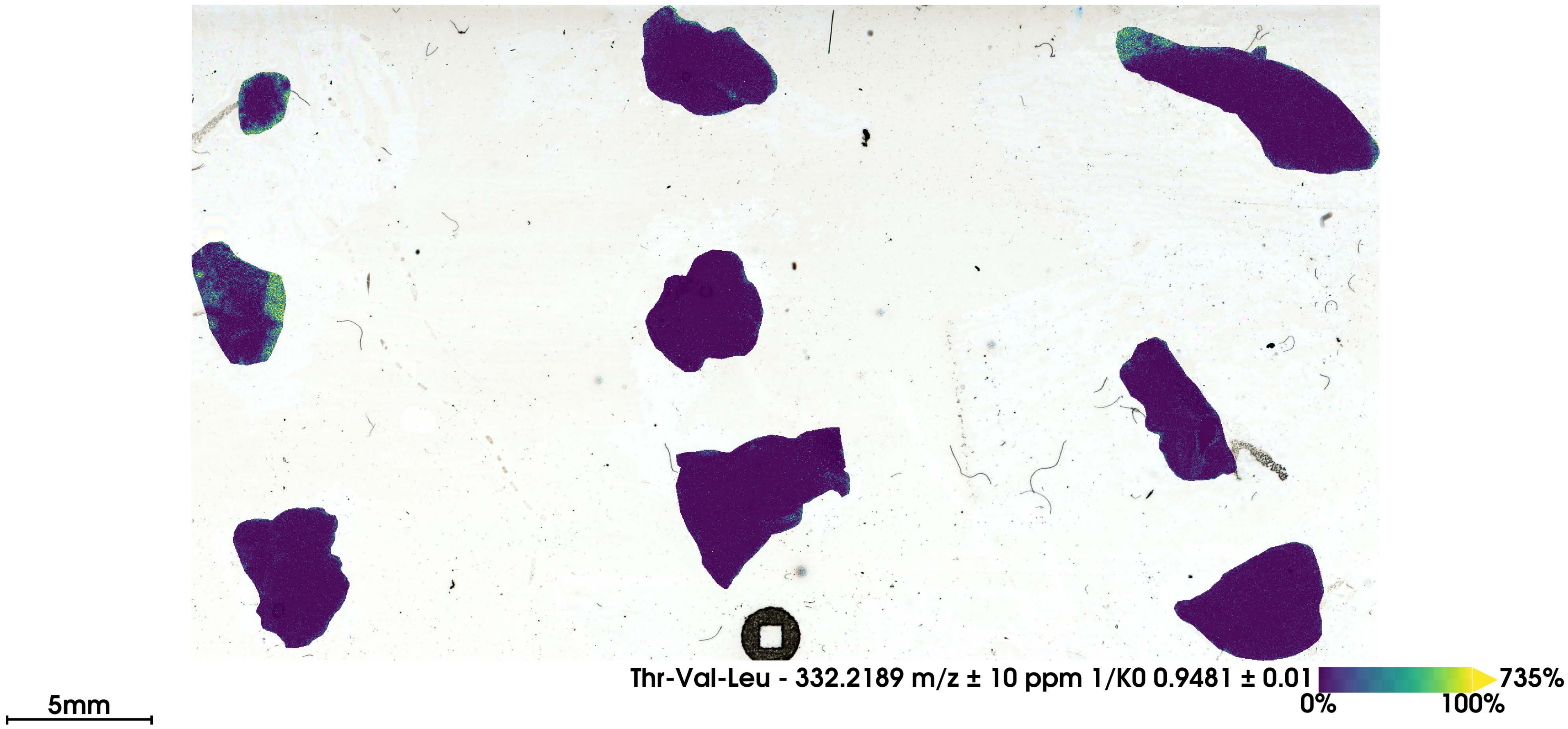
Thr-Val-Leu - 332.2189 m/z ± 10 ppm 1/K0 0.9481 ± 0.01
735%
0%
100%
5mm

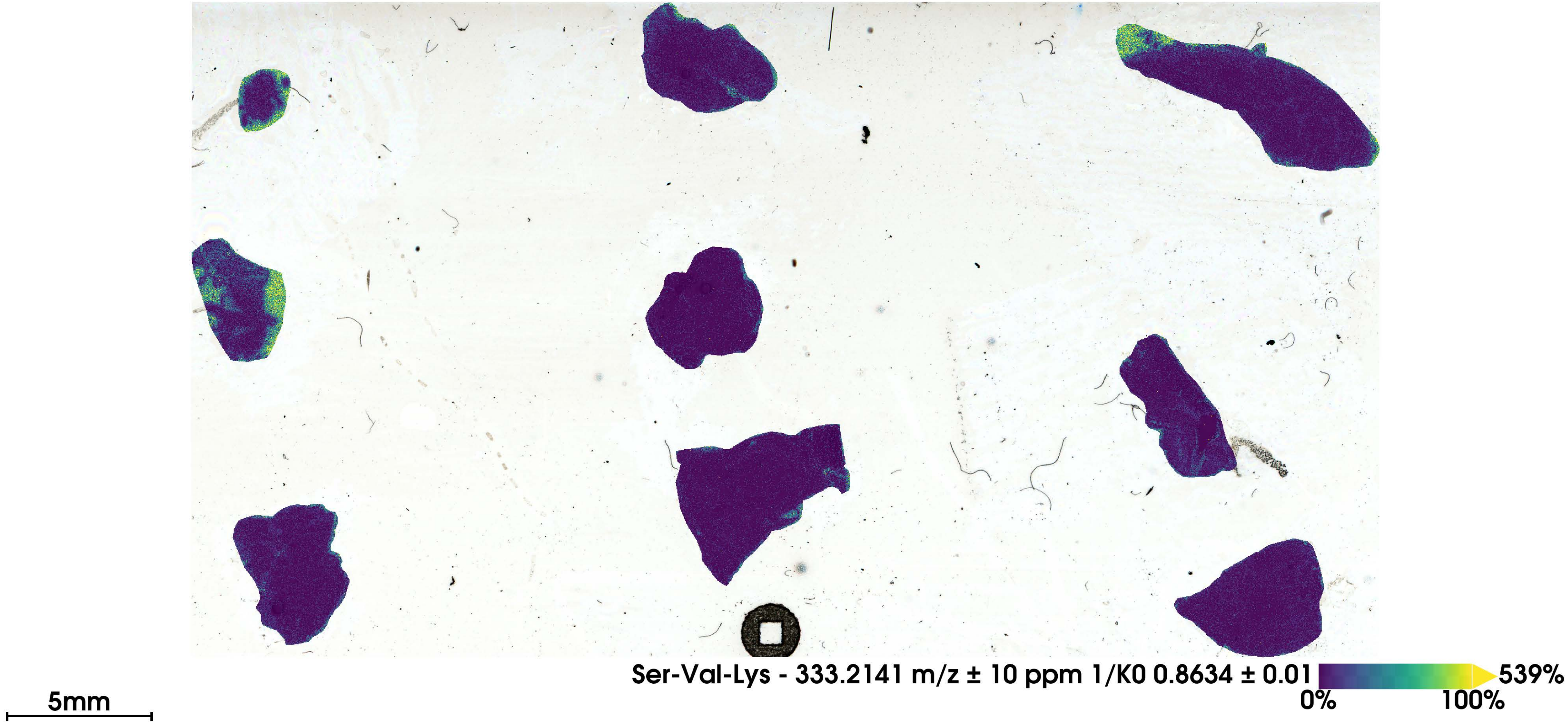
Ser-Val-Lys - 333.2141 m/z ± 10 ppm 1/K0 0.8634 ± 0.01
539%
0%
100%
5mm

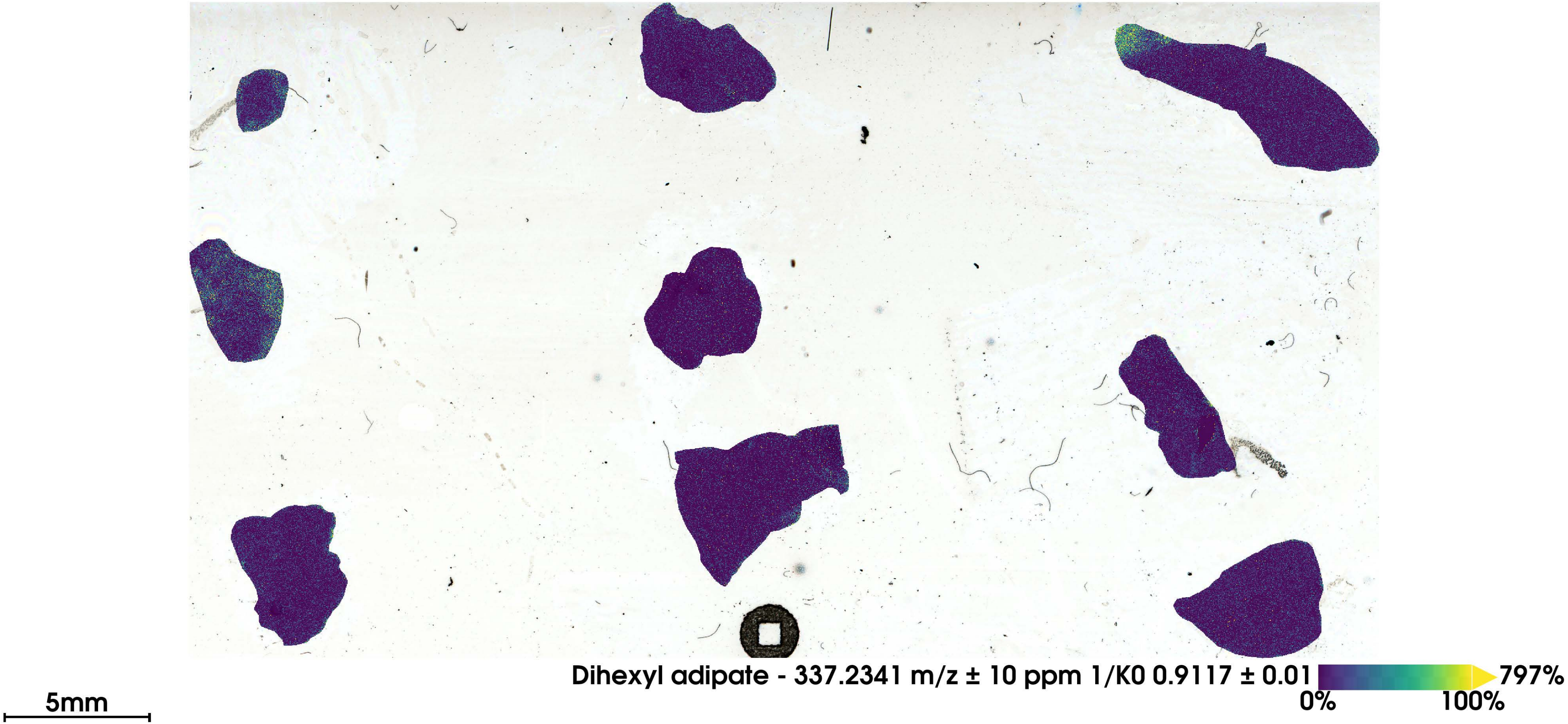
Dihexyl adipate - 337.2341 m/z ± 10 ppm 1/K0 0.9117 ± 0.01
797%
0%
100%
5mm

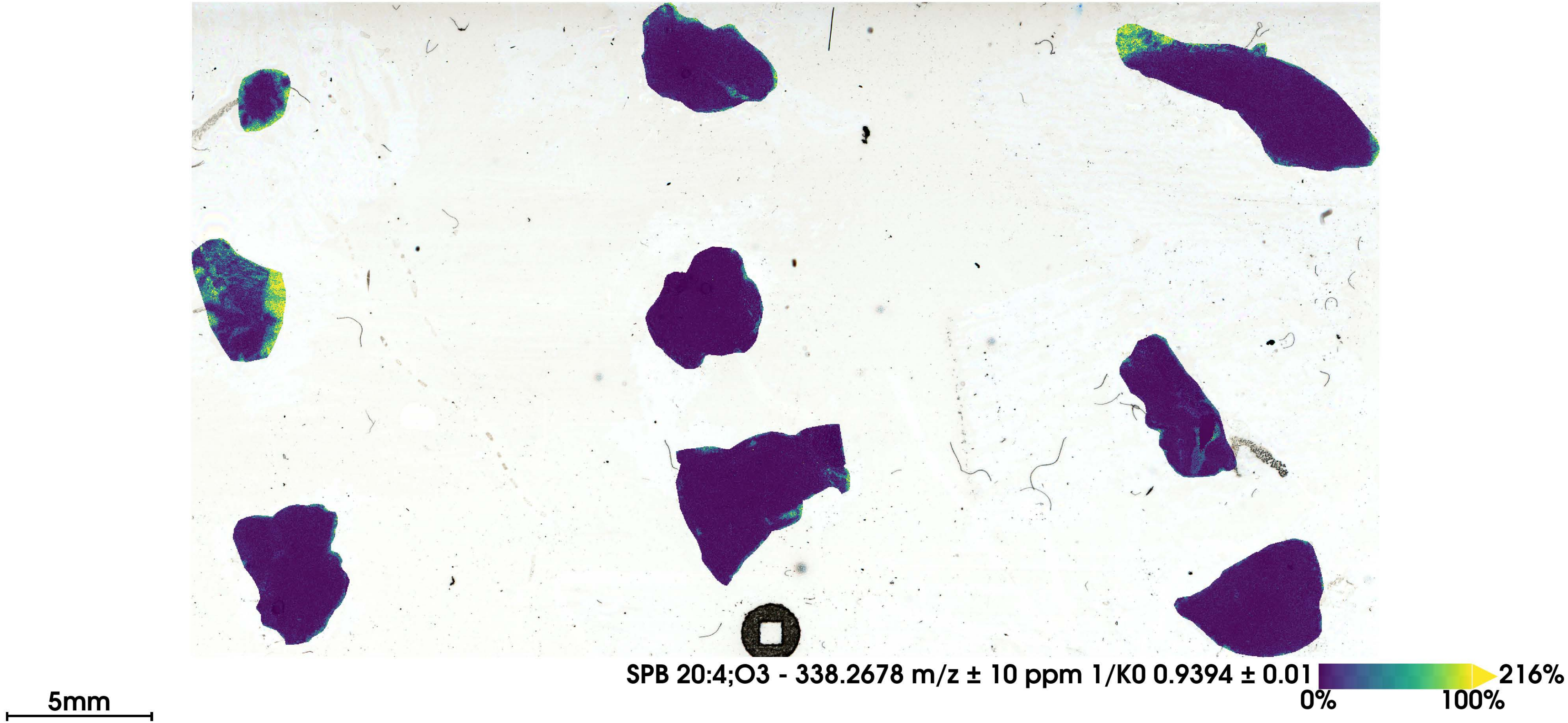

SPB 20:4;O3 - 338.2678 m/z ± 10 ppm 1/K0 0.9394 ± 0.01
216%
0%
100%
5mm

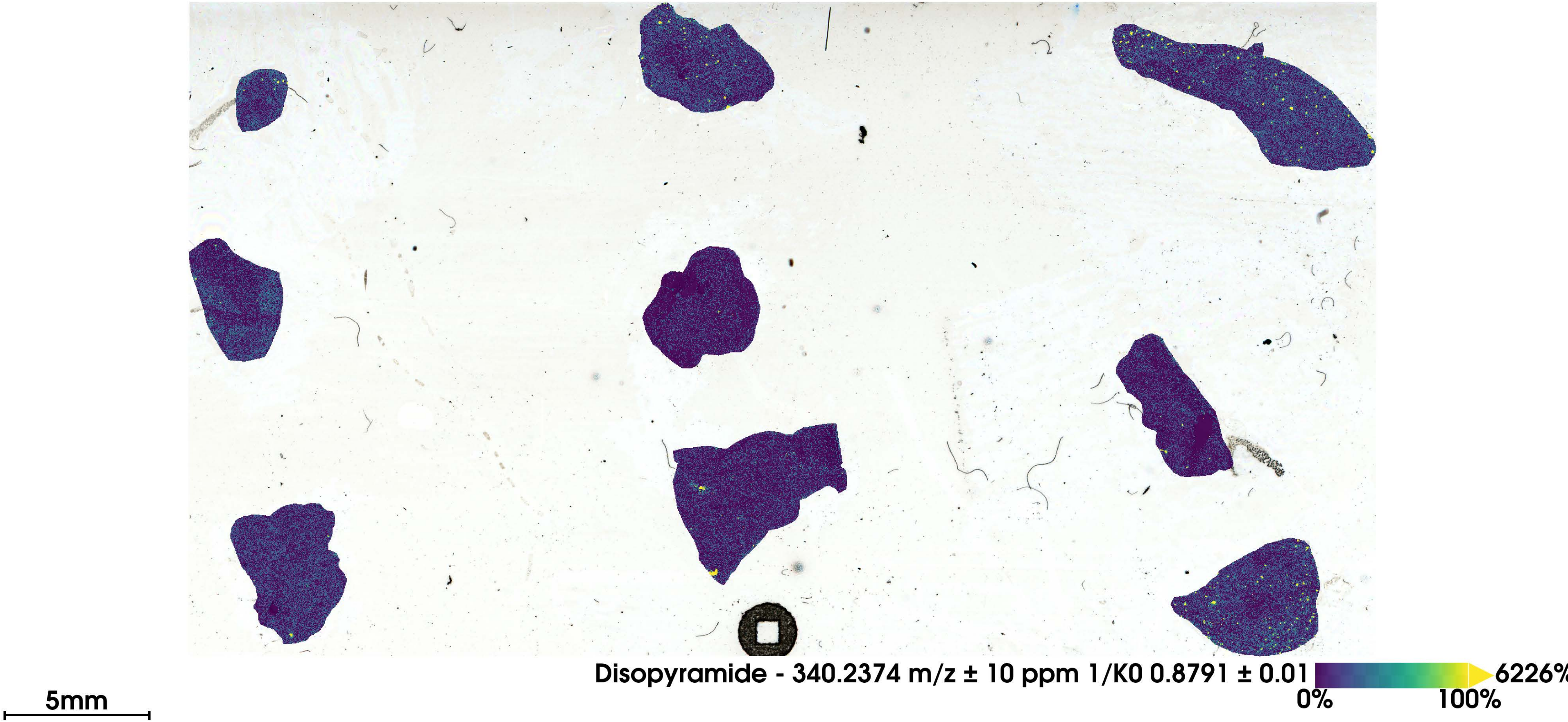
Disopyramide - 340.2374 m/z ± 10 ppm 1/K0 0.8791 ± 0.01
6226%
0%
100%
5mm

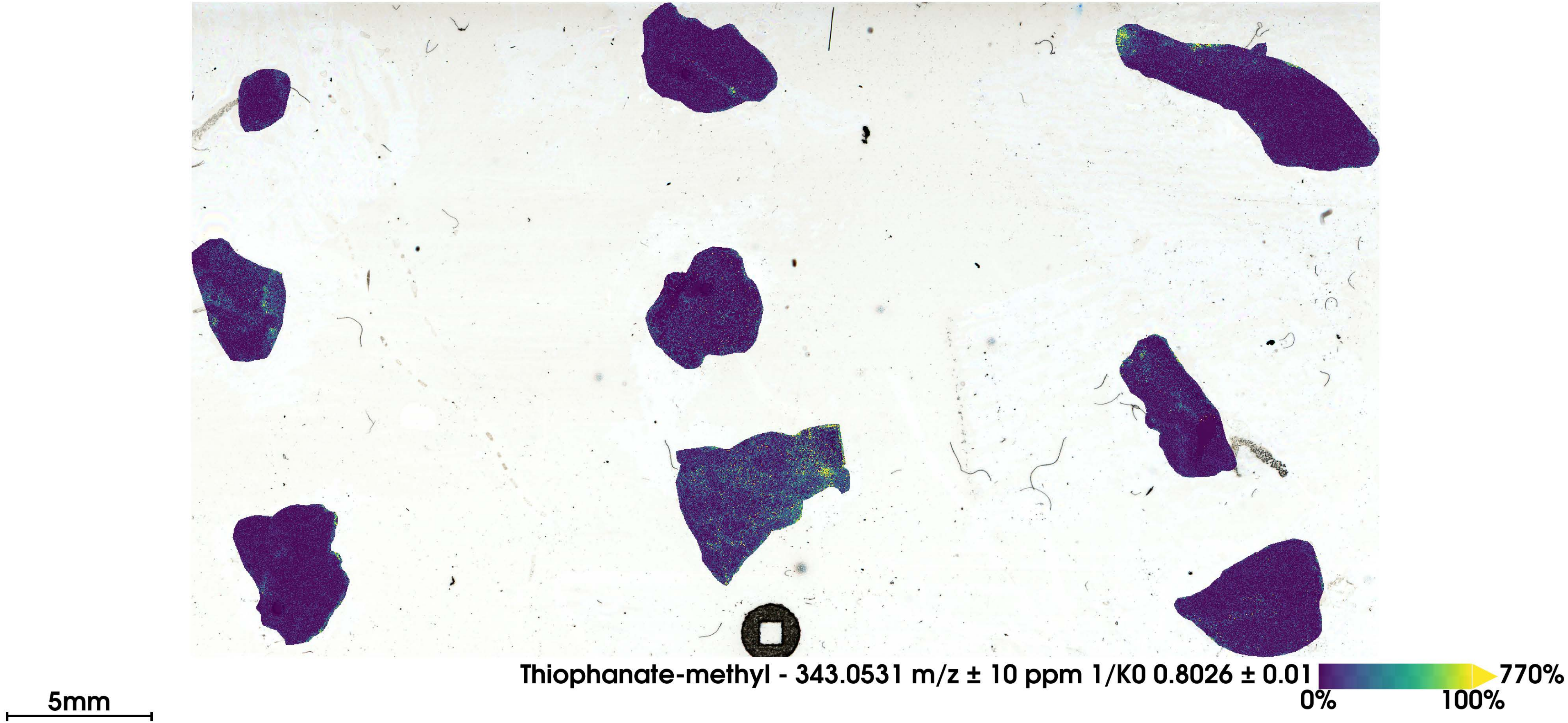
Thiophanate-methyl - 343.0531 m/z ± 10 ppm 1/K0 0.8026 ± 0.01
770%
0%
100%
5mm

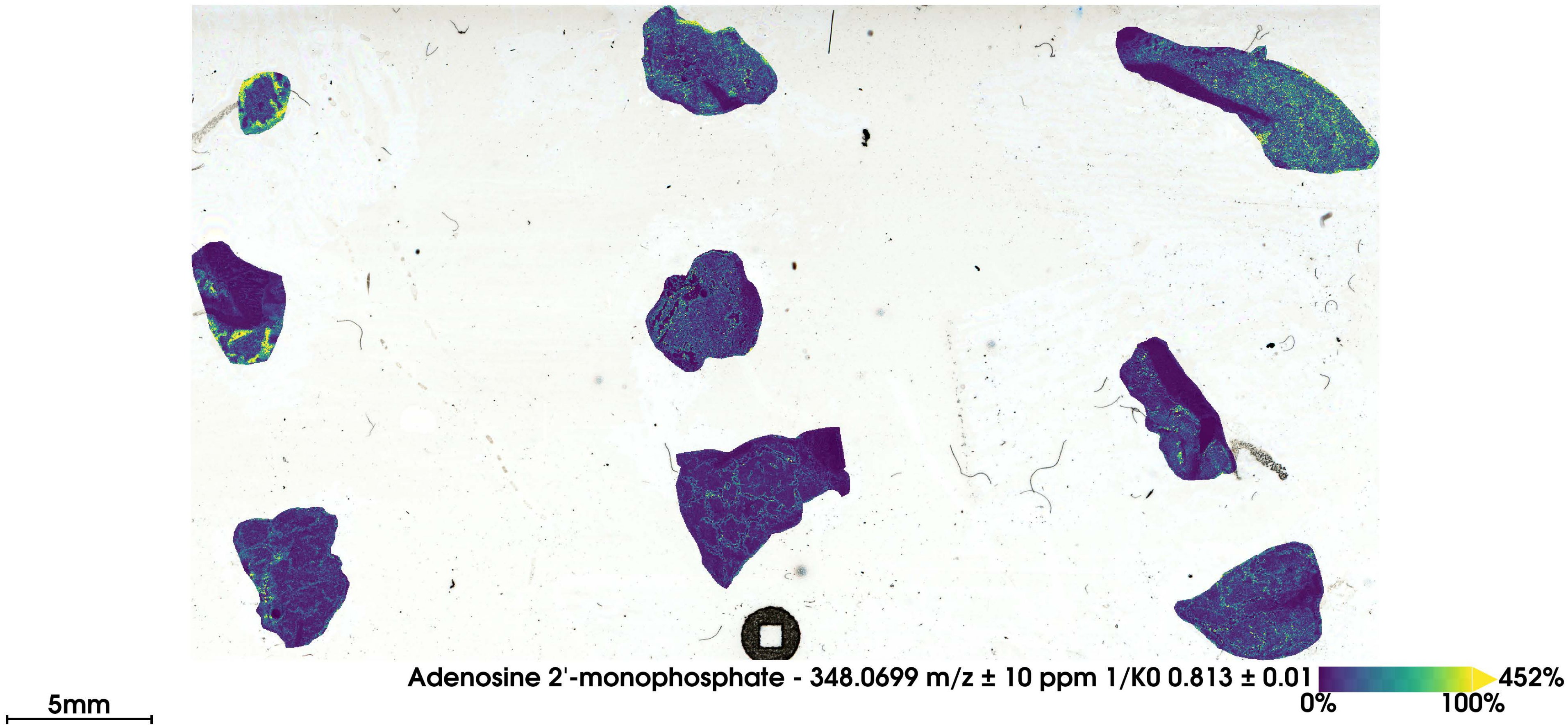
Adenosine 2'-monophosphate - 348.0699 m/z ± 10 ppm 1/K0 0.813 ± 0.01
452%
0%
100%
5mm

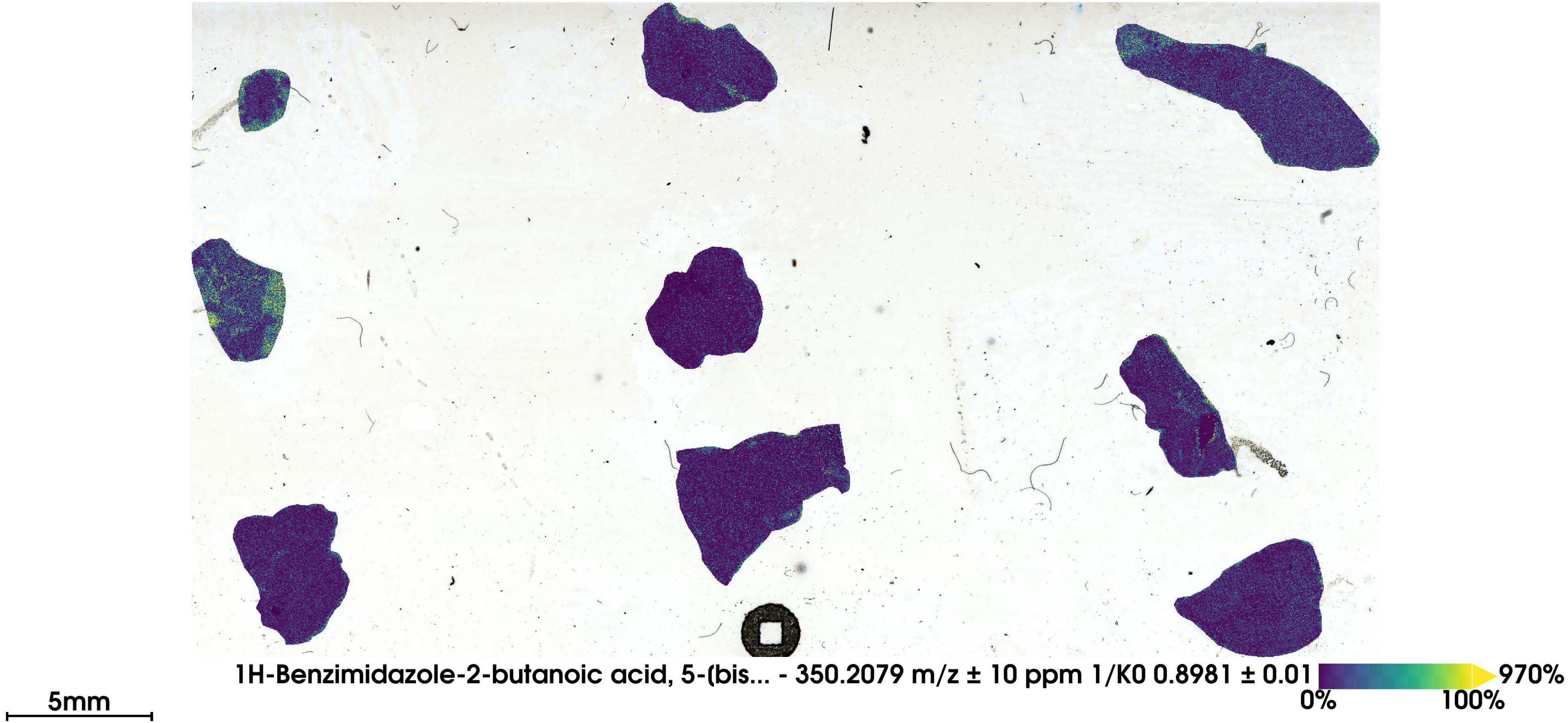

1H-Benzimidazole-2-butanoic acid, 5-(bis... - 350.2079 m/z ± 10 ppm 1/K0 0.8981 ± 0.01
970%
0%
100%
5mm

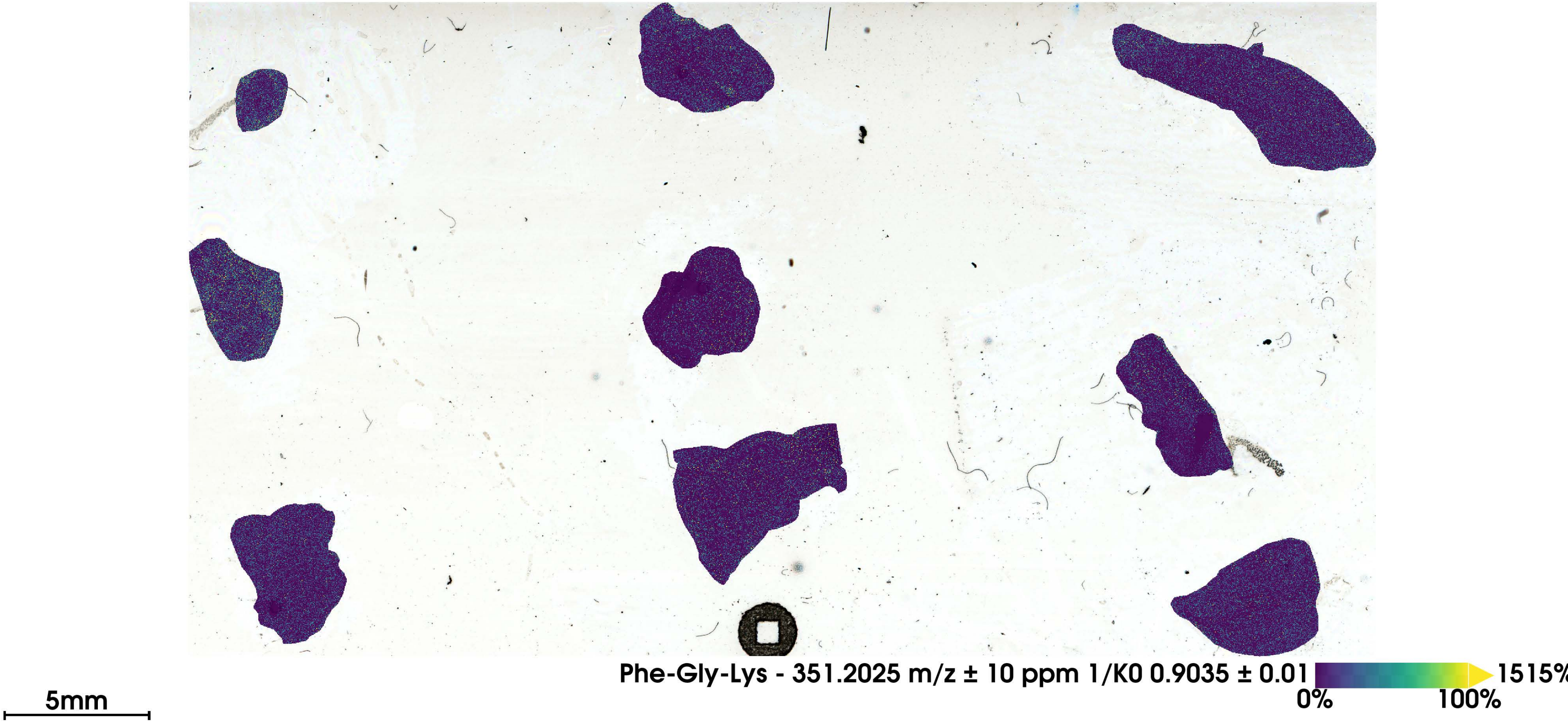
Phe-Gly-Lys - 351.2025 m/z ± 10 ppm 1/K0 0.9035 ± 0.01
1515%
0%
100%
5mm

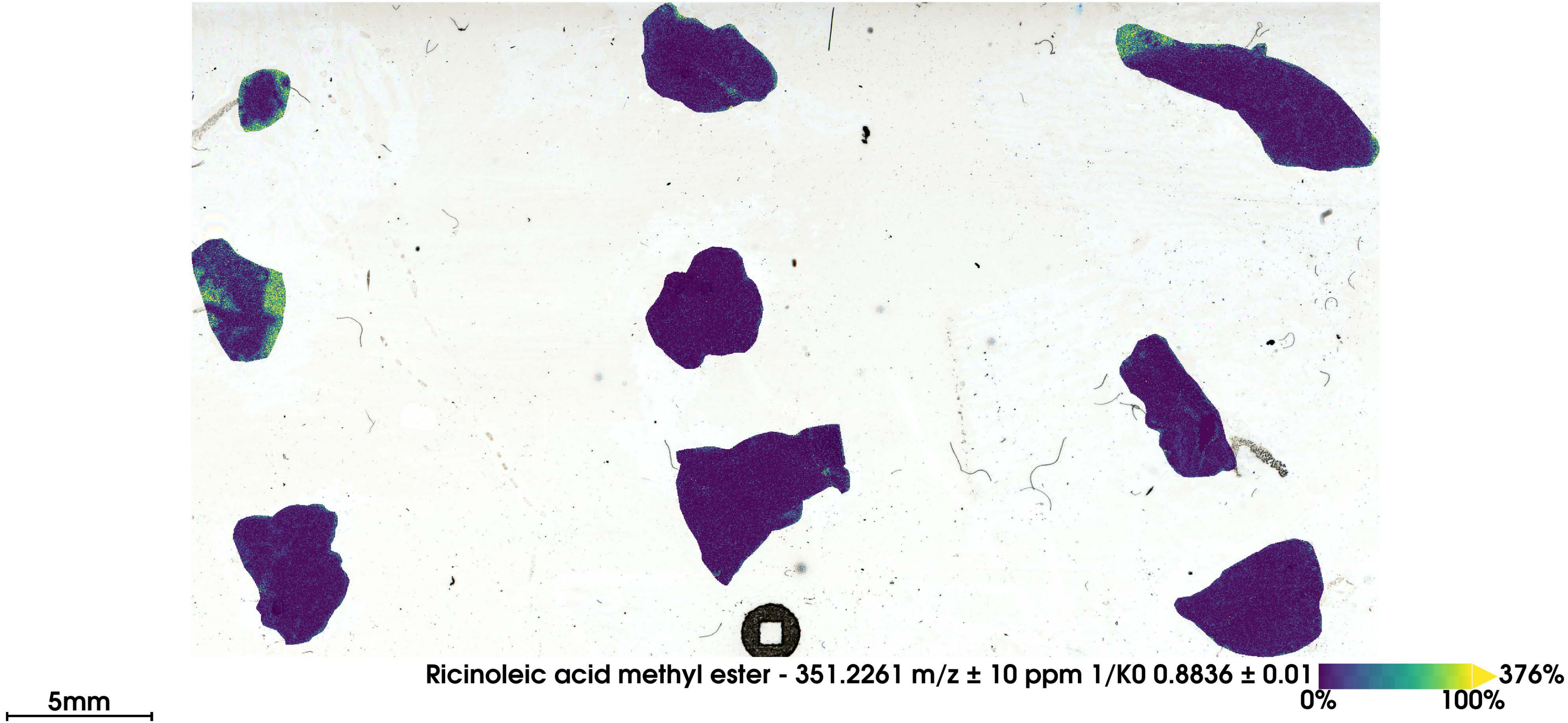
Ricinoleic acid methyl ester - 351.2261 m/z ± 10 ppm 1/K0 0.8836 ± 0.01
376%
0%
100%
5mm

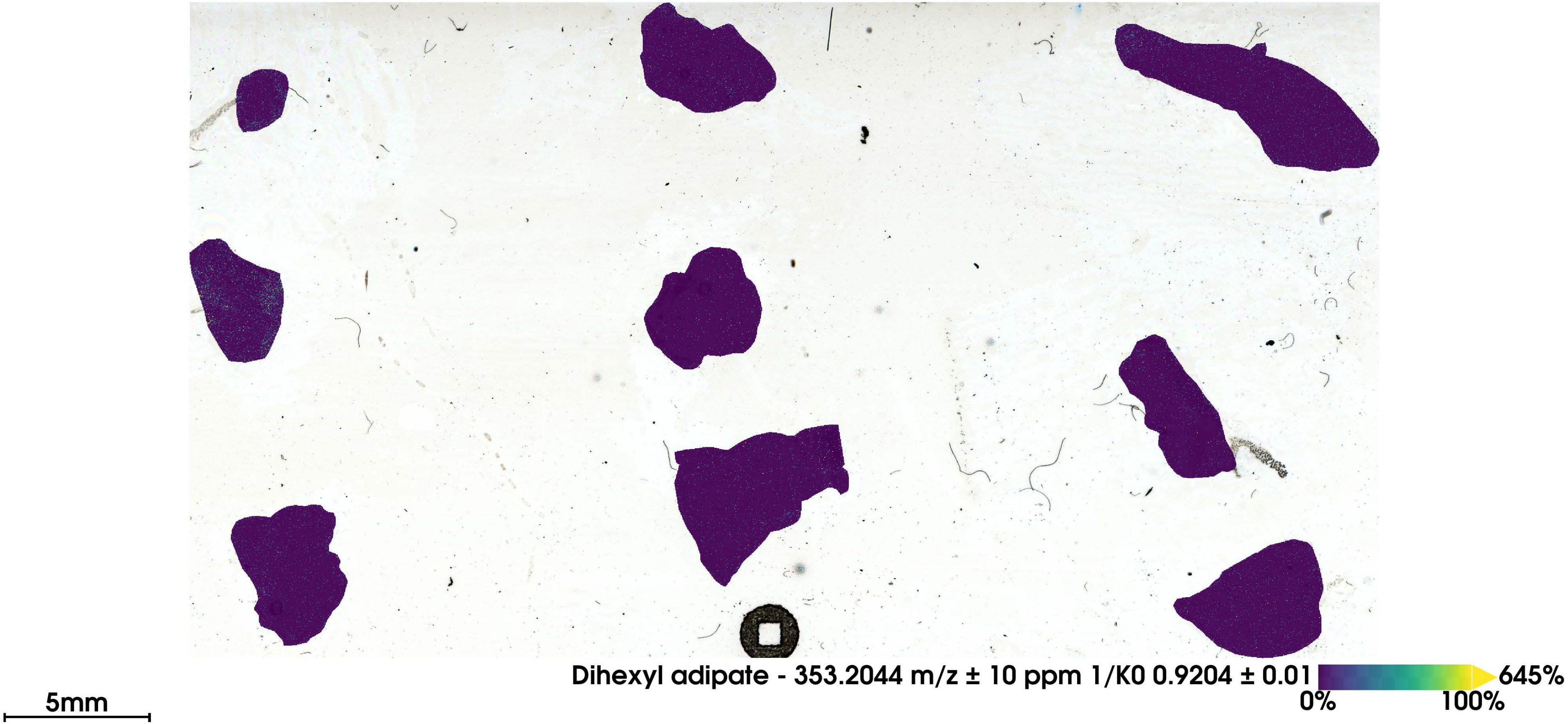

Dihexyl adipate - 353.2044 m/z ± 10 ppm 1/K0 0.9204 ± 0.01
645%
0%
100%
5mm

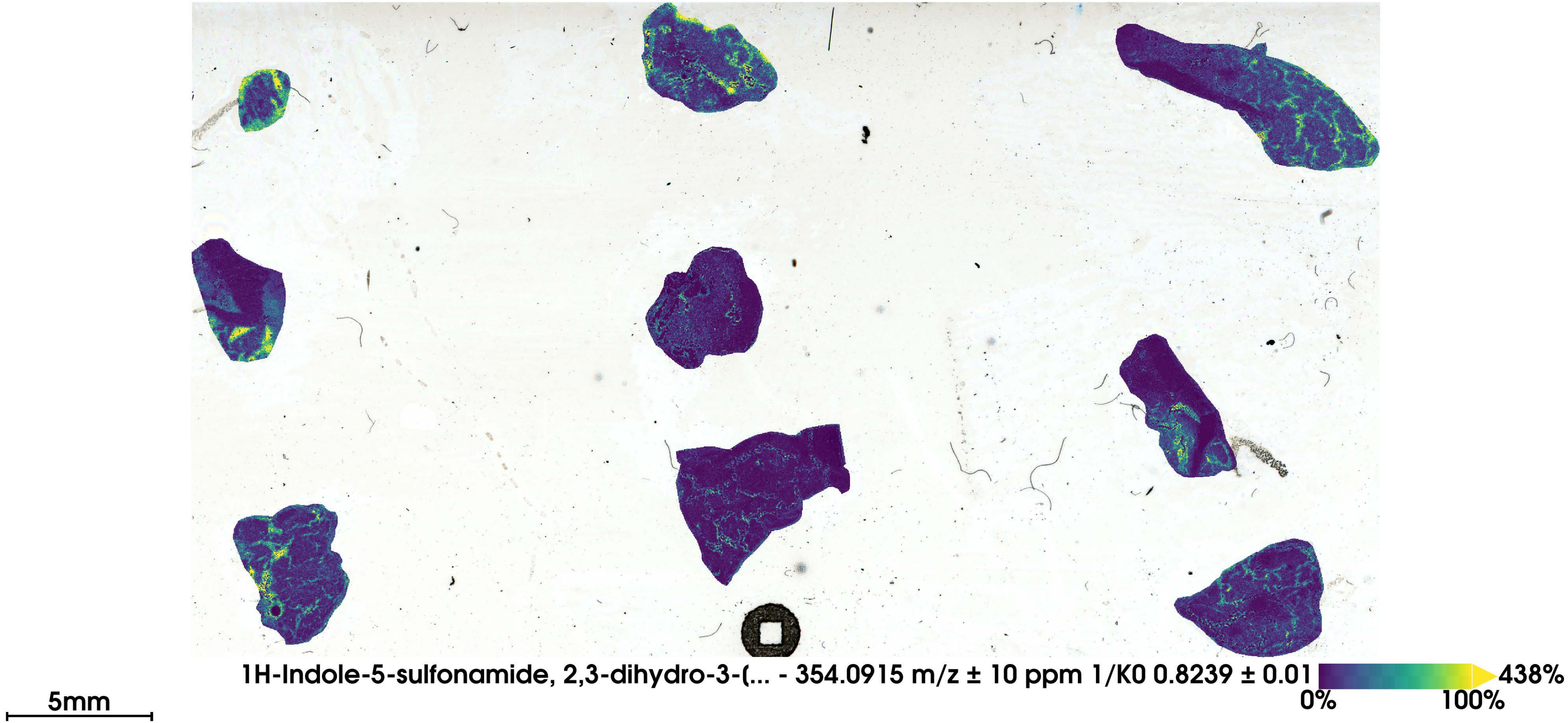
1H-Indole-5-sulfonamide, 2,3-dihydro-3-(... - 354.0915 m/z ± 10 ppm 1/K0 0.8239 ± 0.01
0%
100%
438%
5mm

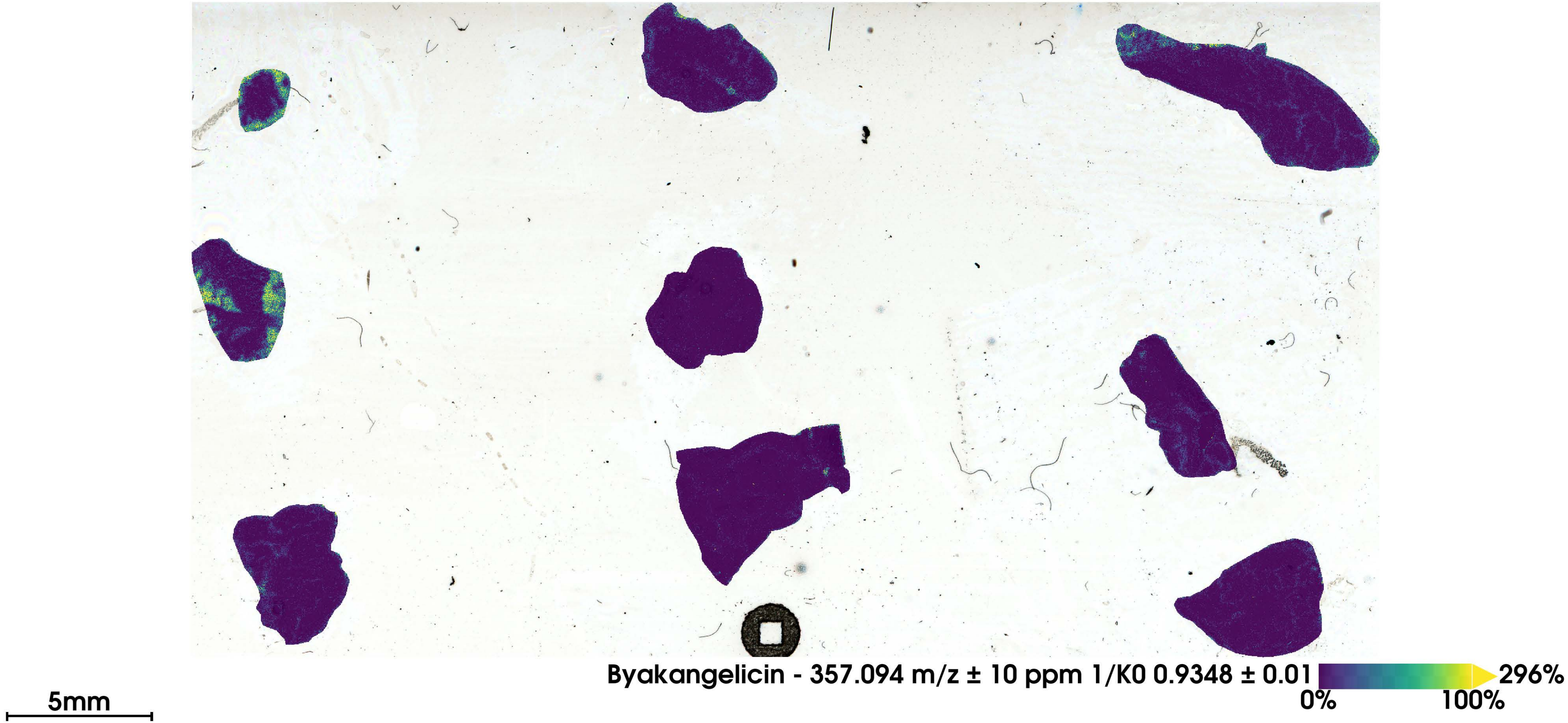

Byakangelicin - 357.094 m/z ± 10 ppm 1/K0 0.9348 ± 0.01
296%
0%
100%
5mm

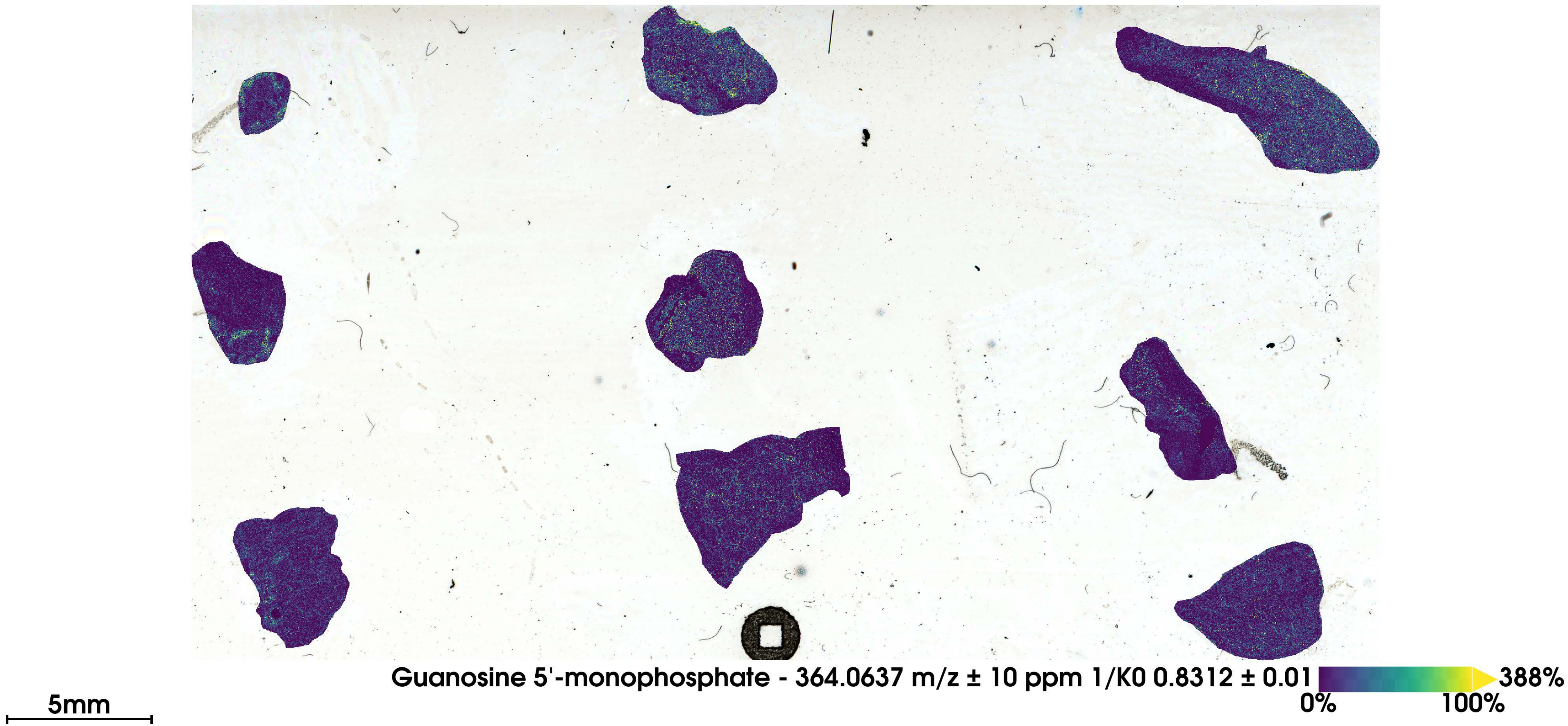
5mm
Guanosine 5'-monophosphate - 364.0637 m/z ± 10 ppm 1/K0 0.8312 ± 0.01
388%
0%
100%

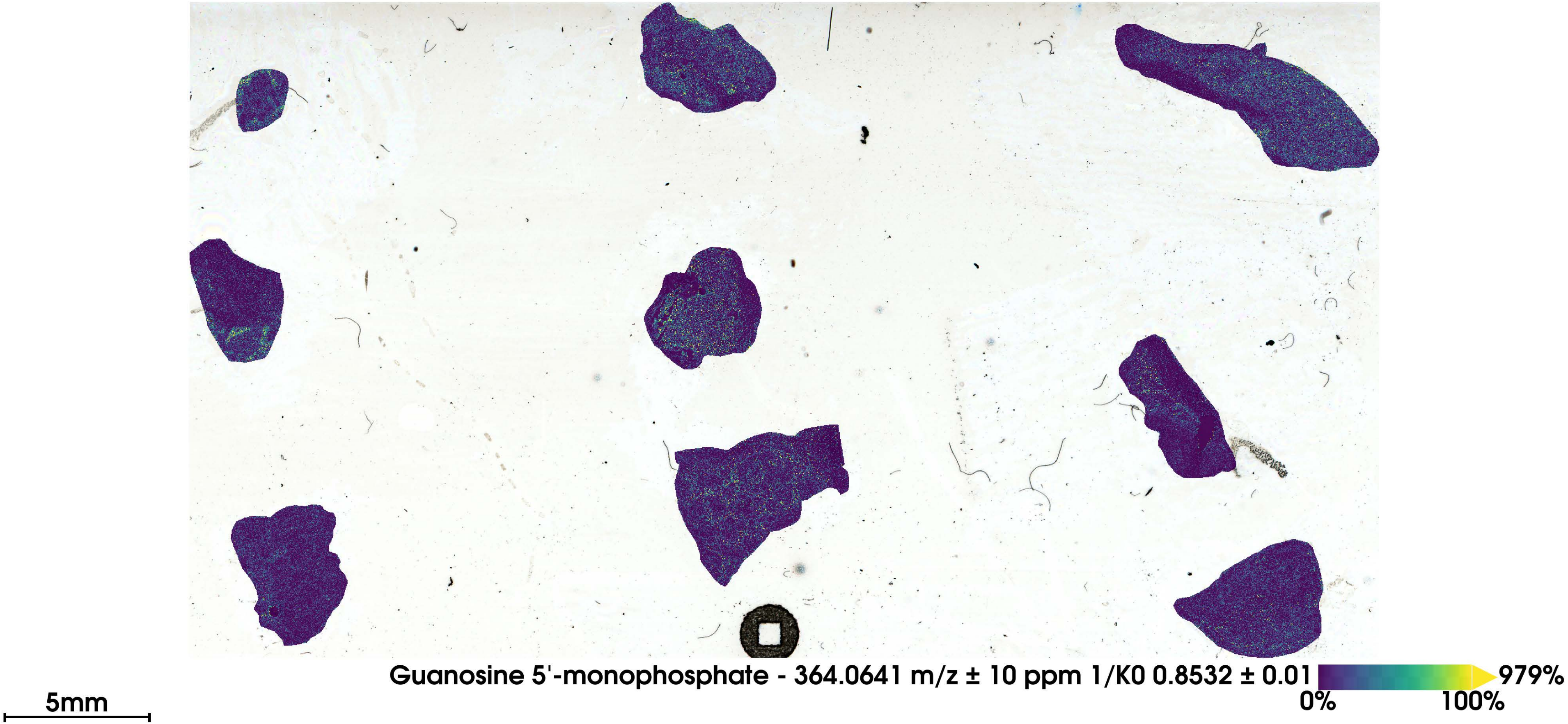
Guanosine 5'-monophosphate - 364.0641 m/z ± 10 ppm 1/K0 0.8532 ± 0.01
979%
0%
100%
5mm

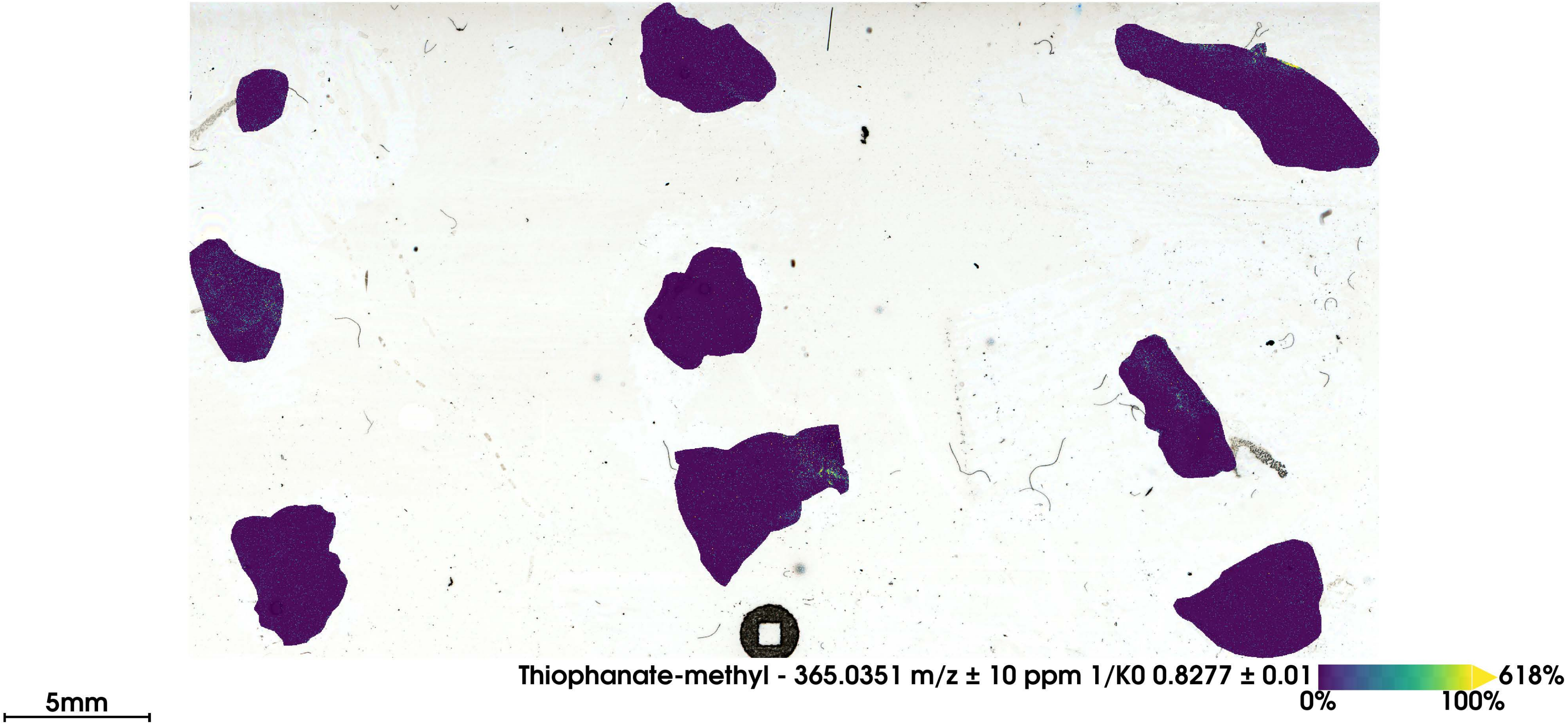
Thiophanate-methyl - 365.0351 m/z ± 10 ppm 1/K0 0.8277 ± 0.01
618%
0%
100%
5mm

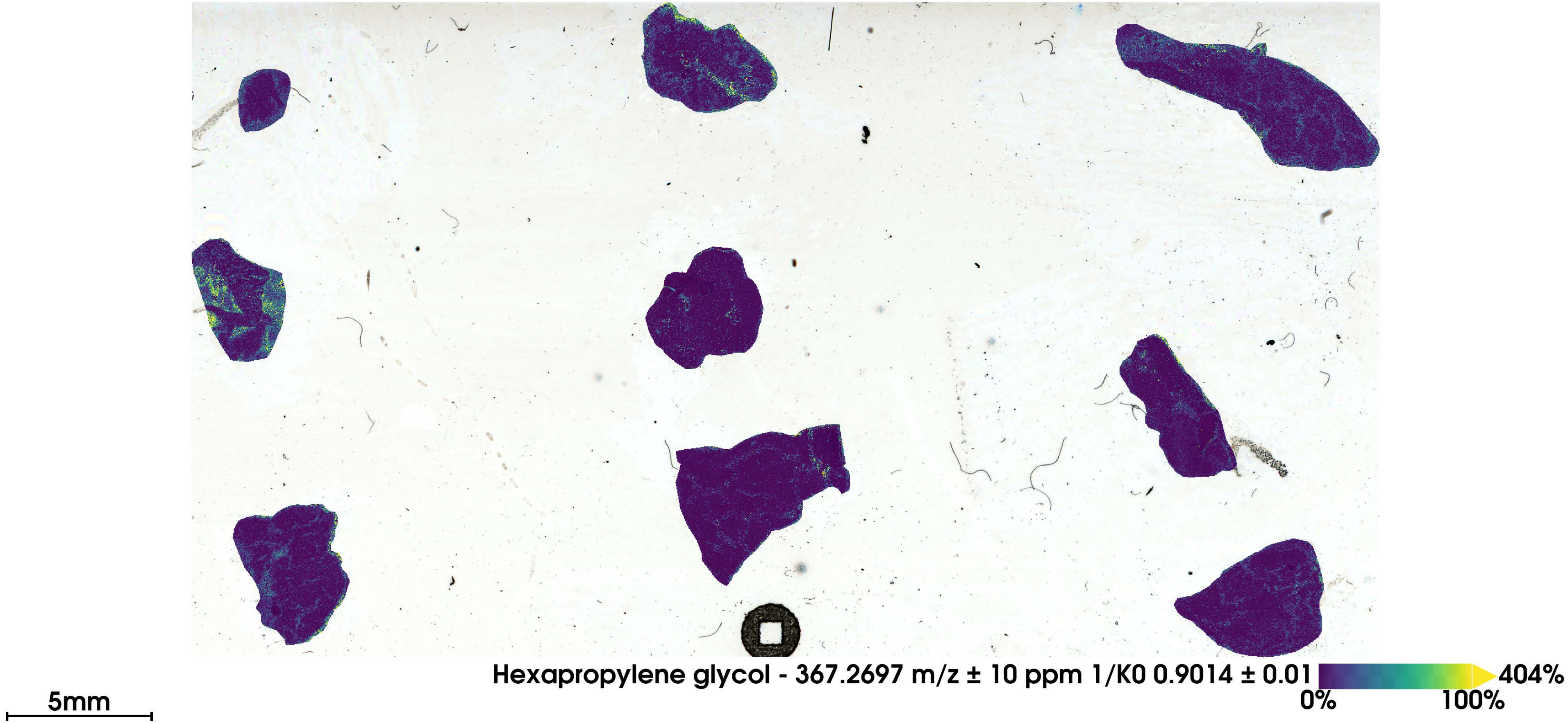
Hexapropylene glycol - 367.2697 m/z ± 10 ppm 1/K0 0.9014 ± 0.01
404%
0%
100%
5mm

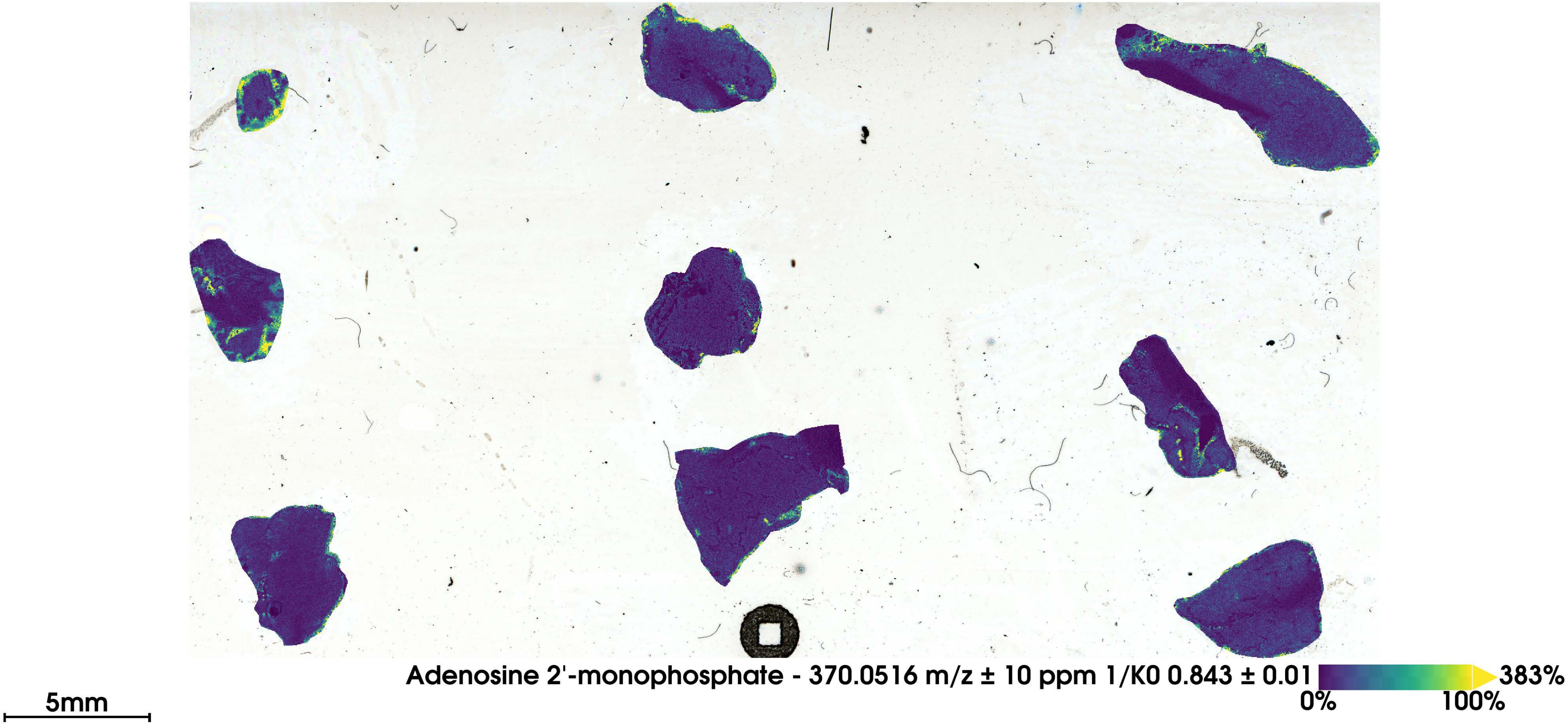
Adenosine 2'-monophosphate - 370.0516 m/z ± 10 ppm 1/K0 0.843 ± 0.01
383%
0%
100%
5mm

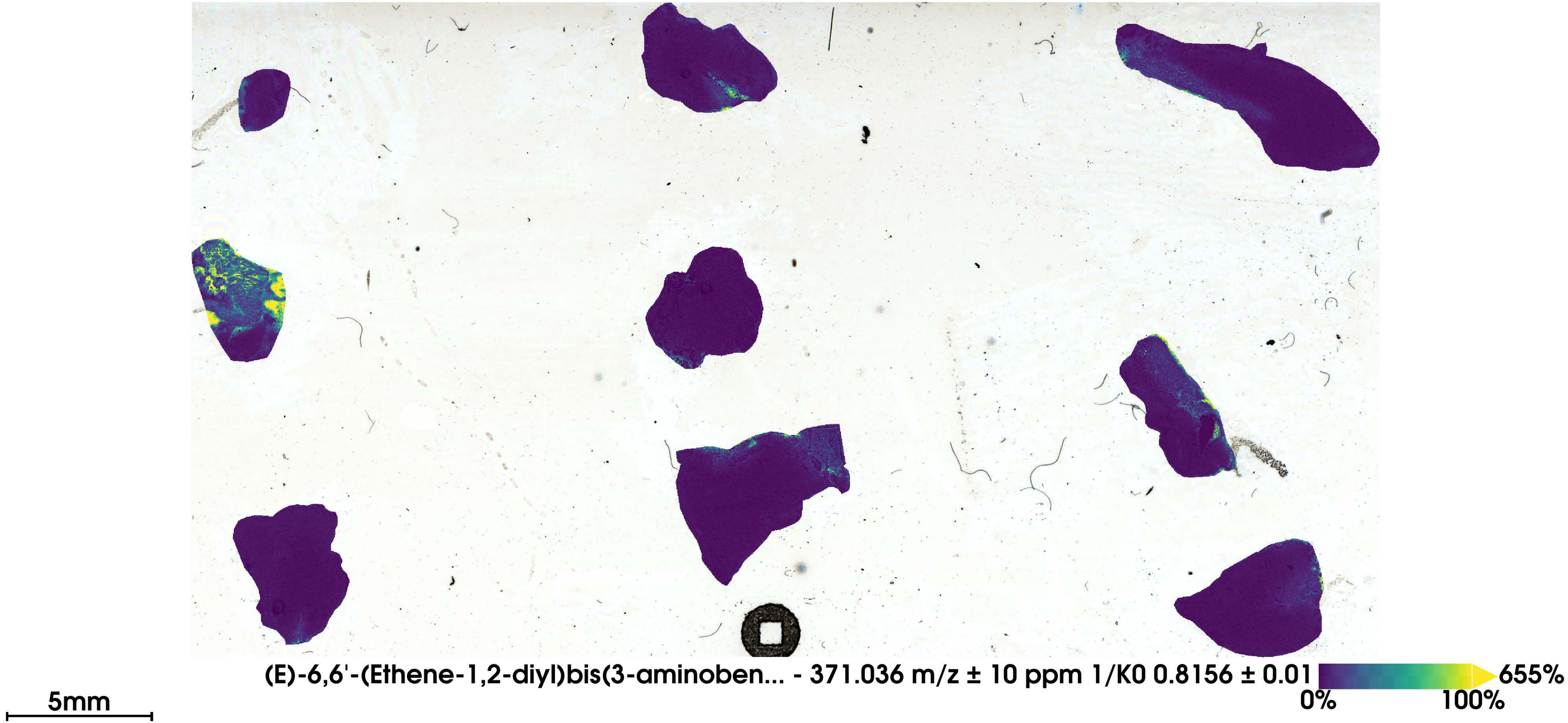
(E)-6,6'-(Ethene-1,2-diyl)bis(3-aminoben... - 371.036 m/z ± 10 ppm 1/K0 0.8156 ± 0.01
655%
0%
100%
5mm

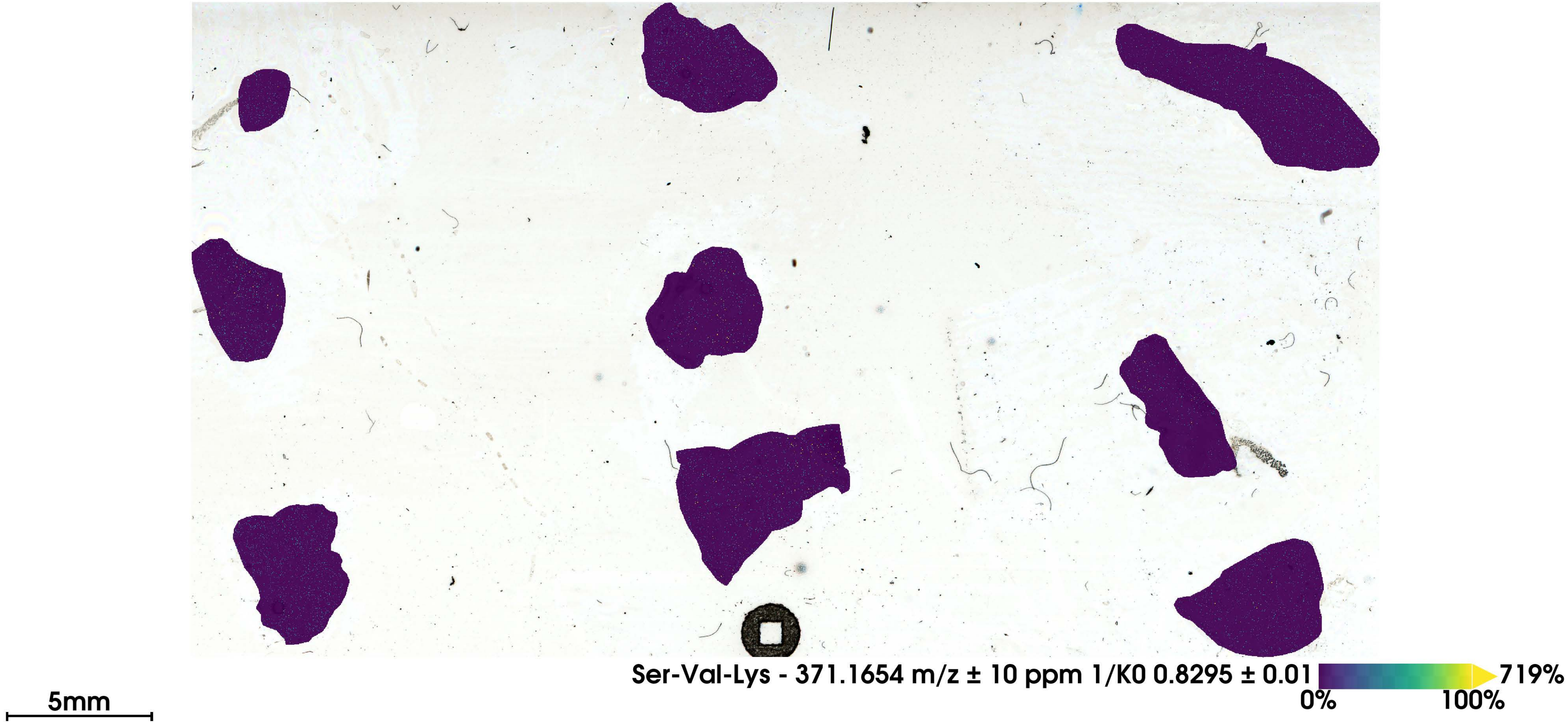

Ser-Val-Lys - 371.1654 m/z ± 10 ppm 1/K0 0.8295 ± 0.01
719%
0%
100%
5mm

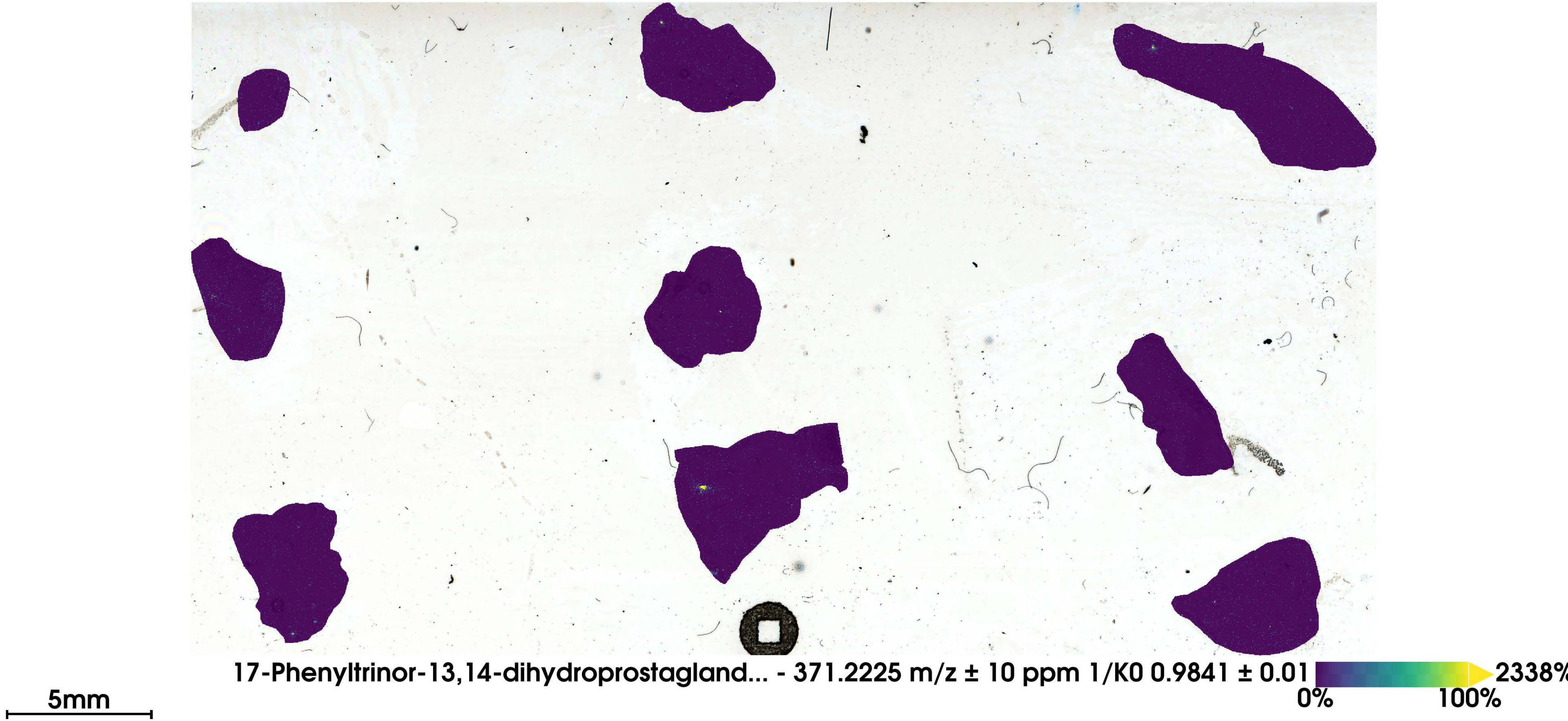
17-Phenyltrinor-13,14-dihydroprostagland... - 371.2225 m/z ± 10 ppm 1/K0 0.9841 ± 0.01
2338%
0%
100%
5mm

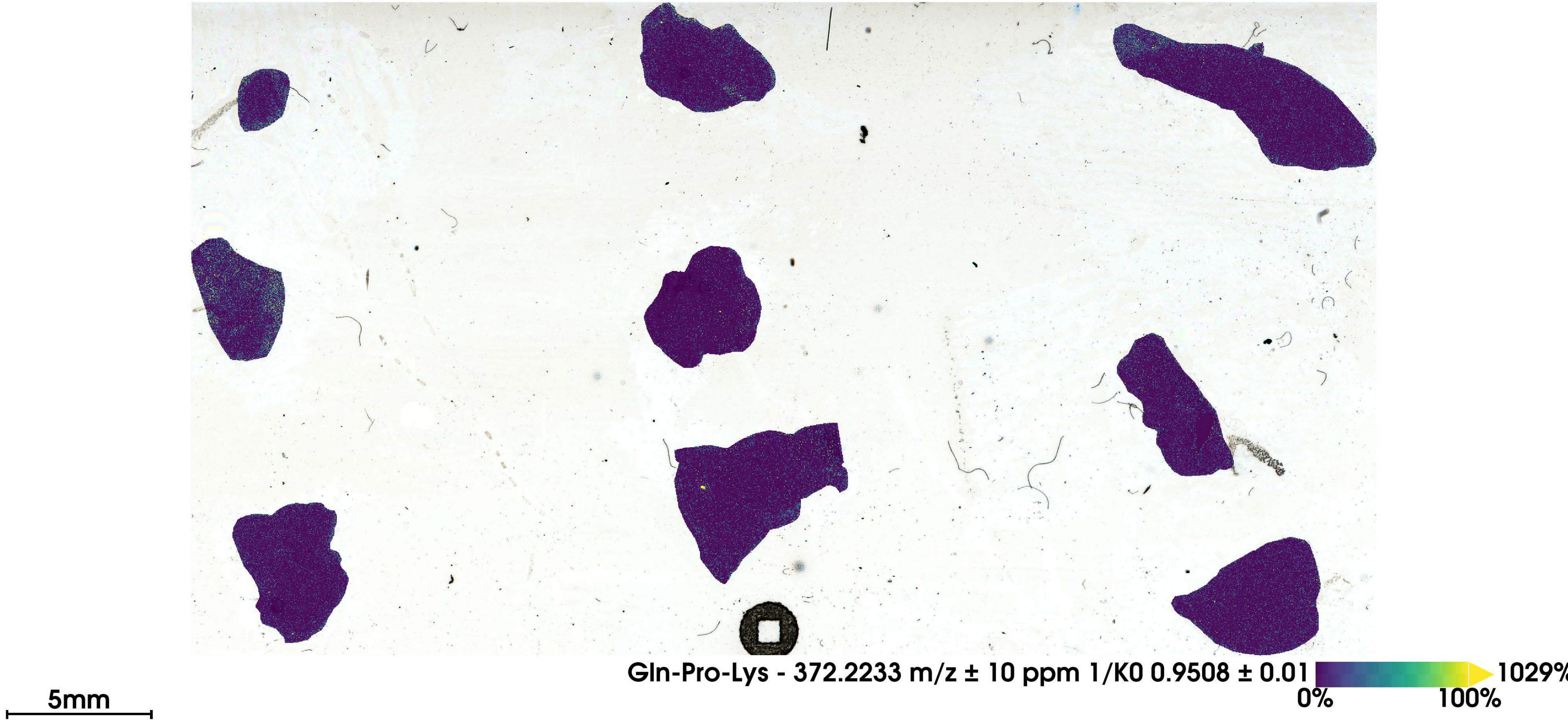

Gln-Pro-Lys - 372.2233 m/z ± 10 ppm 1/K0 0.9508 ± 0.01
1029%
0%
100%
5mm

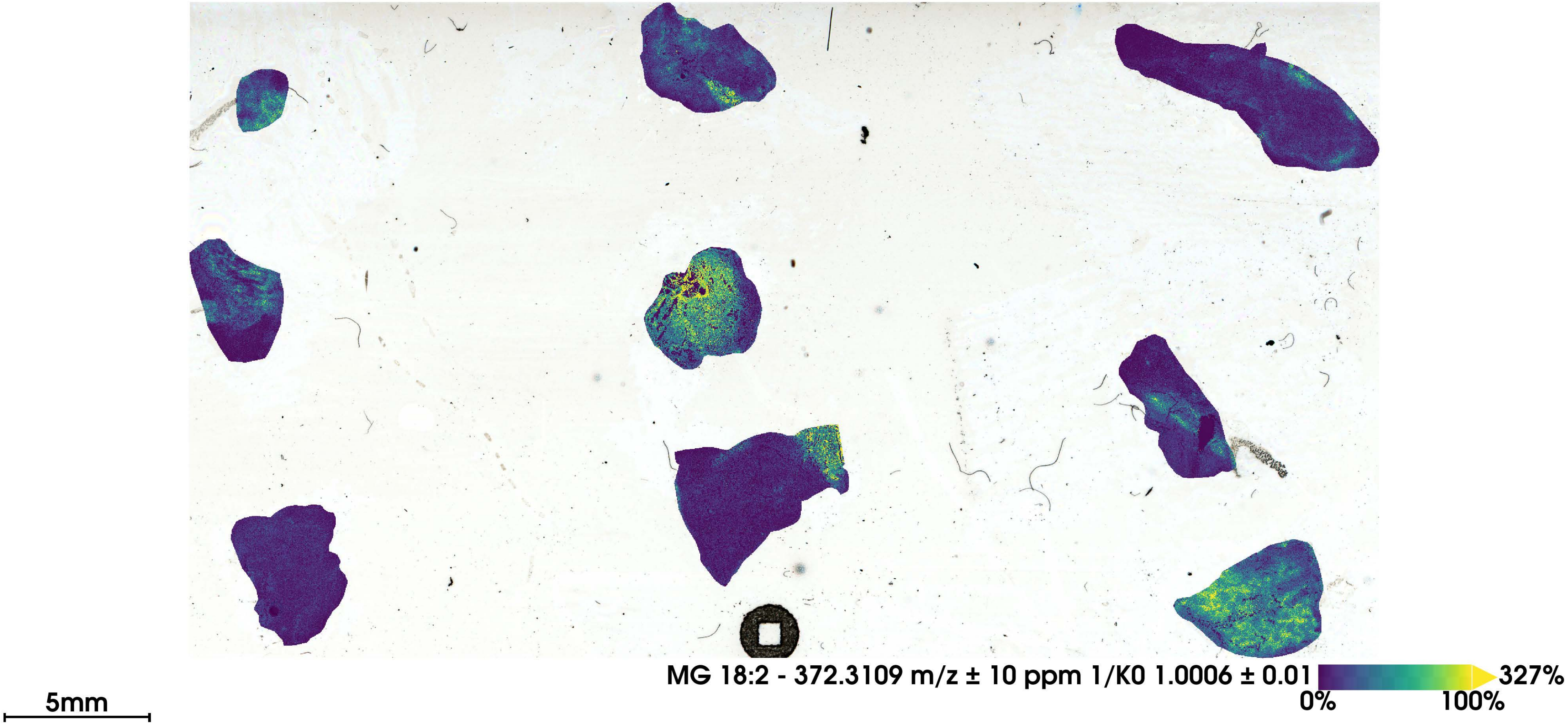
MG 18:2 - 372.3109 m/z ± 10 ppm 1/K0 1.0006 ± 0.01
327%
0%
100%
5mm

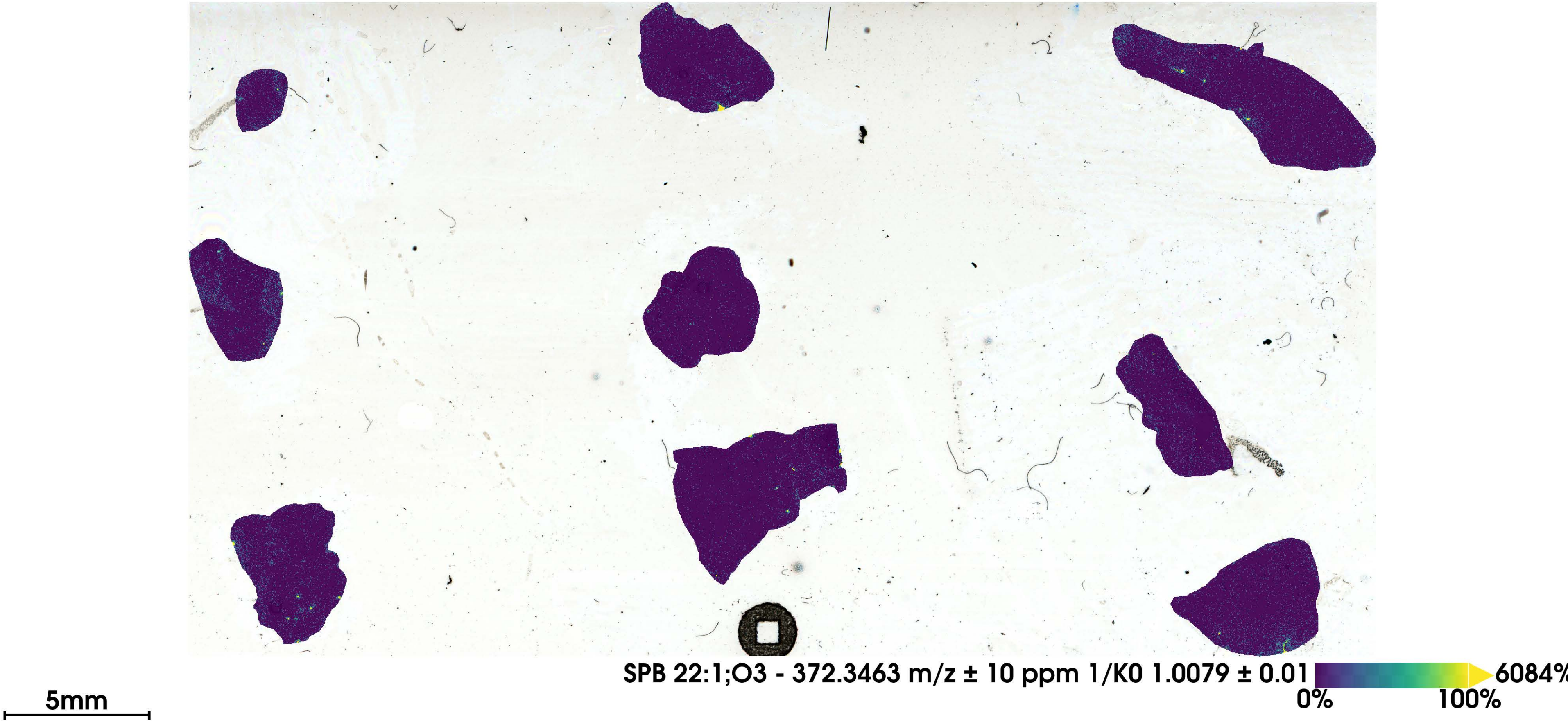
SPB 22:1;O3 - 372.3463 m/z ± 10 ppm 1/K0 1.0079 ± 0.01
6084%
0%
100%
5mm

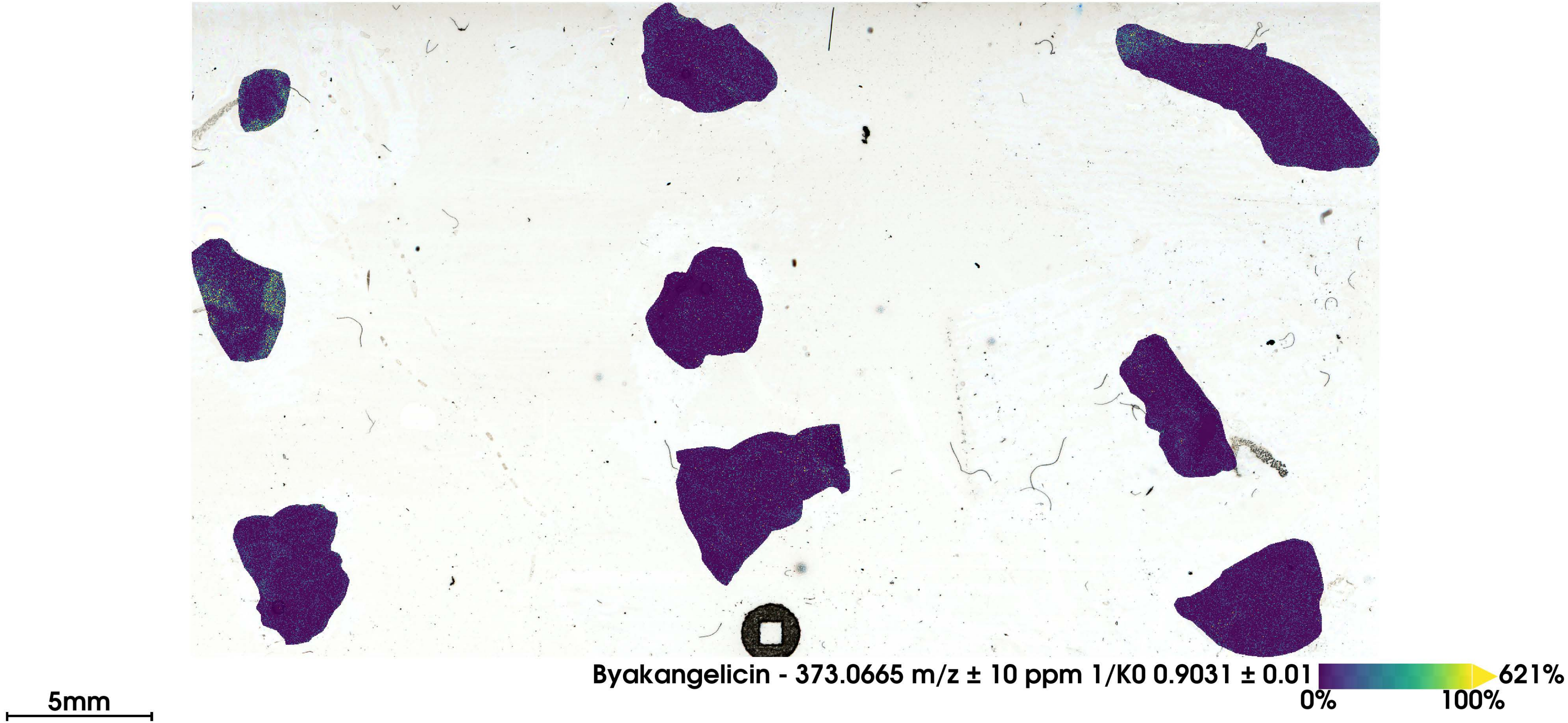

Byakangelicin - 373.0665 m/z ± 10 ppm 1/K0 0.9031 ± 0.01
621%
0%
100%
5mm

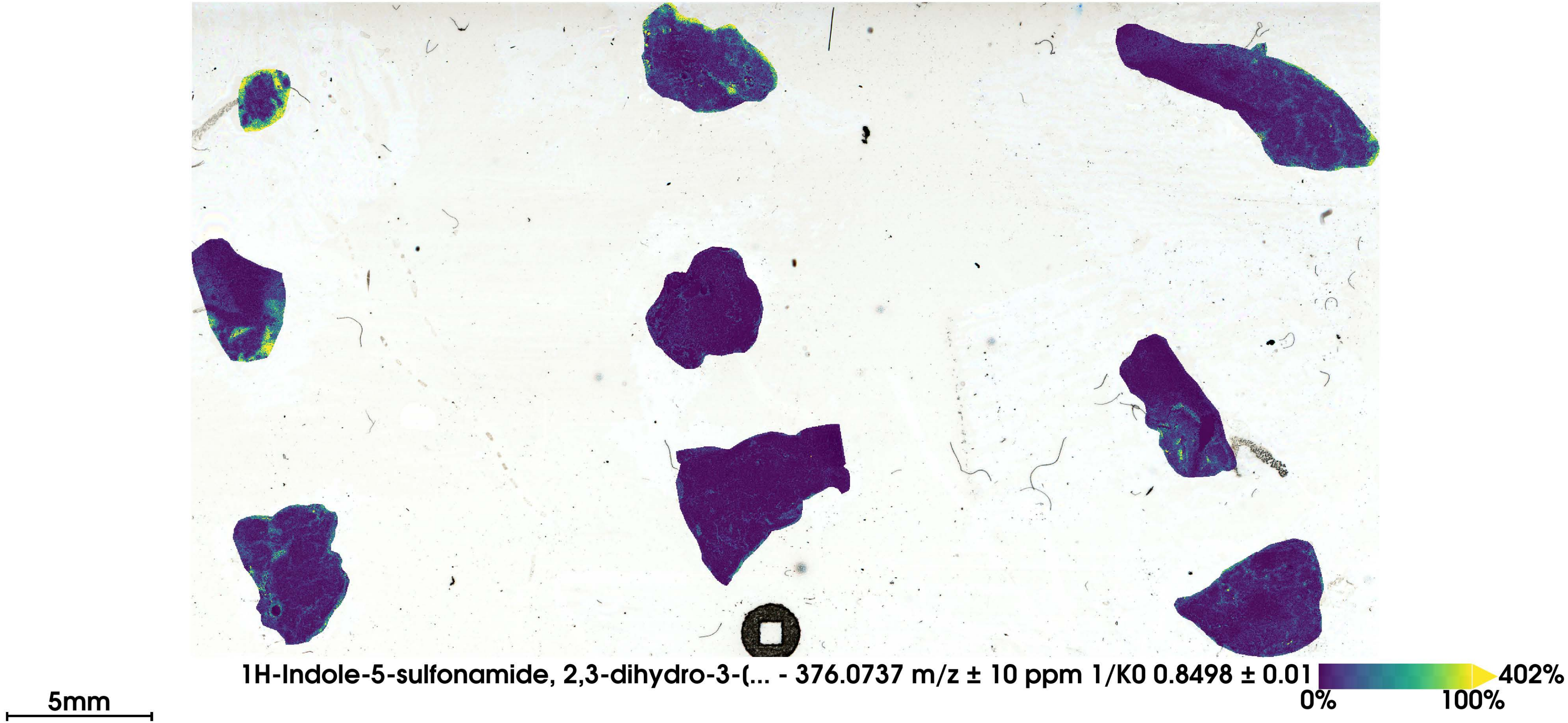
1H-Indole-5-sulfonamide, 2,3-dihydro-3-(... - 376.0737 m/z ± 10 ppm 1/K0 0.8498 ± 0.01
402%
0%
100%
5mm

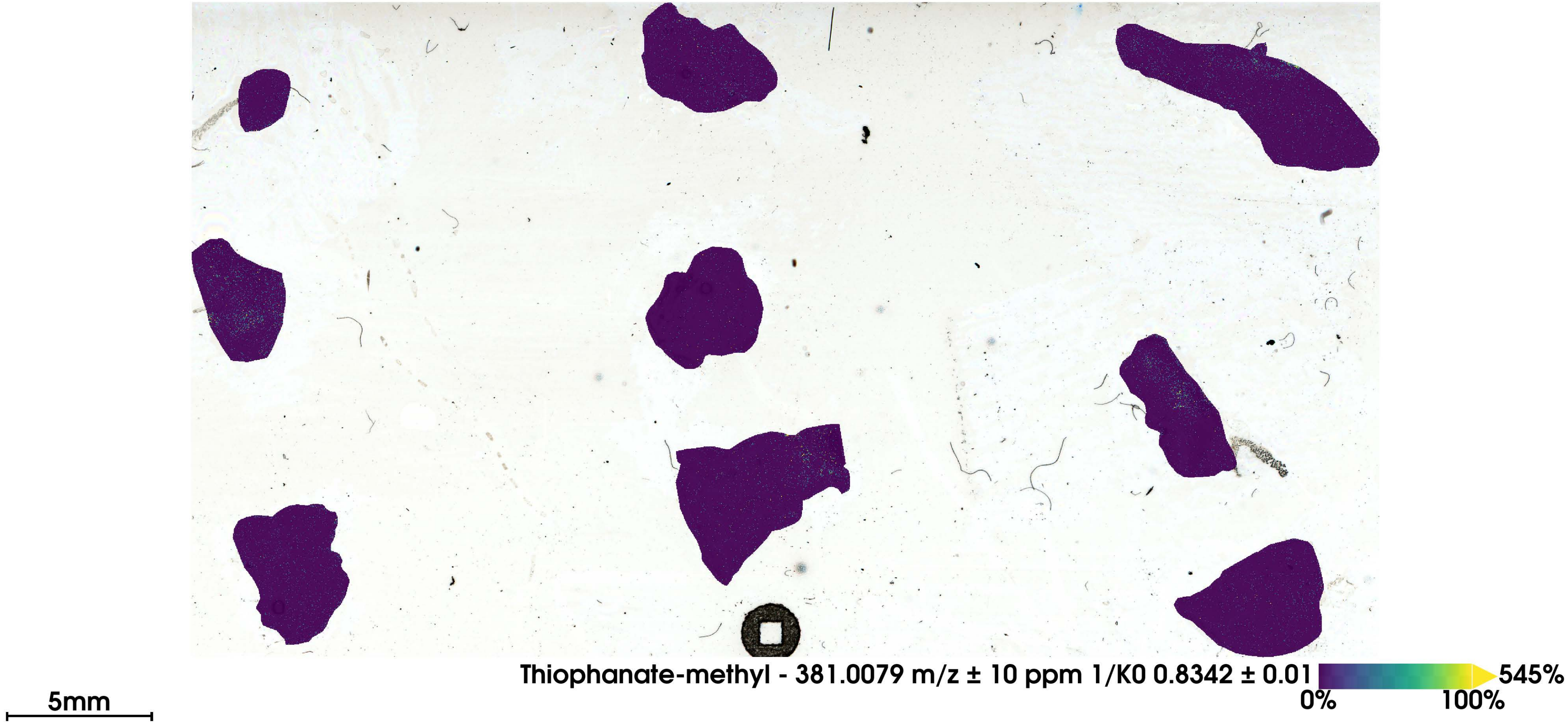
Thiophanate-methyl - 381.0079 m/z ± 10 ppm 1/K0 0.8342 ± 0.01
545%
0%
100%
5mm

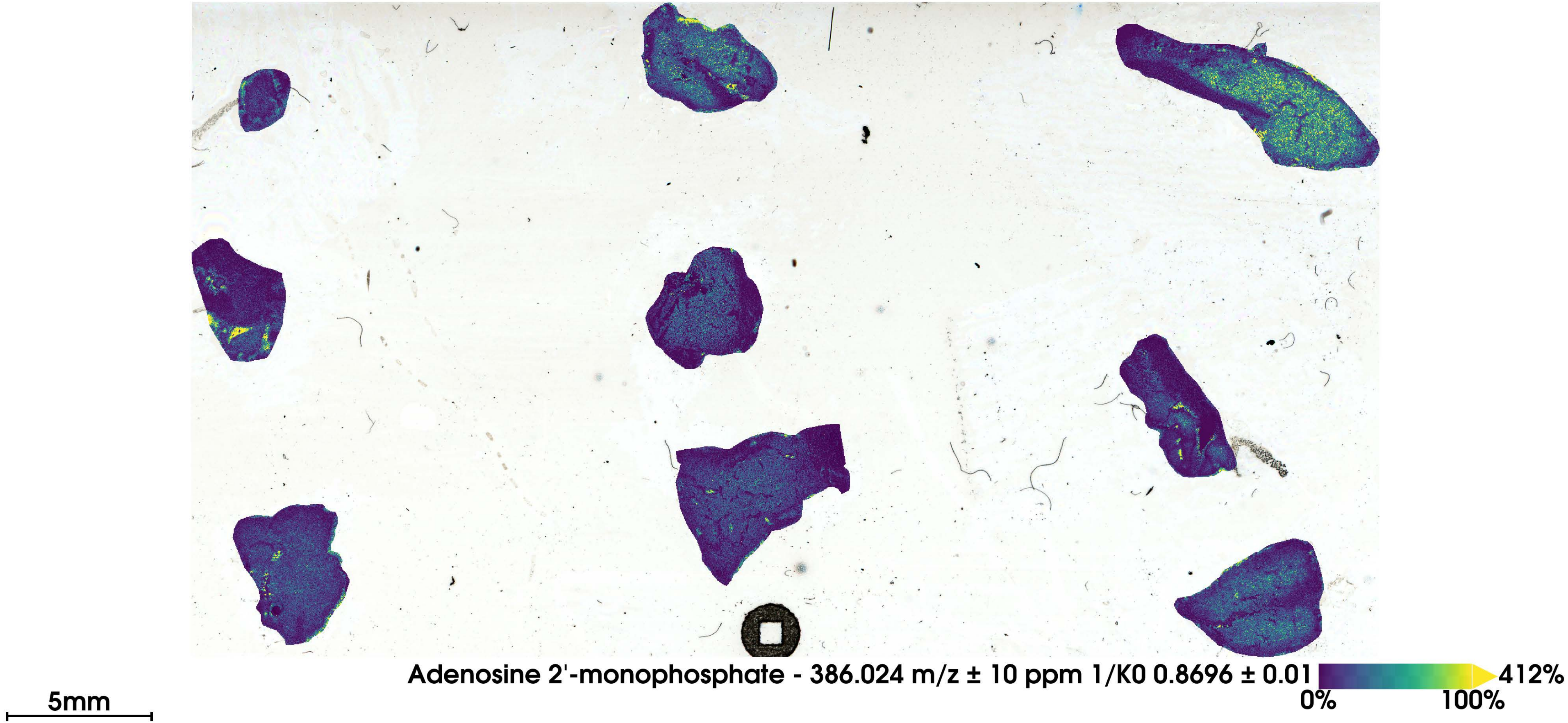

Adenosine 2'-monophosphate - 386.024 m/z ± 10 ppm 1/K0 0.8696 ± 0.01
412%
0%
100%
5mm

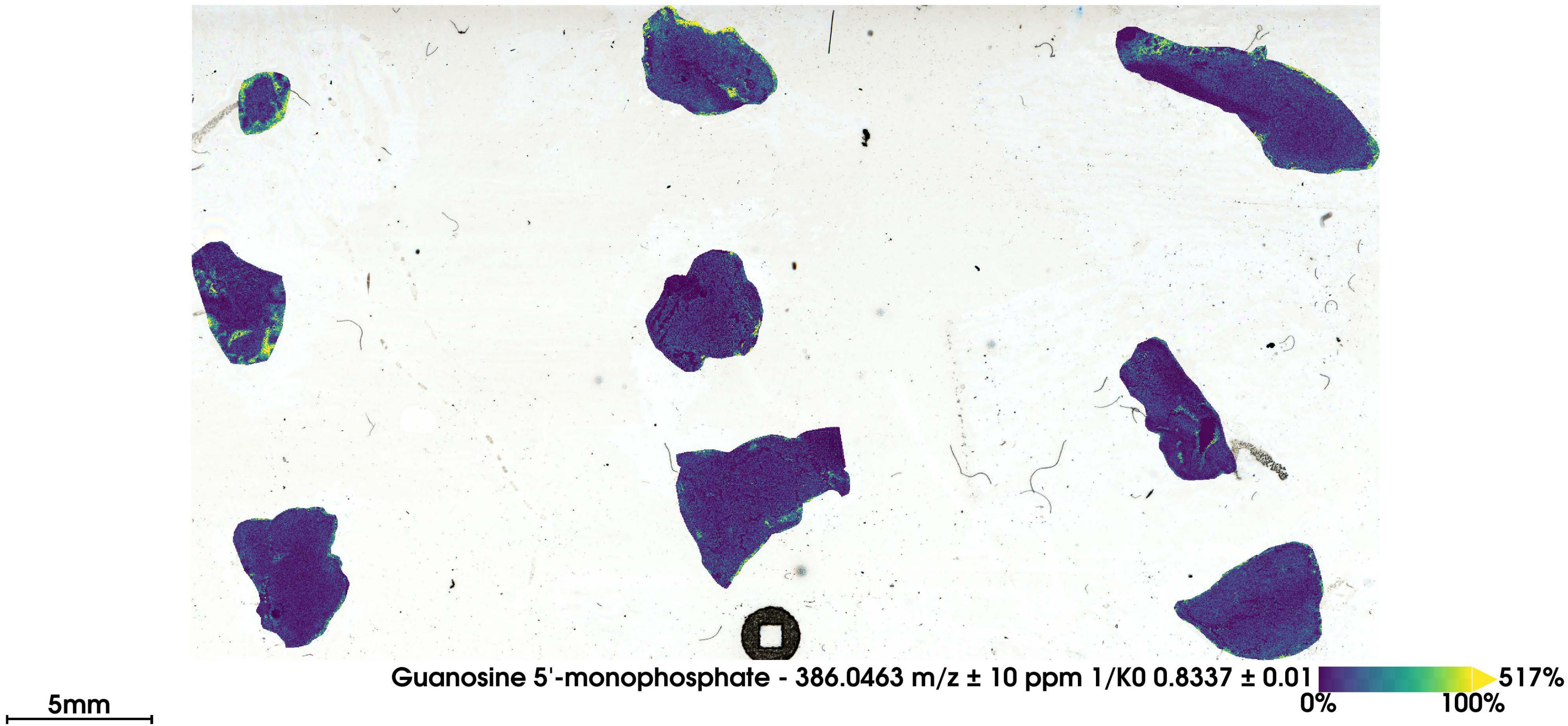
Guanosine 5'-monophosphate - 386.0463 m/z ± 10 ppm 1/K0 0.8337 ± 0.01
517%
0%
100%
5mm

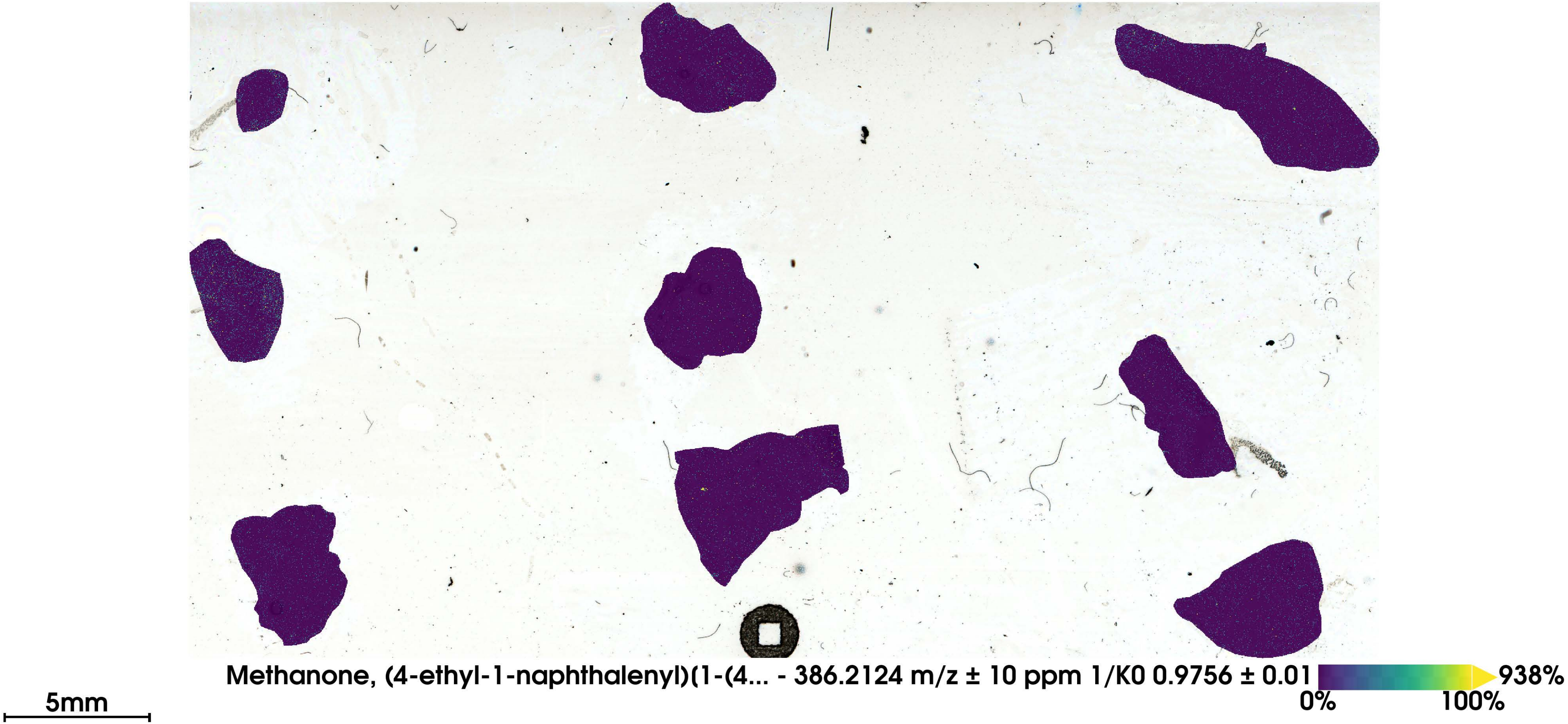
Methanone, (4-ethyl-1-naphthalenyl)(1-(4... - 386.2124 m/z ± 10 ppm 1/K0 0.9756 ± 0.01
938%
0%
100%
5mm

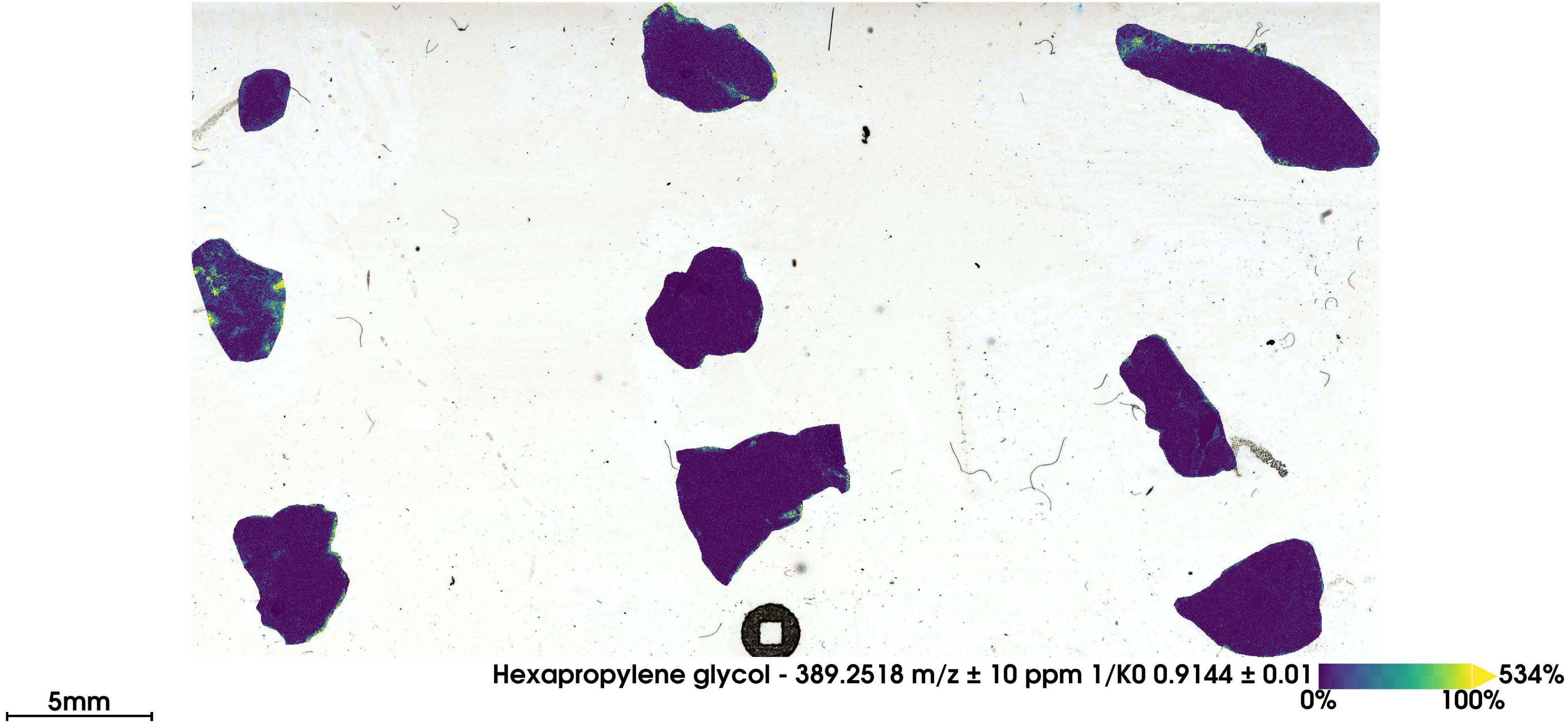

Hexapropylene glycol - 389.2518 m/z ± 10 ppm 1/K0 0.9144 ± 0.01
534%
0%
100%
5mm

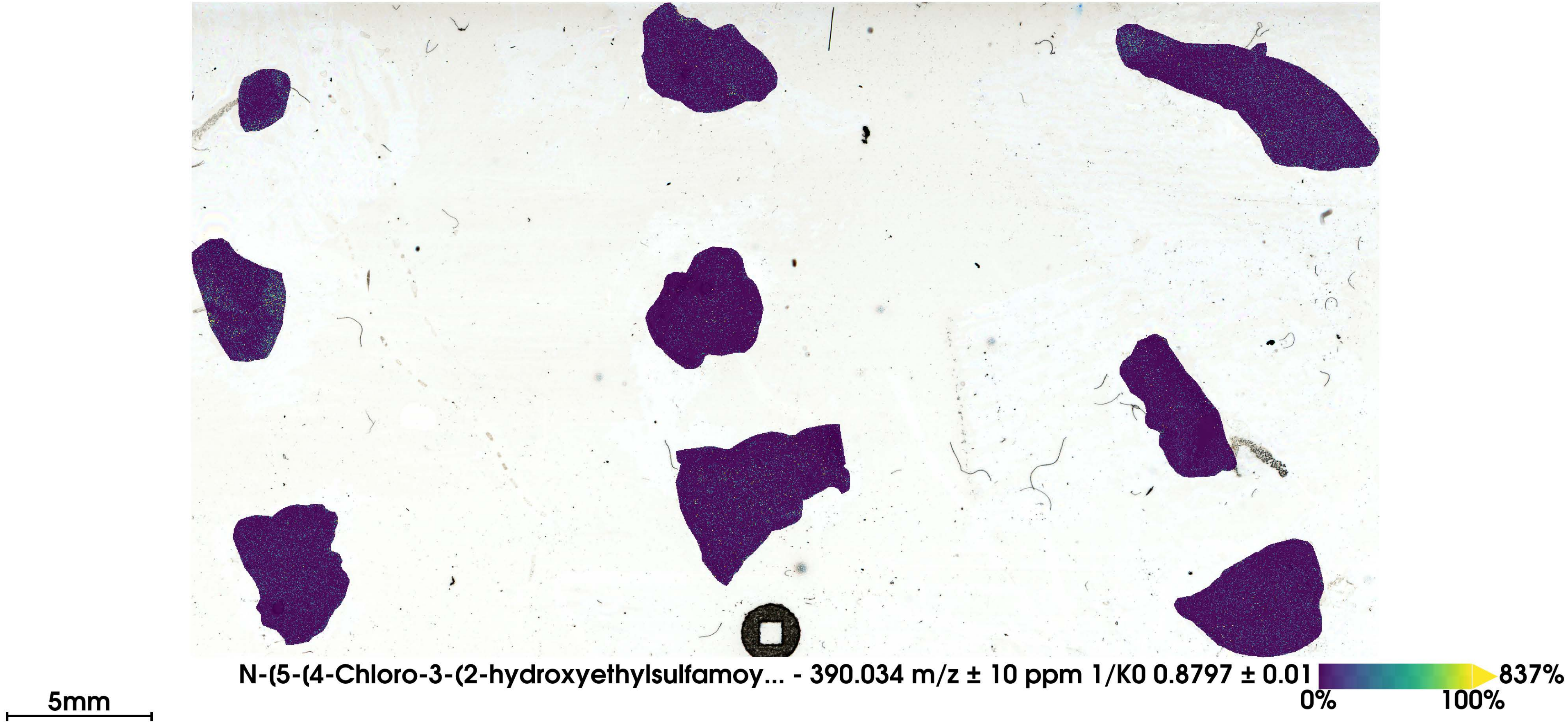
N-(5-(4-Chloro-3-(2-hydroxyethylsulfamoy... - 390.034 m/z ± 10 ppm 1/K0 0.8797 ± 0.01
837%
0%
100%
5mm

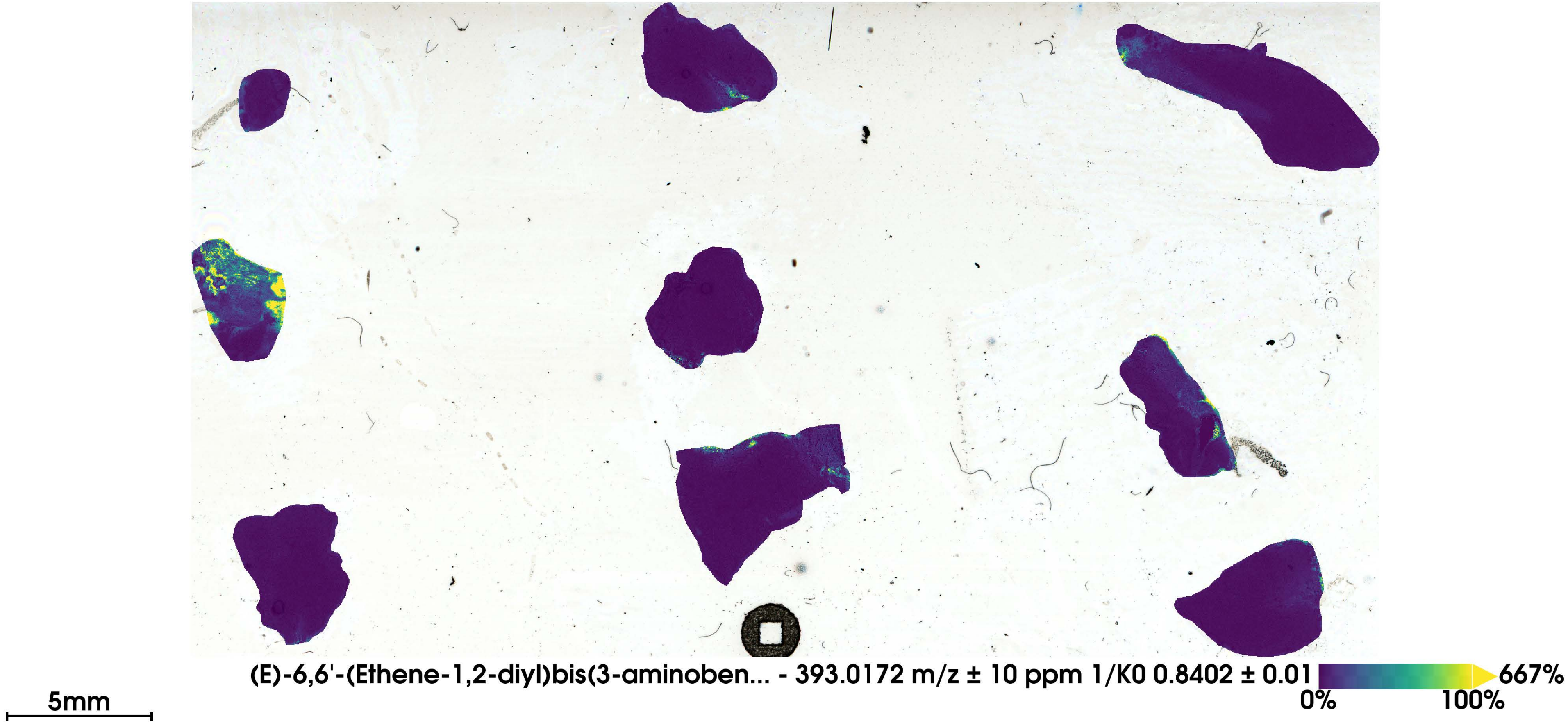
5mm
(E)-6,6'-(Ethene-1,2-diyl)bis(3-aminoben... - 393.0172 m/z ± 10 ppm 1/K0 0.8402 ± 0.01
667%
0%
100%

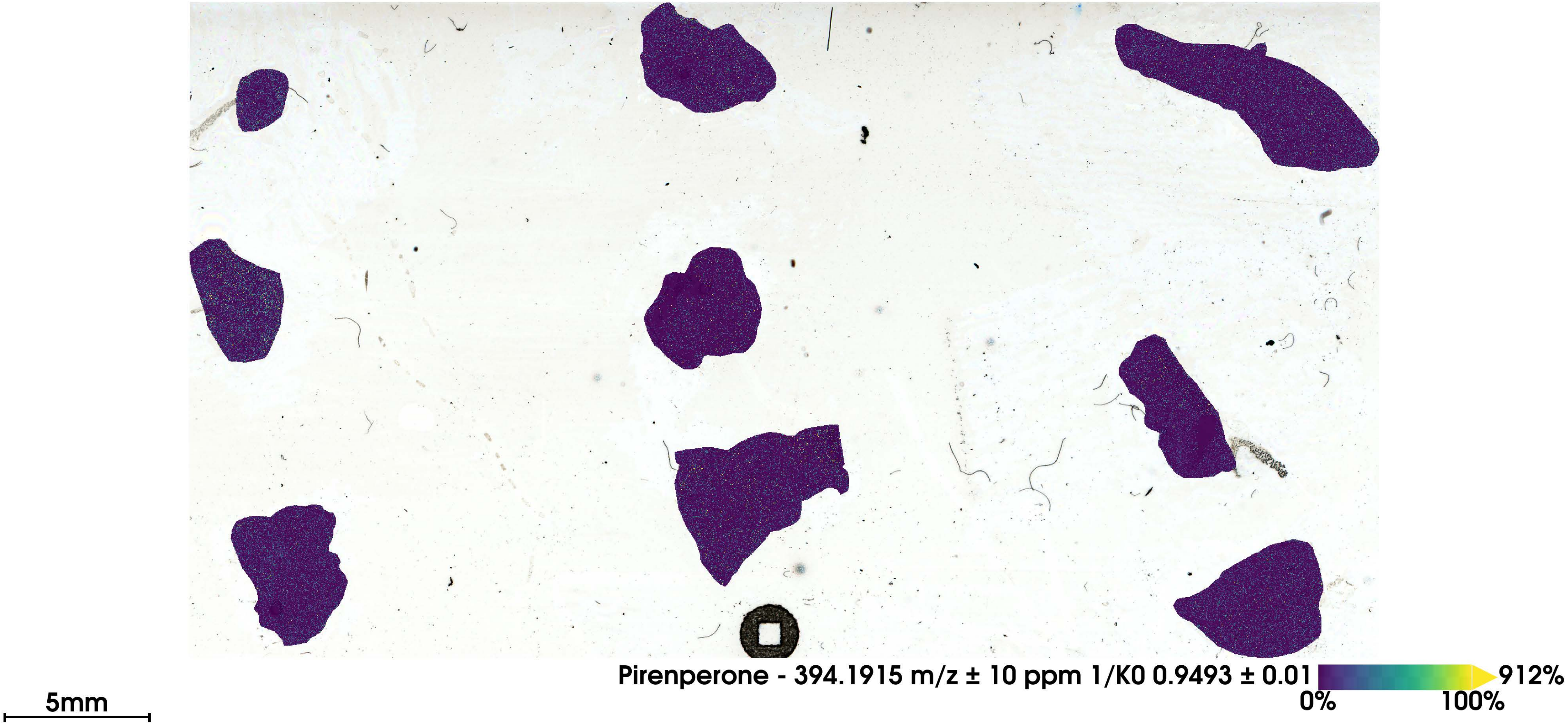

Pirenperone - 394.1915 m/z ± 10 ppm 1/K0 0.9493 ± 0.01
912%
0%
100%
5mm

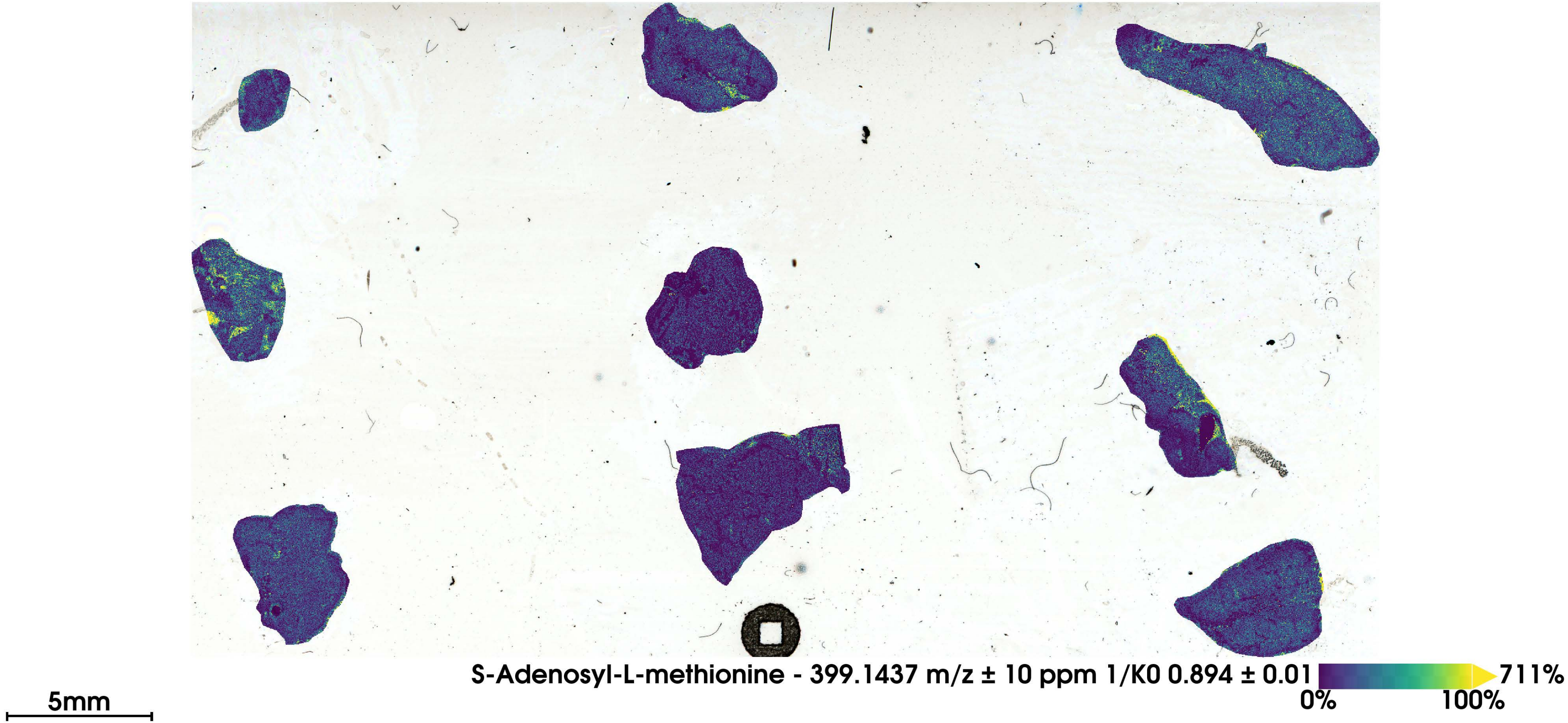
S-Adenosyl-L-methionine - 399.1437 m/z ± 10 ppm 1/K0 0.894 ± 0.01
711%
0%
100%
5mm

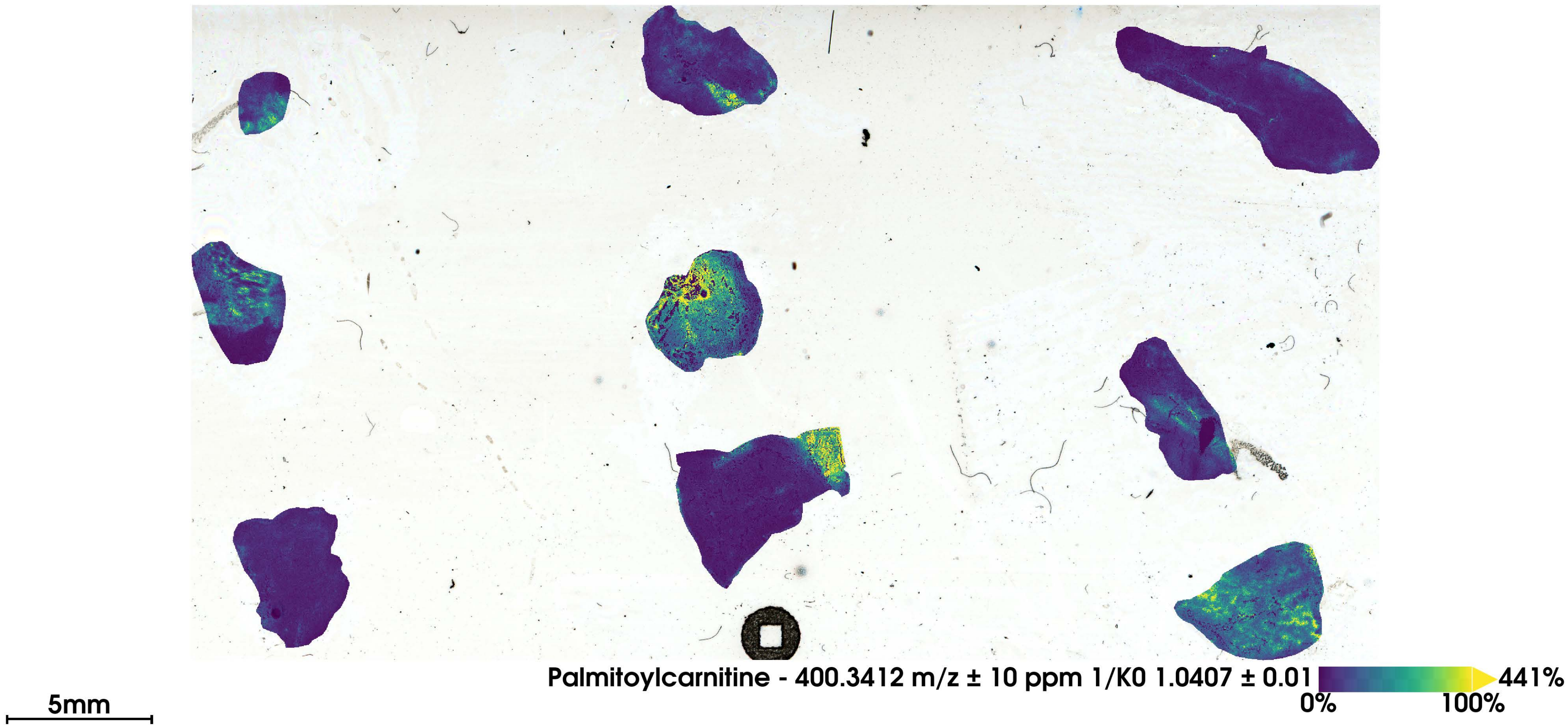

Palmitoylcarnitine - 400.3412 m/z ± 10 ppm 1/K0 1.0407 ± 0.01
441%
0%
100%
5mm

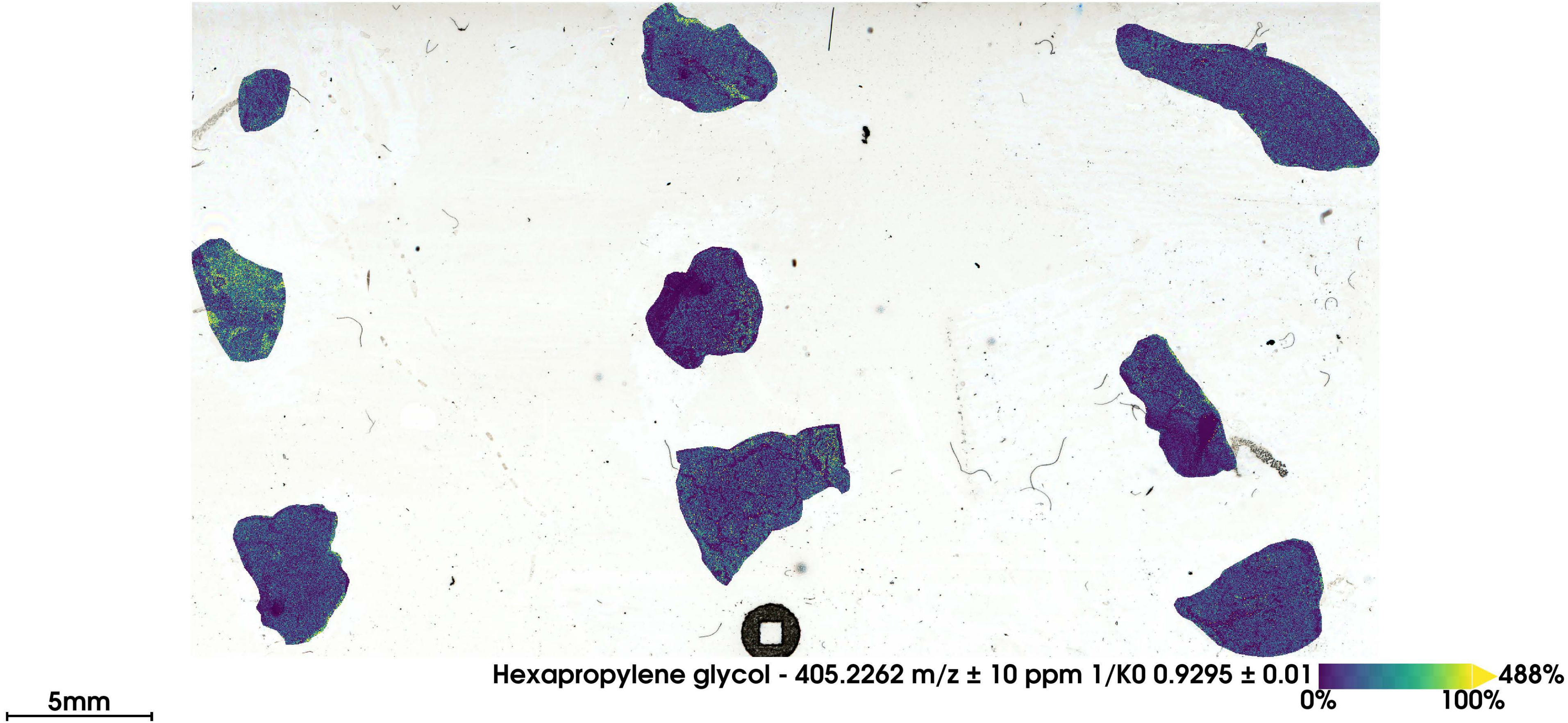
Hexapropylene glycol - 405.2262 m/z ± 10 ppm 1/K0 0.9295 ± 0.01
488%
0%
100%
5mm

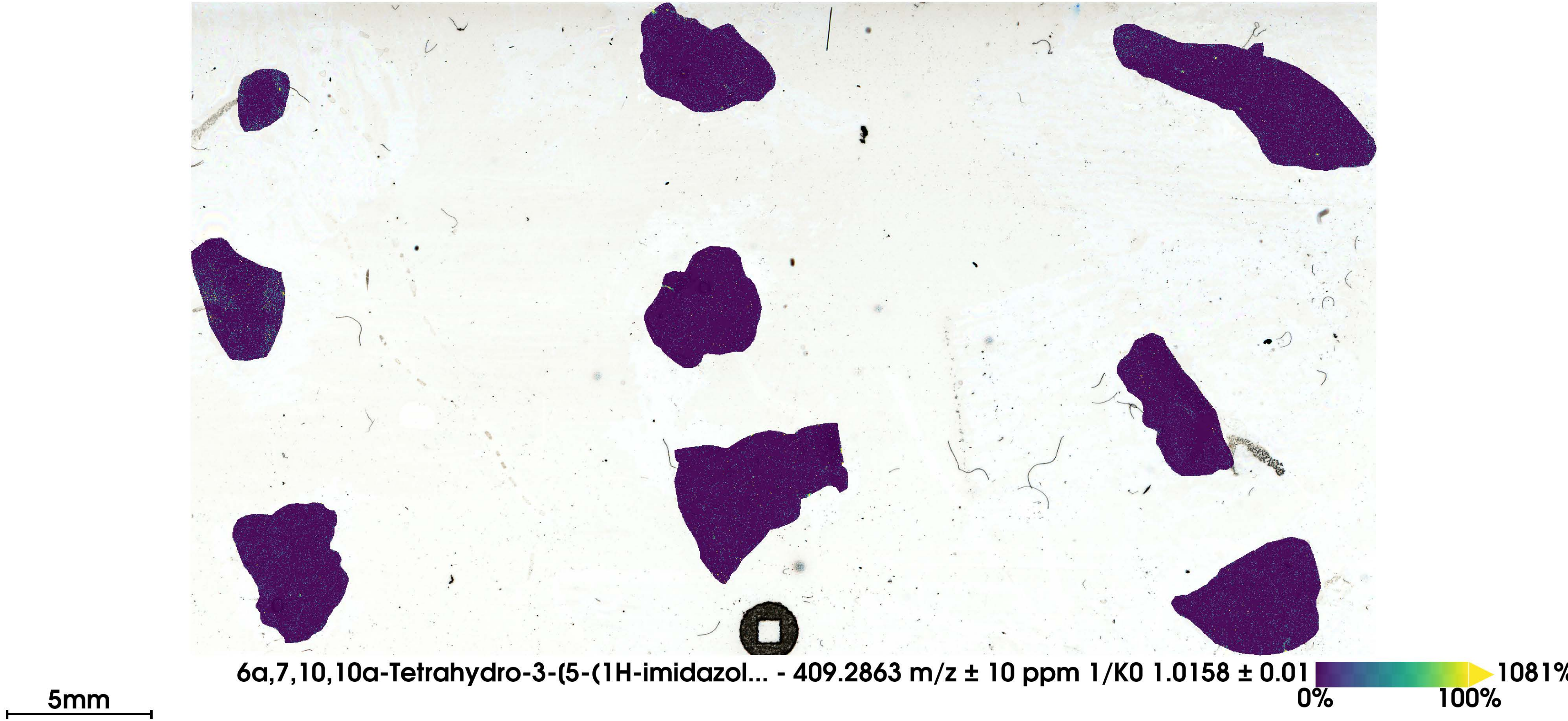
6a,7,10,10a-Tetrahydro-3-(5-(1H-imidazol... - 409.2863 m/z ± 10 ppm 1/K0 1.0158 ± 0.01
1081%
0%
100%
5mm

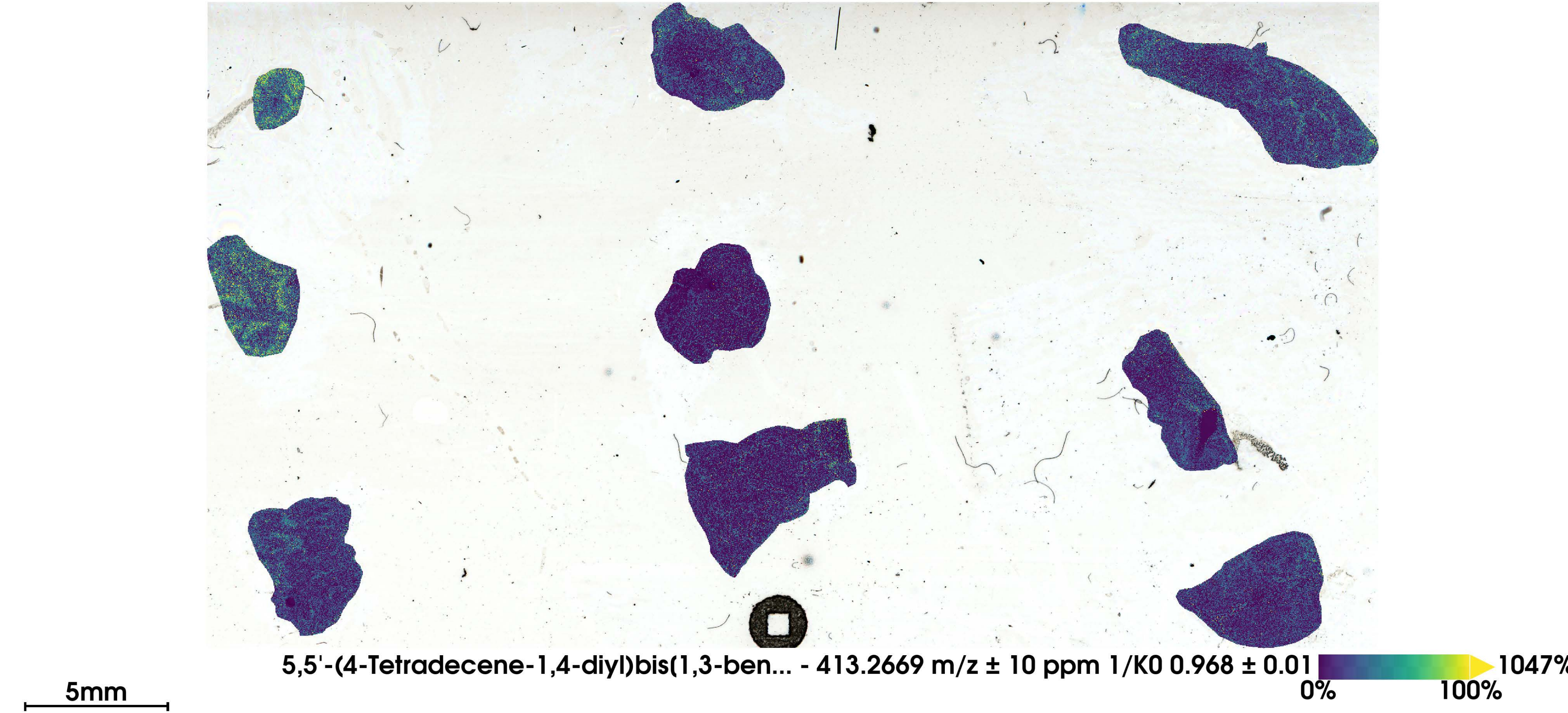

5,5'-(4-Tetradecene-1,4-diyl)bis(1,3-ben... - 413.2669 m/z ± 10 ppm 1/K0 0.968 ± 0.01
1047%
0%
100%
5mm

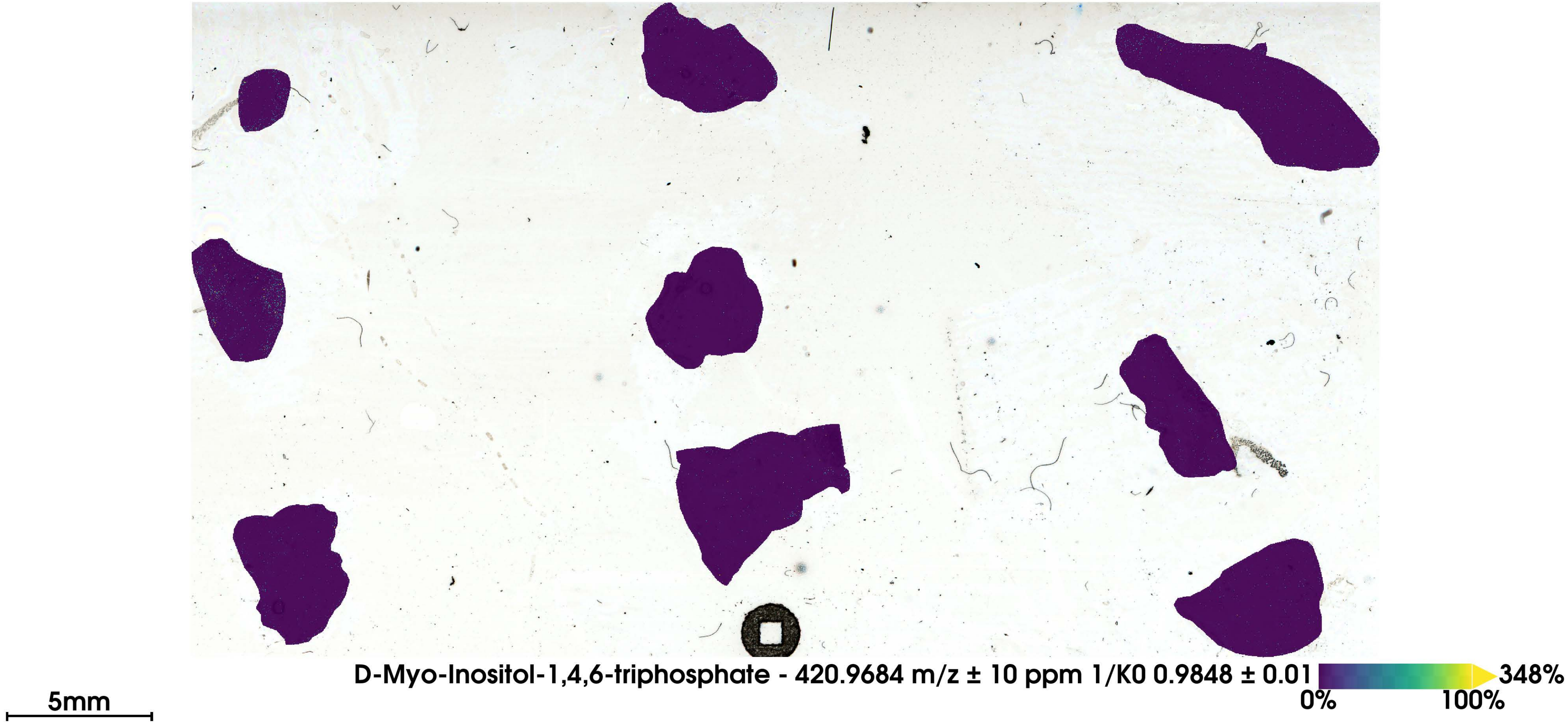
D-Myo-Inositol-1,4,6-triphosphate - 420.9684 m/z ± 10 ppm 1/K0 0.9848 ± 0.01
348%
0%
100%
5mm

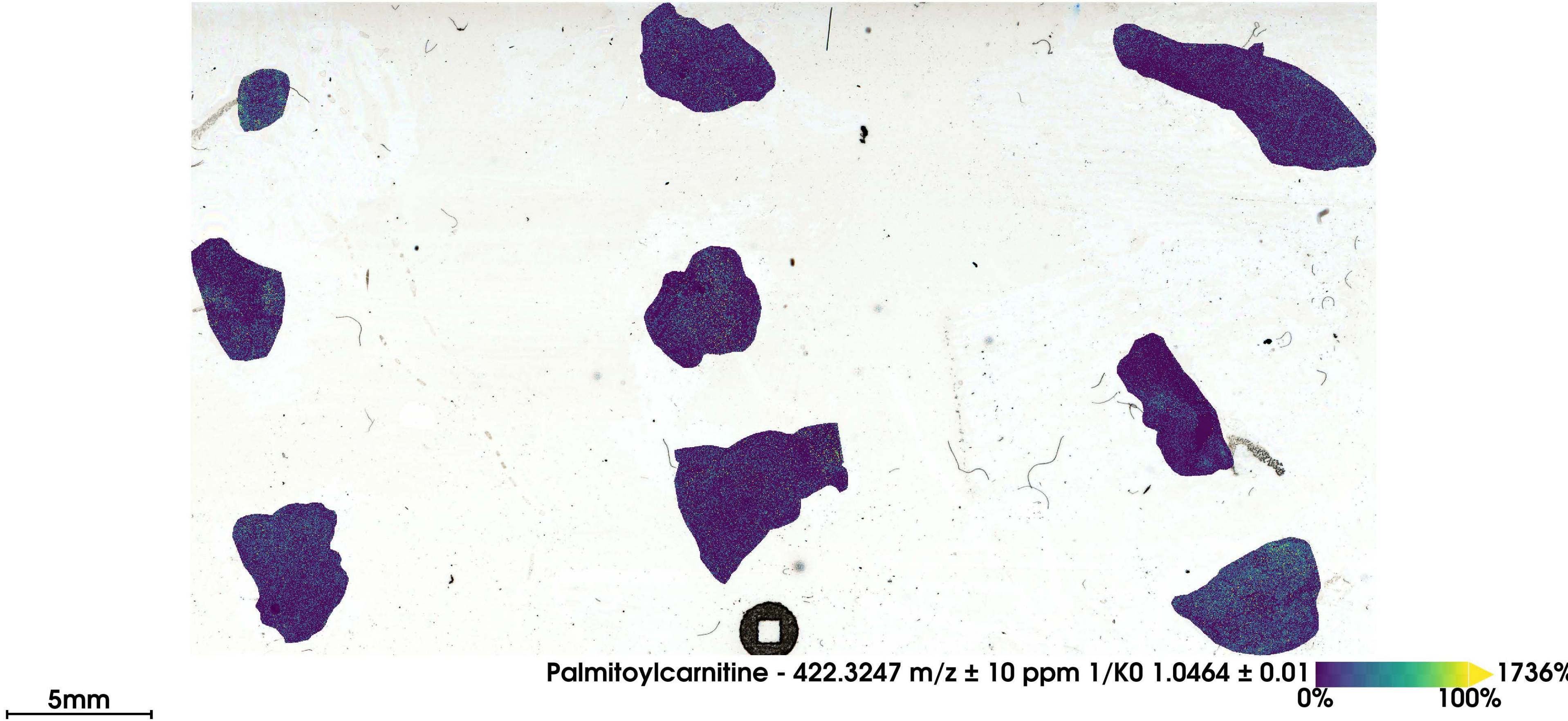

Palmitoylcarnitine - 422.3247 m/z ± 10 ppm 1/K0 1.0464 ± 0.01
1736%
0%
100%
5mm

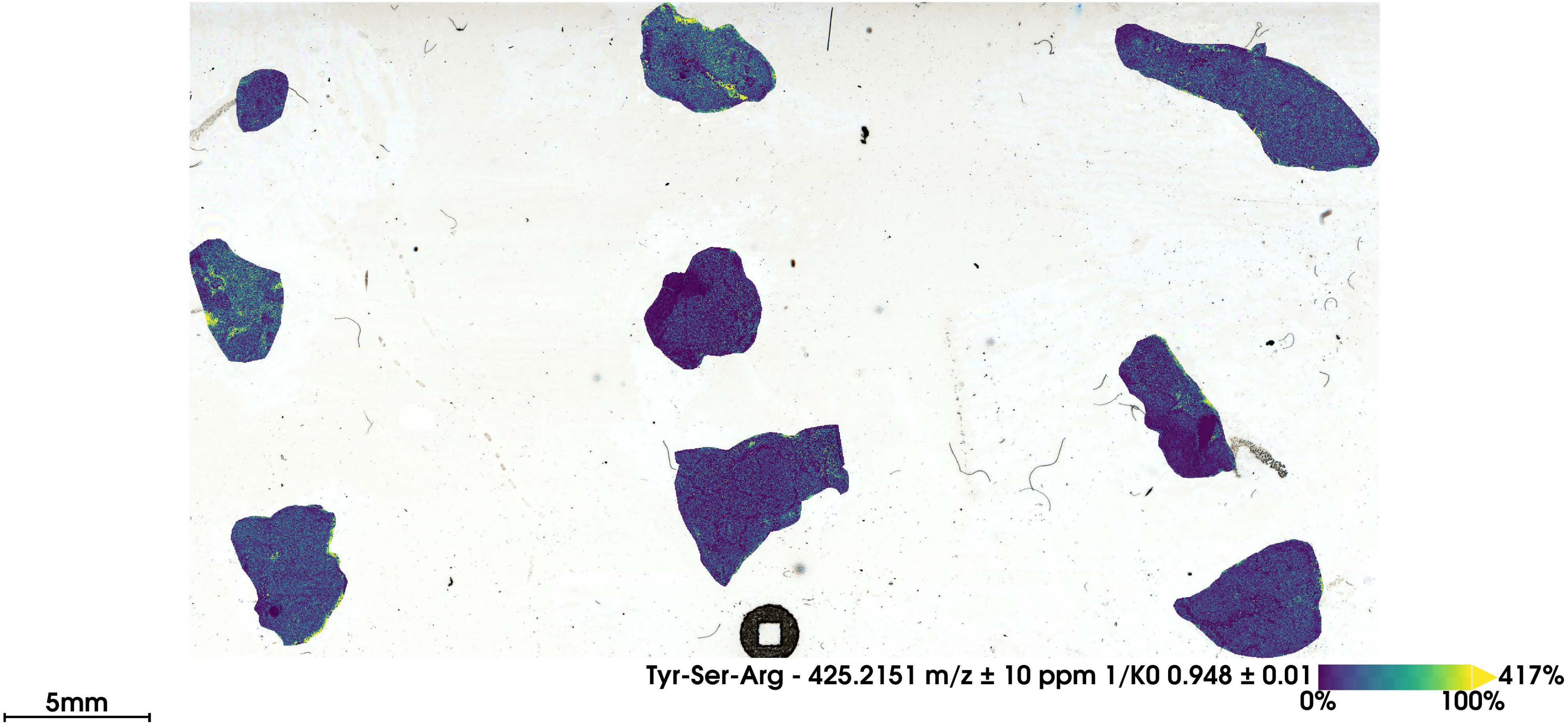
Tyr-Ser-Arg - 425.2151 m/z ± 10 ppm 1/K0 0.948 ± 0.01
417%
0%
100%
5mm

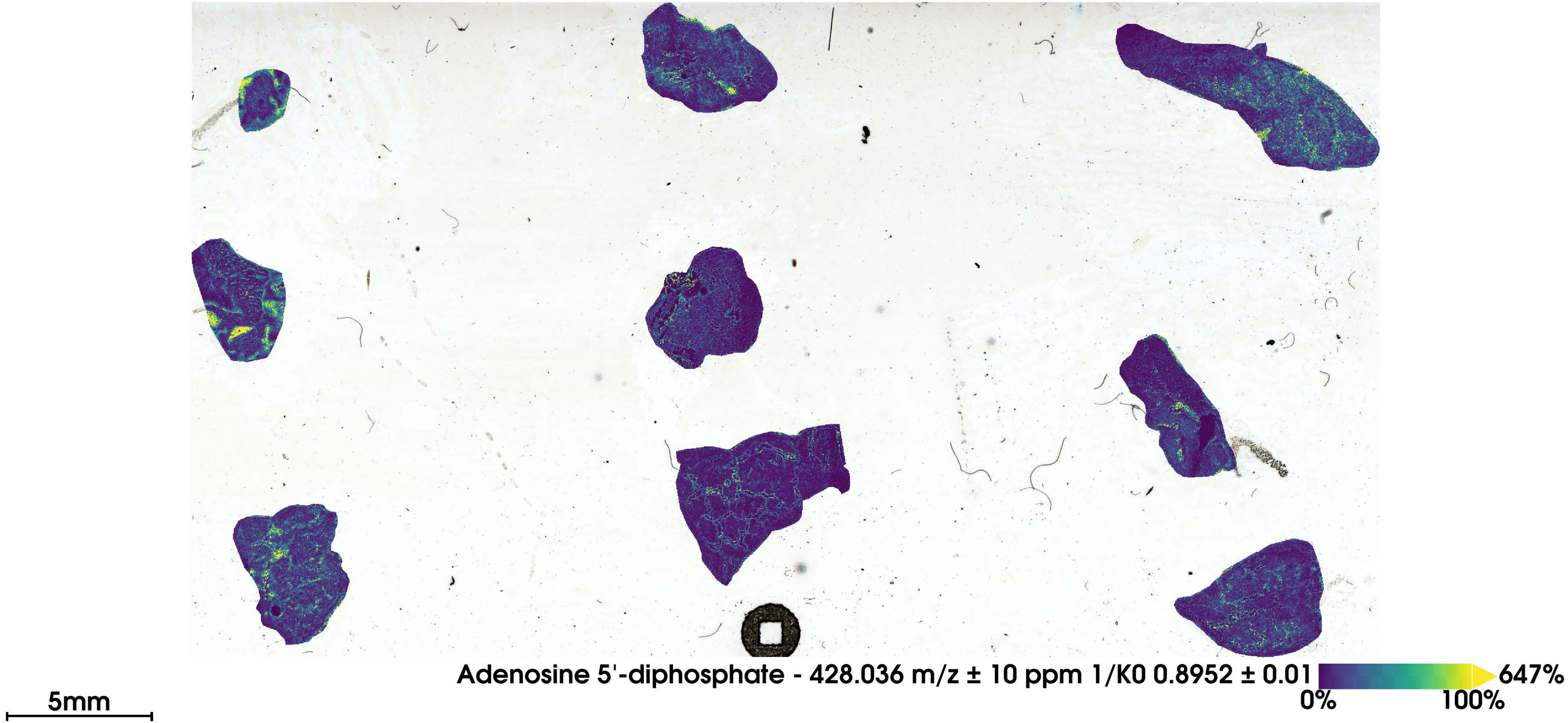

Adenosine 5'-diphosphate - 428.036 m/z ± 10 ppm 1/K0 0.8952 ± 0.01
647%
0%
100%
5mm

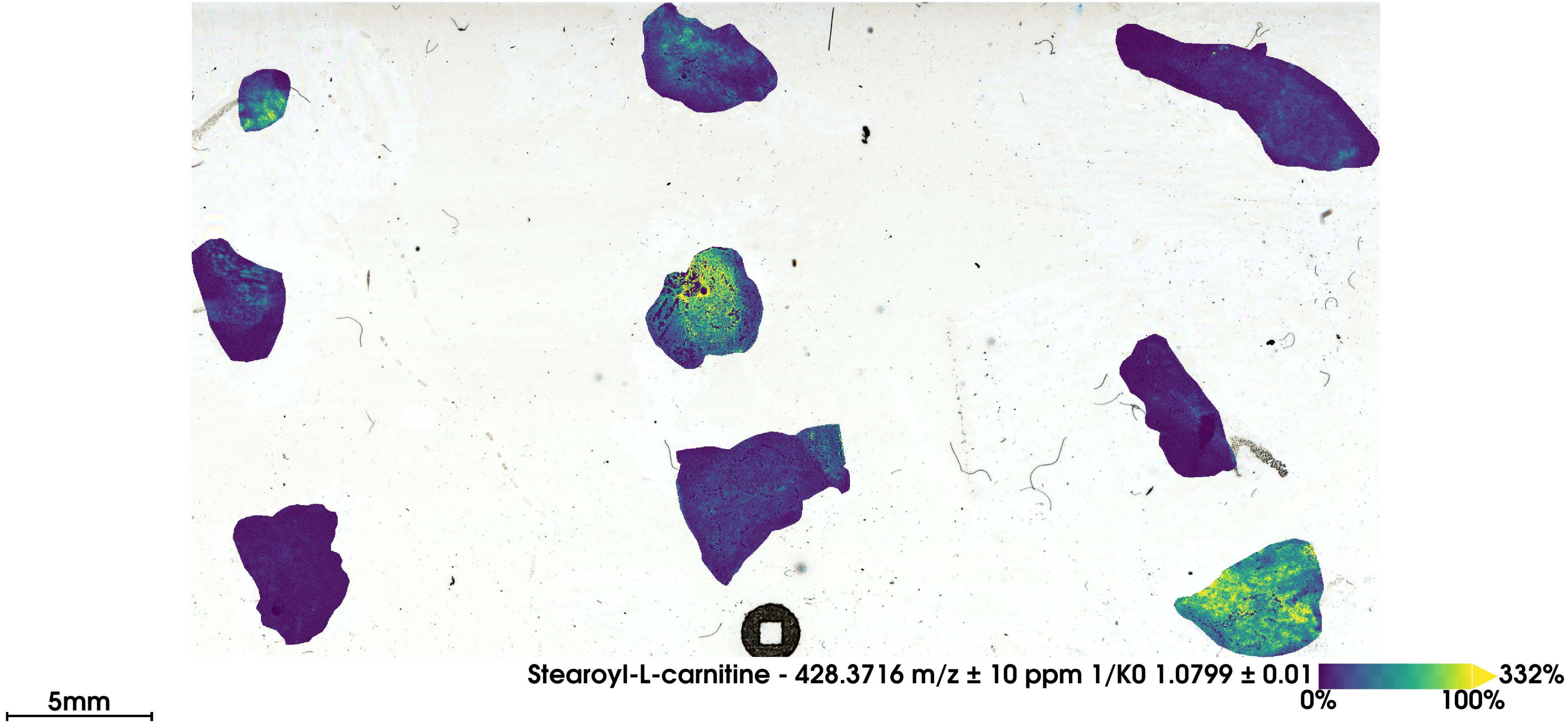
Stearoyl-L-carnitine - 428.3716 m/z ± 10 ppm 1/K0 1.0799 ± 0.01
332%
0%
100%
5mm

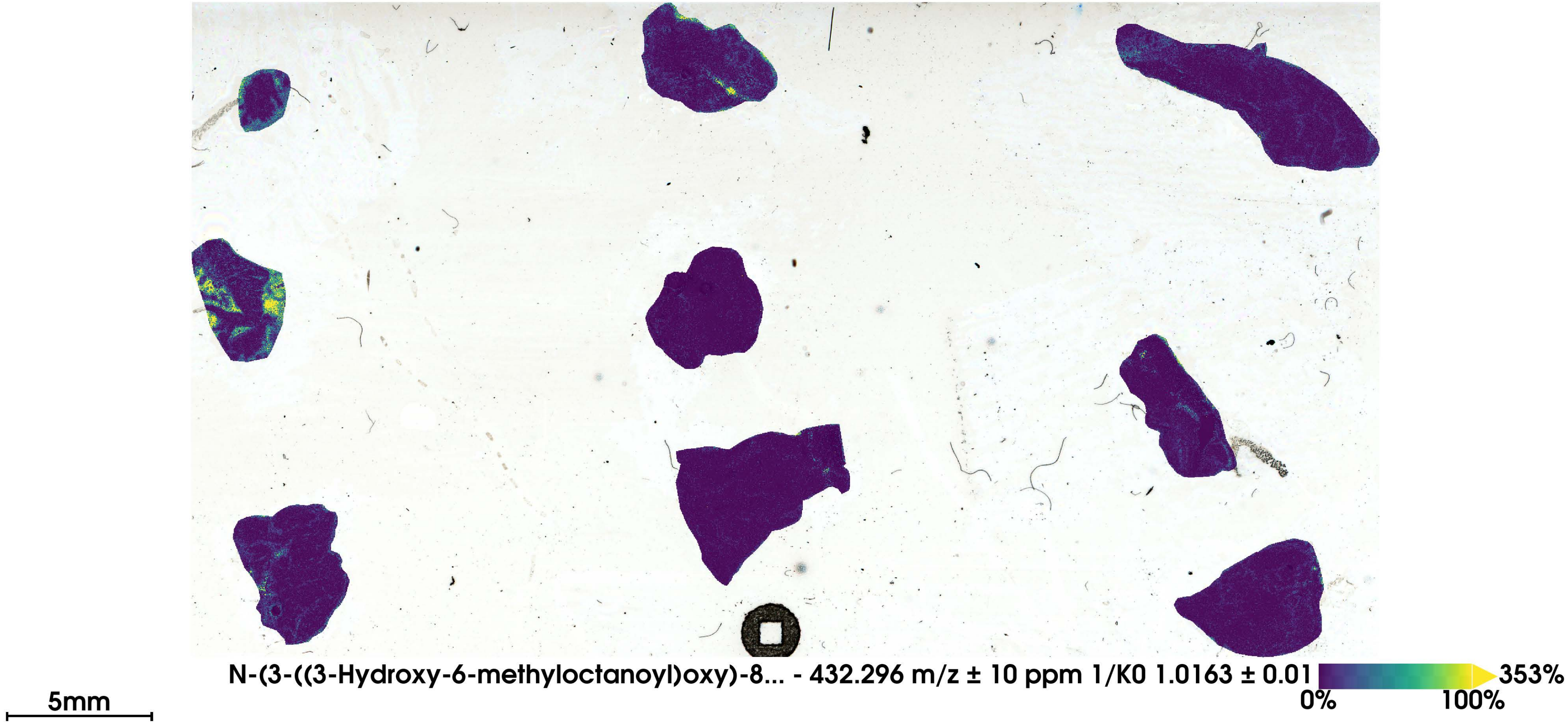

N-(3-((3-Hydroxy-6-methyloctanoyl)oxy)-8... - 432.296 m/z ± 10 ppm 1/K0 1.0163 ± 0.01
353%
0%
100%
5mm

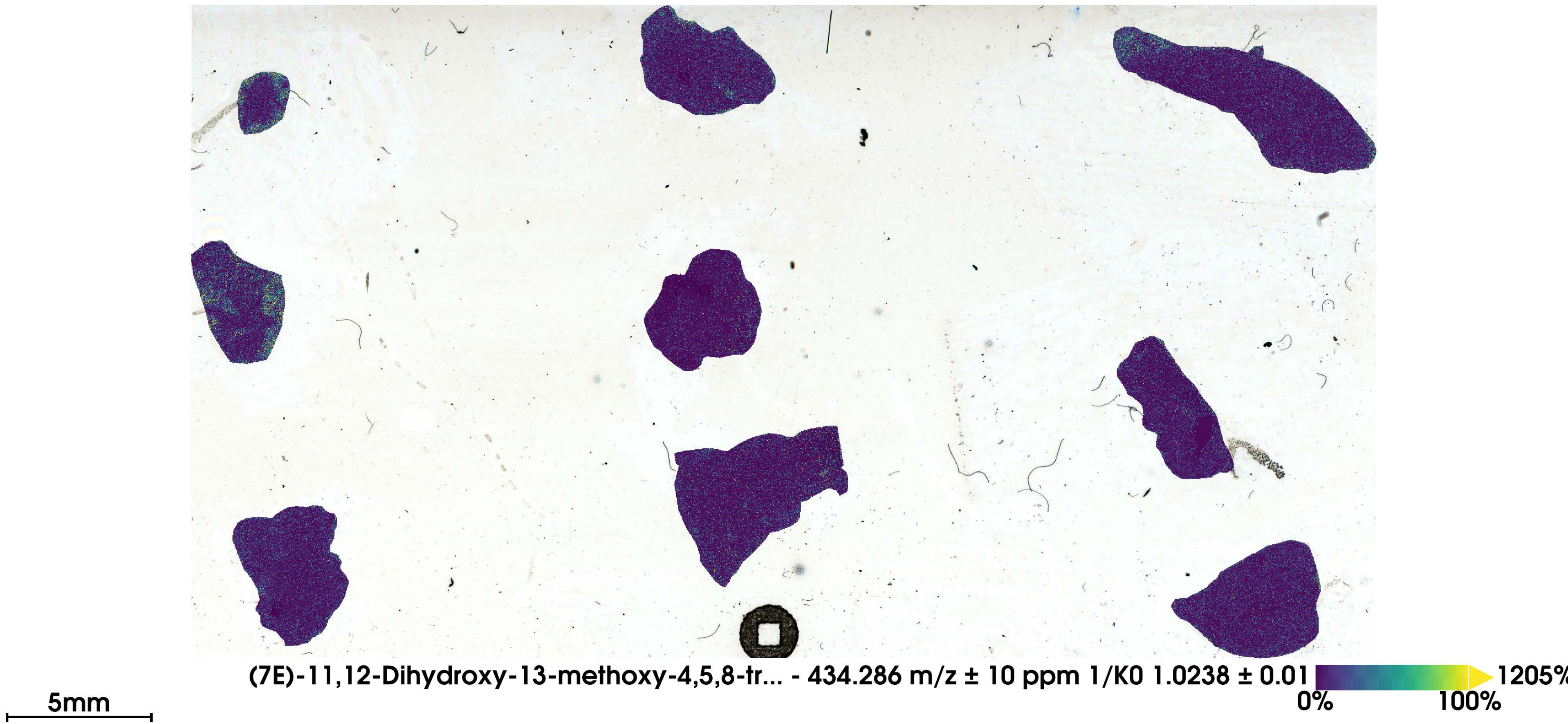
(7E)-11,12-Dihydroxy-13-methoxy-4,5,8-tr... - 434.286 m/z ± 10 ppm 1/K0 1.0238 ± 0.01
1205%
0%
100%
5mm

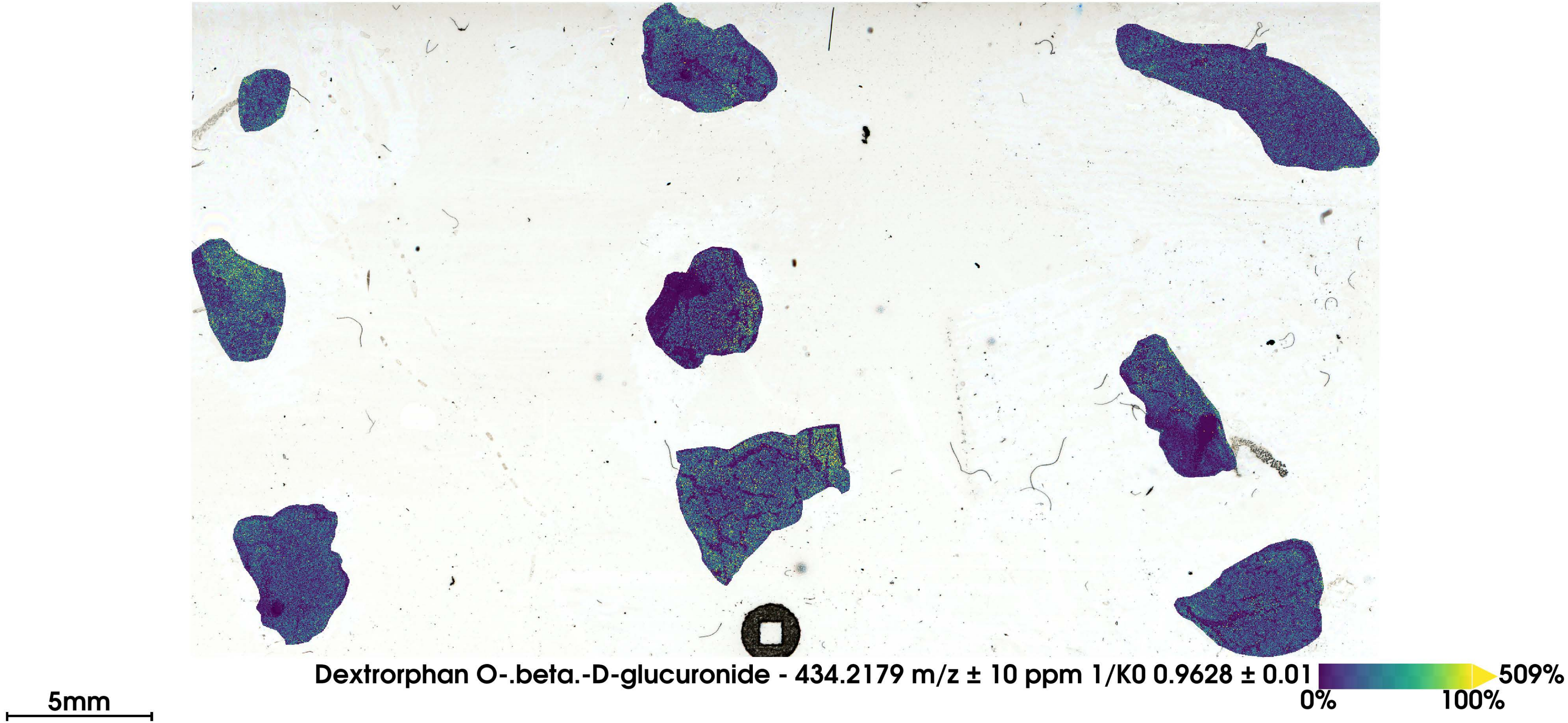

Dextrorphan O-.beta.-D-glucuronide - 434.2179 m/z ± 10 ppm 1/K0 0.9628 ± 0.01
509%
0%
100%
5mm

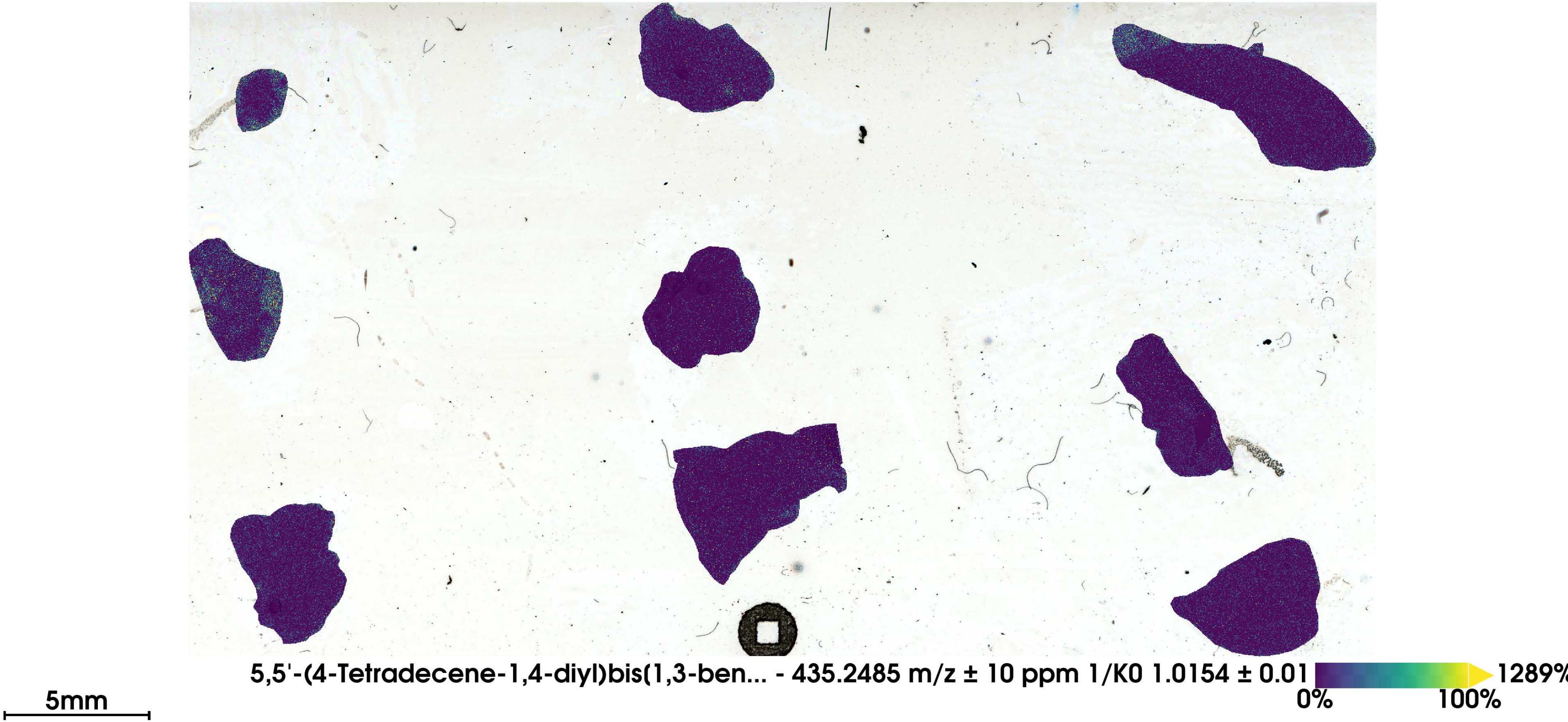
5,5'-(4-Tetradecene-1,4-diyl)bis(1,3-ben... - 435.2485 m/z ± 10 ppm 1/K0 1.0154 ± 0.01
1289%
0%
100%
5mm

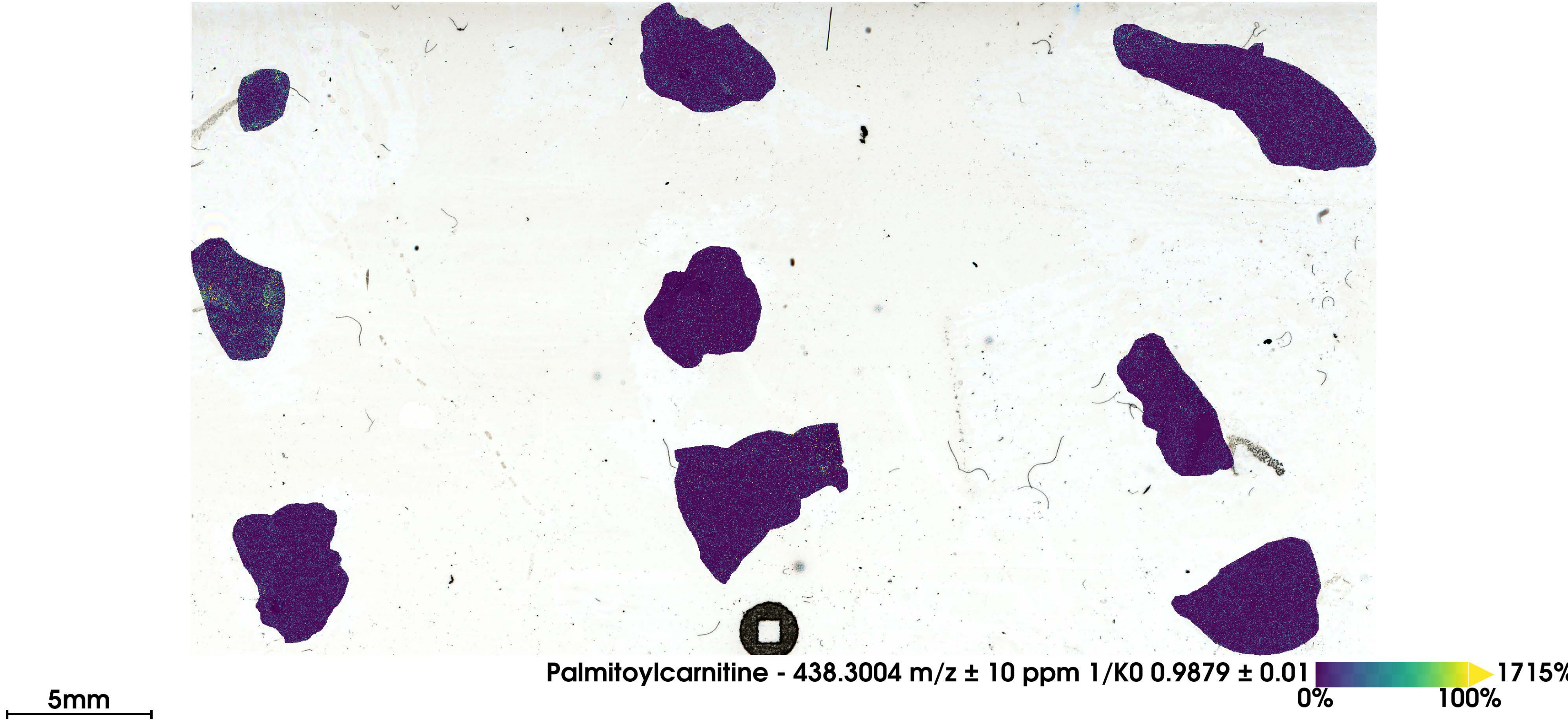

5mm
Palmitoylcarnitine - 438.3004 m/z ± 10 ppm 1/K0 0.9879 ± 0.01
0%
100%
1715%

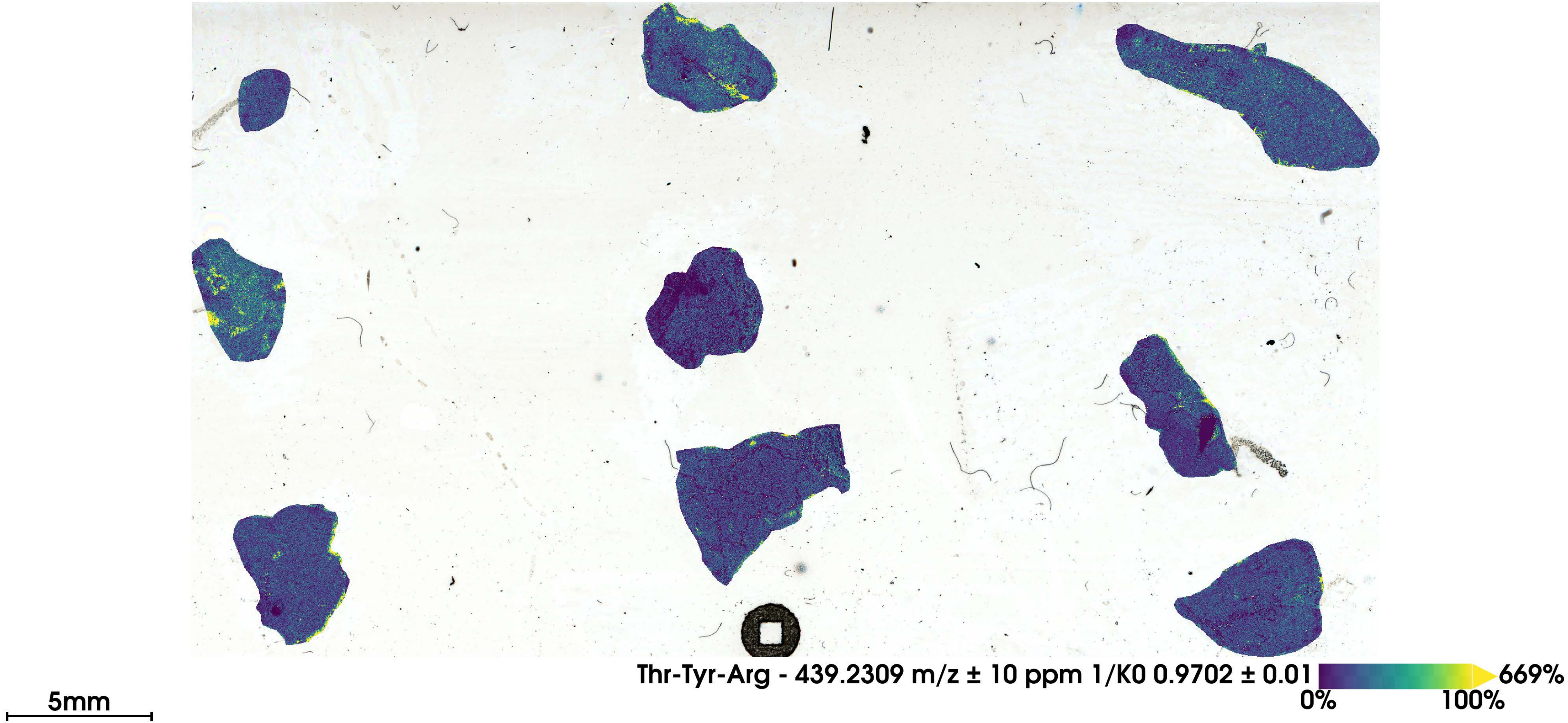
Thr-Tyr-Arg - 439.2309 m/z ± 10 ppm 1/K0 0.9702 ± 0.01
0%
100%
669%
5mm

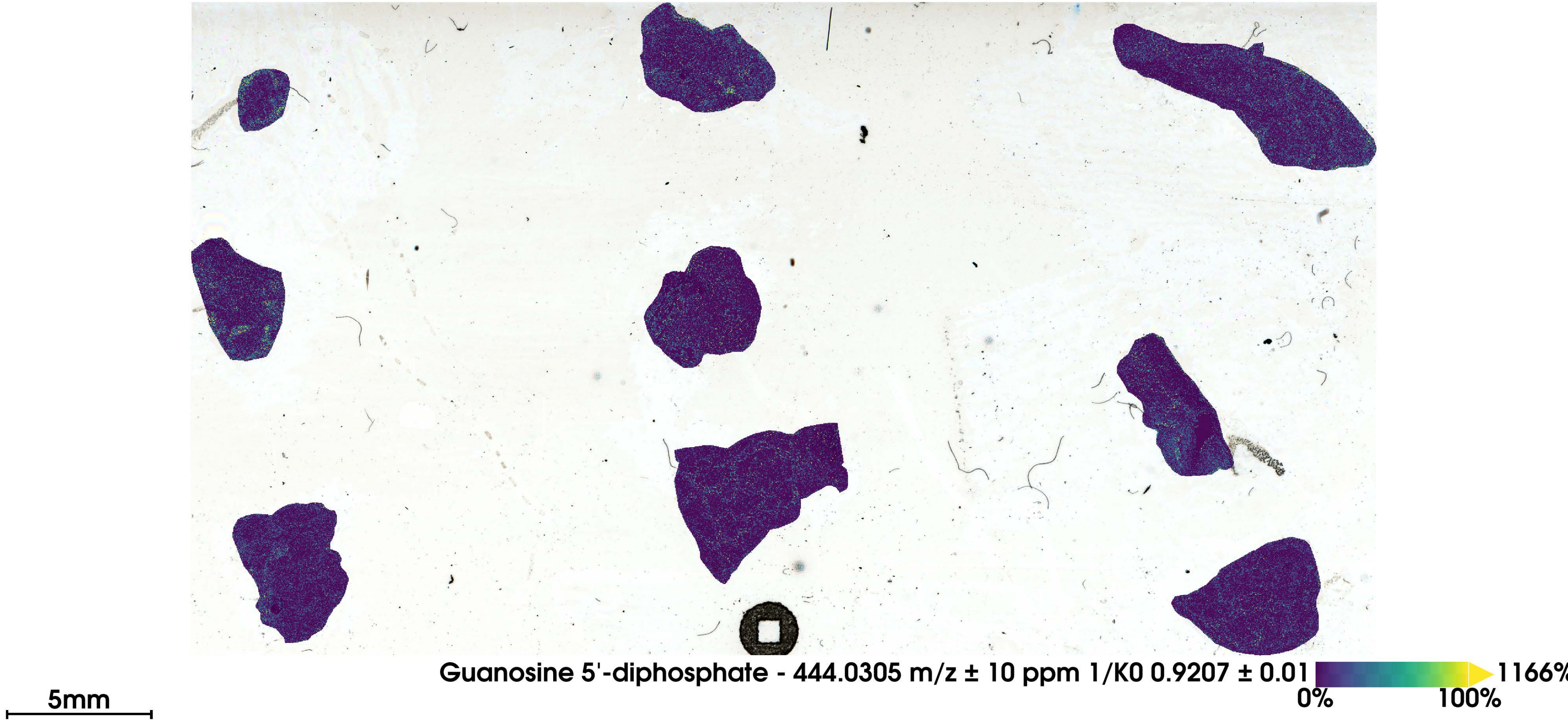
Guanosine 5'-diphosphate - 444.0305 m/z ± 10 ppm 1/K0 0.9207 ± 0.01
1166%
0%
100%
5mm

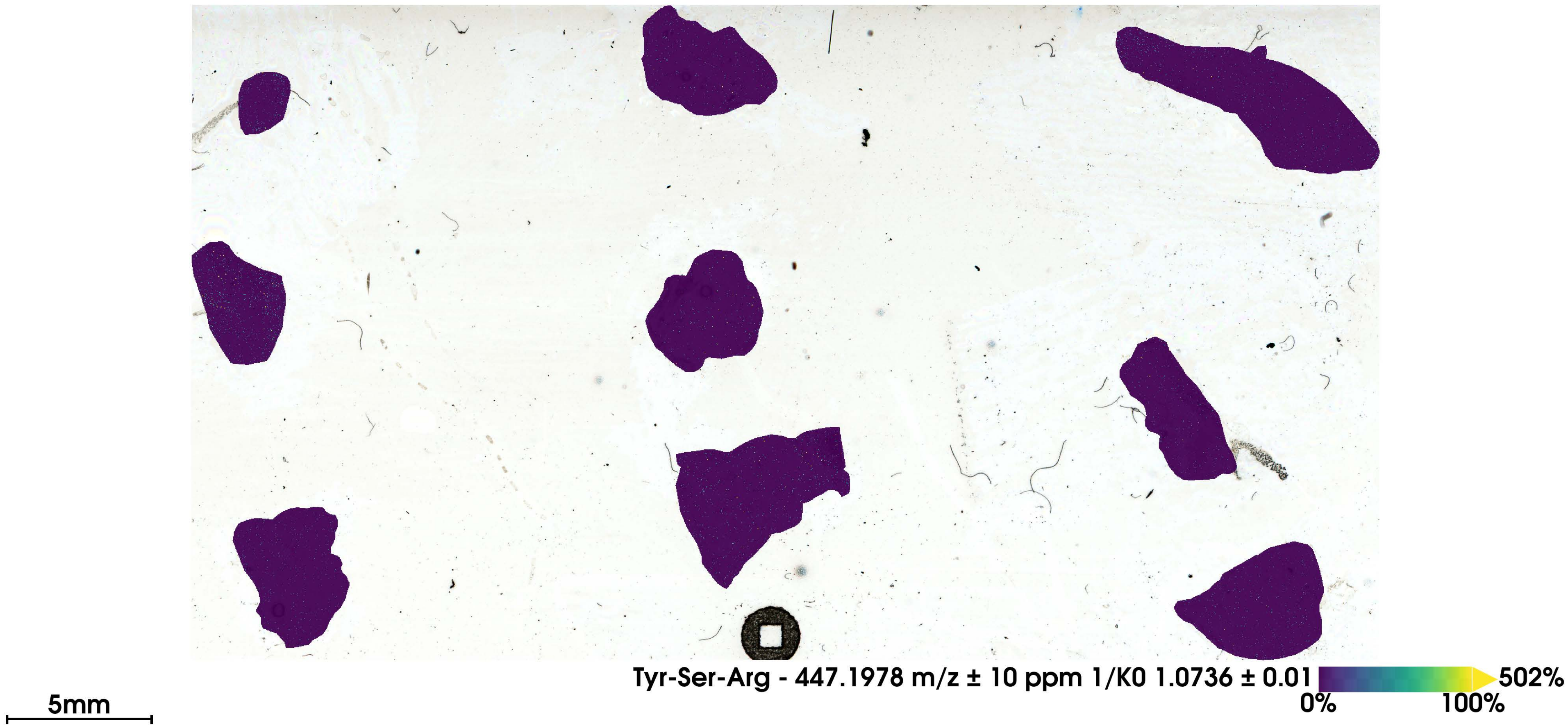
Tyr-Ser-Arg - 447.1978 m/z ± 10 ppm 1/K0 1.0736 ± 0.01
502%
0%
100%
5mm

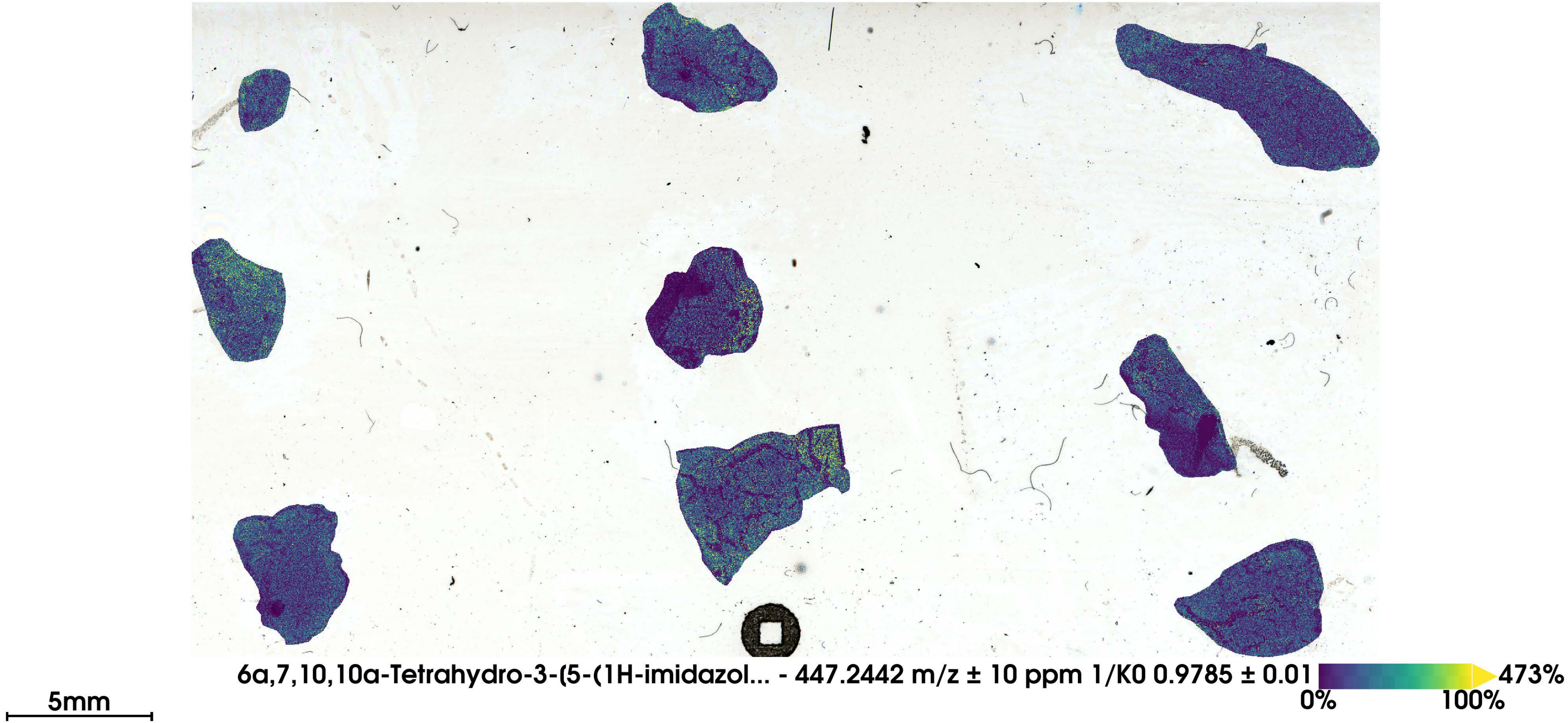
6a,7,10,10a-Tetrahydro-3-(5-(1H-imidazol... - 447.2442 m/z ± 10 ppm 1/K0 0.9785 ± 0.01
473%
0%
100%
5mm

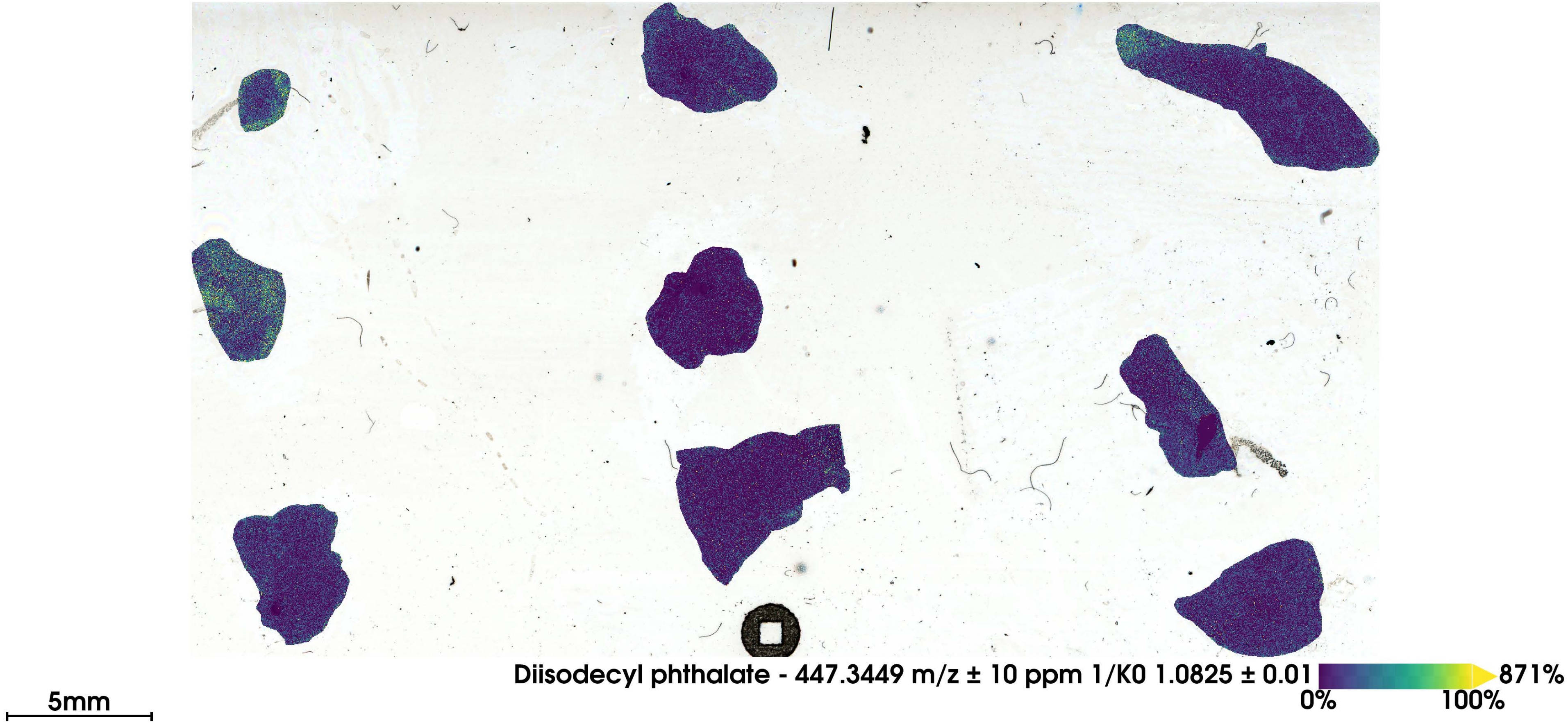

Diisodecyl phthalate - 447.3449 m/z ± 10 ppm 1/K0 1.0825 ± 0.01
871%
0%
100%
5mm

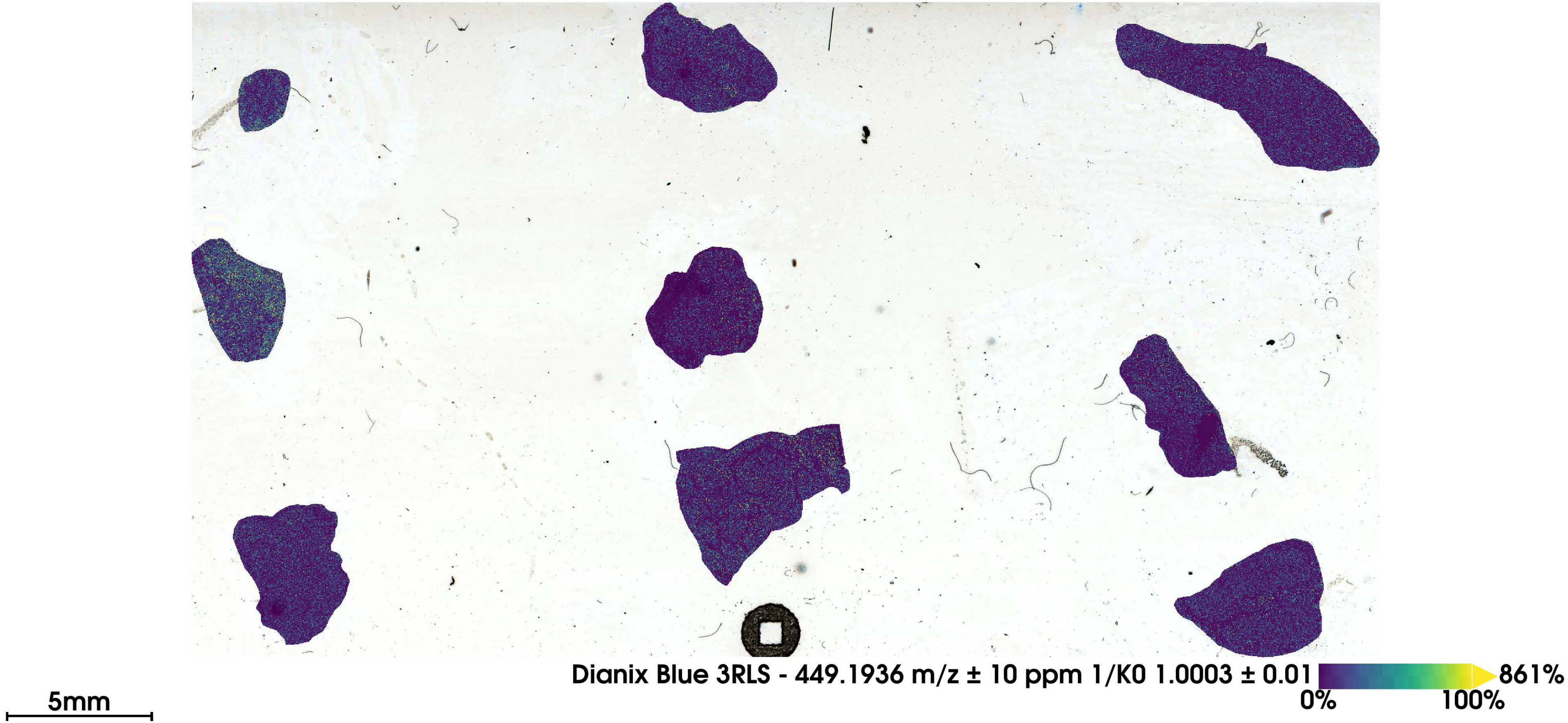
Dianix Blue 3RLS - 449.1936 m/z ± 10 ppm 1/K0 1.0003 ± 0.01
861%
0%
100%
5mm

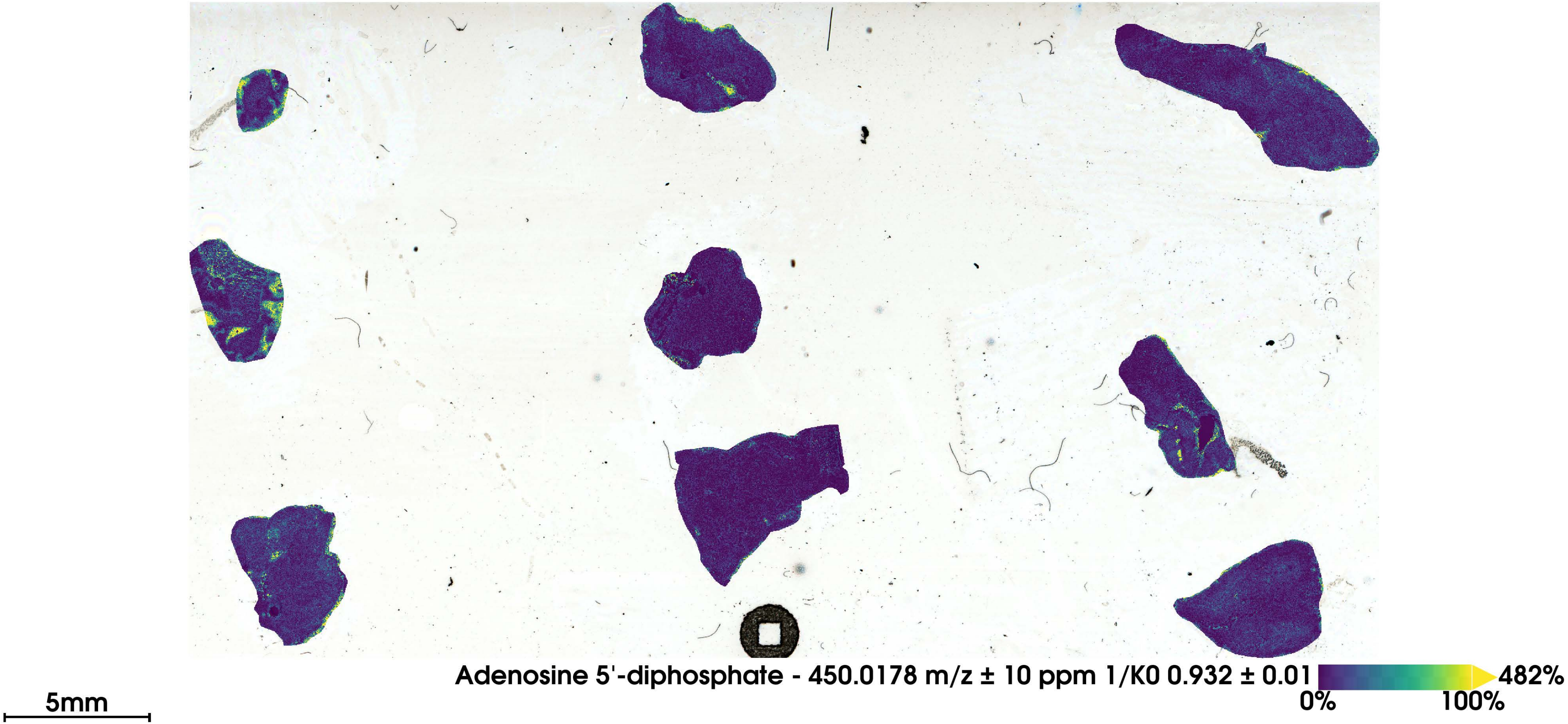

Adenosine 5'-diphosphate - 450.0178 m/z ± 10 ppm 1/K0 0.932 ± 0.01
482%
0%
100%
5mm

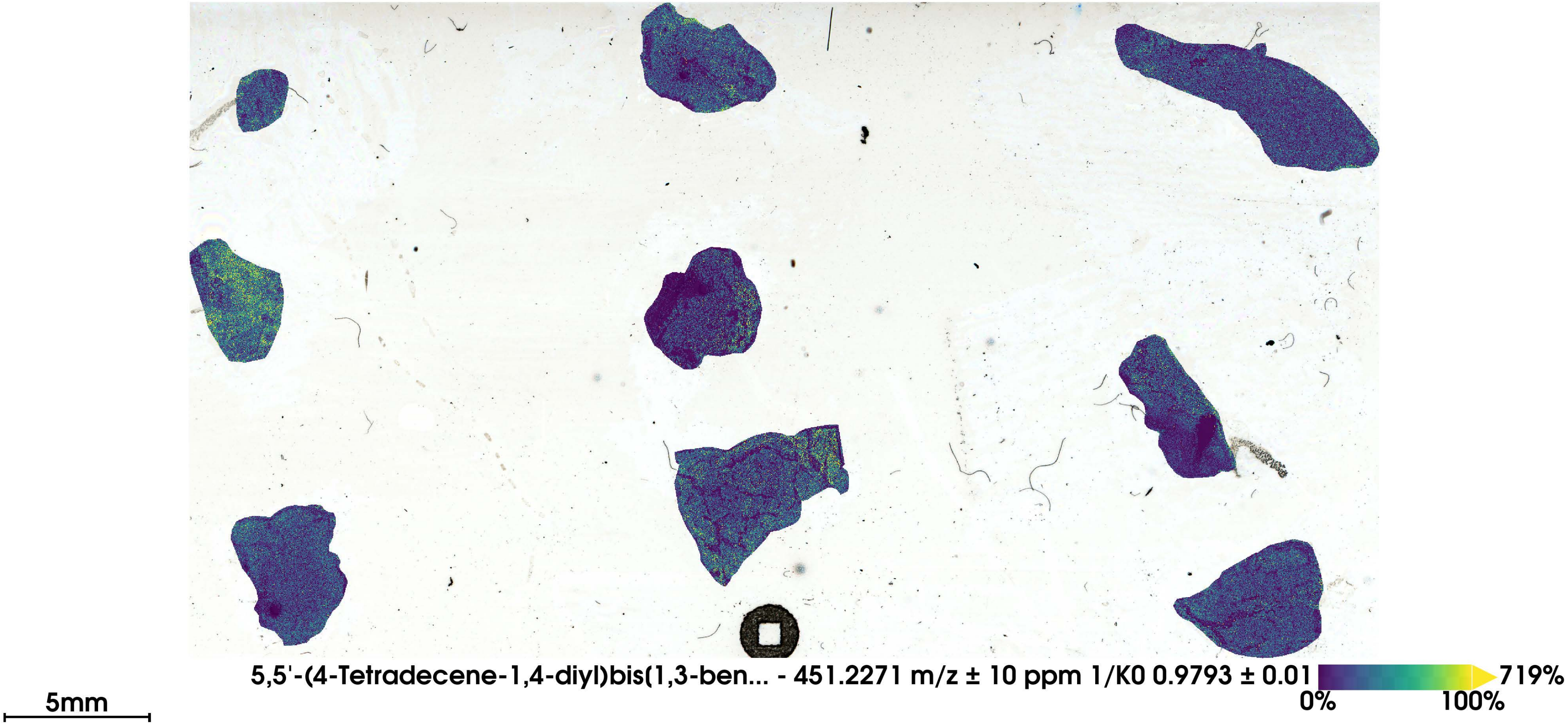

5,5'-(4-Tetradecene-1,4-diyl)bis(1,3-ben... - 451.2271 m/z ± 10 ppm 1/K0 0.9793 ± 0.01
719%
0%
100%
5mm

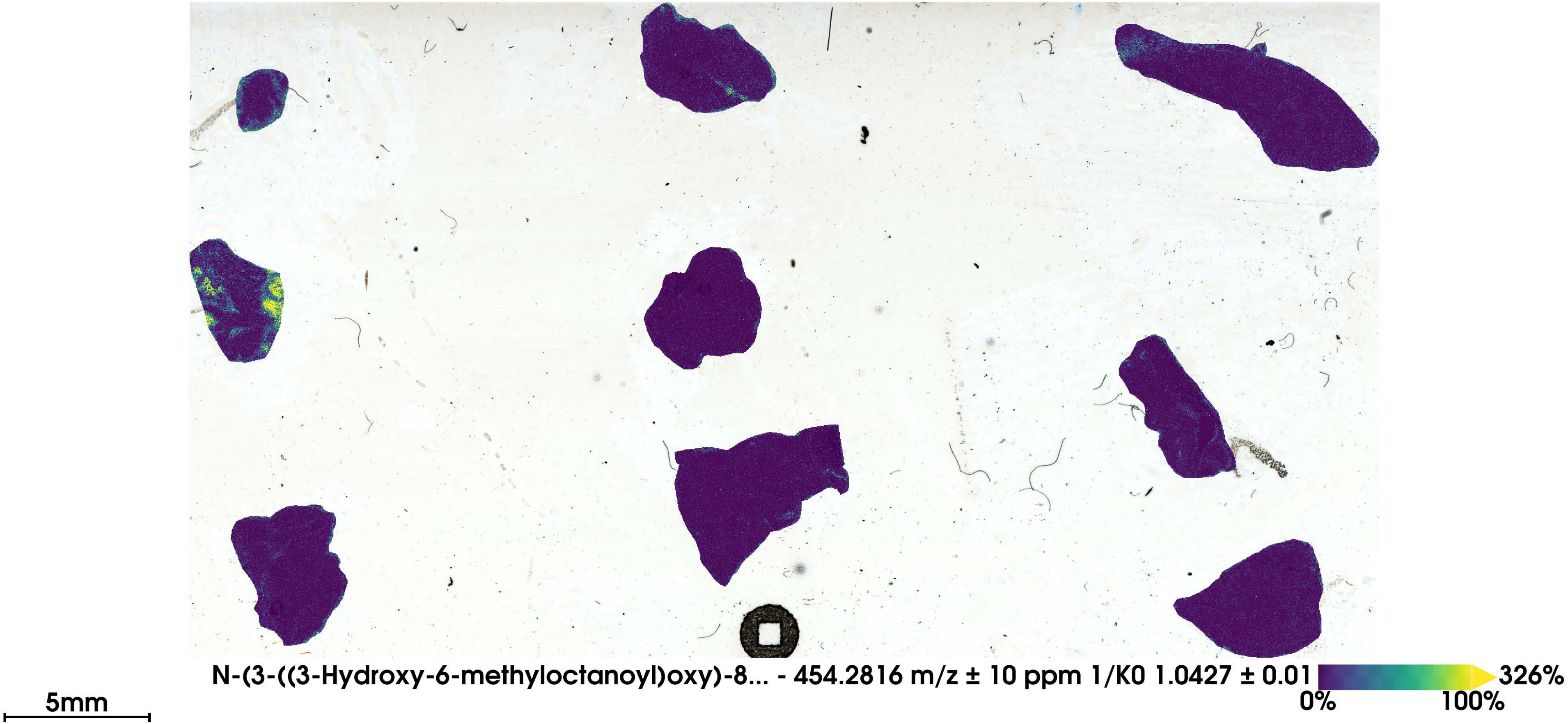
N-(3-((3-Hydroxy-6-methyloctanoyl)oxy)-8... - 454.2816 m/z ± 10 ppm 1/K0 1.0427 ± 0.01
326%
0%
100%
5mm

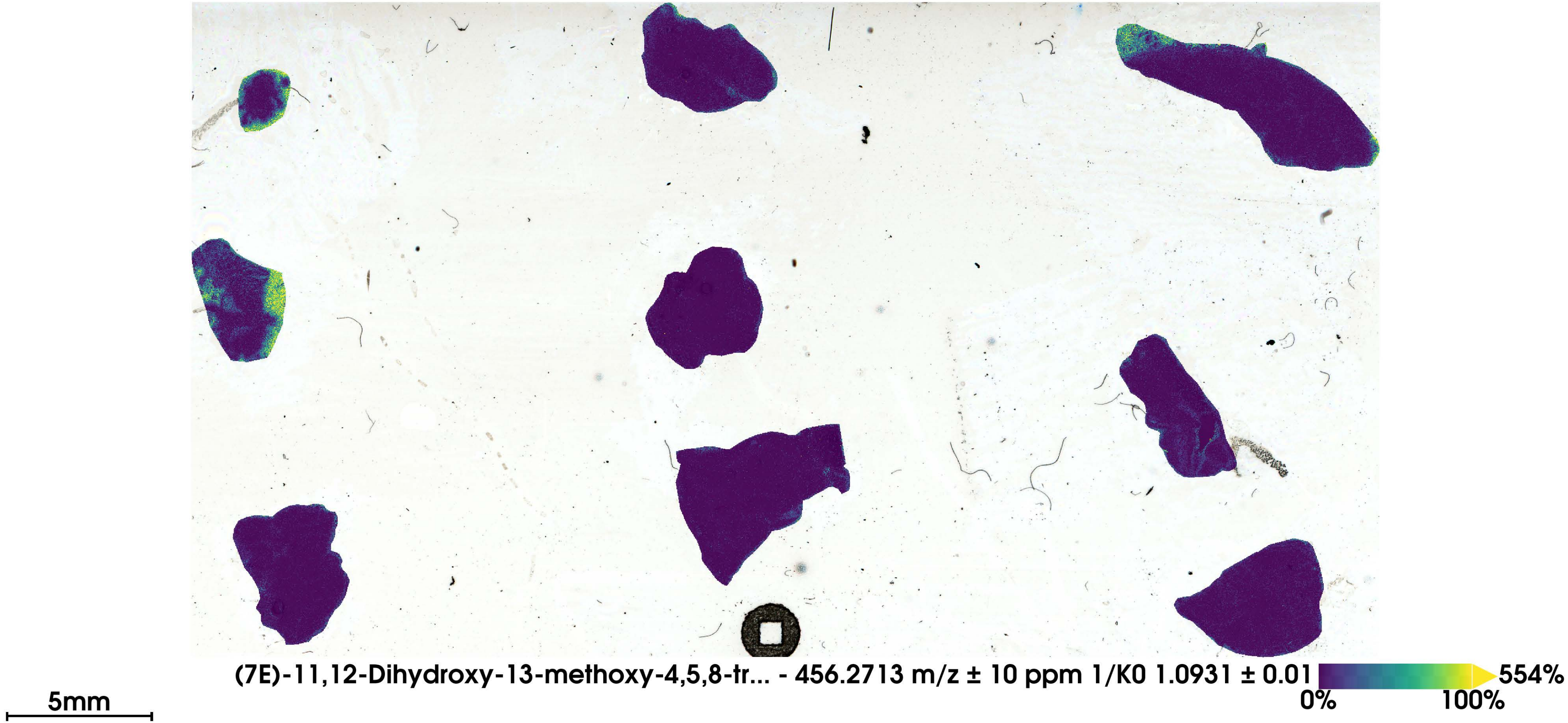

(7E)-11,12-Dihydroxy-13-methoxy-4,5,8-tr... - 456.2713 m/z ± 10 ppm 1/K0 1.0931 ± 0.01
554%
0%
100%
5mm

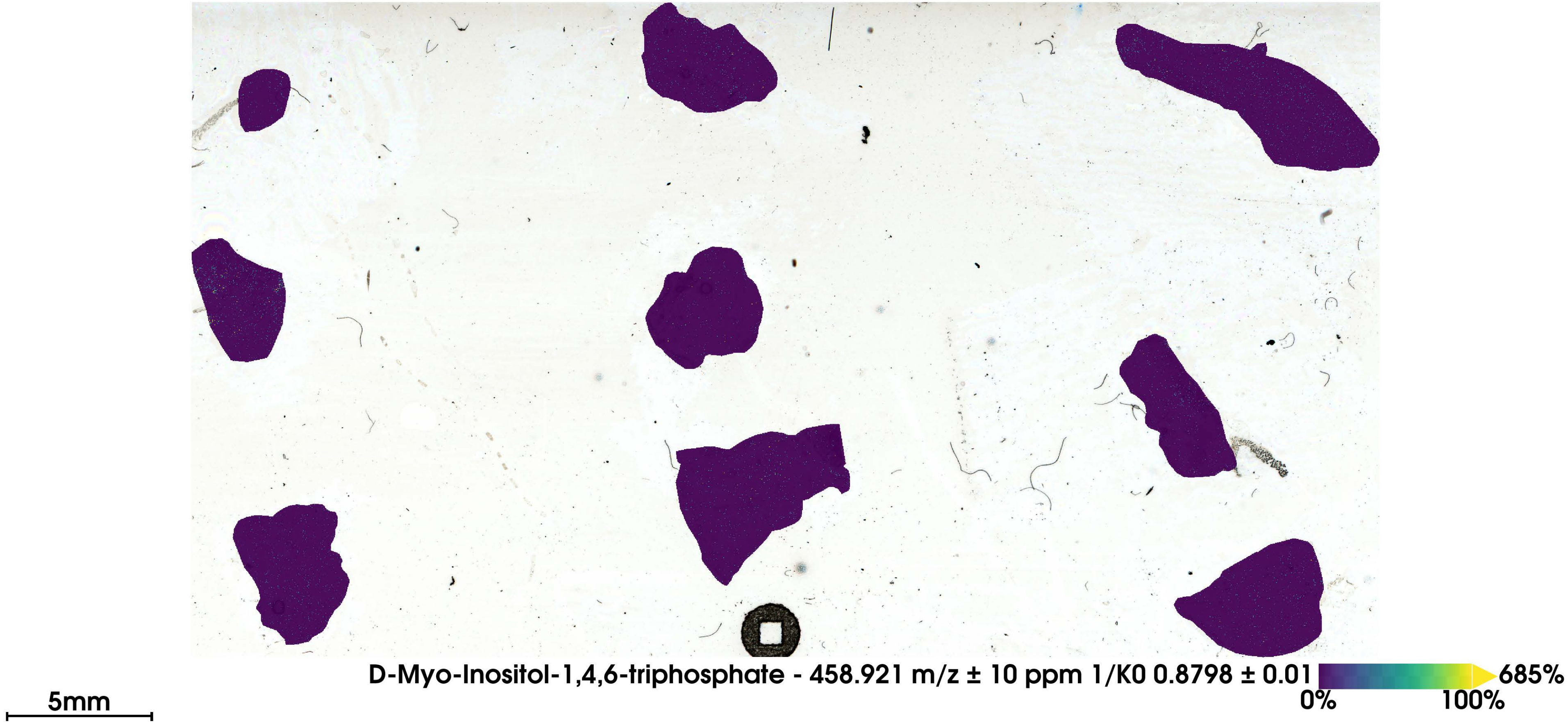

D-Myo-Inositol-1,4,6-triphosphate - 458.921 m/z ± 10 ppm 1/K0 0.8798 ± 0.01
685%
0%
100%
5mm

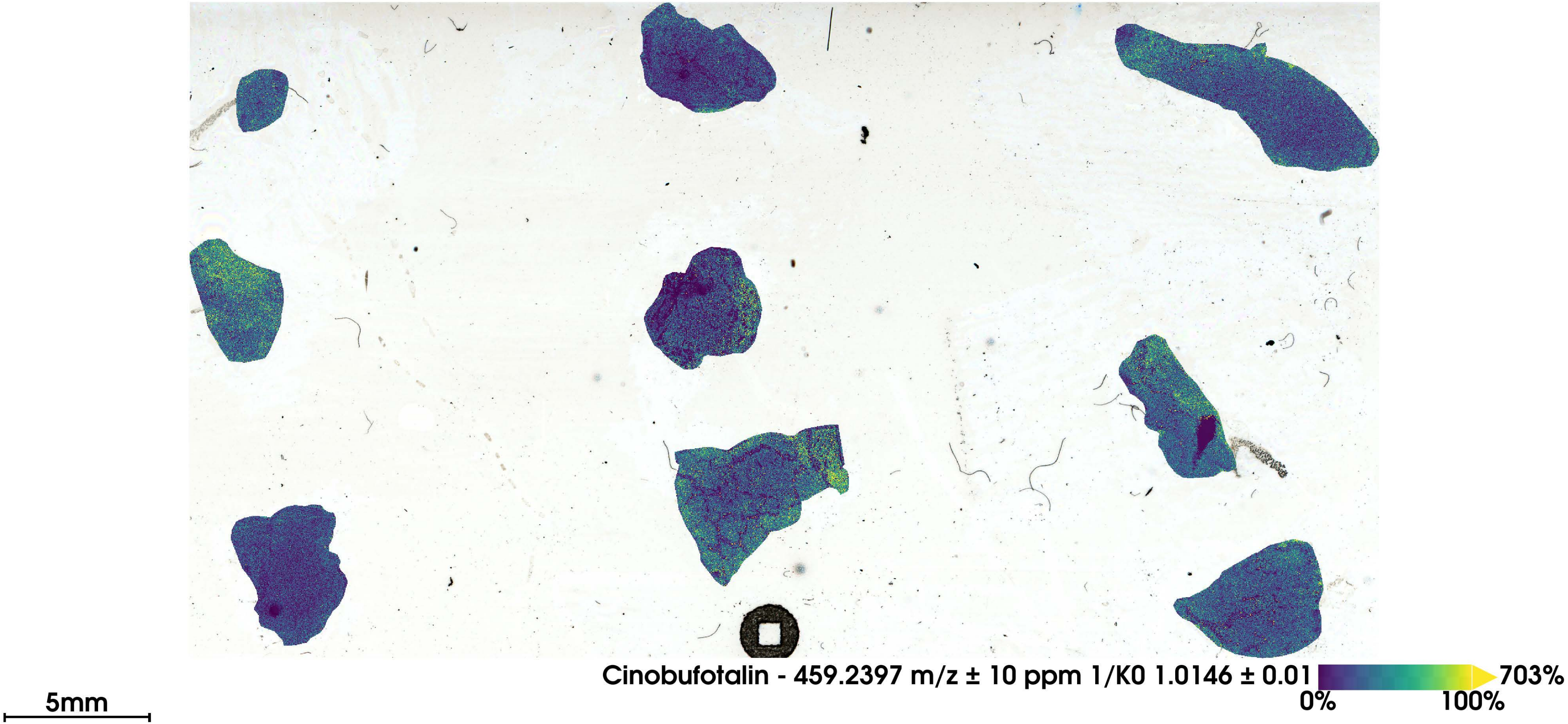
Cinobufotalin - 459.2397 m/z ± 10 ppm 1/K0 1.0146 ± 0.01
703%
0%
100%
5mm

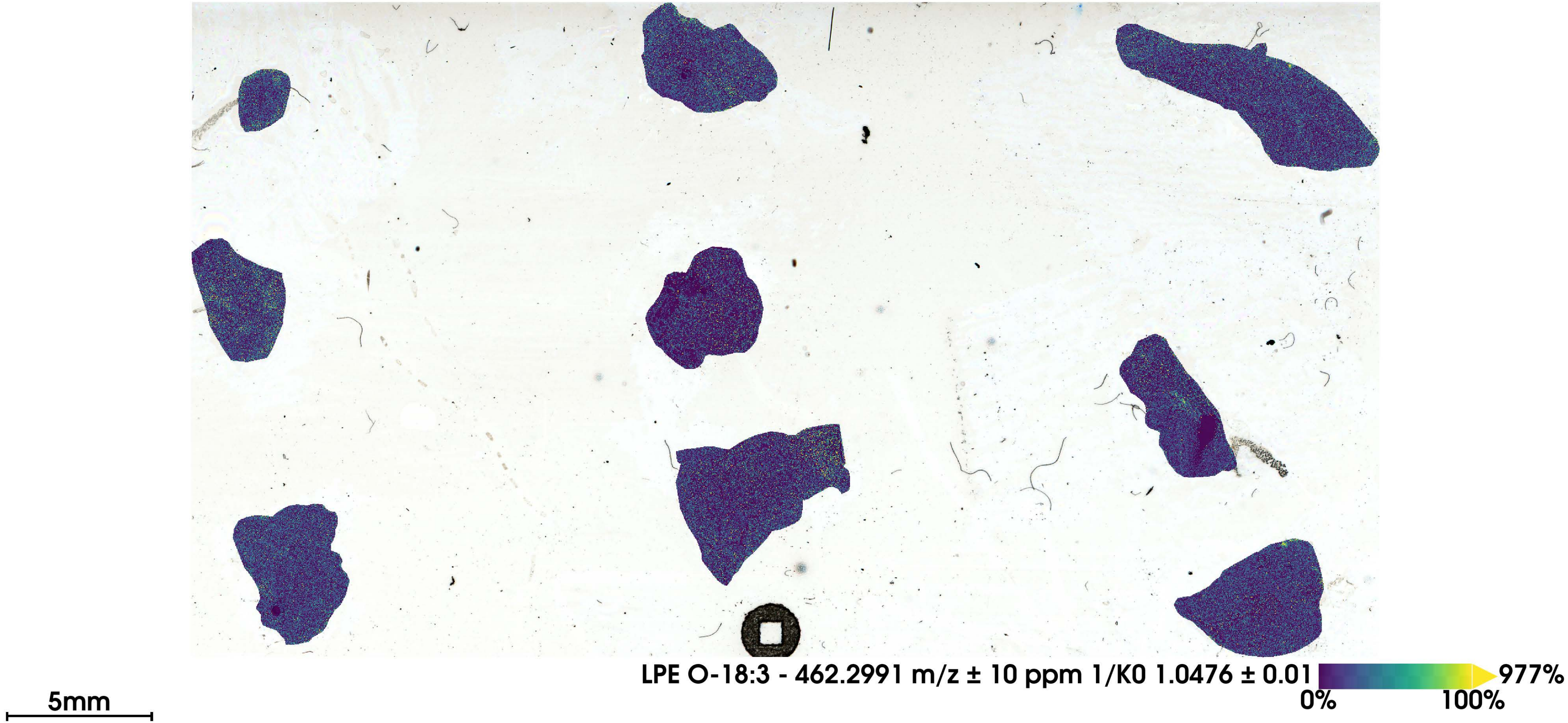

LPE O-18:3 - 462.2991 m/z ± 10 ppm 1/K0 1.0476 ± 0.01
977%
0%
100%
5mm

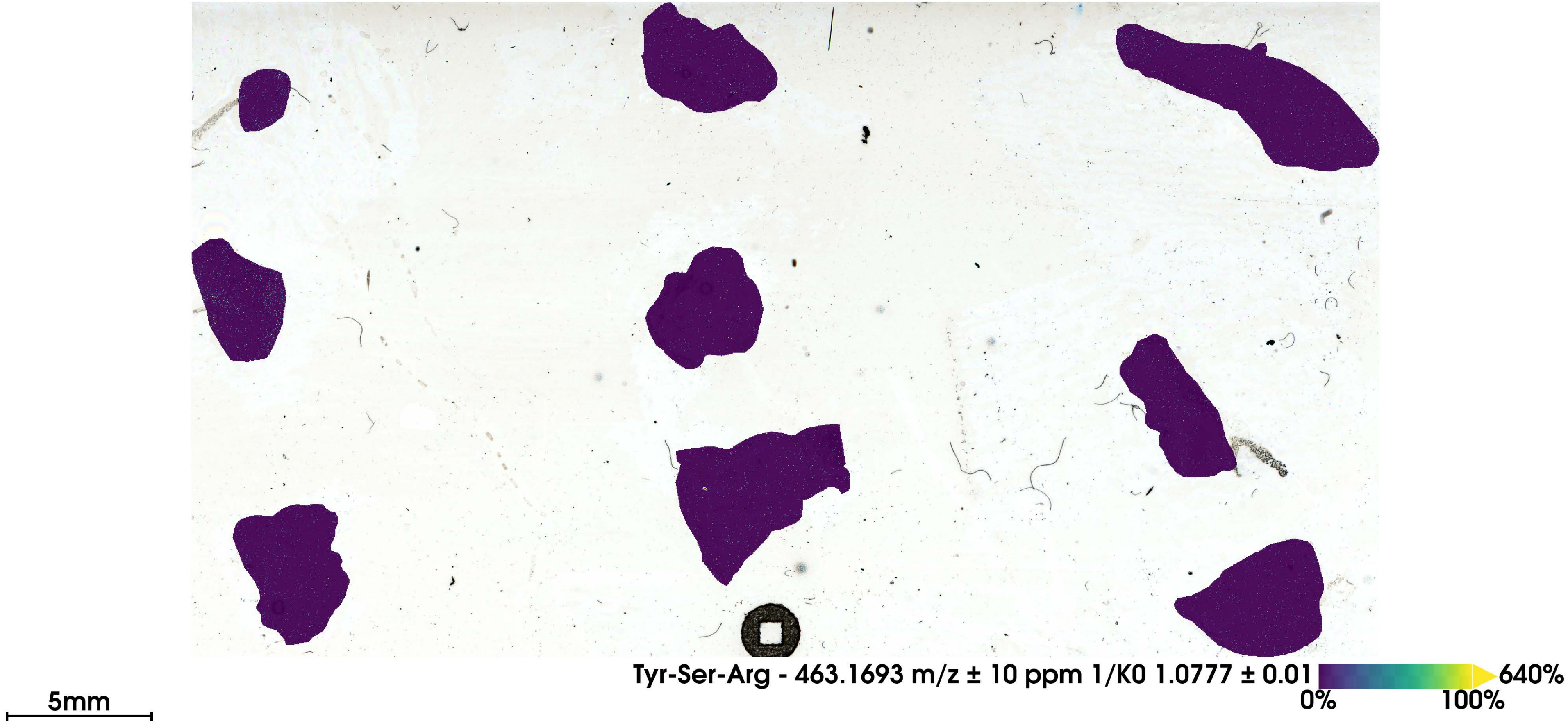
Tyr-Ser-Arg - 463.1693 m/z ± 10 ppm 1/K0 1.0777 ± 0.01
640%
0%
100%
5mm

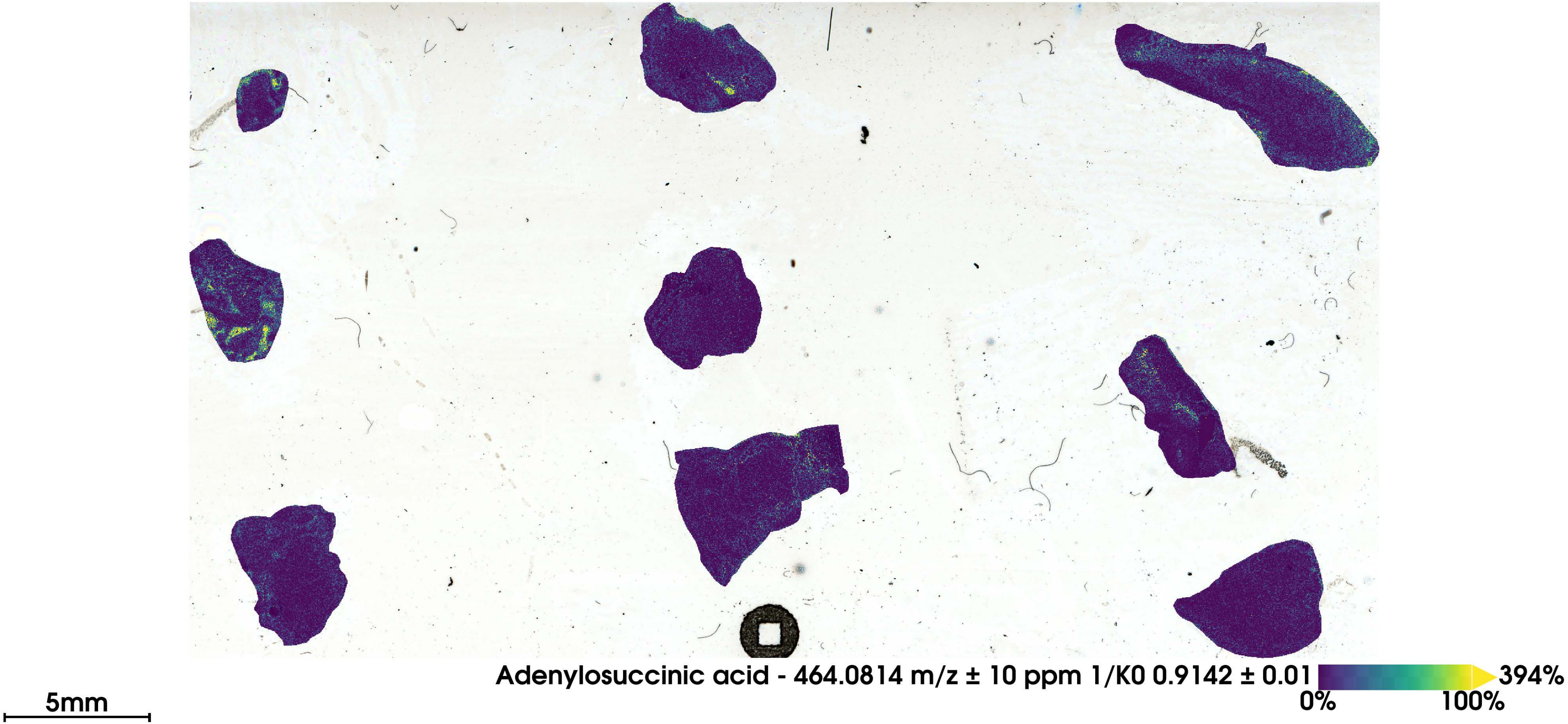
Adenylosuccinic acid - 464.0814 m/z ± 10 ppm 1/K0 0.9142 ± 0.01
394%
0%
100%
5mm

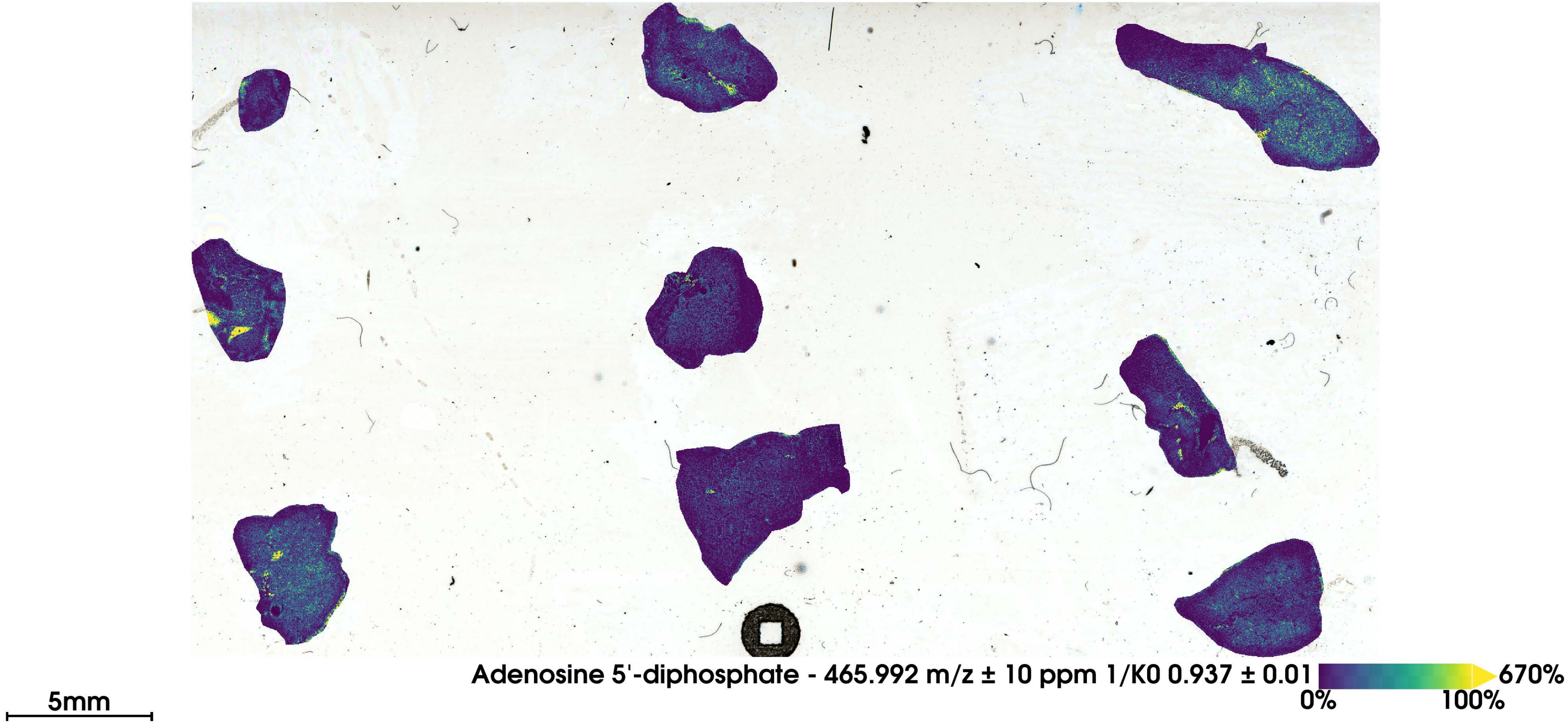
Adenosine 5'-diphosphate - 465.992 m/z ± 10 ppm 1/K0 0.937 ± 0.01
670%
0%
100%
5mm

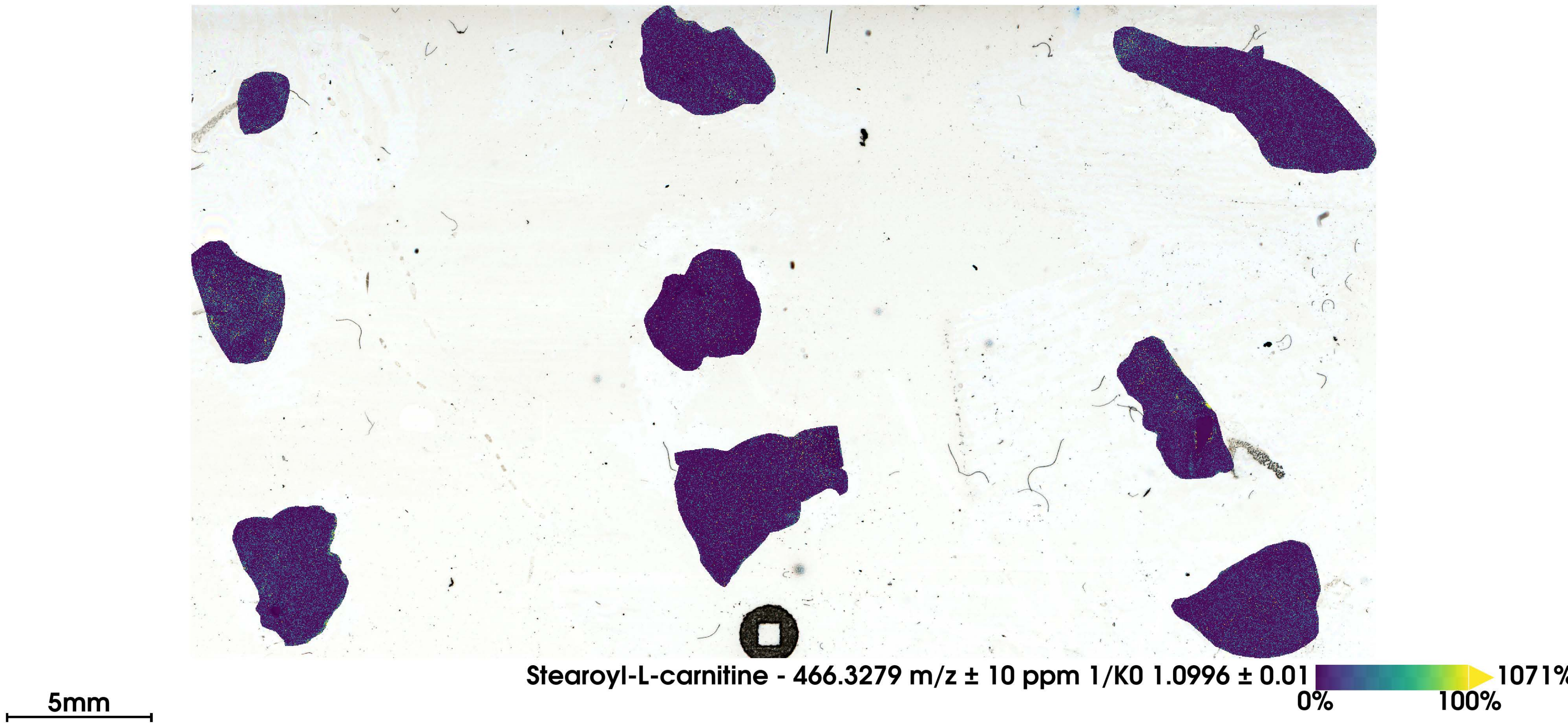
Stearoyl-L-carnitine - 466.3279 m/z ± 10 ppm 1/K0 1.0996 ± 0.01
1071%
0%
100%
5mm

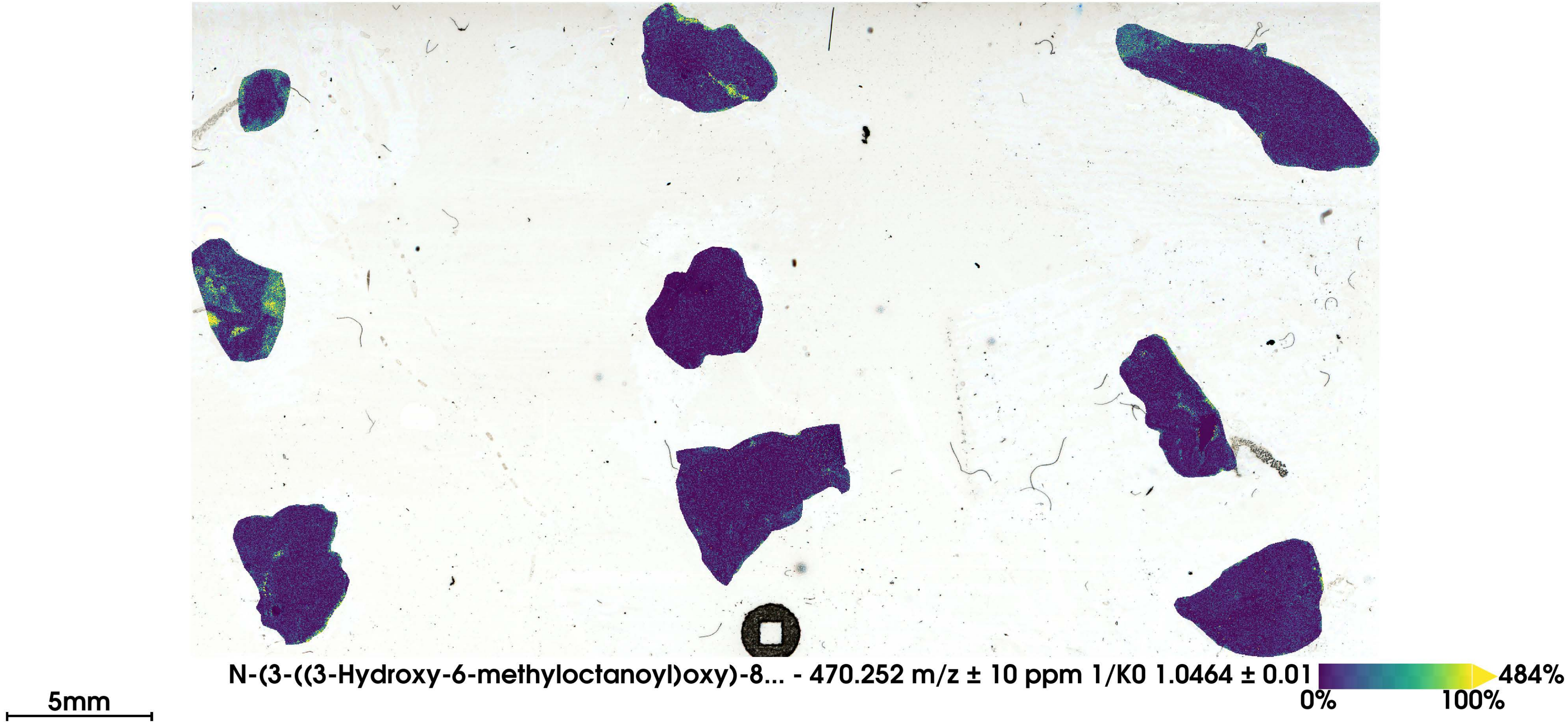
N-(3-((3-Hydroxy-6-methyloctanoyl)oxy)-8... - 470.252 m/z ± 10 ppm 1/K0 1.0464 ± 0.01
484%
0%
100%
5mm

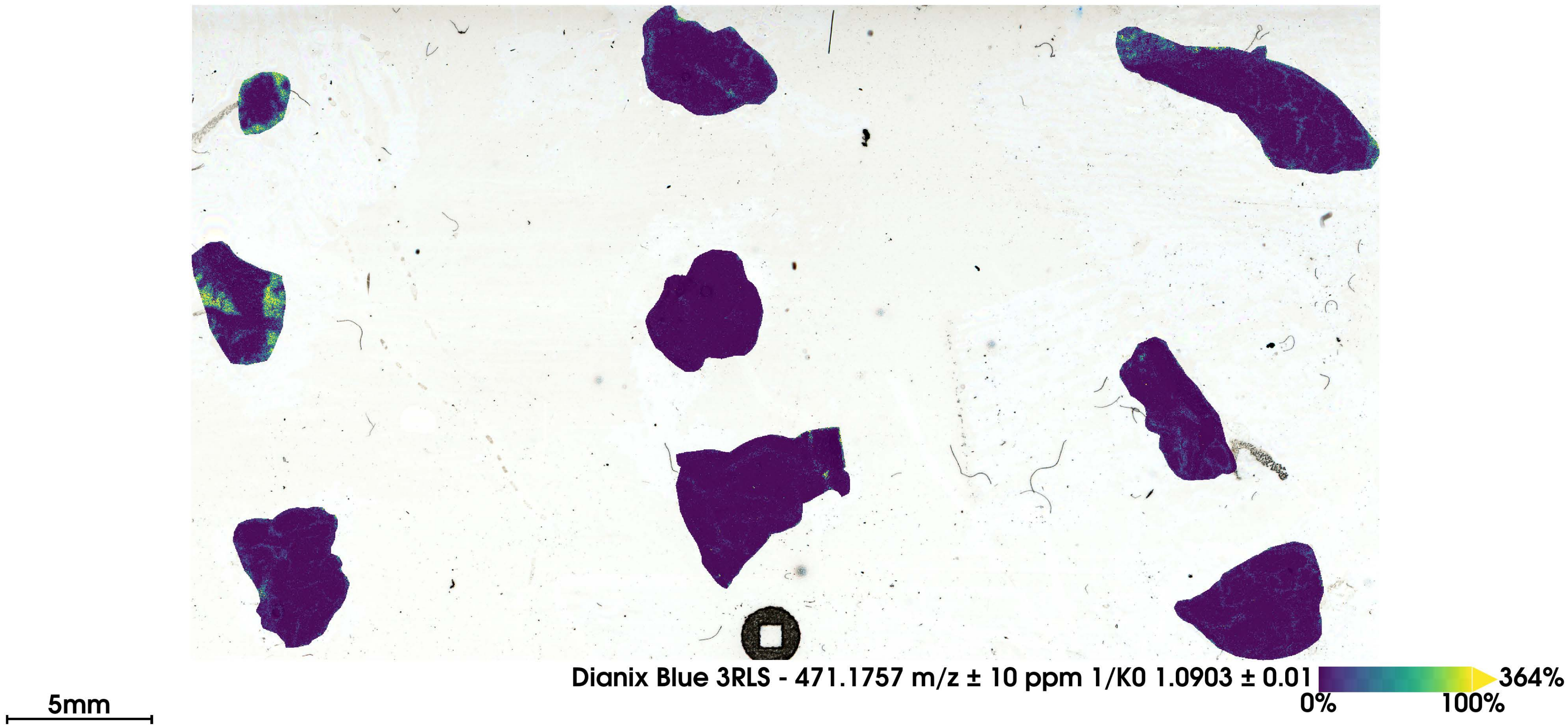
Dianix Blue 3RLS - 471.1757 m/z ± 10 ppm 1/K0 1.0903 ± 0.01
364%
0%
100%
5mm

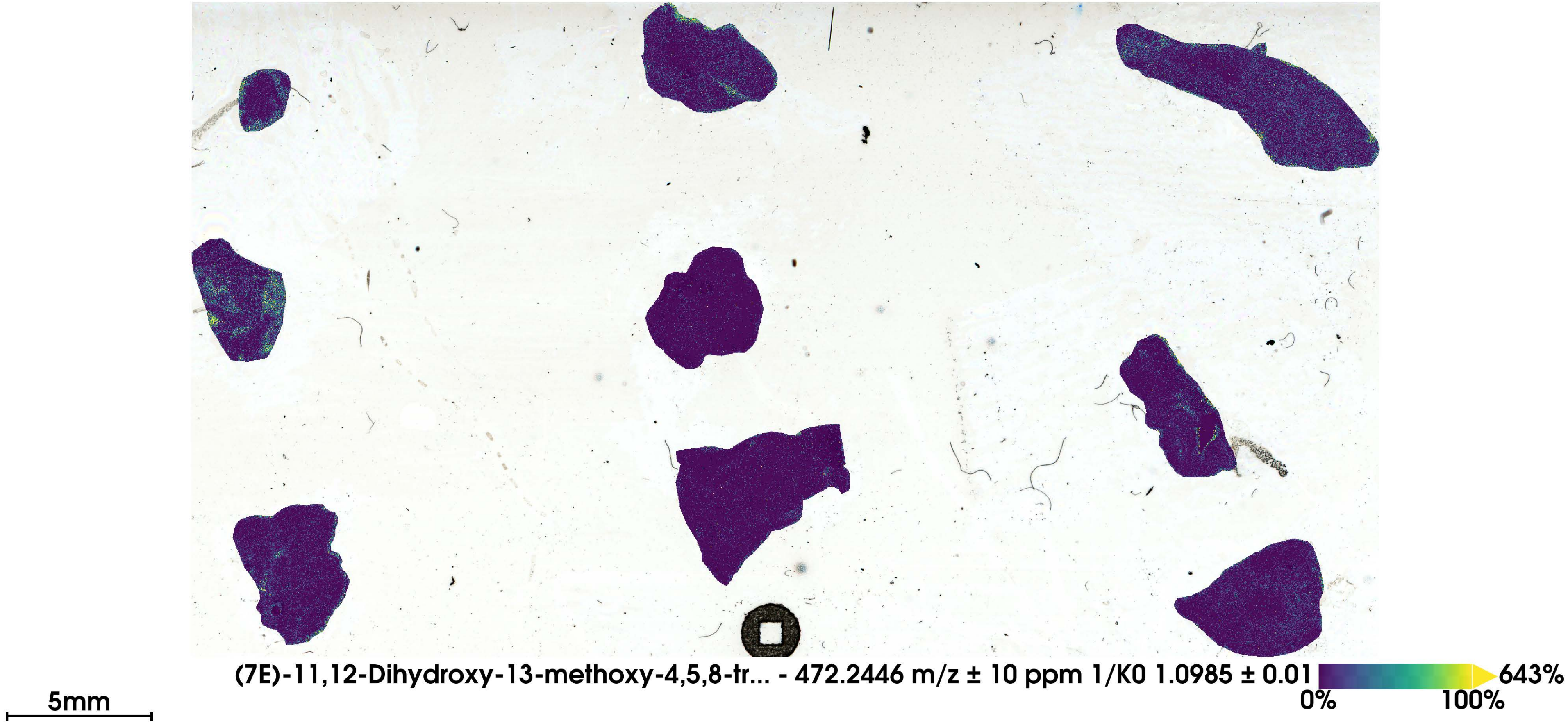
(7E)-11,12-Dihydroxy-13-methoxy-4,5,8-tr... - 472.2446 m/z ± 10 ppm 1/K0 1.0985 ± 0.01
643%
0%
100%
5mm

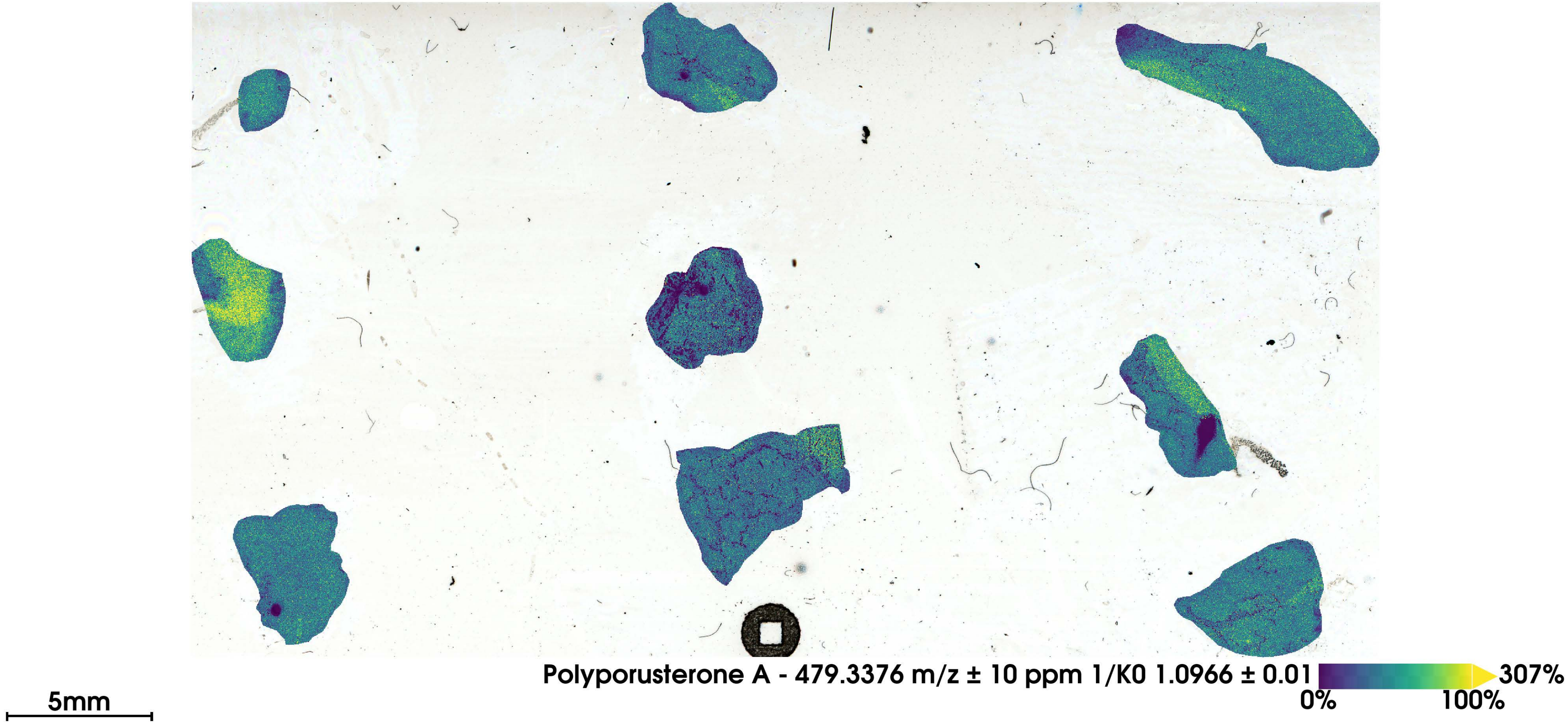
Polyporusterone A - 479.3376 m/z ± 10 ppm 1/K0 1.0966 ± 0.01
307%
0%
100%
5mm

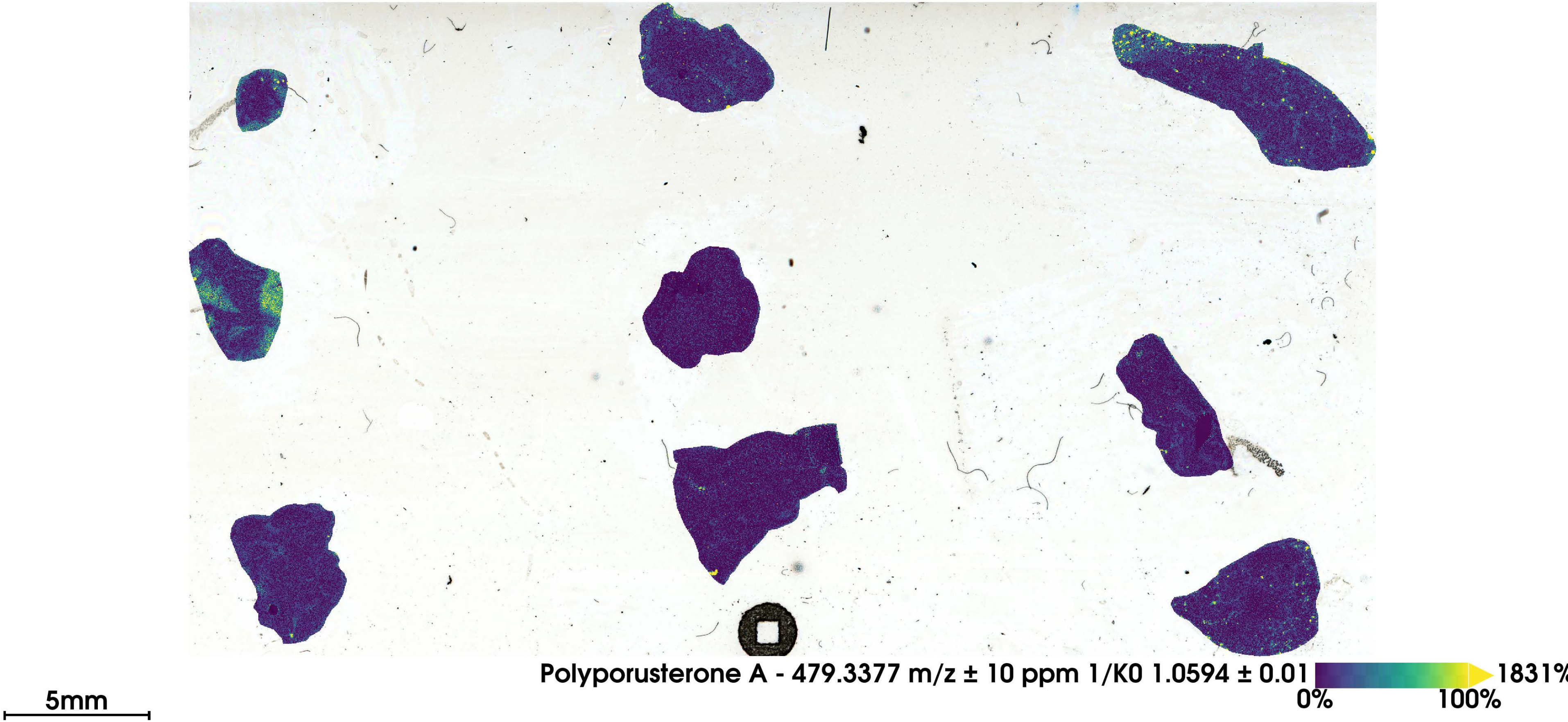

Polyporusterone A - 479.3377 m/z ± 10 ppm 1/K0 1.0594 ± 0.01
1831%
0%
100%
5mm

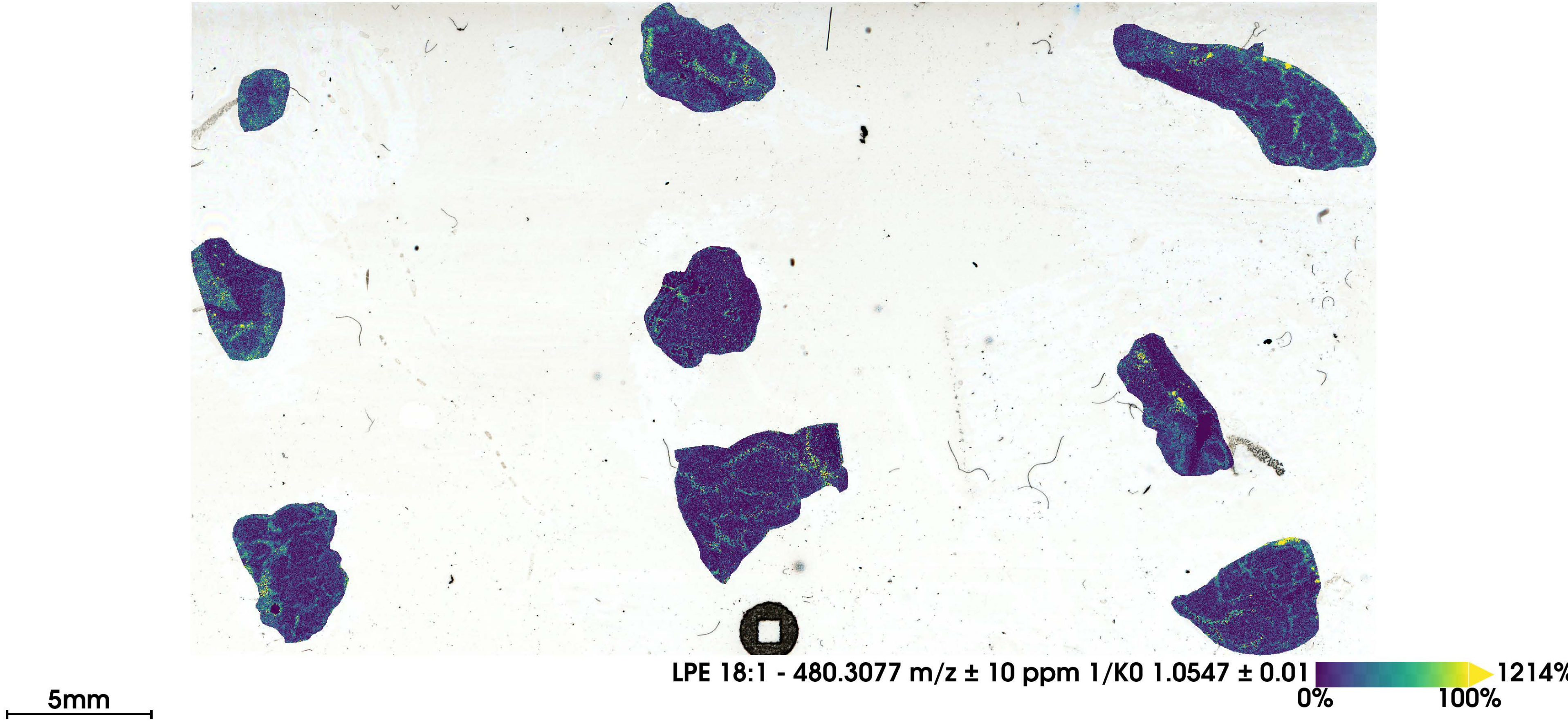

LPE 18:1 - 480.3077 m/z ± 10 ppm 1/K0 1.0547 ± 0.01
1214%
0%
100%
5mm

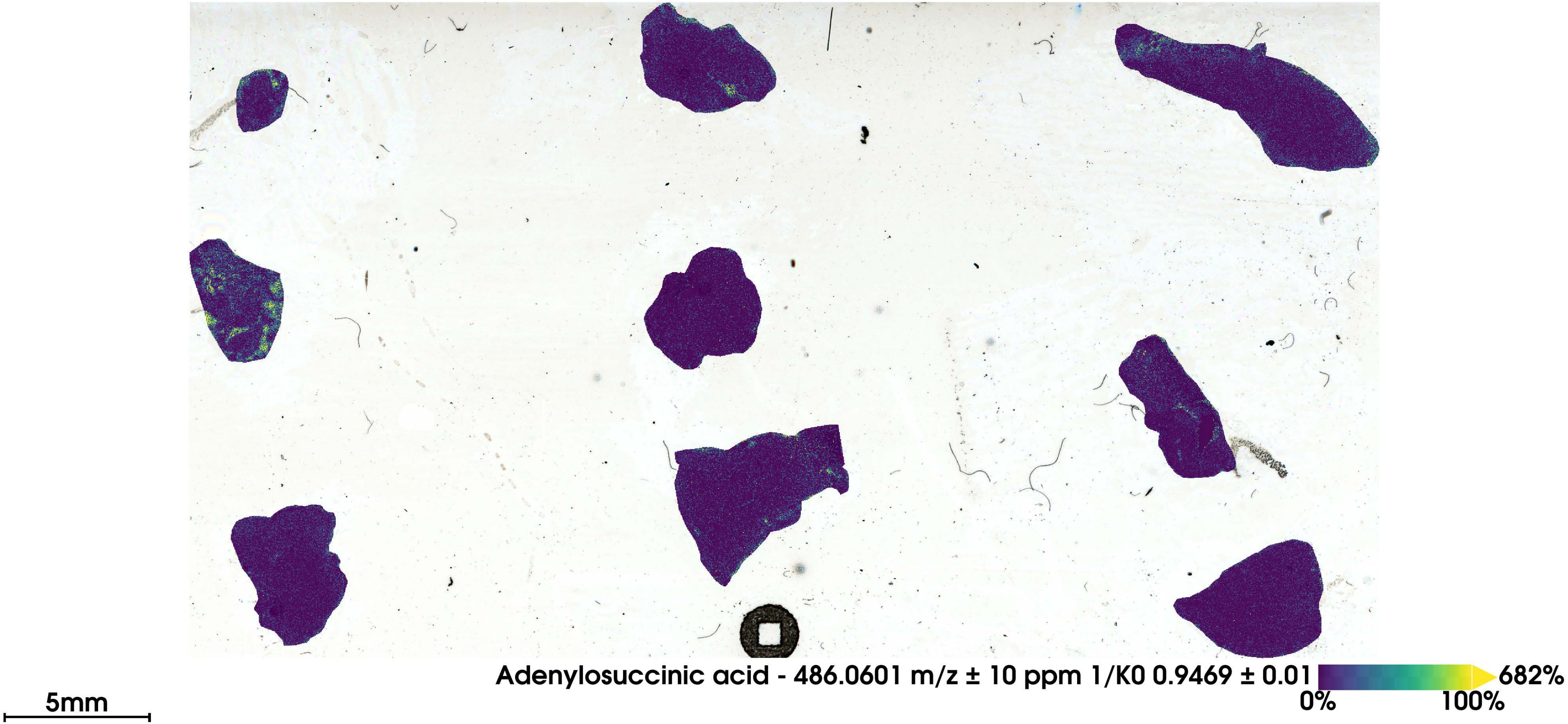
Adenylosuccinic acid - 486.0601 m/z ± 10 ppm 1/K0 0.9469 ± 0.01
682%
0%
100%
5mm

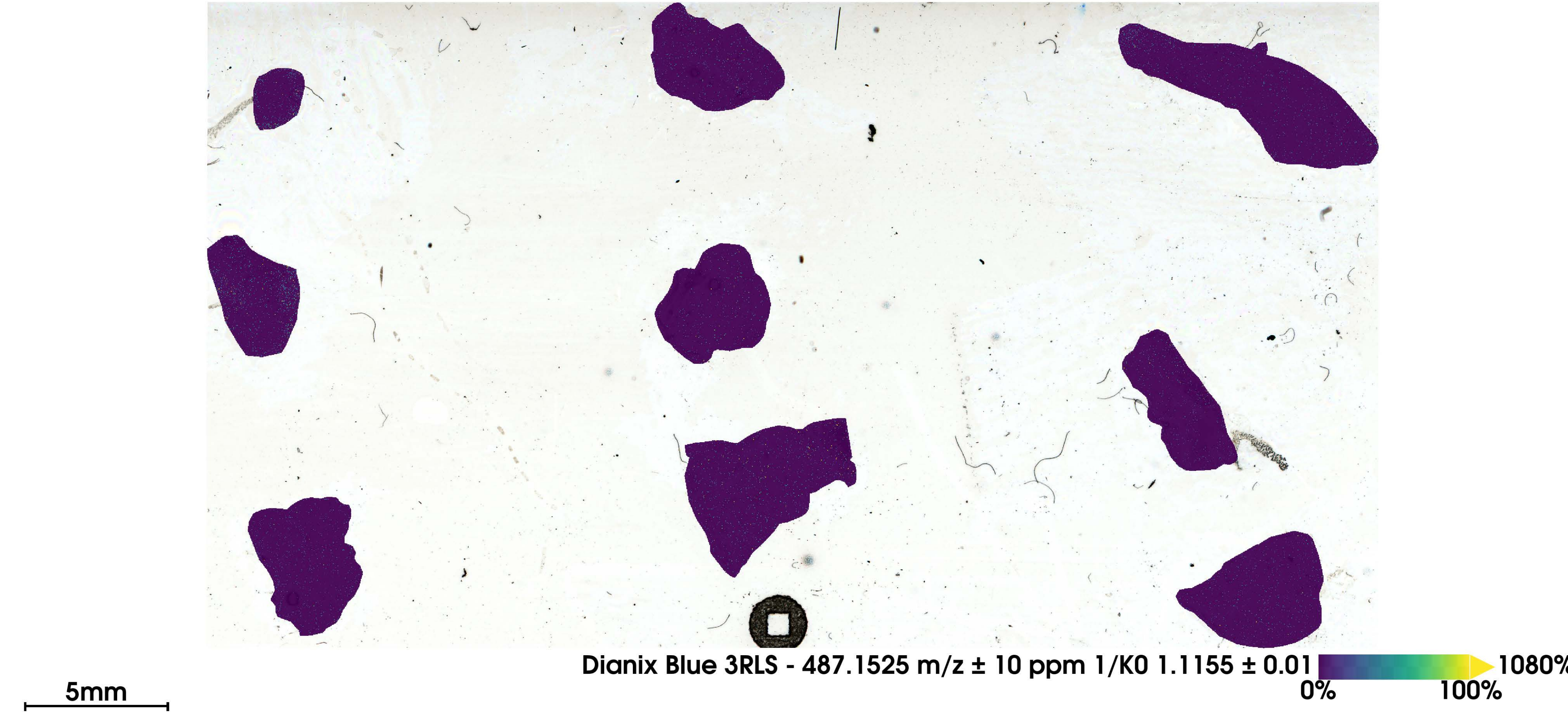

Dianix Blue 3RLS - 487.1525 m/z ± 10 ppm 1/K0 1.1155 ± 0.01
1080%
0%
100%
5mm

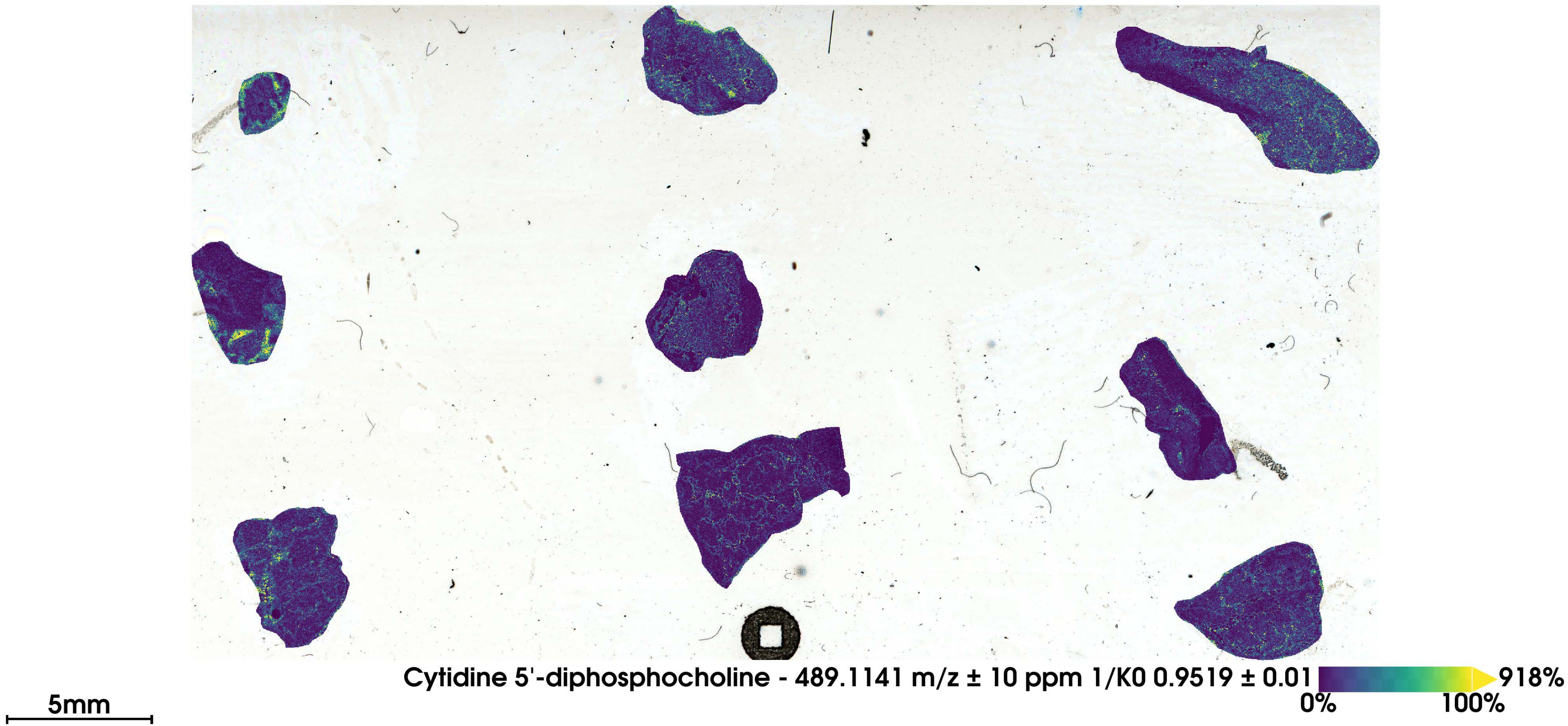
Cytidine 5'-diphosphocholine - 489.1141 m/z ± 10 ppm 1/K0 0.9519 ± 0.01
918%
0%
100%
5mm

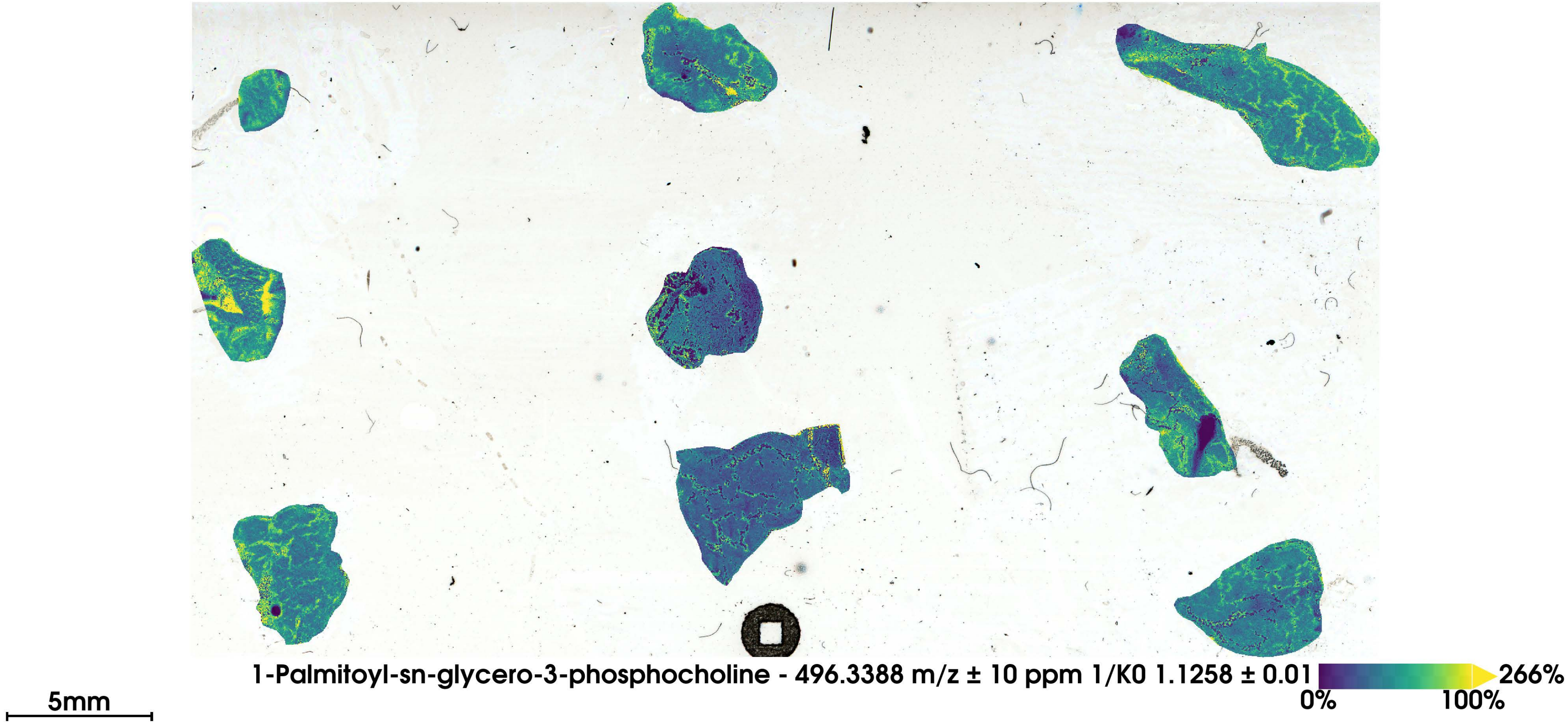

1-Palmitoyl-sn-glycero-3-phosphocholine - 496.3388 m/z ± 10 ppm 1/K0 1.1258 ± 0.01
266%
0%
100%
5mm

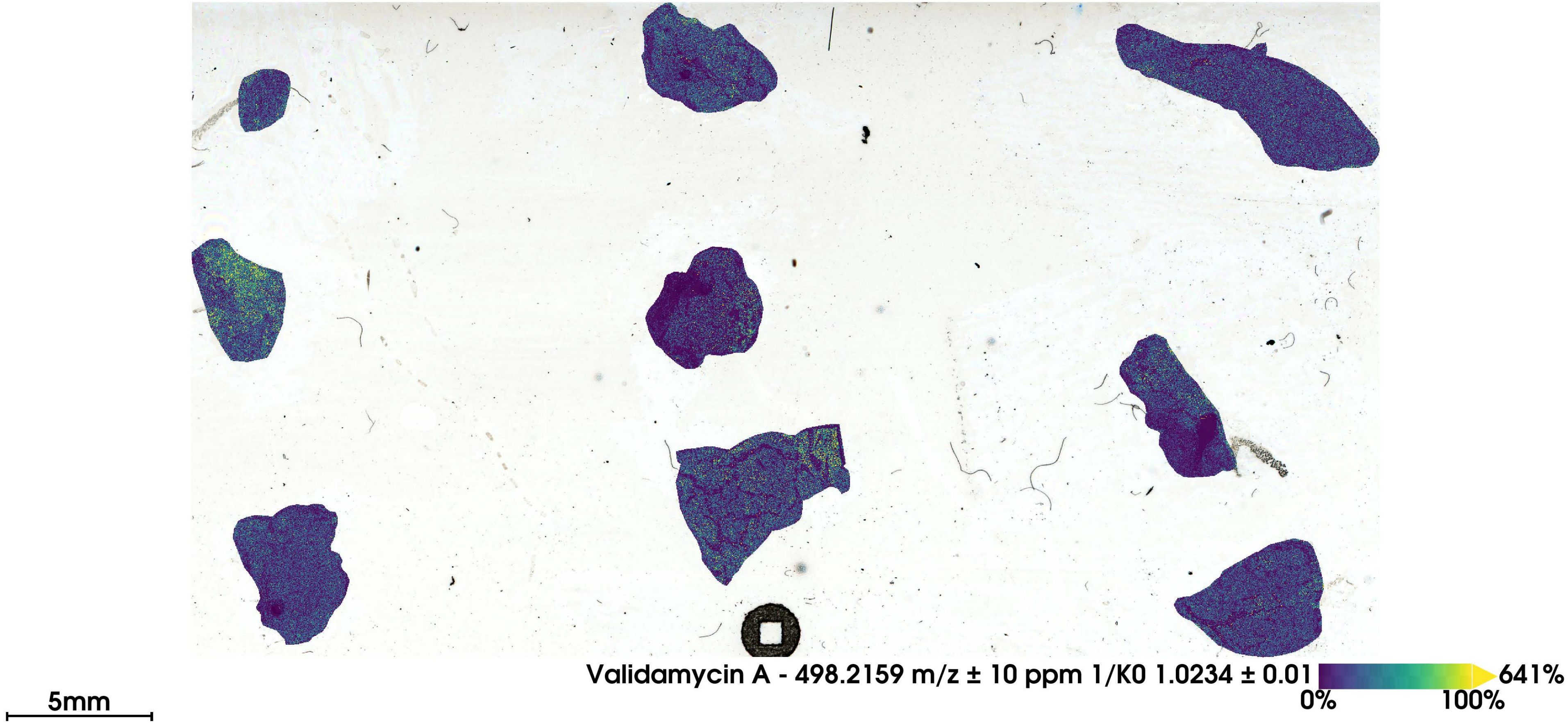
Validamycin A - 498.2159 m/z ± 10 ppm 1/K0 1.0234 ± 0.01
641%
0%
100%
5mm

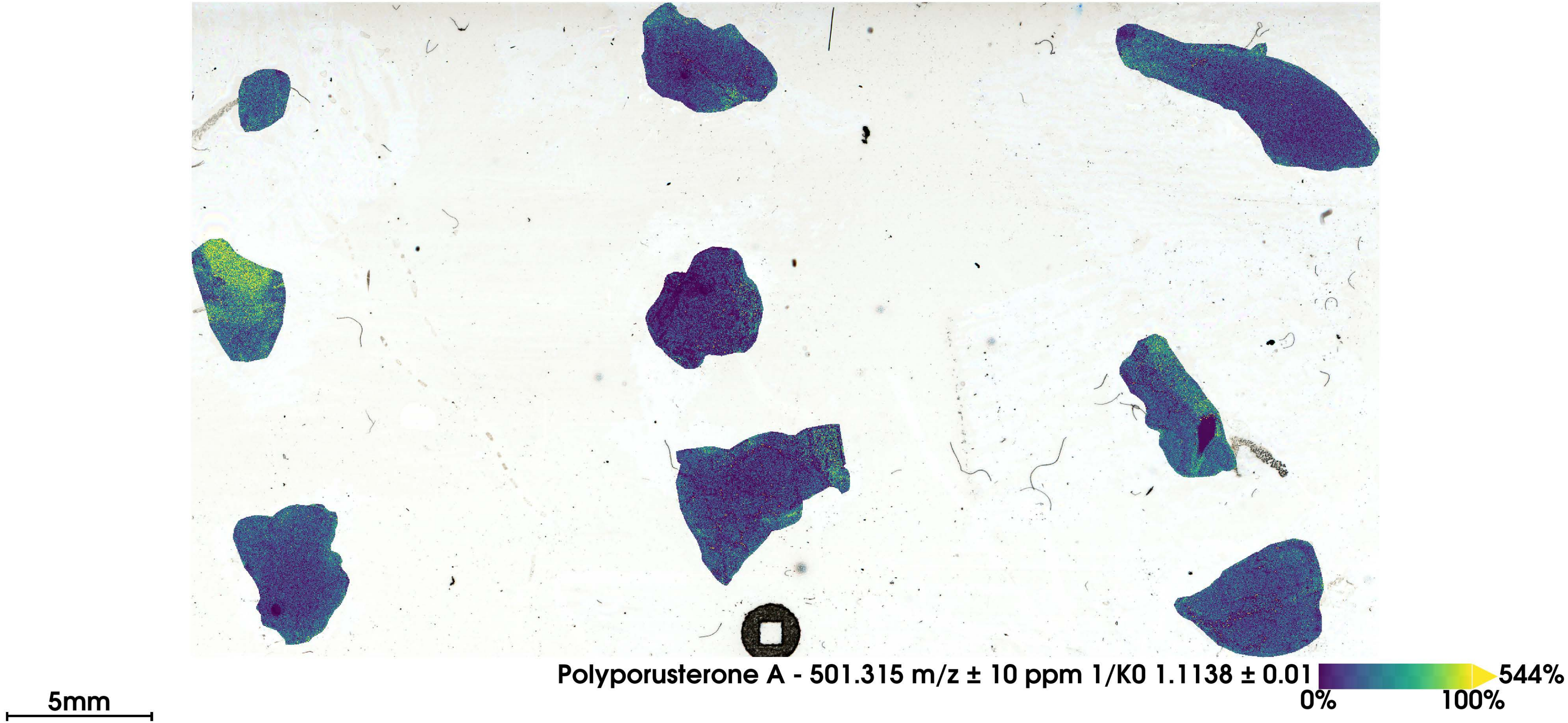
Polyporusterone A - 501.315 m/z ± 10 ppm 1/K0 1.1138 ± 0.01
544%
0%
100%
5mm

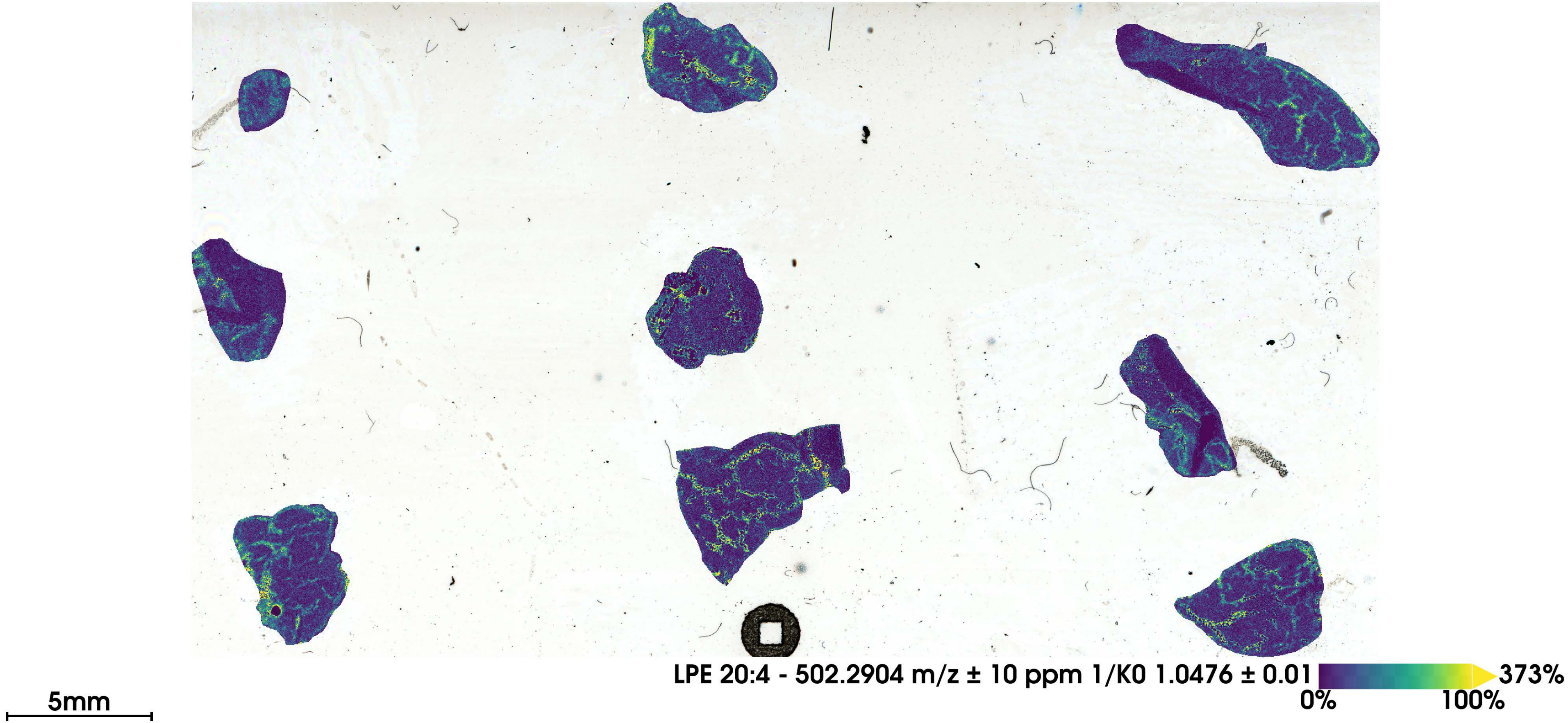
LPE 20:4 - 502.2904 m/z ± 10 ppm 1/K0 1.0476 ± 0.01
373%
0%
100%
5mm

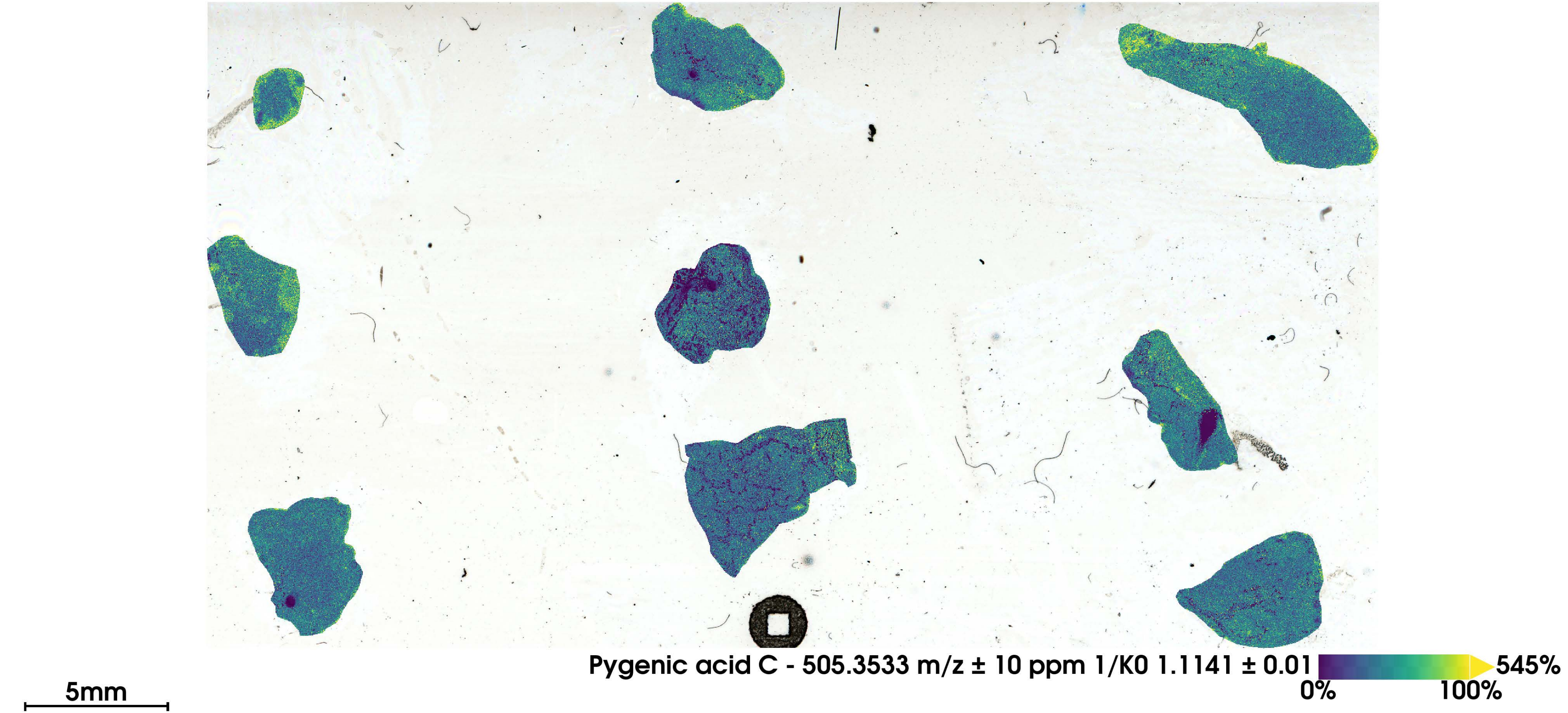

Pygenic acid C - 505.3533 m/z ± 10 ppm 1/K0 1.1141 ± 0.01
545%
0%
100%
5mm

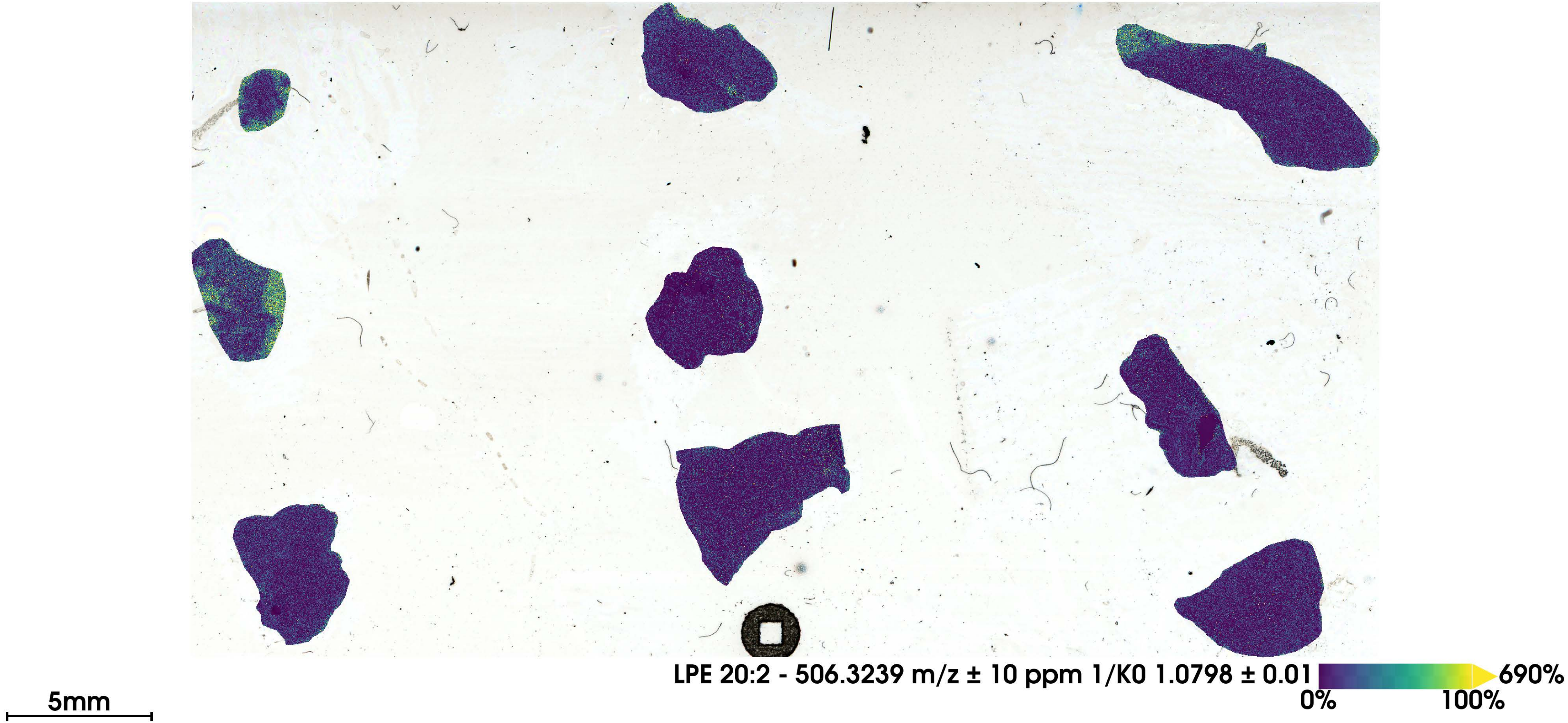
LPE 20:2 - 506.3239 m/z ± 10 ppm 1/K0 1.0798 ± 0.01
690%
0%
100%
5mm

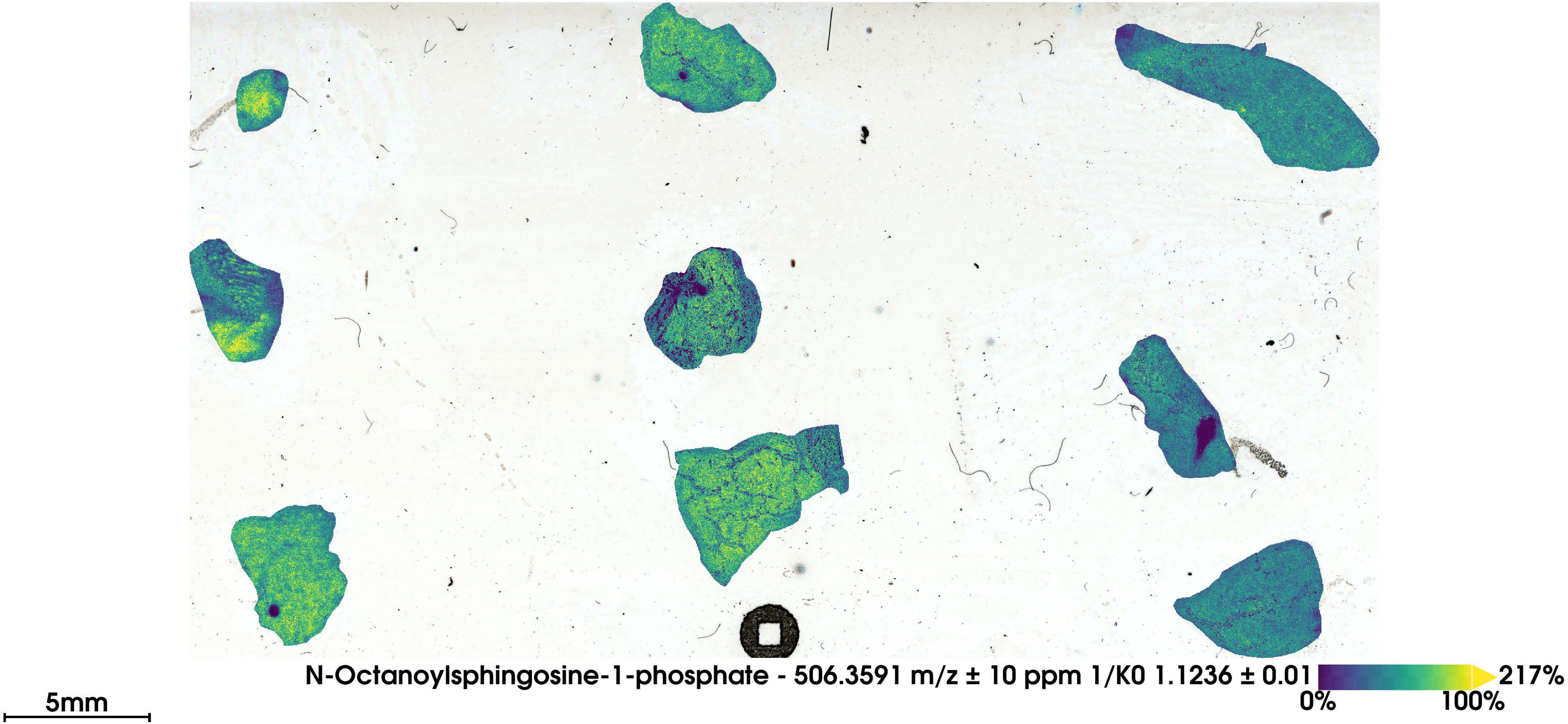
N-Octanoylsphingosine-1-phosphate - 506.3591 m/z ± 10 ppm 1/K0 1.1236 ± 0.01
217%
0%
100%
5mm

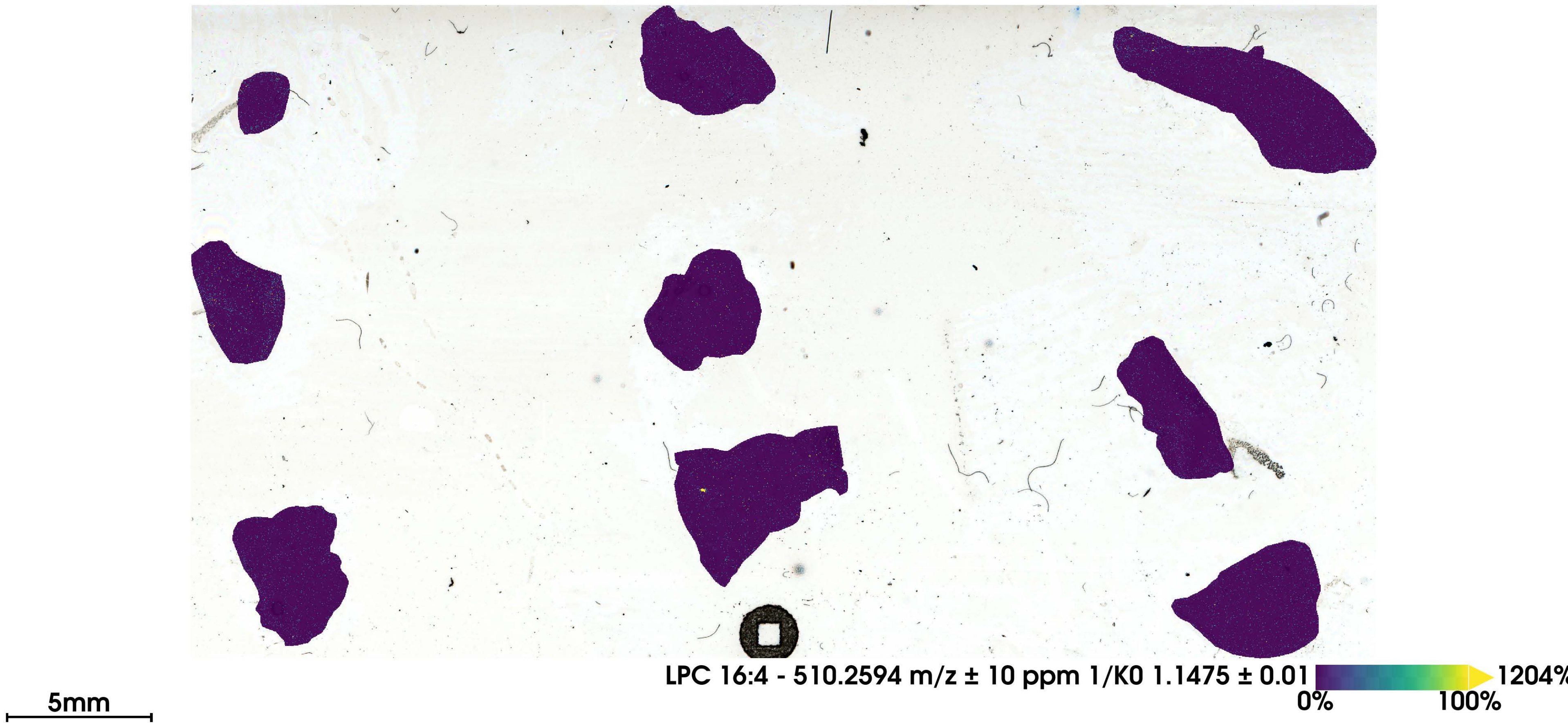

LPC 16:4 - 510.2594 m/z ± 10 ppm 1/K0 1.1475 ± 0.01
1204%
0%
100%
5mm

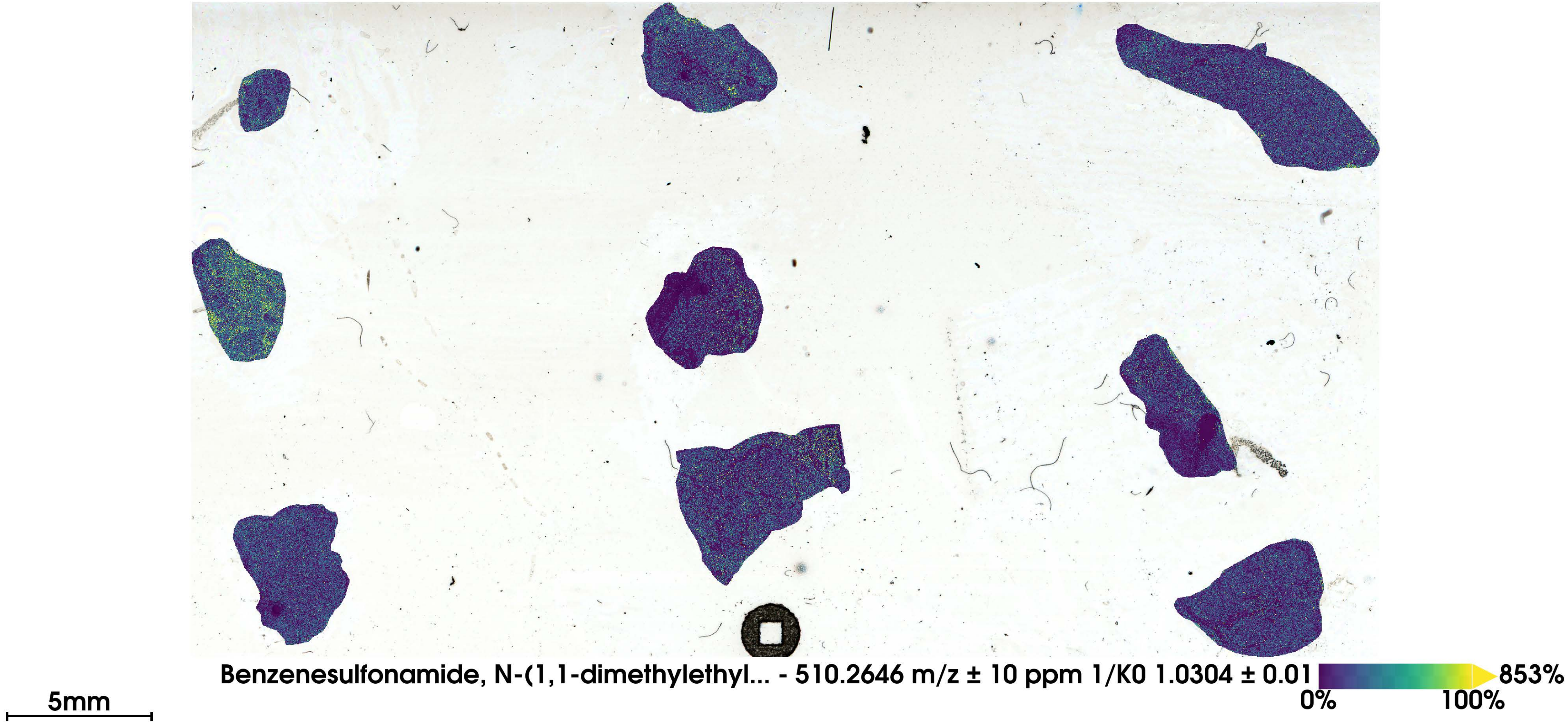
Benzenesulfonamide, N-(1,1-dimethylethyl... - 510.2646 m/z ± 10 ppm 1/K0 1.0304 ± 0.01
853%
0%
100%
5mm

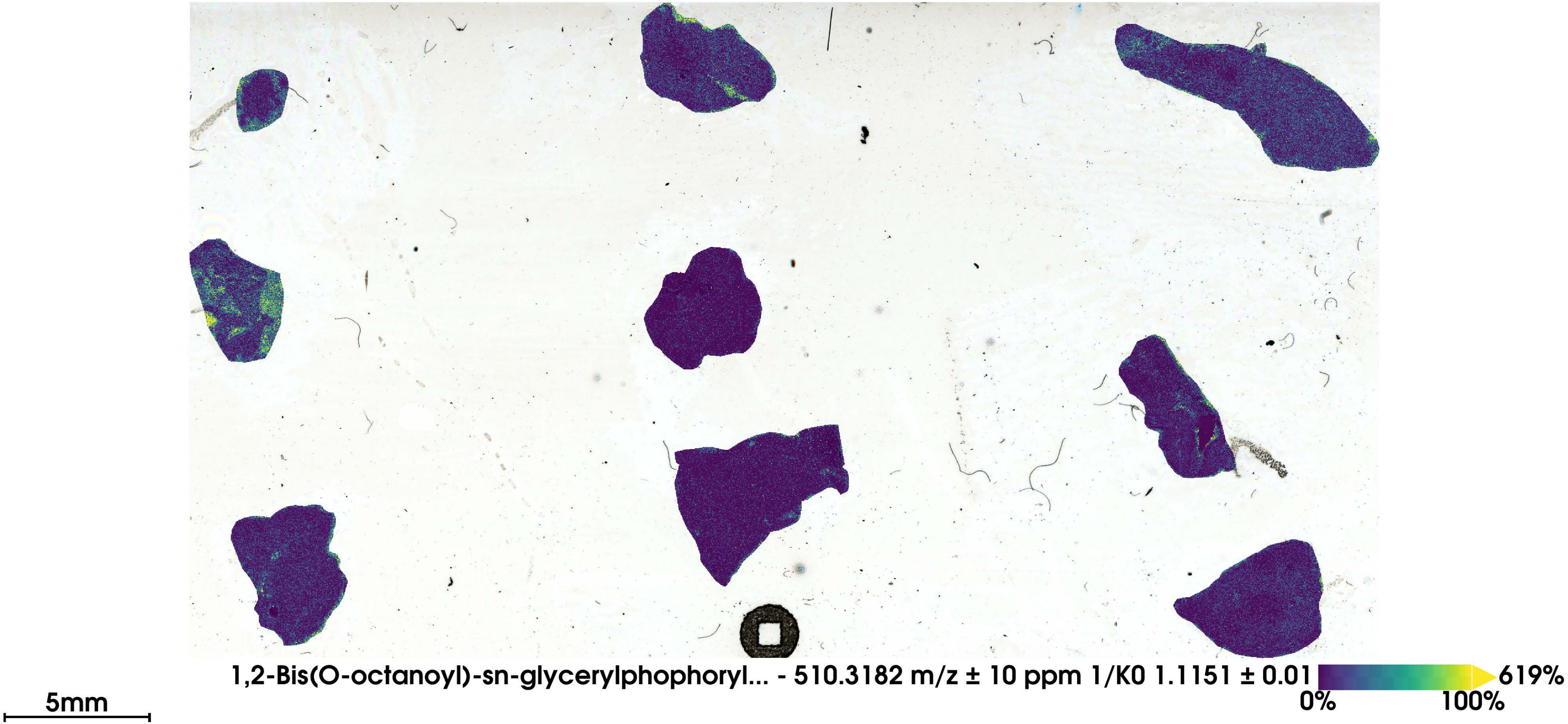

1,2-Bis(O-octanoyl)-sn-glycerylphophoryl... - 510.3182 m/z ± 10 ppm 1/K0 1.1151 ± 0.01
619%
0%
100%
5mm

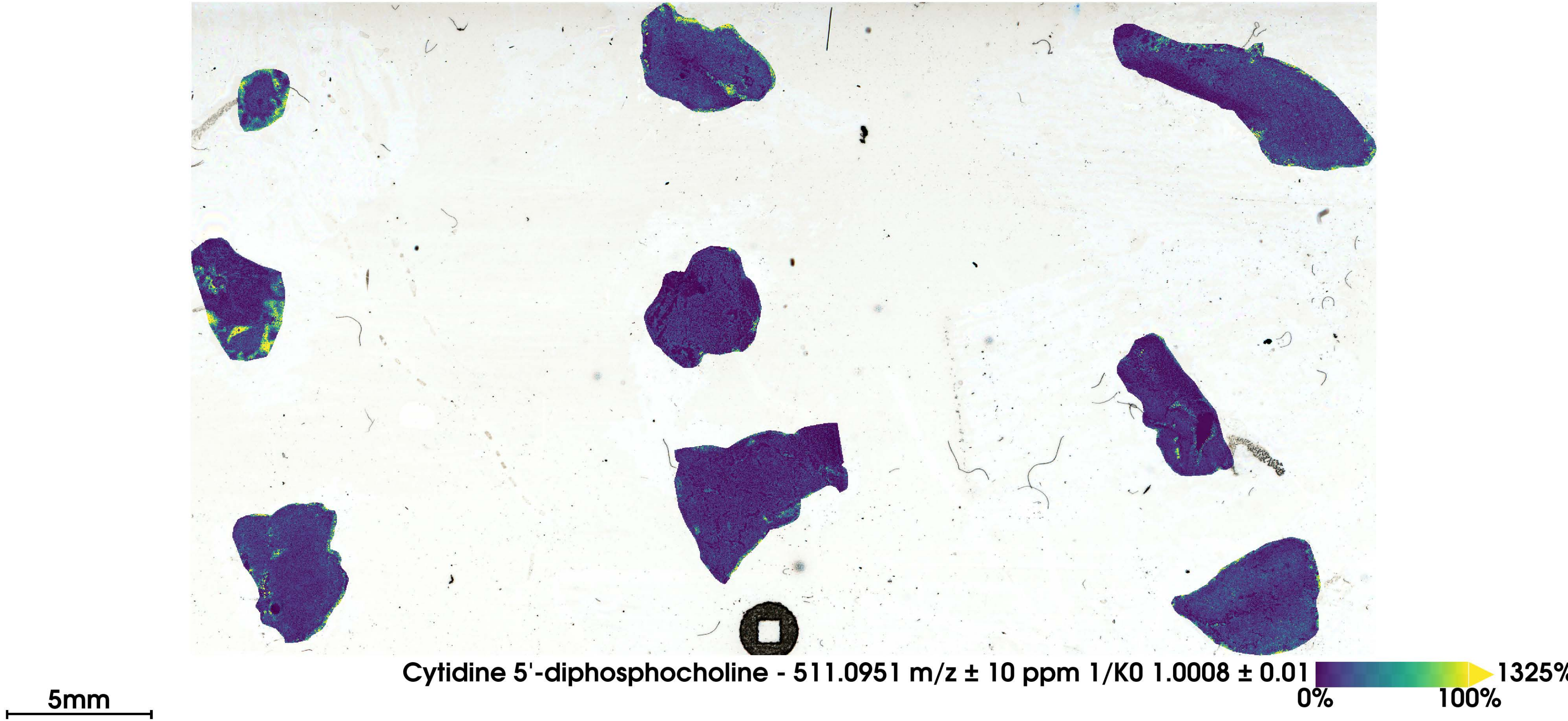
Cytidine 5'-diphosphocholine - 511.0951 m/z ± 10 ppm 1/K0 1.0008 ± 0.01
1325%
0%
100%
5mm

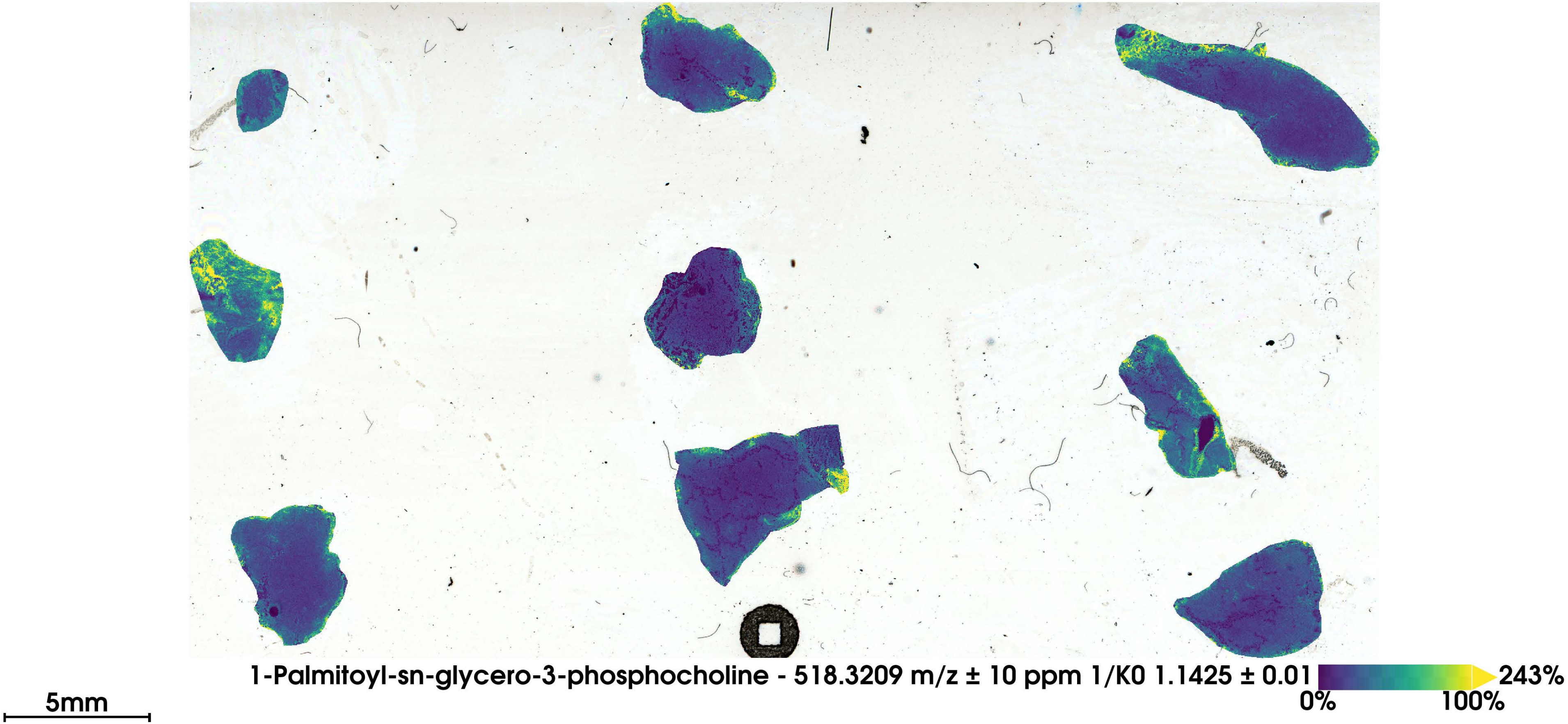

1-Palmitoyl-sn-glycero-3-phosphocholine - 518.3209 m/z ± 10 ppm 1/K0 1.1425 ± 0.01
243%
0%
100%
5mm

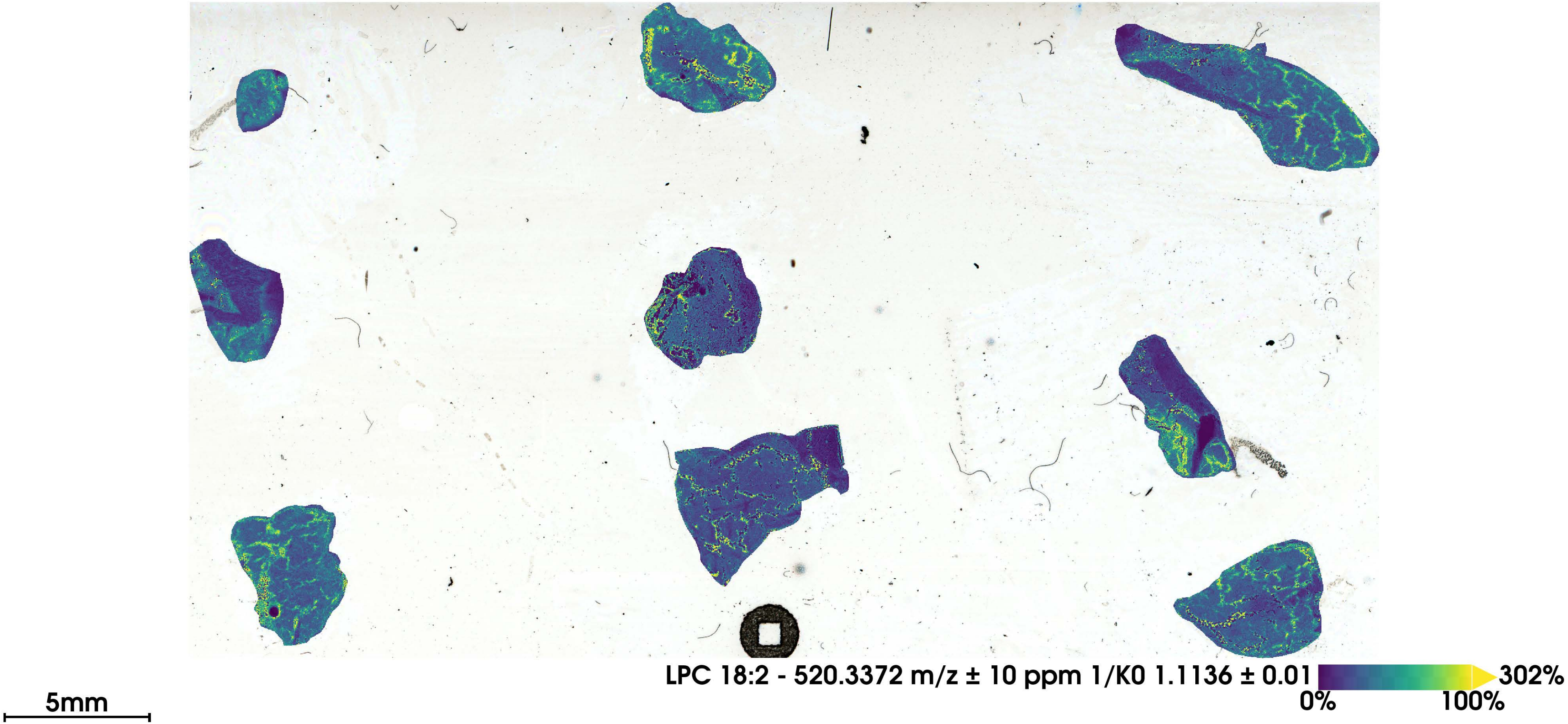
LPC 18:2 - 520.3372 m/z ± 10 ppm 1/K0 1.1136 ± 0.01
302%
0%
100%
5mm

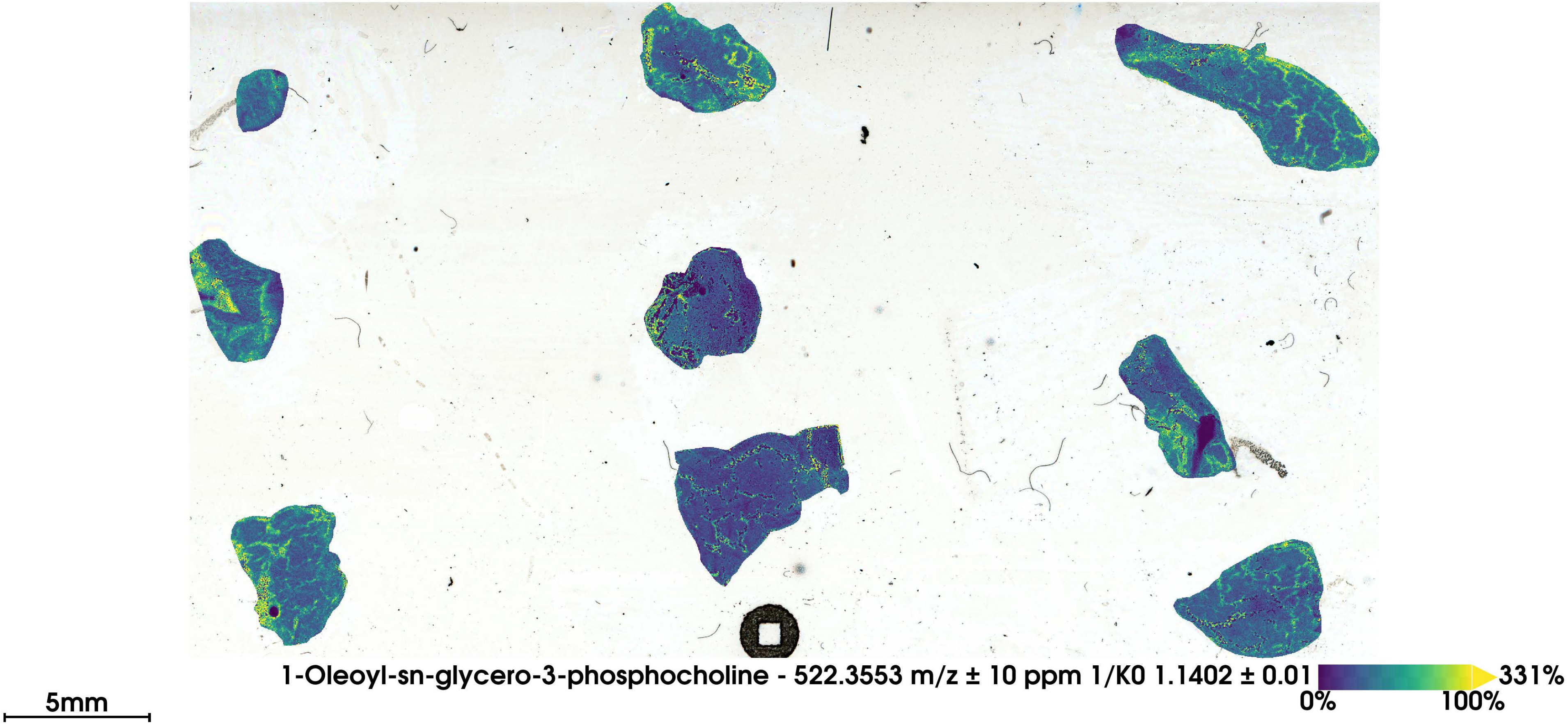
1-Oleoyl-sn-glycero-3-phosphocholine - 522.3553 m/z ± 10 ppm 1/K0 1.1402 ± 0.01
331%
0%
100%
5mm

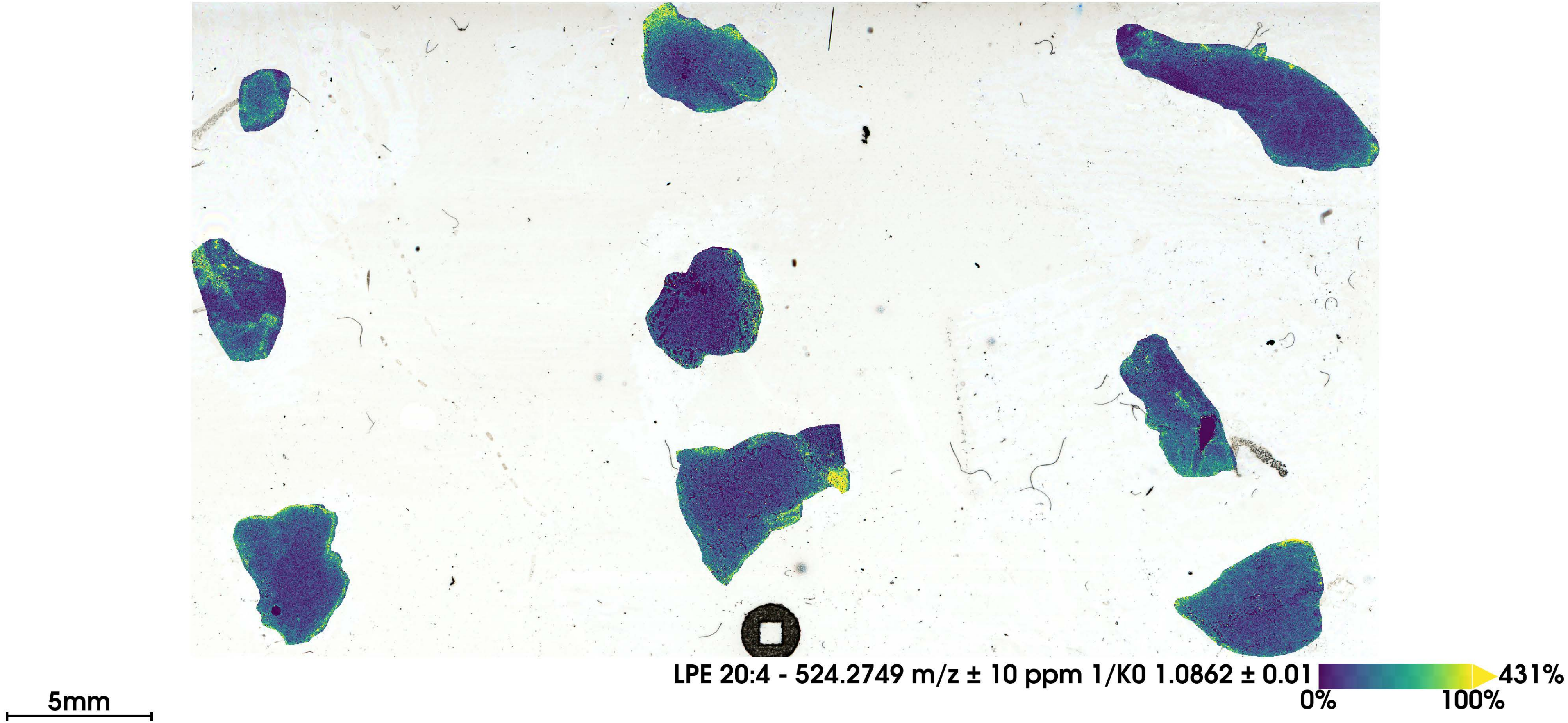
LPE 20:4 - 524.2749 m/z ± 10 ppm 1/K0 1.0862 ± 0.01
431%
0%
100%
5mm

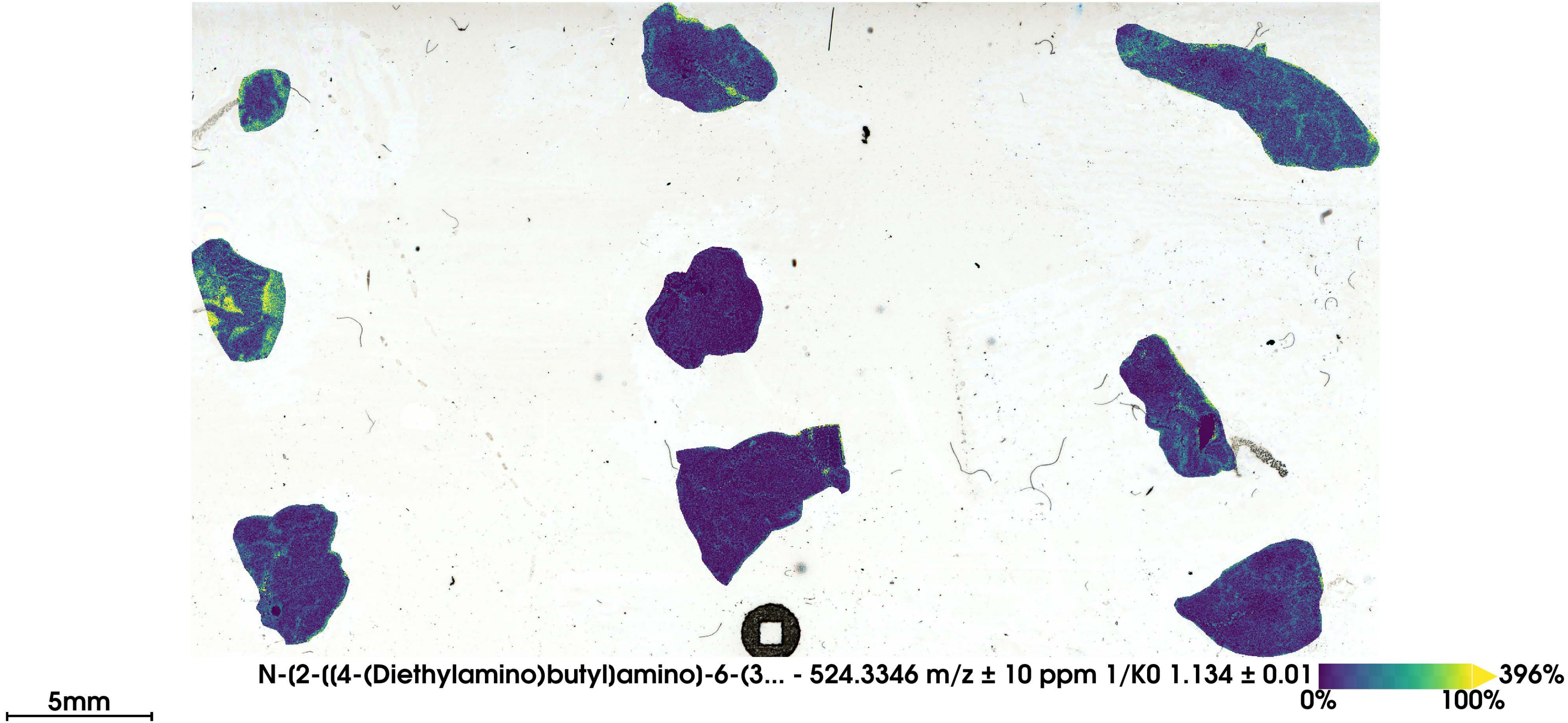
N-(2-((4-(Diethylamino)butyl)amino)-6-(3... - 524.3346 m/z ± 10 ppm 1/K0 1.134 ± 0.01
396%
0%
100%
5mm

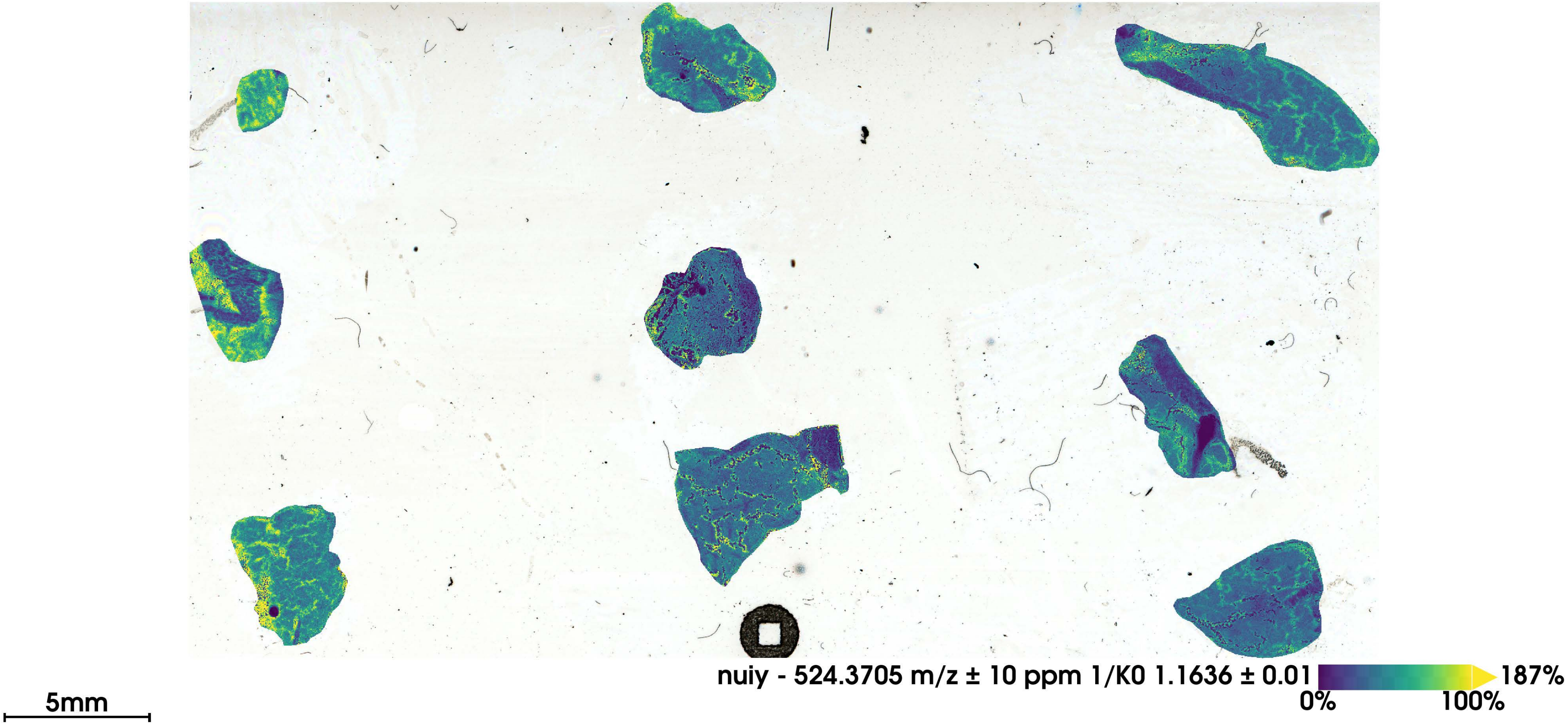
nuiy - 524.3705 m/z ± 10 ppm 1/K0 1.1636 ± 0.01
187%
0%
100%
5mm

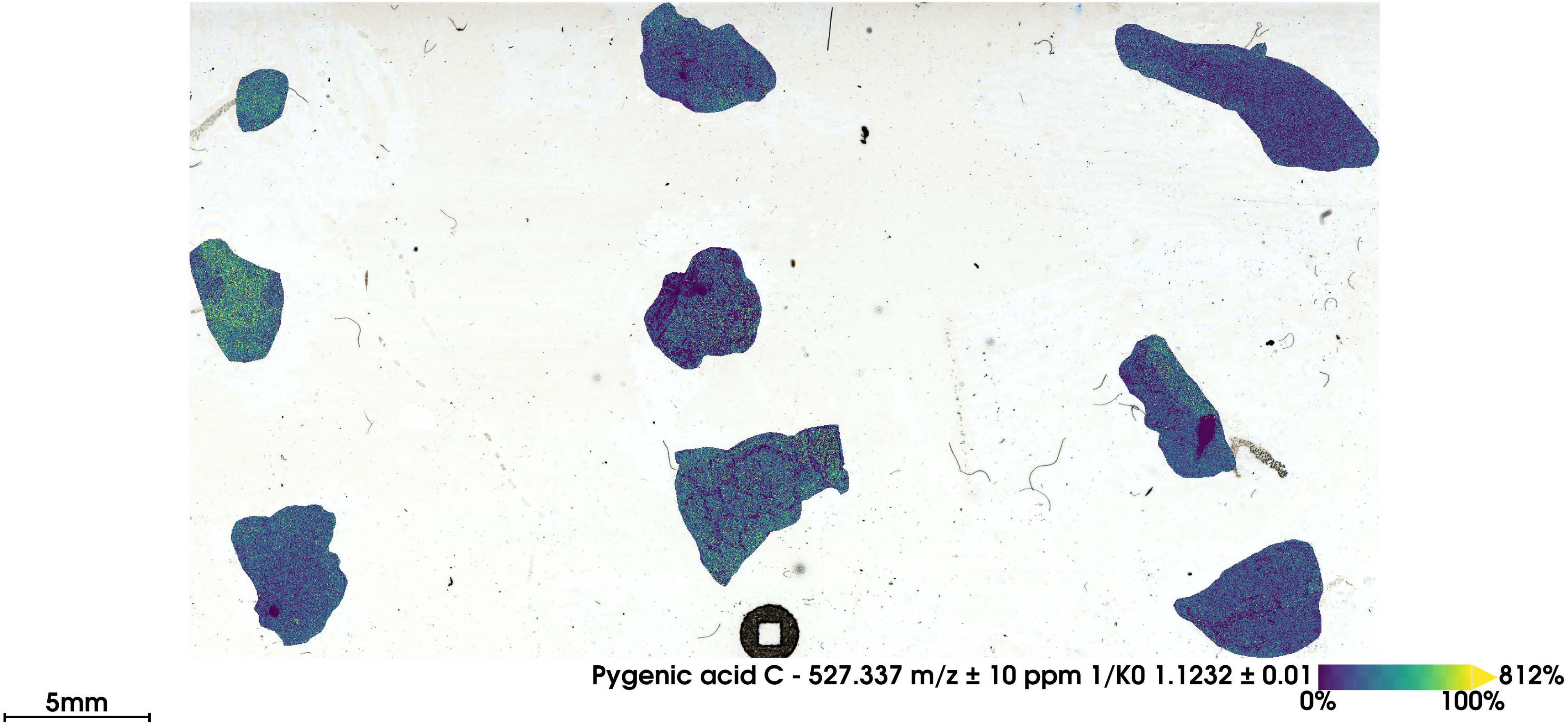

Pygenic acid C - 527.337 m/z ± 10 ppm 1/K0 1.1232 ± 0.01
812%
0%
100%
5mm

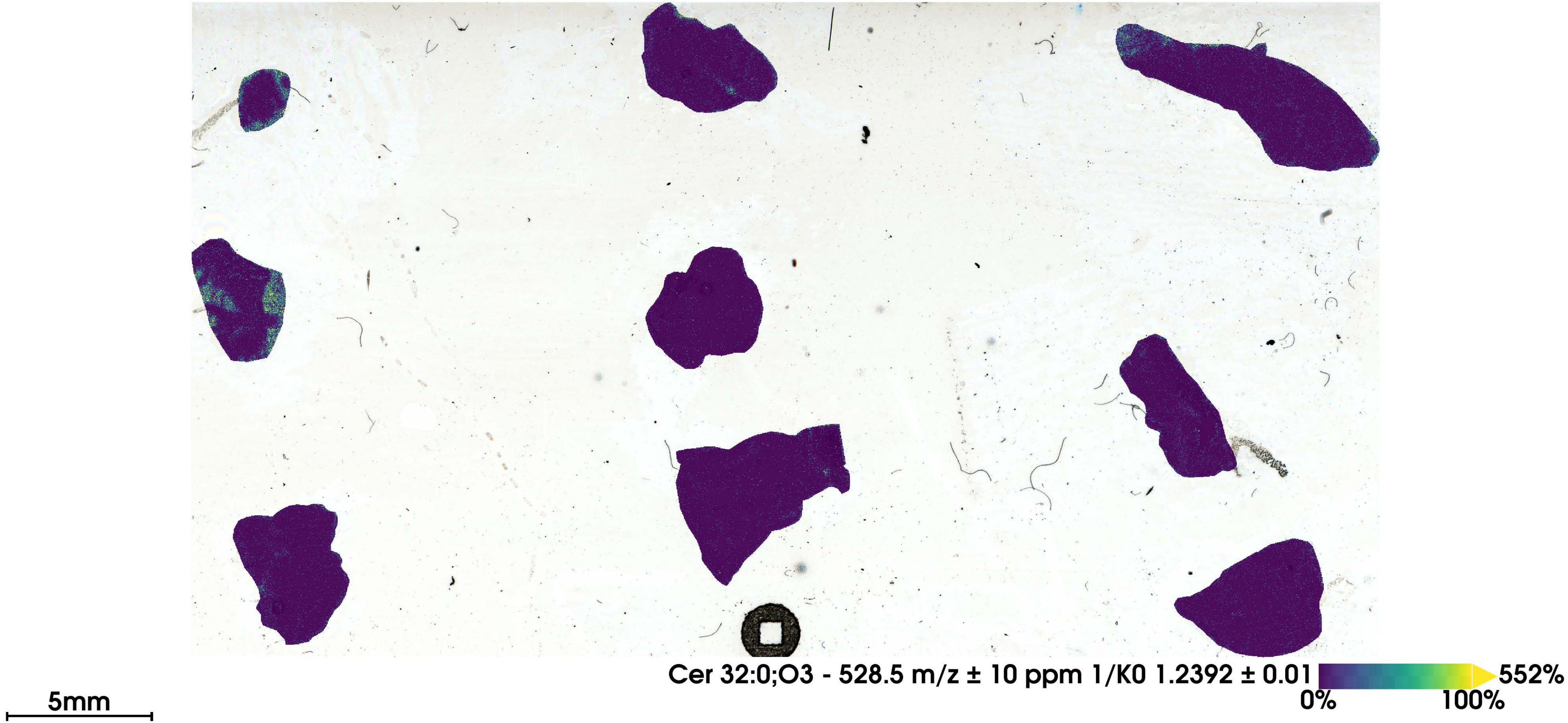
Cer 32:0;O3 - 528.5 m/z ± 10 ppm 1/K0 1.2392 ± 0.01
552%
0%
100%
5mm

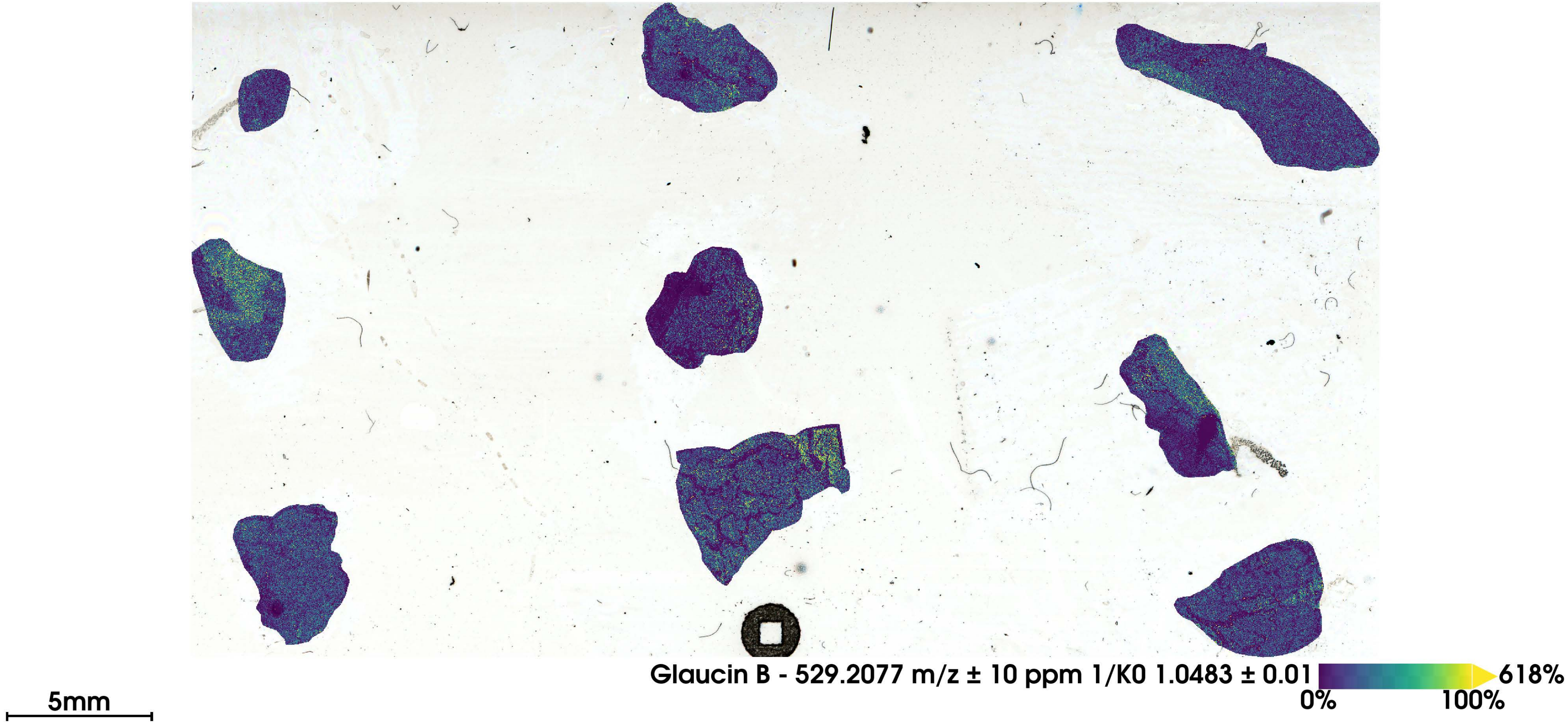
Glaucin B - 529.2077 m/z ± 10 ppm 1/K0 1.0483 ± 0.01
618%
0%
100%
5mm

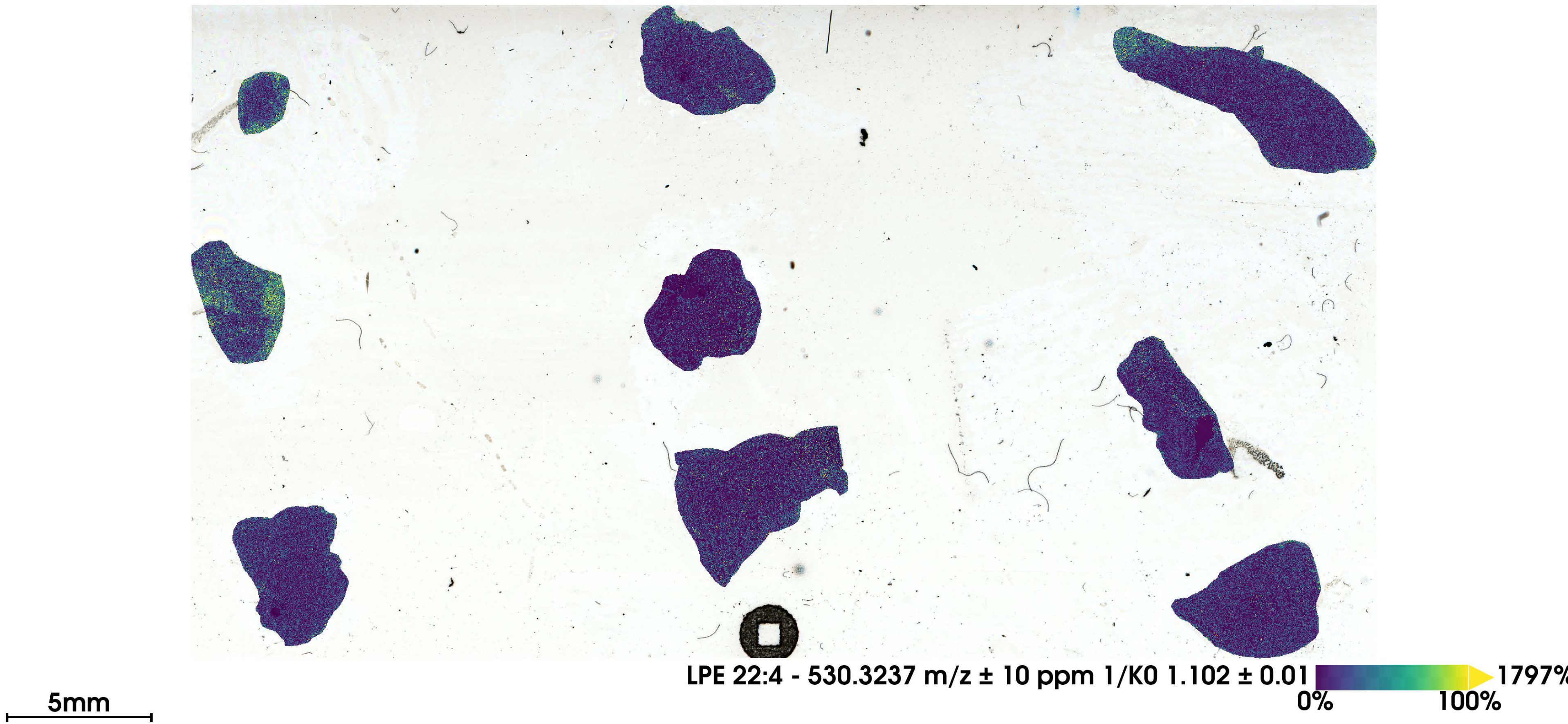

LPE 22:4 - 530.3237 m/z ± 10 ppm 1/K0 1.102 ± 0.01
1797%
0%
100%
5mm

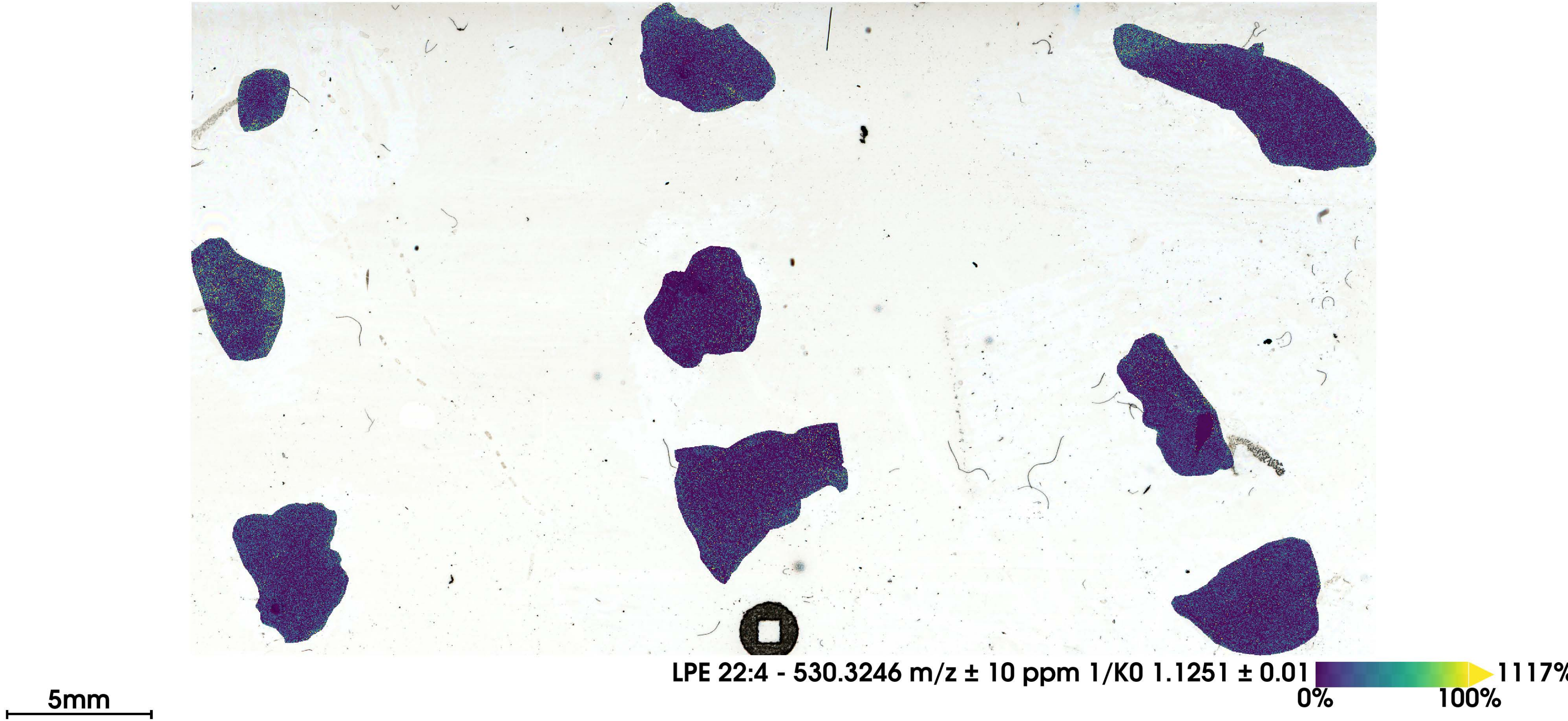

LPE 22:4 - 530.3246 m/z ± 10 ppm 1/K0 1.1251 ± 0.01
0%
100%
1117%
5mm

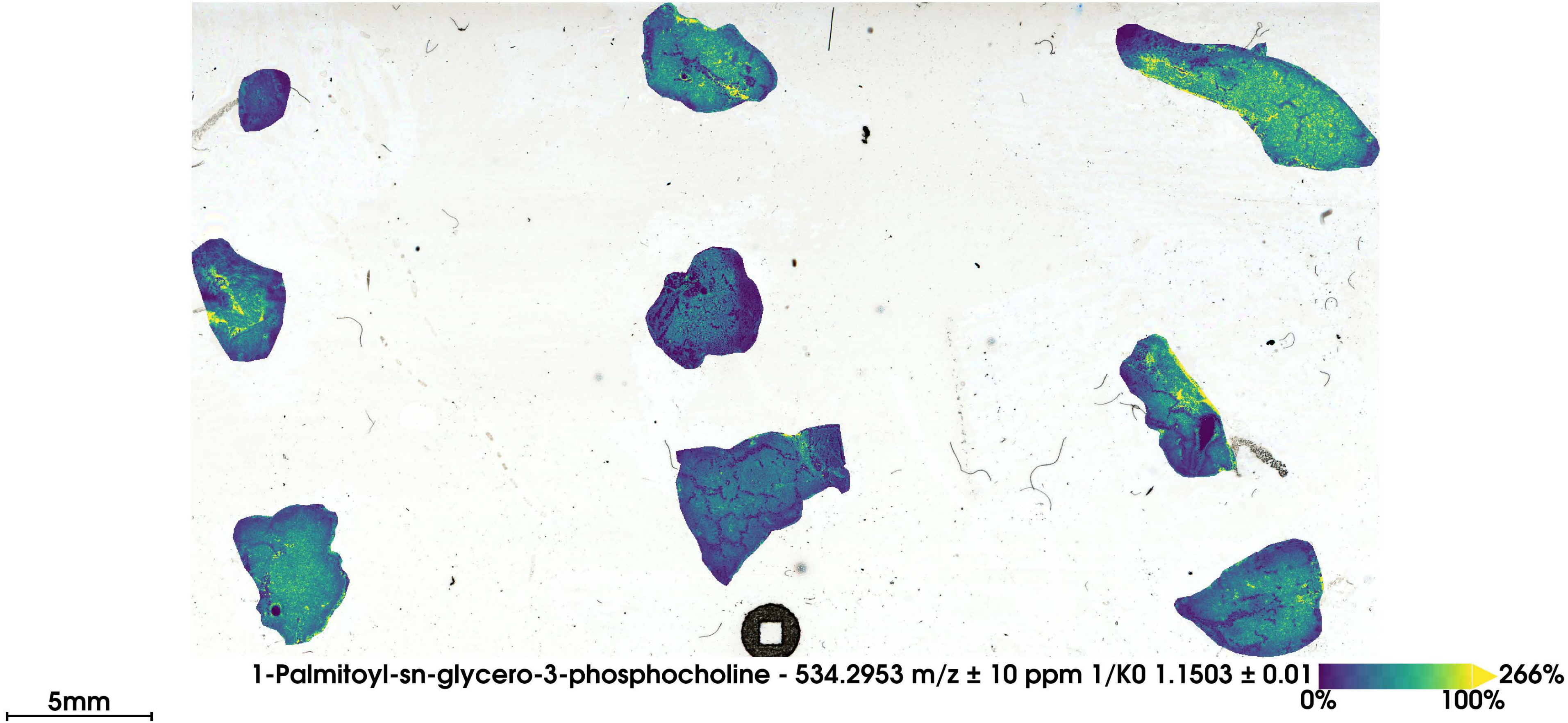

1-Palmitoyl-sn-glycero-3-phosphocholine - 534.2953 m/z ± 10 ppm 1/K0 1.1503 ± 0.01
266%
0%
100%
5mm

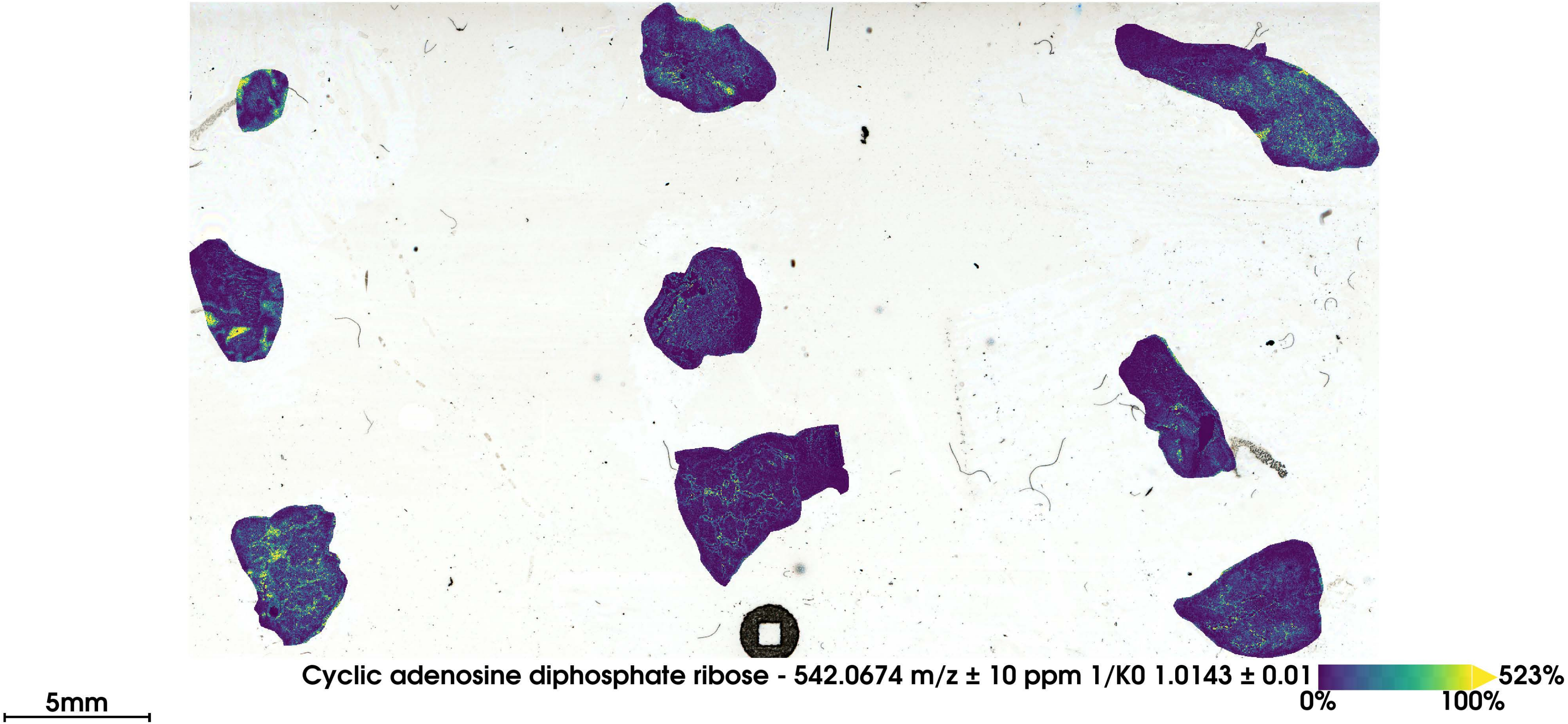
Cyclic adenosine diphosphate ribose - 542.0674 m/z ± 10 ppm 1/K0 1.0143 ± 0.01
523%
0%
100%
5mm

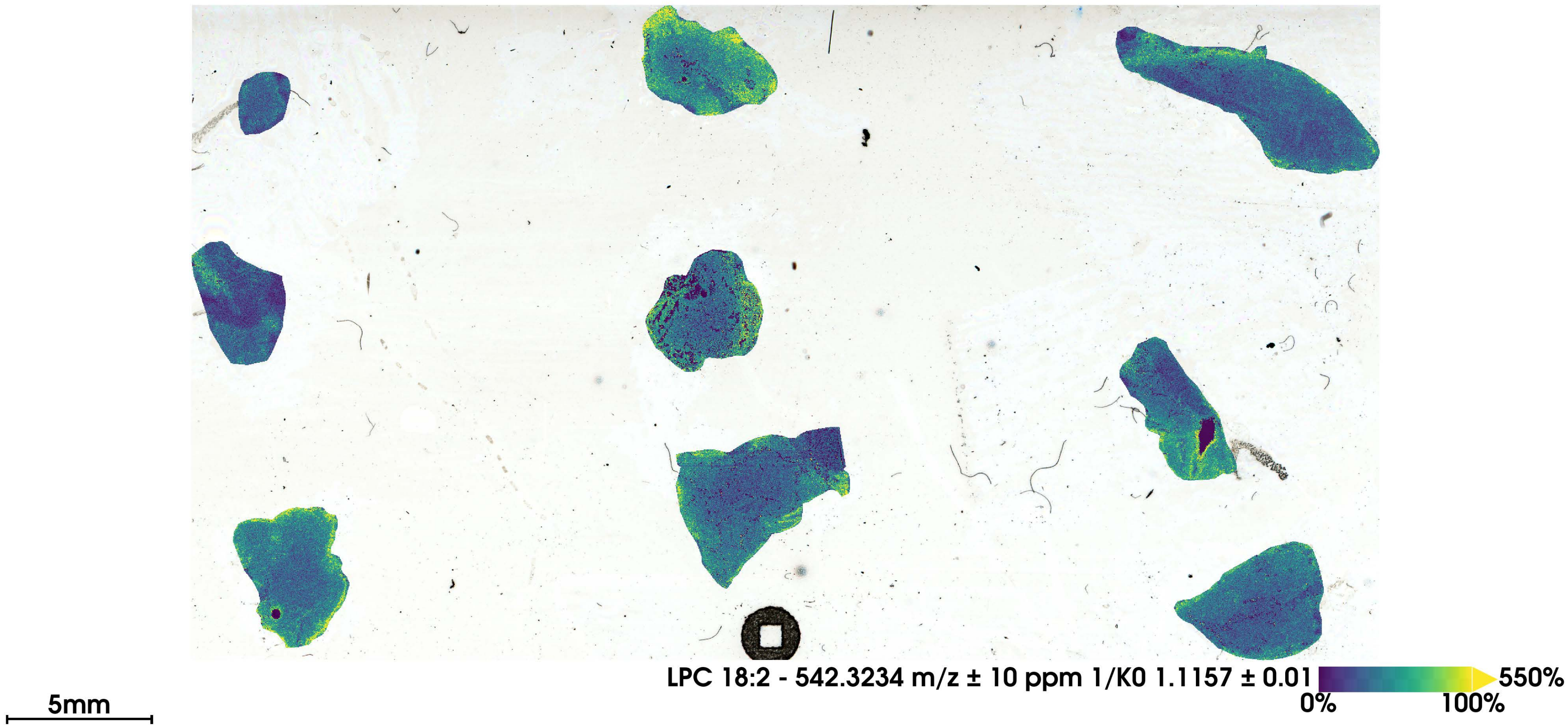

LPC 18:2 - 542.3234 m/z ± 10 ppm 1/K0 1.1157 ± 0.01
550%
0%
100%
5mm

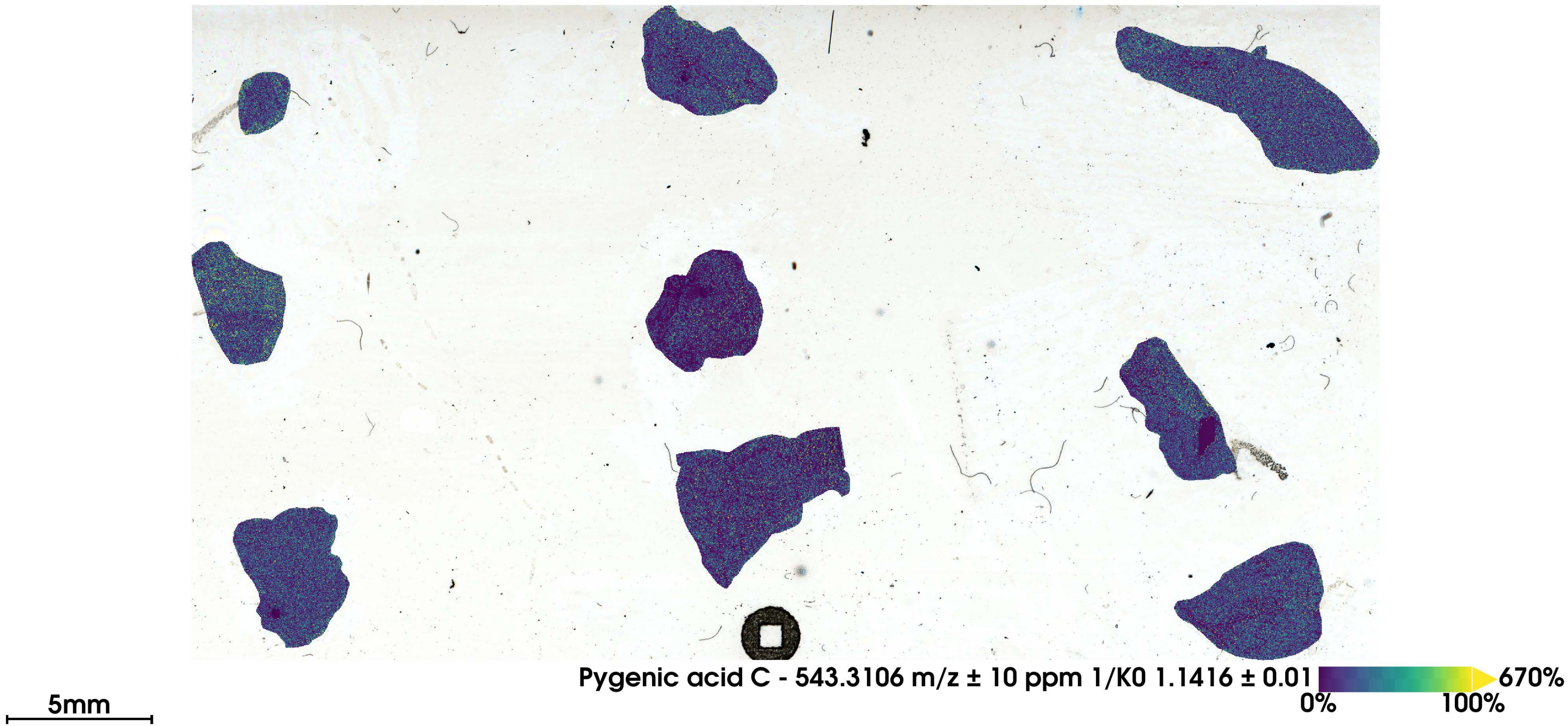
Pygenic acid C - 543.3106 m/z ± 10 ppm 1/K0 1.1416 ± 0.01
670%
0%
100%
5mm

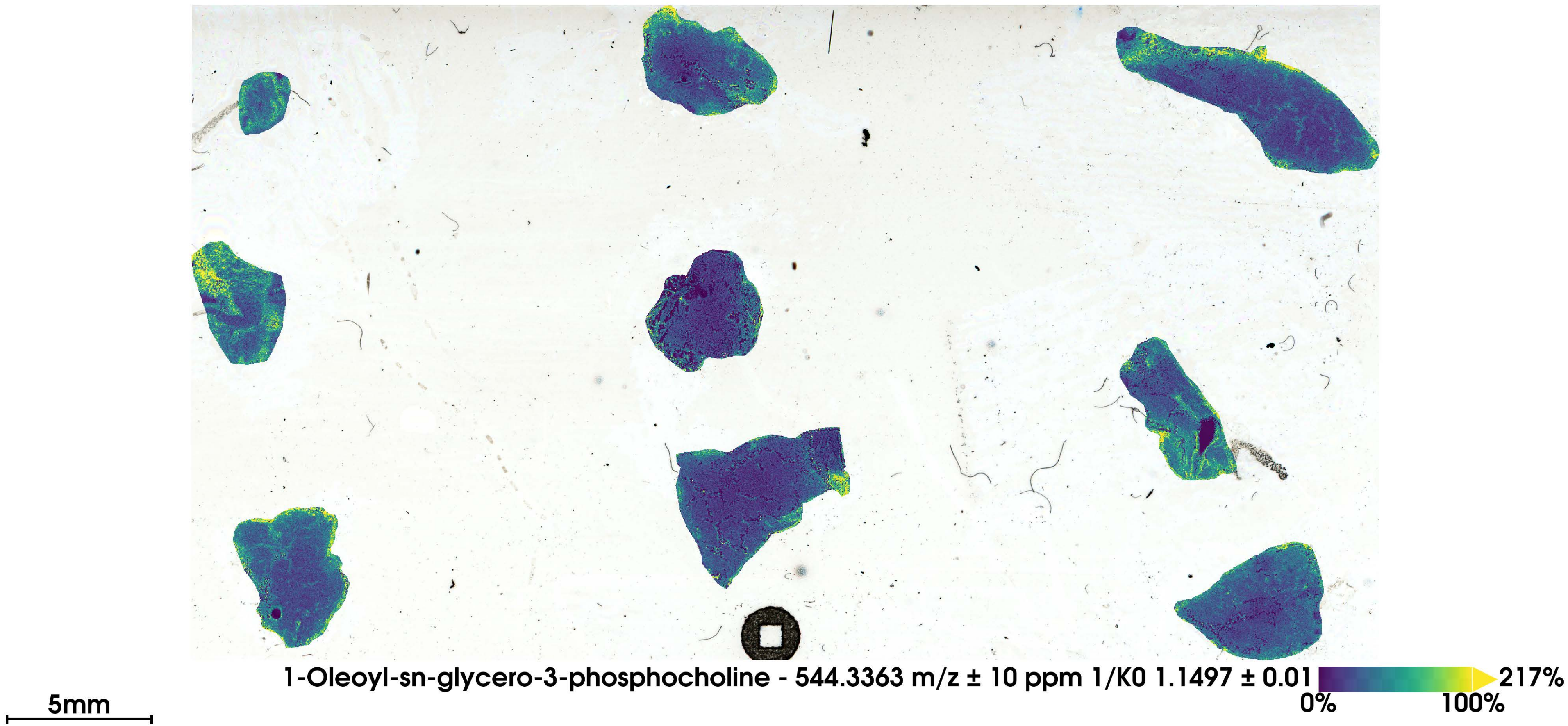
1-Oleoyl-sn-glycero-3-phosphocholine - 544.3363 m/z ± 10 ppm 1/K0 1.1497 ± 0.01
217%
0%
100%
5mm

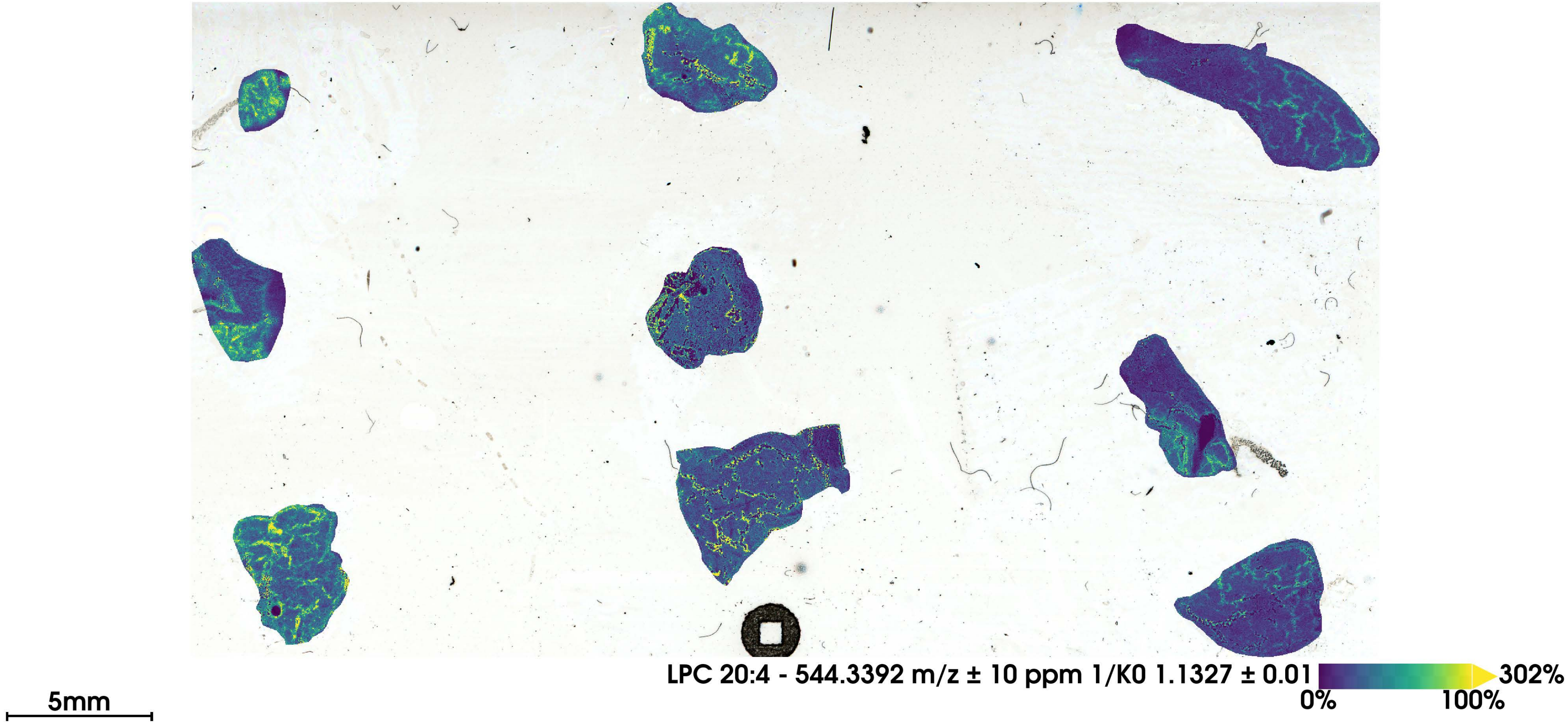
LPC 20:4 - 544.3392 m/z ± 10 ppm 1/K0 1.1327 ± 0.01
302%
0%
100%
5mm

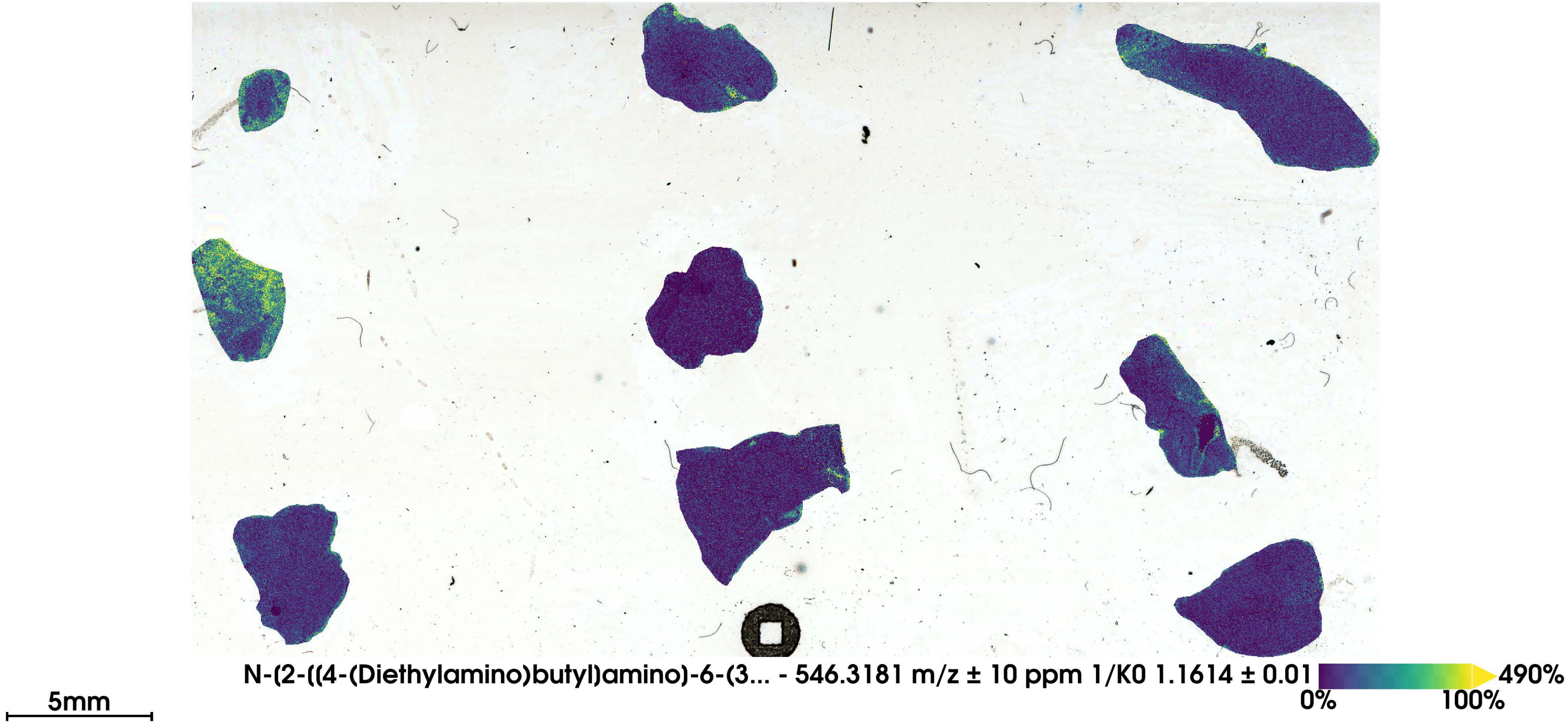
N-(2-((4-(Diethylamino)butyl)amino)-6-(3... - 546.3181 m/z ± 10 ppm 1/K0 1.1614 ± 0.01
490%
0%
100%
5mm

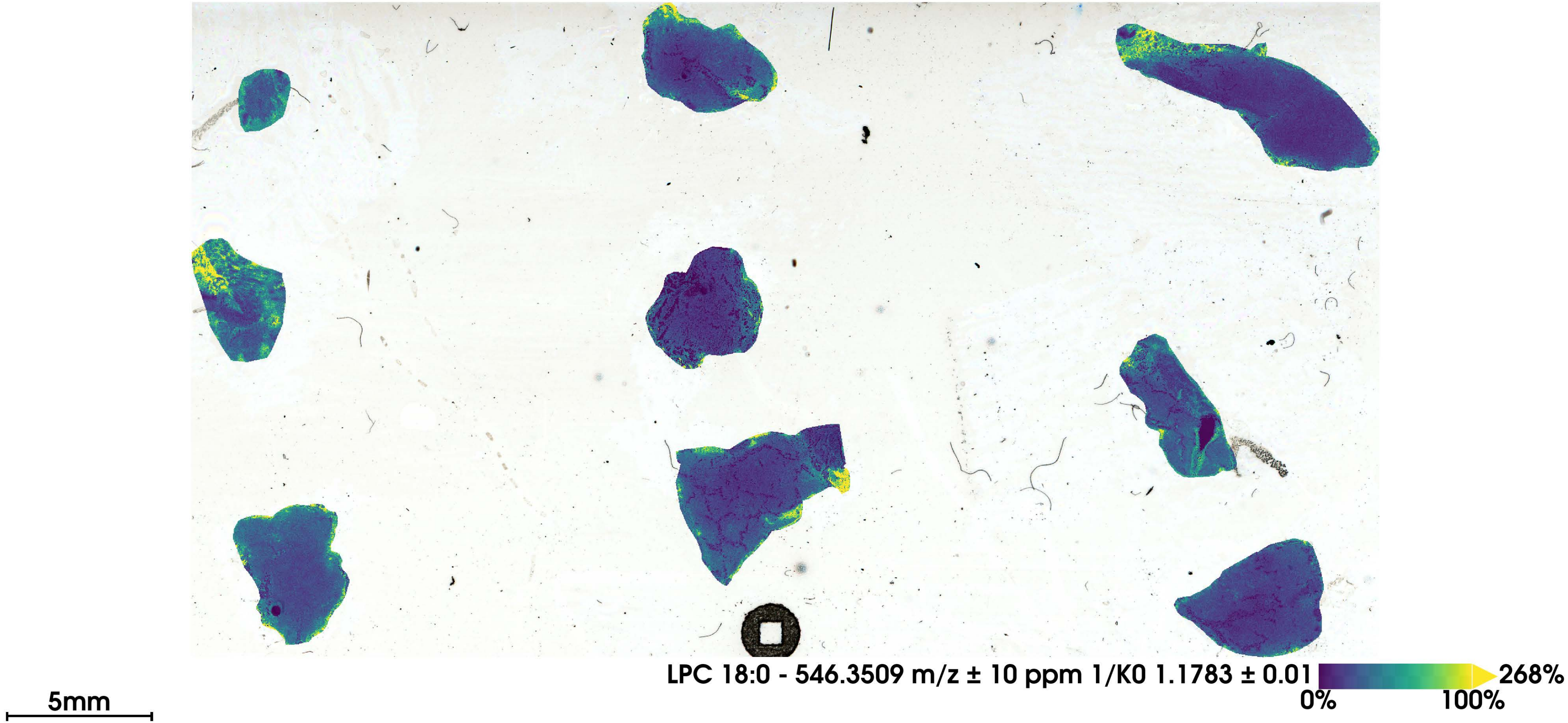
LPC 18:0 - 546.3509 m/z ± 10 ppm 1/K0 1.1783 ± 0.01
268%
0%
100%
5mm

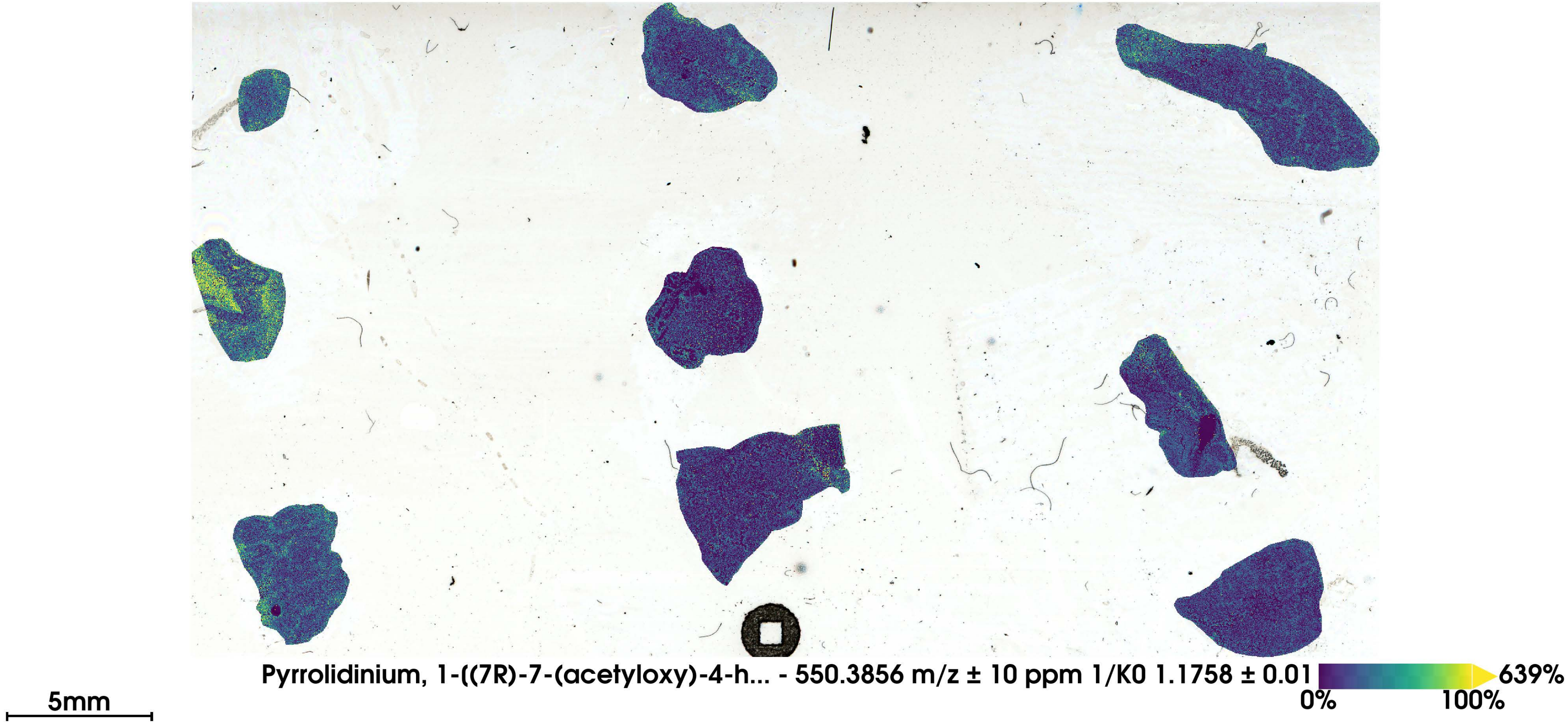

Pyrrolidinium, 1-((7R)-7-(acetyloxy)-4-h... - 550.3856 m/z ± 10 ppm 1/K0 1.1758 ± 0.01
639%
0%
100%
5mm

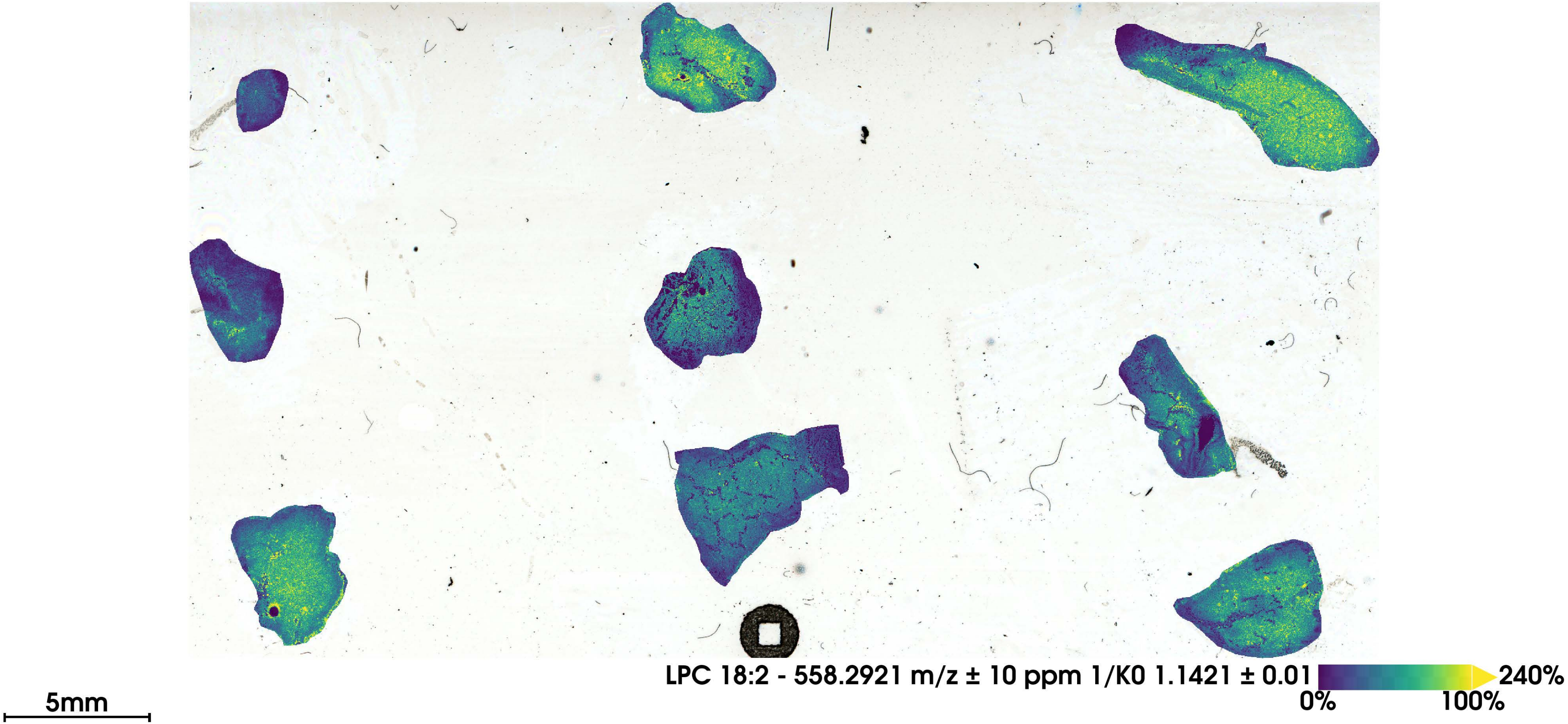

LPC 18:2 - 558.2921 m/z ± 10 ppm 1/K0 1.1421 ± 0.01
240%
0%
100%
5mm

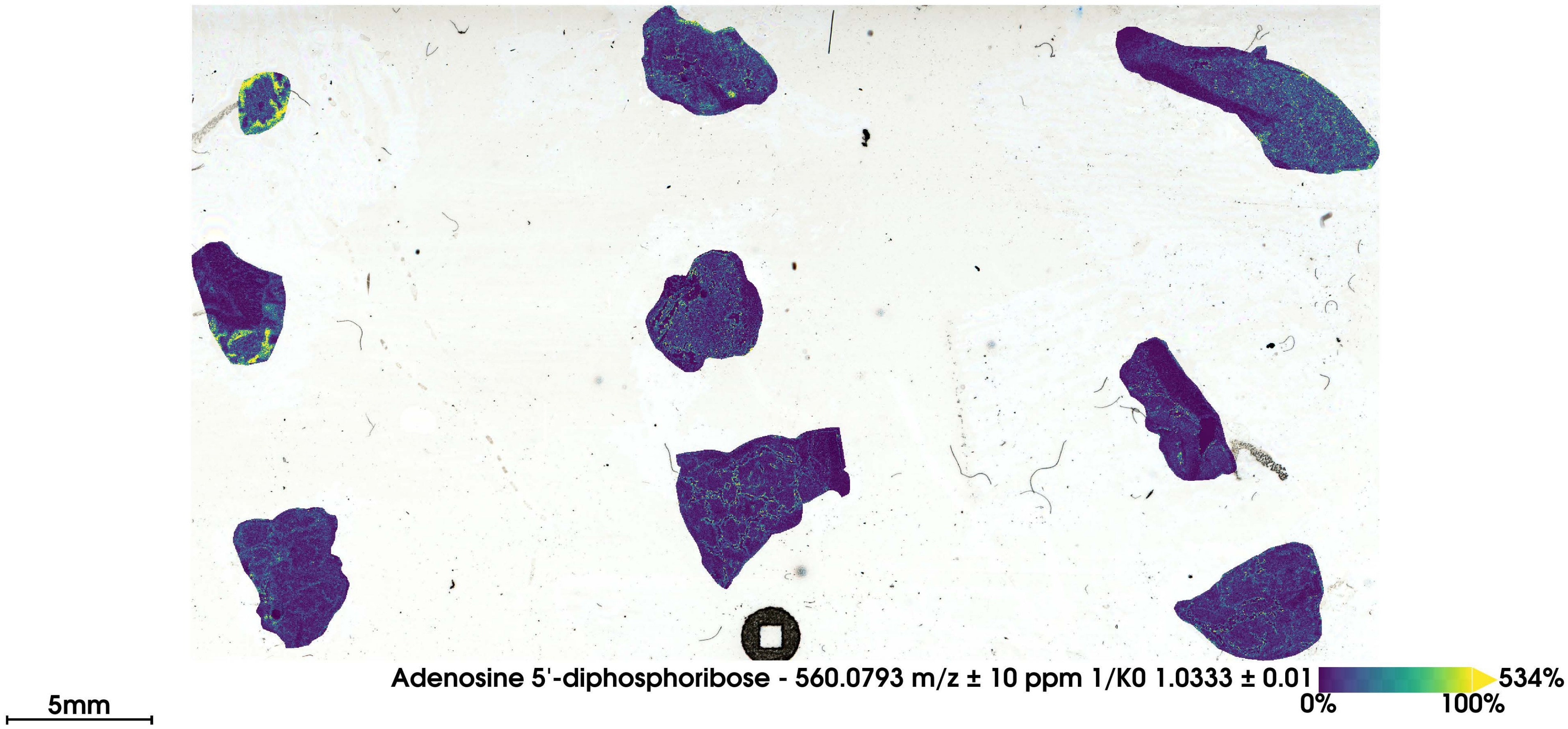
Adenosine 5'-diphosphoribose - 560.0793 m/z ± 10 ppm 1/K0 1.0333 ± 0.01
534%
0%
100%
5mm

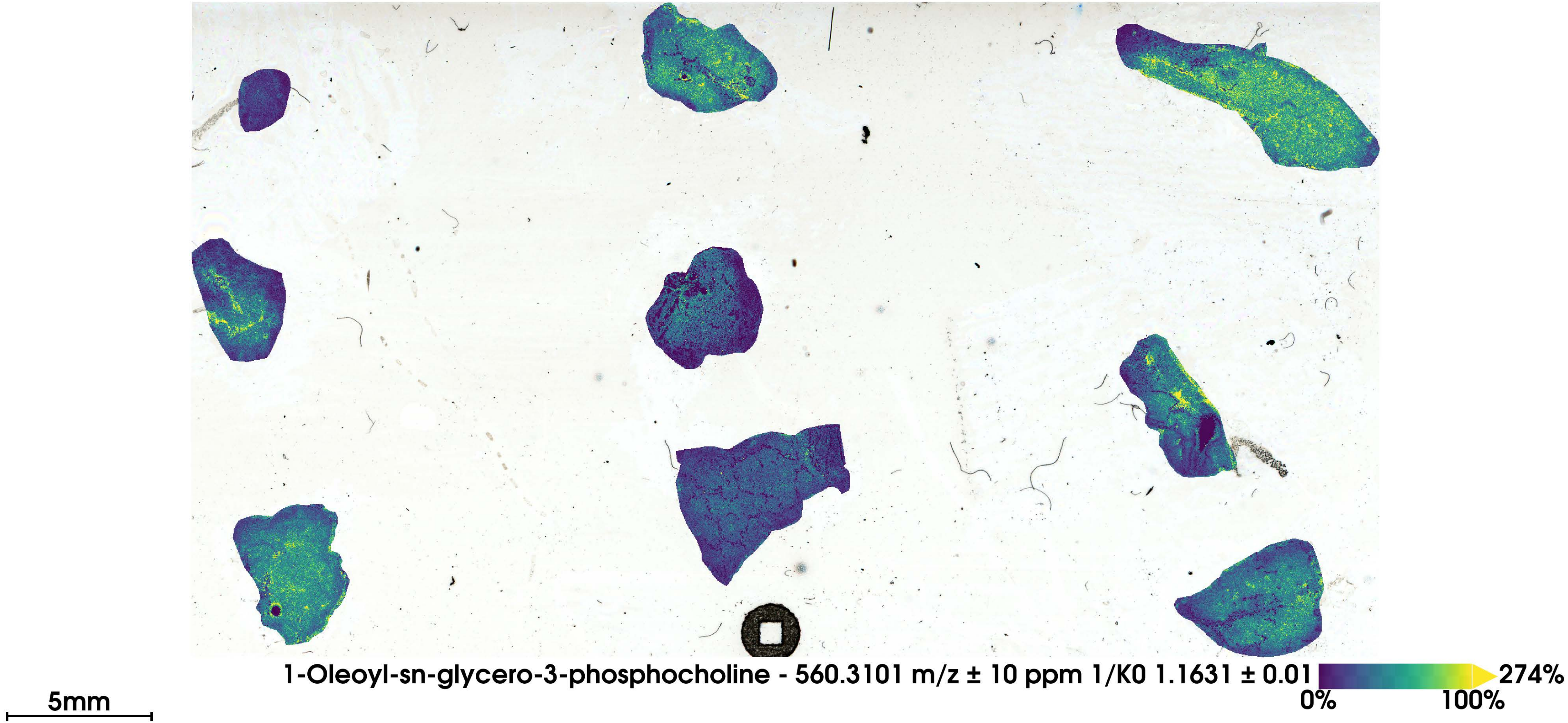
1-Oleoyl-sn-glycero-3-phosphocholine - 560.3101 m/z ± 10 ppm 1/K0 1.1631 ± 0.01
274%
0%
100%
5mm

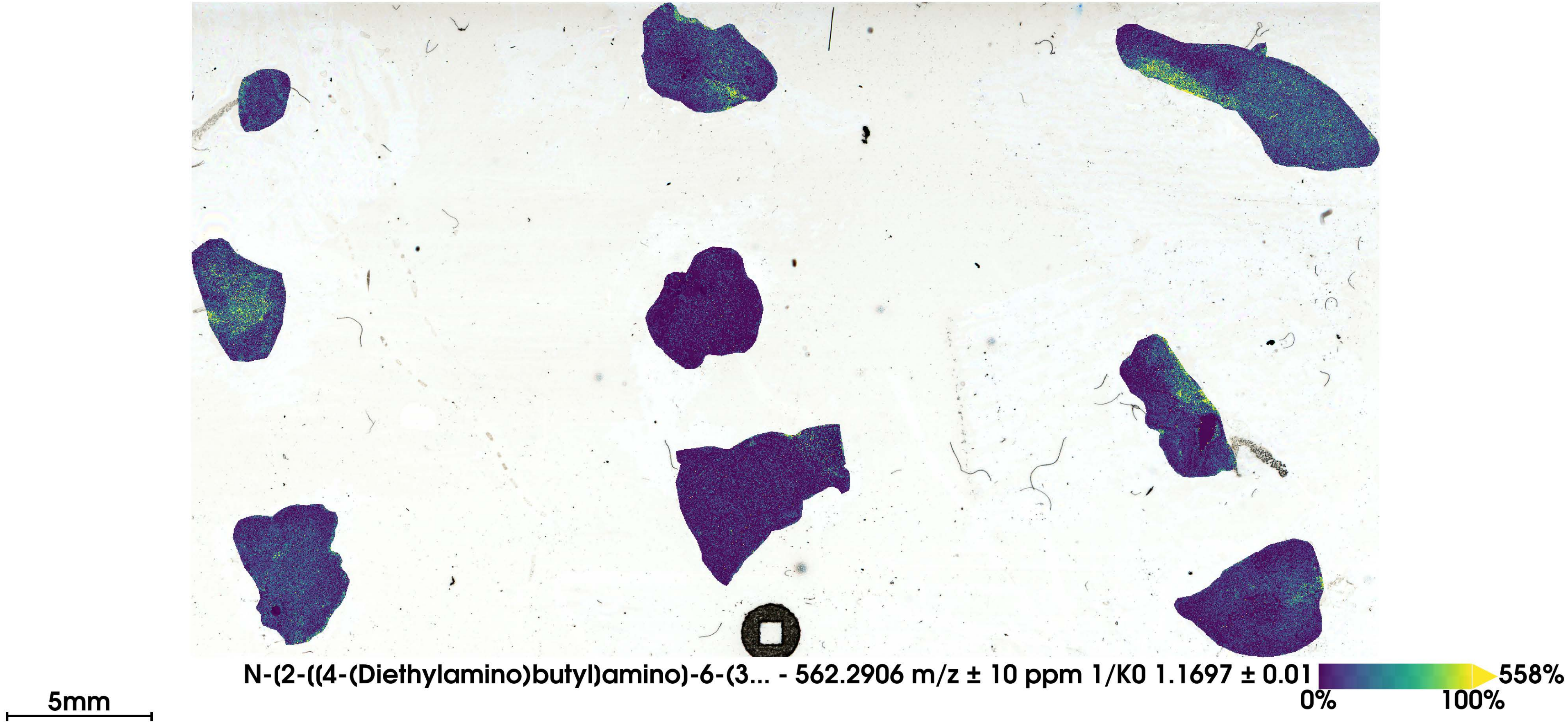
N-(2-((4-(Diethylamino)butyl)amino)-6-(3... - 562.2906 m/z ± 10 ppm 1/K0 1.1697 ± 0.01
558%
0%
100%
5mm

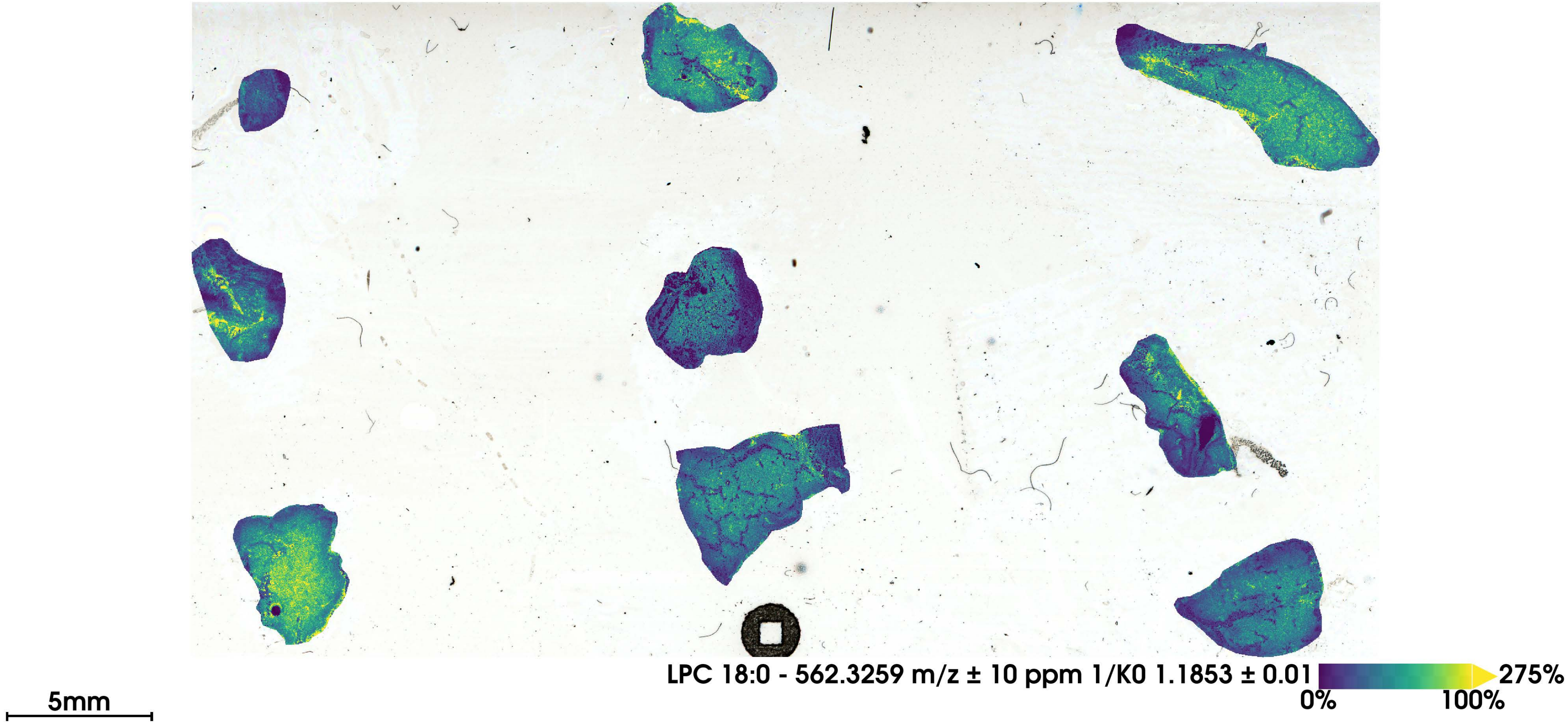

LPC 18:0 - 562.3259 m/z ± 10 ppm 1/K0 1.1853 ± 0.01
275%
0%
100%
5mm

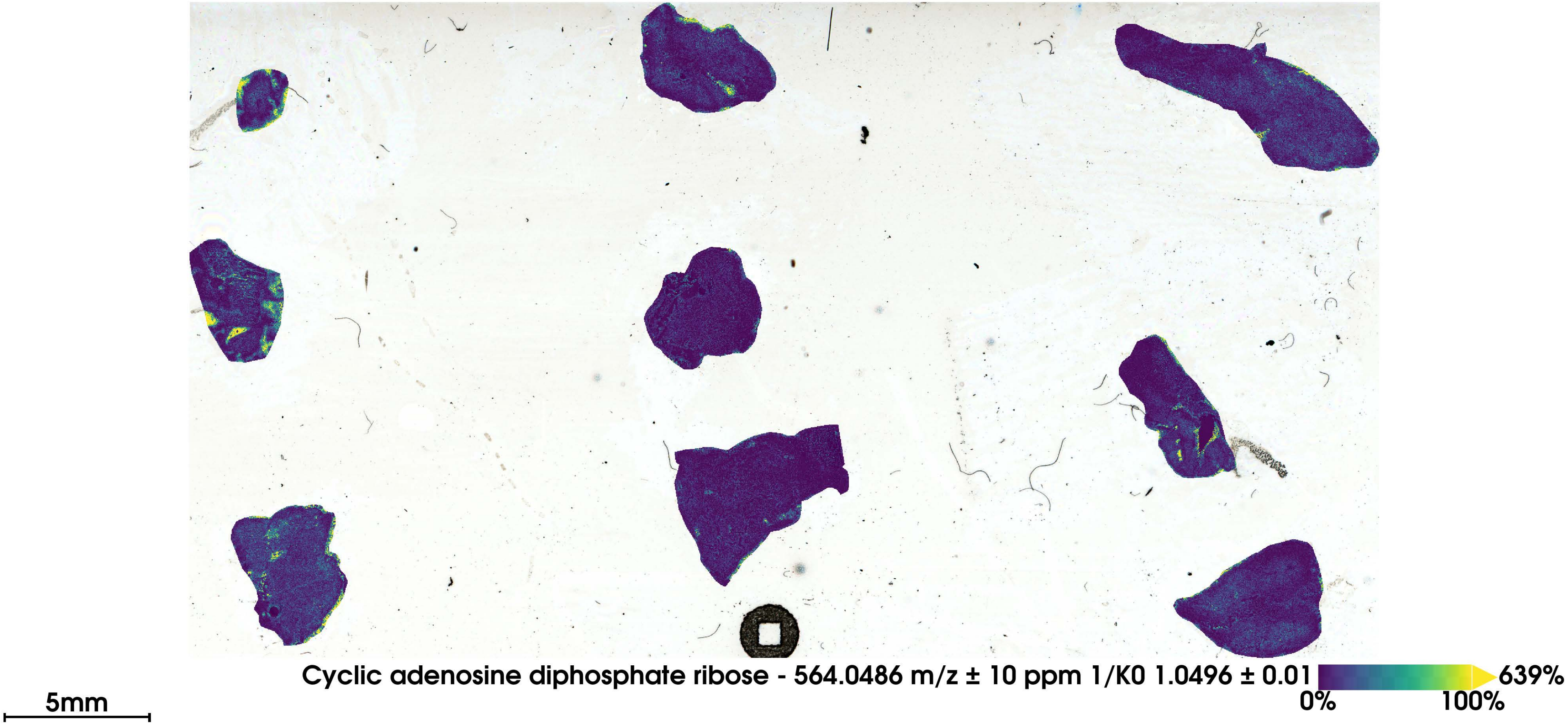
Cyclic adenosine diphosphate ribose - 564.0486 m/z ± 10 ppm 1/K0 1.0496 ± 0.01
639%
0%
100%
5mm

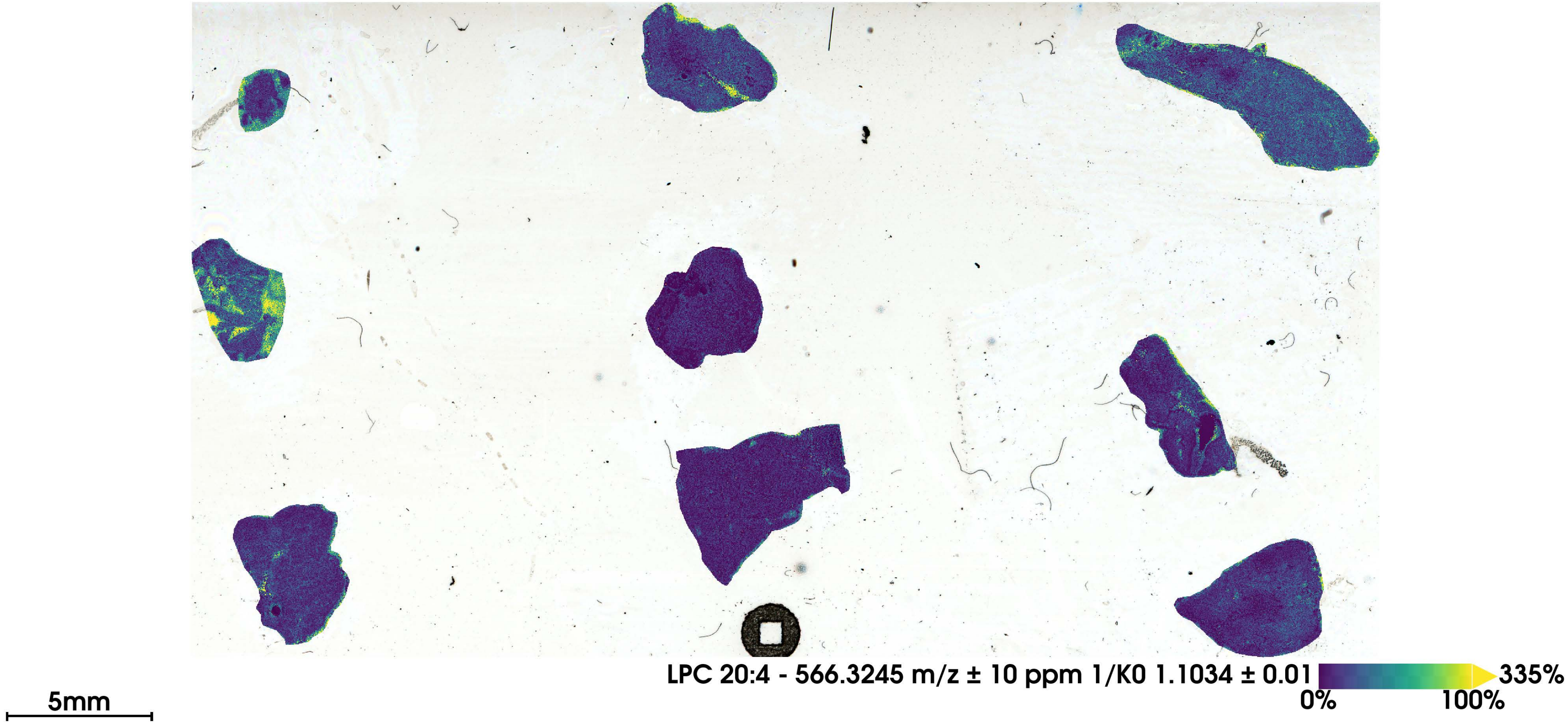

LPC 20:4 - 566.3245 m/z ± 10 ppm 1/K0 1.1034 ± 0.01
335%
0%
100%
5mm

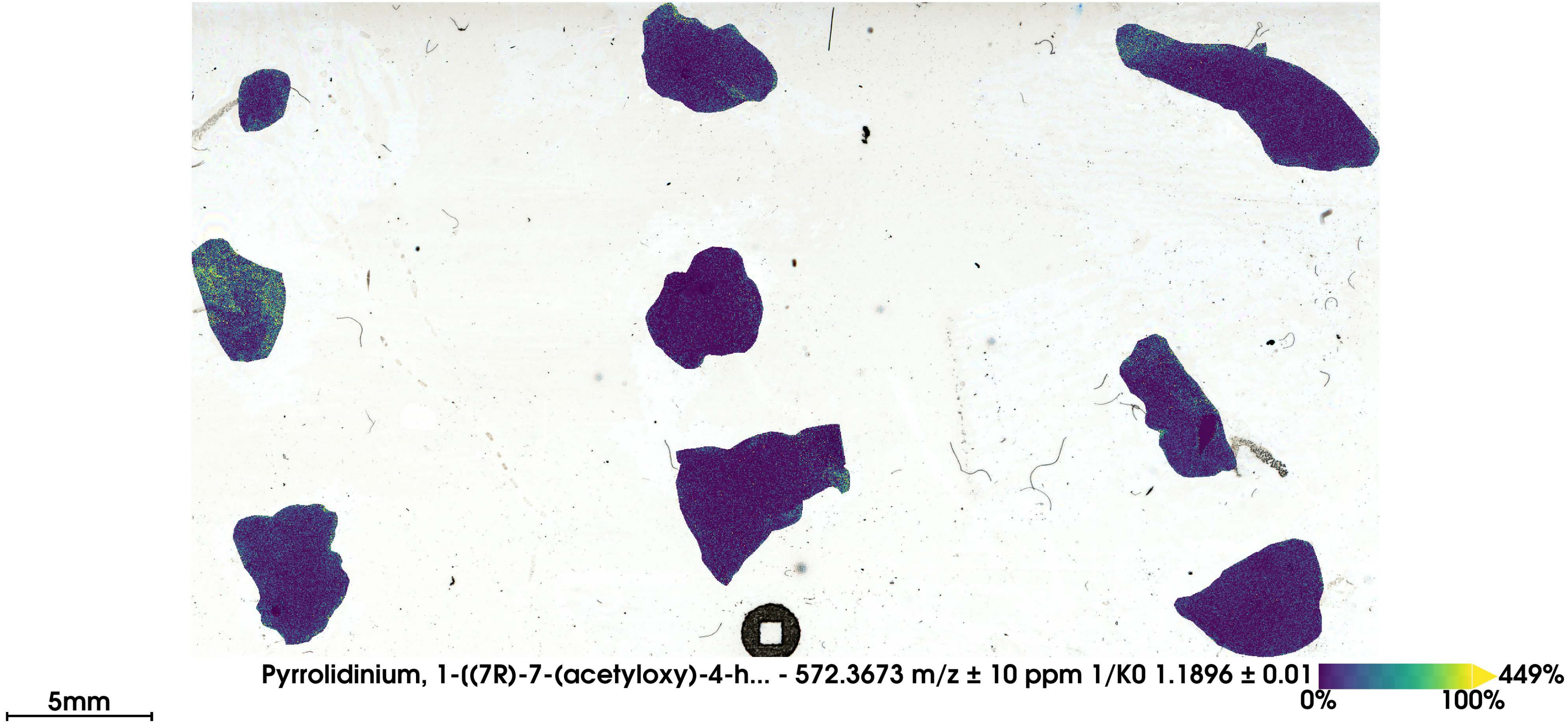

Pyrrolidinium, 1-((7R)-7-(acetyloxy)-4-h... - 572.3673 m/z ± 10 ppm 1/K0 1.1896 ± 0.01
449%
0%
100%
5mm

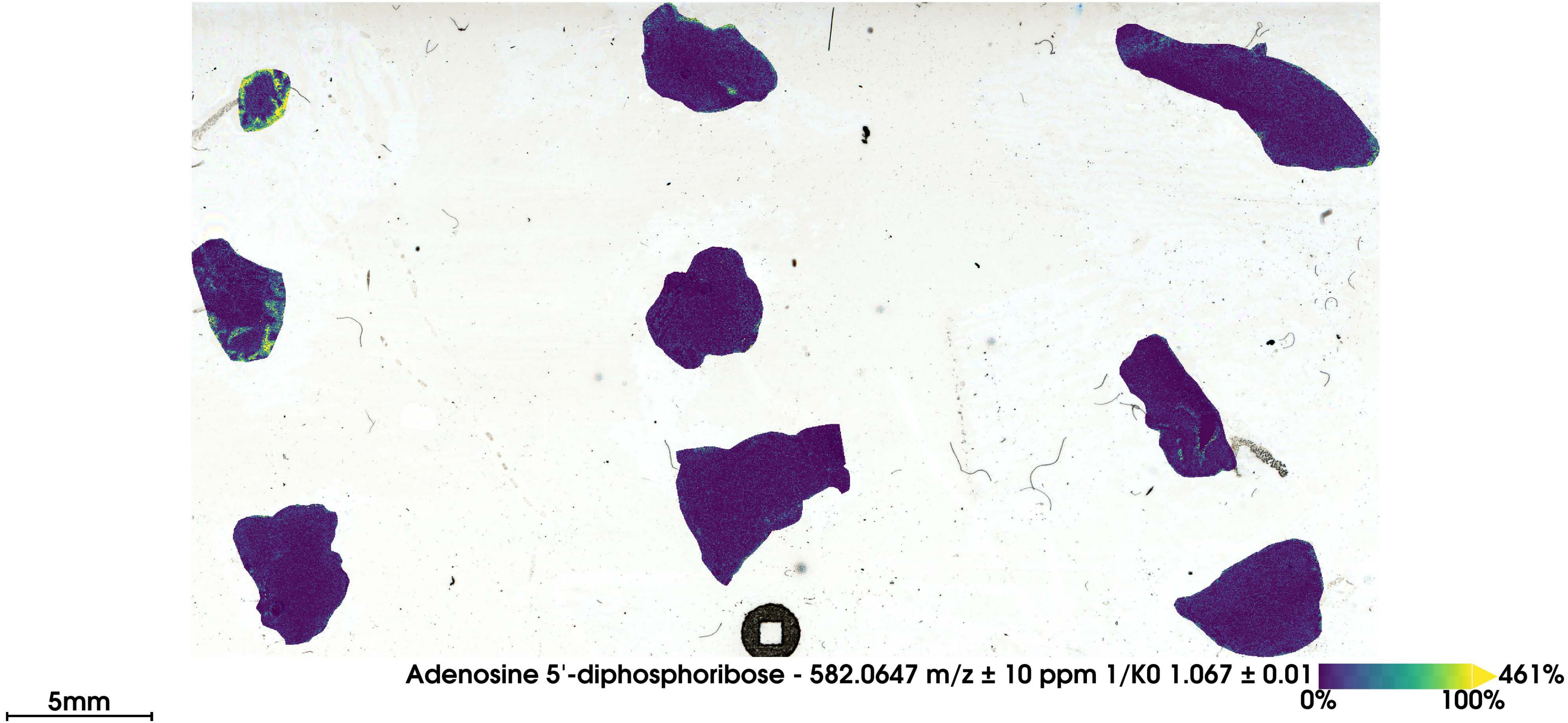

Adenosine 5'-diphosphoribose - 582.0647 m/z ± 10 ppm 1/K0 1.067 ± 0.01
461%
0%
100%
5mm

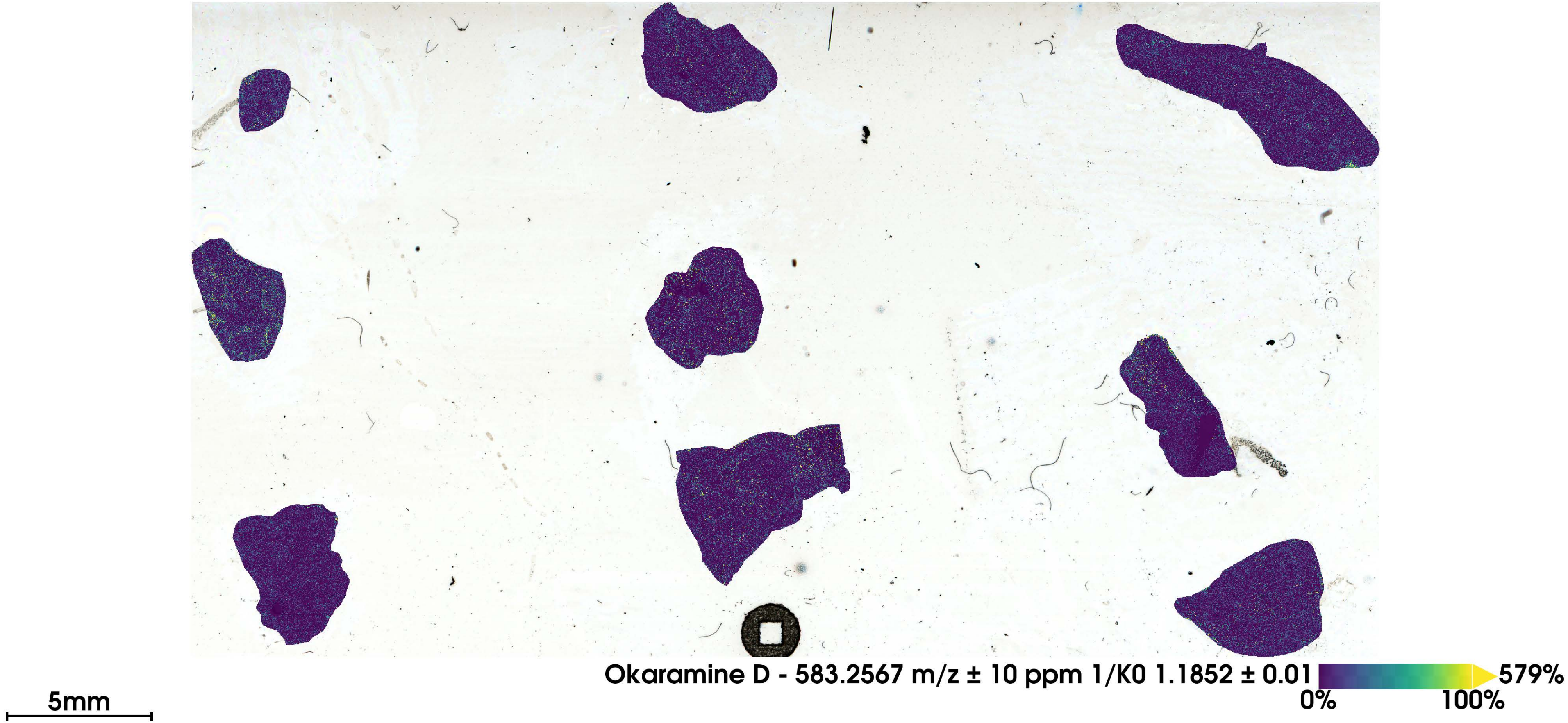

Okaramine D - 583.2567 m/z ± 10 ppm 1/K0 1.1852 ± 0.01
579%
0%
100%
5mm

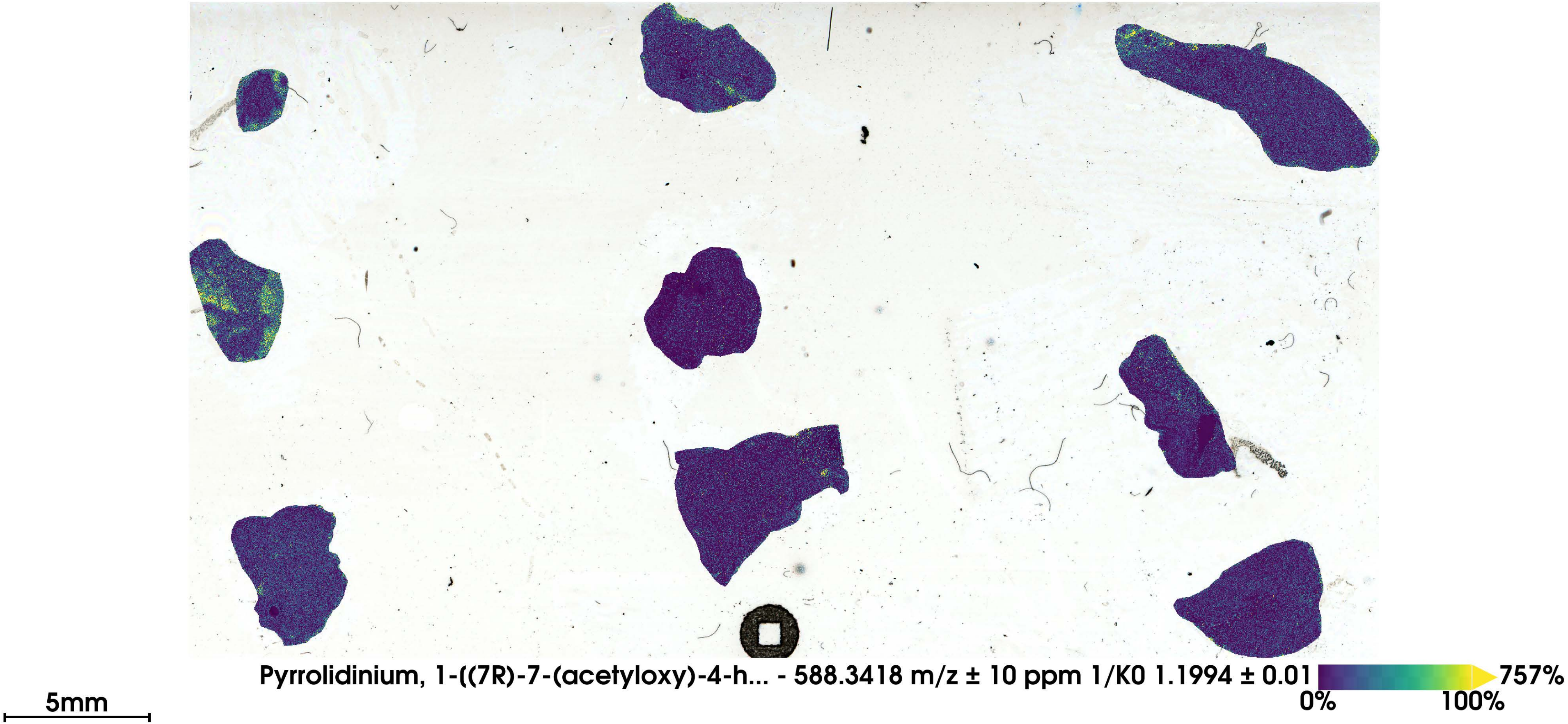

Pyrrolidinium, 1-((7R)-7-(acetyloxy)-4-h... - 588.3418 m/z ± 10 ppm 1/K0 1.1994 ± 0.01
757%
0%
100%
5mm

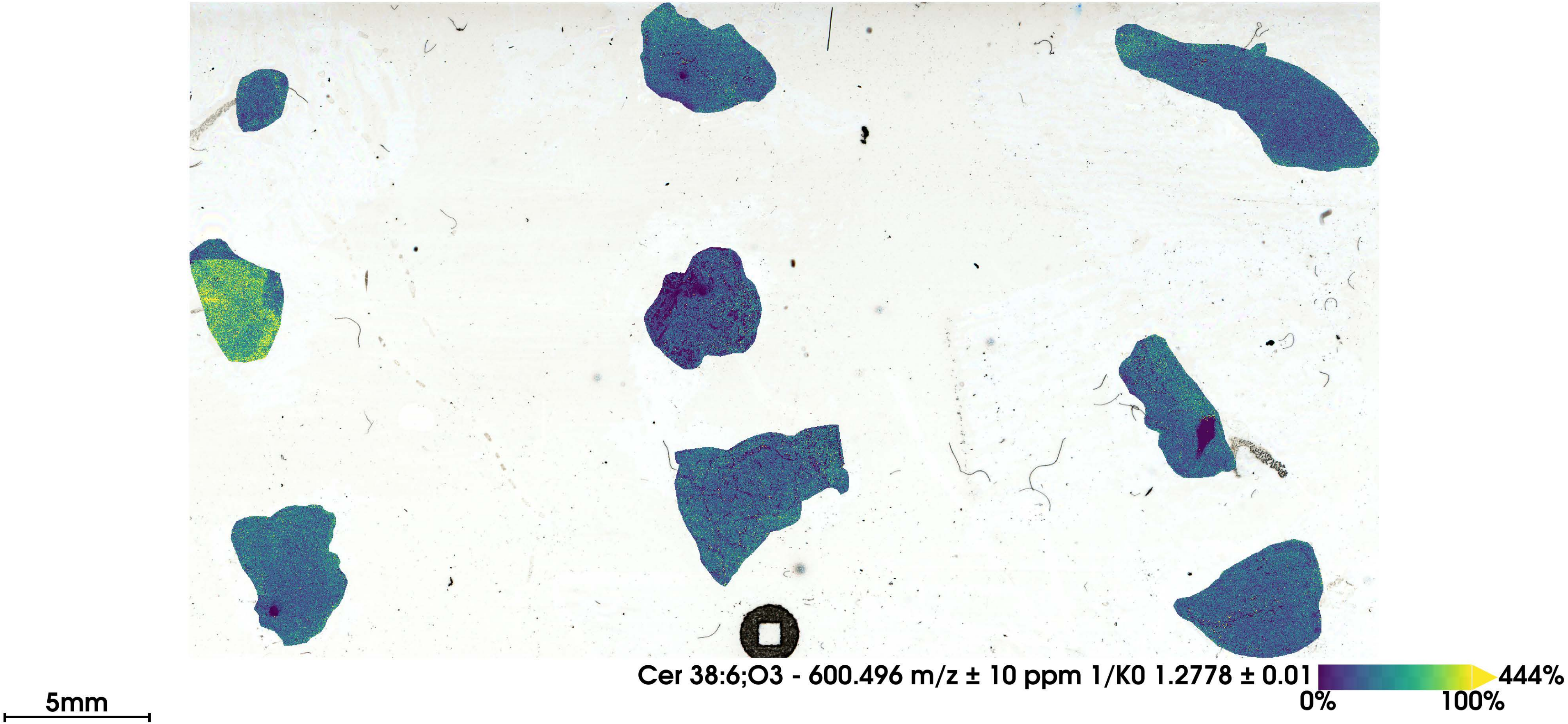
Cer 38:6;O3 - 600.496 m/z ± 10 ppm 1/K0 1.2778 ± 0.01
444%
0%
100%
5mm

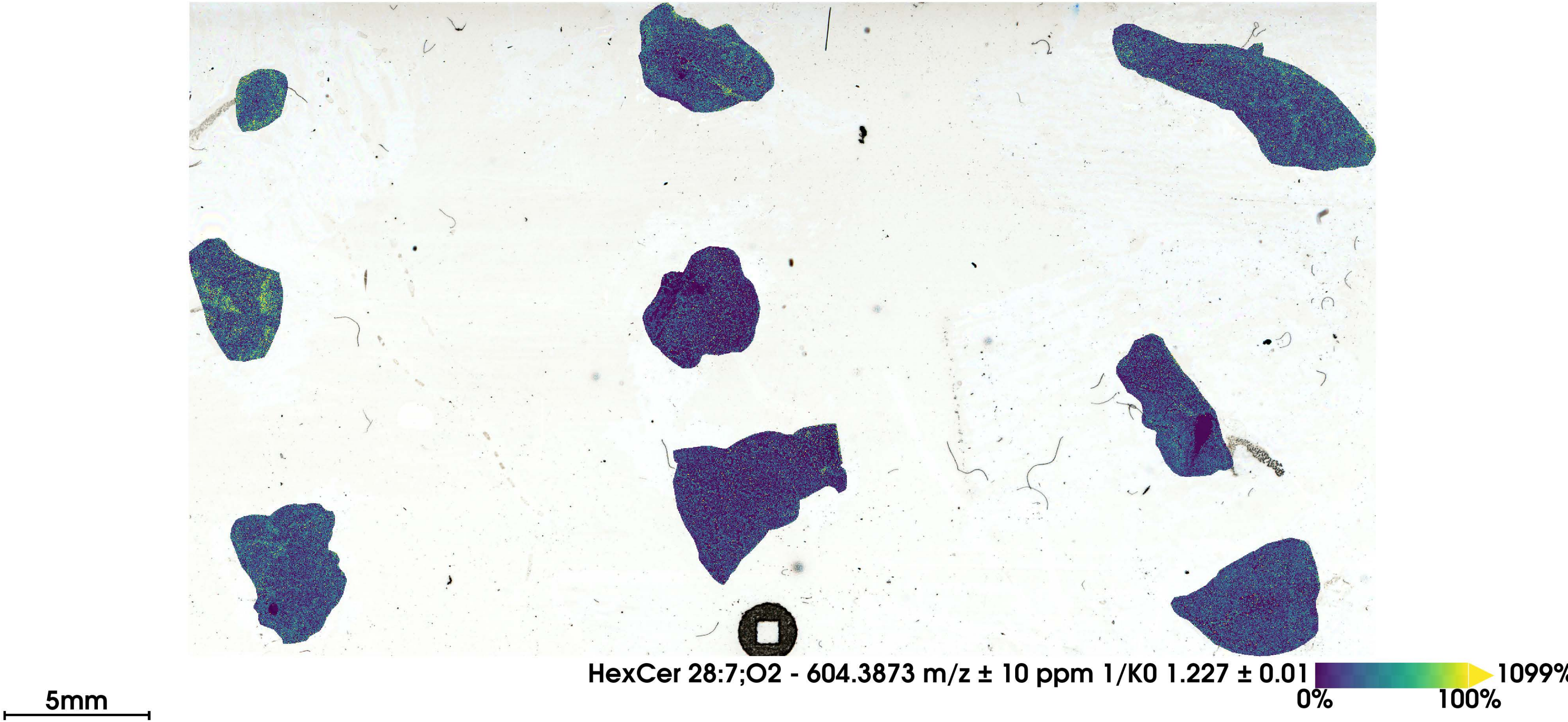
HexCer 28:7;O2 - 604.3873 m/z ± 10 ppm 1/K0 1.227 ± 0.01
1099%
0%
100%
5mm

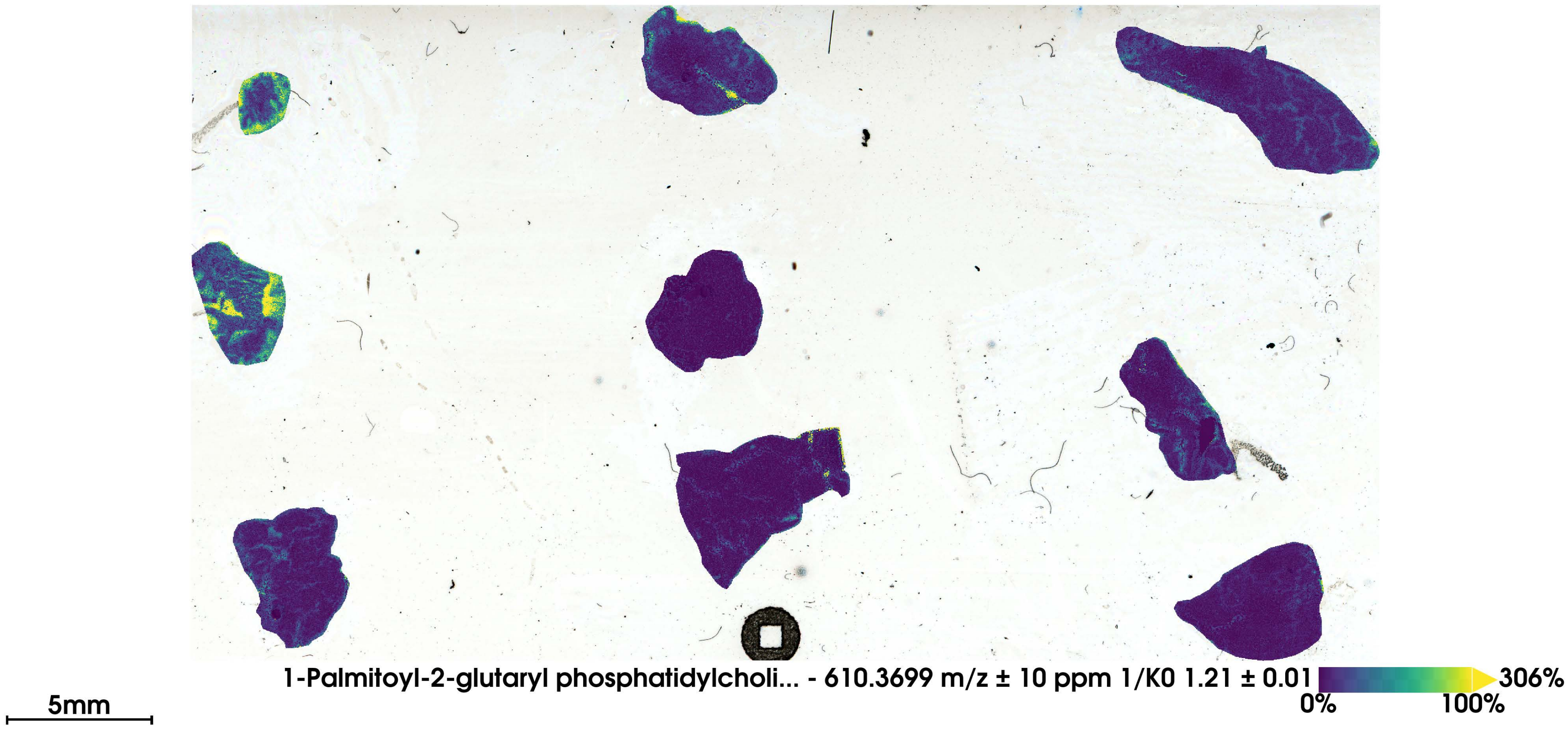
1-Palmitoyl-2-glutaryl phosphatidylcholi... - 610.3699 m/z ± 10 ppm 1/K0 1.21 ± 0.01
306%
0%
100%
5mm

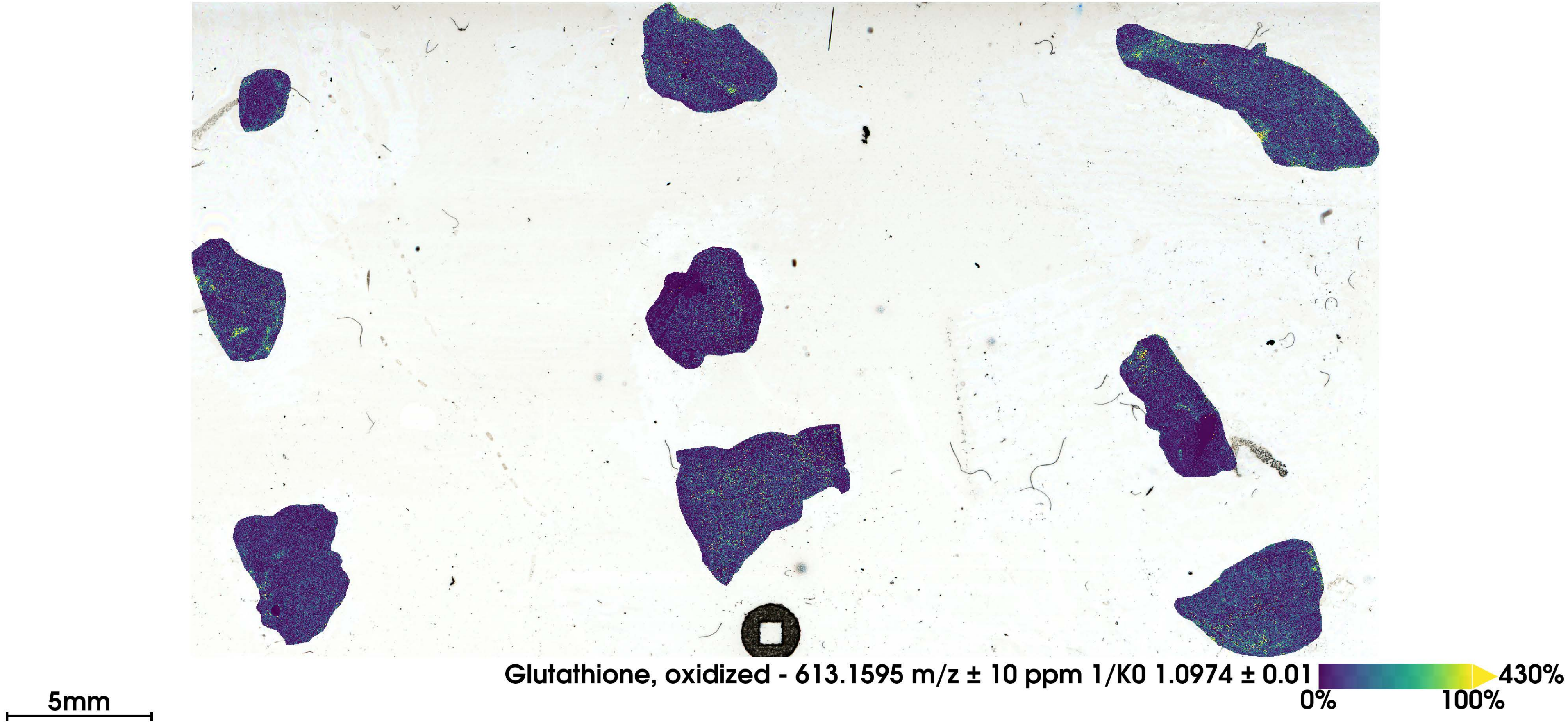
Glutathione, oxidized - 613.1595 m/z ± 10 ppm 1/K0 1.0974 ± 0.01
430%
0%
100%
5mm

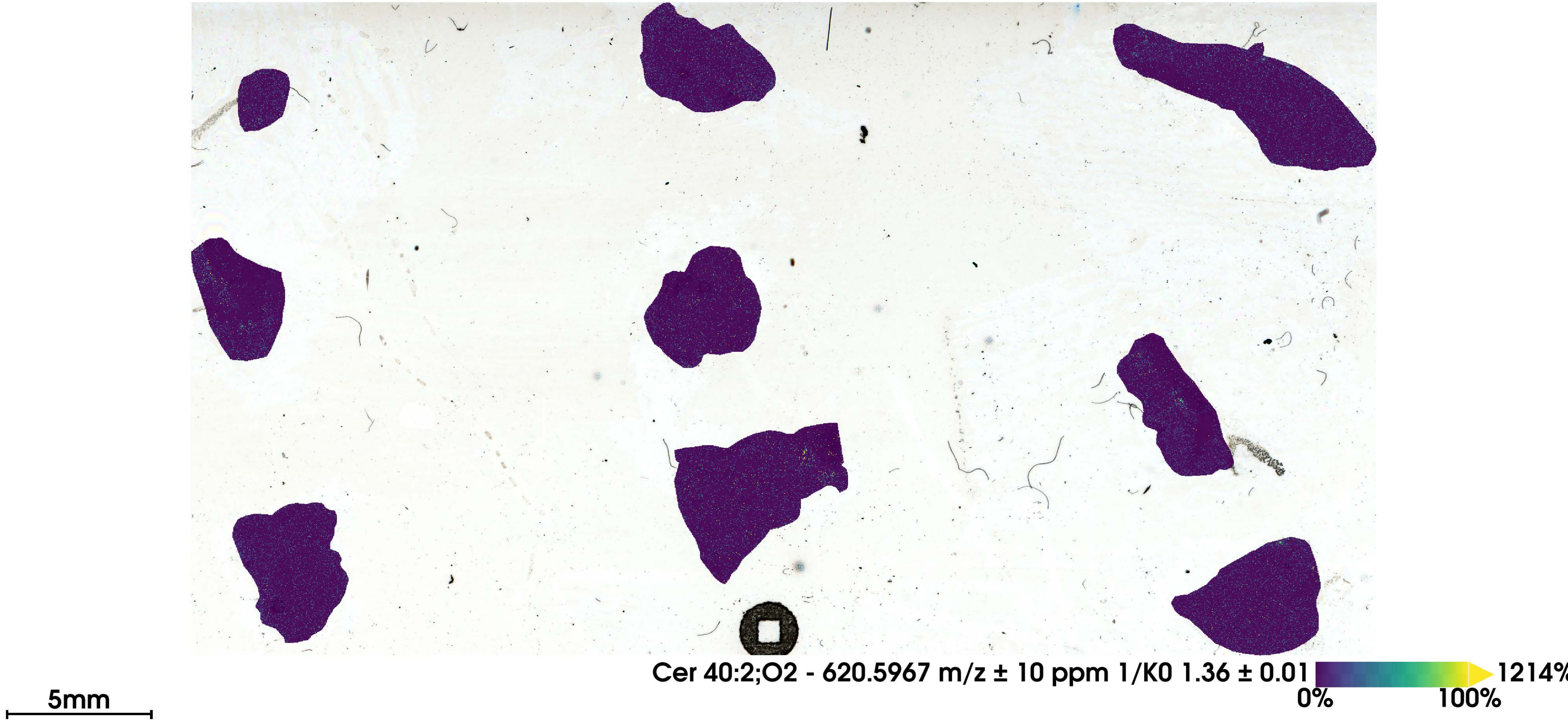

Cer 40:2;O2 - 620.5967 m/z ± 10 ppm 1/K0 1.36 ± 0.01
0%
100%
1214%
5mm

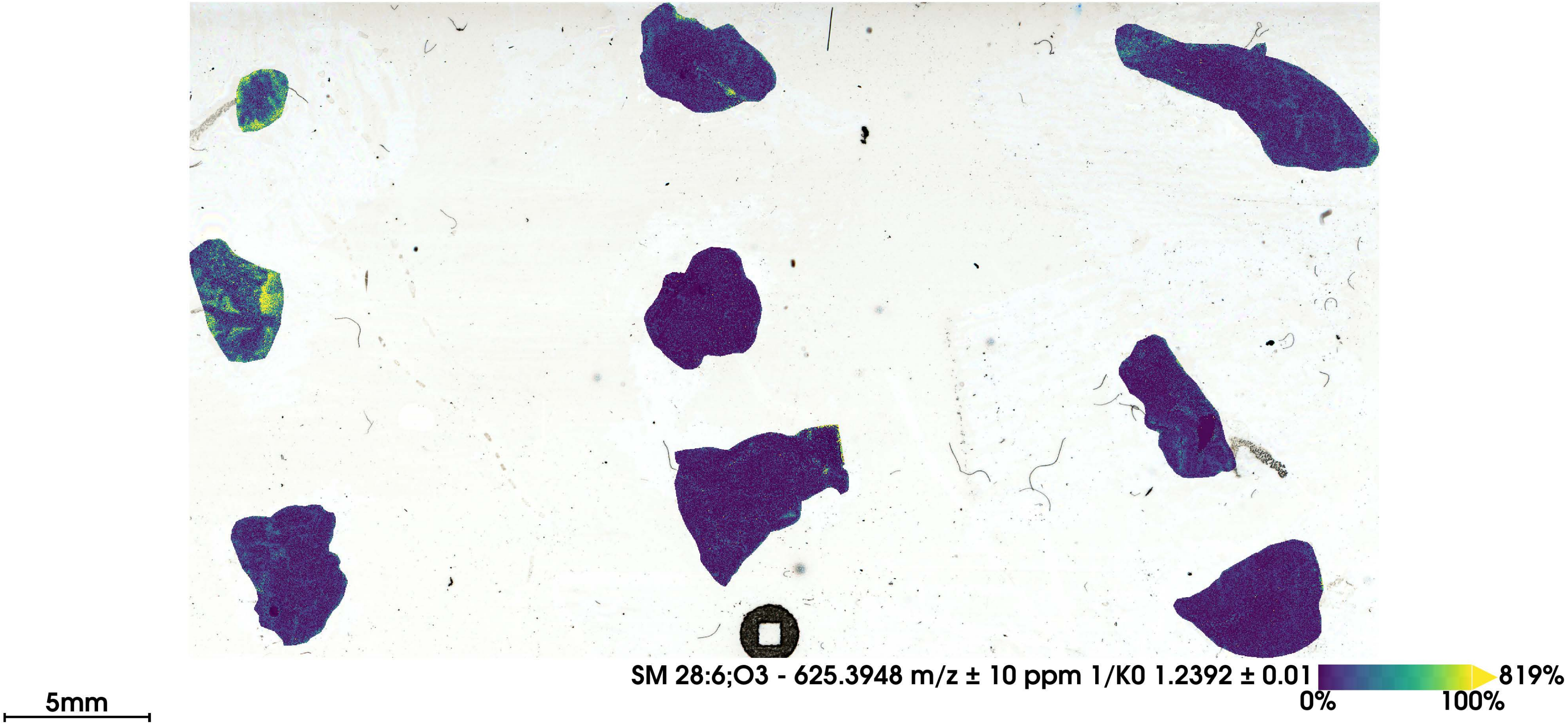
SM 28:6;O3 - 625.3948 m/z ± 10 ppm 1/K0 1.2392 ± 0.01
819%
0%
100%
5mm

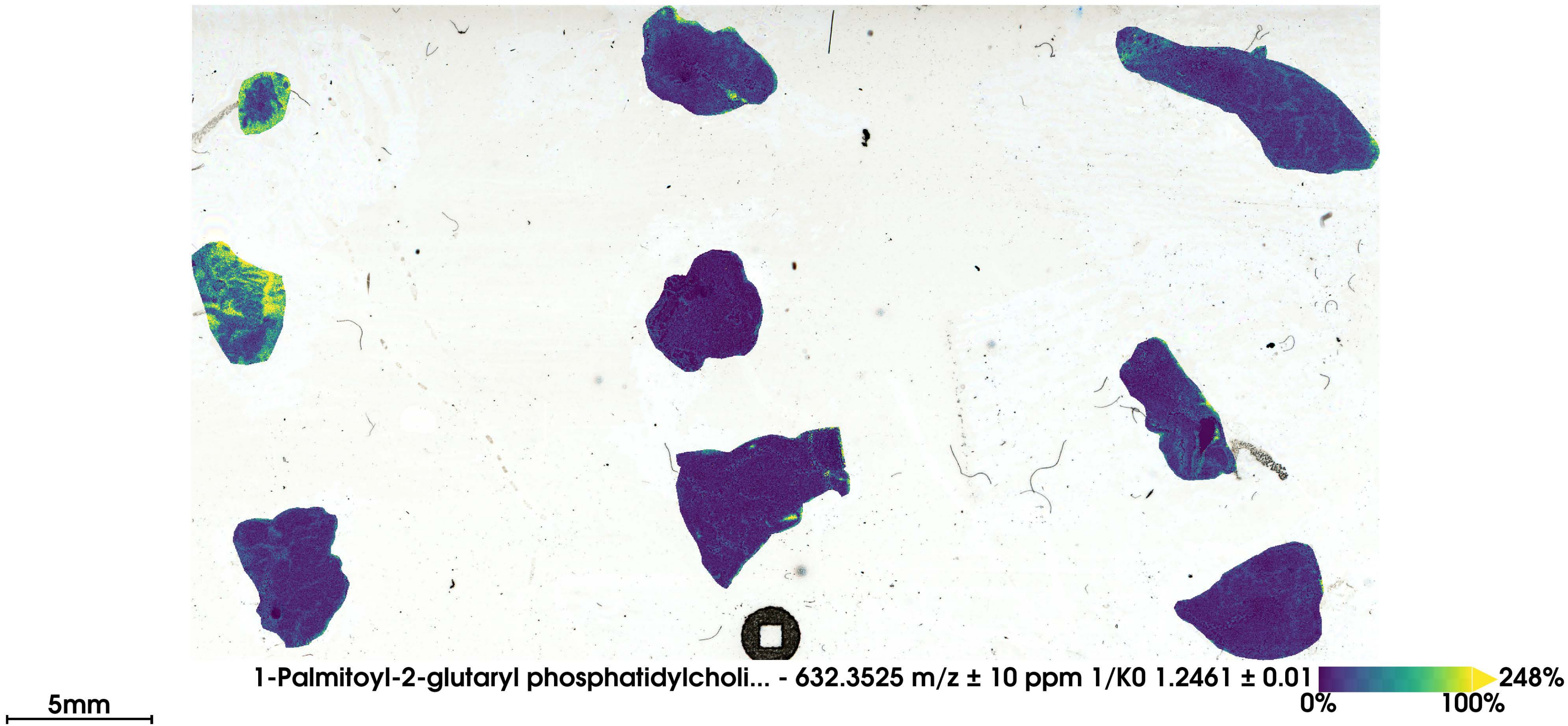
1-Palmitoyl-2-glutaryl phosphatidylcholi... - 632.3525 m/z ± 10 ppm 1/K0 1.2461 ± 0.01
248%
0%
100%
5mm

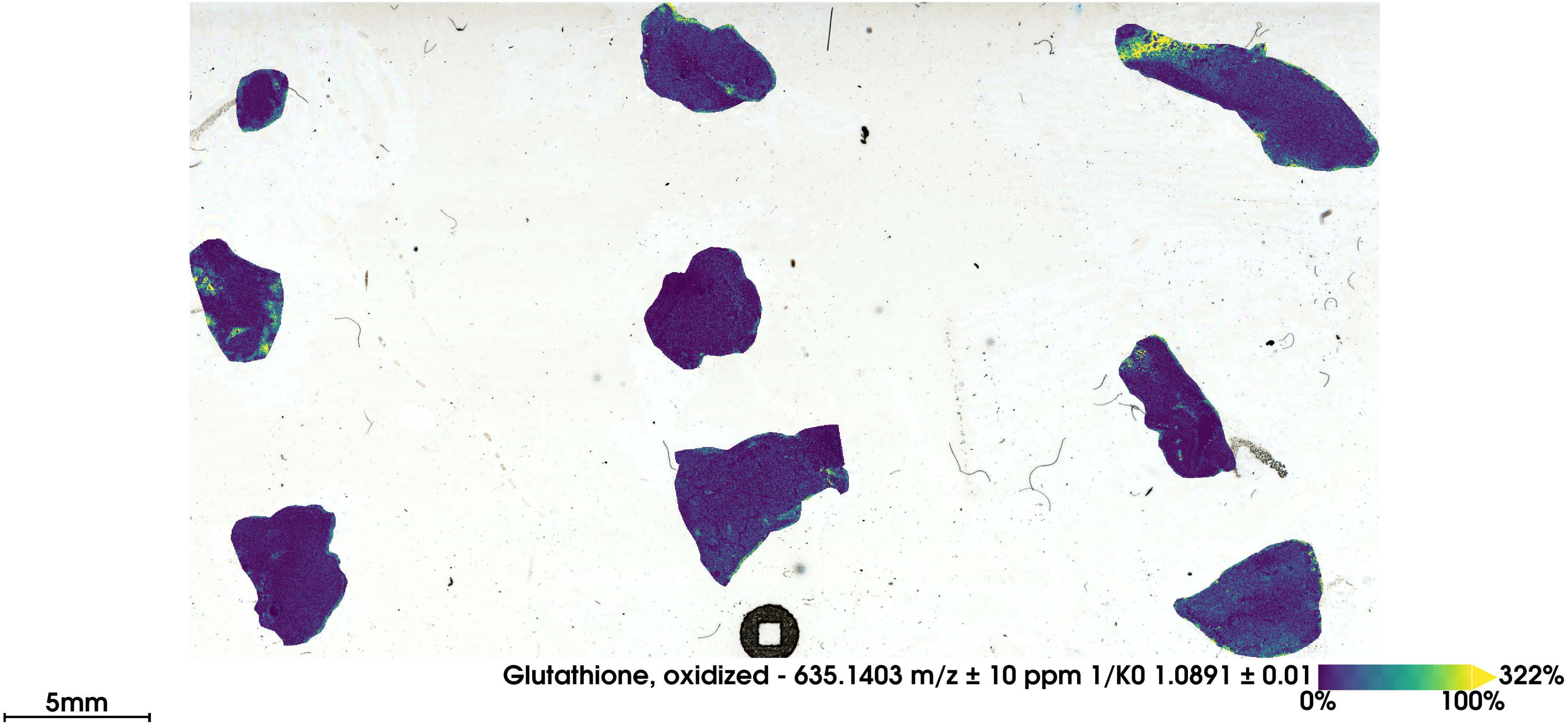

5mm
Glutathione, oxidized - 635.1403 m/z ± 10 ppm 1/K0 1.0891 ± 0.01
0%
100%
322%

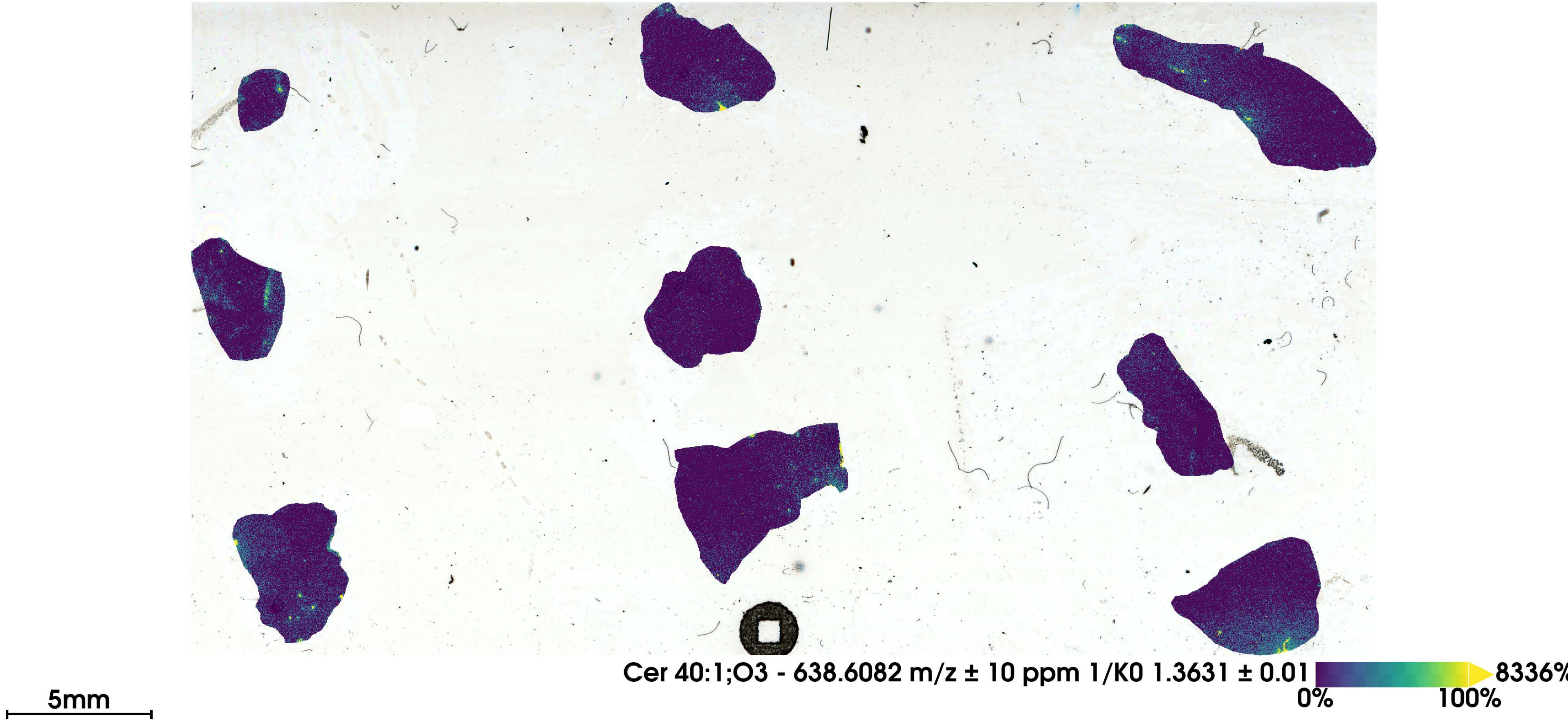

Cer 40:1;O3 - 638.6082 m/z ± 10 ppm 1/K0 1.3631 ± 0.01
8336%
0%
100%
5mm

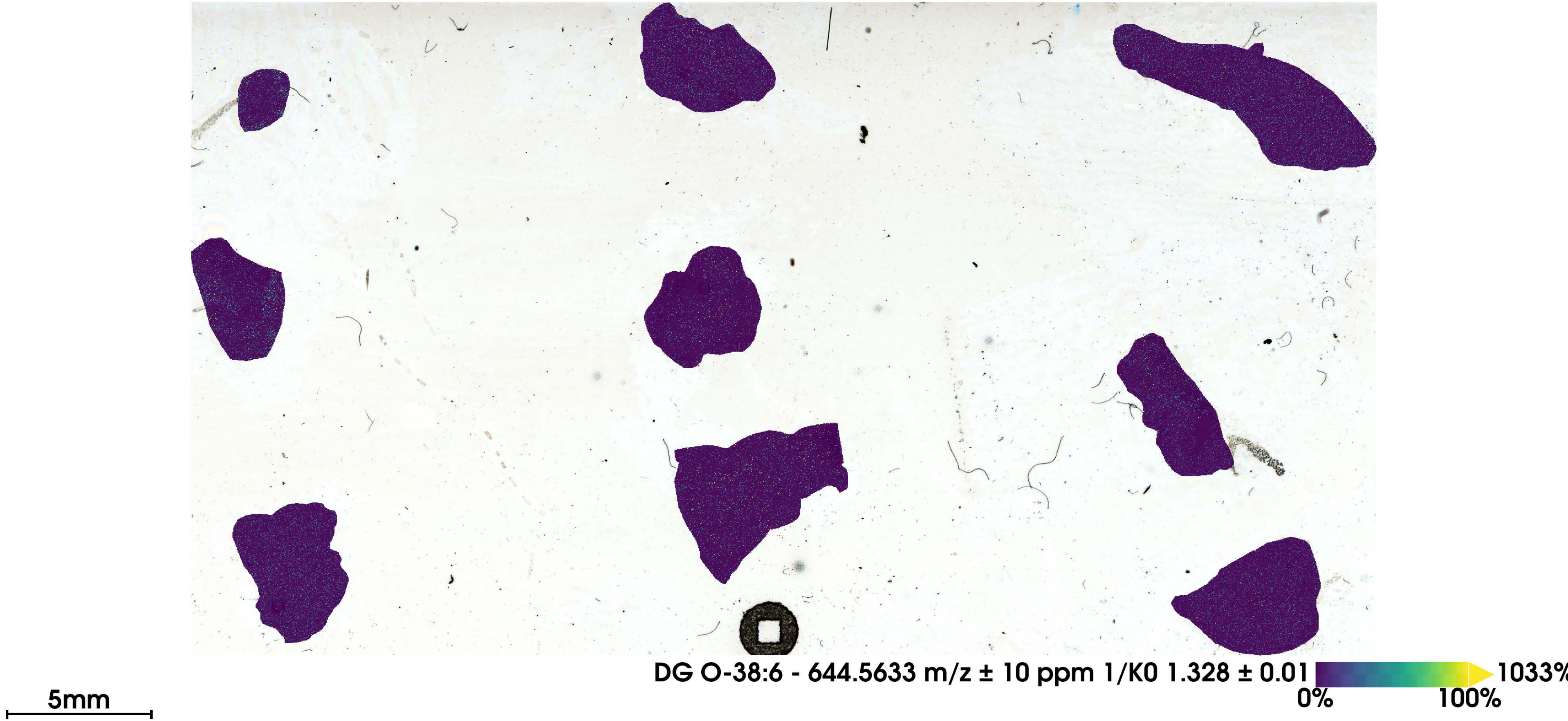
DG O-38:6 - 644.5633 m/z ± 10 ppm 1/K0 1.328 ± 0.01
1033%
0%
100%
5mm

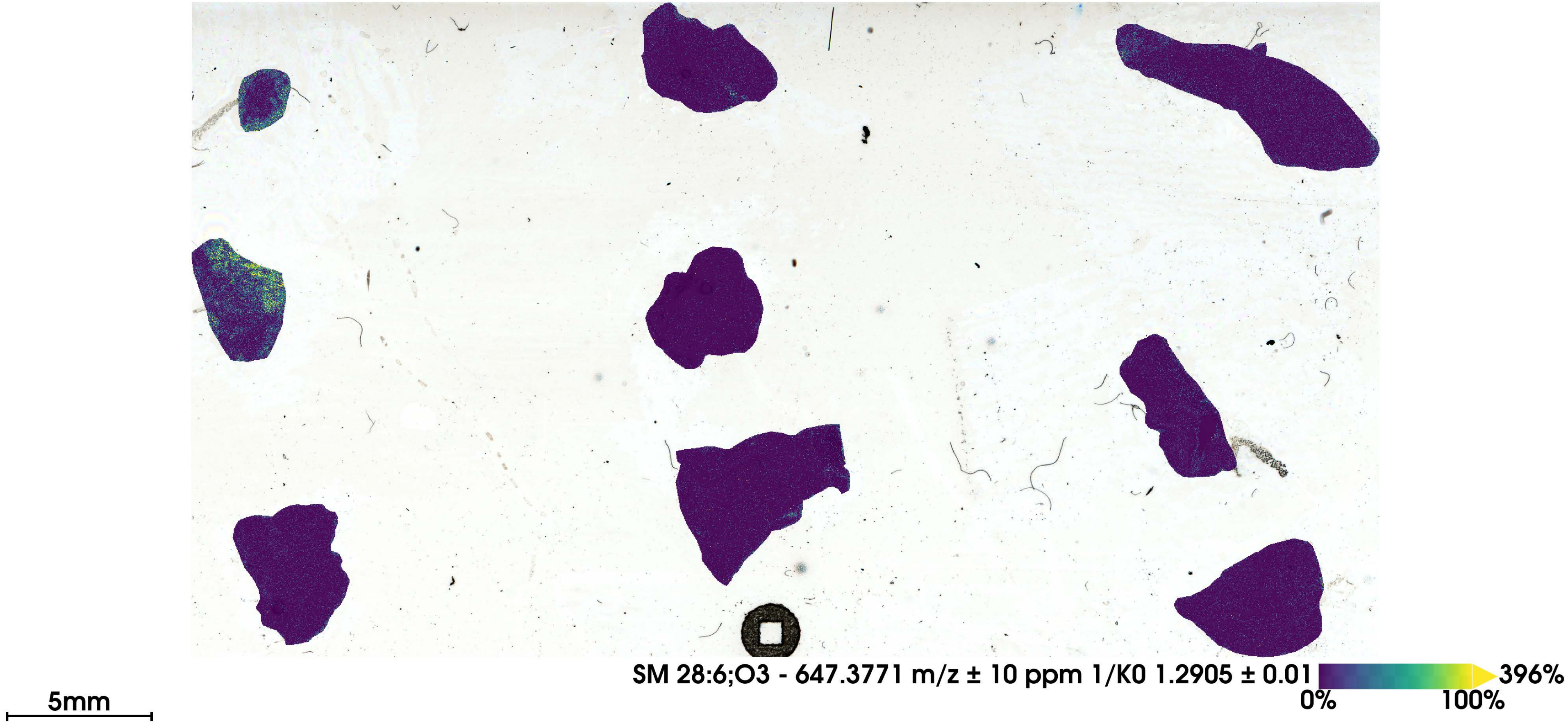

SM 28:6;O3 - 647.3771 m/z ± 10 ppm 1/K0 1.2905 ± 0.01
396%
0%
100%
5mm

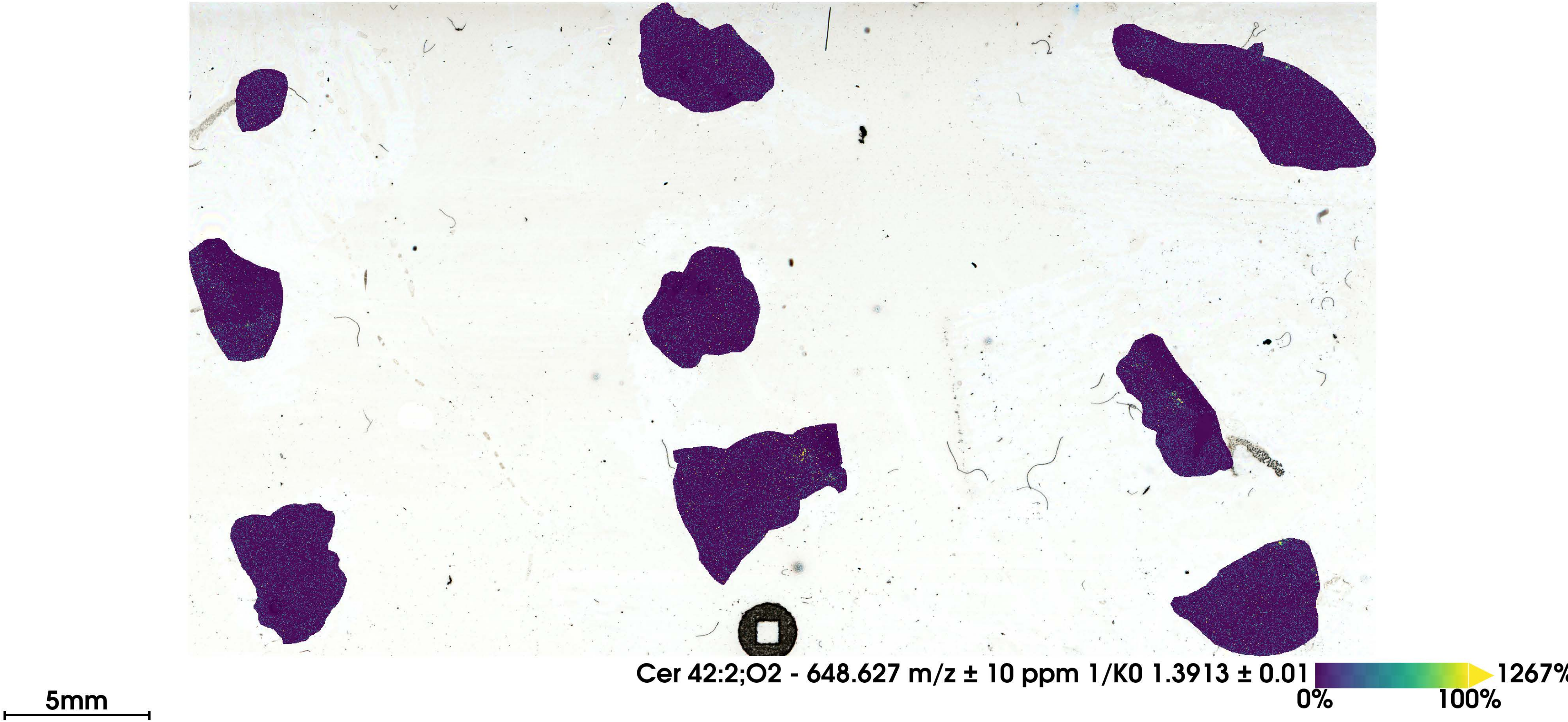
Cer 42:2;O2 - 648.627 m/z ± 10 ppm 1/K0 1.3913 ± 0.01
1267%
0%
100%
5mm

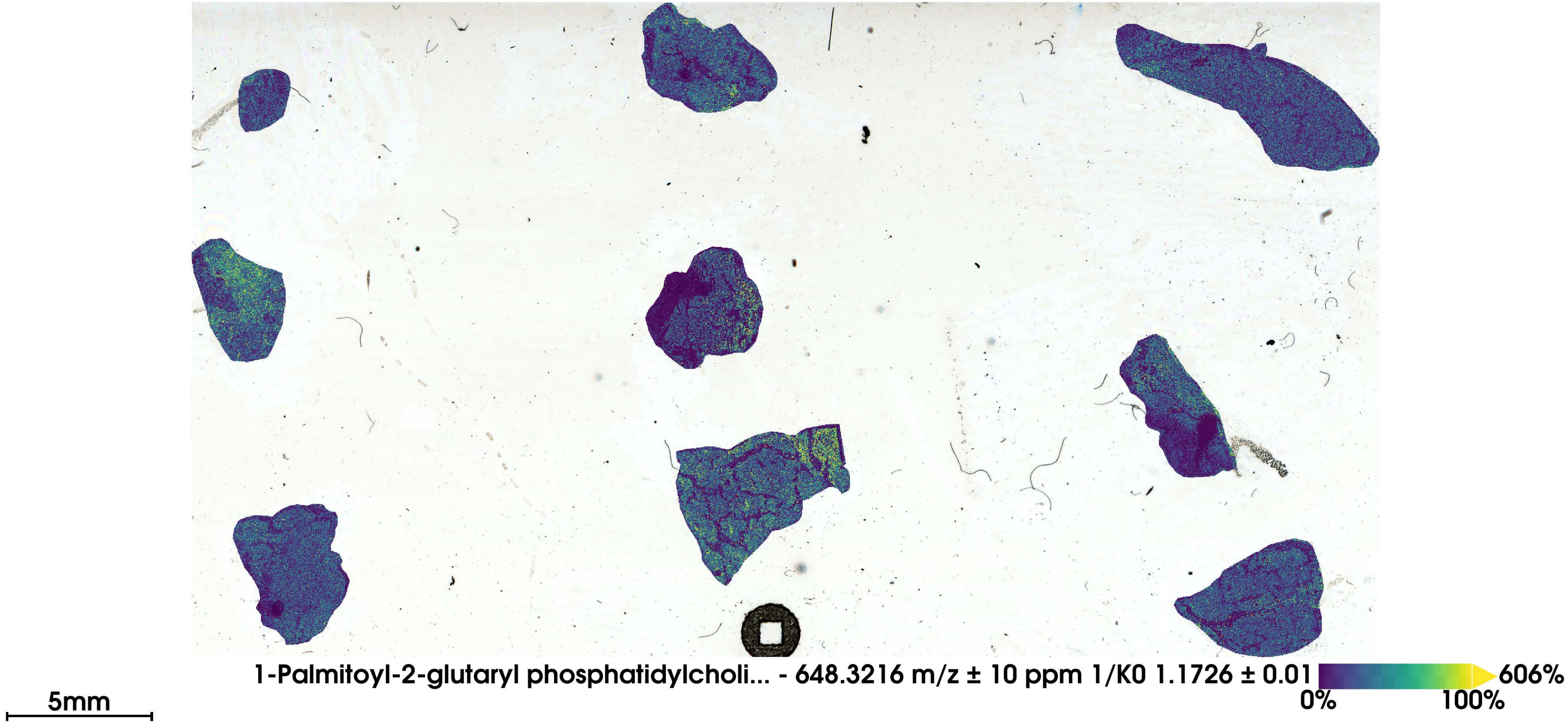
1-Palmitoyl-2-glutaryl phosphatidylcholi... - 648.3216 m/z ± 10 ppm 1/K0 1.1726 ± 0.01
606%
0%
100%
5mm

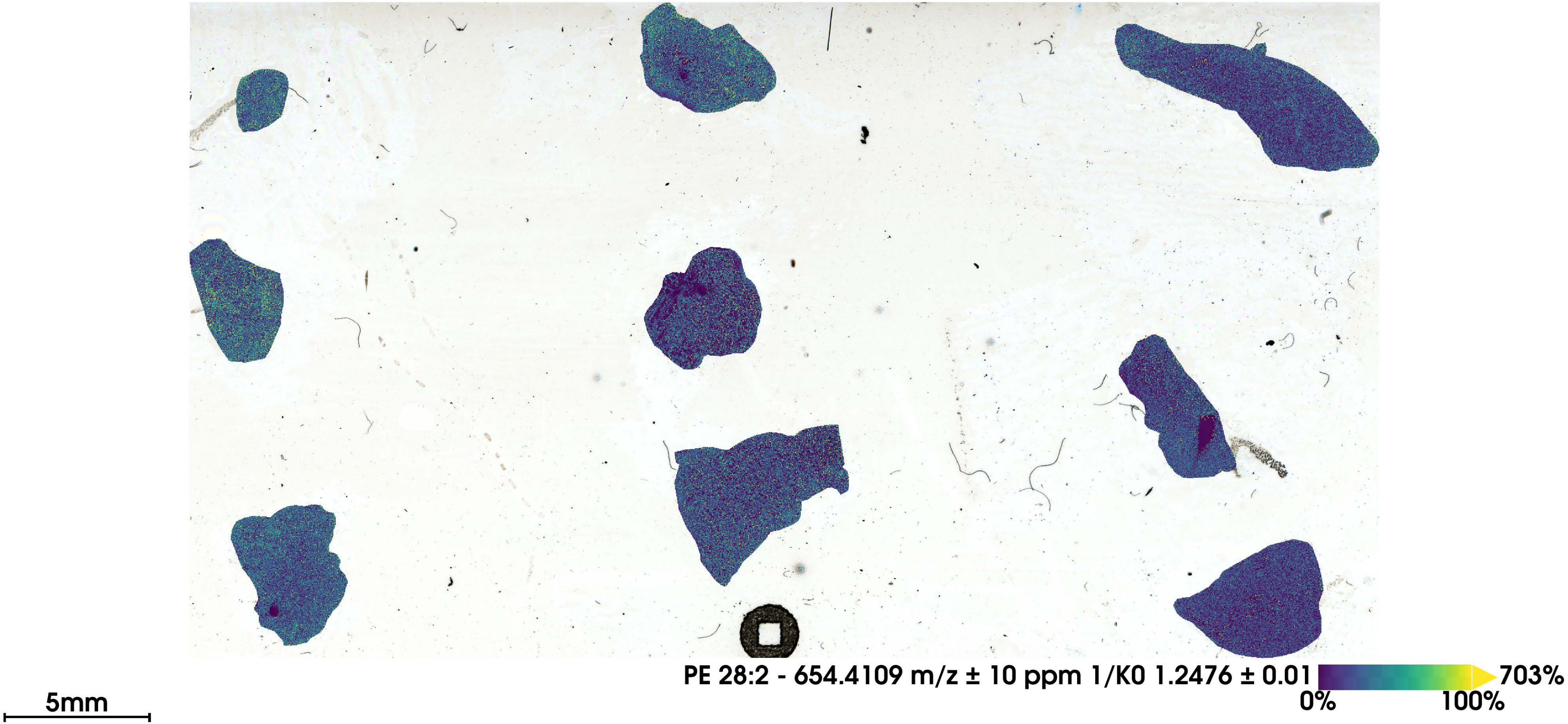
PE 28:2 - 654.4109 m/z ± 10 ppm 1/K0 1.2476 ± 0.01
703%
0%
100%
5mm

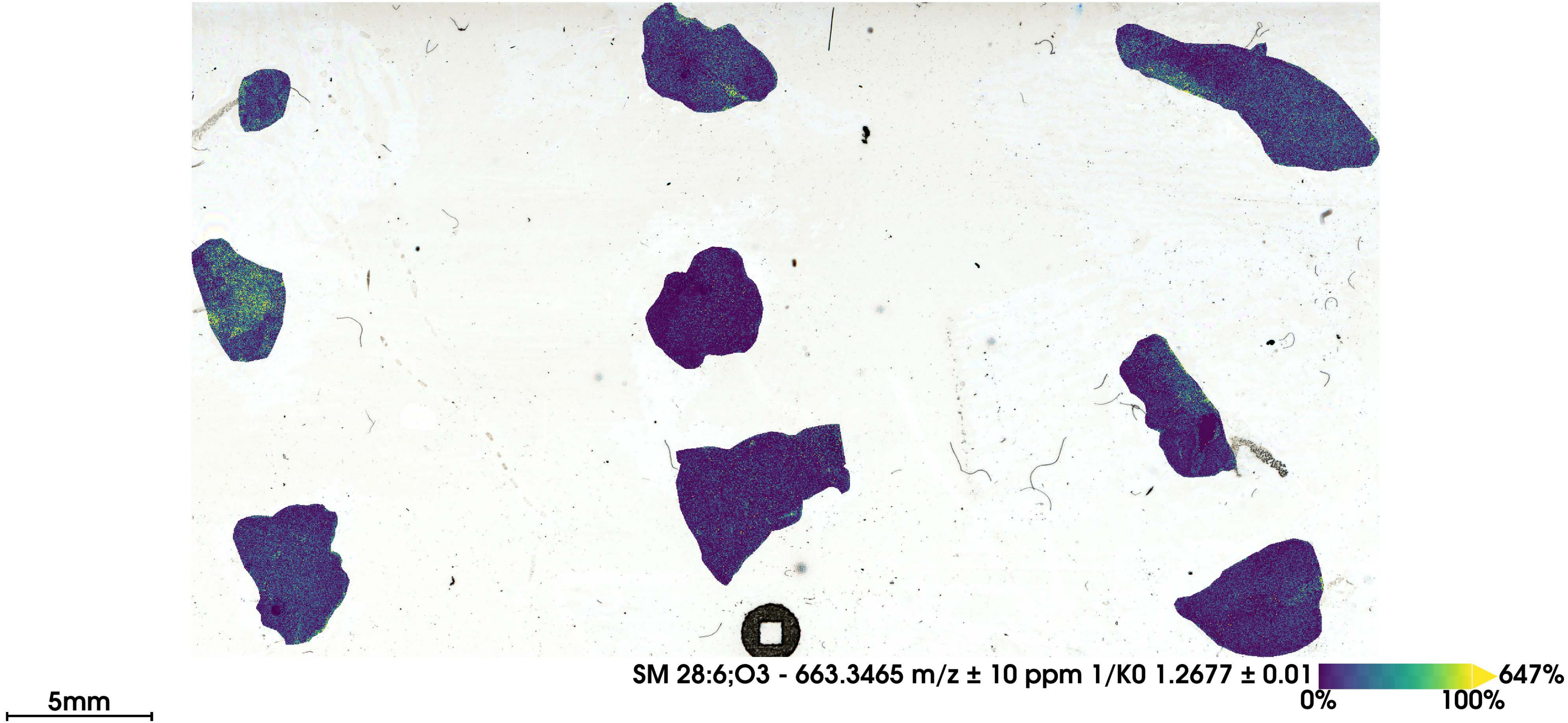

SM 28:6;O3 - 663.3465 m/z ± 10 ppm 1/K0 1.2677 ± 0.01
0%
100%
647%
5mm

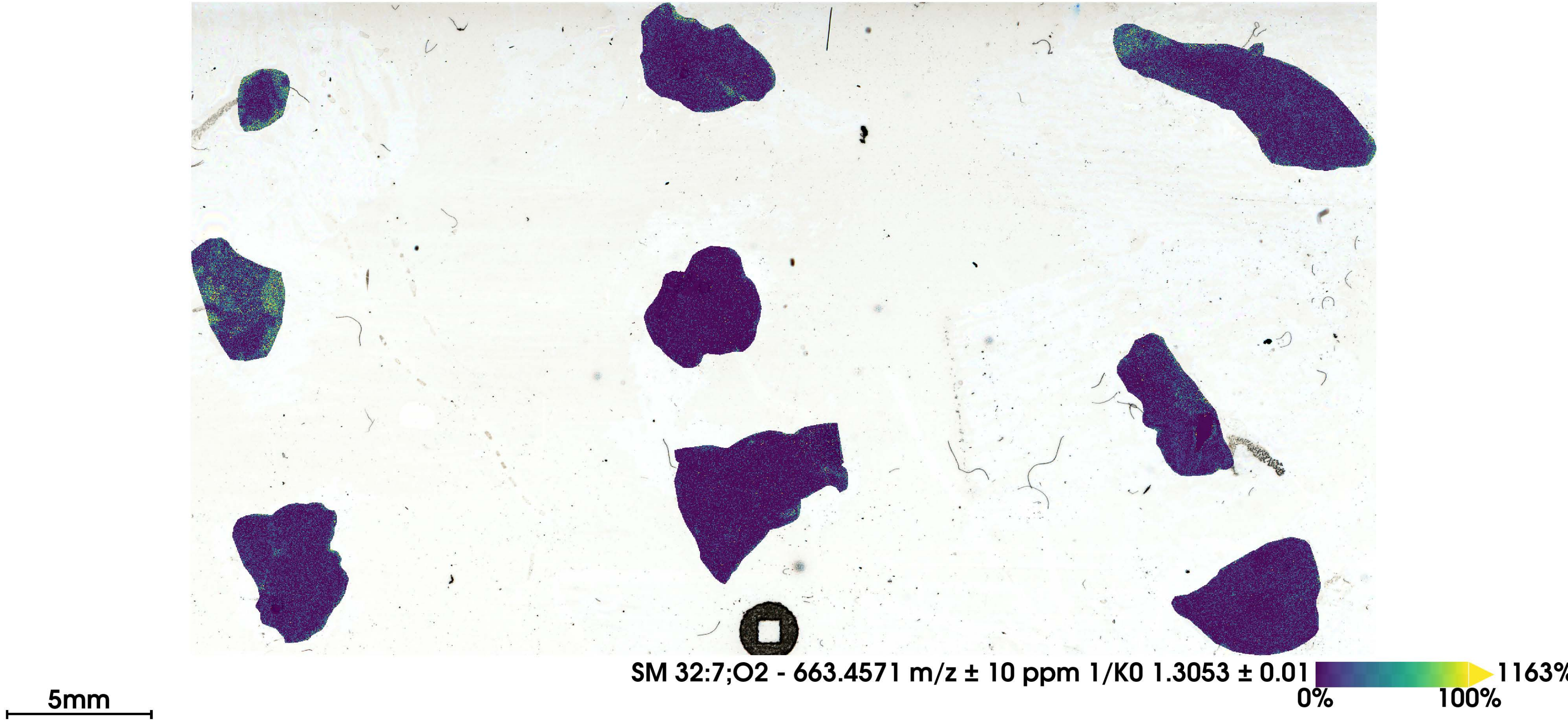
SM 32:7;O2 - 663.4571 m/z ± 10 ppm 1/K0 1.3053 ± 0.01
1163%
0%
100%
5mm

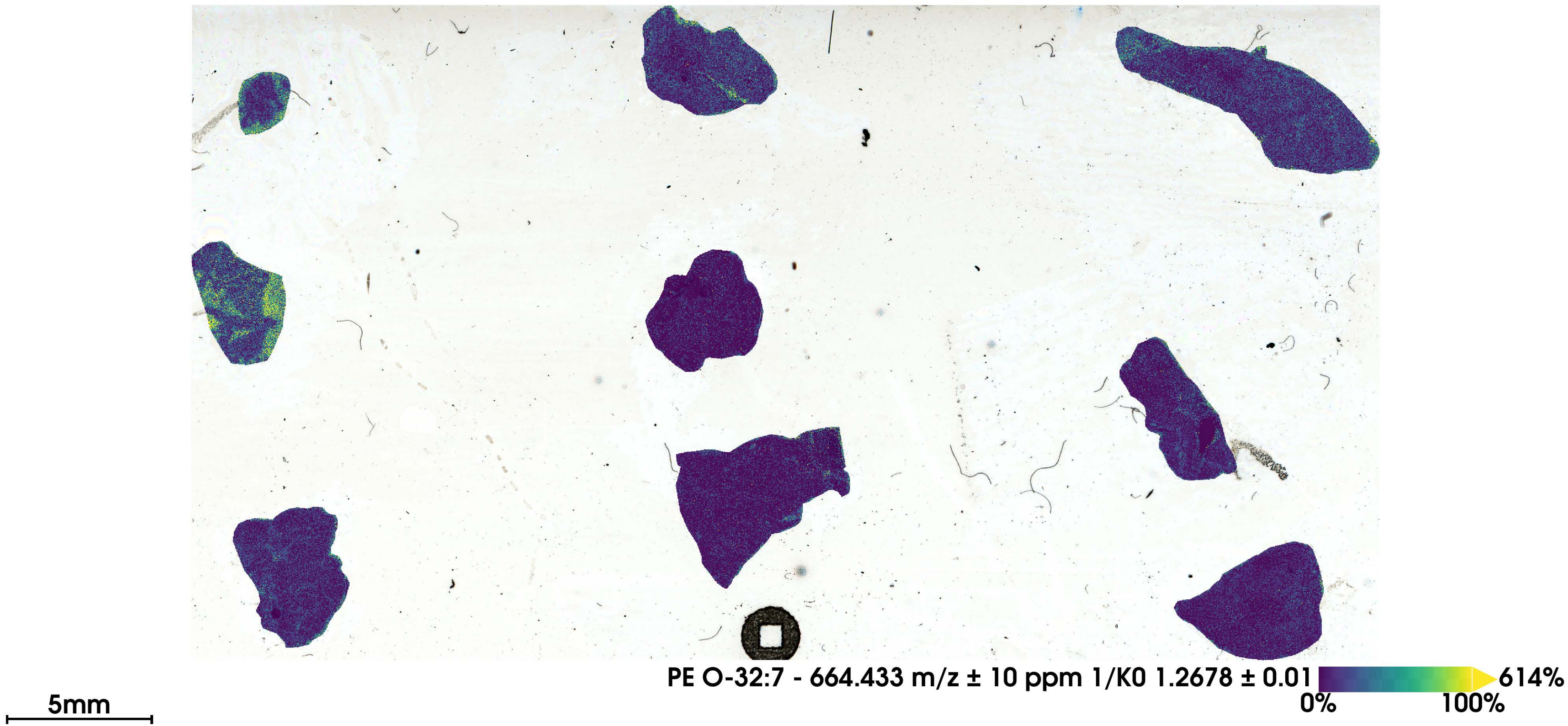

5mm
PE O-32:7 - 664.433 m/z ± 10 ppm 1/K0 1.2678 ± 0.01
614%
0%
100%

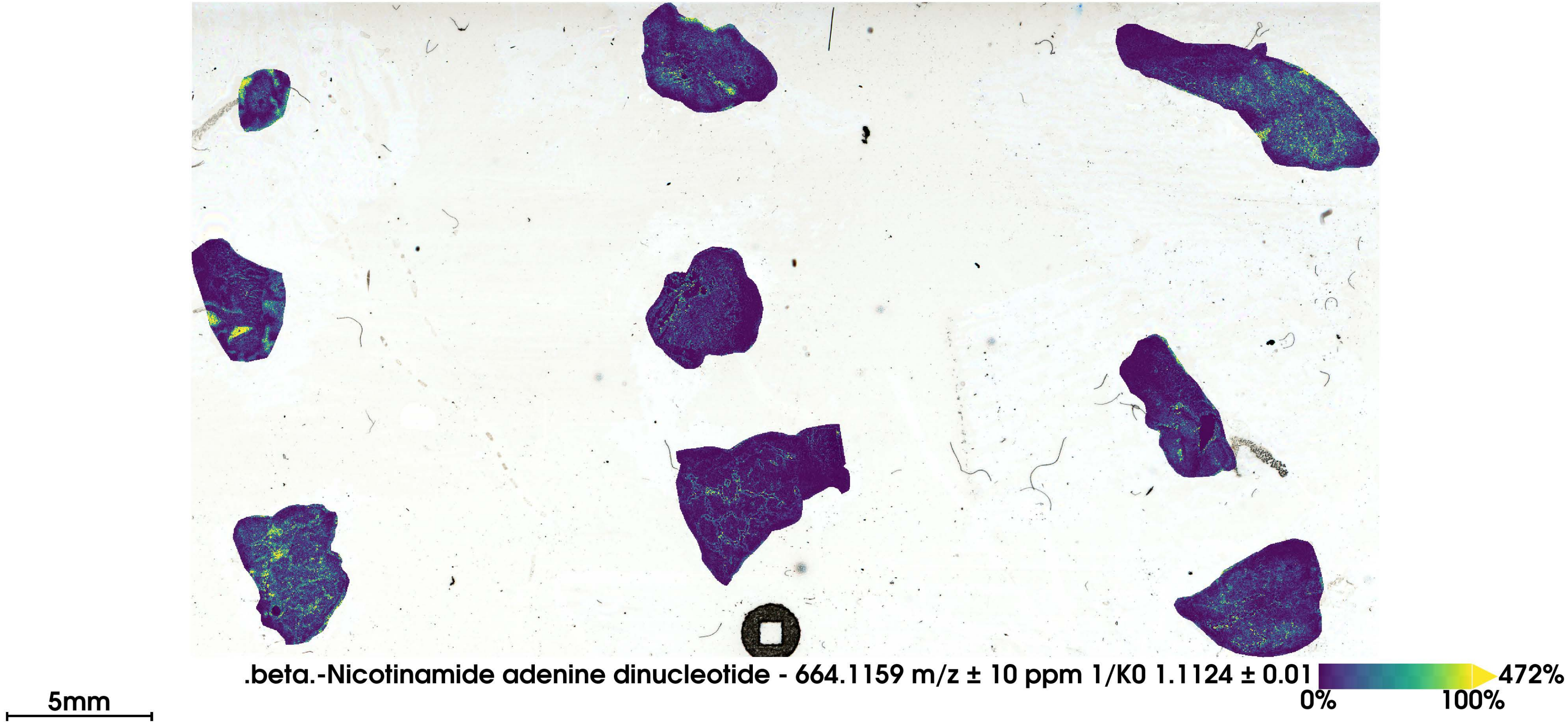
.beta.-Nicotinamide adenine dinucleotide - 664.1159 m/z ± 10 ppm 1/K0 1.1124 ± 0.01
472%
0%
100%
5mm

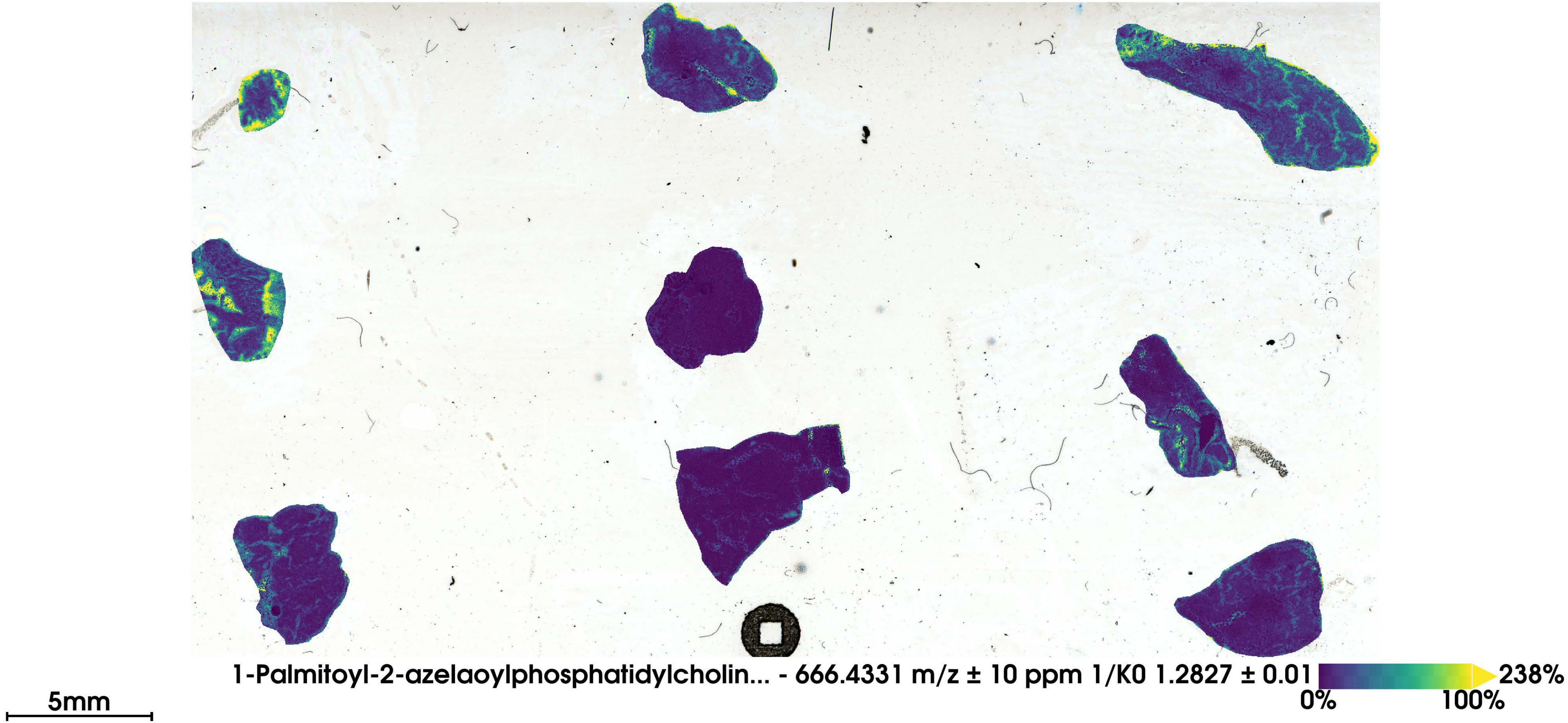

1-Palmitoyl-2-azelaoylphosphatidylcholin... - 666.4331 m/z ± 10 ppm 1/K0 1.2827 ± 0.01
238%
0%
100%
5mm

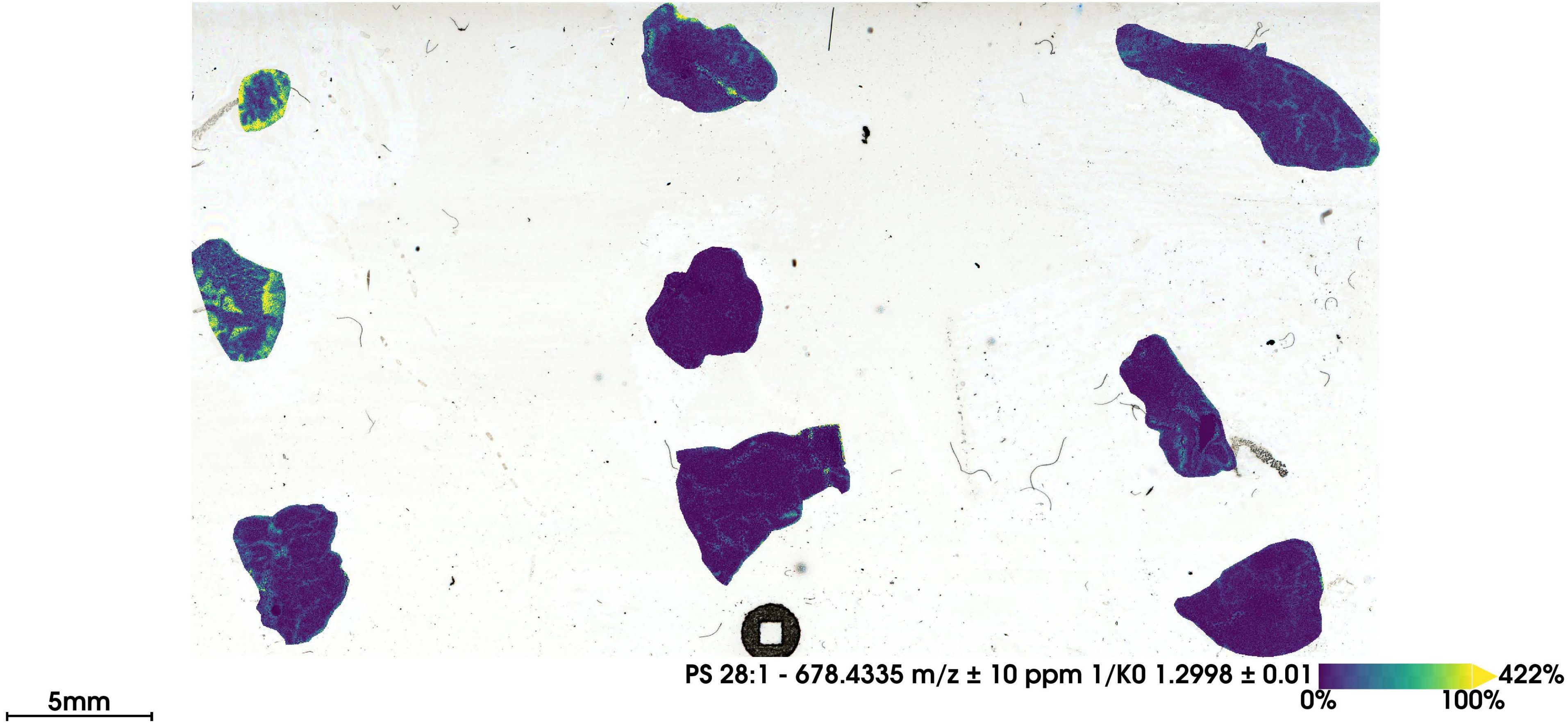
PS 28:1 - 678.4335 m/z ± 10 ppm 1/K0 1.2998 ± 0.01
422%
0%
100%
5mm

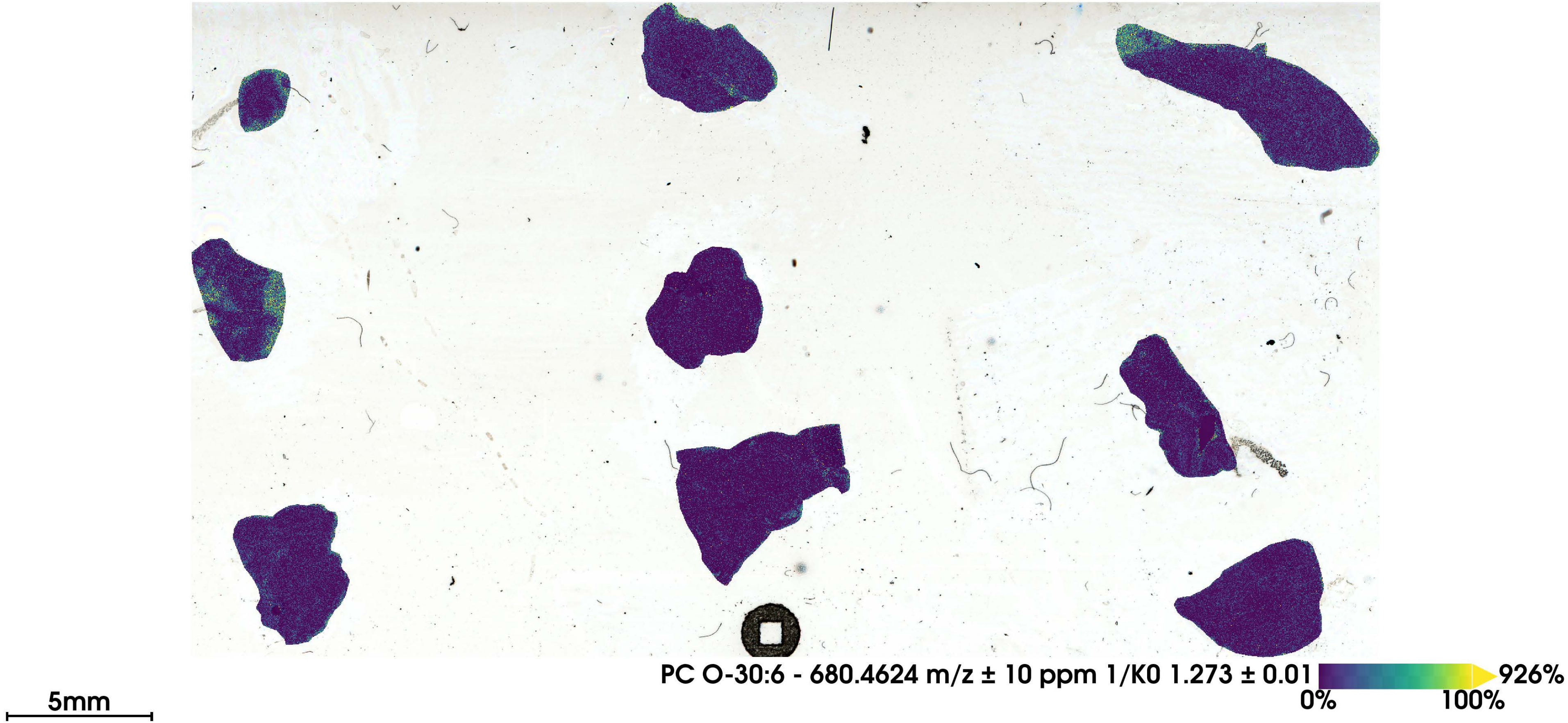
PC O-30:6 - 680.4624 m/z ± 10 ppm 1/K0 1.273 ± 0.01
926%
0%
100%
5mm

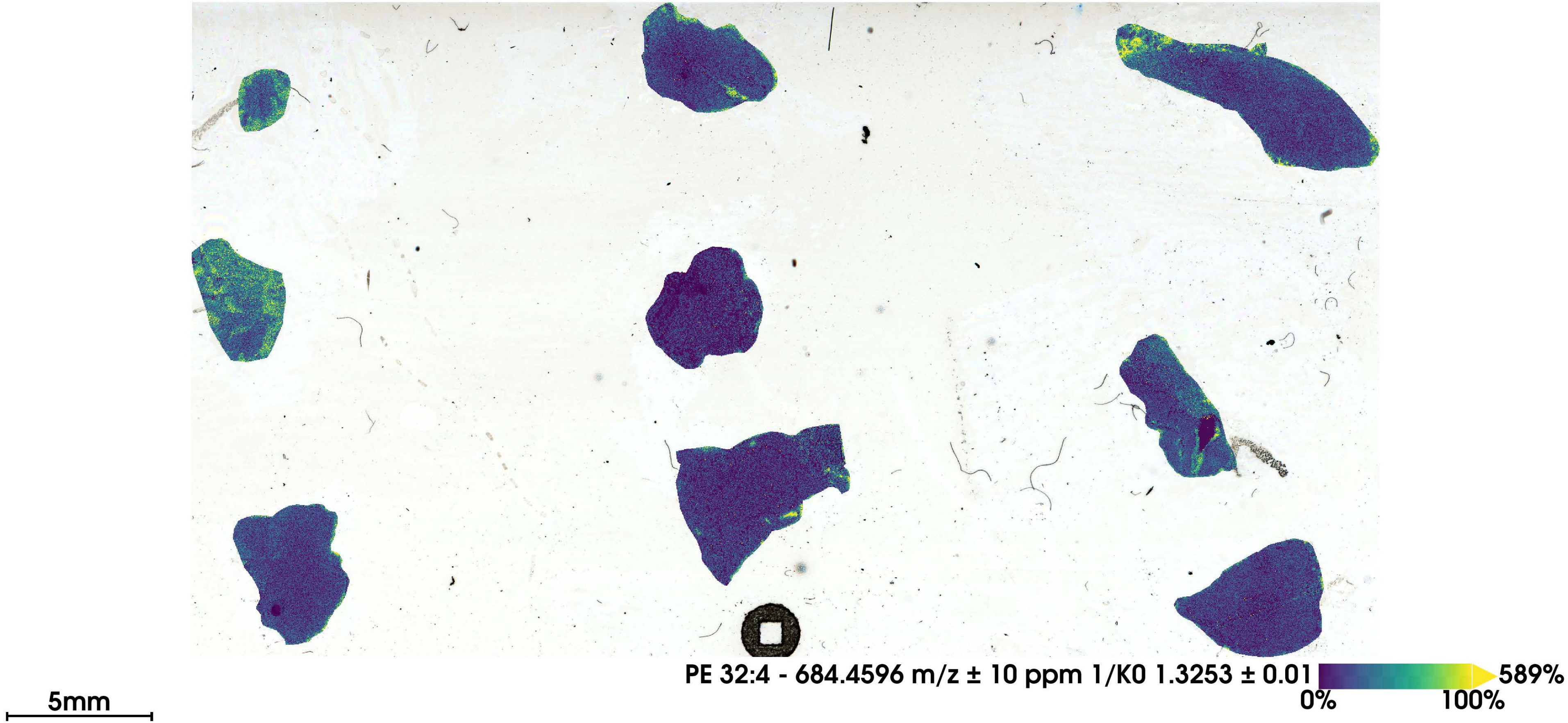

PE 32:4 - 684.4596 m/z ± 10 ppm 1/K0 1.3253 ± 0.01
589%
0%
100%
5mm

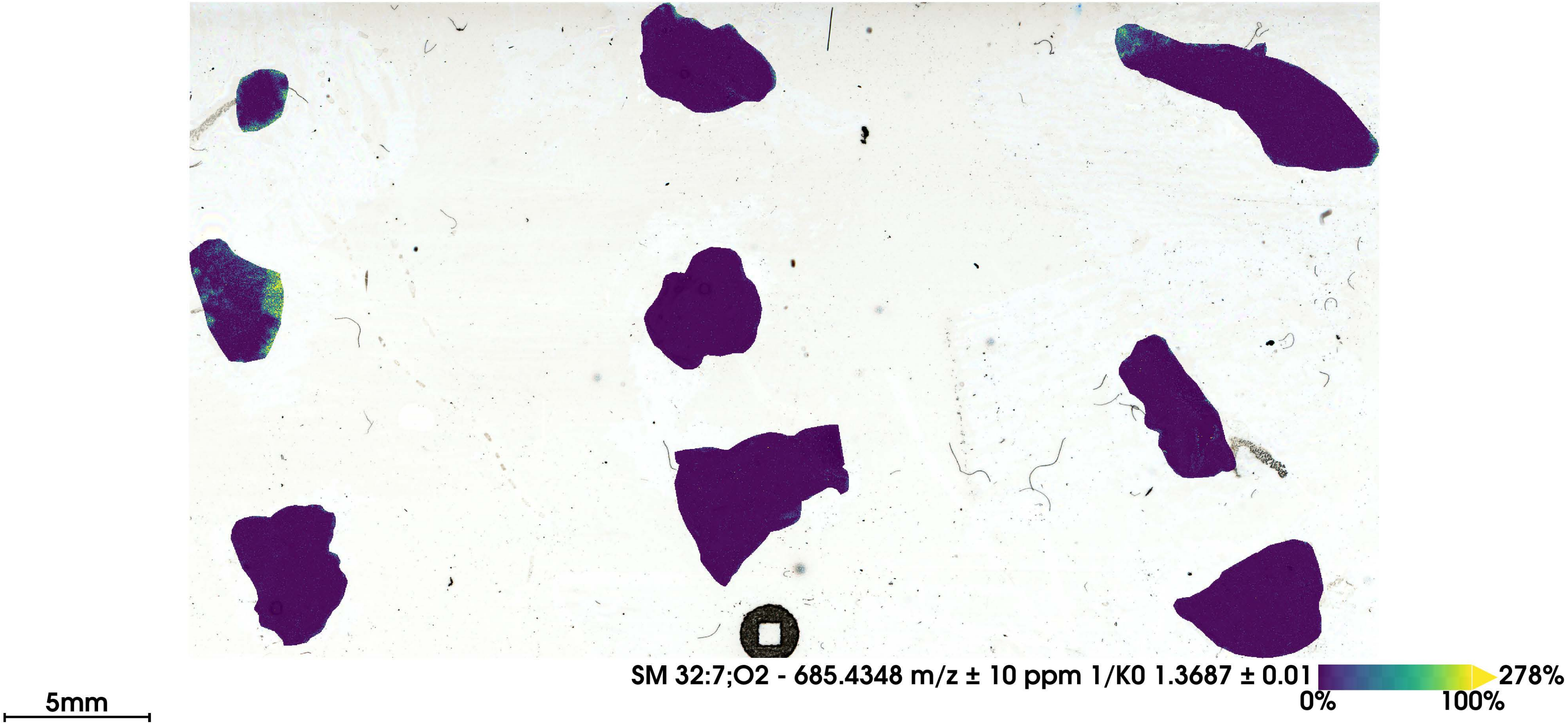
SM 32:7;O2 - 685.4348 m/z ± 10 ppm 1/K0 1.3687 ± 0.01
278%
0%
100%
5mm

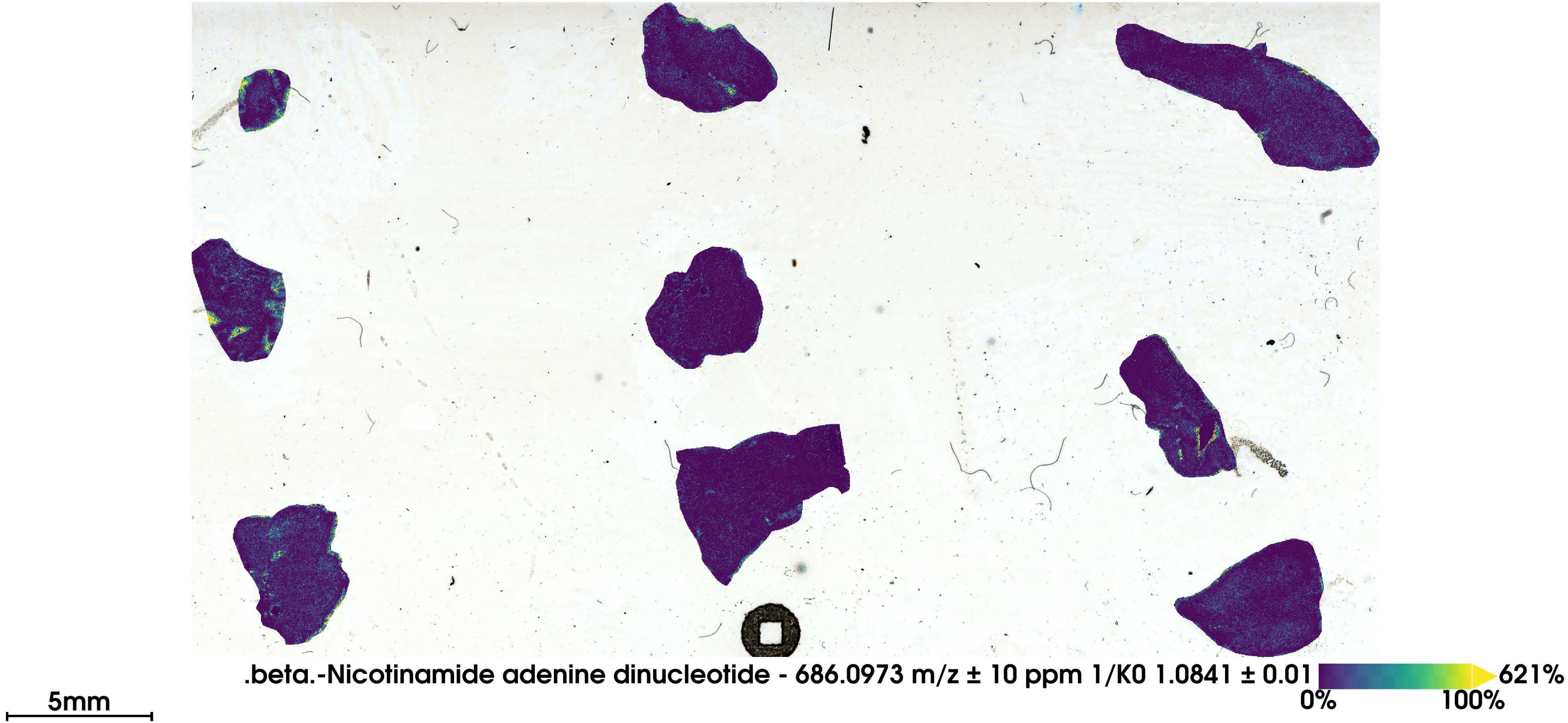
5mm
.beta.-Nicotinamide adenine dinucleotide - 686.0973 m/z ± 10 ppm 1/K0 1.0841 ± 0.01
621%
0%
100%

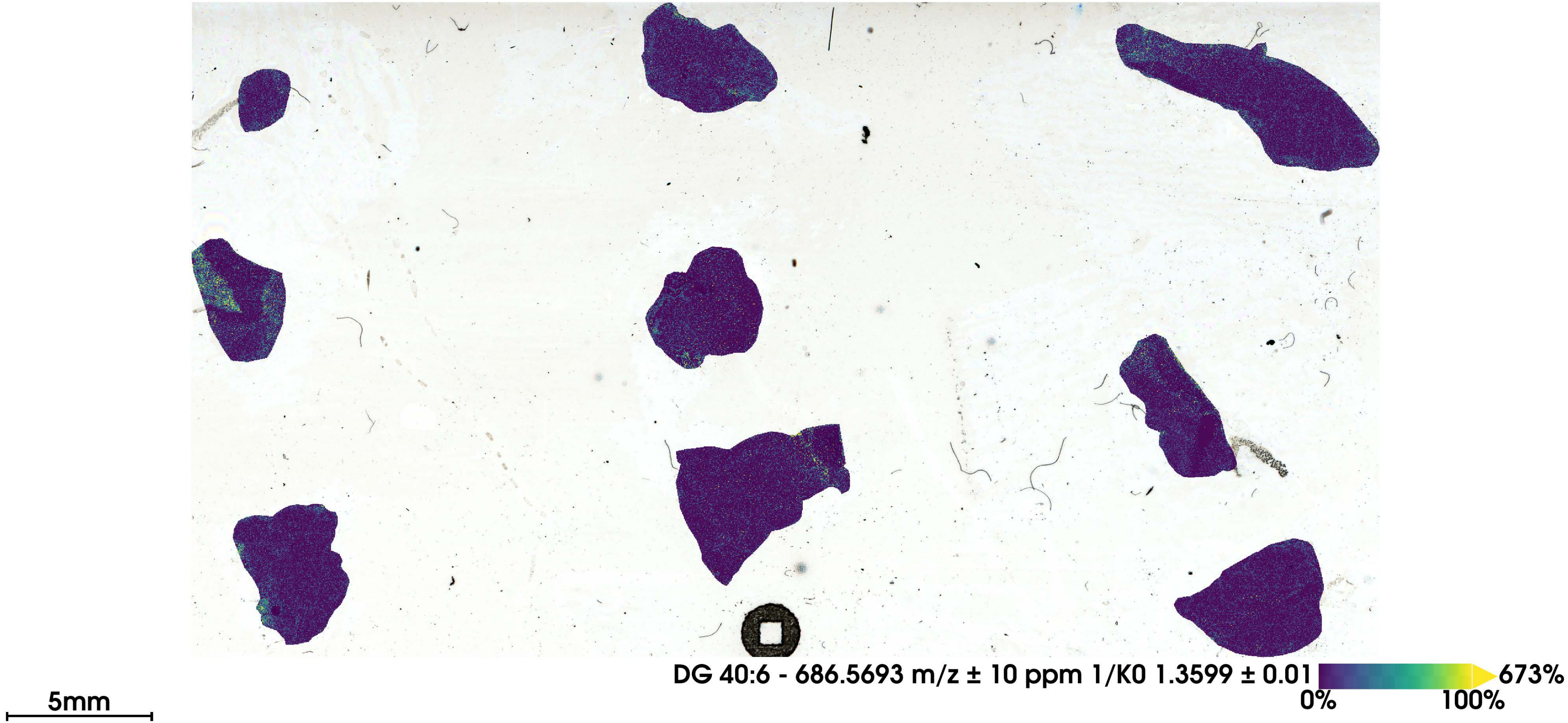

DG 40:6 - 686.5693 m/z ± 10 ppm 1/K0 1.3599 ± 0.01
673%
0%
100%
5mm

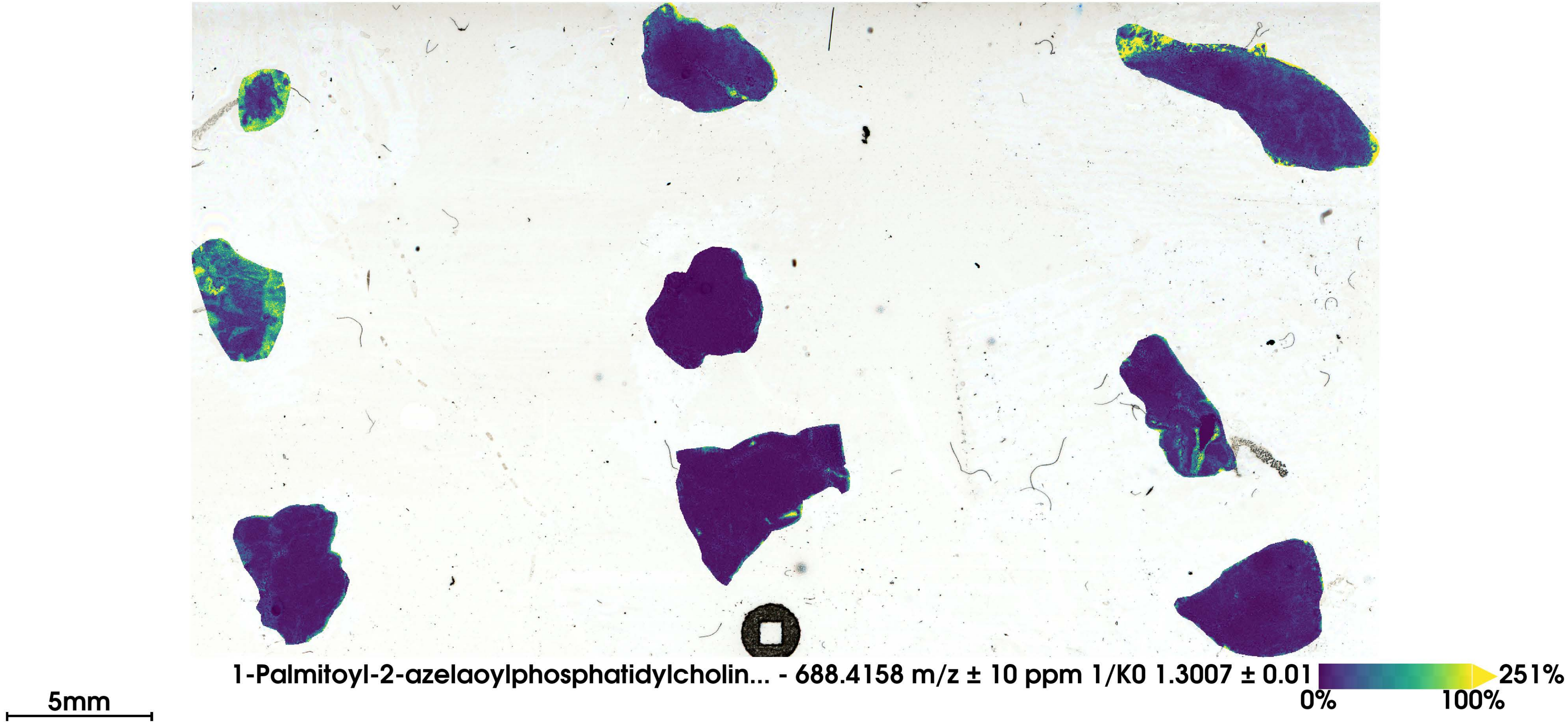

1-Palmitoyl-2-azelaoylphosphatidylcholin... - 688.4158 m/z ± 10 ppm 1/K0 1.3007 ± 0.01
251%
0%
100%
5mm

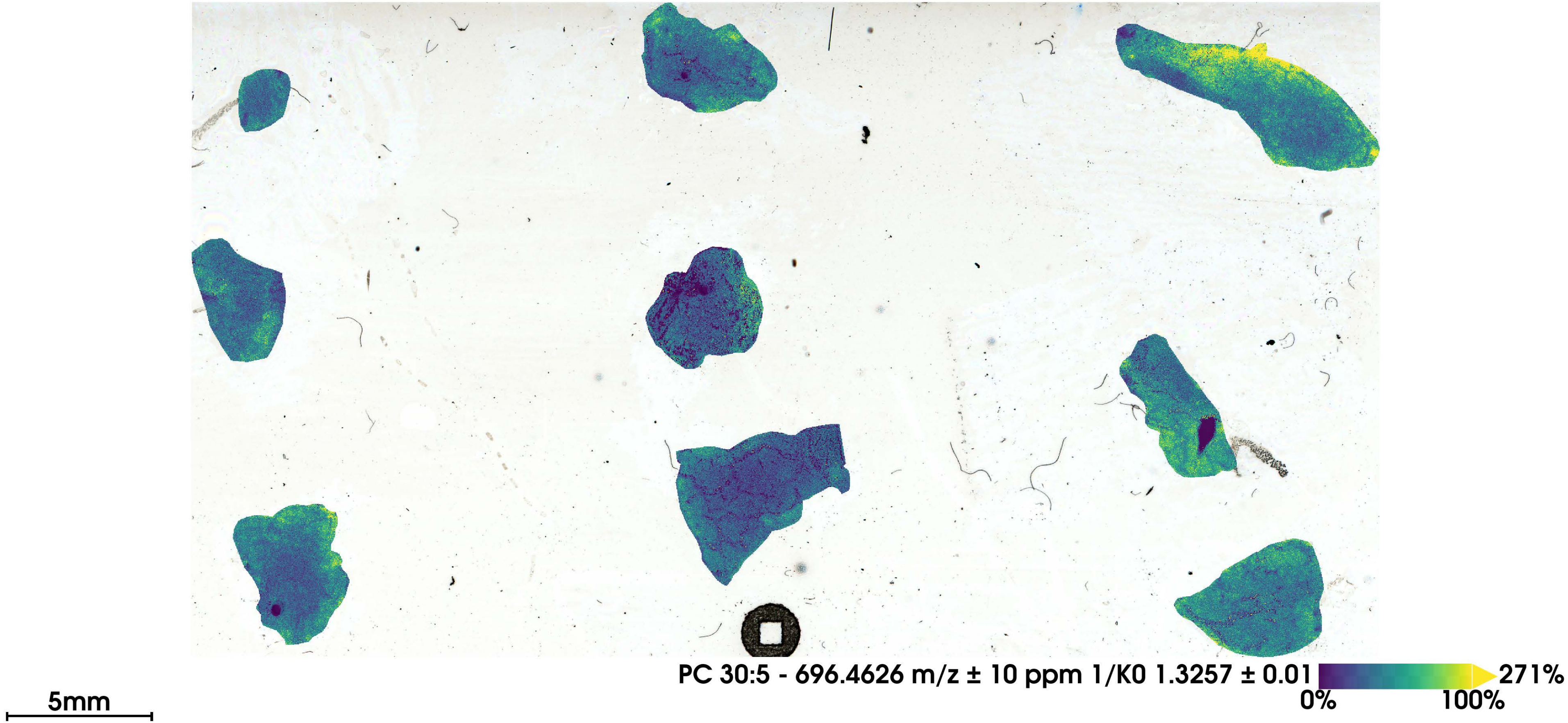
PC 30:5 - 696.4626 m/z ± 10 ppm 1/K0 1.3257 ± 0.01
271%
0%
100%
5mm

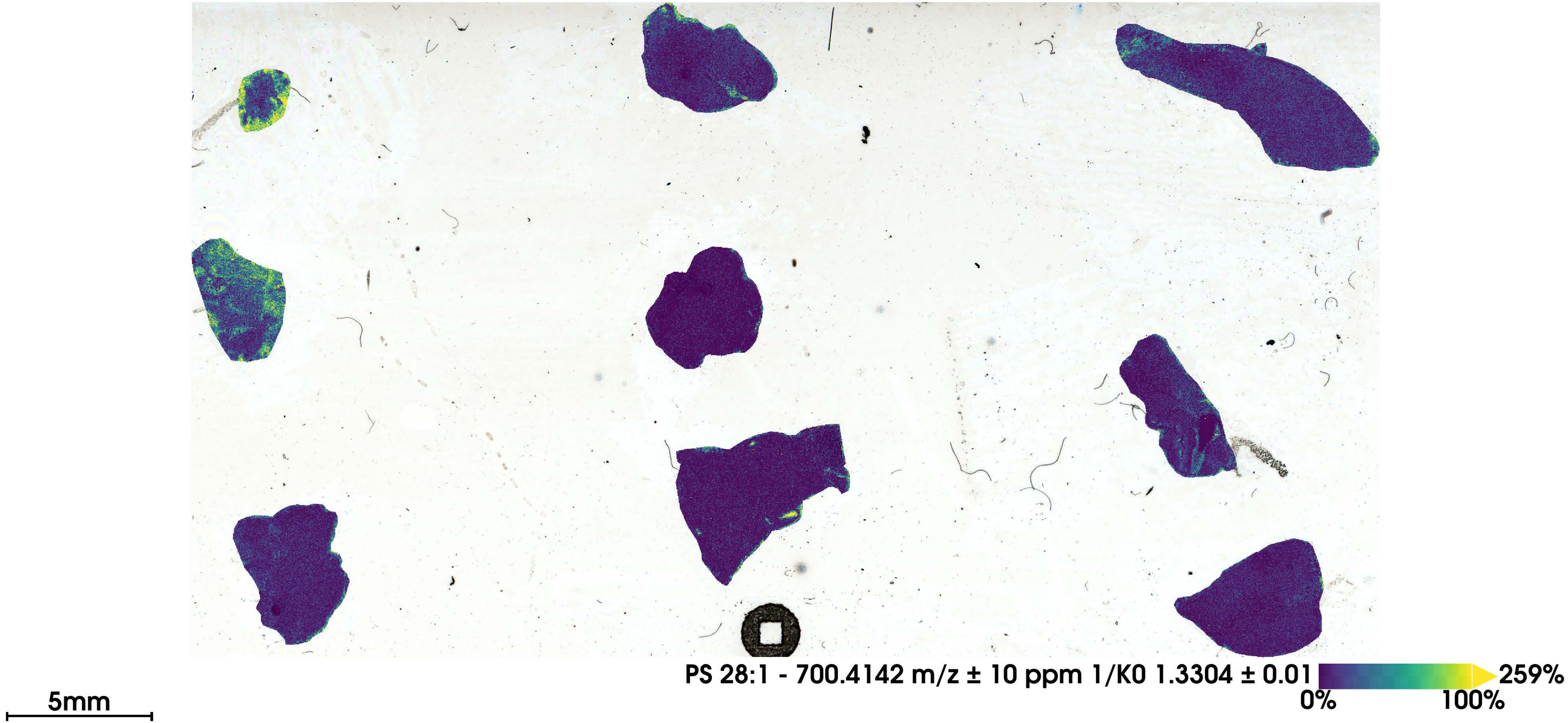

PS 28:1 - 700.4142 m/z ± 10 ppm 1/K0 1.3304 ± 0.01
259%
0%
100%
5mm

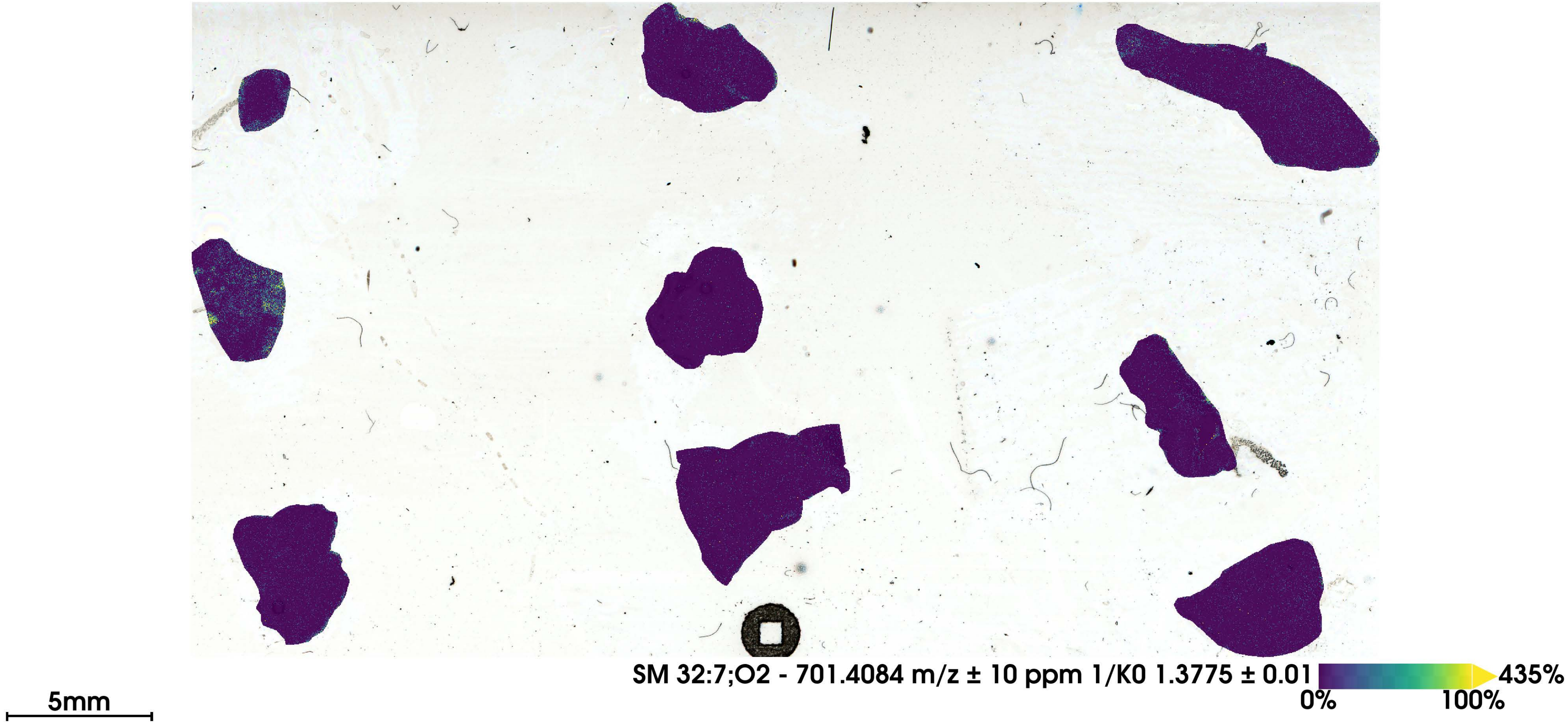
SM 32:7;O2 - 701.4084 m/z ± 10 ppm 1/K0 1.3775 ± 0.01
435%
0%
100%
5mm

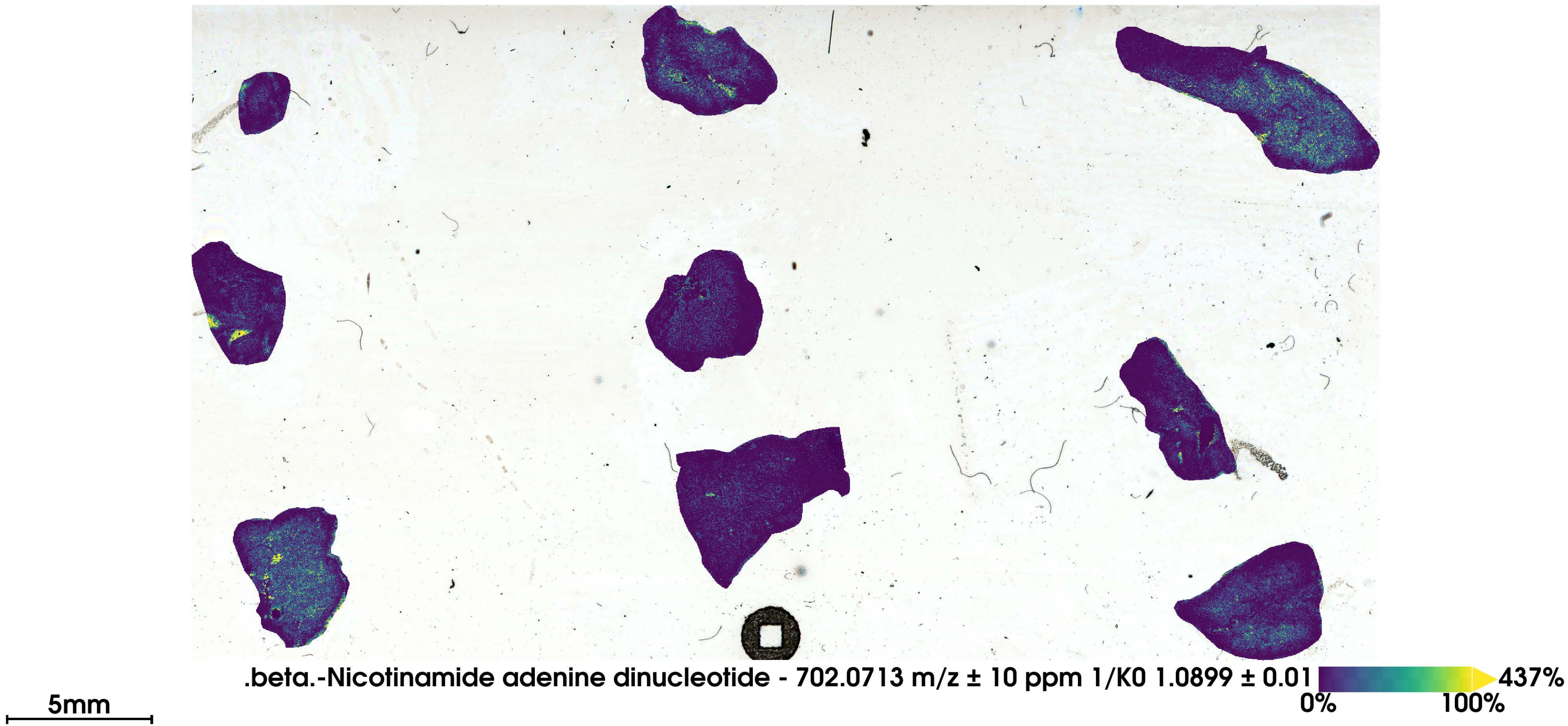
.beta.-Nicotinamide adenine dinucleotide - 702.0713 m/z ± 10 ppm 1/K0 1.0899 ± 0.01
437%
0%
100%
5mm

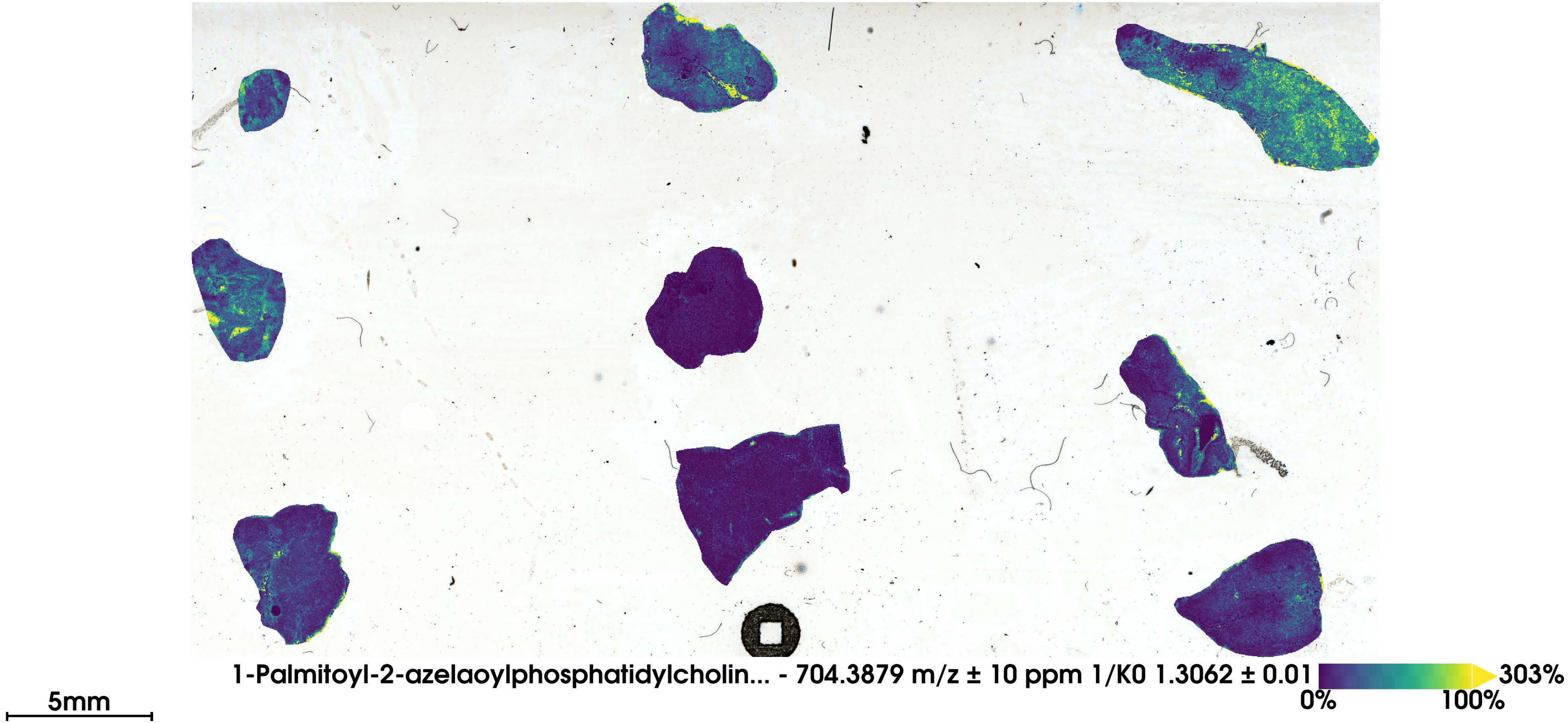
1-Palmitoyl-2-azelaoylphosphatidylcholin... - 704.3879 m/z ± 10 ppm 1/K0 1.3062 ± 0.01
303%
0%
100%
5mm

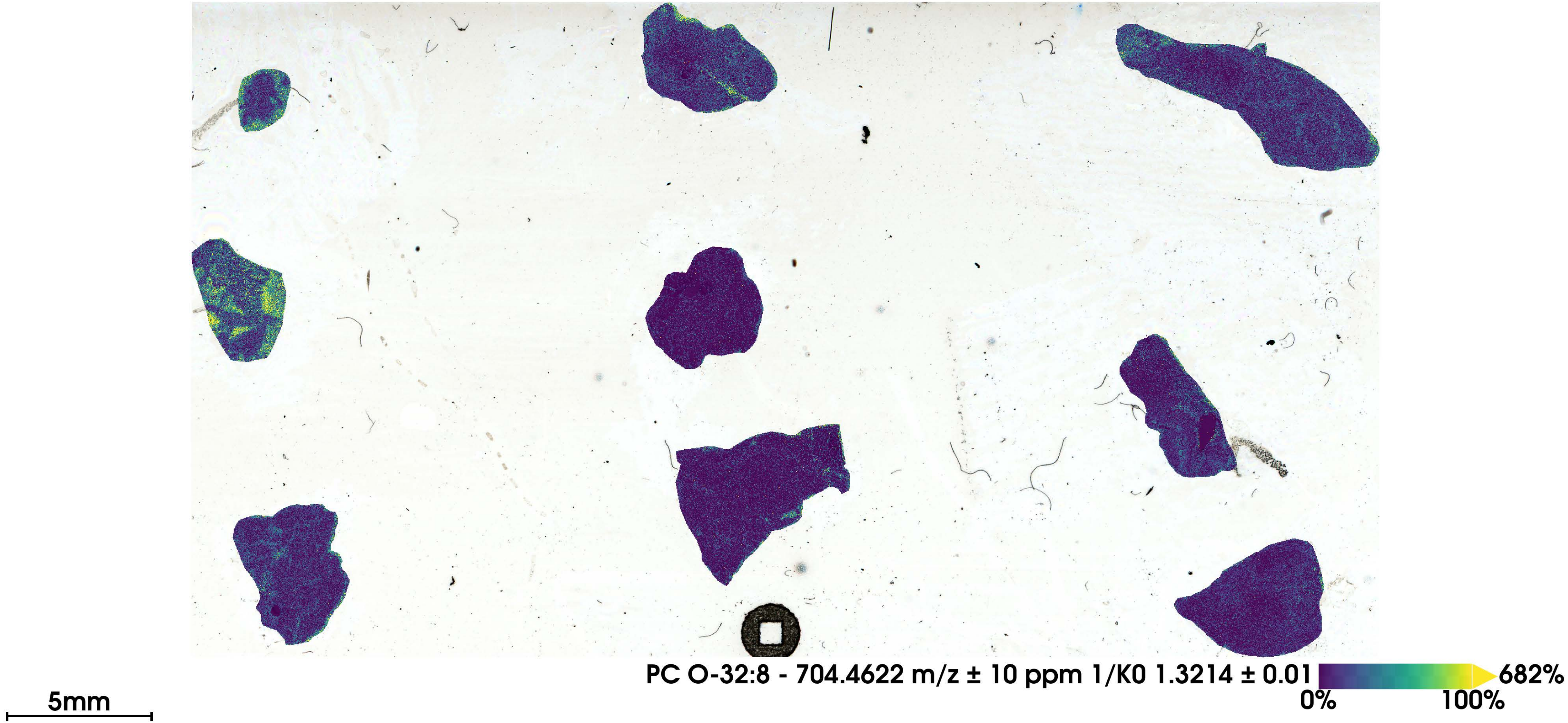
PC O-32:8 - 704.4622 m/z ± 10 ppm 1/K0 1.3214 ± 0.01
682%
0%
100%
5mm

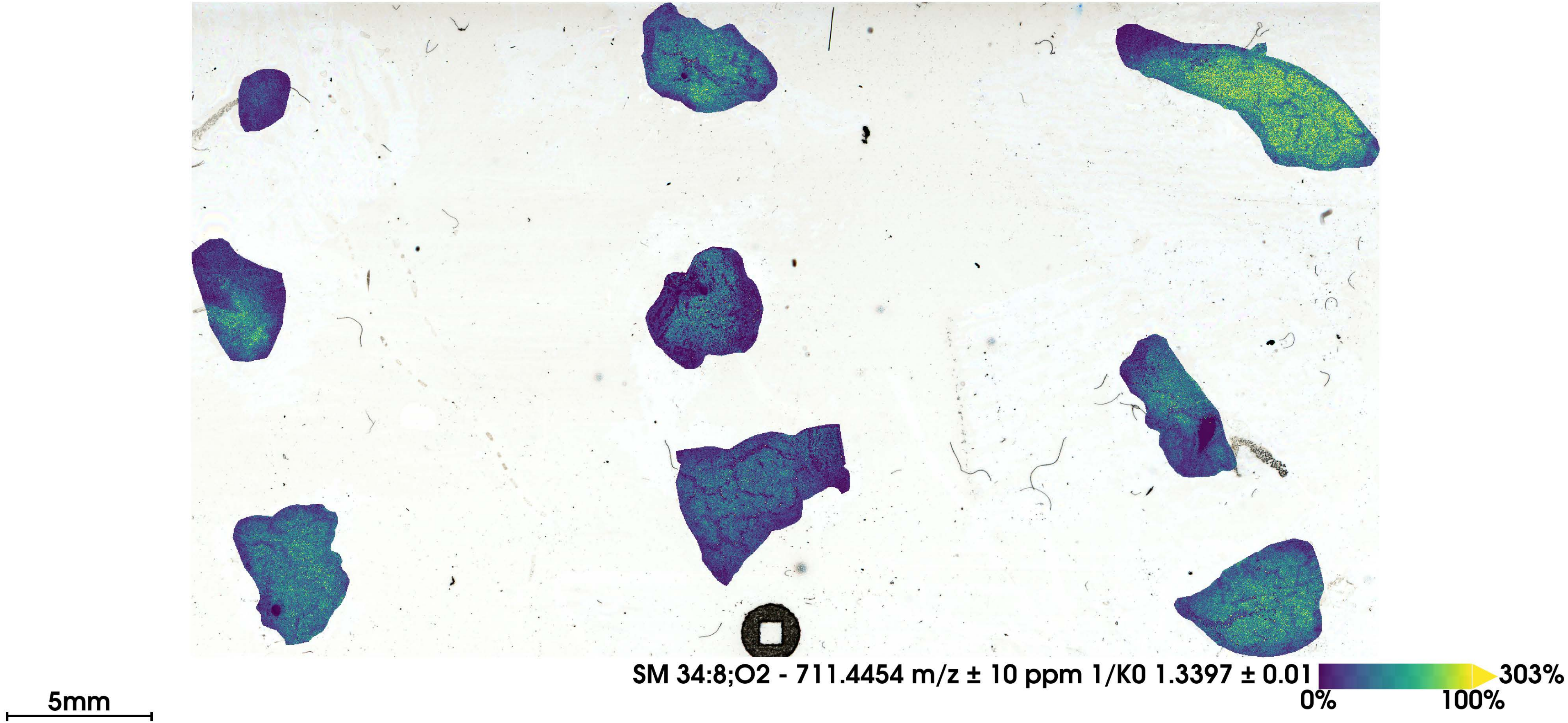

5mm
SM 34:8;O2 - 711.4454 m/z ± 10 ppm 1/K0 1.3397 ± 0.01
0%
100%
303%

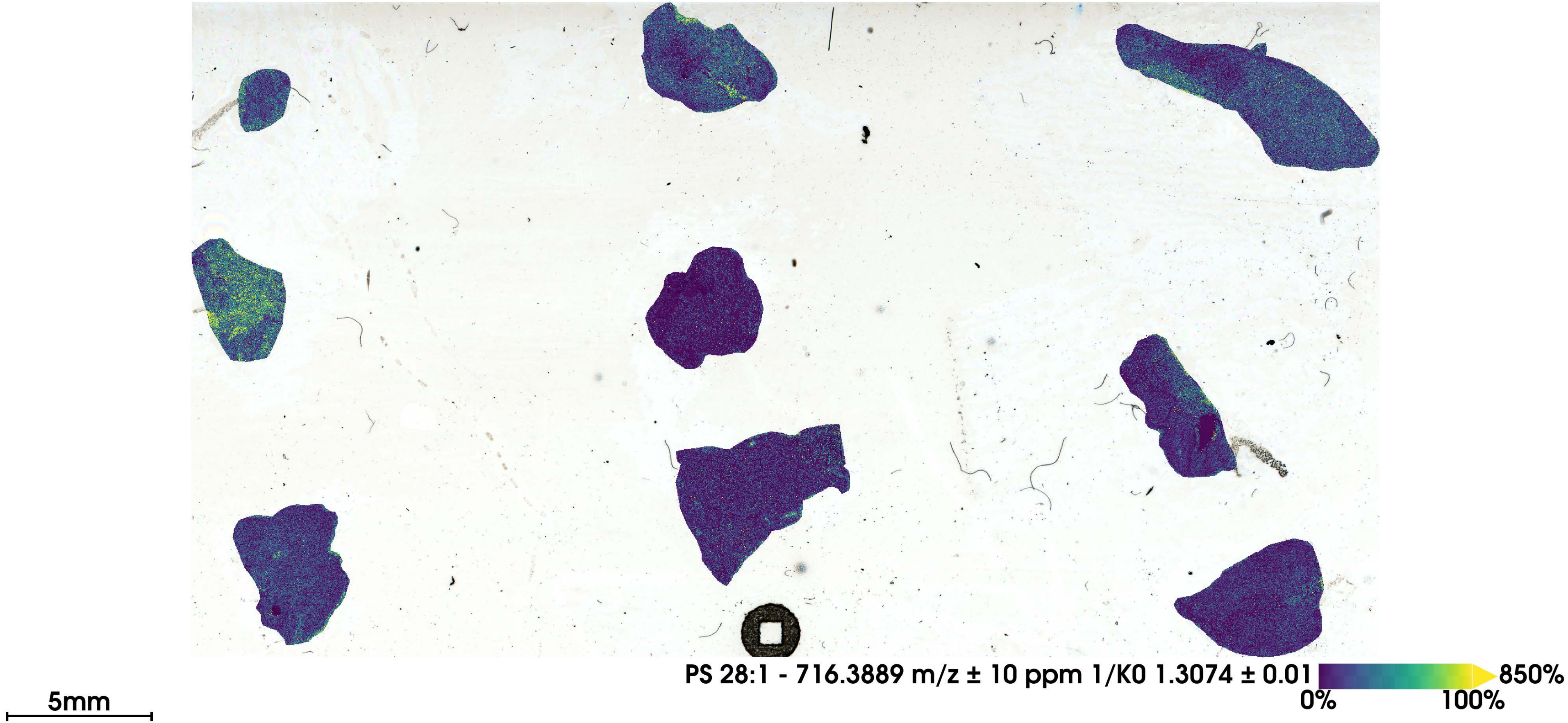
PS 28:1 - 716.3889 m/z ± 10 ppm 1/K0 1.3074 ± 0.01
850%
0%
100%
5mm

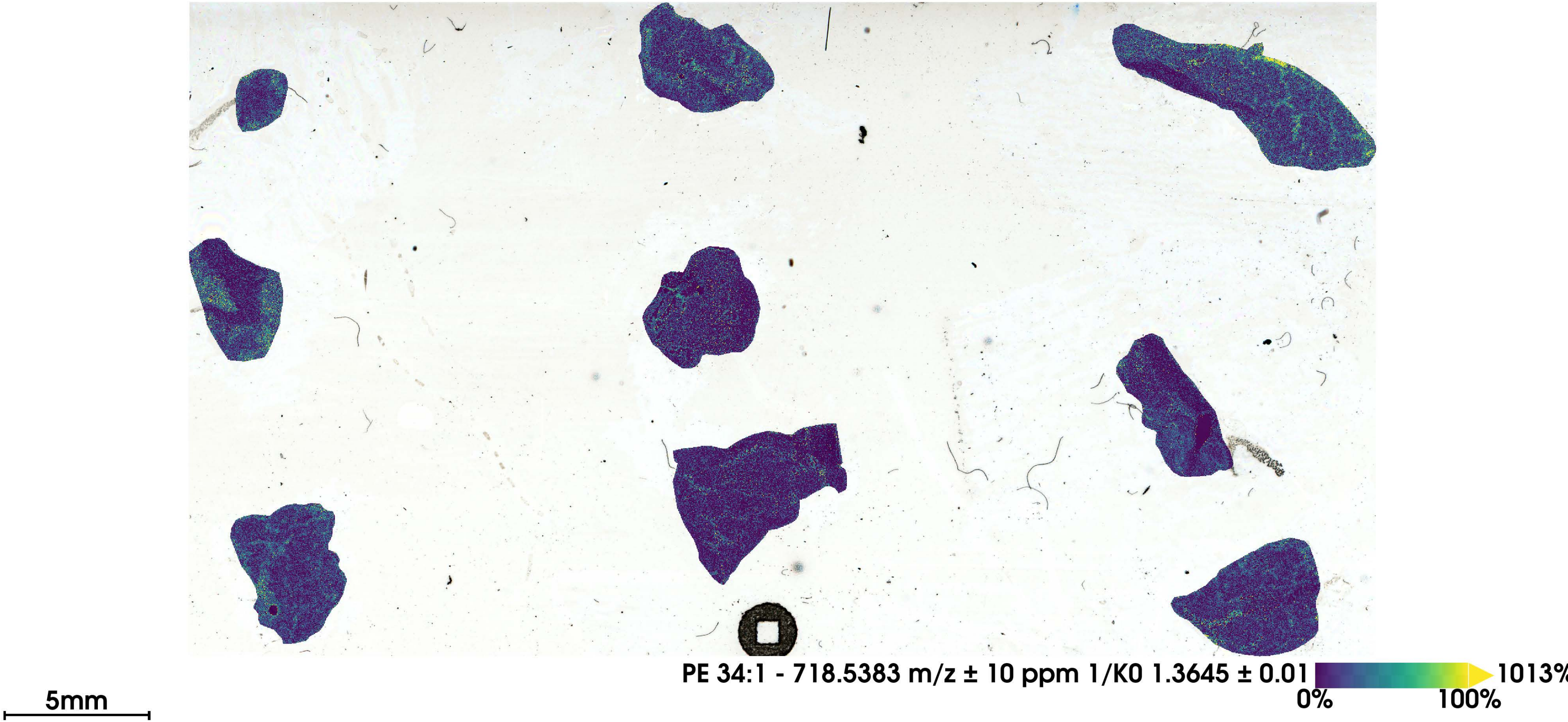
PE 34:1 - 718.5383 m/z ± 10 ppm 1/K0 1.3645 ± 0.01
1013%
0%
100%
5mm

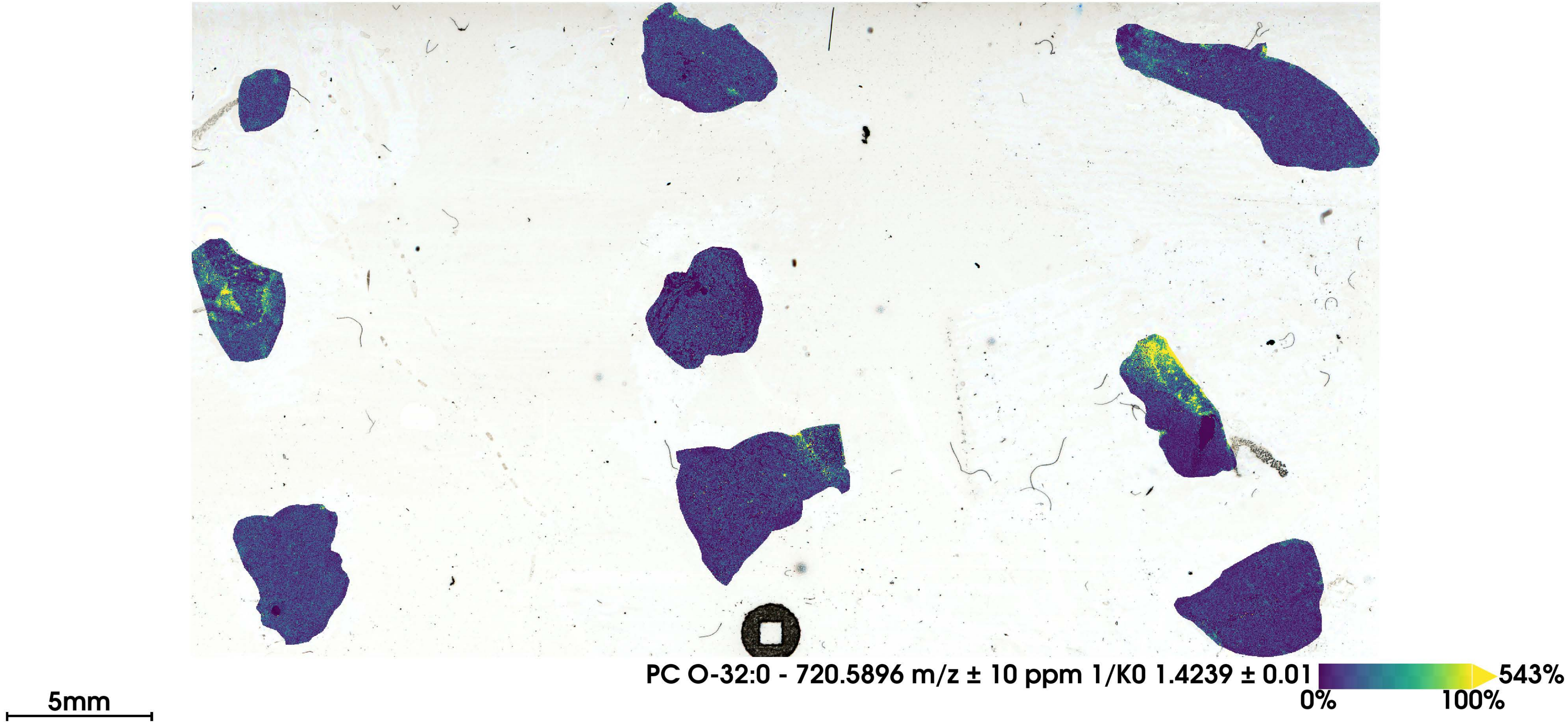
PC O-32:0 - 720.5896 m/z ± 10 ppm 1/K0 1.4239 ± 0.01
543%
0%
100%
5mm

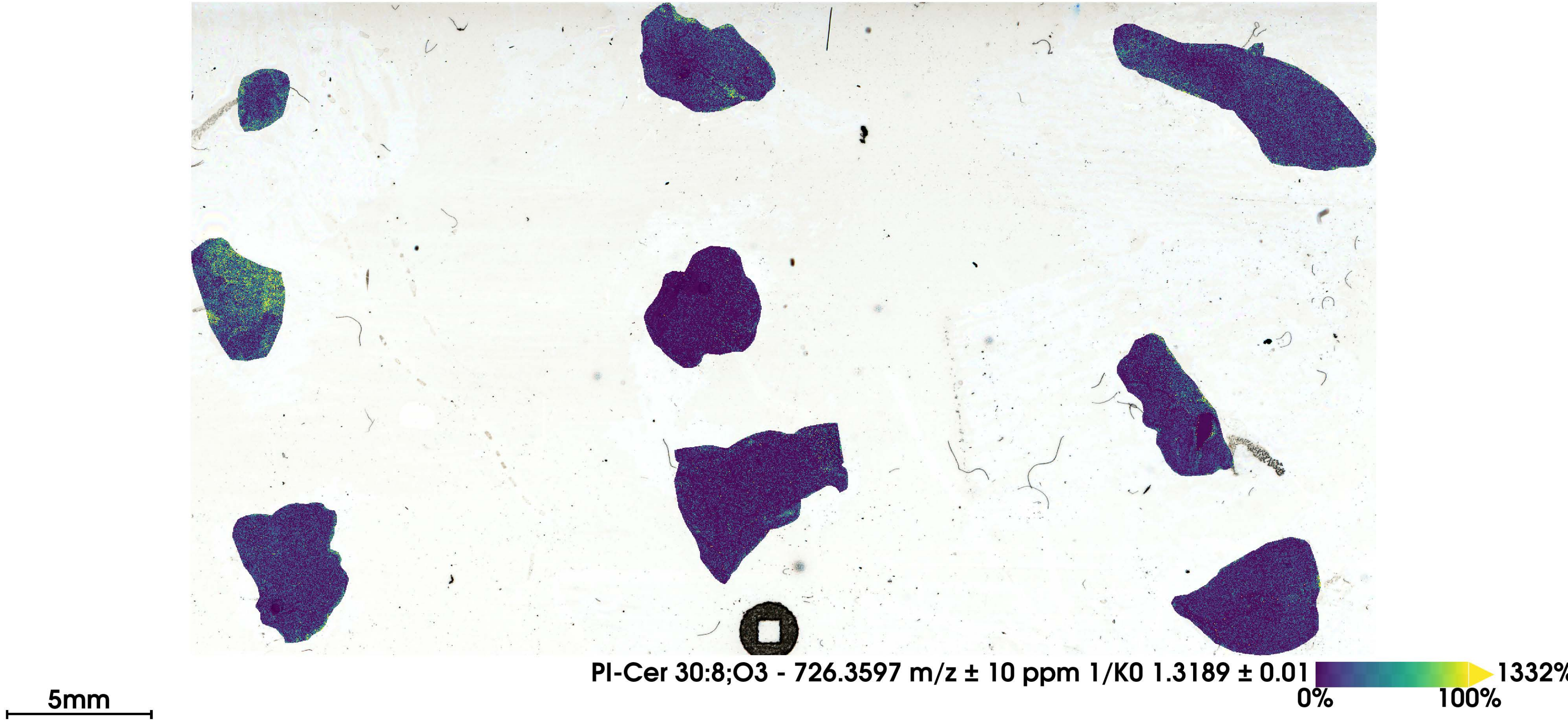
PI-Cer 30:8;O3 - 726.3597 m/z ± 10 ppm 1/K0 1.3189 ± 0.01
1332%
0%
100%
5mm

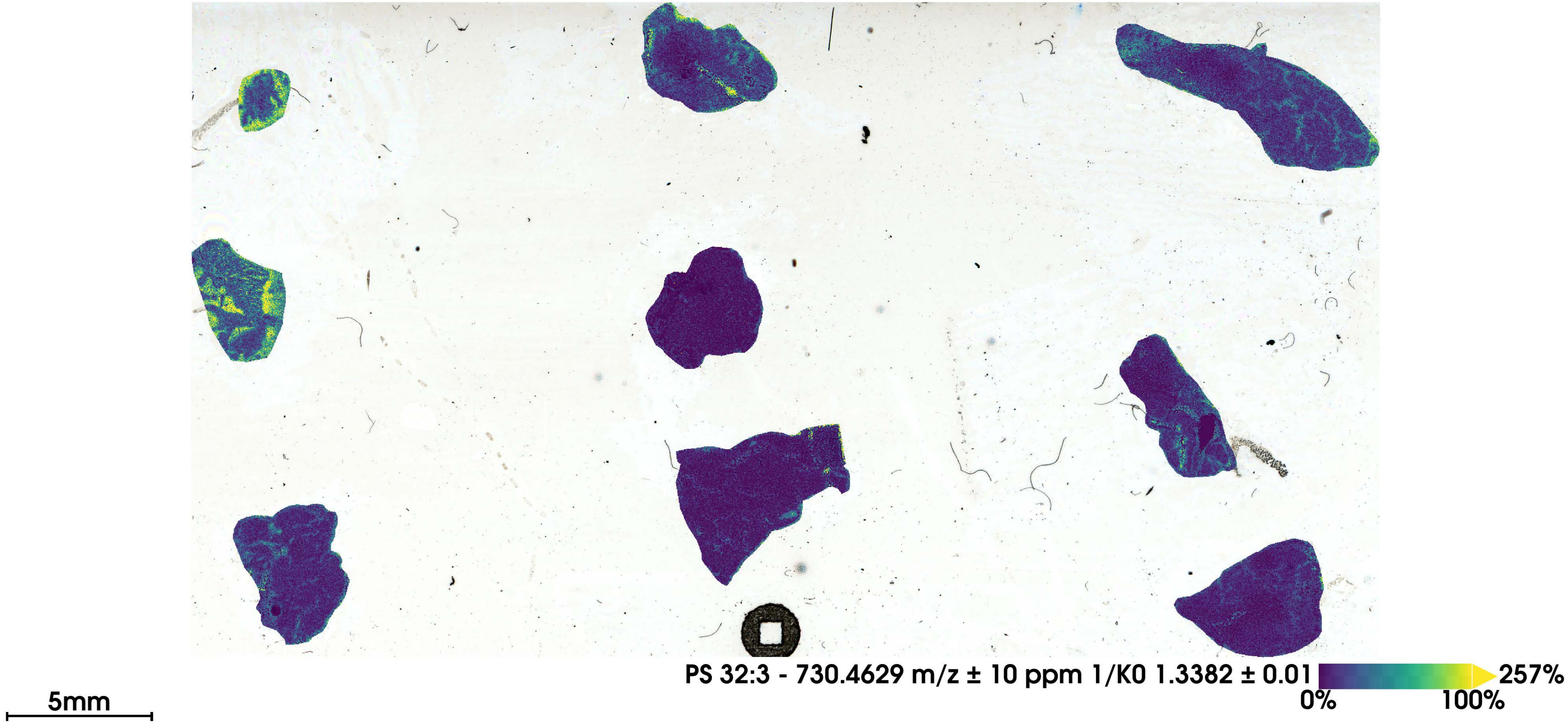

PS 32:3 - 730.4629 m/z ± 10 ppm 1/K0 1.3382 ± 0.01
257%
0%
100%
5mm

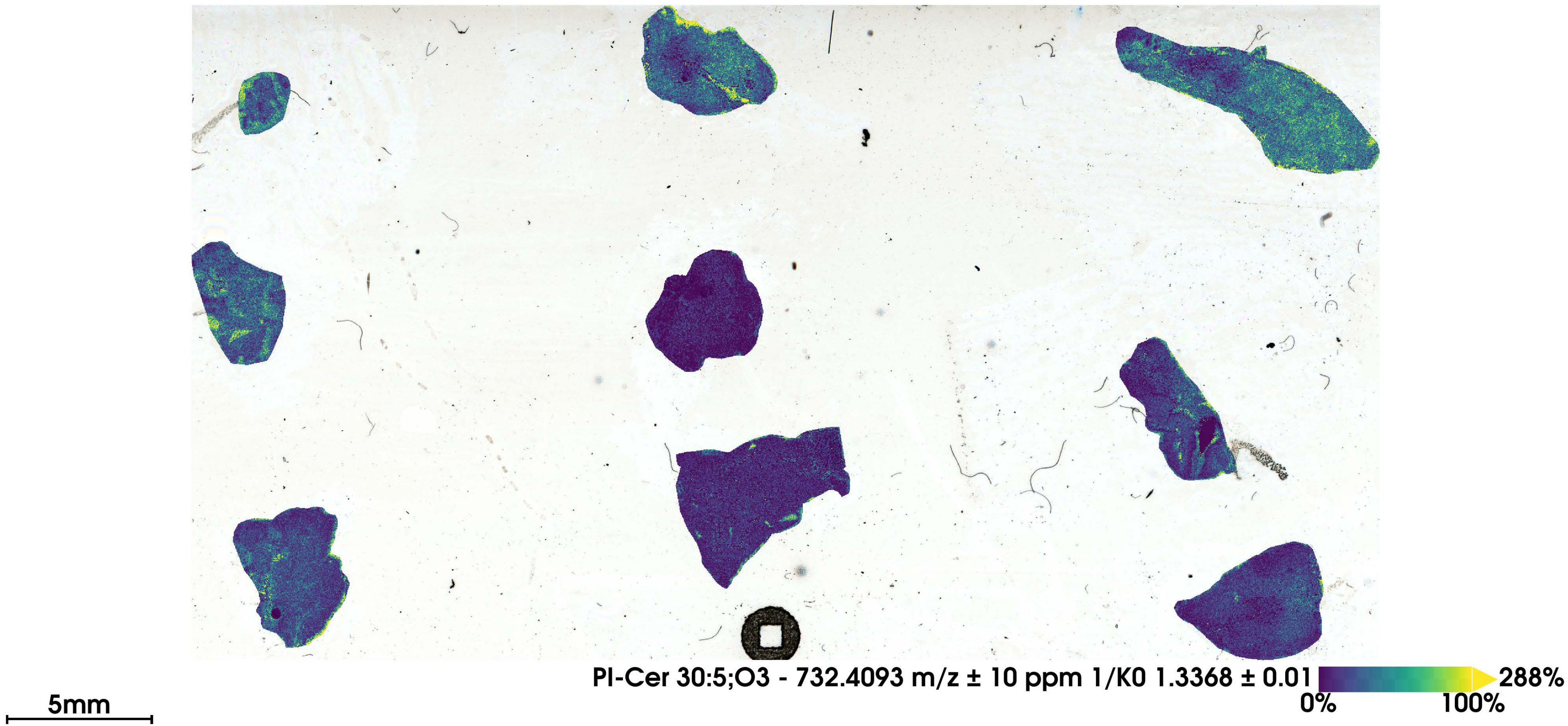
5mm
PI-Cer 30:5;O3 - 732.4093 m/z ± 10 ppm 1/K0 1.3368 ± 0.01
0%
100%
288%

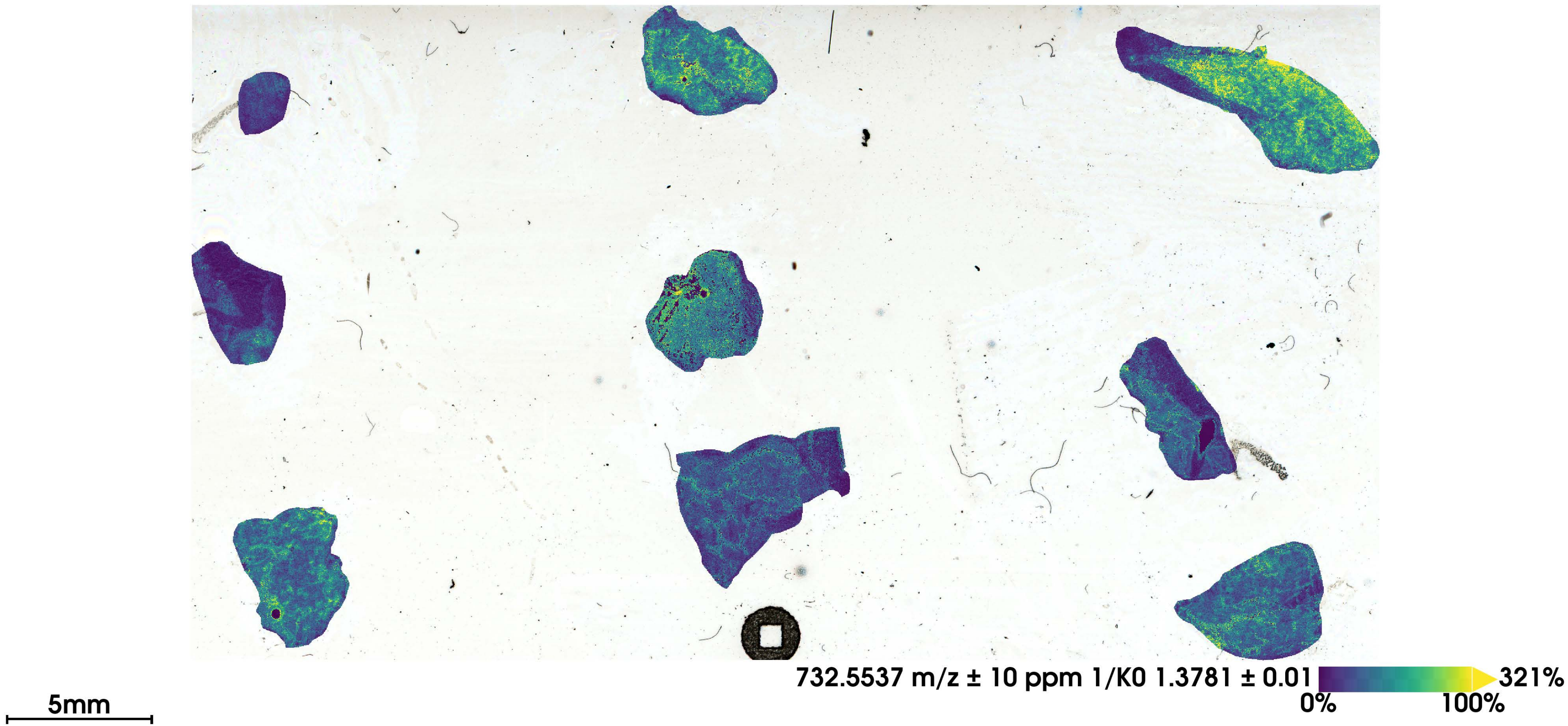
732.5537 m/z ± 10 ppm 1/K0 1.3781 ± 0.01
0%
100%
321%
5mm

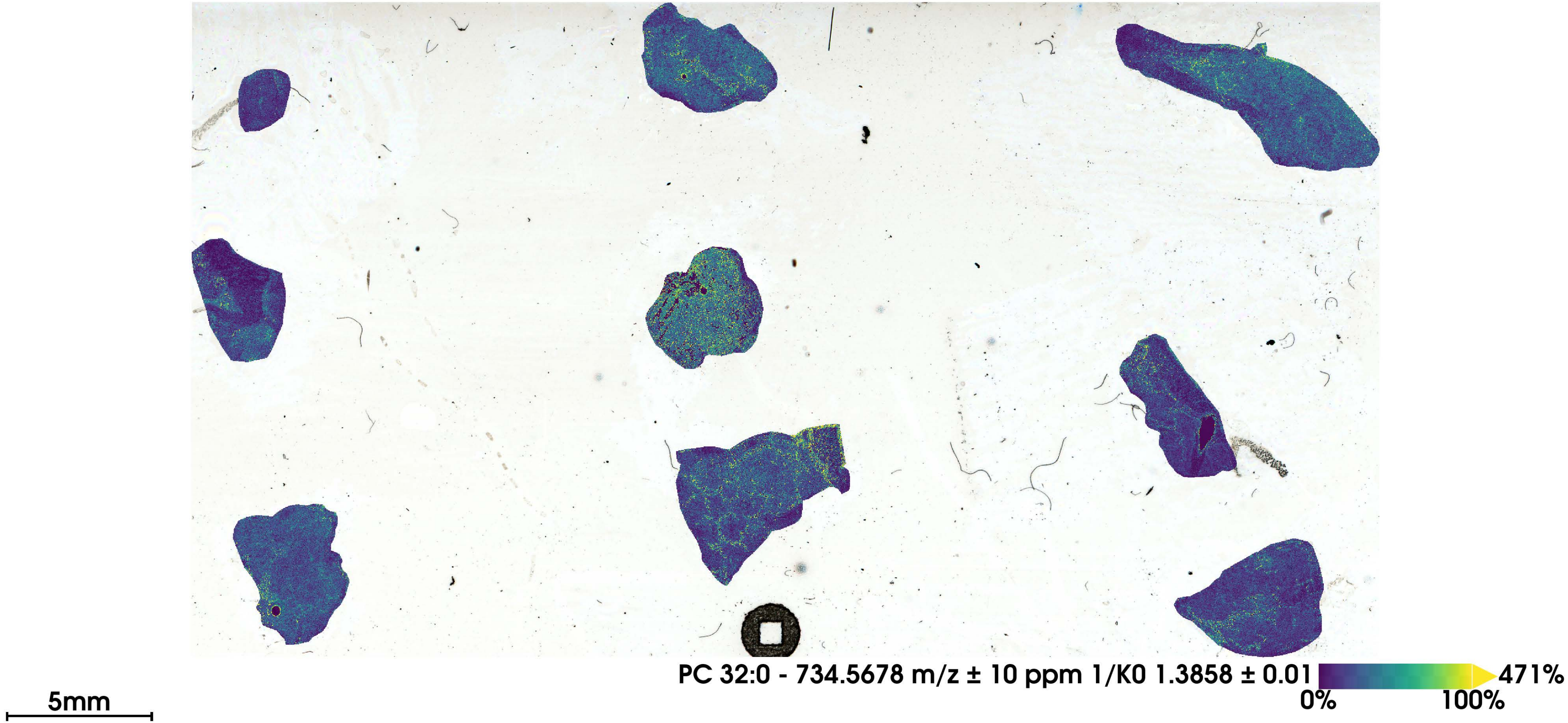

PC 32:0 - 734.5678 m/z ± 10 ppm 1/K0 1.3858 ± 0.01
471%
0%
100%
5mm

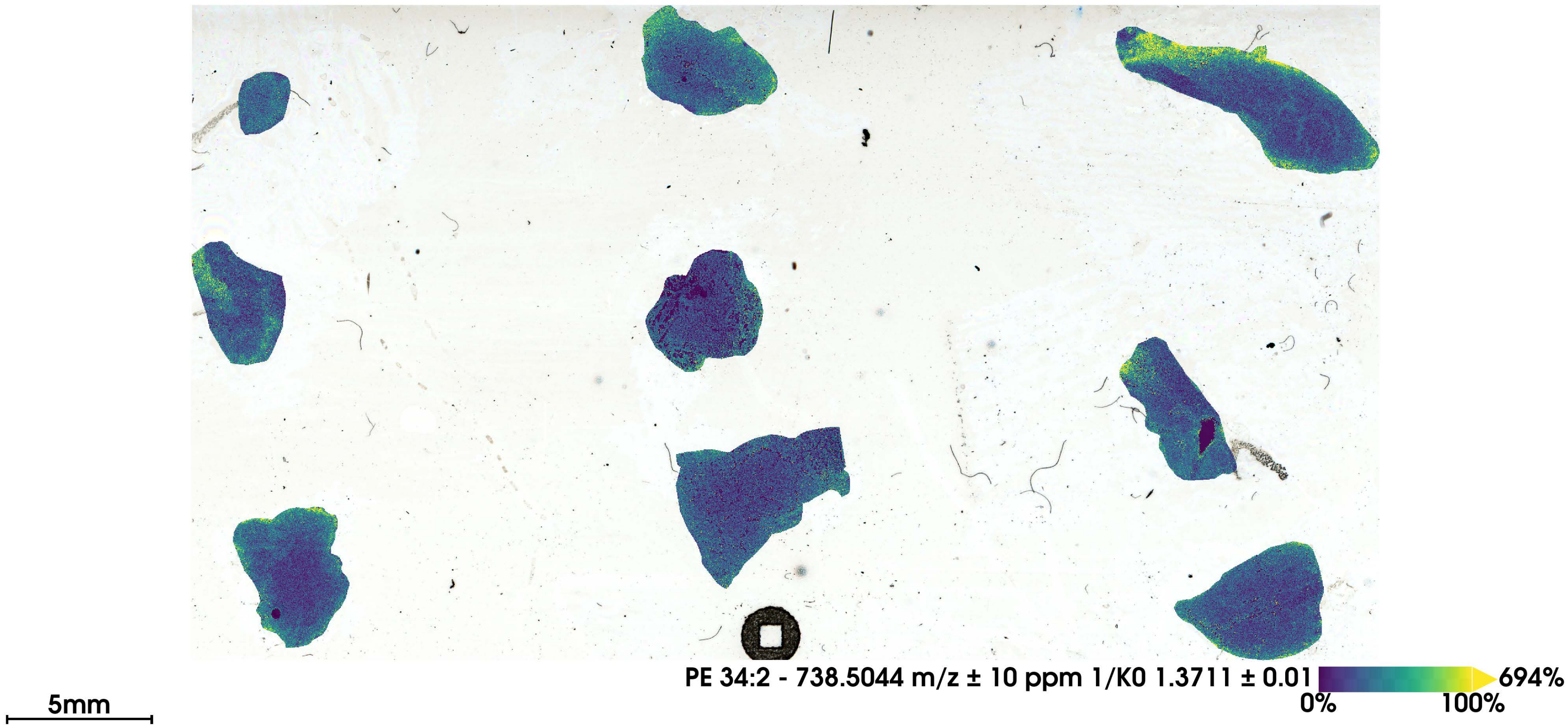
PE 34:2 - 738.5044 m/z ± 10 ppm 1/K0 1.3711 ± 0.01
694%
0%
100%
5mm

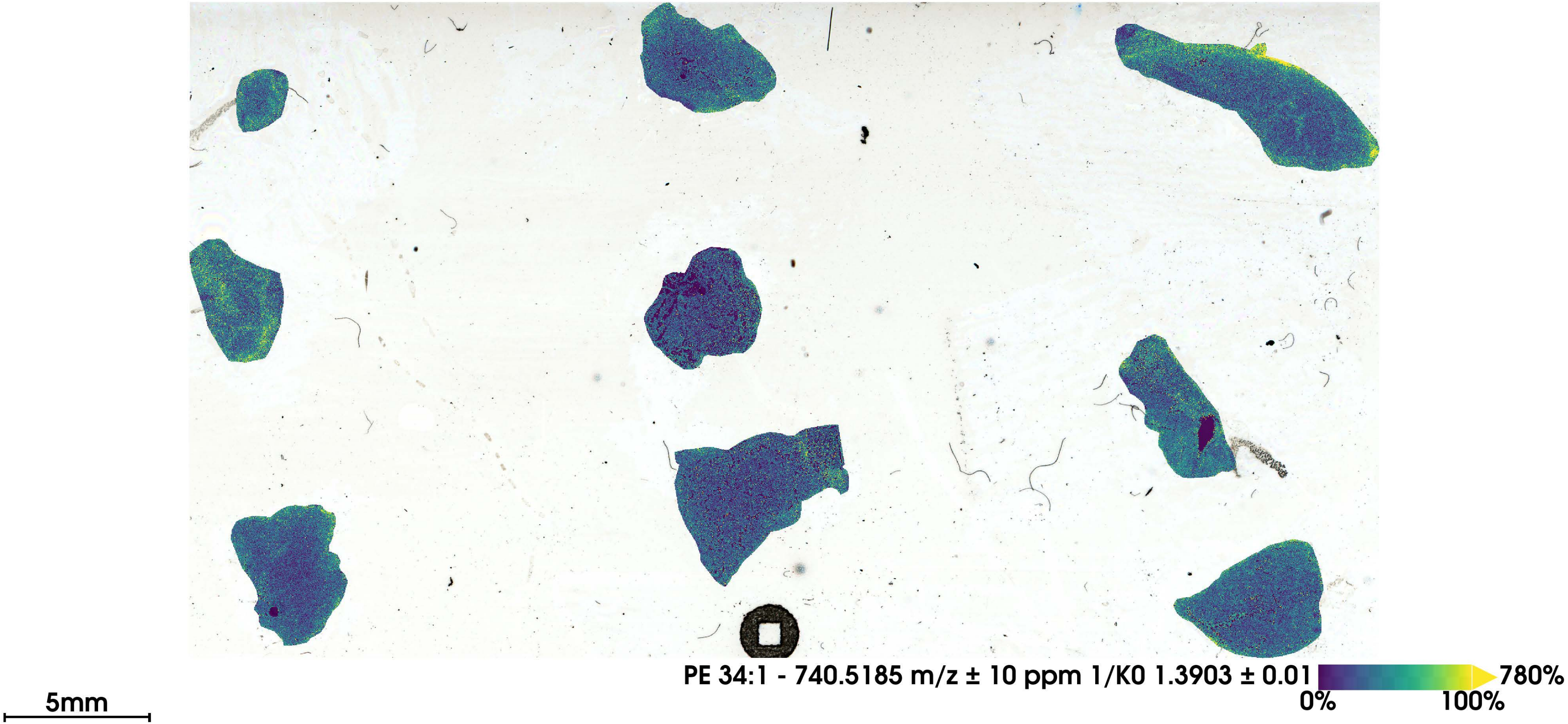
5mm
PE 34:1 - 740.5185 m/z ± 10 ppm 1/K0 1.3903 ± 0.01
0%
100%
780%

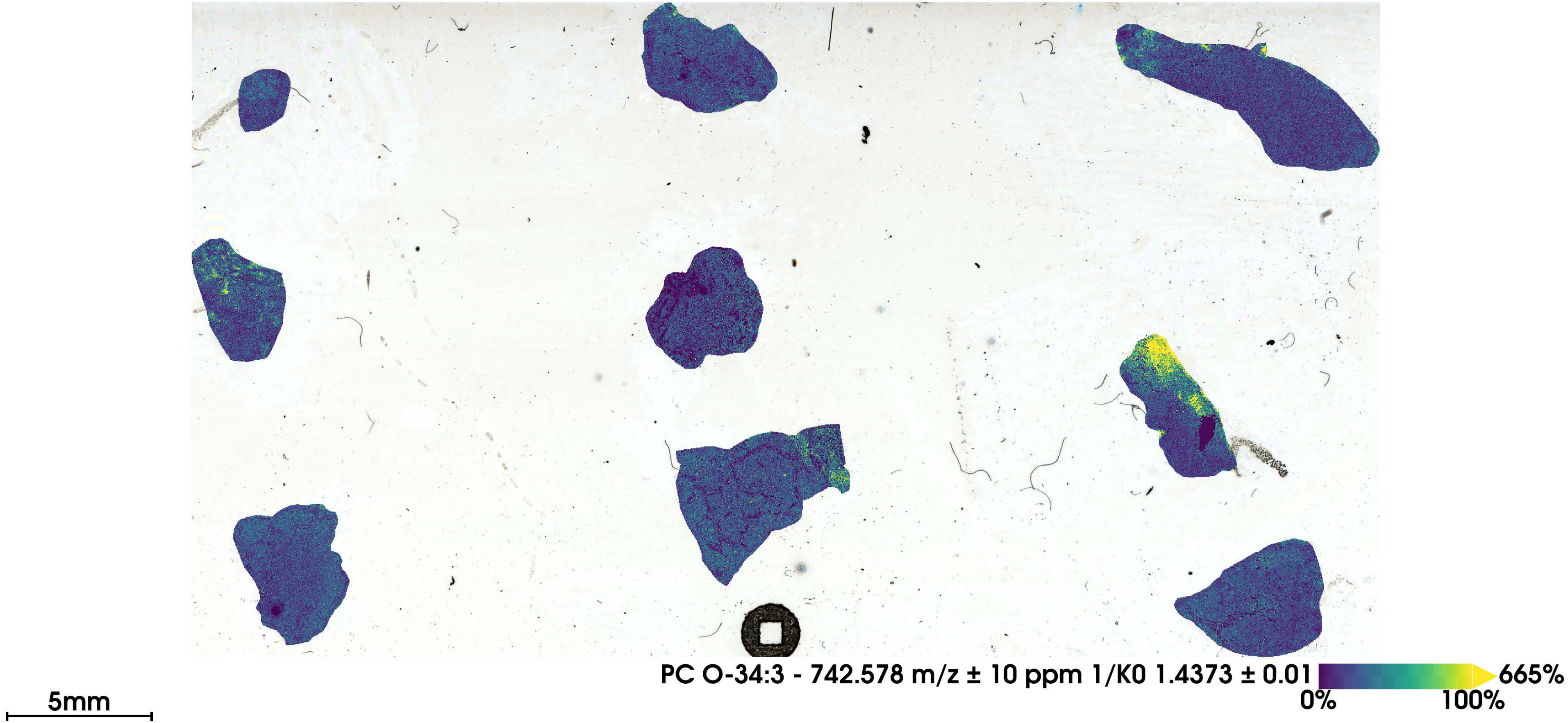
PC O-34:3 - 742.578 m/z ± 10 ppm 1/K0 1.4373 ± 0.01
665%
0%
100%
5mm

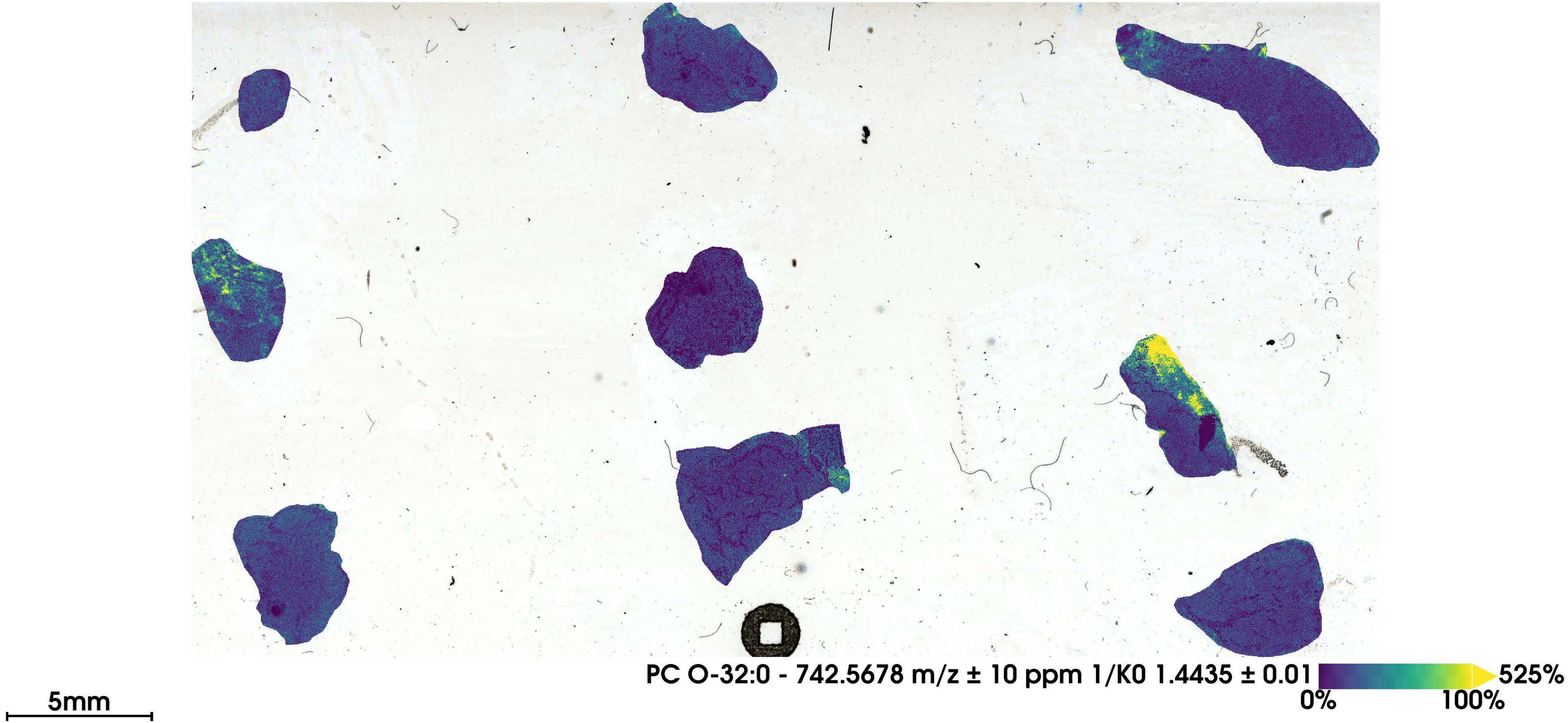
PC O-32:0 - 742.5678 m/z ± 10 ppm 1/K0 1.4435 ± 0.01
525%
0%
100%
5mm

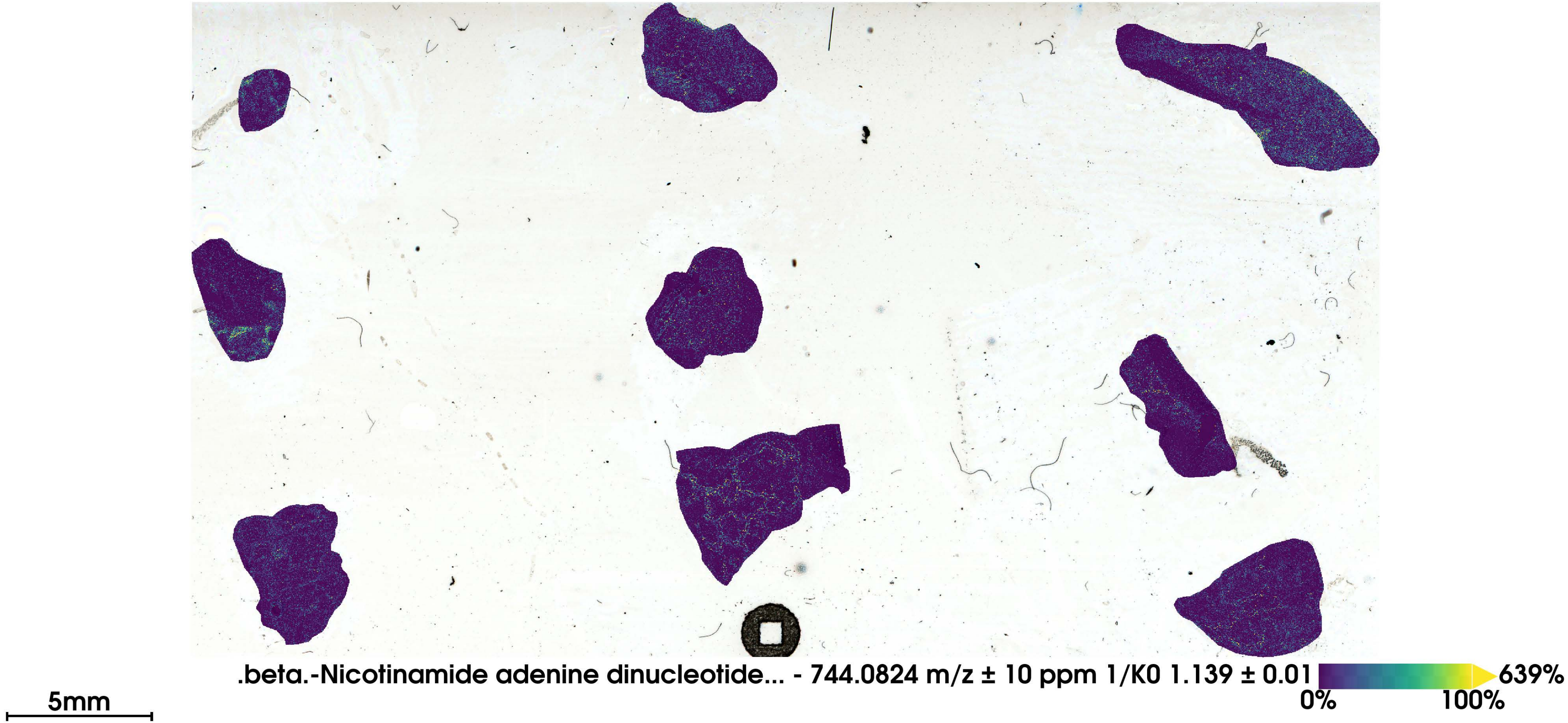

.beta.-Nicotinamide adenine dinucleotide... - 744.0824 m/z ± 10 ppm 1/K0 1.139 ± 0.01
639%
0%
100%
5mm

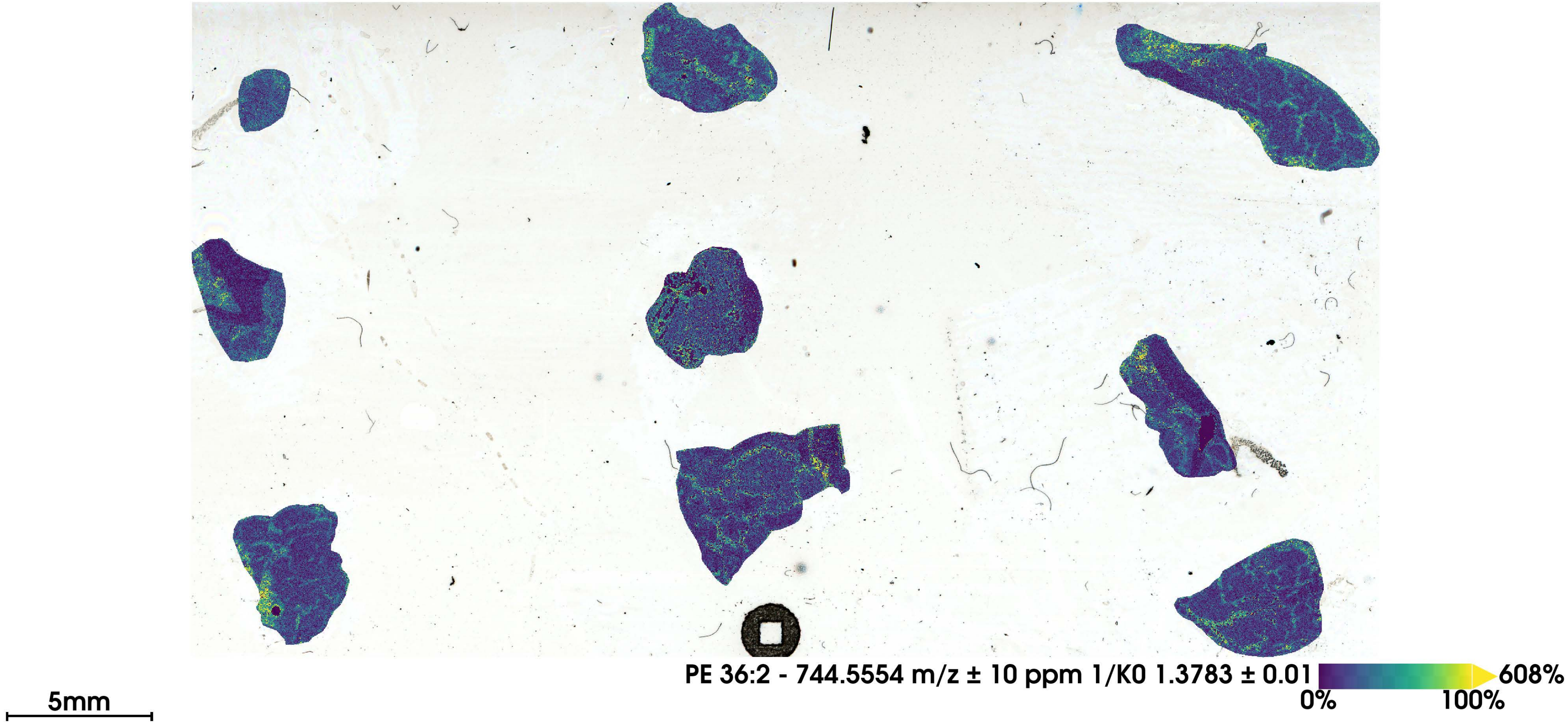
PE 36:2 - 744.5554 m/z ± 10 ppm 1/K0 1.3783 ± 0.01
608%
0%
100%
5mm

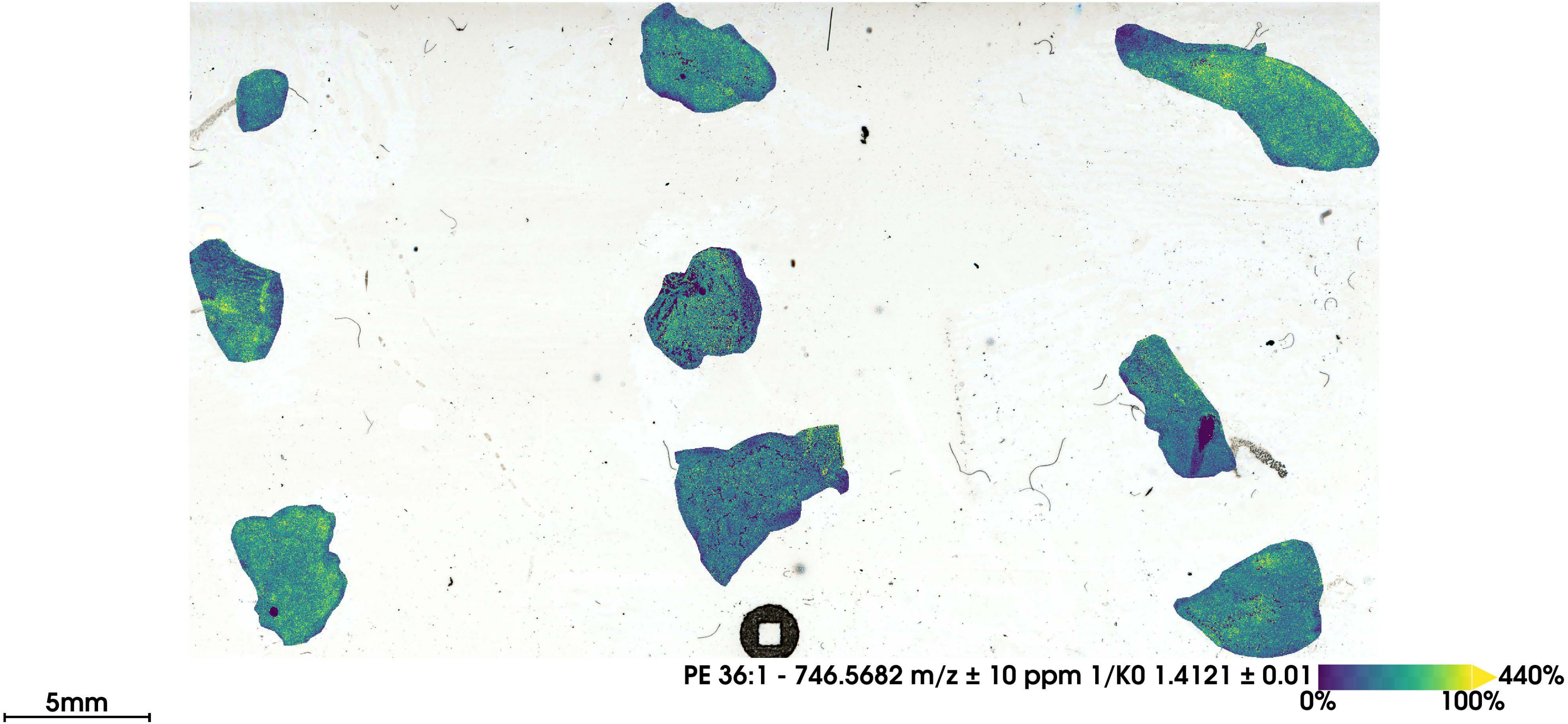

5mm
PE 36:1 - 746.5682 m/z ± 10 ppm 1/K0 1.4121 ± 0.01
440%
0%
100%

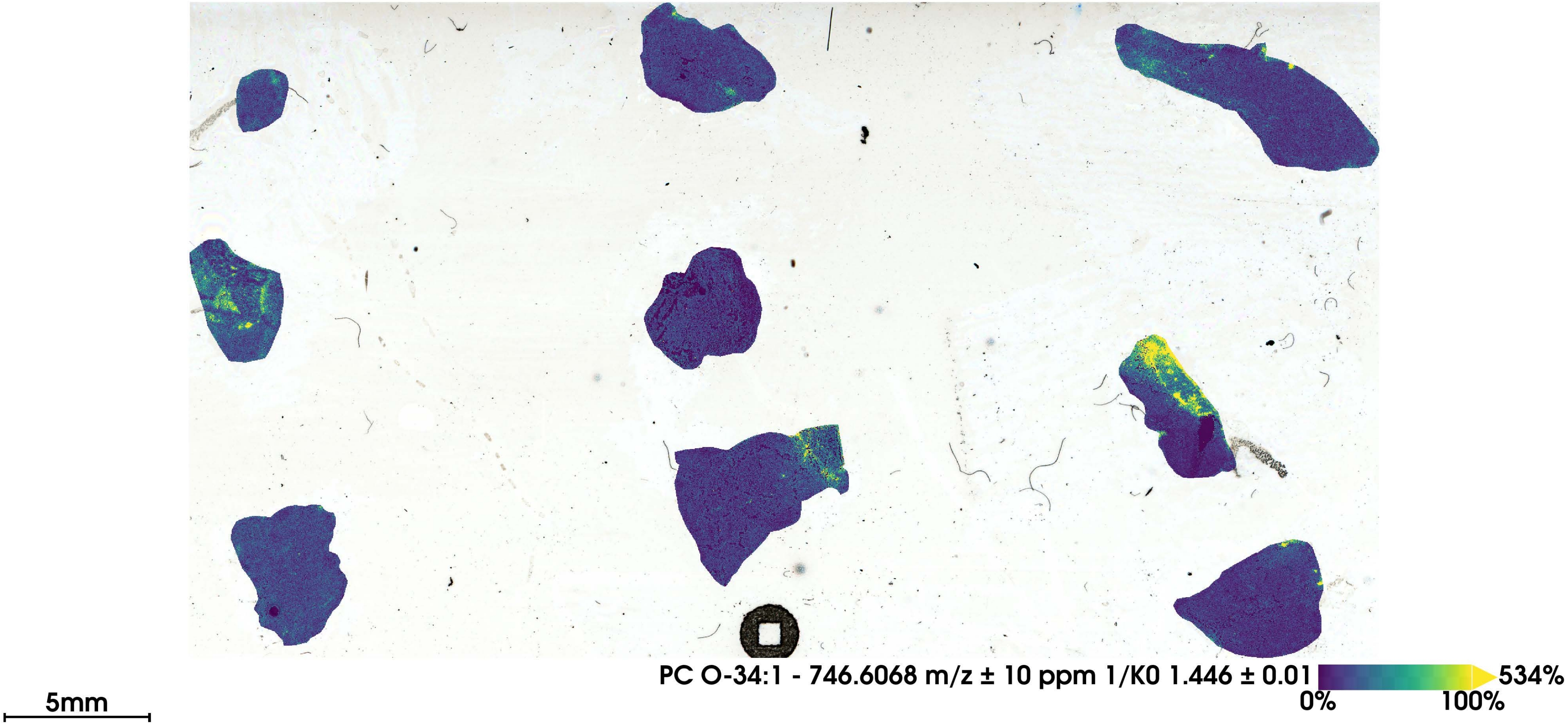

PC O-34:1 - 746.6068 m/z ± 10 ppm 1/K0 1.446 ± 0.01
534%
0%
100%
5mm

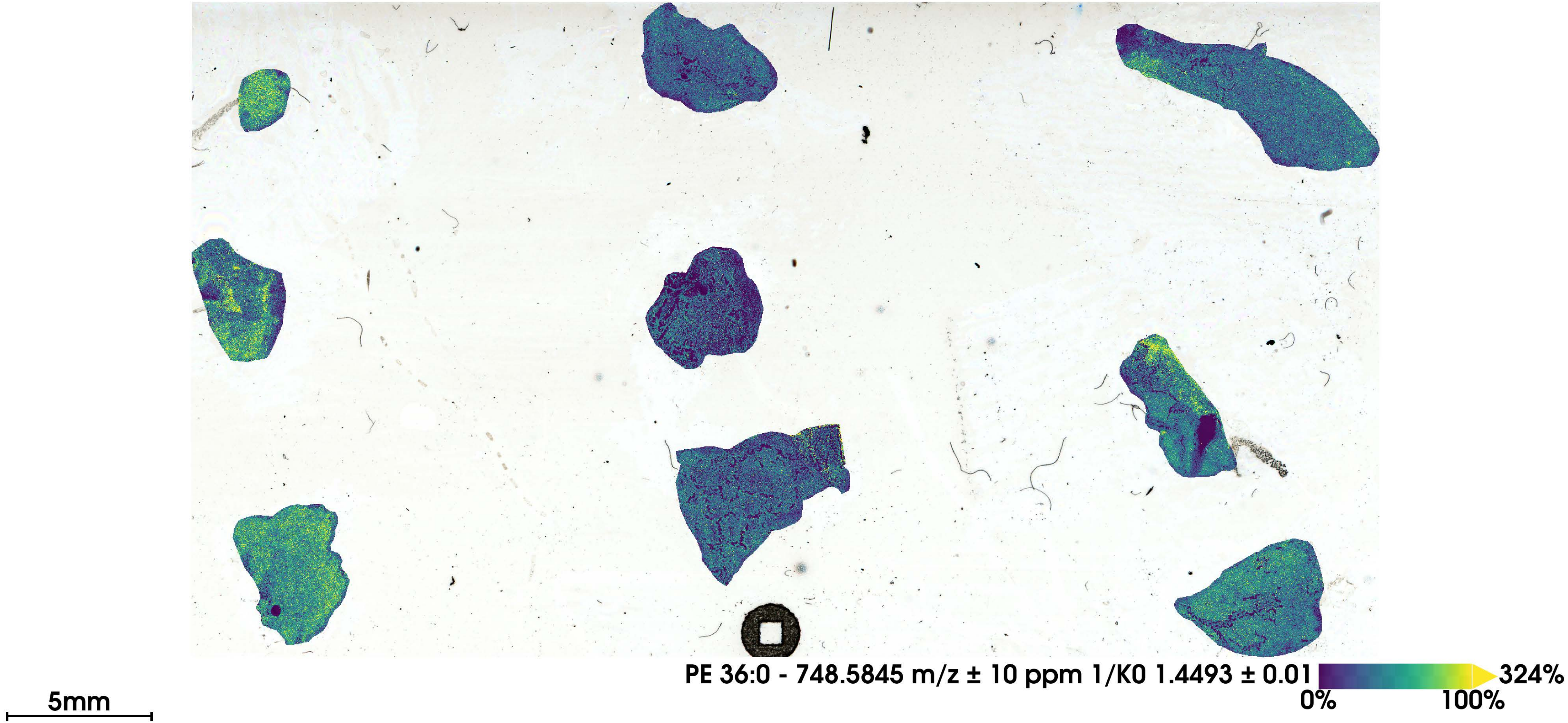

PE 36:0 - 748.5845 m/z ± 10 ppm 1/K0 1.4493 ± 0.01
324%
0%
100%
5mm

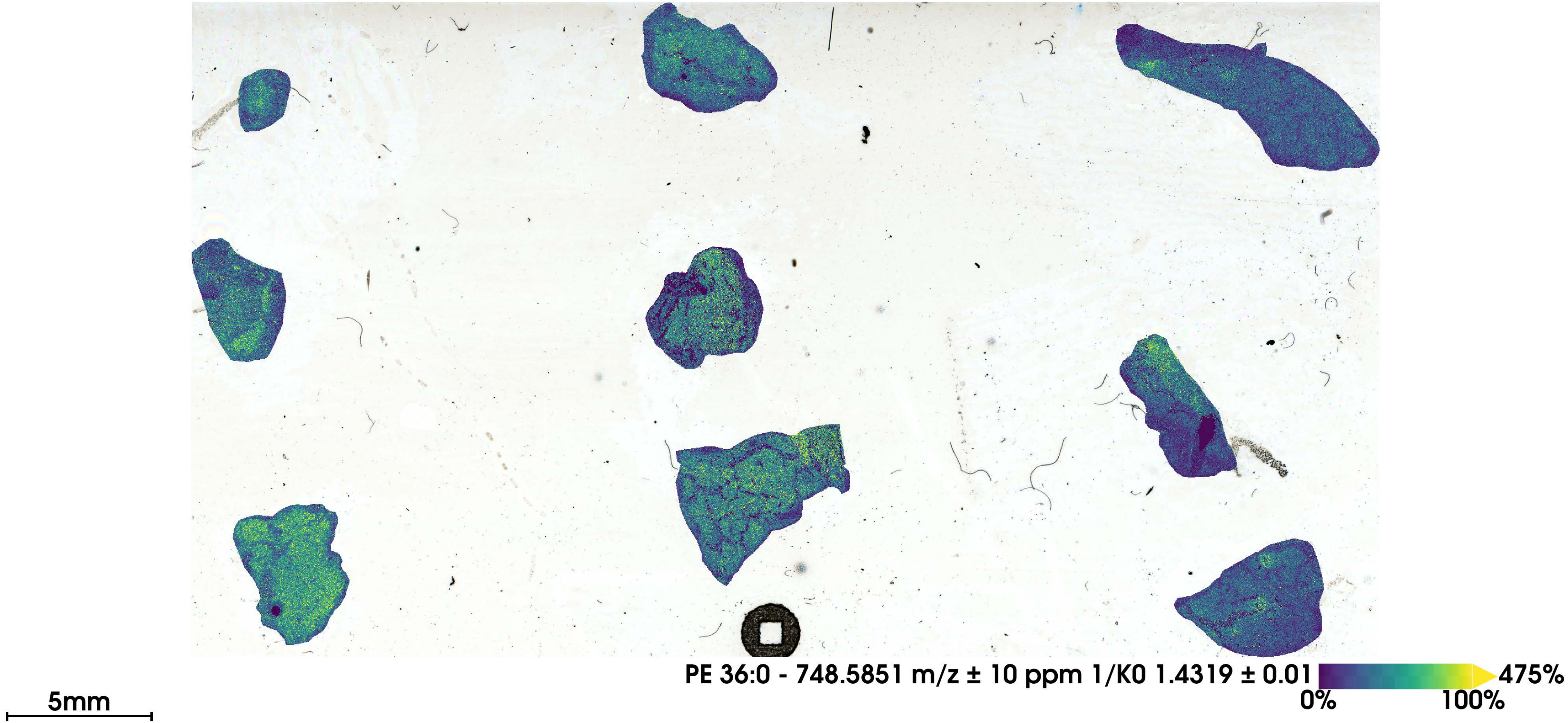
PE 36:0 - 748.5851 m/z ± 10 ppm 1/K0 1.4319 ± 0.01
475%
0%
100%
5mm

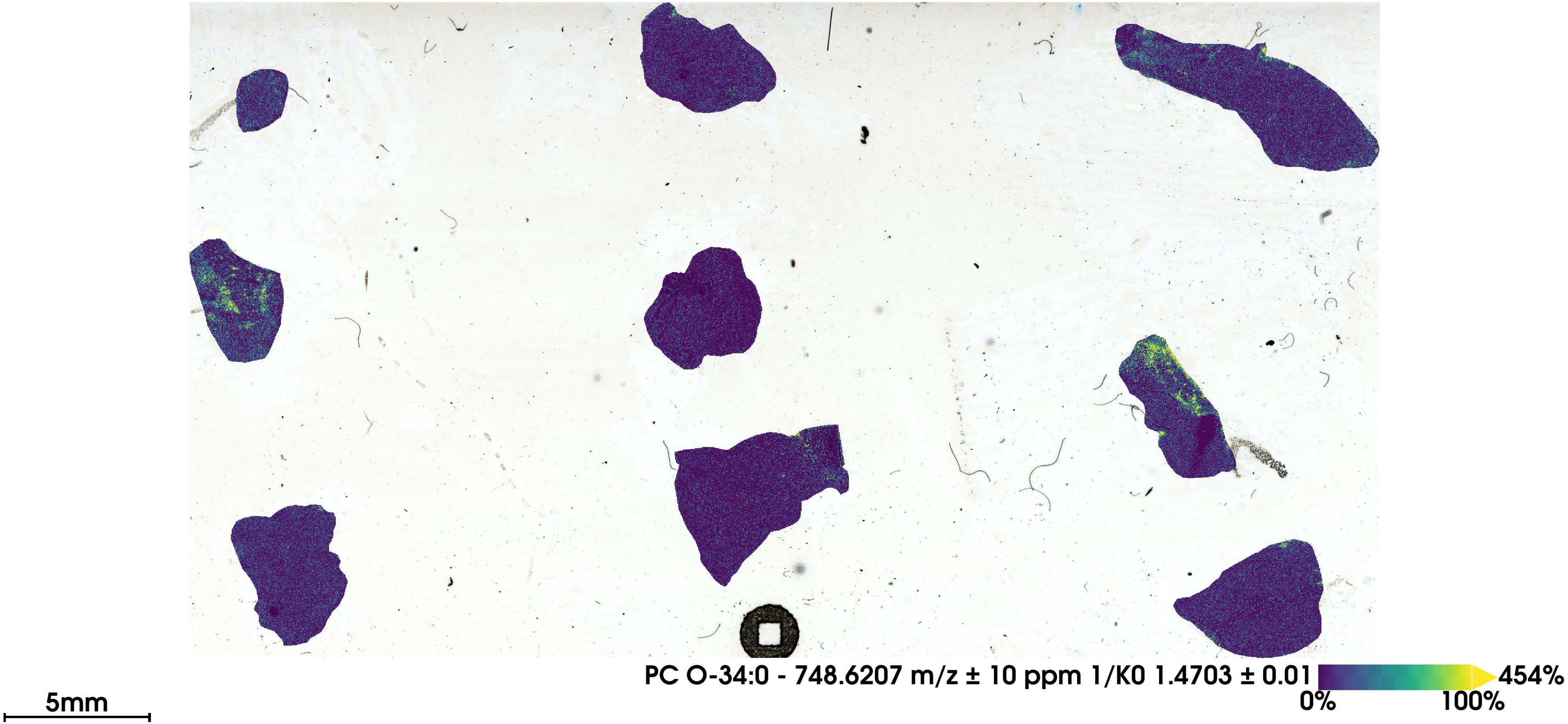
PC O-34:0 - 748.6207 m/z ± 10 ppm 1/K0 1.4703 ± 0.01
0%
100%
454%
5mm

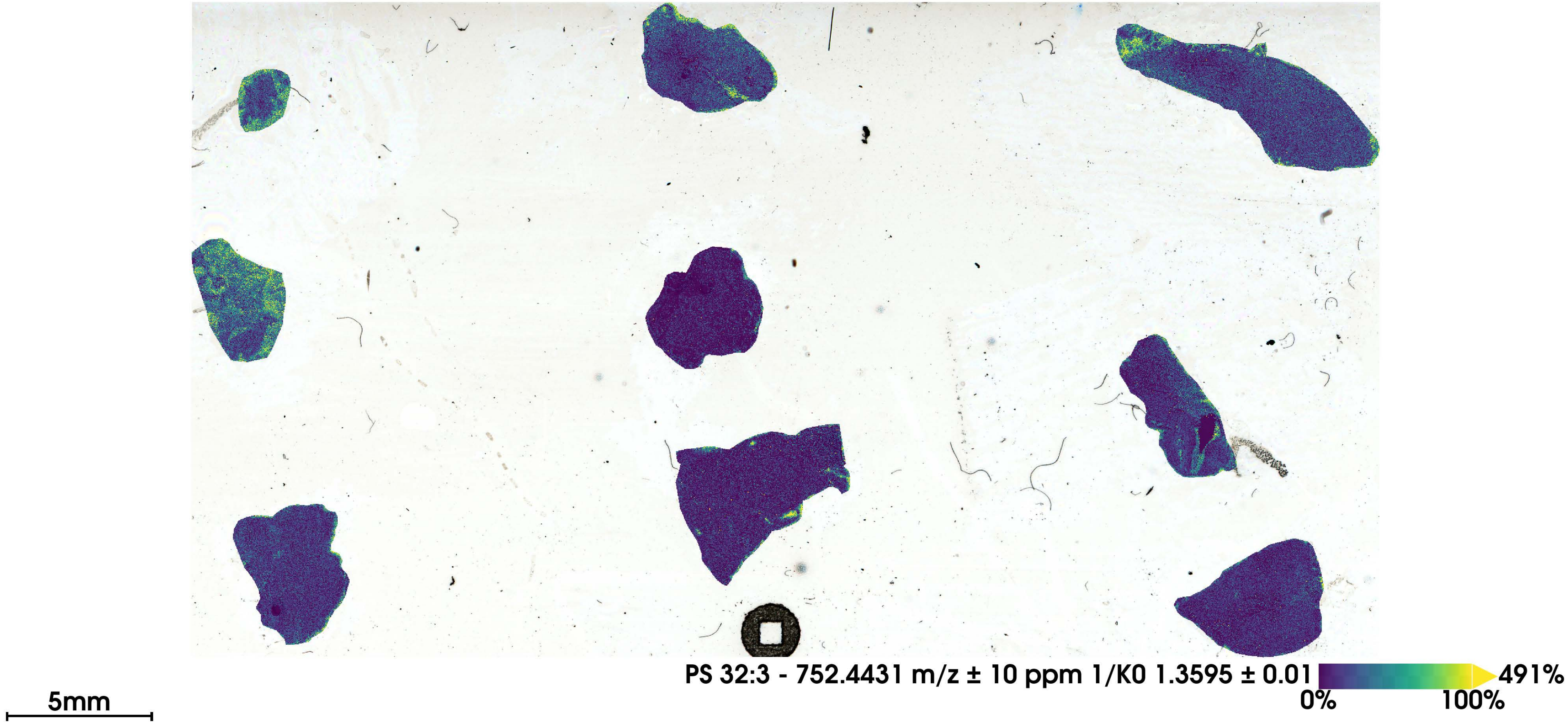

PS 32:3 - 752.4431 m/z ± 10 ppm 1/K0 1.3595 ± 0.01
491%
0%
100%
5mm

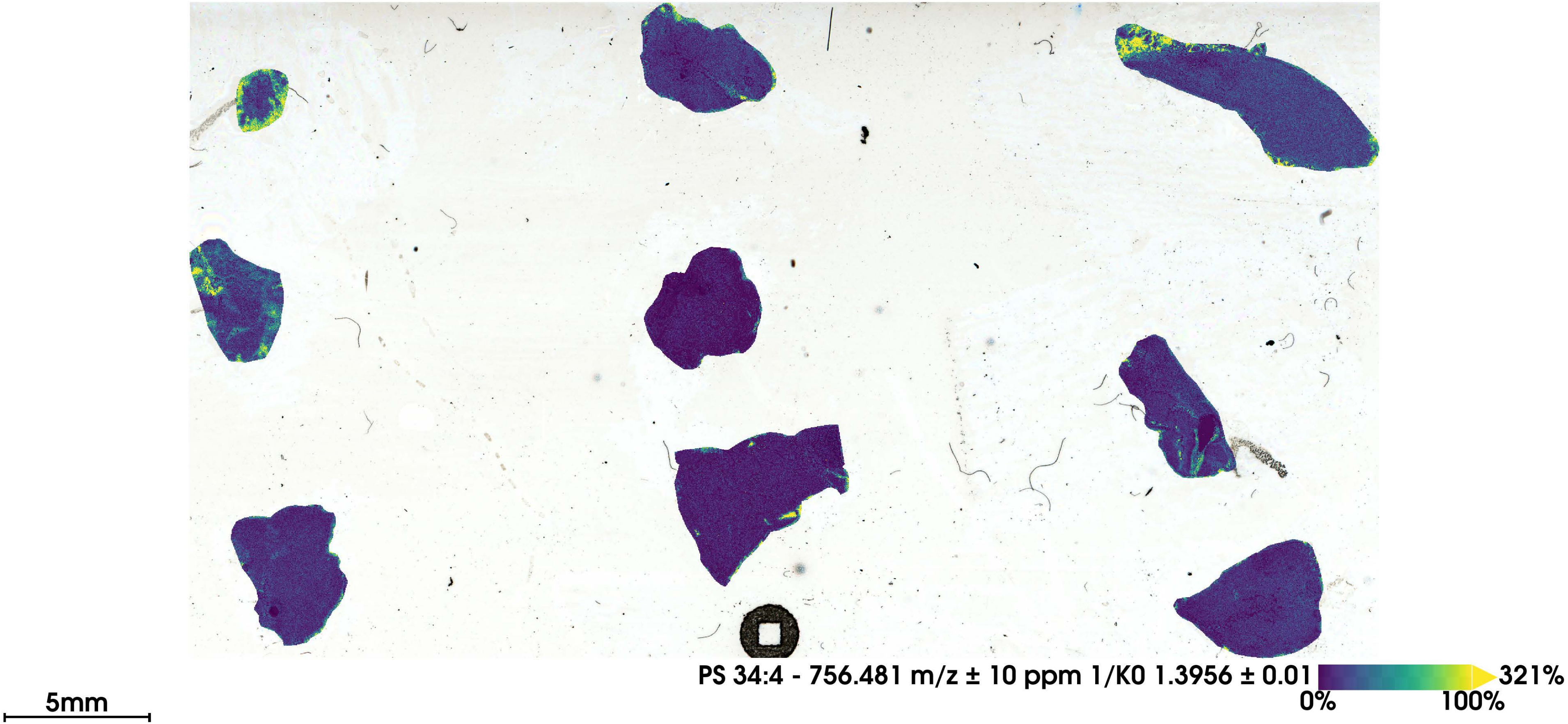

PS 34:4 - 756.481 m/z ± 10 ppm 1/K0 1.3956 ± 0.01
0%
100%
321%
5mm

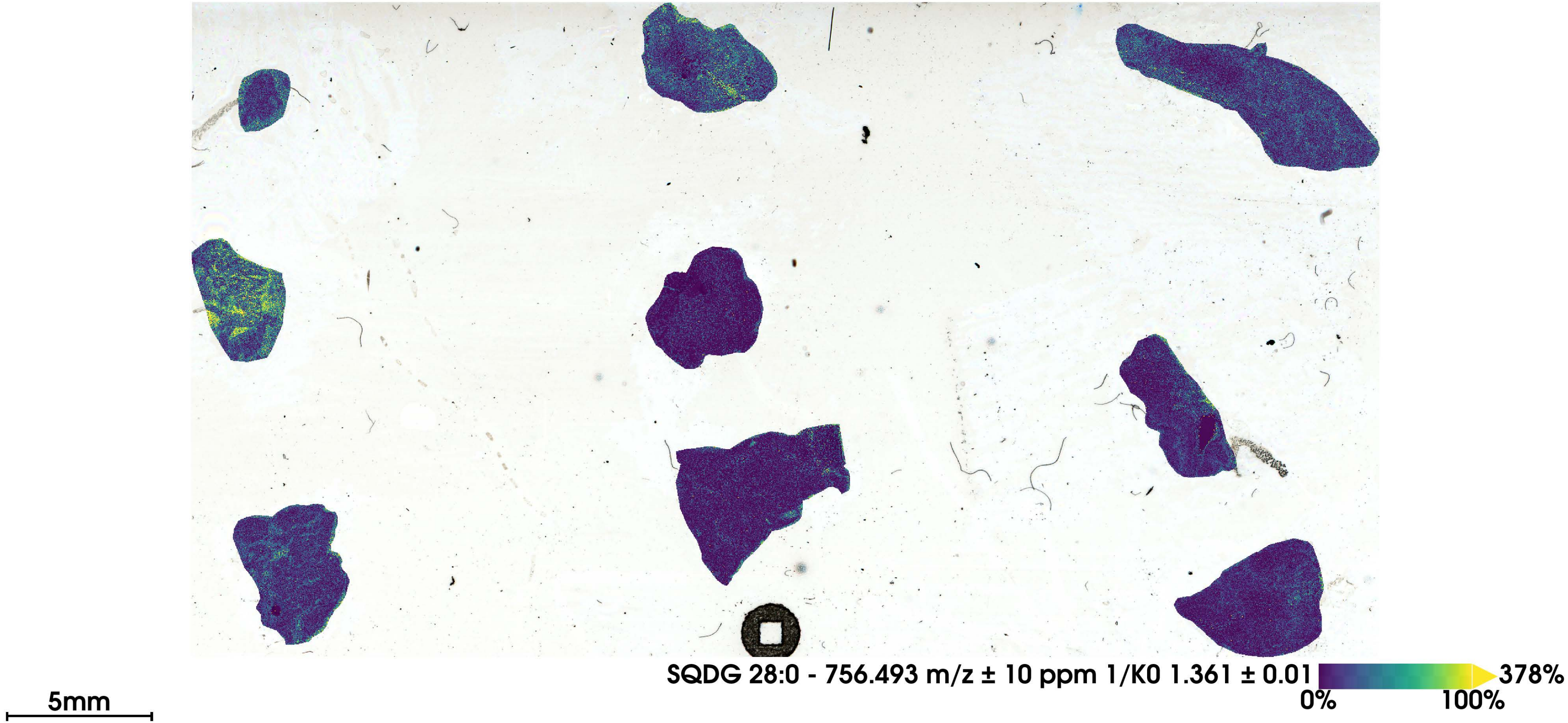

SQDG 28:0 - 756.493 m/z ± 10 ppm 1/K0 1.361 ± 0.01
378%
0%
100%
5mm

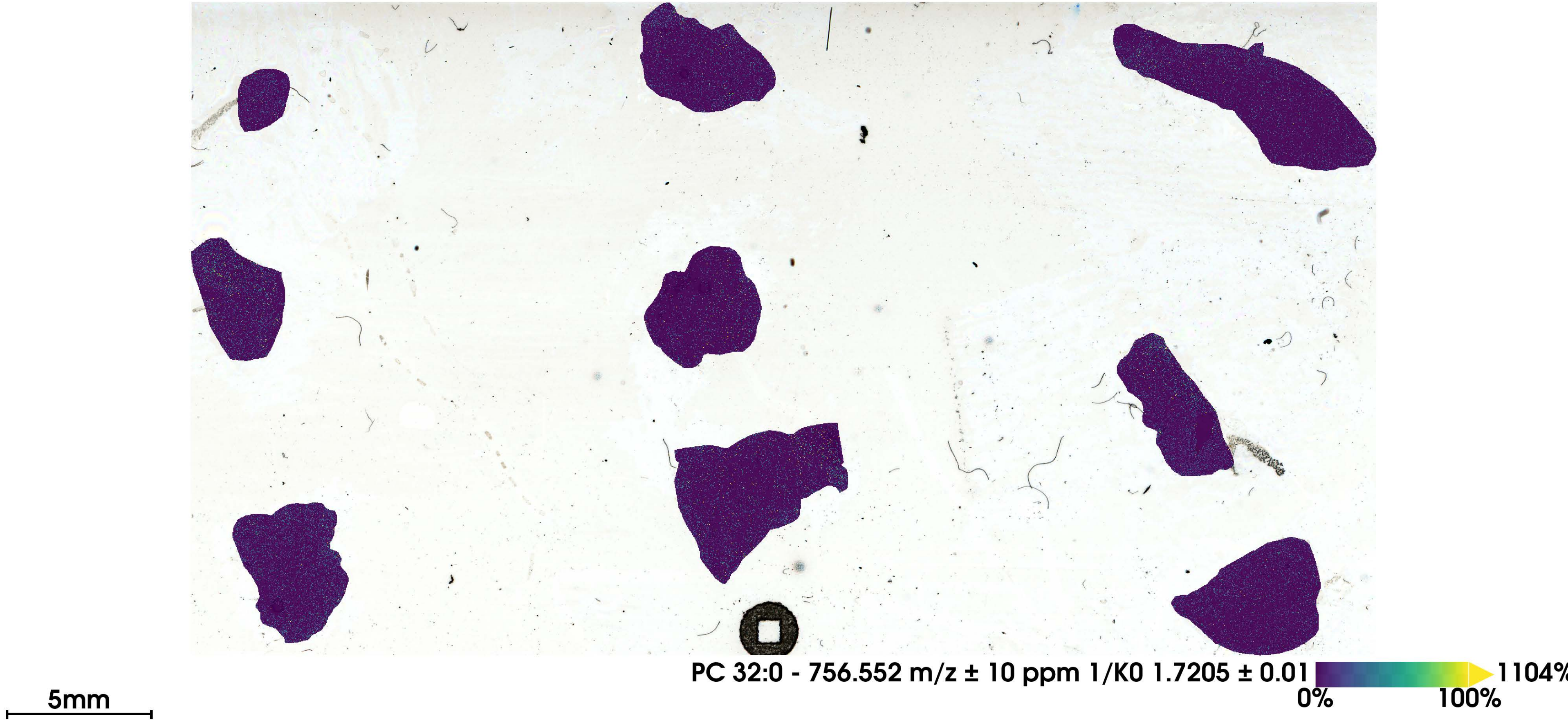
PC 32:0 - 756.552 m/z ± 10 ppm 1/K0 1.7205 ± 0.01
1104%
0%
100%
5mm

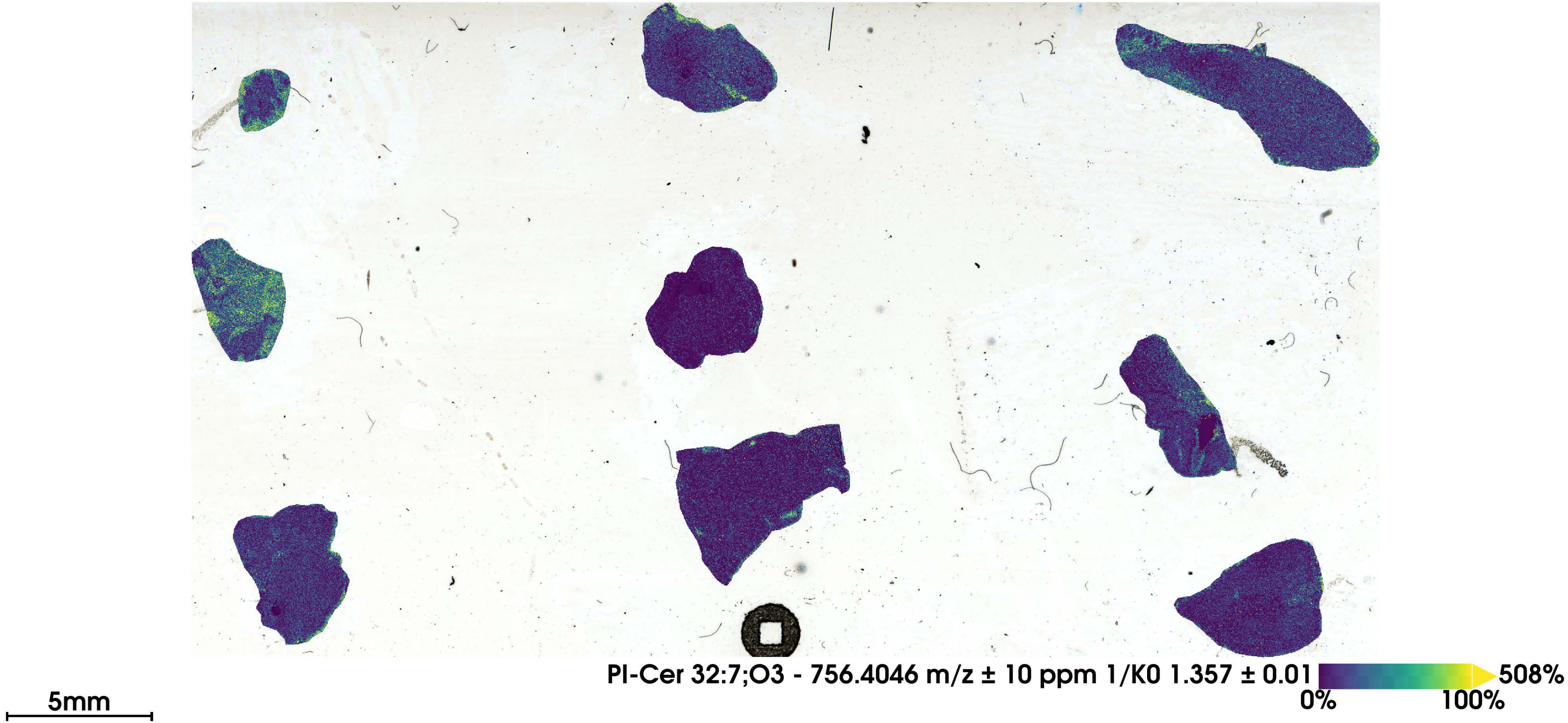
PI-Cer 32:7;O3 - 756.4046 m/z ± 10 ppm 1/K0 1.357 ± 0.01
508%
0%
100%
5mm

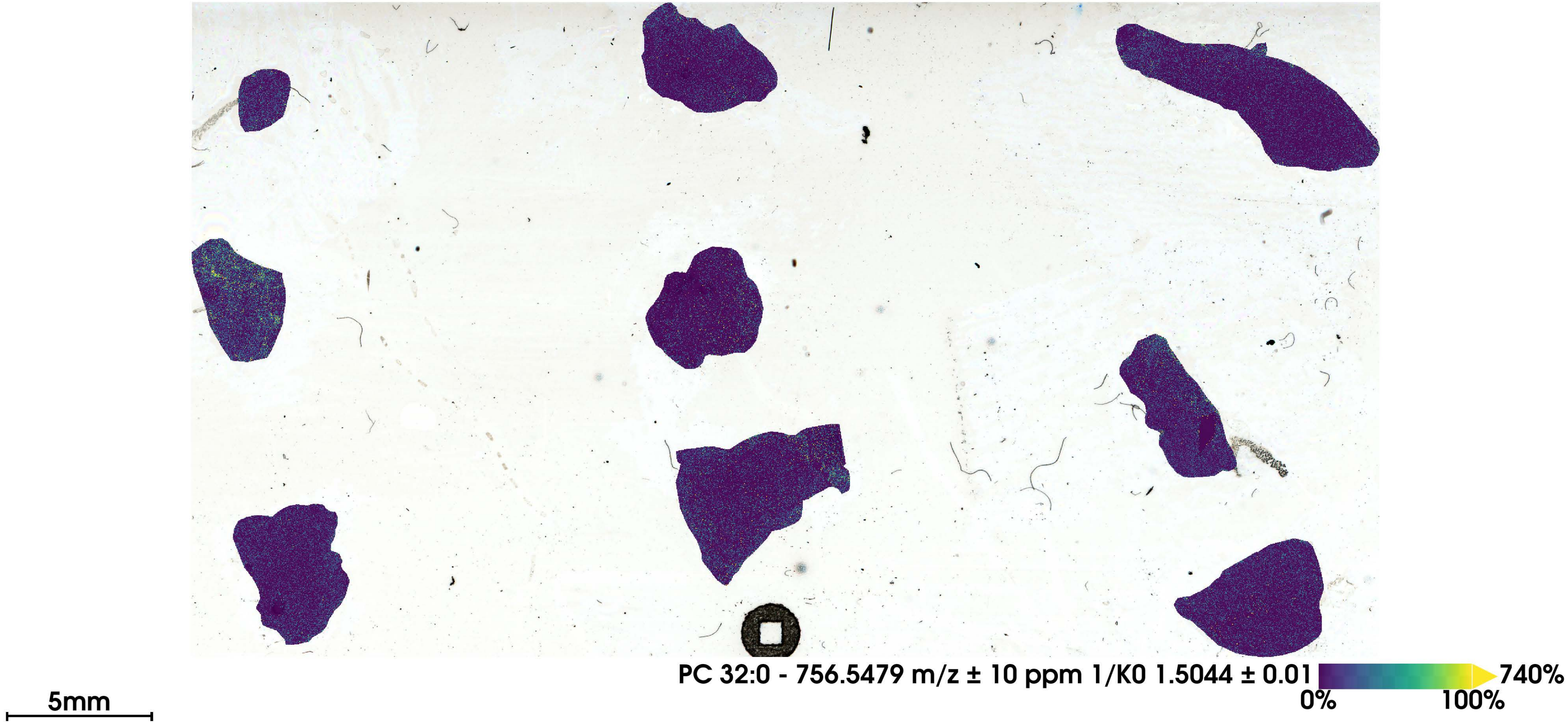

PC 32:0 - 756.5479 m/z ± 10 ppm 1/K0 1.5044 ± 0.01
740%
0%
100%
5mm

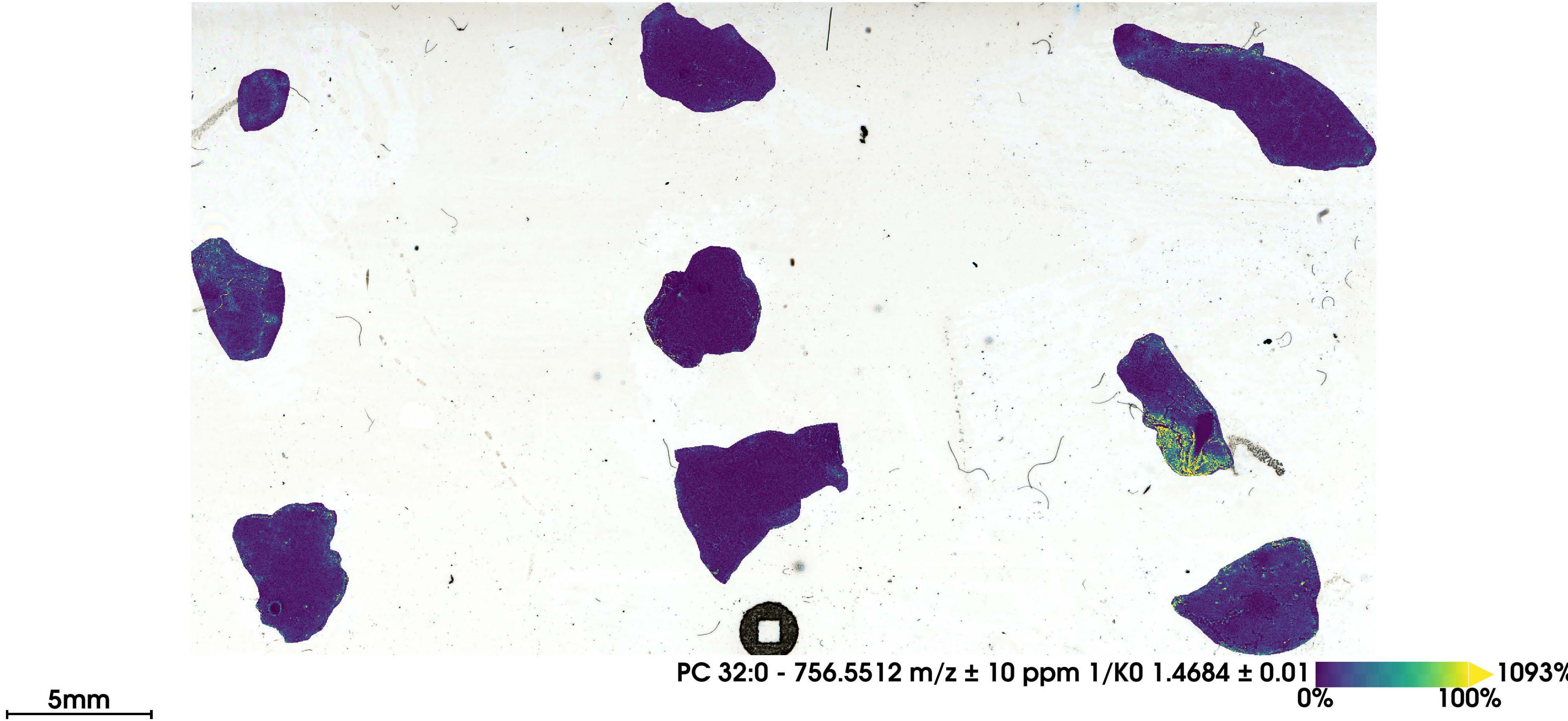
PC 32:0 - 756.5512 m/z ± 10 ppm 1/K0 1.4684 ± 0.01
1093%
0%
100%
5mm

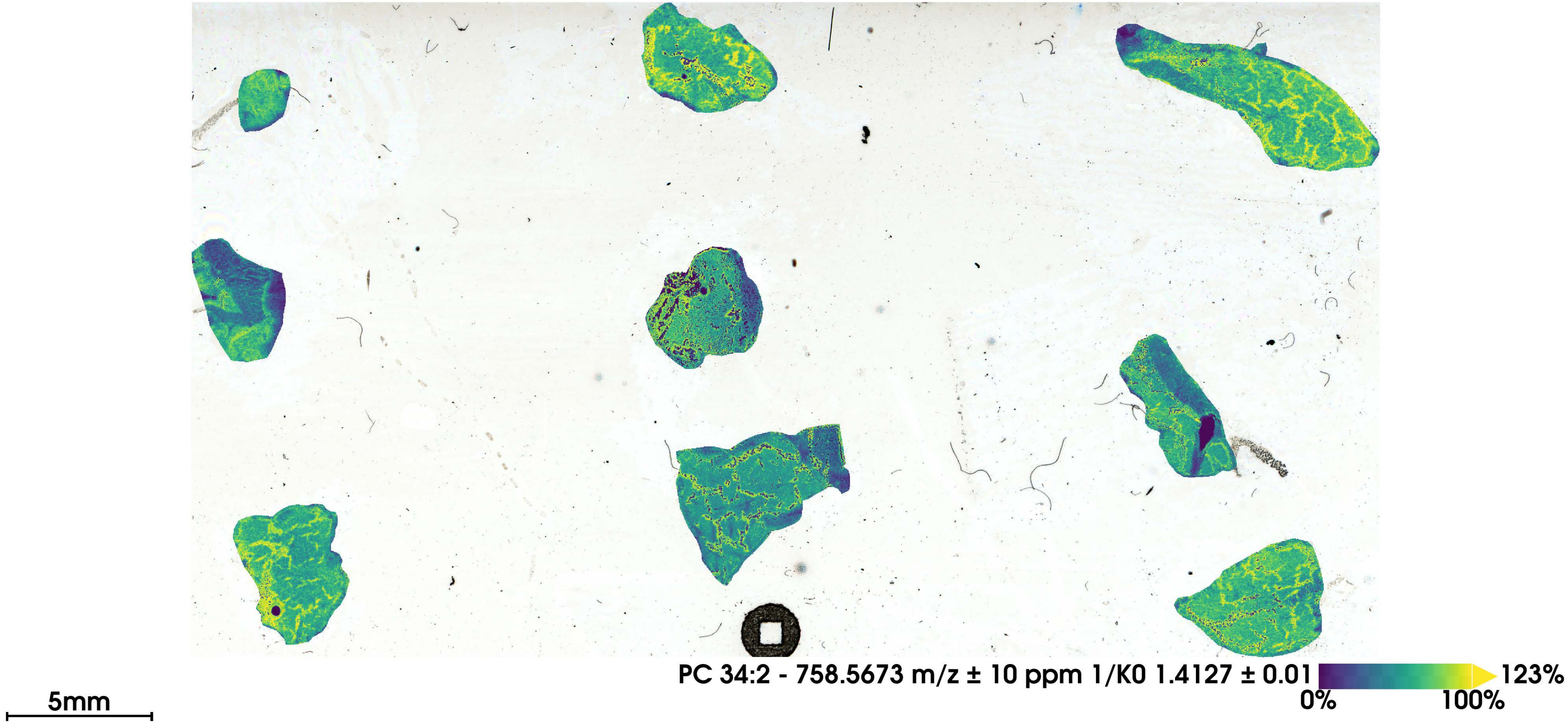
PC 34:2 - 758.5673 m/z ± 10 ppm 1/K0 1.4127 ± 0.01
0%
100%
123%
5mm

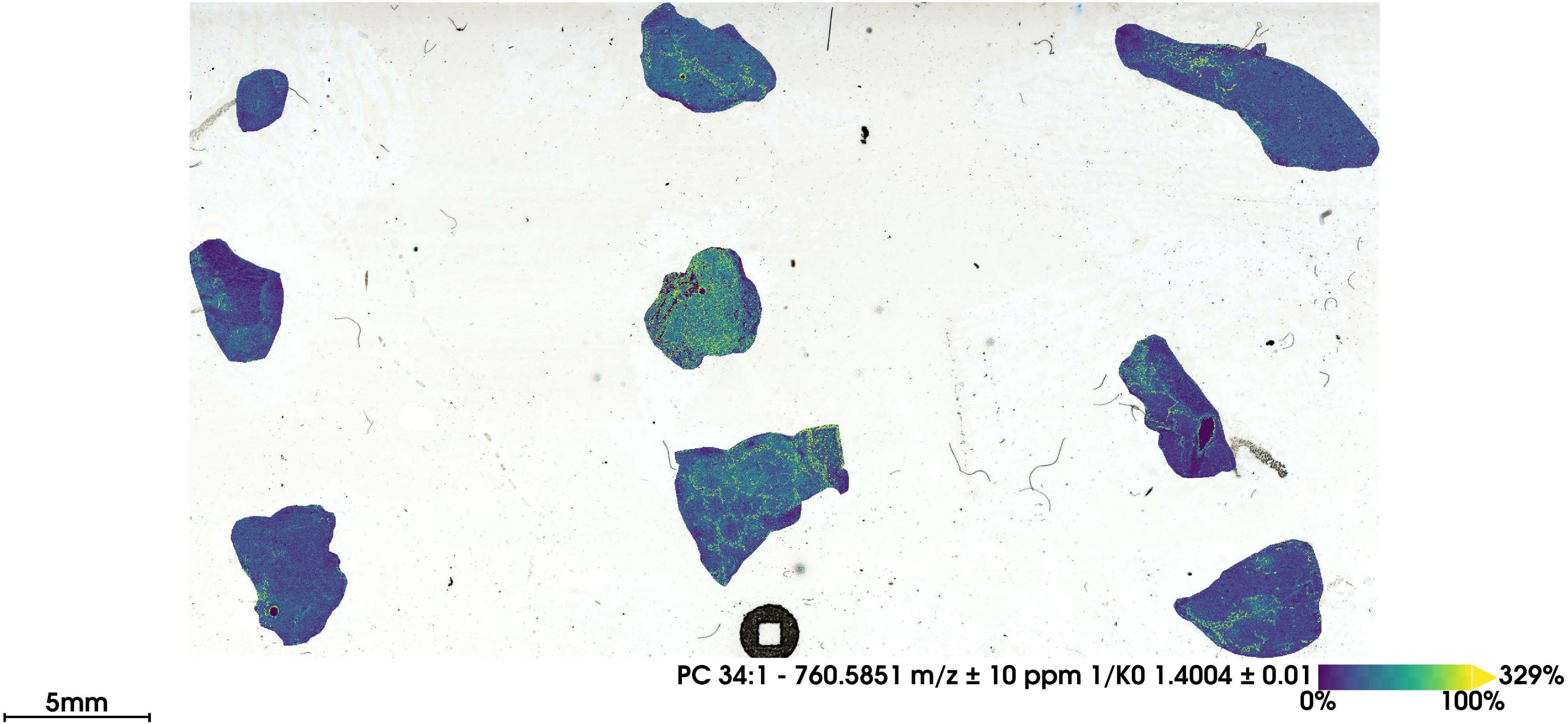
PC 34:1 - 760.5851 m/z ± 10 ppm 1/K0 1.4004 ± 0.01
0%
100%
329%
5mm

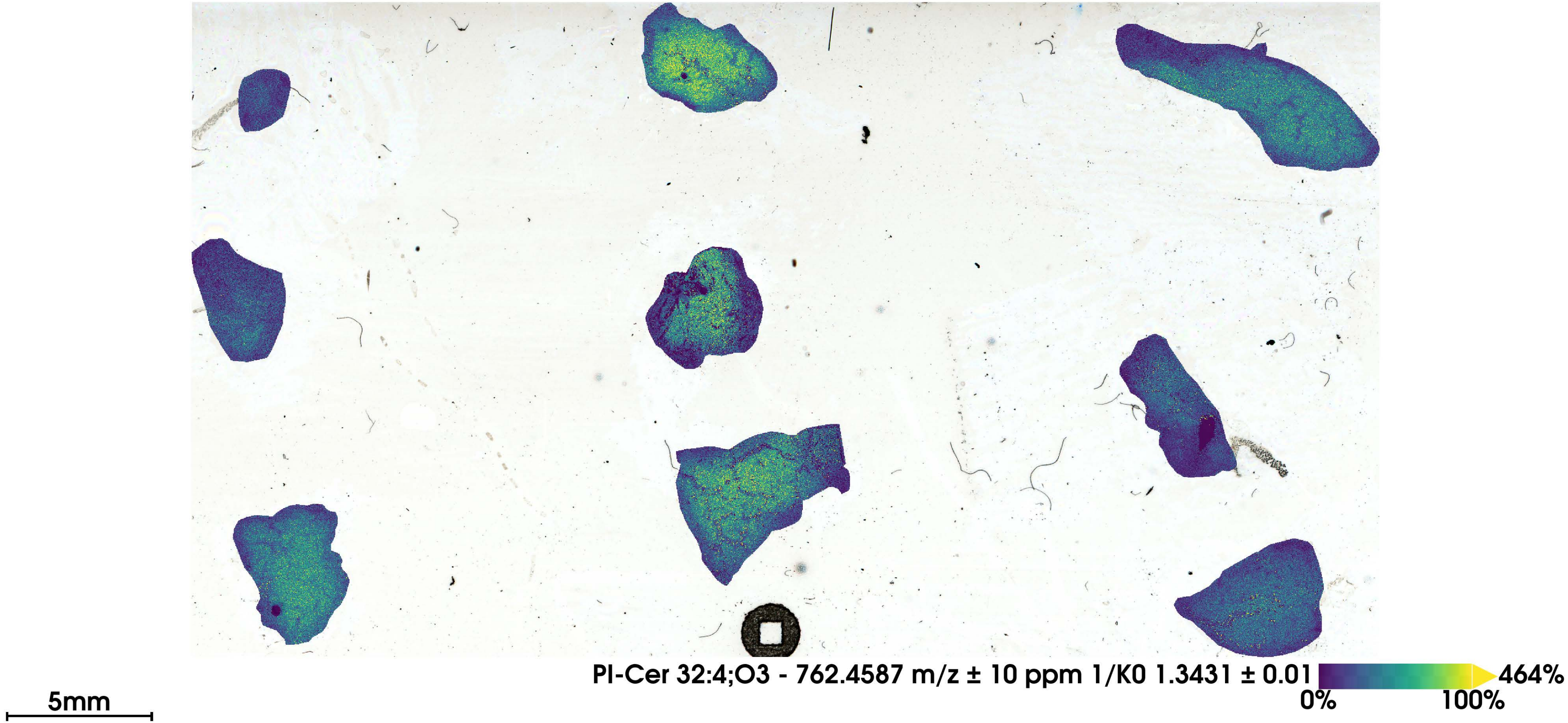
PI-Cer 32:4;O3 - 762.4587 m/z ± 10 ppm 1/K0 1.3431 ± 0.01
0%
100%
464%
5mm

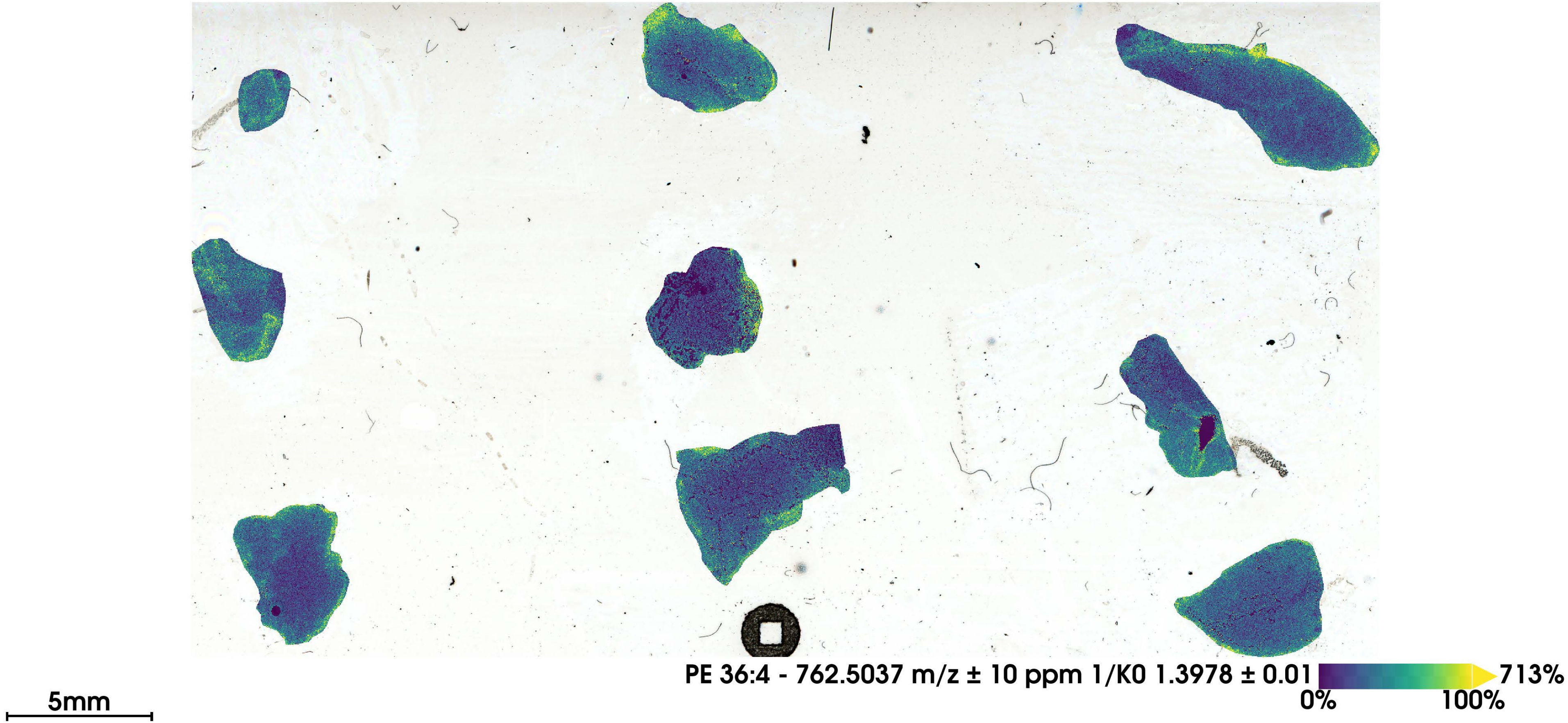
5mm
PE 36:4 - 762.5037 m/z ± 10 ppm 1/K0 1.3978 ± 0.01
713%
0%
100%

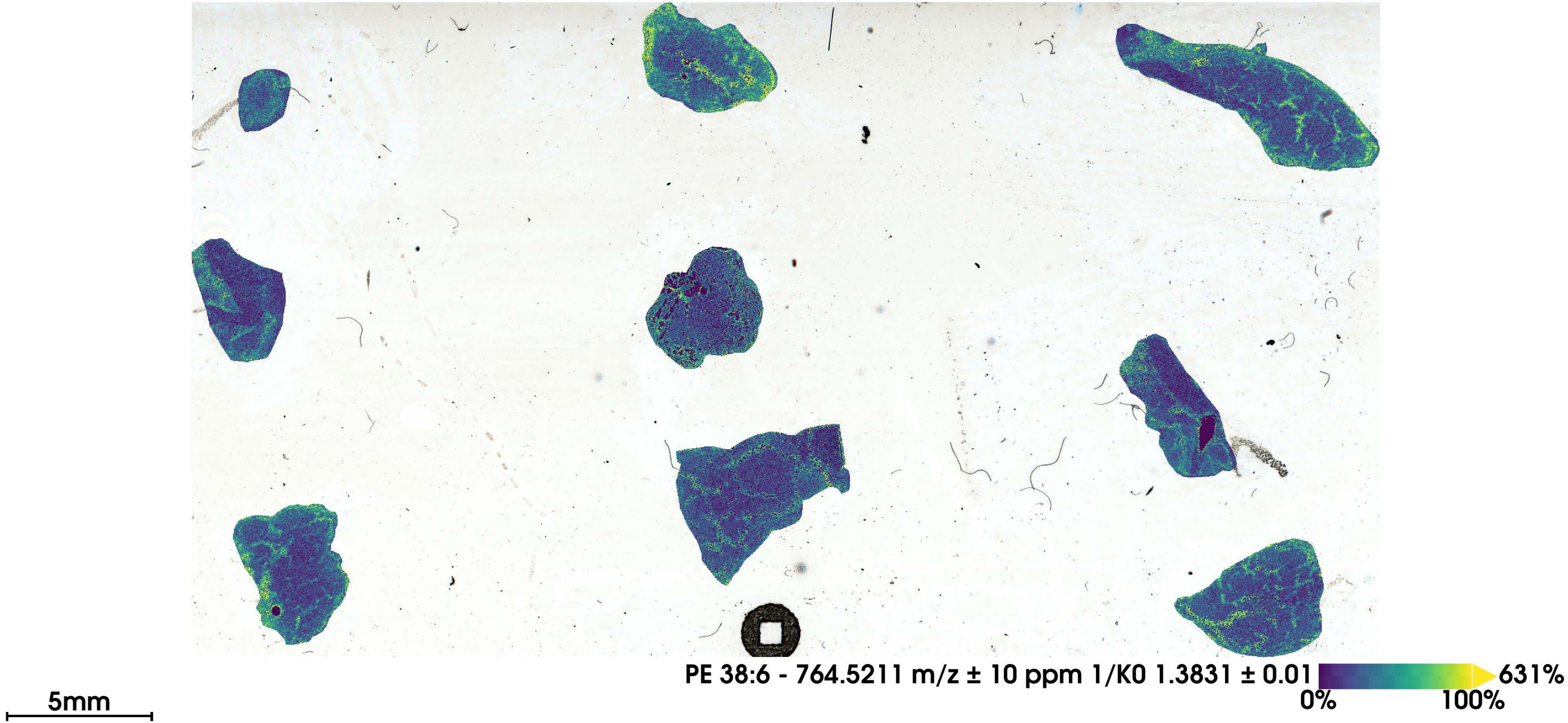
PE 38:6 - 764.5211 m/z ± 10 ppm 1/K0 1.3831 ± 0.01
631%
0%
100%
5mm

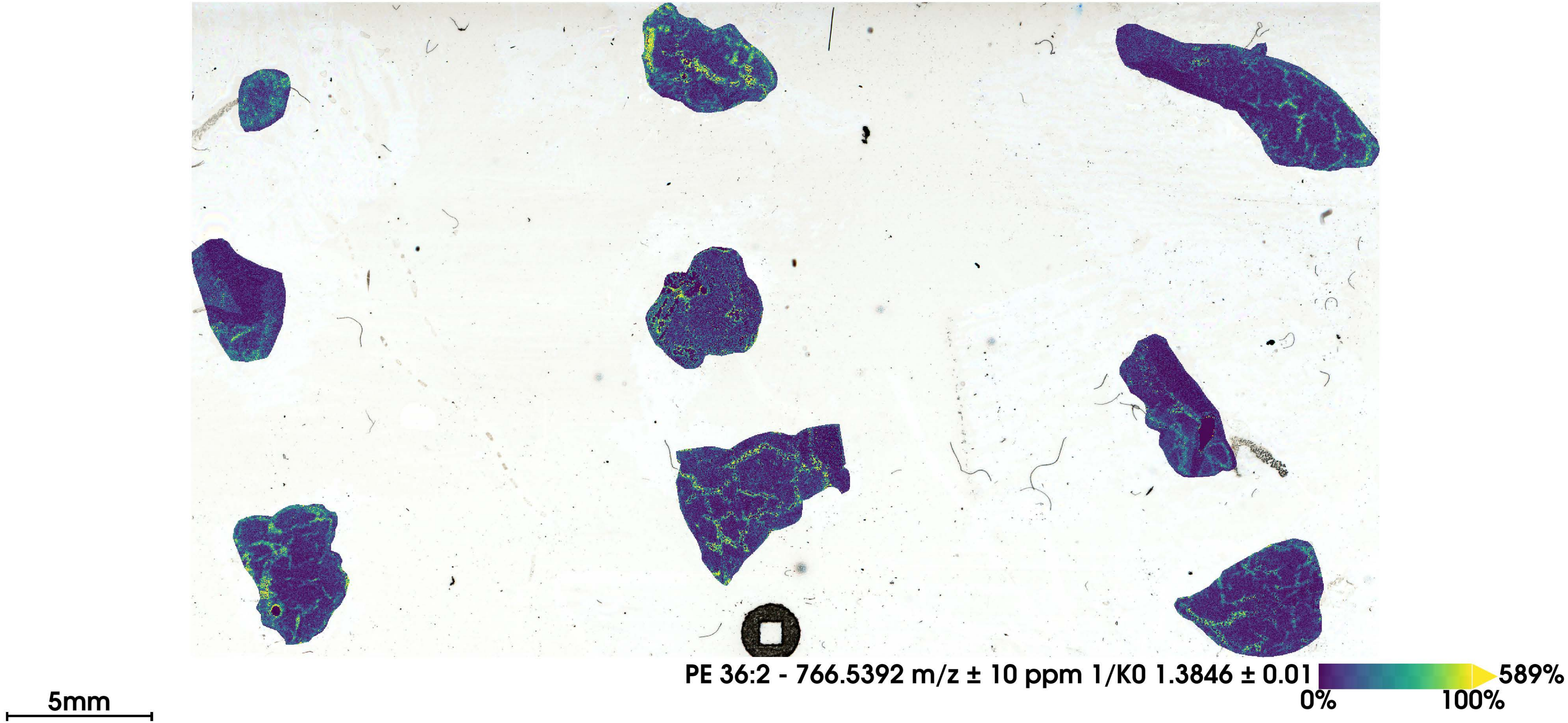

PE 36:2 - 766.5392 m/z ± 10 ppm 1/K0 1.3846 ± 0.01
589%
0%
100%
5mm

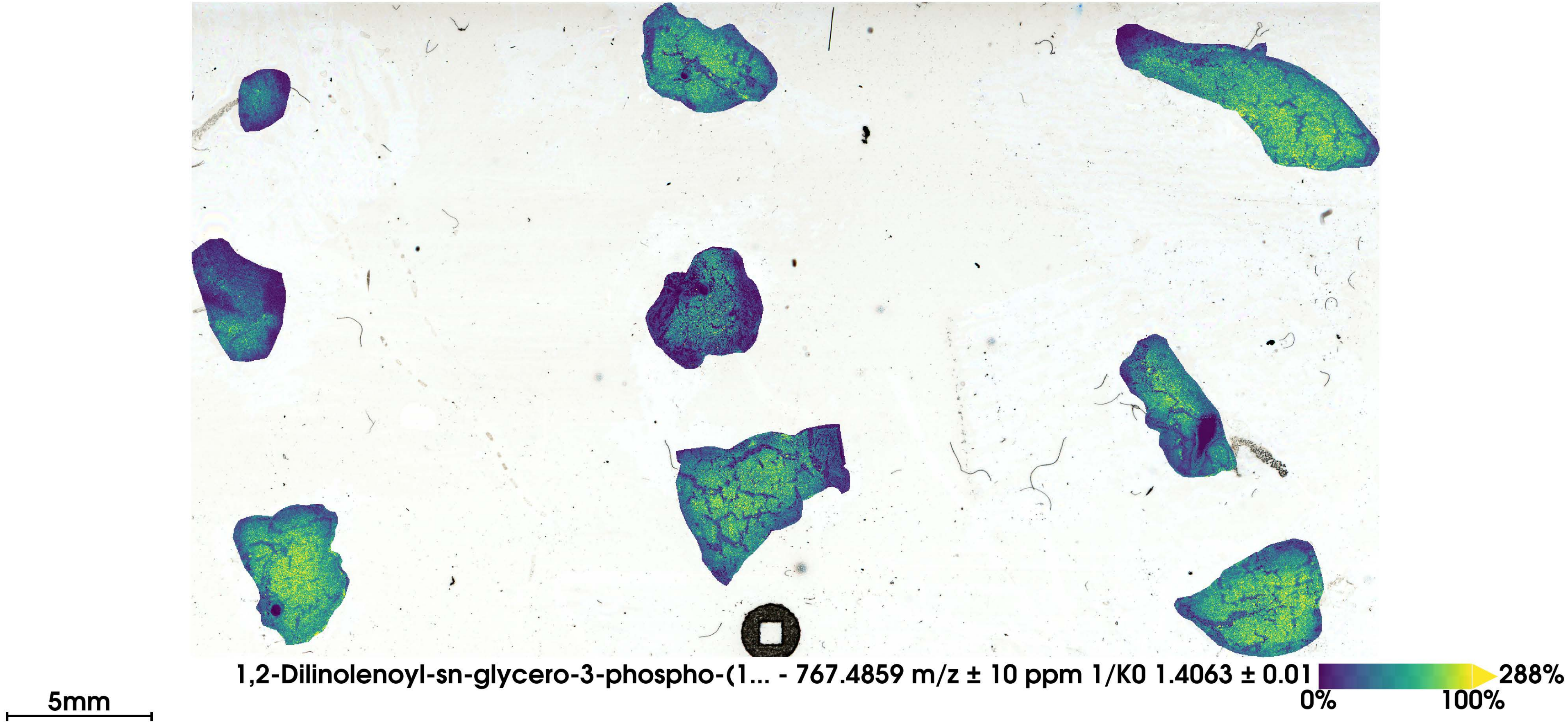

1,2-Dilinolenoyl-sn-glycero-3-phospho-(1... - 767.4859 m/z ± 10 ppm 1/K0 1.4063 ± 0.01
288%
0%
100%
5mm

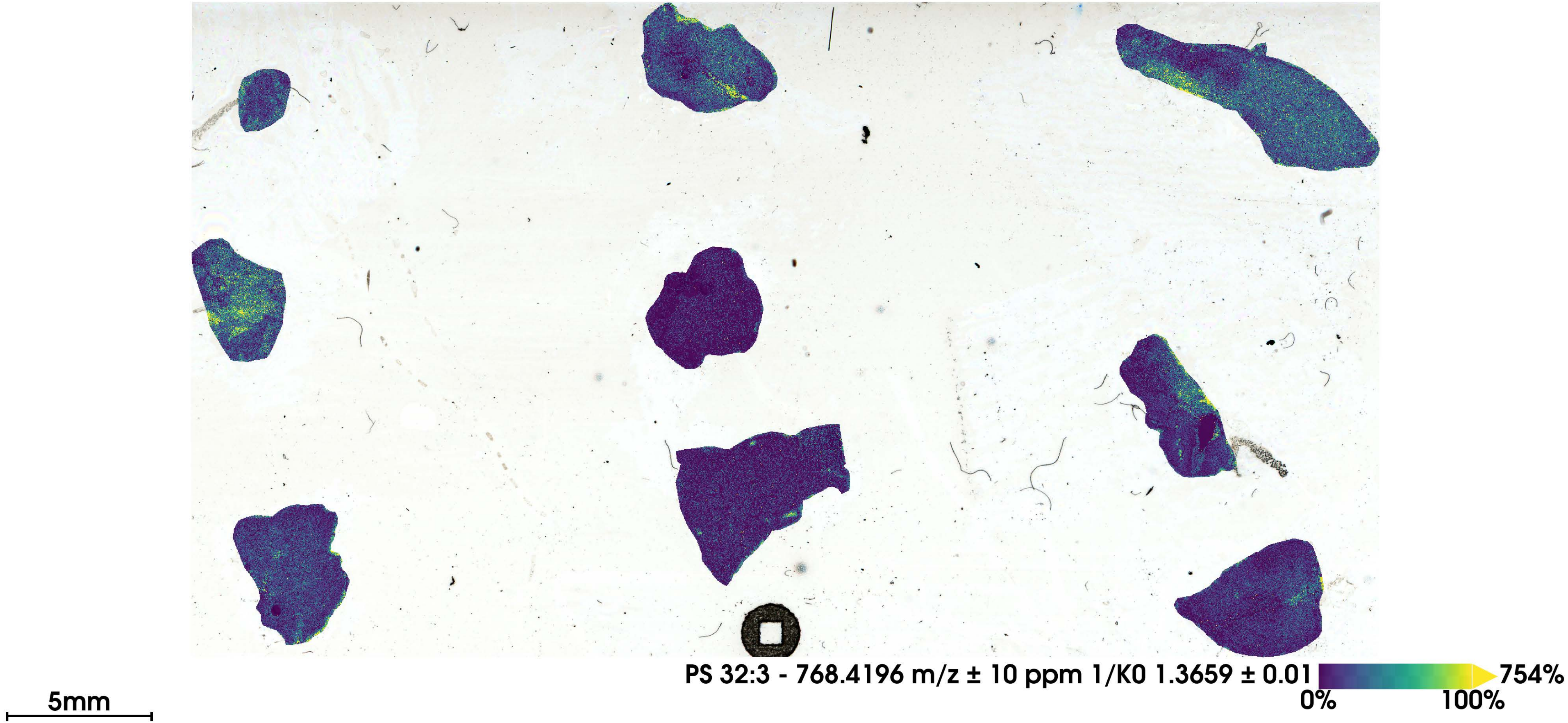

5mm
PS 32:3 - 768.4196 m/z ± 10 ppm 1/K0 1.3659 ± 0.01
754%
0%
100%

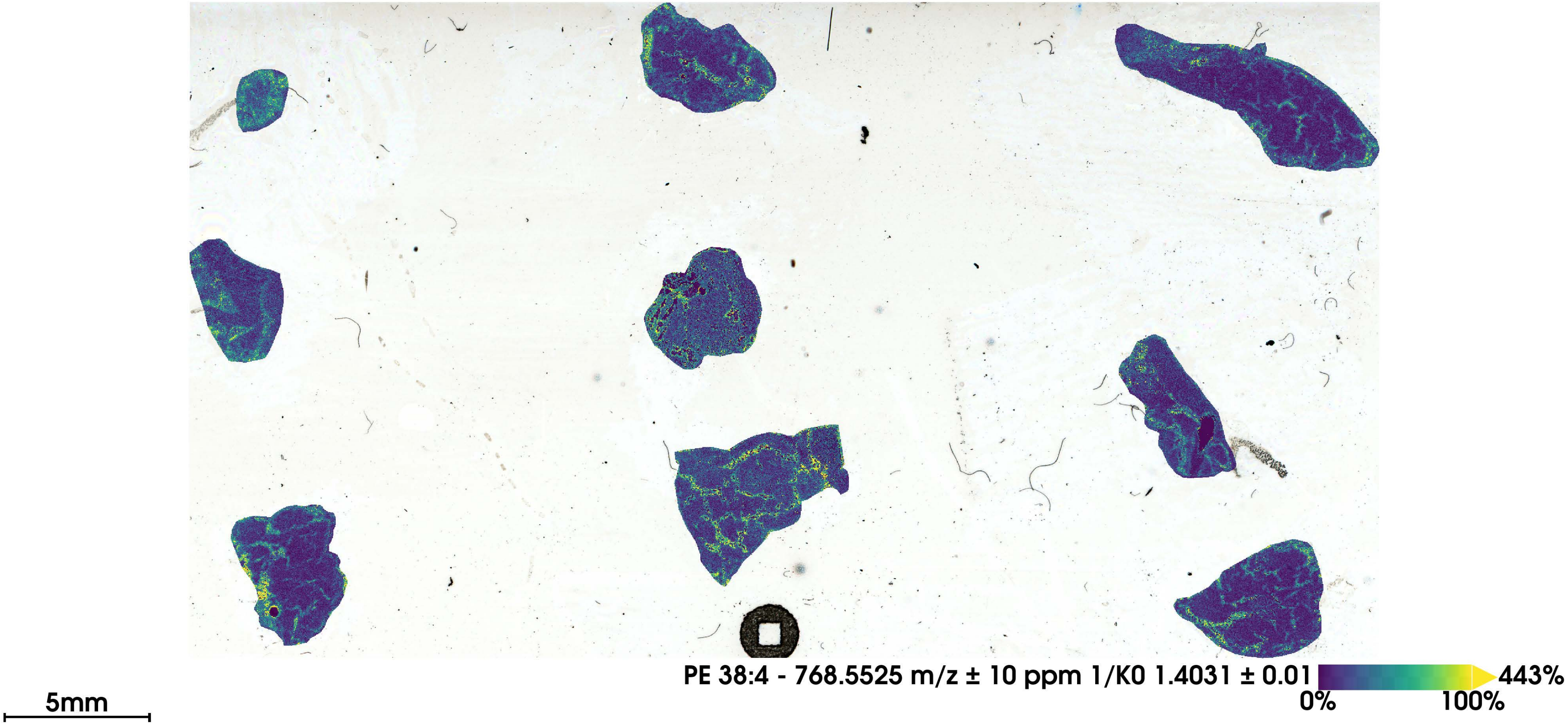
PE 38:4 - 768.5525 m/z ± 10 ppm 1/K0 1.4031 ± 0.01
443%
0%
100%
5mm

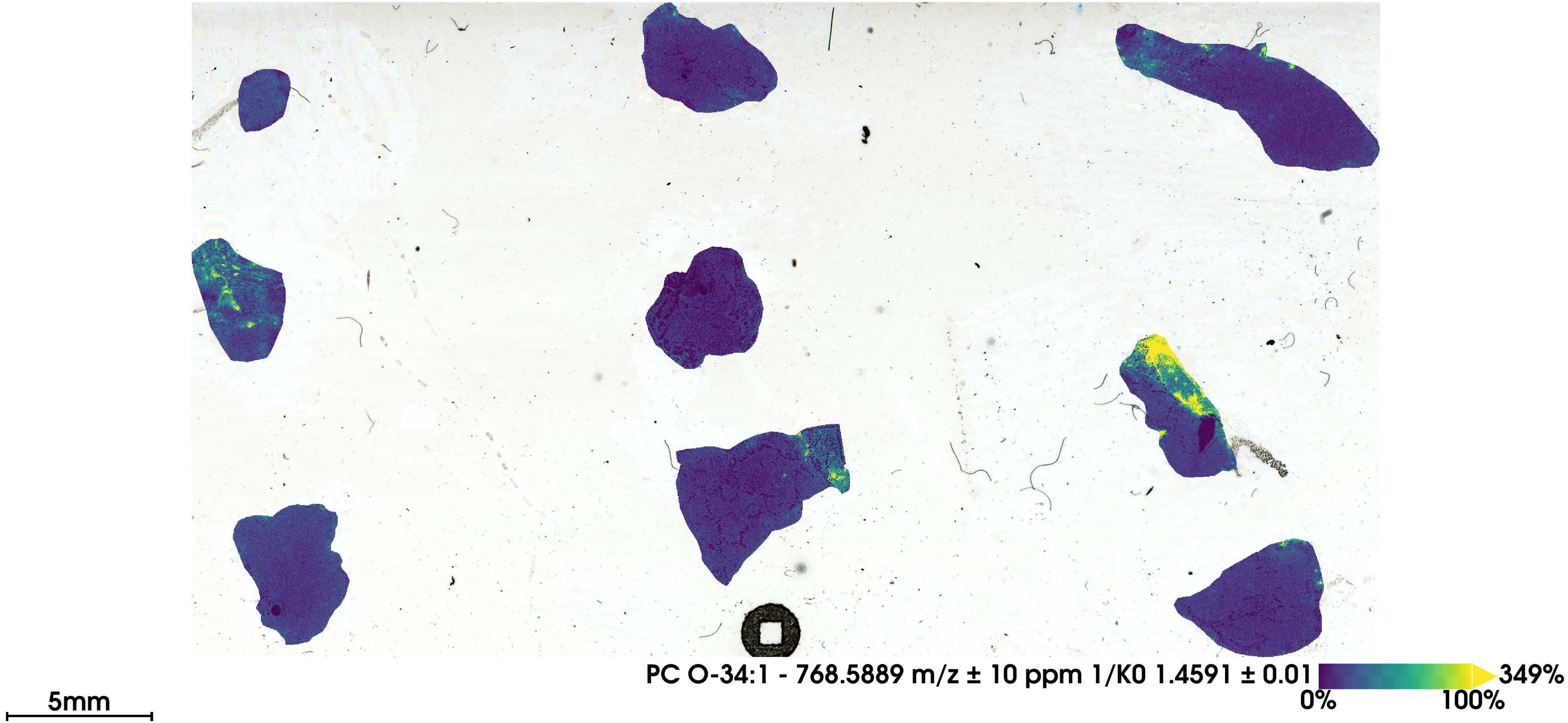
PC O-34:1 - 768.5889 m/z ± 10 ppm 1/K0 1.4591 ± 0.01
349%
0%
100%
5mm

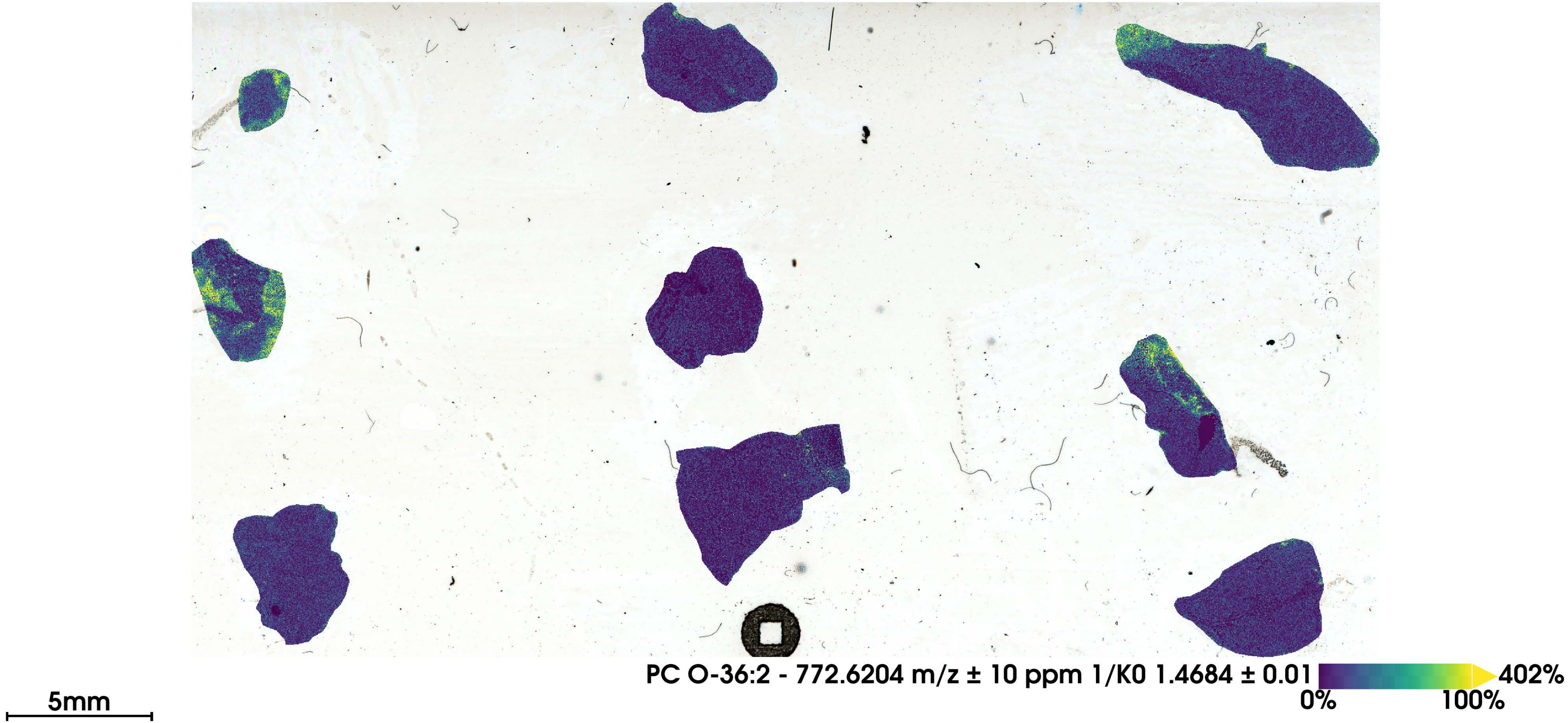

PC O-36:2 - 772.6204 m/z ± 10 ppm 1/K0 1.4684 ± 0.01
0%
100%
402%
5mm

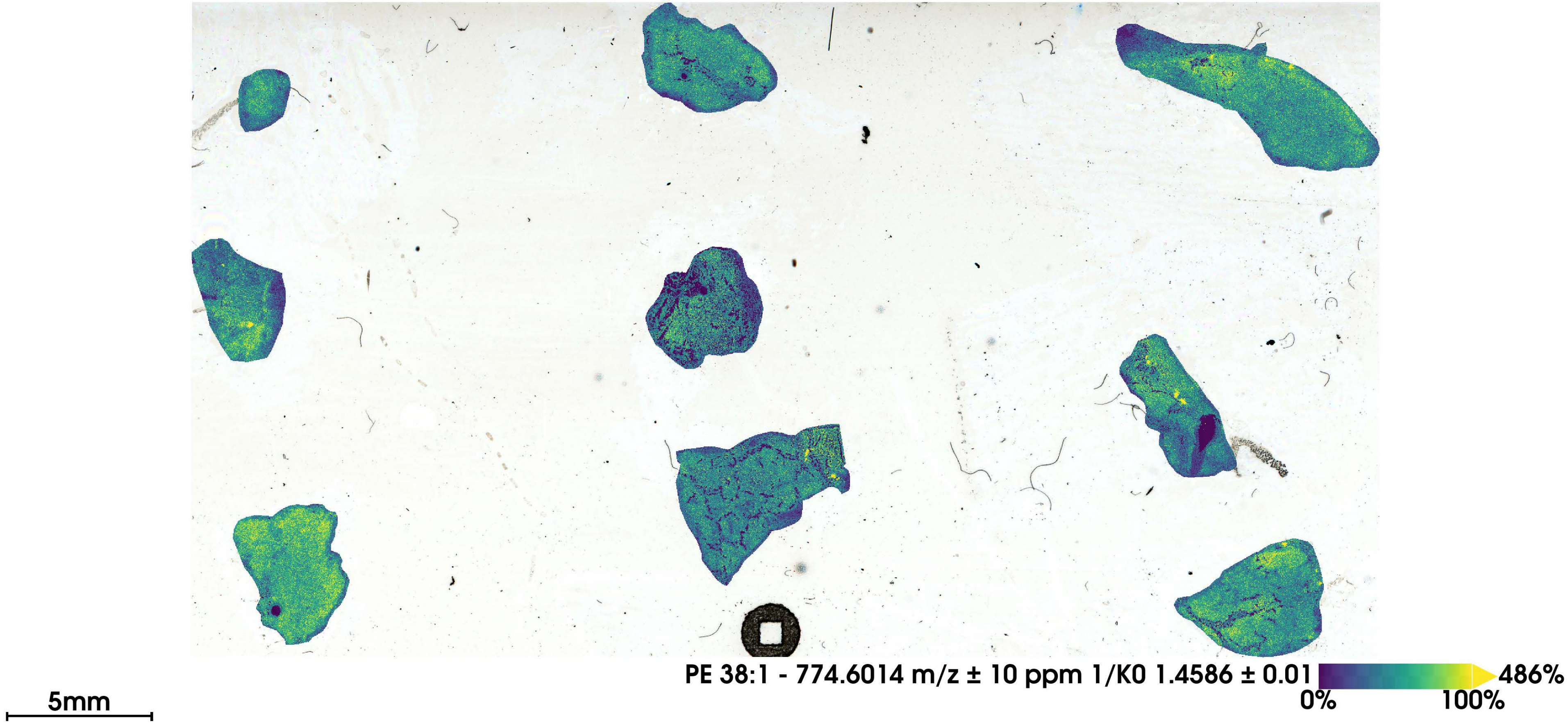

PE 38:1 - 774.6014 m/z ± 10 ppm 1/K0 1.4586 ± 0.01
486%
0%
100%
5mm

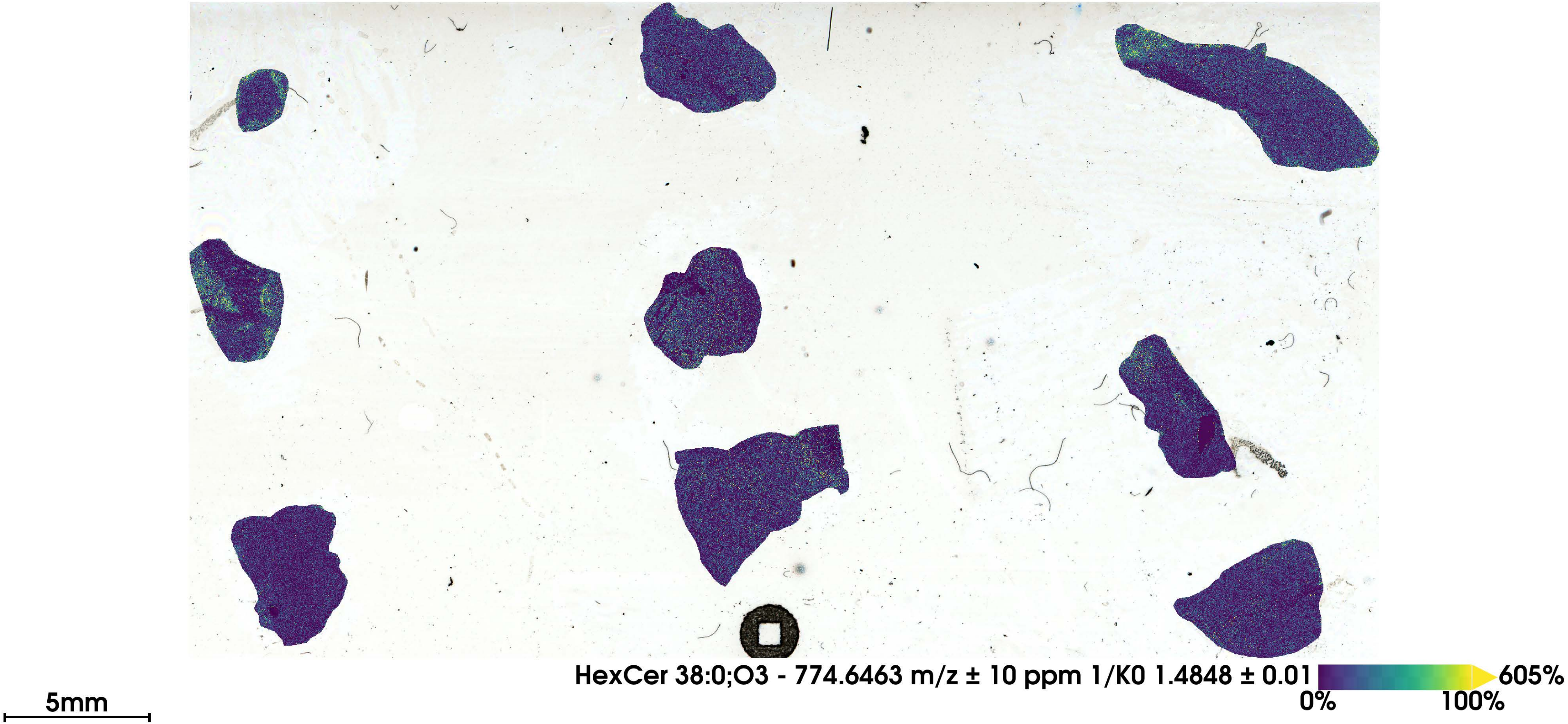

HexCer 38:0;O3 - 774.6463 m/z ± 10 ppm 1/K0 1.4848 ± 0.01
605%
0%
100%
5mm

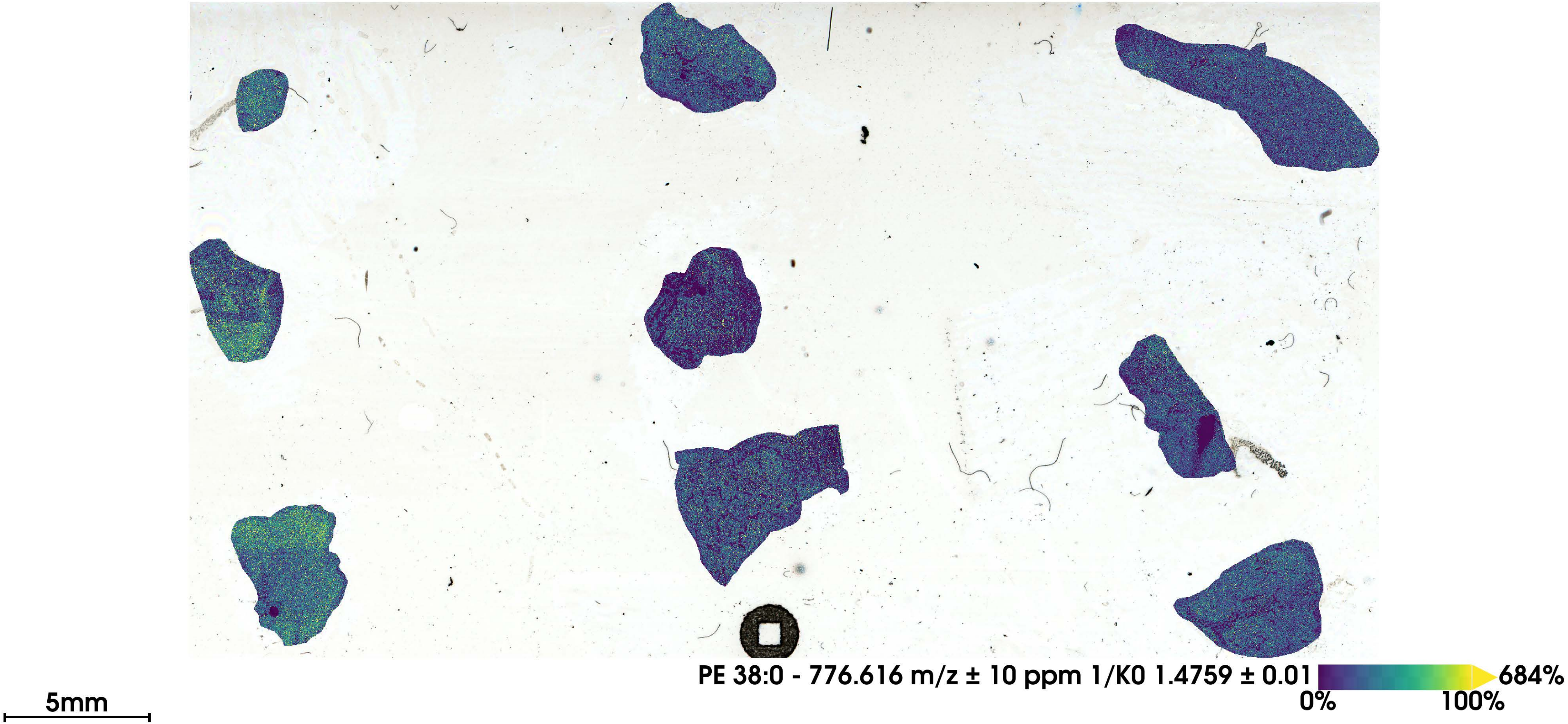
PE 38:0 - 776.616 m/z ± 10 ppm 1/K0 1.4759 ± 0.01
684%
0%
100%
5mm

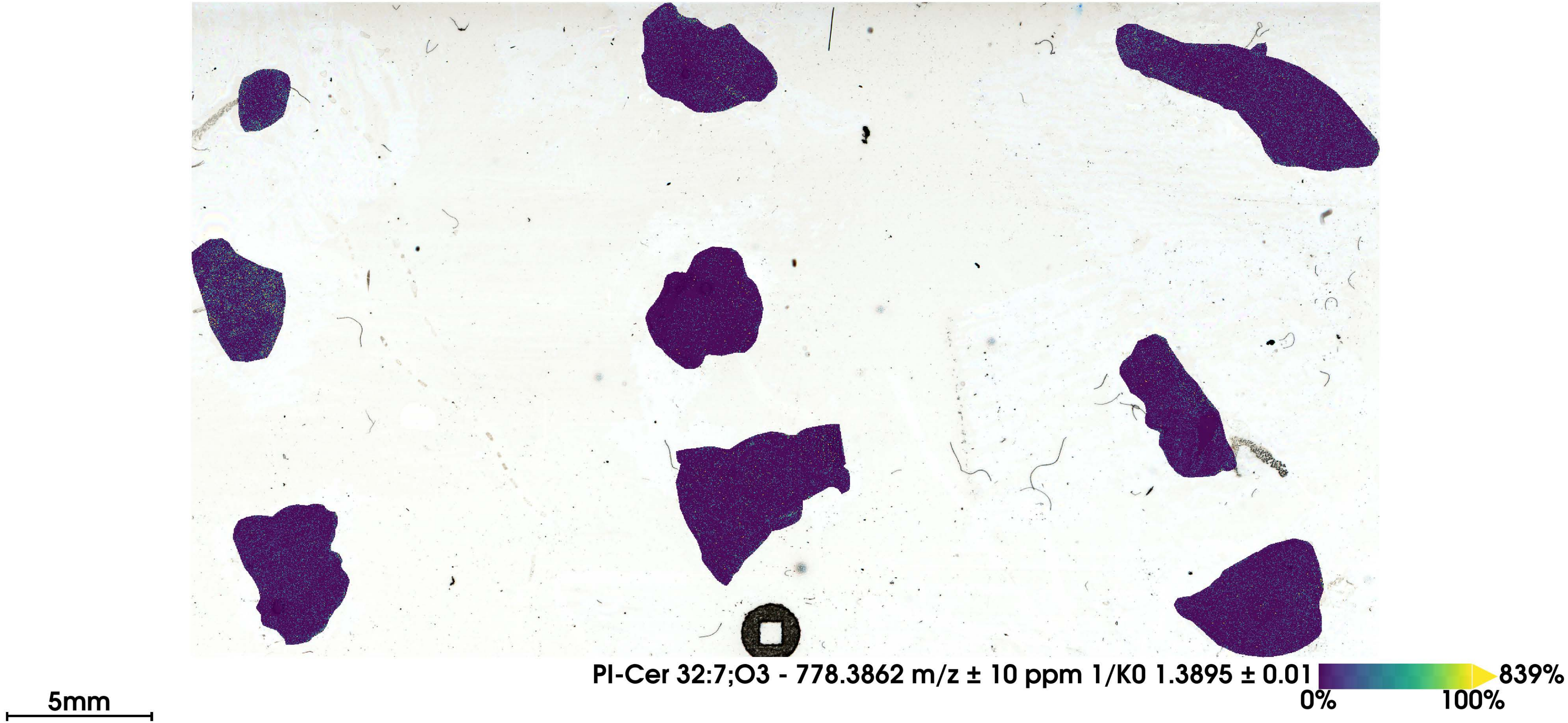
PI-Cer 32:7;O3 - 778.3862 m/z ± 10 ppm 1/K0 1.3895 ± 0.01
839%
0%
100%
5mm

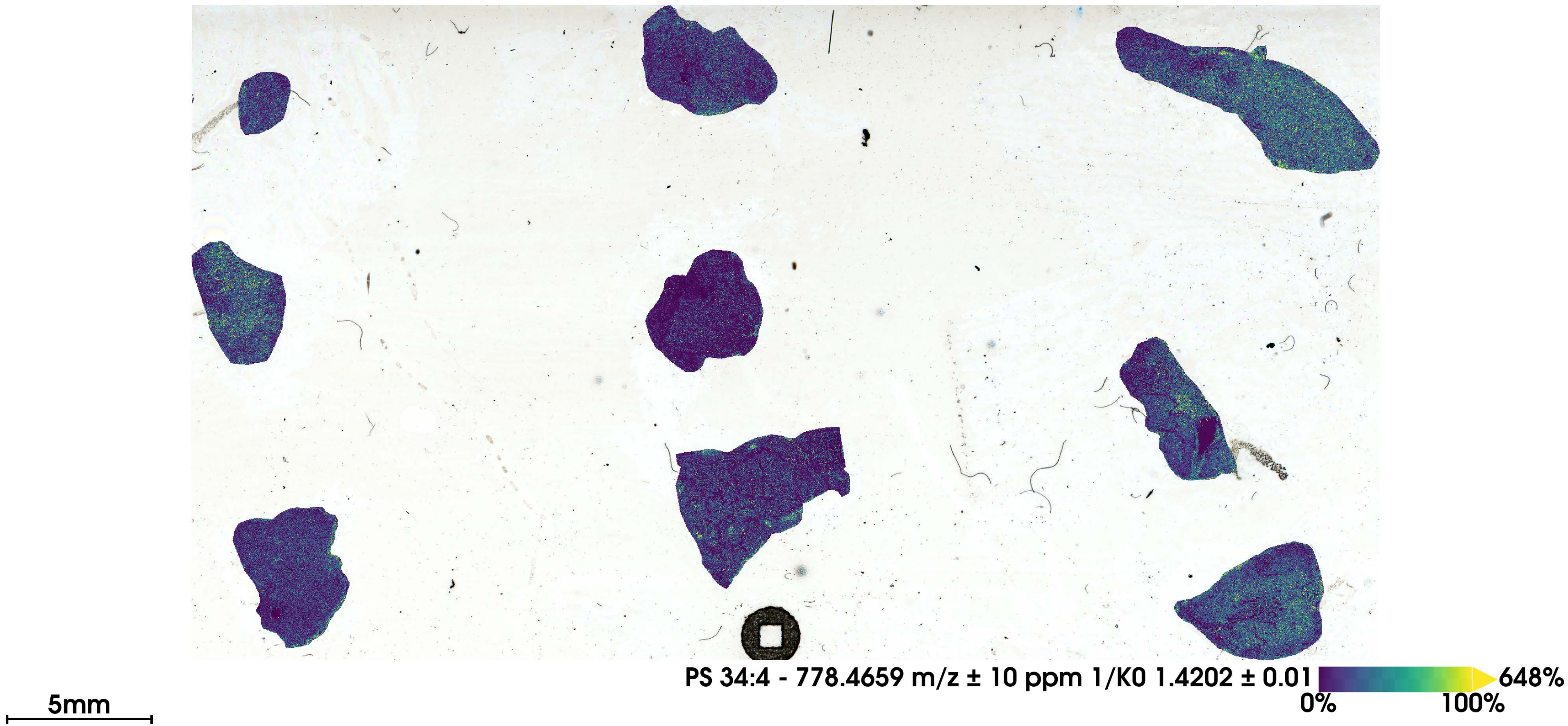
PS 34:4 - 778.4659 m/z ± 10 ppm 1/K0 1.4202 ± 0.01
648%
0%
100%
5mm

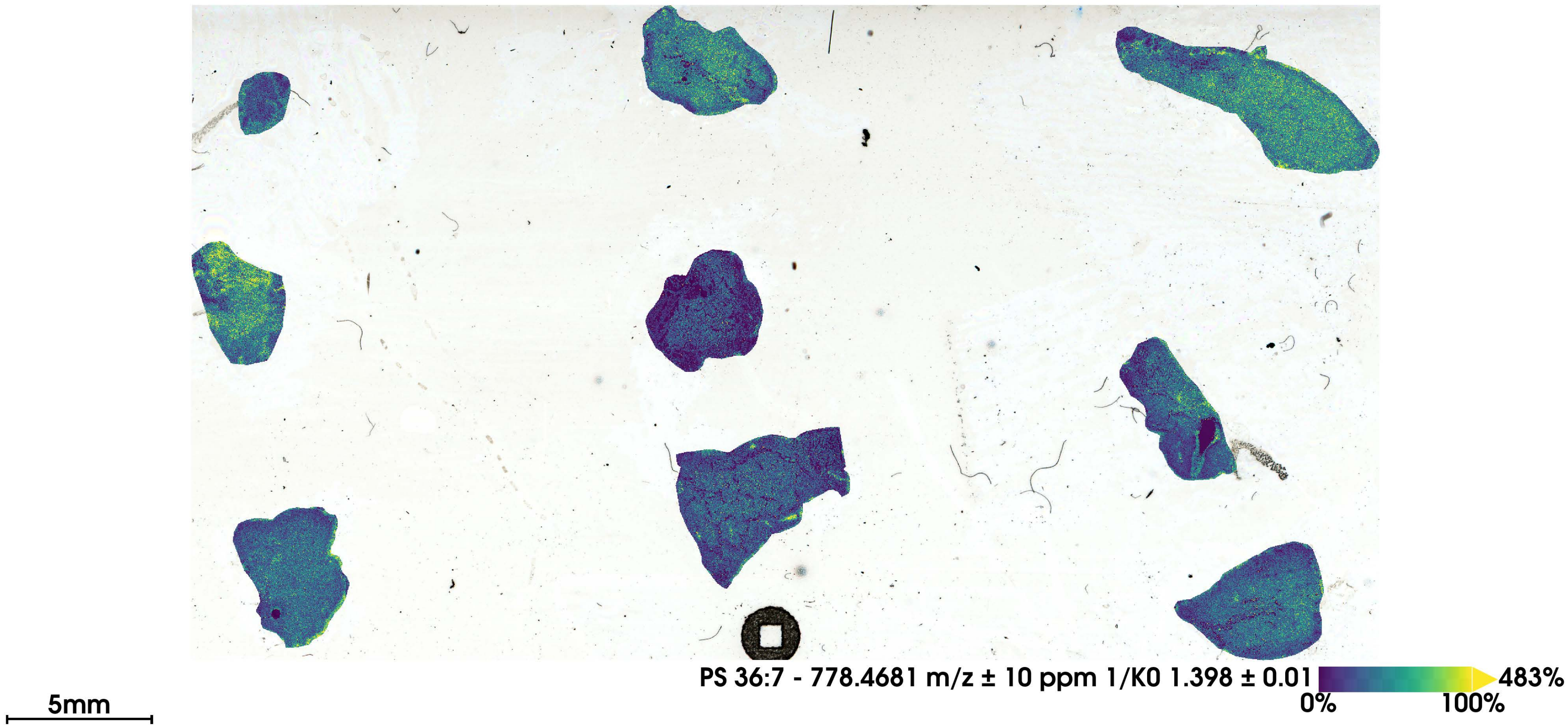
PS 36:7 - 778.4681 m/z ± 10 ppm 1/K0 1.398 ± 0.01
483%
0%
100%
5mm

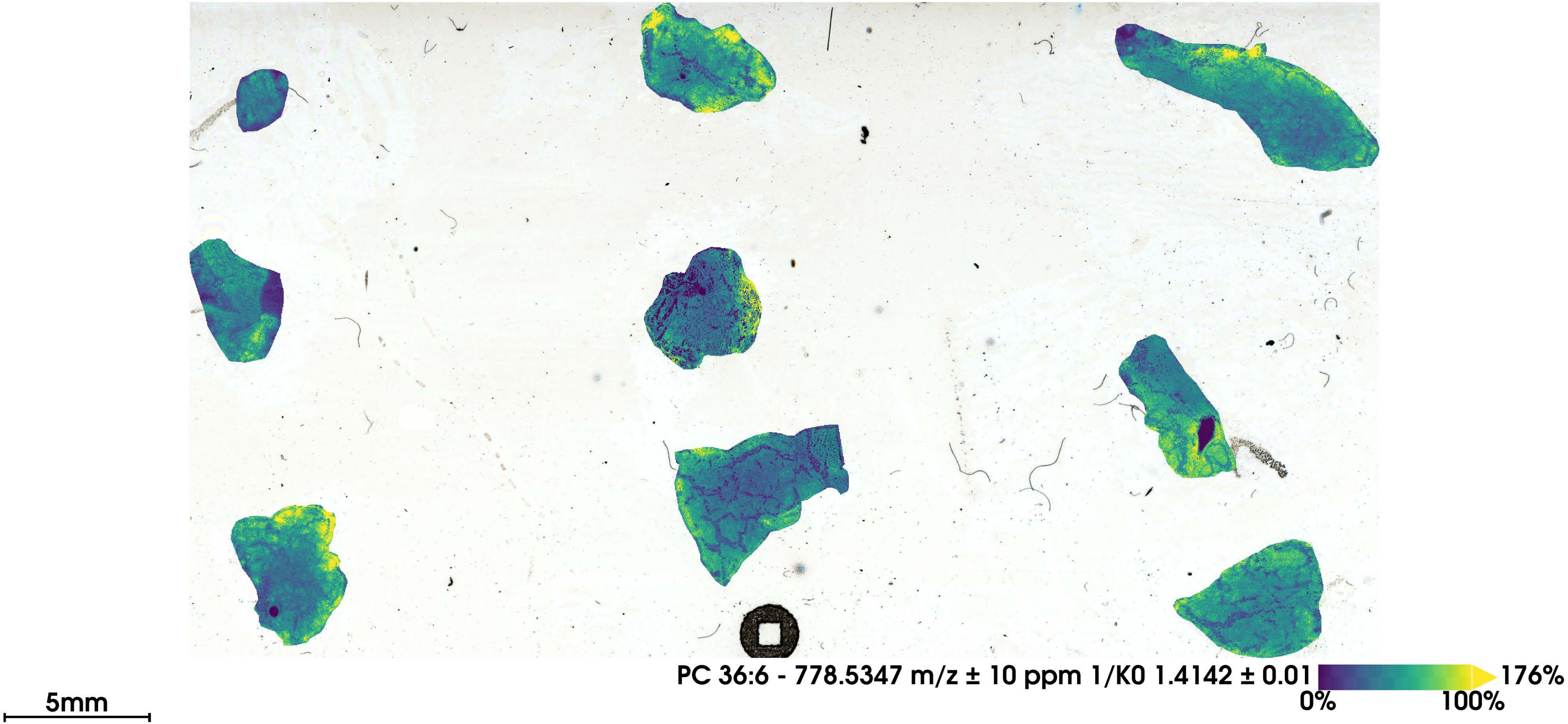
PC 36:6 - 778.5347 m/z ± 10 ppm 1/K0 1.4142 ± 0.01
176%
0%
100%
5mm

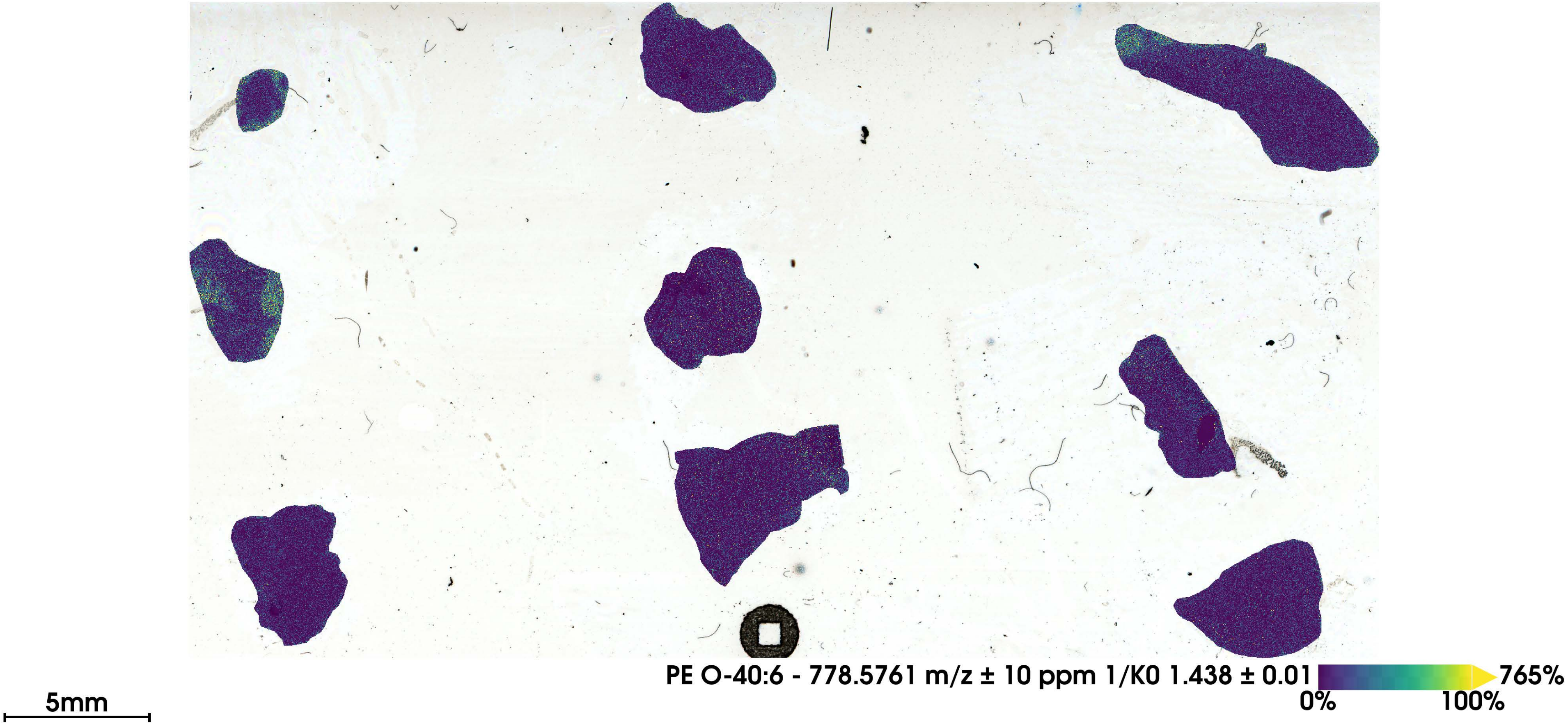
PE O-40:6 - 778.5761 m/z ± 10 ppm 1/K0 1.438 ± 0.01
765%
0%
100%
5mm

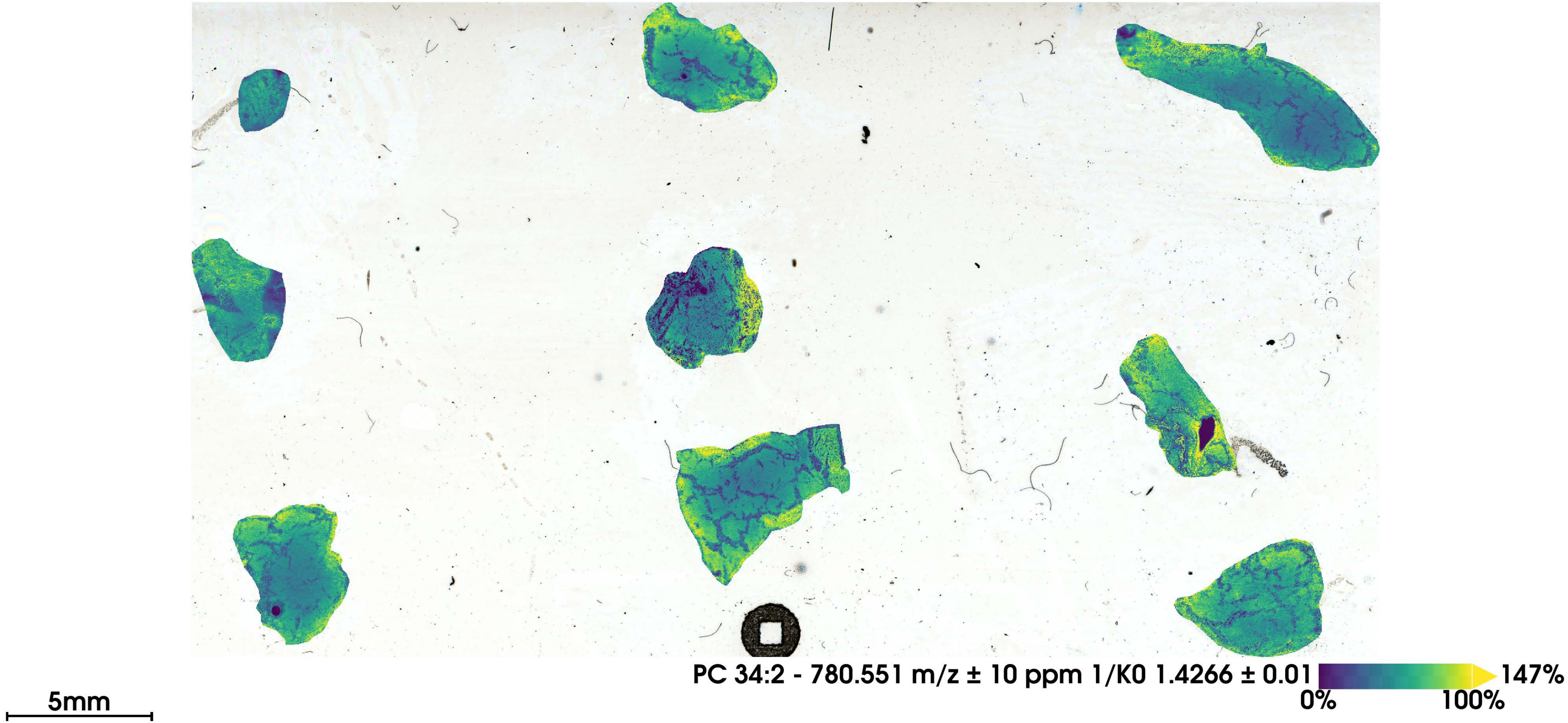
PC 34:2 - 780.551 m/z ± 10 ppm 1/K0 1.4266 ± 0.01
0%
100%
147%
5mm

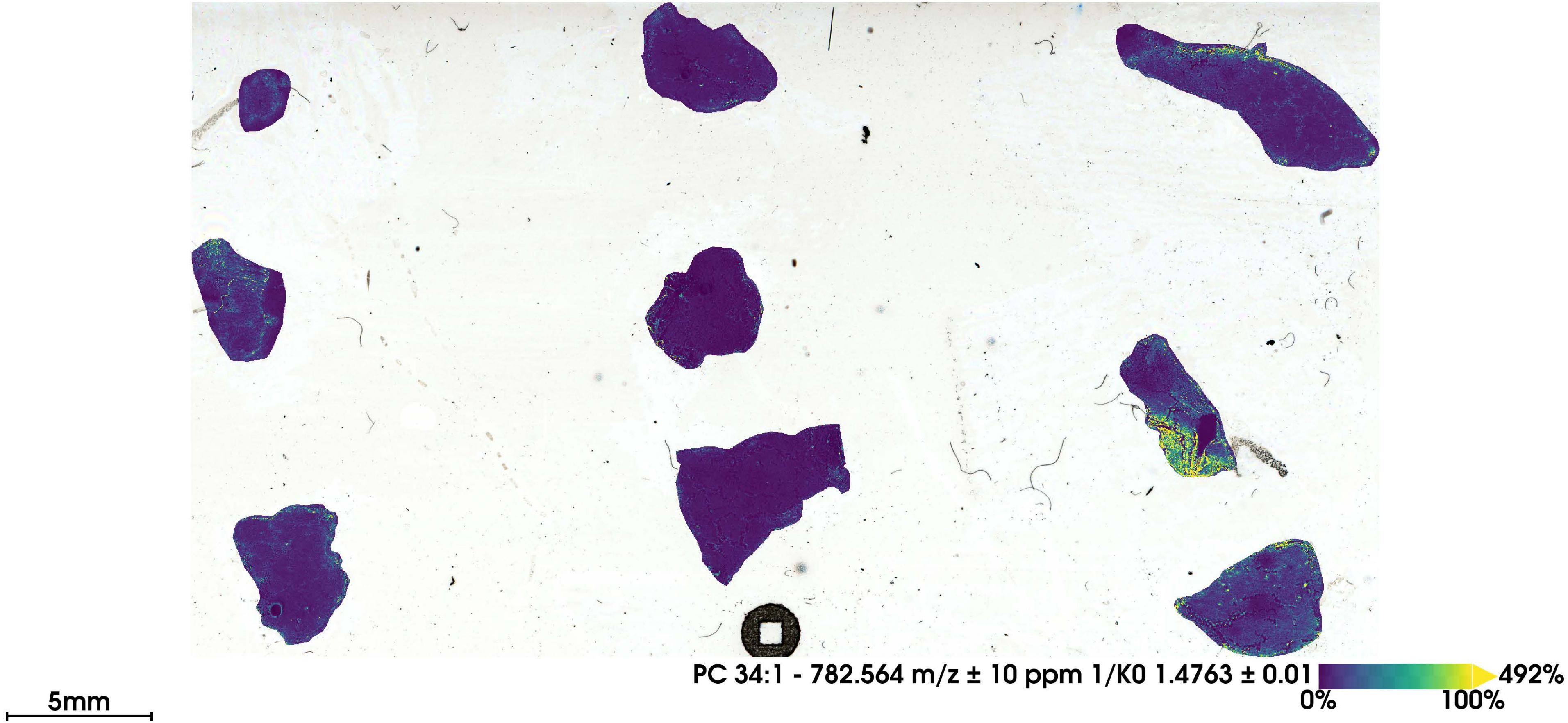
PC 34:1 - 782.564 m/z ± 10 ppm 1/K0 1.4763 ± 0.01
492%
0%
100%
5mm

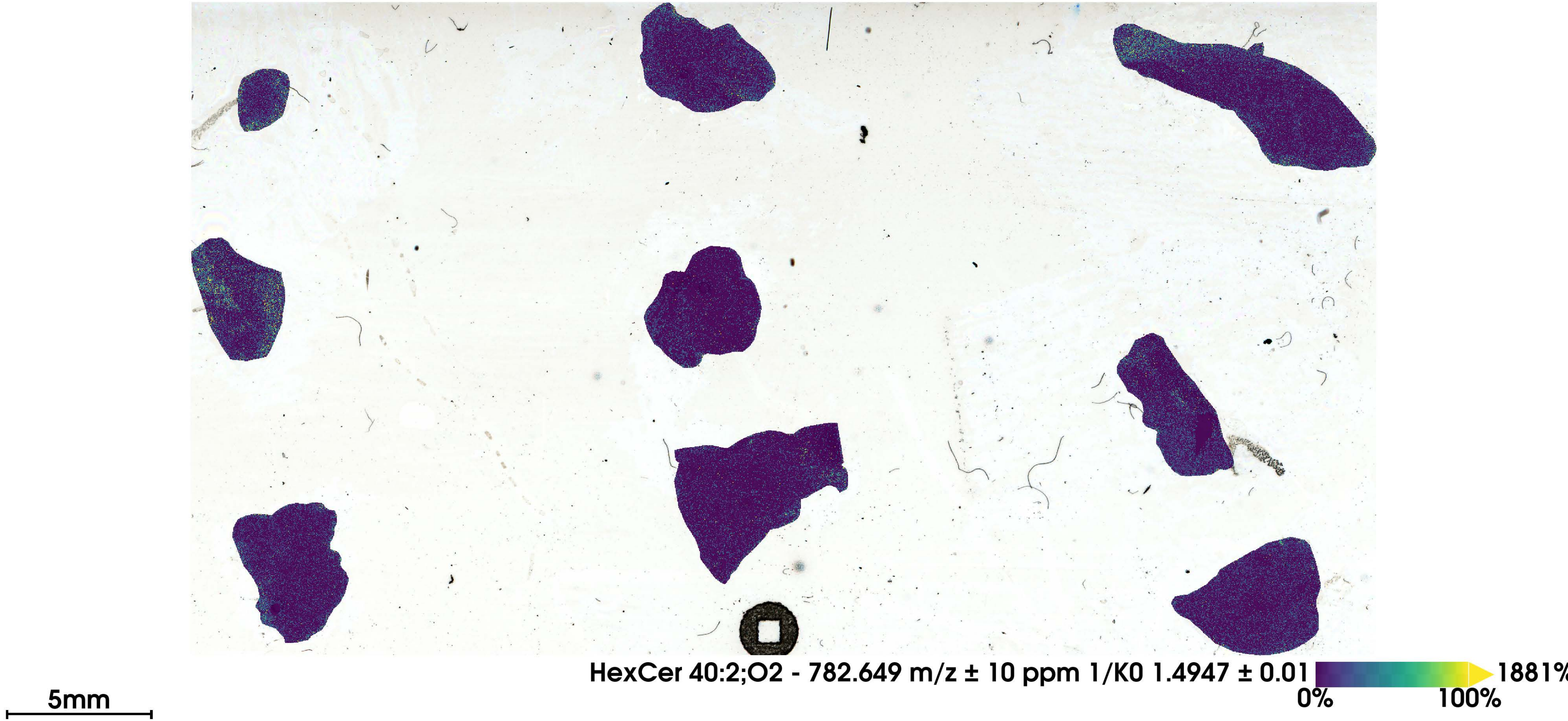
HexCer 40:2;O2 - 782.649 m/z ± 10 ppm 1/K0 1.4947 ± 0.01
1881%
0%
100%
5mm

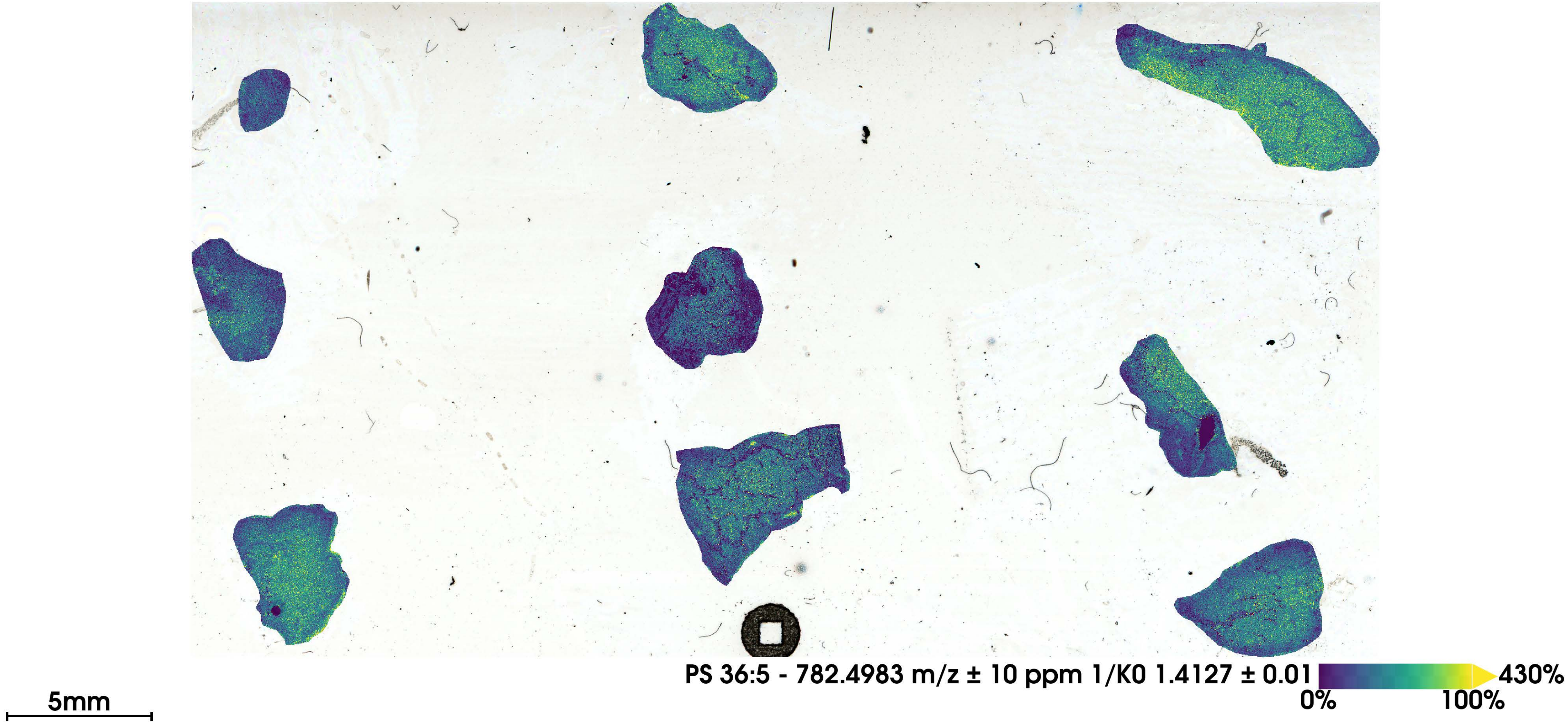
PS 36:5 - 782.4983 m/z ± 10 ppm 1/K0 1.4127 ± 0.01
0%
100%
430%
5mm

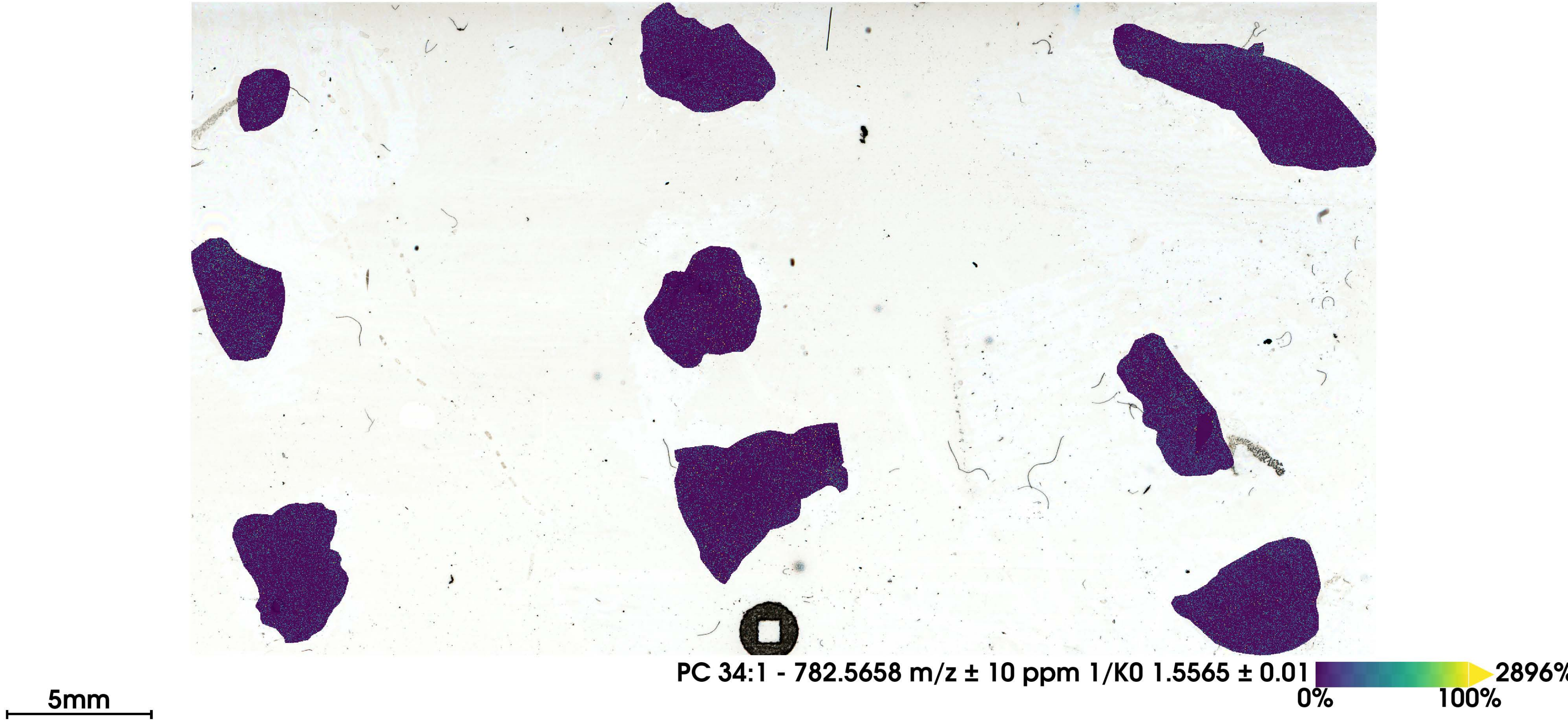
PC 34:1 - 782.5658 m/z ± 10 ppm 1/K0 1.5565 ± 0.01
2896%
0%
100%
5mm

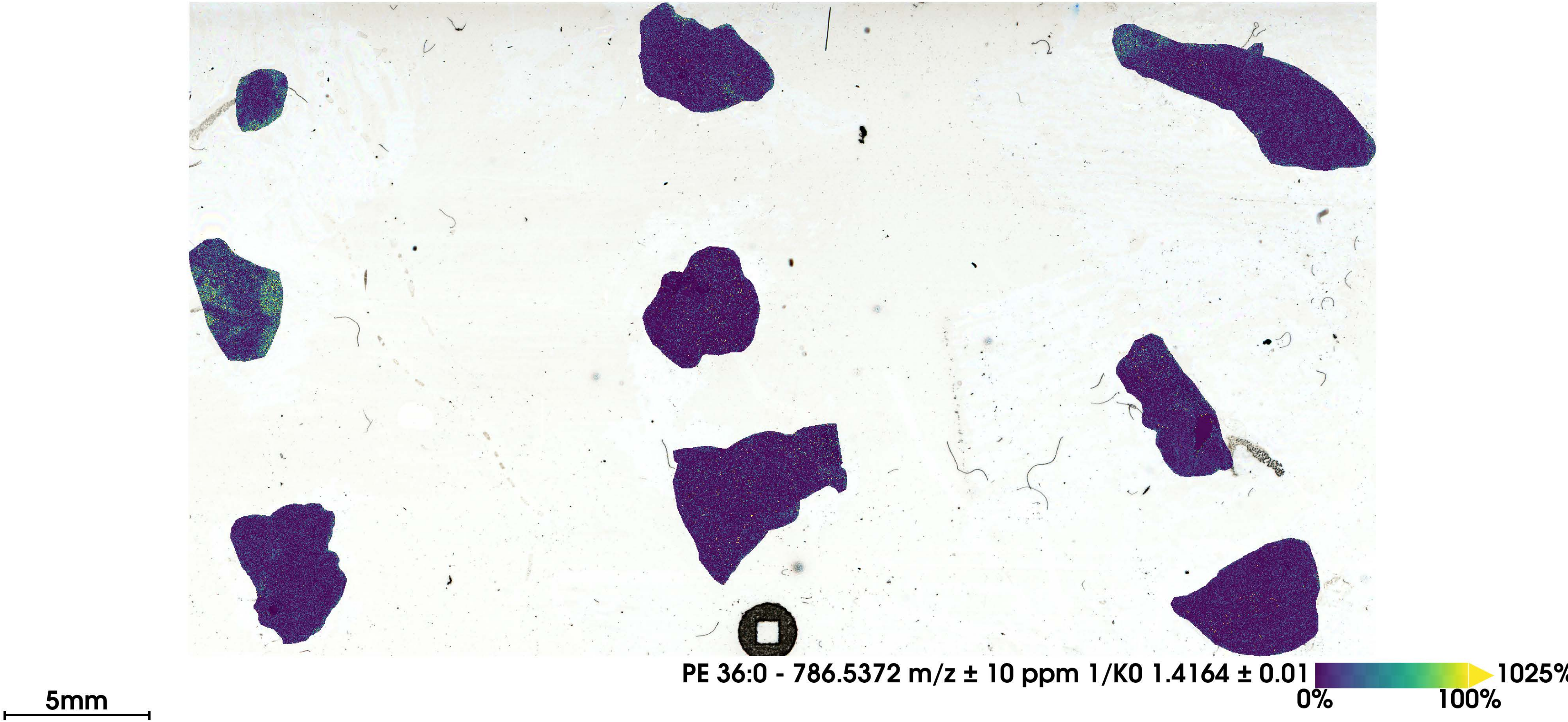
PE 36:0 - 786.5372 m/z ± 10 ppm 1/K0 1.4164 ± 0.01
1025%
0%
100%
5mm

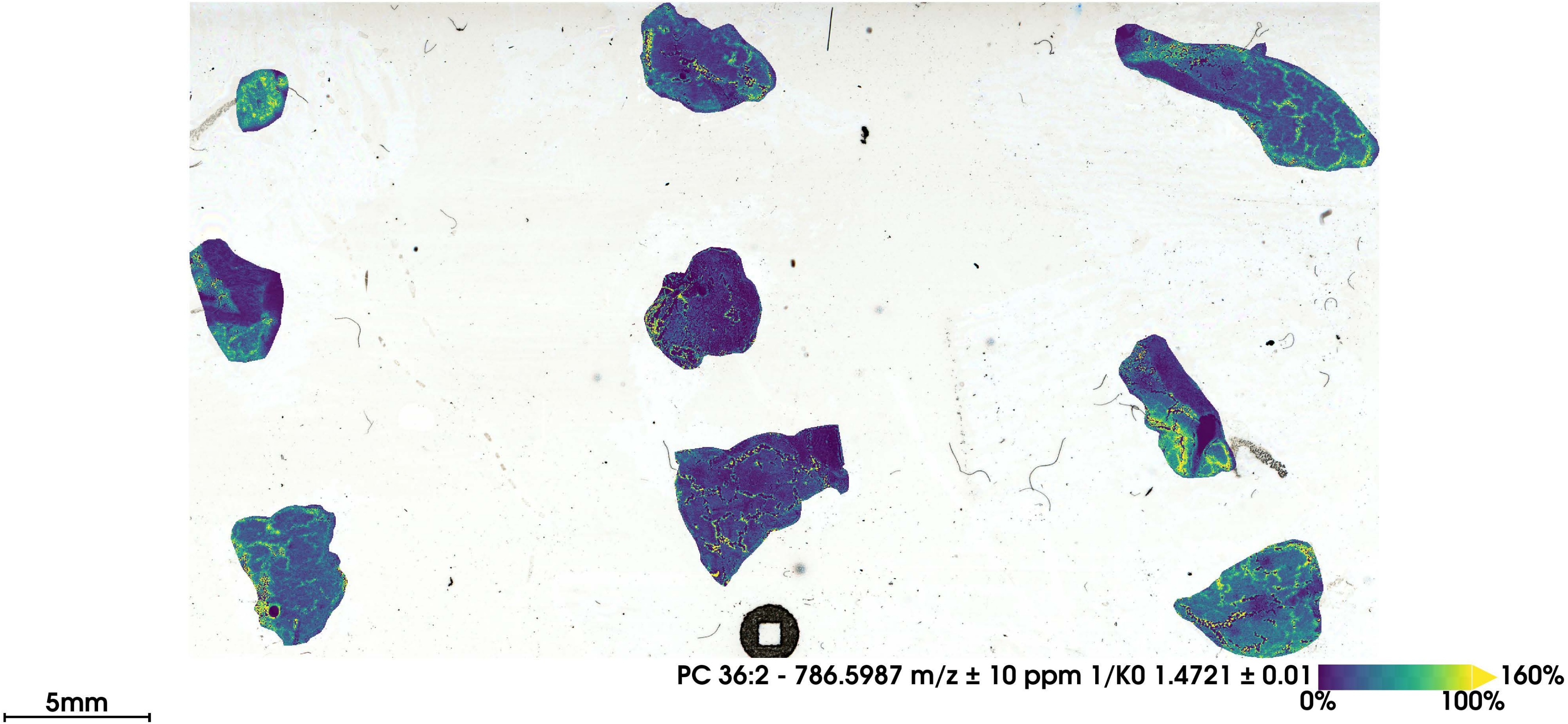

PC 36:2 - 786.5987 m/z ± 10 ppm 1/K0 1.4721 ± 0.01
160%
0%
100%
5mm

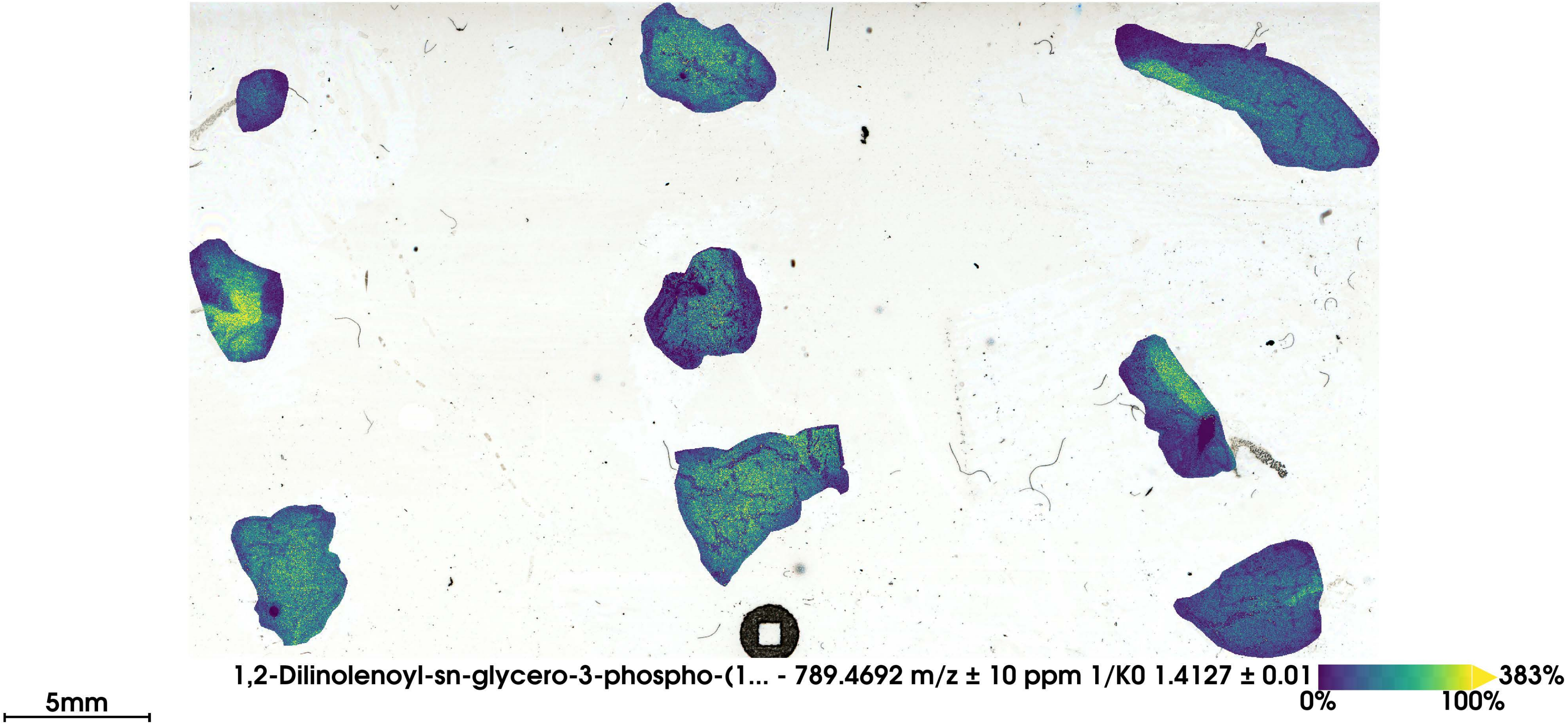
1,2-Dilinolenoyl-sn-glycero-3-phospho-(1... - 789.4692 m/z ± 10 ppm 1/K0 1.4127 ± 0.01
383%
0%
100%
5mm

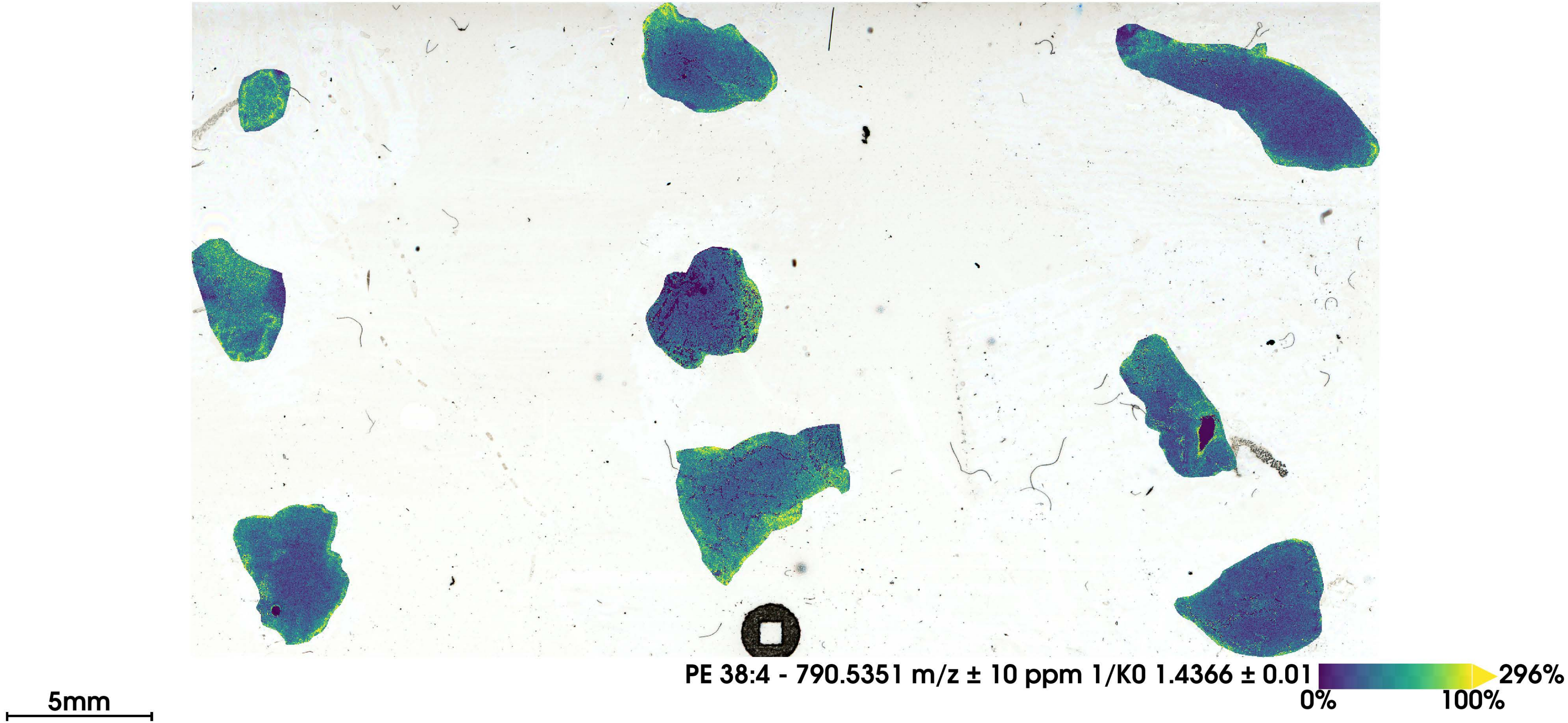
5mm
PE 38:4 - 790.5351 m/z ± 10 ppm 1/K0 1.4366 ± 0.01
296%
0%
100%

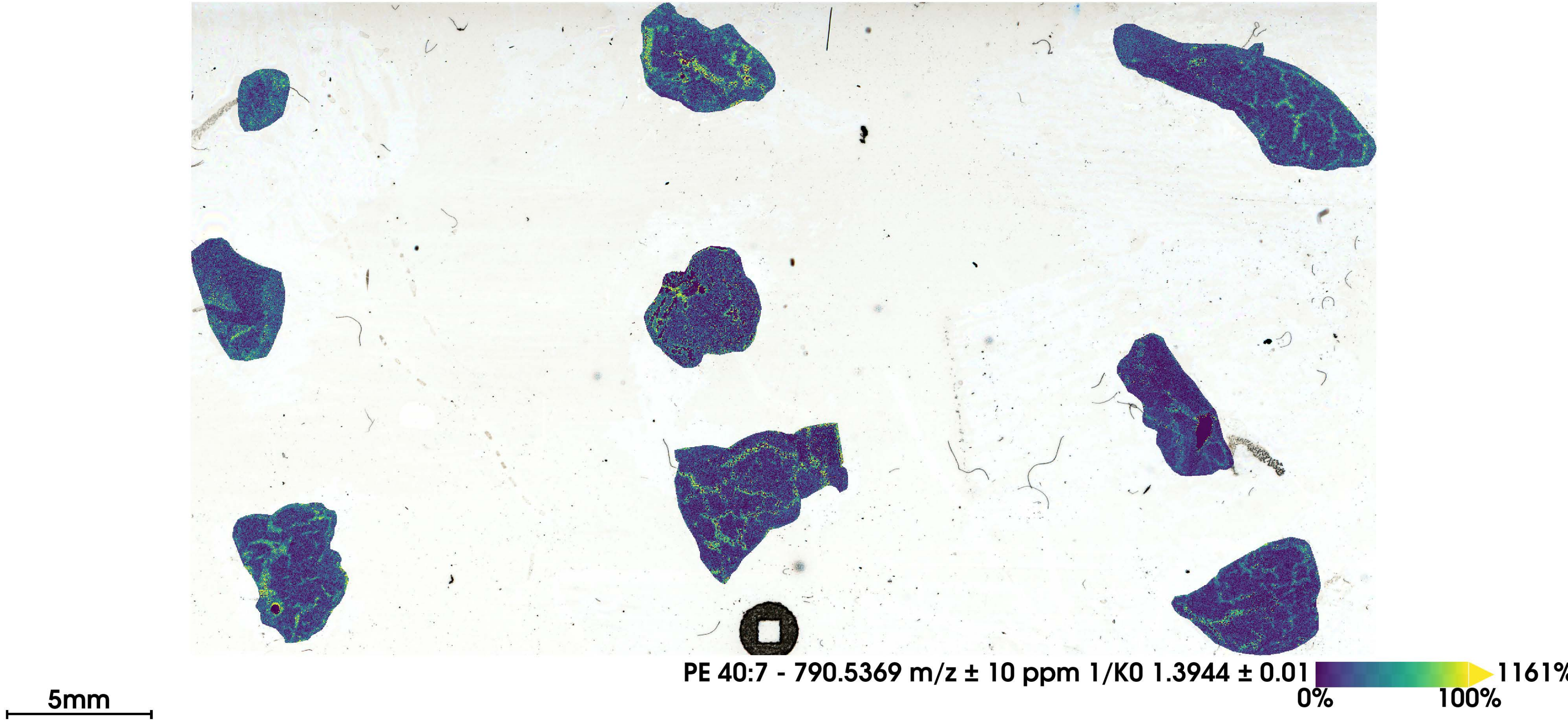

PE 40:7 - 790.5369 m/z ± 10 ppm 1/K0 1.3944 ± 0.01
1161%
0%
100%
5mm

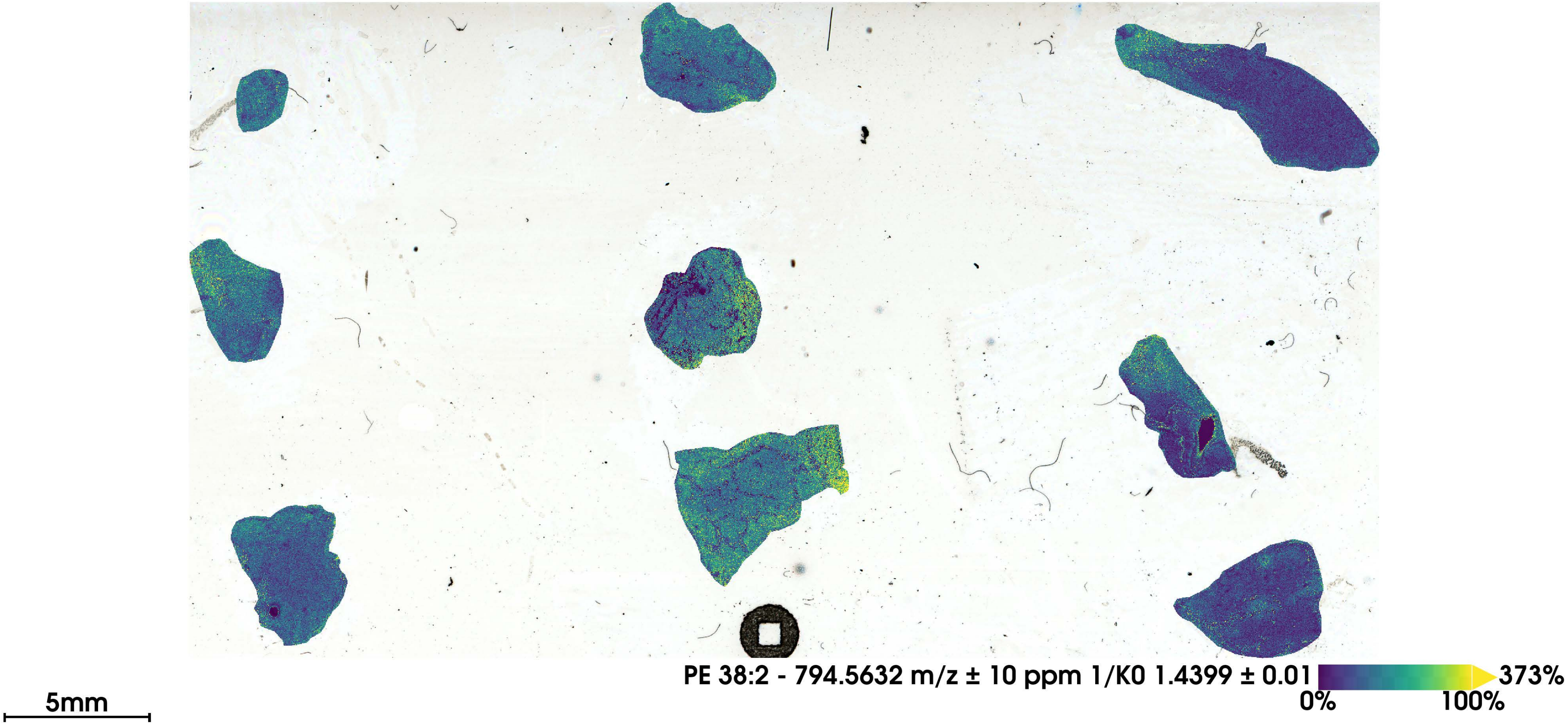
PE 38:2 - 794.5632 m/z ± 10 ppm 1/K0 1.4399 ± 0.01
373%
0%
100%
5mm

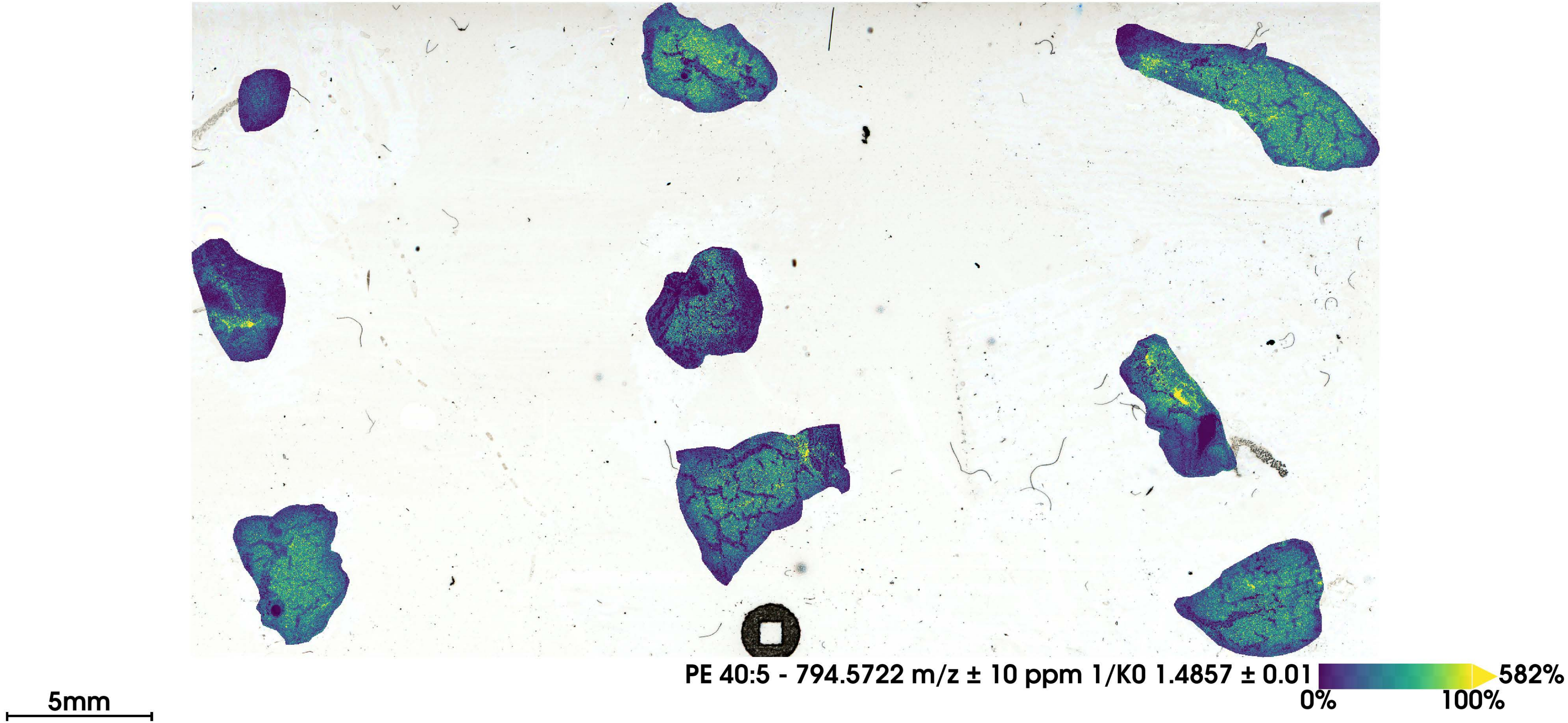
5mm
PE 40:5 - 794.5722 m/z ± 10 ppm 1/K0 1.4857 ± 0.01
582%
0%
100%

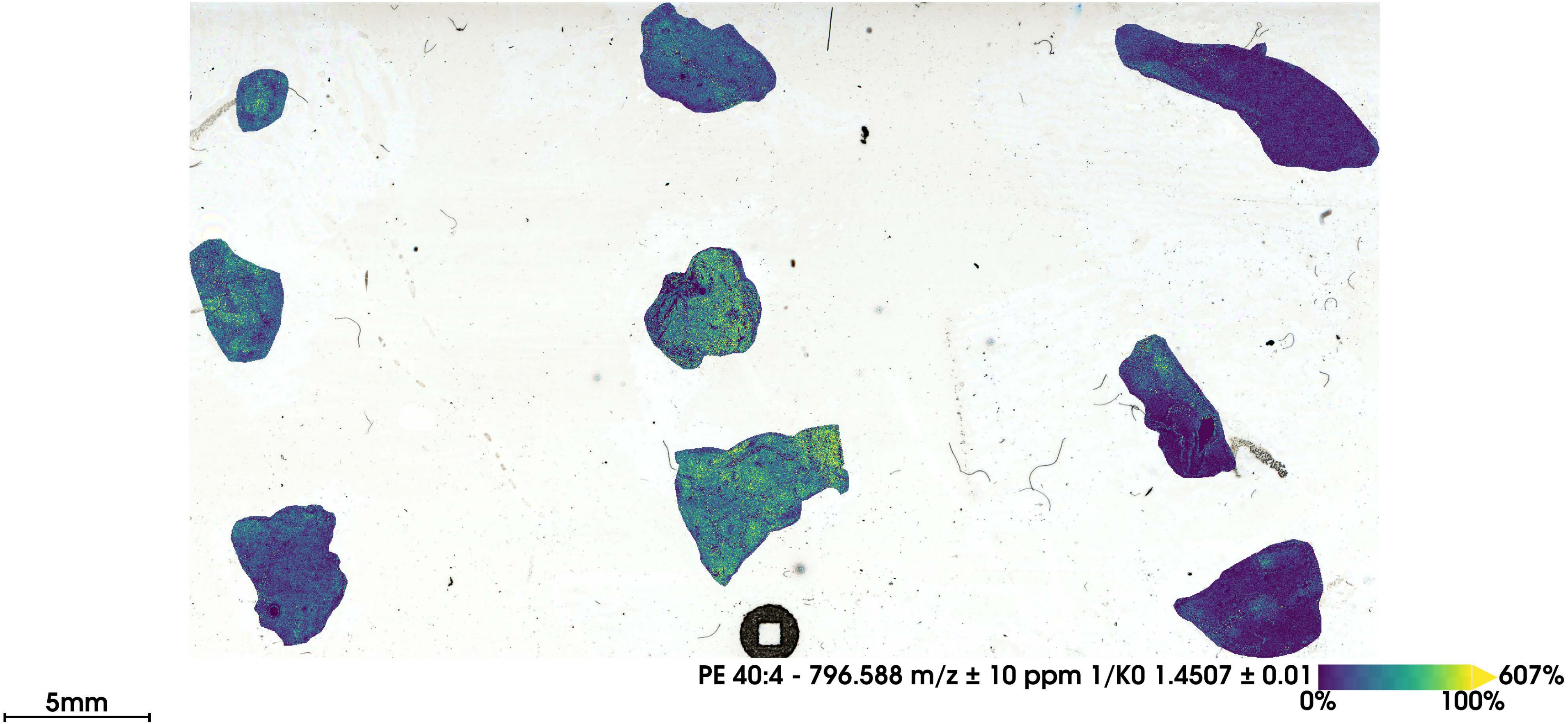
PE 40:4 - 796.588 m/z ± 10 ppm 1/K0 1.4507 ± 0.01
607%
0%
100%
5mm

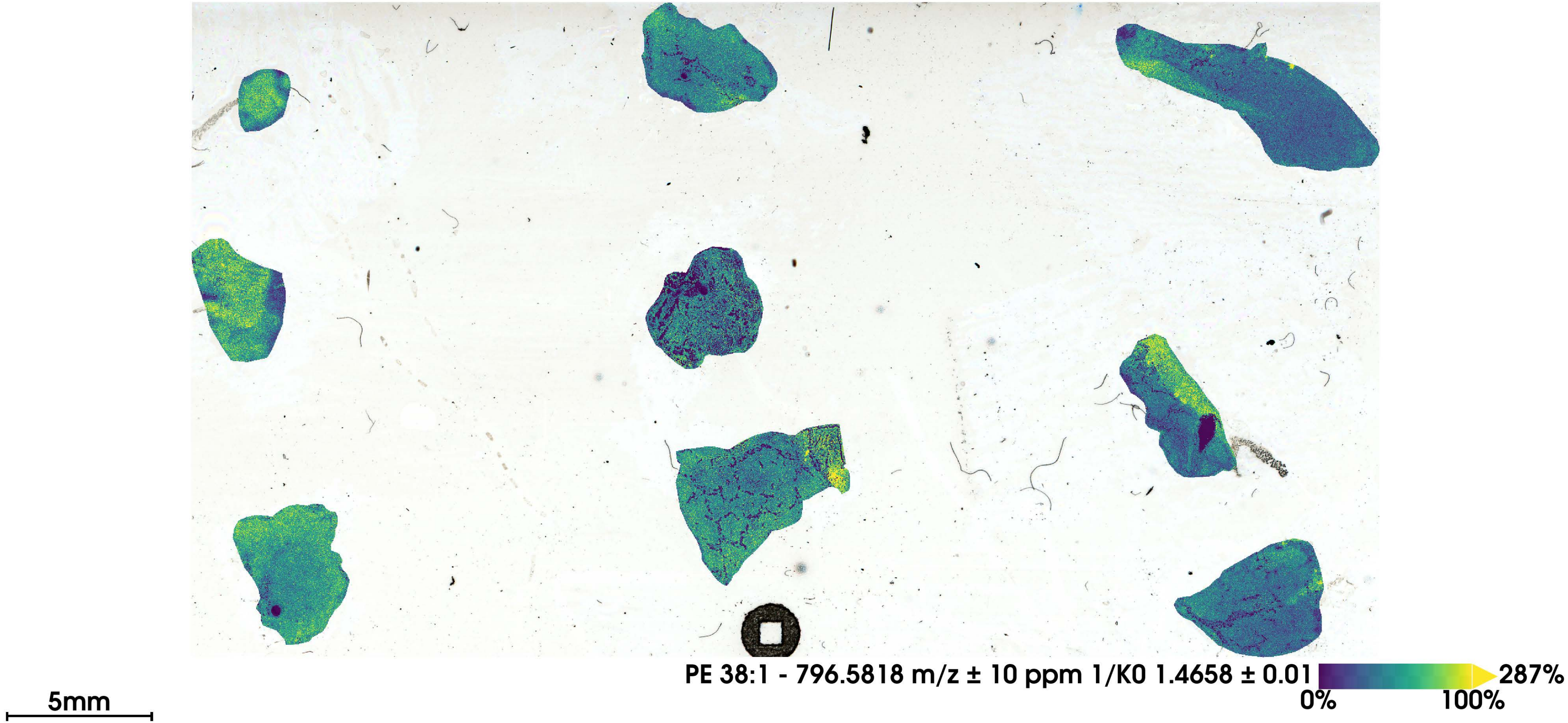
PE 38:1 - 796.5818 m/z ± 10 ppm 1/K0 1.4658 ± 0.01
287%
0%
100%
5mm

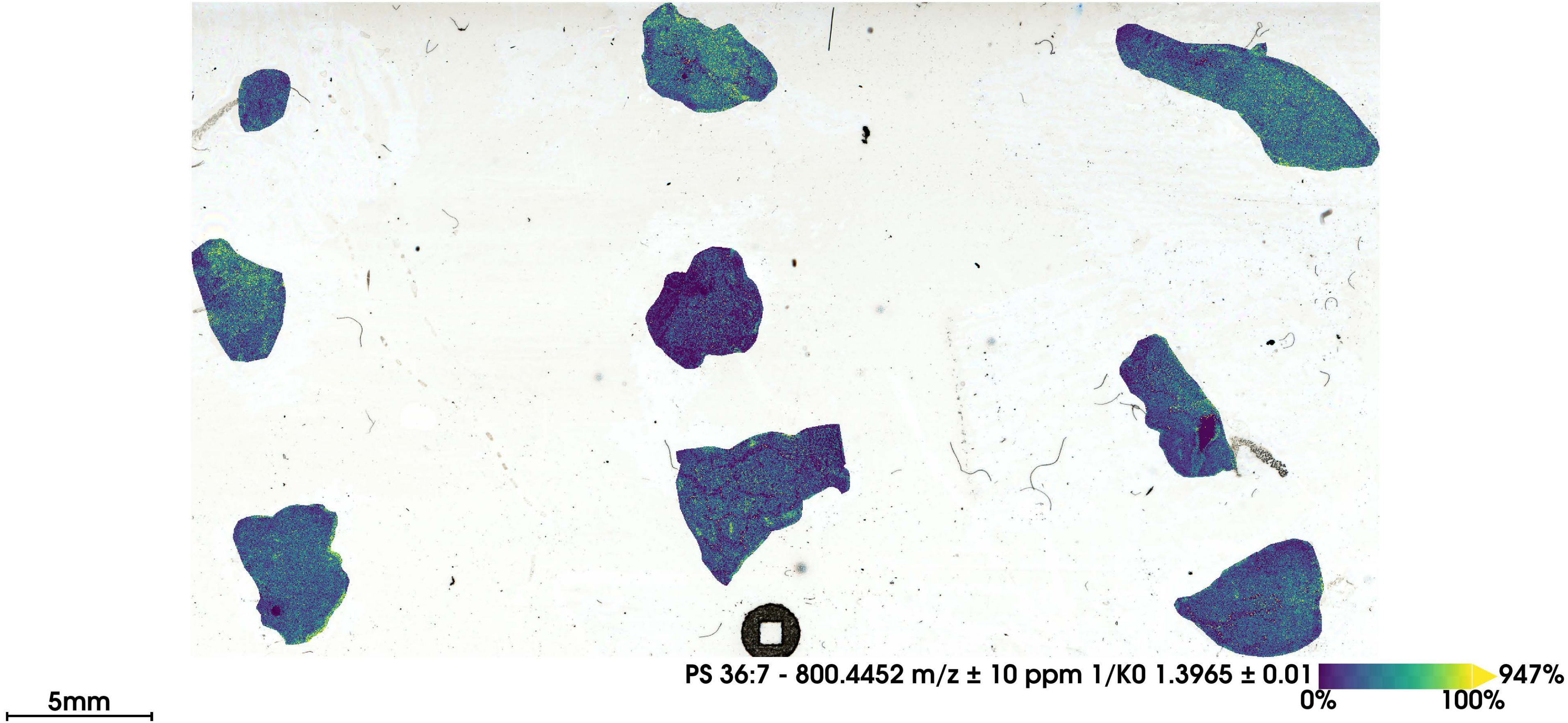
PS 36:7 - 800.4452 m/z ± 10 ppm 1/K0 1.3965 ± 0.01
947%
0%
100%
5mm

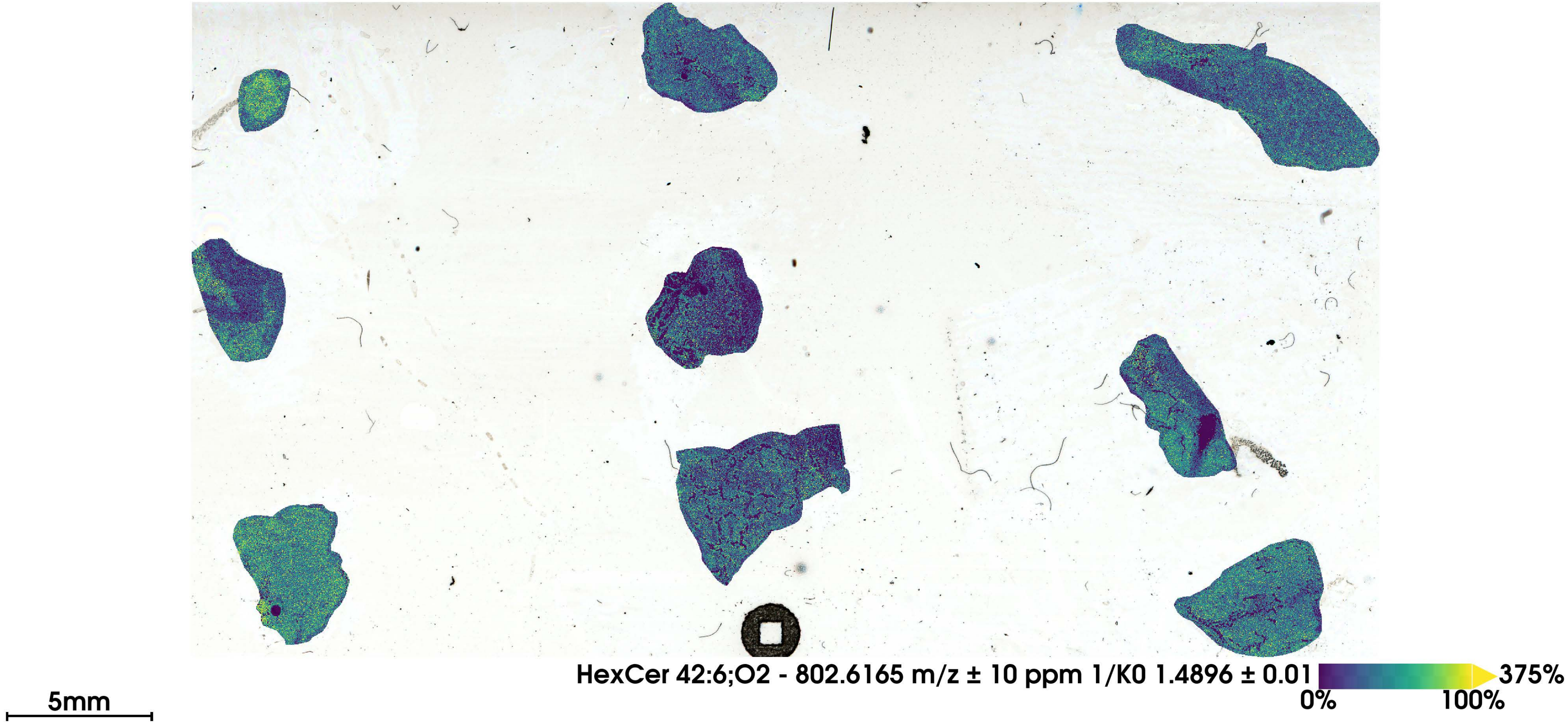
HexCer 42:6;O2 - 802.6165 m/z ± 10 ppm 1/K0 1.4896 ± 0.01
375%
0%
100%
5mm

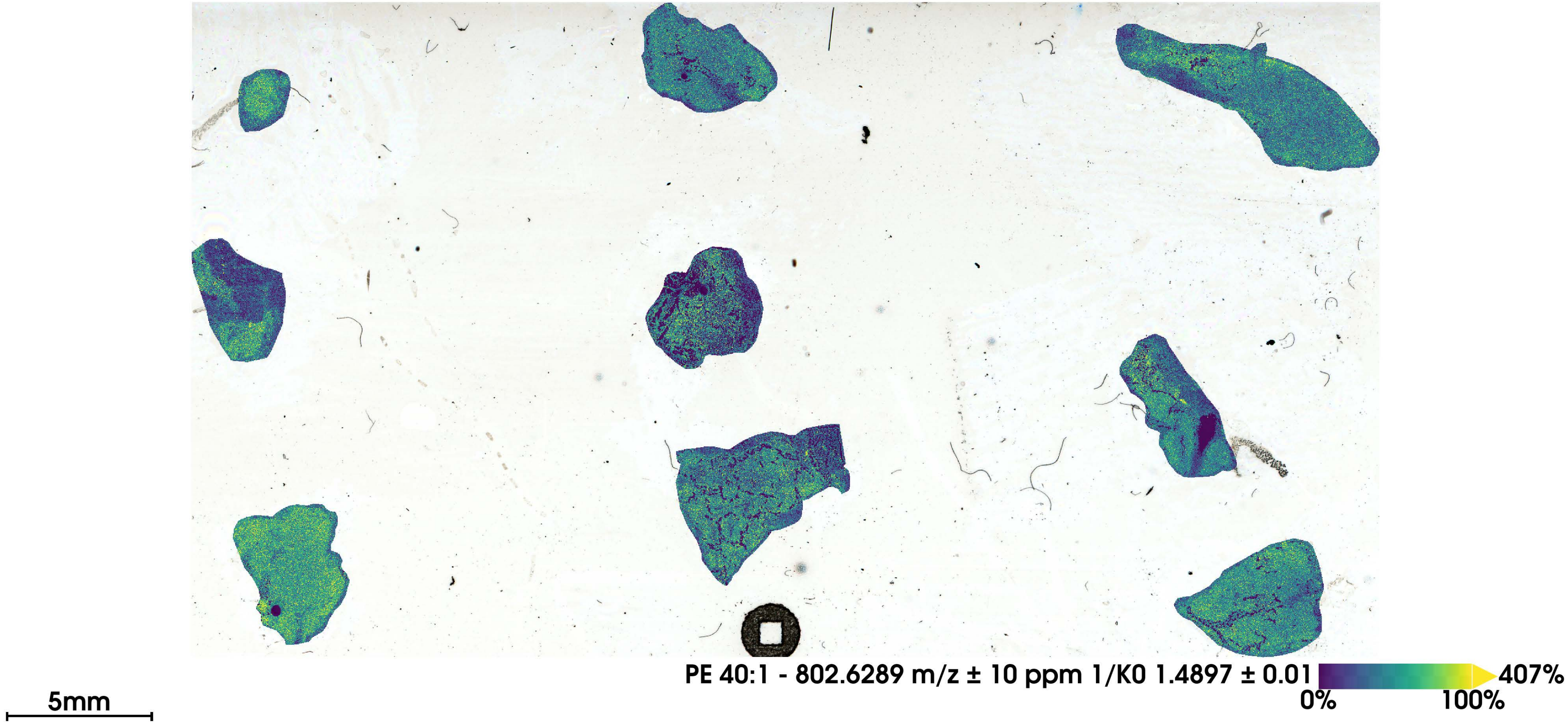

PE 40:1 - 802.6289 m/z ± 10 ppm 1/K0 1.4897 ± 0.01
407%
0%
100%
5mm

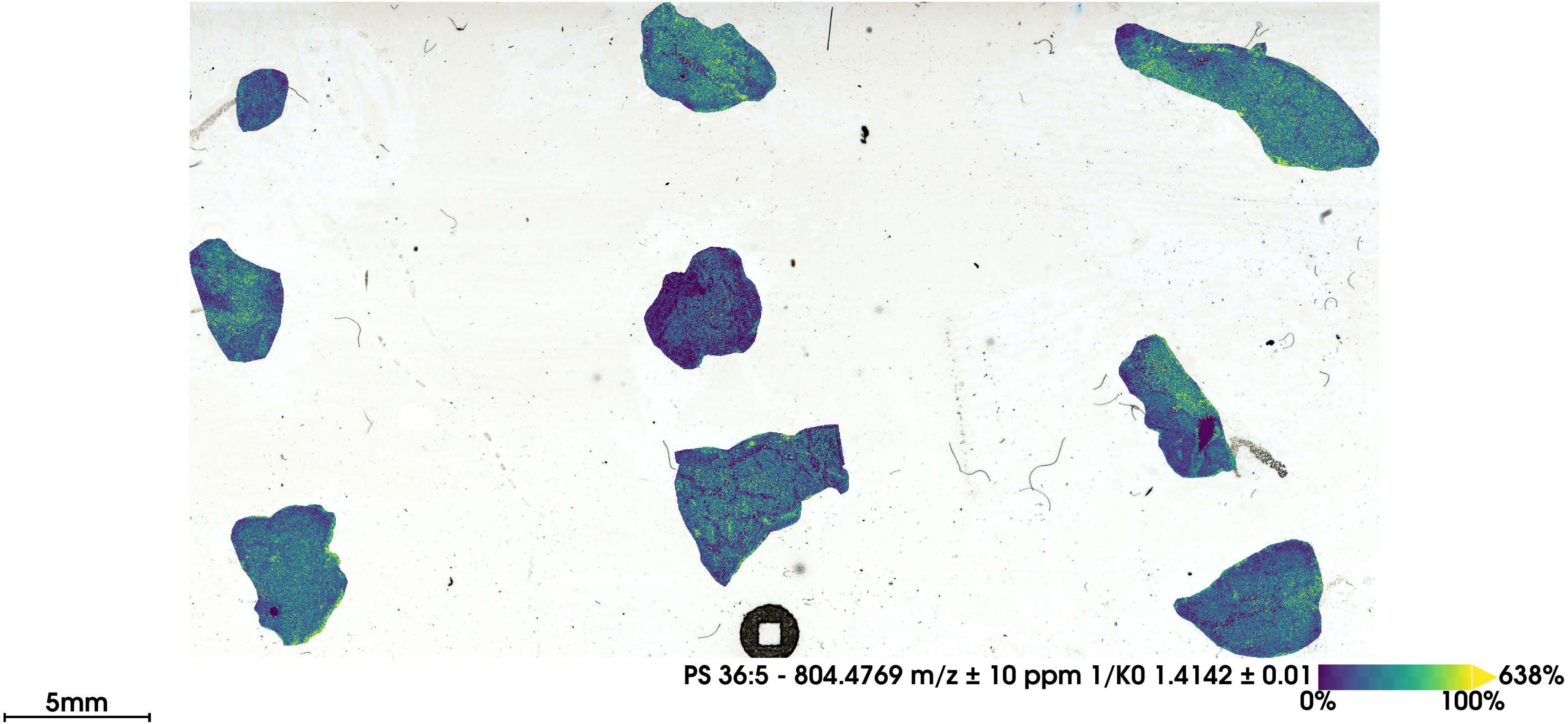
PS 36:5 - 804.4769 m/z ± 10 ppm 1/K0 1.4142 ± 0.01
638%
0%
100%
5mm

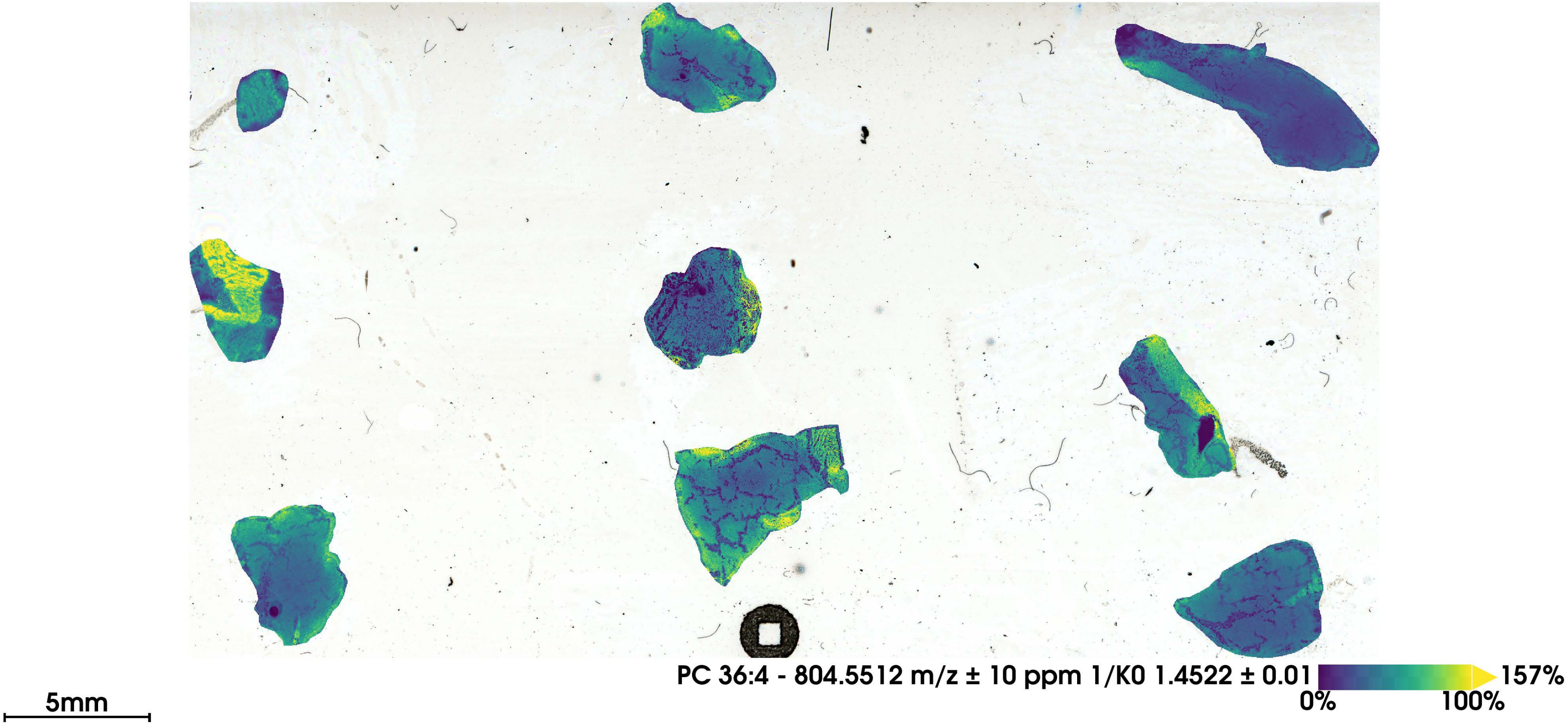
5mm
PC 36:4 - 804.5512 m/z ± 10 ppm 1/K0 1.4522 ± 0.01
157%
0%
100%

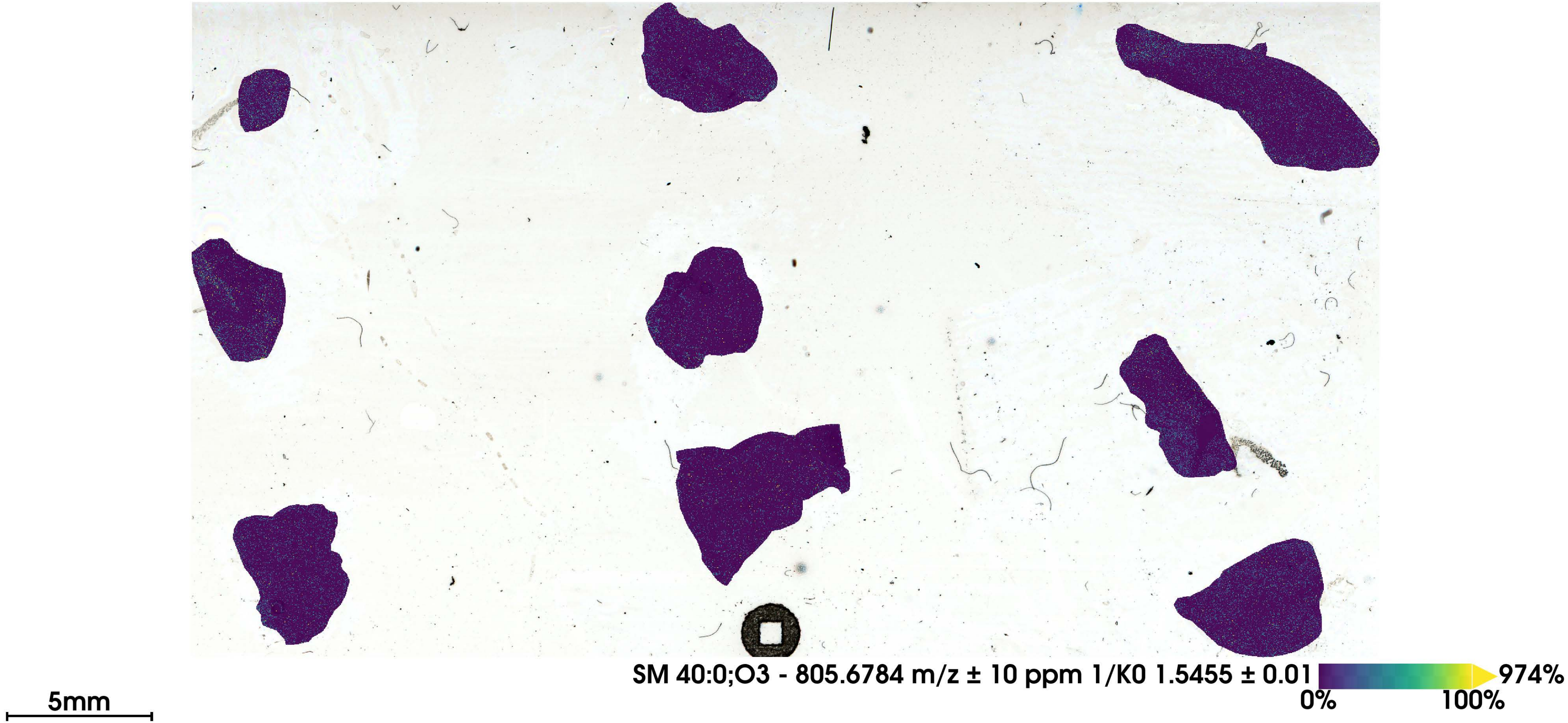
SM 40:0;O3 - 805.6784 m/z ± 10 ppm 1/K0 1.5455 ± 0.01
974%
0%
100%
5mm

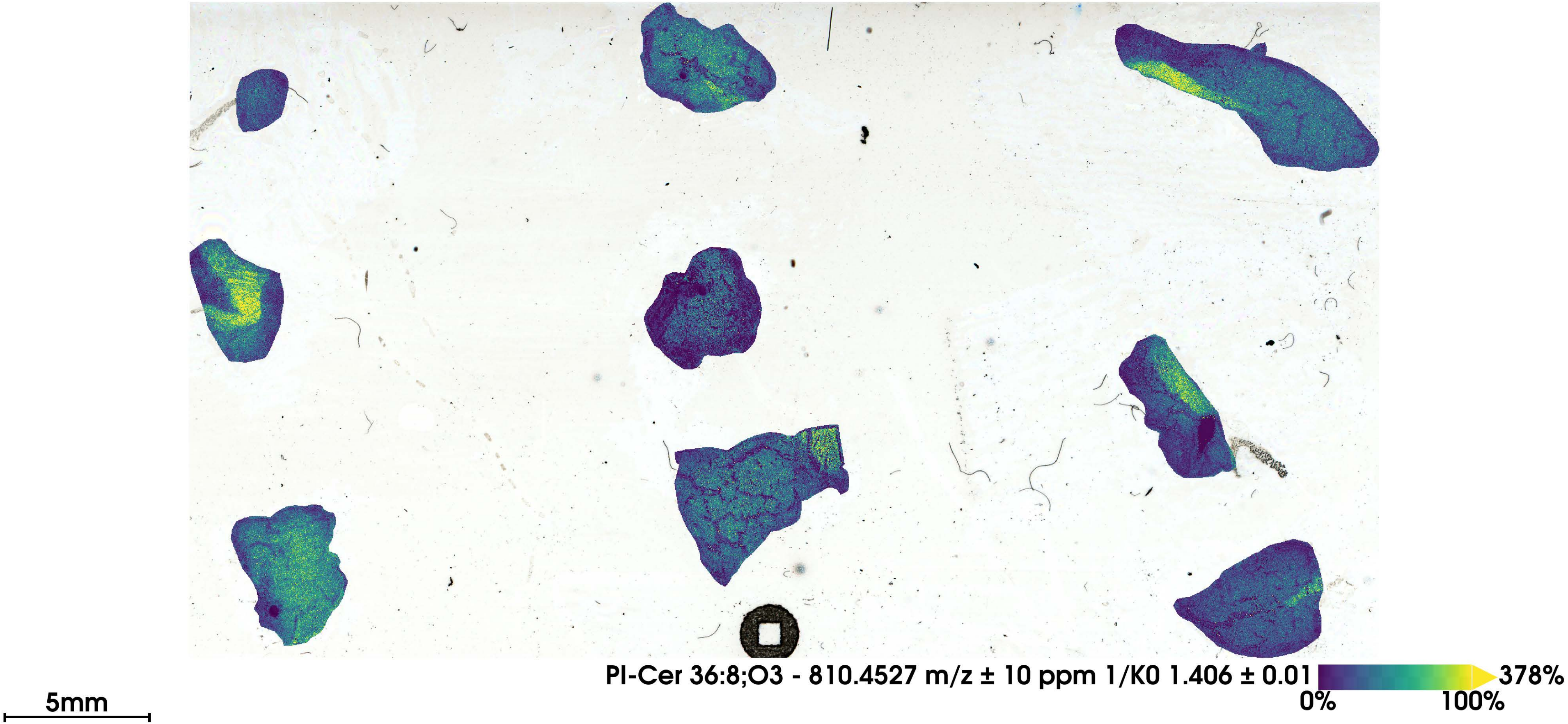
PI-Cer 36:8;O3 - 810.4527 m/z ± 10 ppm 1/K0 1.406 ± 0.01
378%
0%
100%
5mm

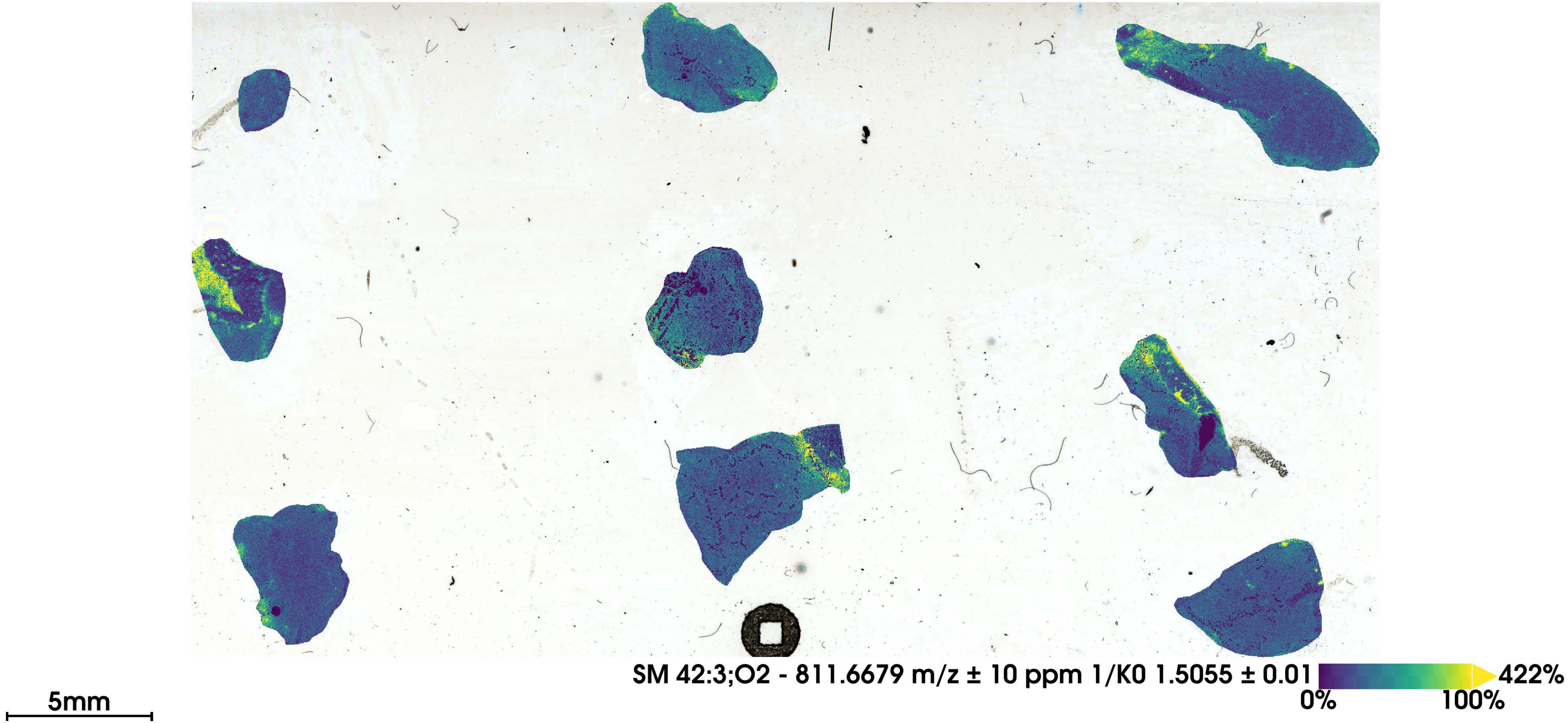

SM 42:3;O2 - 811.6679 m/z ± 10 ppm 1/K0 1.5055 ± 0.01
422%
0%
100%
5mm

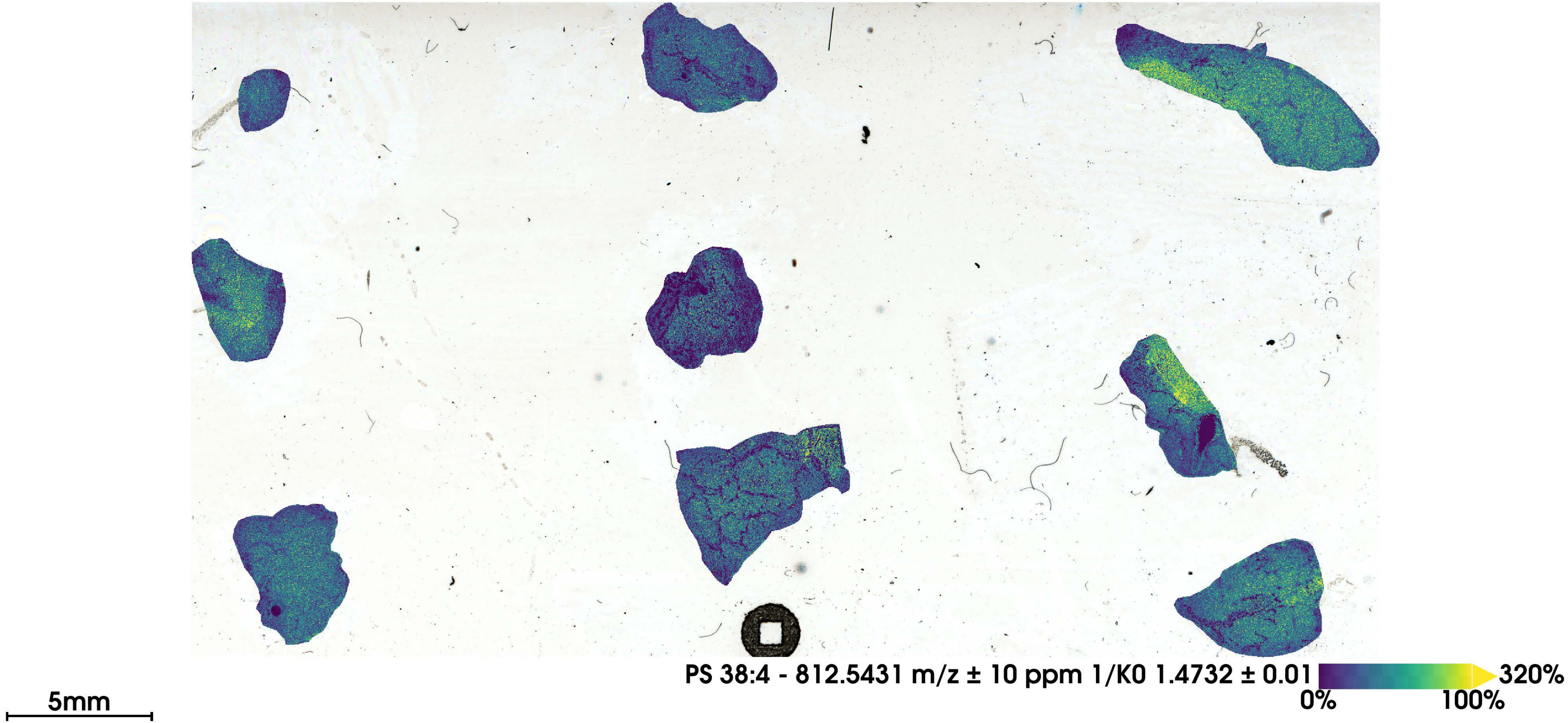
PS 38:4 - 812.5431 m/z ± 10 ppm 1/K0 1.4732 ± 0.01
320%
0%
100%
5mm

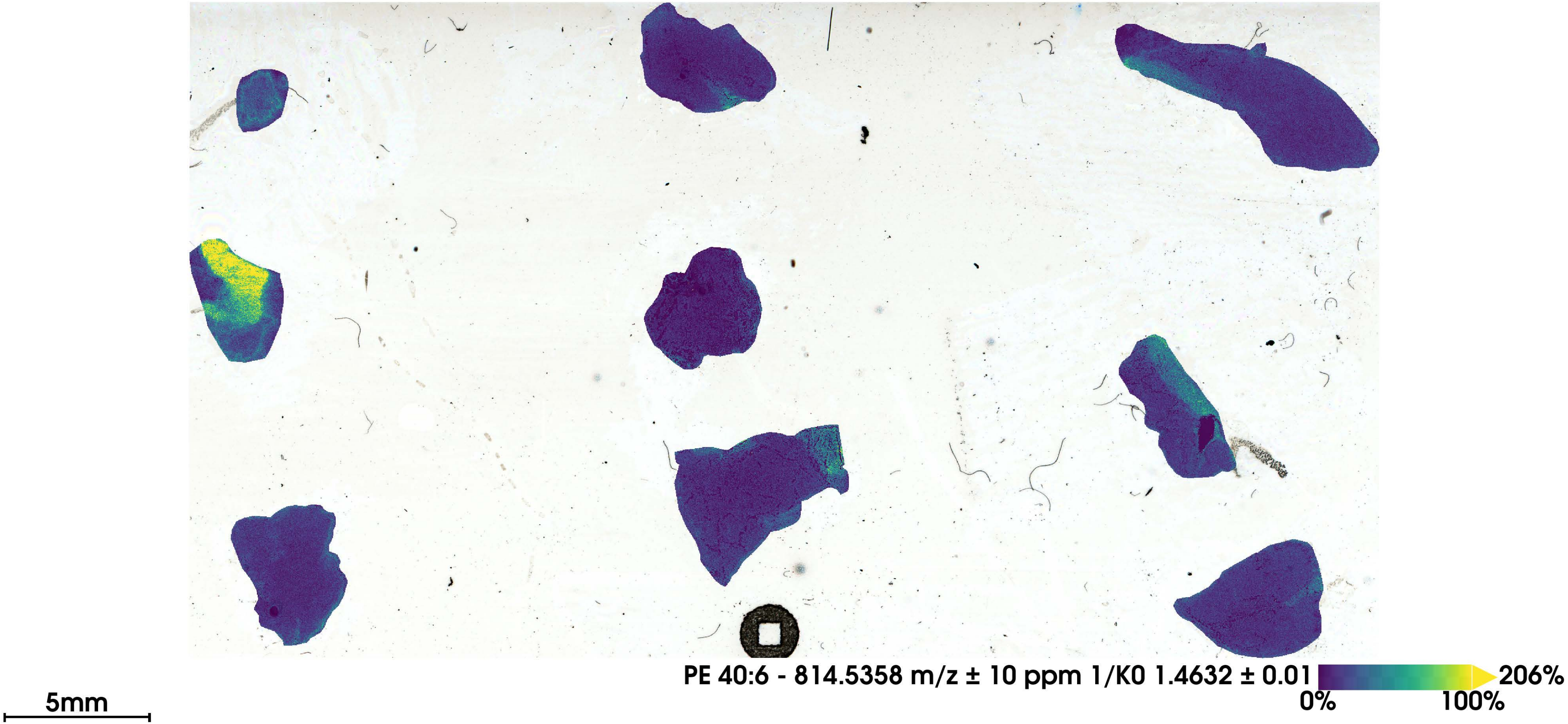
PE 40:6 - 814.5358 m/z ± 10 ppm 1/K0 1.4632 ± 0.01
206%
0%
100%
5mm

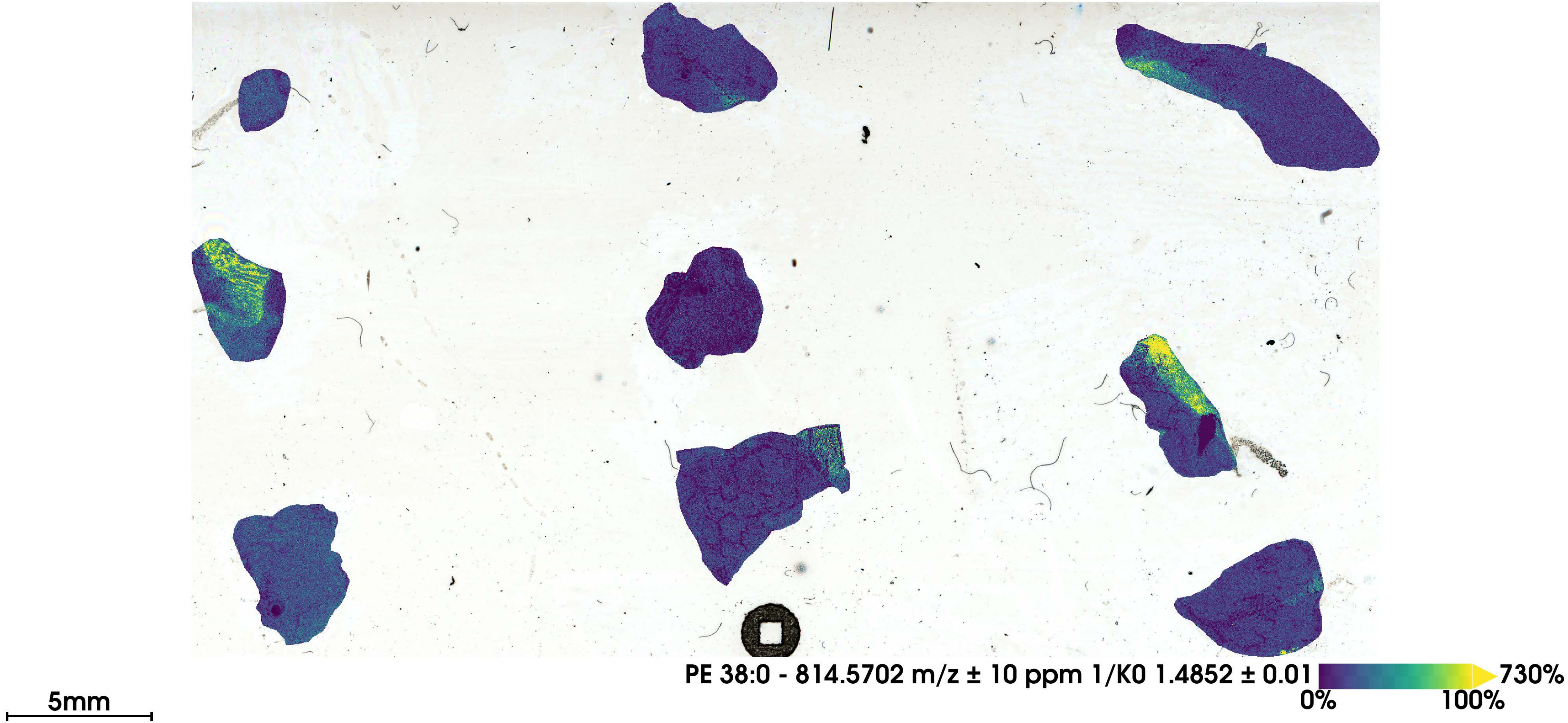
PE 38:0 - 814.5702 m/z ± 10 ppm 1/K0 1.4852 ± 0.01
730%
0%
100%
5mm

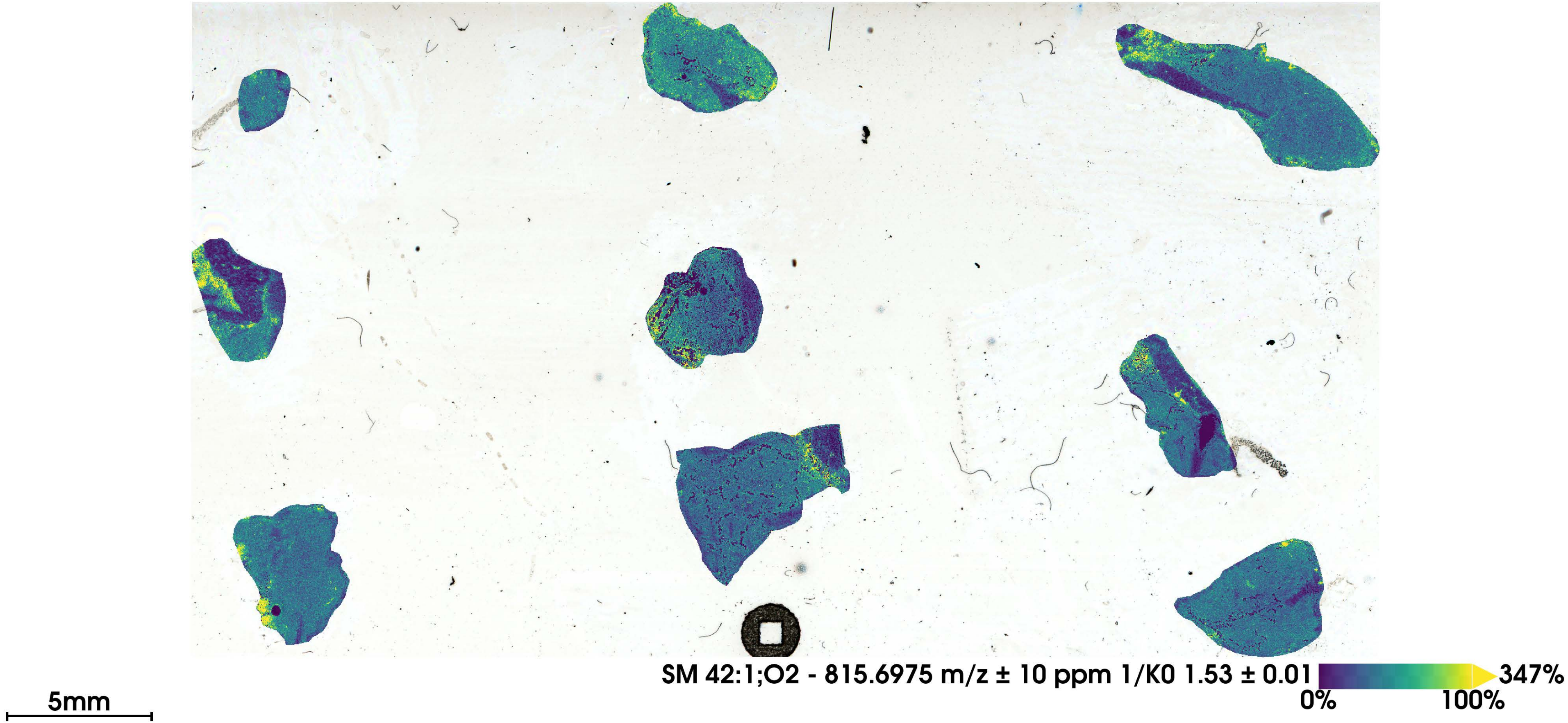
SM 42:1;O2 - 815.6975 m/z ± 10 ppm 1/K0 1.53 ± 0.01
347%
0%
100%
5mm

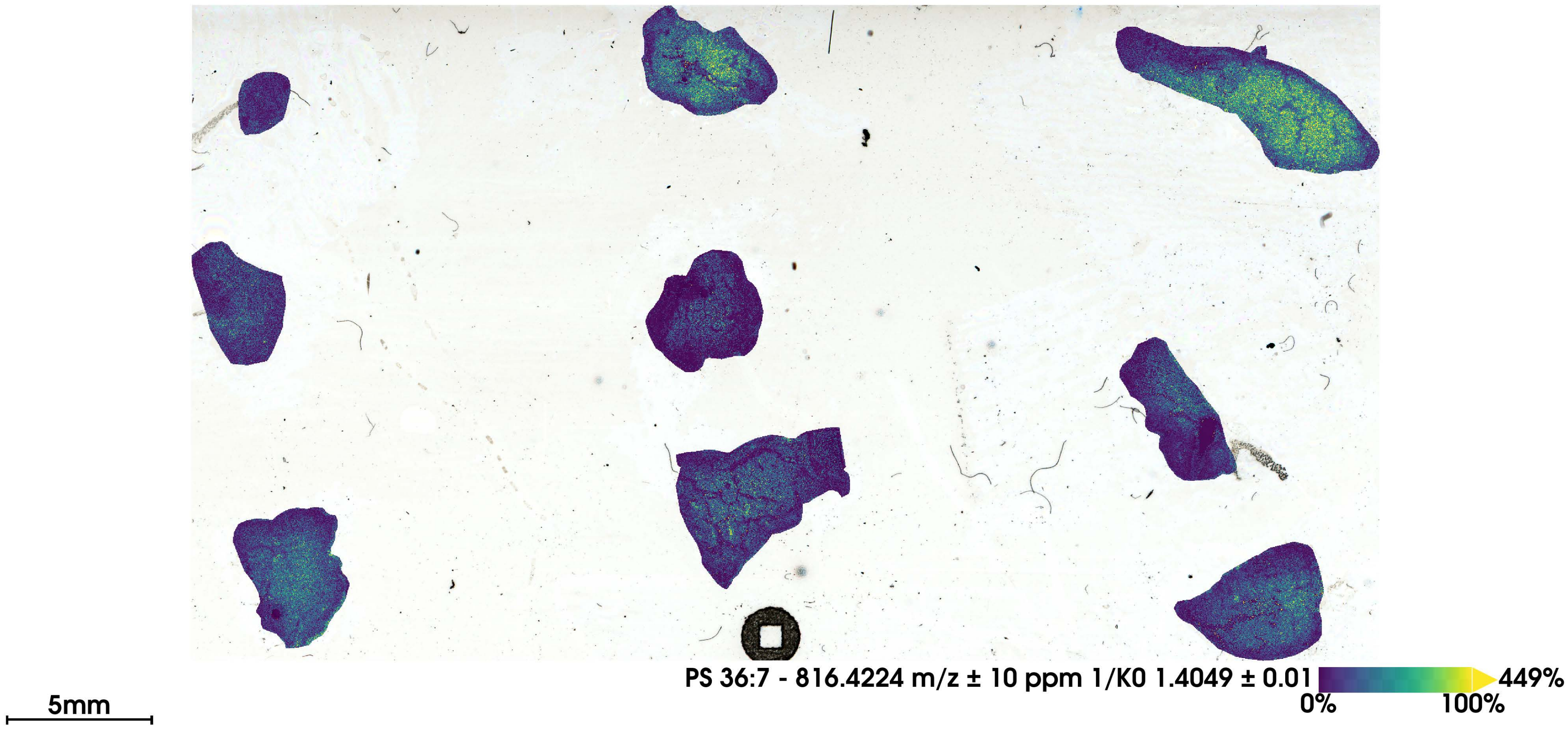
PS 36:7 - 816.4224 m/z ± 10 ppm 1/K0 1.4049 ± 0.01
449%
0%
100%
5mm

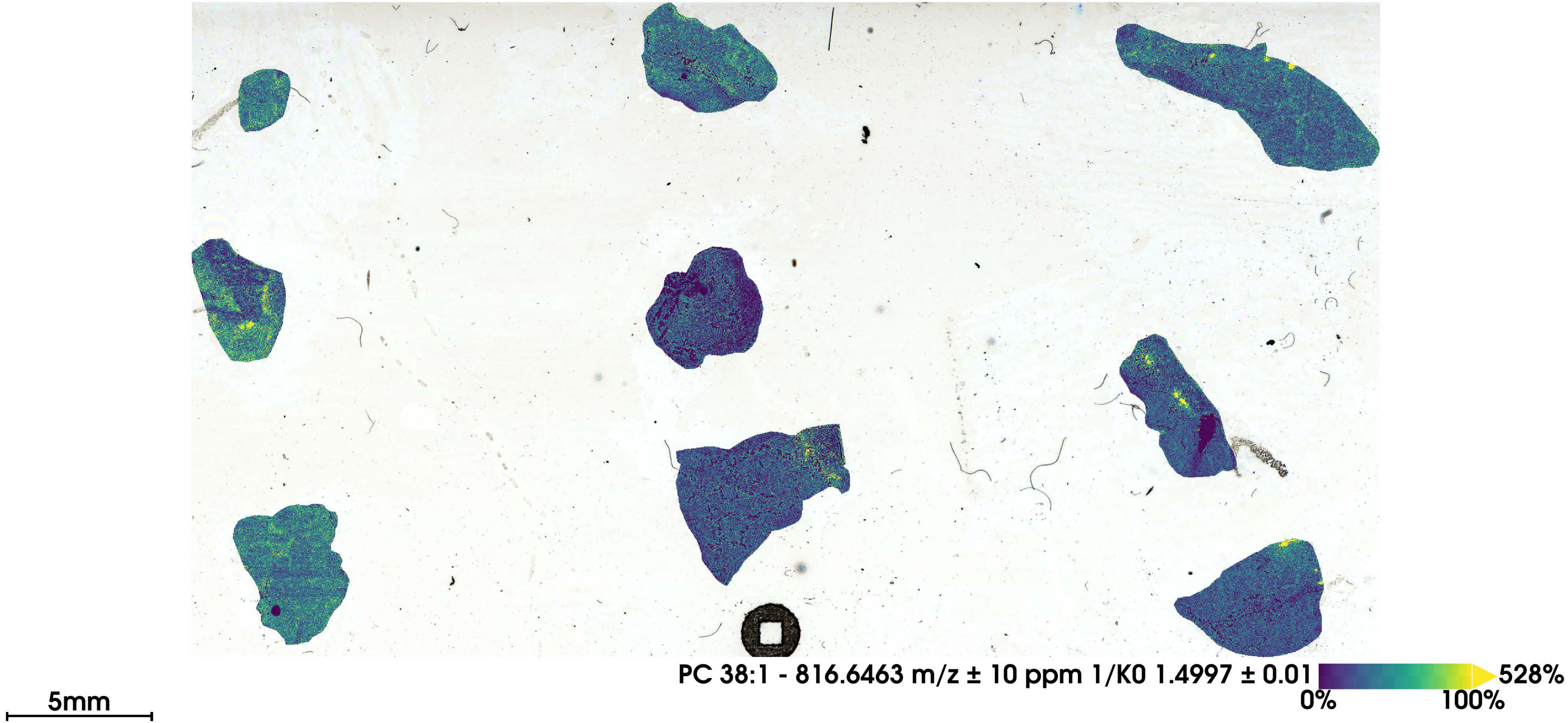
PC 38:1 - 816.6463 m/z ± 10 ppm 1/K0 1.4997 ± 0.01
528%
0%
100%
5mm

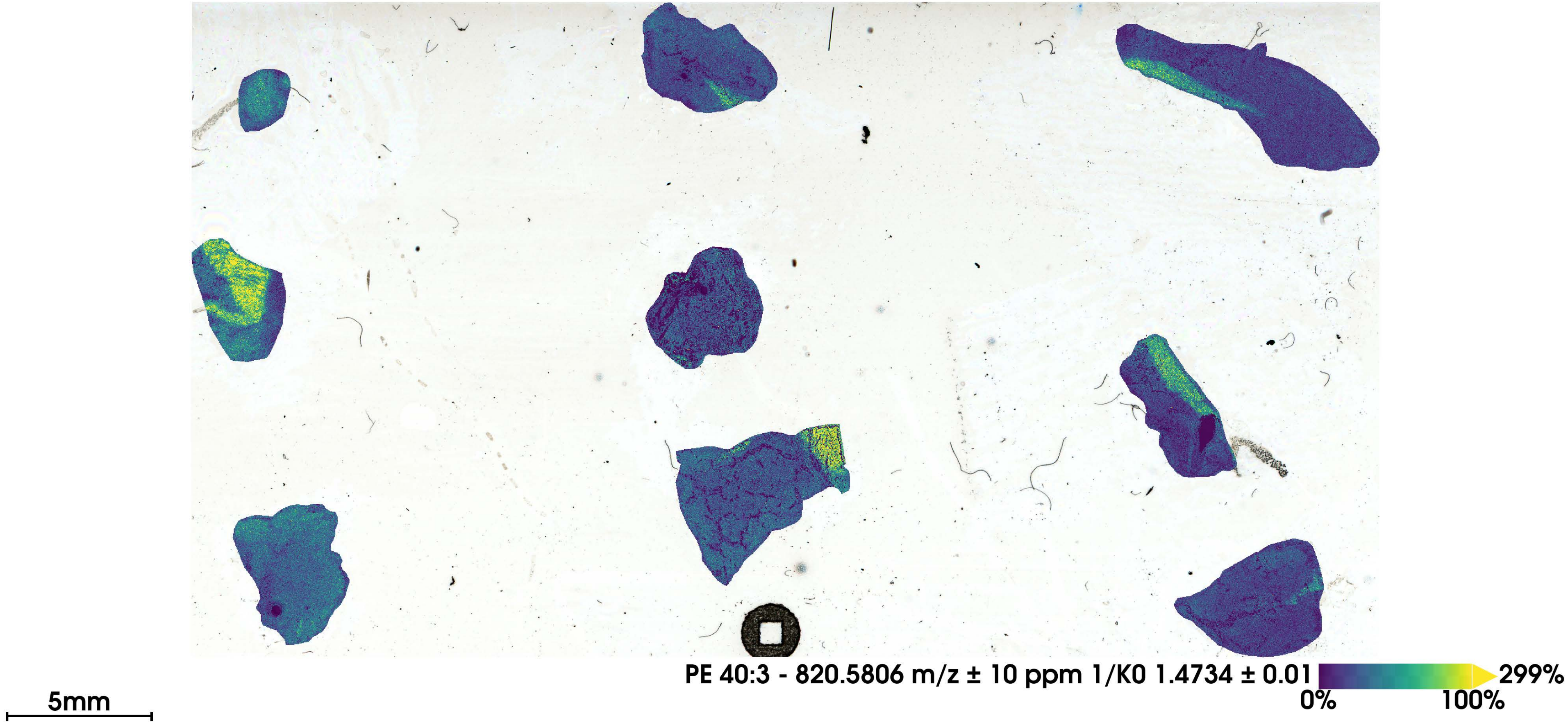
PE 40:3 - 820.5806 m/z ± 10 ppm 1/K0 1.4734 ± 0.01
299%
0%
100%
5mm

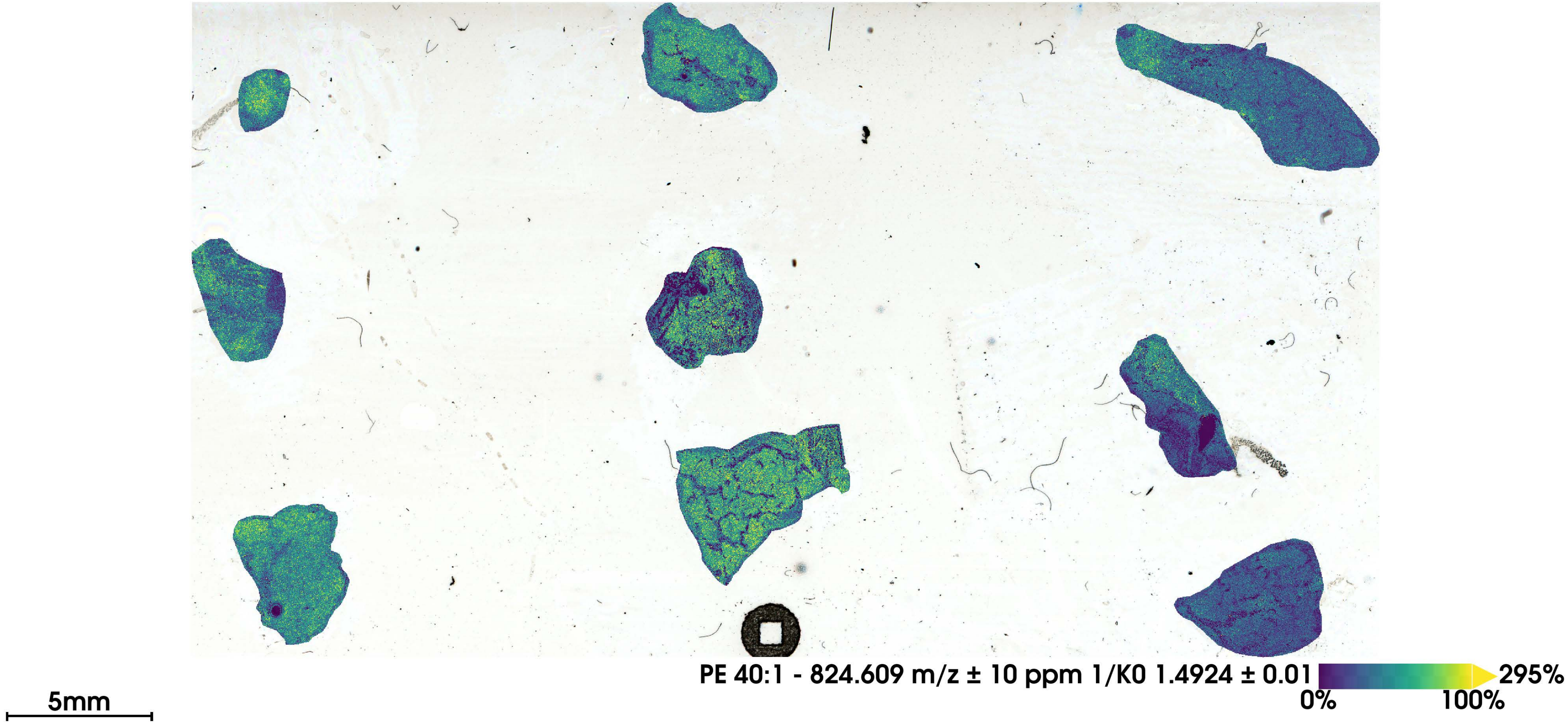
PE 40:1 - 824.609 m/z ± 10 ppm 1/K0 1.4924 ± 0.01
295%
0%
100%
5mm

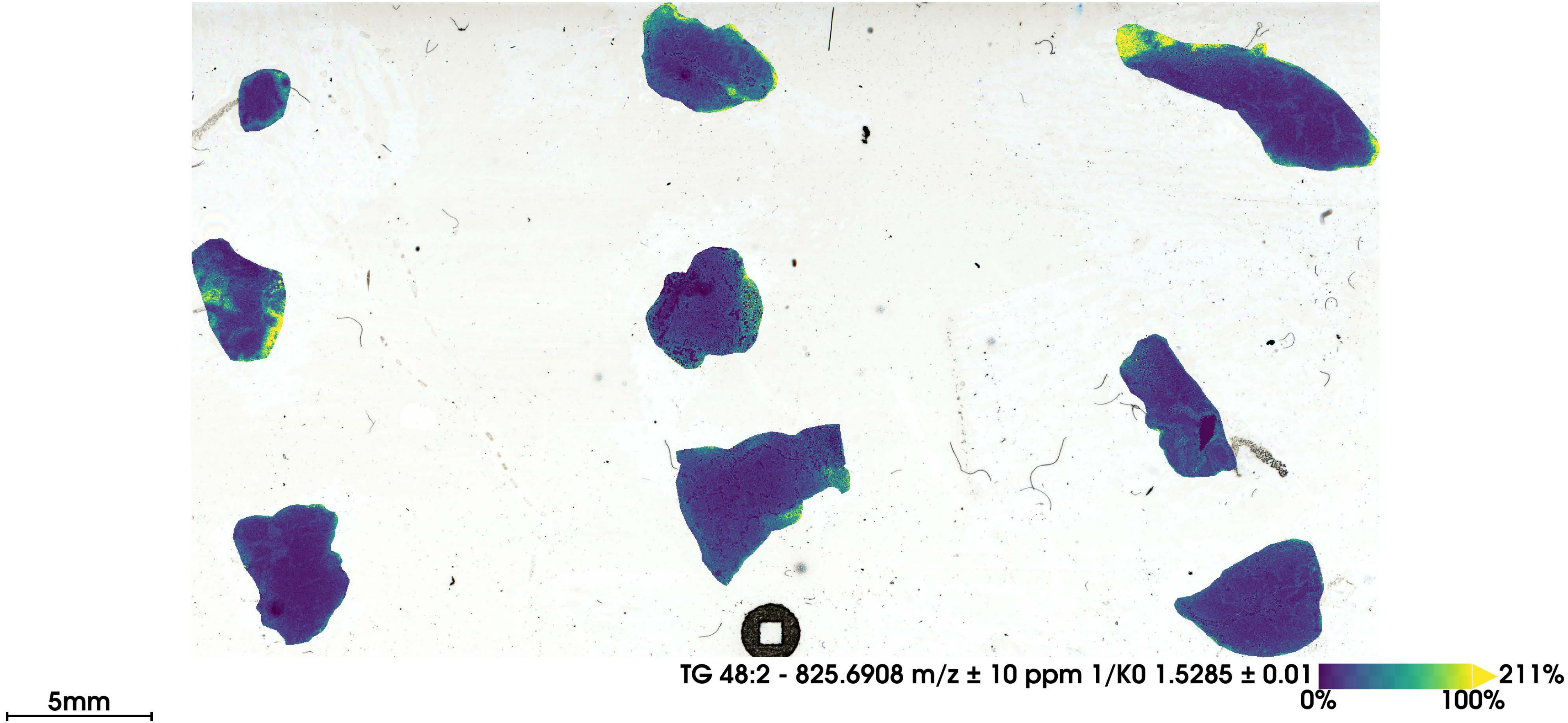
TG 48:2 - 825.6908 m/z ± 10 ppm 1/K0 1.5285 ± 0.01
211%
0%
100%
5mm

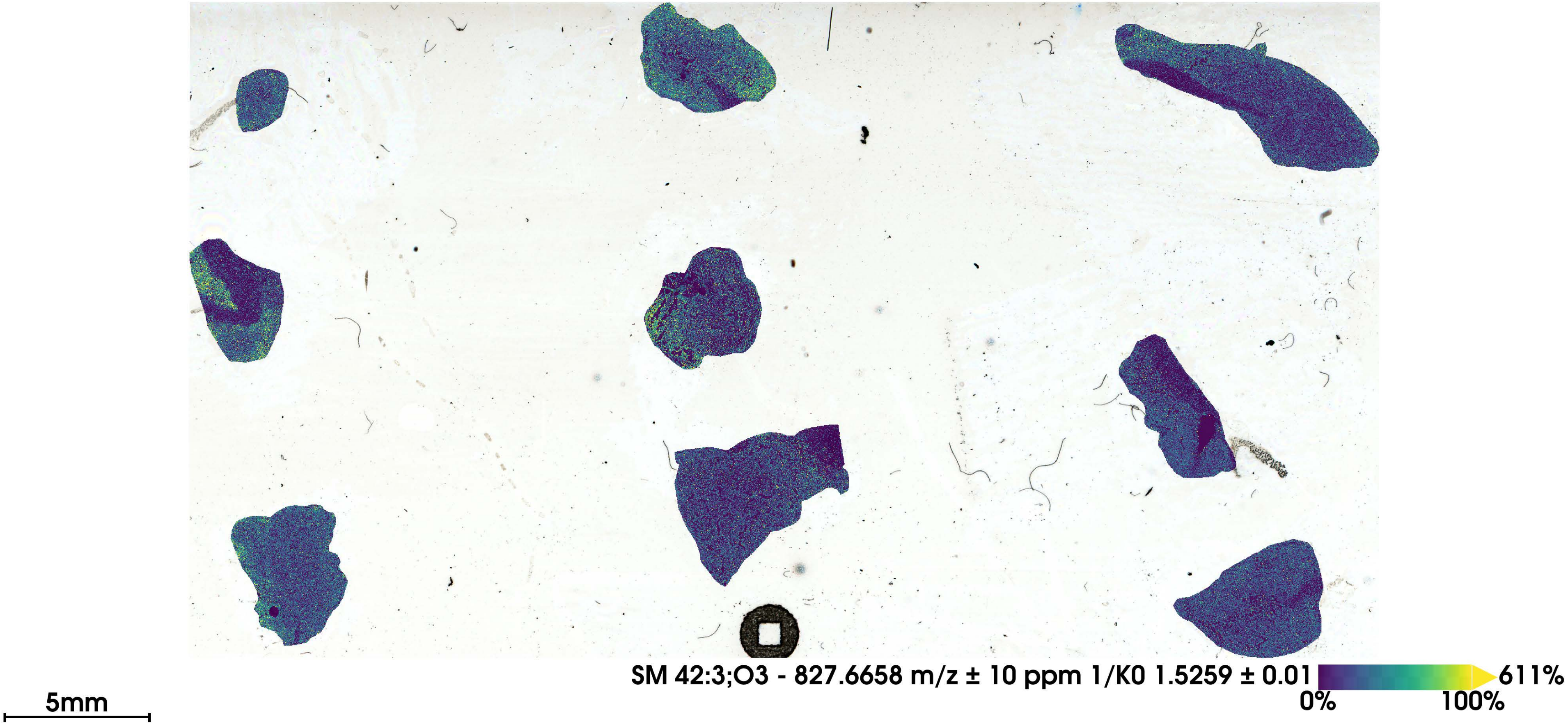

SM 42:3;O3 - 827.6658 m/z ± 10 ppm 1/K0 1.5259 ± 0.01
611%
0%
100%
5mm

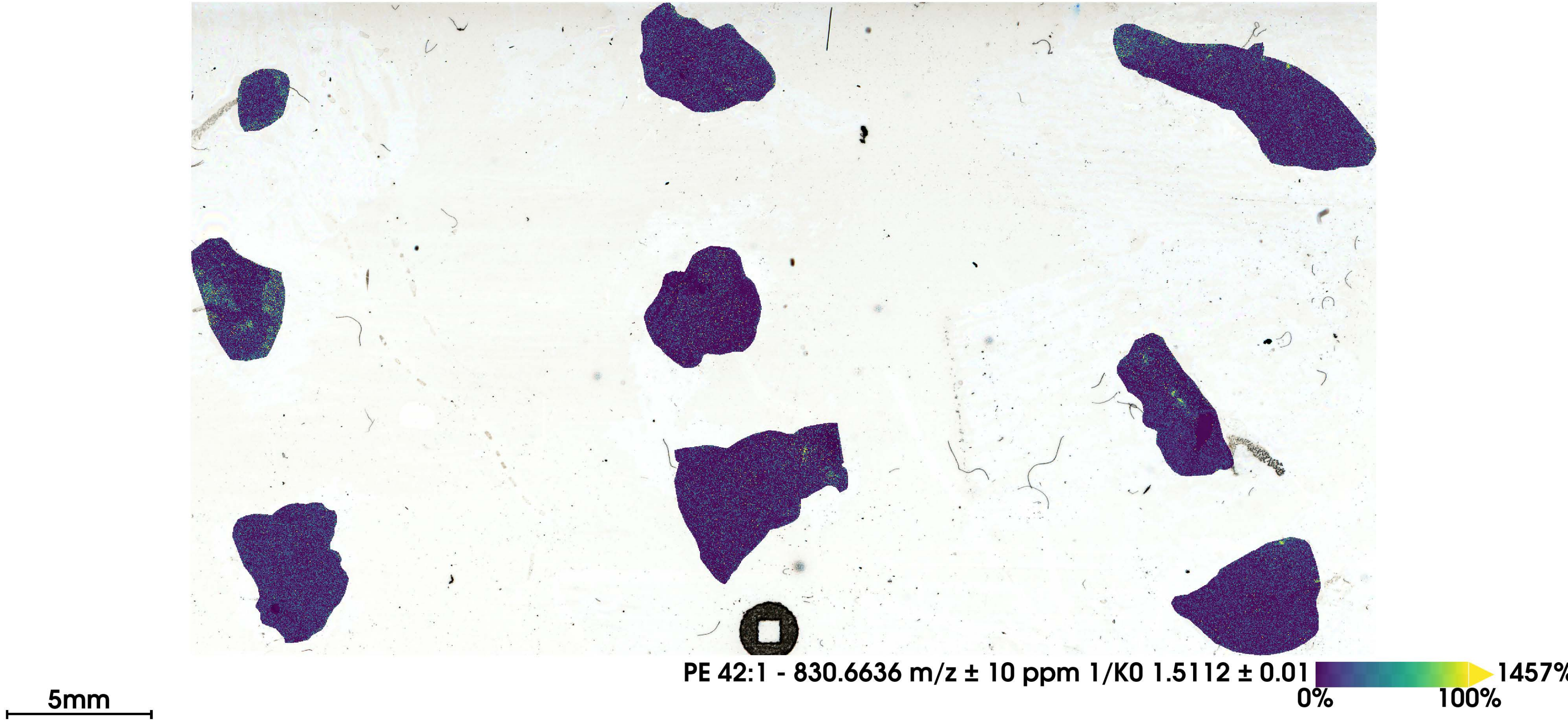
PE 42:1 - 830.6636 m/z ± 10 ppm 1/K0 1.5112 ± 0.01
1457%
0%
100%
5mm

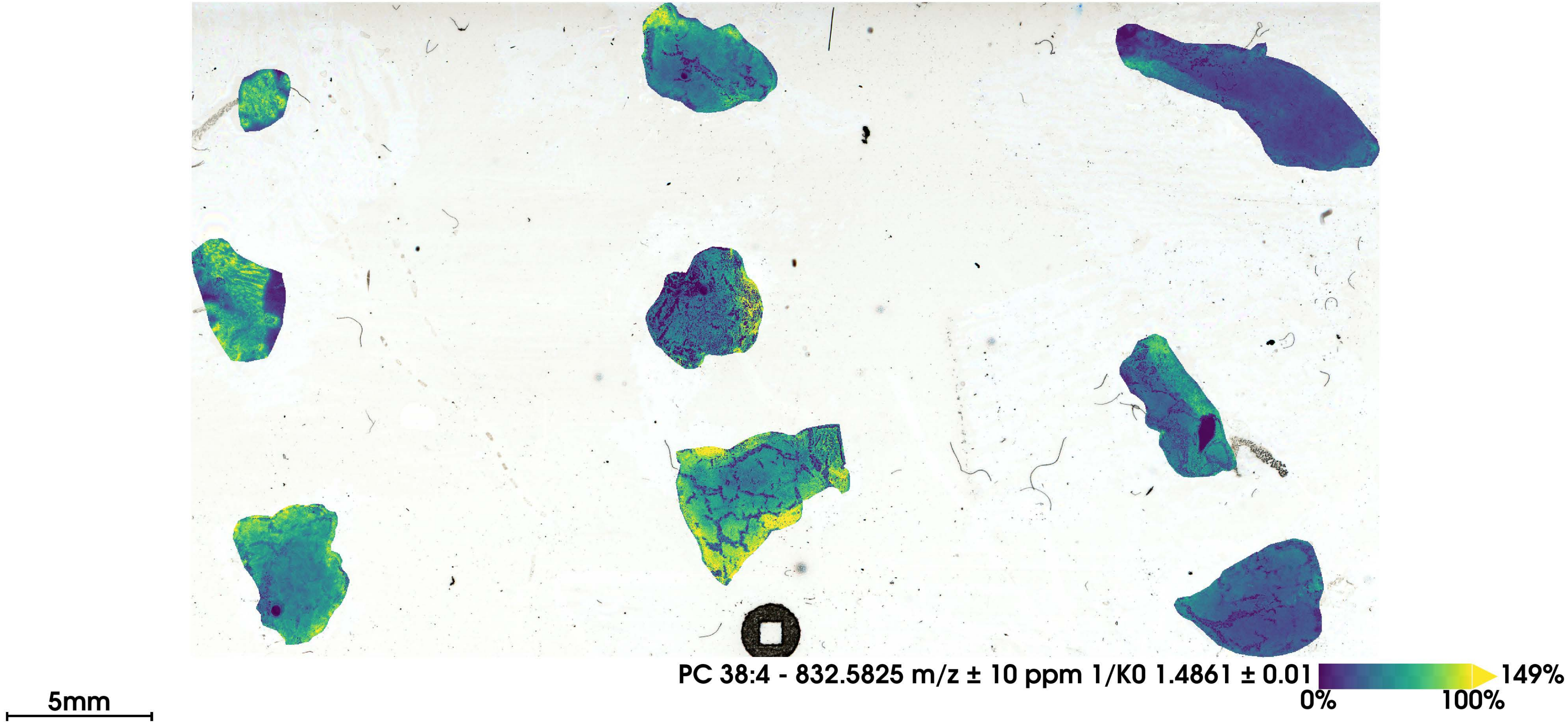

PC 38:4 - 832.5825 m/z ± 10 ppm 1/K0 1.4861 ± 0.01
149%
0%
100%
5mm

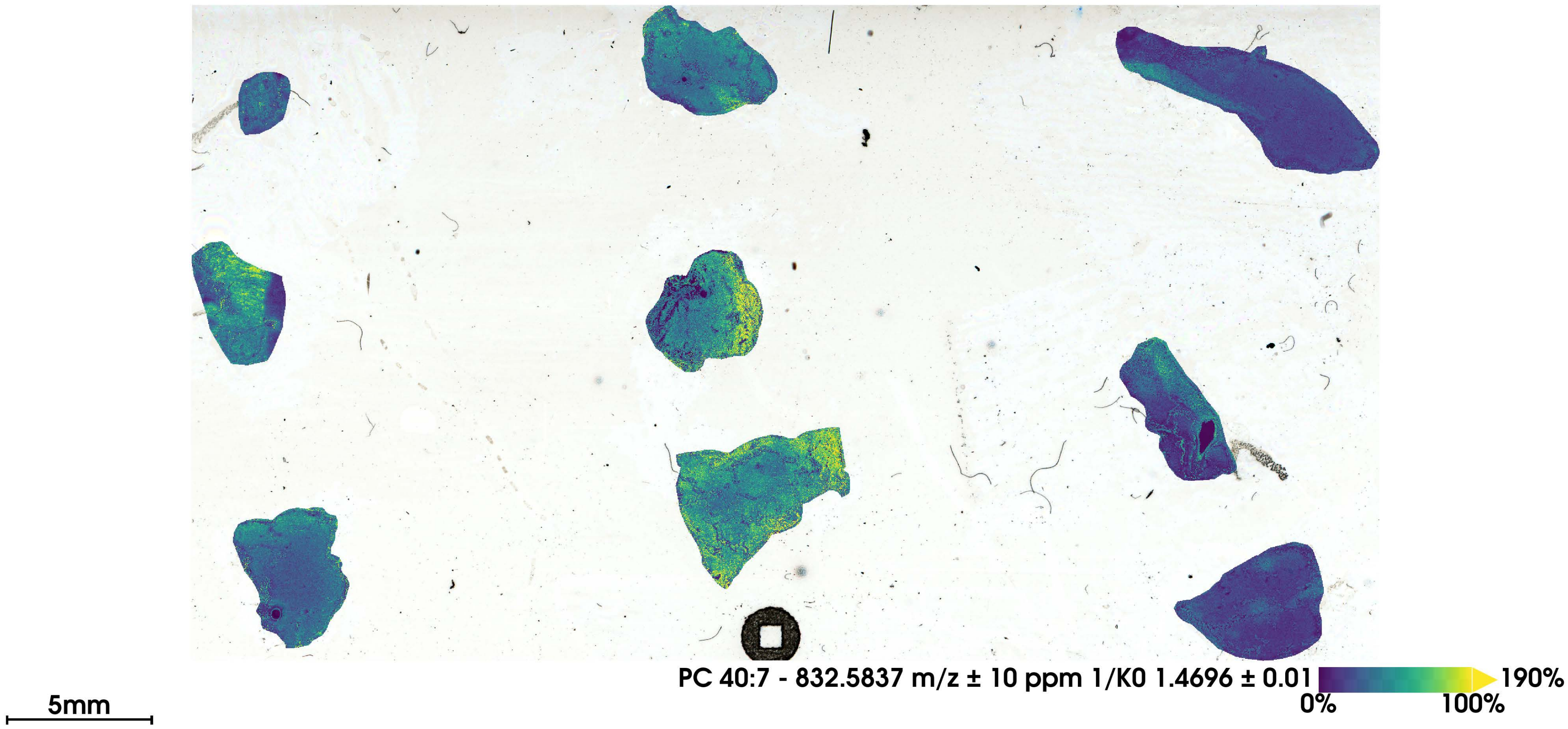
PC 40:7 - 832.5837 m/z ± 10 ppm 1/K0 1.4696 ± 0.01
190%
0%
100%
5mm

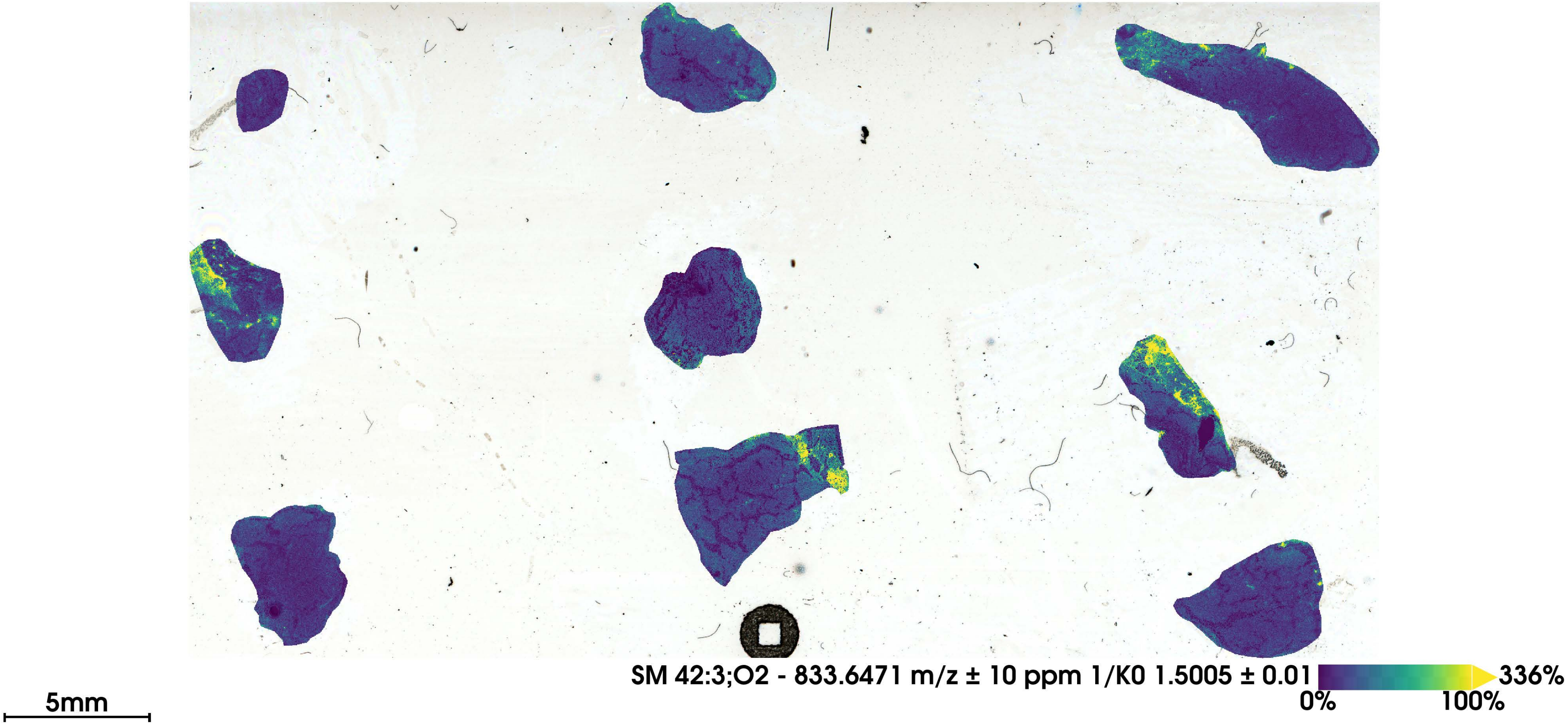

5mm
SM 42:3;O2 - 833.6471 m/z ± 10 ppm 1/K0 1.5005 ± 0.01
336%
0%
100%

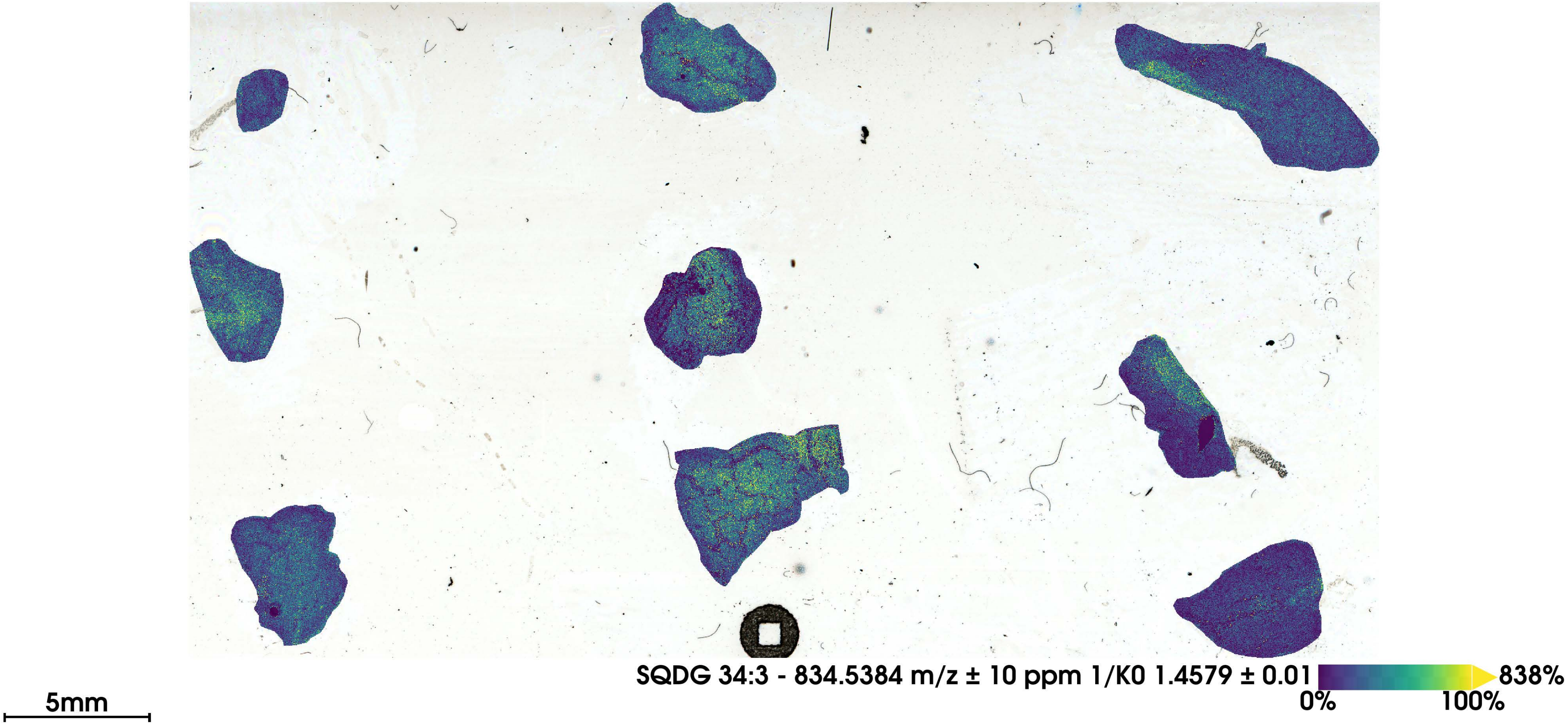
SQDG 34:3 - 834.5384 m/z ± 10 ppm 1/K0 1.4579 ± 0.01
838%
0%
100%
5mm

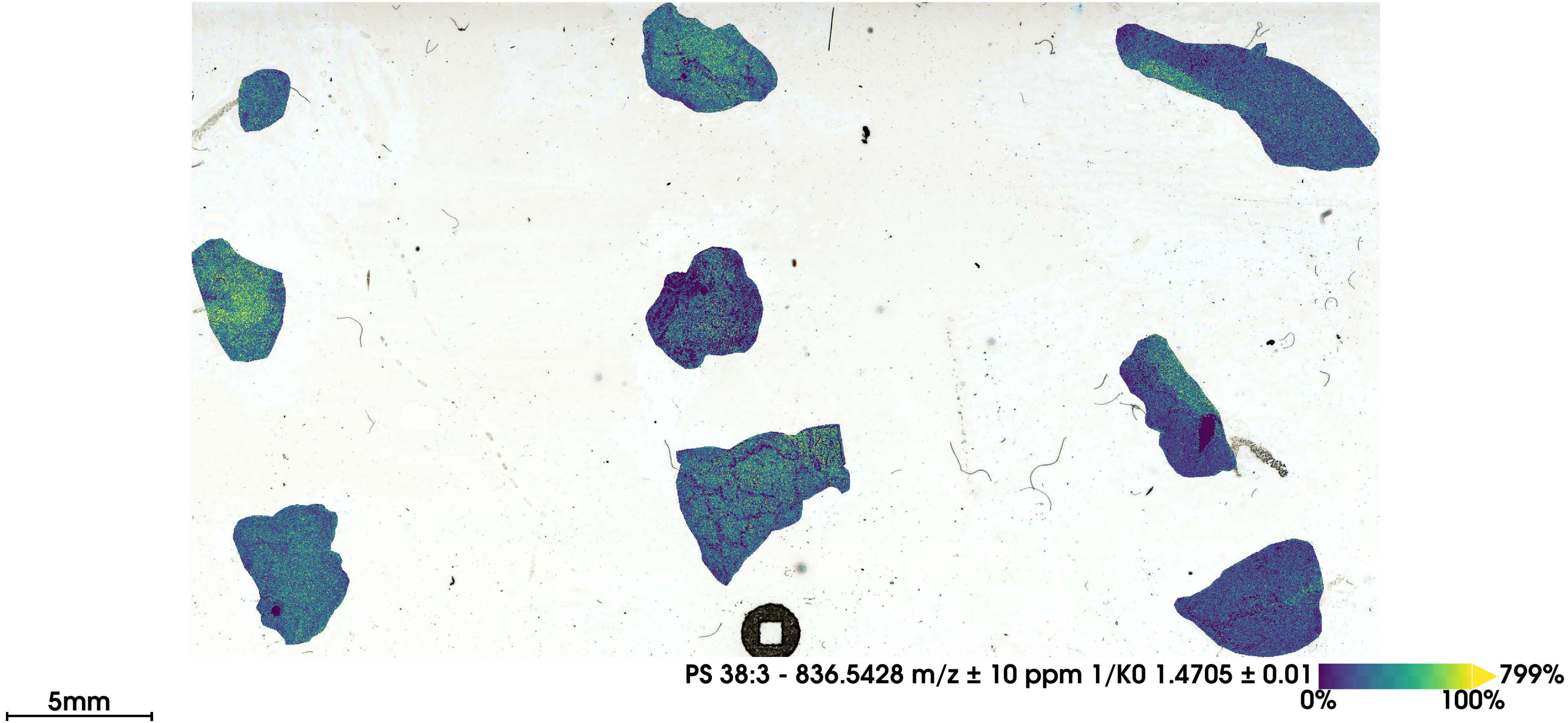
PS 38:3 - 836.5428 m/z ± 10 ppm 1/K0 1.4705 ± 0.01
799%
0%
100%
5mm

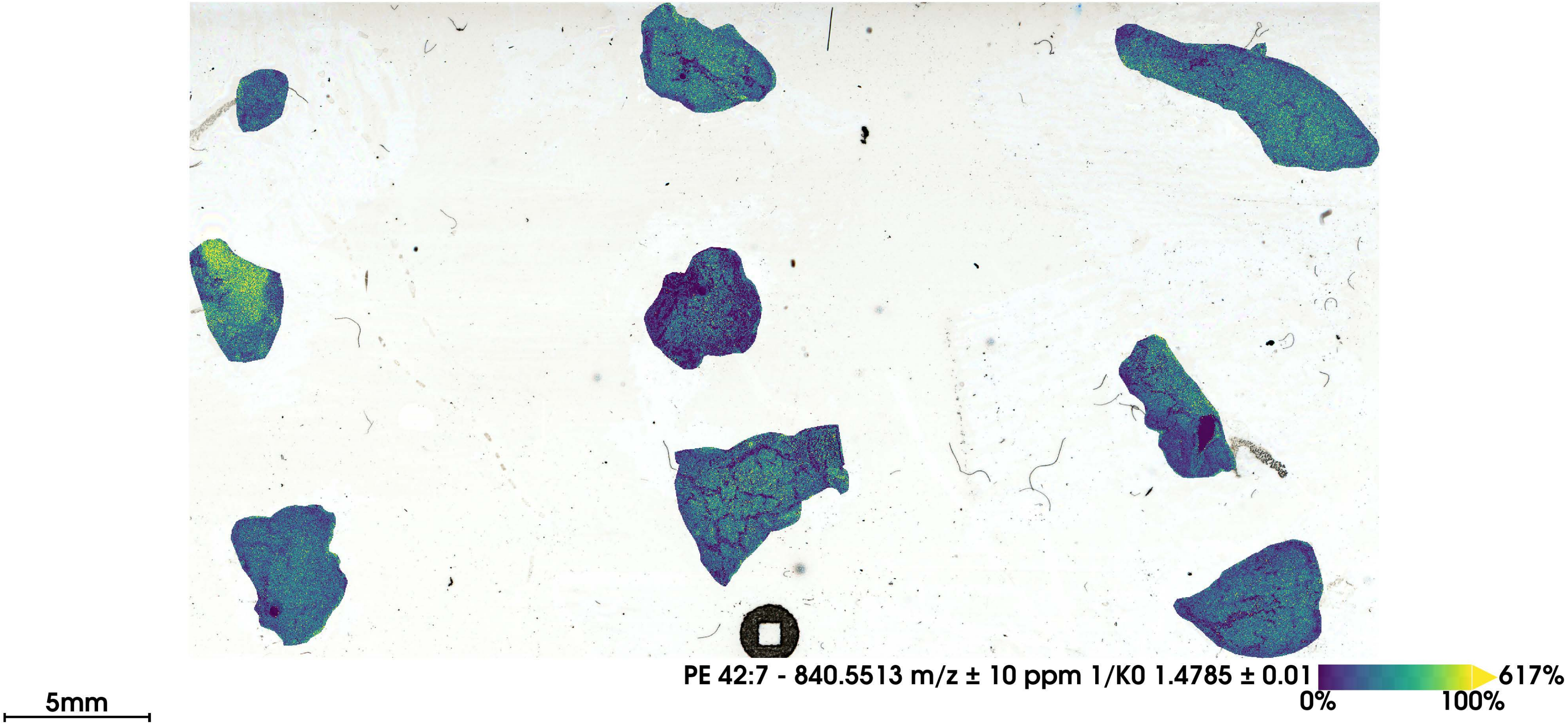
PE 42:7 - 840.5513 m/z ± 10 ppm 1/K0 1.4785 ± 0.01
617%
0%
100%
5mm

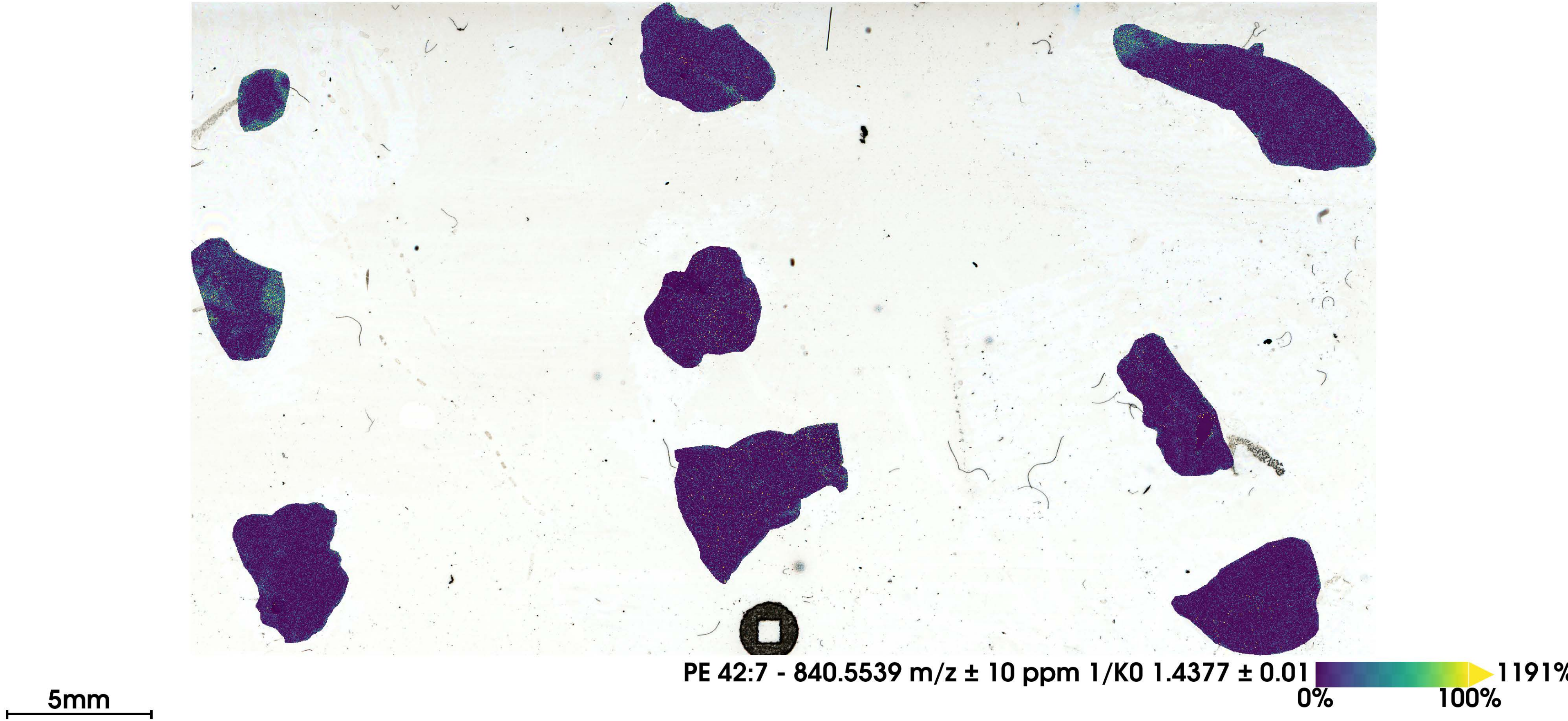

PE 42:7 - 840.5539 m/z ± 10 ppm 1/K0 1.4377 ± 0.01
1191%
0%
100%
5mm

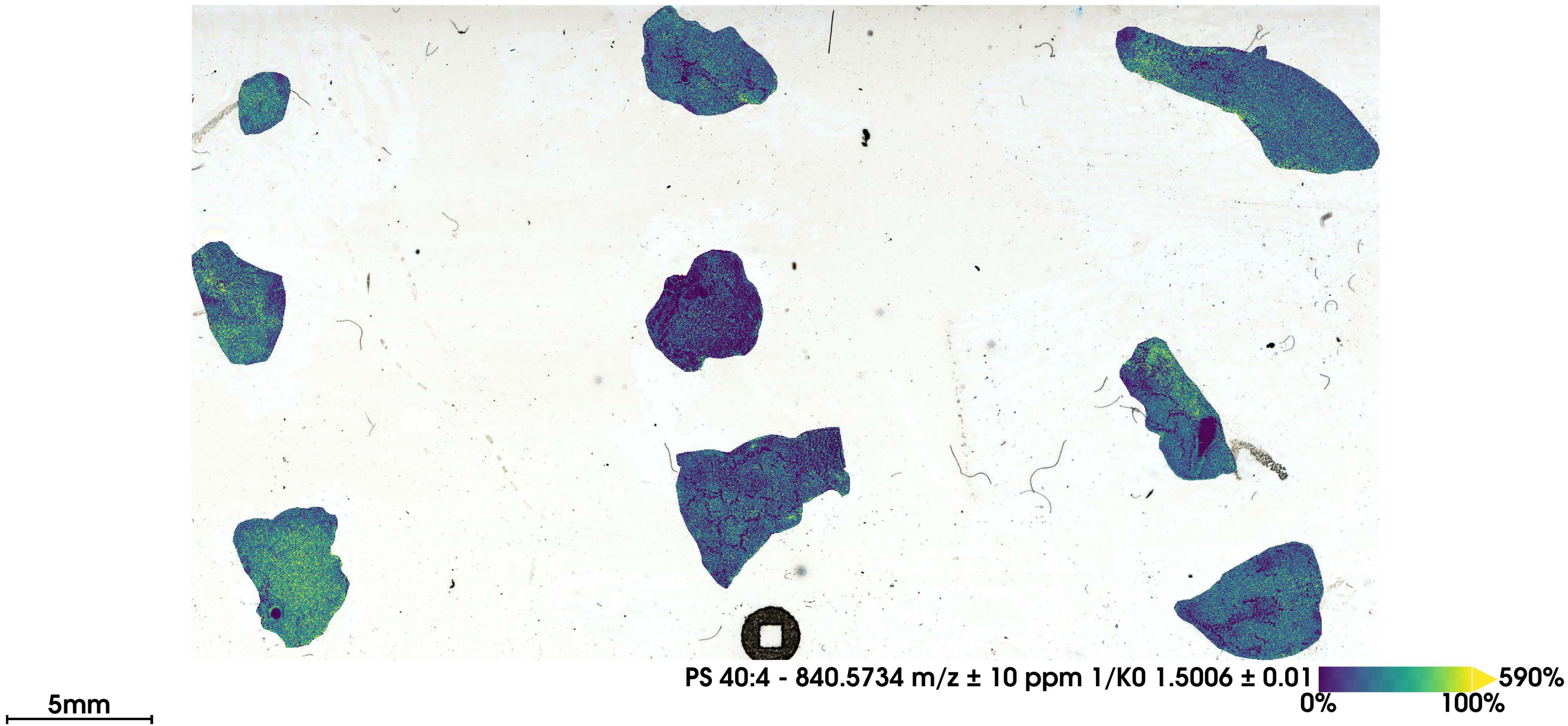
PS 40:4 - 840.5734 m/z ± 10 ppm 1/K0 1.5006 ± 0.01
590%
0%
100%
5mm

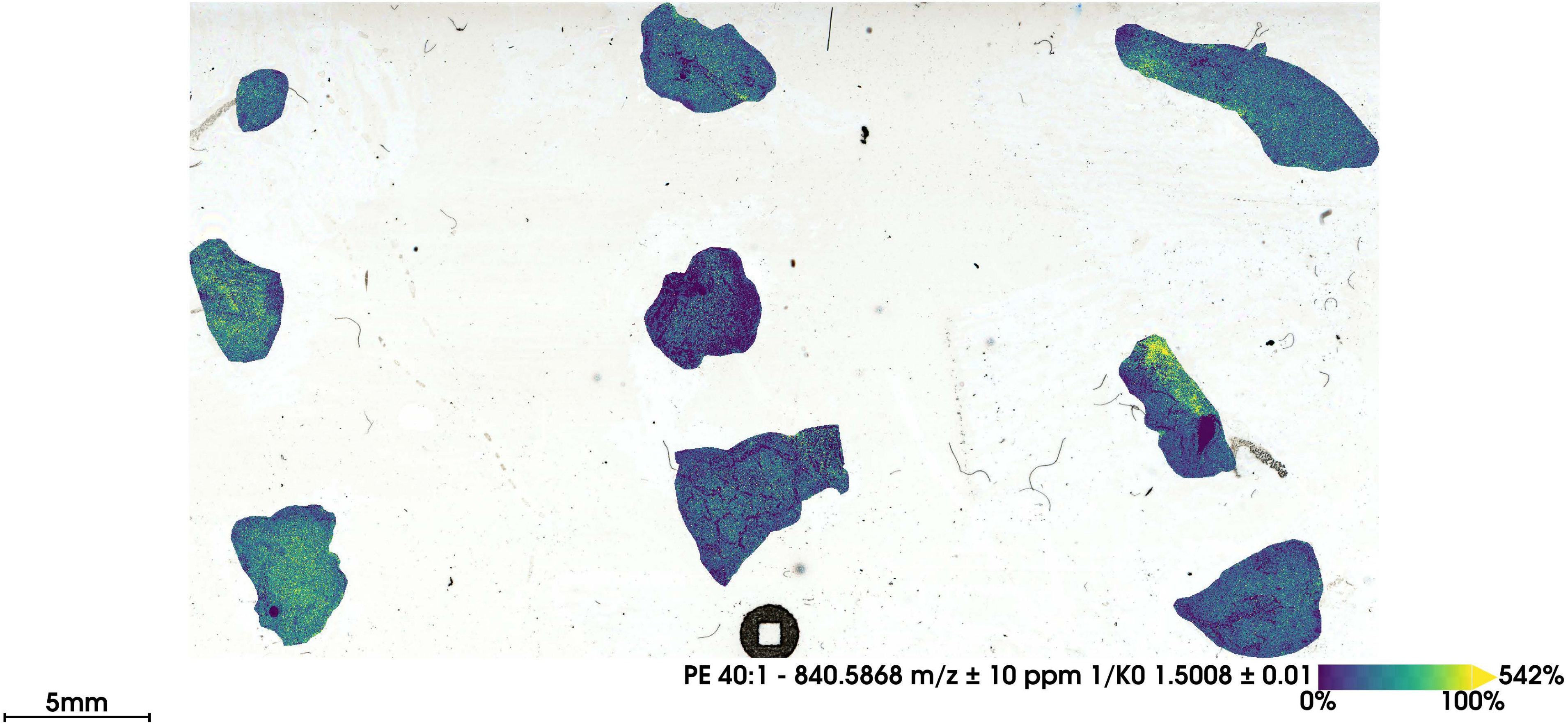
PE 40:1 - 840.5868 m/z ± 10 ppm 1/K0 1.5008 ± 0.01
542%
0%
100%
5mm

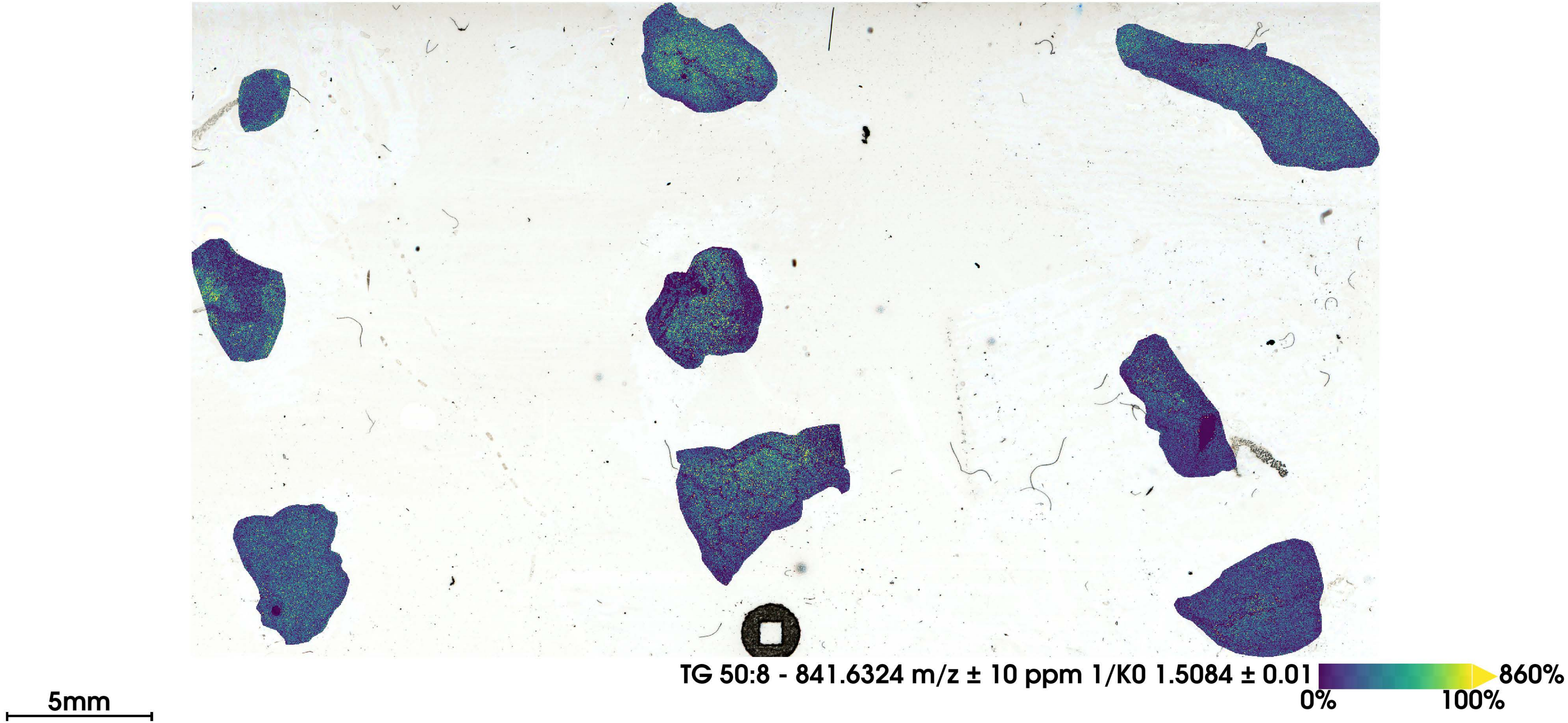
TG 50:8 - 841.6324 m/z ± 10 ppm 1/K0 1.5084 ± 0.01
860%
0%
100%
5mm

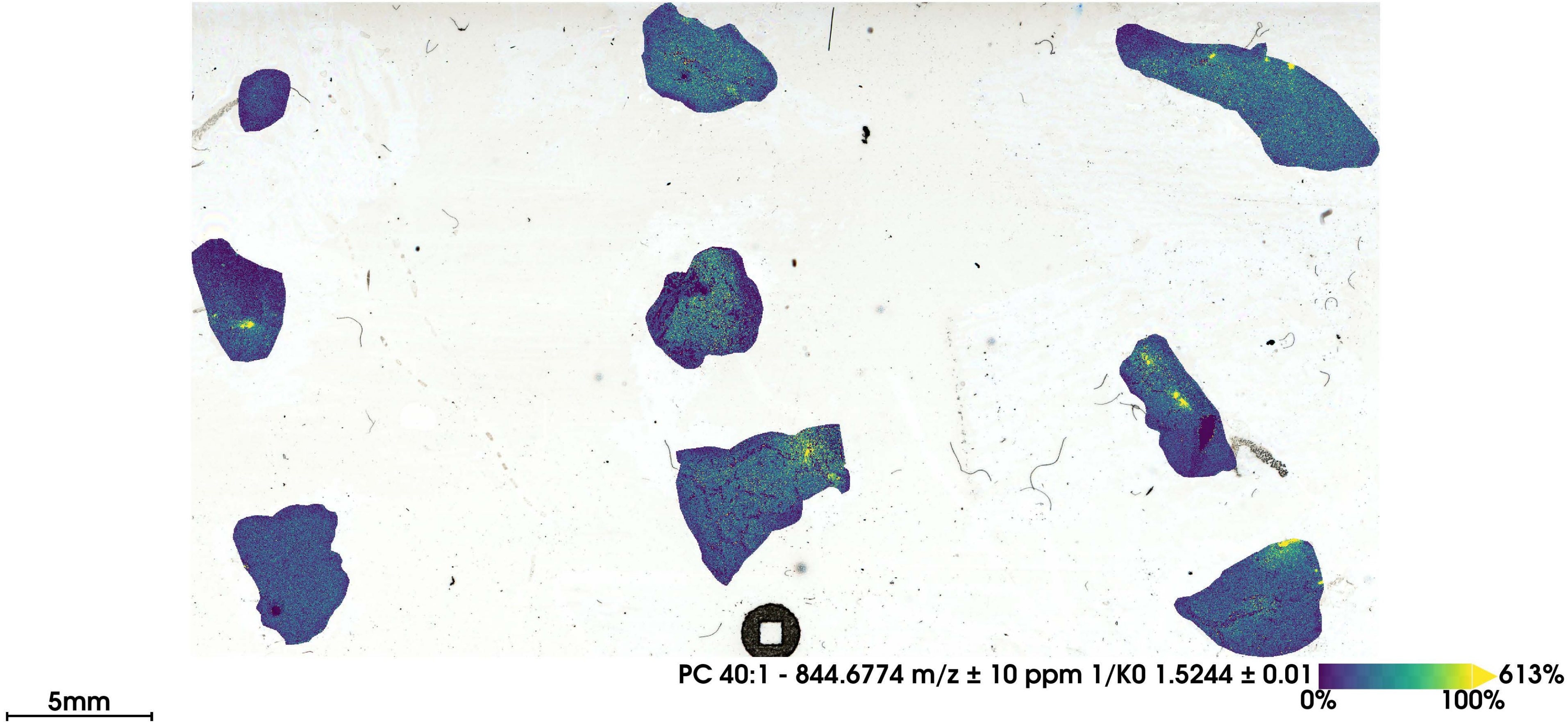
PC 40:1 - 844.6774 m/z ± 10 ppm 1/K0 1.5244 ± 0.01
613%
0%
100%
5mm

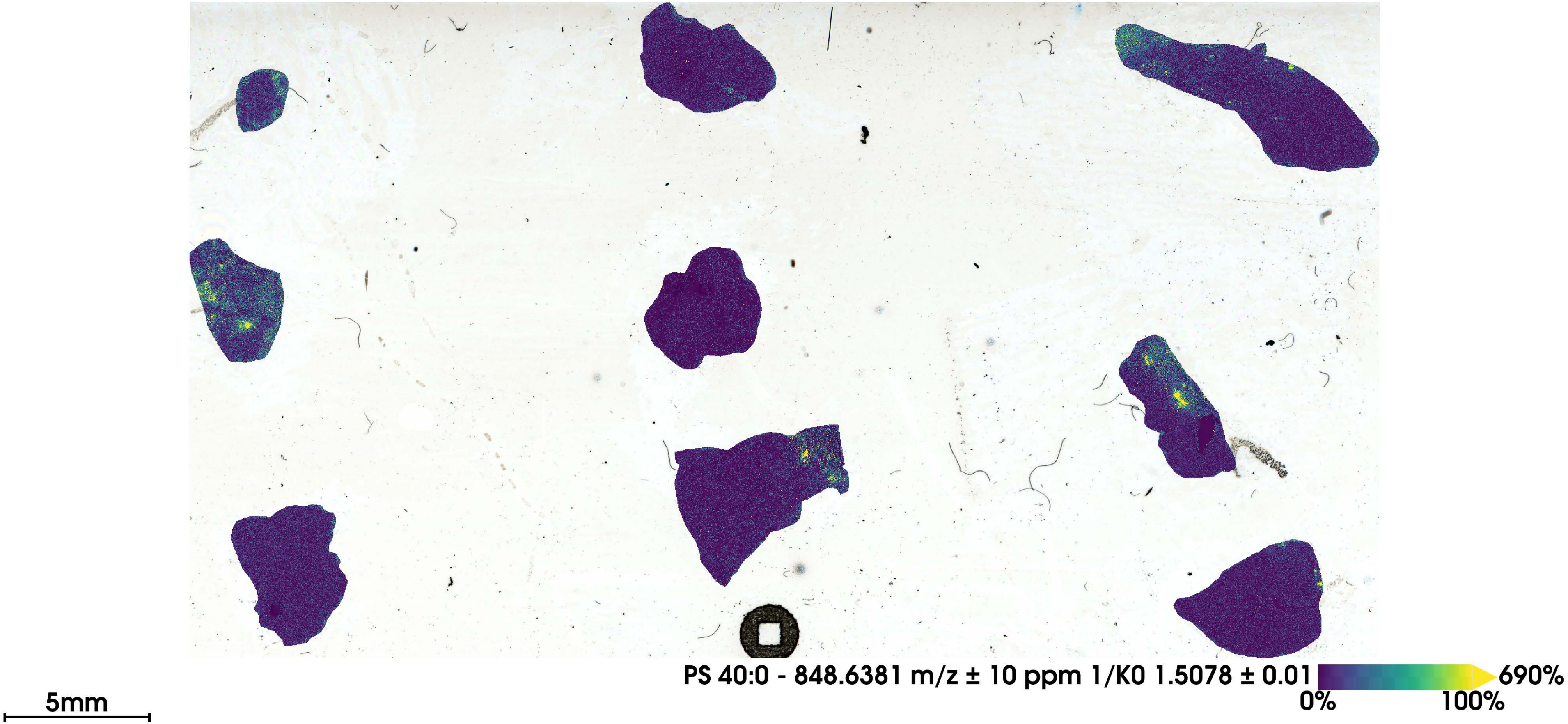
PS 40:0 - 848.6381 m/z ± 10 ppm 1/K0 1.5078 ± 0.01
690%
0%
100%
5mm

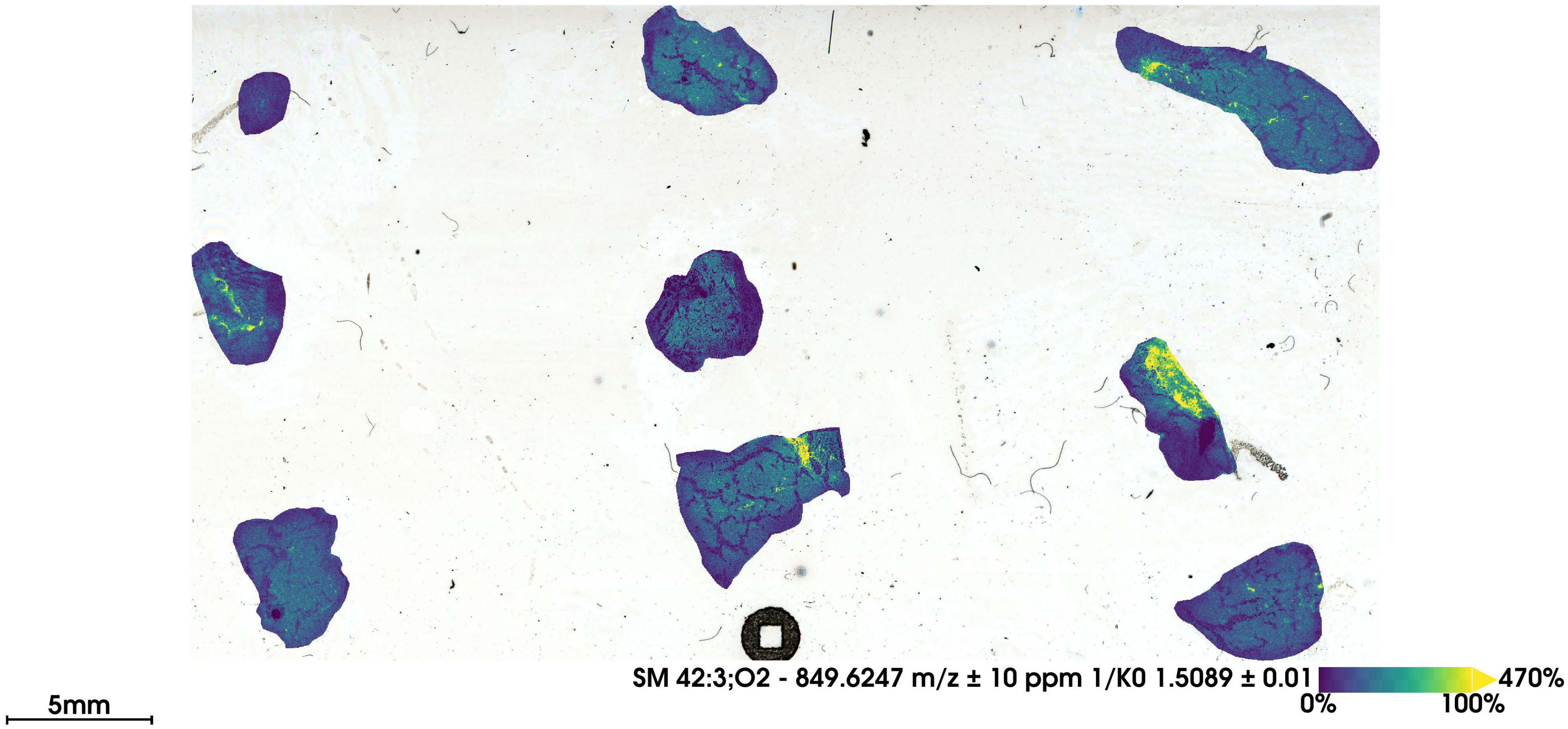

SM 42:3;O2 - 849.6247 m/z ± 10 ppm 1/K0 1.5089 ± 0.01
470%
0%
100%
5mm

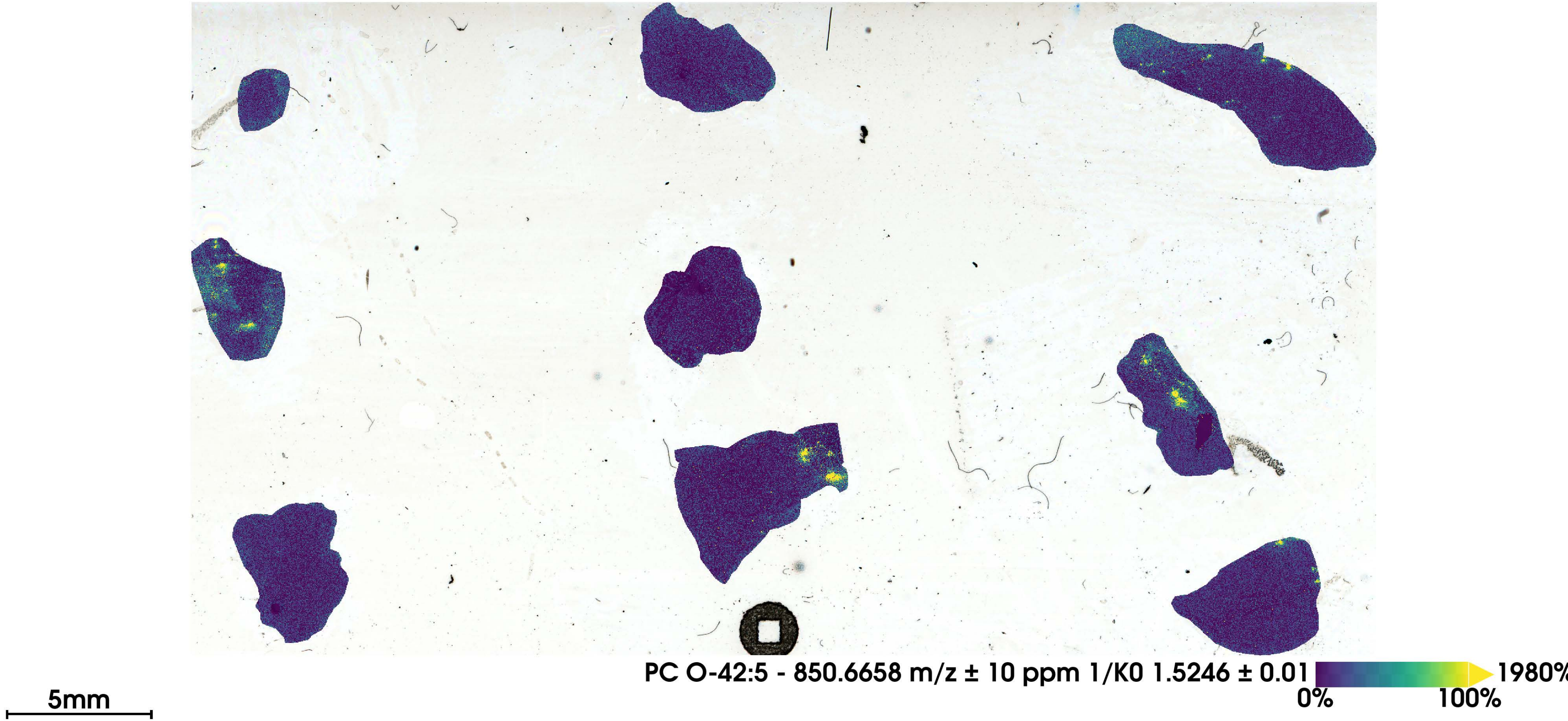

PC O-42:5 - 850.6658 m/z ± 10 ppm 1/K0 1.5246 ± 0.01
1980%
0%
100%
5mm

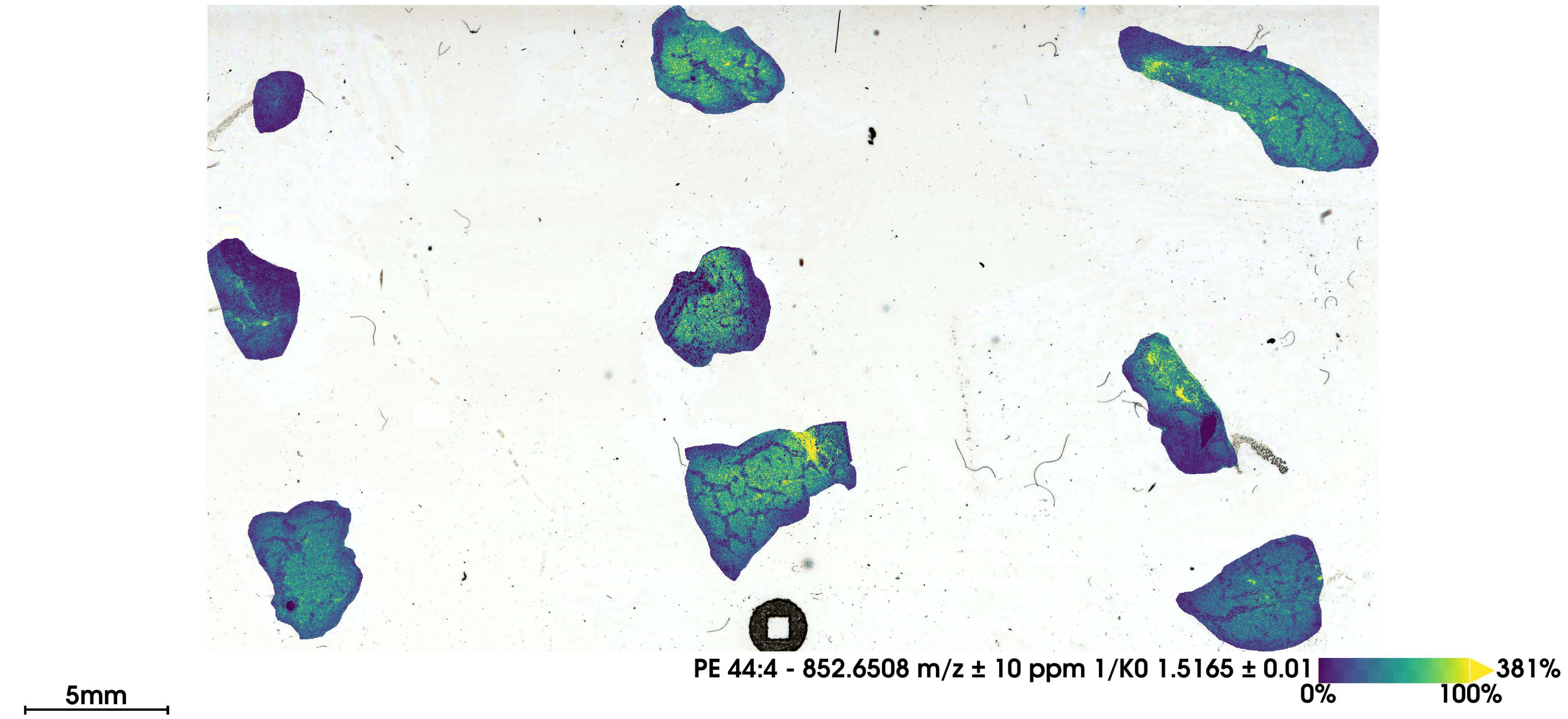

PE 44:4 - 852.6508 m/z ± 10 ppm 1/K0 1.5165 ± 0.01
381%
0%
100%
5mm

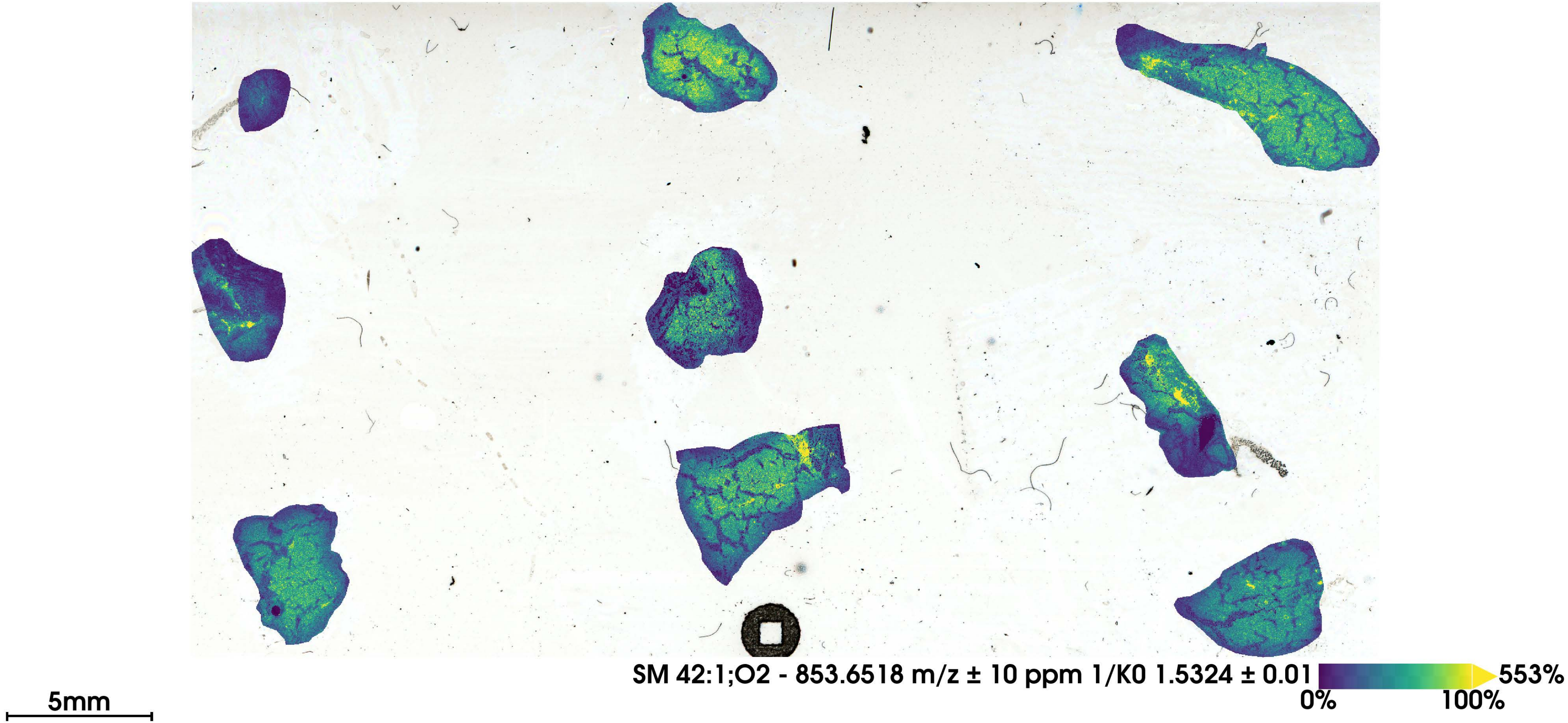

SM 42:1;O2 - 853.6518 m/z ± 10 ppm 1/K0 1.5324 ± 0.01
553%
0%
100%
5mm

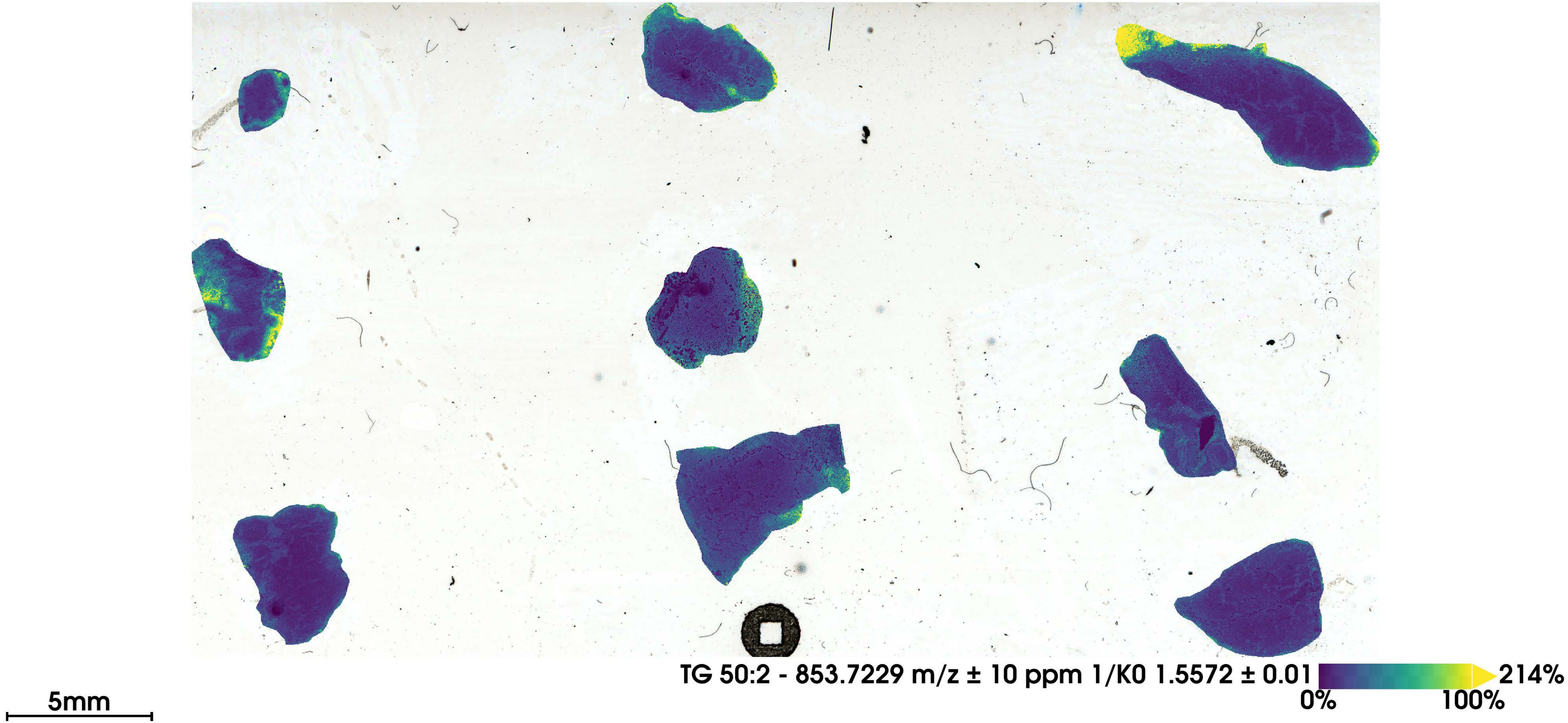
TG 50:2 - 853.7229 m/z ± 10 ppm 1/K0 1.5572 ± 0.01
214%
0%
100%
5mm

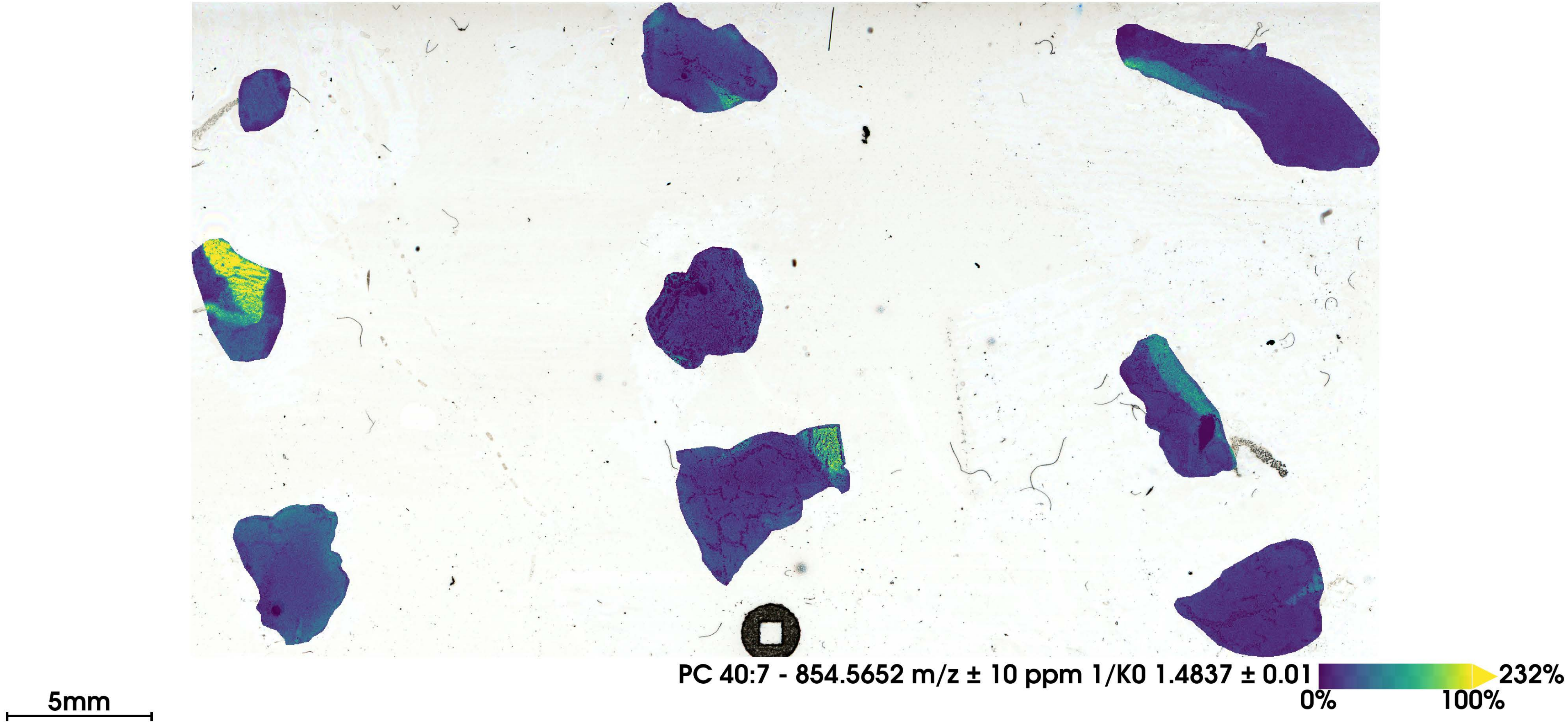

PC 40:7 - 854.5652 m/z ± 10 ppm 1/K0 1.4837 ± 0.01
232%
0%
100%
5mm

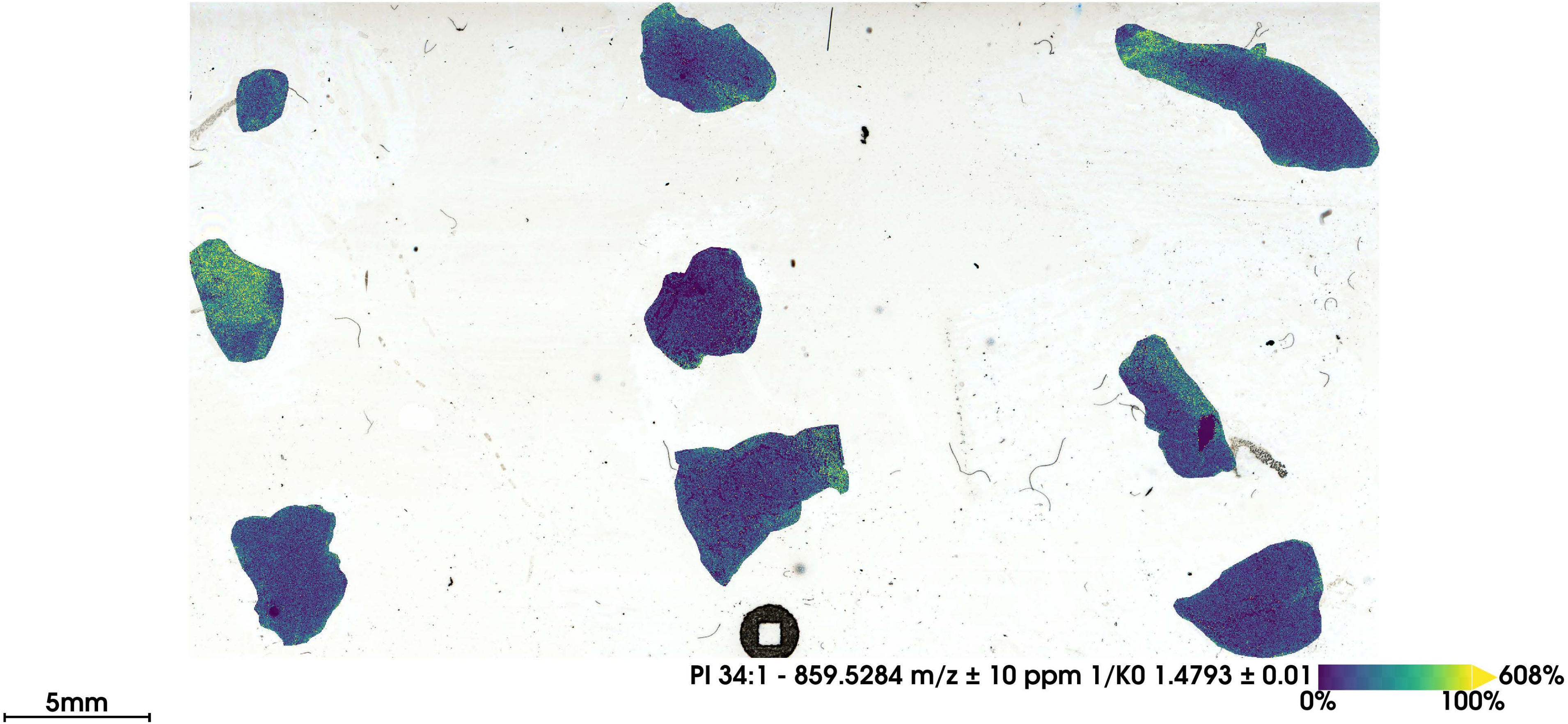
PI 34:1 - 859.5284 m/z ± 10 ppm 1/K0 1.4793 ± 0.01
608%
0%
100%
5mm

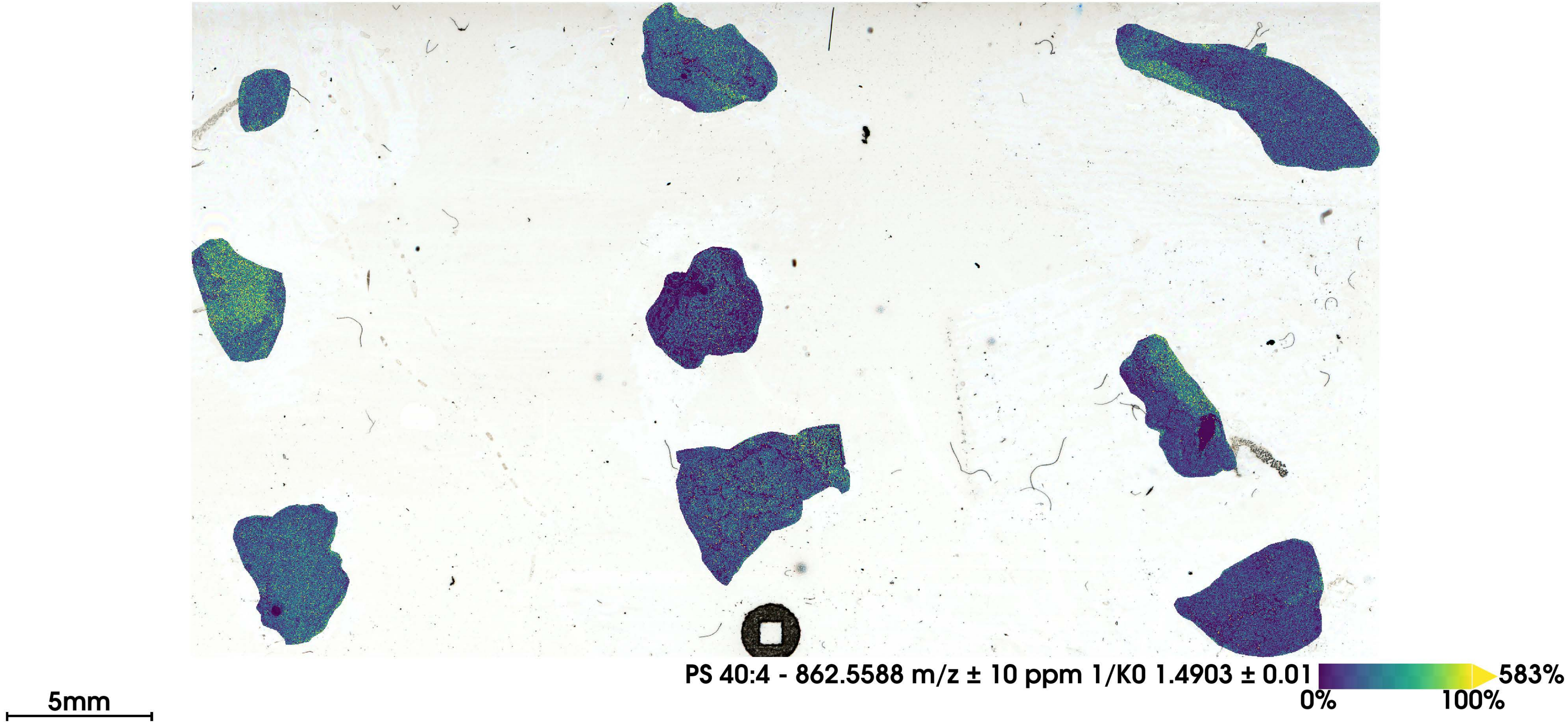
PS 40:4 - 862.5588 m/z ± 10 ppm 1/K0 1.4903 ± 0.01
583%
0%
100%
5mm

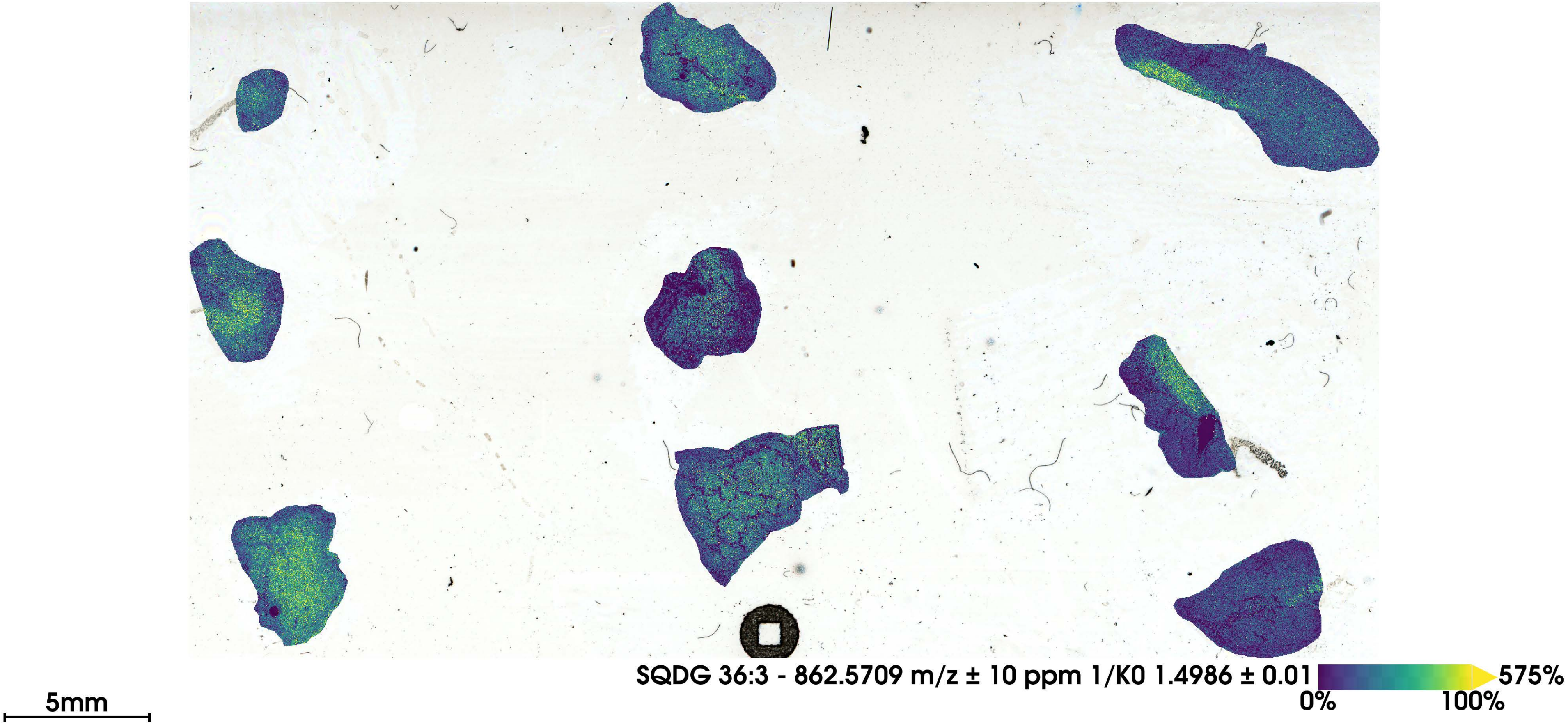

SQDG 36:3 - 862.5709 m/z ± 10 ppm 1/K0 1.4986 ± 0.01
575%
0%
100%
5mm

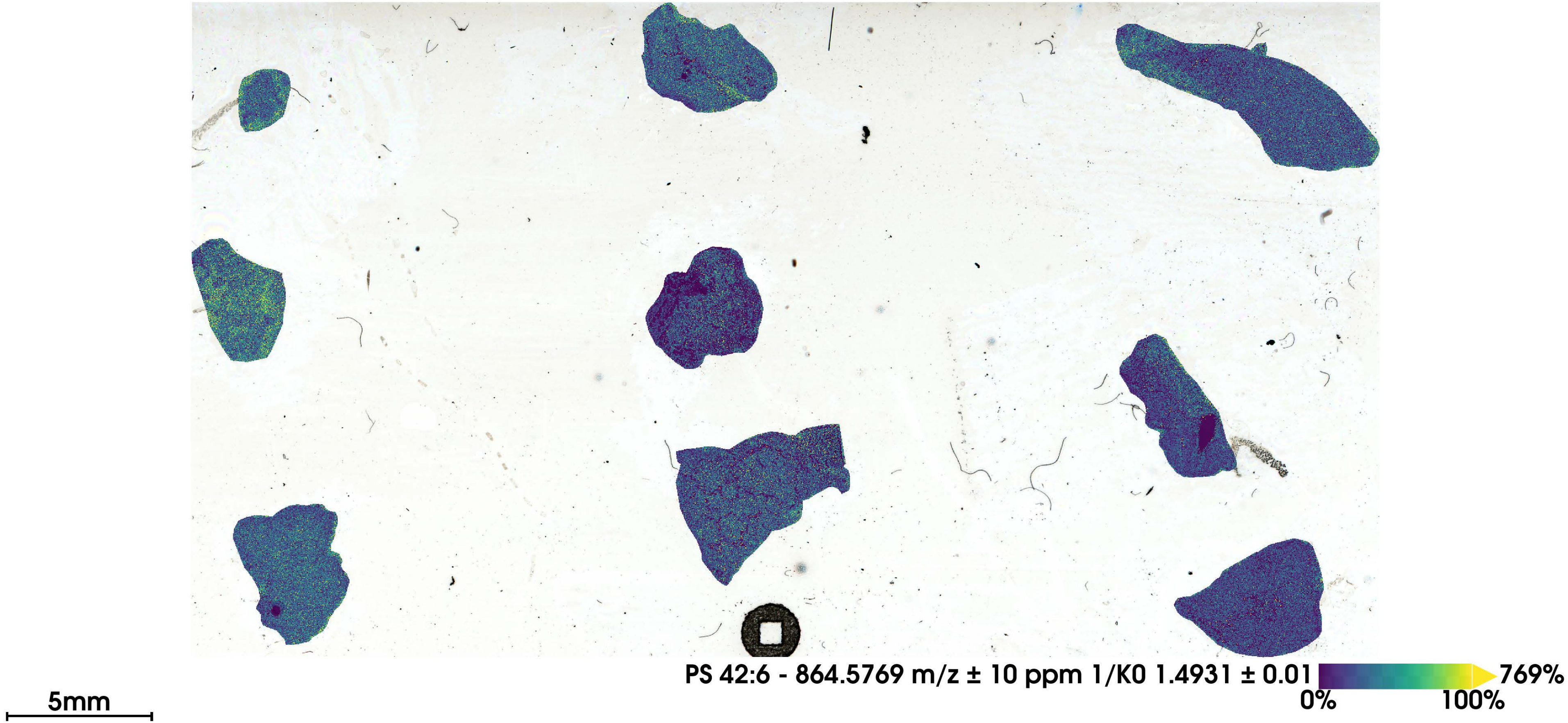

PS 42:6 - 864.5769 m/z ± 10 ppm 1/K0 1.4931 ± 0.01
769%
0%
100%
5mm

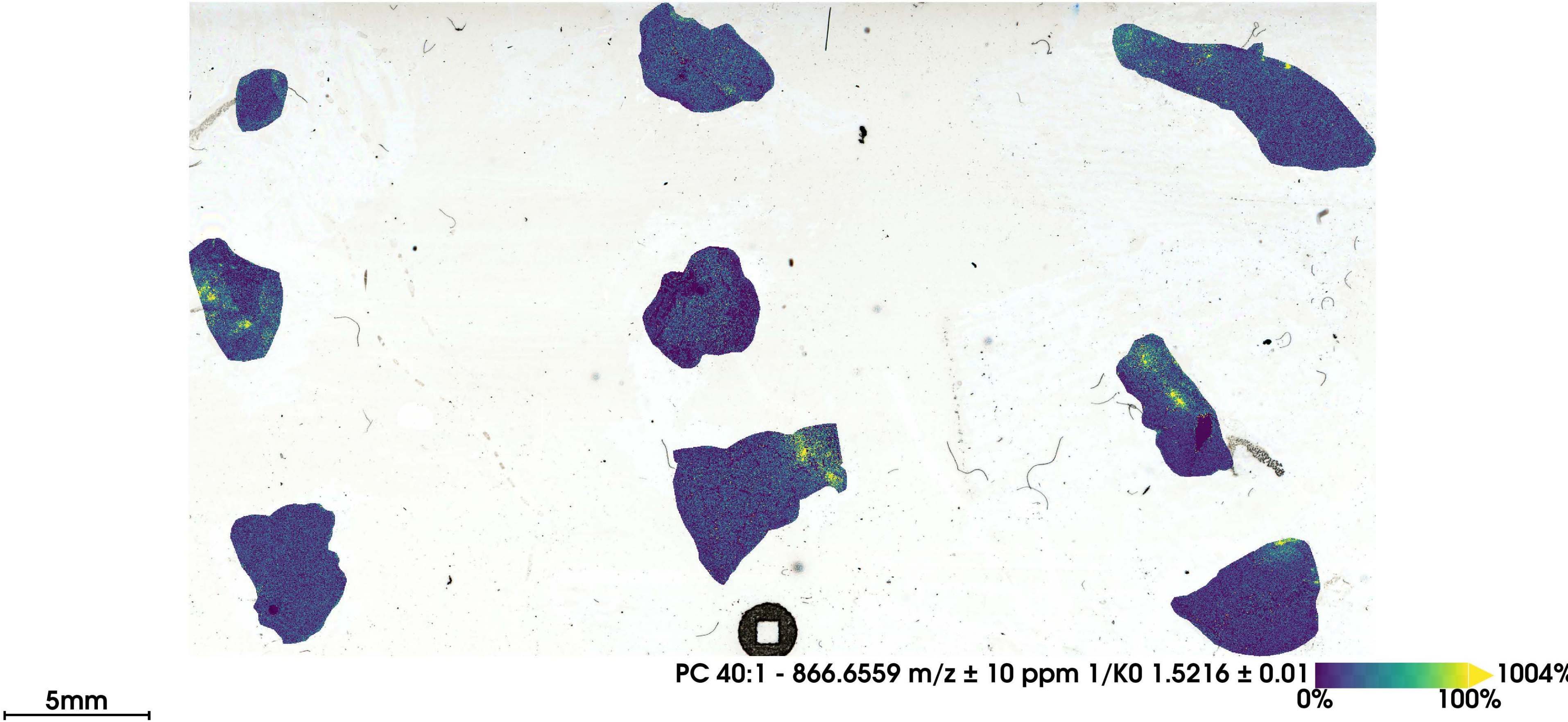
PC 40:1 - 866.6559 m/z ± 10 ppm 1/K0 1.5216 ± 0.01
1004%
0%
100%
5mm

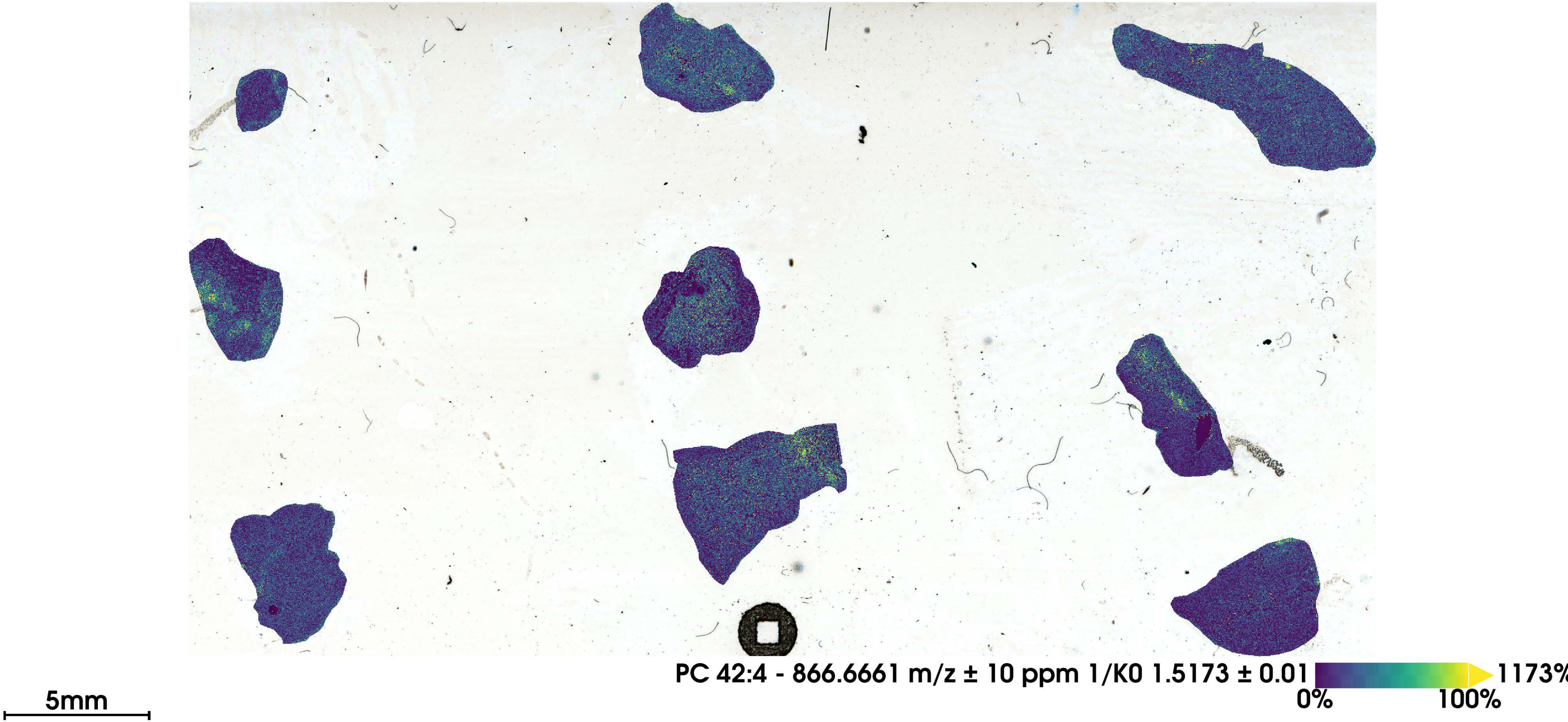
PC 42:4 - 866.6661 m/z ± 10 ppm 1/K0 1.5173 ± 0.01
1173%
0%
100%
5mm

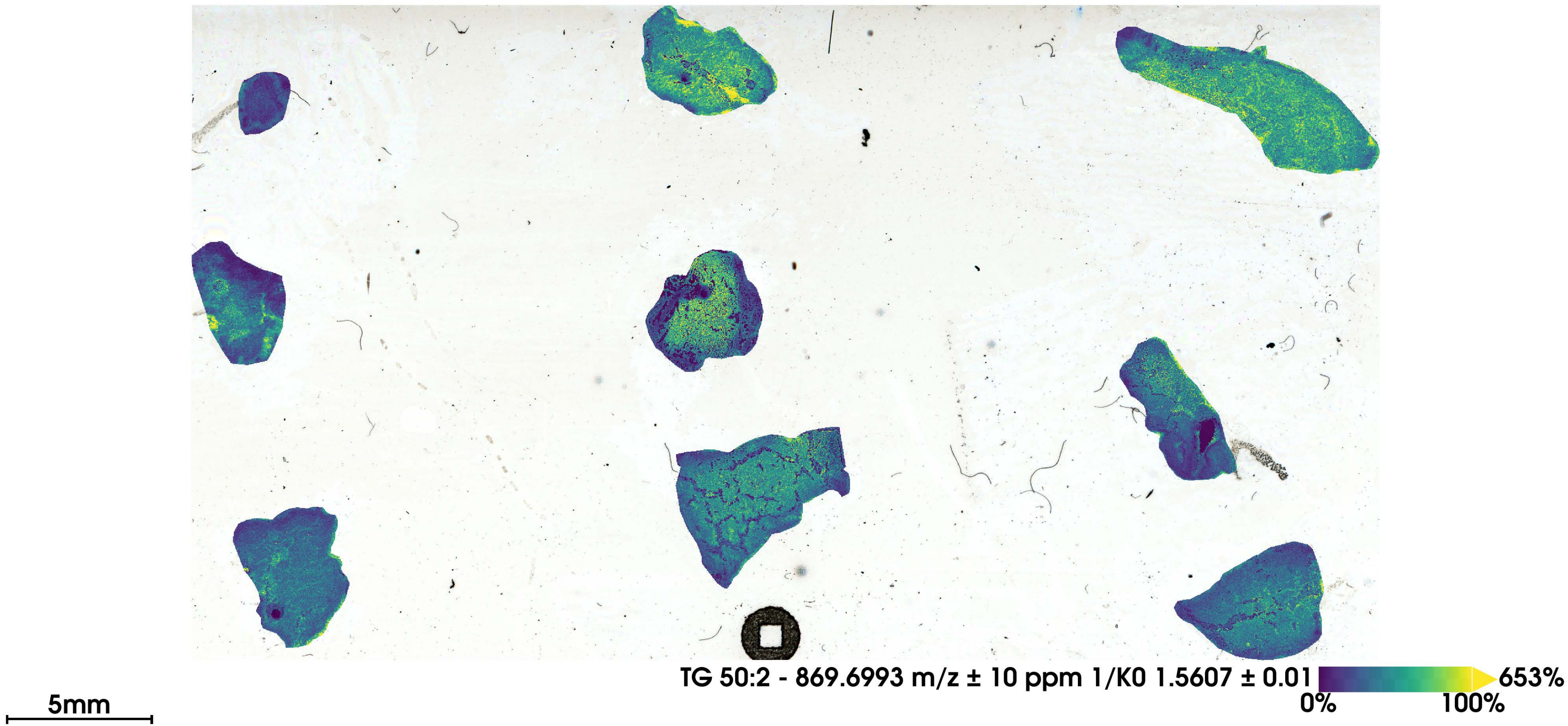
TG 50:2 - 869.6993 m/z ± 10 ppm 1/K0 1.5607 ± 0.01
653%
0%
100%
5mm

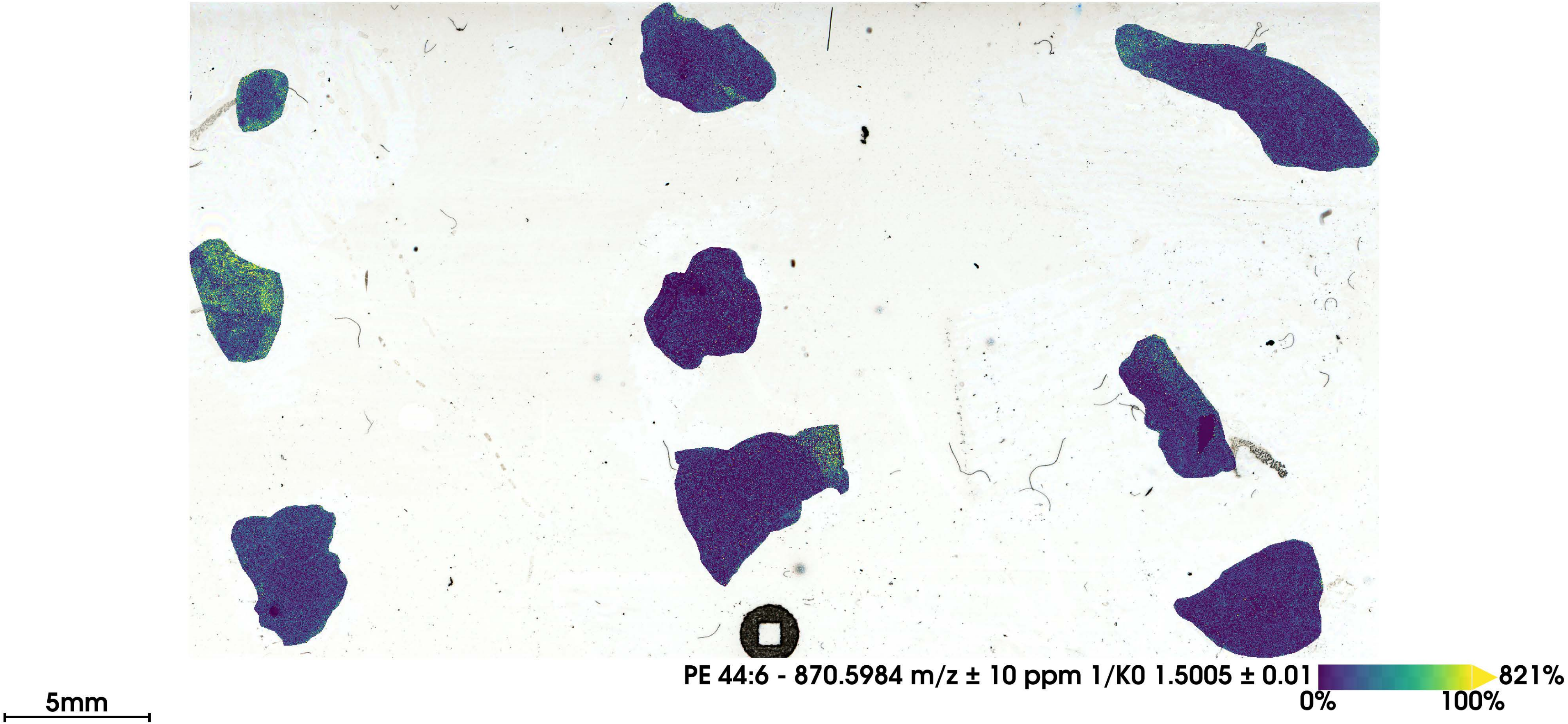
PE 44:6 - 870.5984 m/z ± 10 ppm 1/K0 1.5005 ± 0.01
821%
0%
100%
5mm

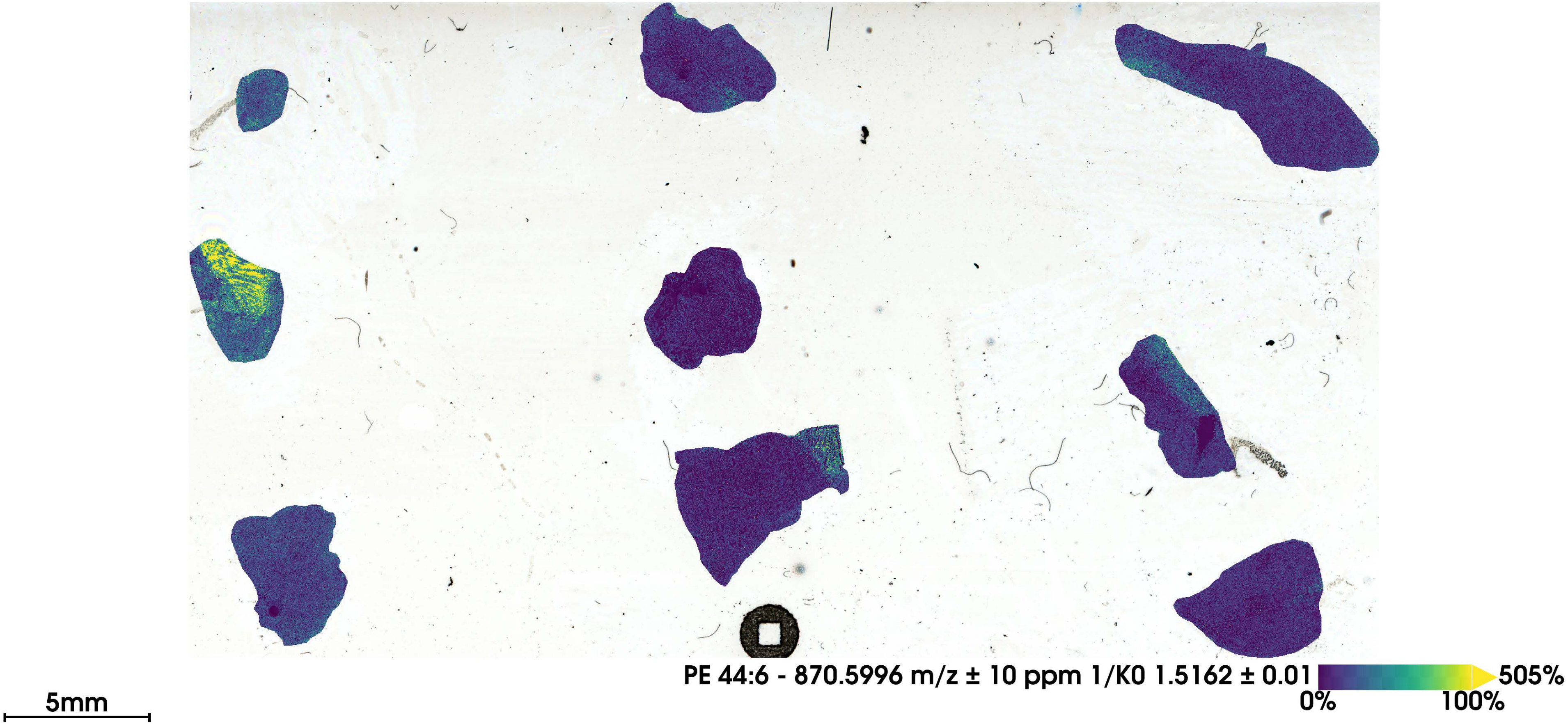
PE 44:6 - 870.5996 m/z ± 10 ppm 1/K0 1.5162 ± 0.01
505%
0%
100%
5mm

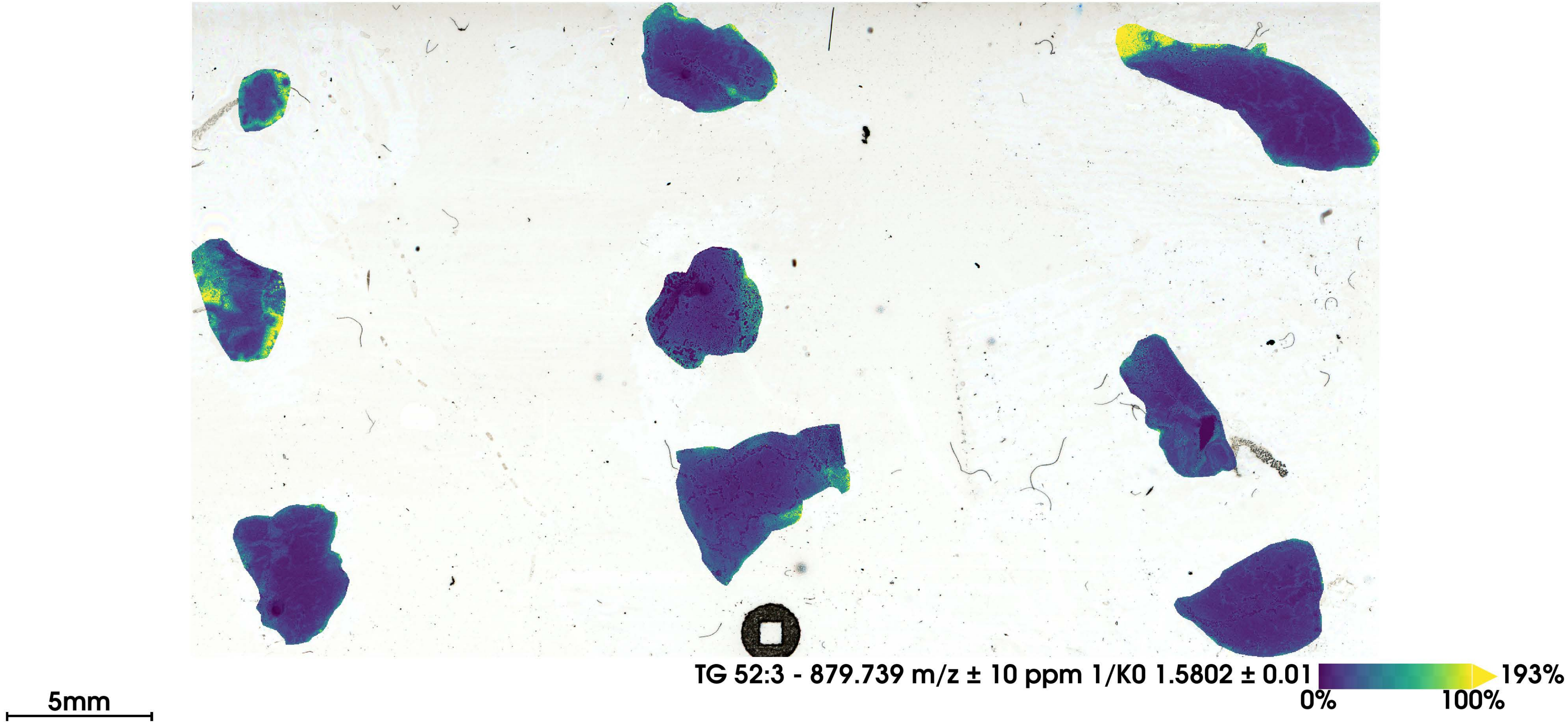

TG 52:3 - 879.739 m/z ± 10 ppm 1/K0 1.5802 ± 0.01
193%
0%
100%
5mm

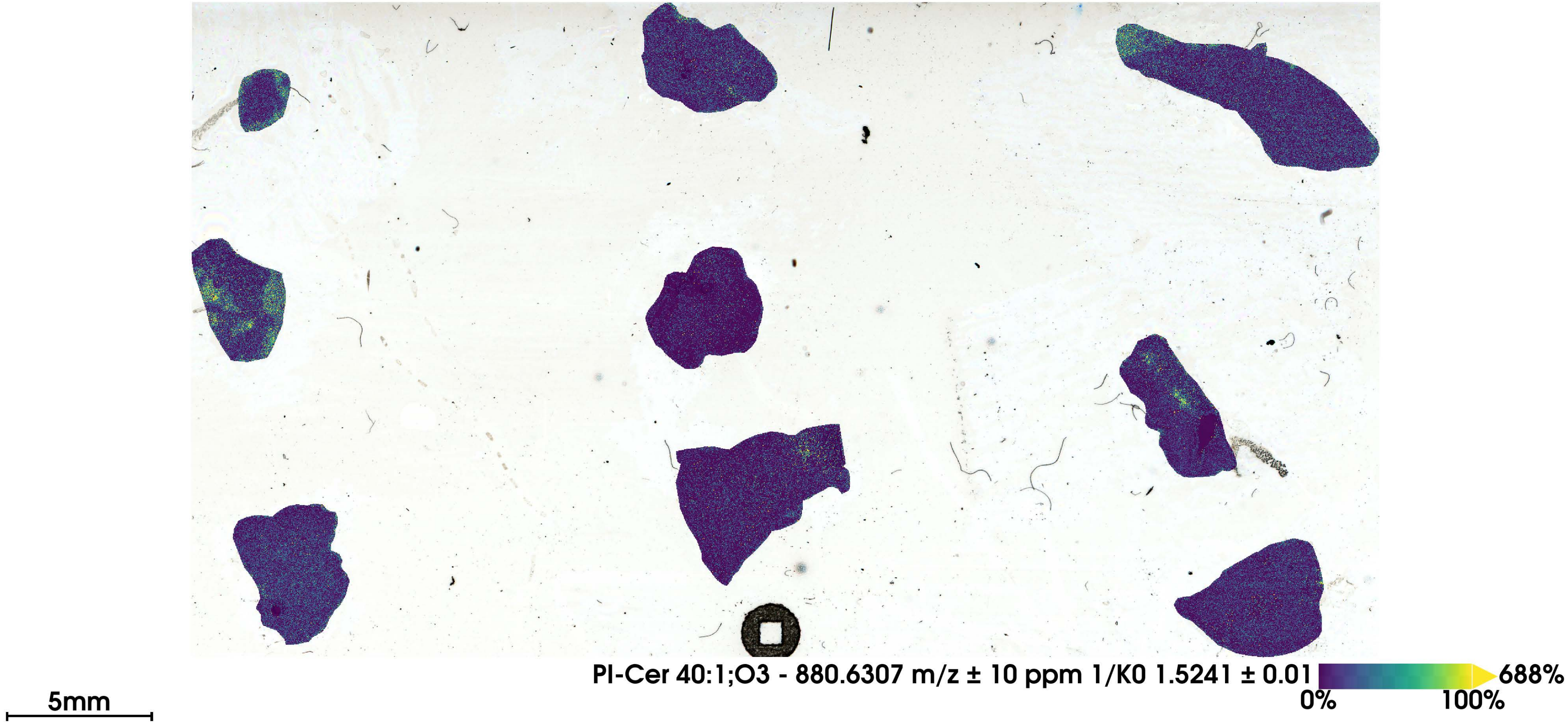

PI-Cer 40:1;O3 - 880.6307 m/z ± 10 ppm 1/K0 1.5241 ± 0.01
688%
0%
100%
5mm

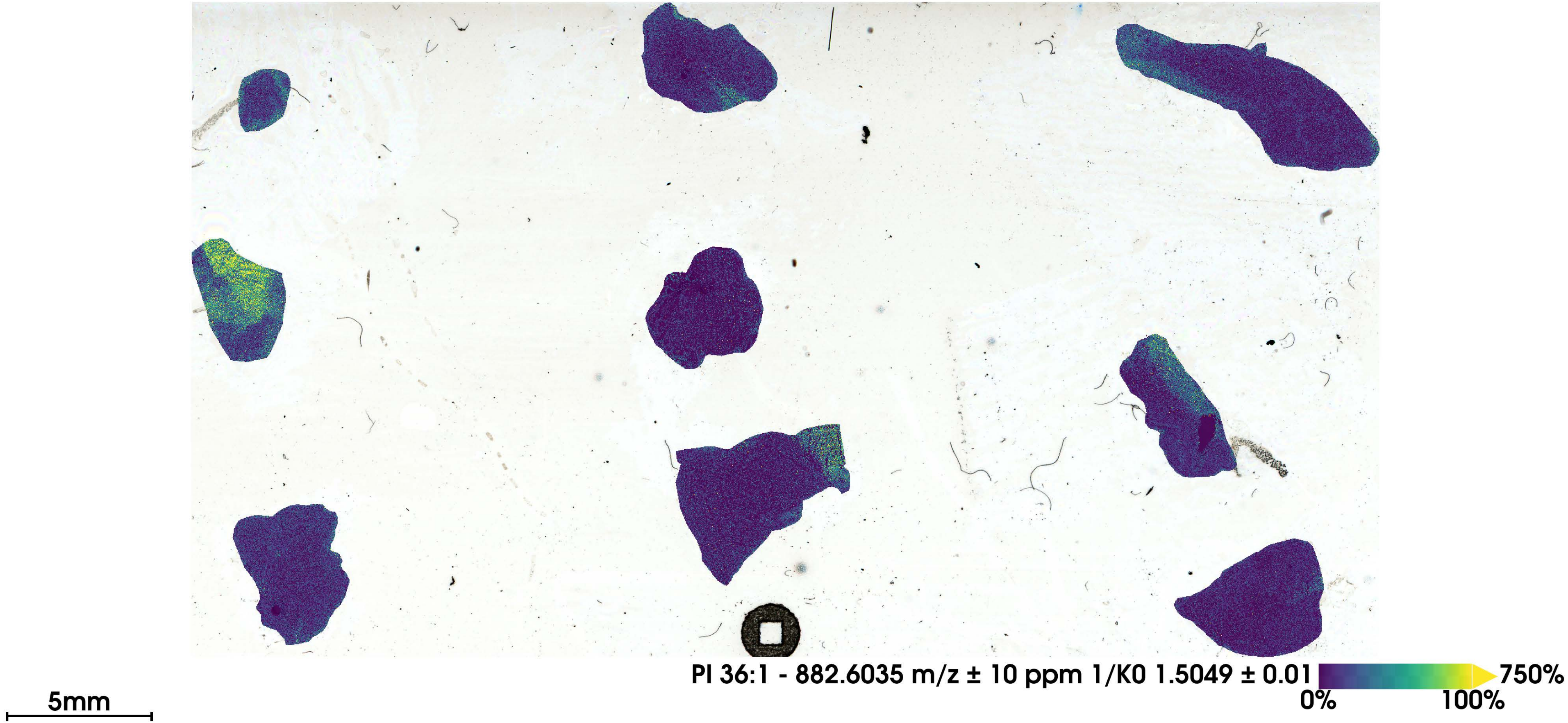
PI 36:1 - 882.6035 m/z ± 10 ppm 1/K0 1.5049 ± 0.01
750%
0%
100%
5mm

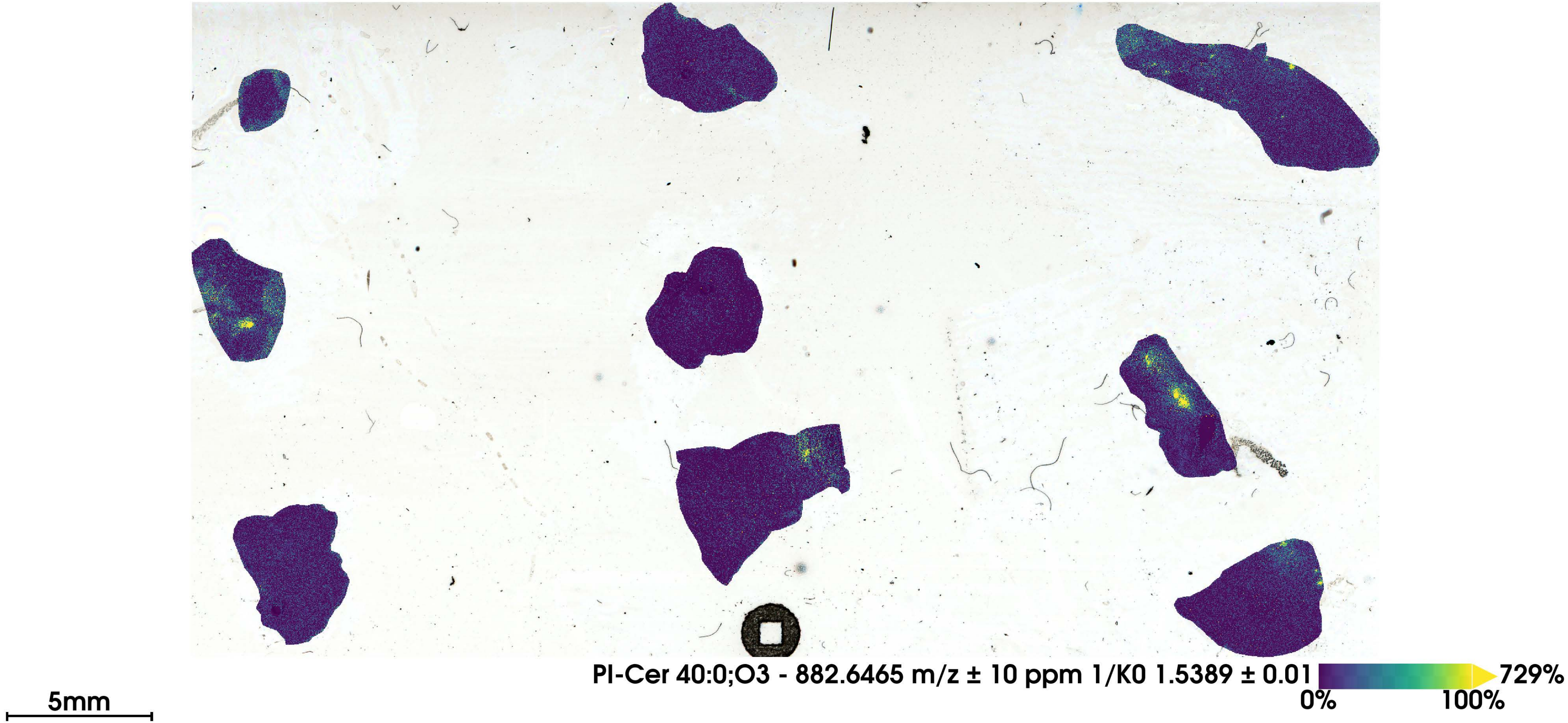

PI-Cer 40:0;O3 - 882.6465 m/z ± 10 ppm 1/K0 1.5389 ± 0.01
729%
0%
100%
5mm

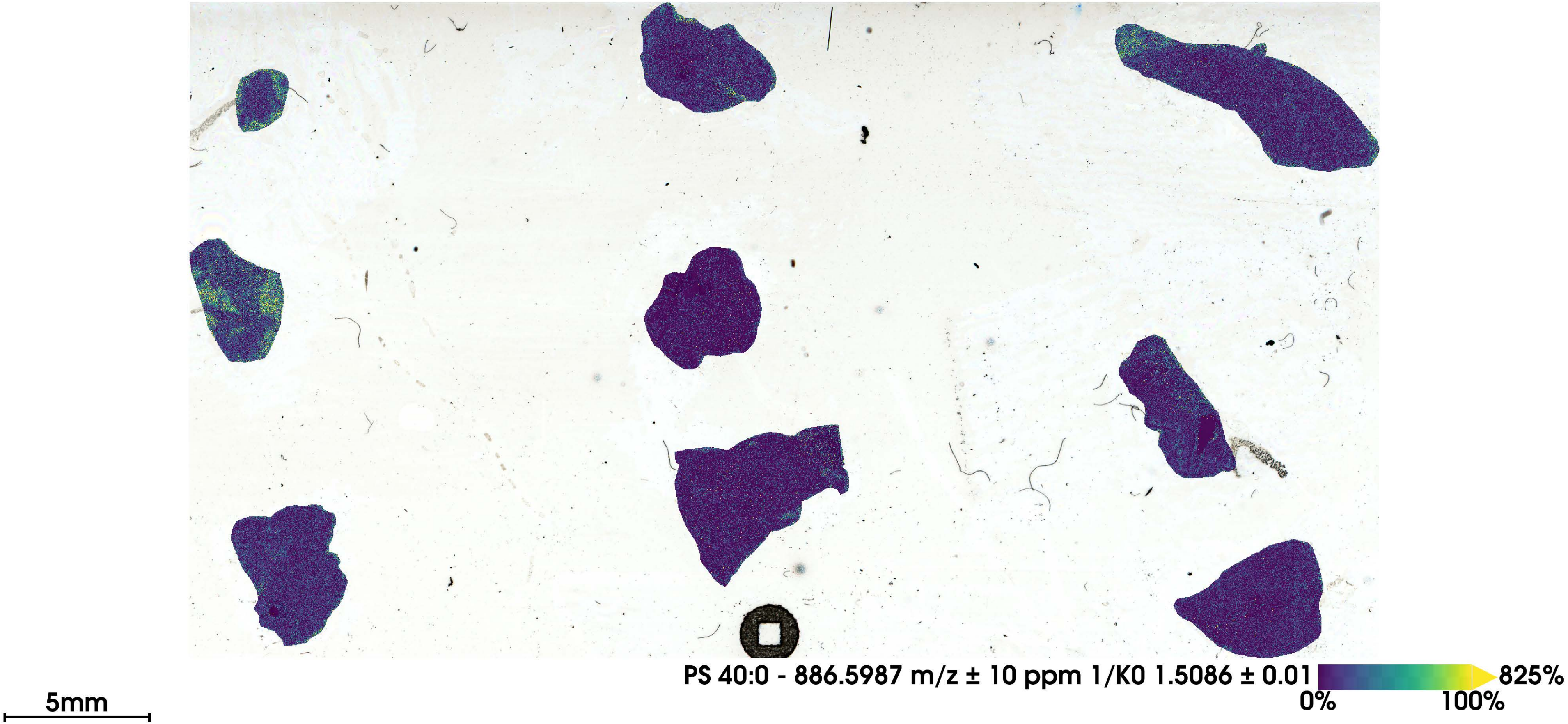
PS 40:0 - 886.5987 m/z ± 10 ppm 1/K0 1.5086 ± 0.01
825%
0%
100%
5mm

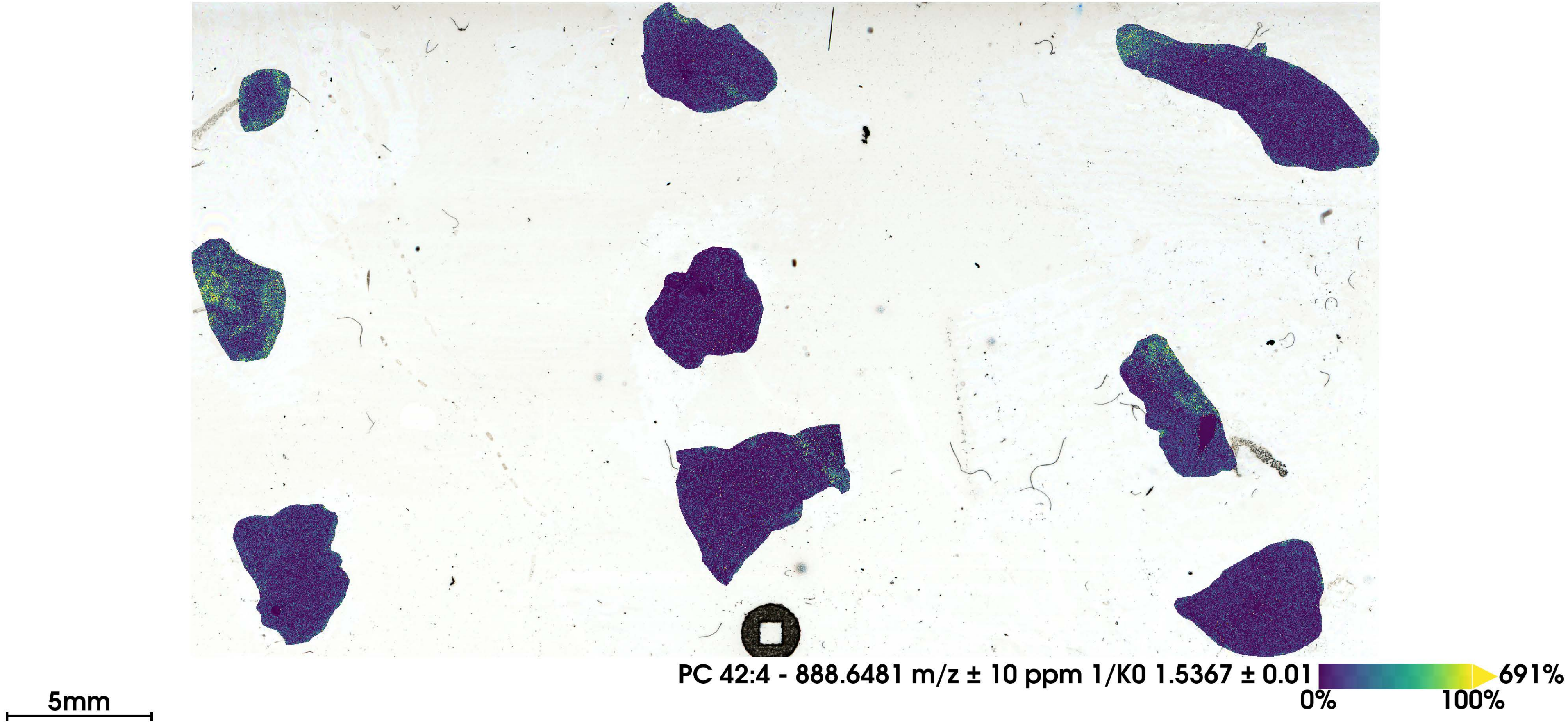

PC 42:4 - 888.6481 m/z ± 10 ppm 1/K0 1.5367 ± 0.01
691%
0%
100%
5mm

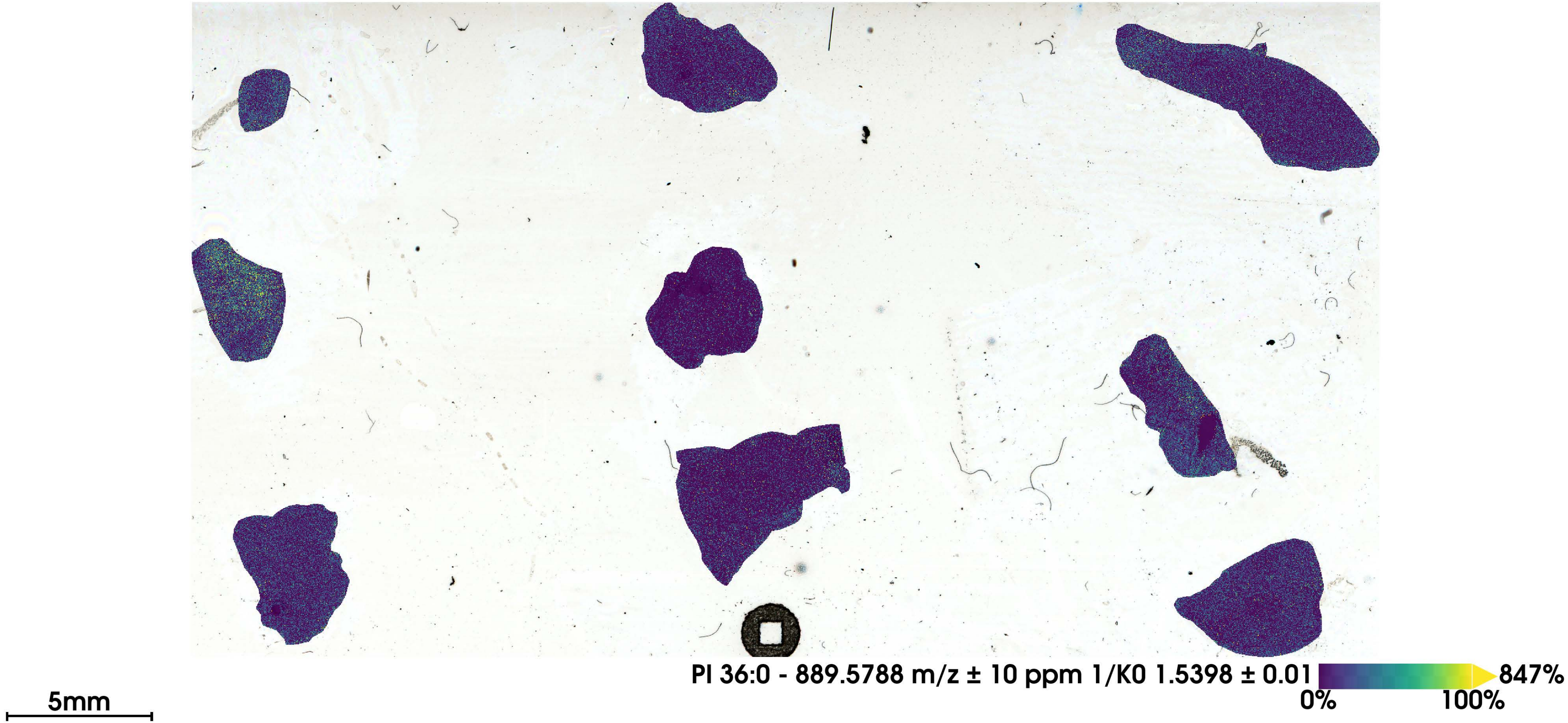
PI 36:0 - 889.5788 m/z ± 10 ppm 1/K0 1.5398 ± 0.01
847%
0%
100%
5mm

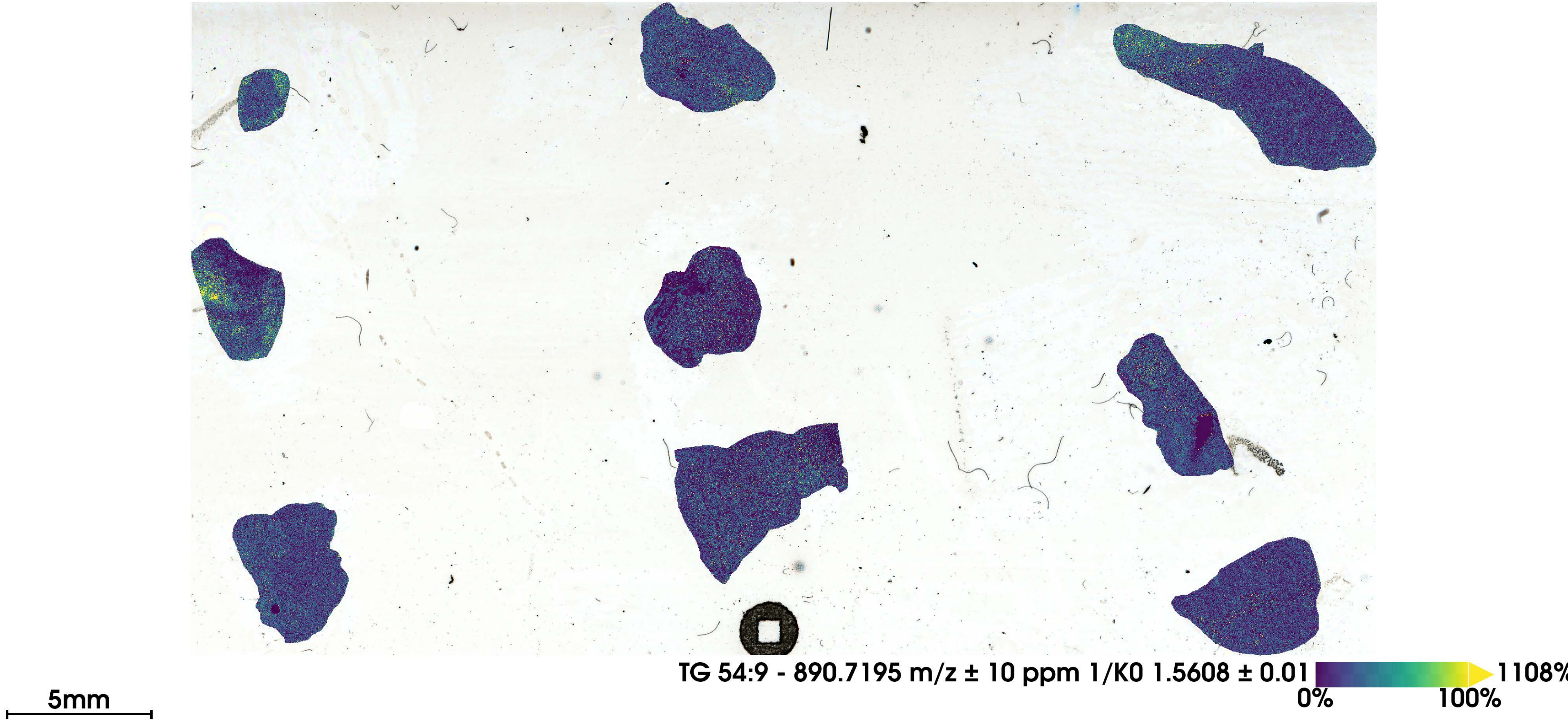
TG 54:9 - 890.7195 m/z ± 10 ppm 1/K0 1.5608 ± 0.01
1108%
0%
100%
5mm

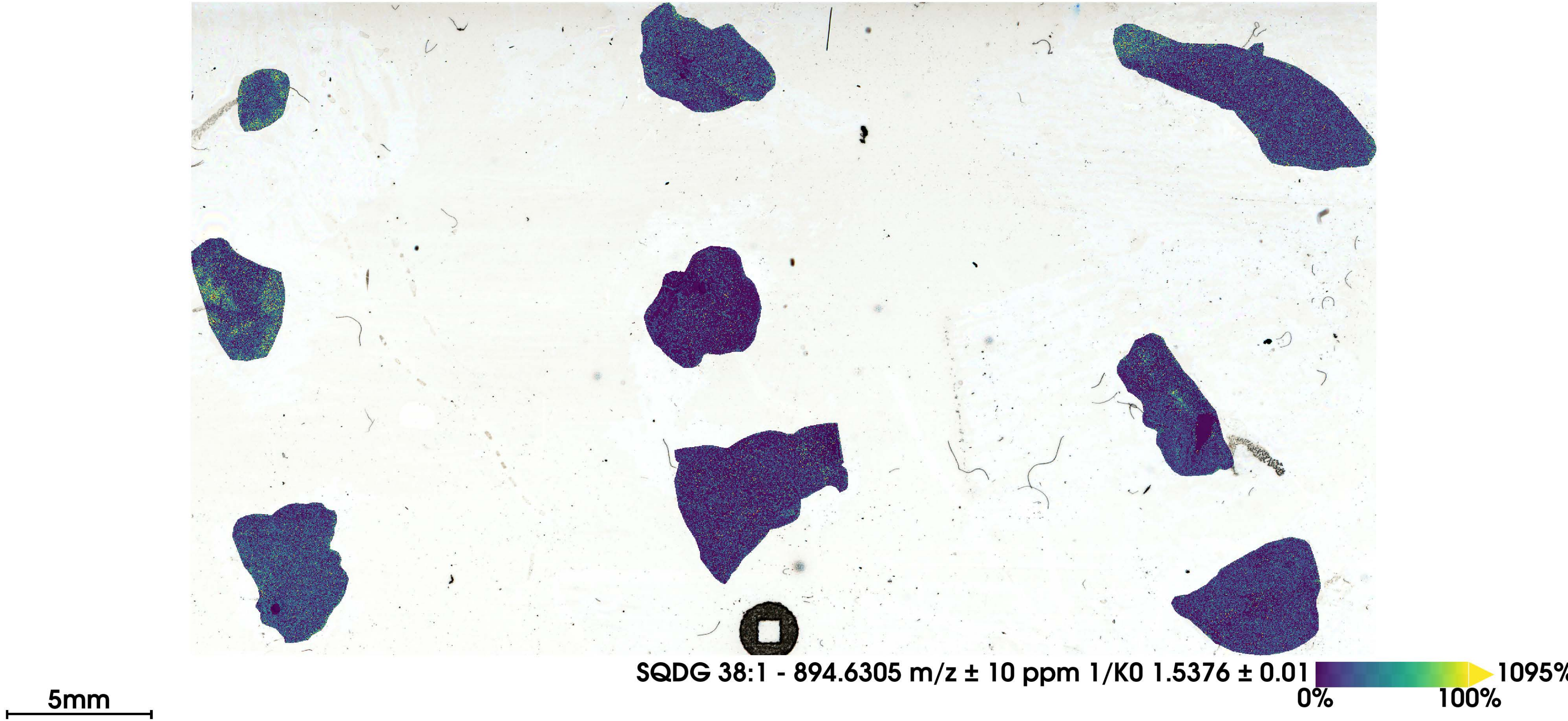
SQDG 38:1 - 894.6305 m/z ± 10 ppm 1/K0 1.5376 ± 0.01
1095%
0%
100%
5mm

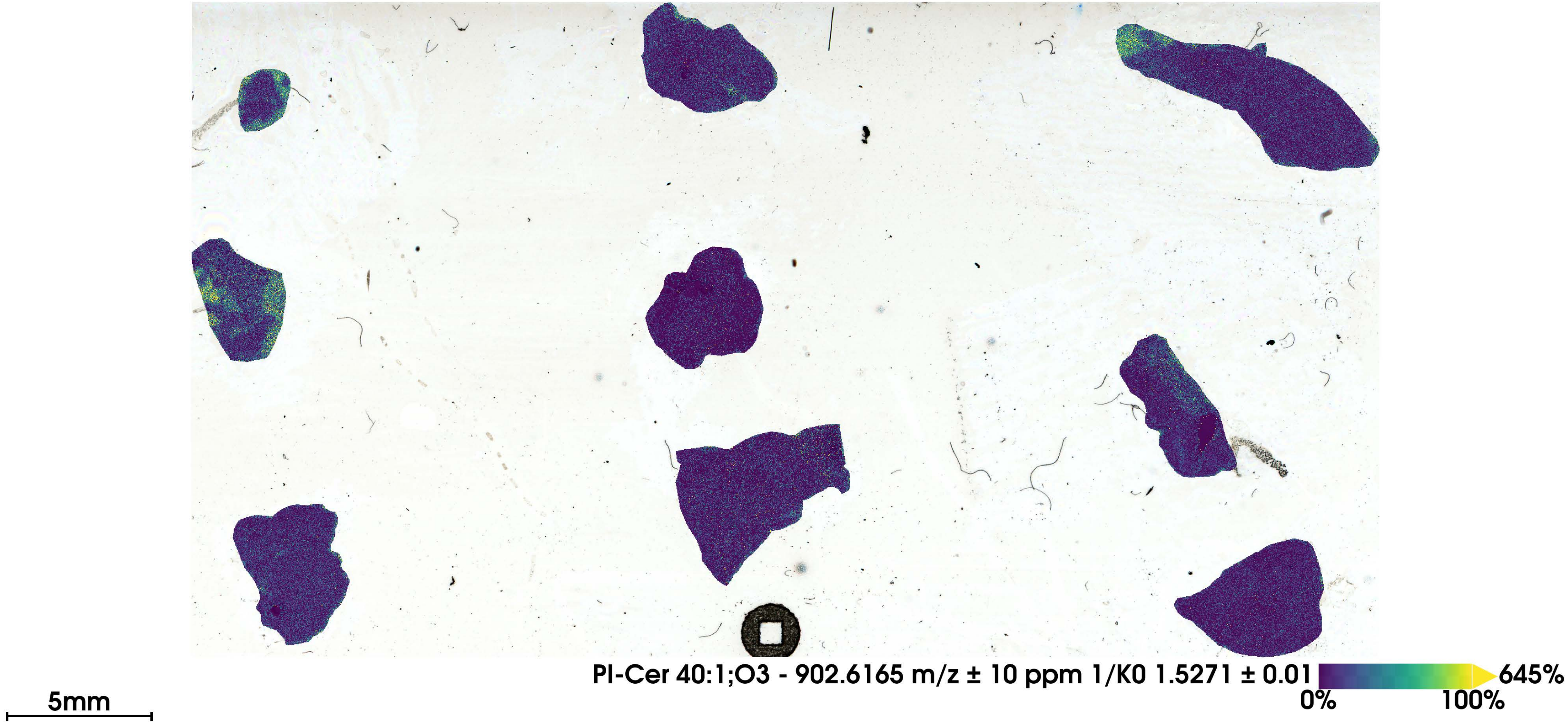

PI-Cer 40:1;O3 - 902.6165 m/z ± 10 ppm 1/K0 1.5271 ± 0.01
645%
0%
100%
5mm

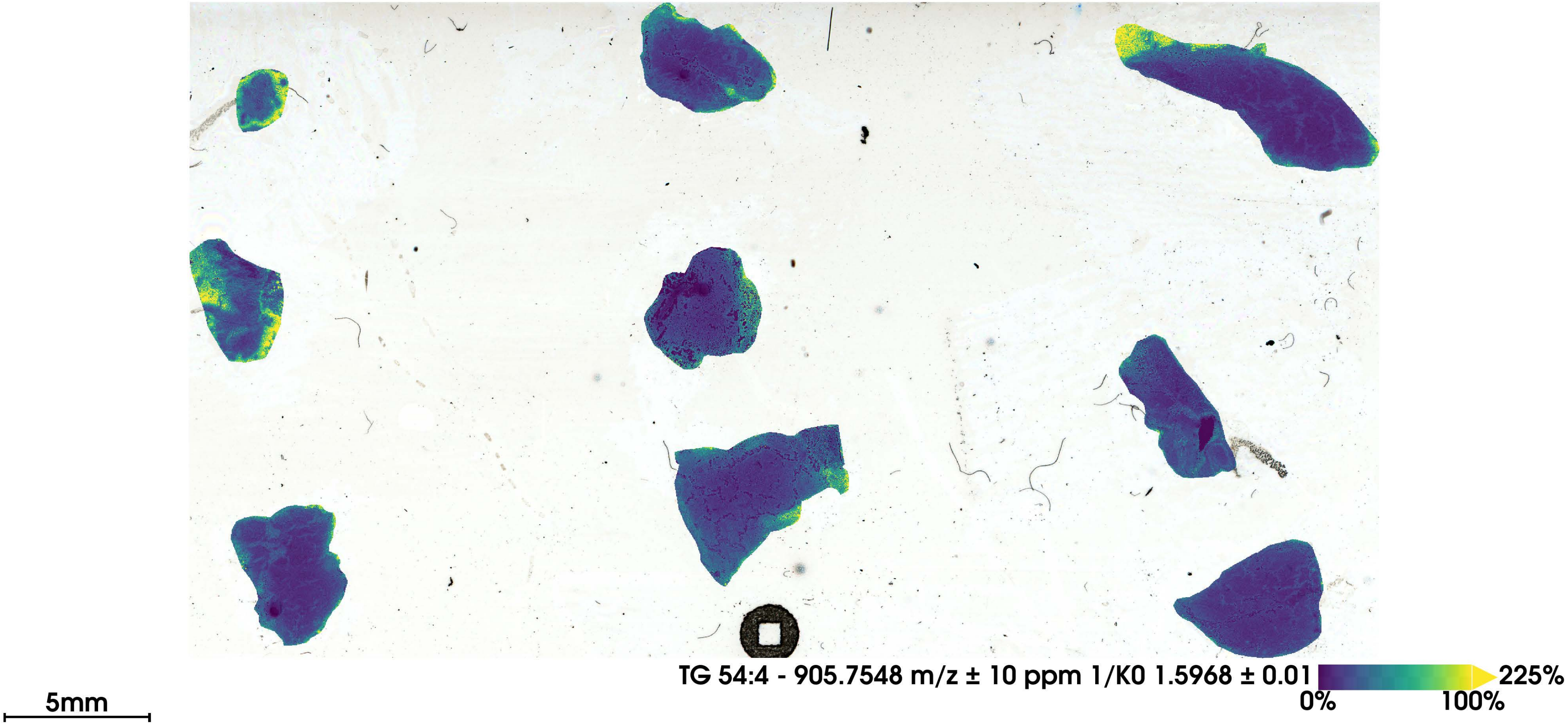
TG 54:4 - 905.7548 m/z ± 10 ppm 1/K0 1.5968 ± 0.01
225%
0%
100%
5mm

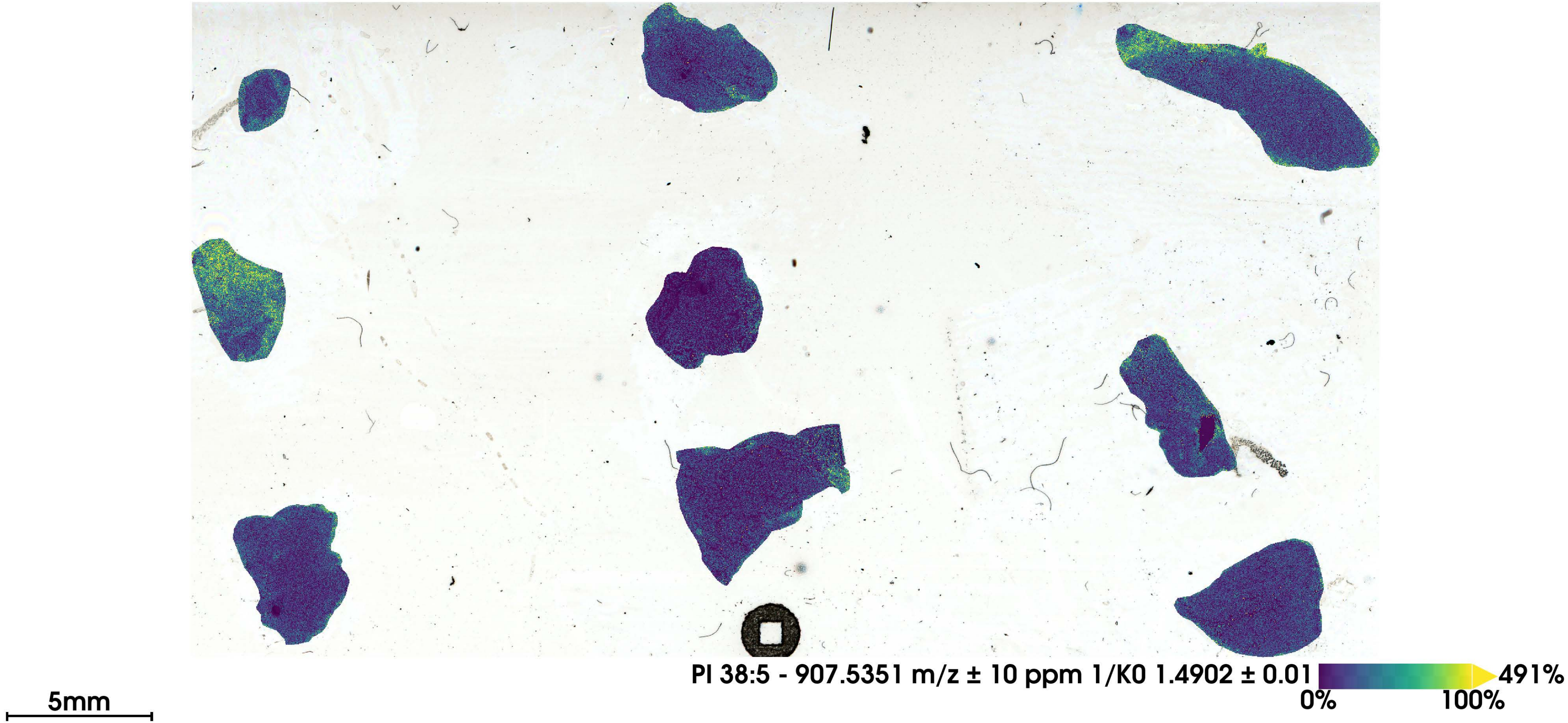
PI 38:5 - 907.5351 m/z ± 10 ppm 1/K0 1.4902 ± 0.01
491%
0%
100%
5mm

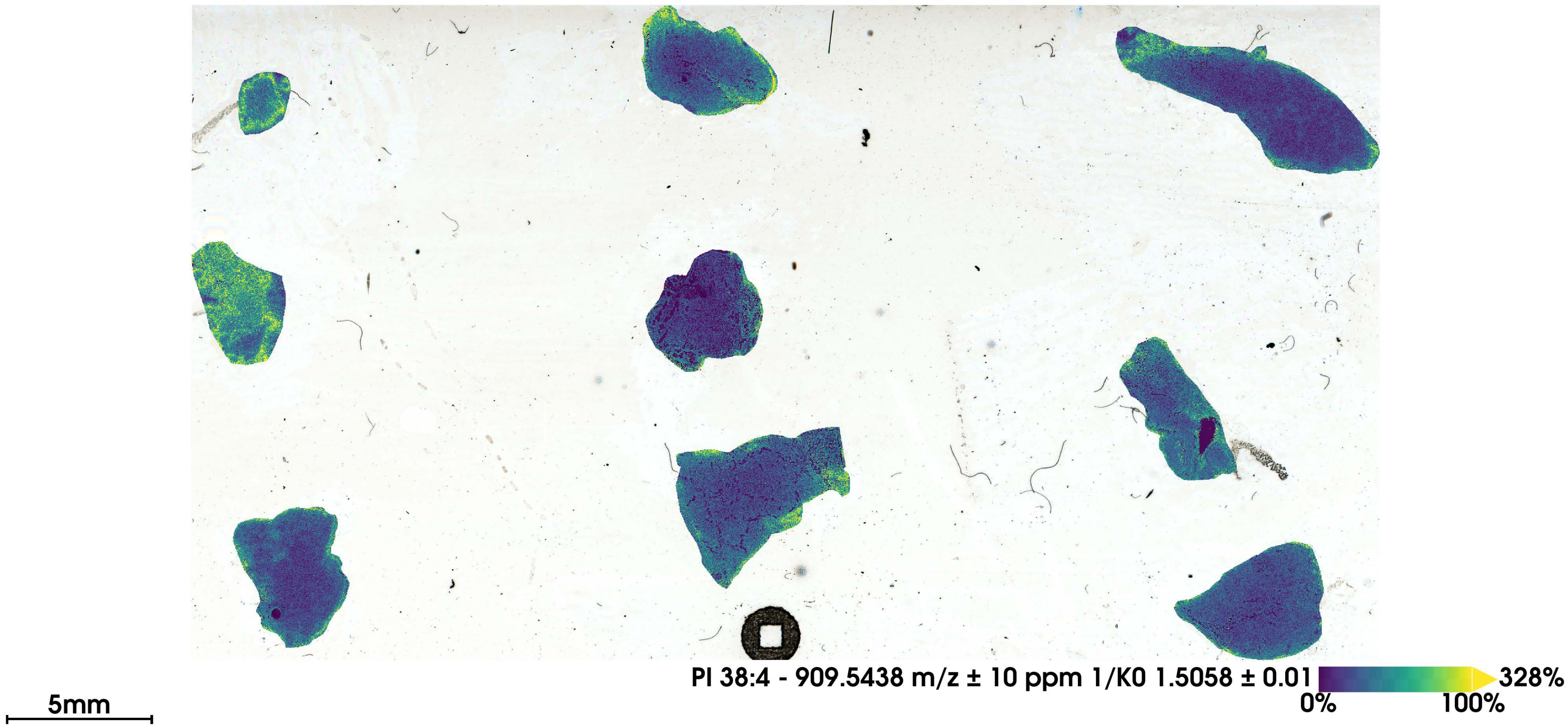
PI 38:4 - 909.5438 m/z ± 10 ppm 1/K0 1.5058 ± 0.01
328%
0%
100%
5mm

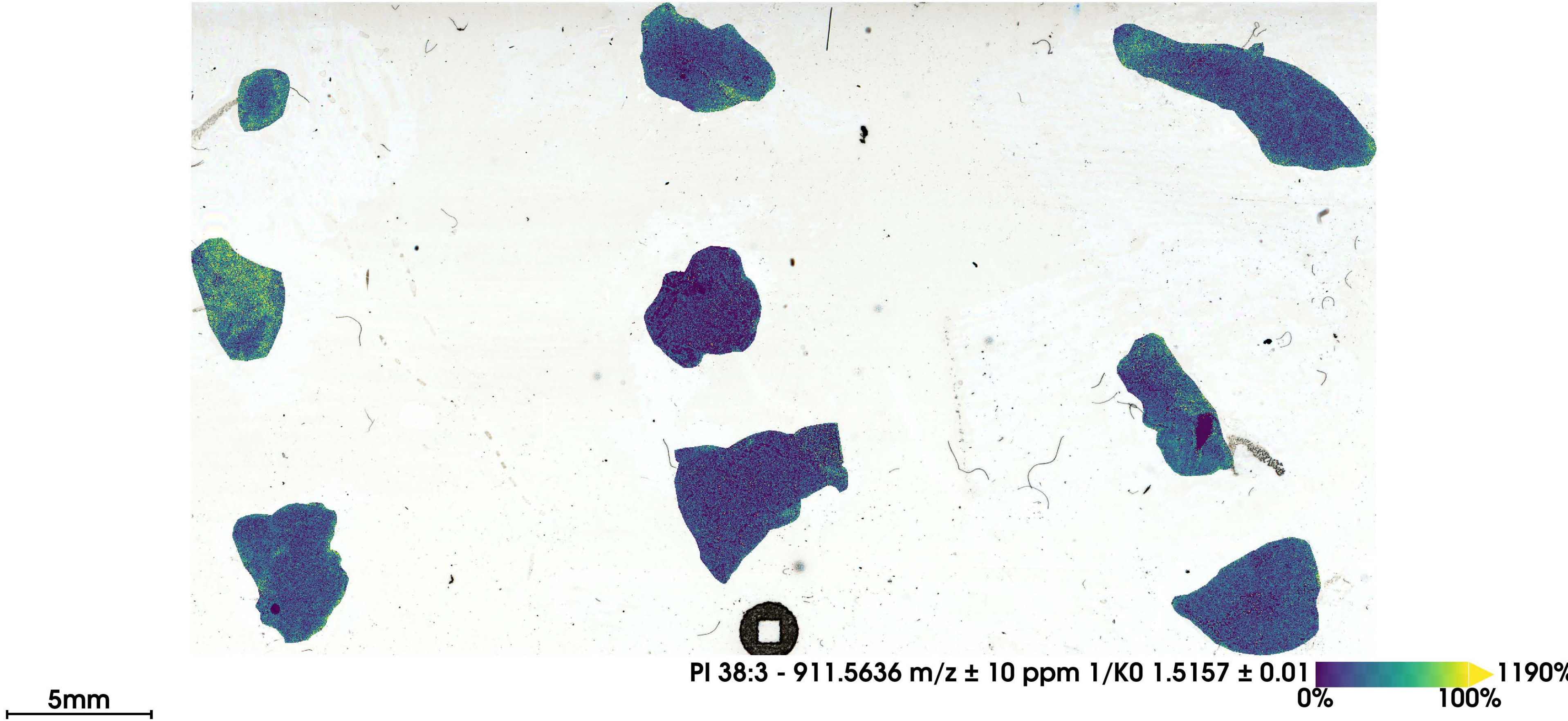
PI 38:3 - 911.5636 m/z ± 10 ppm 1/K0 1.5157 ± 0.01
0%
100%
1190%
5mm

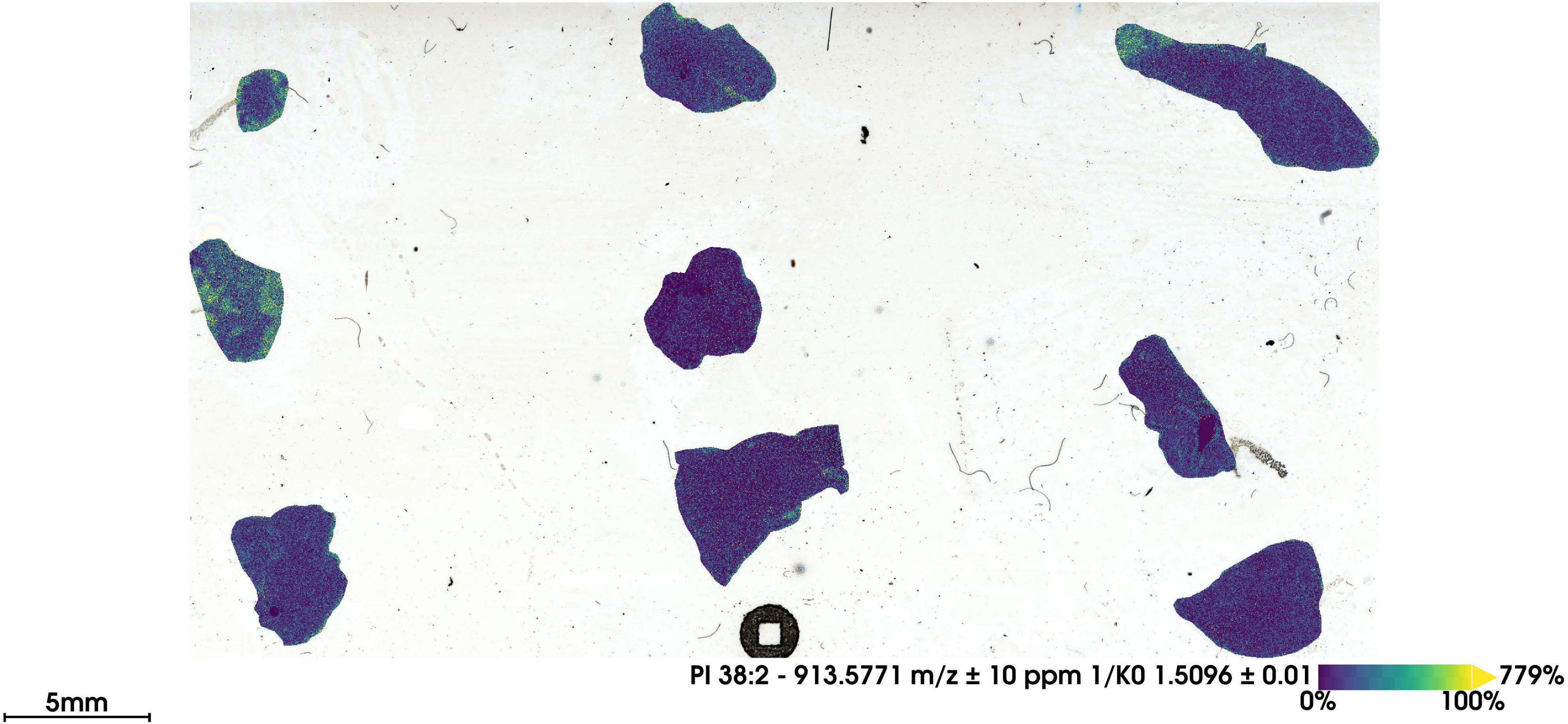
PI 38:2 - 913.5771 m/z ± 10 ppm 1/K0 1.5096 ± 0.01
779%
0%
100%
5mm

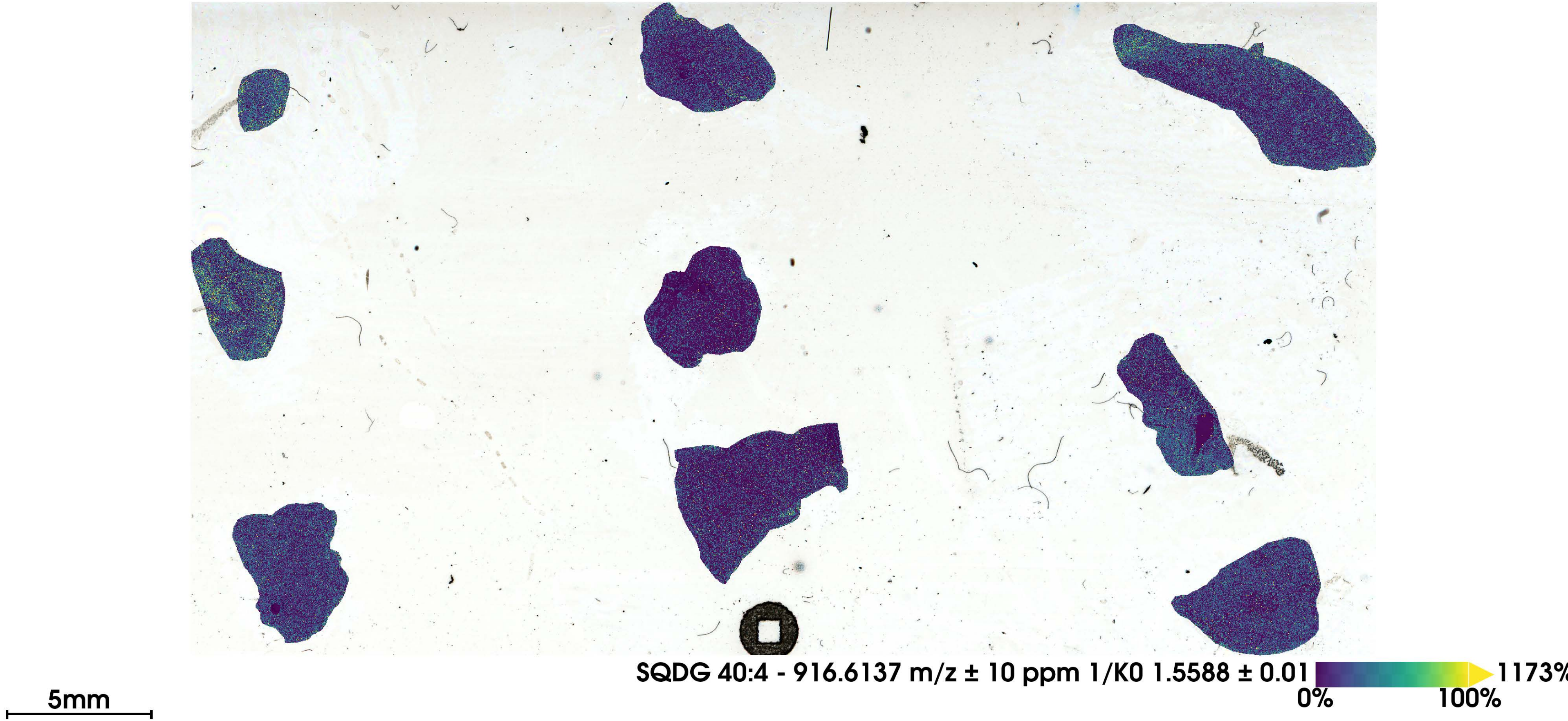

SQDG 40:4 - 916.6137 m/z ± 10 ppm 1/K0 1.5588 ± 0.01
1173%
0%
100%
5mm

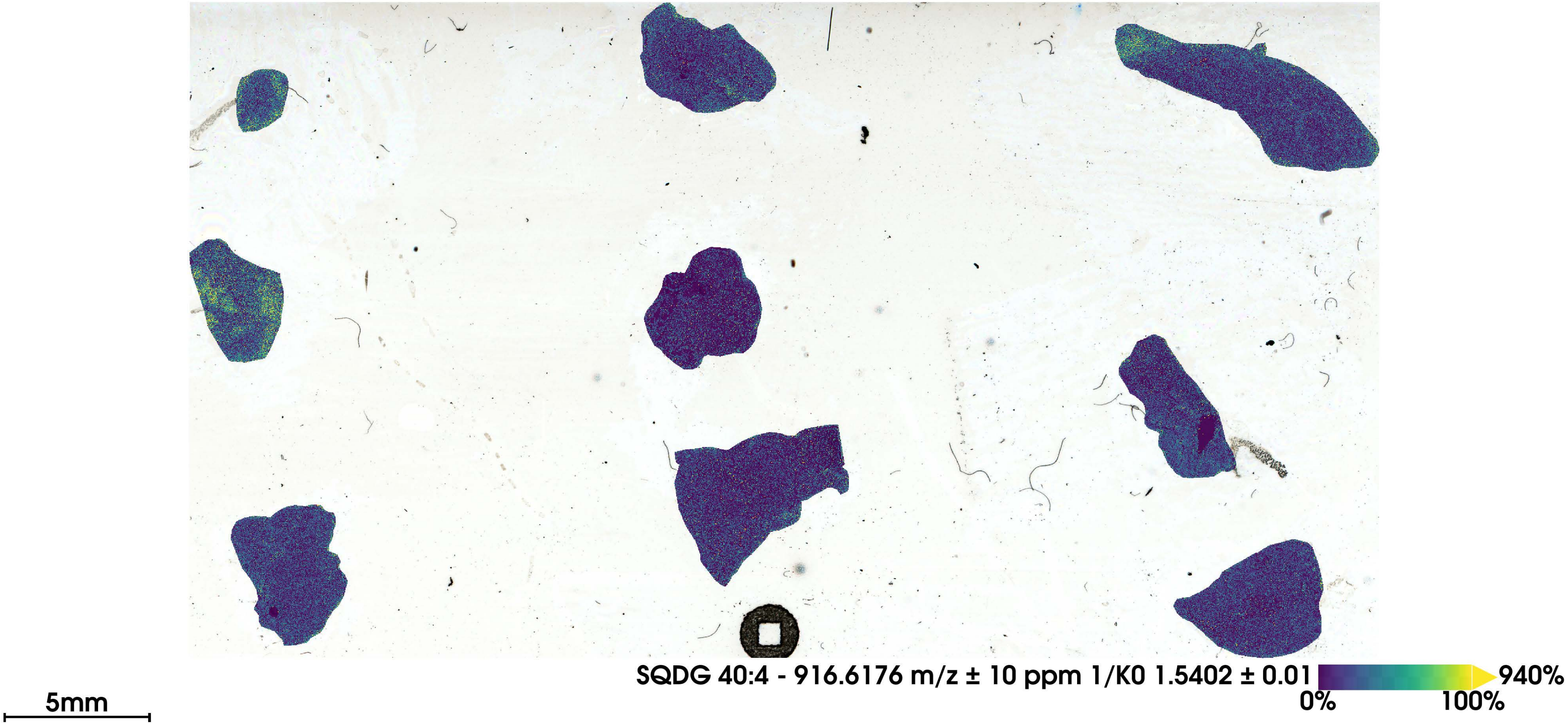

SQDG 40:4 - 916.6176 m/z ± 10 ppm 1/K0 1.5402 ± 0.01
940%
0%
100%
5mm

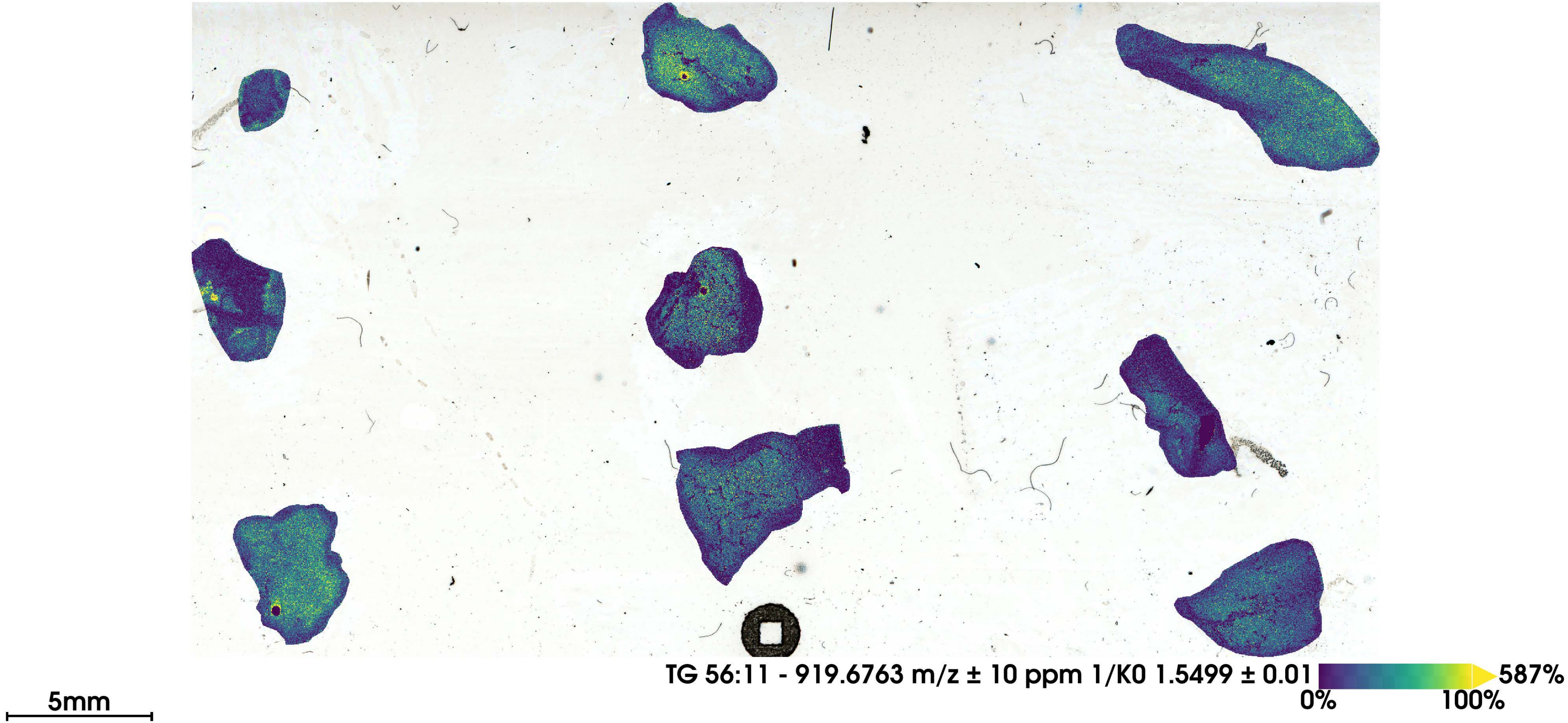
TG 56:11 - 919.6763 m/z ± 10 ppm 1/K0 1.5499 ± 0.01
587%
0%
100%
5mm

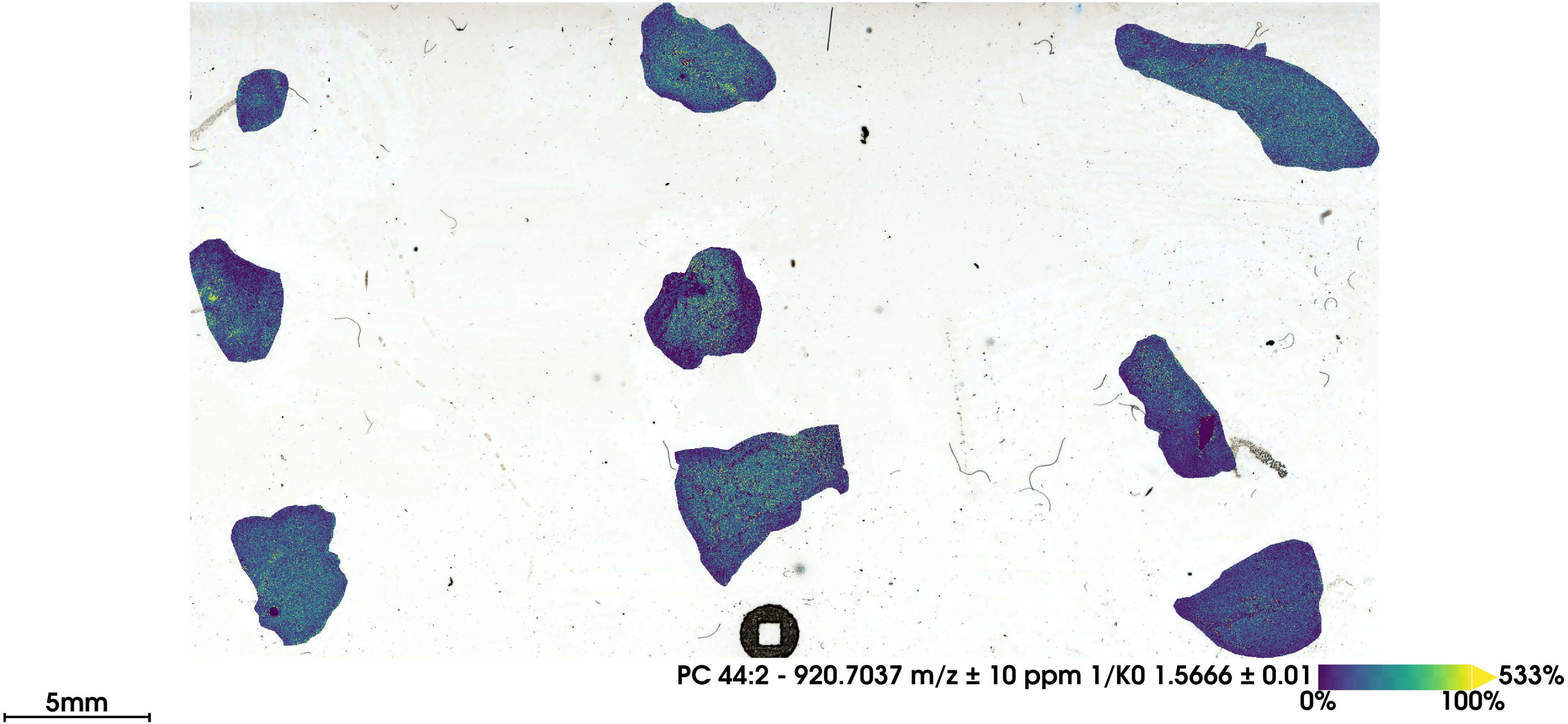

PC 44:2 - 920.7037 m/z ± 10 ppm 1/K0 1.5666 ± 0.01
533%
0%
100%
5mm

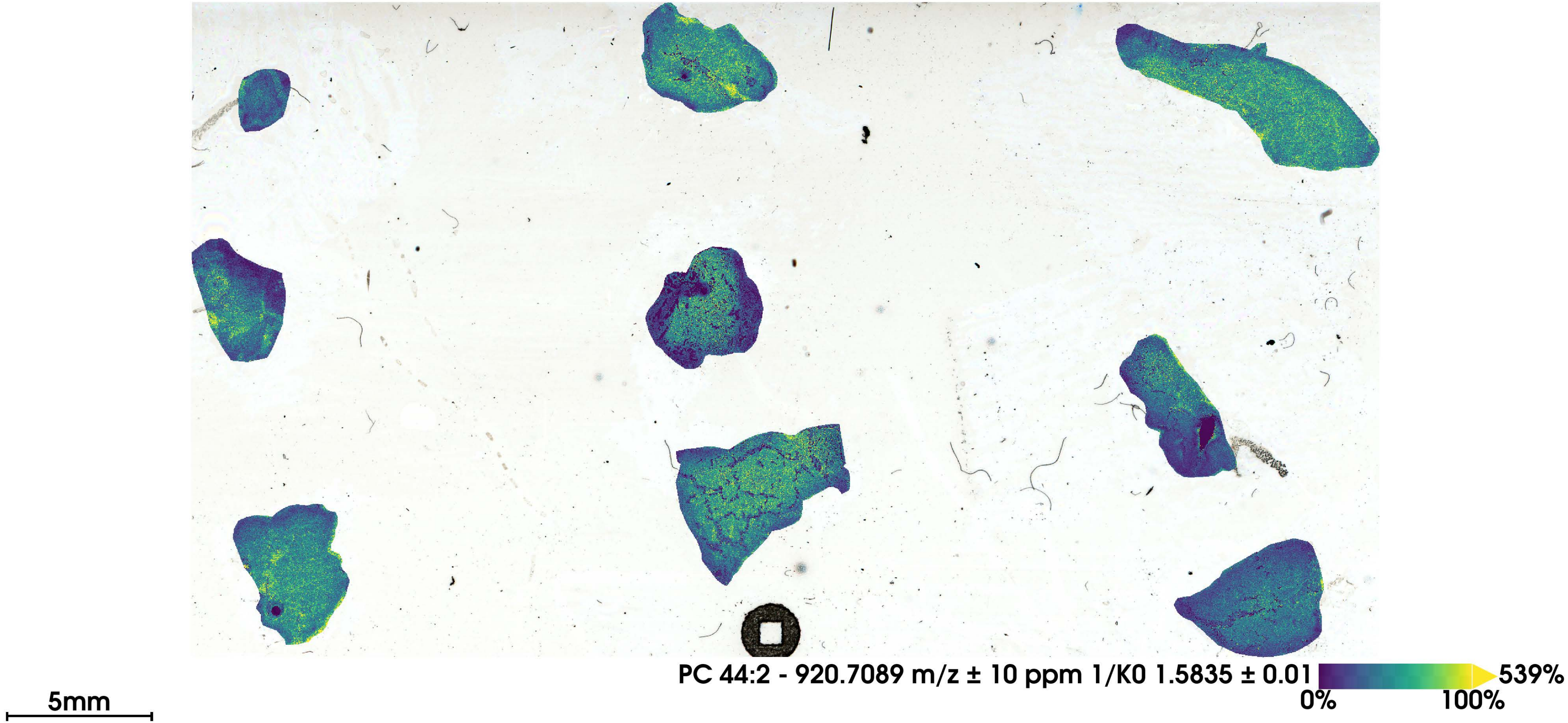
PC 44:2 - 920.7089 m/z ± 10 ppm 1/K0 1.5835 ± 0.01
539%
0%
100%
5mm

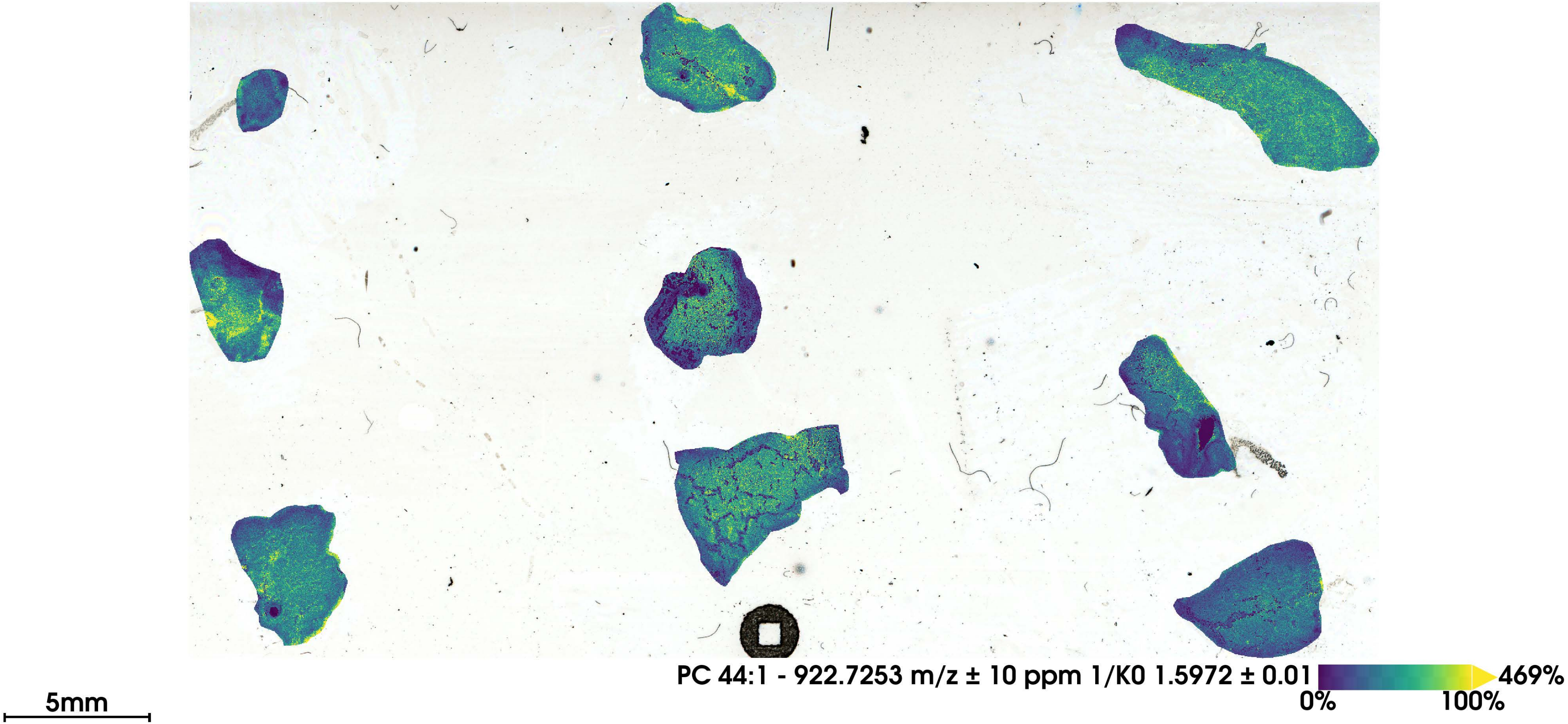
PC 44:1 - 922.7253 m/z ± 10 ppm 1/K0 1.5972 ± 0.01
469%
0%
100%
5mm

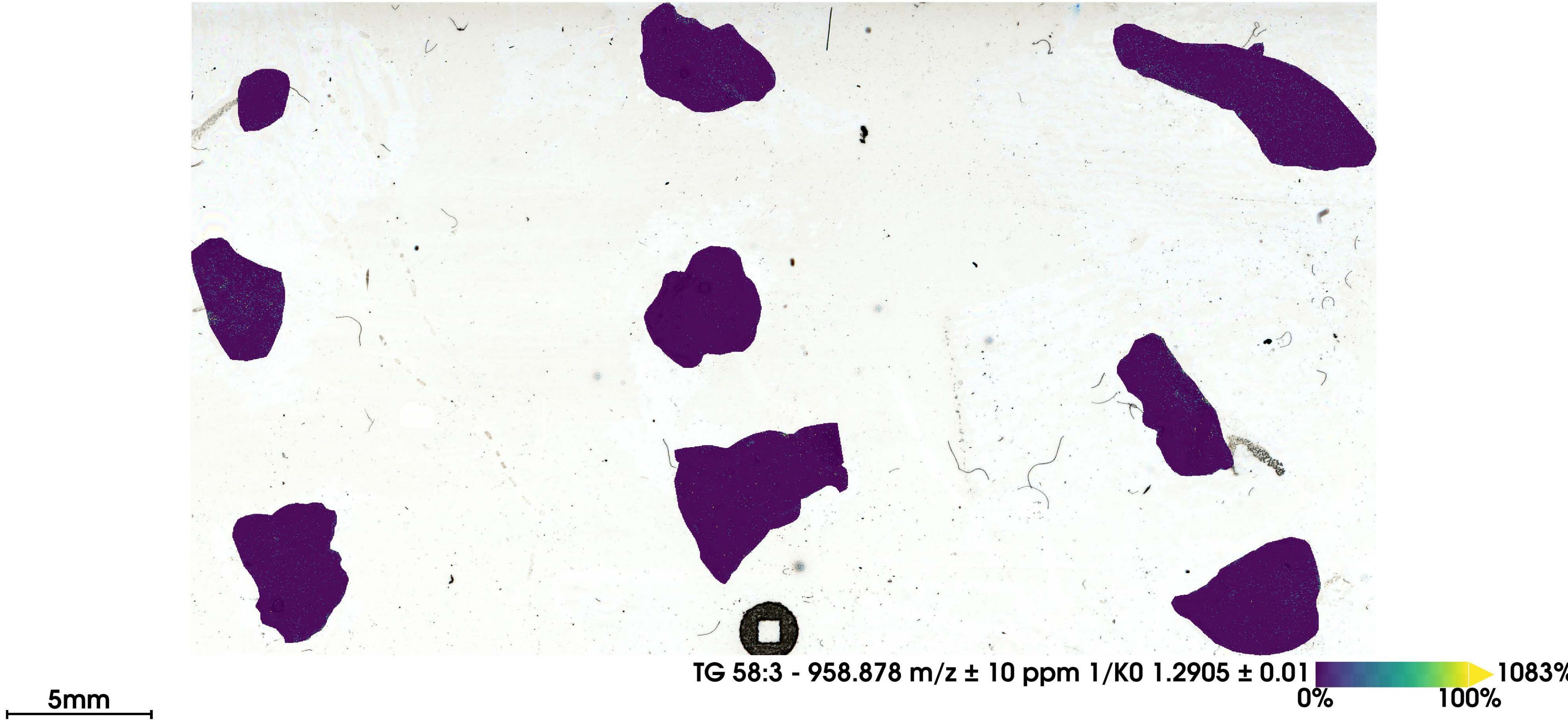

TG 58:3 - 958.878 m/z ± 10 ppm 1/K0 1.2905 ± 0.01
1083%
0%
100%
5mm

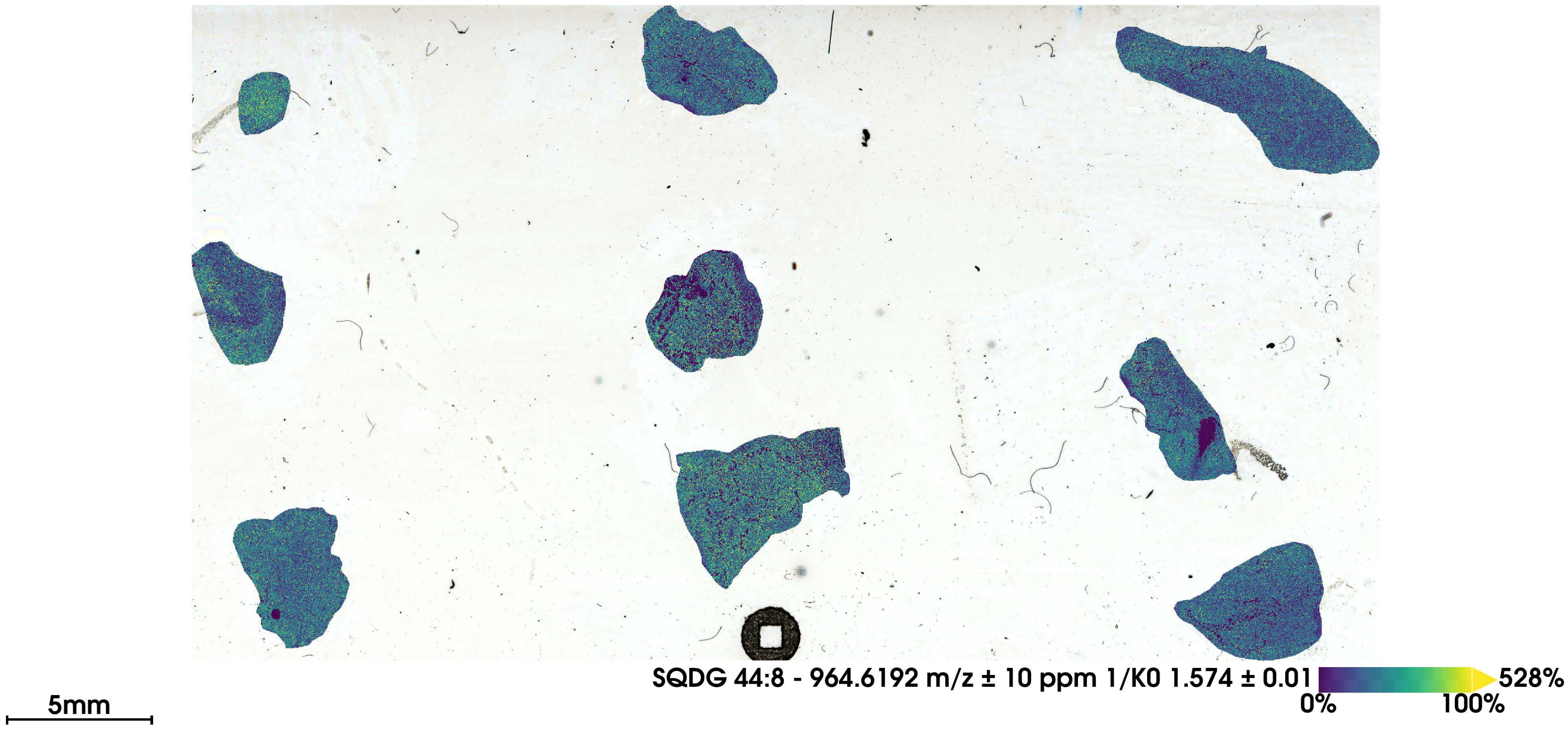

SQDG 44:8 - 964.6192 m/z ± 10 ppm 1/K0 1.574 ± 0.01
528%
0%
100%
5mm

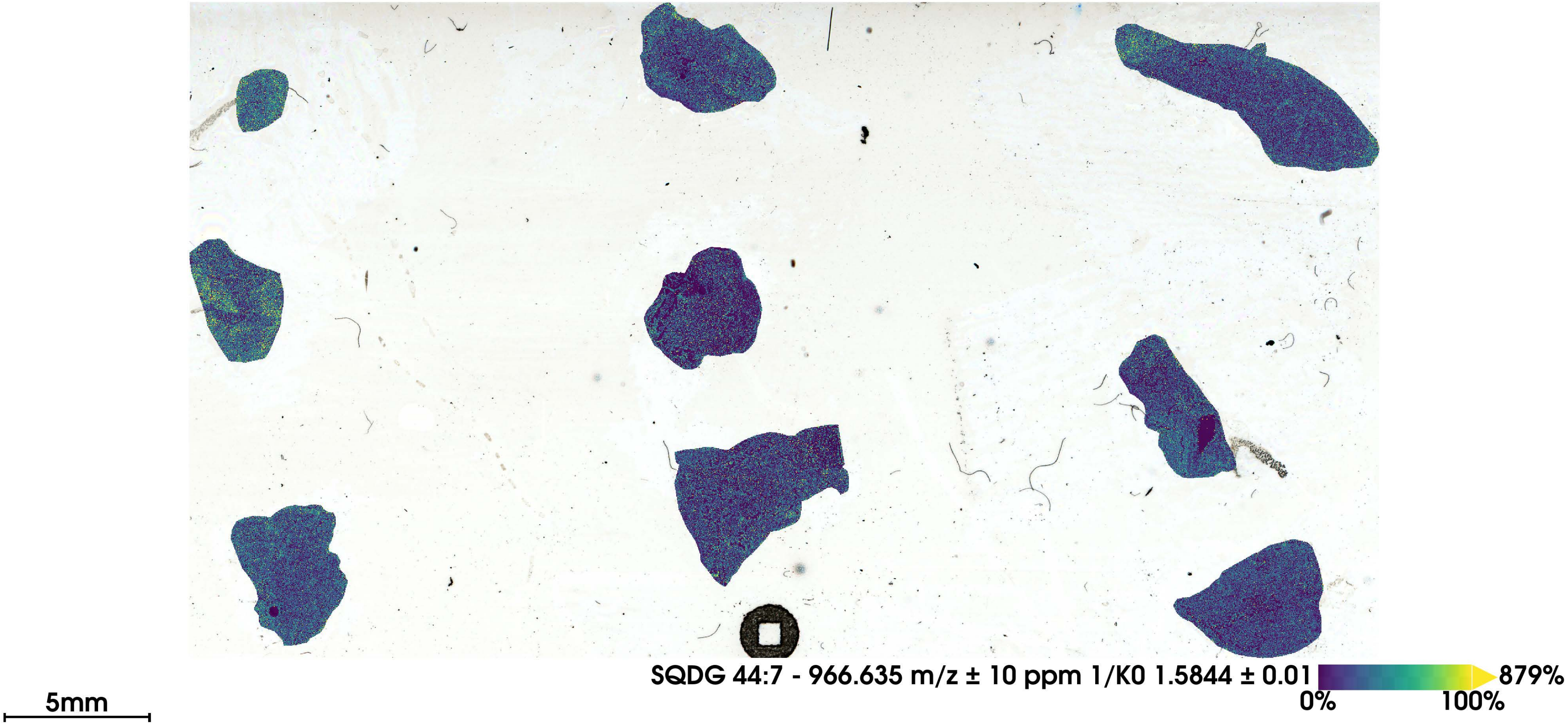
SQDG 44:7 - 966.635 m/z ± 10 ppm 1/K0 1.5844 ± 0.01
879%
0%
100%
5mm

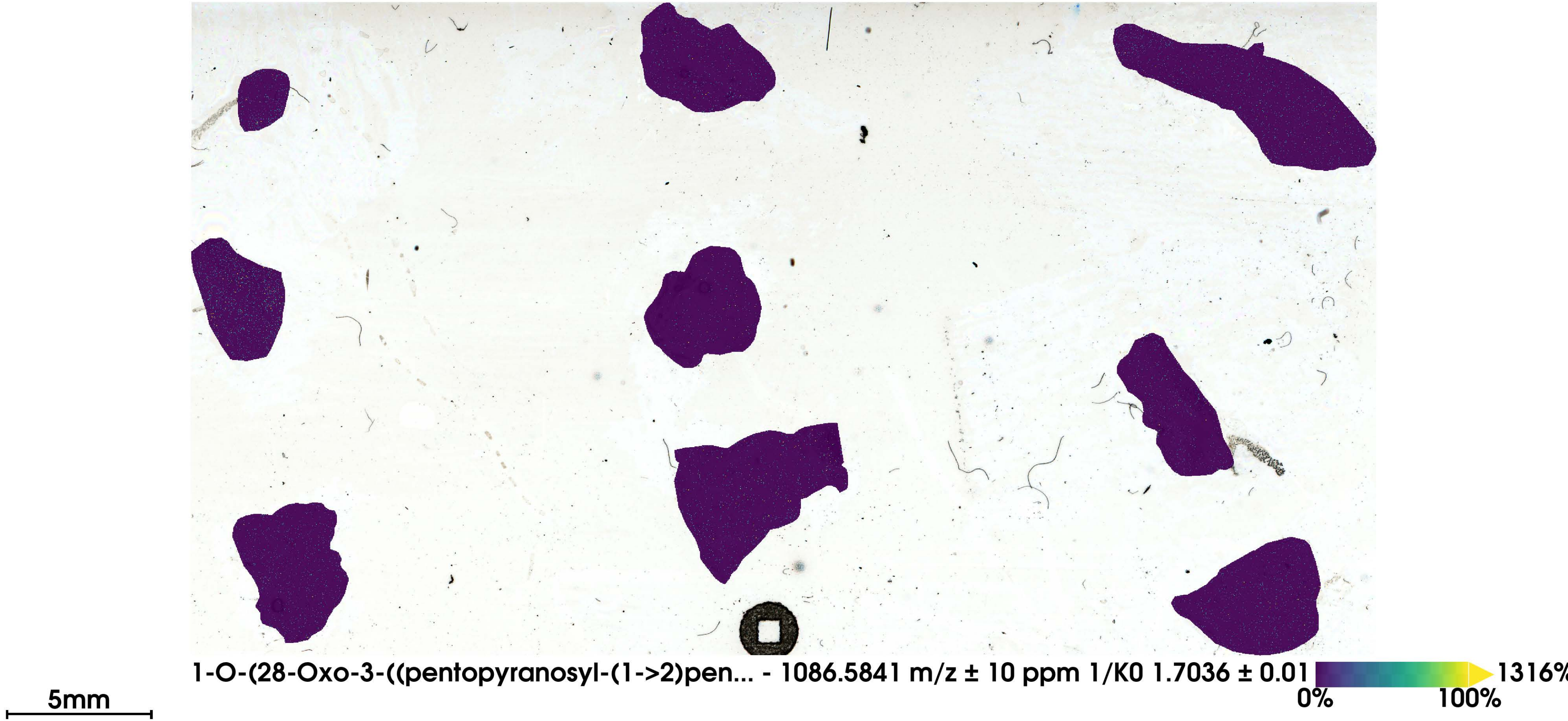

1-O-(28-Oxo-3-((pentopyranosyl-(1->2)pen... - 1086.5841 m/z ± 10 ppm 1/K0 1.7036 ± 0.01
1316%
0%
100%
5mm

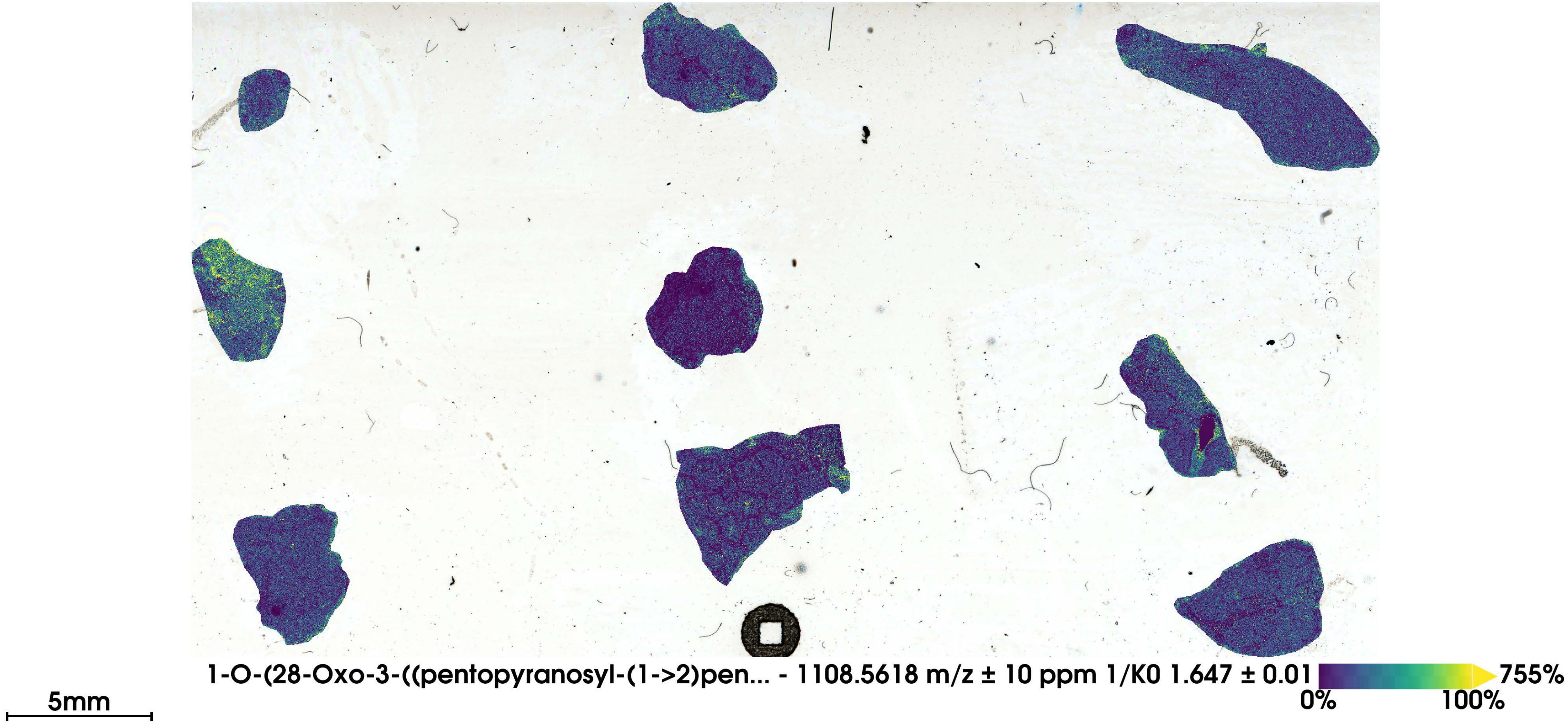
1-O-(28-Oxo-3-((pentopyranosyl-(1->2)pen... - 1108.5618 m/z ± 10 ppm 1/K0 1.647 ± 0.01
755%
0%
100%
5mm

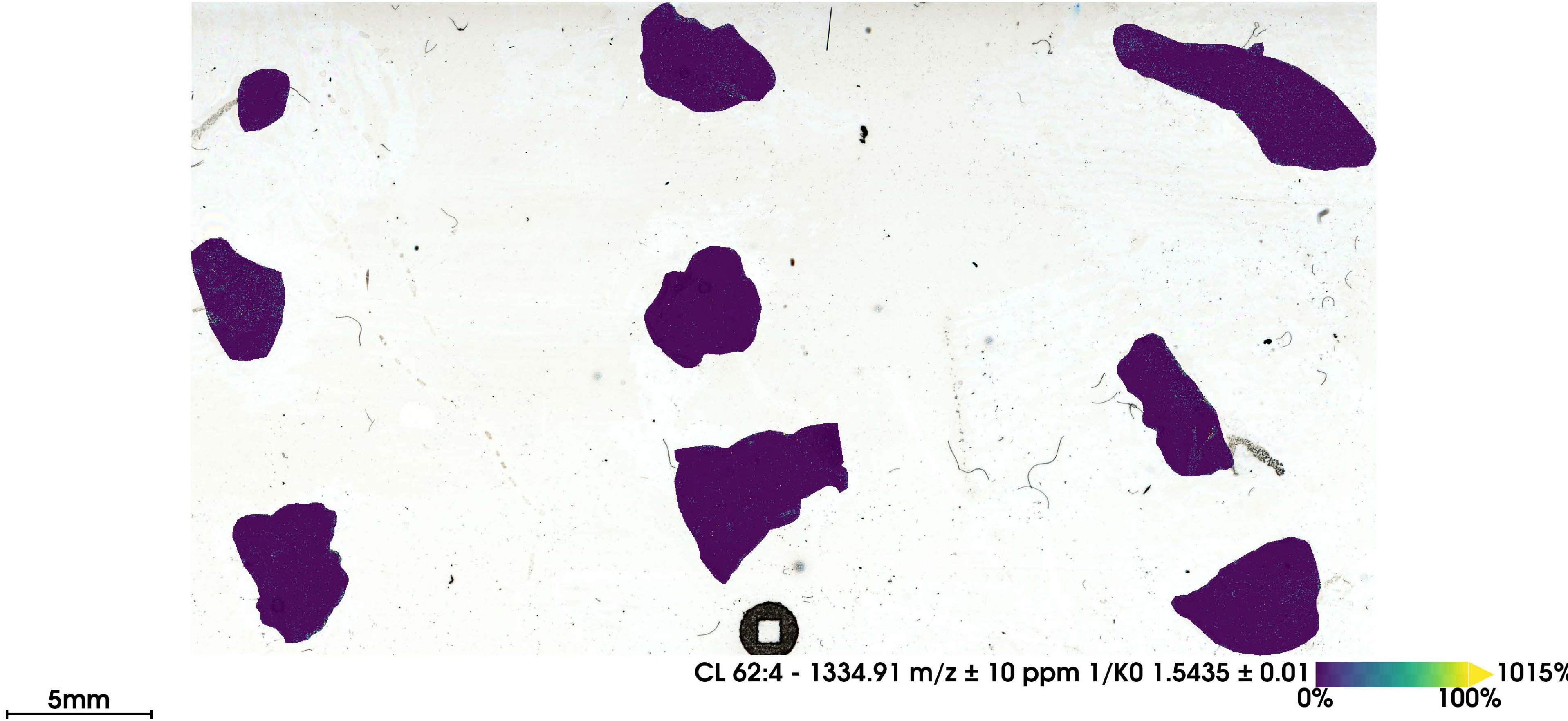

5mm
CL 62:4 - 1334.91 m/z ± 10 ppm 1/K0 1.5435 ± 0.01
0%
100%
1015%
