# Supplementary material for: Periodic fasting and refeeding re-shapes lipid saturation, storage, and distribution in brown adipose tissue
Source: PLoS Biol. 2026 Jan 12;24(1):e3003593. doi: 10.1371/journal.pbio.3003593 (PMC12795461; doi:10.1371/journal.pbio.3003593)
Supplement: S4 Raw Images — The results showed lipid and metabolite profile in Raptor KO BAT under AD, Refed, and FAS conditions at room temperature (22 ℃) (n = 5–6/group). (PDF) [file pbio.3003593.s019.pdf]

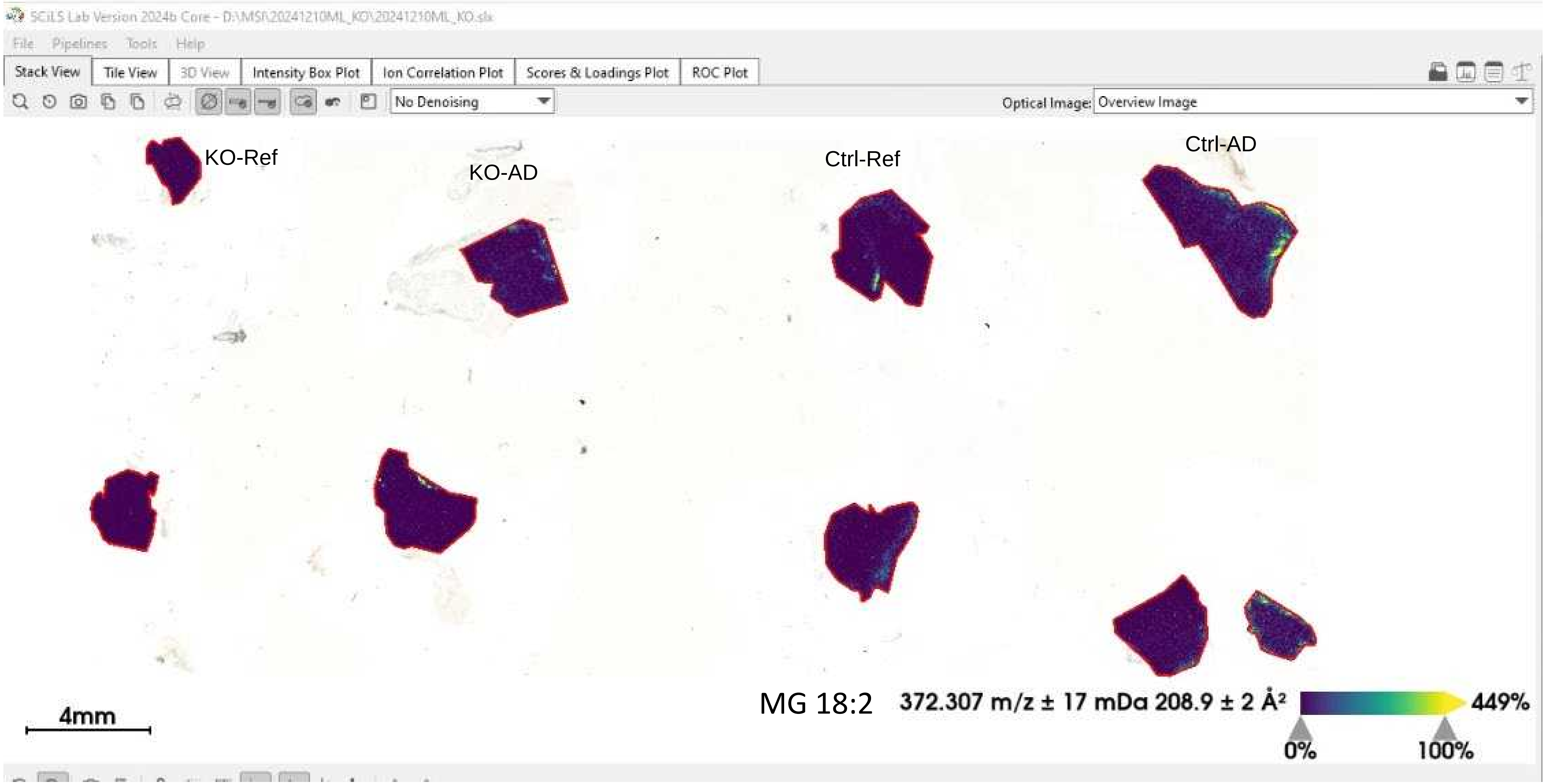

SCiLS Lab Version 2024b Core - D:\MSI\20241210ML_KO\20241210ML_KO.slx
File Pipelines Tools Help
Stack View
Tile View
3D View
Intensity Box Plot
Ion Correlation Plot
Scores & Loadings Plot
ROC Plot
No Denoising
Optical Image: Overview Image
KO-Ref
KO-AD
Ctrl-Ref
Ctrl-AD
4mm
MG 18:2
372.307 m/z ± 17 mDa 208.9 ± 2 Å²
449%
0%
100%

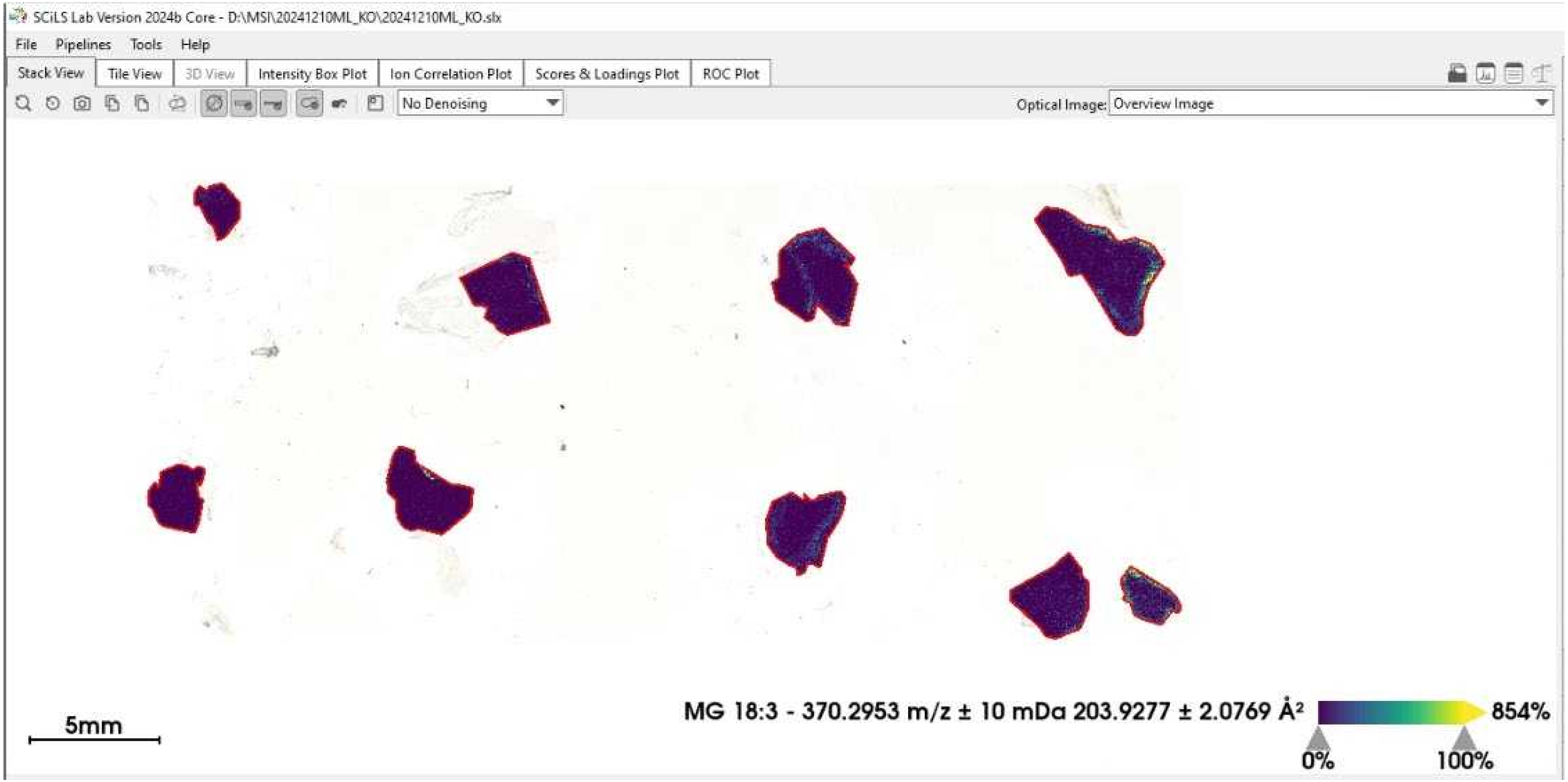
SCiLS Lab Version 2024b Core - D:\MSI\20241210ML_KO\20241210ML_KO.slx
File
Pipelines
Tools
Help
Stack View
Tile View
3D View
Intensity Box Plot
Ion Correlation Plot
Scores & Loadings Plot
ROC Plot
No Denoising
Optical Image:
Overview Image
5mm
MG 18:3 - 370.2953 m/z ± 10 mDa 203.9277 ± 2.0769 Å²
854%
0%
100%

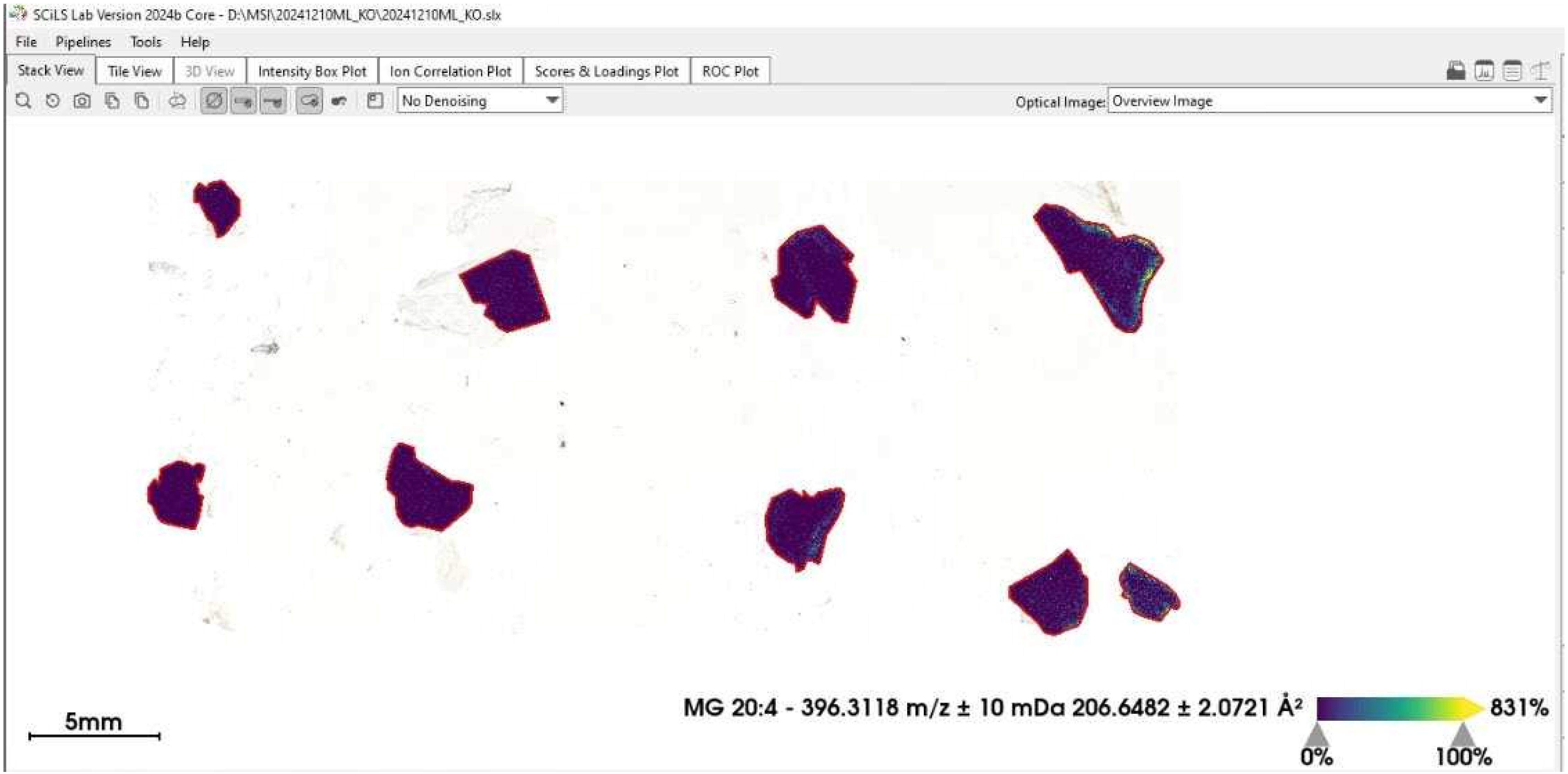
SCiLS Lab Version 2024b Core - D:\MSI\20241210ML_KO\20241210ML_KO.slx
File
Pipelines
Tools
Help
Stack View
Tile View
3D View
Intensity Box Plot
Ion Correlation Plot
Scores & Loadings Plot
ROC Plot
No Denoising
Optical Image:
Overview Image
5mm
MG 20:4 - 396.3118 m/z ± 10 mDa 206.6482 ± 2.0721 Å²
831%
0%
100%

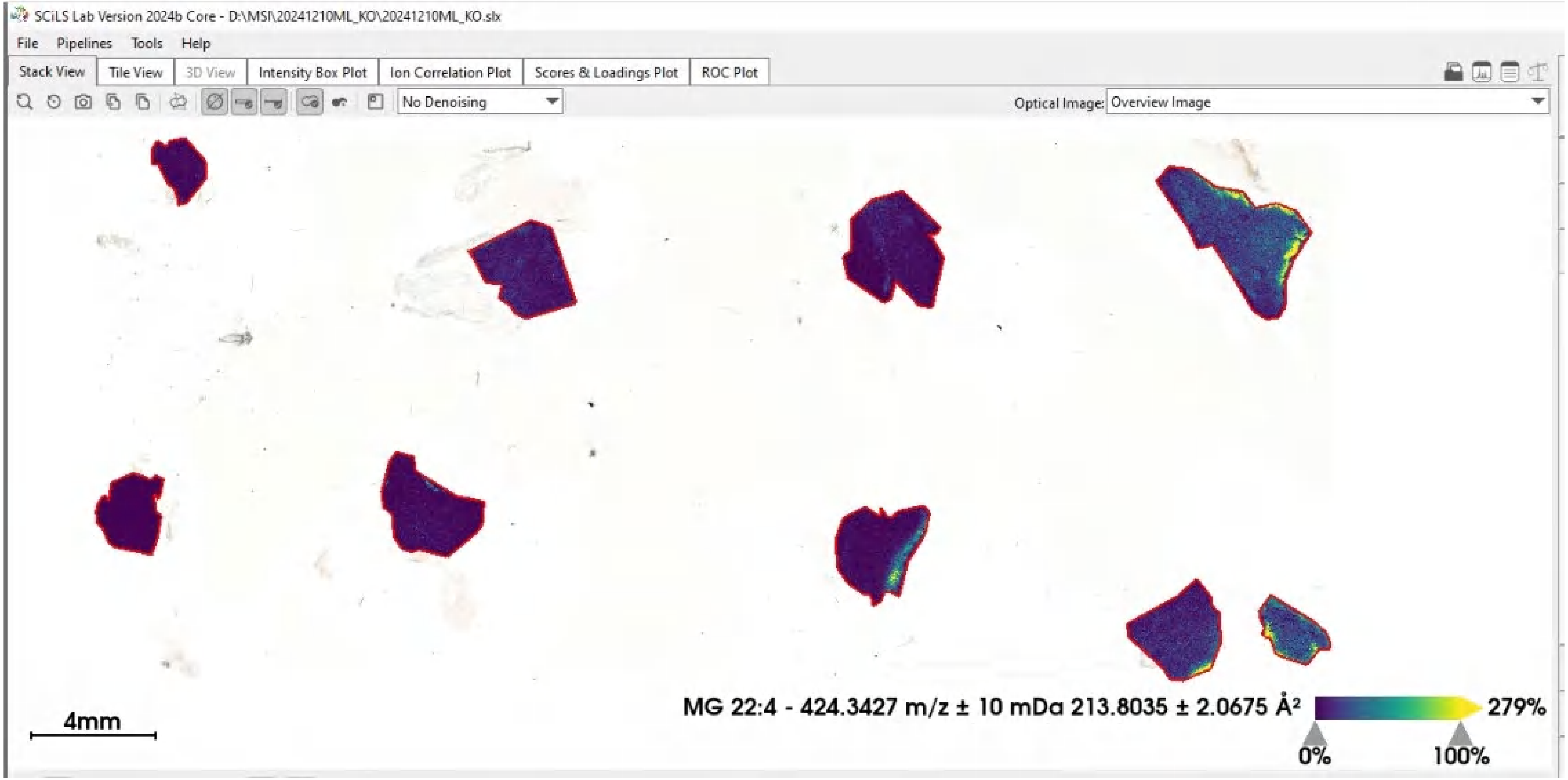
SCiLS Lab Version 2024b Core - D:\MSI\20241210ML_KO\20241210ML_KO.slx
File Pipelines Tools Help
Stack View
Tile View
3D View
Intensity Box Plot
Ion Correlation Plot
Scores & Loadings Plot
ROC Plot
No Denoising
Optical Image: Overview Image
4mm
MG 22:4 - 424.3427 m/z ± 10 mDa 213.8035 ± 2.0675 Å²
279%
0%
100%

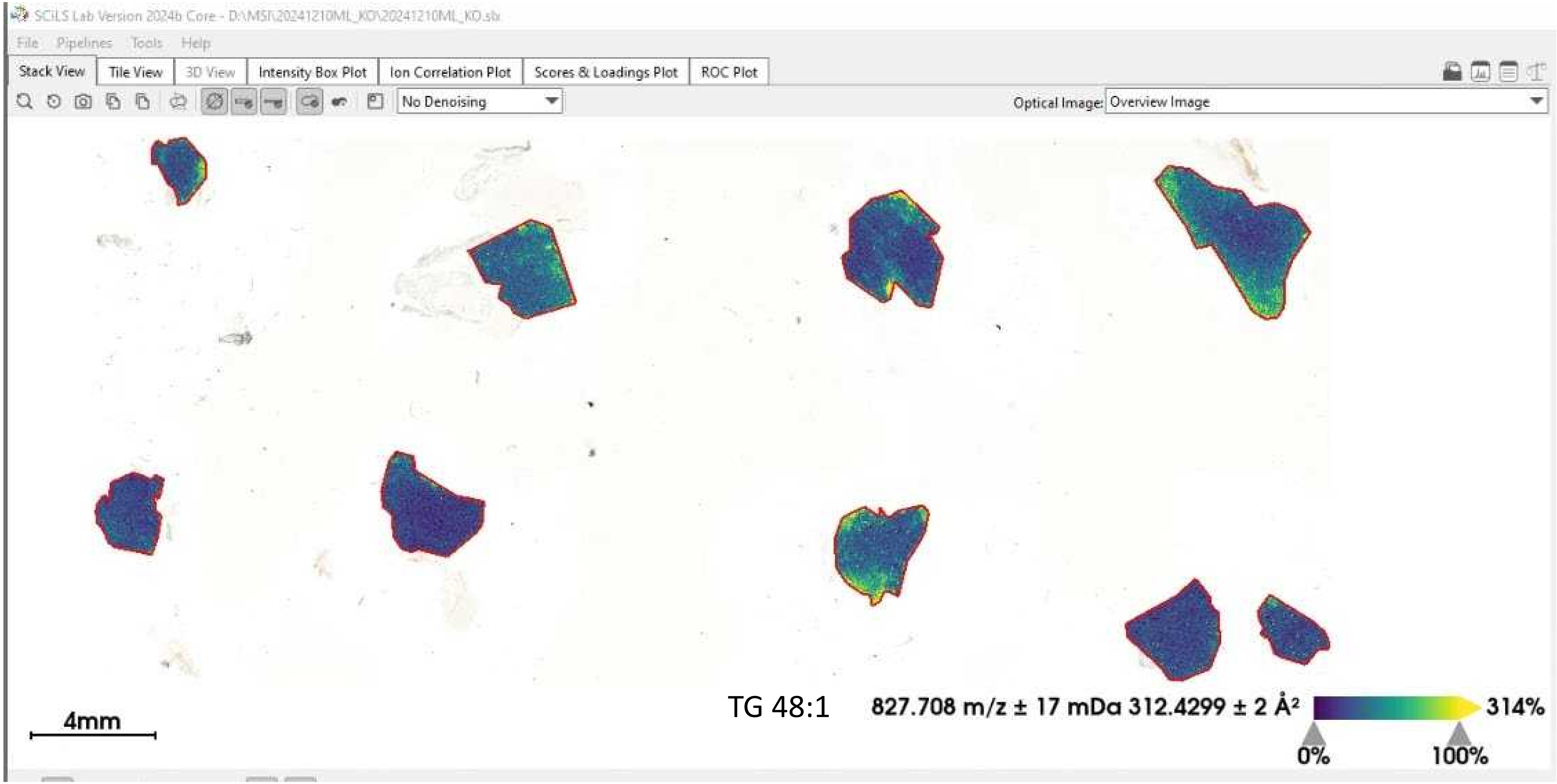
SCiLS Lab Version 2024b Core - D:\MSI\20241210ML_KO\20241210ML_KO.slx
File
Pipelines
Tools
Help
Stack View
Tile View
3D View
Intensity Box Plot
Ion Correlation Plot
Scores & Loadings Plot
ROC Plot
No Denoising
Optical Image:
Overview Image
4mm
TG 48:1
827.708 m/z ± 17 mDa 312.4299 ± 2 Å²
314%
0%
100%

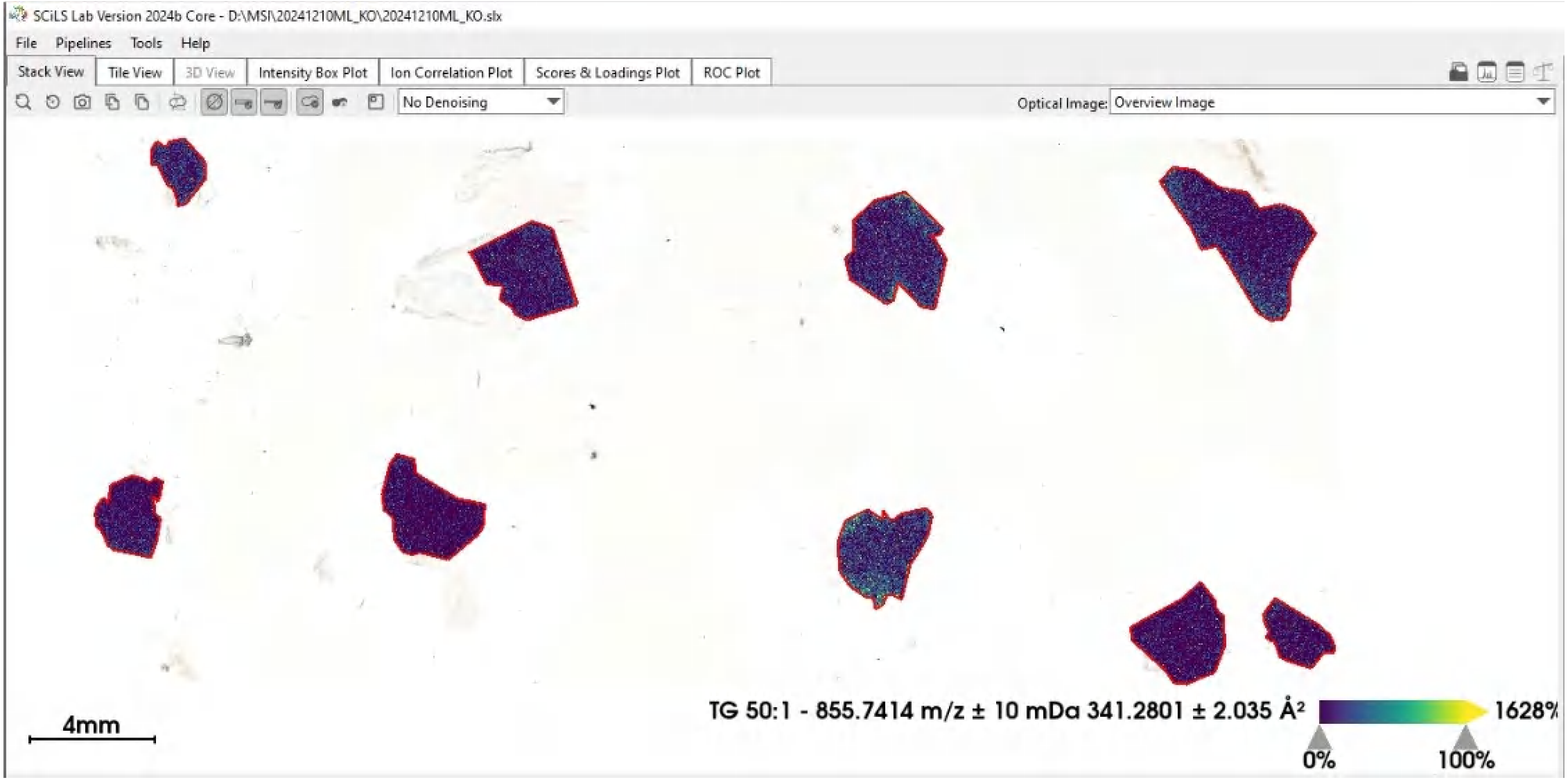
SCiLS Lab Version 2024b Core - D:\MSI\20241210ML_KO\20241210ML_KO.slx
File
Pipelines
Tools
Help
Stack View
Tile View
3D View
Intensity Box Plot
Ion Correlation Plot
Scores & Loadings Plot
ROC Plot
No Denoising
Optical Image:
Overview Image
4mm
TG 50:1 - 855.7414 m/z ± 10 mDa 341.2801 ± 2.035 Å²
0%
100%

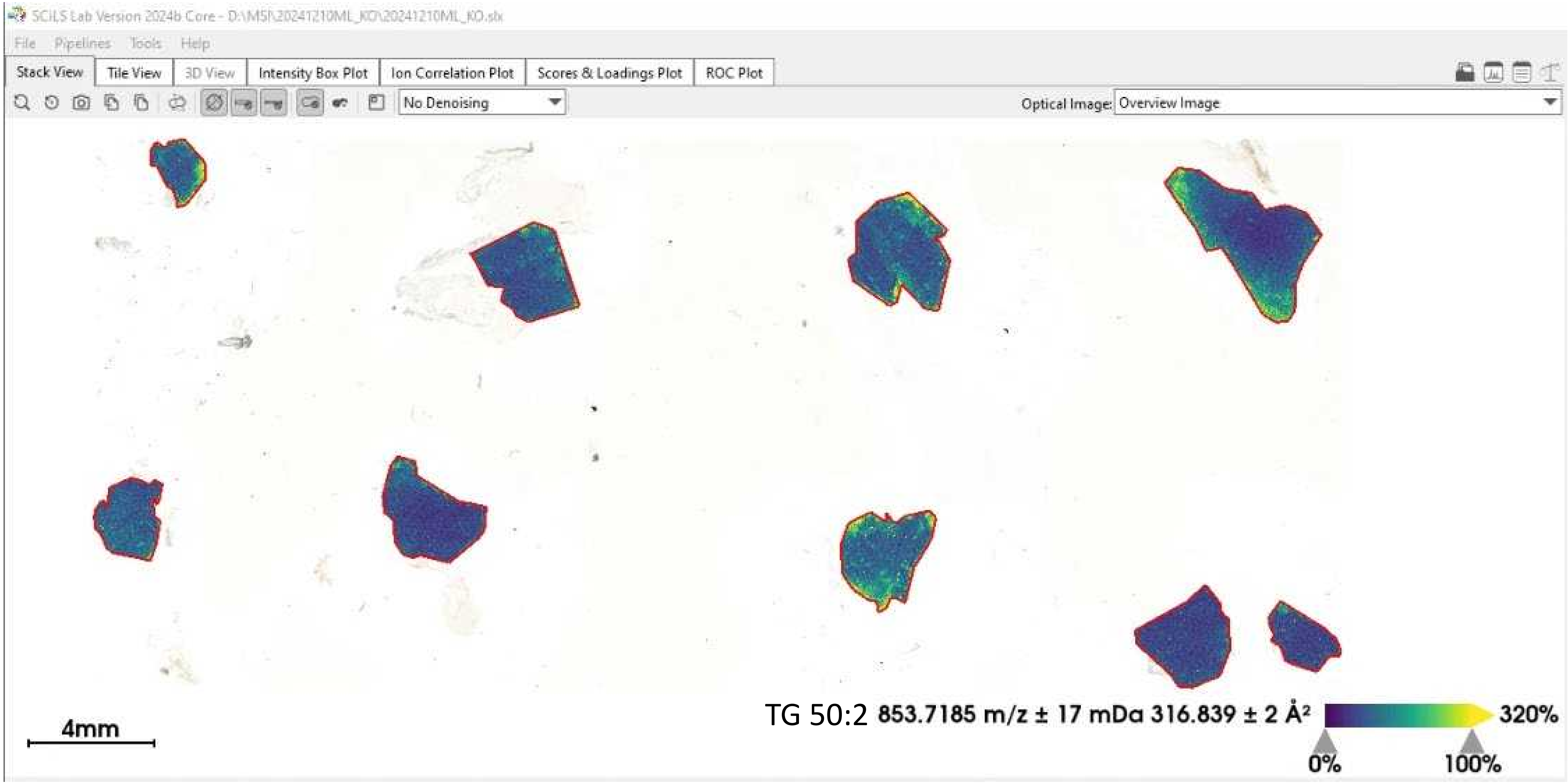
SCiLS Lab Version 2024b Core - D:\MSI\20241210ML_KO\20241210ML_KO.slx
File Pipelines Tools Help
Stack View
Tile View
3D View
Intensity Box Plot
Ion Correlation Plot
Scores & Loadings Plot
ROC Plot
No Denoising
Optical Image: Overview Image
4mm
TG 50:2 853.7185 m/z ± 17 mDa 316.839 ± 2 Å²
320%
0%
100%

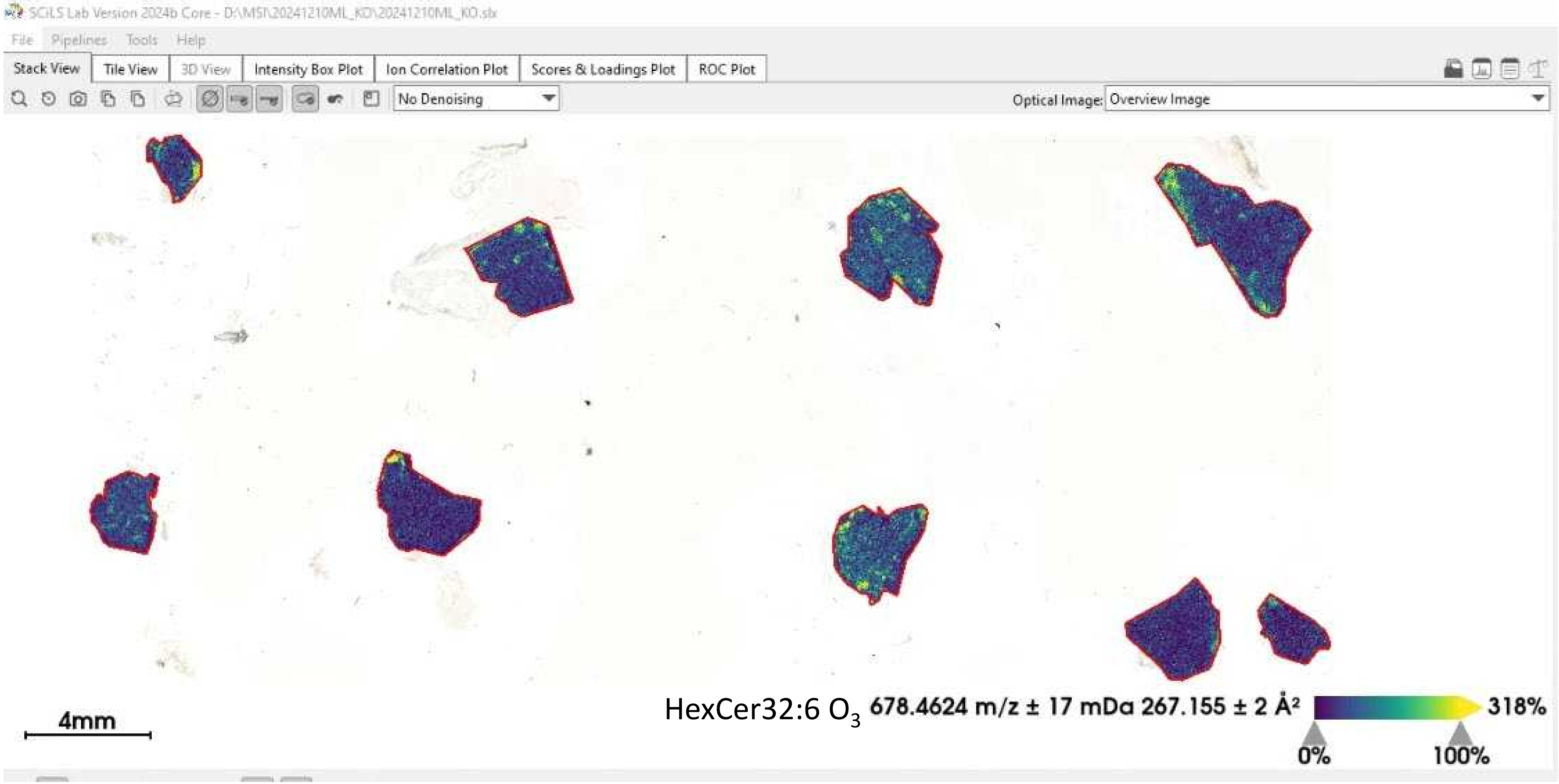
SCiLS Lab Version 2024b Core - D:\MSI\20241210ML_KO\20241210ML_KO.slx
File
Pipelines
Tools
Help
Stack View
Tile View
3D View
Intensity Box Plot
Ion Correlation Plot
Scores & Loadings Plot
ROC Plot
No Denoising
Optical Image:
Overview Image
4mm
HexCer32:6 O3 678.4624 m/z ± 17 mDa 267.155 ± 2 Å²
318%
0%
100%

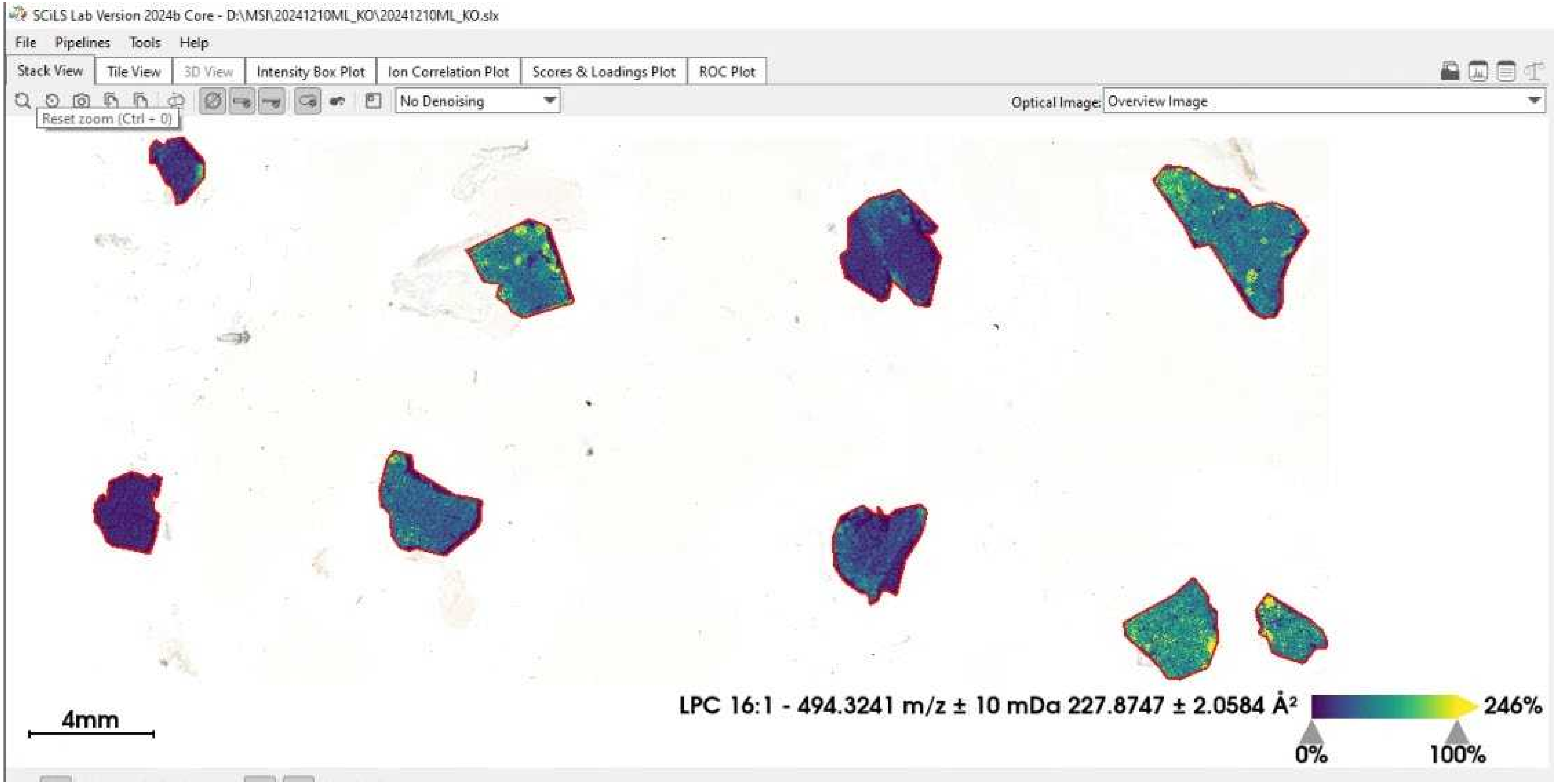
SCiLS Lab Version 2024b Core - D:\MSI\20241210ML_KO\20241210ML_KO.slx
File
Pipelines
Tools
Help
Stack View
Tile View
3D View
Intensity Box Plot
Ion Correlation Plot
Scores & Loadings Plot
ROC Plot
No Denoising
Optical Image:
Overview Image
Reset zoom (Ctrl + 0)
4mm
LPC 16:1 - 494.3241 m/z ± 10 mDa 227.8747 ± 2.0584 Å²
246%
0%
100%

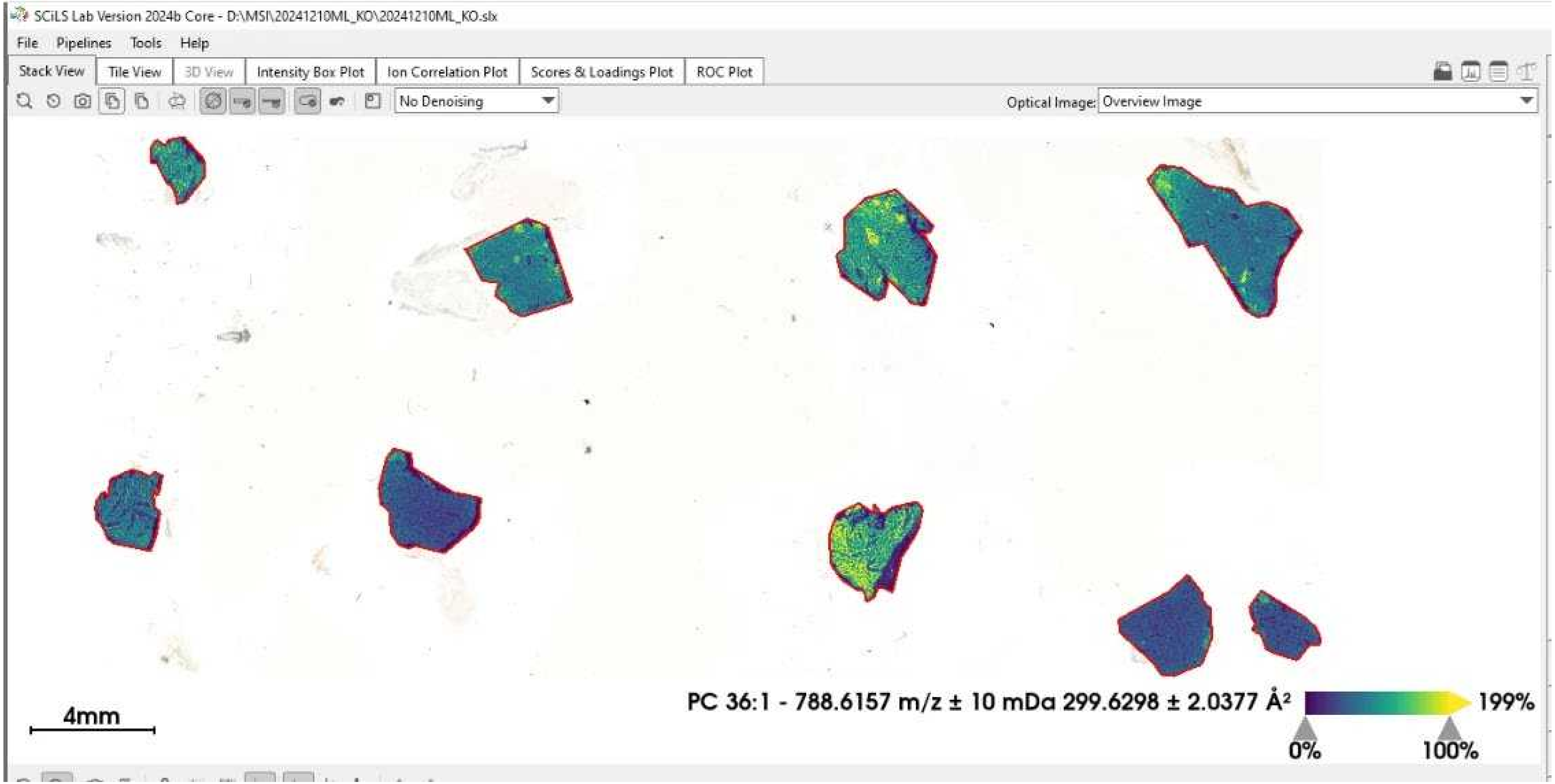
SCiLS Lab Version 2024b Core - D:\MSI\20241210ML_KO\20241210ML_KO.slx
File
Pipelines
Tools
Help
Stack View
Tile View
3D View
Intensity Box Plot
Ion Correlation Plot
Scores & Loadings Plot
ROC Plot
No Denoising
Optical Image:
Overview Image
4mm
PC 36:1 - 788.6157 m/z ± 10 mDa 299.6298 ± 2.0377 Å²
199%
0%
100%

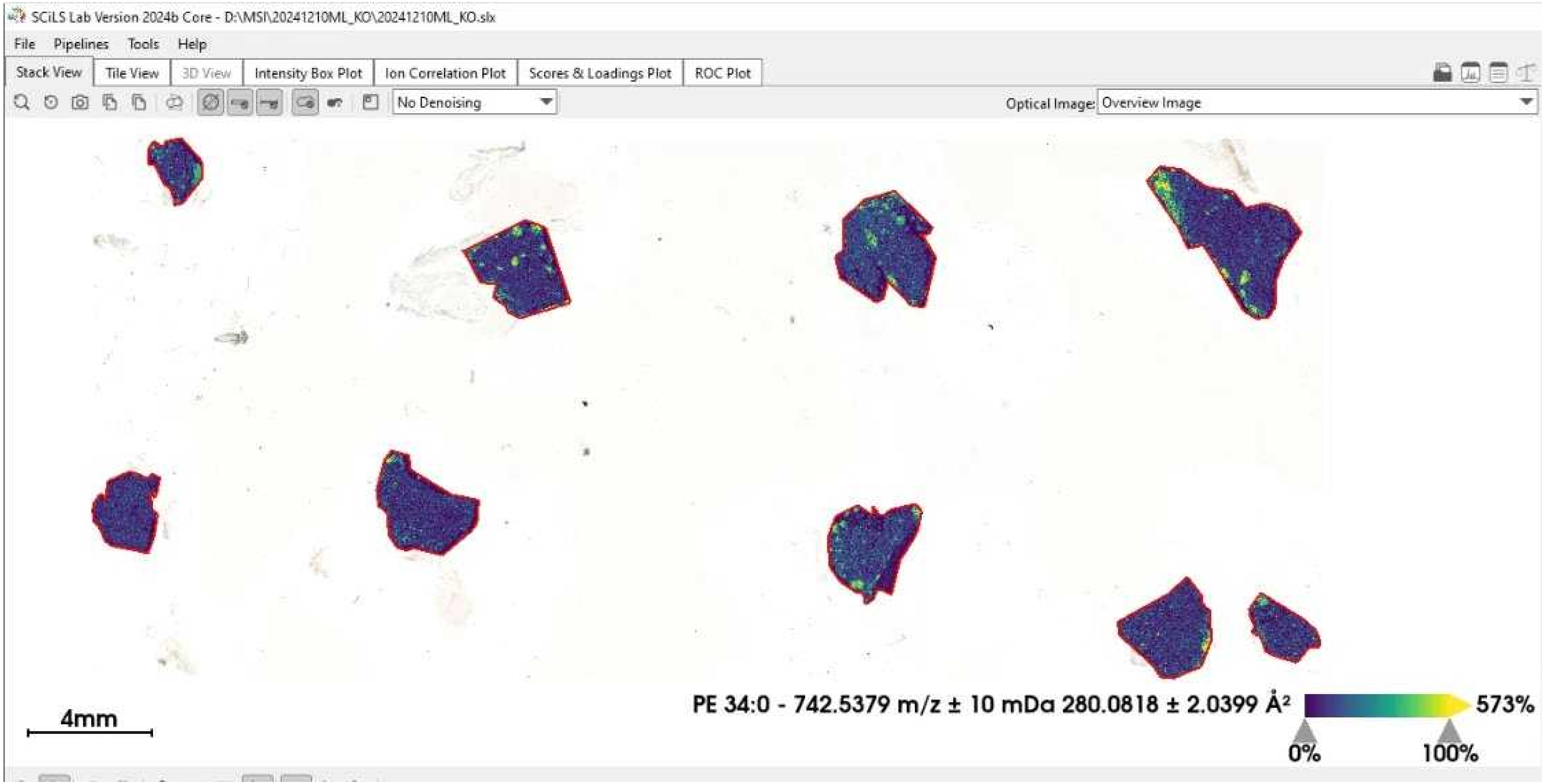
SCiLS Lab Version 2024b Core - D:\MSI\20241210ML_KO\20241210ML_KO.slx
File
Pipelines
Tools
Help
Stack View
Tile View
3D View
Intensity Box Plot
Ion Correlation Plot
Scores & Loadings Plot
ROC Plot
No Denoising
Optical Image:
Overview Image
4mm
PE 34:0 - 742.5379 m/z ± 10 mDa 280.0818 ± 2.0399 Å²
573%
0%
100%

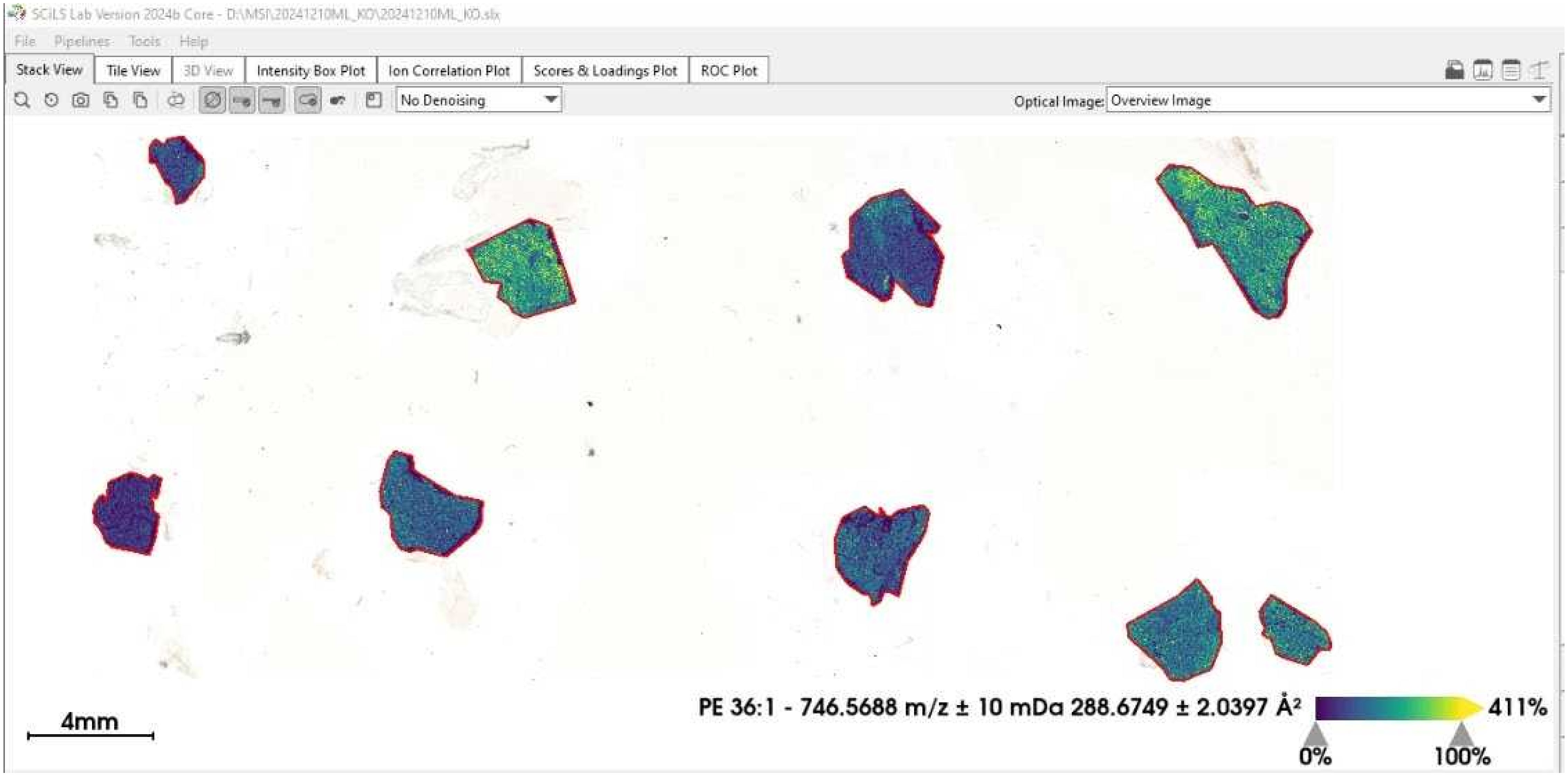
SCiLS Lab Version 2024b Core - D:\MSI\20241210ML_KO\20241210ML_KO.slx
File
Pipelines
Tools
Help
Stack View
Tile View
3D View
Intensity Box Plot
Ion Correlation Plot
Scores & Loadings Plot
ROC Plot
No Denoising
Optical Image:
Overview Image
4mm
PE 36:1 - 746.5688 m/z ± 10 mDa 288.6749 ± 2.0397 Å²
411%
0%
100%

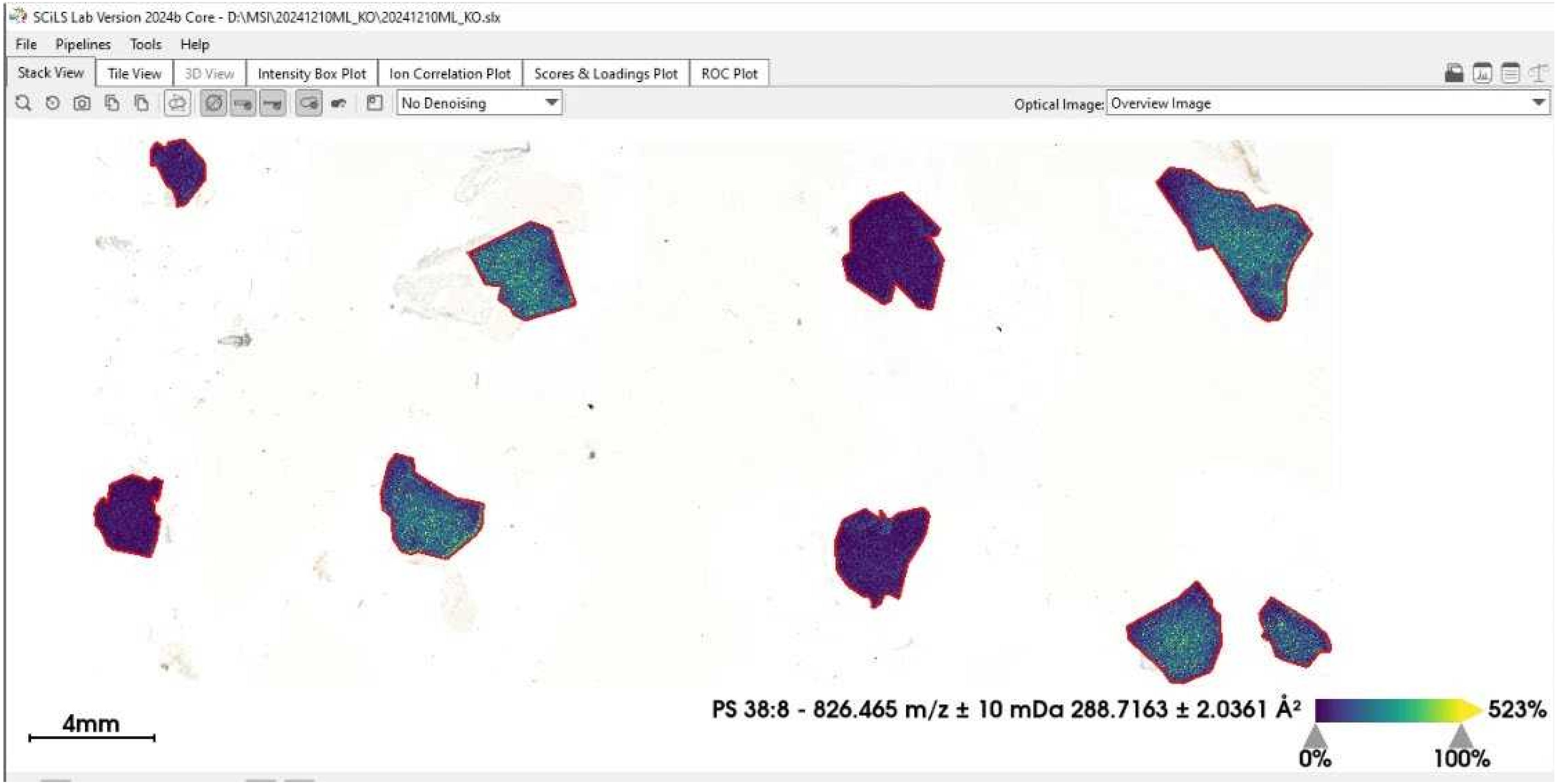

SCiLS Lab Version 2024b Core - D:\MSI\20241210ML_KO\20241210ML_KO.slx
File Pipelines Tools Help
Stack View
Tile View
3D View
Intensity Box Plot
Ion Correlation Plot
Scores & Loadings Plot
ROC Plot
No Denoising
Optical Image: Overview Image
4mm
PS 38:8 - 826.465 m/z ± 10 mDa 288.7163 ± 2.0361 Å²
523%
0%
100%

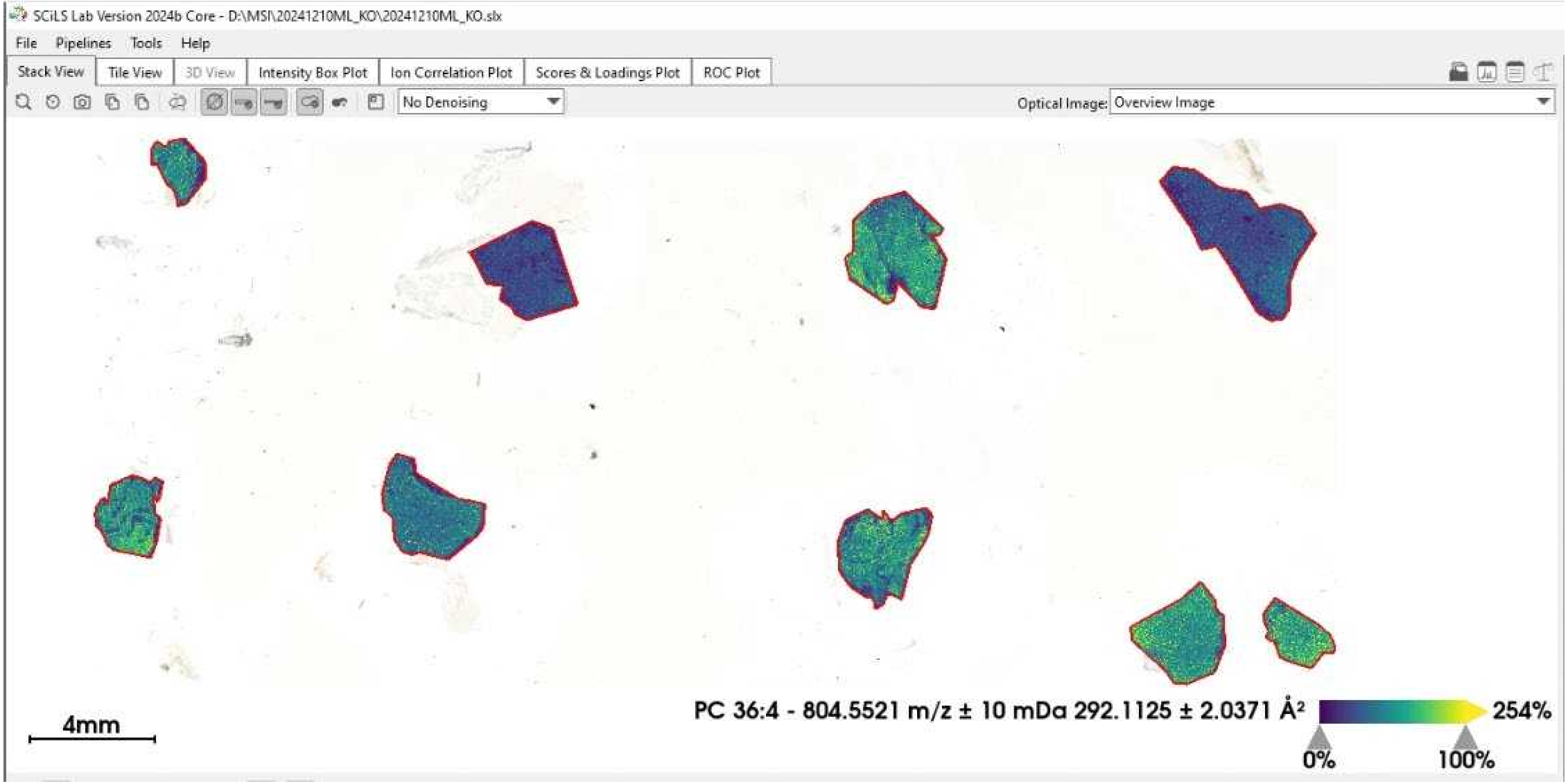
SCiLS Lab Version 2024b Core - D:\MSI\20241210ML_KO\20241210ML_KO.slx
File
Pipelines
Tools
Help
Stack View
Tile View
3D View
Intensity Box Plot
Ion Correlation Plot
Scores & Loadings Plot
ROC Plot
No Denoising
Optical Image:
Overview Image
4mm
PC 36:4 - 804.5521 m/z ± 10 mDa 292.1125 ± 2.0371 Å²
254%
0%
100%

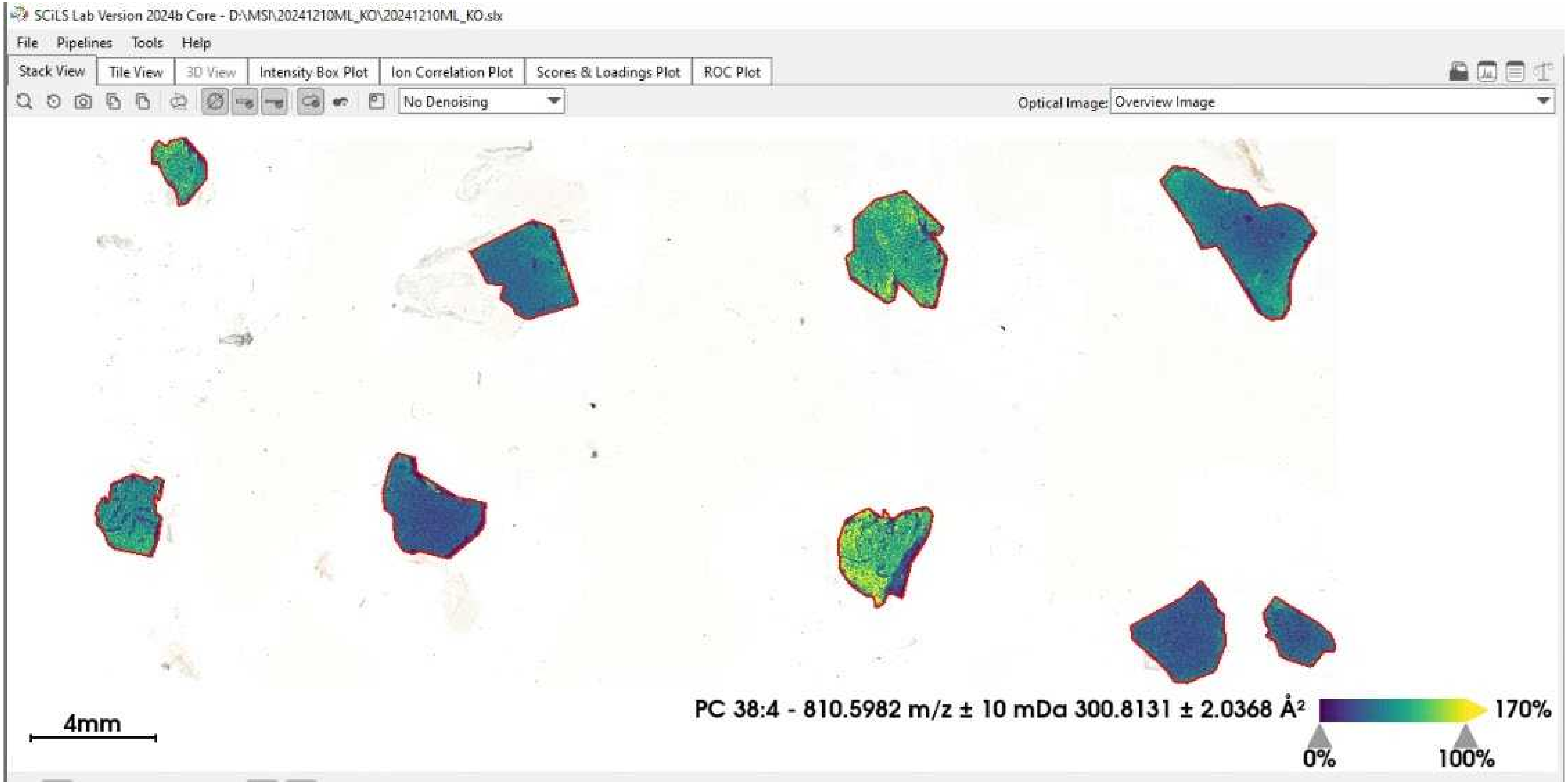
SCiLS Lab Version 2024b Core - D:\MSI\20241210ML_KO\20241210ML_KO.slx
File
Pipelines
Tools
Help
Stack View
Tile View
3D View
Intensity Box Plot
Ion Correlation Plot
Scores & Loadings Plot
ROC Plot
No Denoising
Optical Image:
Overview Image
4mm
PC 38:4 - 810.5982 m/z ± 10 mDa 300.8131 ± 2.0368 Å²
170%
0%
100%

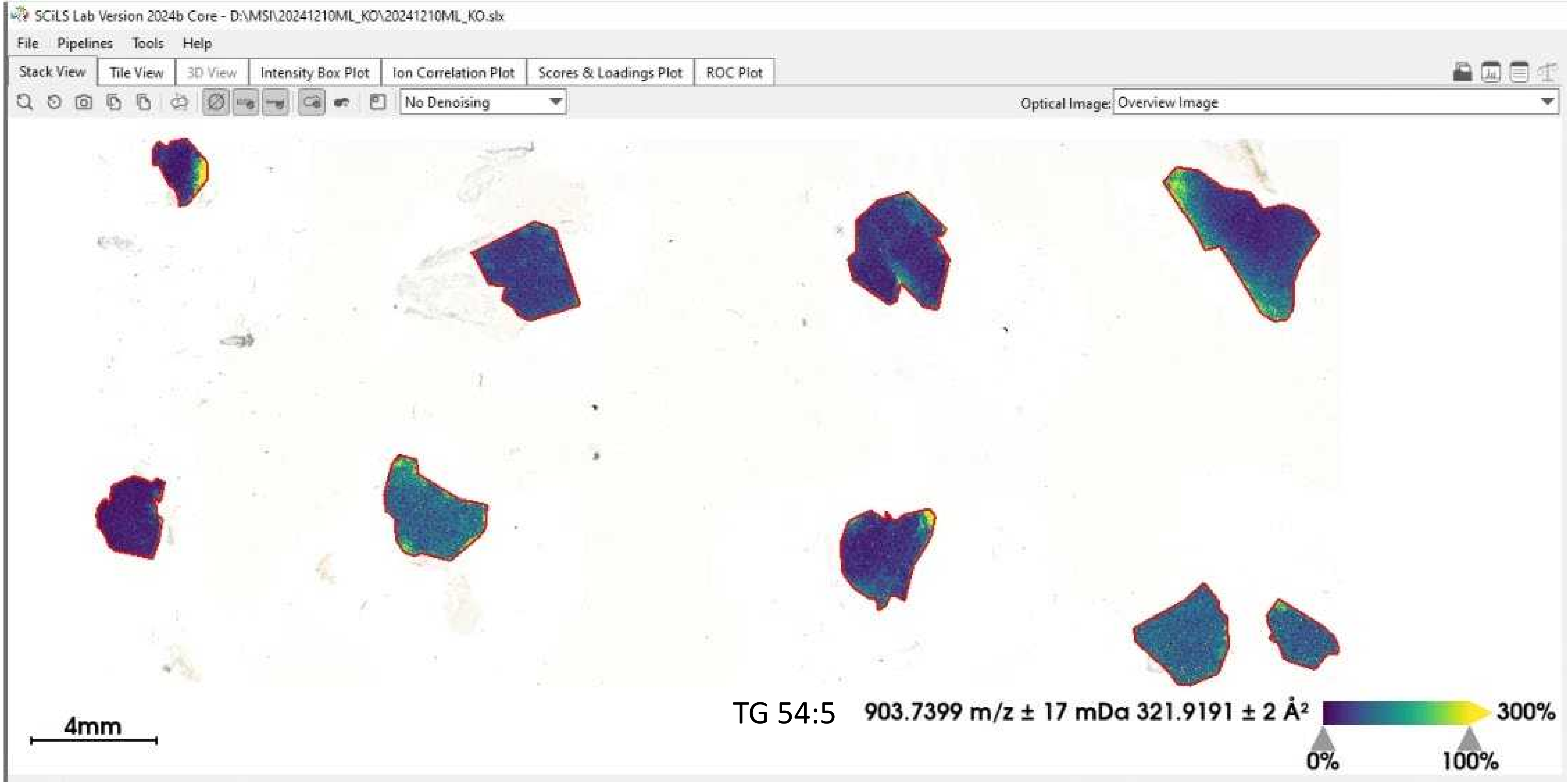
SCiLS Lab Version 2024b Core - D:\MSI\20241210ML_KO\20241210ML_KO.slx
File Pipelines Tools Help
Stack View
Tile View
3D View
Intensity Box Plot
Ion Correlation Plot
Scores & Loadings Plot
ROC Plot
No Denoising
Optical Image: Overview Image
4mm
TG 54:5
903.7399 m/z ± 17 mDa 321.9191 ± 2 Å²
300%
0%
100%

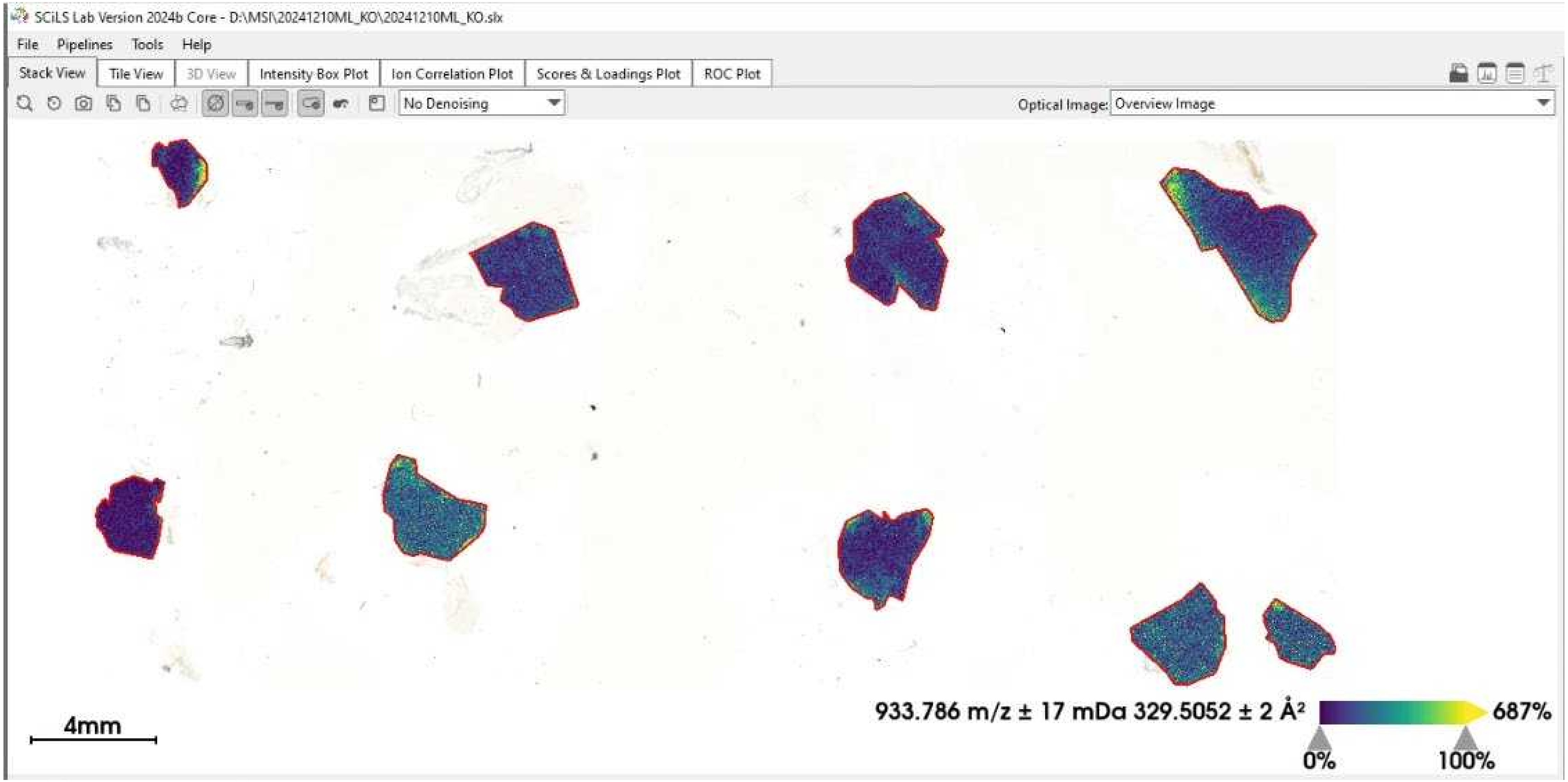
SCiLS Lab Version 2024b Core - D:\MSI\20241210ML_KO\20241210ML_KO.slx
File
Pipelines
Tools
Help
Stack View
Tile View
3D View
Intensity Box Plot
Ion Correlation Plot
Scores & Loadings Plot
ROC Plot
No Denoising
Optical Image:
Overview Image
4mm
933.786 m/z ± 17 mDa 329.5052 ± 2 Å²
687%
0%
100%

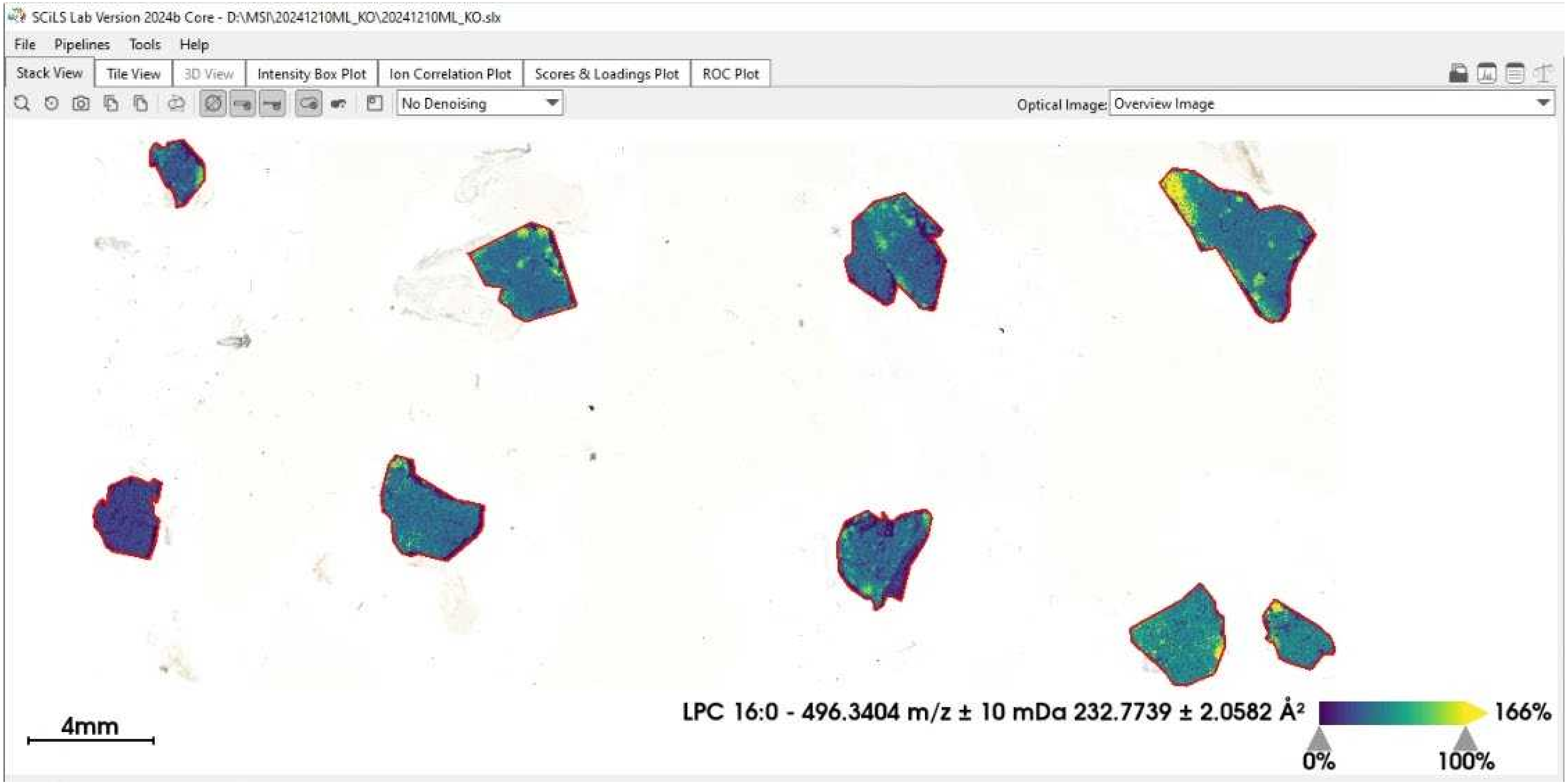

SCiLS Lab Version 2024b Core - D:\MSI\20241210ML_KO\20241210ML_KO.slx
File
Pipelines
Tools
Help
Stack View
Tile View
3D View
Intensity Box Plot
Ion Correlation Plot
Scores & Loadings Plot
ROC Plot
No Denoising
Optical Image:
Overview Image
4mm
LPC 16:0 - 496.3404 m/z ± 10 mDa 232.7739 ± 2.0582 Å²
166%
0%
100%

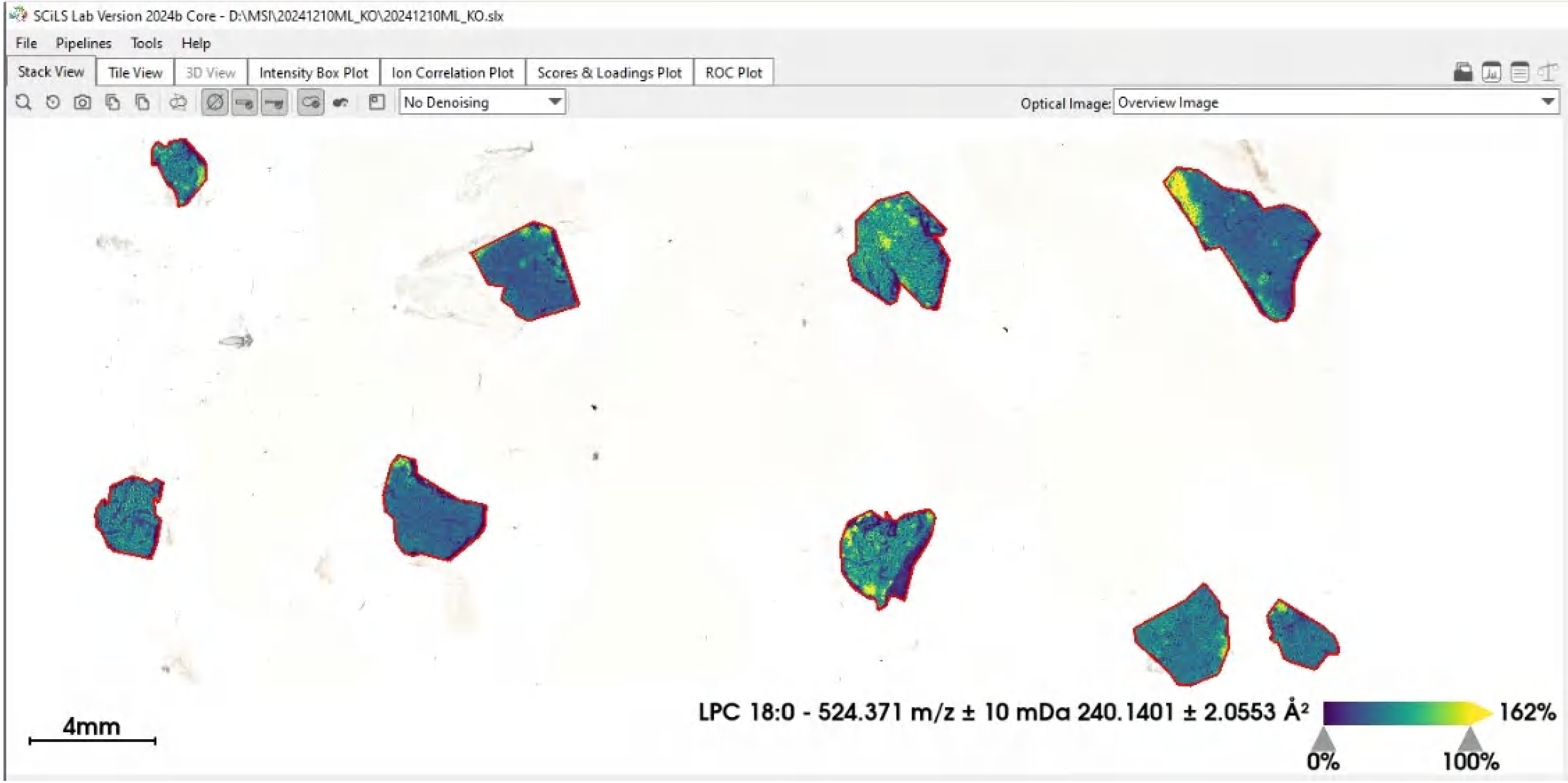

SCiLS Lab Version 2024b Core - D:\MSI\20241210ML_KO\20241210ML_KO.slx
File
Pipelines
Tools
Help
Stack View
Tile View
3D View
Intensity Box Plot
Ion Correlation Plot
Scores & Loadings Plot
ROC Plot
No Denoising
Optical Image:
Overview Image
4mm
LPC 18:0 - 524.371 m/z ± 10 mDa 240.1401 ± 2.0553 Å²
162%
0%
100%

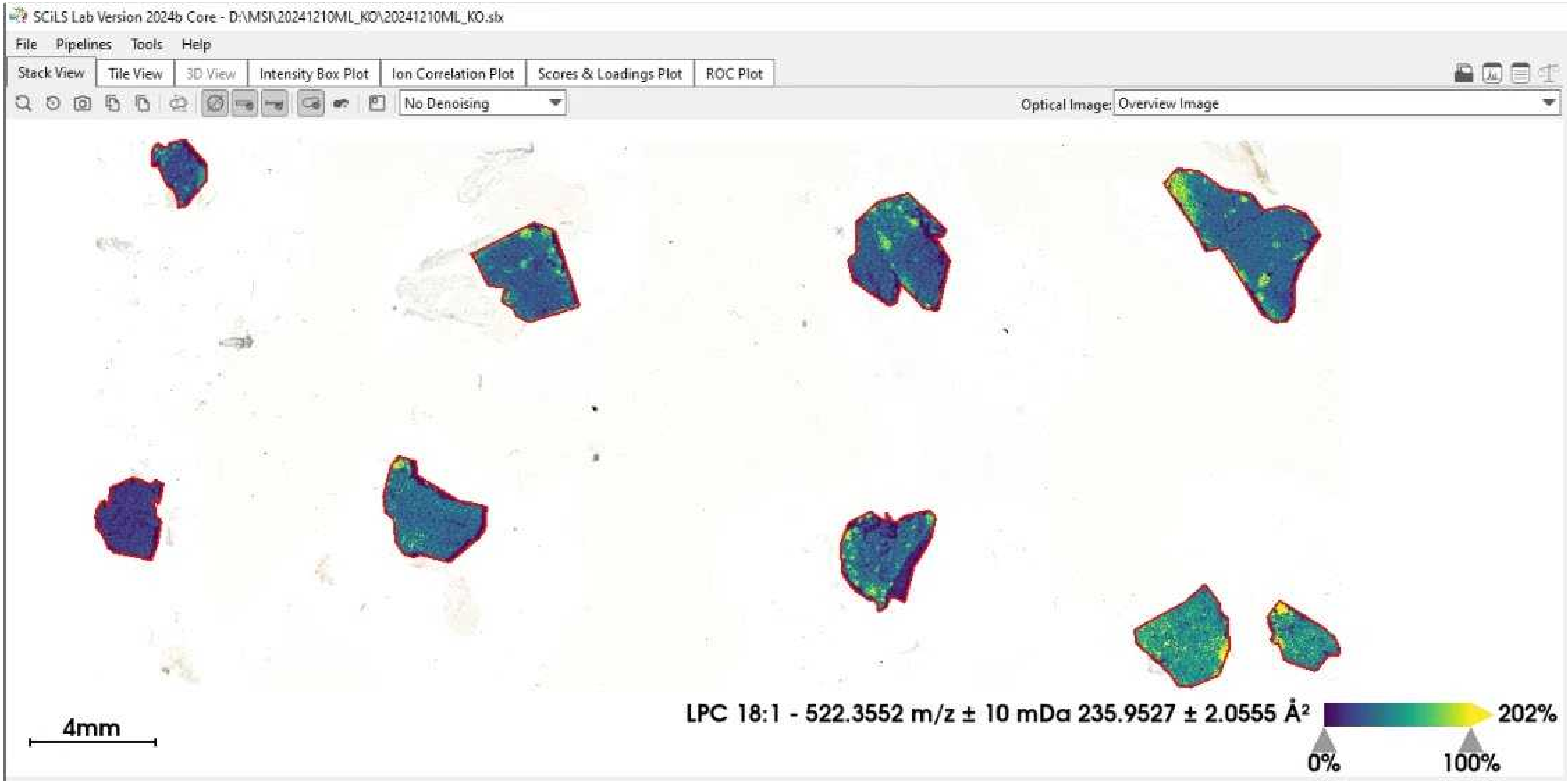
SCiLS Lab Version 2024b Core - D:\MSI\20241210ML_KO\20241210ML_KO.slx
File
Pipelines
Tools
Help
Stack View
Tile View
3D View
Intensity Box Plot
Ion Correlation Plot
Scores & Loadings Plot
ROC Plot
No Denoising
Optical Image:
Overview Image
4mm
LPC 18:1 - 522.3552 m/z ± 10 mDa 235.9527 ± 2.0555 Å²
202%
0%
100%

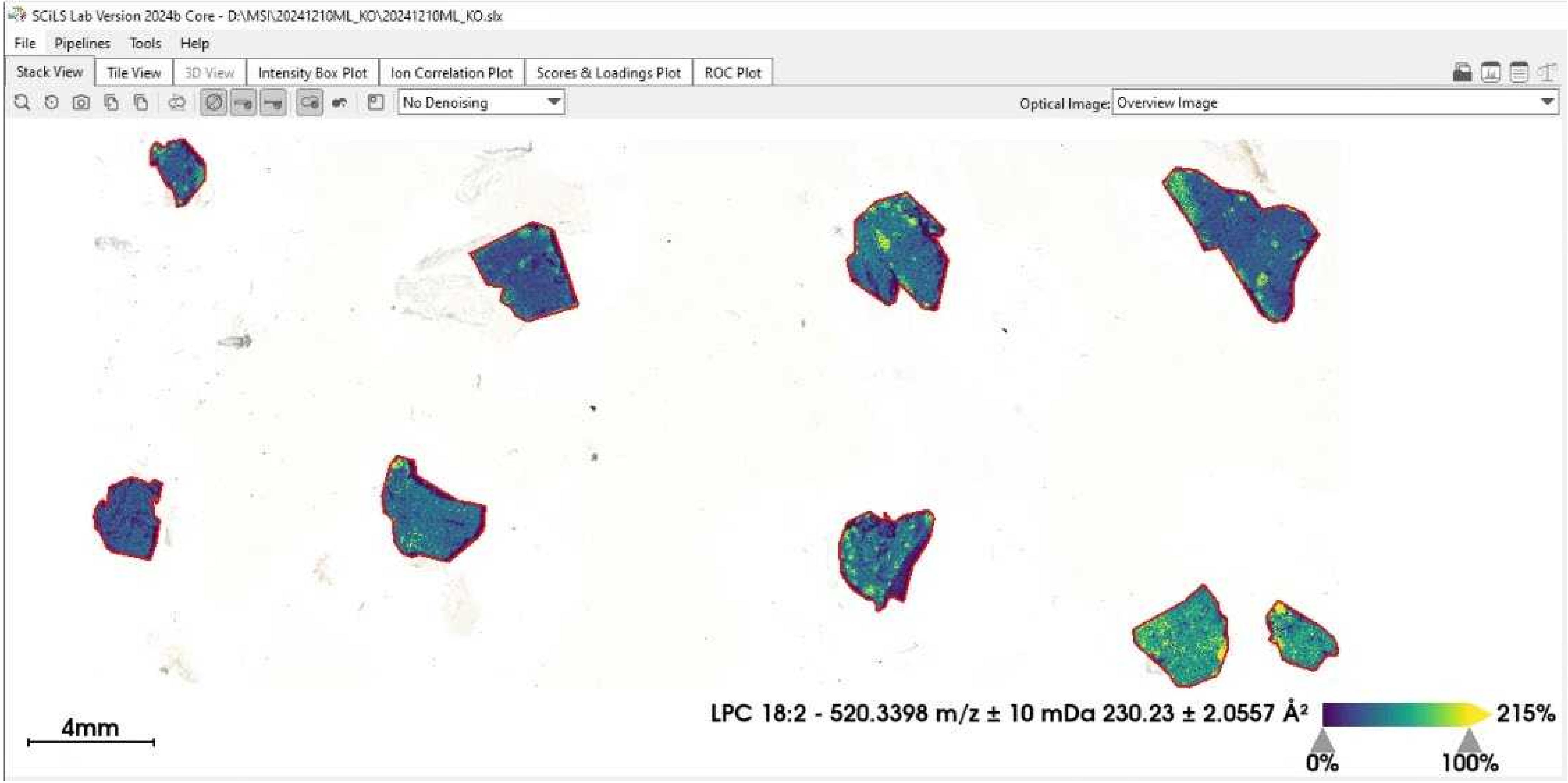
SCiLS Lab Version 2024b Core - D:\MSI\20241210ML_KO\20241210ML_KO.slx
File
Pipelines
Tools
Help
Stack View
Tile View
3D View
Intensity Box Plot
Ion Correlation Plot
Scores & Loadings Plot
ROC Plot
No Denoising
Optical Image:
Overview Image
4mm
LPC 18:2 - 520.3398 m/z ± 10 mDa 230.23 ± 2.0557 Å²
215%
0%
100%

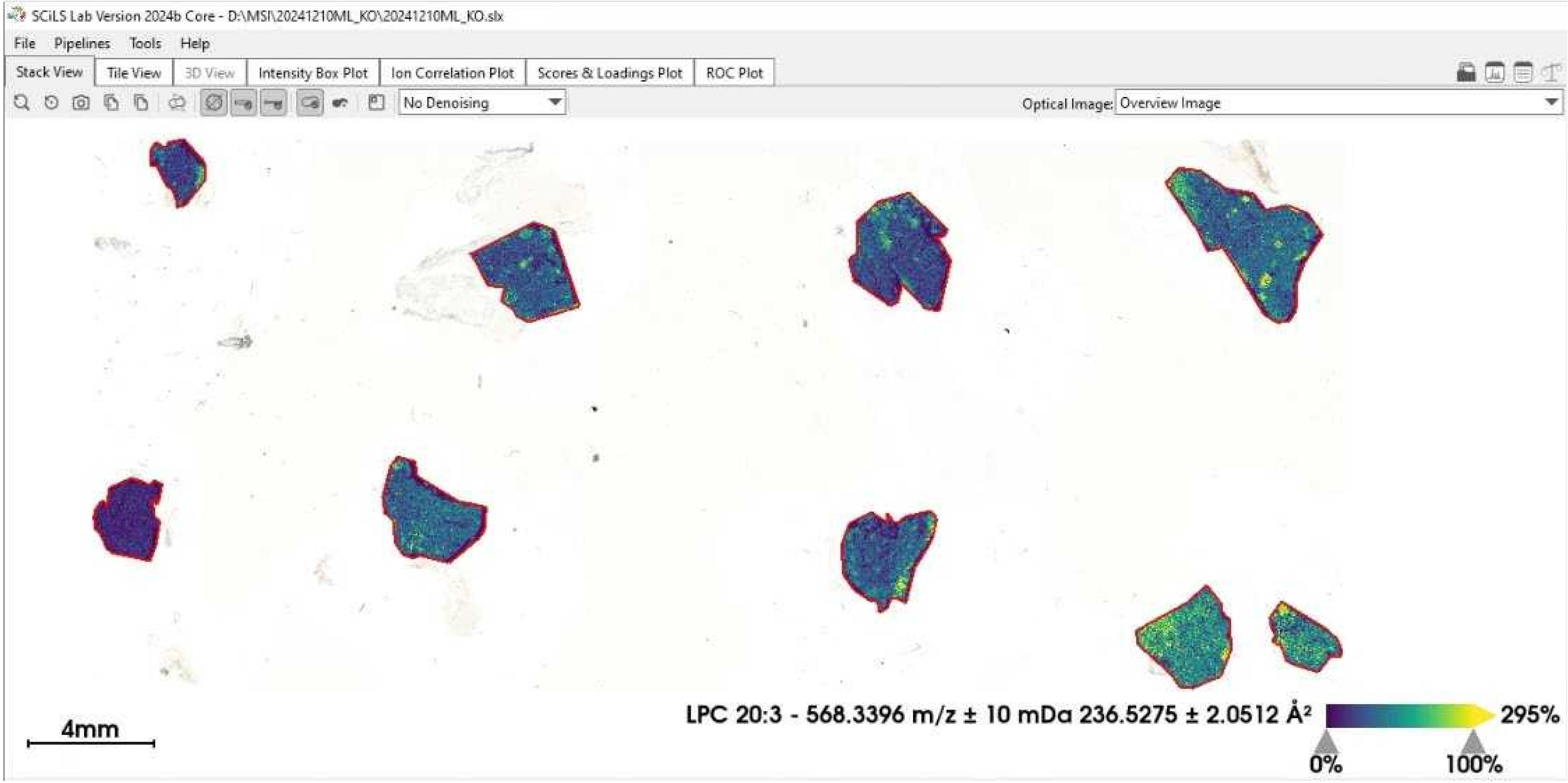
SCiLS Lab Version 2024b Core - D:\MSI\20241210ML_KO\20241210ML_KO.slx
File
Pipelines
Tools
Help
Stack View
Tile View
3D View
Intensity Box Plot
Ion Correlation Plot
Scores & Loadings Plot
ROC Plot
No Denoising
Optical Image:
Overview Image
4mm
LPC 20:3 - 568.3396 m/z ± 10 mDa 236.5275 ± 2.0512 Å²
295%
0%
100%

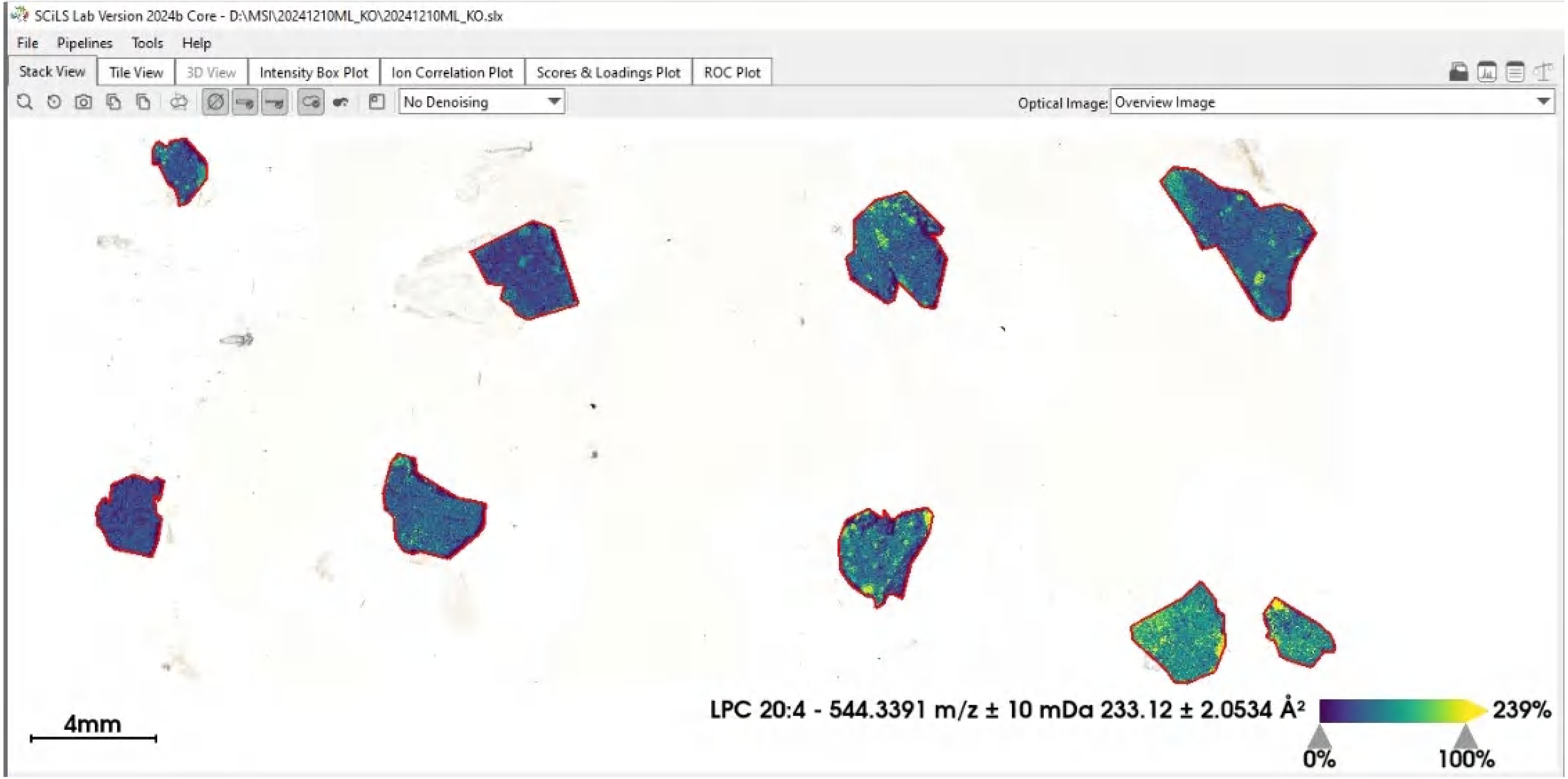

SCiLS Lab Version 2024b Core - D:\MSI\20241210ML_KO\20241210ML_KO.slx
File Pipelines Tools Help
Stack View
Tile View
3D View
Intensity Box Plot
Ion Correlation Plot
Scores & Loadings Plot
ROC Plot
No Denoising
Optical Image: Overview Image
4mm
LPC 20:4 - 544.3391 m/z ± 10 mDa 233.12 ± 2.0534 Å²
239%
0%
100%

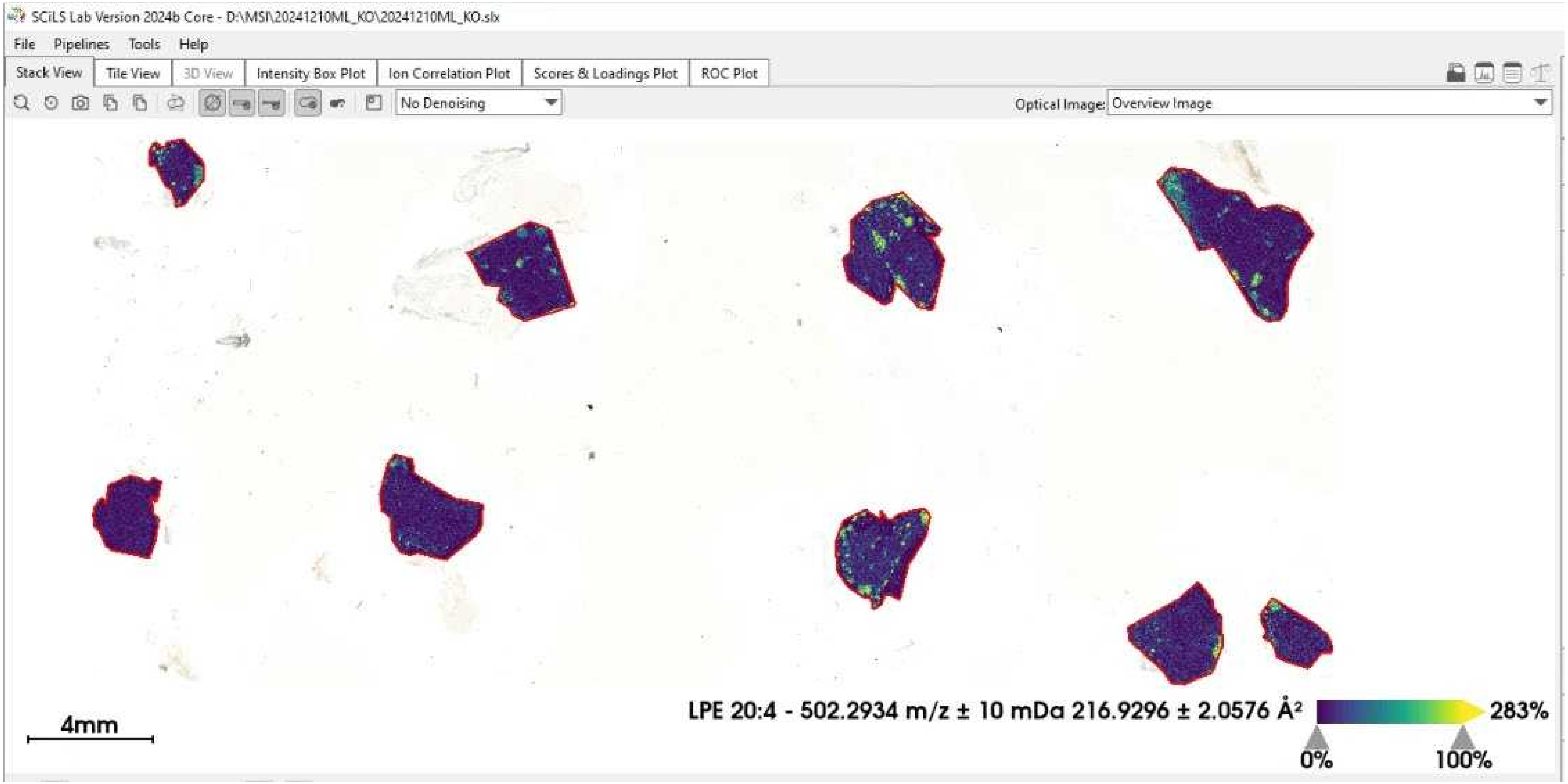
SCiLS Lab Version 2024b Core - D:\MSI\20241210ML_KO\20241210ML_KO.slx
File Pipelines Tools Help
Stack View
Tile View
3D View
Intensity Box Plot
Ion Correlation Plot
Scores & Loadings Plot
ROC Plot
No Denoising
Optical Image: Overview Image
4mm
LPE 20:4 - 502.2934 m/z ± 10 mDa 216.9296 ± 2.0576 Å²
283%
0%
100%

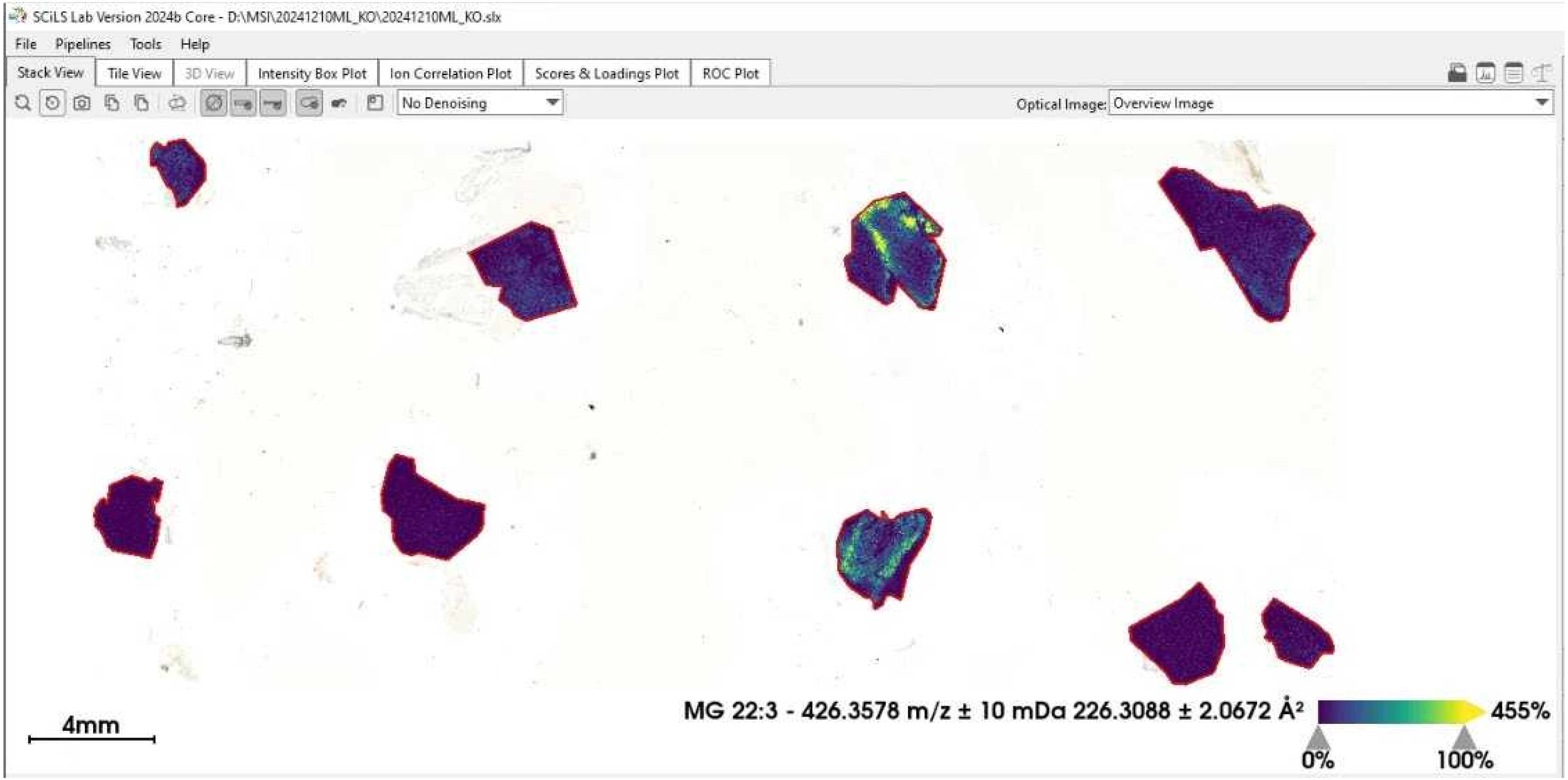
SCiLS Lab Version 2024b Core - D:\MSI\20241210ML_KO\20241210ML_KO.slx
File
Pipelines
Tools
Help
Stack View
Tile View
3D View
Intensity Box Plot
Ion Correlation Plot
Scores & Loadings Plot
ROC Plot
No Denoising
Optical Image:
Overview Image
4mm
MG 22:3 - 426.3578 m/z ± 10 mDa 226.3088 ± 2.0672 Å²
455%
0%
100%

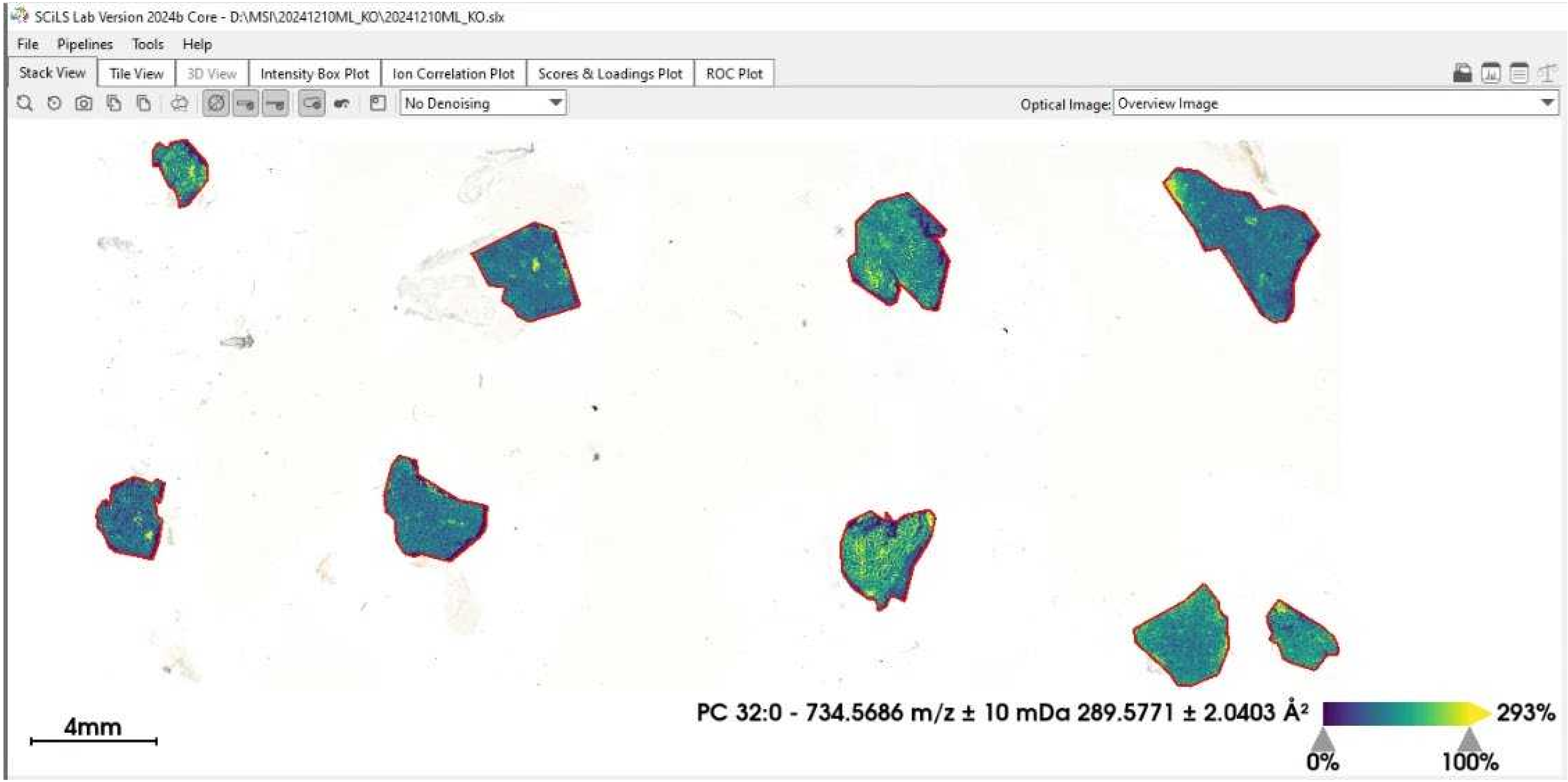
SCiLS Lab Version 2024b Core - D:\MSI\20241210ML_KO\20241210ML_KO.slx
File
Pipelines
Tools
Help
Stack View
Tile View
3D View
Intensity Box Plot
Ion Correlation Plot
Scores & Loadings Plot
ROC Plot
No Denoising
Optical Image:
Overview Image
4mm
PC 32:0 - 734.5686 m/z ± 10 mDa 289.5771 ± 2.0403 Å²
293%
0%
100%

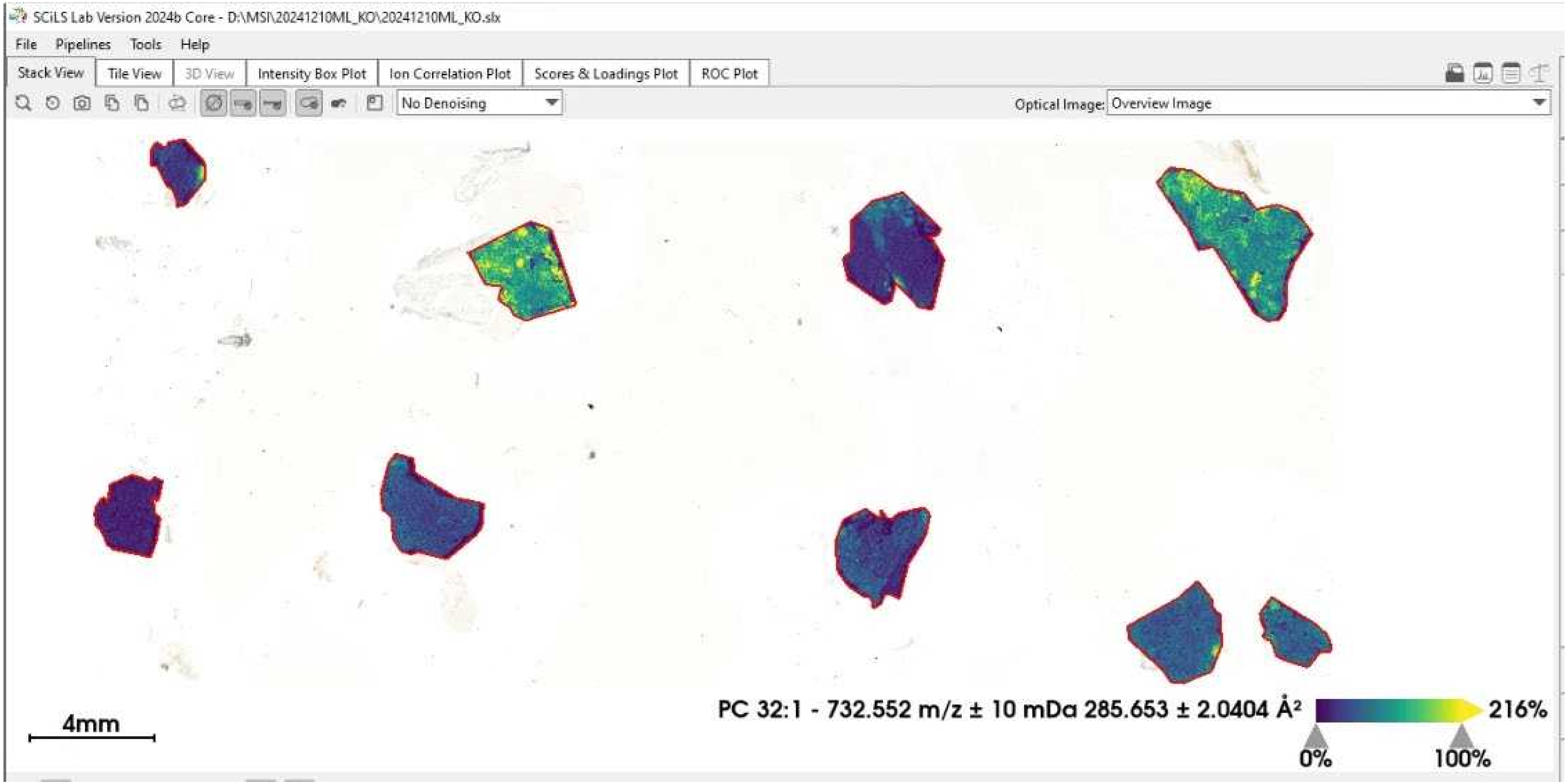
SCiLS Lab Version 2024b Core - D:\MSI\20241210ML_KO\20241210ML_KO.slx
File
Pipelines
Tools
Help
Stack View
Tile View
3D View
Intensity Box Plot
Ion Correlation Plot
Scores & Loadings Plot
ROC Plot
No Denoising
Optical Image:
Overview Image
4mm
PC 32:1 - 732.552 m/z ± 10 mDa 285.653 ± 2.0404 Å²
216%
0%
100%

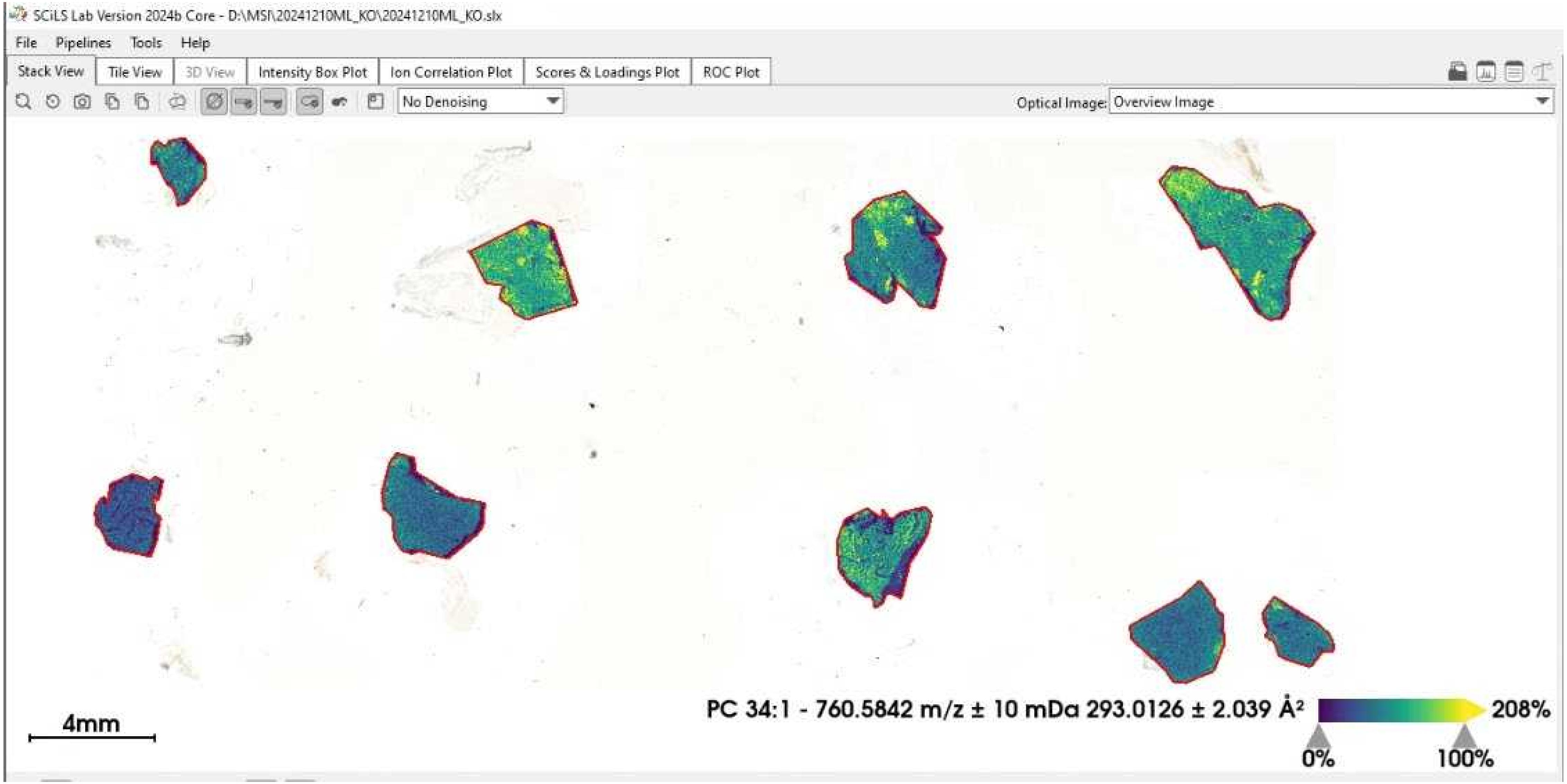
SCiLS Lab Version 2024b Core - D:\MSI\20241210ML_KO\20241210ML_KO.slx
File
Pipelines
Tools
Help
Stack View
Tile View
3D View
Intensity Box Plot
Ion Correlation Plot
Scores & Loadings Plot
ROC Plot
No Denoising
Optical Image:
Overview Image
4mm
PC 34:1 - 760.5842 m/z ± 10 mDa 293.0126 ± 2.039 Å²
208%
0%
100%

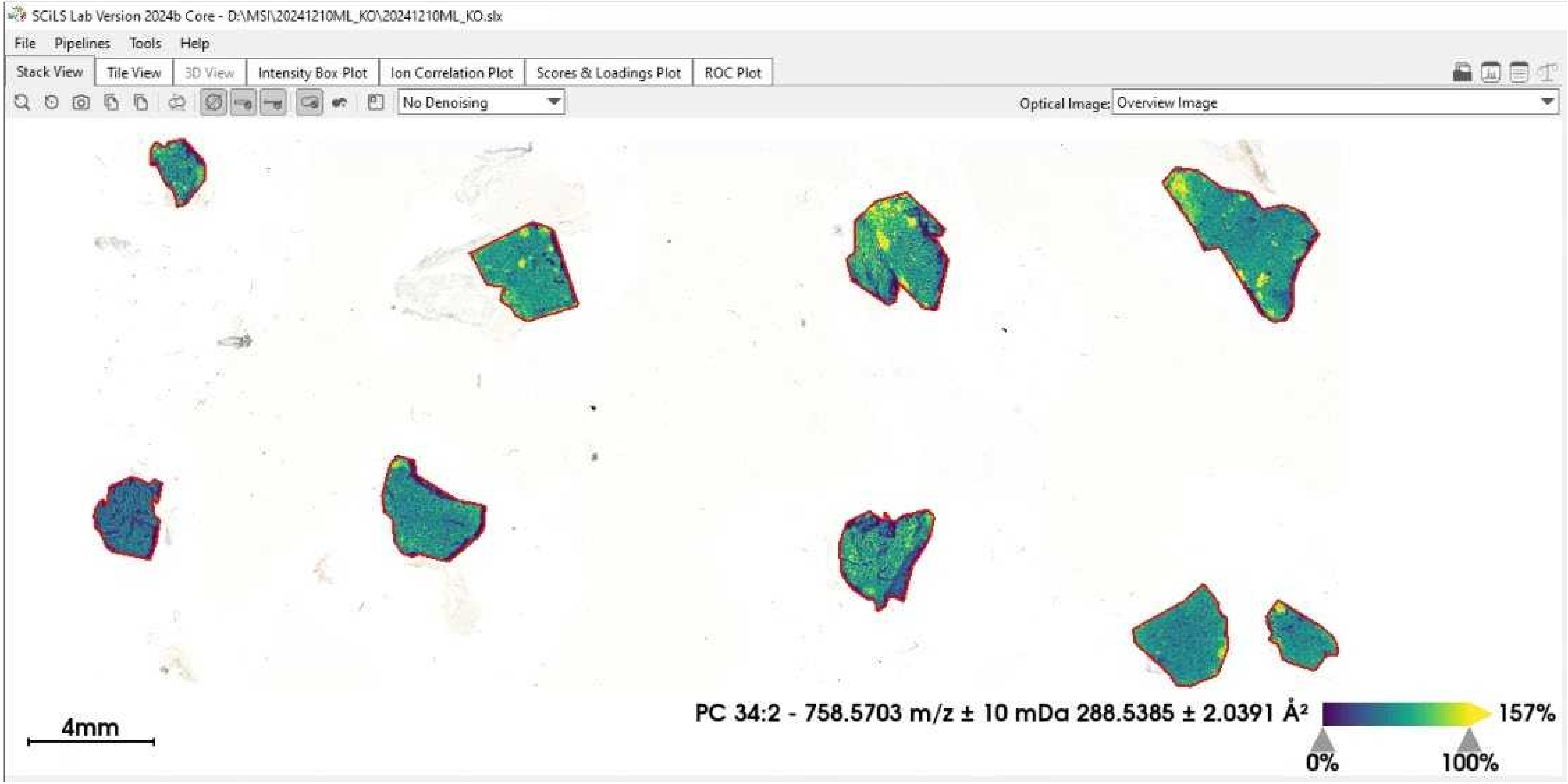
SCiLS Lab Version 2024b Core - D:\MSI\20241210ML_KO\20241210ML_KO.slx
File
Pipelines
Tools
Help
Stack View
Tile View
3D View
Intensity Box Plot
Ion Correlation Plot
Scores & Loadings Plot
ROC Plot
No Denoising
Optical Image:
Overview Image
4mm
PC 34:2 - 758.5703 m/z ± 10 mDa 288.5385 ± 2.0391 Å²
157%
0%
100%

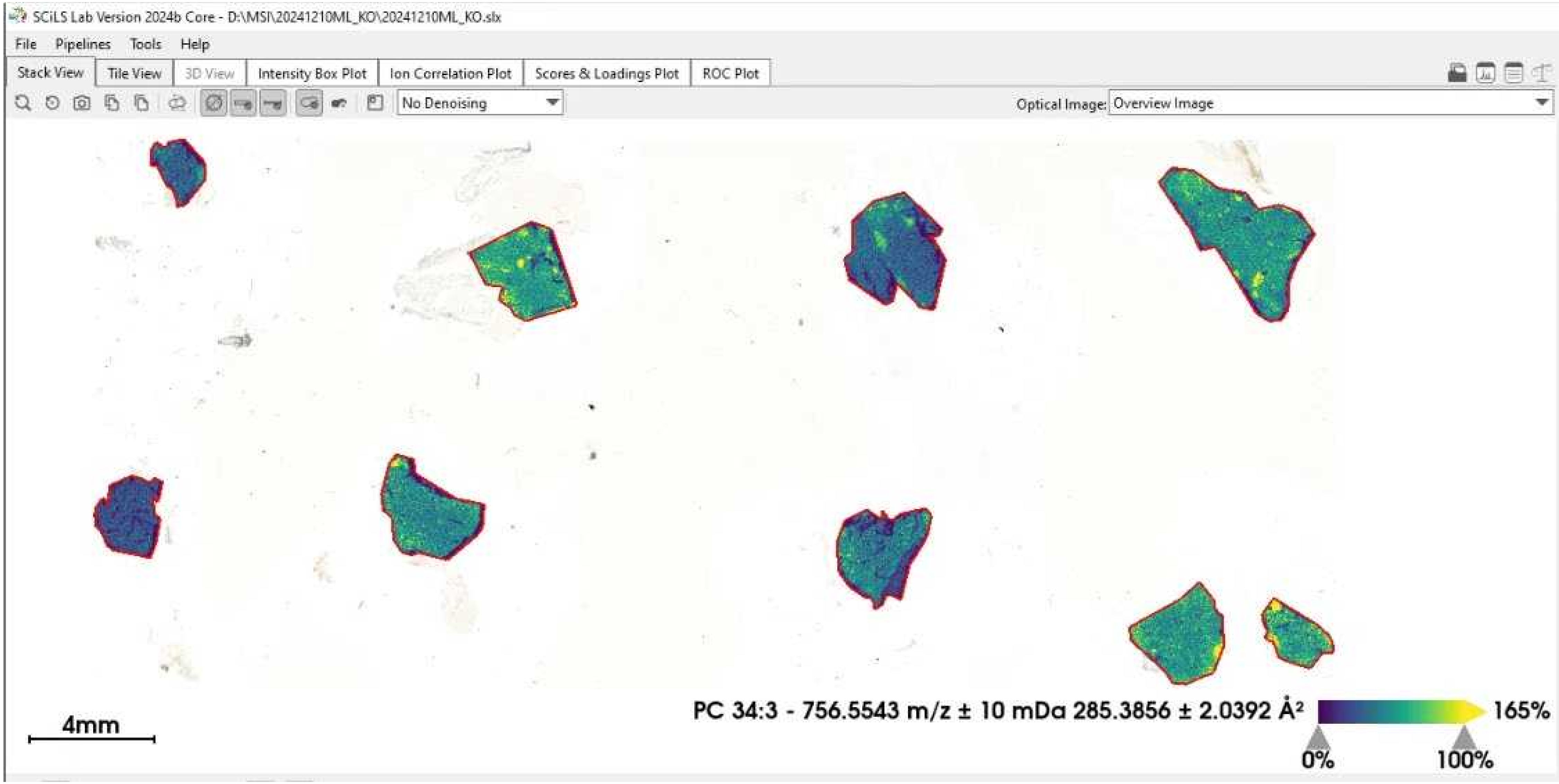
SCiLS Lab Version 2024b Core - D:\MSI\20241210ML_KO\20241210ML_KO.slx
File
Pipelines
Tools
Help
Stack View
Tile View
3D View
Intensity Box Plot
Ion Correlation Plot
Scores & Loadings Plot
ROC Plot
No Denoising
Optical Image:
Overview Image
4mm
PC 34:3 - 756.5543 m/z ± 10 mDa 285.3856 ± 2.0392 Å²
165%
0%
100%

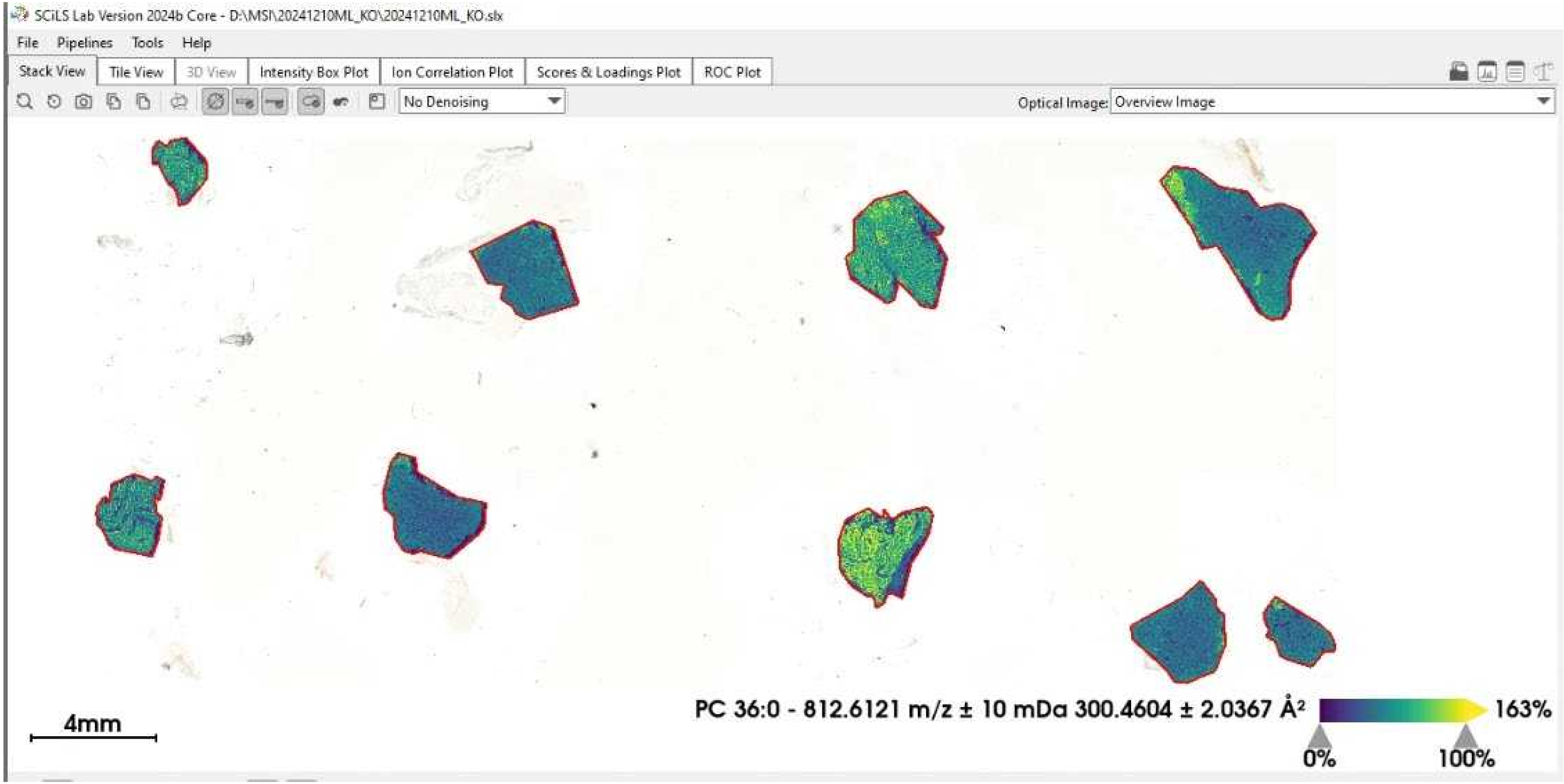

SCiLS Lab Version 2024b Core - D:\MSI\20241210ML_KO\20241210ML_KO.slx
File Pipelines Tools Help
Stack View Tile View 3D View Intensity Box Plot Ion Correlation Plot Scores & Loadings Plot ROC Plot
No Denoising
Optical Image: Overview Image
4mm
PC 36:0 - 812.6121 m/z ± 10 mDa 300.4604 ± 2.0367 Å²
163%
0%
100%

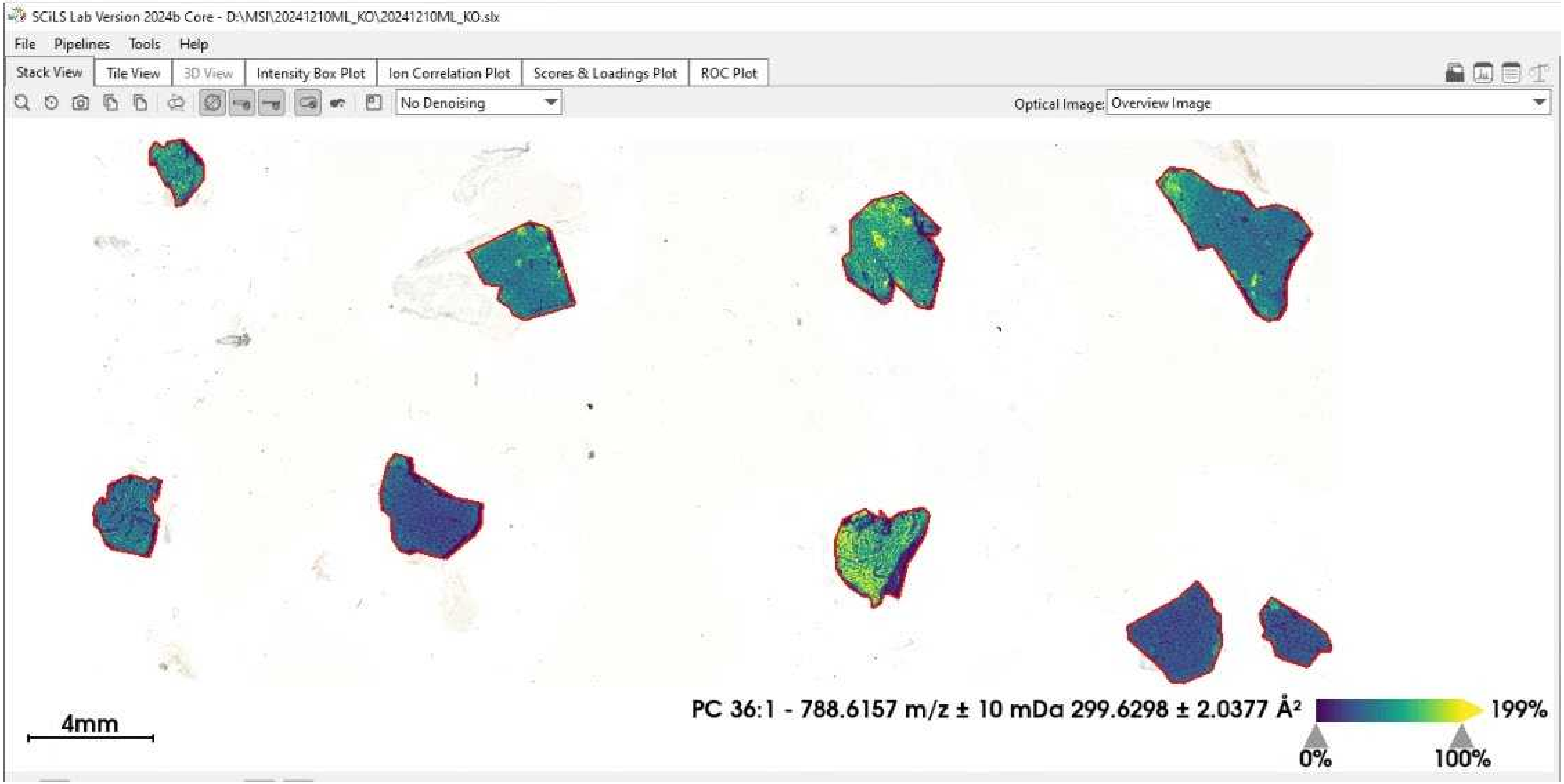
SCiLS Lab Version 2024b Core - D:\MSI\20241210ML_KO\20241210ML_KO.slx
File
Pipelines
Tools
Help
Stack View
Tile View
3D View
Intensity Box Plot
Ion Correlation Plot
Scores & Loadings Plot
ROC Plot
No Denoising
Optical Image:
Overview Image
4mm
PC 36:1 - 788.6157 m/z ± 10 mDa 299.6298 ± 2.0377 Å²
199%
0%
100%

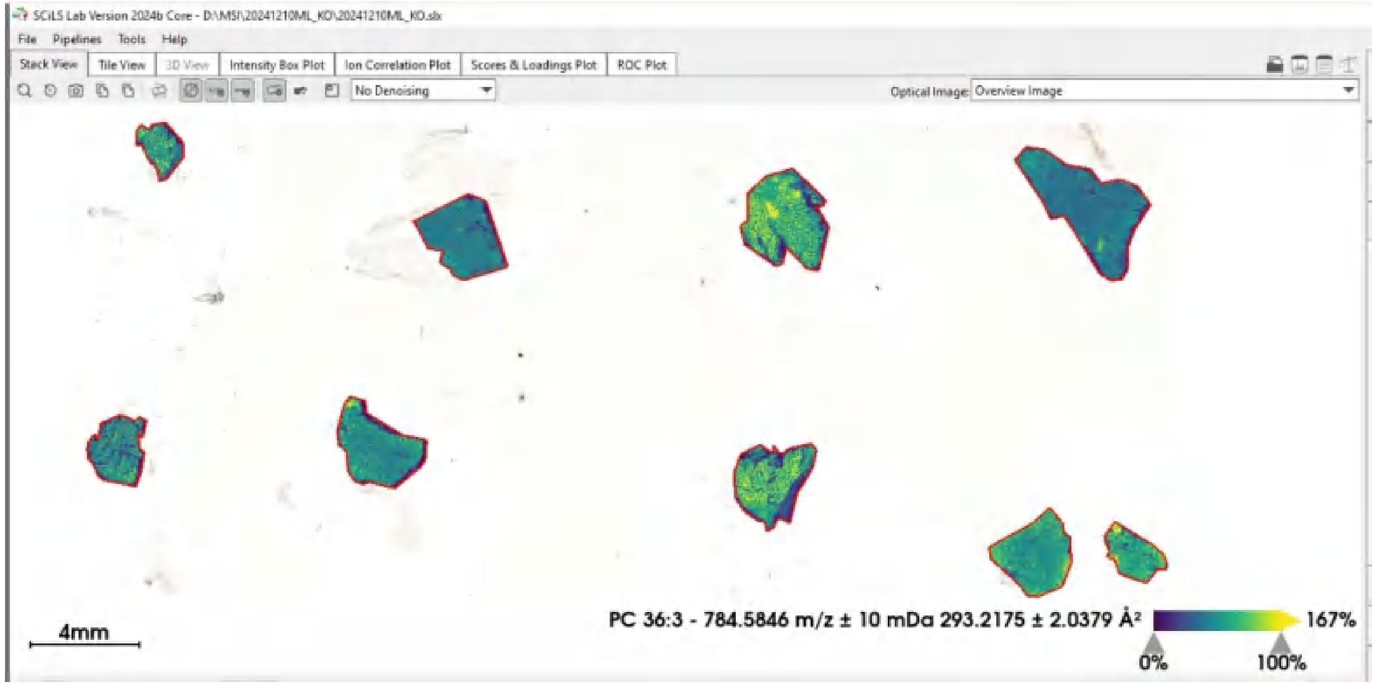

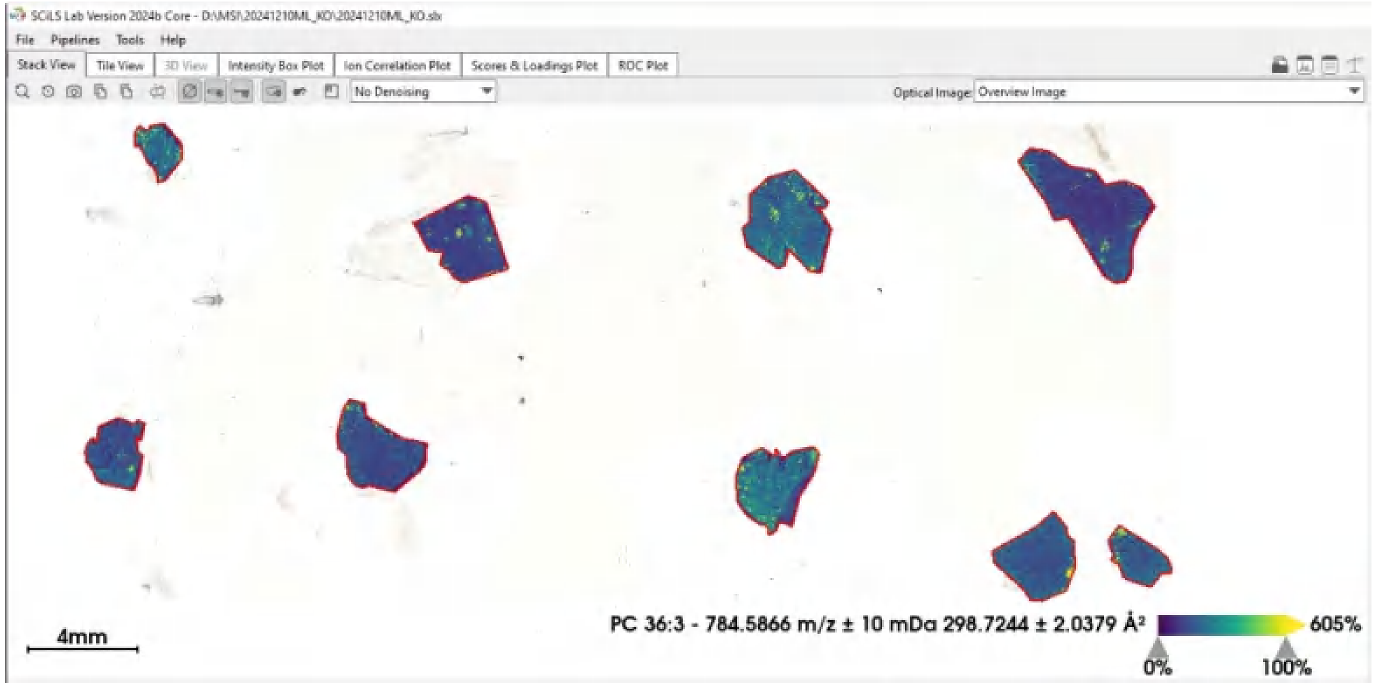

There are 2 features with 2 different CCS for PC 36:3, further tests needed to confirm the real one.

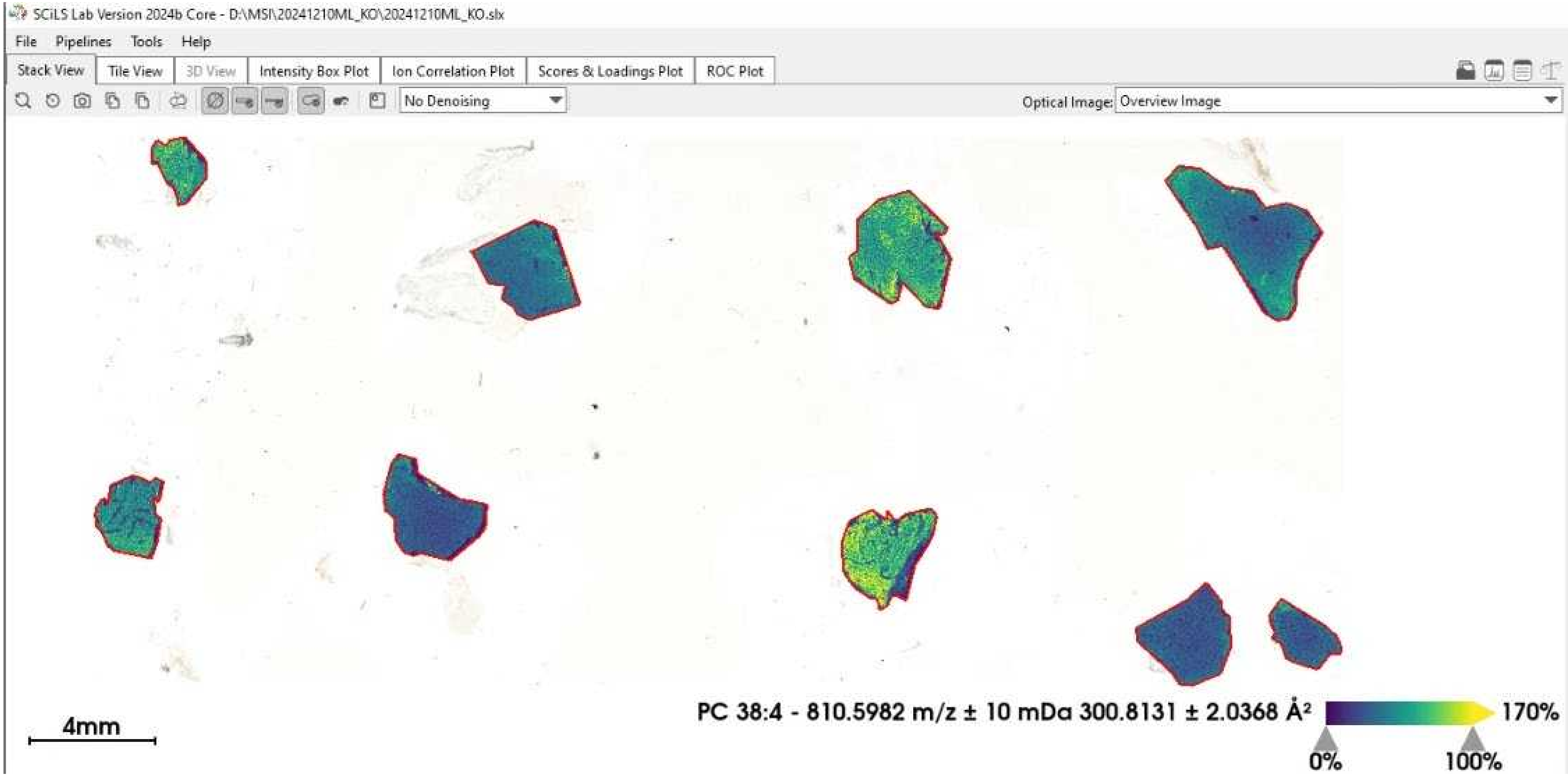
SCiLS Lab Version 2024b Core - D:\MSI\20241210ML_KO\20241210ML_KO.slx
File Pipelines Tools Help
Stack View
Tile View
3D View
Intensity Box Plot
Ion Correlation Plot
Scores & Loadings Plot
ROC Plot
No Denoising
Optical Image: Overview Image
4mm
PC 38:4 - 810.5982 m/z ± 10 mDa 300.8131 ± 2.0368 Å²
170%
0%
100%

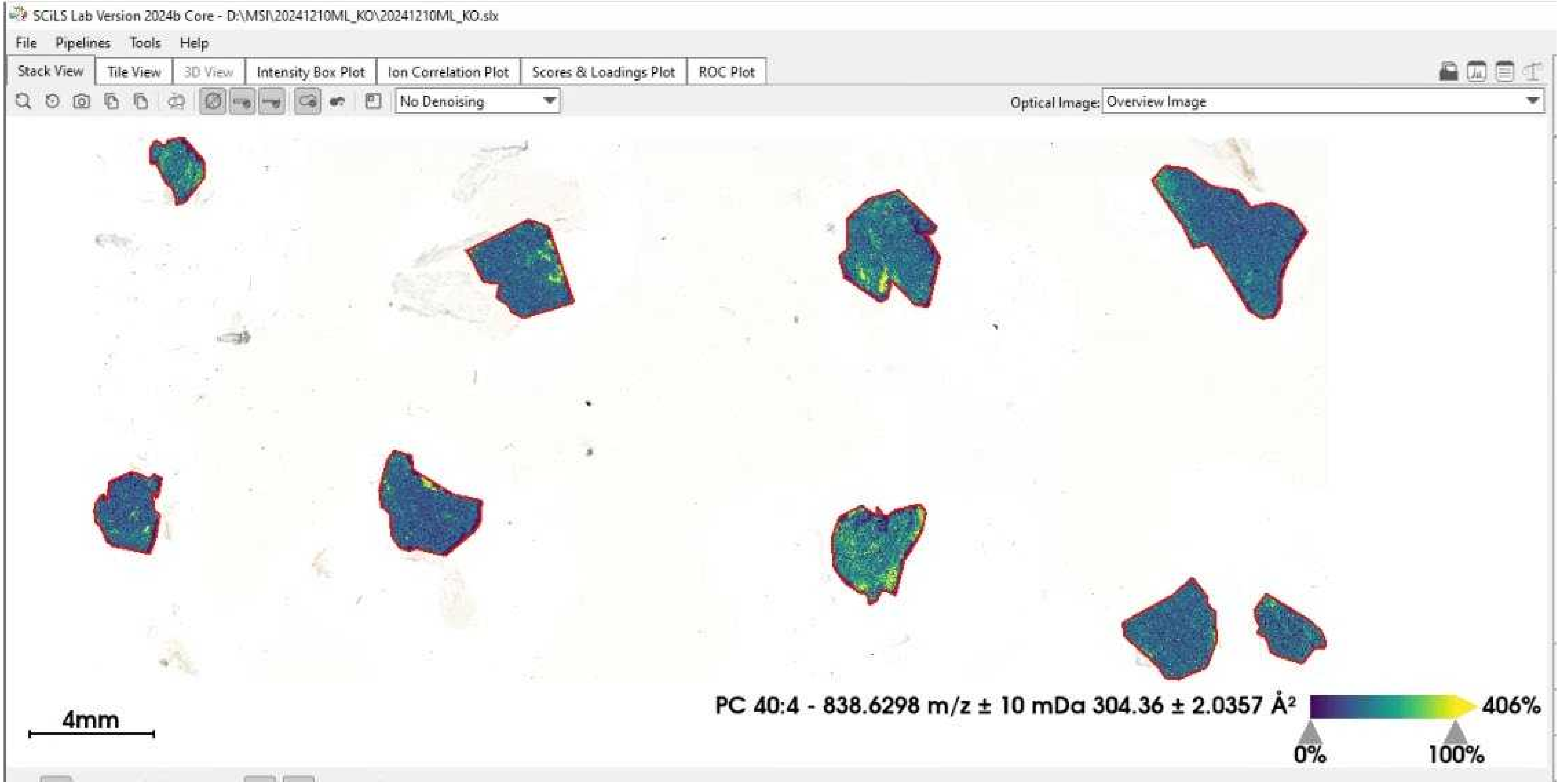
SCiLS Lab Version 2024b Core - D:\MSI\20241210ML_KO\20241210ML_KO.slx
File
Pipelines
Tools
Help
Stack View
Tile View
3D View
Intensity Box Plot
Ion Correlation Plot
Scores & Loadings Plot
ROC Plot
No Denoising
Optical Image:
Overview Image
4mm
PC 40:4 - 838.6298 m/z ± 10 mDa 304.36 ± 2.0357 Å²
406%
0%
100%

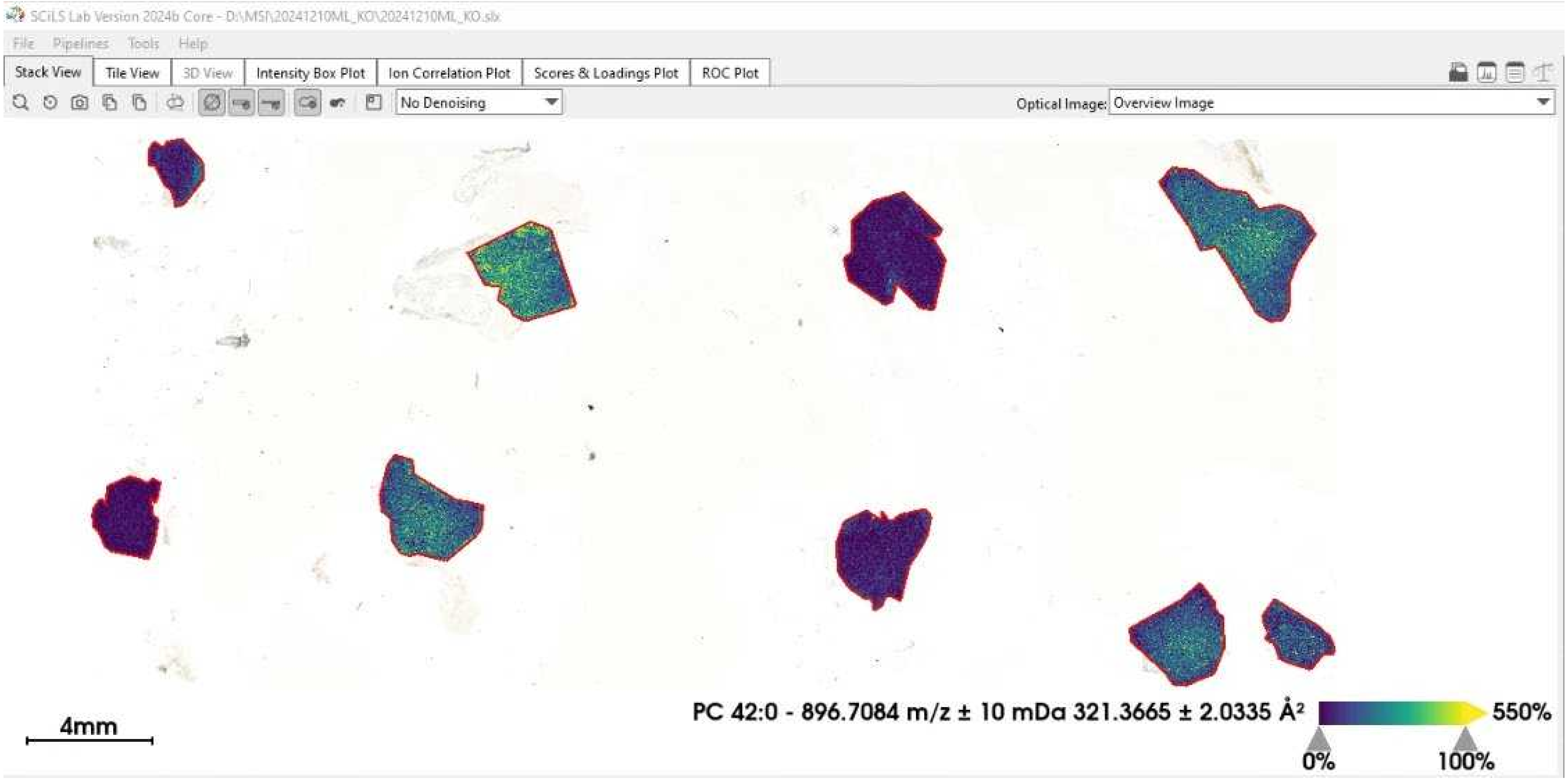
SCiLS Lab Version 2024b Core - D:\MSI\20241210ML_KO\20241210ML_KO.slx
File
Pipelines
Tools
Help
Stack View
Tile View
3D View
Intensity Box Plot
Ion Correlation Plot
Scores & Loadings Plot
ROC Plot
No Denoising
Optical Image:
Overview Image
4mm
PC 42:0 - 896.7084 m/z ± 10 mDa 321.3665 ± 2.0335 Å²
550%
0%
100%

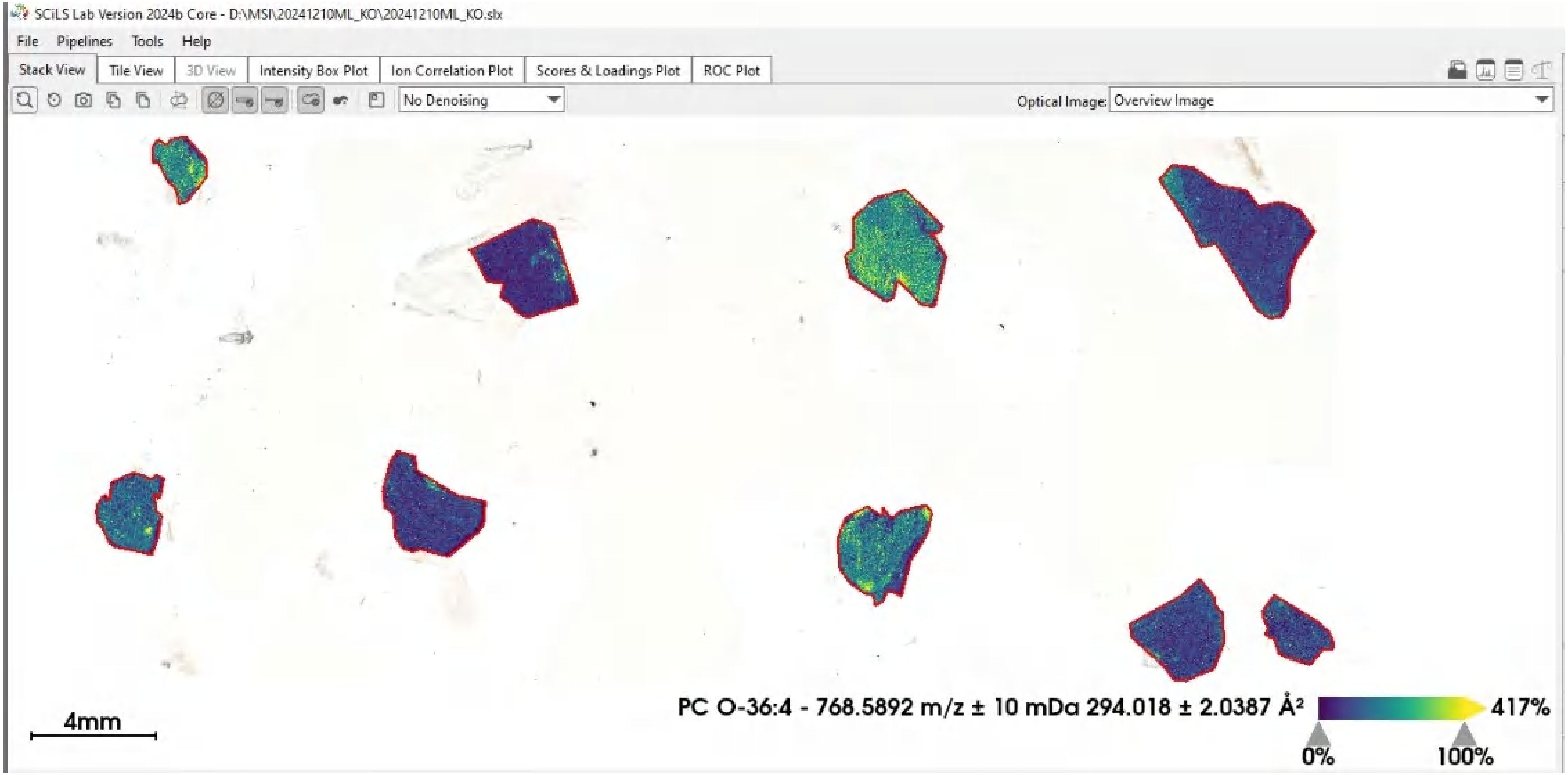
SCiLS Lab Version 2024b Core - D:\MSI\20241210ML_KO\20241210ML_KO.slx
File
Pipelines
Tools
Help
Stack View
Tile View
3D View
Intensity Box Plot
Ion Correlation Plot
Scores & Loadings Plot
ROC Plot
No Denoising
Optical Image:
Overview Image
4mm
PC O-36:4 - 768.5892 m/z ± 10 mDa 294.018 ± 2.0387 Å²
417%
0%
100%

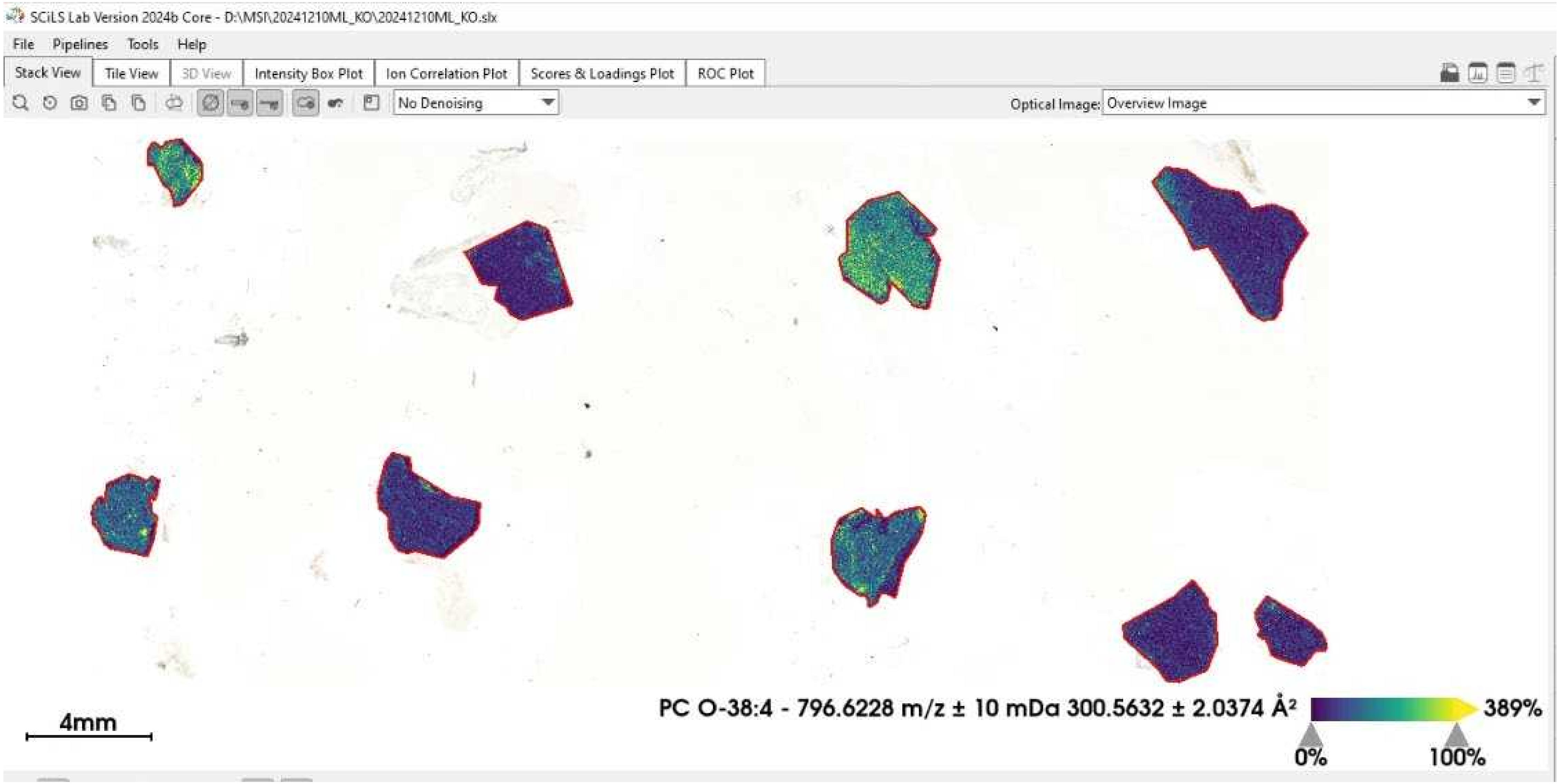

SCiLS Lab Version 2024b Core - D:\MSI\20241210ML_KO\20241210ML_KO.slx
File
Pipelines
Tools
Help
Stack View
Tile View
3D View
Intensity Box Plot
Ion Correlation Plot
Scores & Loadings Plot
ROC Plot
No Denoising
Optical Image:
Overview Image
4mm
PC O-38:4 - 796.6228 m/z ± 10 mDa 300.5632 ± 2.0374 Å²
389%
0%
100%

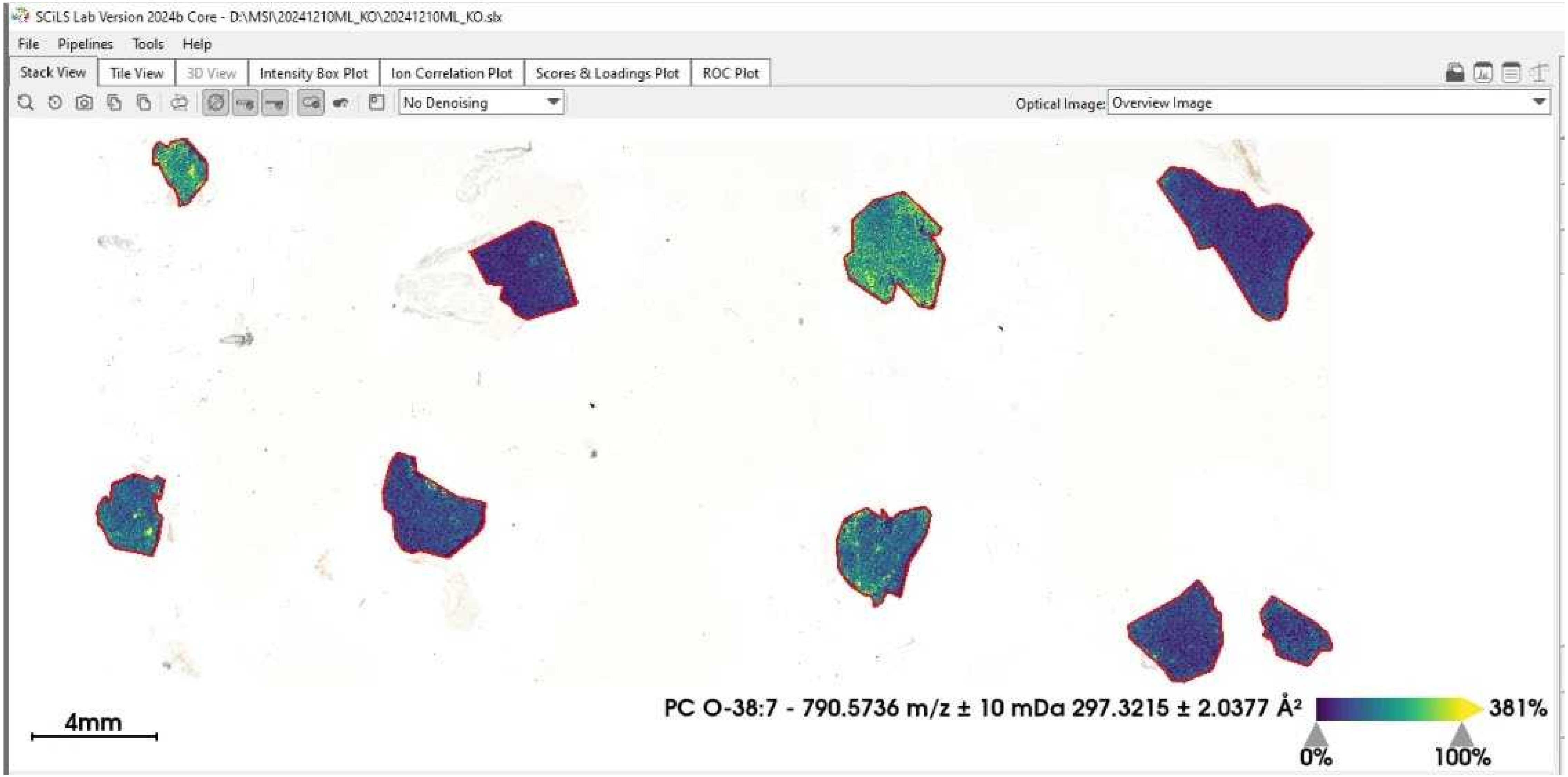
SCiLS Lab Version 2024b Core - D:\MSI\20241210ML_KO\20241210ML_KO.slx
File Pipelines Tools Help
Stack View
Tile View
3D View
Intensity Box Plot
Ion Correlation Plot
Scores & Loadings Plot
ROC Plot
No Denoising
Optical Image: Overview Image
4mm
PC O-38:7 - 790.5736 m/z ± 10 mDa 297.3215 ± 2.0377 Å²
381%
0%
100%

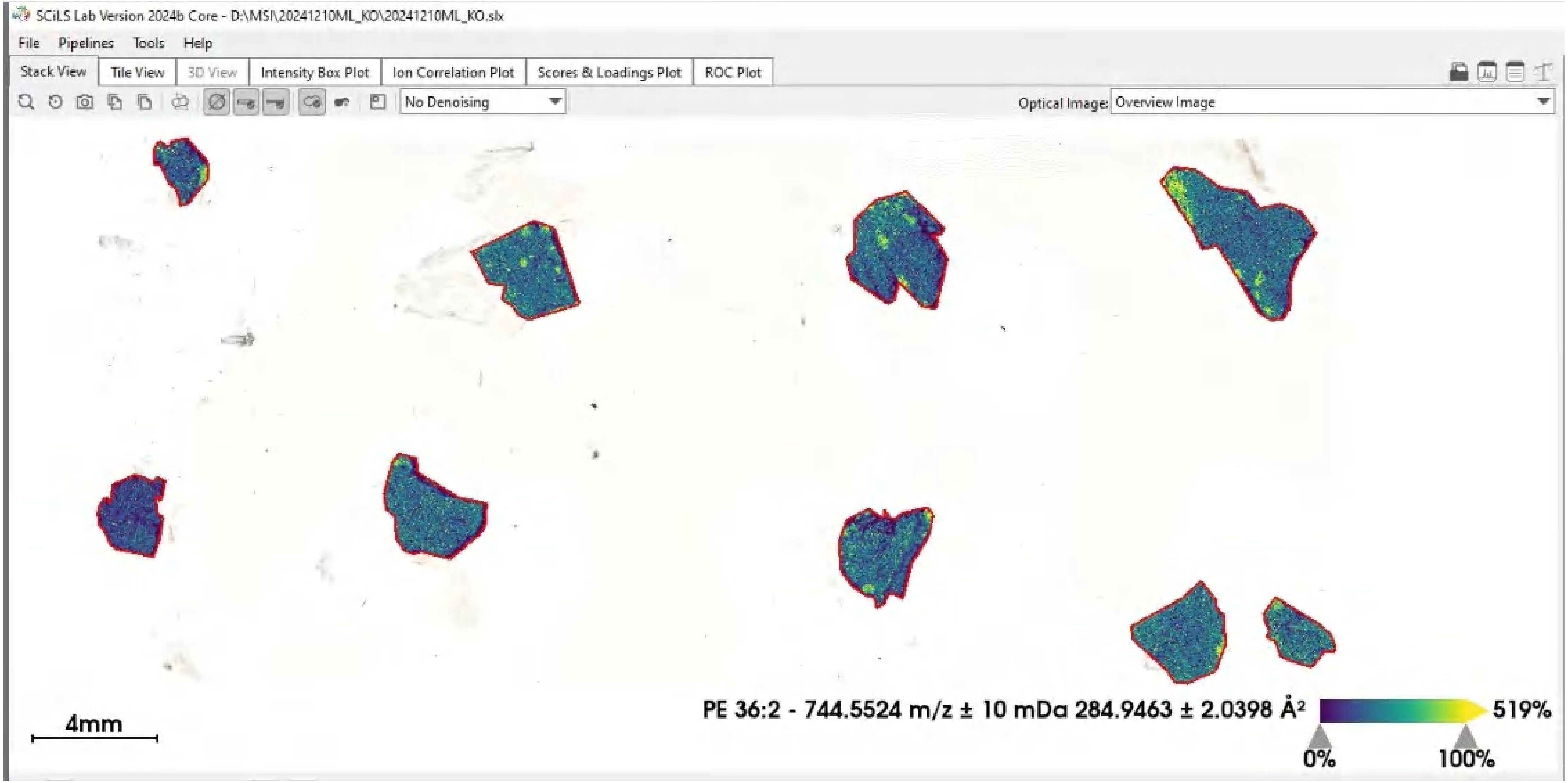
SCiLS Lab Version 2024b Core - D:\MSI\20241210ML_KO\20241210ML_KO.slx
File
Pipelines
Tools
Help
Stack View
Tile View
3D View
Intensity Box Plot
Ion Correlation Plot
Scores & Loadings Plot
ROC Plot
No Denoising
Optical Image:
Overview Image
4mm
PE 36:2 - 744.5524 m/z ± 10 mDa 284.9463 ± 2.0398 Å²
519%
0%
100%

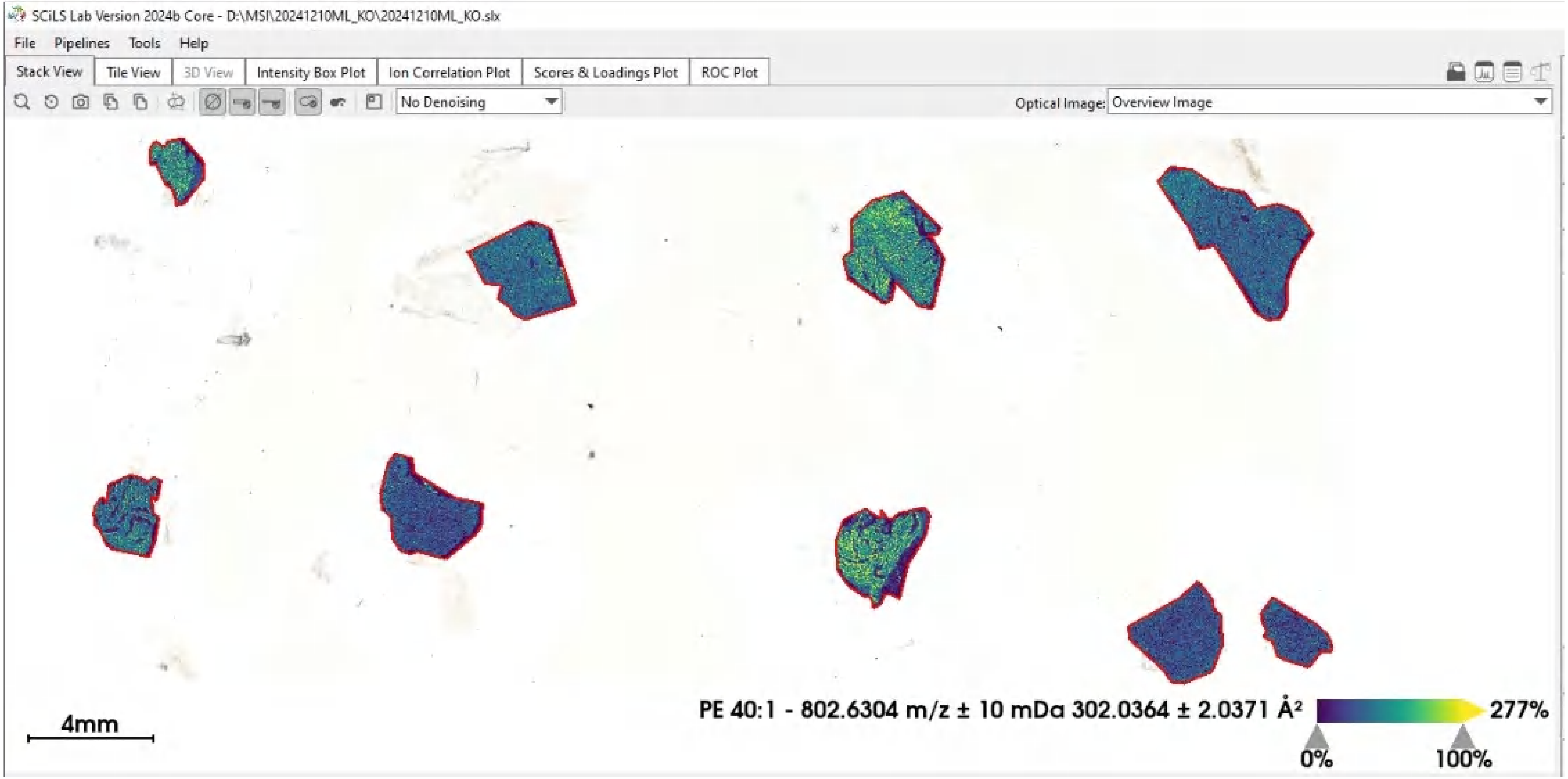
SCiLS Lab Version 2024b Core - D:\MSI\20241210ML_KO\20241210ML_KO.slx
File
Pipelines
Tools
Help
Stack View
Tile View
3D View
Intensity Box Plot
Ion Correlation Plot
Scores & Loadings Plot
ROC Plot
No Denoising
Optical Image:
Overview Image
4mm
PE 40:1 - 802.6304 m/z ± 10 mDa 302.0364 ± 2.0371 Å²
277%
0%
100%

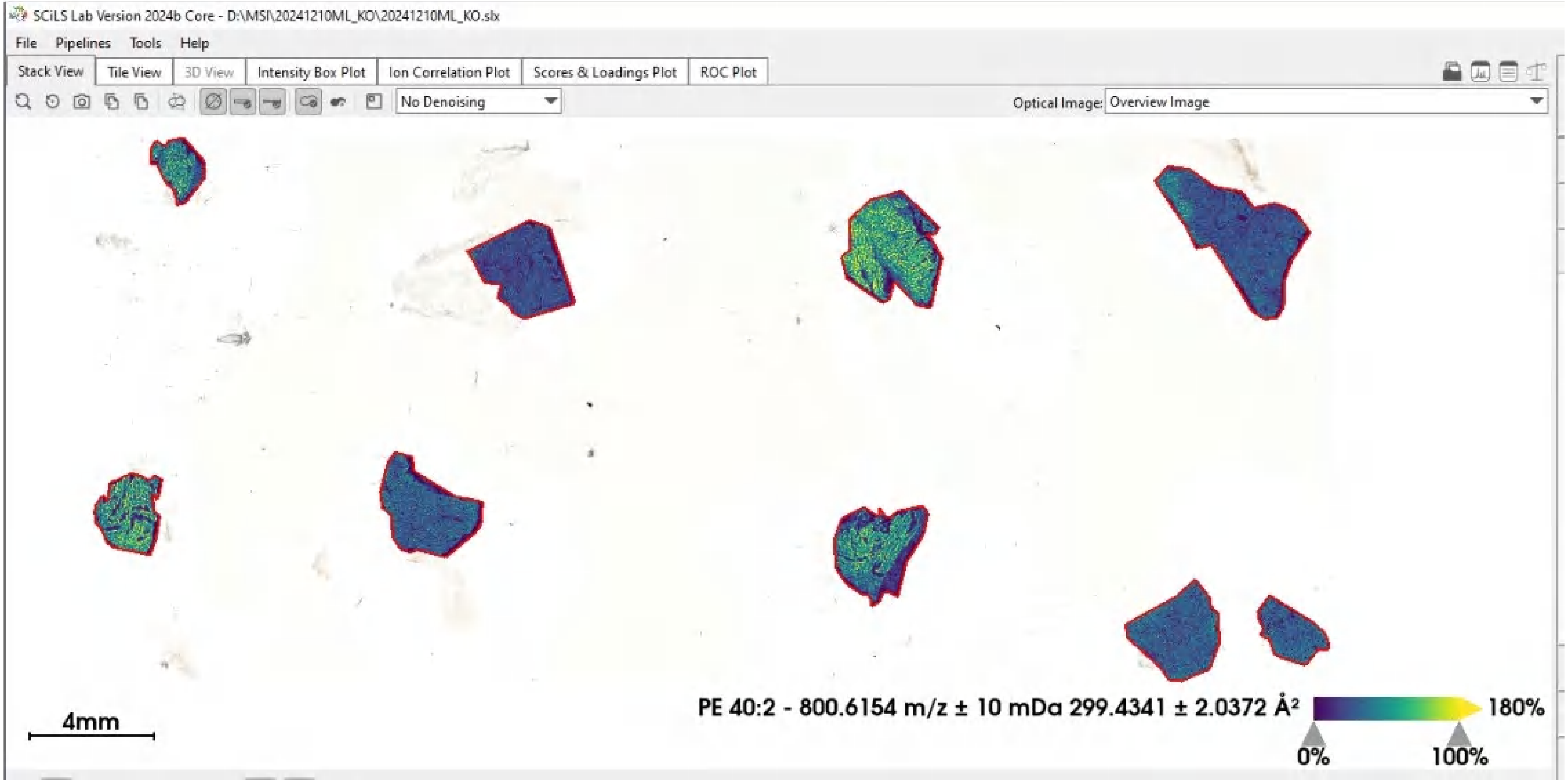
SCiLS Lab Version 2024b Core - D:\MSI\20241210ML_KO\20241210ML_KO.slx
File
Pipelines
Tools
Help
Stack View
Tile View
3D View
Intensity Box Plot
Ion Correlation Plot
Scores & Loadings Plot
ROC Plot
No Denoising
Optical Image:
Overview Image
4mm
PE 40:2 - 800.6154 m/z ± 10 mDa 299.4341 ± 2.0372 Å²
180%
0%
100%

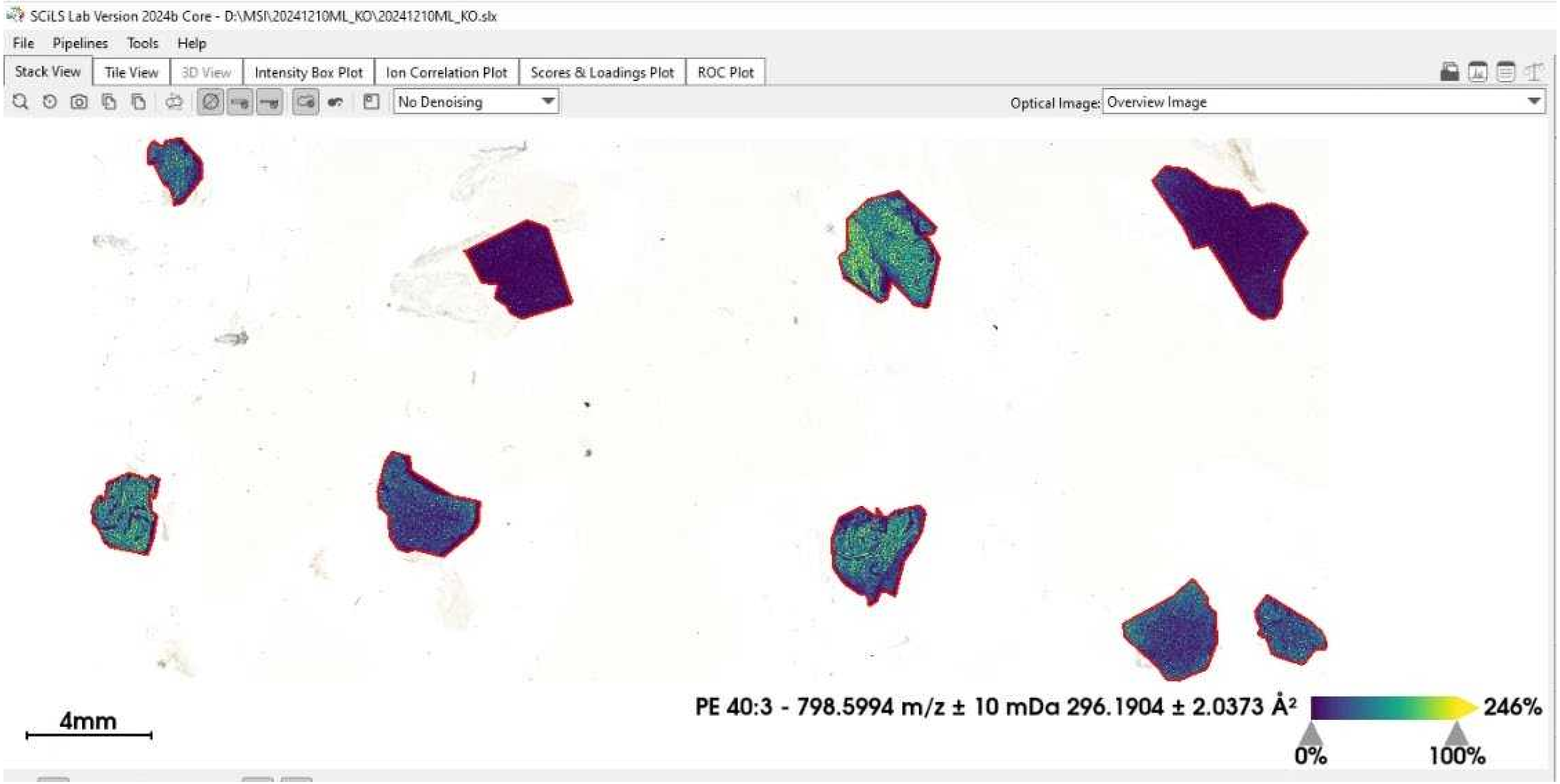
SCiLS Lab Version 2024b Core - D:\MSI\20241210ML_KO\20241210ML_KO.slx
File
Pipelines
Tools
Help
Stack View
Tile View
3D View
Intensity Box Plot
Ion Correlation Plot
Scores & Loadings Plot
ROC Plot
No Denoising
Optical Image:
Overview Image
4mm
PE 40:3 - 798.5994 m/z ± 10 mDa 296.1904 ± 2.0373 Å²
246%
0%
100%

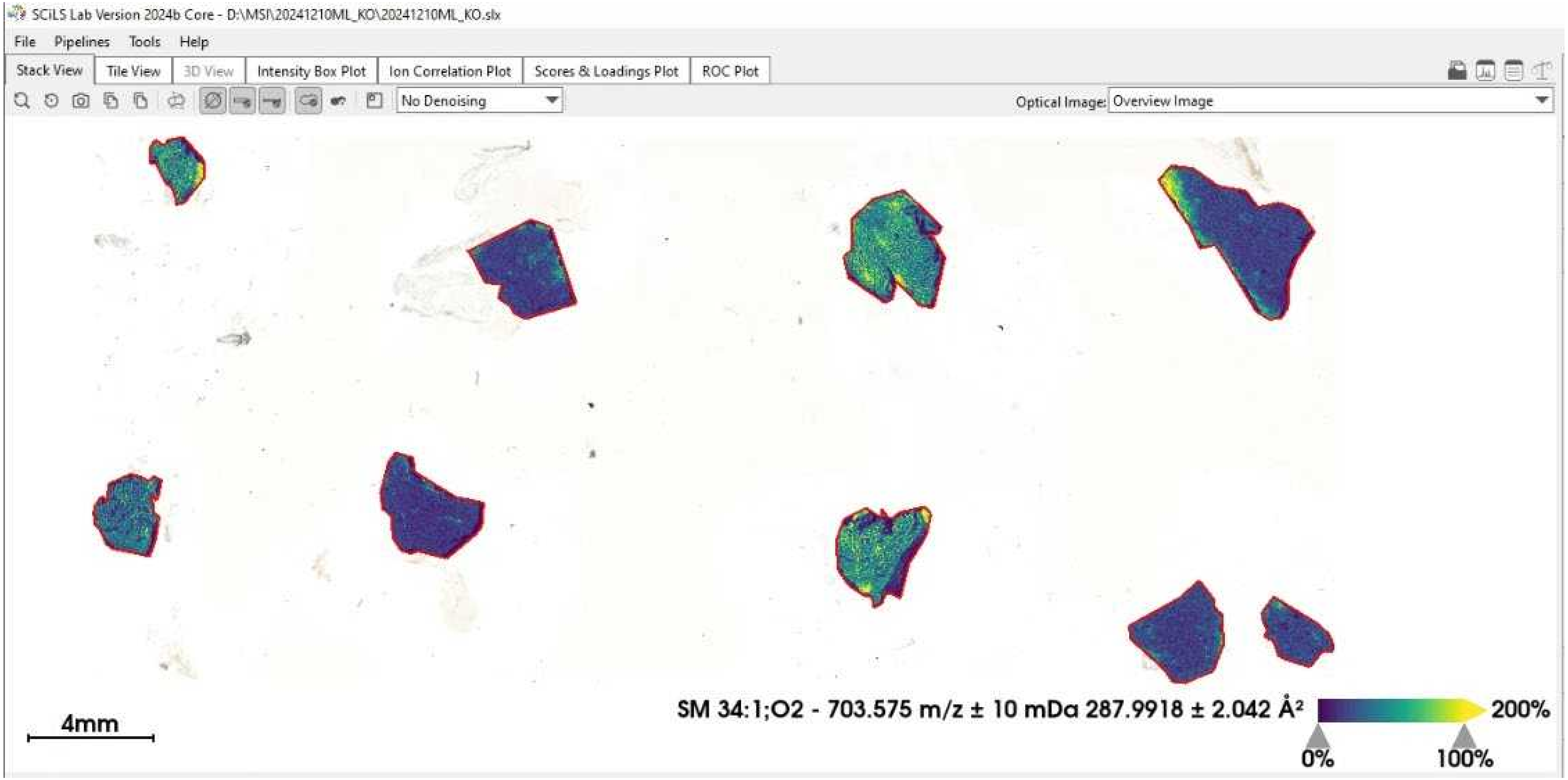
SCiLS Lab Version 2024b Core - D:\MSI\20241210ML_KO\20241210ML_KO.slx
File
Pipelines
Tools
Help
Stack View
Tile View
3D View
Intensity Box Plot
Ion Correlation Plot
Scores & Loadings Plot
ROC Plot
No Denoising
Optical Image:
Overview Image
4mm
SM 34:1;O2 - 703.575 m/z ± 10 mDa 287.9918 ± 2.042 Å²
200%
0%
100%

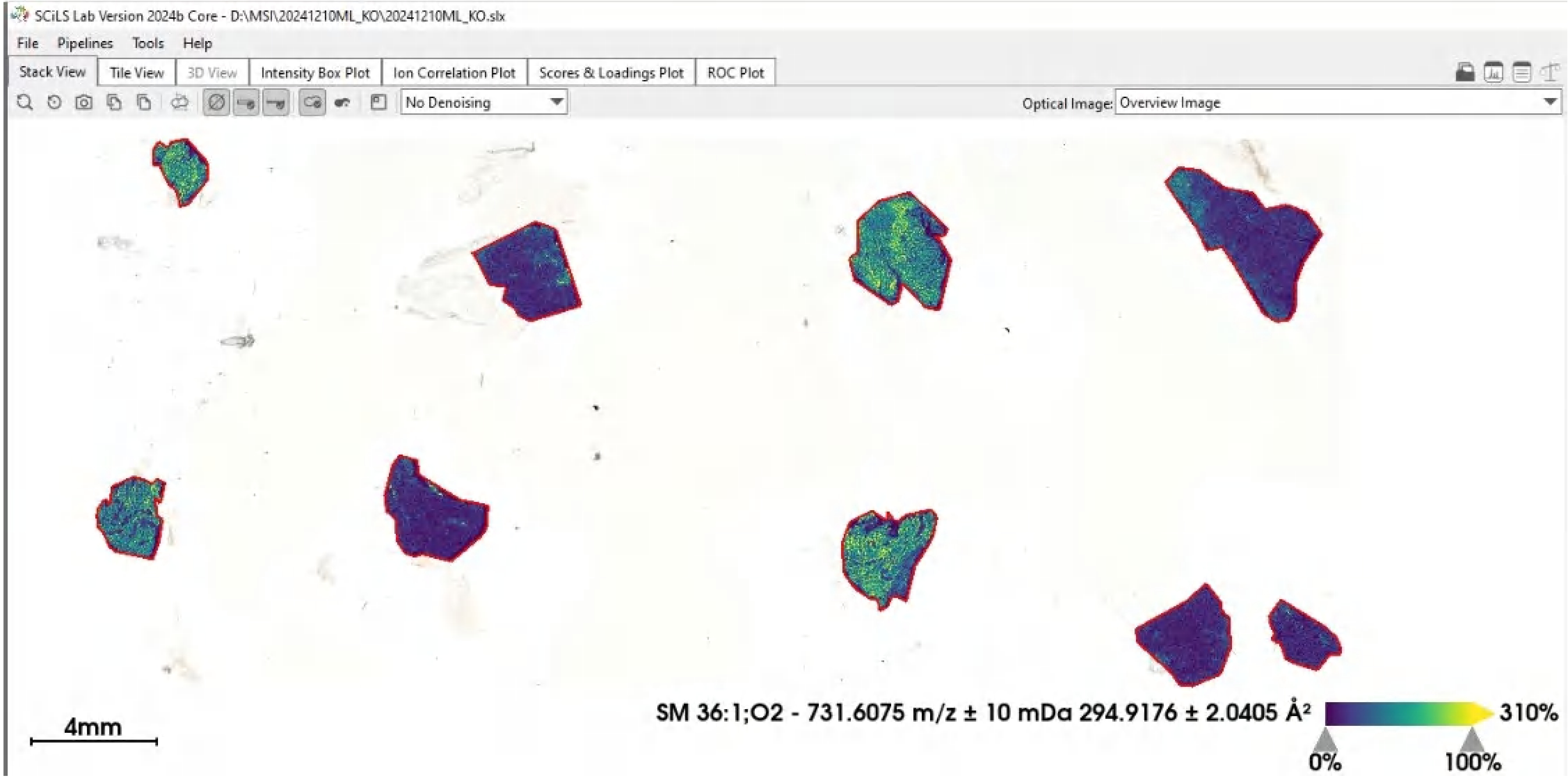
SCiLS Lab Version 2024b Core - D:\MSI\20241210ML_KO\20241210ML_KO.slx
File
Pipelines
Tools
Help
Stack View
Tile View
3D View
Intensity Box Plot
Ion Correlation Plot
Scores & Loadings Plot
ROC Plot
No Denoising
Optical Image:
Overview Image
4mm
SM 36:1;O2 - 731.6075 m/z ± 10 mDa 294.9176 ± 2.0405 Å²
310%
0%
100%

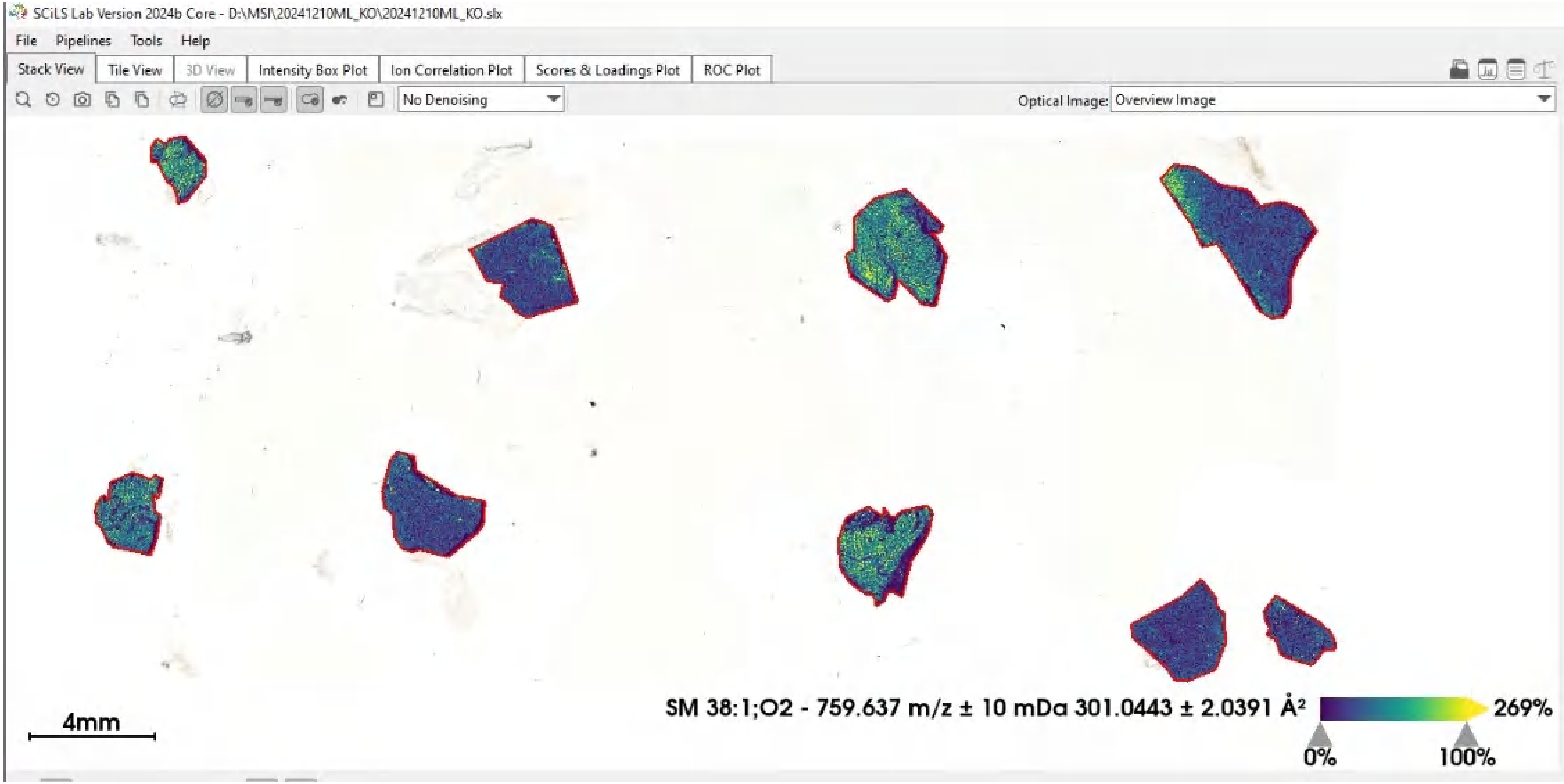
SCiLS Lab Version 2024b Core - D:\MSI\20241210ML_KO\20241210ML_KO.slx
File
Pipelines
Tools
Help
Stack View
Tile View
3D View
Intensity Box Plot
Ion Correlation Plot
Scores & Loadings Plot
ROC Plot
No Denoising
Optical Image:
Overview Image
4mm
SM 38:1;O2 - 759.637 m/z ± 10 mDa 301.0443 ± 2.0391 Å²
269%
0%
100%

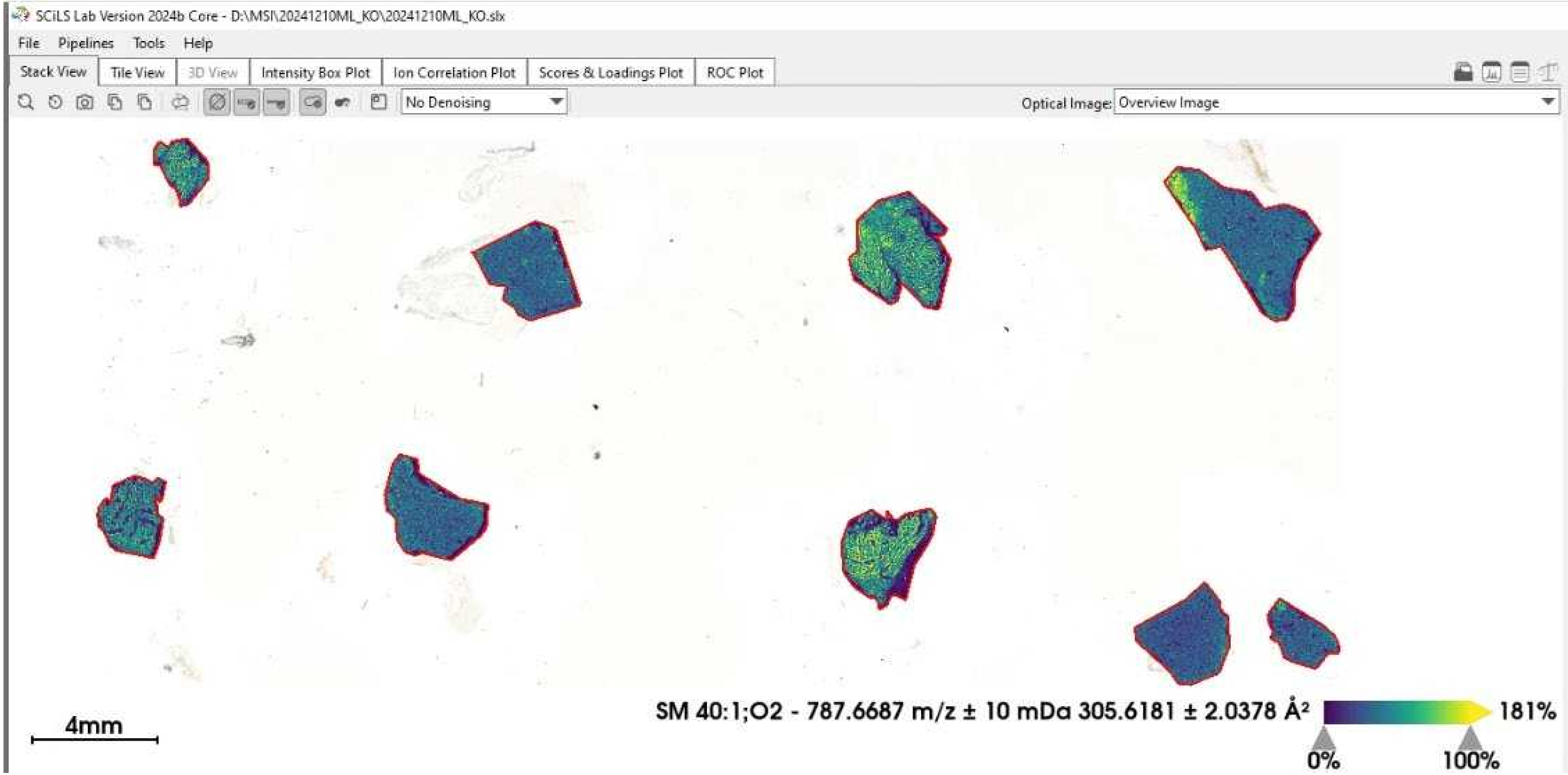
SCiLS Lab Version 2024b Core - D:\MSI\20241210ML_KO\20241210ML_KO.slx
File
Pipelines
Tools
Help
Stack View
Tile View
3D View
Intensity Box Plot
Ion Correlation Plot
Scores & Loadings Plot
ROC Plot
No Denoising
Optical Image:
Overview Image
4mm
SM 40:1;O2 - 787.6687 m/z ± 10 mDa 305.6181 ± 2.0378 Å²
181%
0%
100%

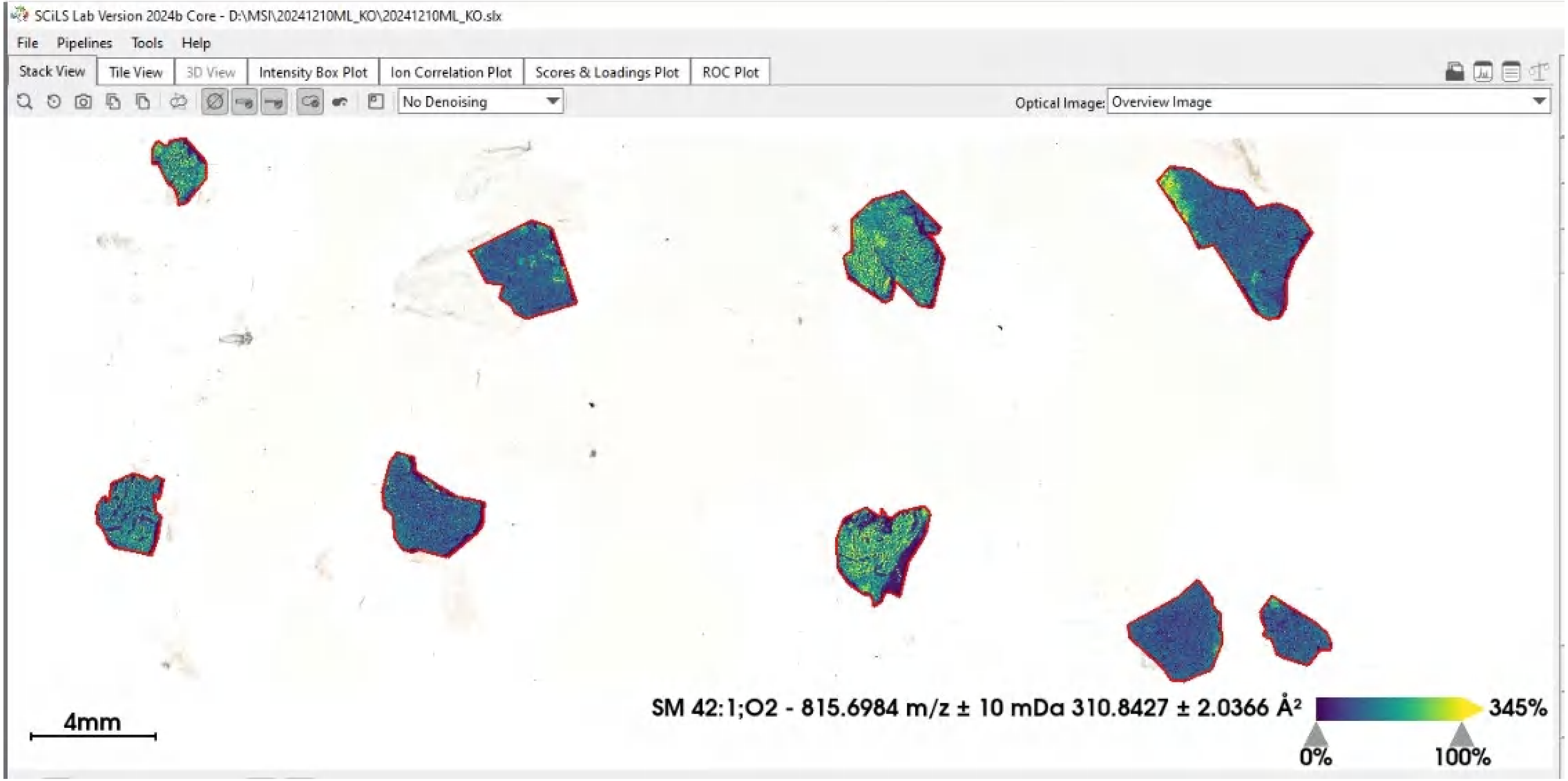

SCiLS Lab Version 2024b Core - D:\MSI\20241210ML_KO\20241210ML_KO.slx
File
Pipelines
Tools
Help
Stack View
Tile View
3D View
Intensity Box Plot
Ion Correlation Plot
Scores & Loadings Plot
ROC Plot
No Denoising
Optical Image:
Overview Image
4mm
SM 42:1;O2 - 815.6984 m/z ± 10 mDa 310.8427 ± 2.0366 Å²
345%
0%
100%

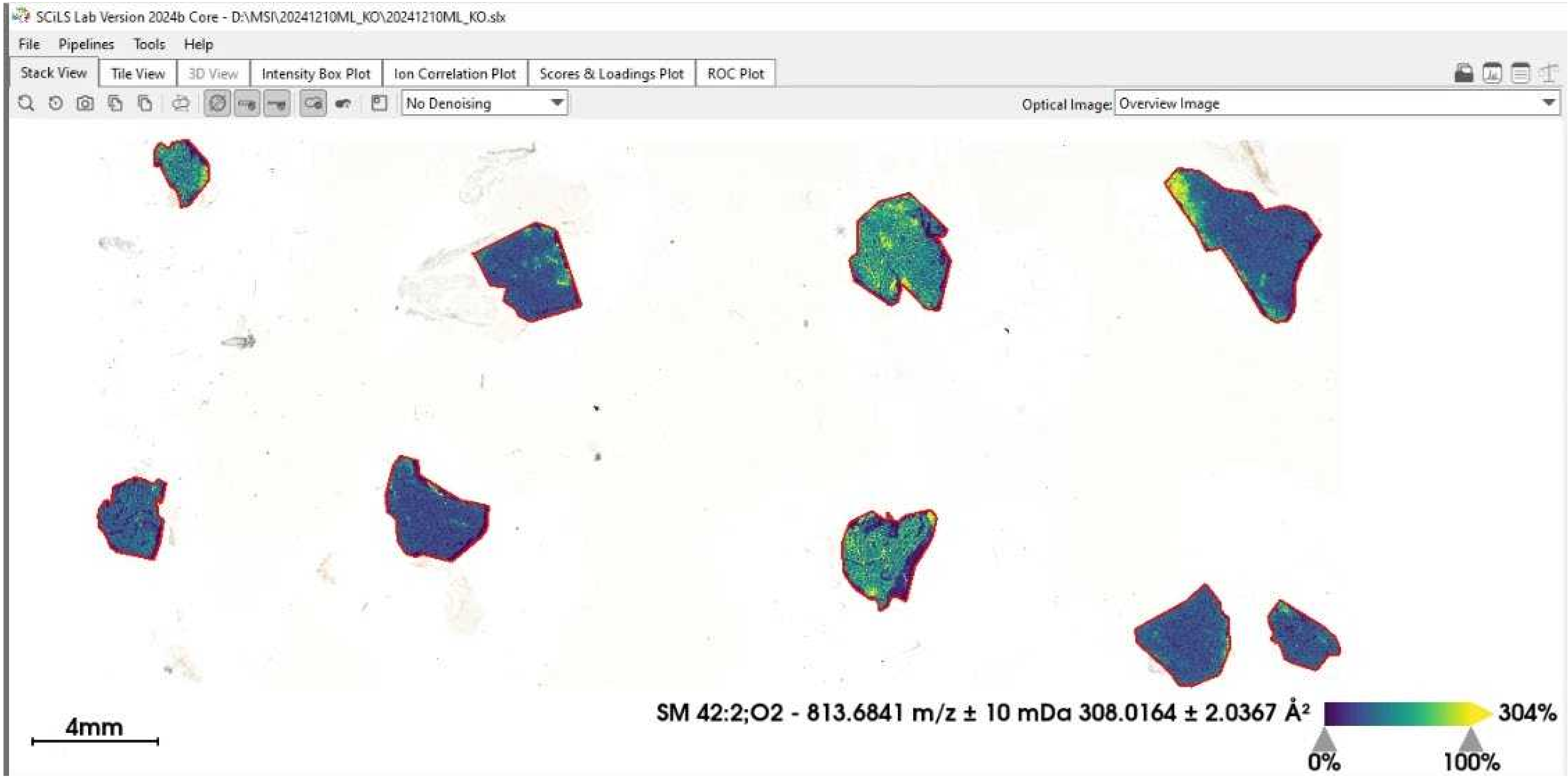
SCiLS Lab Version 2024b Core - D:\MSI\20241210ML_KO\20241210ML_KO.slx
File
Pipelines
Tools
Help
Stack View
Tile View
3D View
Intensity Box Plot
Ion Correlation Plot
Scores & Loadings Plot
ROC Plot
No Denoising
Optical Image:
Overview Image
4mm
SM 42:2;O2 - 813.6841 m/z ± 10 mDa 308.0164 ± 2.0367 Å²
304%
0%
100%

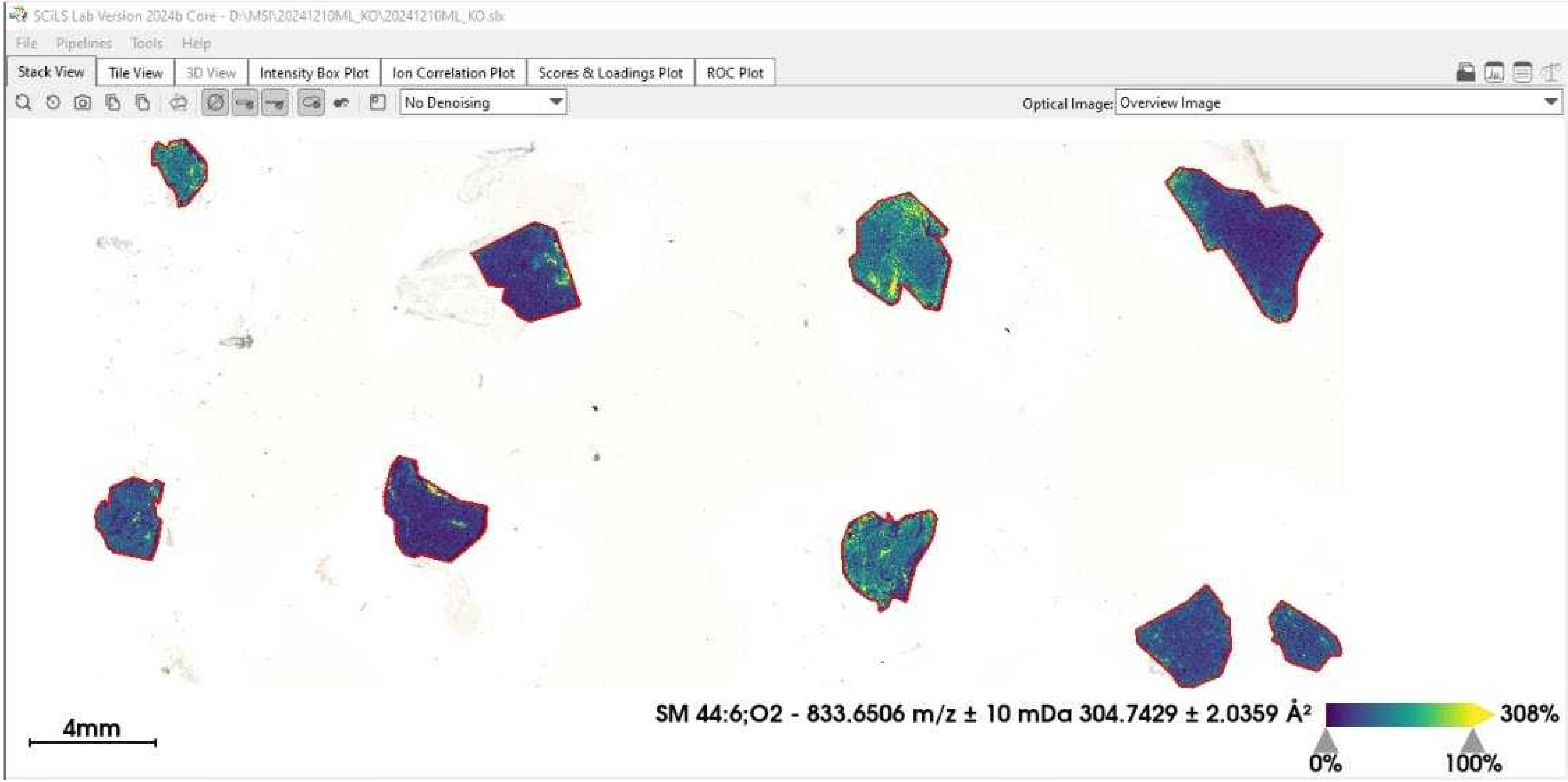
SCiLS Lab Version 2024b Core - D:\MSI\20241210ML_KO\20241210ML_KO.slx
File
Pipelines
Tools
Help
Stack View
Tile View
3D View
Intensity Box Plot
Ion Correlation Plot
Scores & Loadings Plot
ROC Plot
No Denoising
Optical Image:
Overview Image
4mm
SM 44:6;O2 - 833.6506 m/z ± 10 mDa 304.7429 ± 2.0359 Å²
308%
0%
100%

Second Replication from here

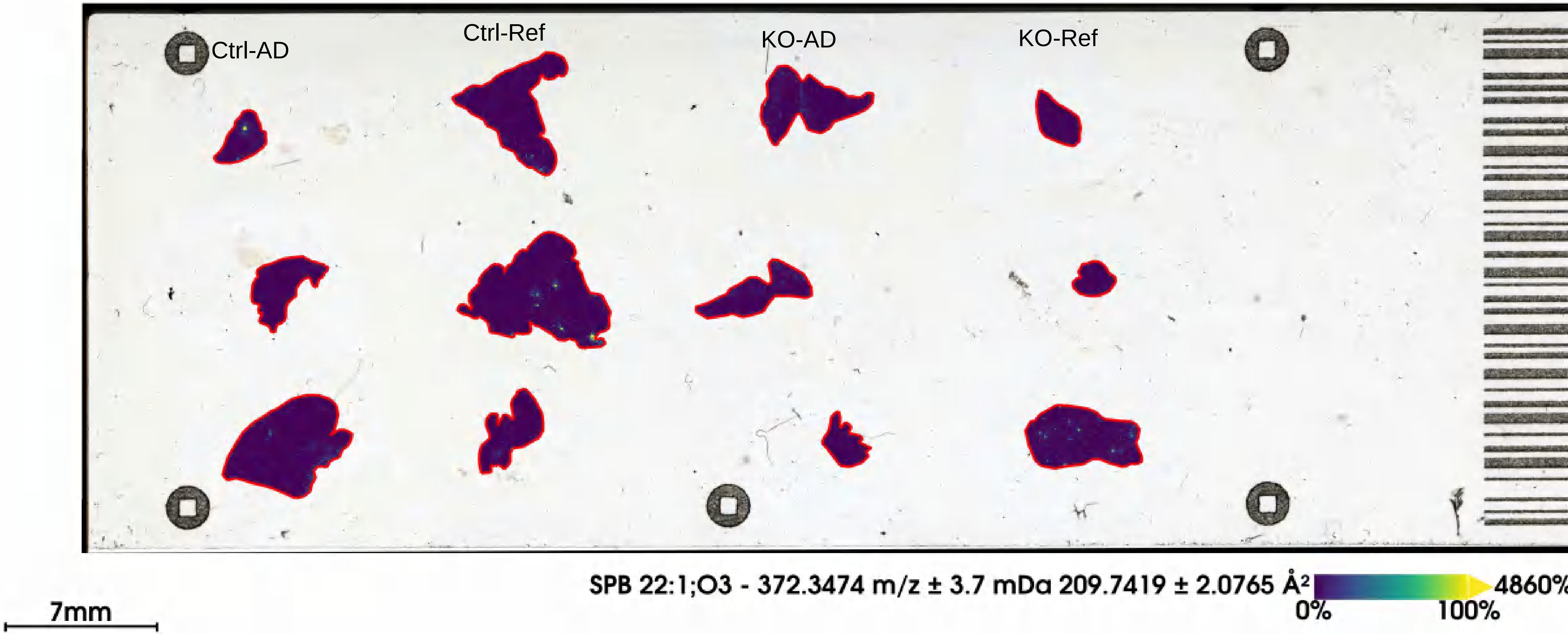

Ctrl-AD
Ctrl-Ref
KO-AD
KO-Ref
7mm
SPB 22:1;O3 - 372.3474 m/z ± 3.7 mDa 209.7419 ± 2.0765 Å²
0%
100%
4860%

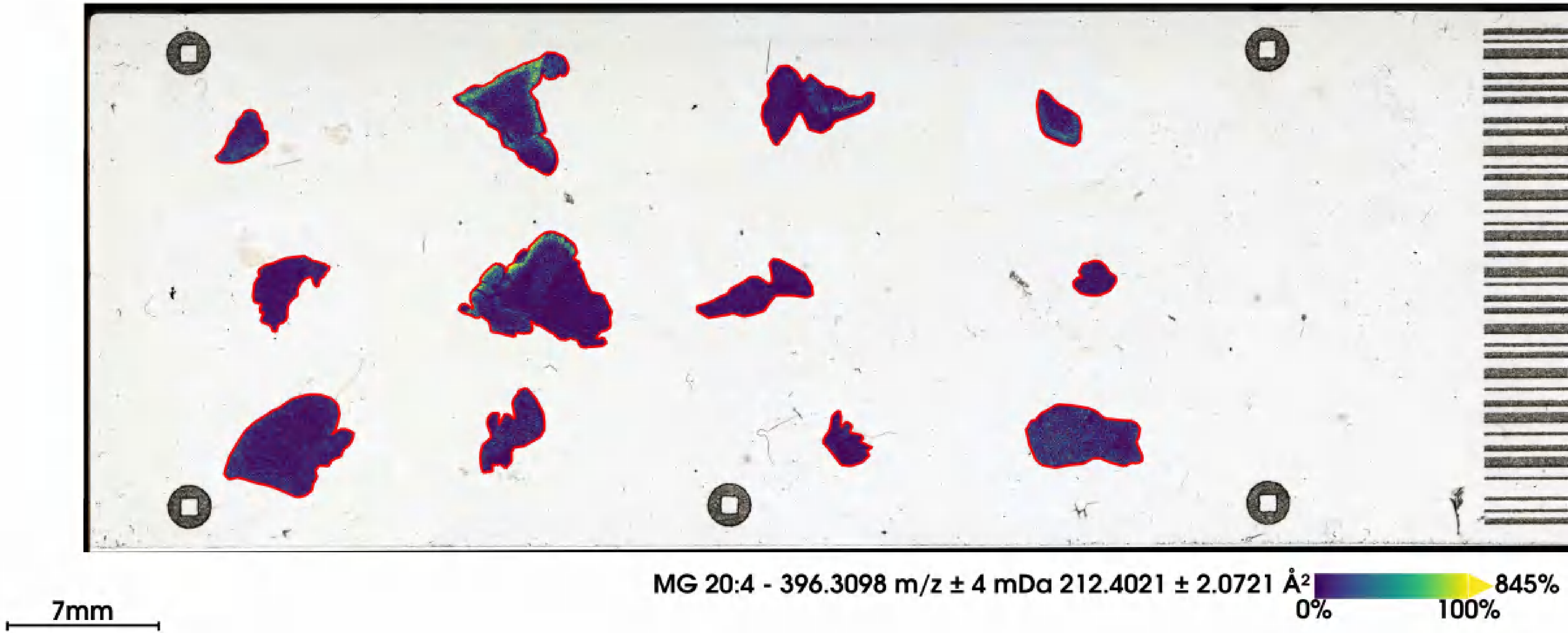

MG 20:4 - 396.3098 m/z ± 4 mDa 212.4021 ± 2.0721 Å²
0%
100%
845%
7mm

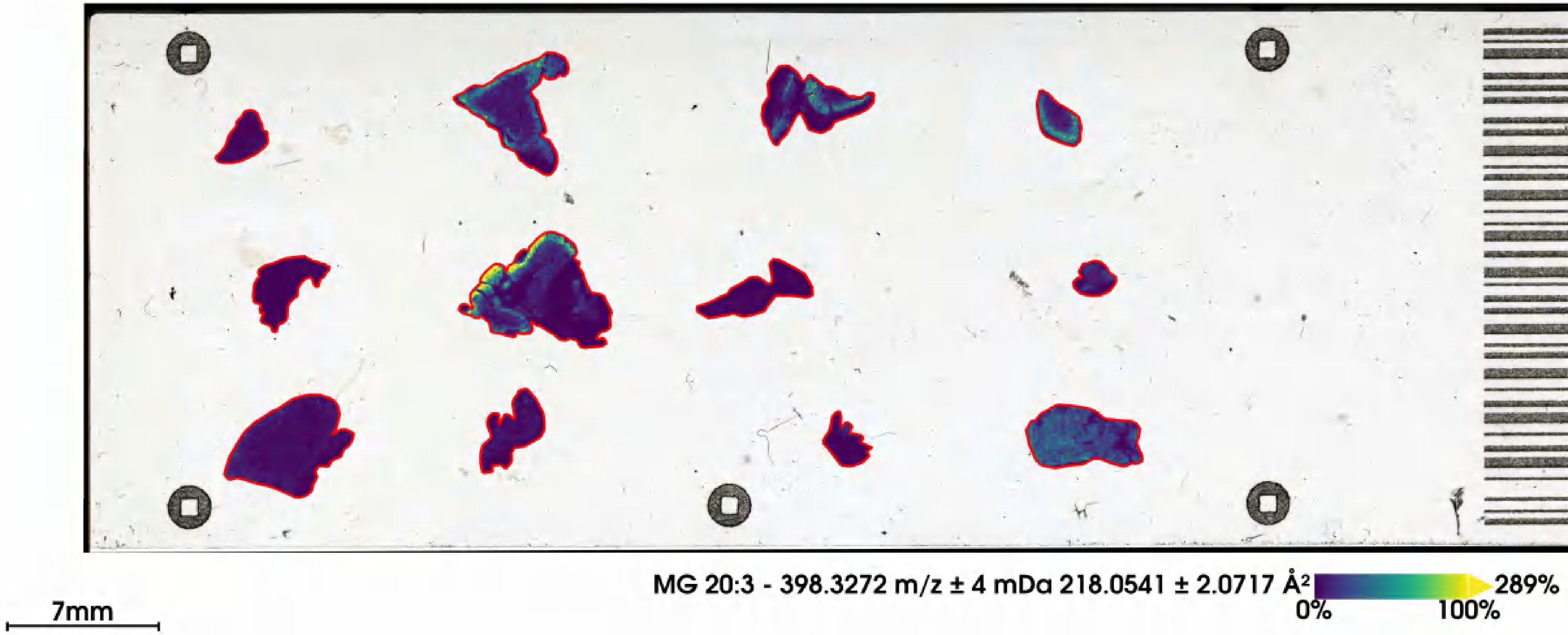
MG 20:3 - 398.3272 m/z ± 4 mDa 218.0541 ± 2.0717 Å²
289%
0%
100%
7mm

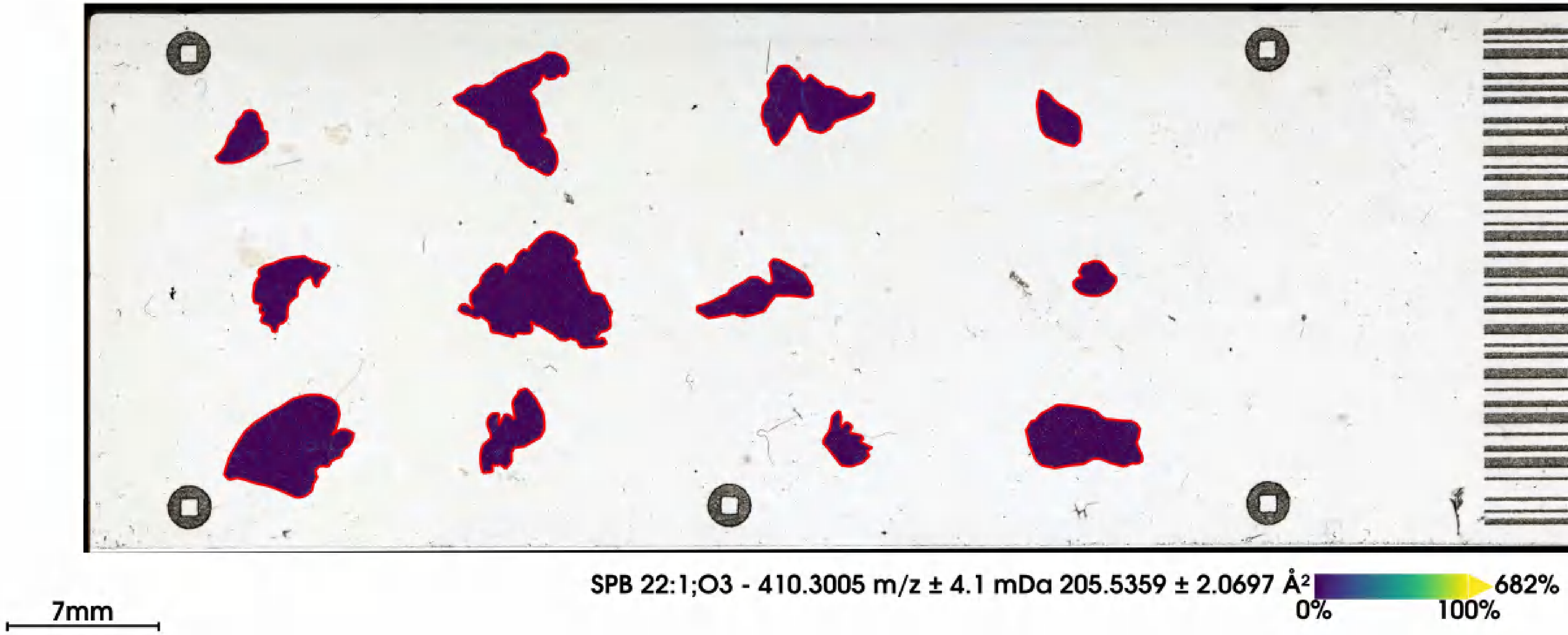
SPB 22:1;O3 - 410.3005 m/z ± 4.1 mDa 205.5359 ± 2.0697 Å²
0%
100%
682%
7mm

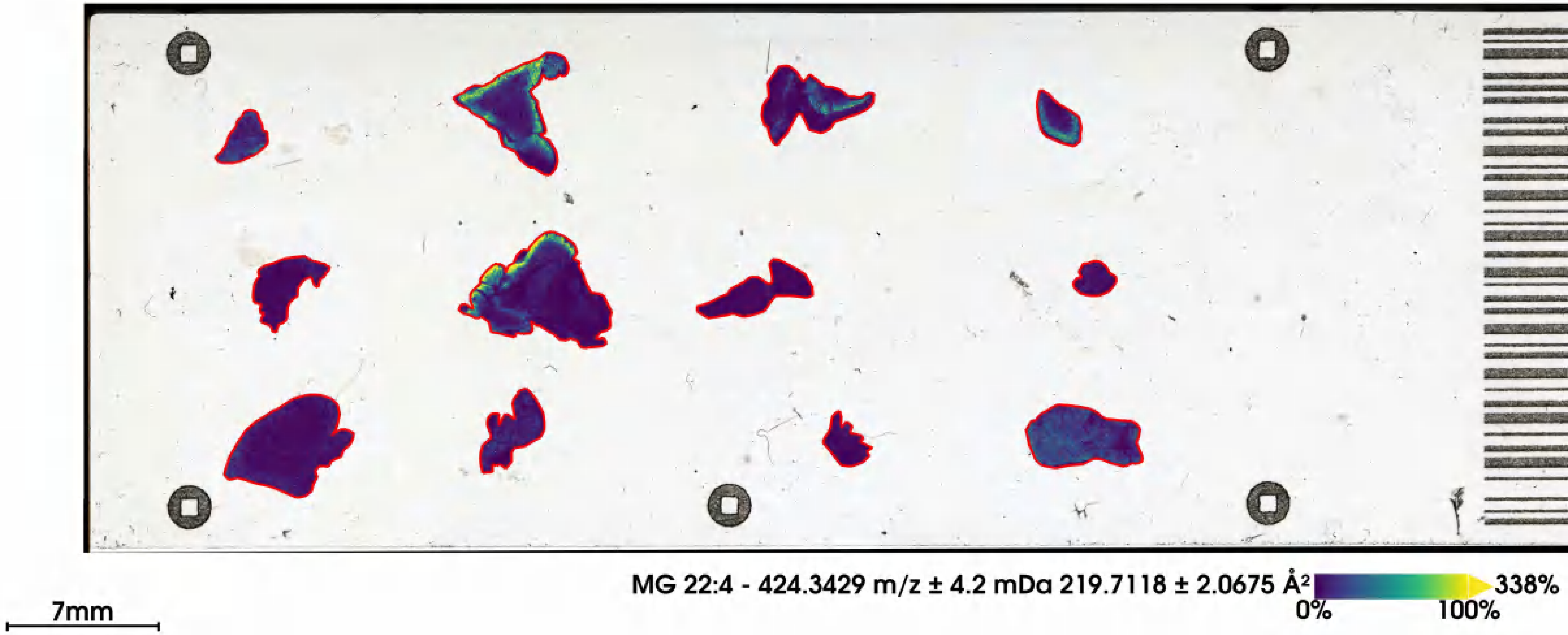

MG 22:4 - 424.3429 m/z ± 4.2 mDa 219.7118 ± 2.0675 Å²
0%
100%
338%
7mm

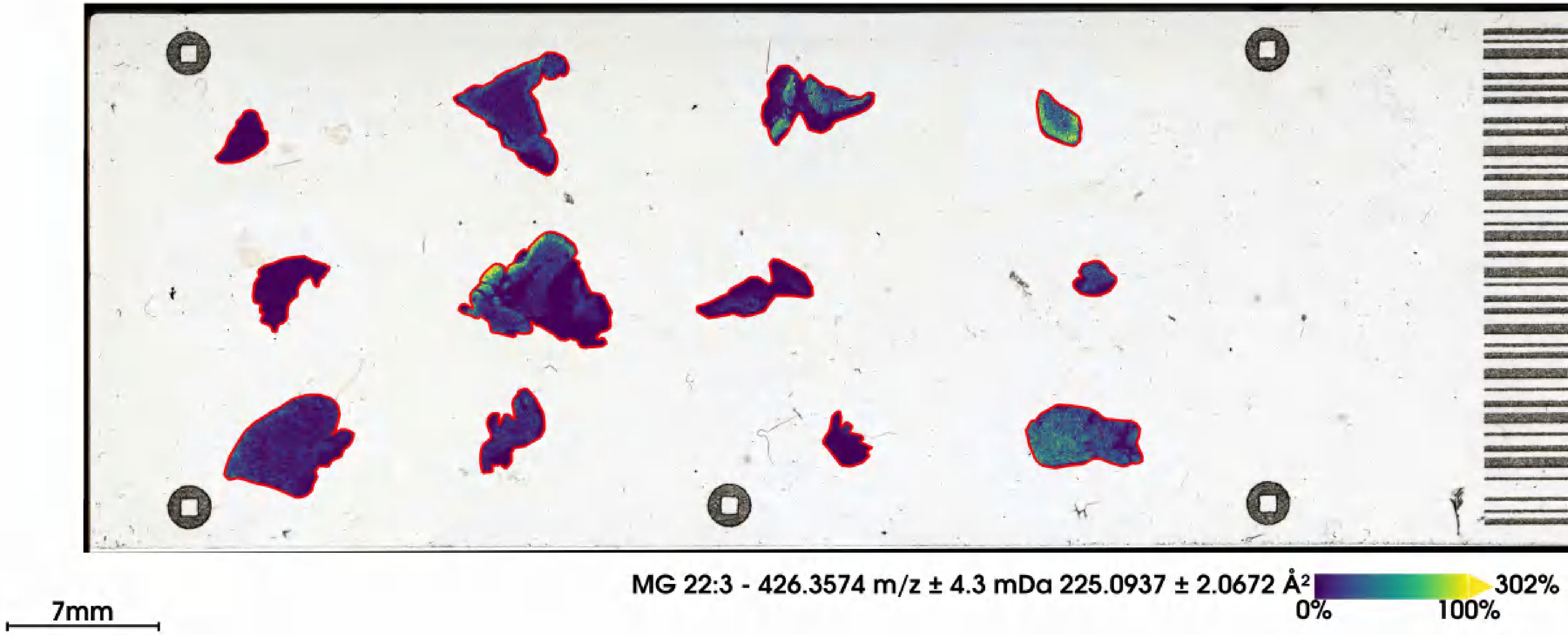
MG 22:3 - 426.3574 m/z ± 4.3 mDa 225.0937 ± 2.0672 Å²
302%
0%
100%
7mm

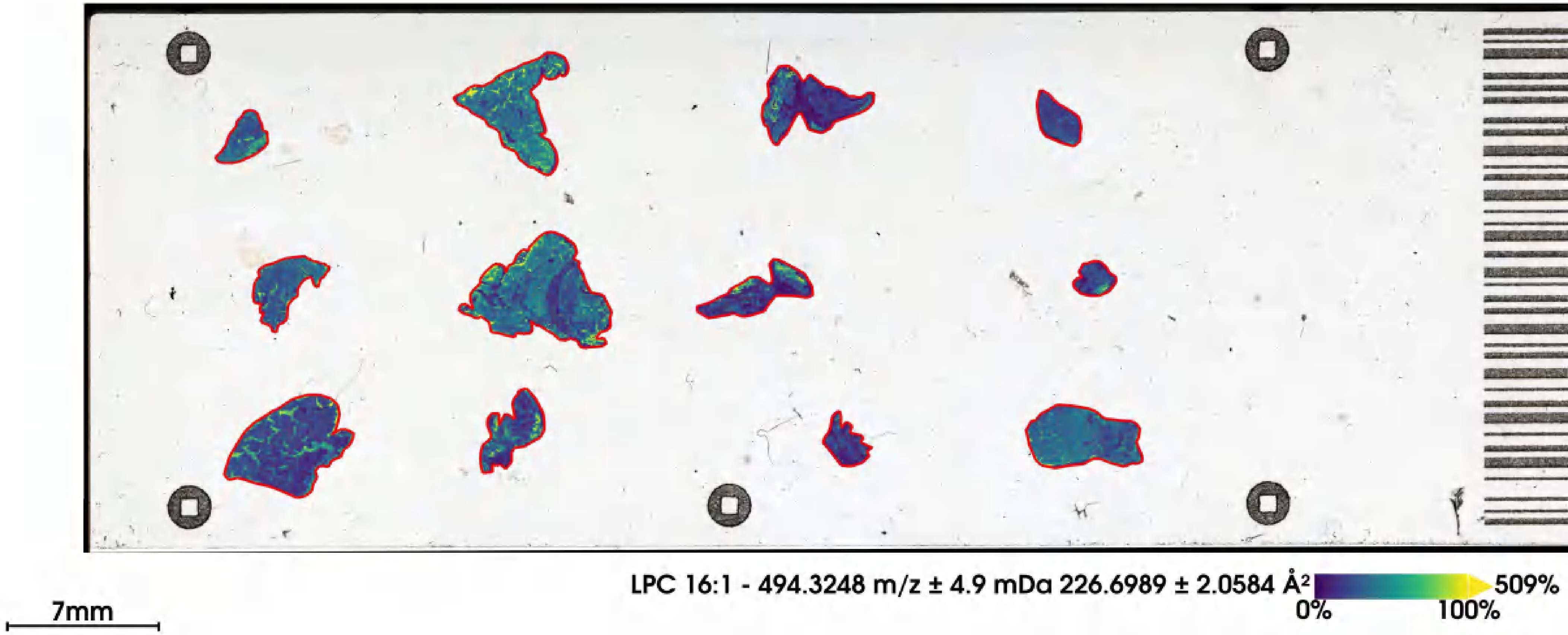
LPC 16:1 - 494.3248 m/z ± 4.9 mDa 226.6989 ± 2.0584 Å²
0%
100%
509%
7mm

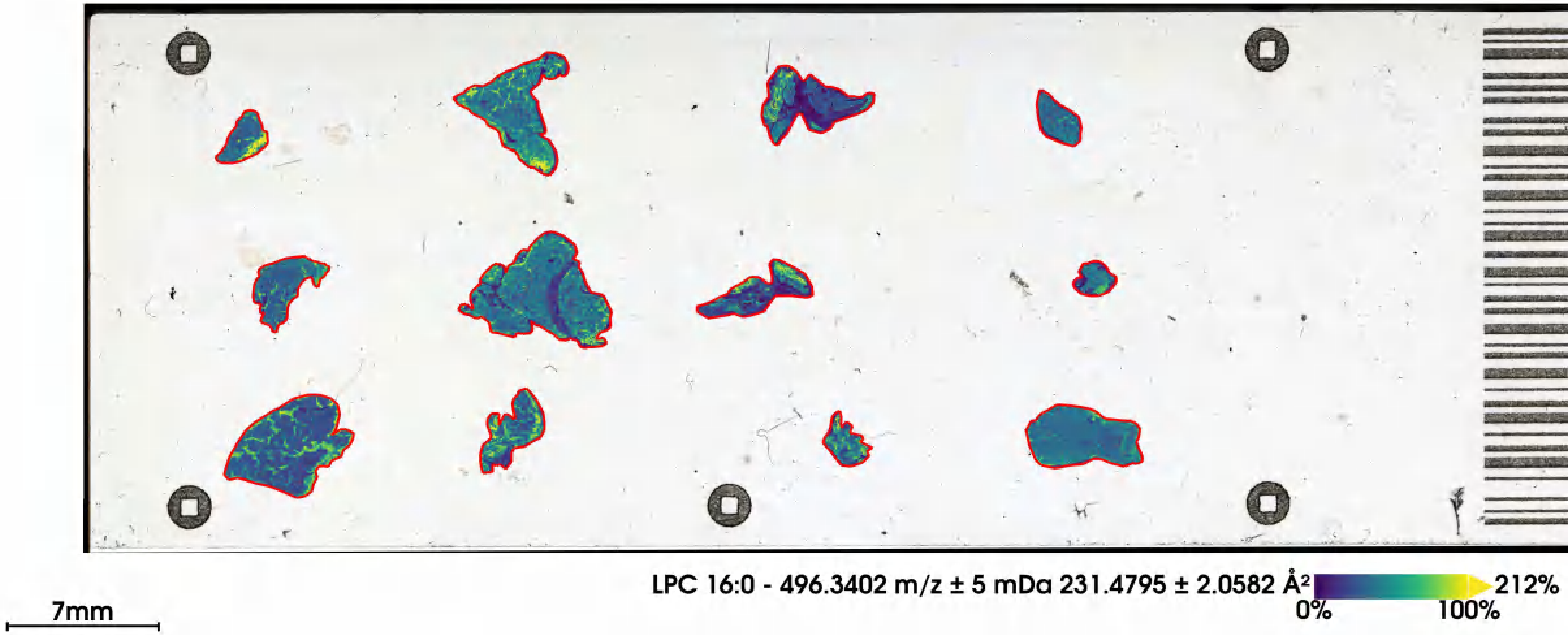
LPC 16:0 - 496.3402 m/z ± 5 mDa 231.4795 ± 2.0582 Å²
0%
100%
212%
7mm

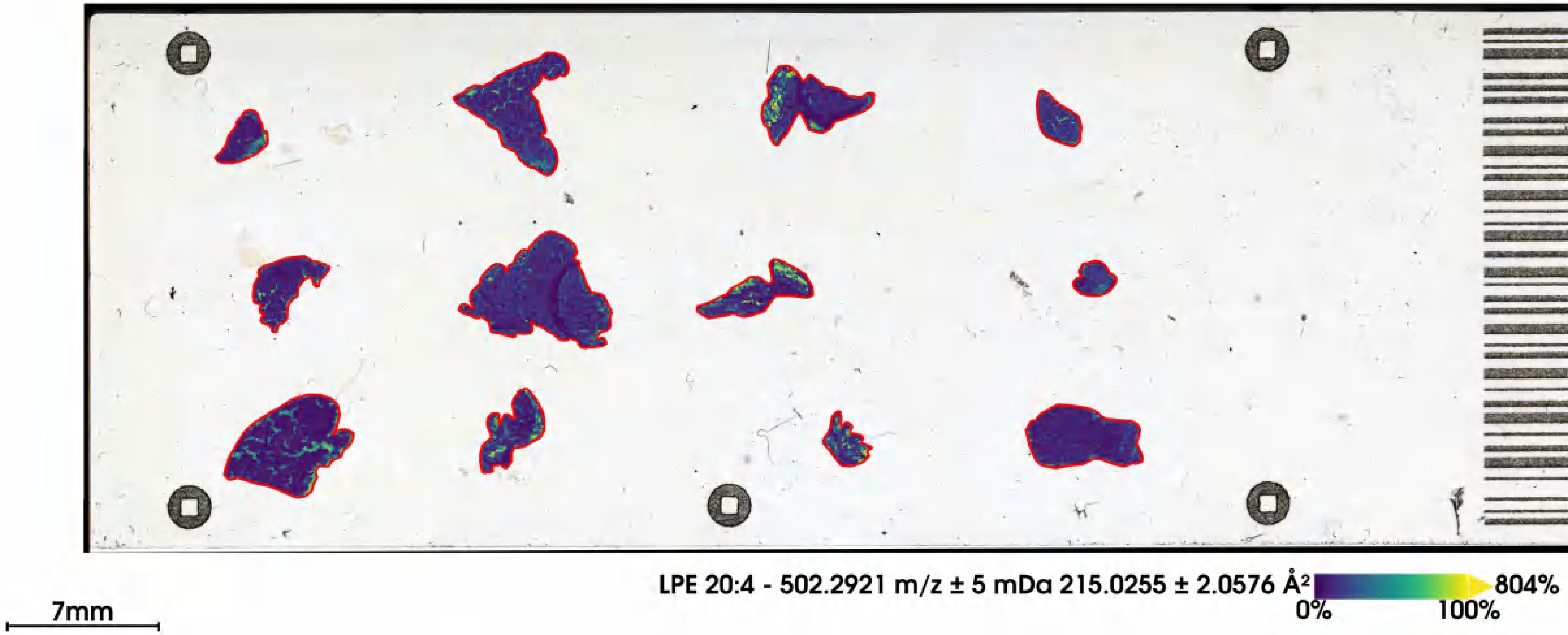
LPE 20:4 - 502.2921 m/z ± 5 mDa 215.0255 ± 2.0576 Å²
0%
100%
804%
7mm

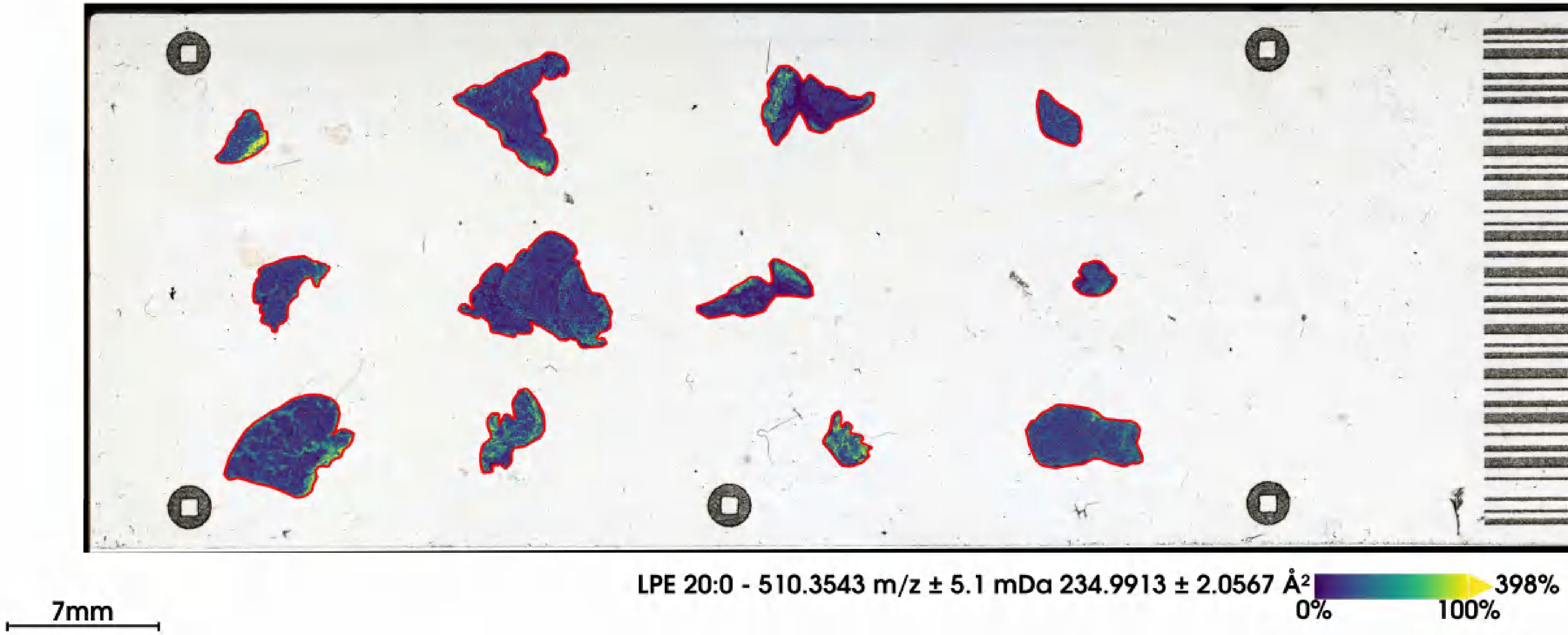
LPE 20:0 - 510.3543 m/z ± 5.1 mDa 234.9913 ± 2.0567 Å²
0%
100%
398%
7mm

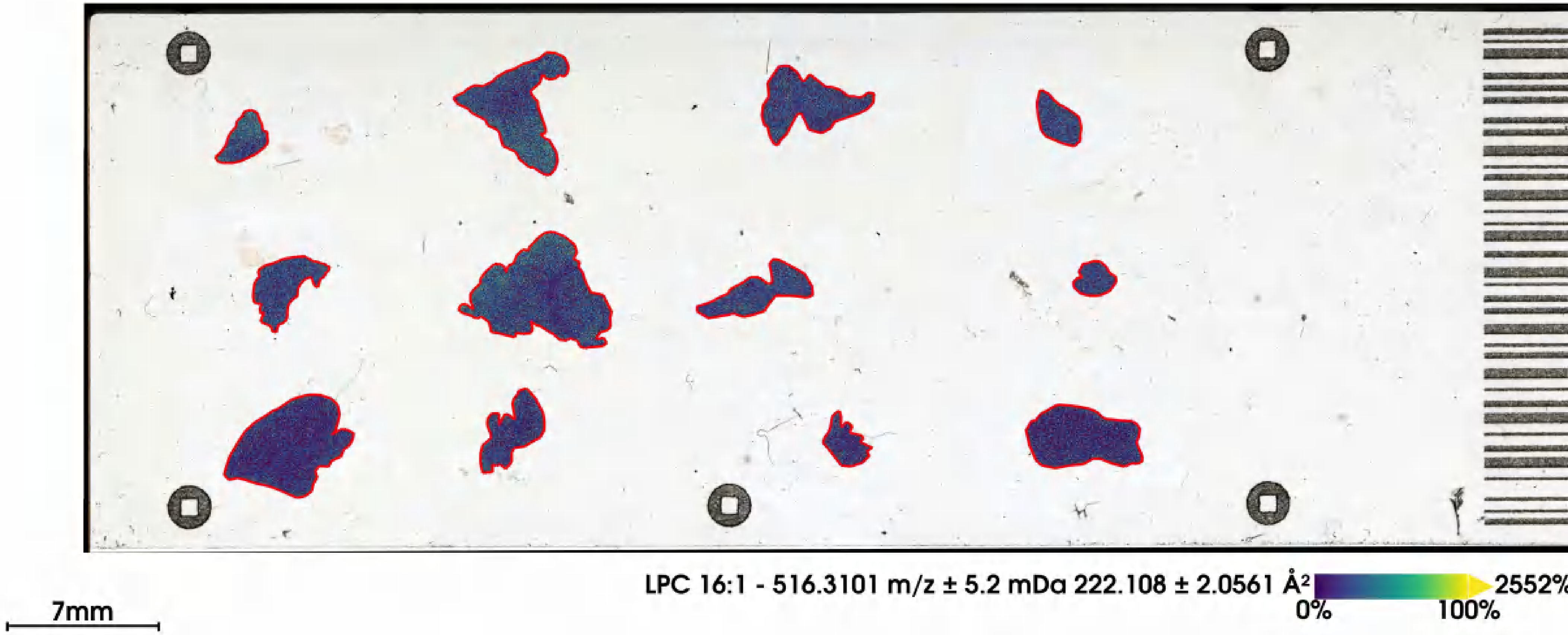
LPC 16:1 - 516.3101 m/z ± 5.2 mDa 222.108 ± 2.0561 Å²
2552%
0%
100%
7mm

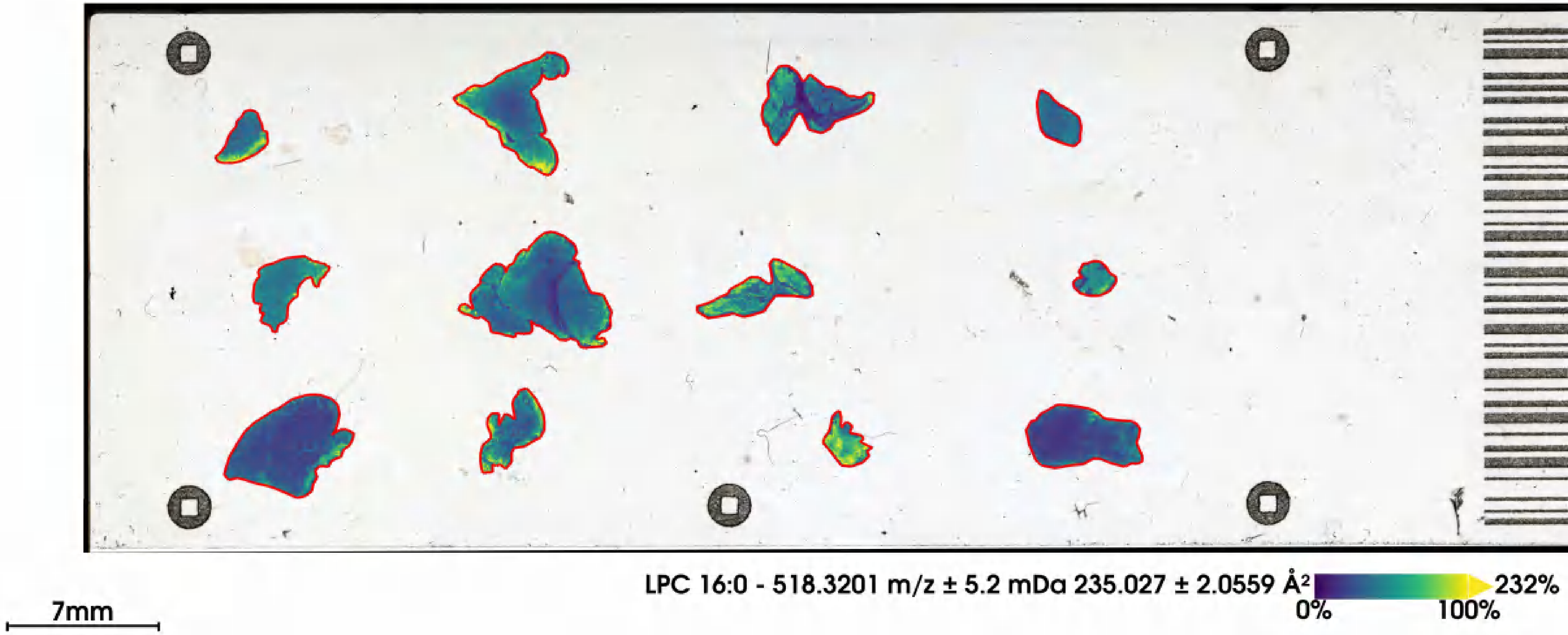
LPC 16:0 - 518.3201 m/z ± 5.2 mDa 235.027 ± 2.0559 Å²
232%
0%
100%
7mm

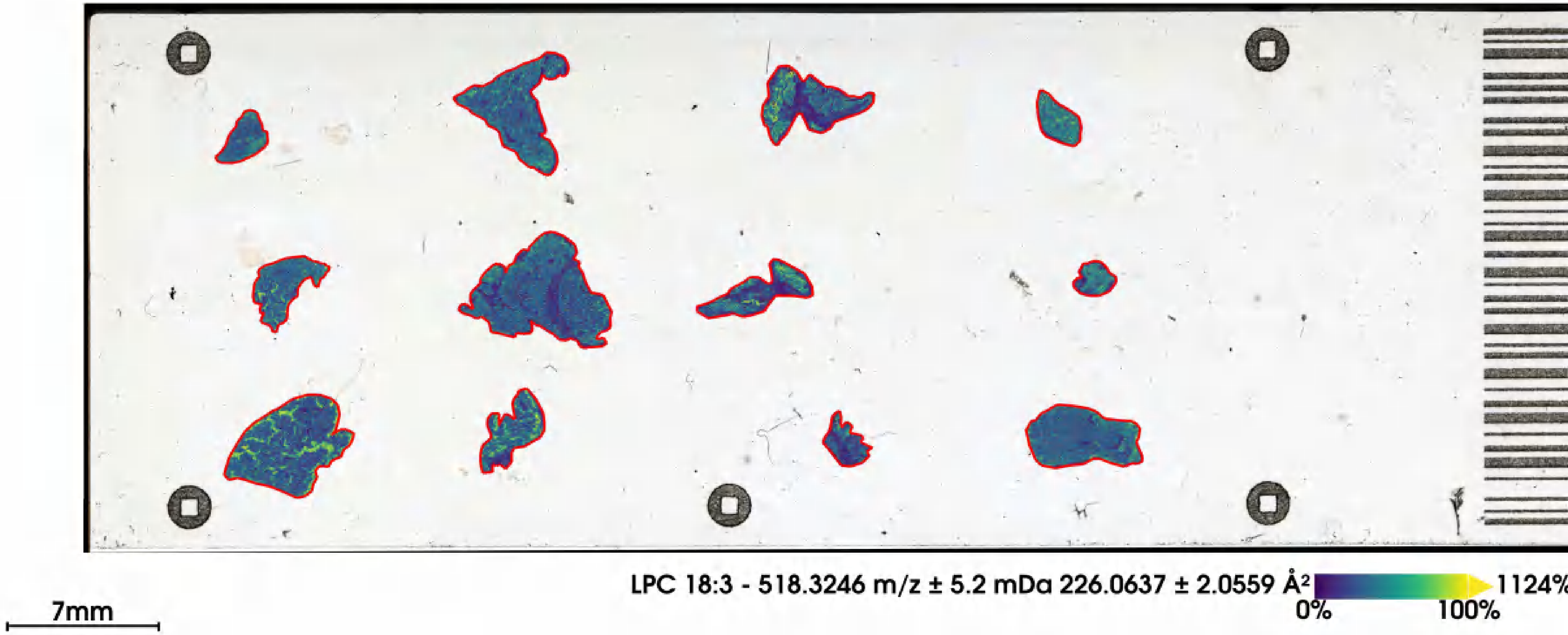

LPC 18:3 - 518.3246 m/z ± 5.2 mDa 226.0637 ± 2.0559 Å²
0%
100%
1124%
7mm

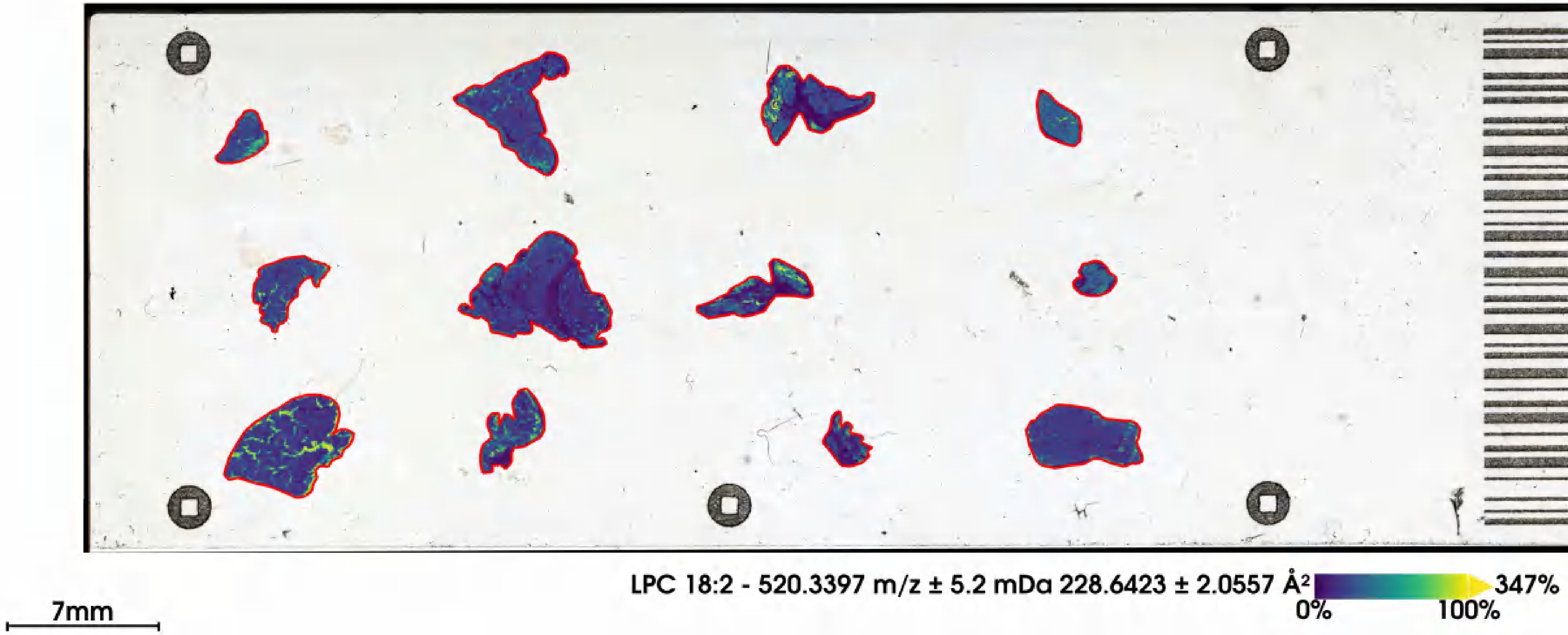
LPC 18:2 - 520.3397 m/z ± 5.2 mDa 228.6423 ± 2.0557 Å²
0%
100%
347%
7mm

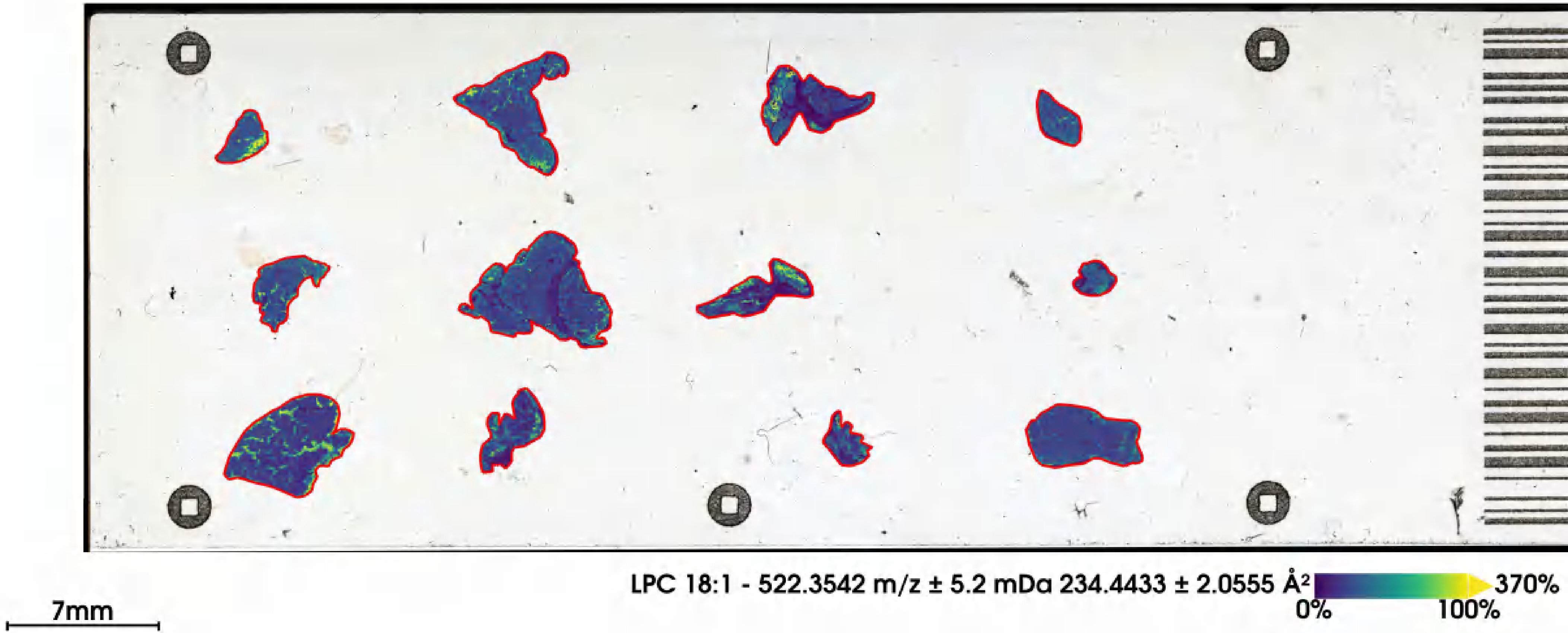

LPC 18:1 - 522.3542 m/z ± 5.2 mDa 234.4433 ± 2.0555 Å²
0%
100%
370%
7mm

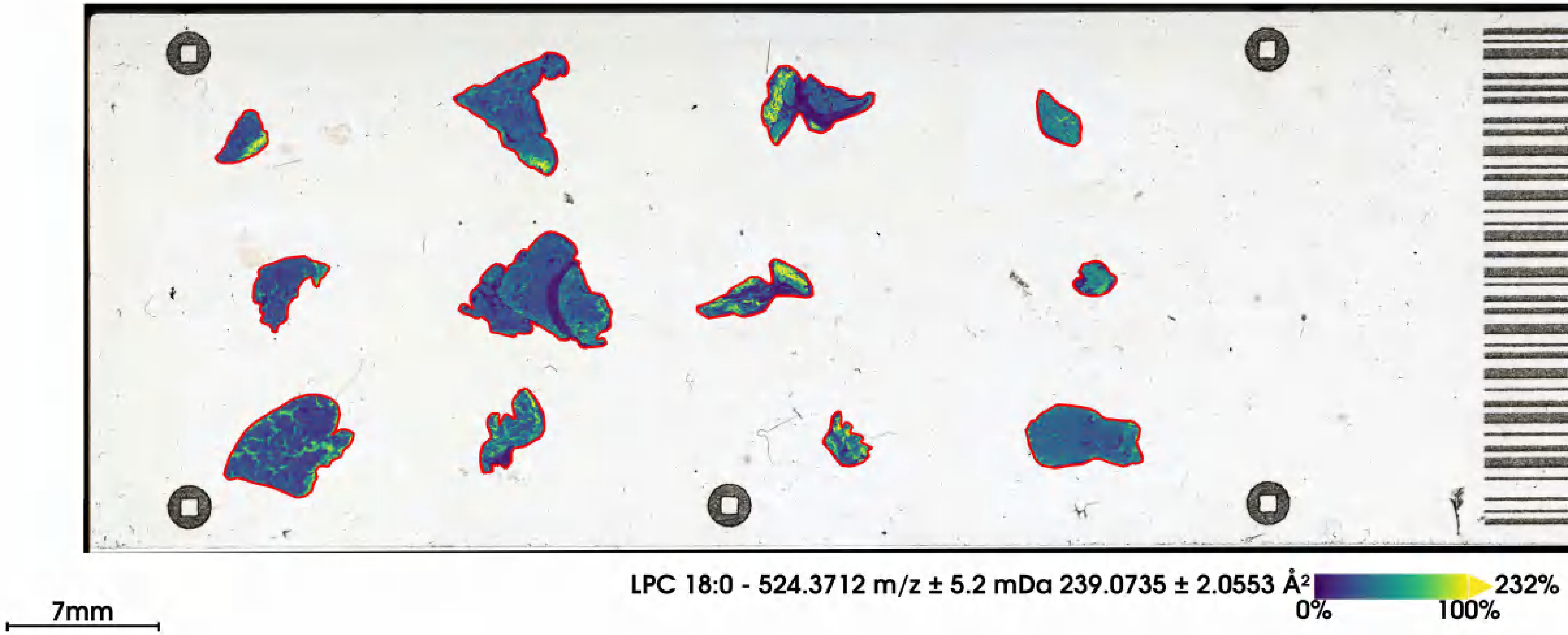

7mm
LPC 18:0 - 524.3712 m/z ± 5.2 mDa 239.0735 ± 2.0553 Å²
0%
100%
232%

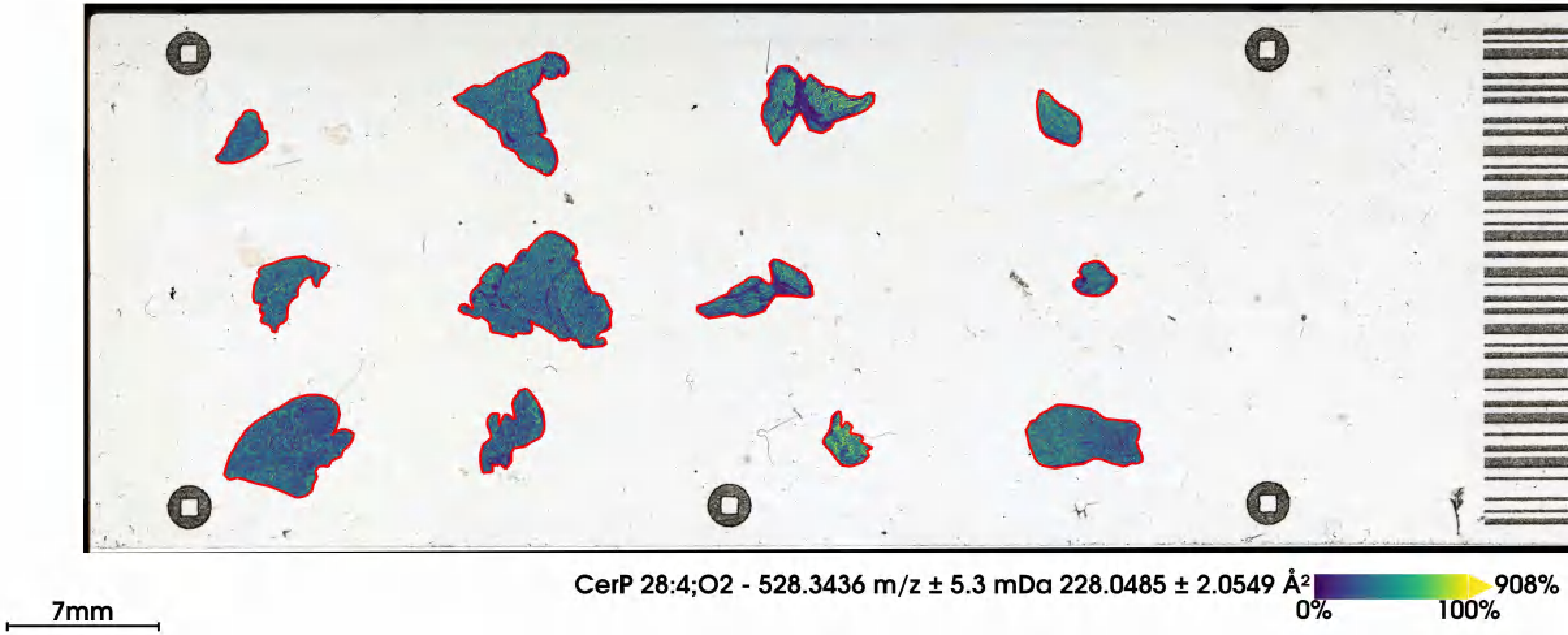
CerP 28:4;O2 - 528.3436 m/z ± 5.3 mDa 228.0485 ± 2.0549 Å²
908%
0%
100%
7mm

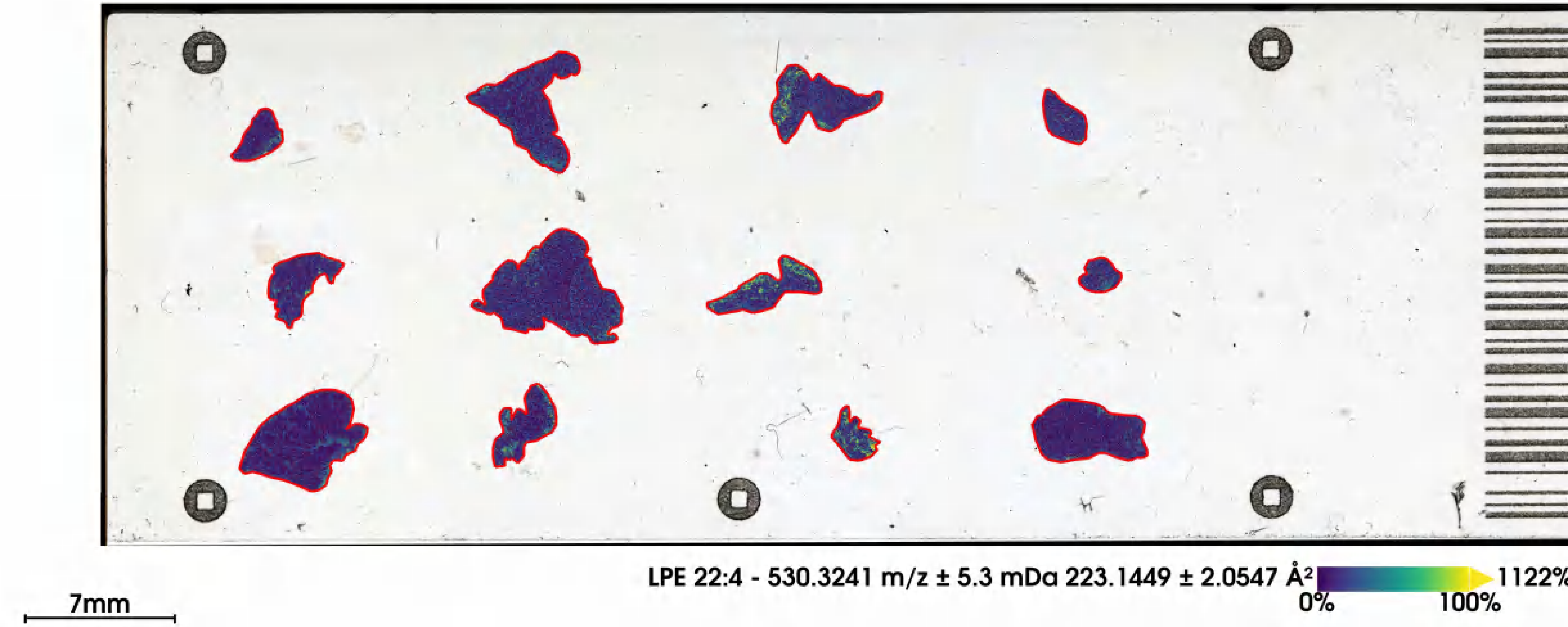
LPE 22:4 - 530.3241 m/z ± 5.3 mDa 223.1449 ± 2.0547 Å²
0%
100%
1122%
7mm

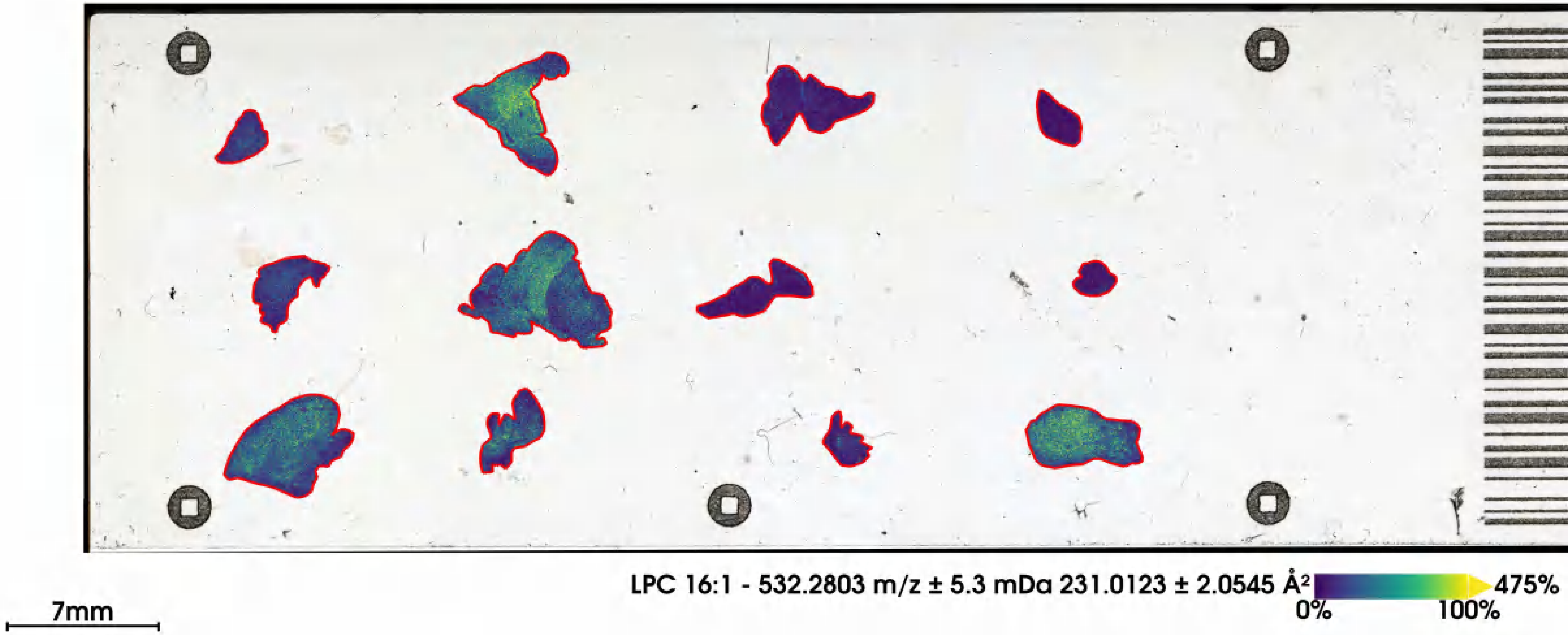

7mm
LPC 16:1 - 532.2803 m/z ± 5.3 mDa 231.0123 ± 2.0545 Å²
0%
100%
475%

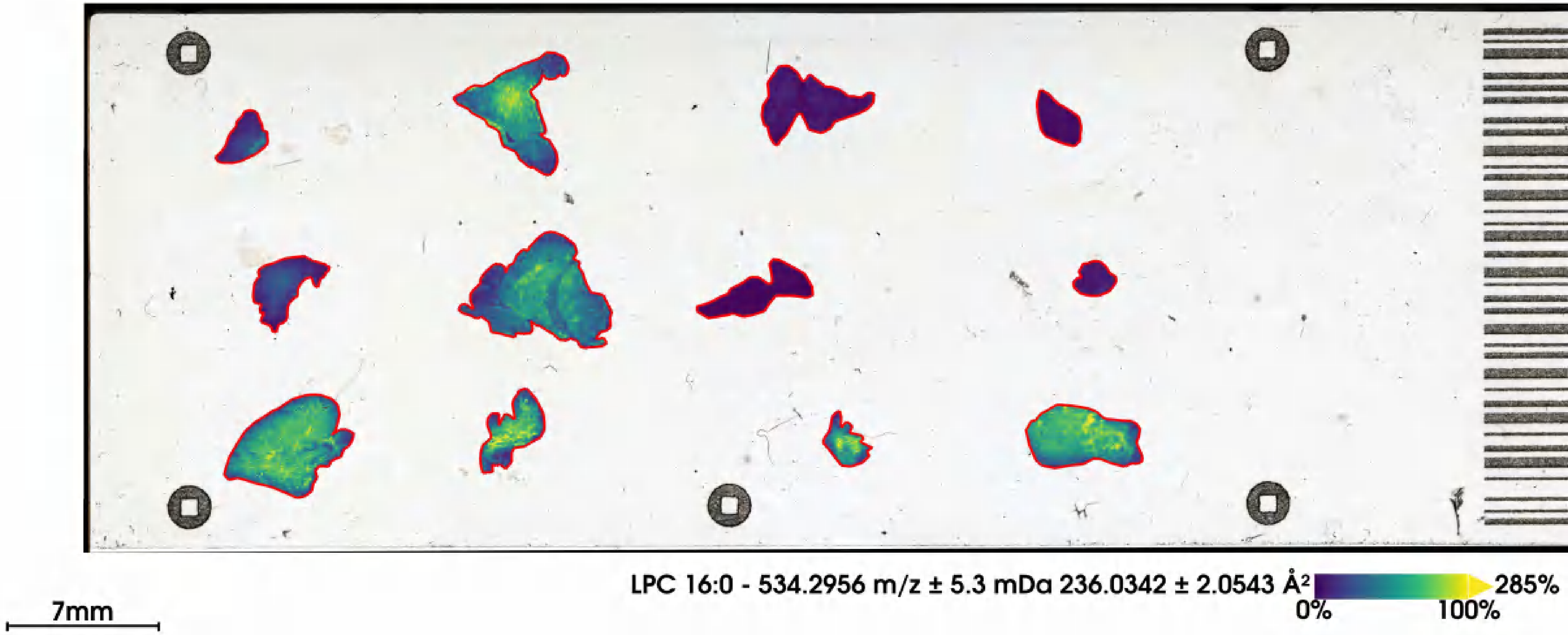
7mm
LPC 16:0 - 534.2956 m/z ± 5.3 mDa 236.0342 ± 2.0543 Å²
0%
100%
285%

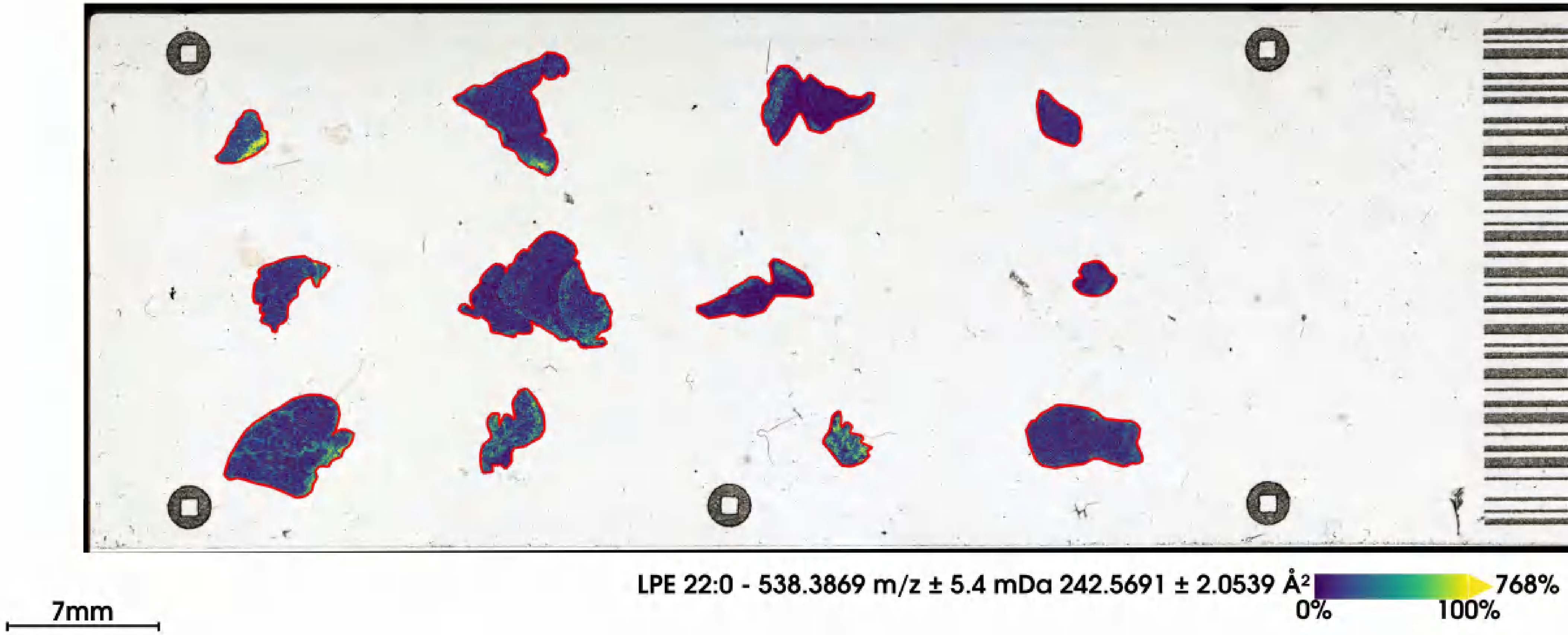
LPE 22:0 - 538.3869 m/z ± 5.4 mDa 242.5691 ± 2.0539 Å²
768%
0%
100%
7mm

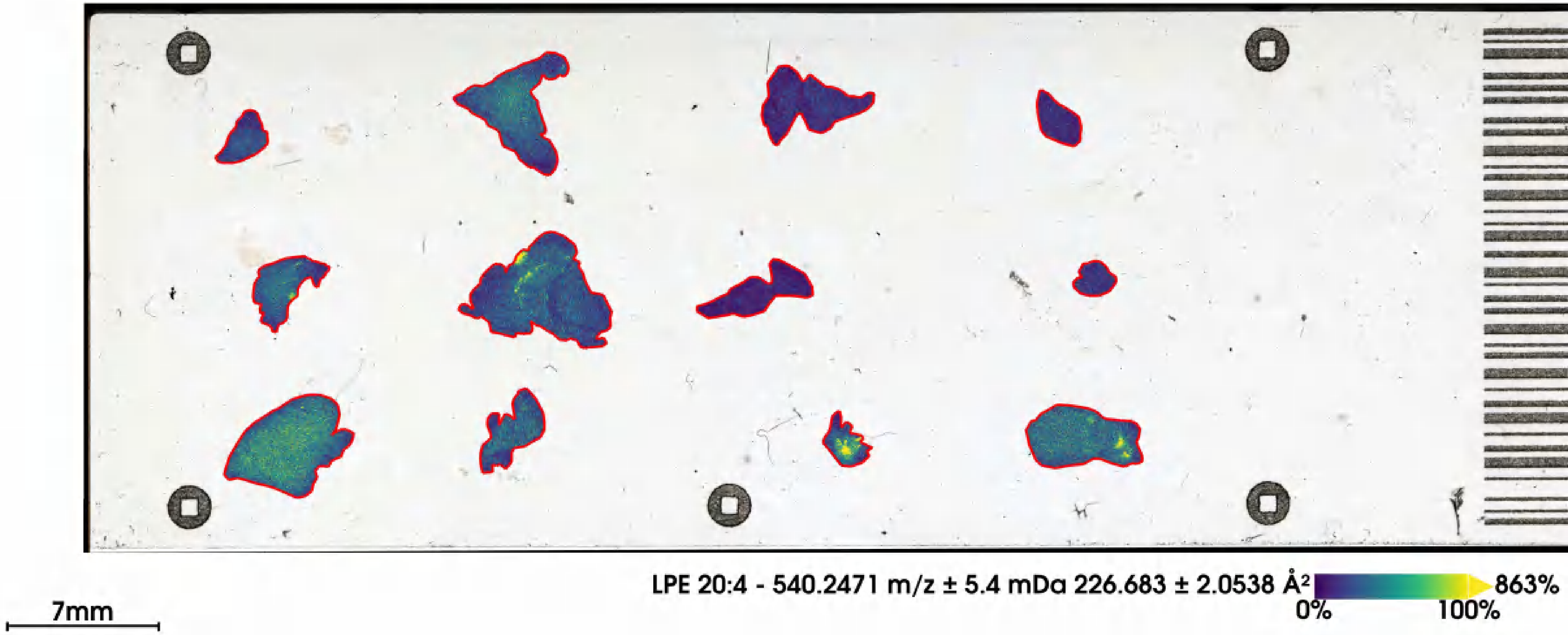

LPE 20:4 - 540.2471 m/z ± 5.4 mDa 226.683 ± 2.0538 Å²
0%
100%
863%
7mm

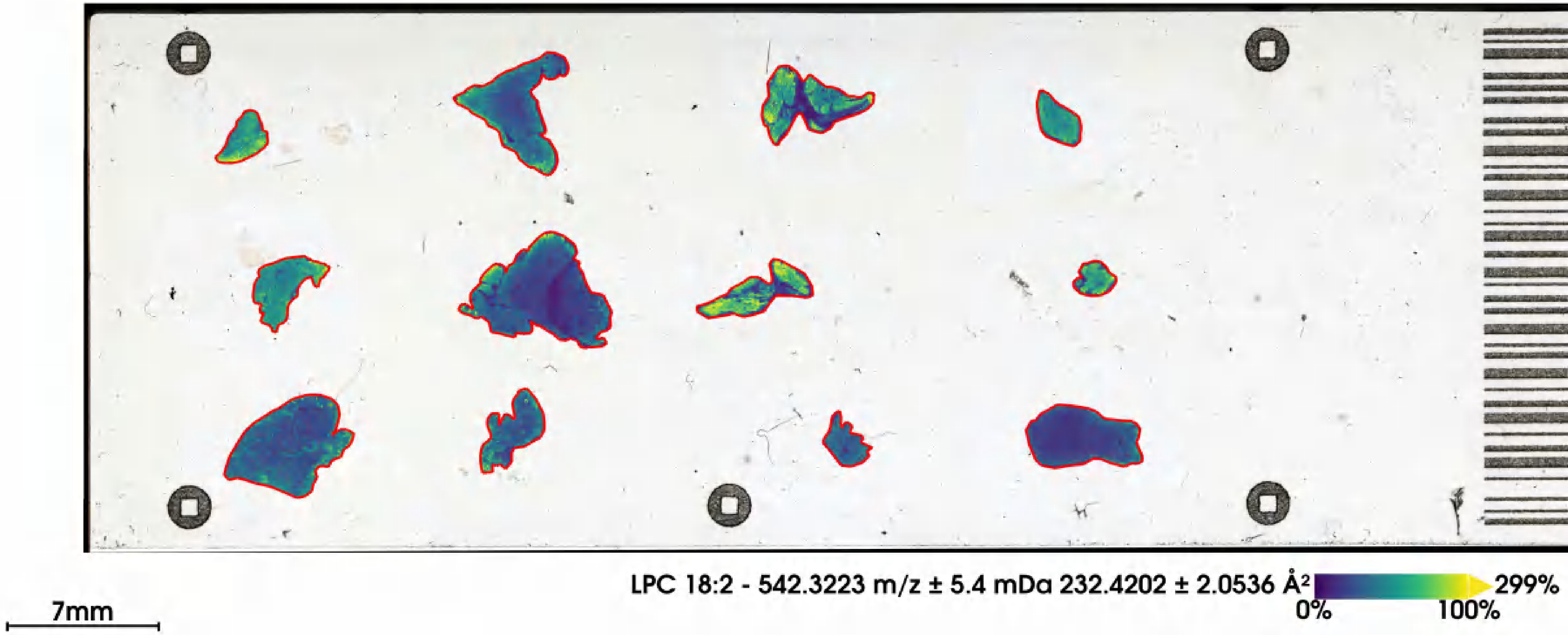
LPC 18:2 - 542.3223 m/z ± 5.4 mDa 232.4202 ± 2.0536 Å²
0%
100%
299%
7mm

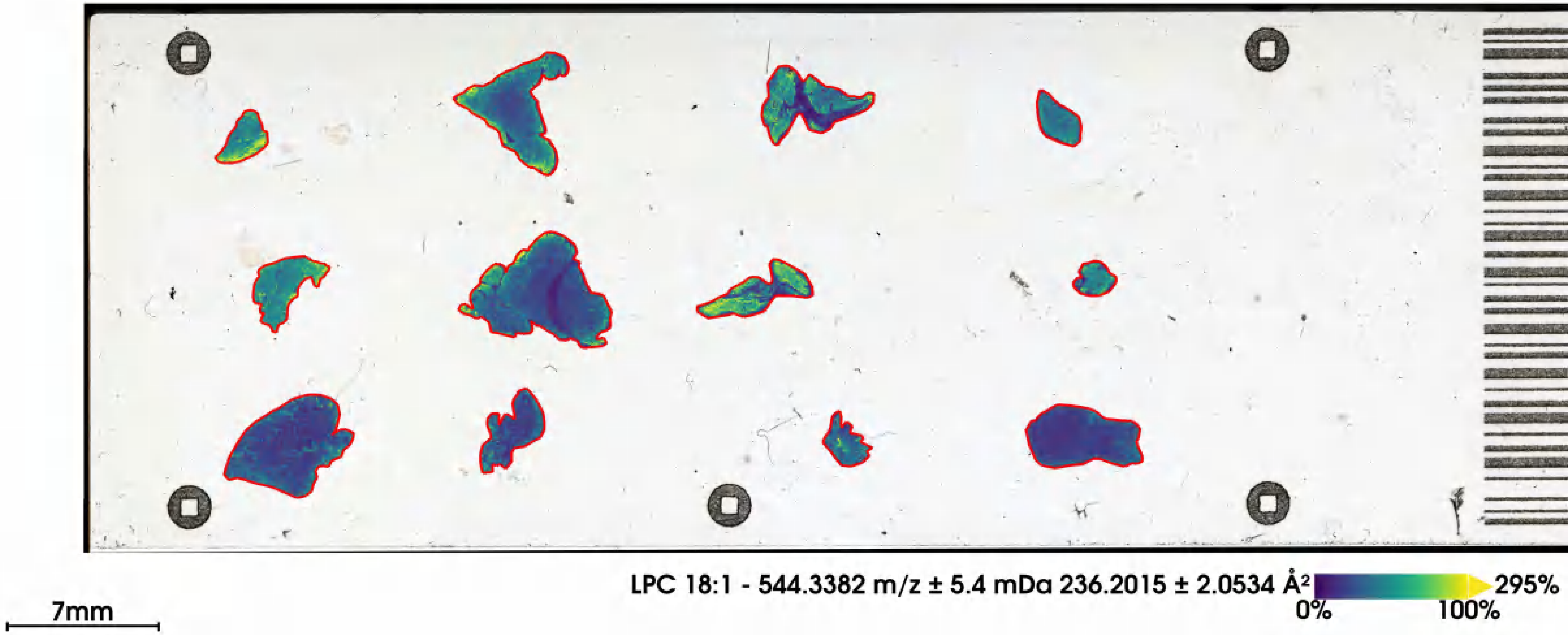
LPC 18:1 - 544.3382 m/z ± 5.4 mDa 236.2015 ± 2.0534 Å²
0%
100%
295%
7mm

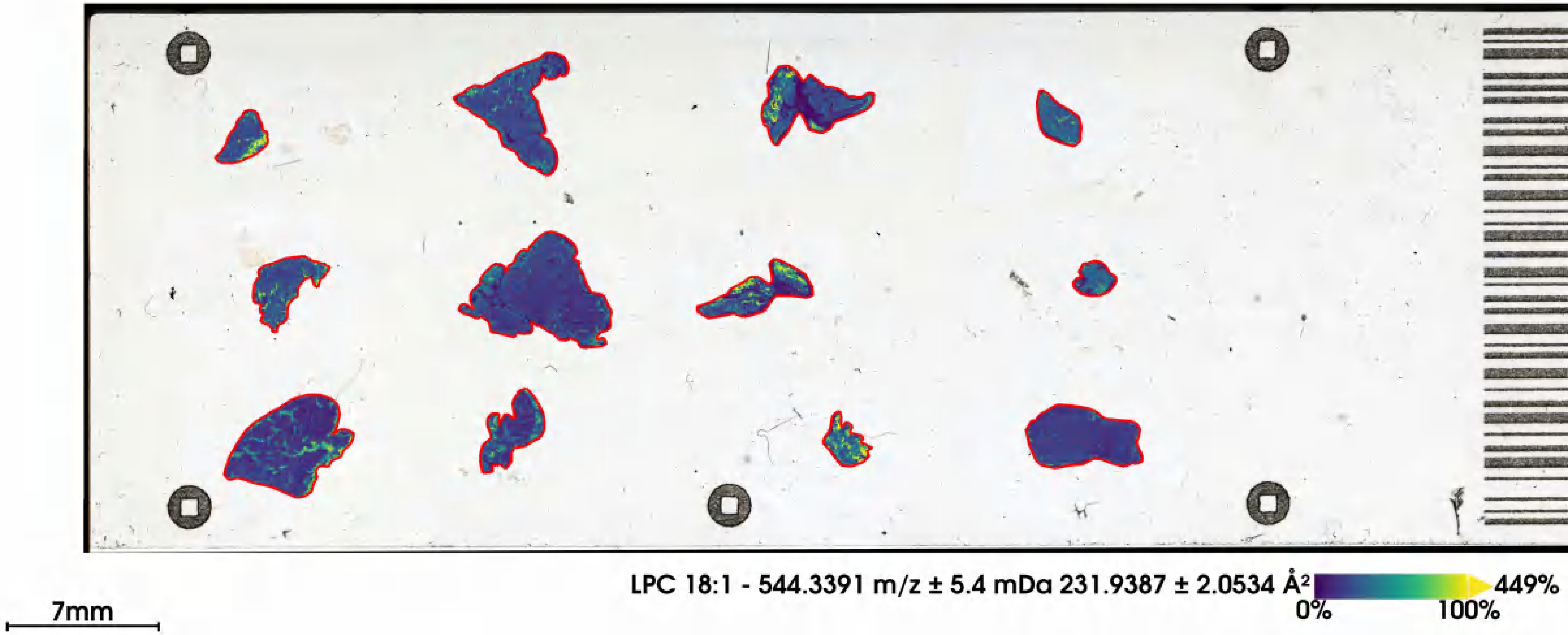

LPC 18:1 - 544.3391 m/z ± 5.4 mDa 231.9387 ± 2.0534 Å²
0%
100%
449%
7mm

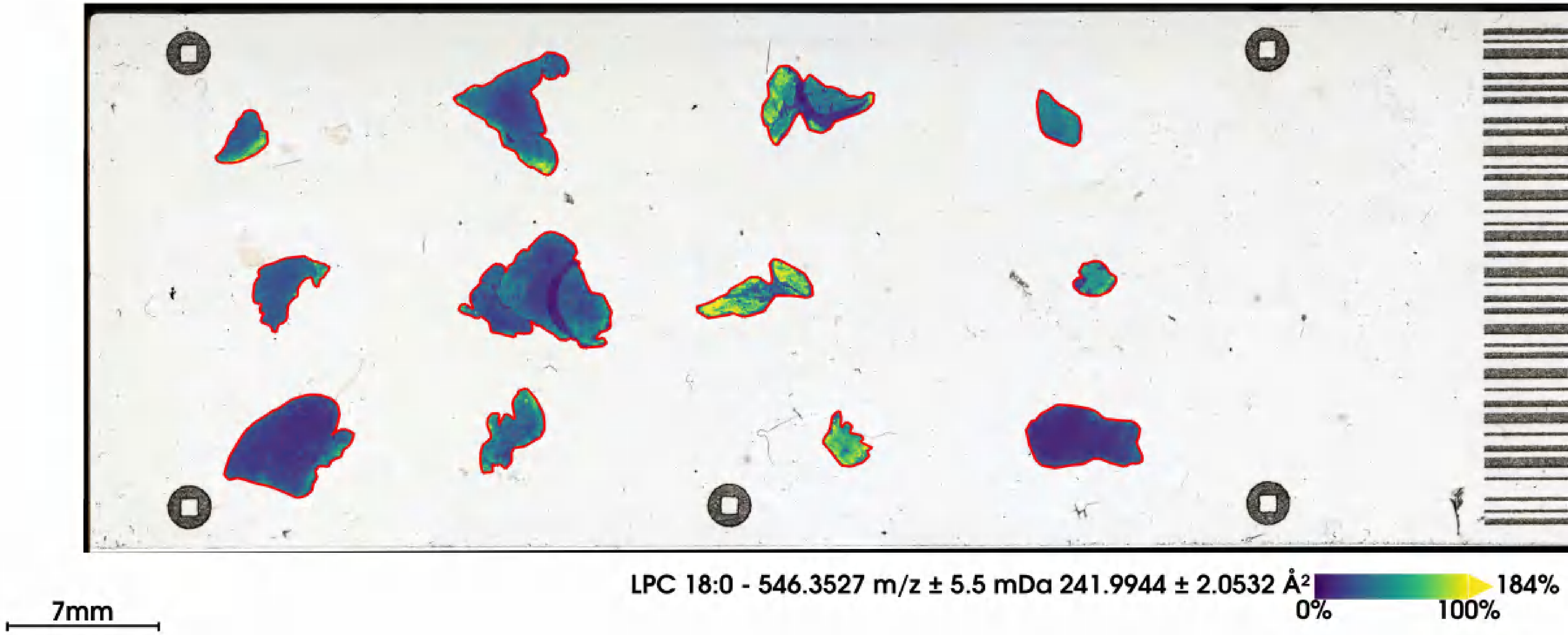
7mm
LPC 18:0 - 546.3527 m/z ± 5.5 mDa 241.9944 ± 2.0532 Å²
0%
100%
184%

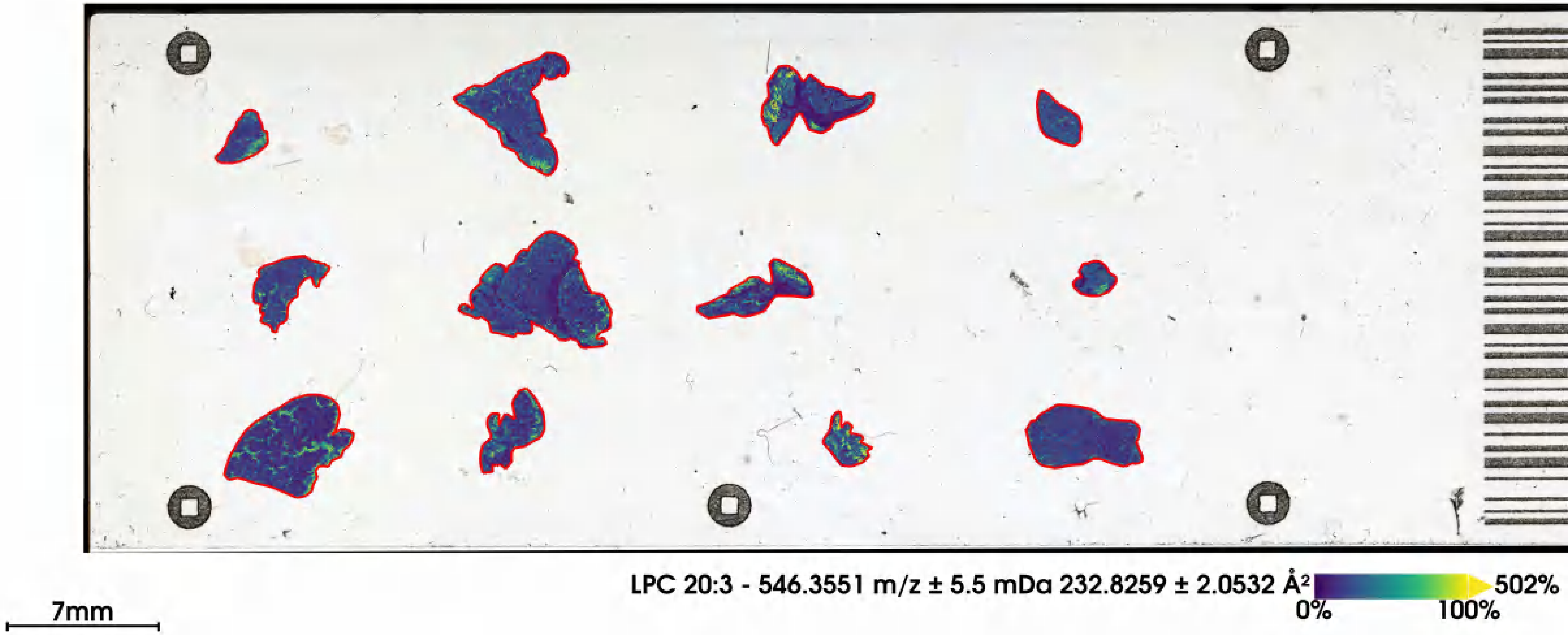

7mm
LPC 20:3 - 546.3551 m/z ± 5.5 mDa 232.8259 ± 2.0532 Å²
0%
100%
502%

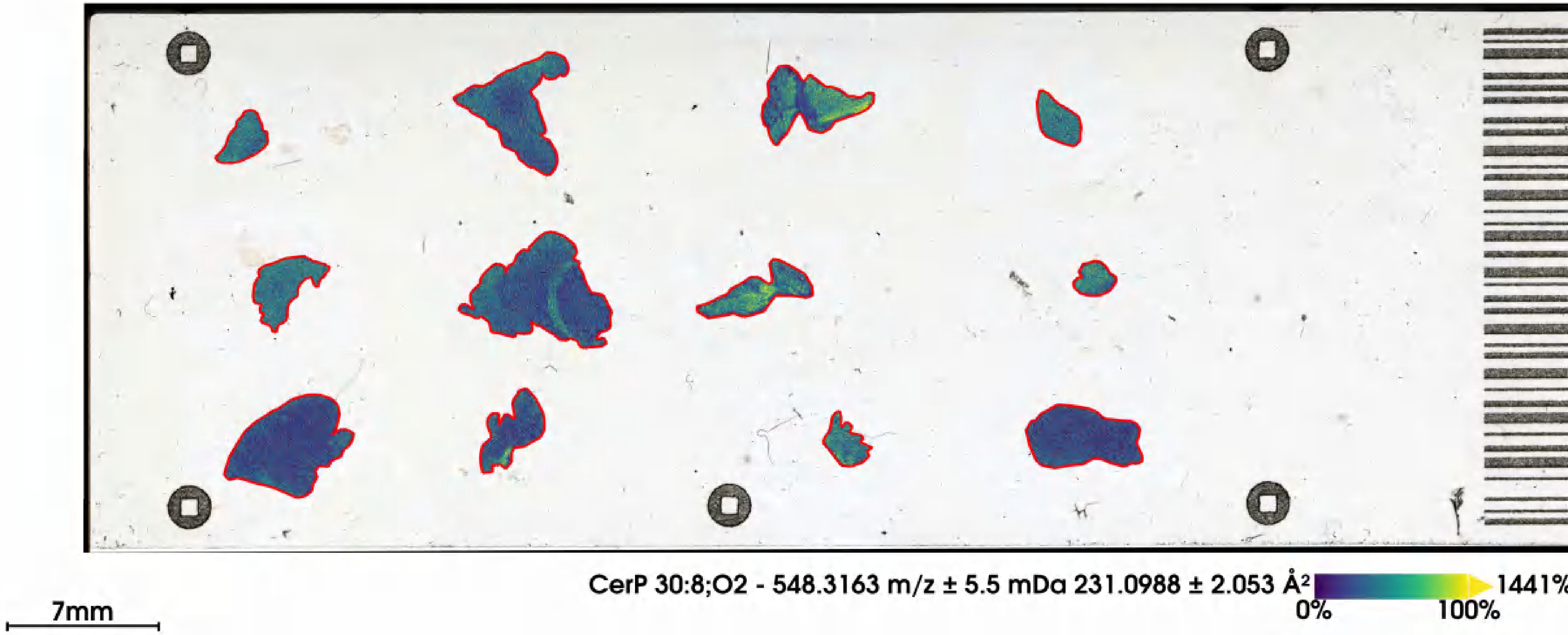
CerP 30:8;O2 - 548.3163 m/z ± 5.5 mDa 231.0988 ± 2.053 Å²
1441%
0%
100%
7mm

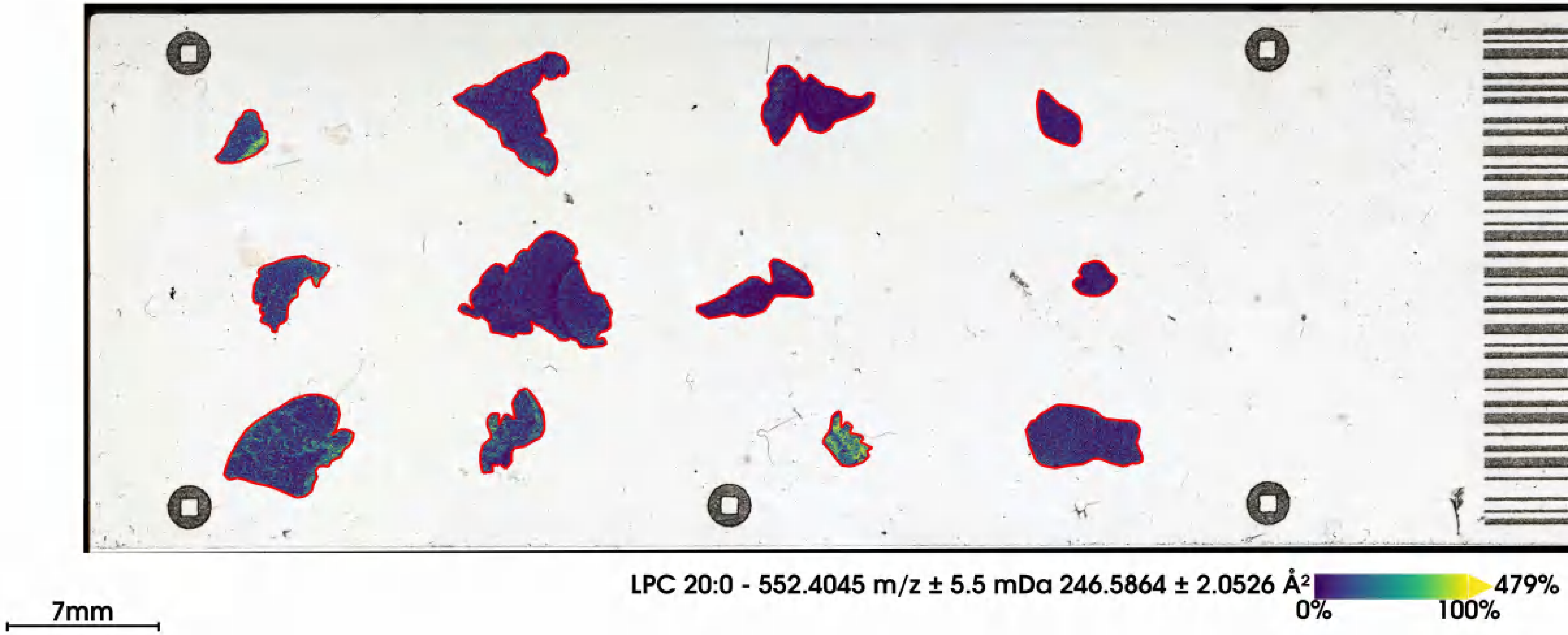

7mm
LPC 20:0 - 552.4045 m/z ± 5.5 mDa 246.5864 ± 2.0526 Å²
0%
100%
479%

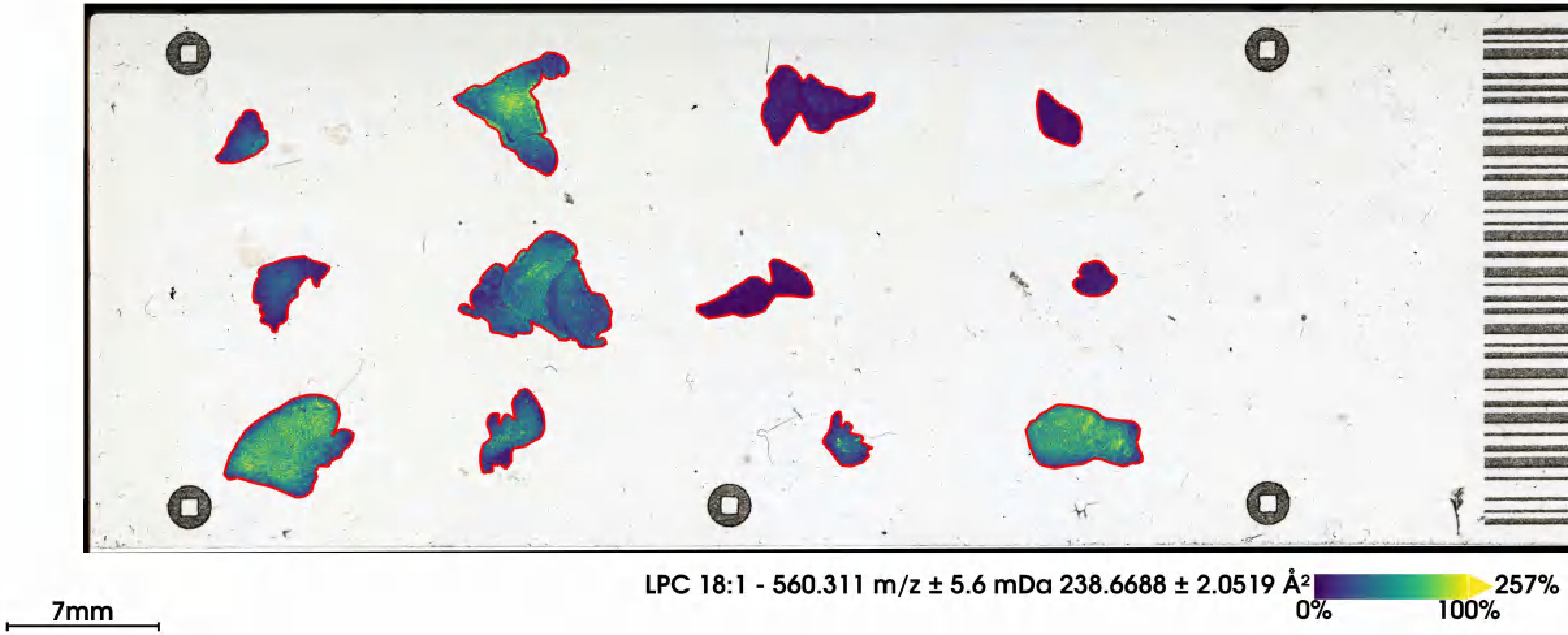
LPC 18:1 - 560.311 m/z ± 5.6 mDa 238.6688 ± 2.0519 Å²
0%
100%
257%
7mm

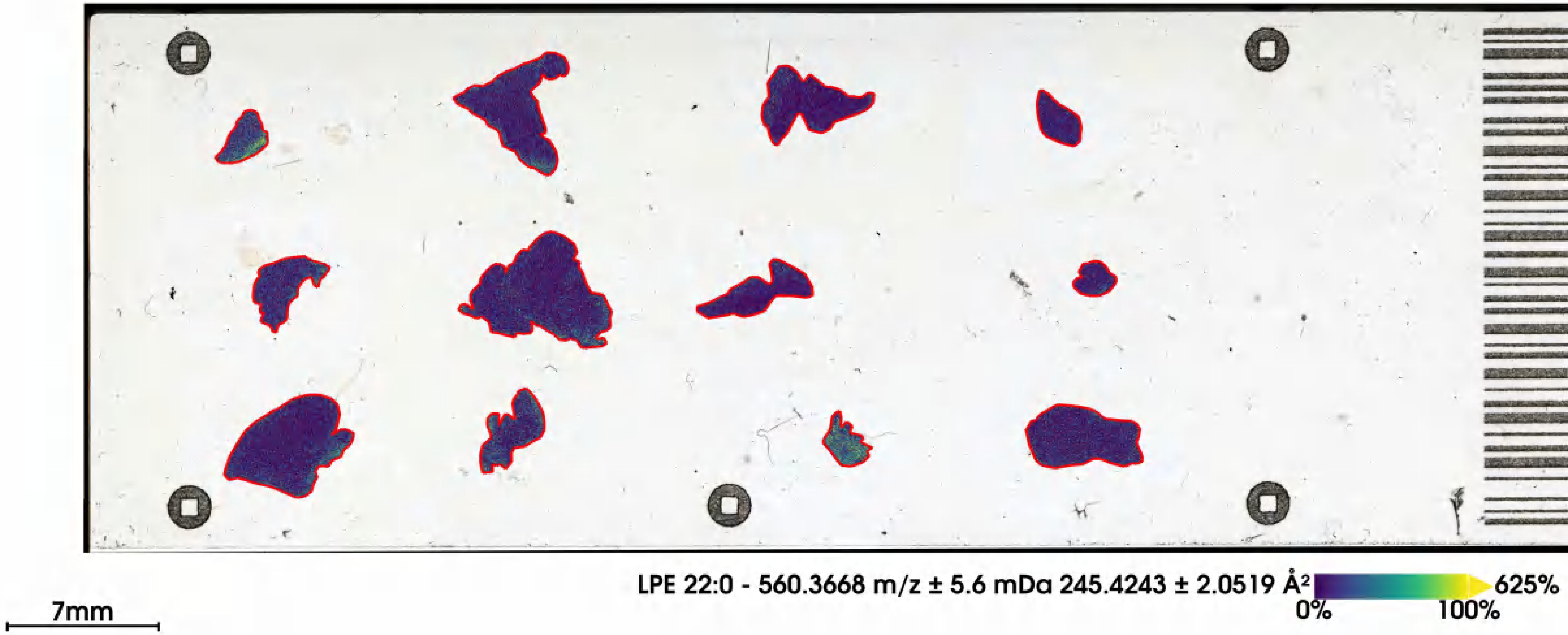
7mm
LPE 22:0 - 560.3668 m/z ± 5.6 mDa 245.4243 ± 2.0519 Å²
0%
100%
625%

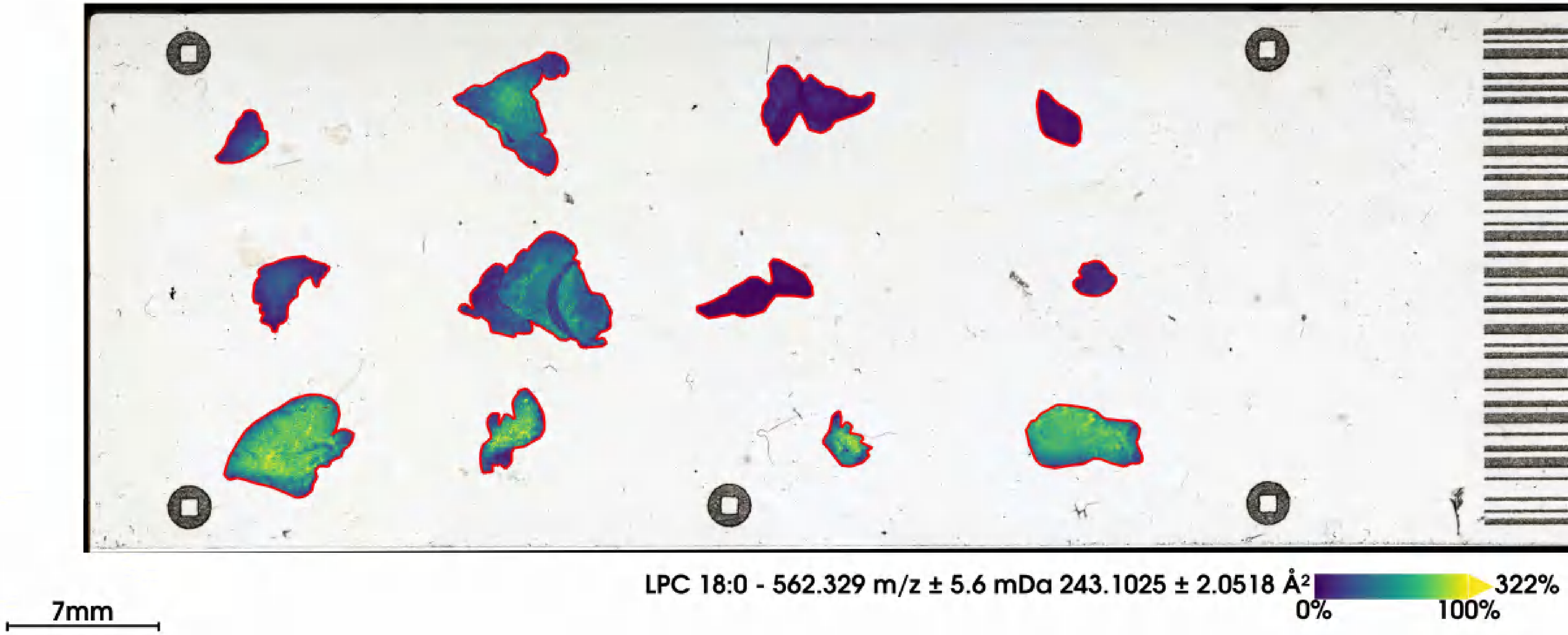
LPC 18:0 - 562.329 m/z ± 5.6 mDa 243.1025 ± 2.0518 Å²
322%
0%
100%
7mm

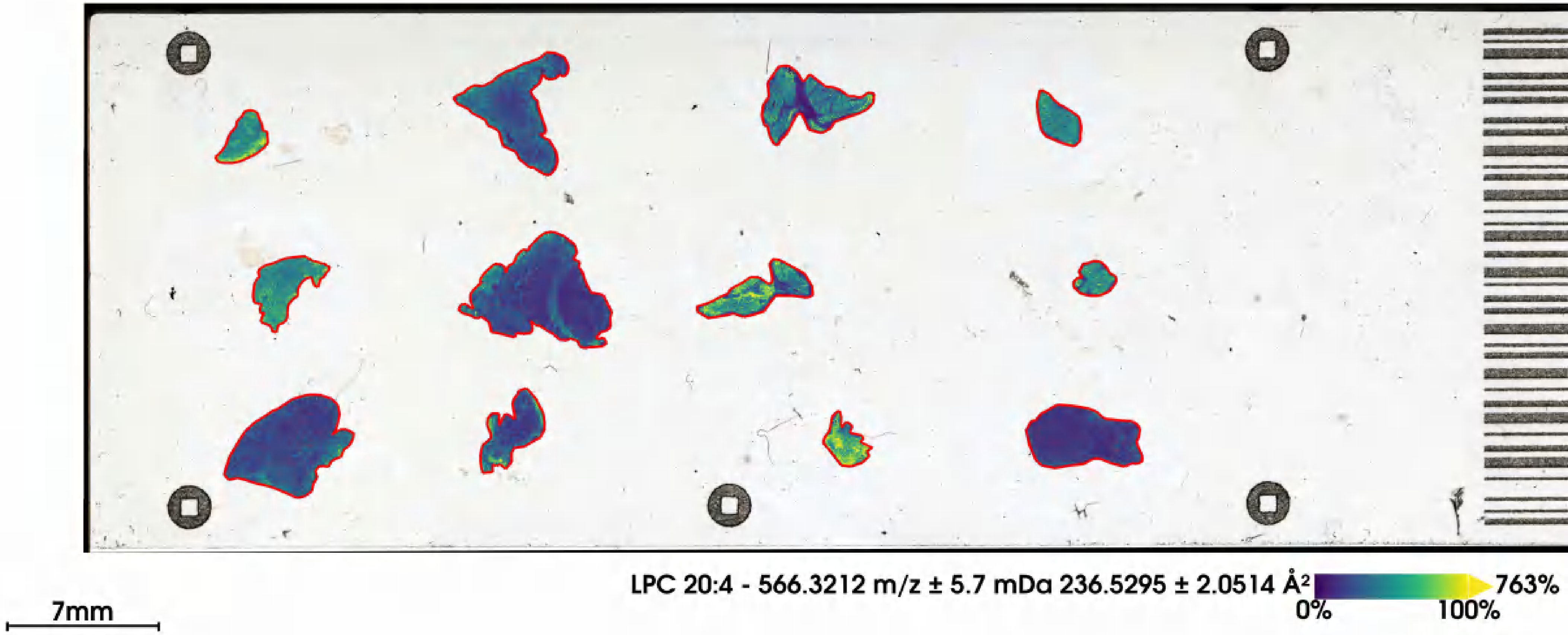
LPC 20:4 - 566.3212 m/z ± 5.7 mDa 236.5295 ± 2.0514 Å²
0%
100%
763%
7mm

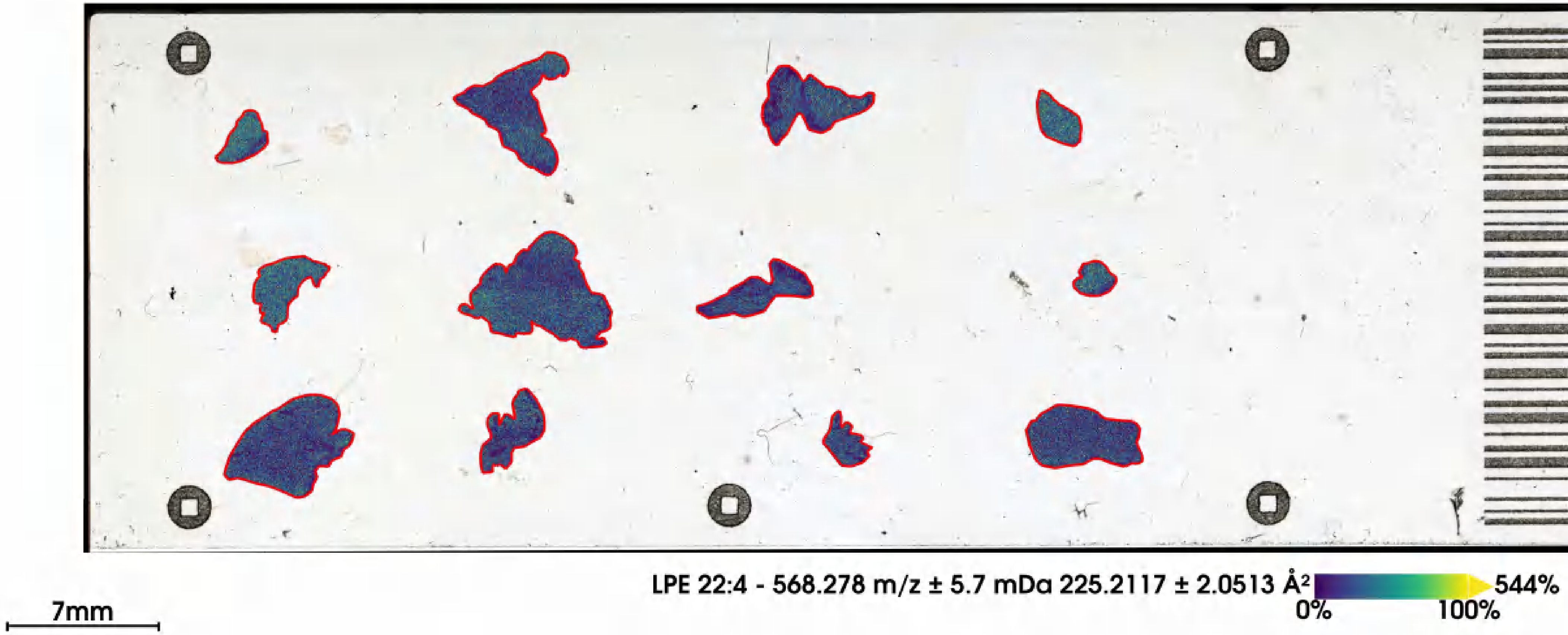

LPE 22:4 - 568.278 m/z ± 5.7 mDa 225.2117 ± 2.0513 Å²
544%
0%
100%
7mm

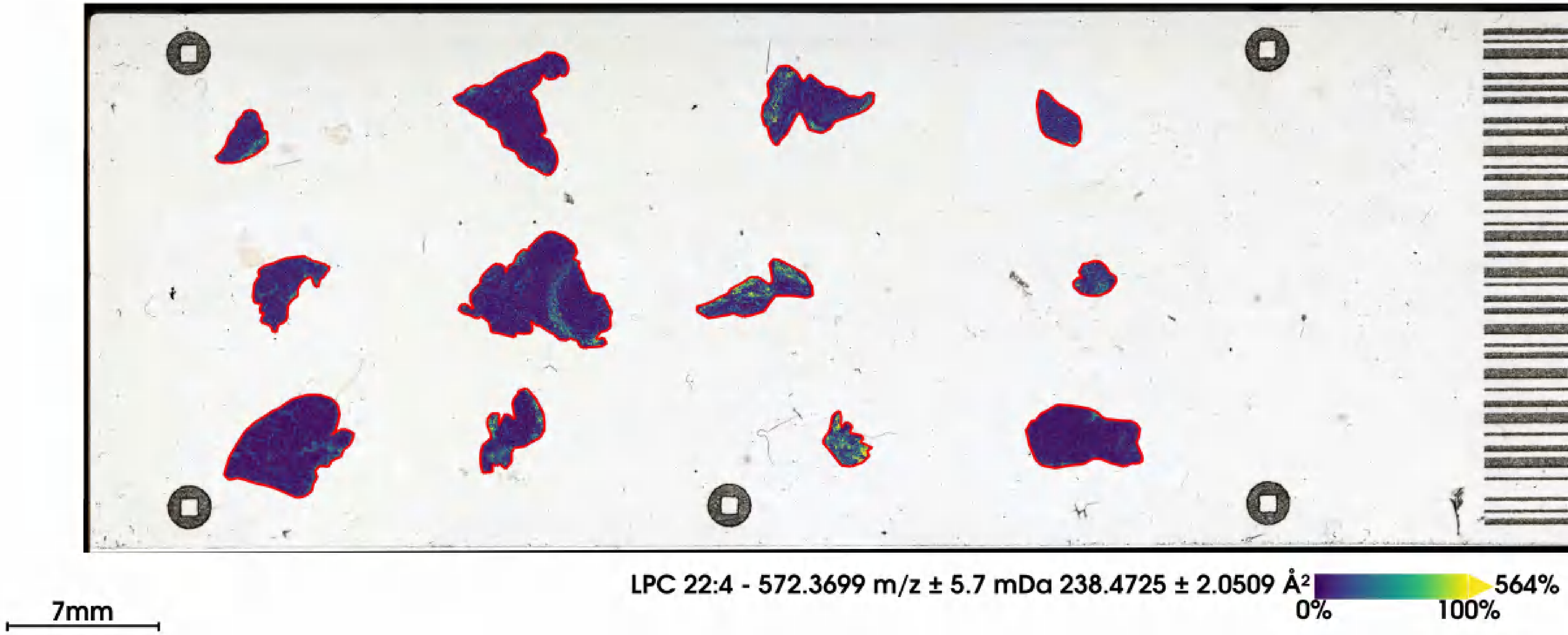

7mm
LPC 22:4 - 572.3699 m/z ± 5.7 mDa 238.4725 ± 2.0509 Å²
0%
100%
564%

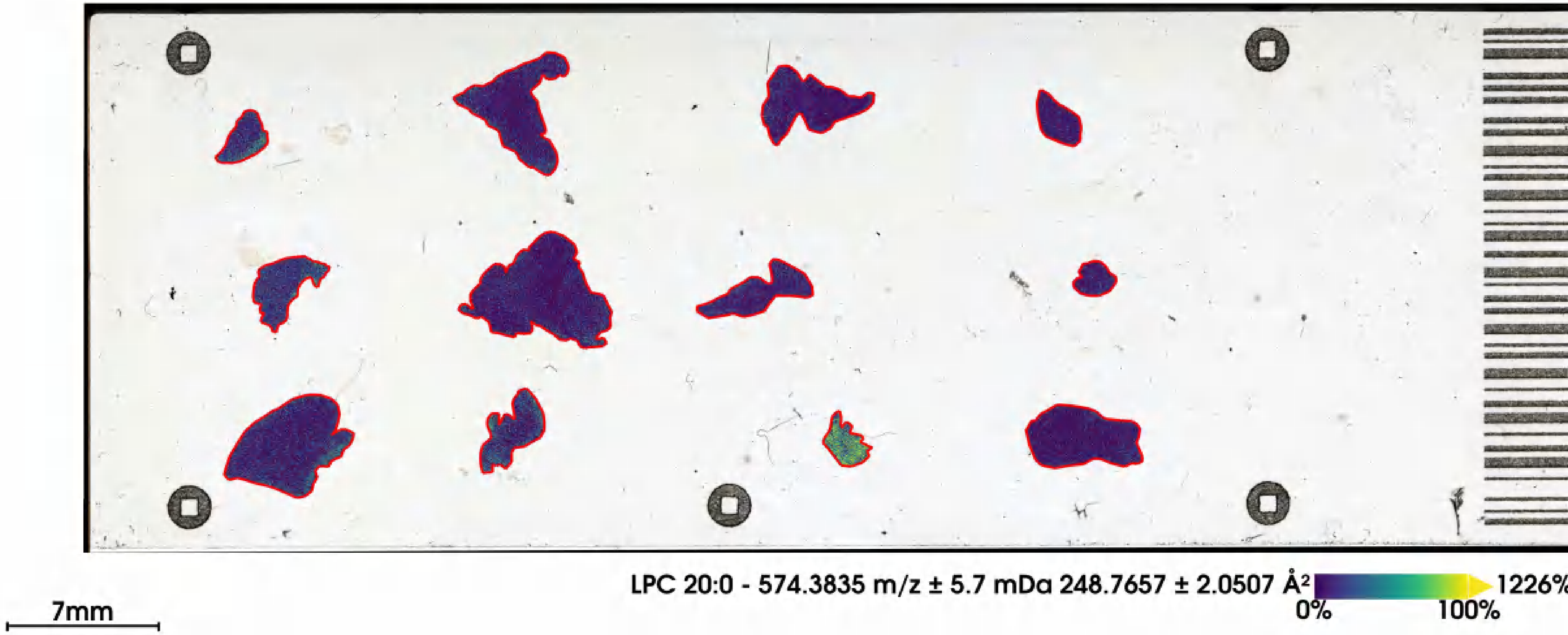

LPC 20:0 - 574.3835 m/z ± 5.7 mDa 248.7657 ± 2.0507 Å²
1226%
0%
100%
7mm

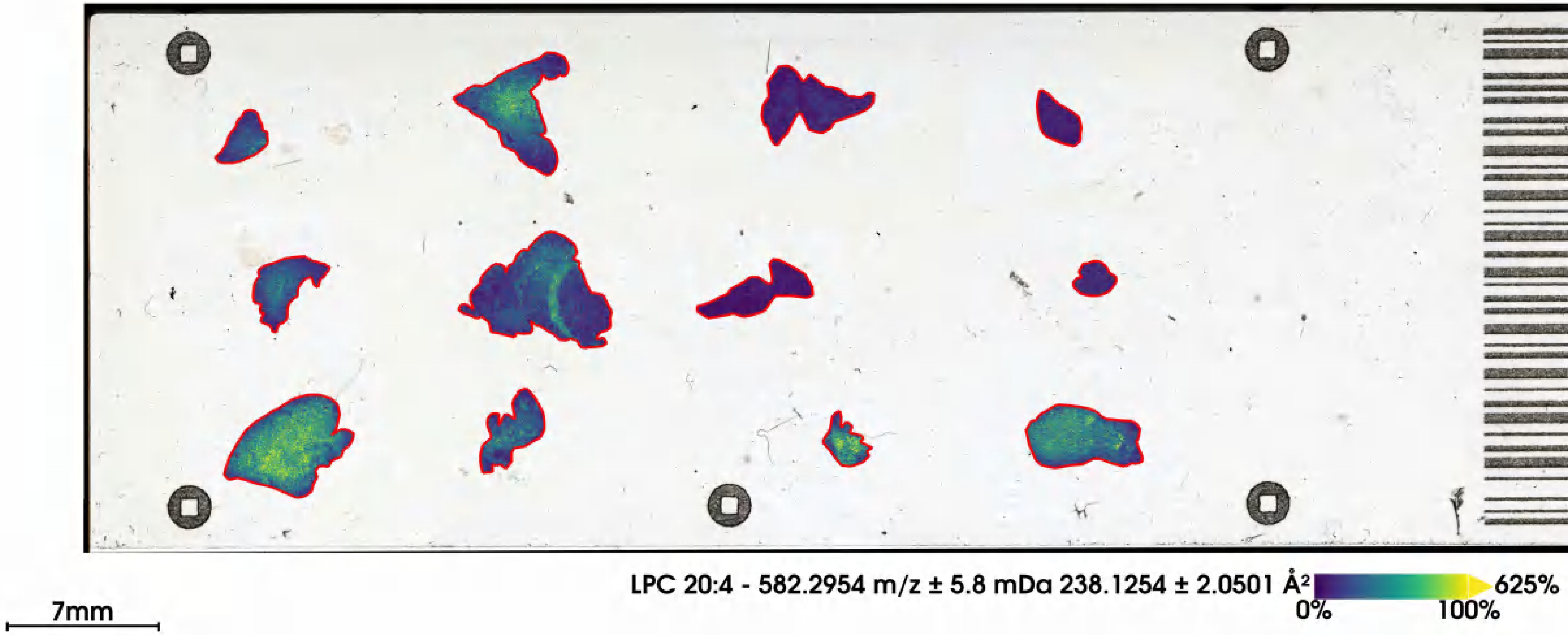
7mm
LPC 20:4 - 582.2954 m/z ± 5.8 mDa 238.1254 ± 2.0501 Å²
0%
100%
625%

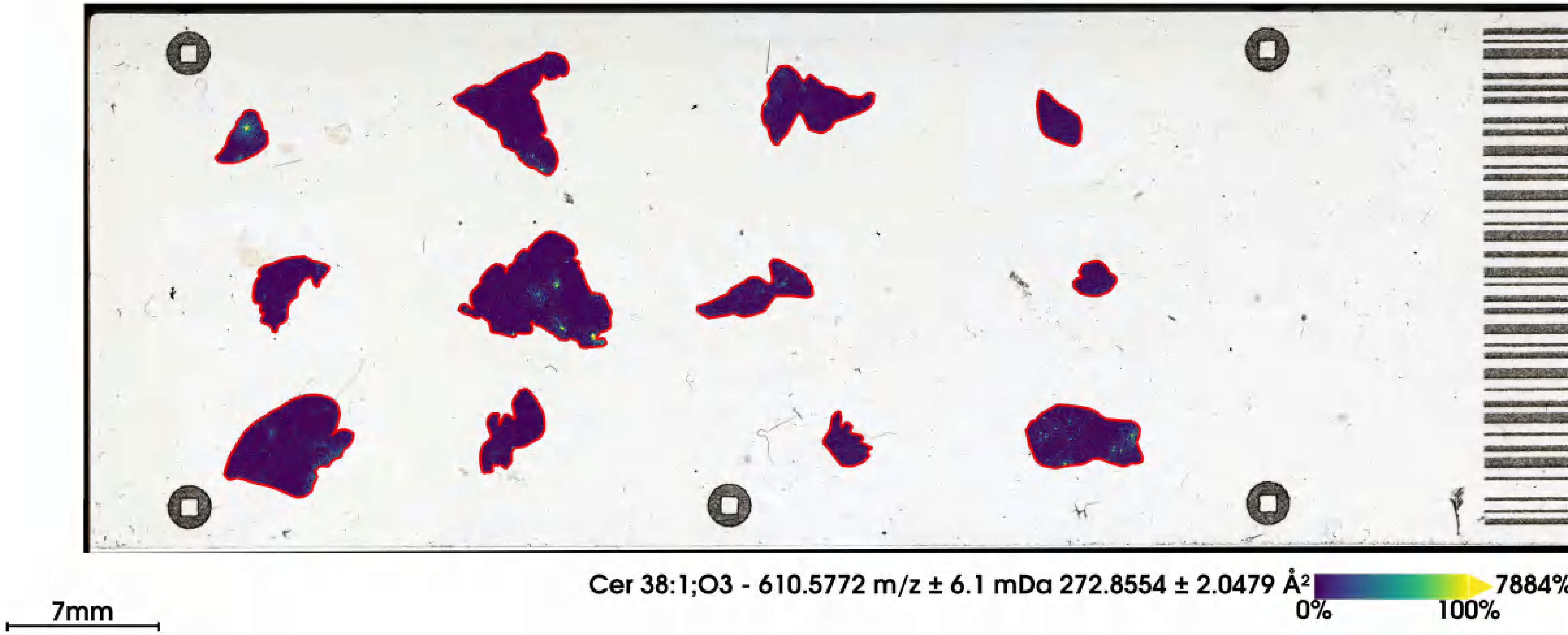
7mm
Cer 38:1;O3 - 610.5772 m/z ± 6.1 mDa 272.8554 ± 2.0479 Å²
7884%
0%
100%

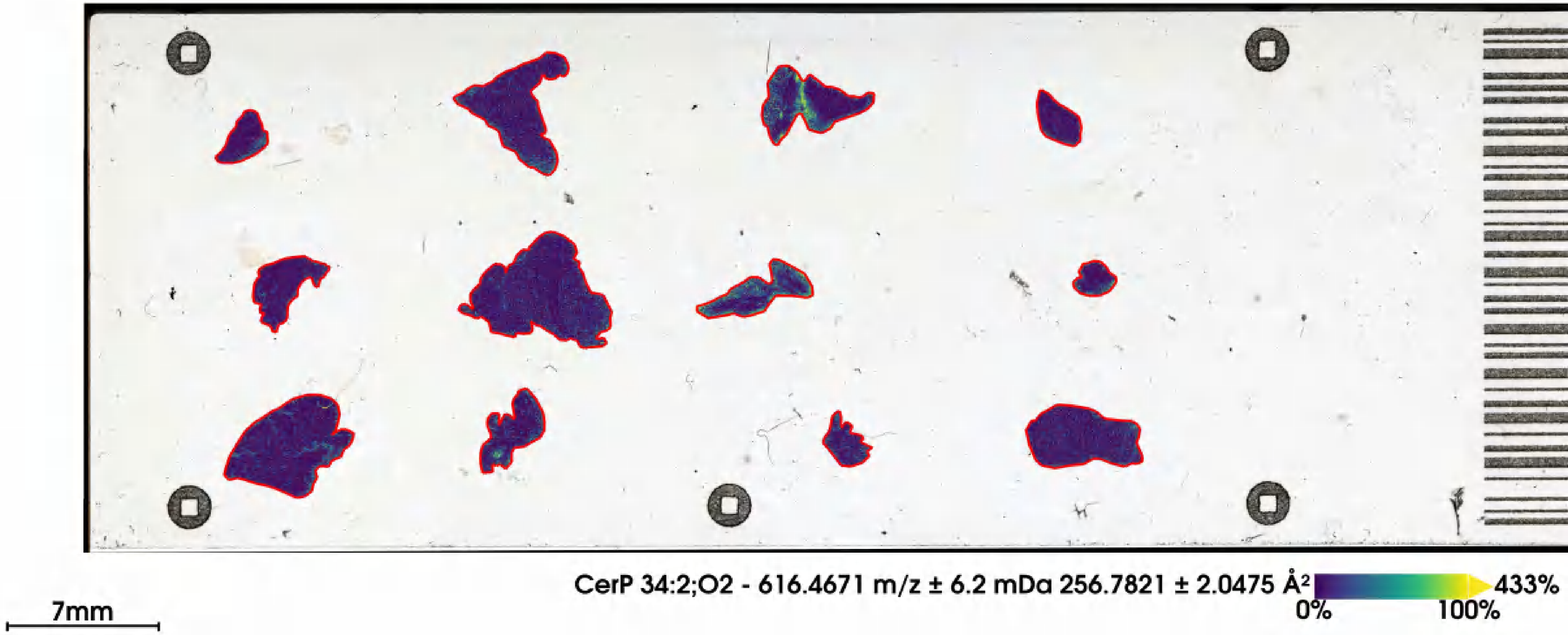

7mm
CerP 34:2;O2 - 616.4671 m/z ± 6.2 mDa 256.7821 ± 2.0475 Å²
0%
100%
433%

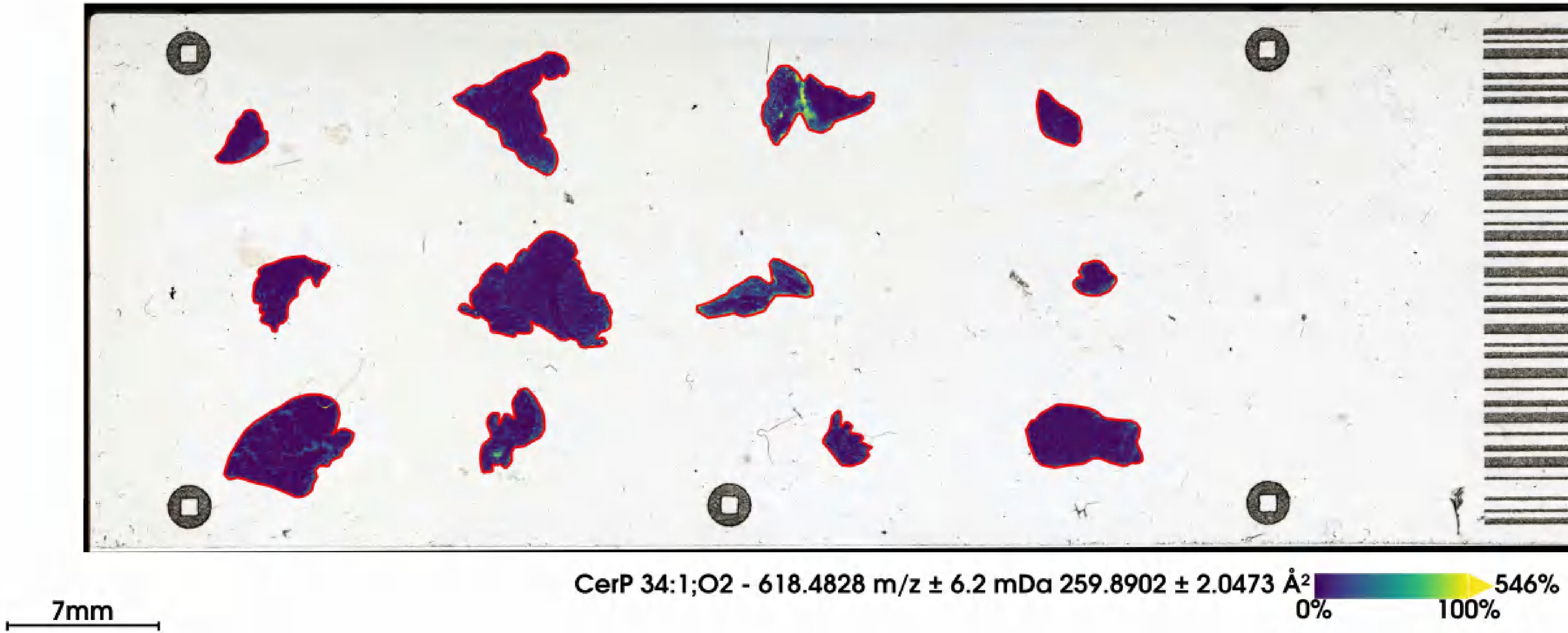
CerP 34:1;O2 - 618.4828 m/z ± 6.2 mDa 259.8902 ± 2.0473 Å²
0%
100%
546%
7mm

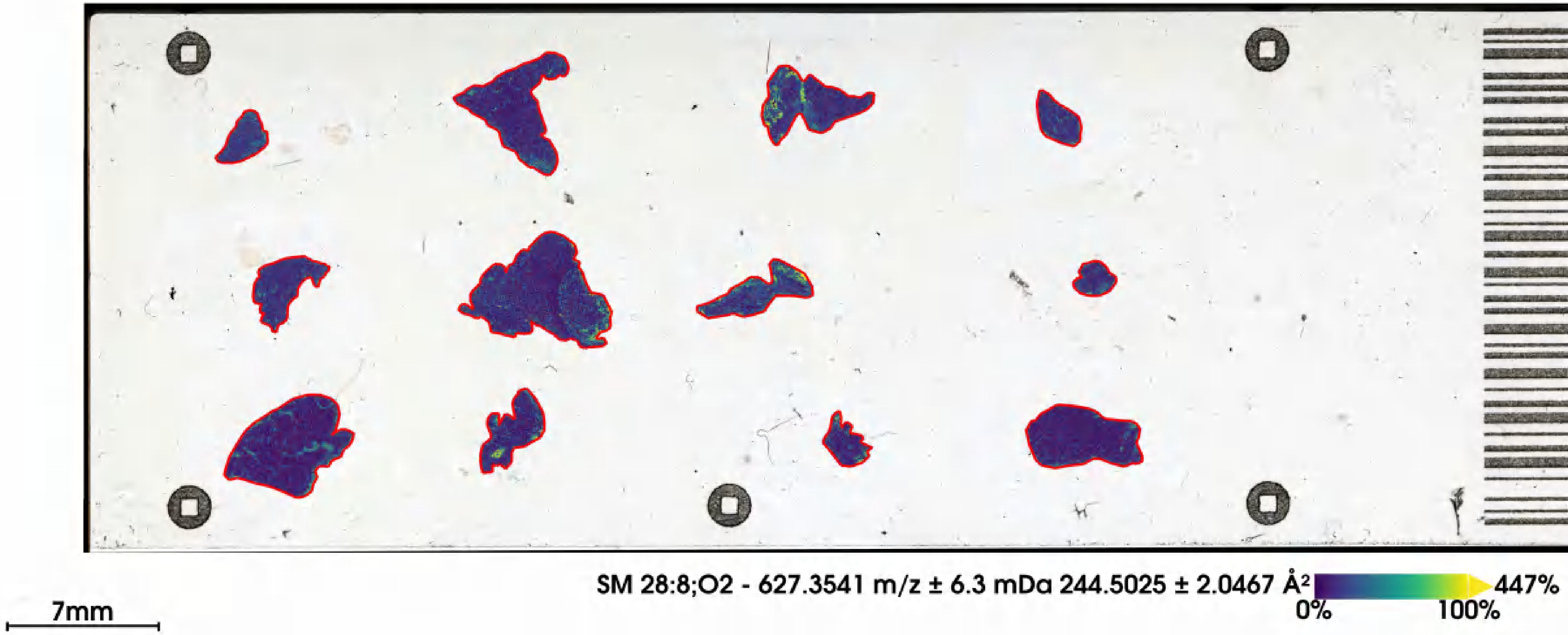
SM 28:8;O2 - 627.3541 m/z ± 6.3 mDa 244.5025 ± 2.0467 Å²
0%
100%
447%
7mm

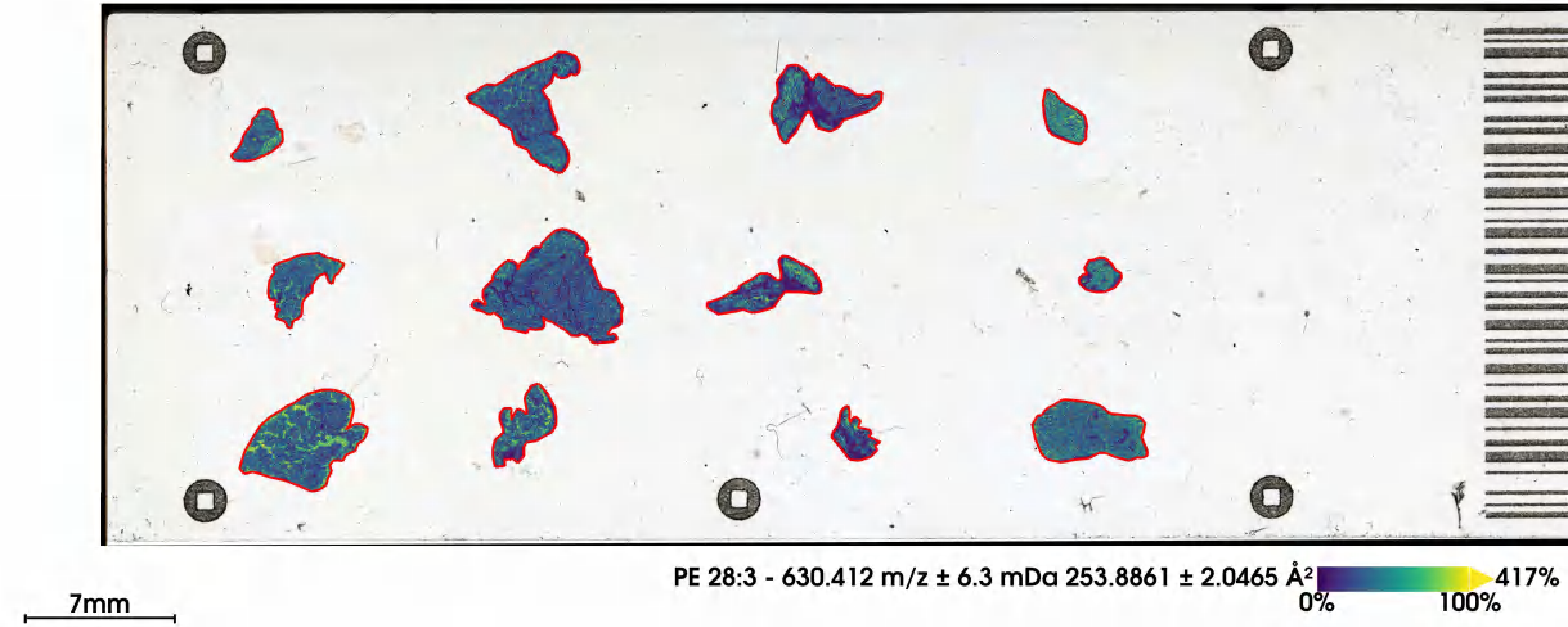
PE 28:3 - 630.412 m/z ± 6.3 mDa 253.8861 ± 2.0465 Å²
0%
100%
417%
7mm

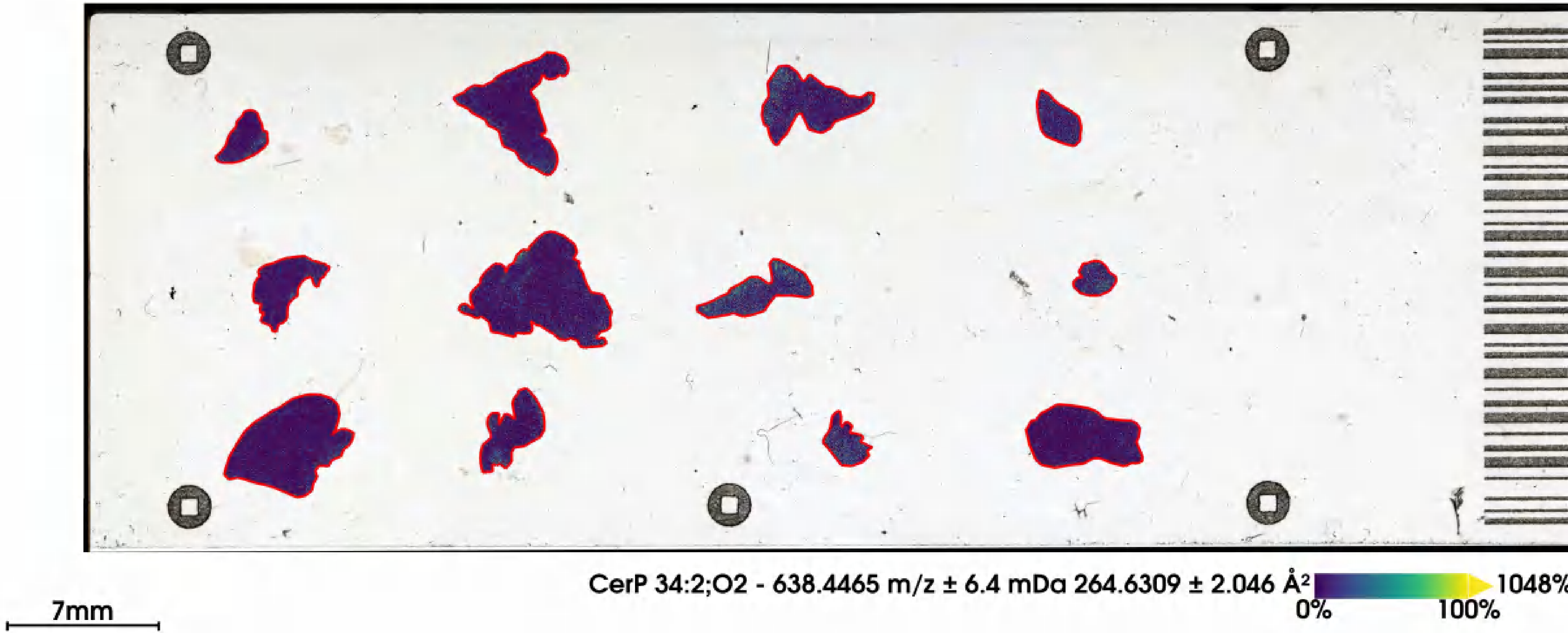
7mm
CerP 34:2;O2 - 638.4465 m/z ± 6.4 mDa 264.6309 ± 2.046 Å²
0%
100%
1048%

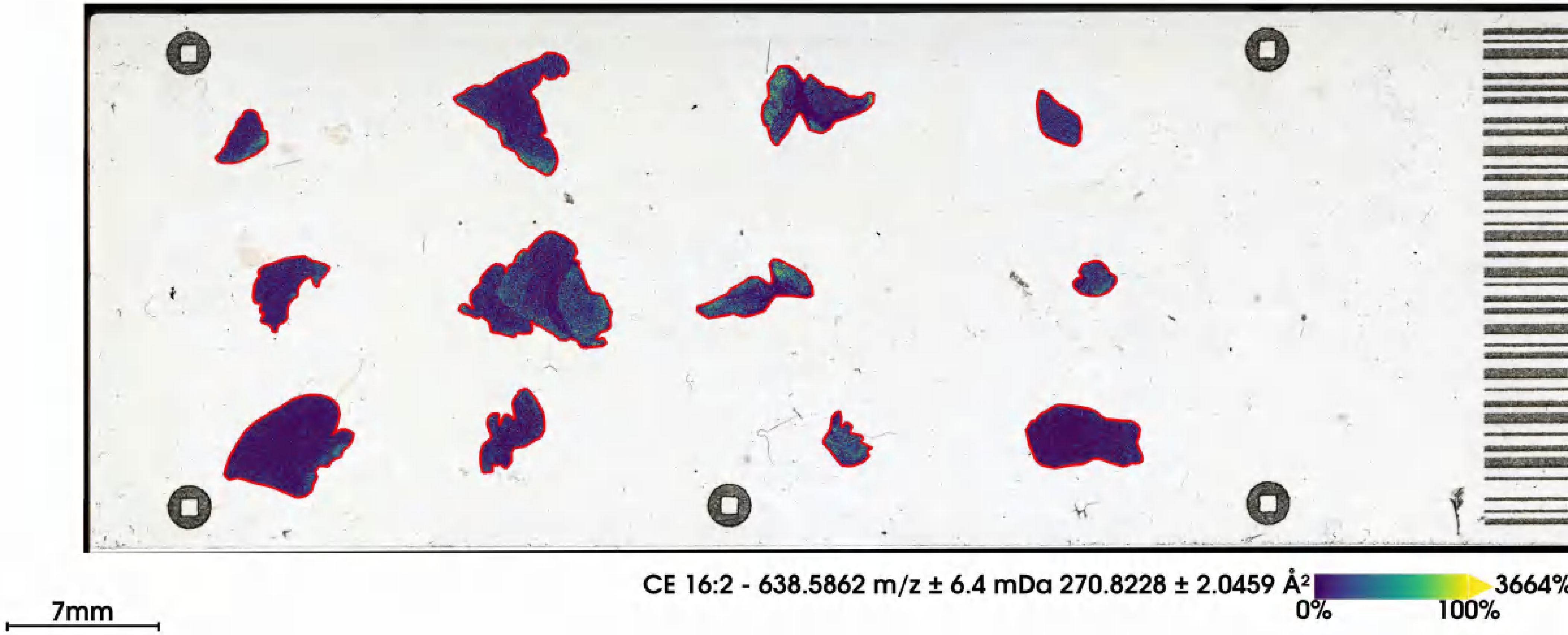

CE 16:2 - 638.5862 m/z ± 6.4 mDa 270.8228 ± 2.0459 Å²
0%
100%
3664%
7mm

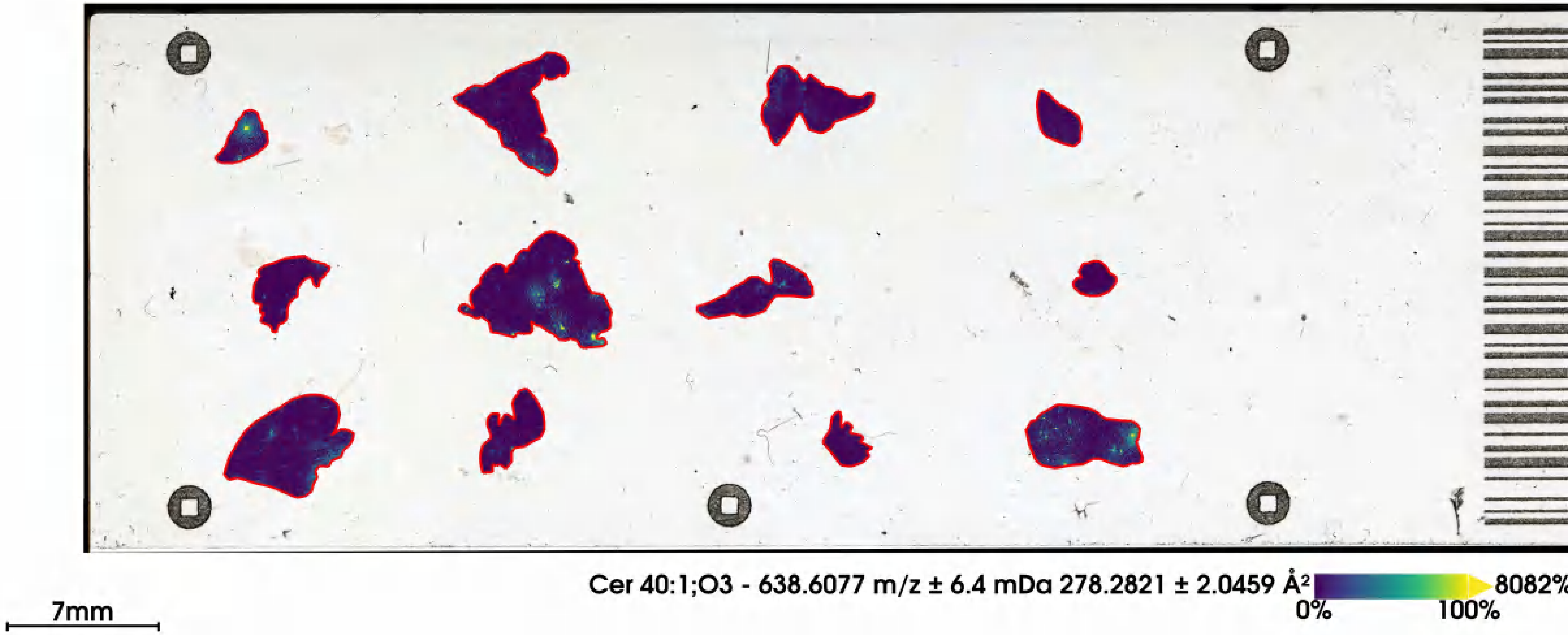

Cer 40:1;O3 - 638.6077 m/z ± 6.4 mDa 278.2821 ± 2.0459 Å²
0%
100%
8082%
7mm

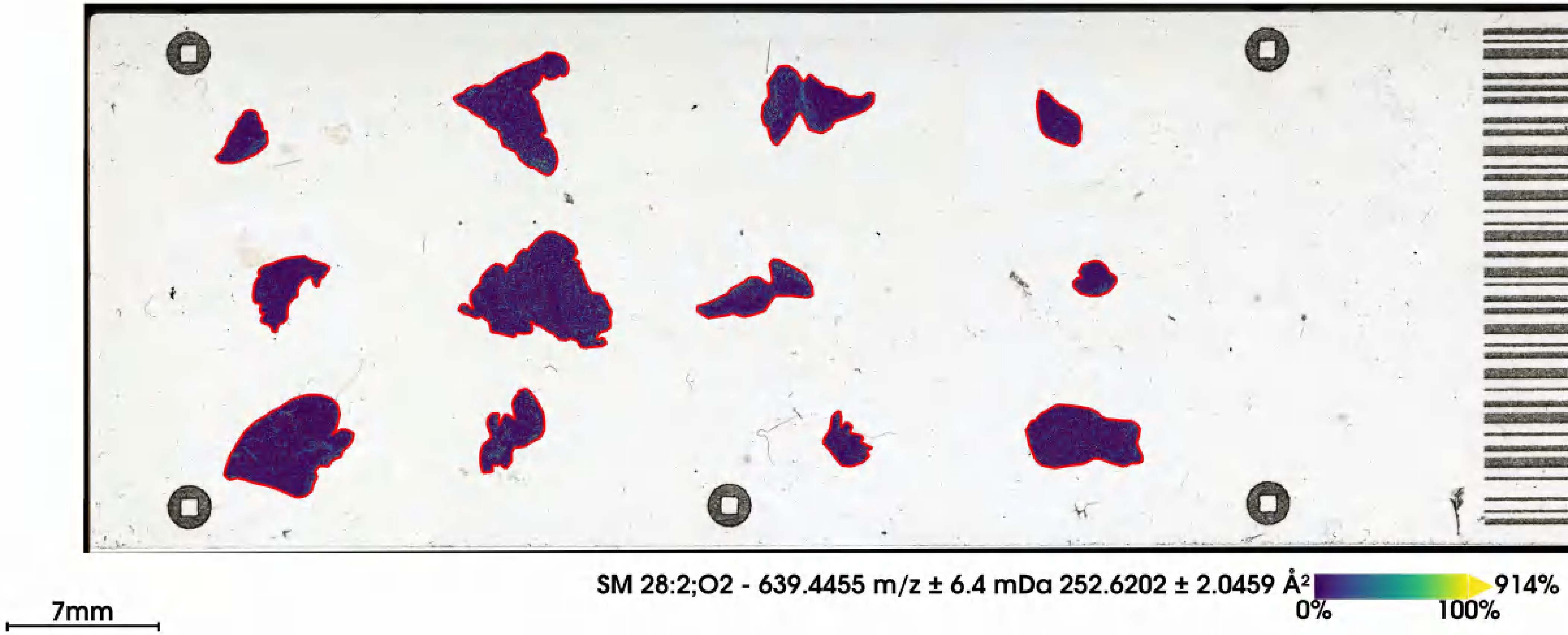
SM 28:2;O2 - 639.4455 m/z ± 6.4 mDa 252.6202 ± 2.0459 Å²
914%
0%
100%
7mm

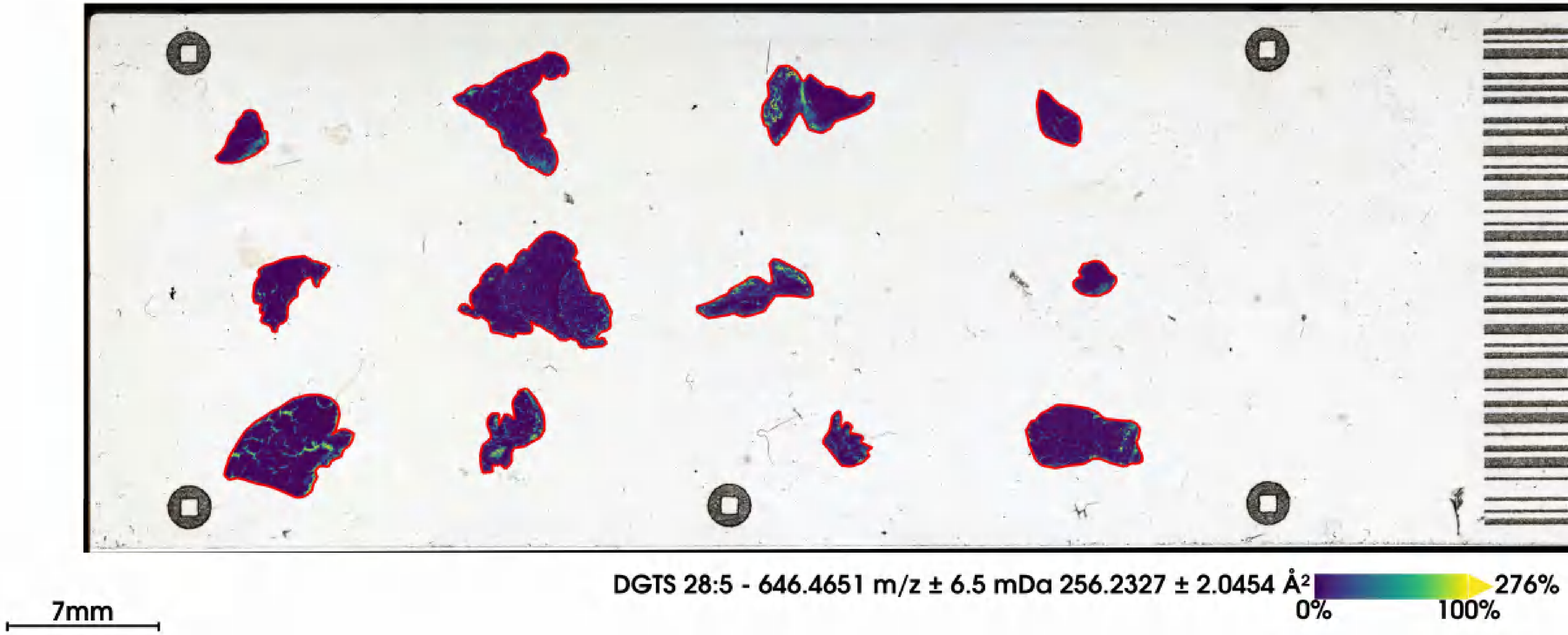
DGTS 28:5 - 646.4651 m/z ± 6.5 mDa 256.2327 ± 2.0454 Å²
0%
100%
276%
7mm

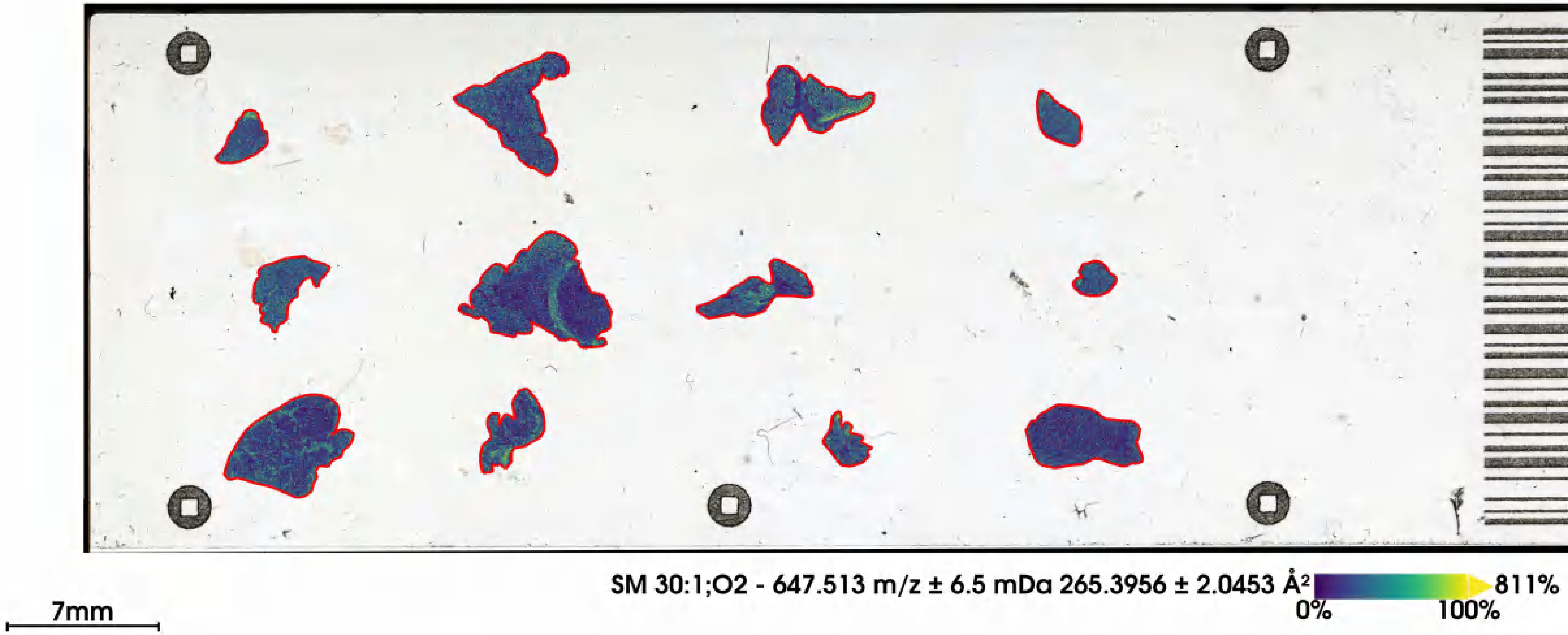
SM 30:1;O2 - 647.513 m/z ± 6.5 mDa 265.3956 ± 2.0453 Å²
0%
100%
811%
7mm

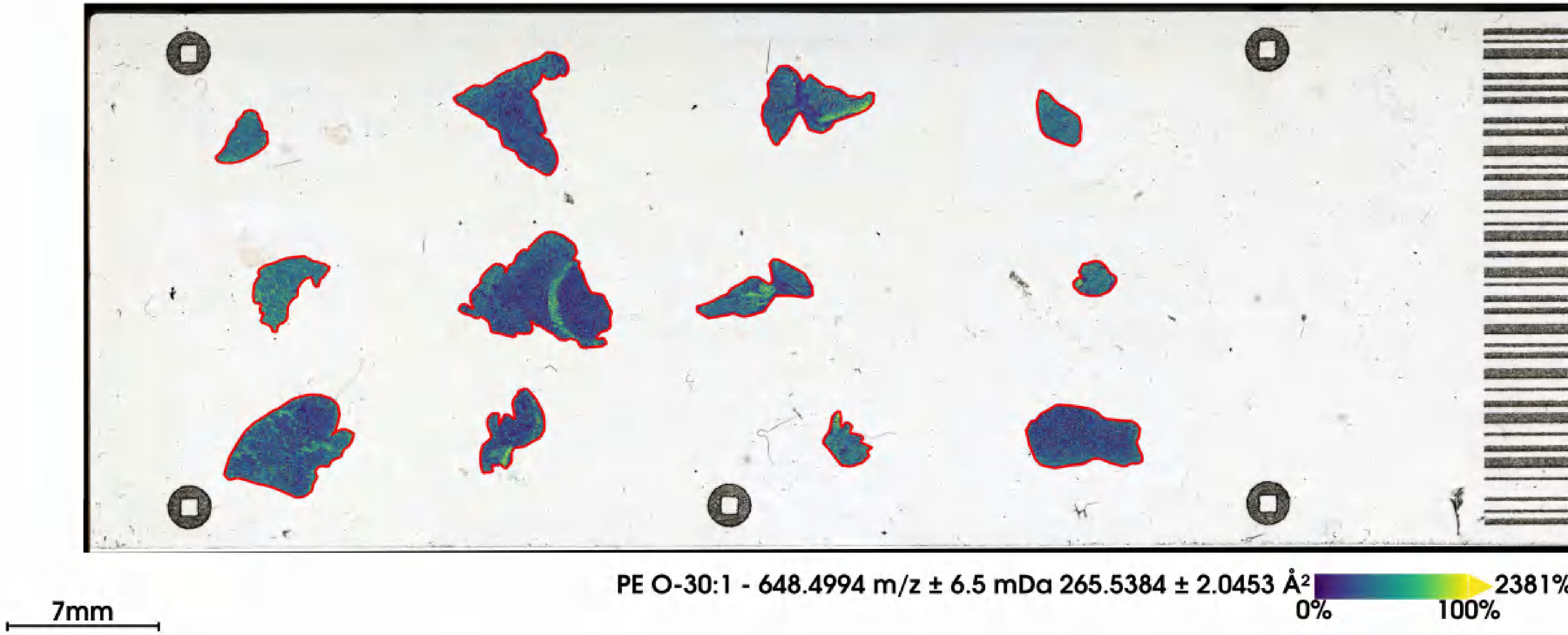
PE O-30:1 - 648.4994 m/z ± 6.5 mDa 265.5384 ± 2.0453 Å²
0%
100%
2381%
7mm

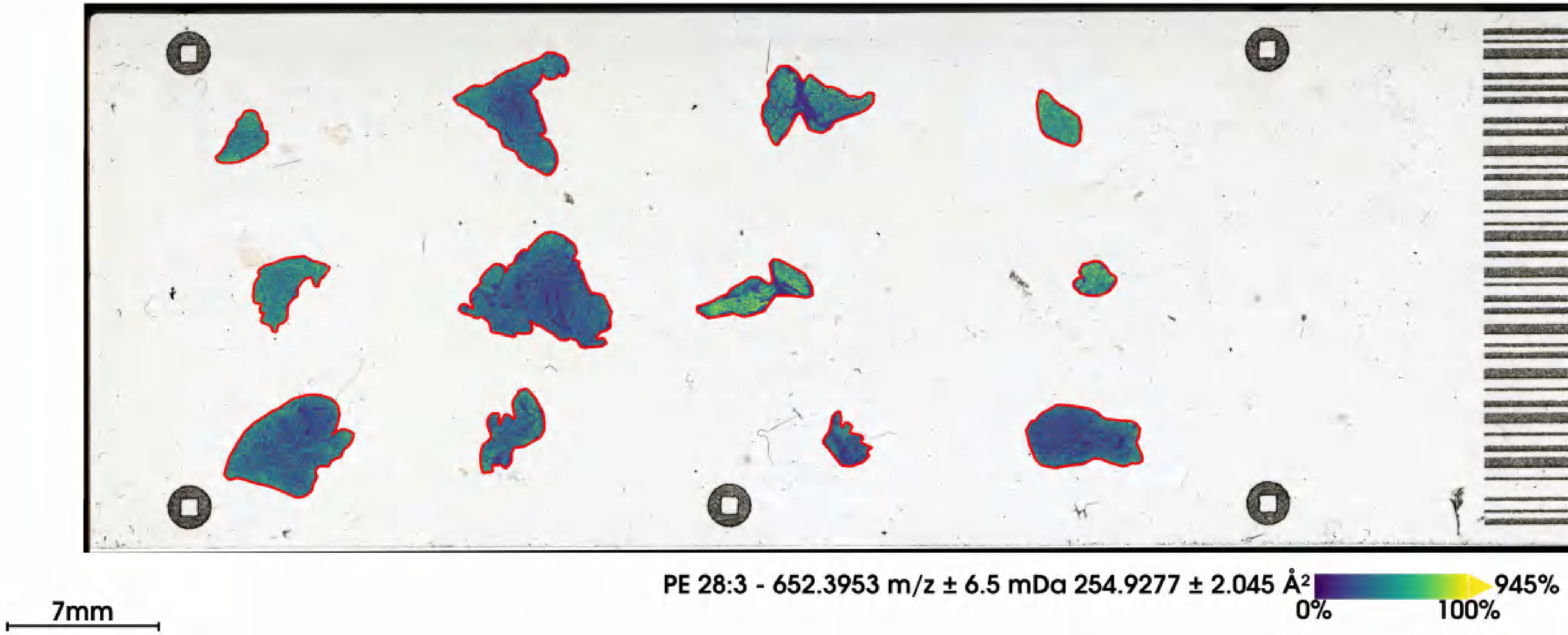
PE 28:3 - 652.3953 m/z ± 6.5 mDa 254.9277 ± 2.045 Å²
945%
0%
100%
7mm

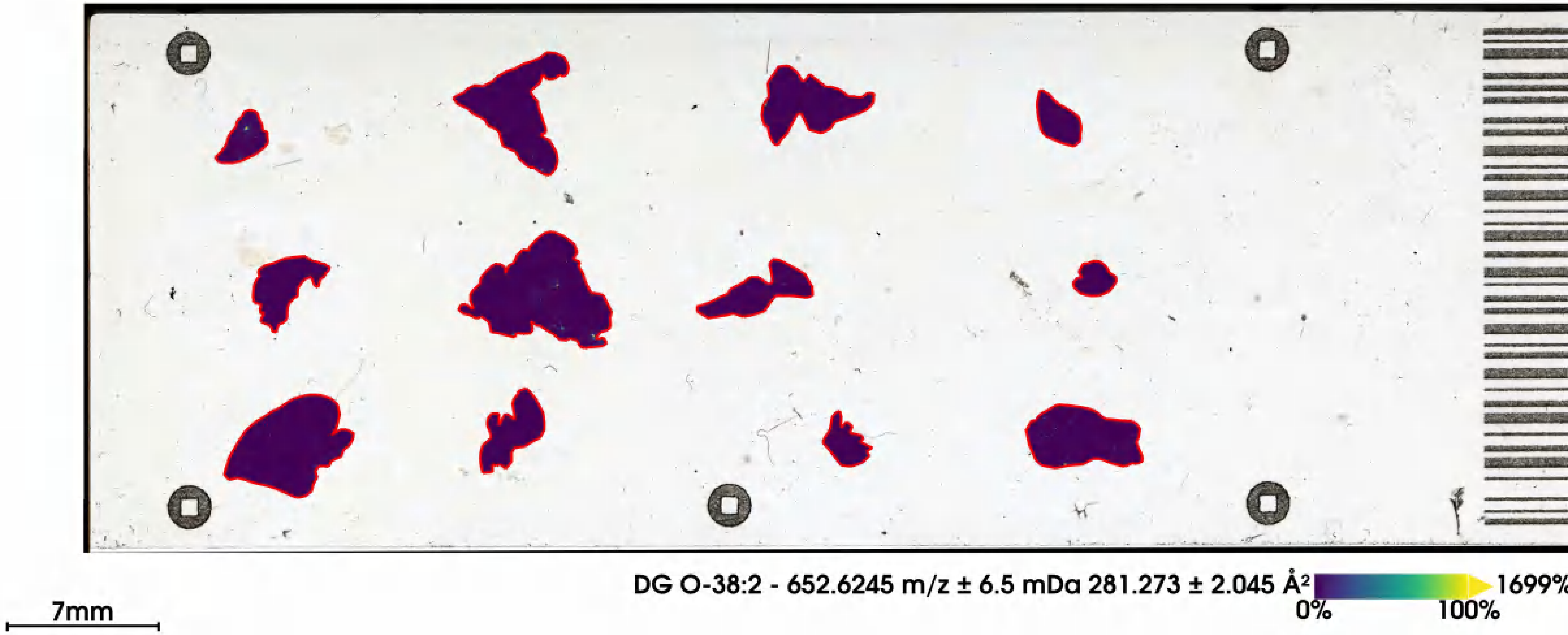
DG O-38:2 - 652.6245 m/z ± 6.5 mDa 281.273 ± 2.045 Å²
0%
100%
1699%
7mm

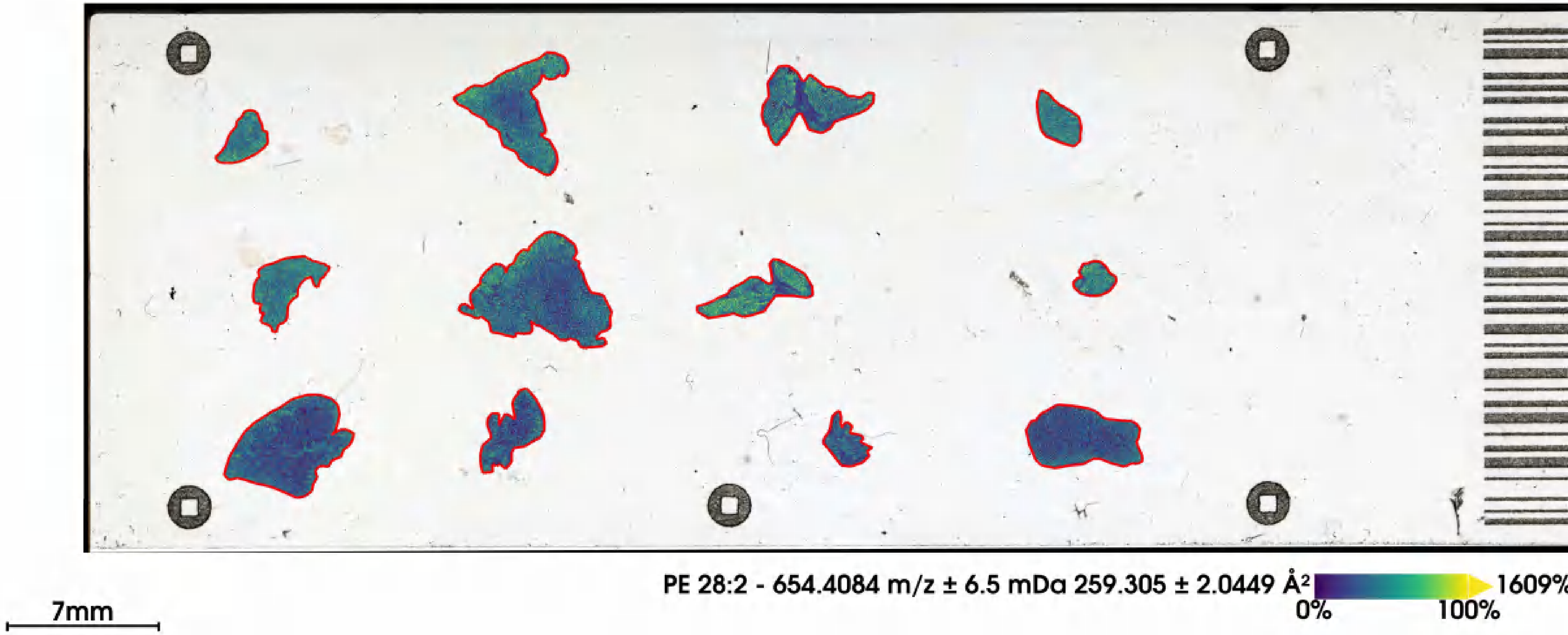
PE 28:2 - 654.4084 m/z ± 6.5 mDa 259.305 ± 2.0449 Å²
0%
100%
1609%
7mm

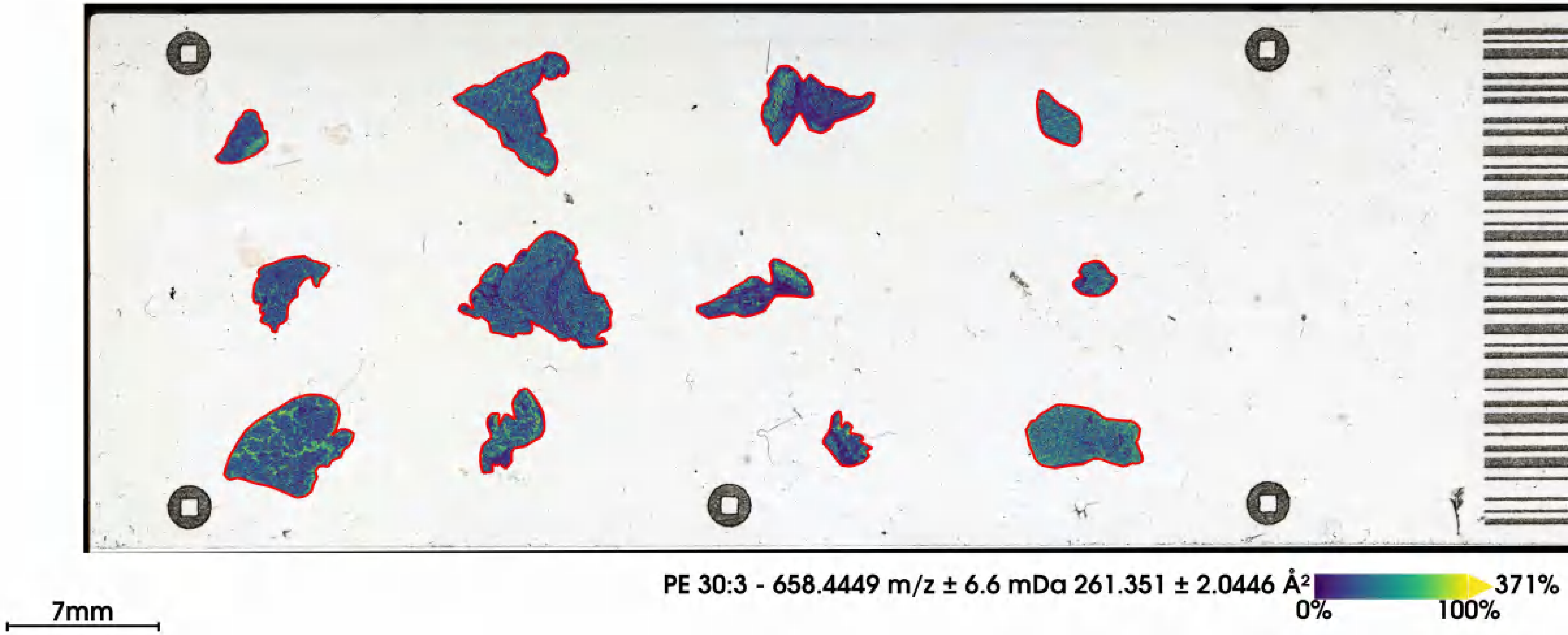

PE 30:3 - 658.4449 m/z ± 6.6 mDa 261.351 ± 2.0446 Å²
0%
100%
371%
7mm

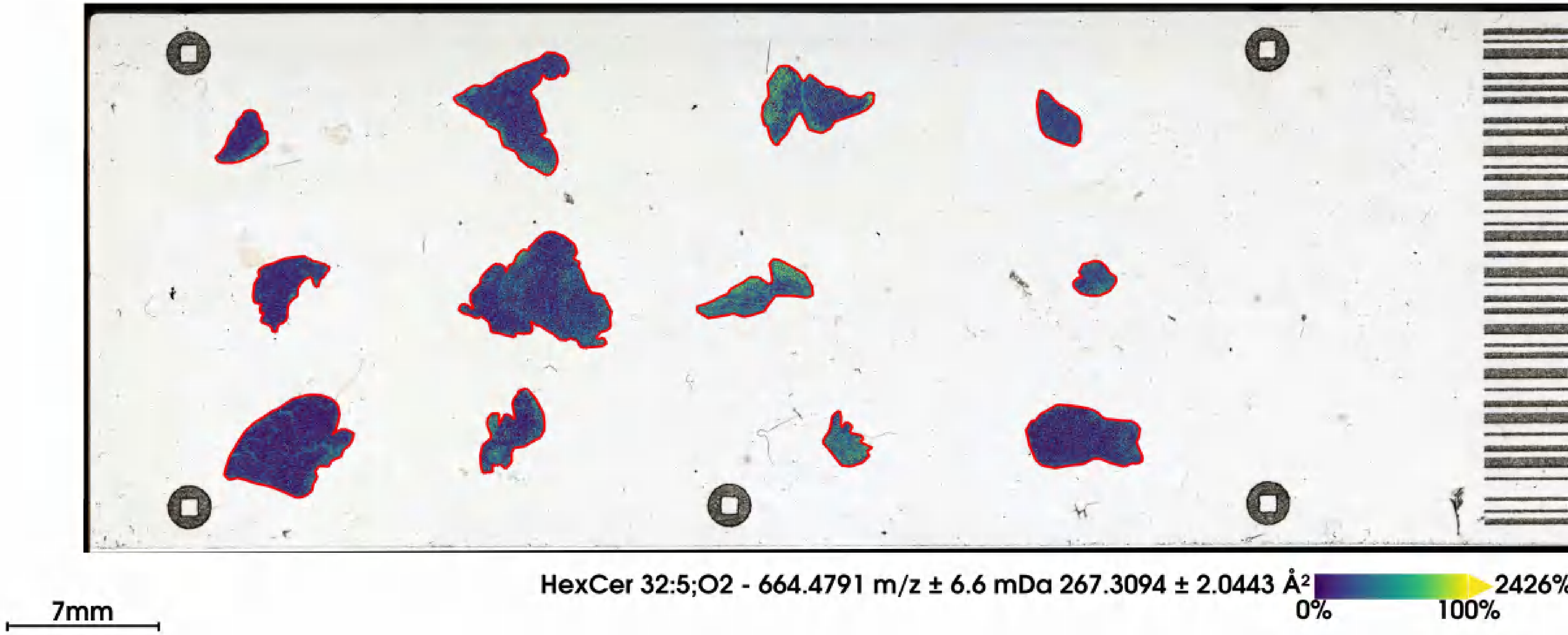
HexCer 32:5;O2 - 664.4791 m/z ± 6.6 mDa 267.3094 ± 2.0443 Å²
0%
100%
2426%
7mm

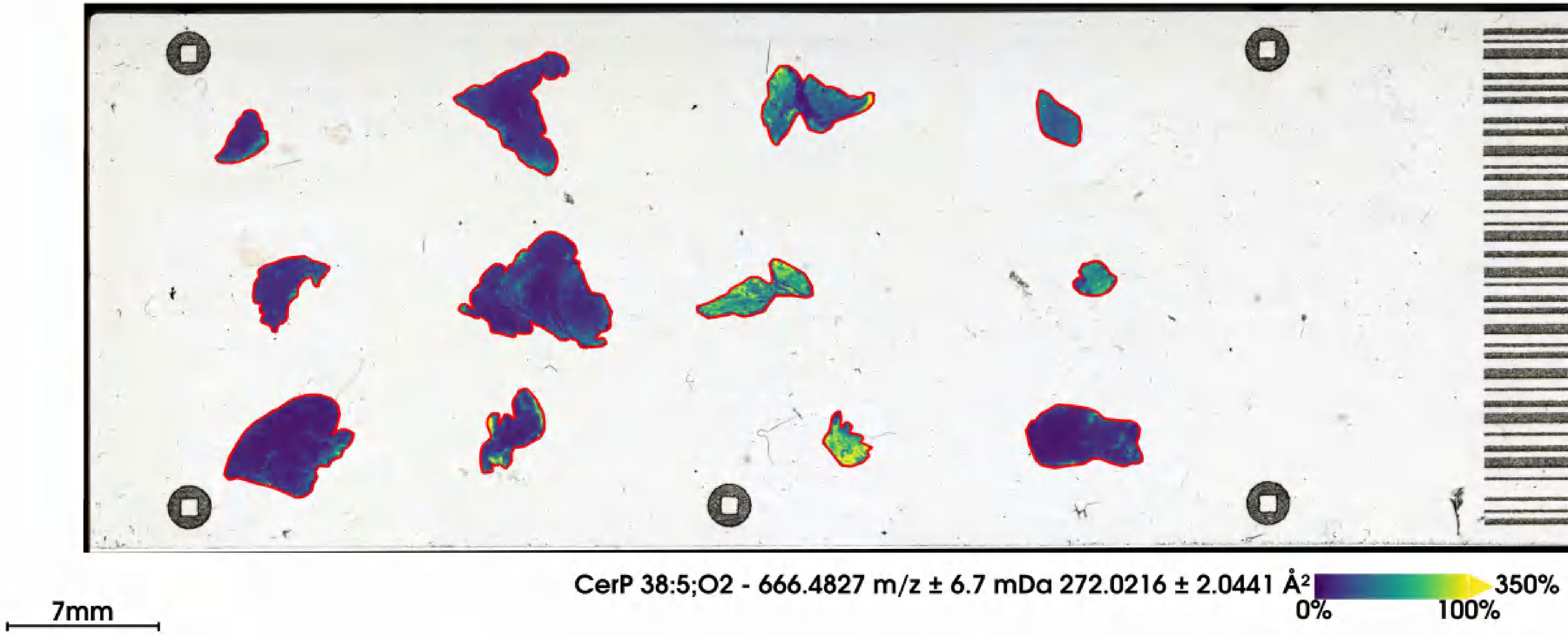
CerP 38:5;O2 - 666.4827 m/z ± 6.7 mDa 272.0216 ± 2.0441 Å²
0%
100%
350%
7mm

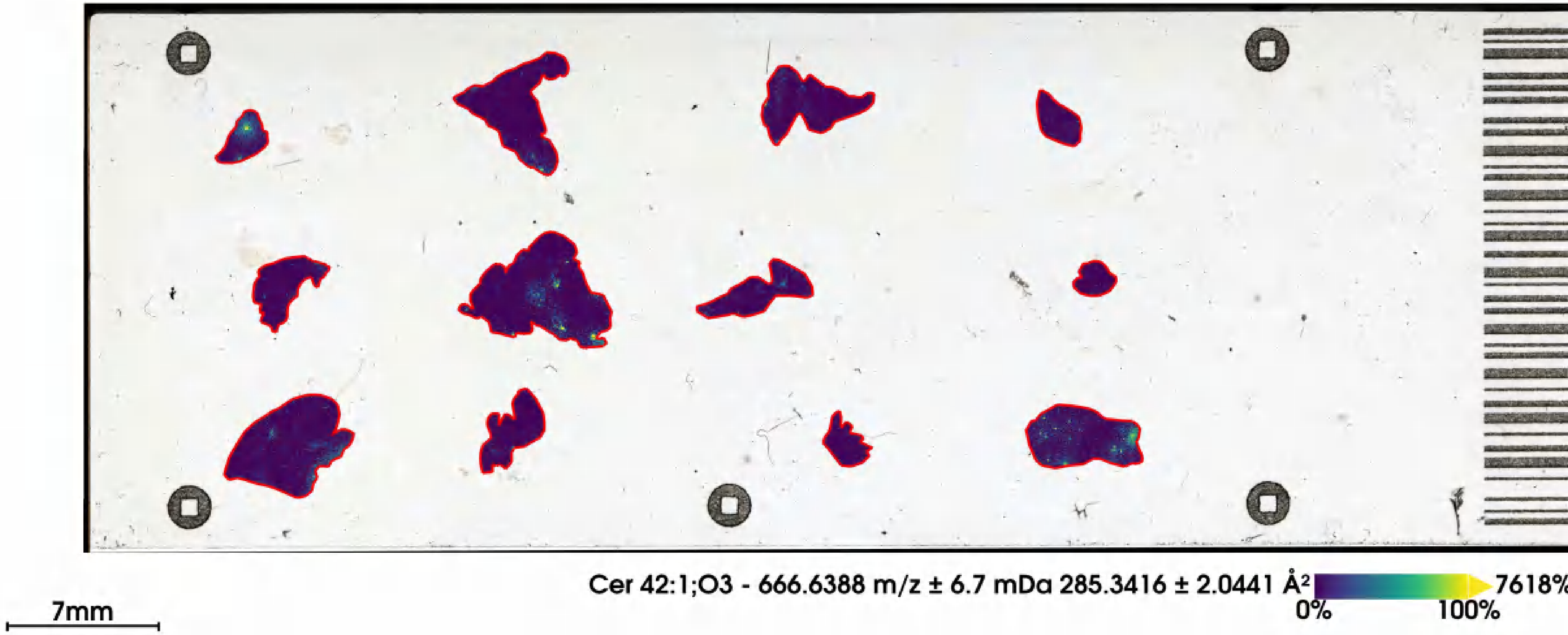
Cer 42:1;O3 - 666.6388 m/z ± 6.7 mDa 285.3416 ± 2.0441 Å²
0%
100%
7618%
7mm

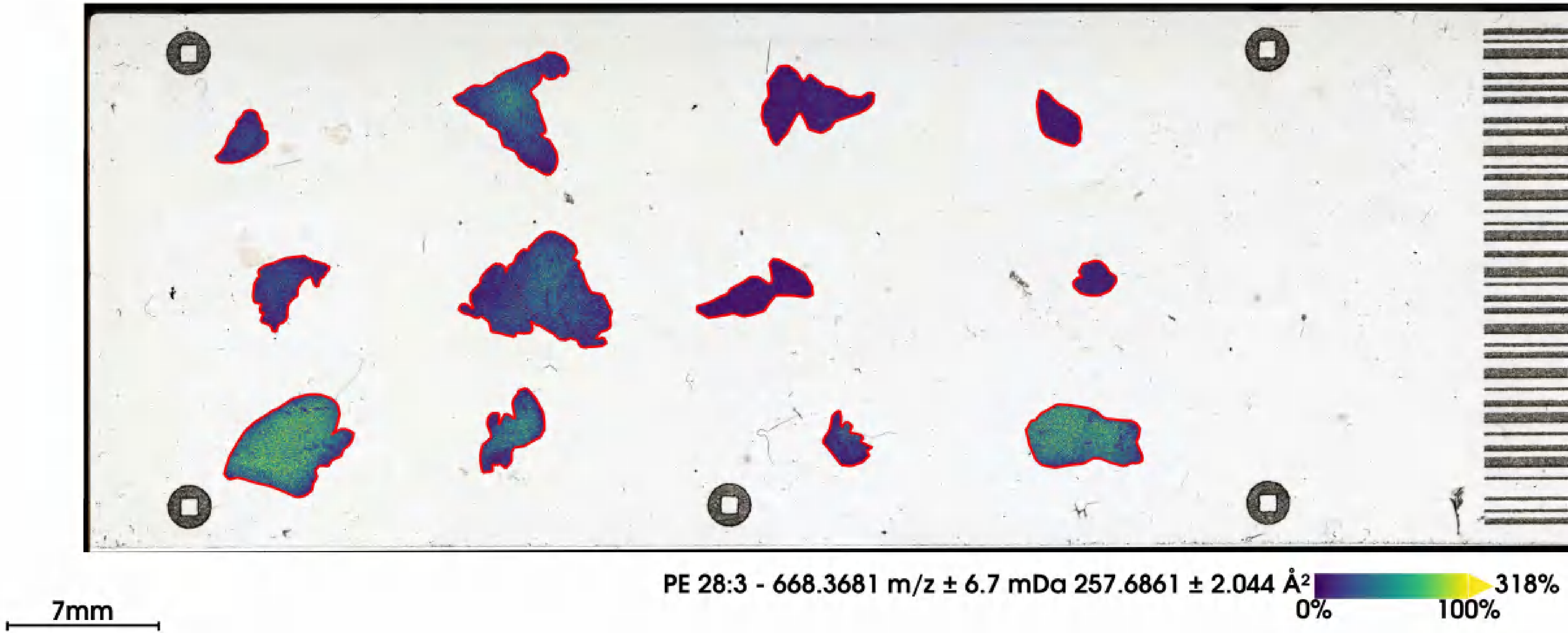

PE 28:3 - 668.3681 m/z ± 6.7 mDa 257.6861 ± 2.044 Å²
0%
100%
318%
7mm

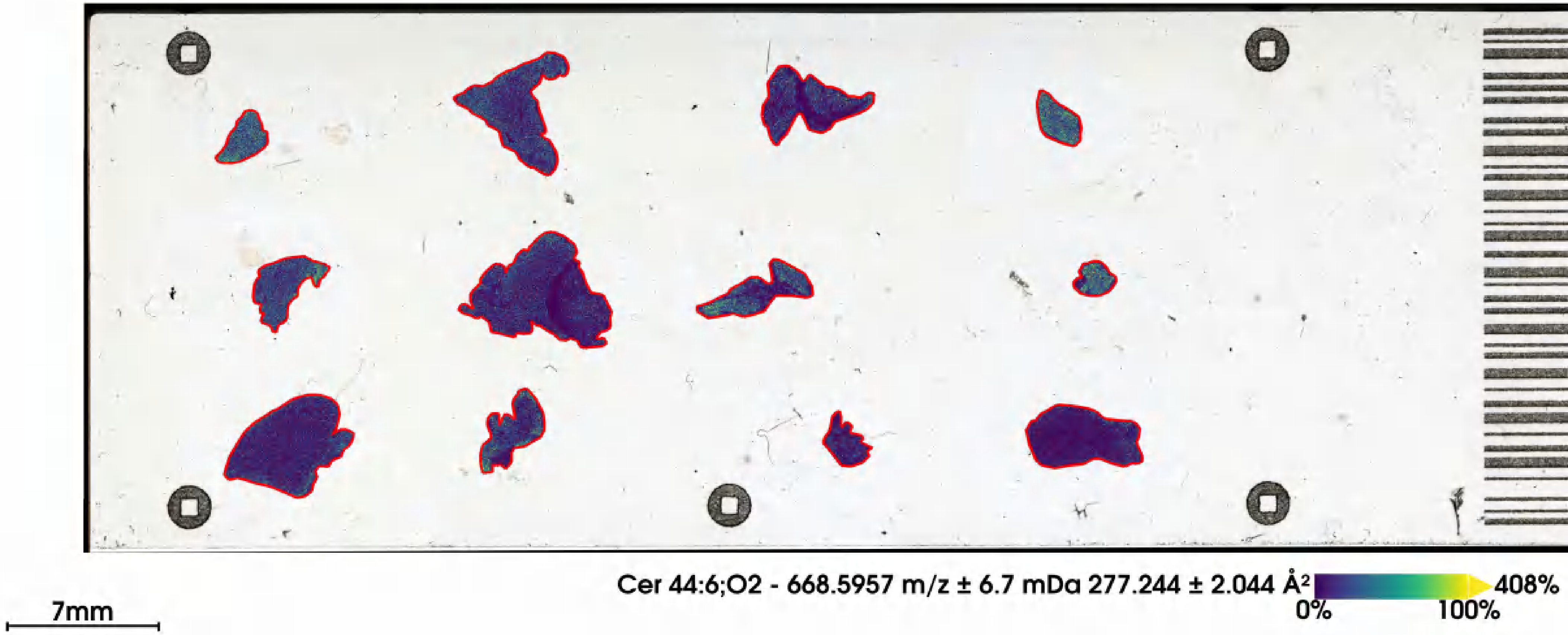

Cer 44:6;O2 - 668.5957 m/z ± 6.7 mDa 277.244 ± 2.044 Å²
0%
100%
408%
7mm

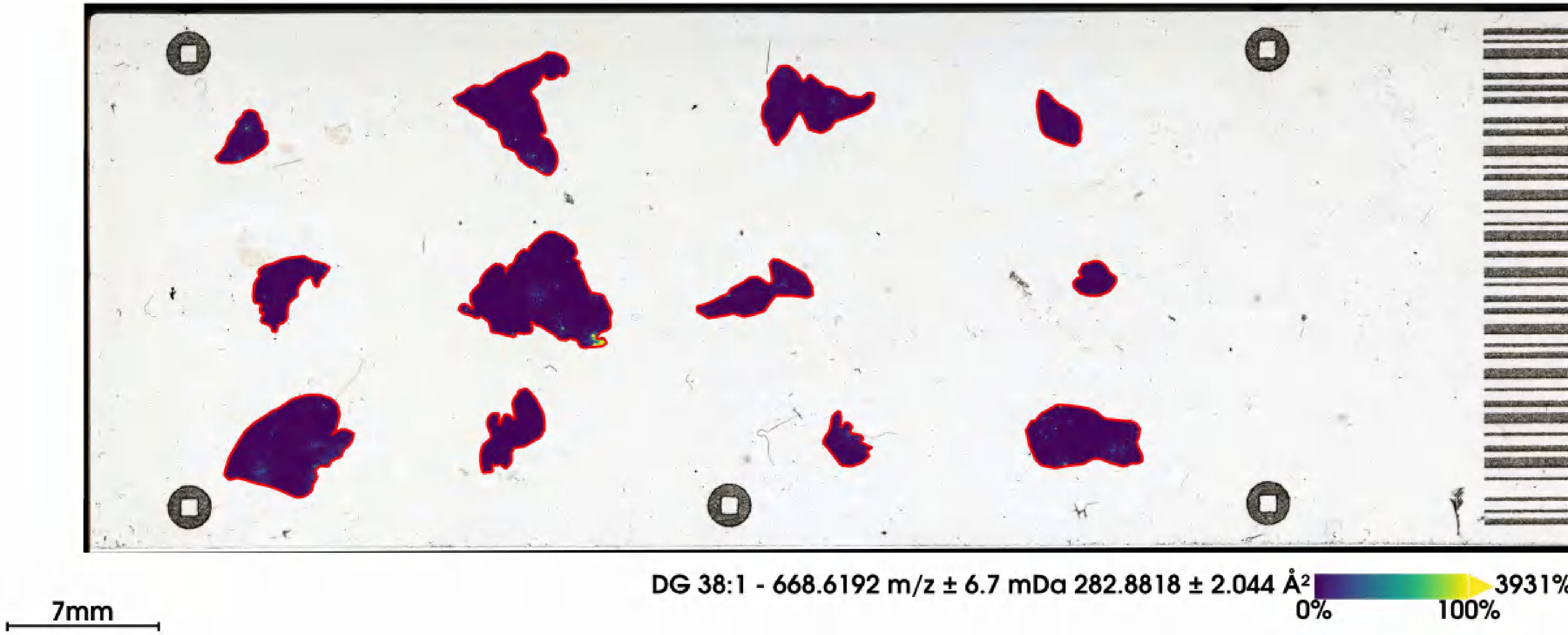
DG 38:1 - 668.6192 m/z ± 6.7 mDa 282.8818 ± 2.044 Å²
3931%
0%
100%
7mm

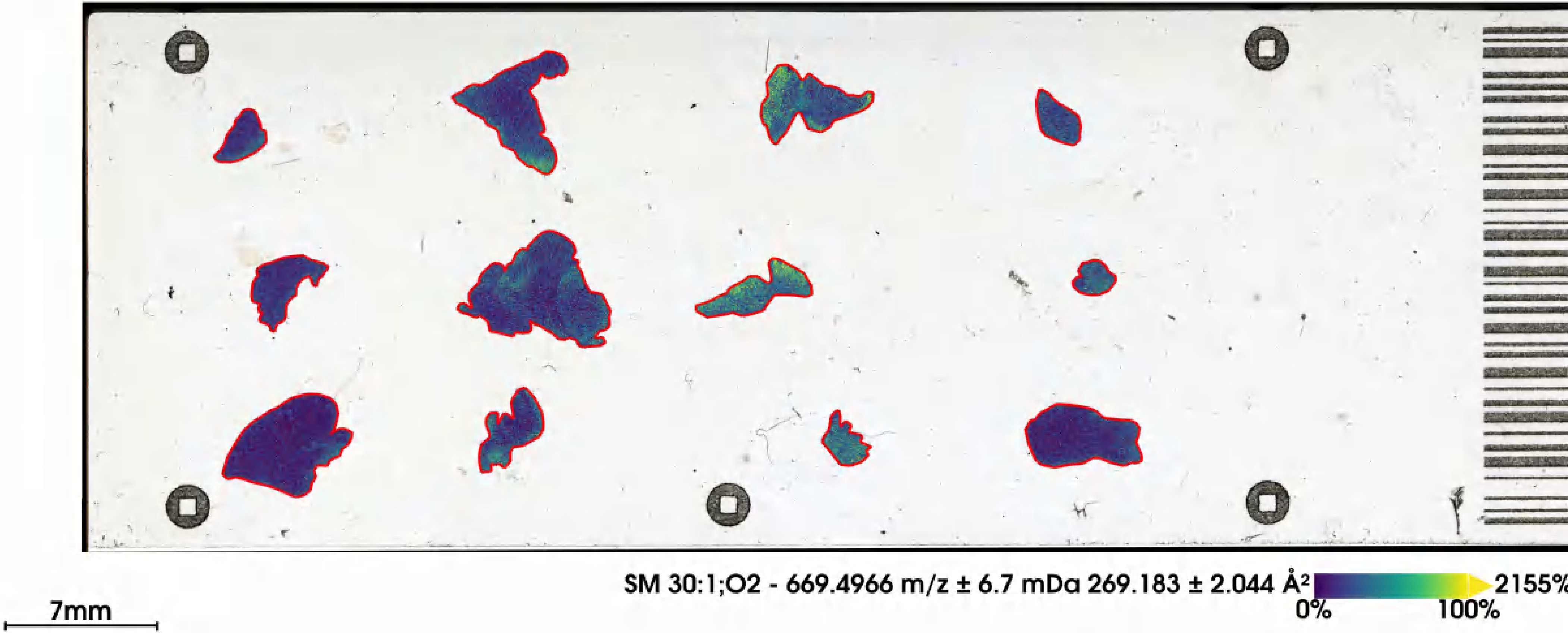
SM 30:1;O2 - 669.4966 m/z ± 6.7 mDa 269.183 ± 2.044 Å²
0%
100%
2155%
7mm

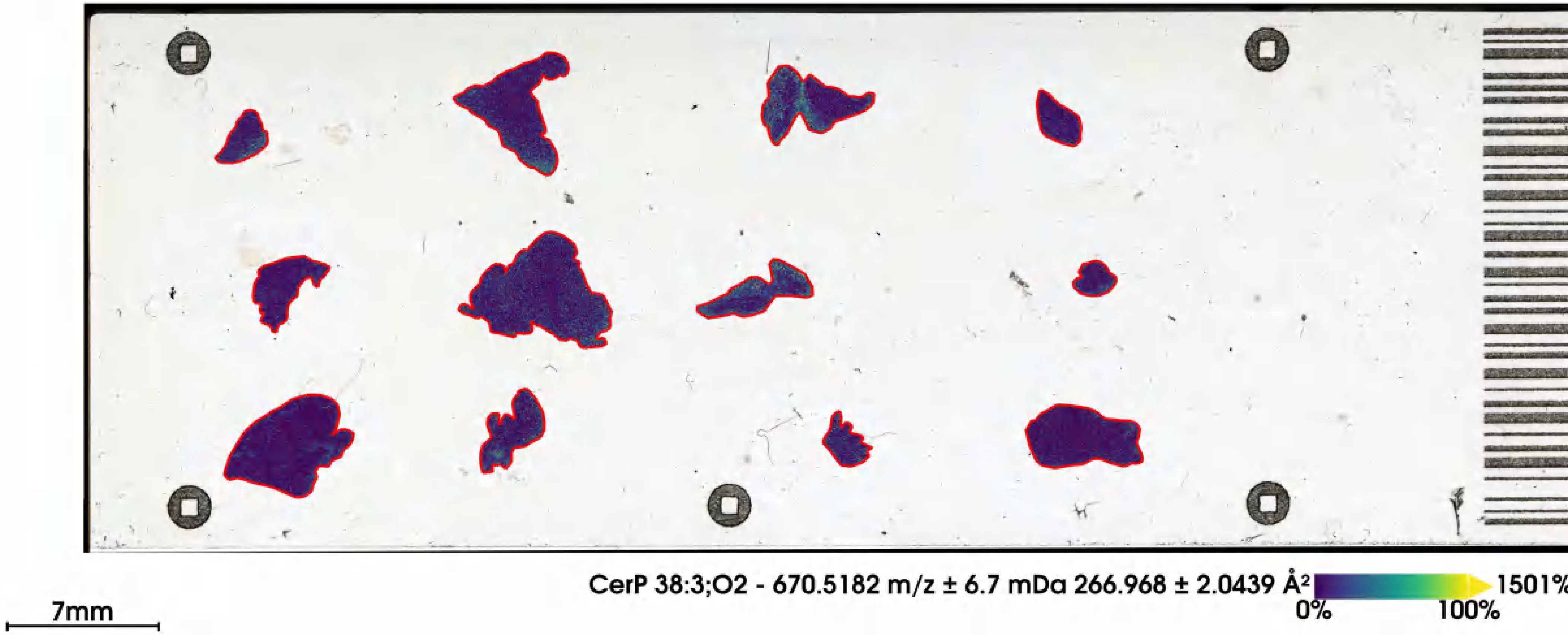
CerP 38:3;O2 - 670.5182 m/z ± 6.7 mDa 266.968 ± 2.0439 Å²
0%
100%
1501%
7mm

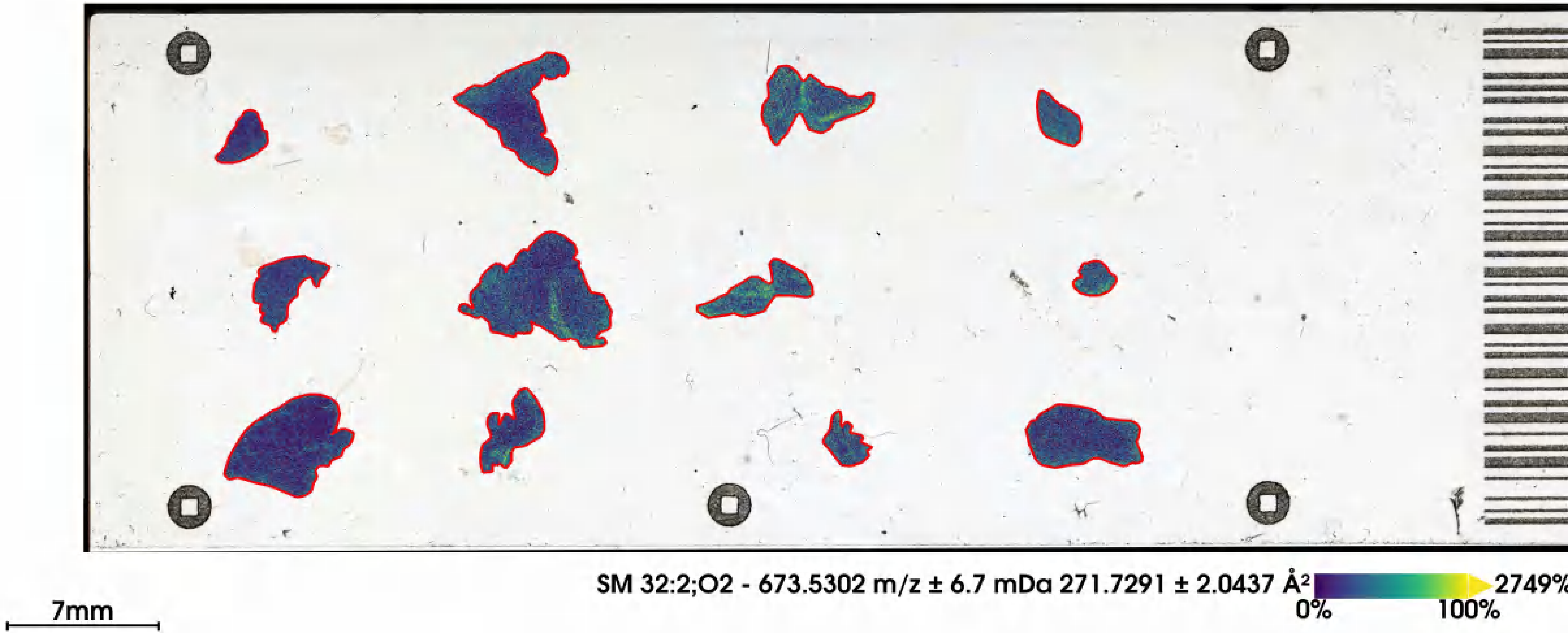
SM 32:2;O2 - 673.5302 m/z ± 6.7 mDa 271.7291 ± 2.0437 Å²
0%
100%
2749%
7mm

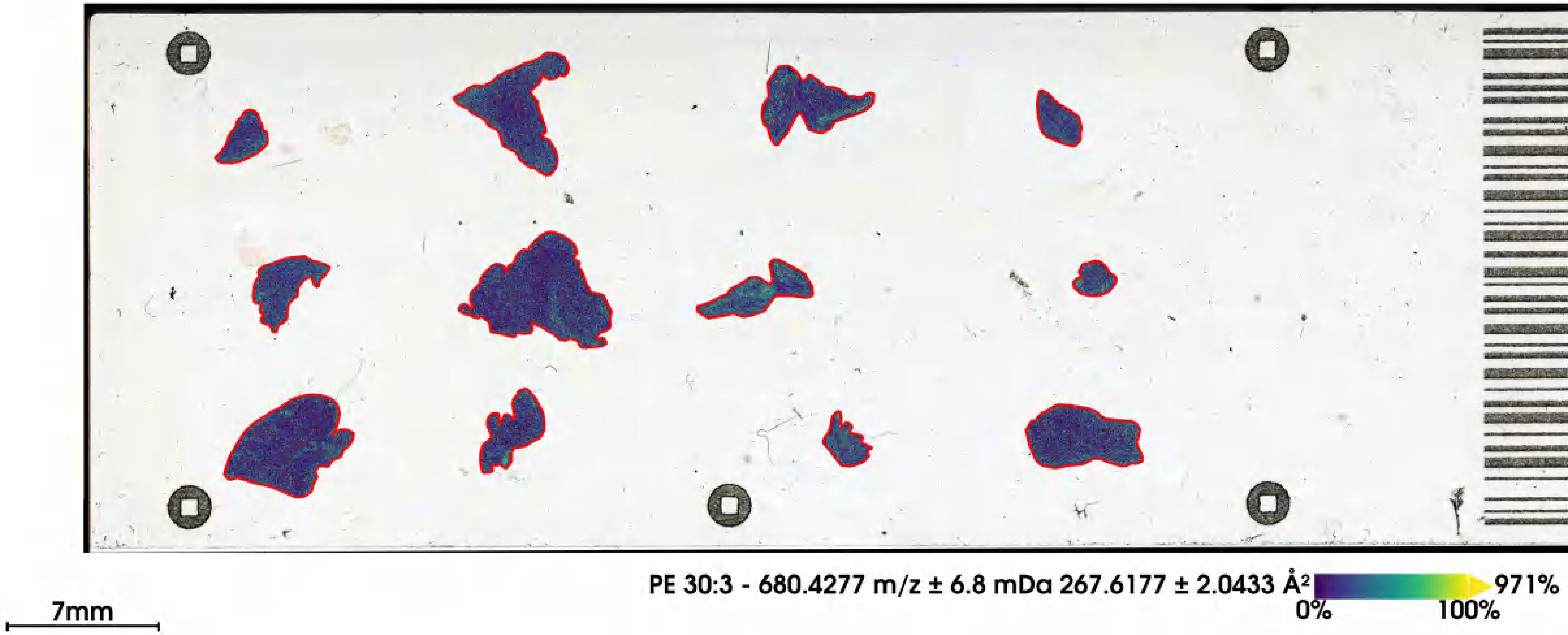

PE 30:3 - 680.4277 m/z ± 6.8 mDa 267.6177 ± 2.0433 Å²
0%
100%
971%
7mm

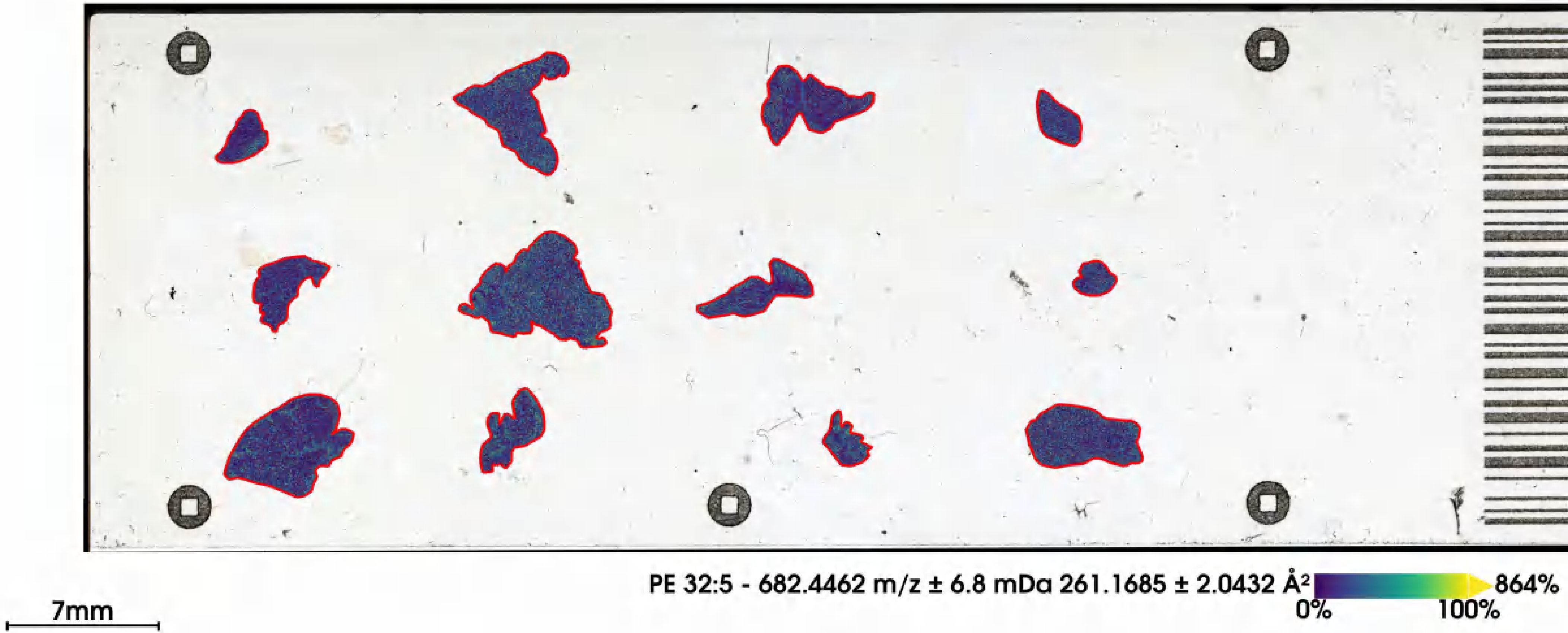
7mm
PE 32:5 - 682.4462 m/z ± 6.8 mDa 261.1685 ± 2.0432 Å²
0%
100%
864%

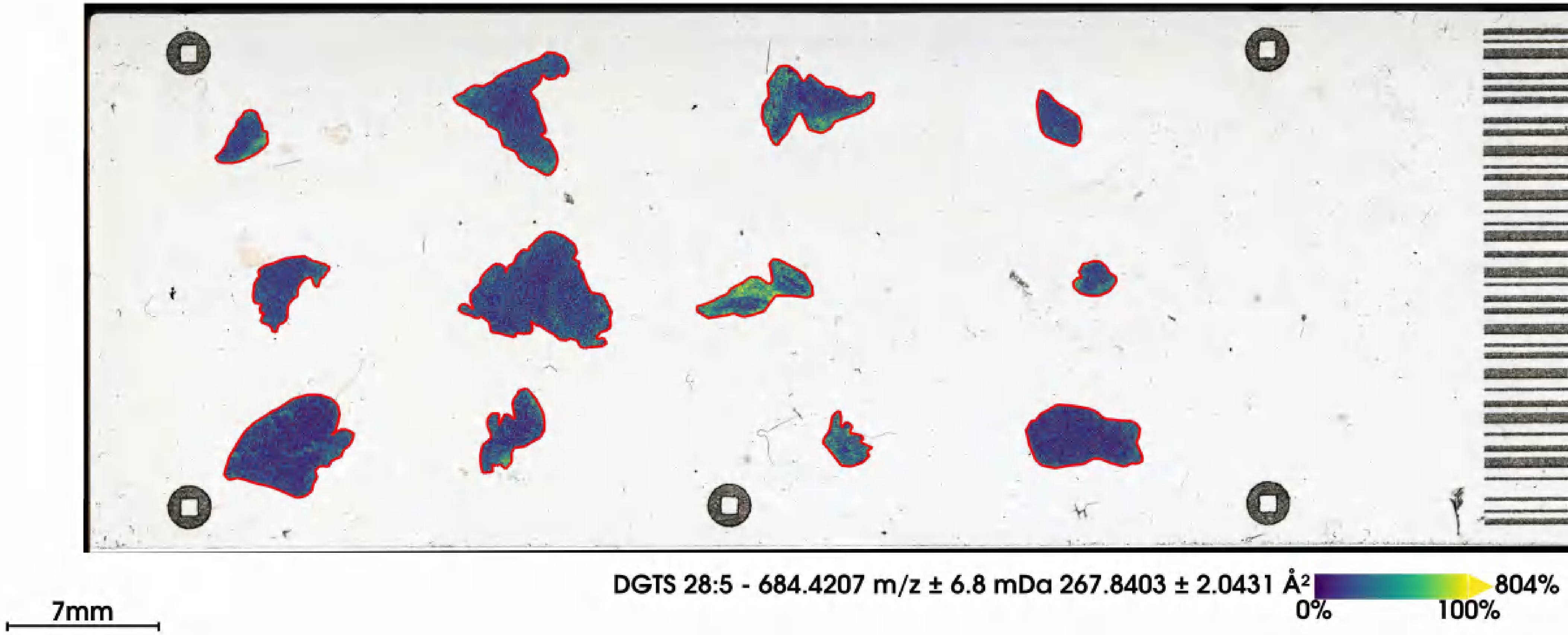

DGTS 28:5 - 684.4207 m/z ± 6.8 mDa 267.8403 ± 2.0431 Å²
804%
0%
100%
7mm

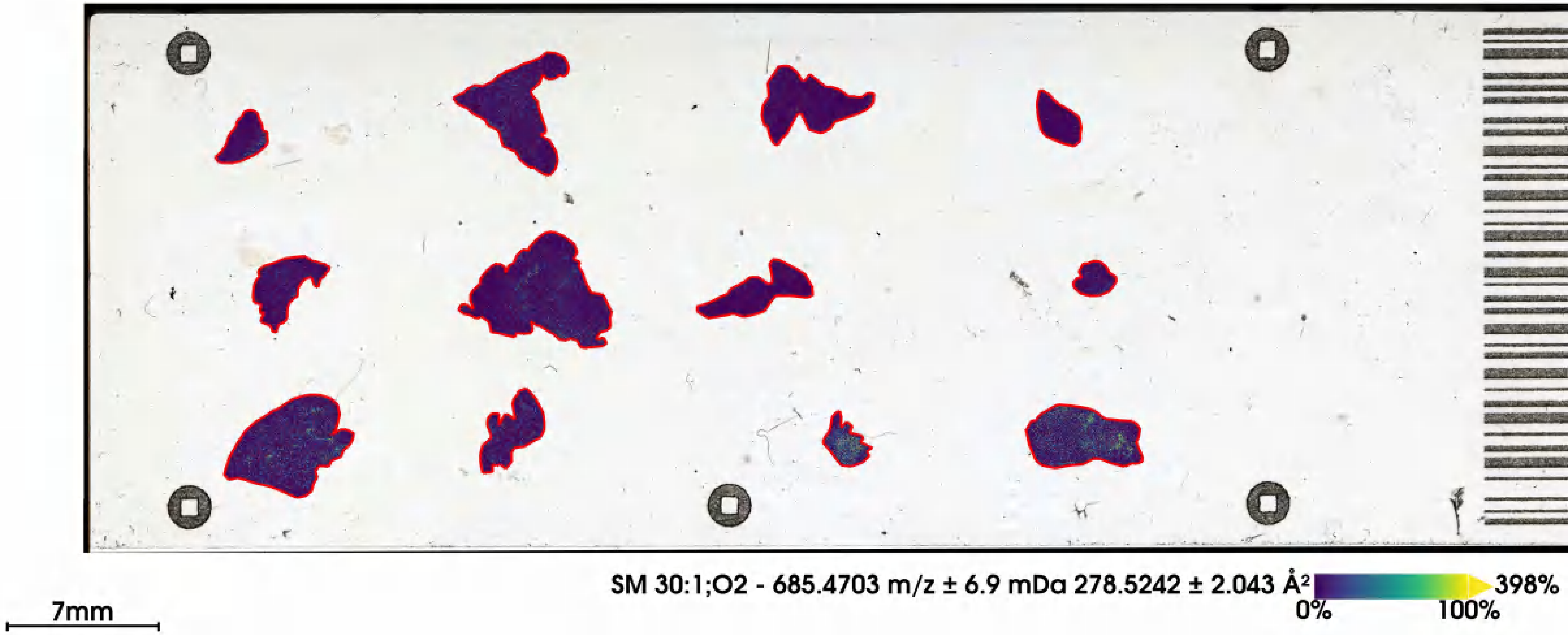
SM 30:1;O2 - 685.4703 m/z ± 6.9 mDa 278.5242 ± 2.043 Å²
398%
0%
100%
7mm

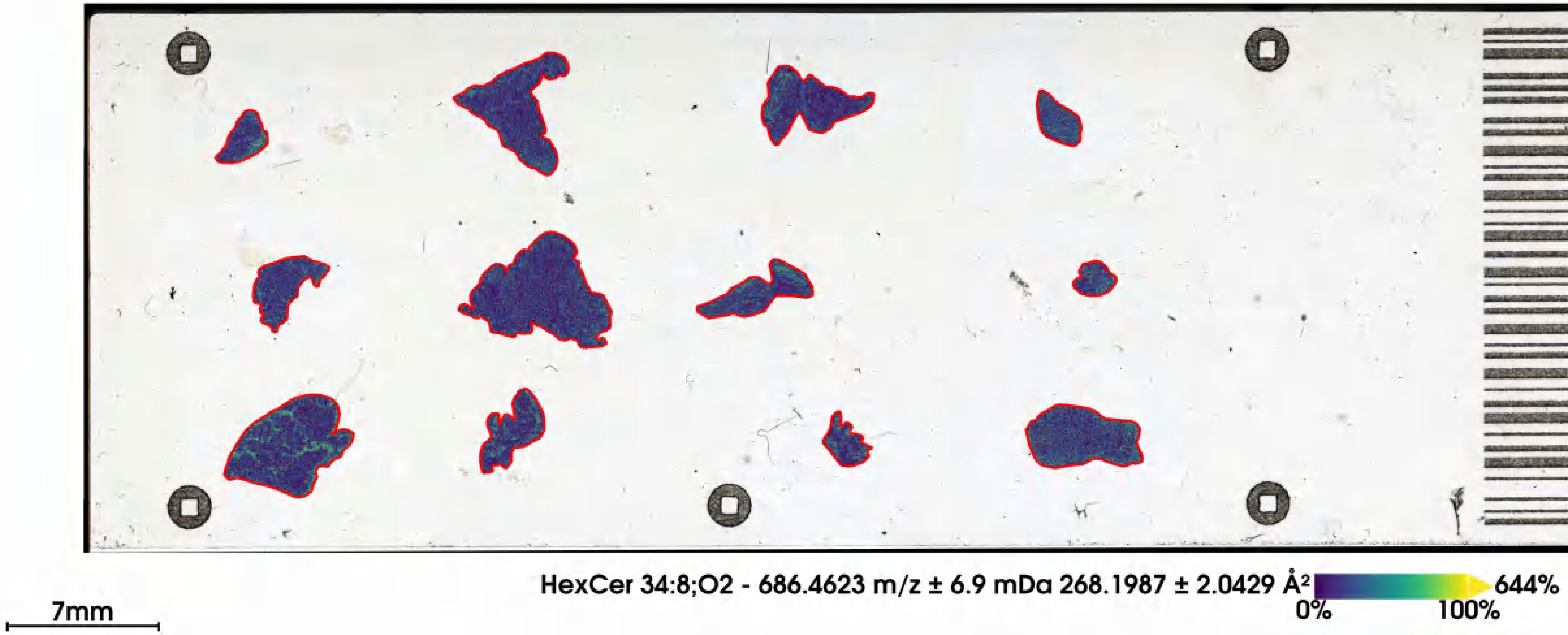

HexCer 34:8;O2 - 686.4623 m/z ± 6.9 mDa 268.1987 ± 2.0429 Å²
0%
100%
644%
7mm

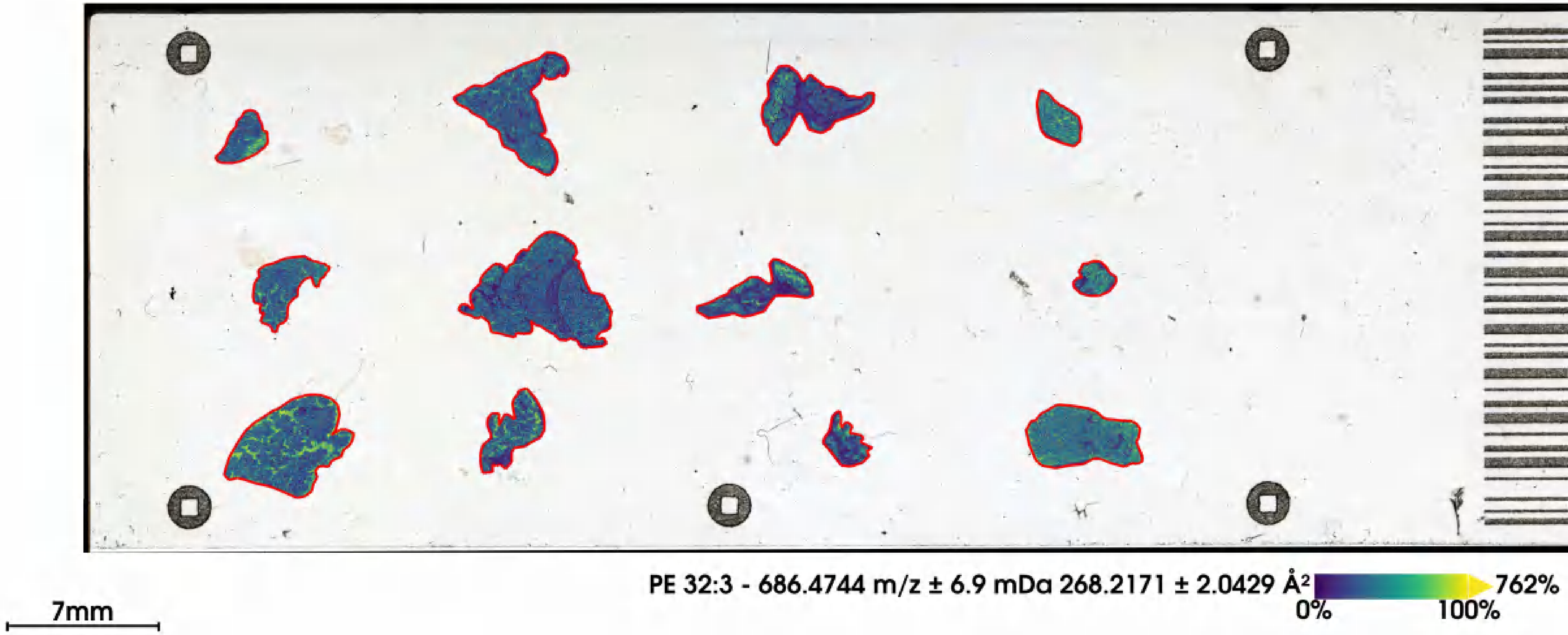
PE 32:3 - 686.4744 m/z ± 6.9 mDa 268.2171 ± 2.0429 Å²
0%
100%
762%
7mm

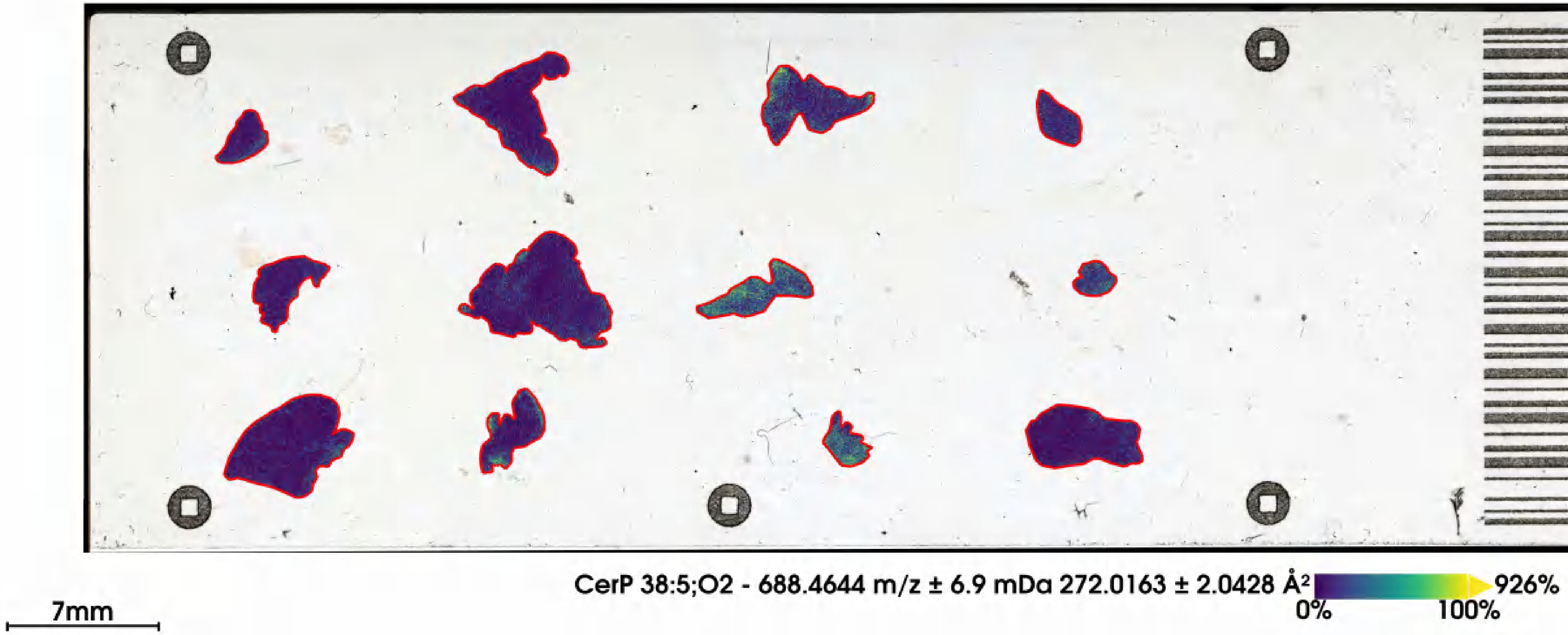
CerP 38:5;O2 - 688.4644 m/z ± 6.9 mDa 272.0163 ± 2.0428 Å²
926%
0%
100%
7mm

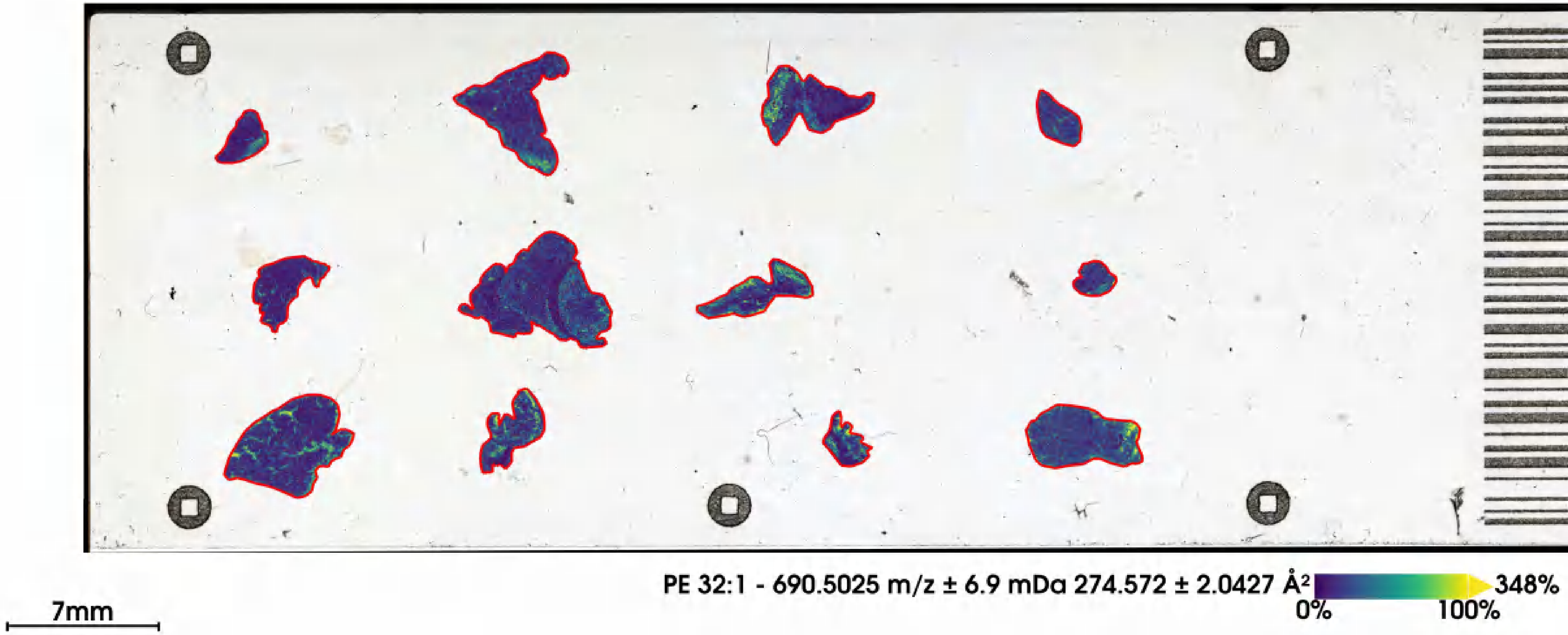
7mm
PE 32:1 - 690.5025 m/z ± 6.9 mDa 274.572 ± 2.0427 Å²
0%
100%
348%

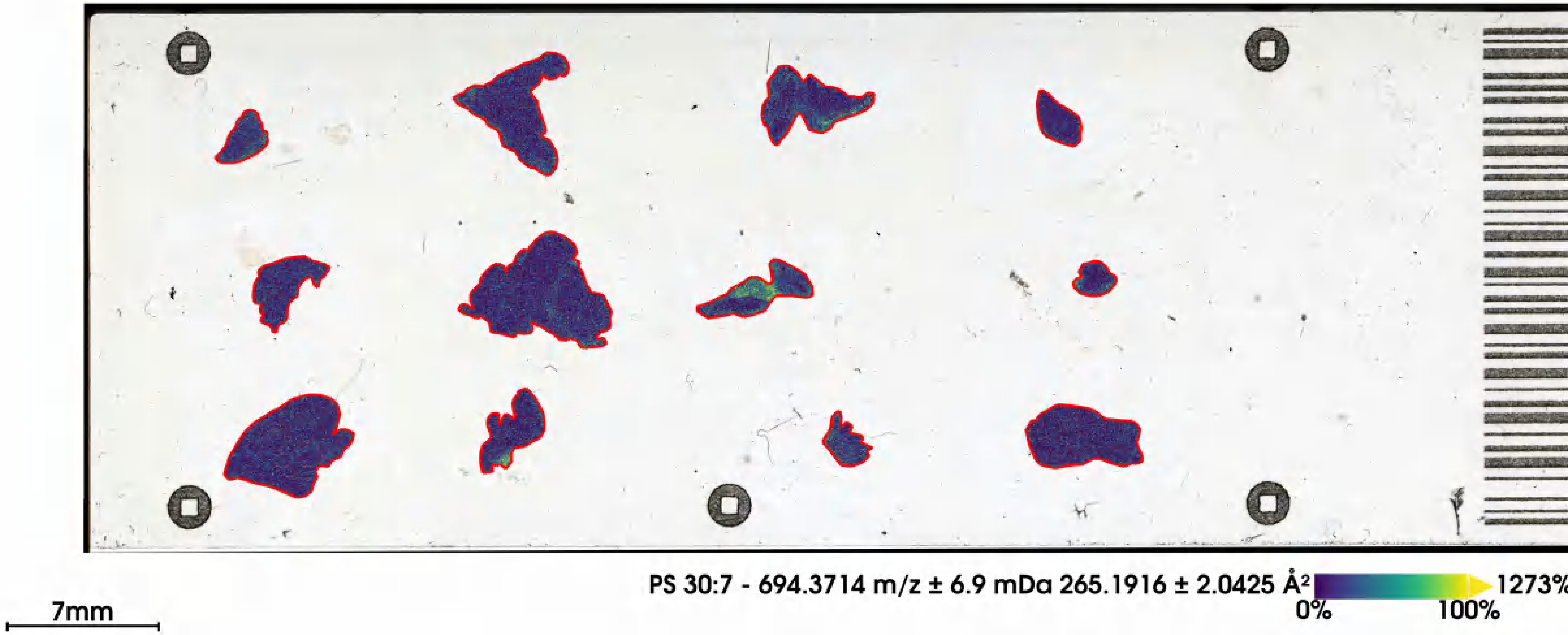
PS 30:7 - 694.3714 m/z ± 6.9 mDa 265.1916 ± 2.0425 Å²
0%
100%
1273%
7mm

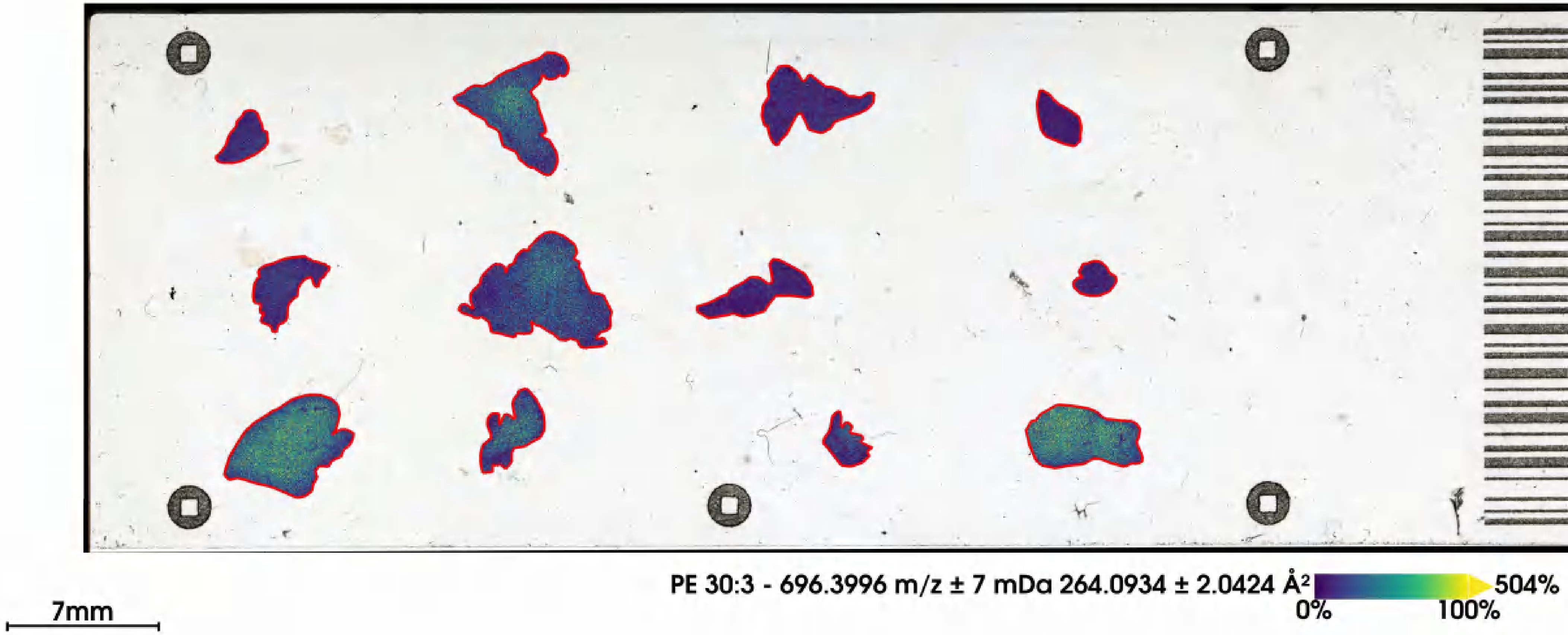
PE 30:3 - 696.3996 m/z ± 7 mDa 264.0934 ± 2.0424 Å²
504%
0%
100%
7mm

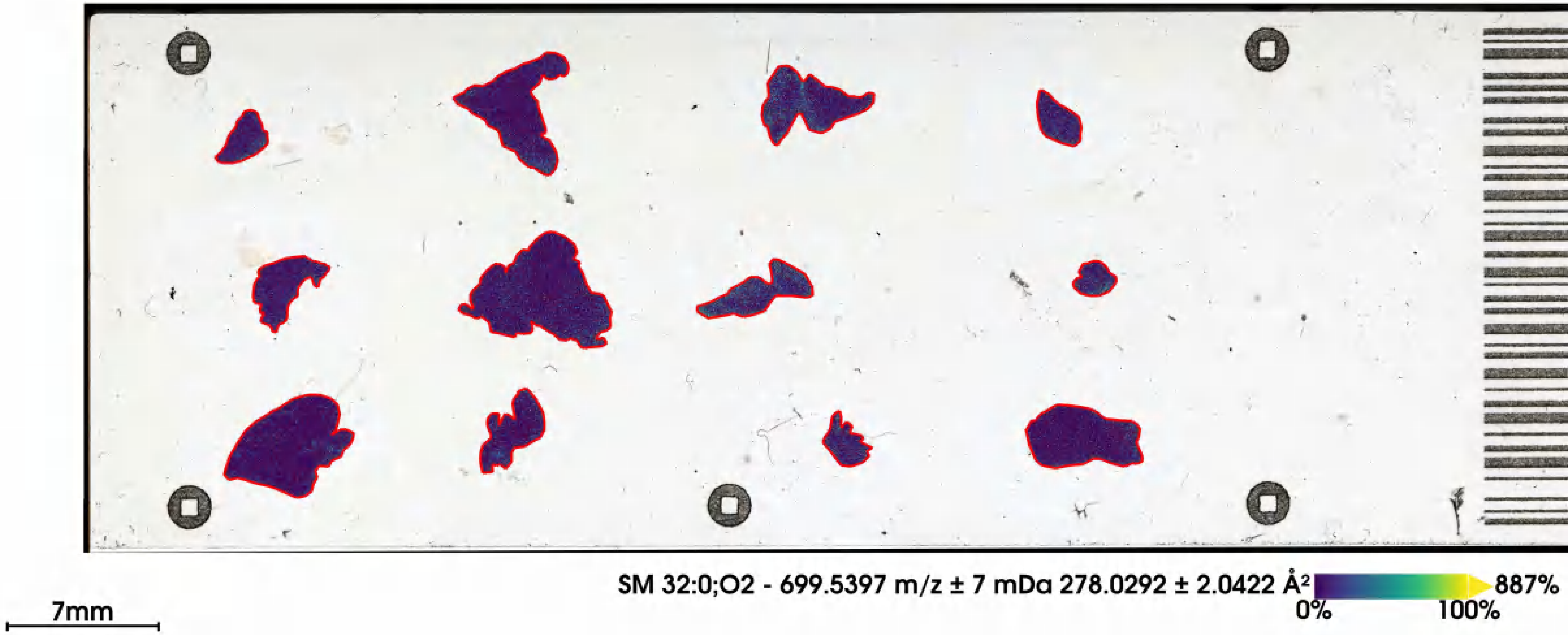

SM 32:0;O2 - 699.5397 m/z ± 7 mDa 278.0292 ± 2.0422 Å²
0%
100%
887%
7mm

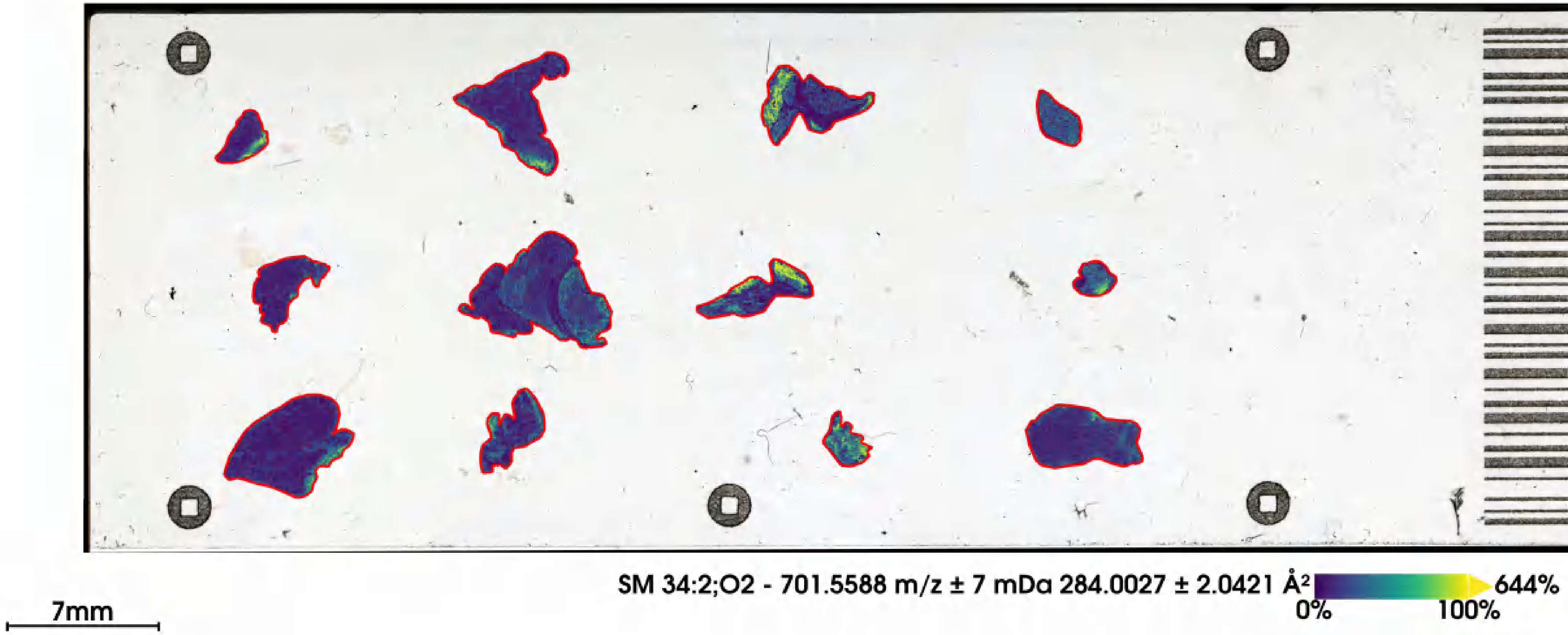
SM 34:2;O2 - 701.5588 m/z ± 7 mDa 284.0027 ± 2.0421 Å²
0%
100%
644%
7mm

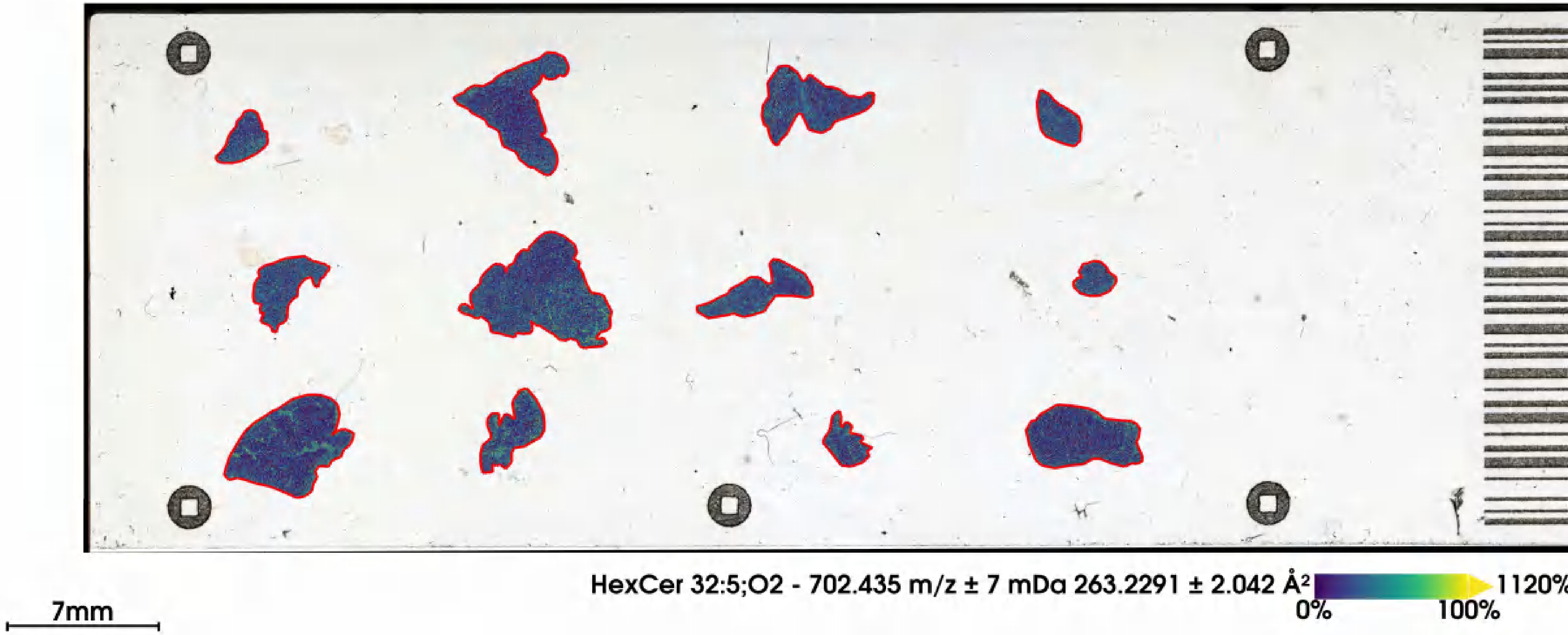
HexCer 32:5;O2 - 702.435 m/z ± 7 mDa 263.2291 ± 2.042 Å²
0%
100%
1120%
7mm

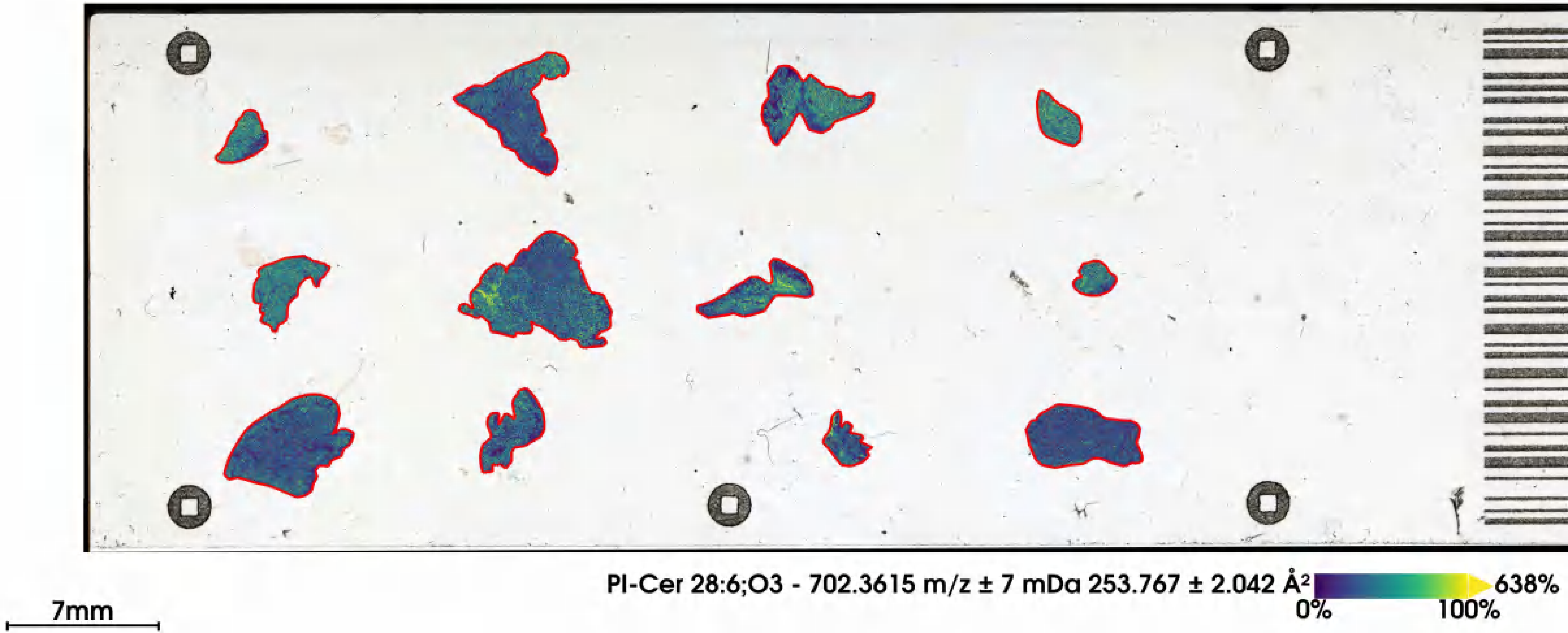

PI-Cer 28:6;O3 - 702.3615 m/z ± 7 mDa 253.767 ± 2.042 Å²
0%
100%
638%
7mm

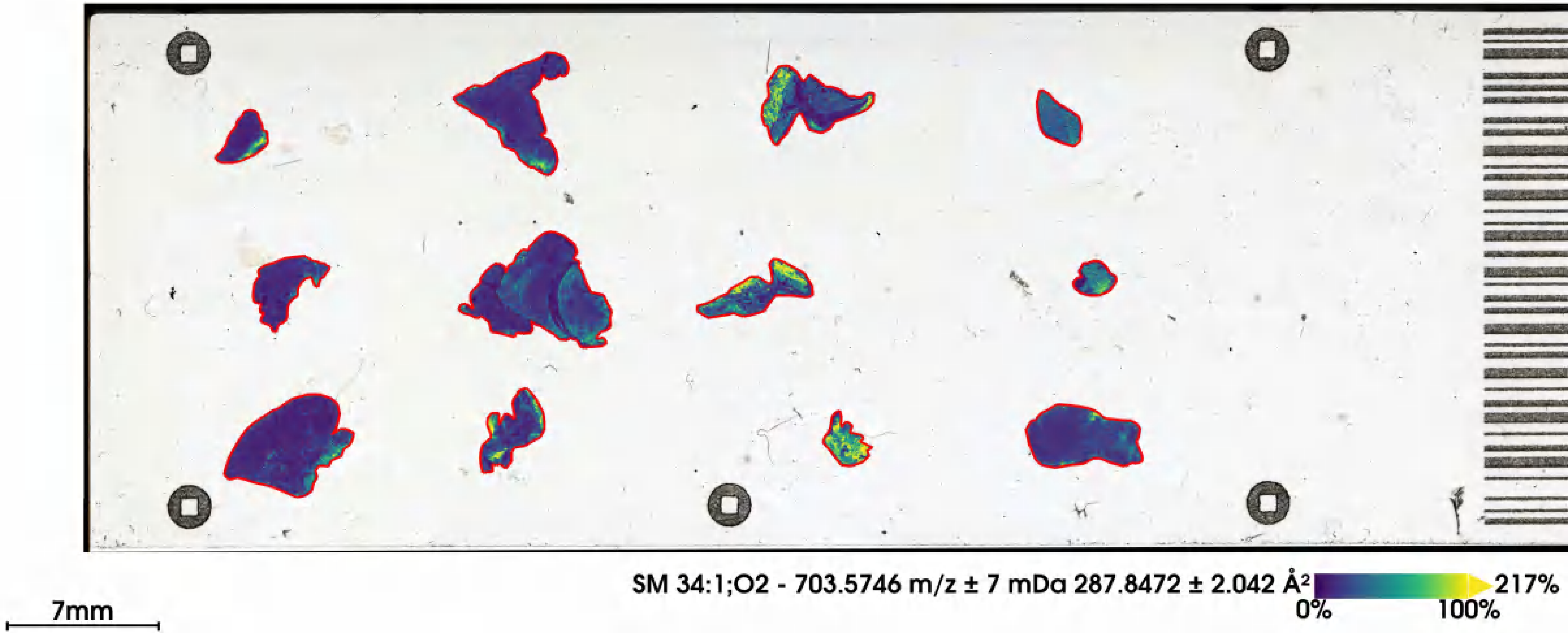
SM 34:1;O2 - 703.5746 m/z ± 7 mDa 287.8472 ± 2.042 Å²
0%
100%
217%
7mm

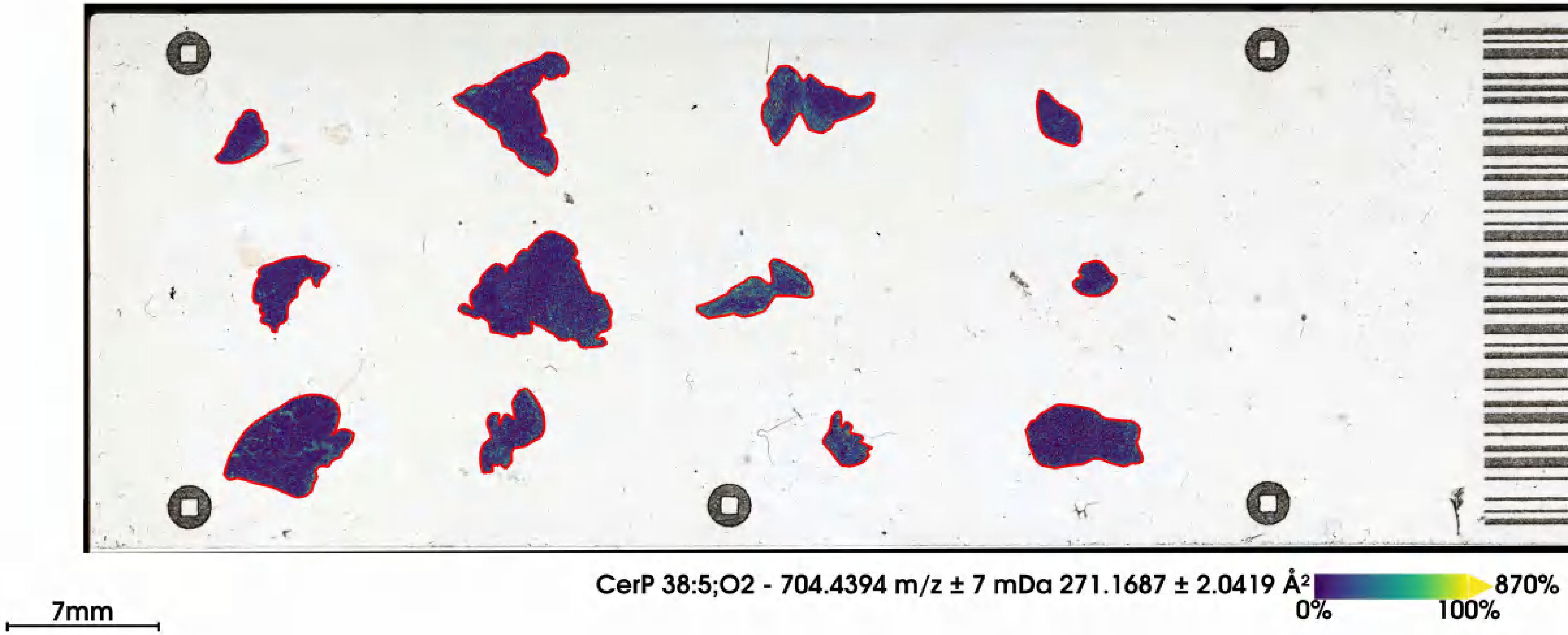
CerP 38:5;O2 - 704.4394 m/z ± 7 mDa 271.1687 ± 2.0419 Å²
0%
100%
870%
7mm

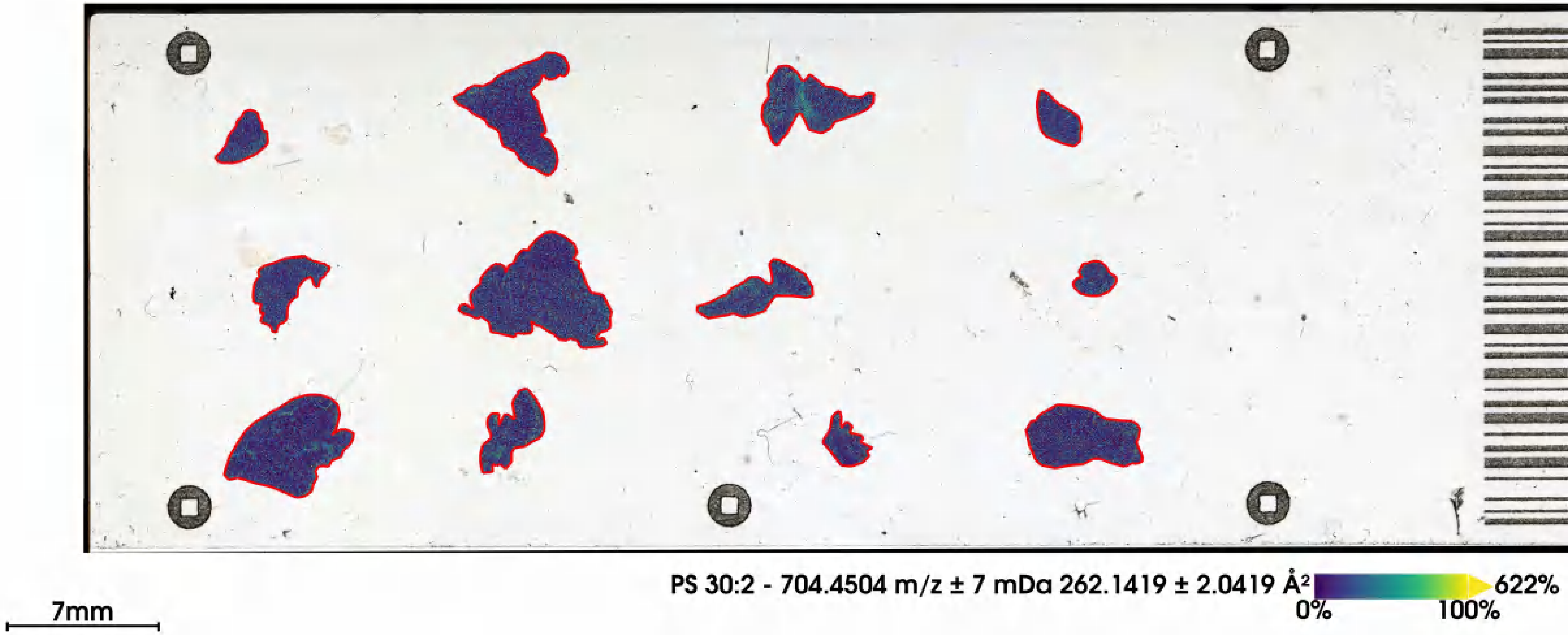
PS 30:2 - 704.4504 m/z ± 7 mDa 262.1419 ± 2.0419 Å²
0%
100%
622%
7mm

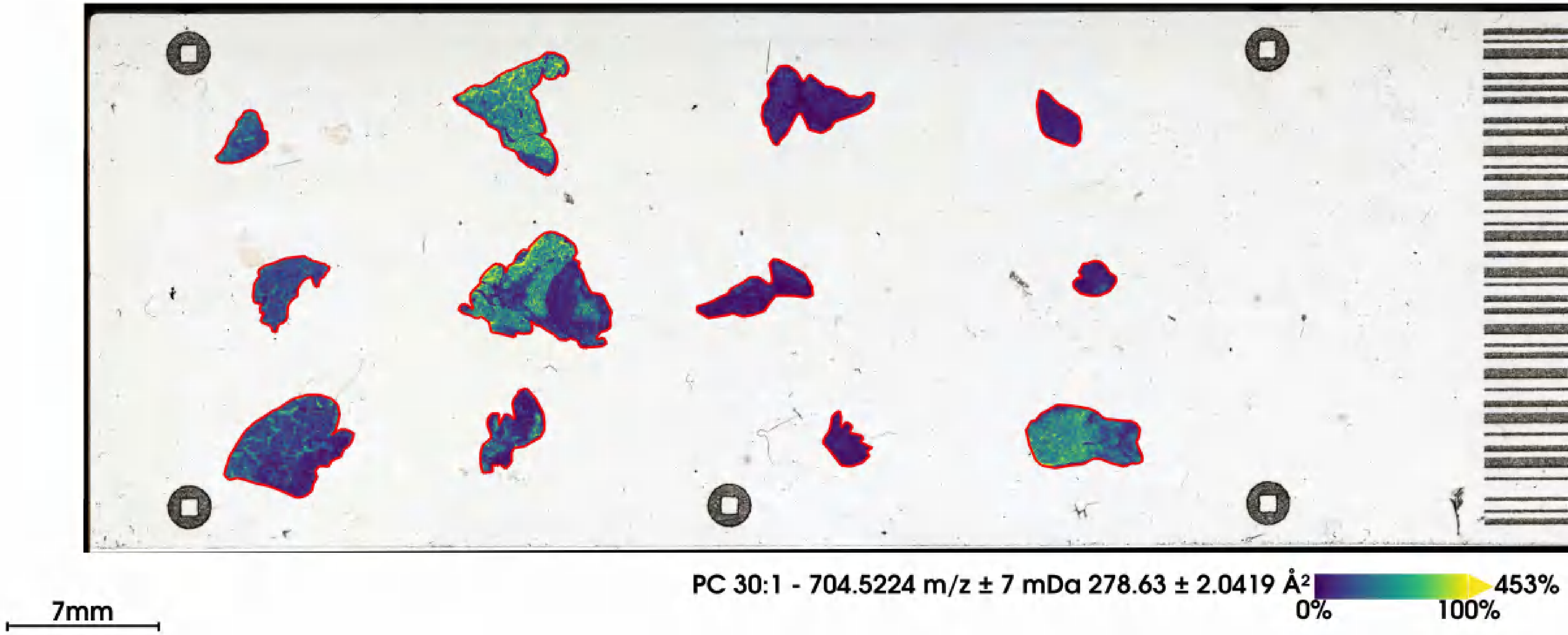
PC 30:1 - 704.5224 m/z ± 7 mDa 278.63 ± 2.0419 Å²
0%
100%
453%
7mm

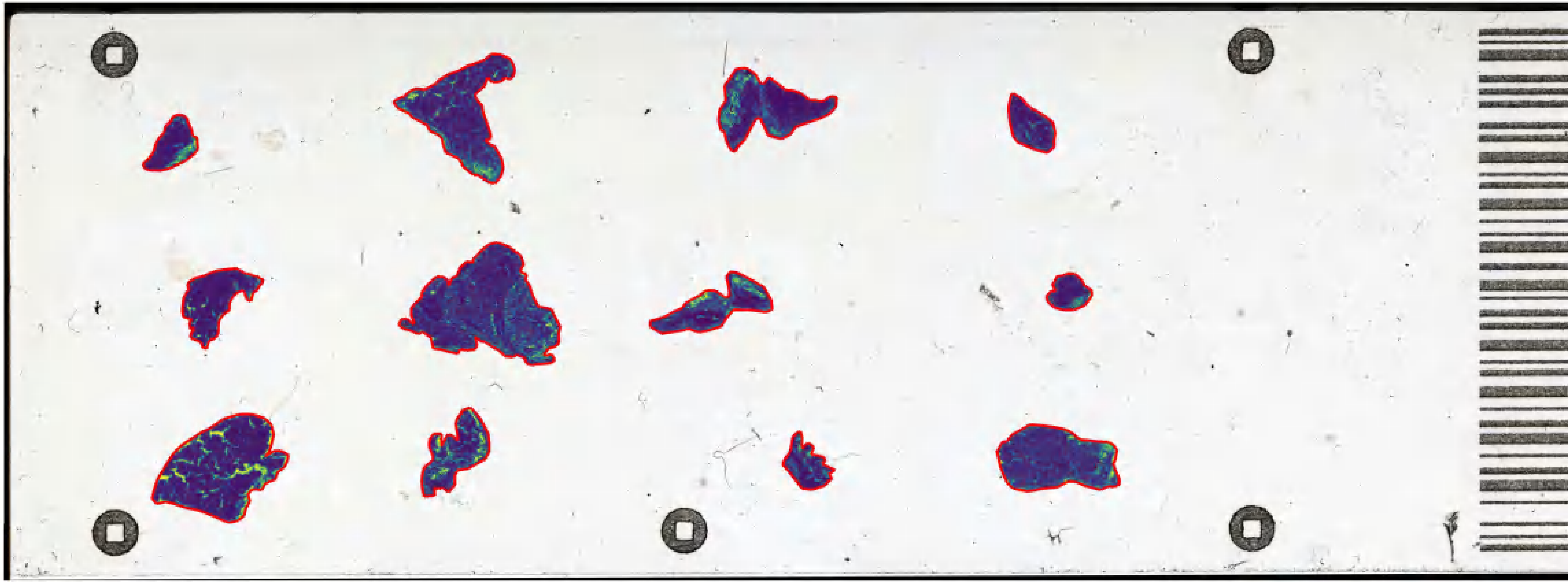

PS 30:1 - 706.4647 m/z ± 7.1 mDa 270.1981 ± 2.0418 Å² 0% 100% 221%

7mm

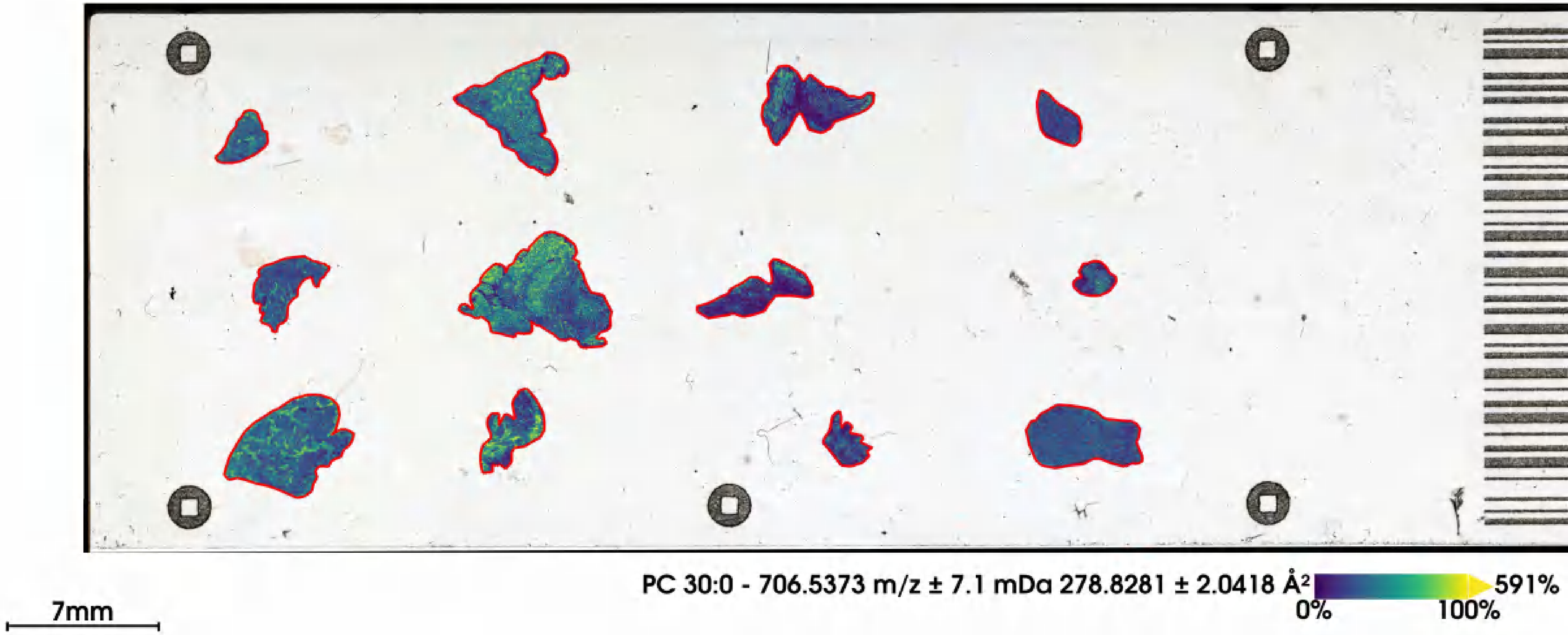
PC 30:0 - 706.5373 m/z ± 7.1 mDa 278.8281 ± 2.0418 Å²
0%
100%
591%
7mm

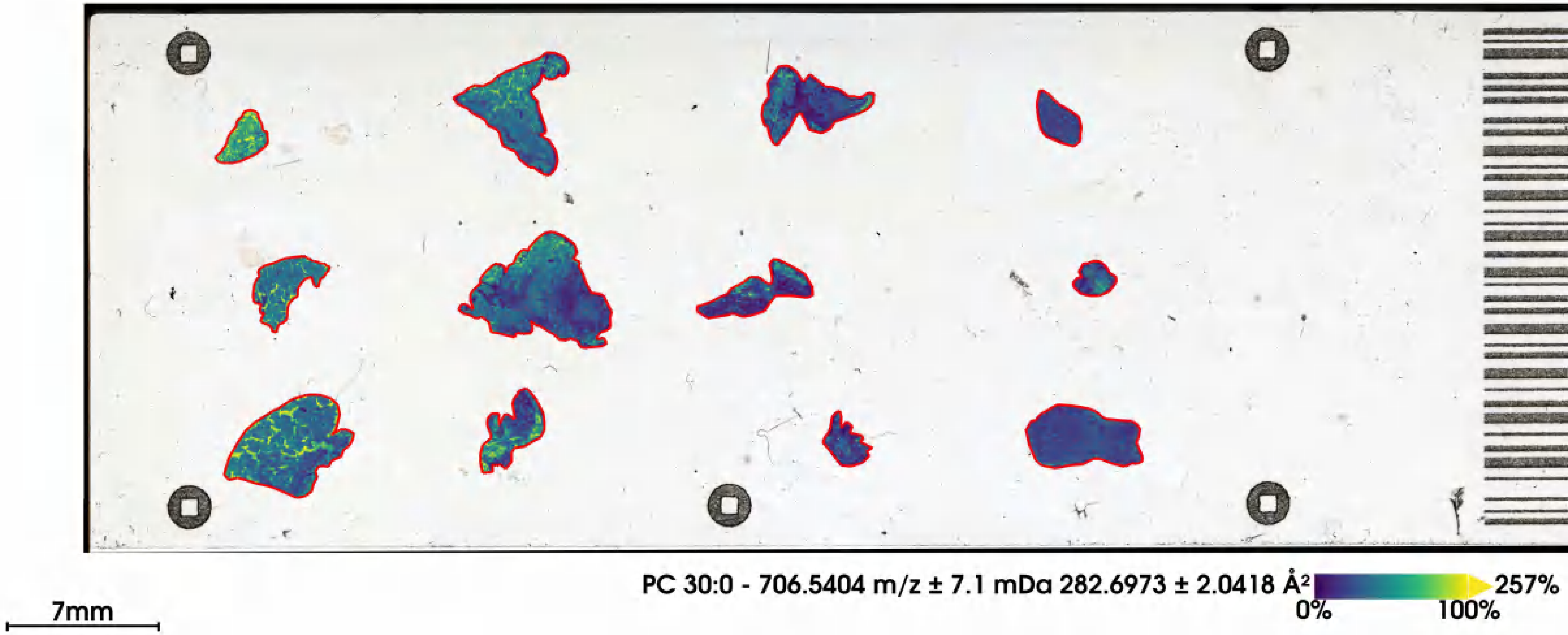
PC 30:0 - 706.5404 m/z ± 7.1 mDa 282.6973 ± 2.0418 Å²
0%
100%
257%
7mm

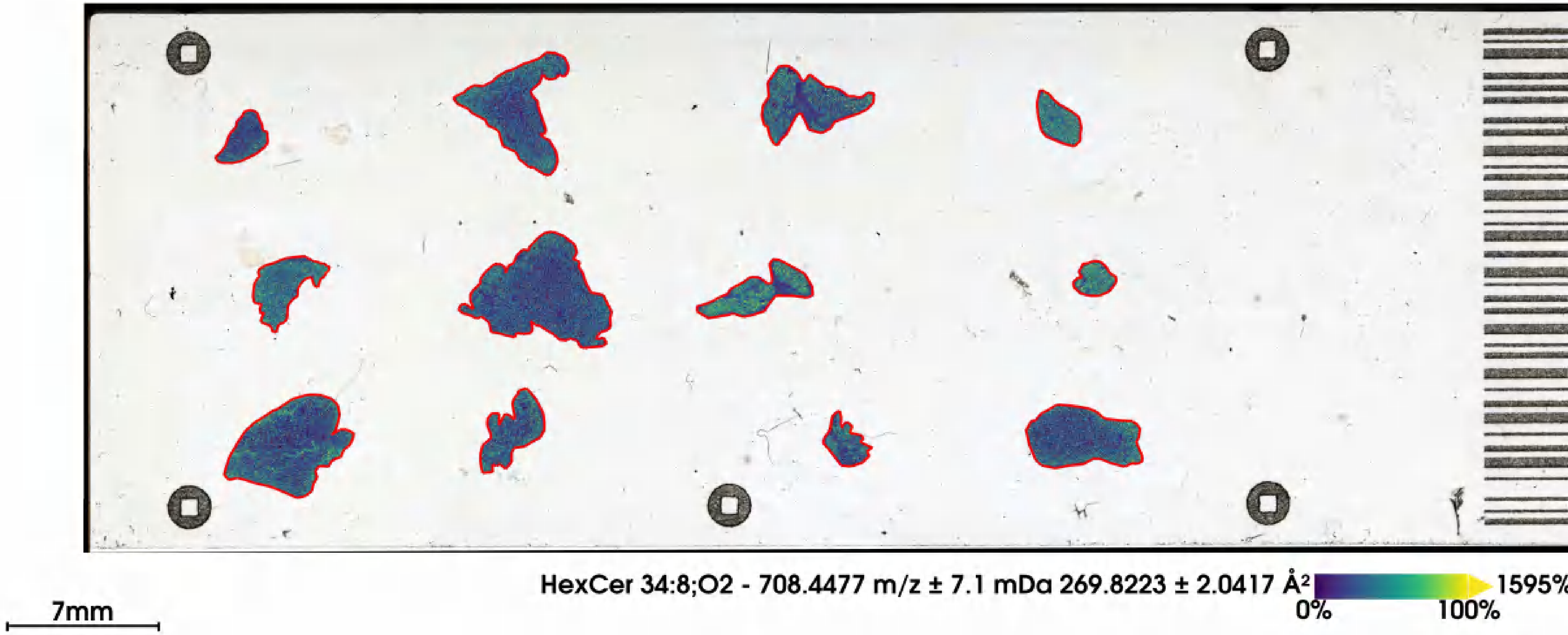
HexCer 34:8;O2 - 708.4477 m/z ± 7.1 mDa 269.8223 ± 2.0417 Å²
0%
100%
1595%
7mm

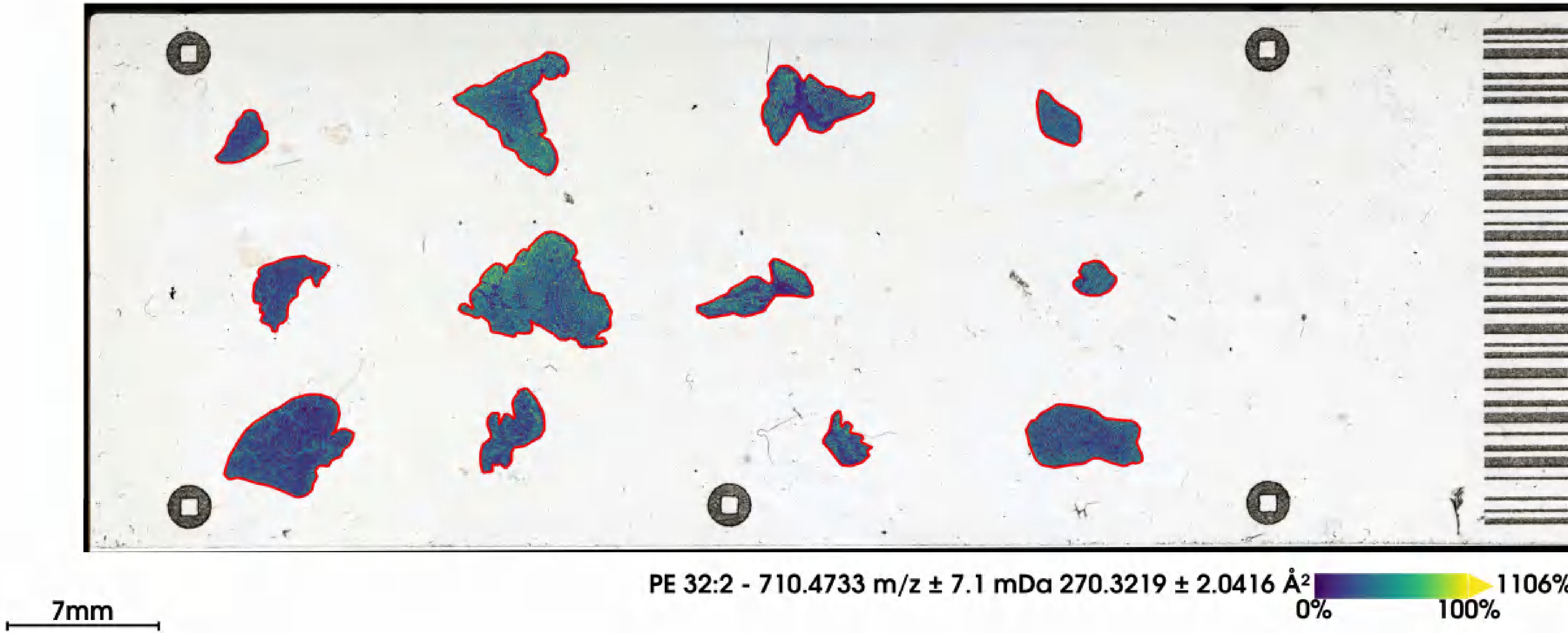

7mm
PE 32:2 - 710.4733 m/z ± 7.1 mDa 270.3219 ± 2.0416 Å²
0%
100%
1106%

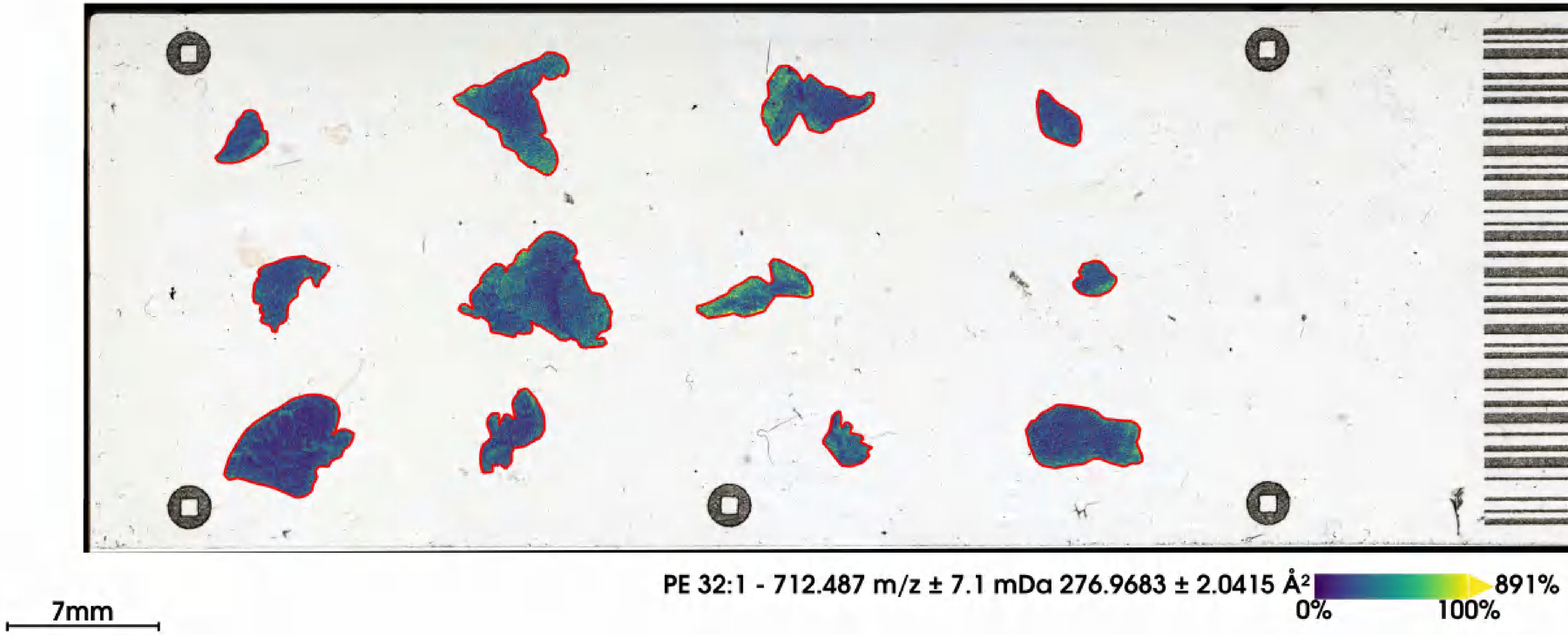
PE 32:1 - 712.487 m/z ± 7.1 mDa 276.9683 ± 2.0415 Å²
0%
100%
891%
7mm

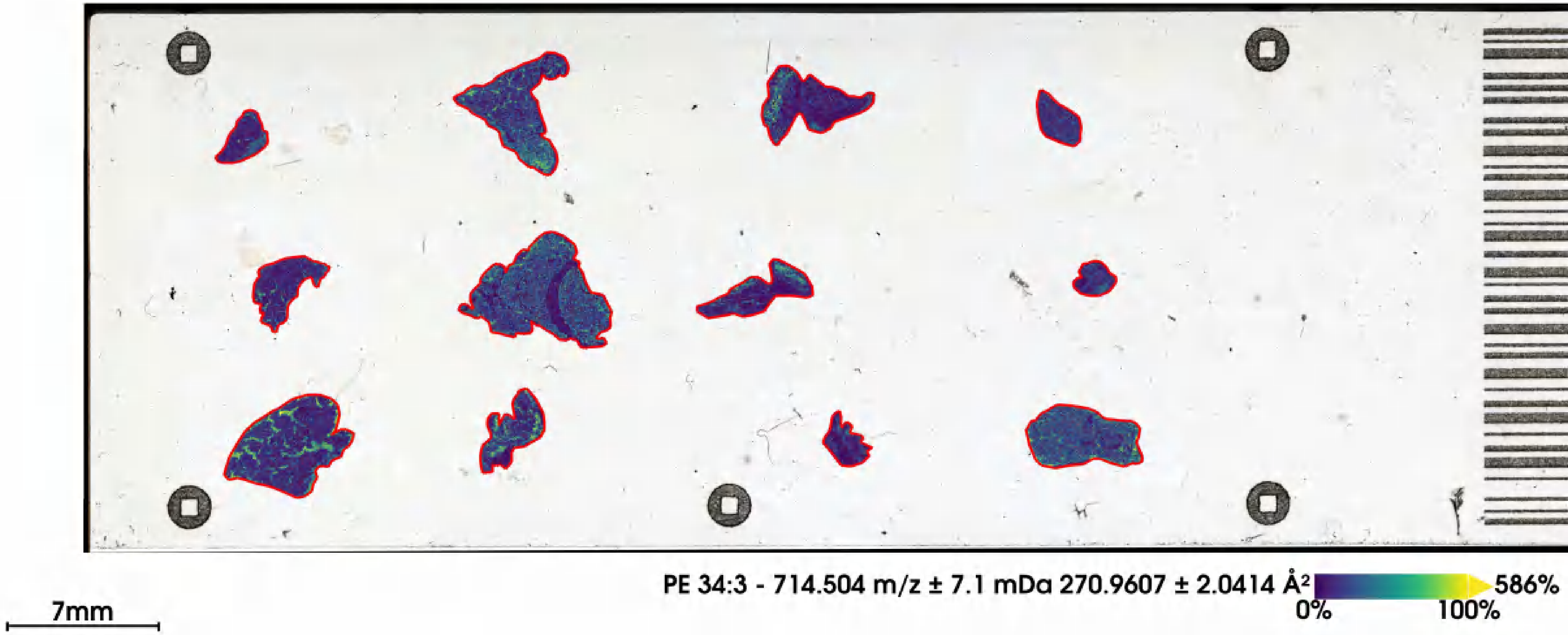
PE 34:3 - 714.504 m/z ± 7.1 mDa 270.9607 ± 2.0414 Å²
0%
100%
586%
7mm

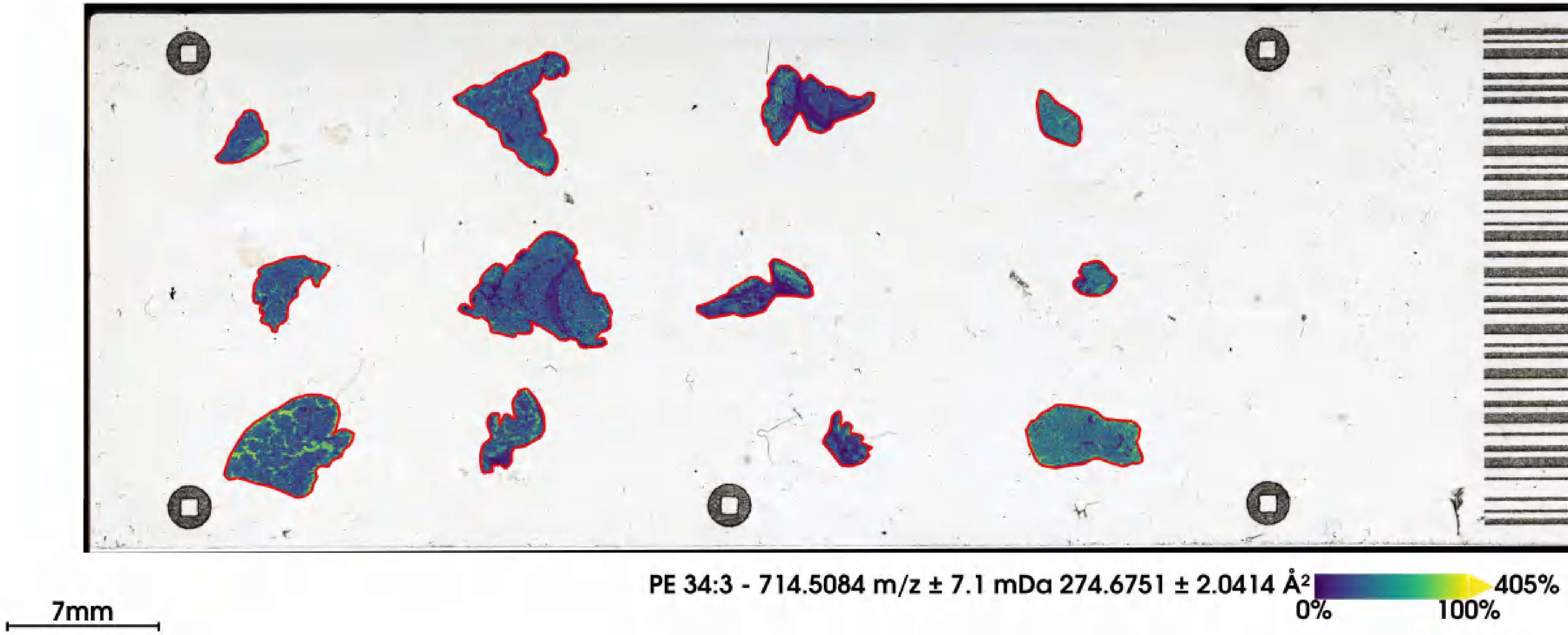

7mm
PE 34:3 - 714.5084 m/z ± 7.1 mDa 274.6751 ± 2.0414 Å²
0%
100%
405%

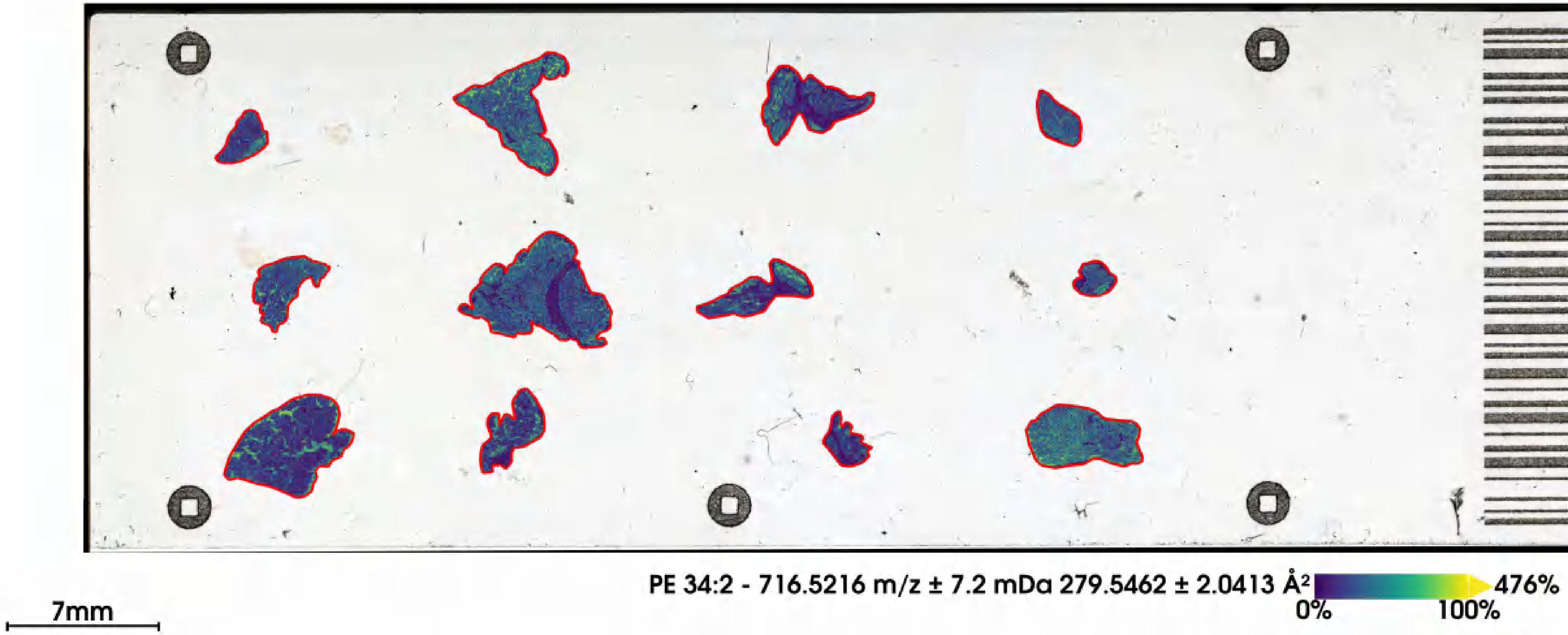

PE 34:2 - 716.5216 m/z ± 7.2 mDa 279.5462 ± 2.0413 Å²
0%
100%
476%
7mm

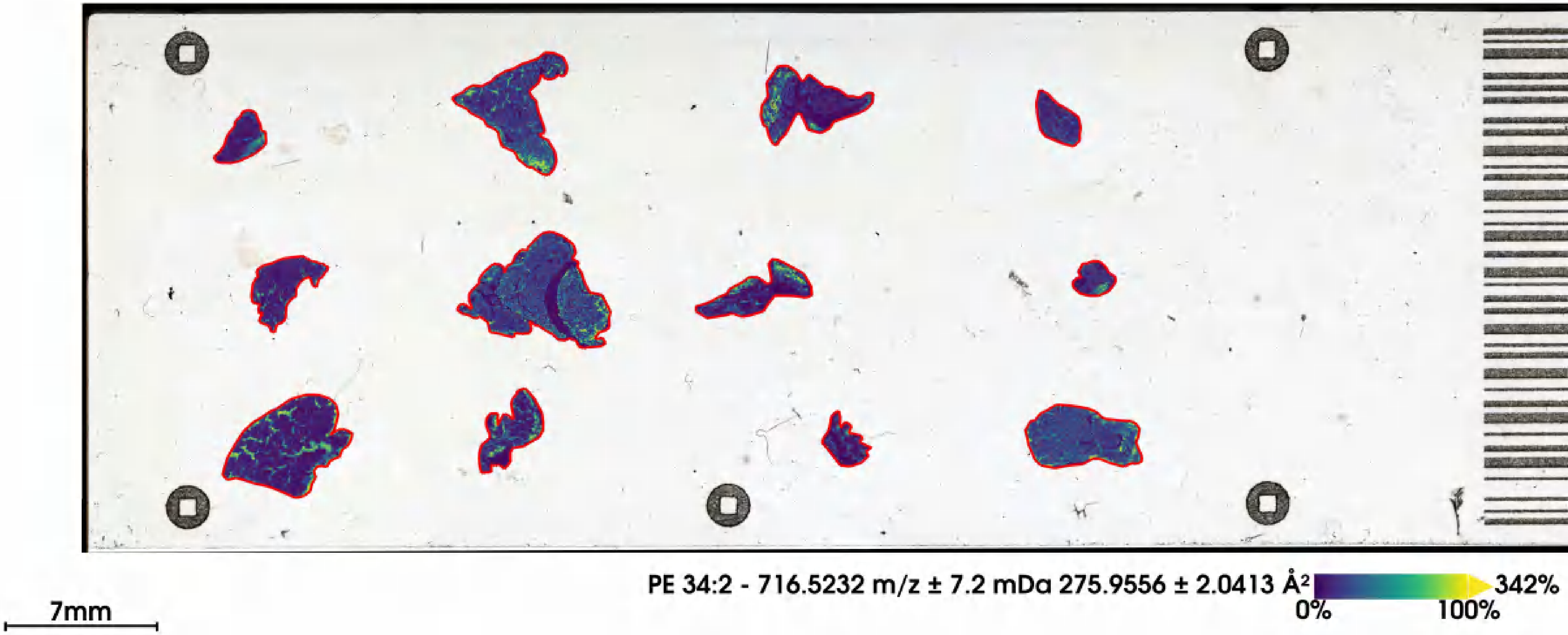

PE 34:2 - 716.5232 m/z ± 7.2 mDa 275.9556 ± 2.0413 Å²
0%
100%
342%
7mm

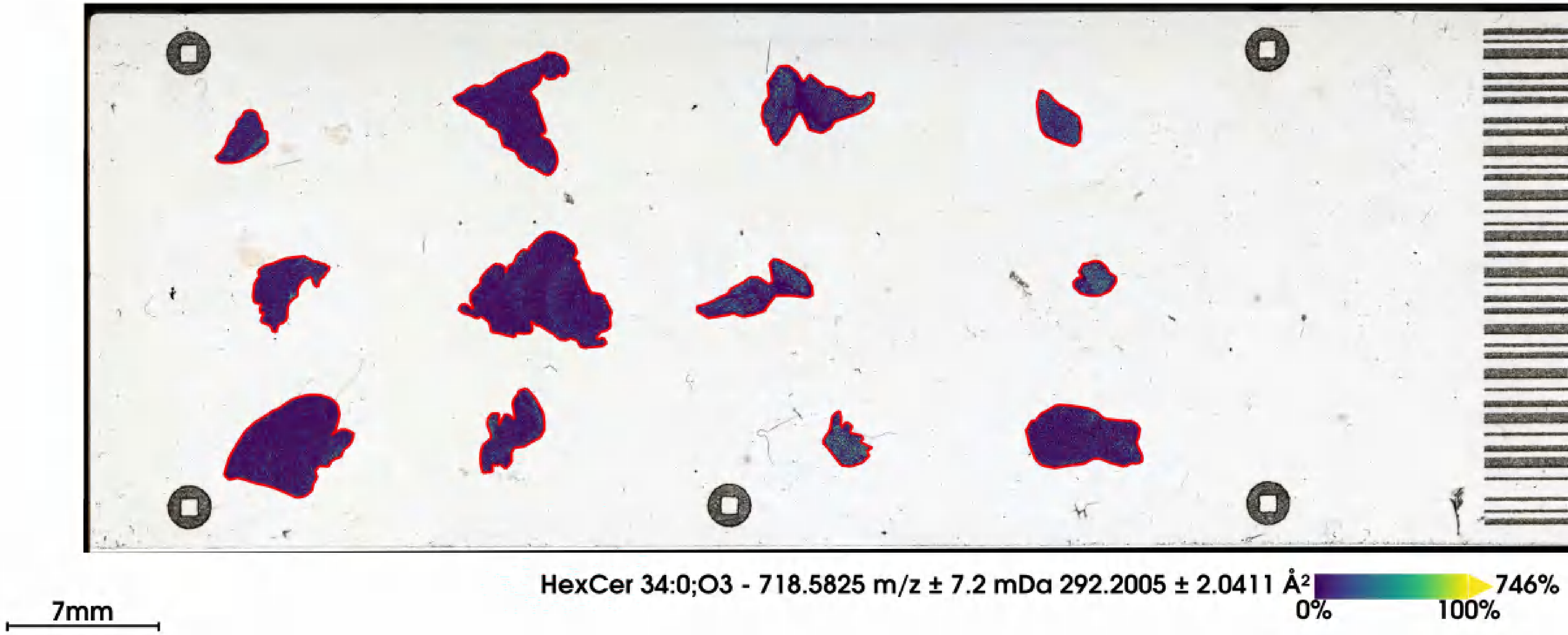

HexCer 34:0;O3 - 718.5825 m/z ± 7.2 mDa 292.2005 ± 2.0411 Å²
746%
0%
100%
7mm

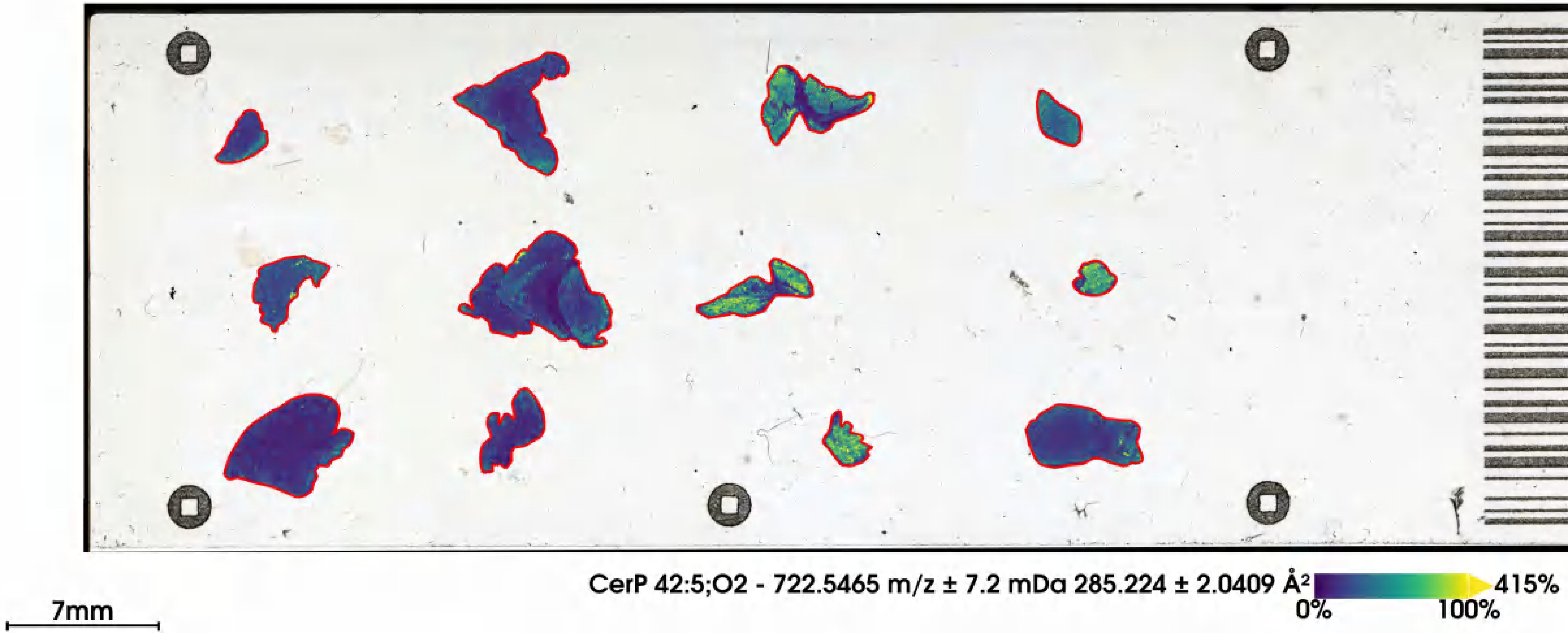
CerP 42:5;O2 - 722.5465 m/z ± 7.2 mDa 285.224 ± 2.0409 Å²
0%
100%
415%
7mm

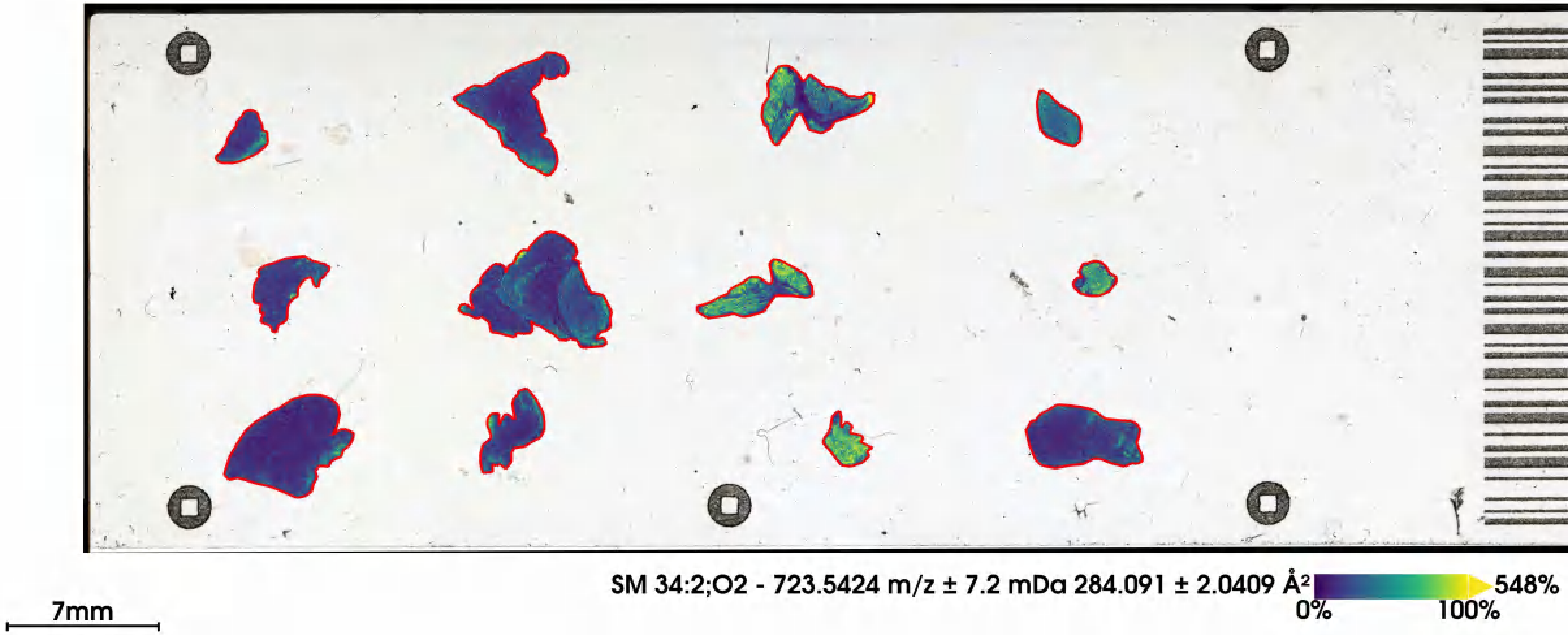
SM 34:2;O2 - 723.5424 m/z ± 7.2 mDa 284.091 ± 2.0409 Å²
0%
100%
548%
7mm

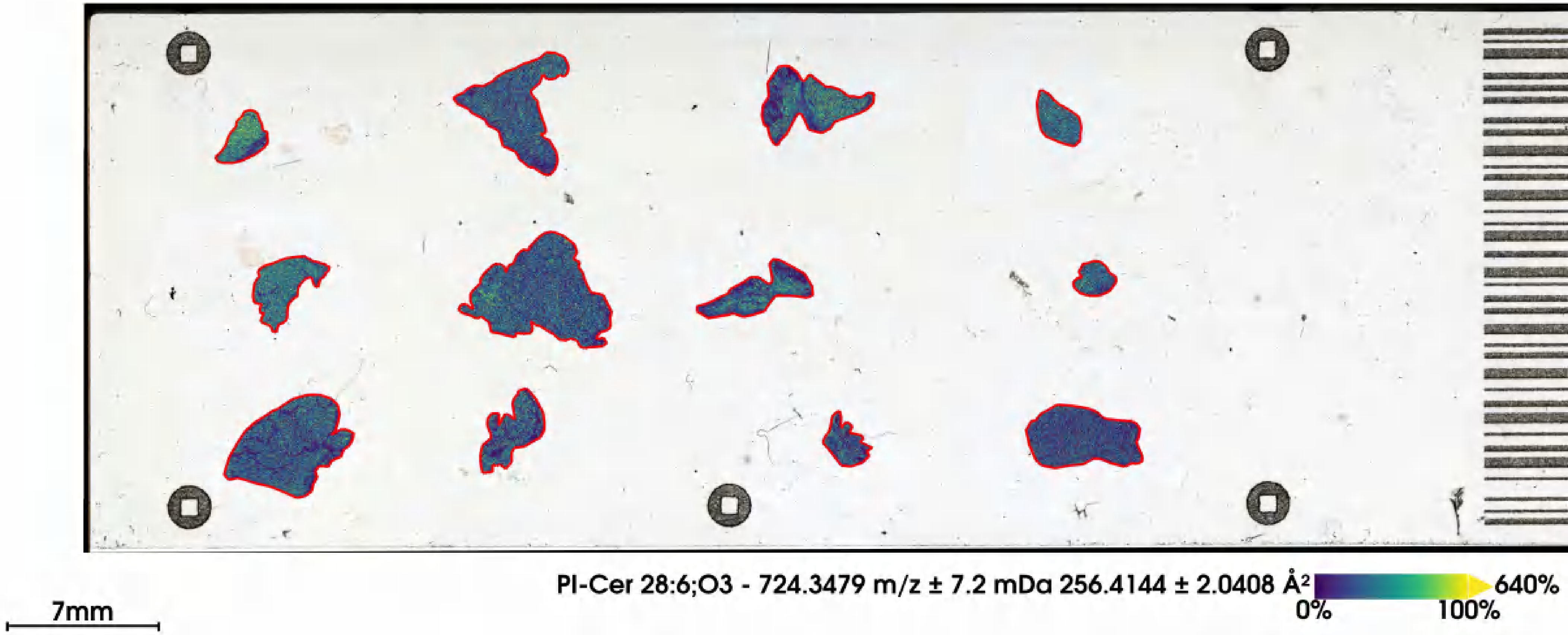
PI-Cer 28:6;O3 - 724.3479 m/z ± 7.2 mDa 256.4144 ± 2.0408 Å²
0%
100%
640%
7mm

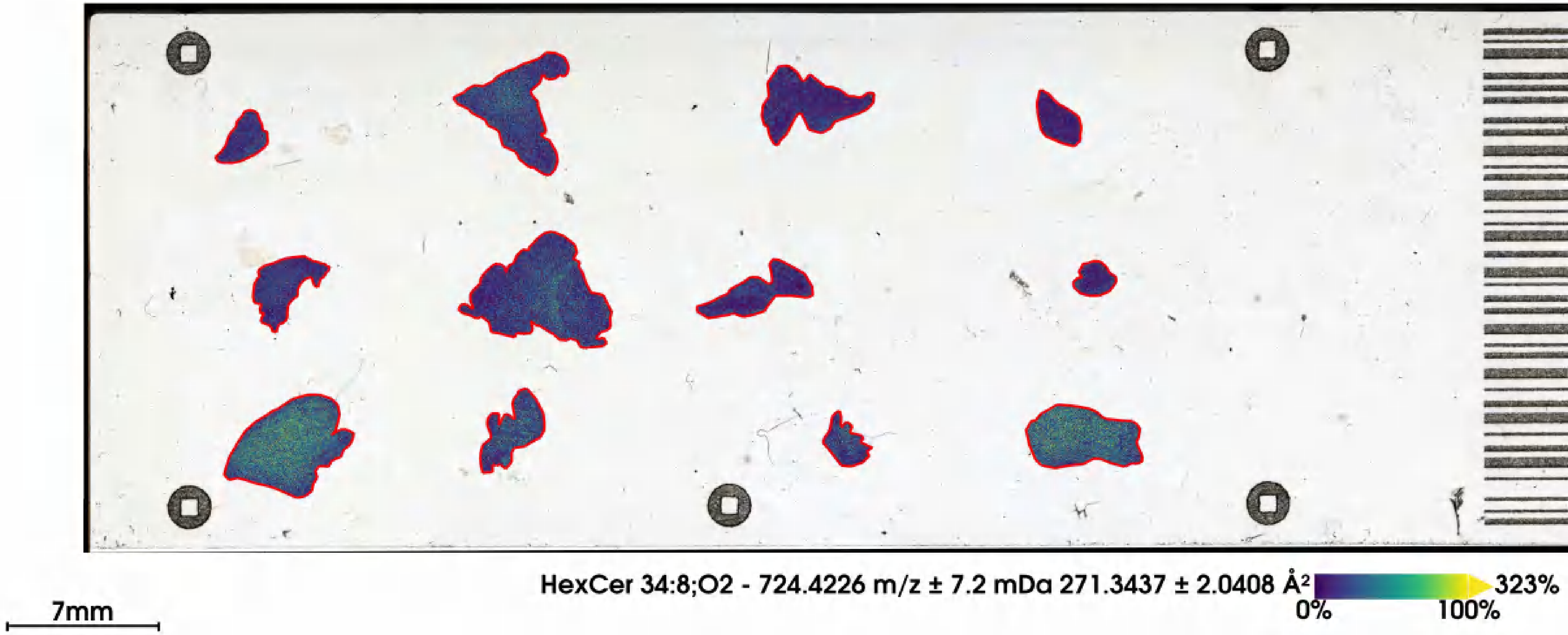
HexCer 34:8;O2 - 724.4226 m/z ± 7.2 mDa 271.3437 ± 2.0408 Å²
0%
100%
323%
7mm

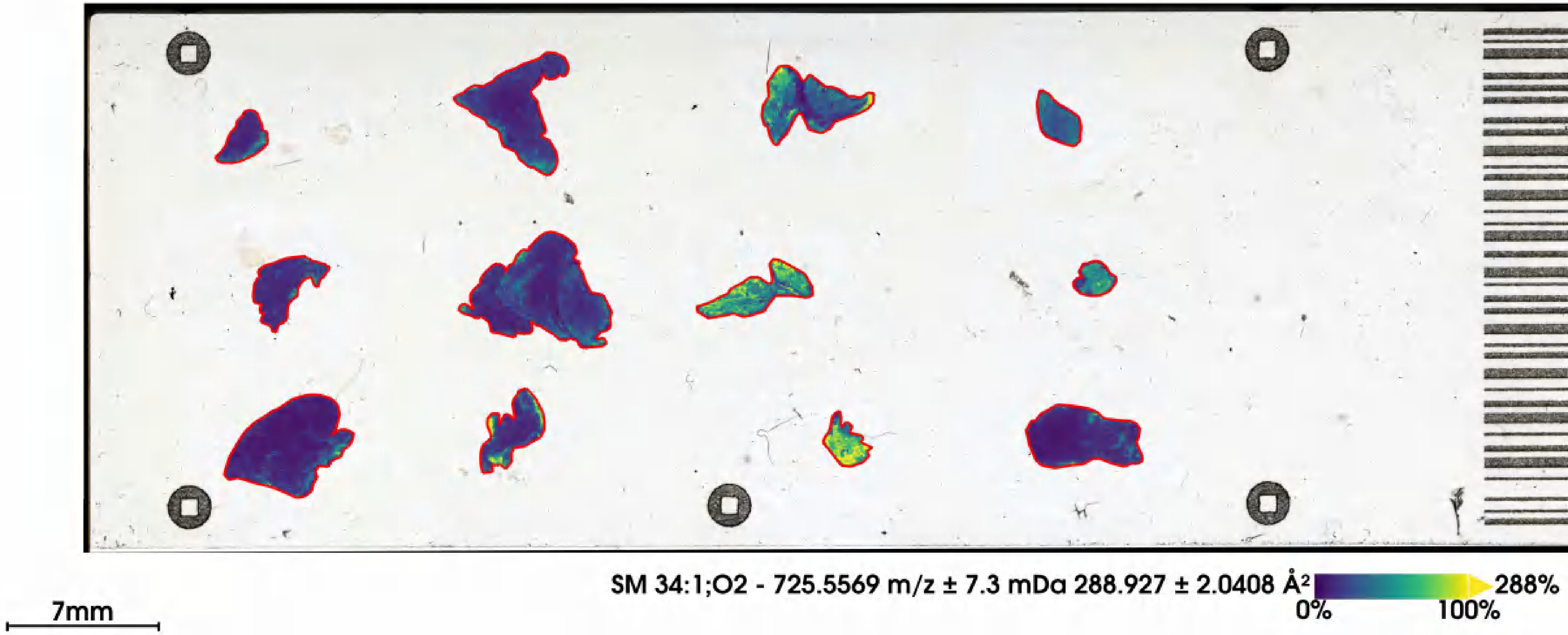
SM 34:1;O2 - 725.5569 m/z ± 7.3 mDa 288.927 ± 2.0408 Å²
288%
0%
100%
7mm

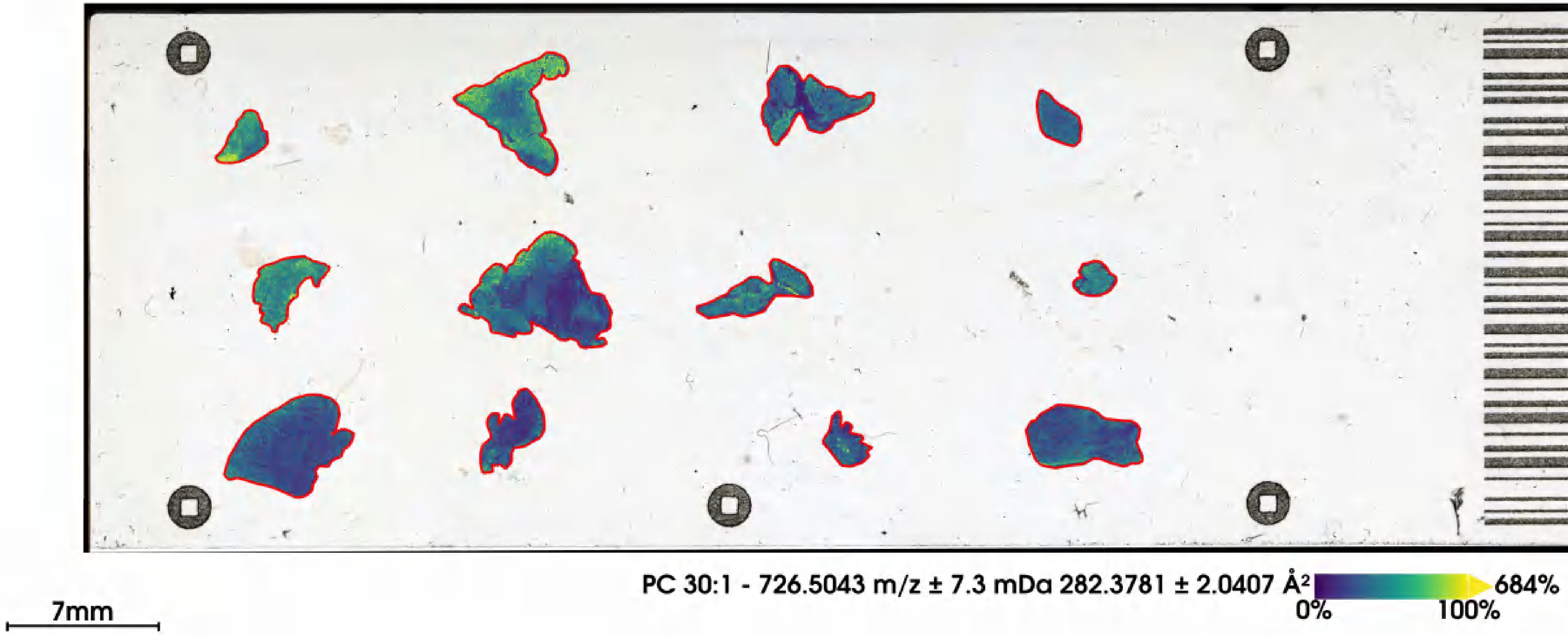
PC 30:1 - 726.5043 m/z ± 7.3 mDa 282.3781 ± 2.0407 Å²
0%
100%
684%
7mm

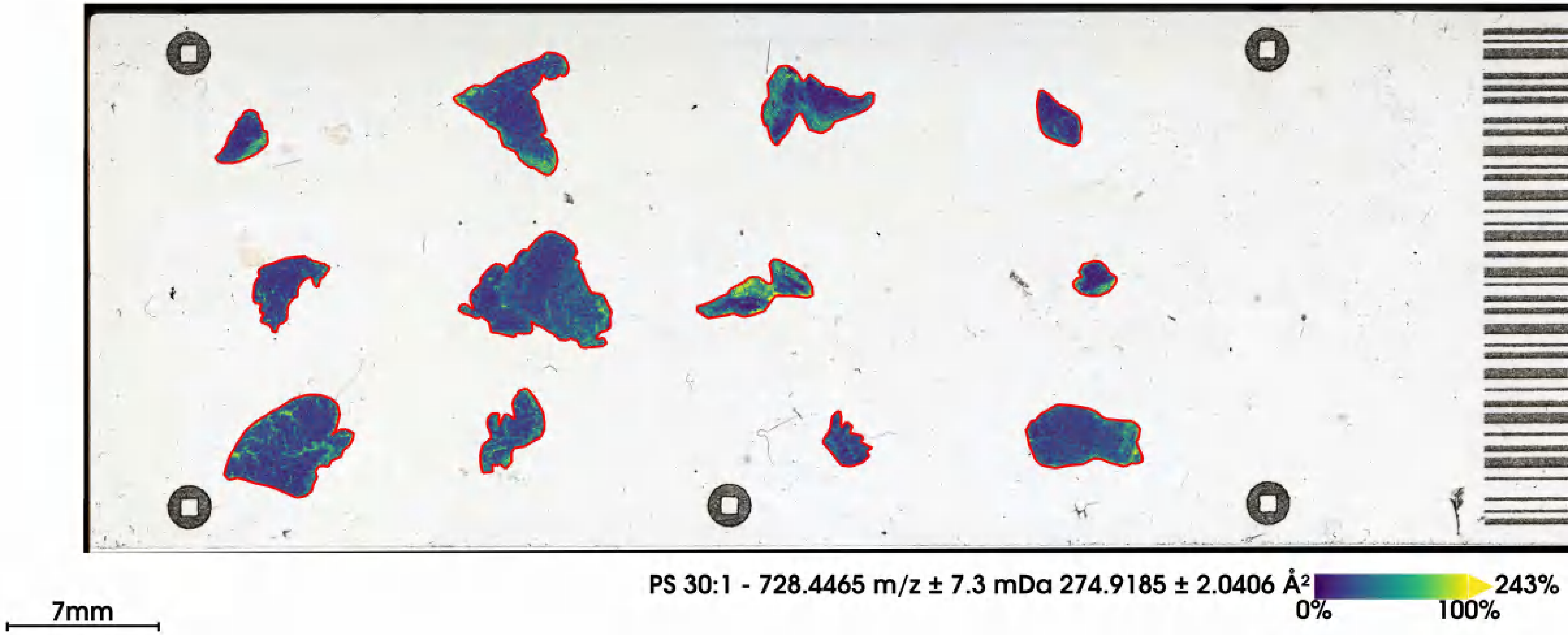

PS 30:1 - 728.4465 m/z ± 7.3 mDa 274.9185 ± 2.0406 Å²
0%
100%
243%
7mm

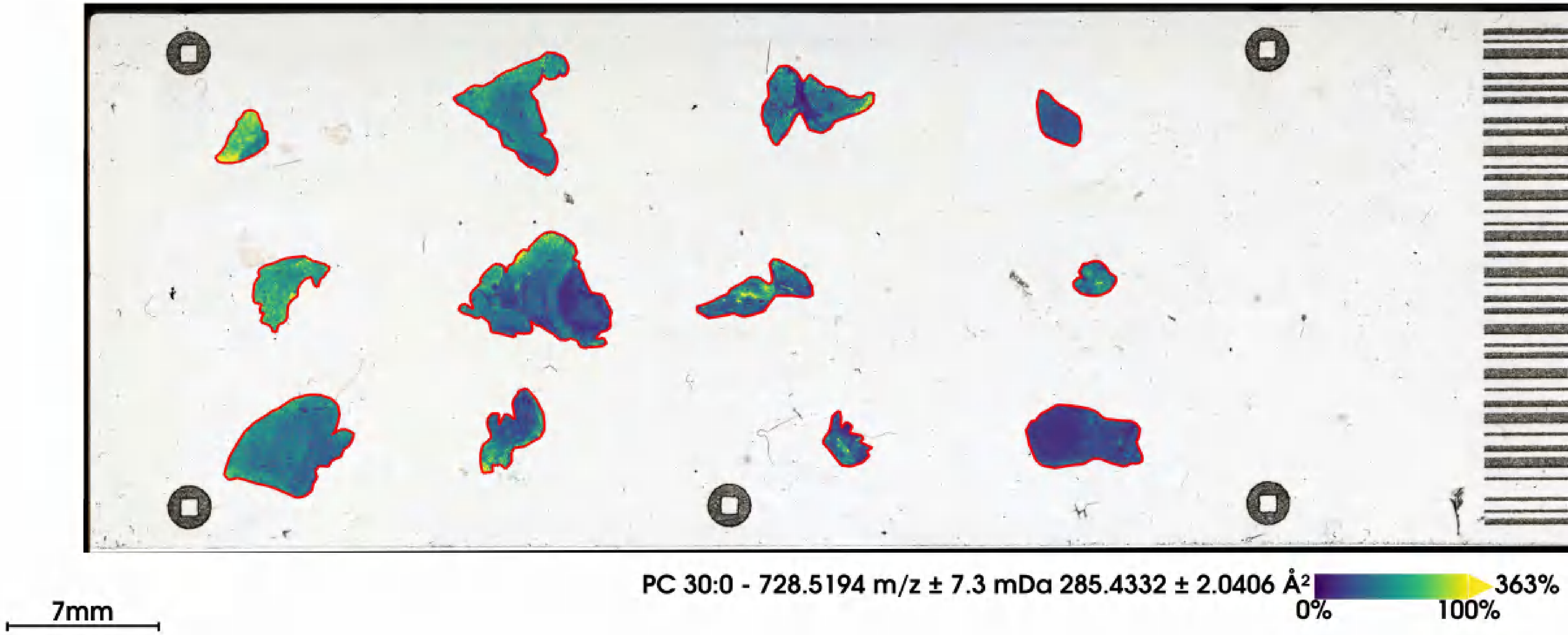

PC 30:0 - 728.5194 m/z ± 7.3 mDa 285.4332 ± 2.0406 Å²
0%
100%
363%
7mm

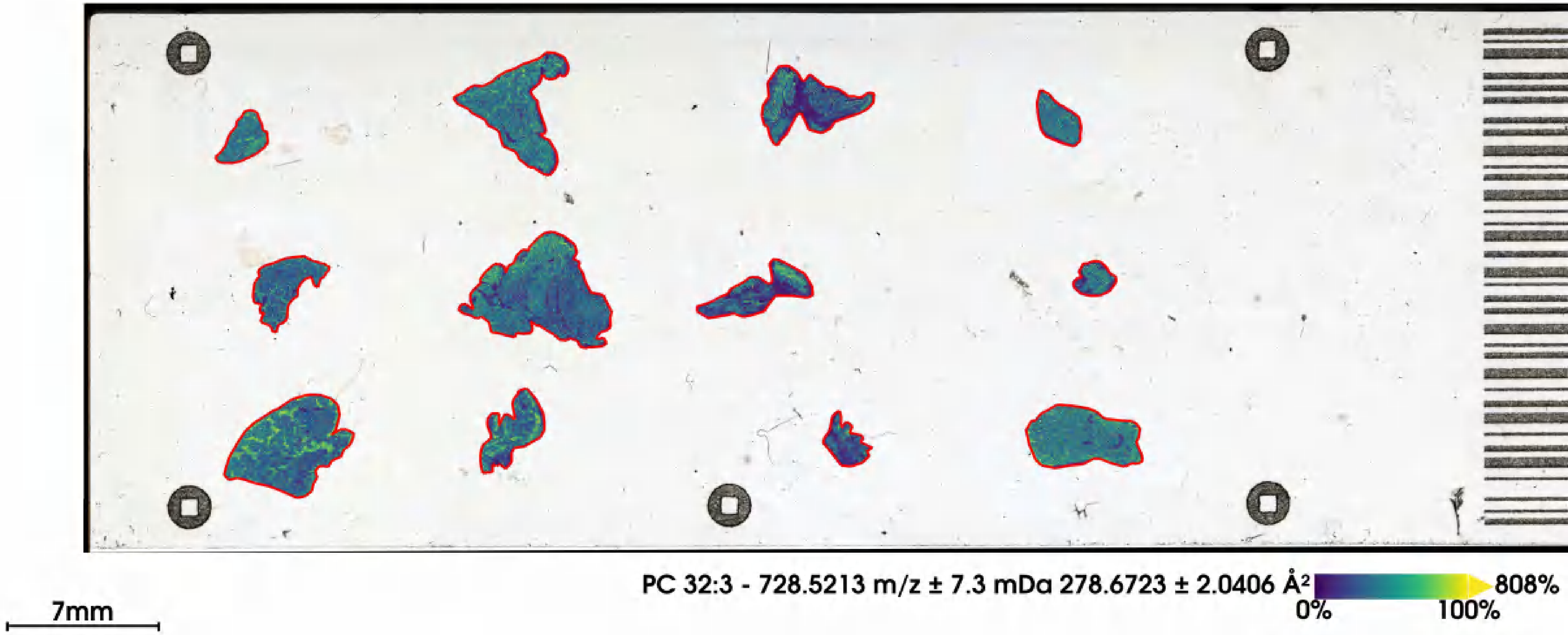
PC 32:3 - 728.5213 m/z ± 7.3 mDa 278.6723 ± 2.0406 Å²
0%
100%
808%
7mm

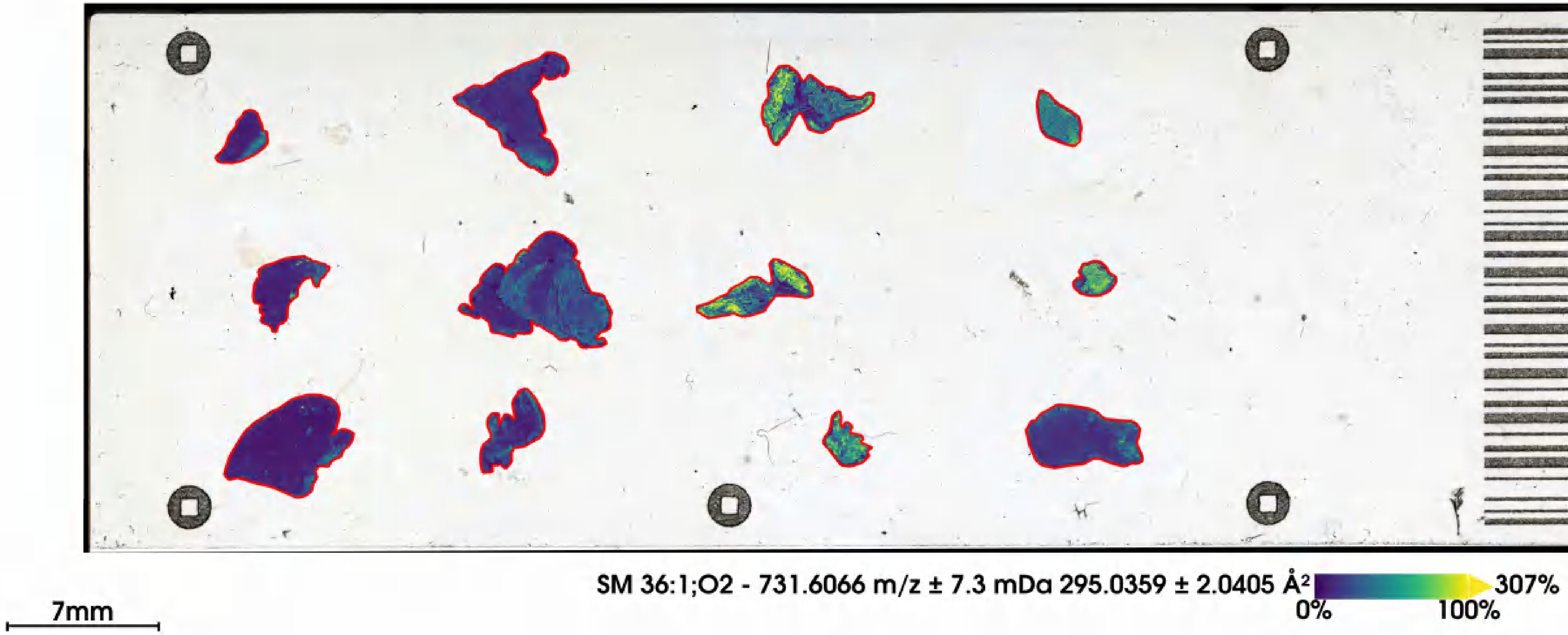
7mm
SM 36:1;O2 - 731.6066 m/z ± 7.3 mDa 295.0359 ± 2.0405 Å²
0%
100%
307%

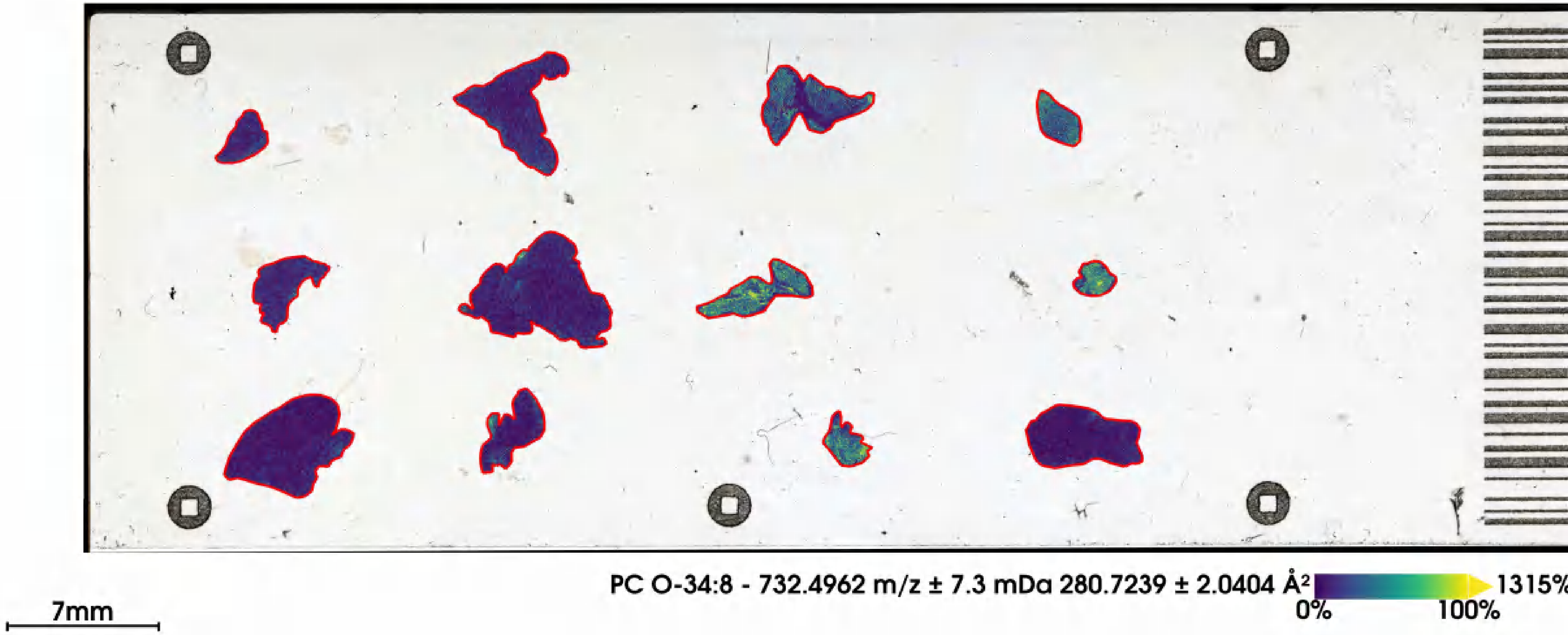
PC O-34:8 - 732.4962 m/z ± 7.3 mDa 280.7239 ± 2.0404 Å²
0%
100%
1315%
7mm

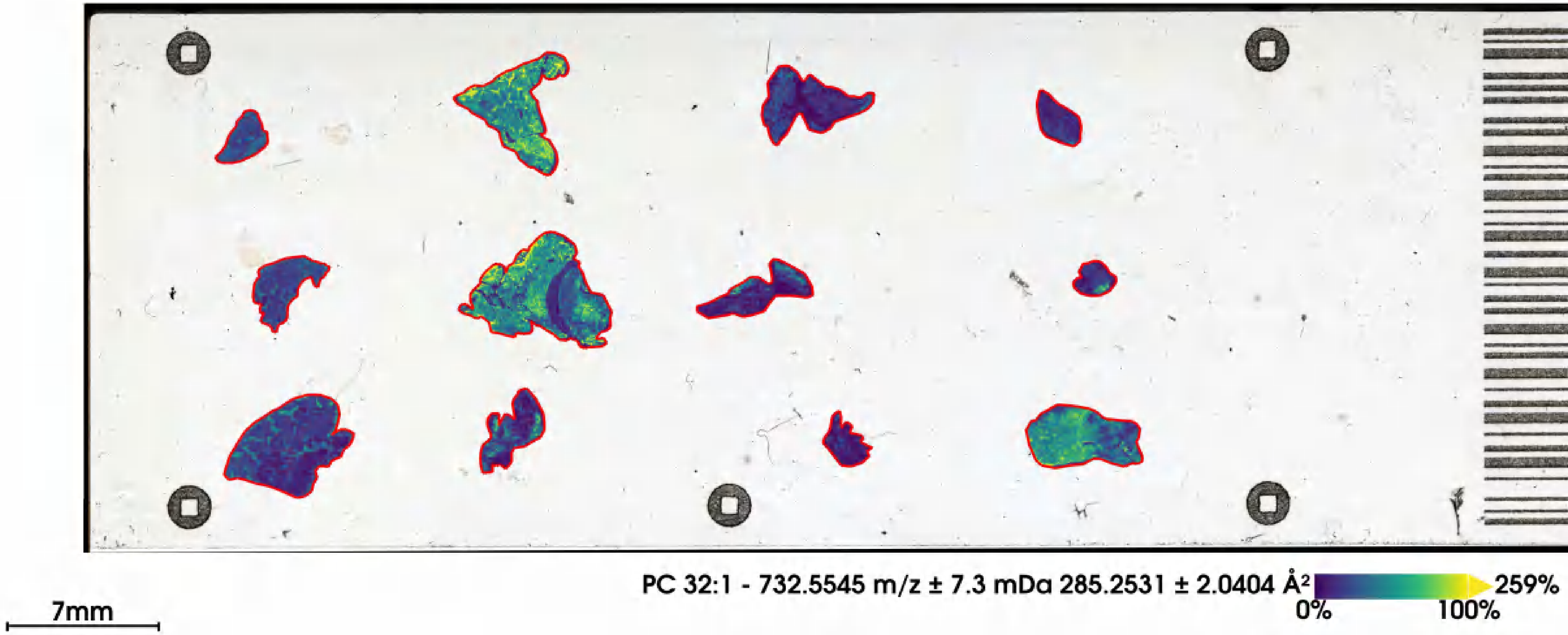
PC 32:1 - 732.5545 m/z ± 7.3 mDa 285.2531 ± 2.0404 Å²
0%
100%
259%
7mm

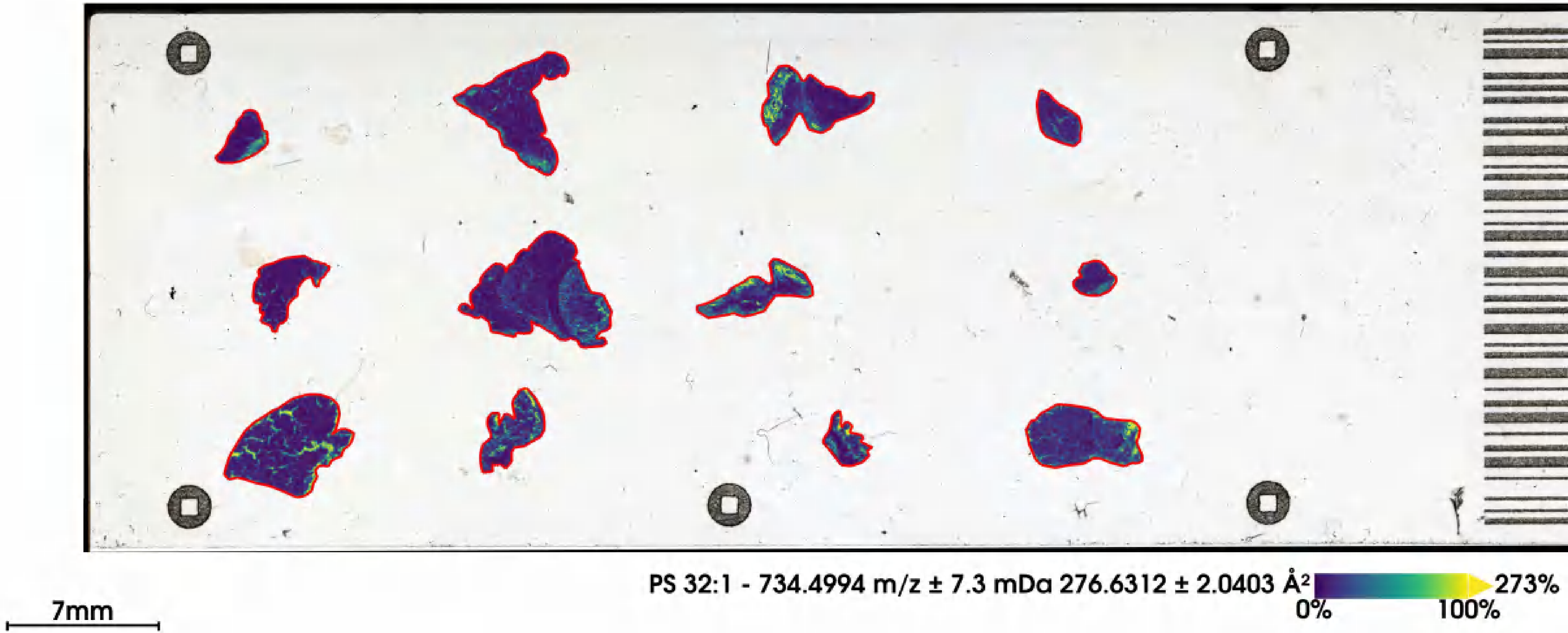

PS 32:1 - 734.4994 m/z ± 7.3 mDa 276.6312 ± 2.0403 Å²
0%
100%
273%
7mm

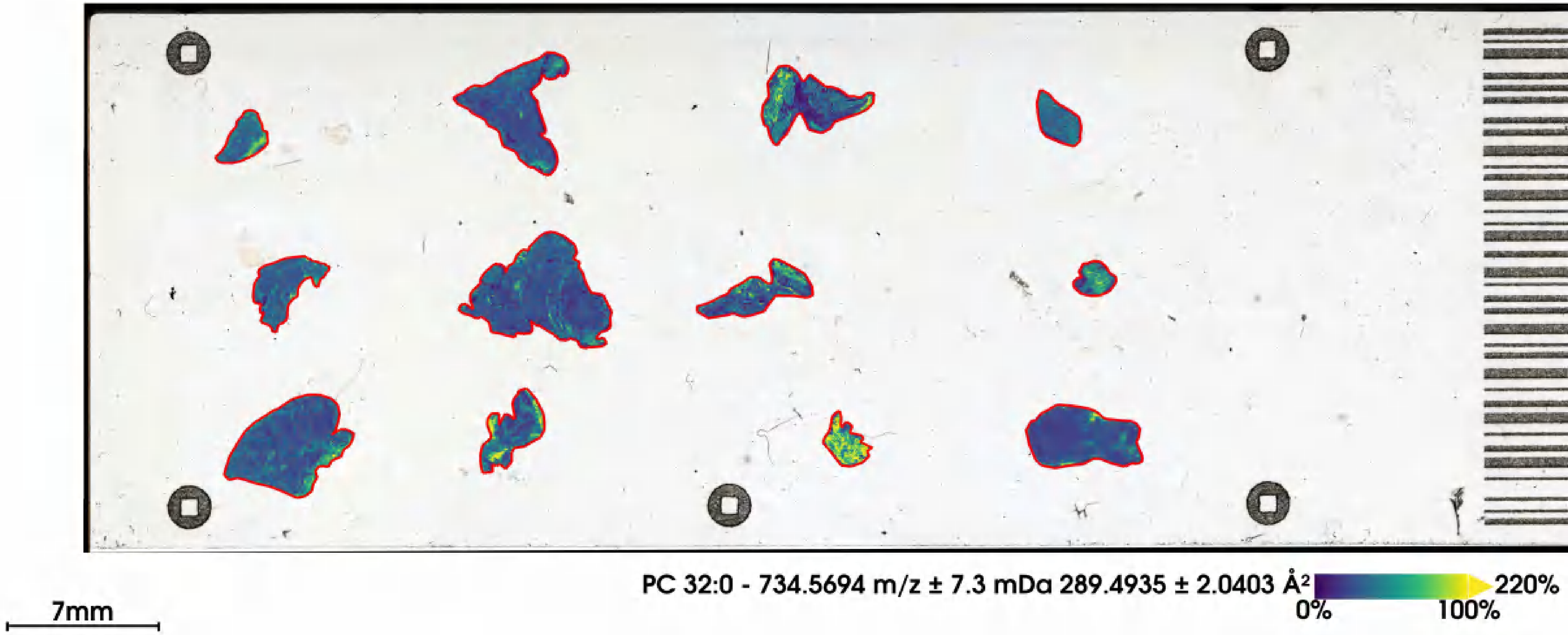

PC 32:0 - 734.5694 m/z ± 7.3 mDa 289.4935 ± 2.0403 Å²
0%
100%
220%
7mm

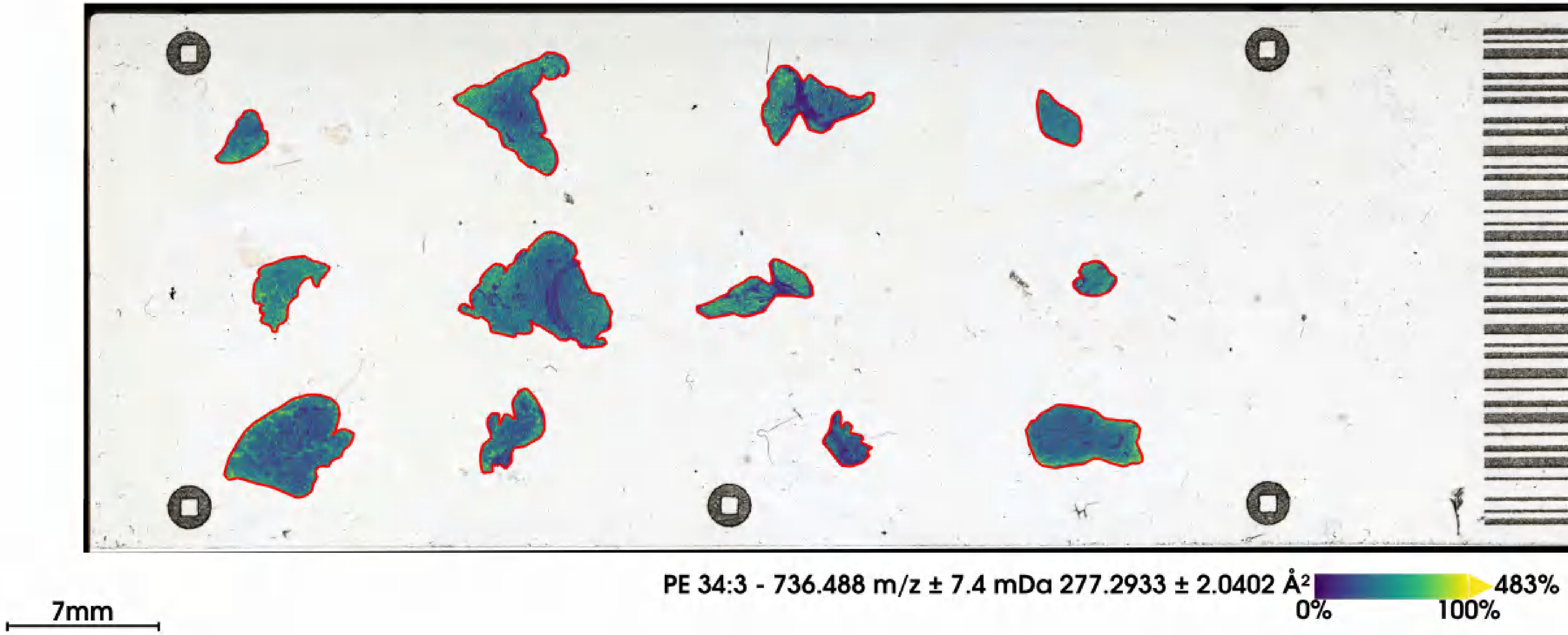

PE 34:3 - 736.488 m/z ± 7.4 mDa 277.2933 ± 2.0402 Å²
0%
100%
483%
7mm

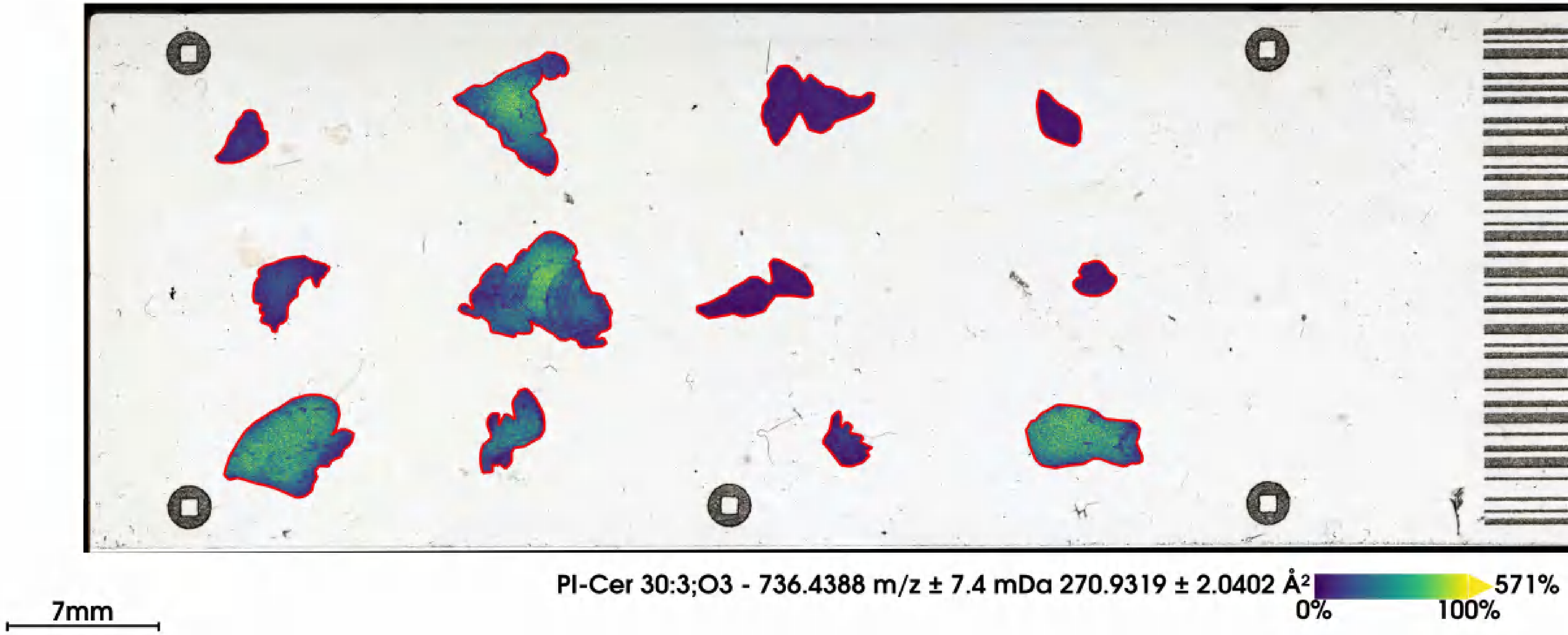
PI-Cer 30:3;O3 - 736.4388 m/z ± 7.4 mDa 270.9319 ± 2.0402 Å²
0%
100%
571%
7mm

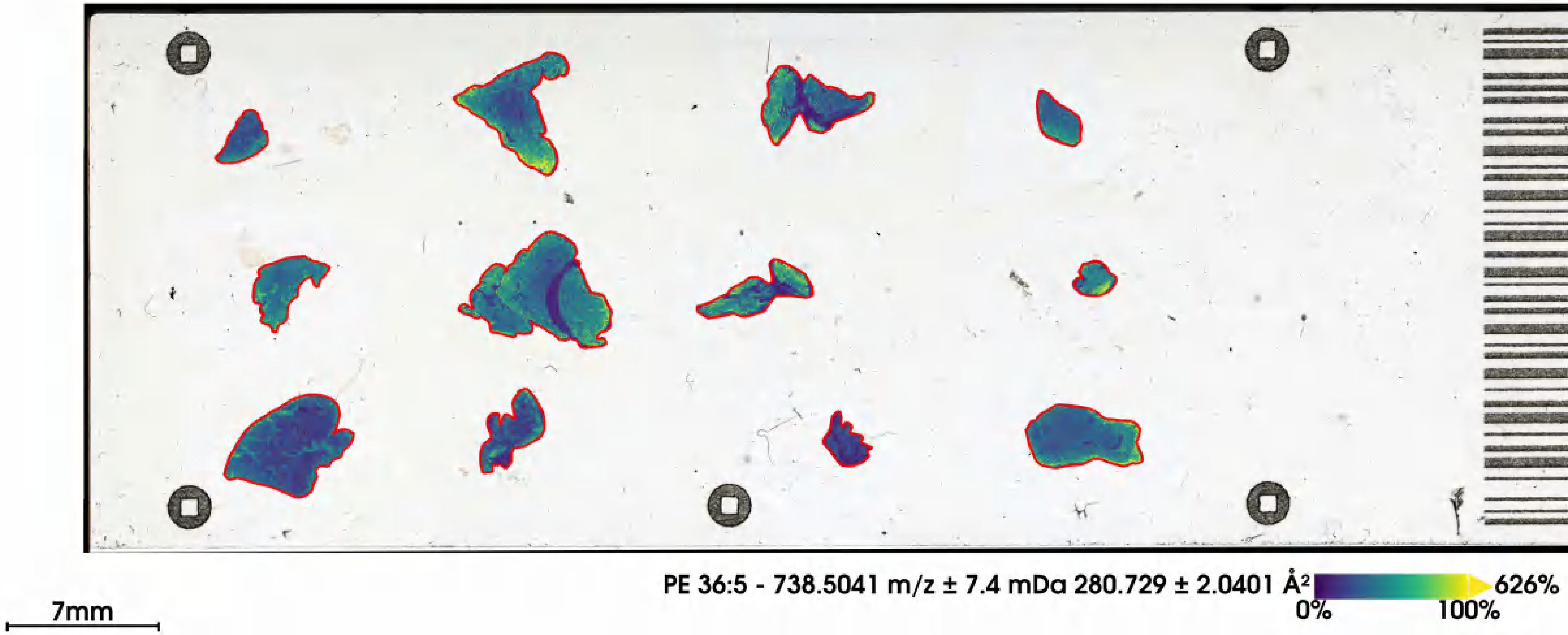

7mm
PE 36:5 - 738.5041 m/z ± 7.4 mDa 280.729 ± 2.0401 Å²
0%
100%
626%

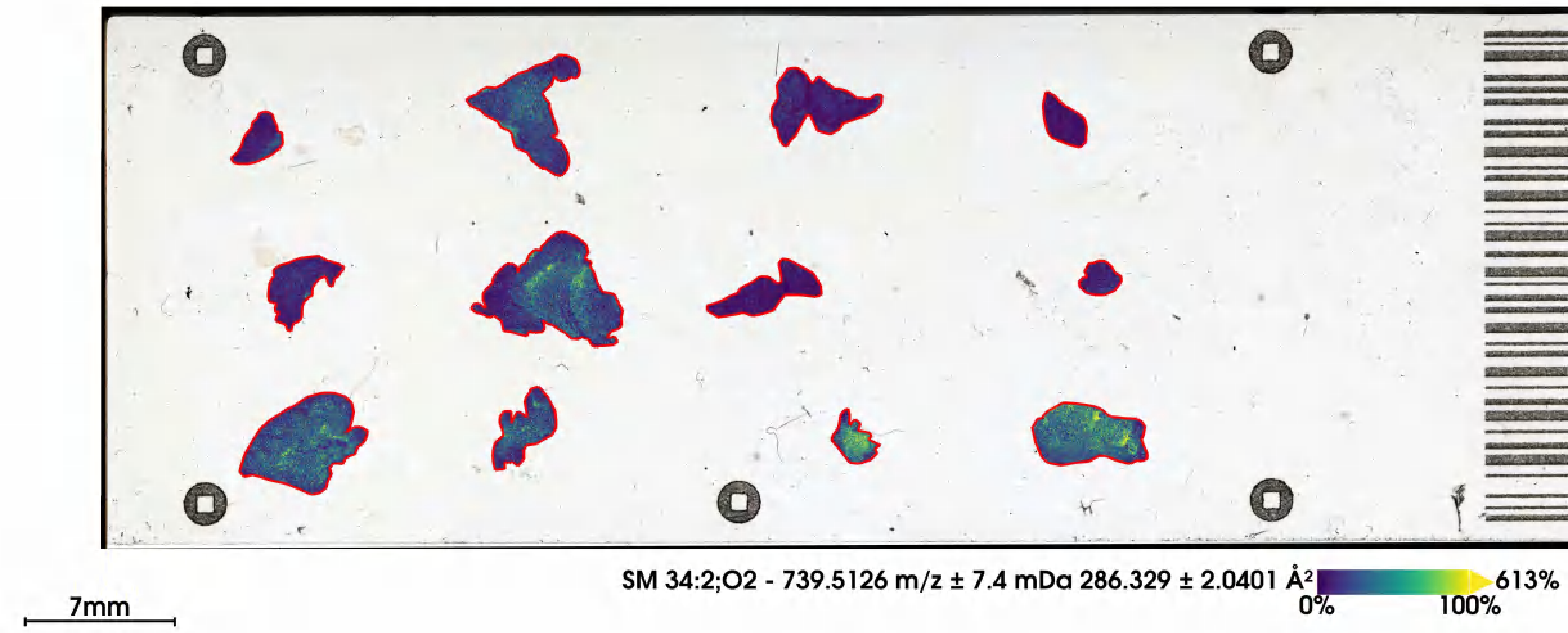
SM 34:2;O2 - 739.5126 m/z ± 7.4 mDa 286.329 ± 2.0401 Å²
0%
100%
613%
7mm

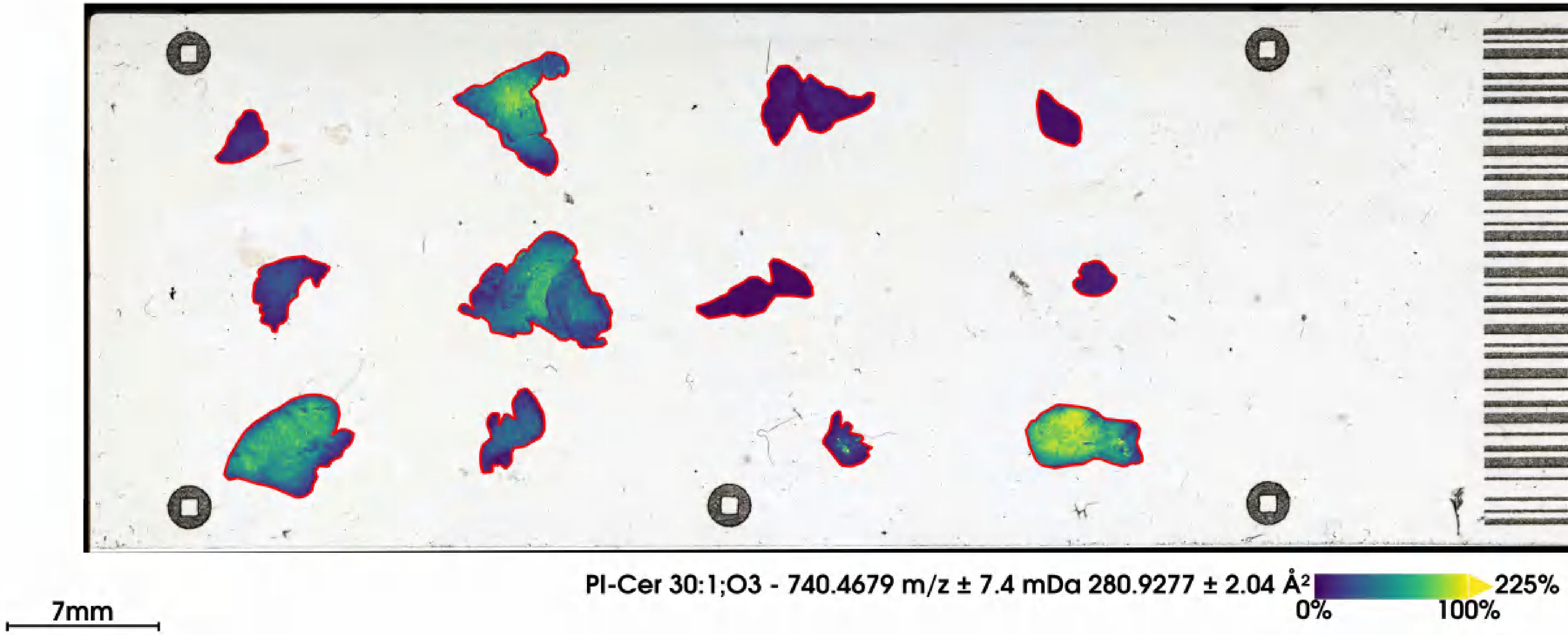
PI-Cer 30:1;O3 - 740.4679 m/z ± 7.4 mDa 280.9277 ± 2.04 Å²
225%
0%
100%
7mm

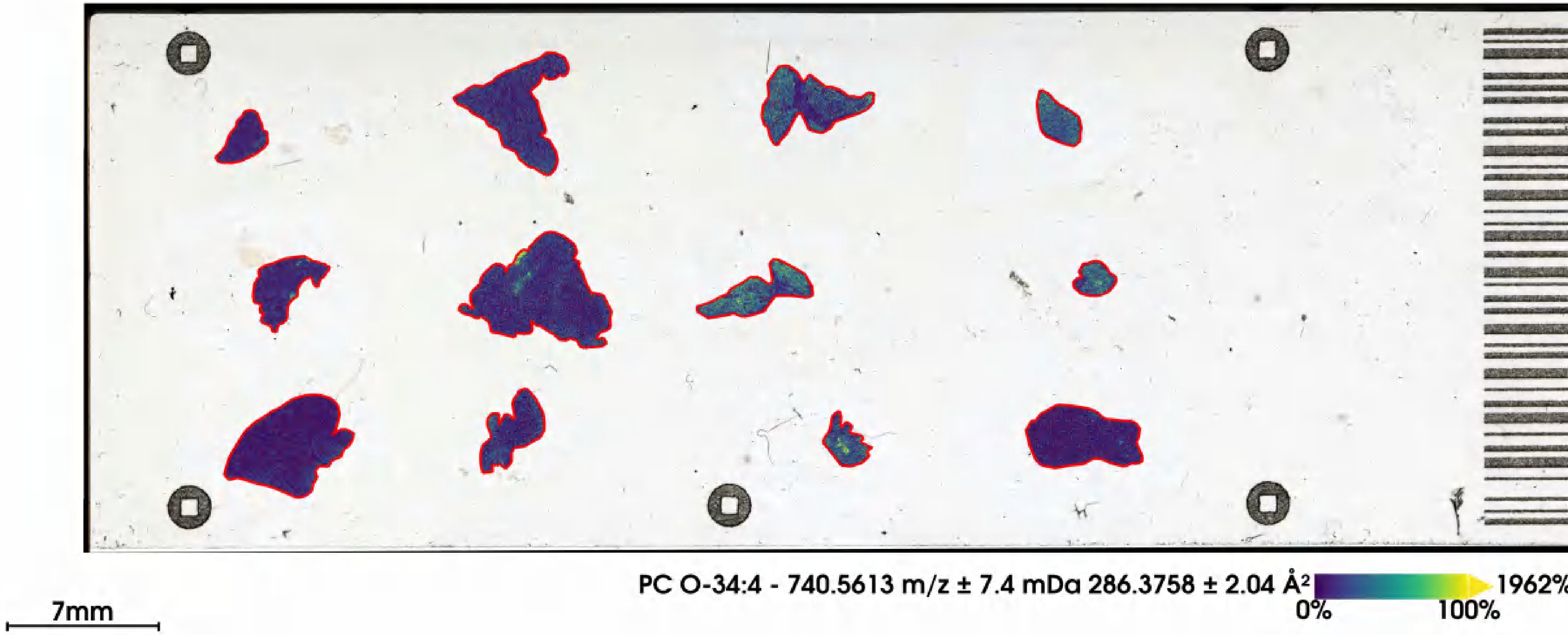
PC O-34:4 - 740.5613 m/z ± 7.4 mDa 286.3758 ± 2.04 Å²
0%
100%
1962%
7mm

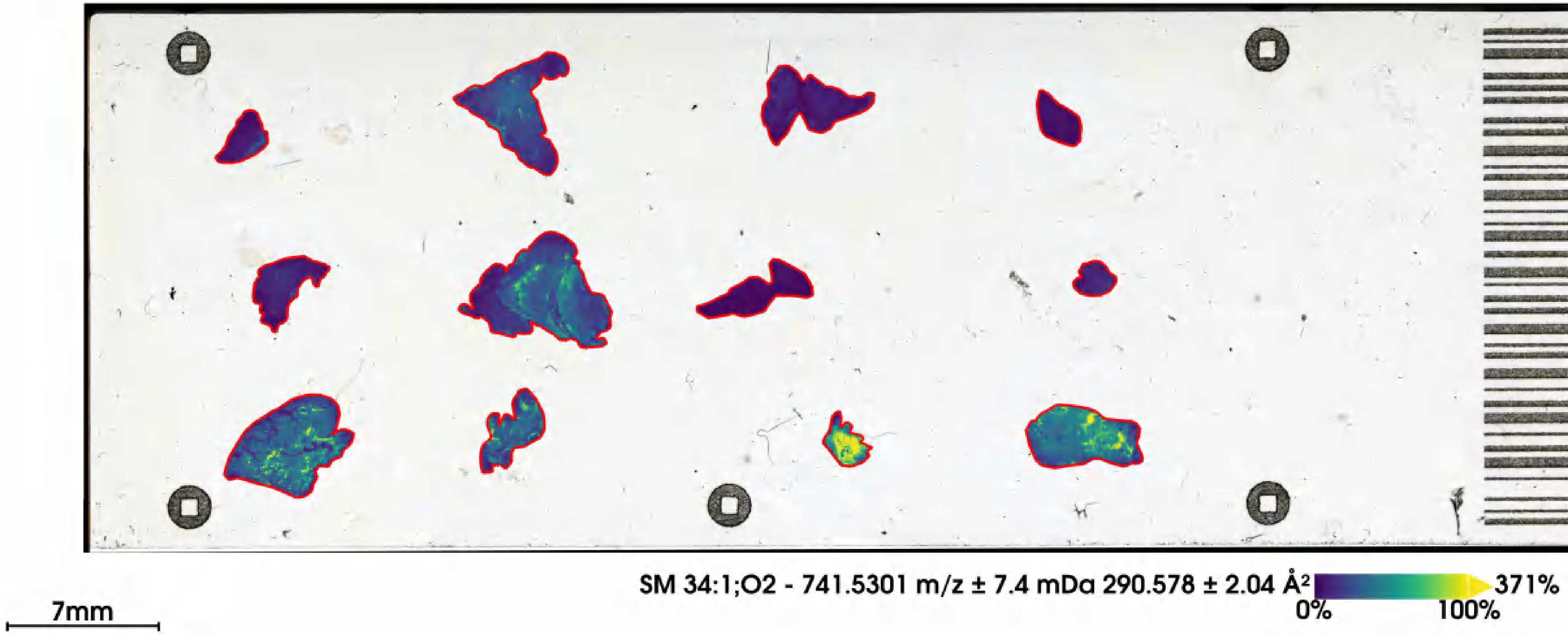
SM 34:1;O2 - 741.5301 m/z ± 7.4 mDa 290.578 ± 2.04 Å²
0%
100%
371%
7mm

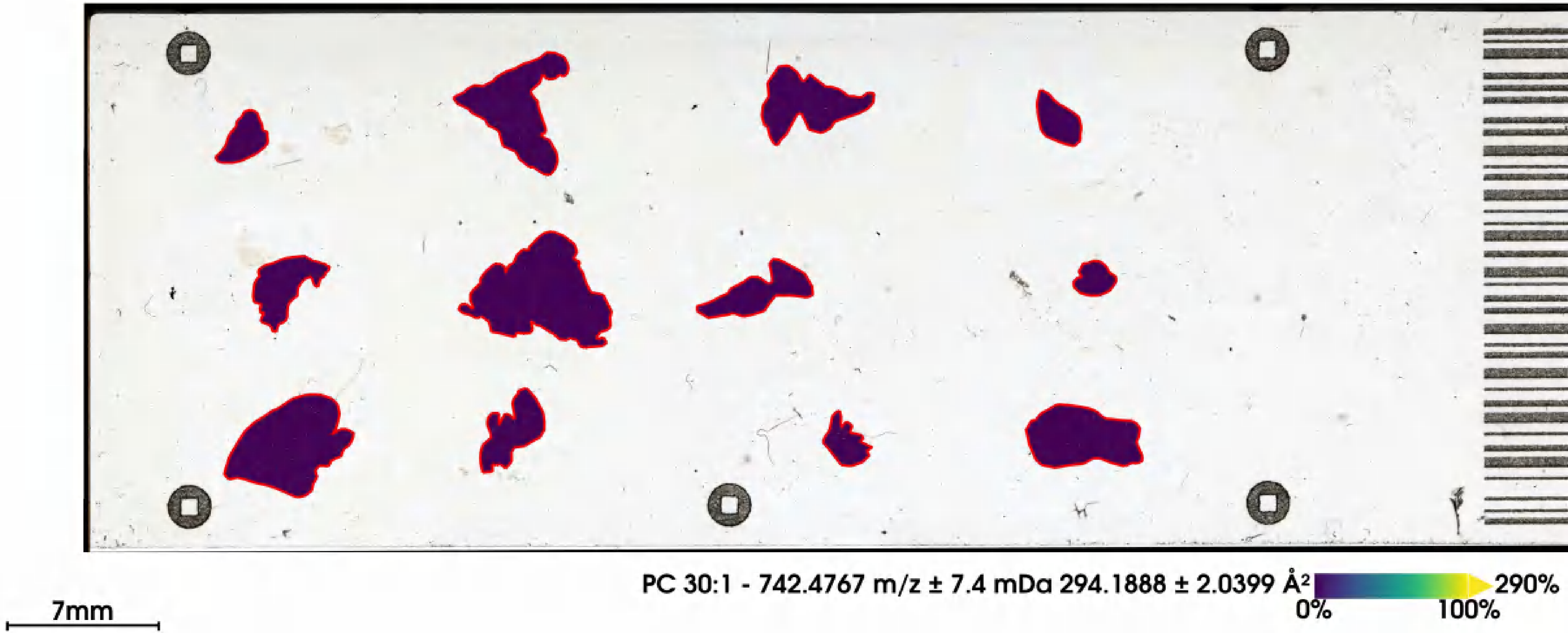
7mm
PC 30:1 - 742.4767 m/z ± 7.4 mDa 294.1888 ± 2.0399 Å²
0%
100%
290%

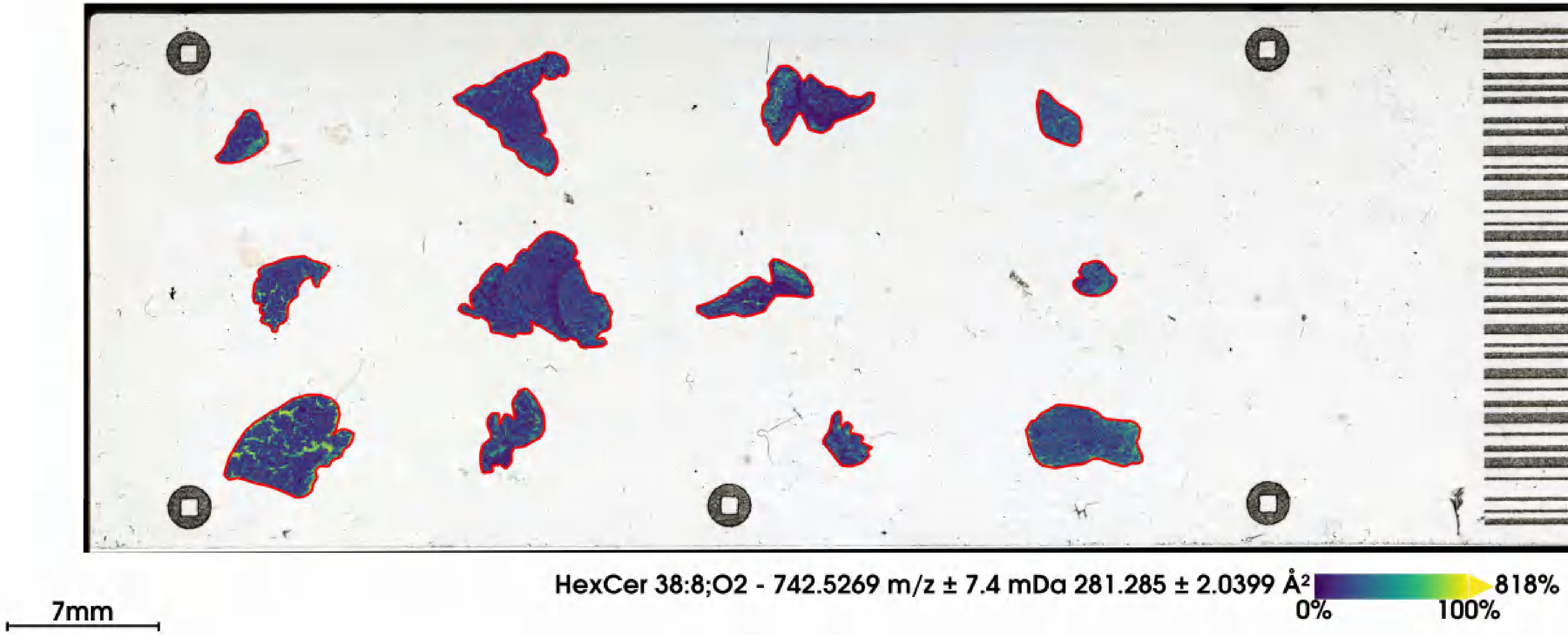
7mm
HexCer 38:8;O2 - 742.5269 m/z ± 7.4 mDa 281.285 ± 2.0399 Å²
0%
100%
818%

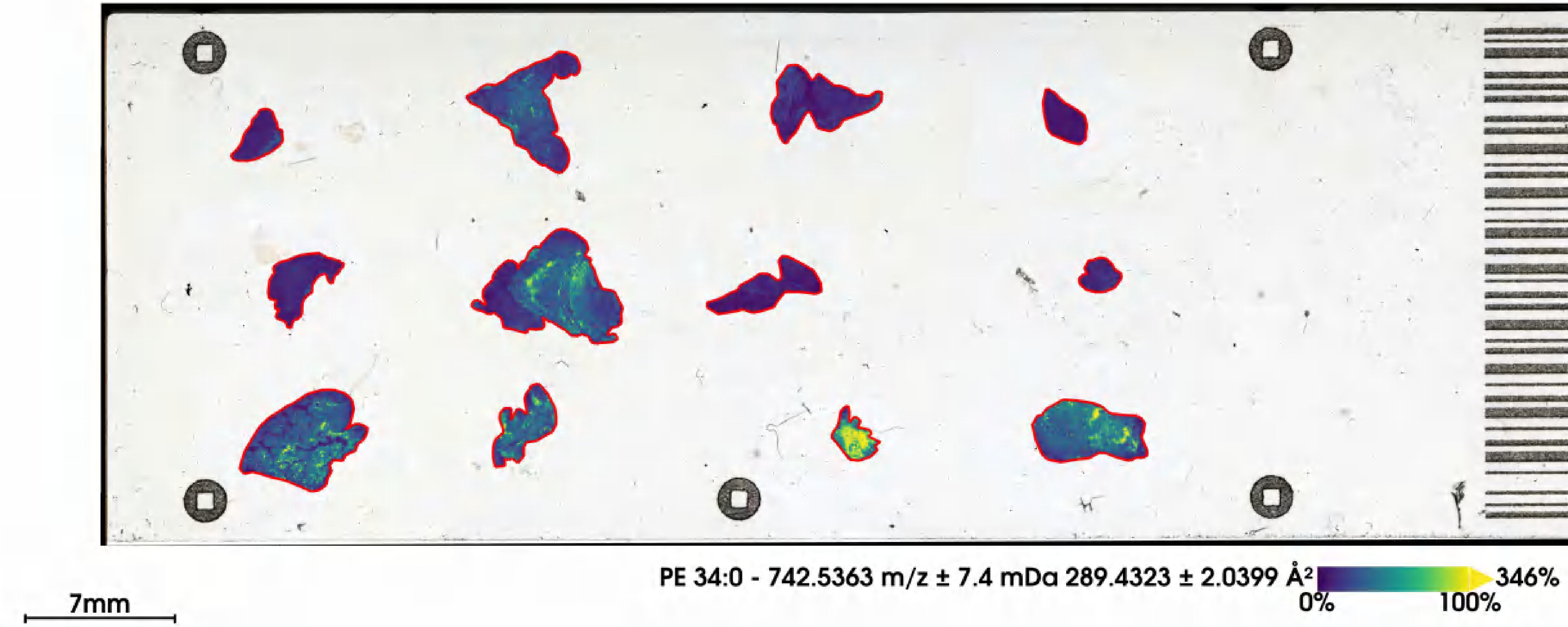
PE 34:0 - 742.5363 m/z ± 7.4 mDa 289.4323 ± 2.0399 Å²
346%
0%
100%
7mm

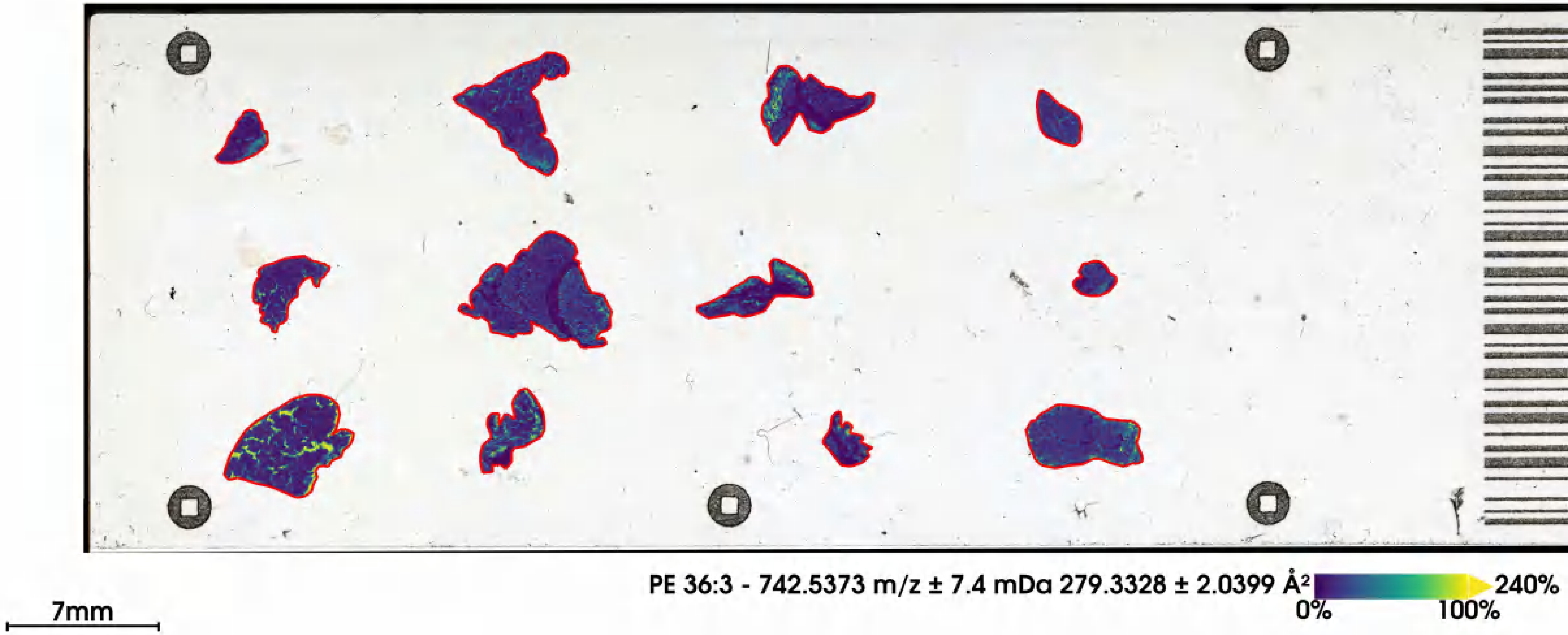

PE 36:3 - 742.5373 m/z ± 7.4 mDa 279.3328 ± 2.0399 Å²
0%
100%
240%
7mm

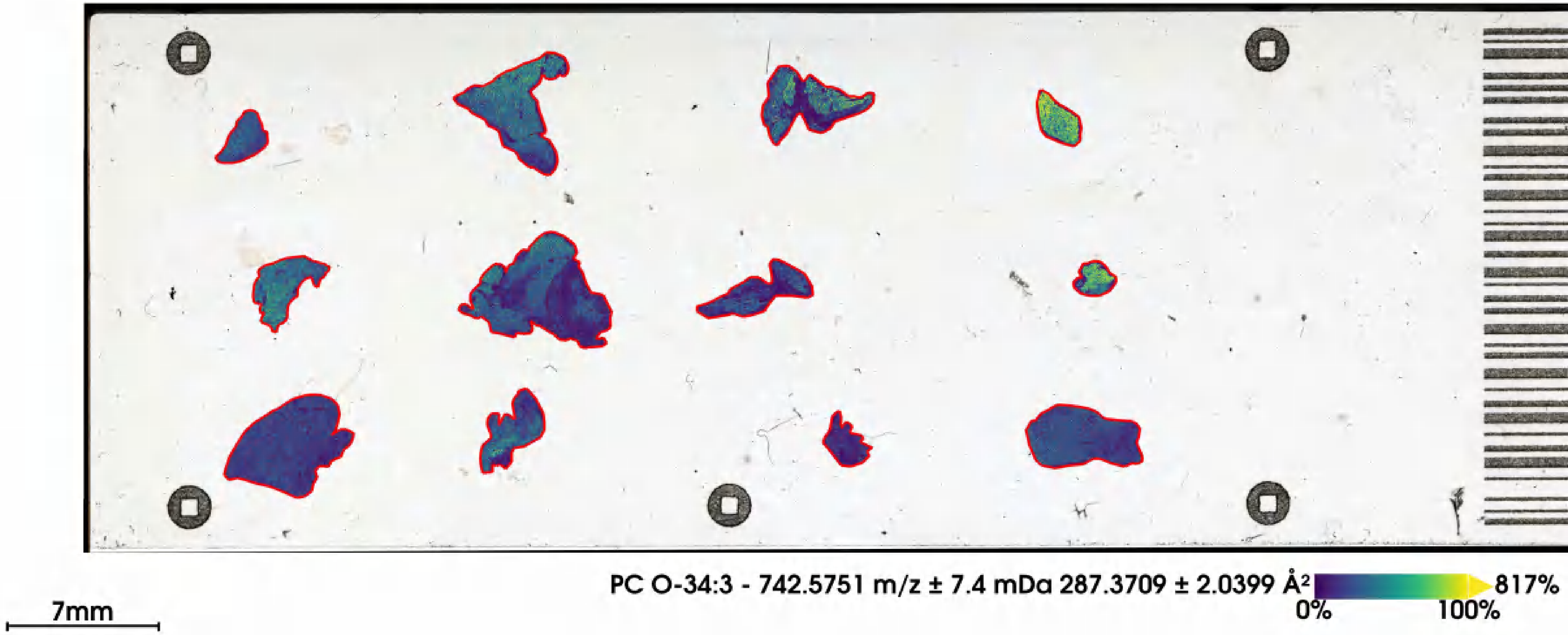
PC O-34:3 - 742.5751 m/z ± 7.4 mDa 287.3709 ± 2.0399 Å²
817%
0%
100%
7mm

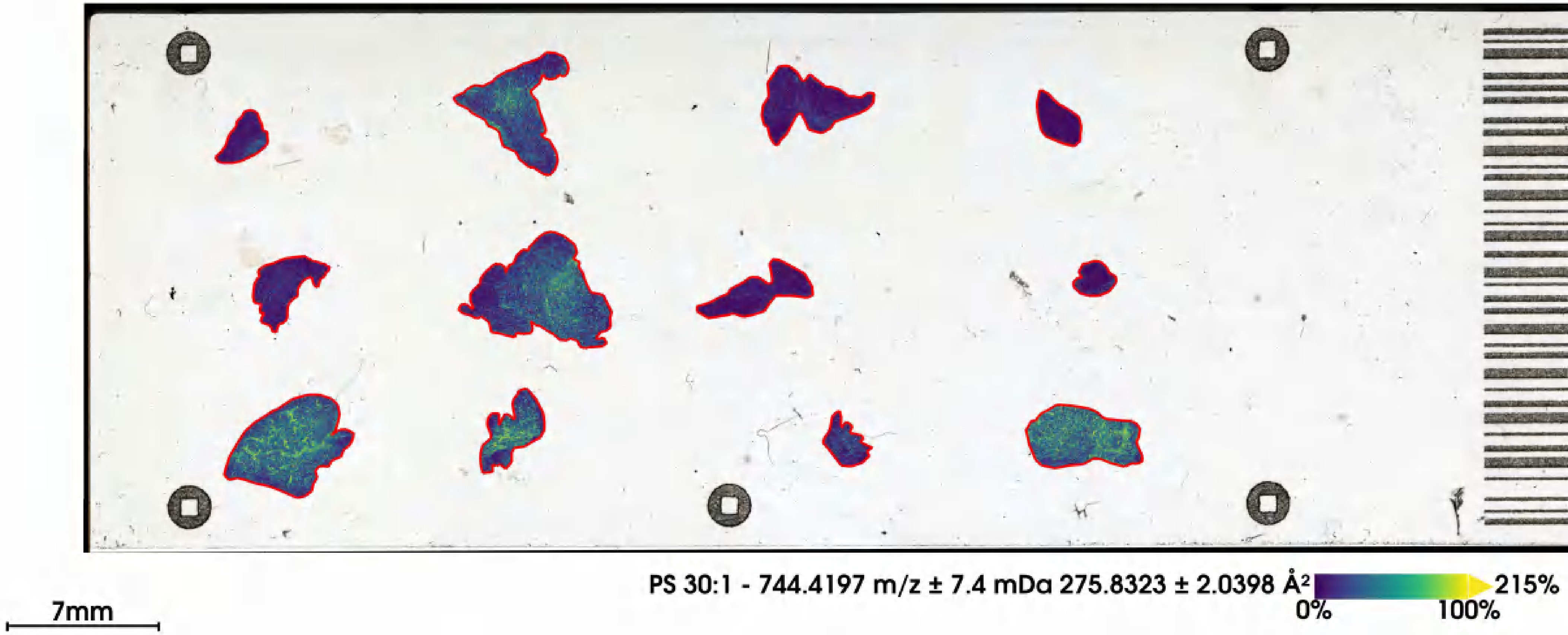
PS 30:1 - 744.4197 m/z ± 7.4 mDa 275.8323 ± 2.0398 Å²
0%
100%
215%
7mm

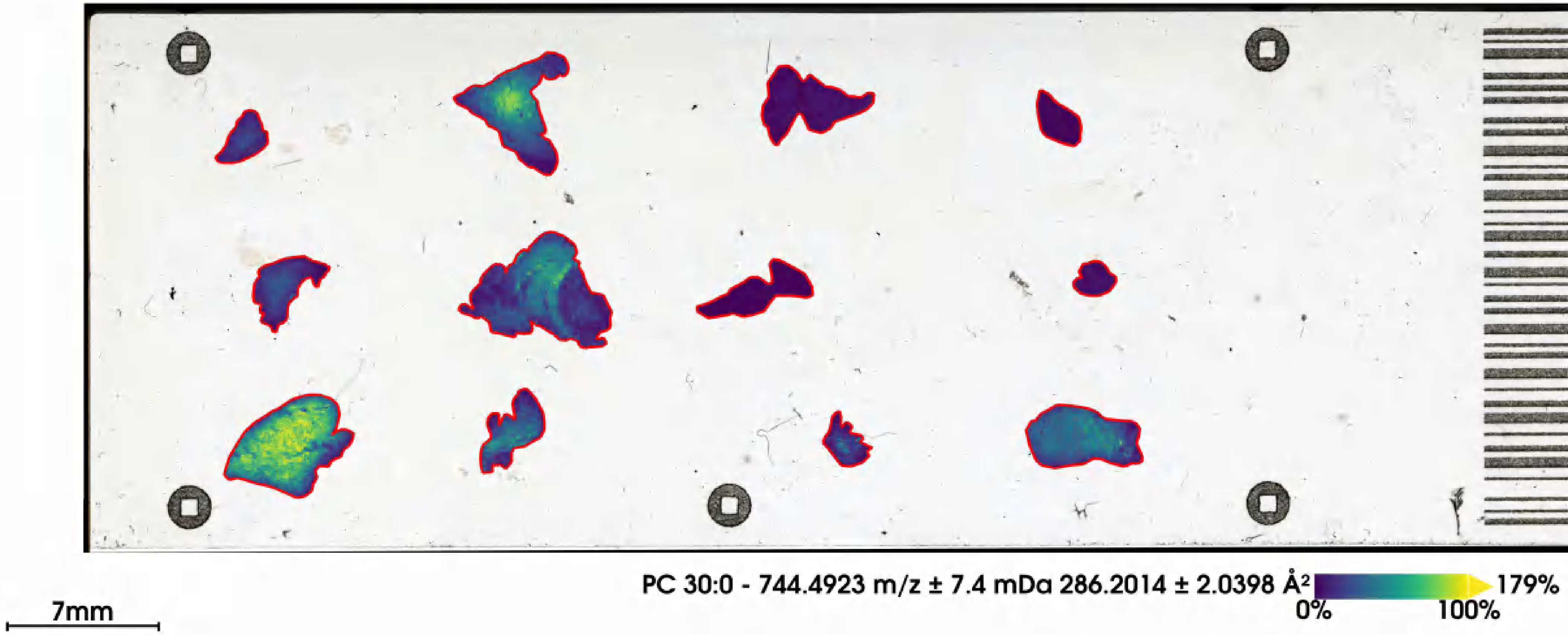
PC 30:0 - 744.4923 m/z ± 7.4 mDa 286.2014 ± 2.0398 Å²
0%
100%
179%
7mm

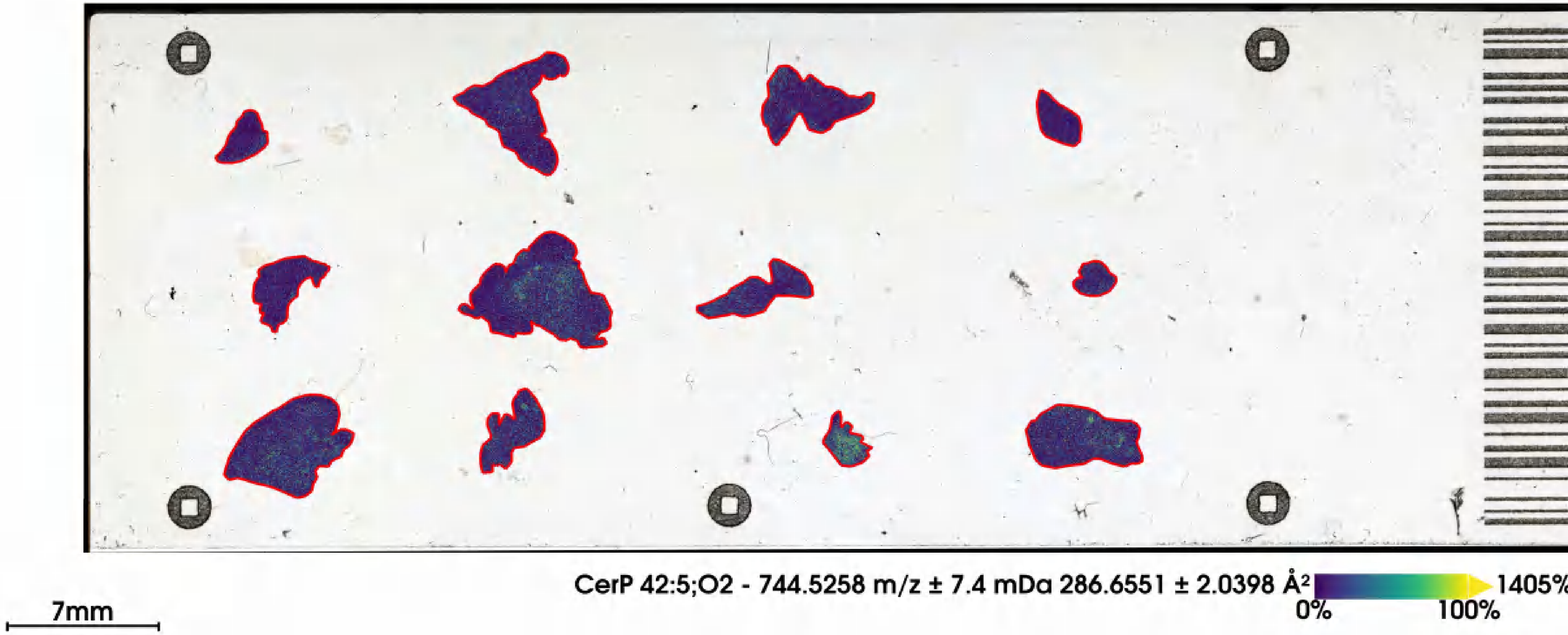

CerP 42:5;O2 - 744.5258 m/z ± 7.4 mDa 286.6551 ± 2.0398 Å²
0%
100%
1405%
7mm

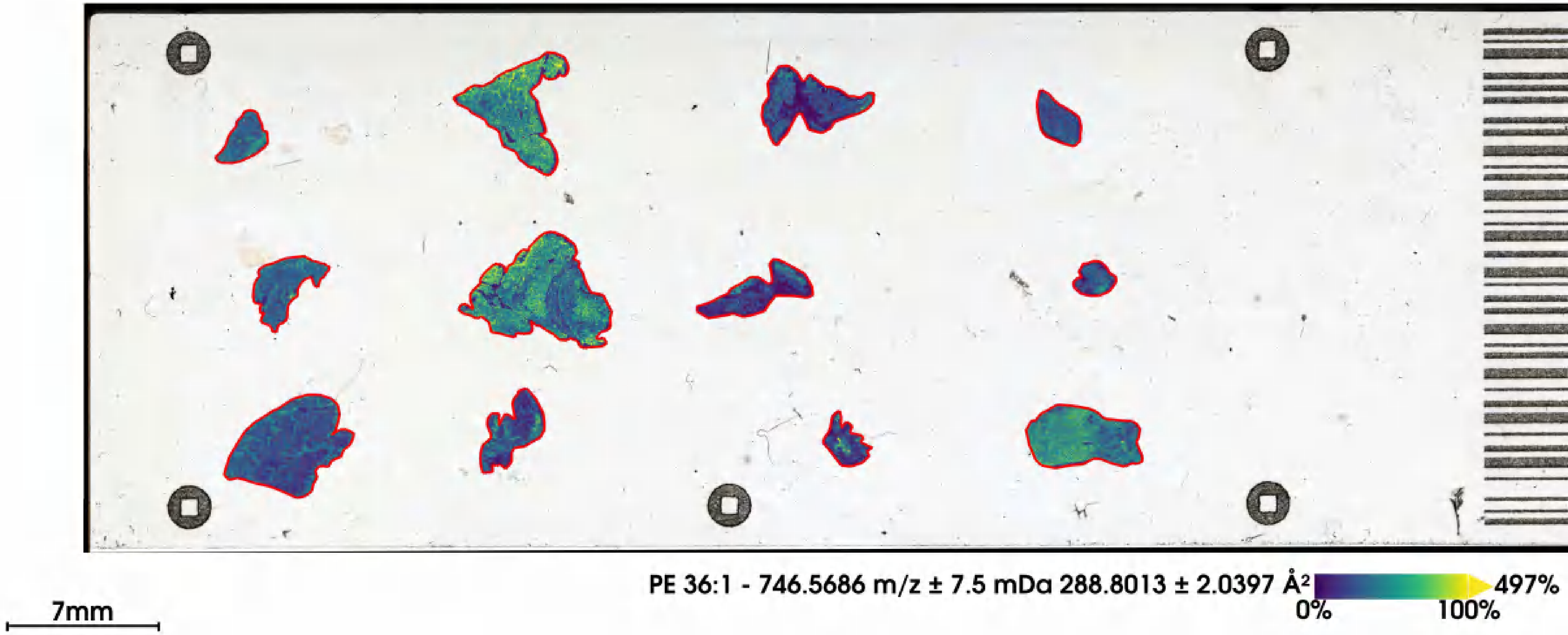
7mm
PE 36:1 - 746.5686 m/z ± 7.5 mDa 288.8013 ± 2.0397 Å²
0%
100%
497%

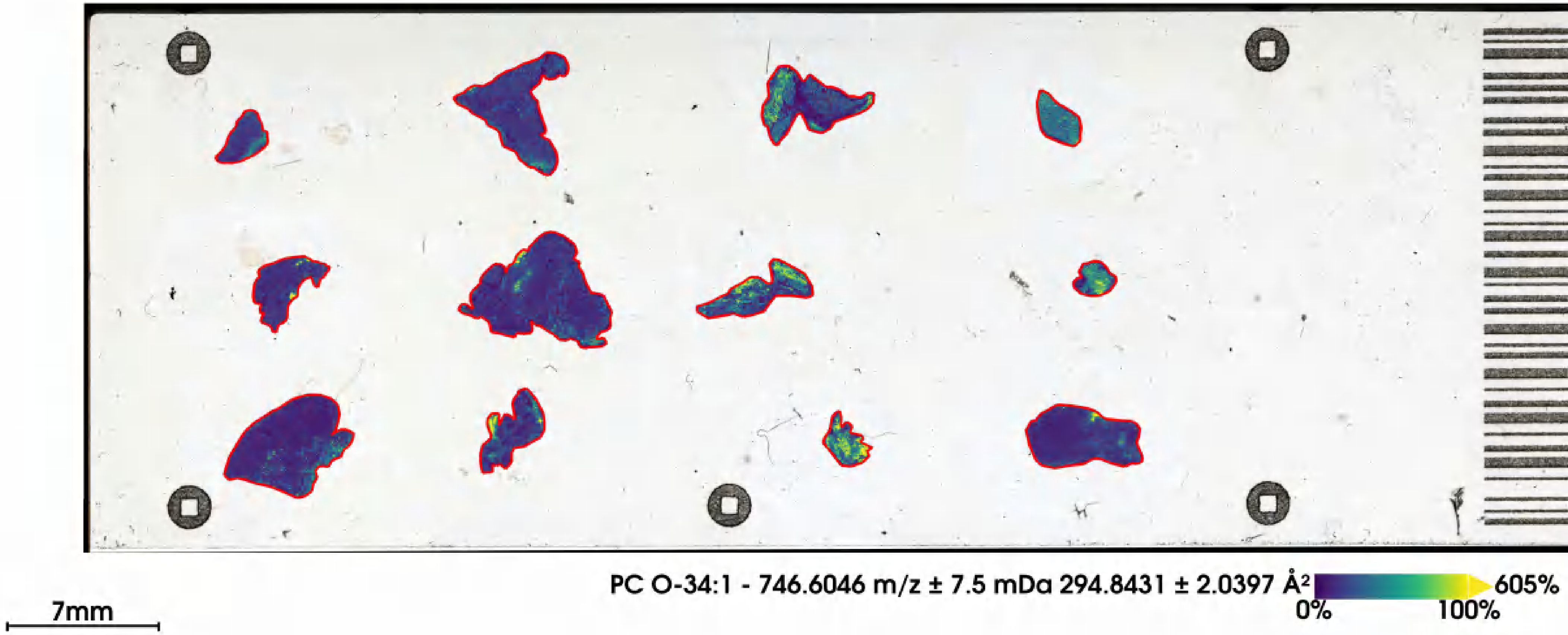
PC O-34:1 - 746.6046 m/z ± 7.5 mDa 294.8431 ± 2.0397 Å²
0%
100%
605%
7mm

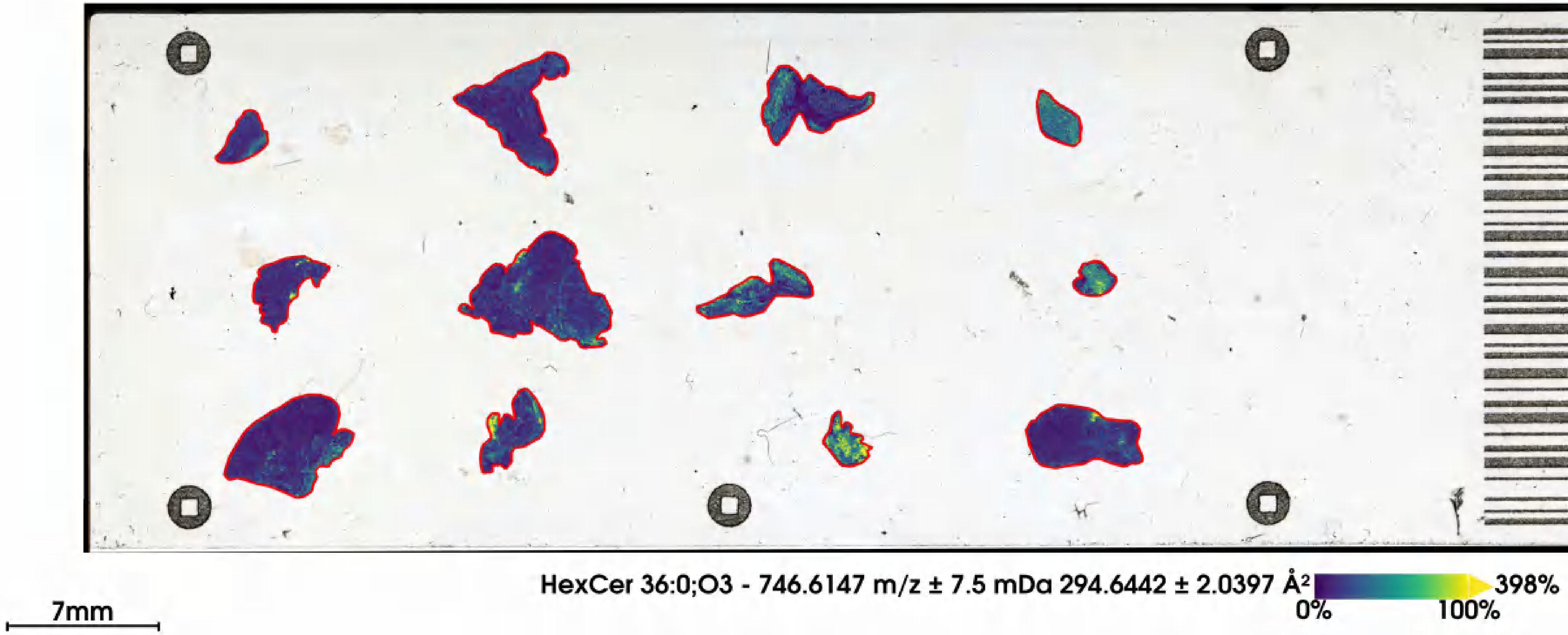

HexCer 36:0;O3 - 746.6147 m/z ± 7.5 mDa 294.6442 ± 2.0397 Å²
0%
100%
398%
7mm

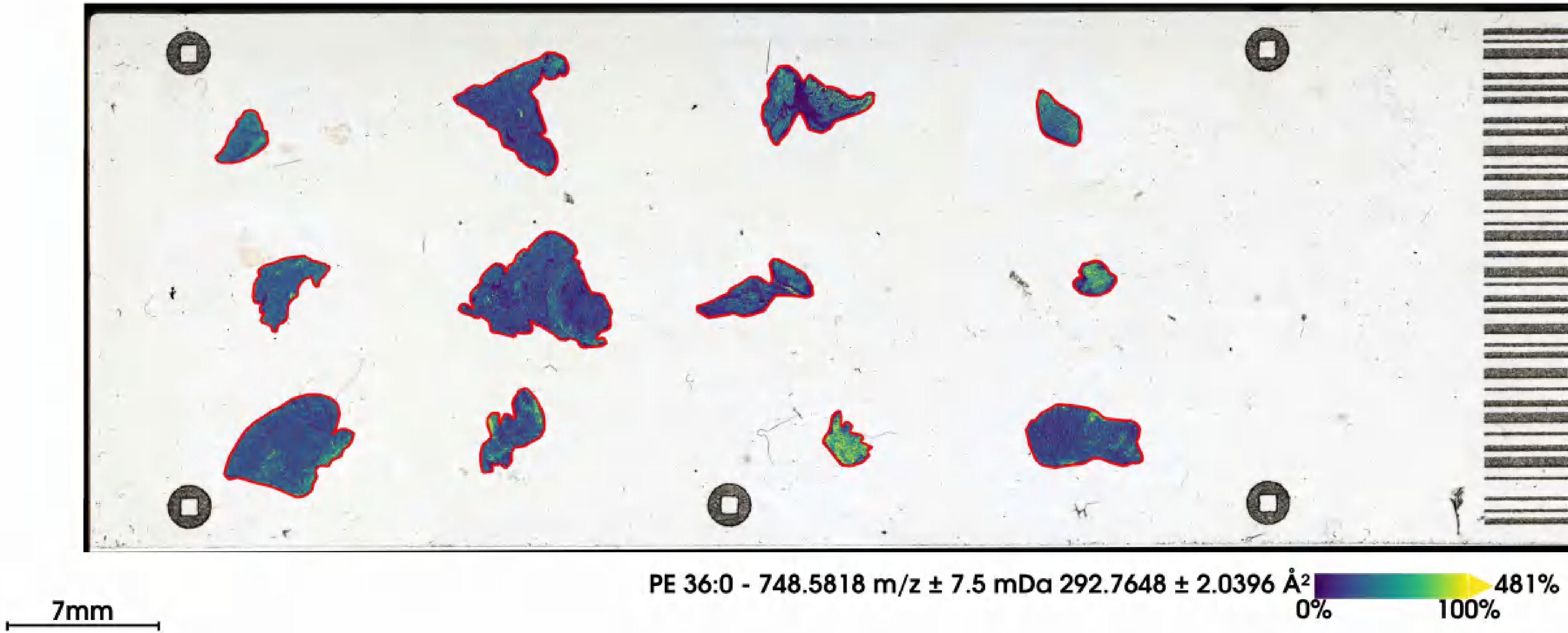
PE 36:0 - 748.5818 m/z ± 7.5 mDa 292.7648 ± 2.0396 Å²
0%
100%
481%
7mm

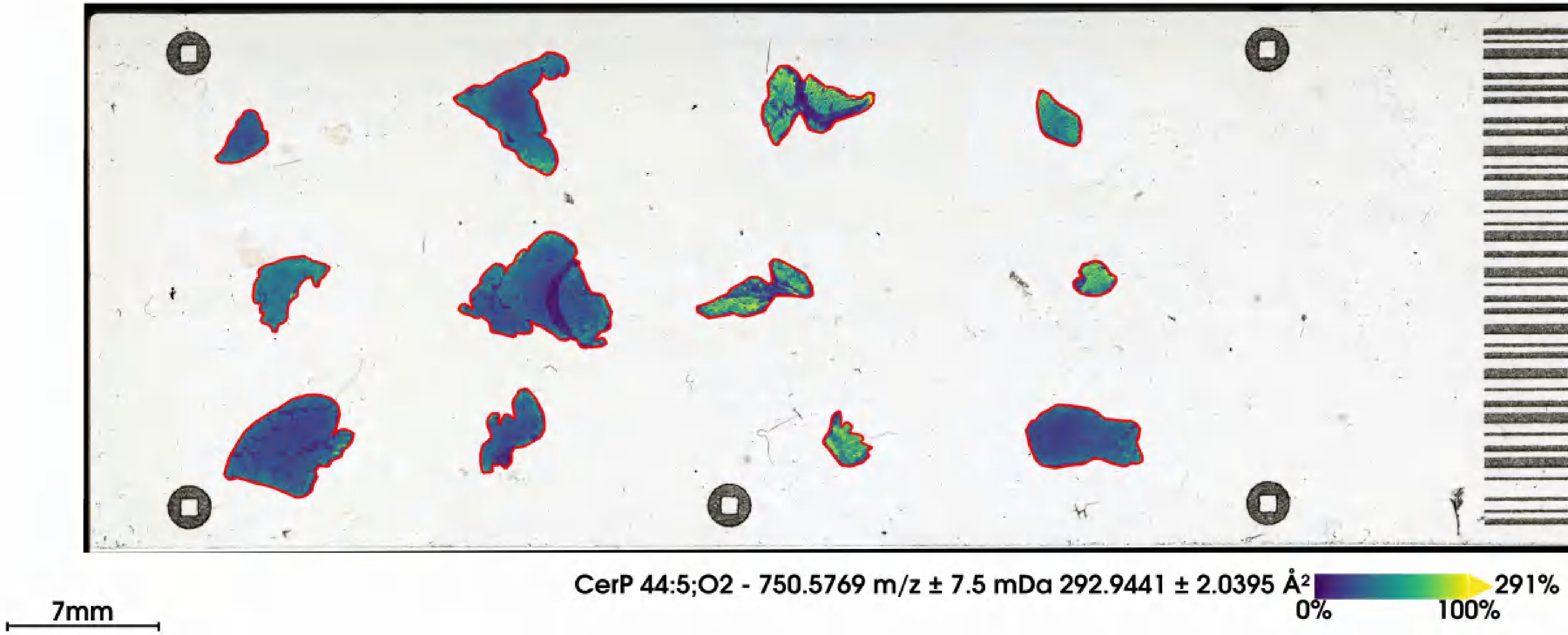
CerP 44:5;O2 - 750.5769 m/z ± 7.5 mDa 292.9441 ± 2.0395 Å²
291%
0%
100%
7mm

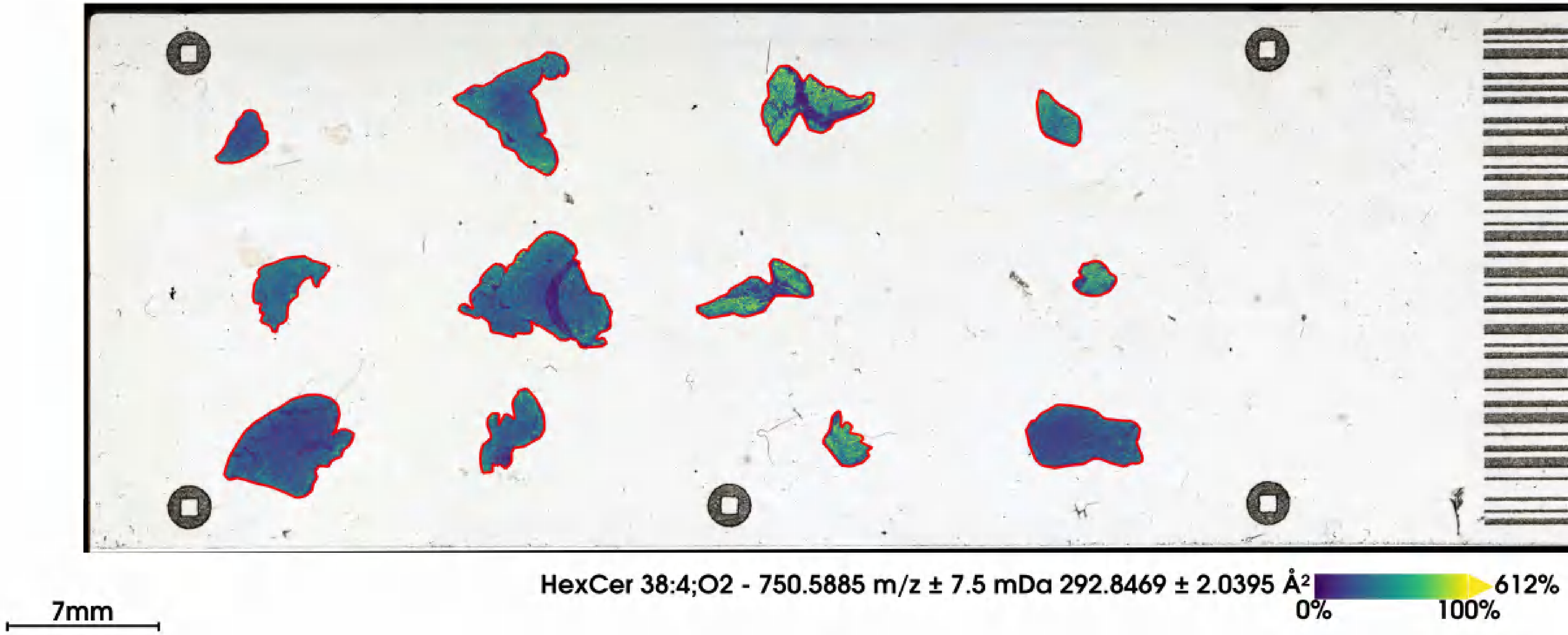

HexCer 38:4;O2 - 750.5885 m/z ± 7.5 mDa 292.8469 ± 2.0395 Å²
612%
0%
100%
7mm

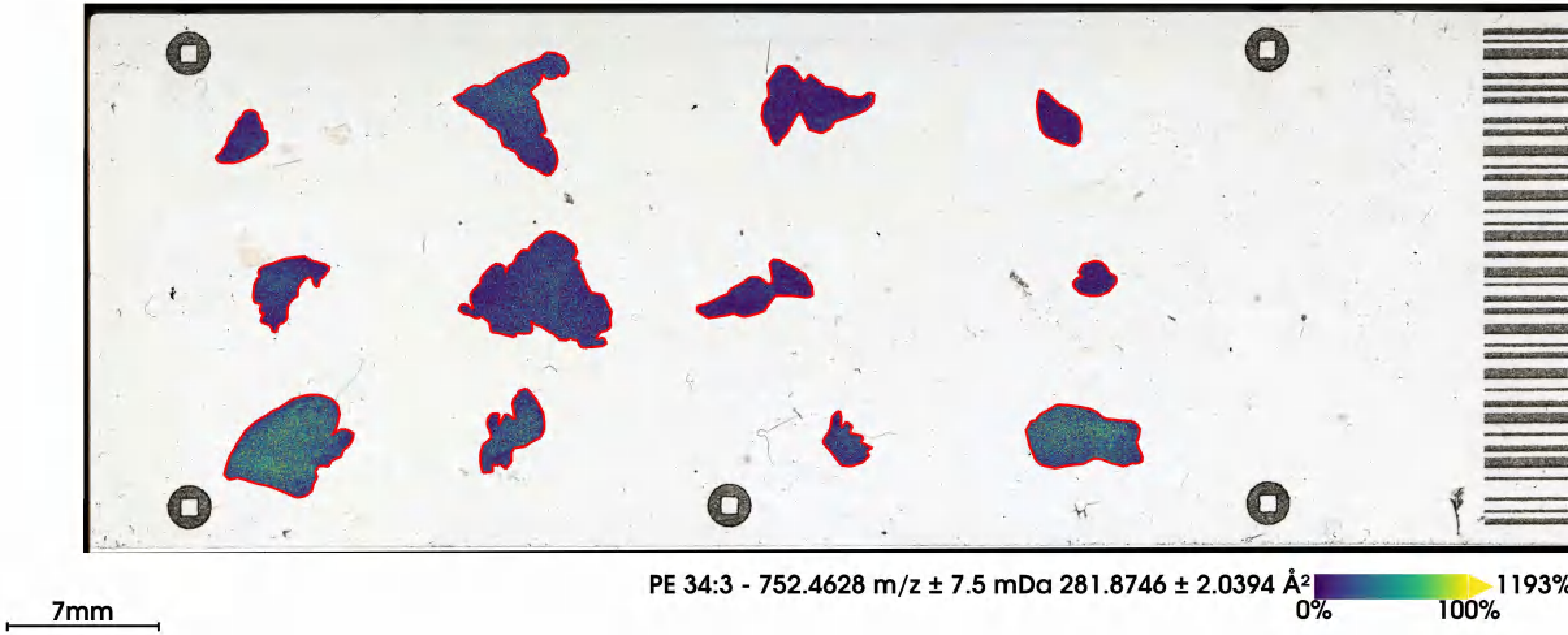

PE 34:3 - 752.4628 m/z ± 7.5 mDa 281.8746 ± 2.0394 Å²
1193%
0%
100%
7mm

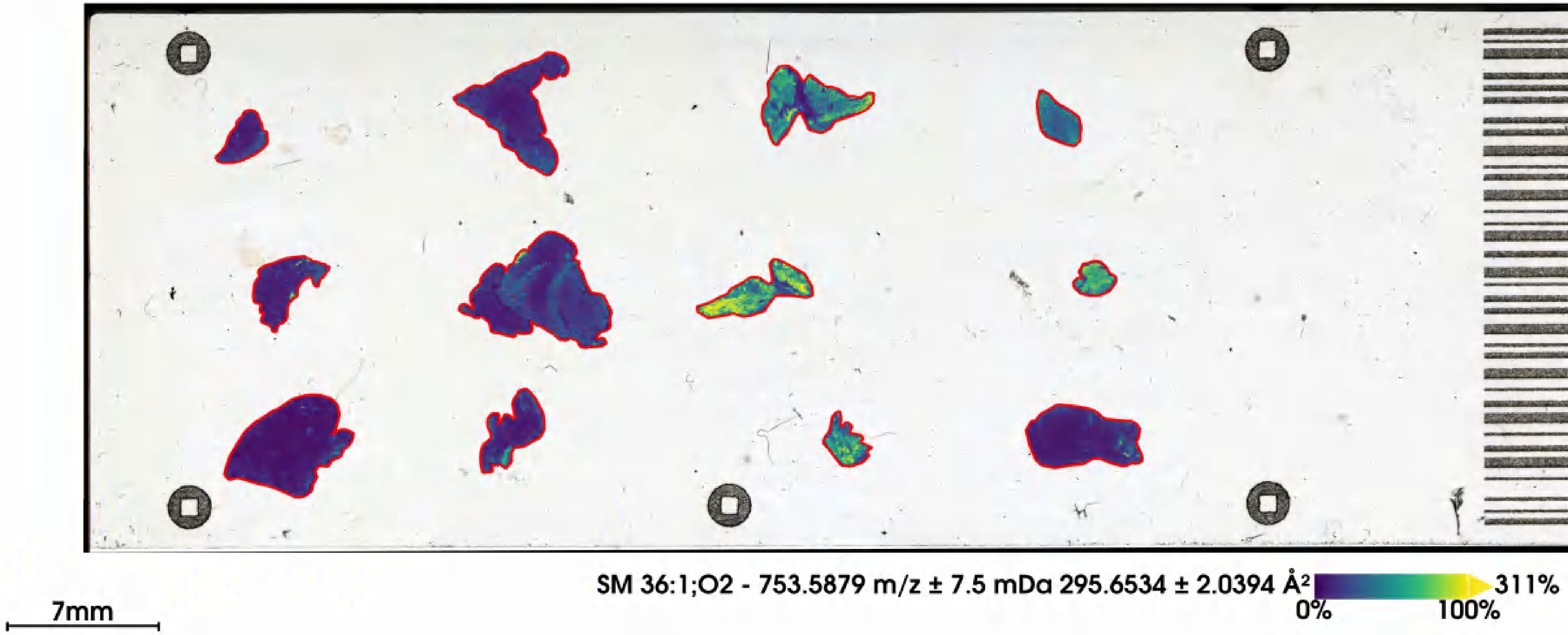
SM 36:1;O2 - 753.5879 m/z ± 7.5 mDa 295.6534 ± 2.0394 Å²
311%
0%
100%
7mm

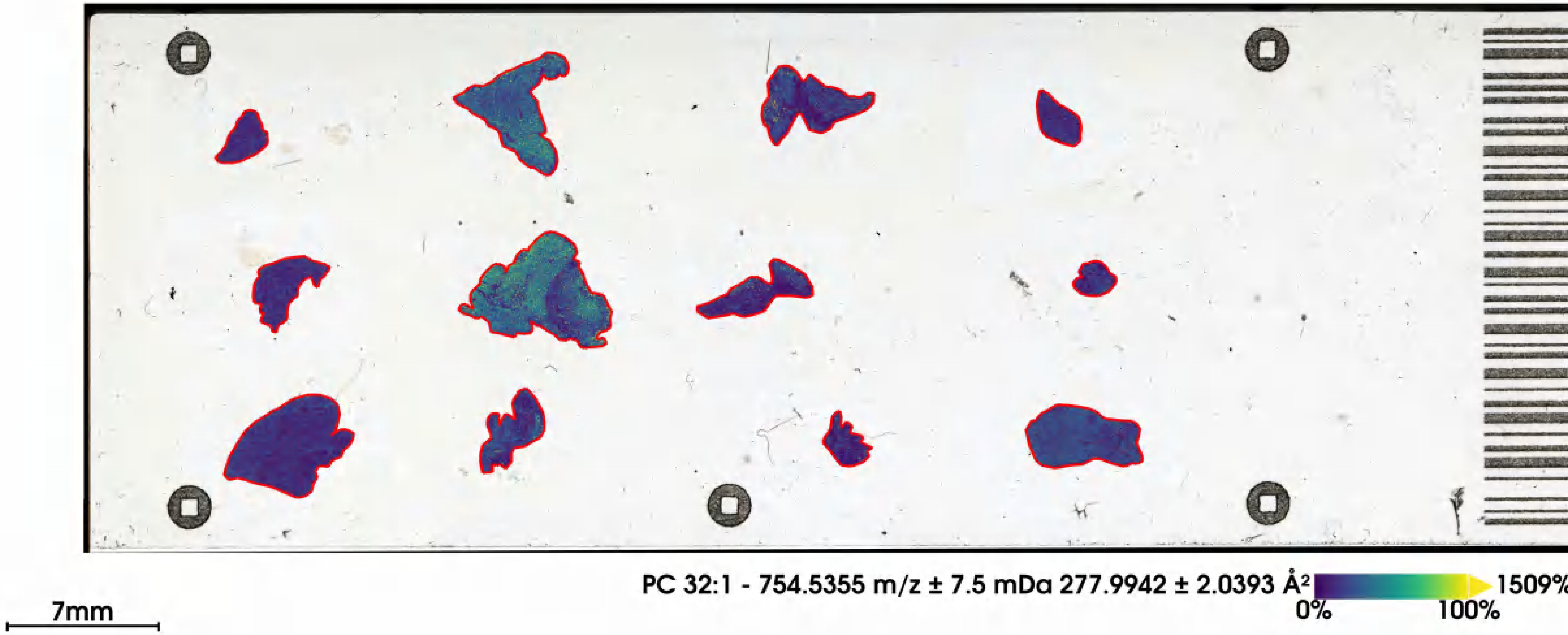

PC 32:1 - 754.5355 m/z ± 7.5 mDa 277.9942 ± 2.0393 Å²
0%
100%
1509%
7mm

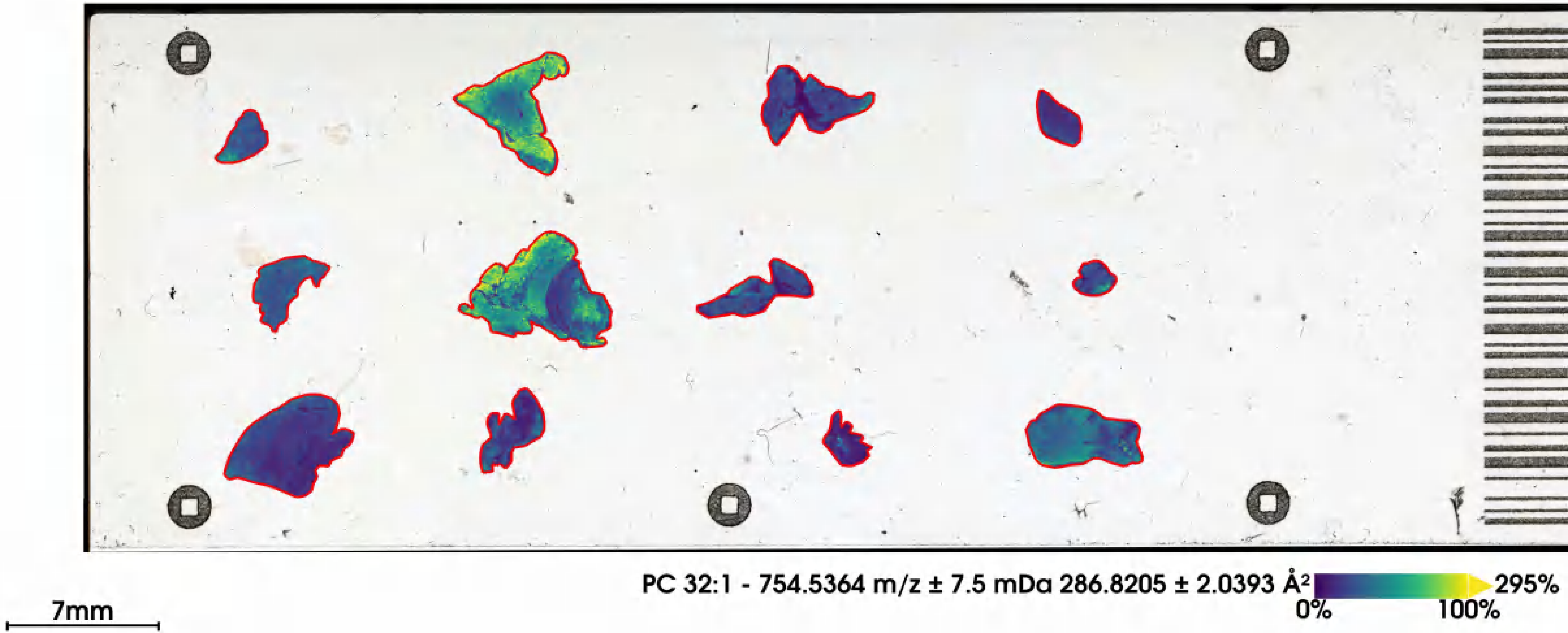
PC 32:1 - 754.5364 m/z ± 7.5 mDa 286.8205 ± 2.0393 Å²
0%
100%
295%
7mm

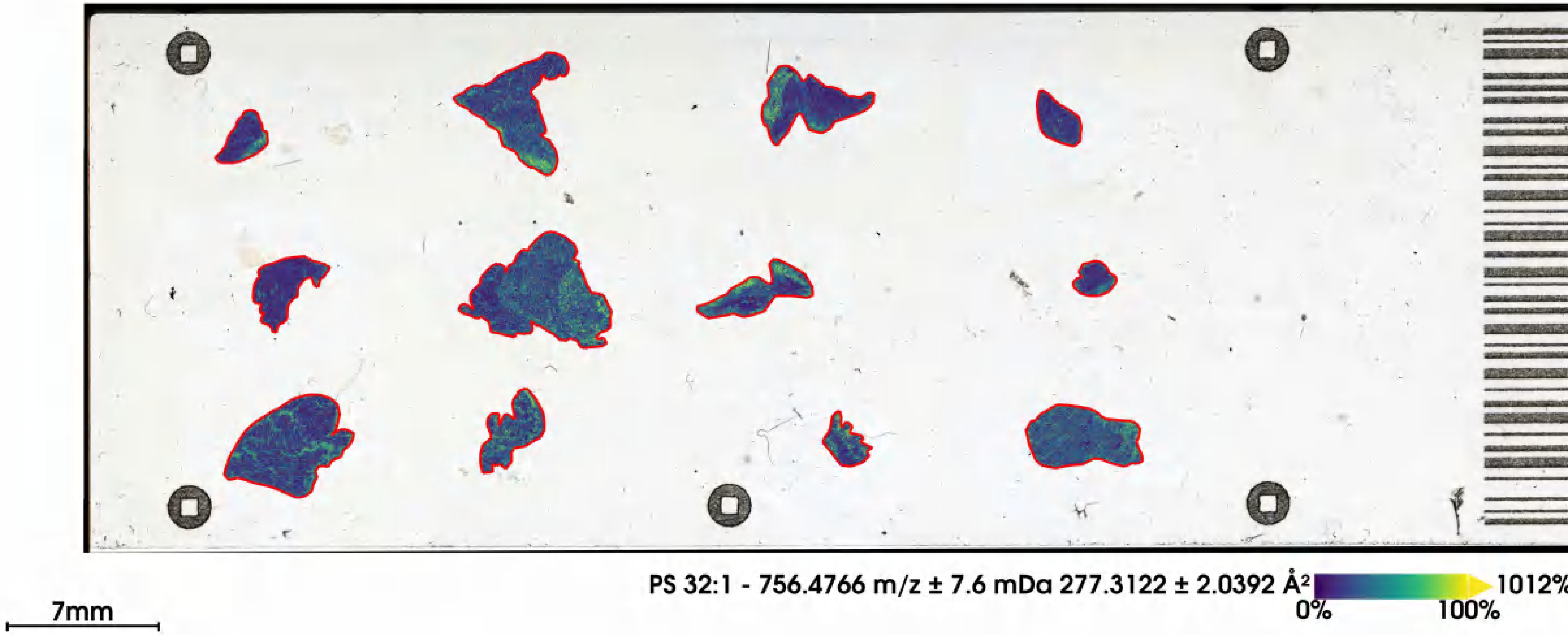
PS 32:1 - 756.4766 m/z ± 7.6 mDa 277.3122 ± 2.0392 Å²
0%
100%
1012%
7mm

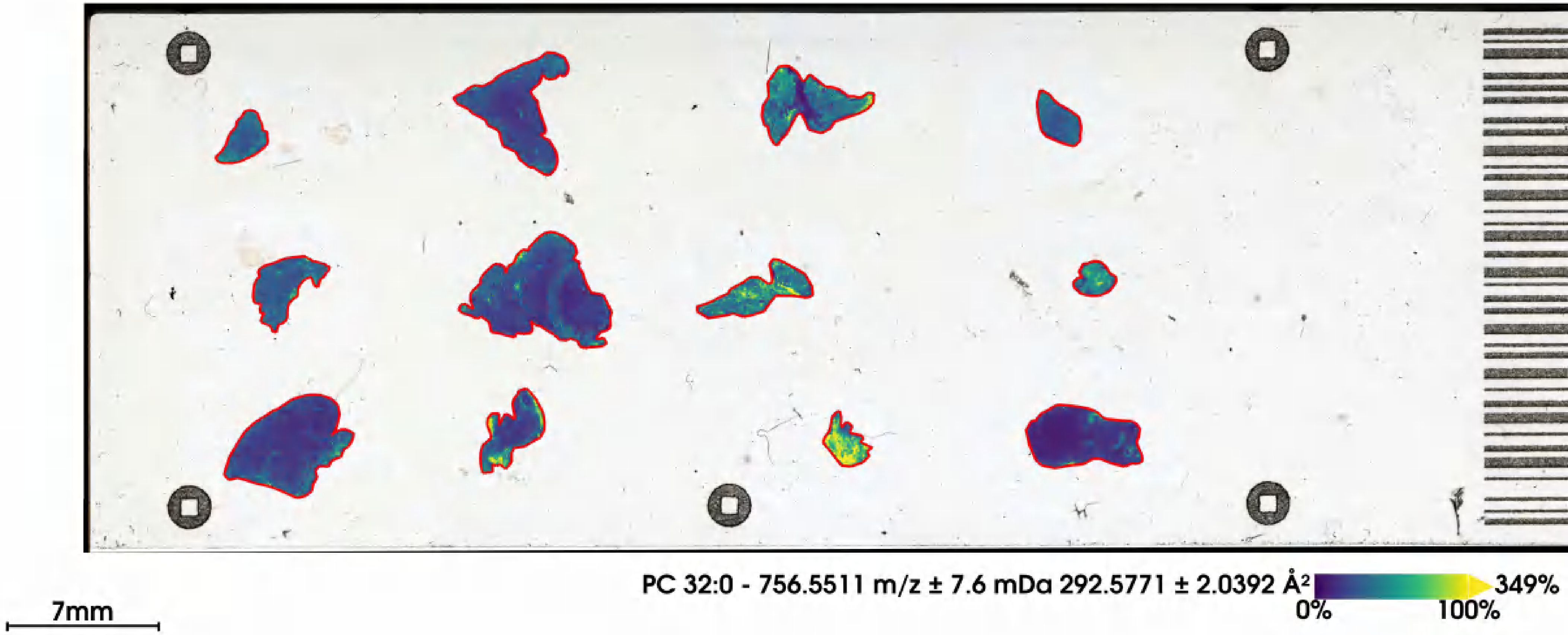
PC 32:0 - 756.5511 m/z ± 7.6 mDa 292.5771 ± 2.0392 Å²
0%
100%
349%
7mm

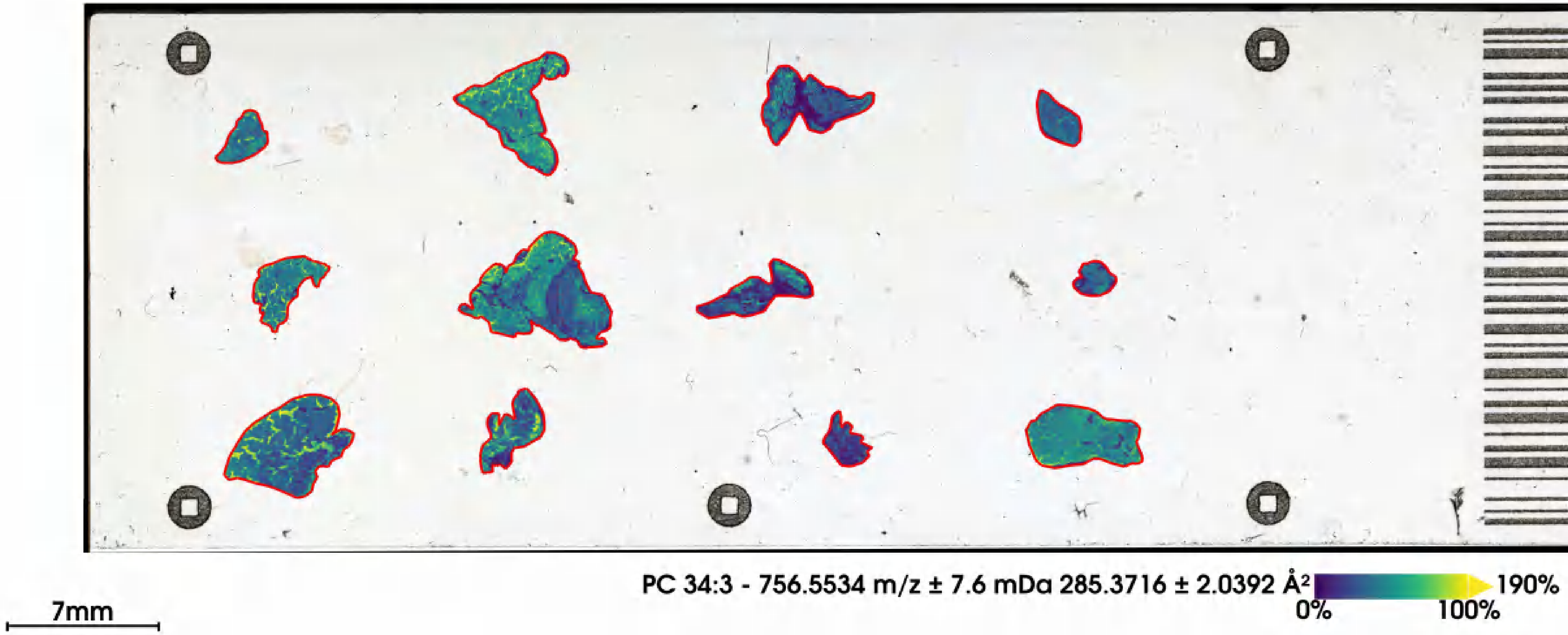
PC 34:3 - 756.5534 m/z ± 7.6 mDa 285.3716 ± 2.0392 Å²
0%
100%
190%
7mm

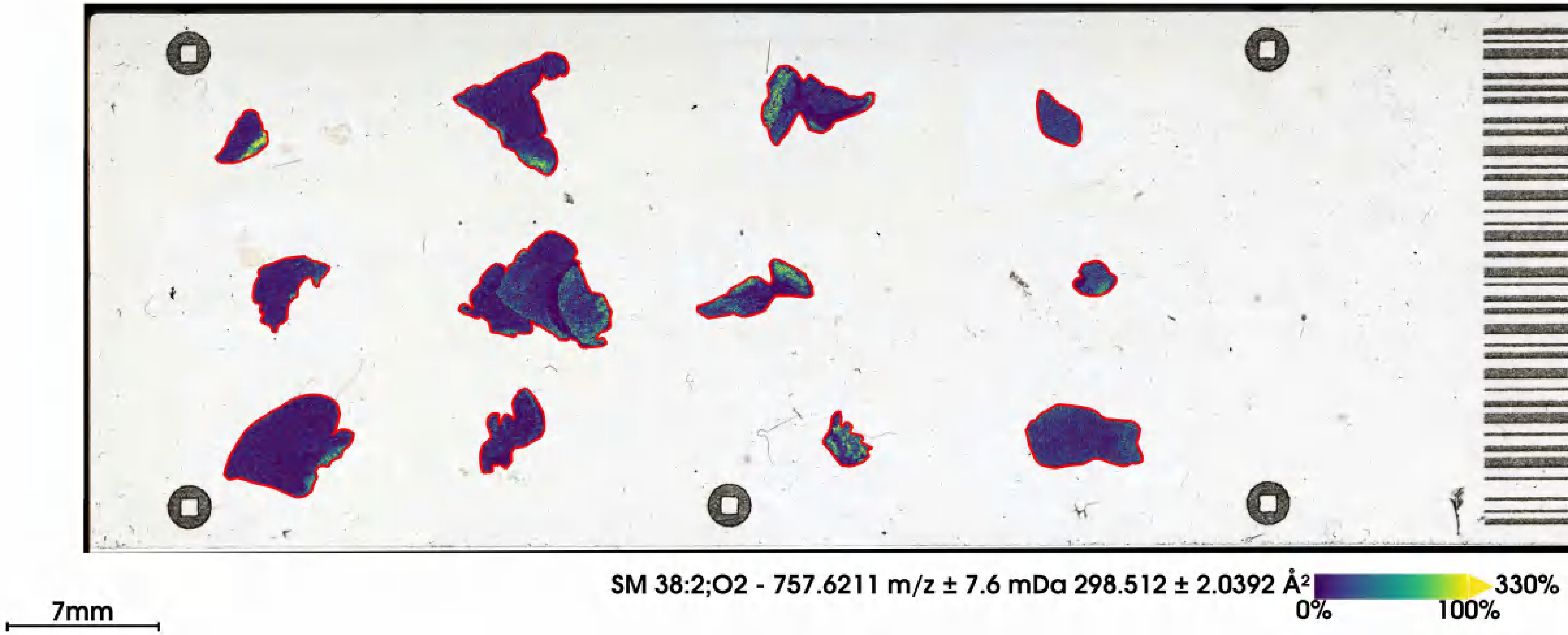
SM 38:2;O2 - 757.6211 m/z ± 7.6 mDa 298.512 ± 2.0392 Å²
0%
100%
330%
7mm

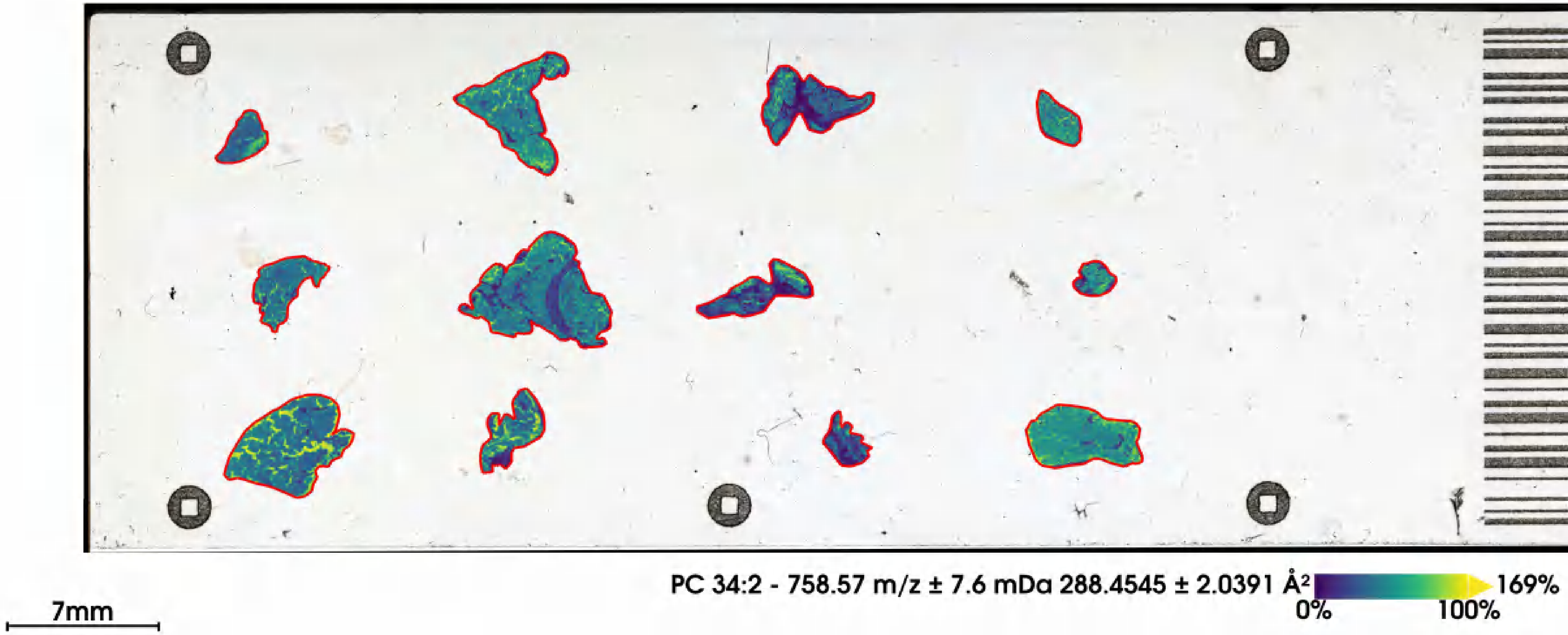
PC 34:2 - 758.57 m/z ± 7.6 mDa 288.4545 ± 2.0391 Å²
0%
100%
169%
7mm

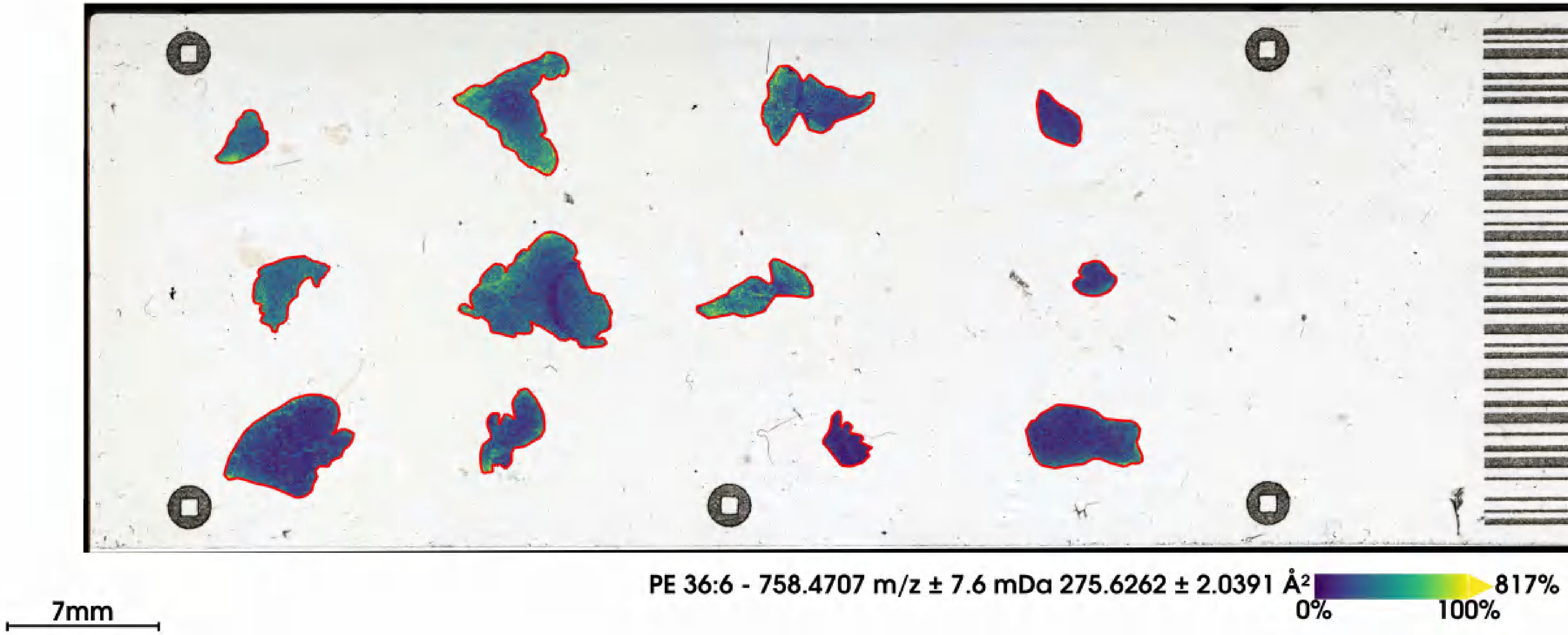

PE 36:6 - 758.4707 m/z ± 7.6 mDa 275.6262 ± 2.0391 Å²
0%
100%
817%
7mm

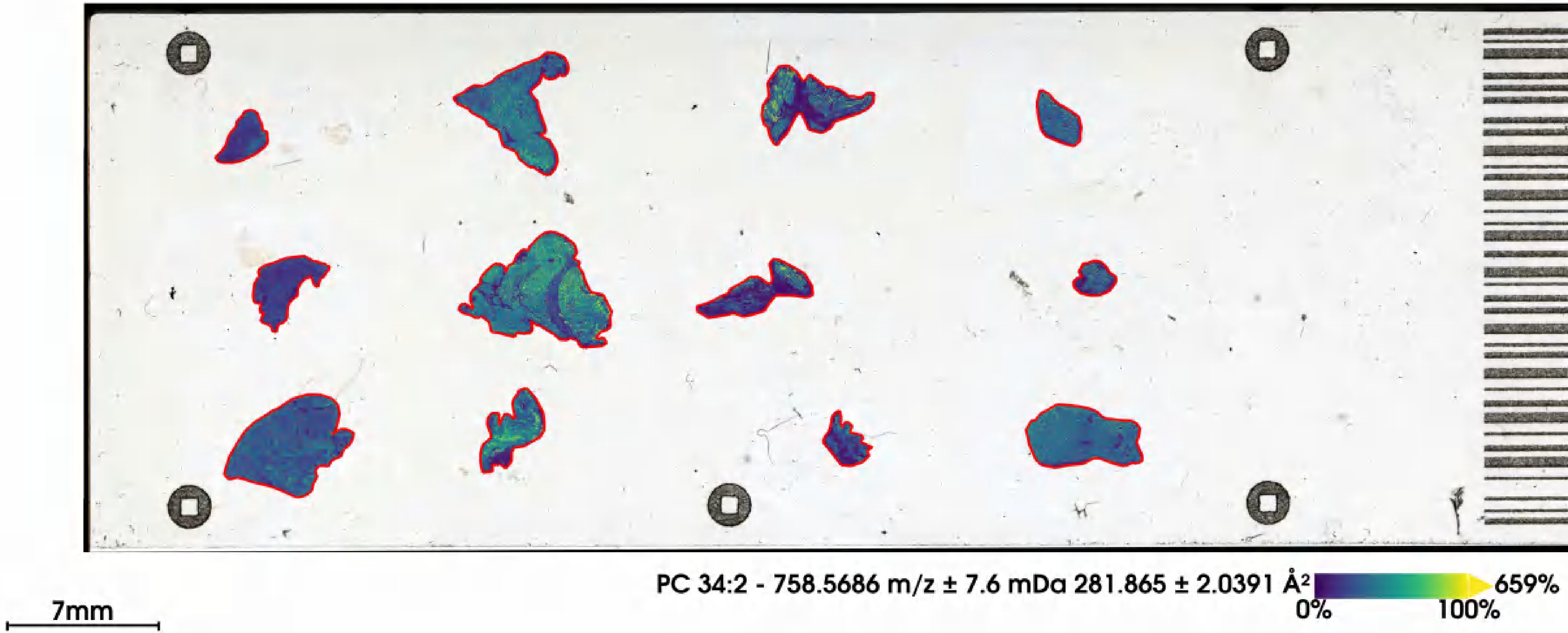
PC 34:2 - 758.5686 m/z ± 7.6 mDa 281.865 ± 2.0391 Å²
0%
100%
659%
7mm

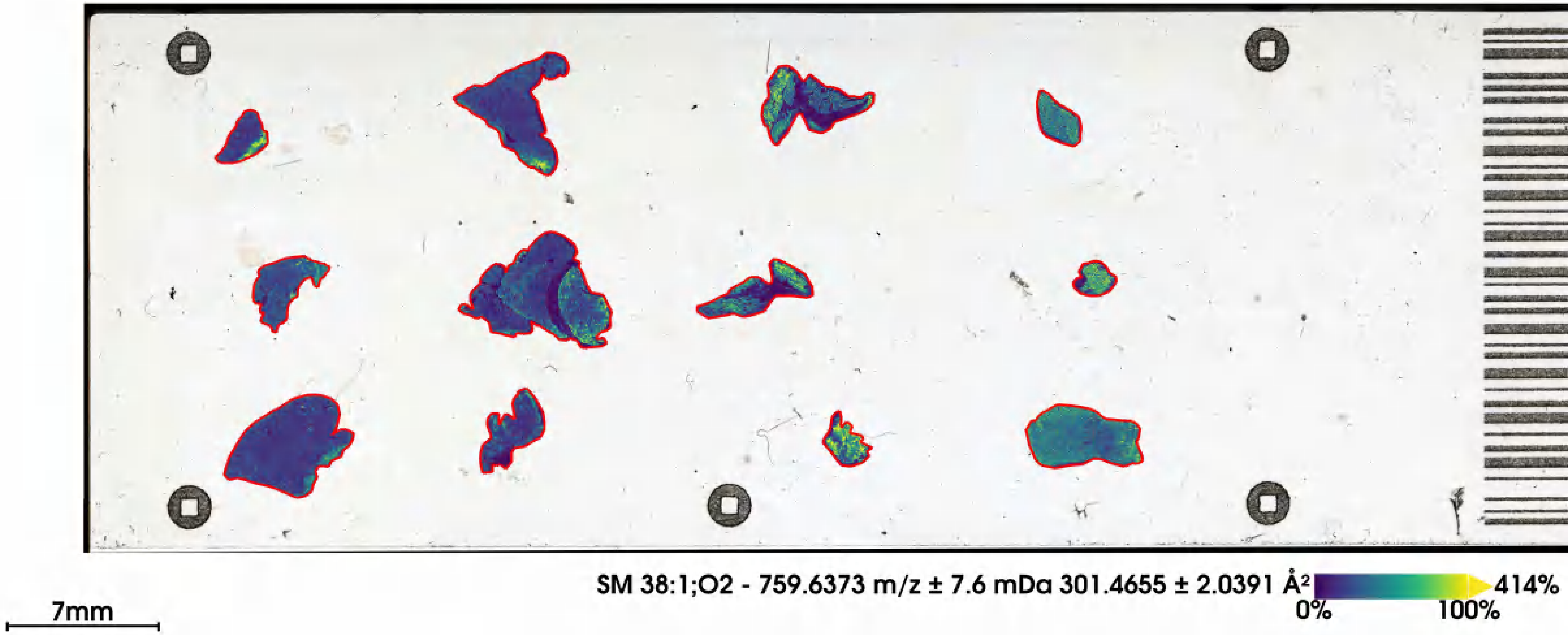
SM 38:1;O2 - 759.6373 m/z ± 7.6 mDa 301.4655 ± 2.0391 Å²
414%
0%
100%
7mm

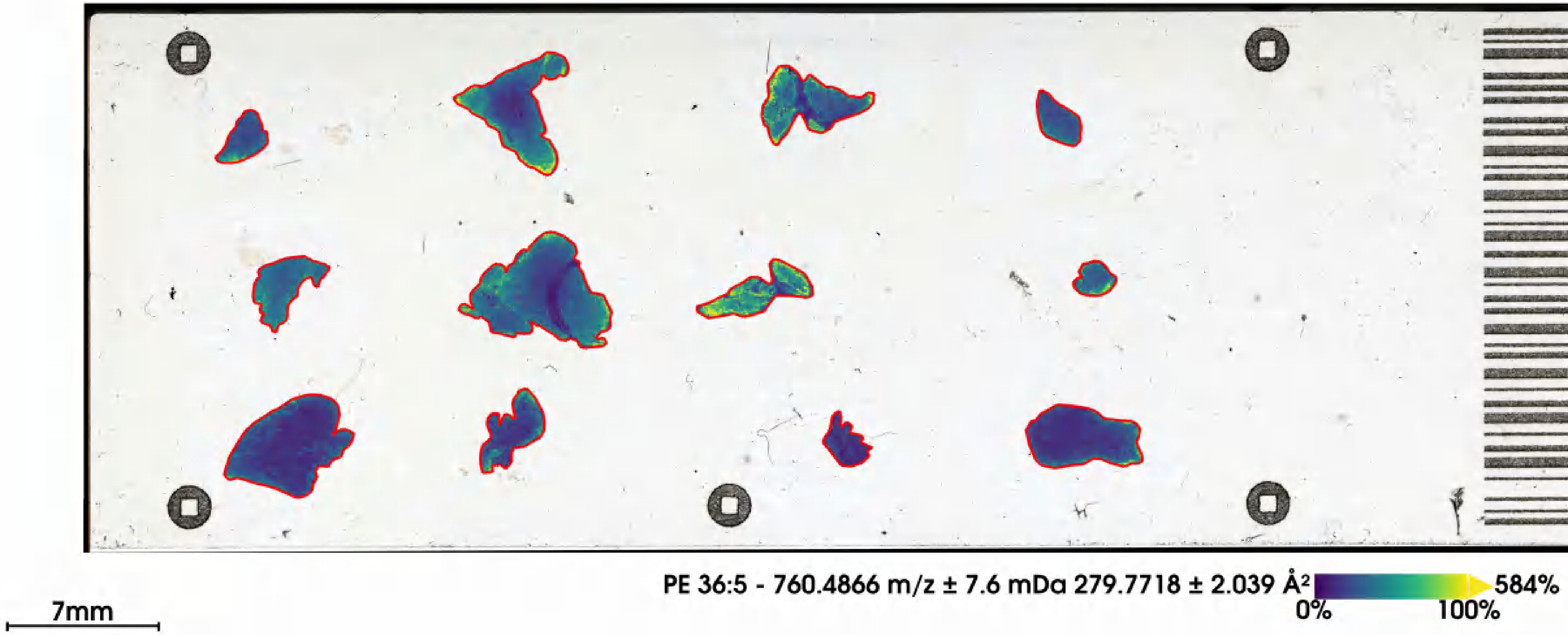

PE 36:5 - 760.4866 m/z ± 7.6 mDa 279.7718 ± 2.039 Å²
0%
100%
584%
7mm

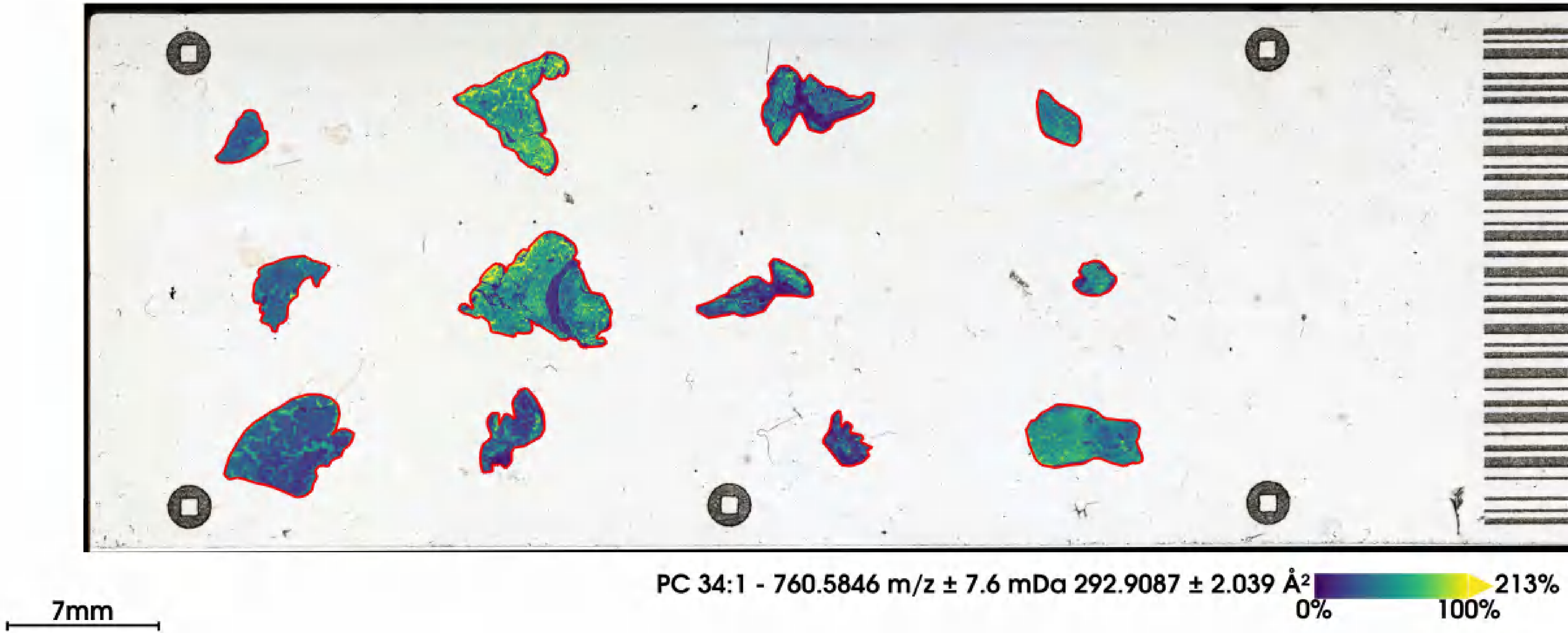
PC 34:1 - 760.5846 m/z ± 7.6 mDa 292.9087 ± 2.039 Å²
0%
100%
213%
7mm

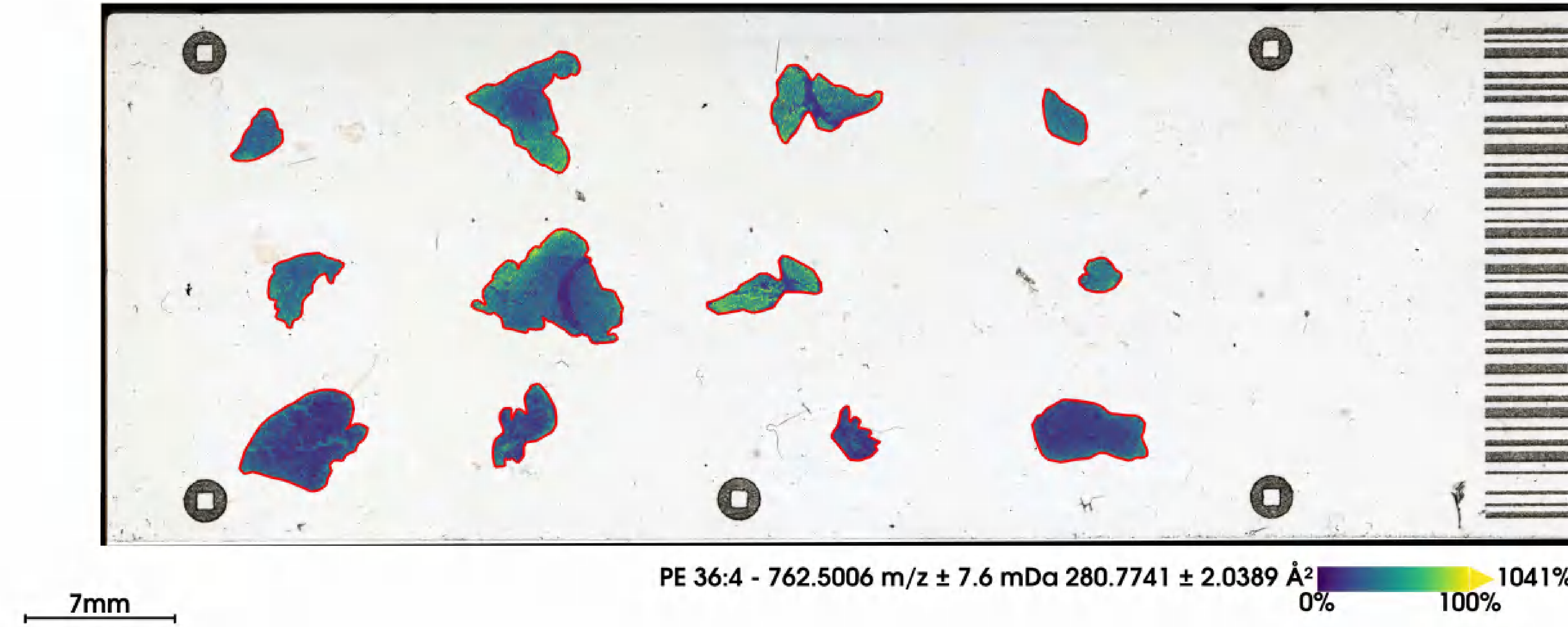
PE 36:4 - 762.5006 m/z ± 7.6 mDa 280.7741 ± 2.0389 Å²
0%
100%
1041%
7mm

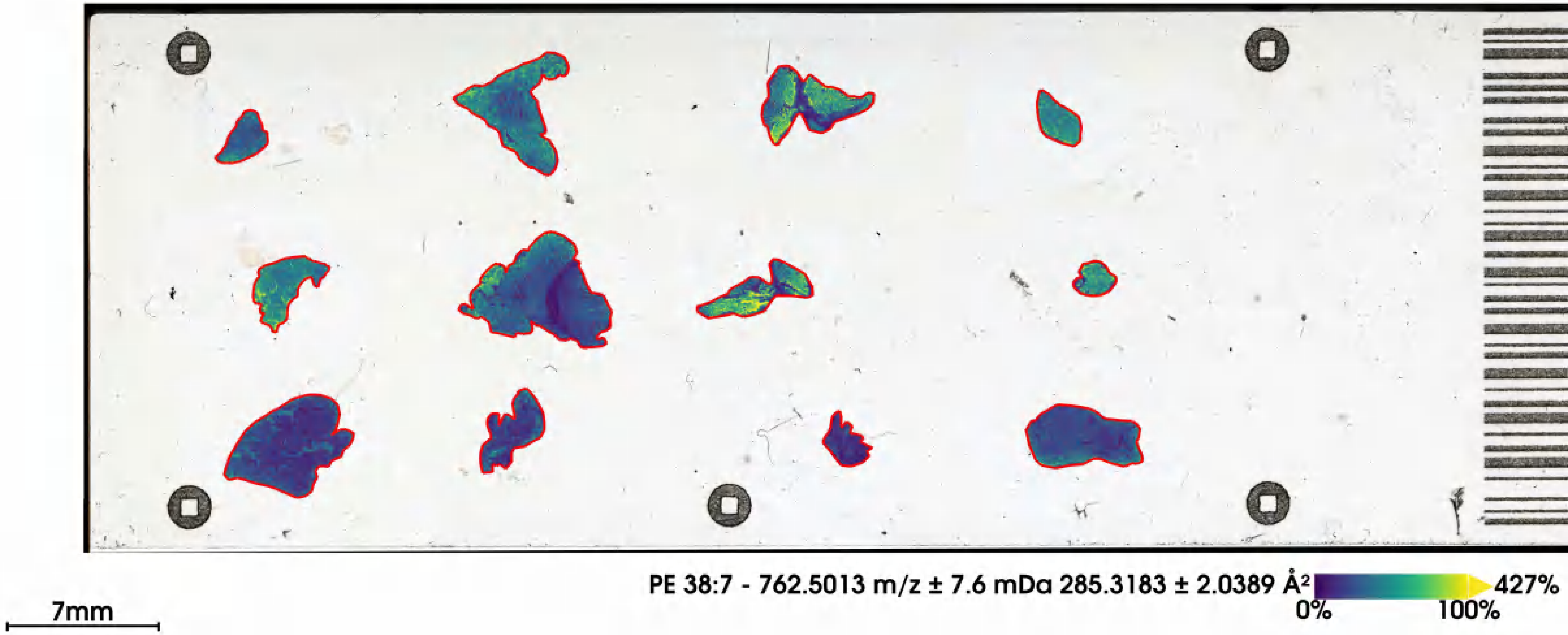
7mm
PE 38:7 - 762.5013 m/z ± 7.6 mDa 285.3183 ± 2.0389 Å²
0%
100%
427%

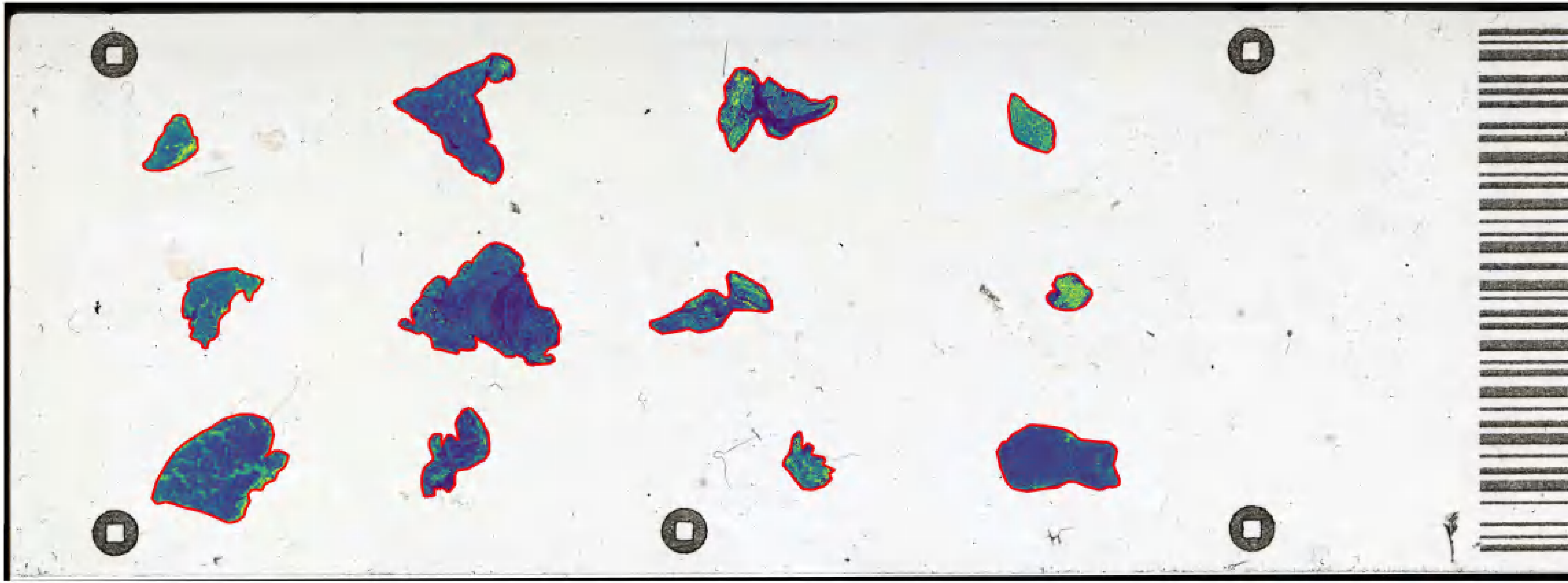

PC 34:0 - 762.6038 m/z ± 7.6 mDa 297.9668 ± 2.0389 Å²
0%
100%
357%
7mm

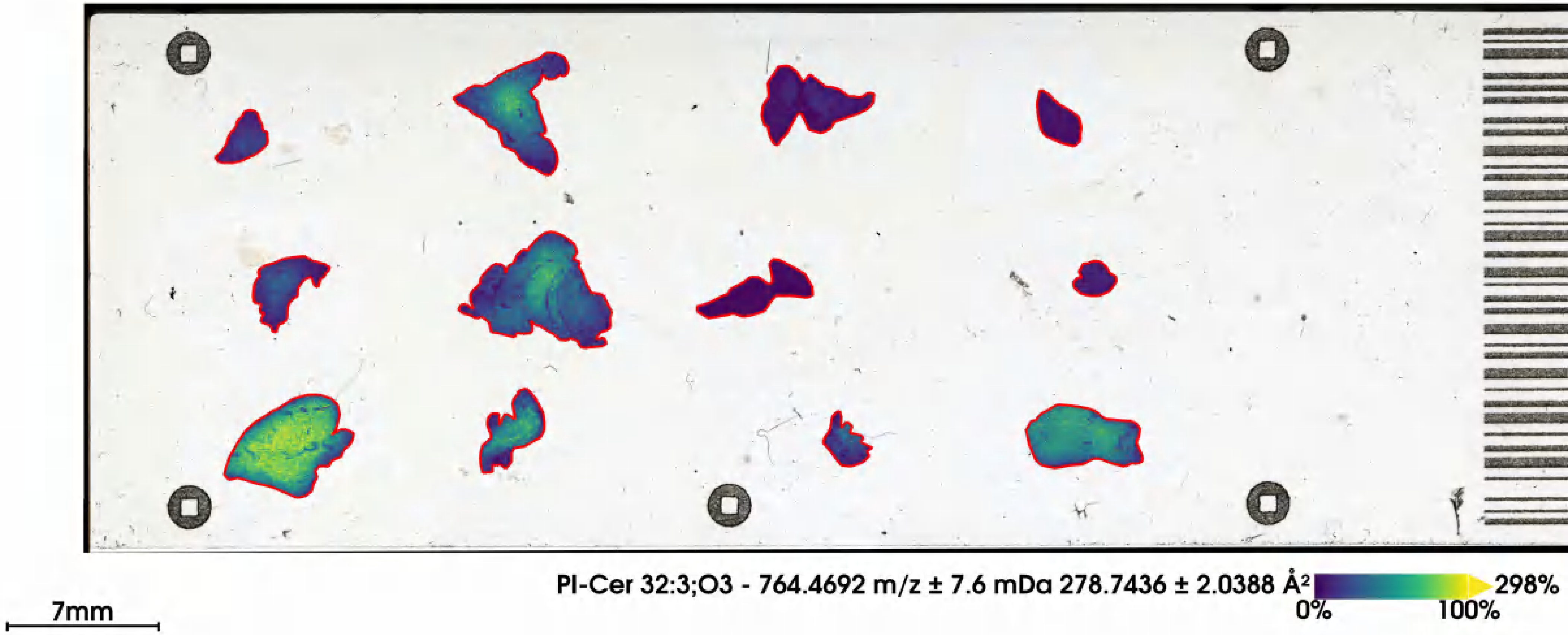

PI-Cer 32:3;O3 - 764.4692 m/z ± 7.6 mDa 278.7436 ± 2.0388 Å²
0%
100%
298%
7mm

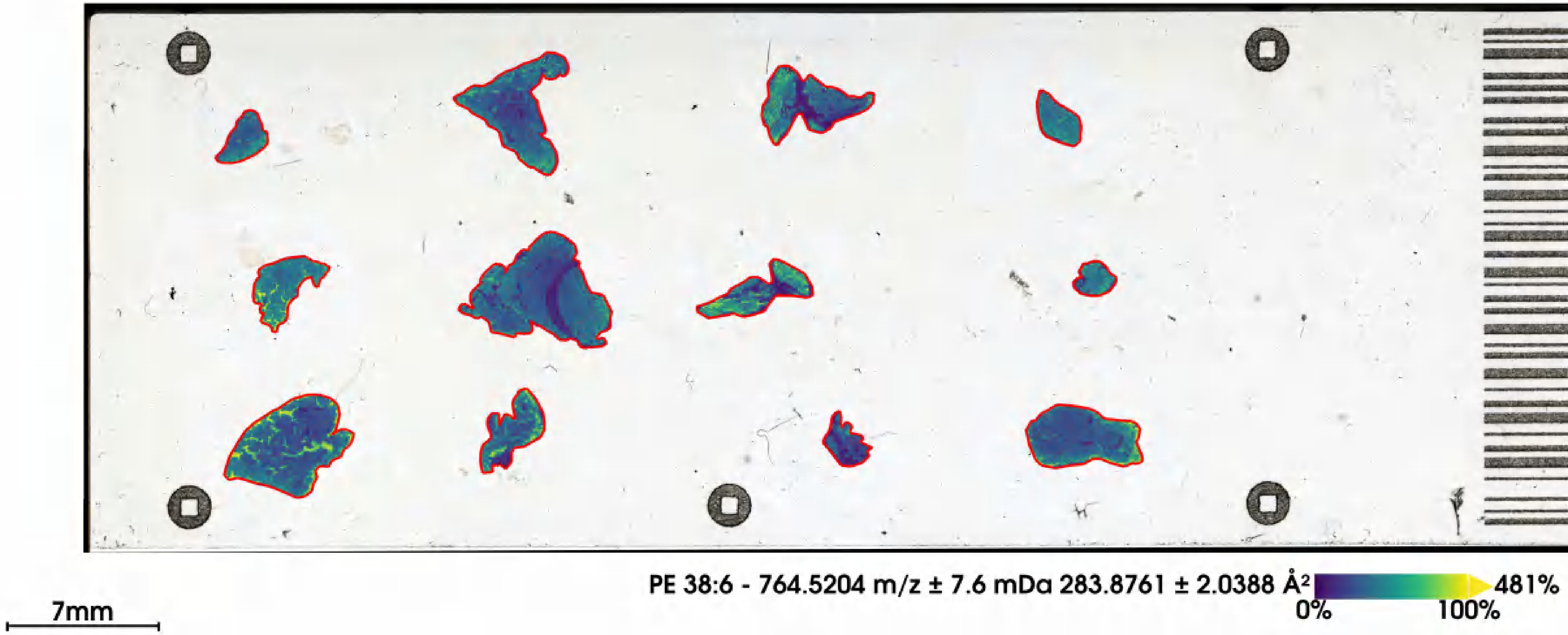
PE 38:6 - 764.5204 m/z ± 7.6 mDa 283.8761 ± 2.0388 Å²
0%
100%
481%
7mm

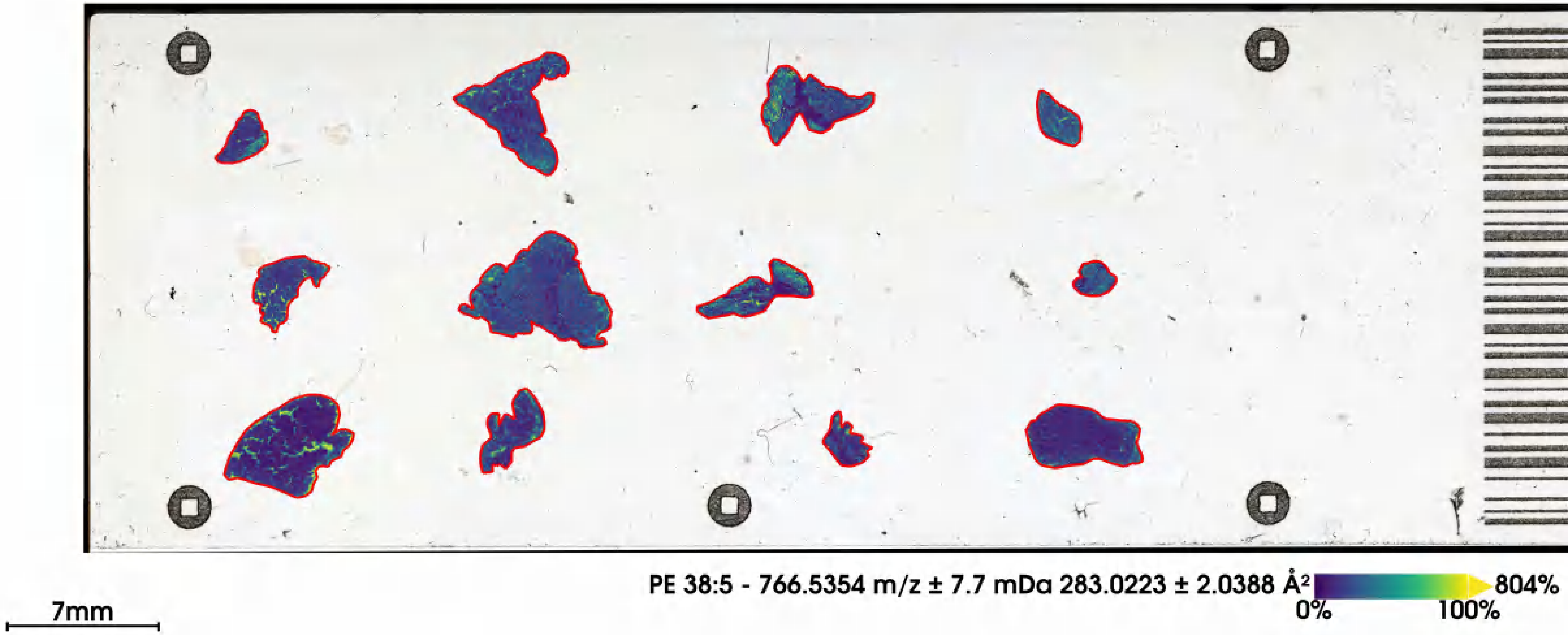
7mm
PE 38:5 - 766.5354 m/z ± 7.7 mDa 283.0223 ± 2.0388 Å²
0%
100%
804%

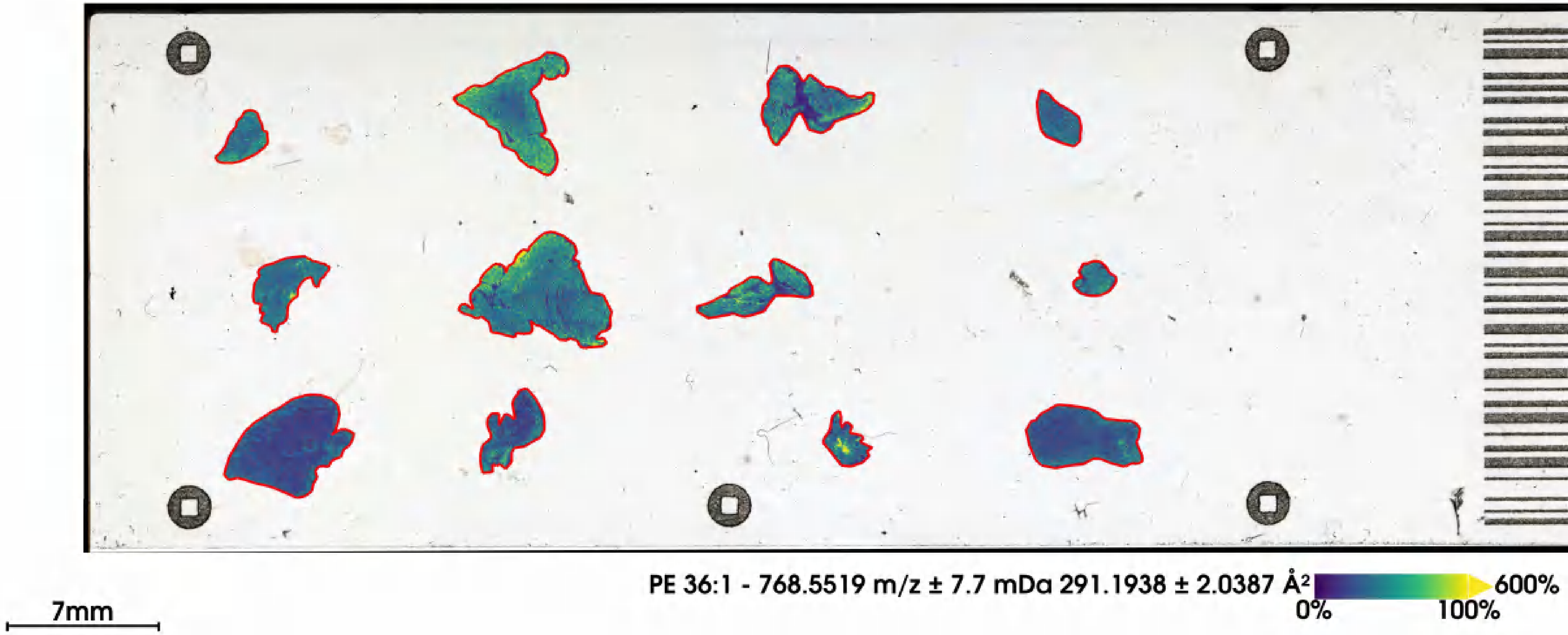

7mm
PE 36:1 - 768.5519 m/z ± 7.7 mDa 291.1938 ± 2.0387 Å²
0%
100%
600%

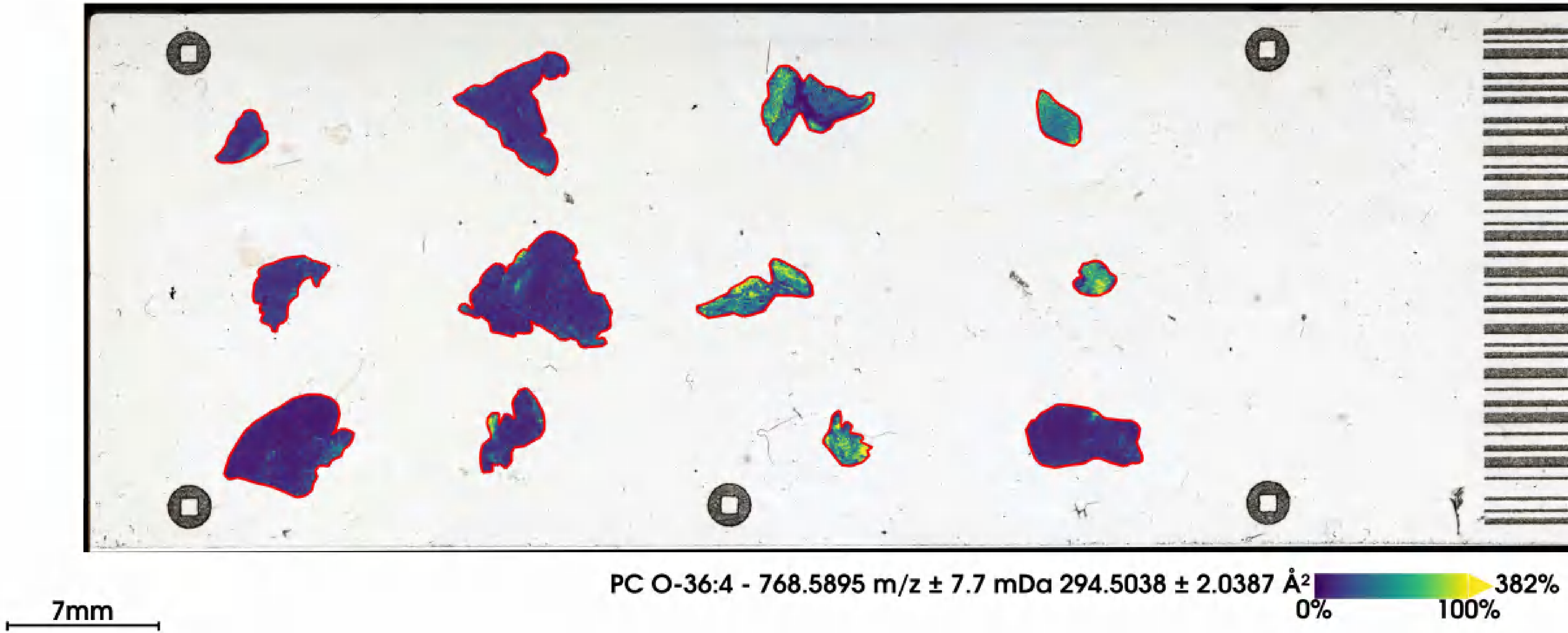
PC O-36:4 - 768.5895 m/z ± 7.7 mDa 294.5038 ± 2.0387 Å²
0%
100%
382%
7mm

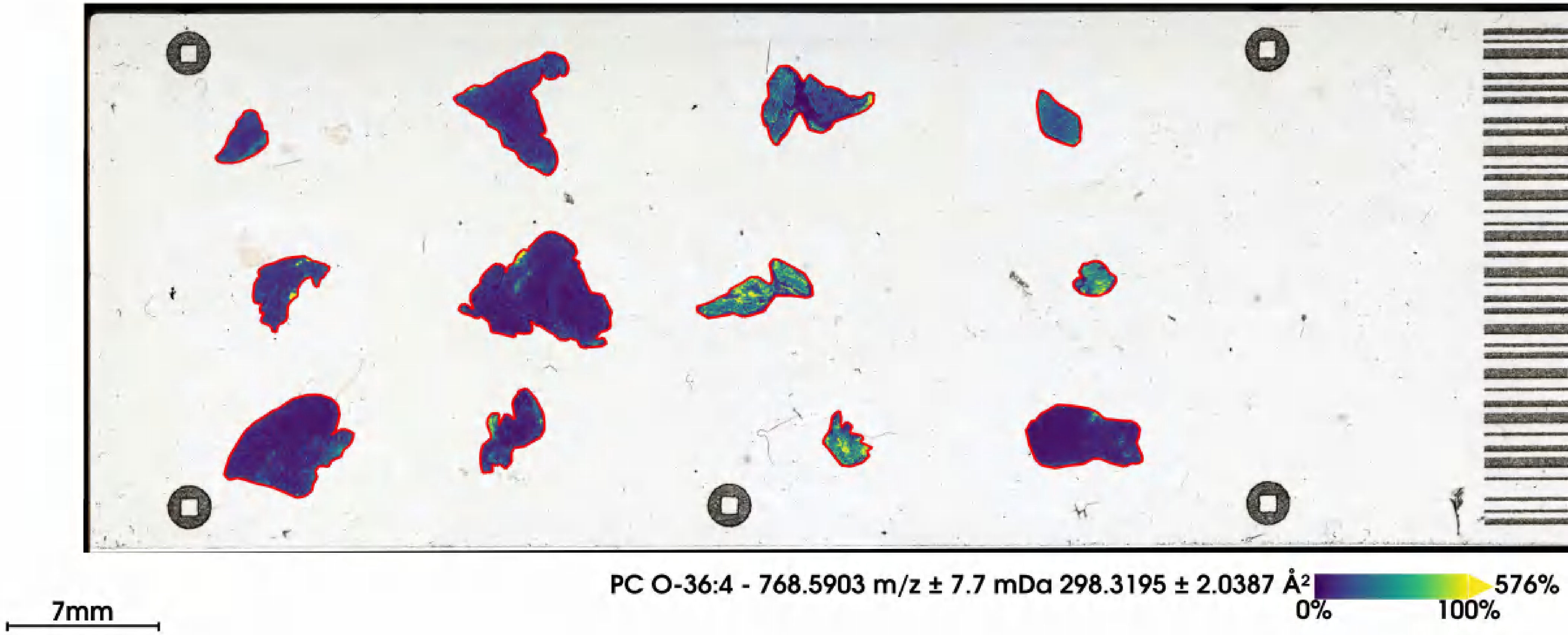
PC O-36:4 - 768.5903 m/z ± 7.7 mDa 298.3195 ± 2.0387 Å²
576%
0%
100%
7mm

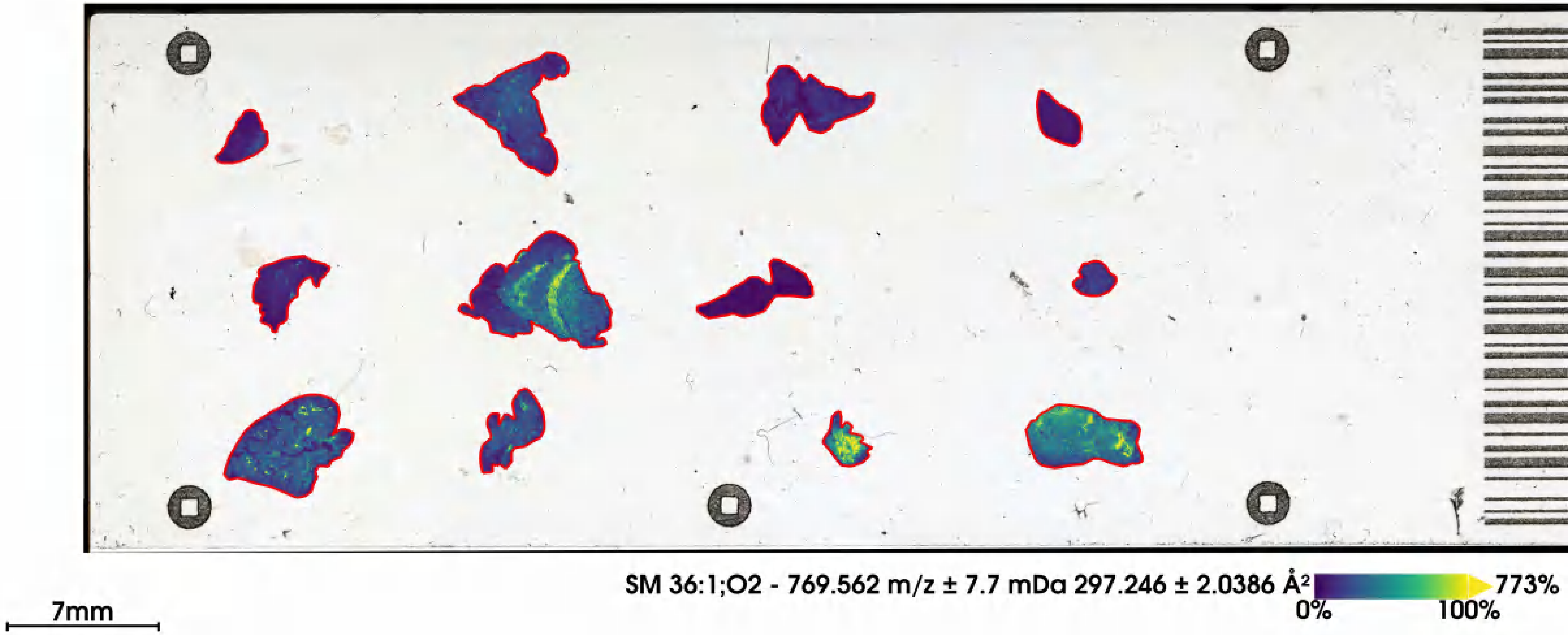
SM 36:1;O2 - 769.562 m/z ± 7.7 mDa 297.246 ± 2.0386 Å²
773%
0%
100%
7mm

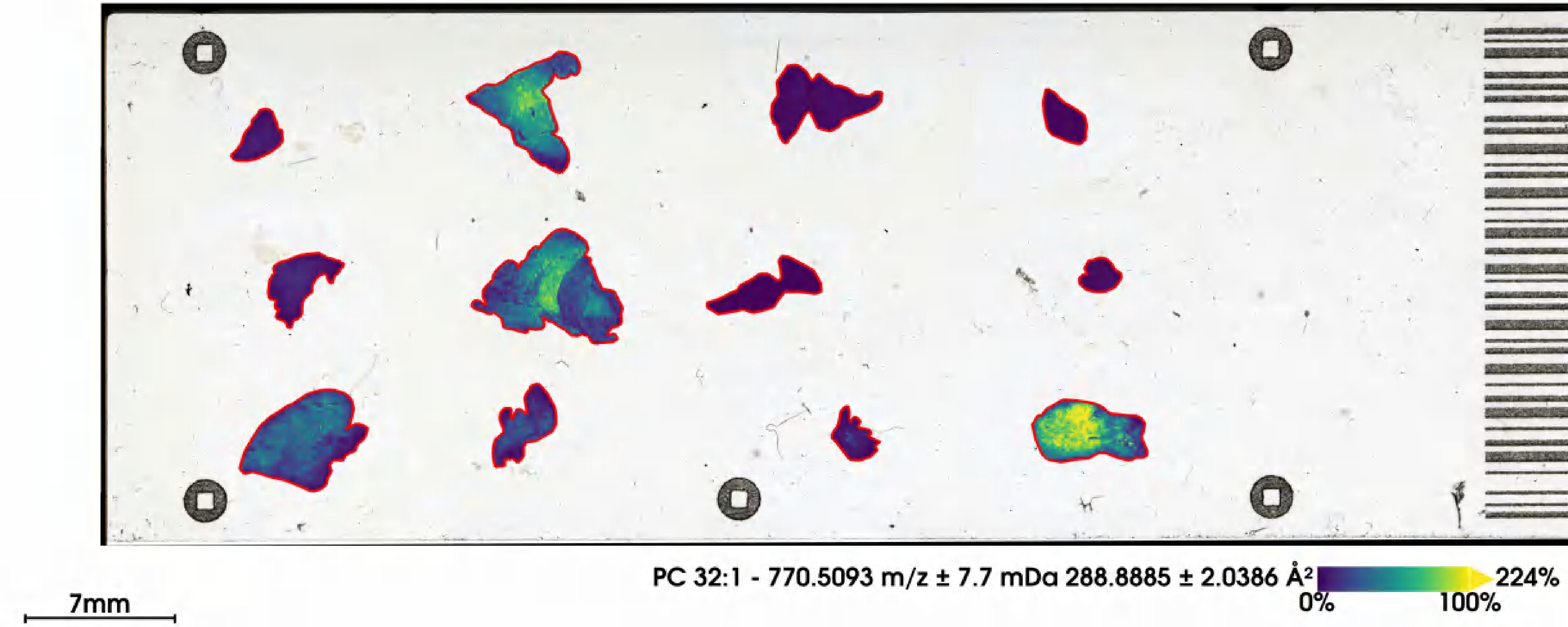
PC 32:1 - 770.5093 m/z ± 7.7 mDa 288.8885 ± 2.0386 Å²
224%
0%
100%
7mm

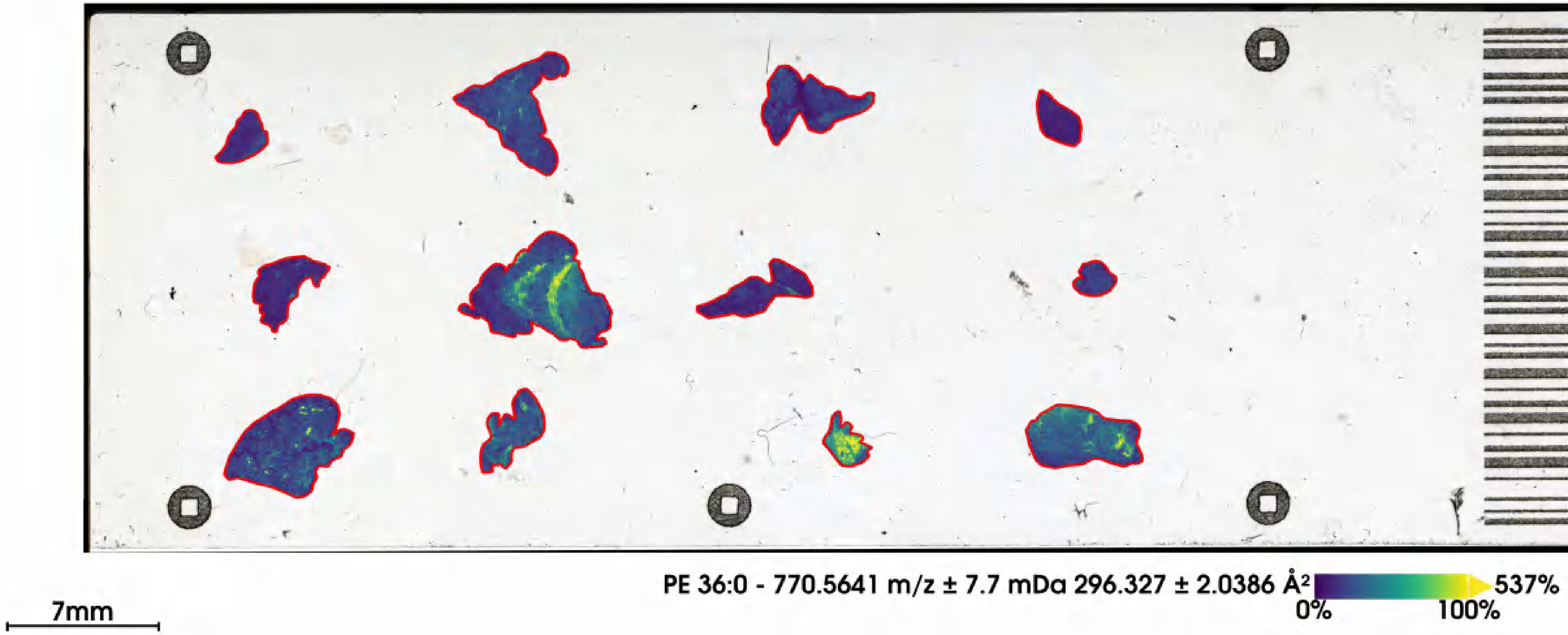

PE 36:0 - 770.5641 m/z ± 7.7 mDa 296.327 ± 2.0386 Å²
0%
100%
537%
7mm

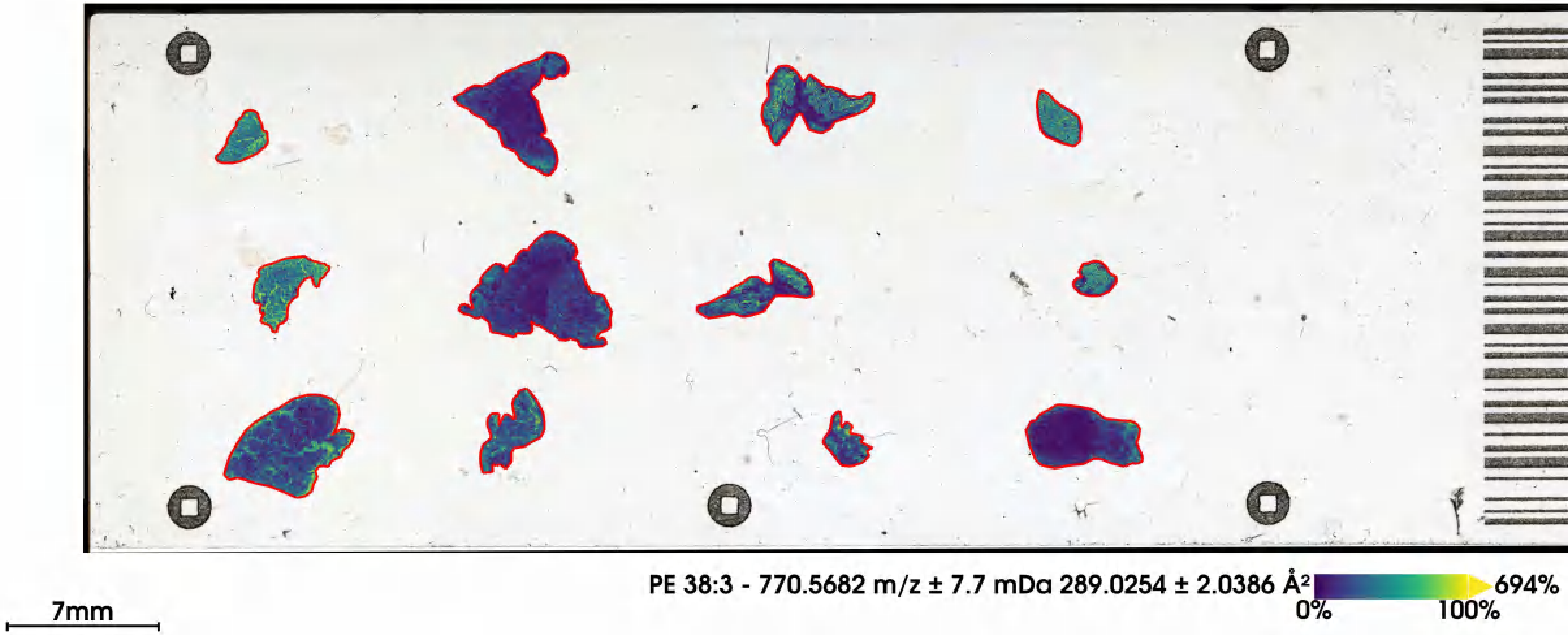
PE 38:3 - 770.5682 m/z ± 7.7 mDa 289.0254 ± 2.0386 Å²
0%
100%
694%
7mm

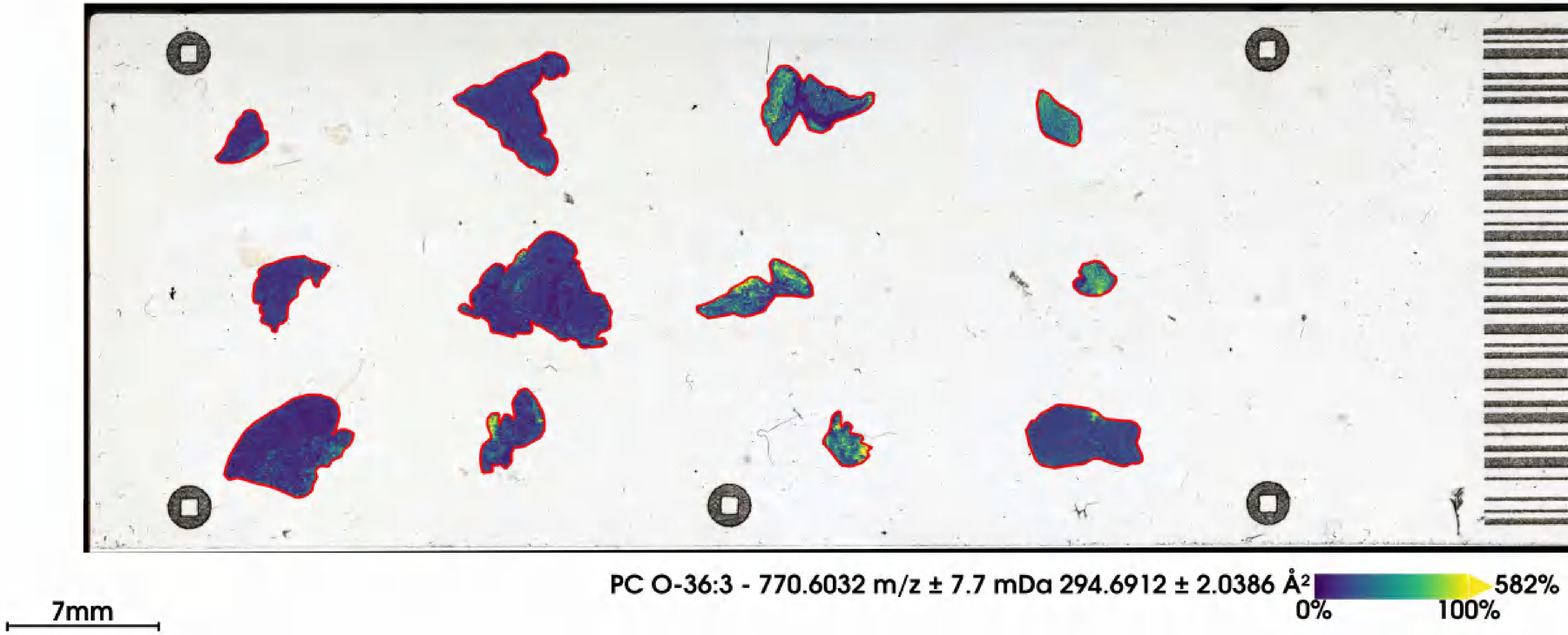
PC O-36:3 - 770.6032 m/z ± 7.7 mDa 294.6912 ± 2.0386 Å²
0%
100%
582%
7mm

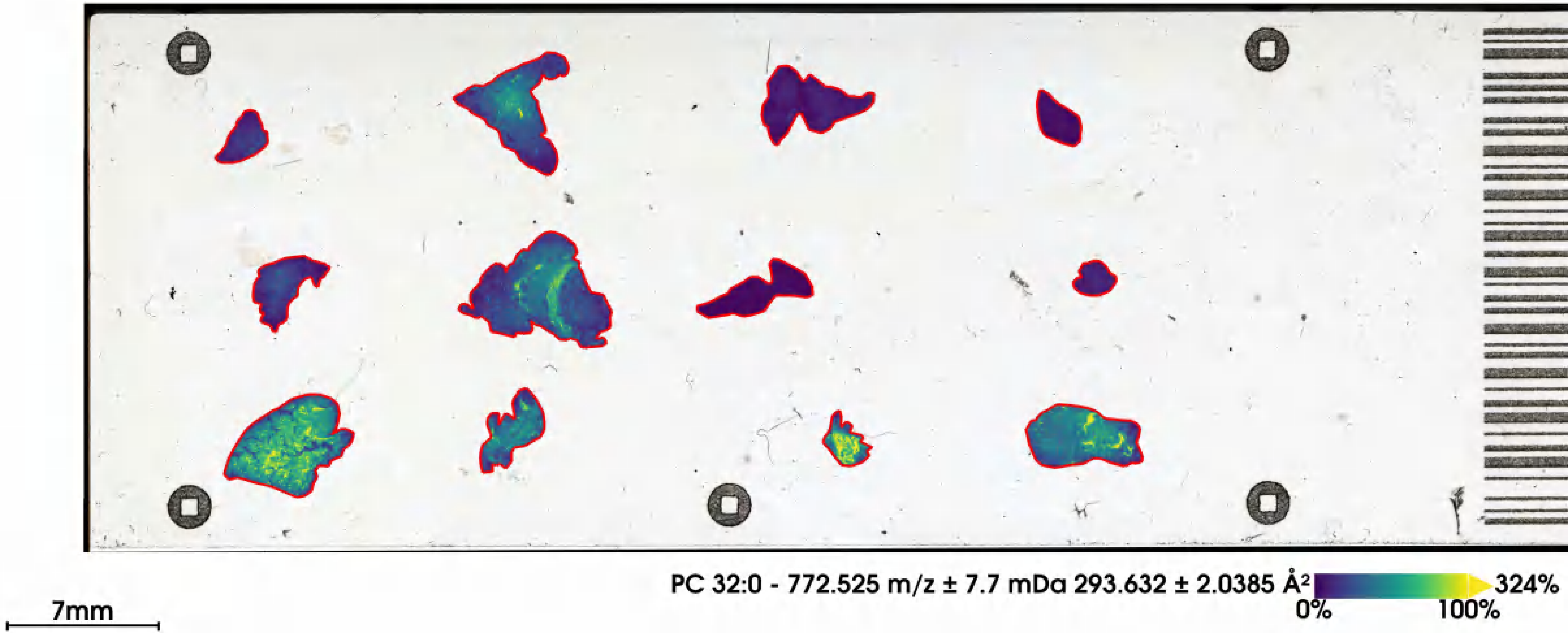

PC 32:0 - 772.525 m/z ± 7.7 mDa 293.632 ± 2.0385 Å²
0%
100%
324%
7mm

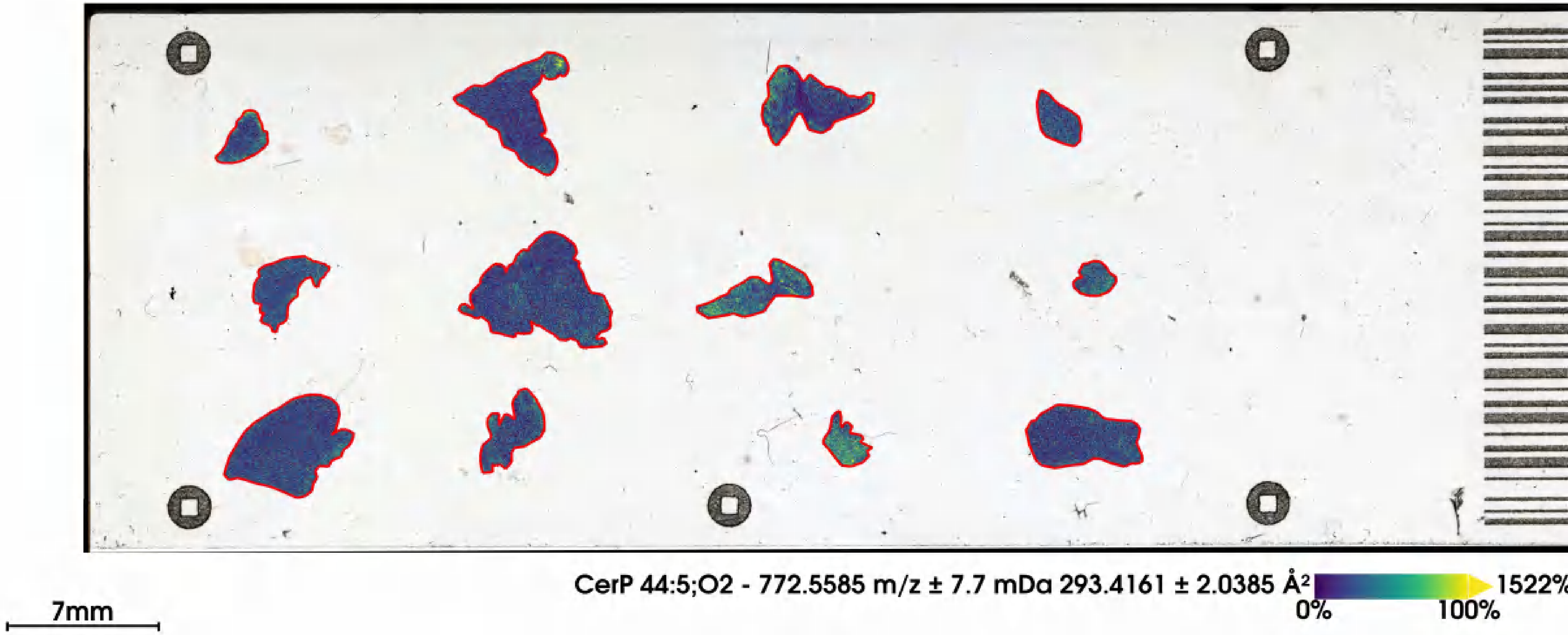

CerP 44:5;O2 - 772.5585 m/z ± 7.7 mDa 293.4161 ± 2.0385 Å²
0%
100%
1522%
7mm

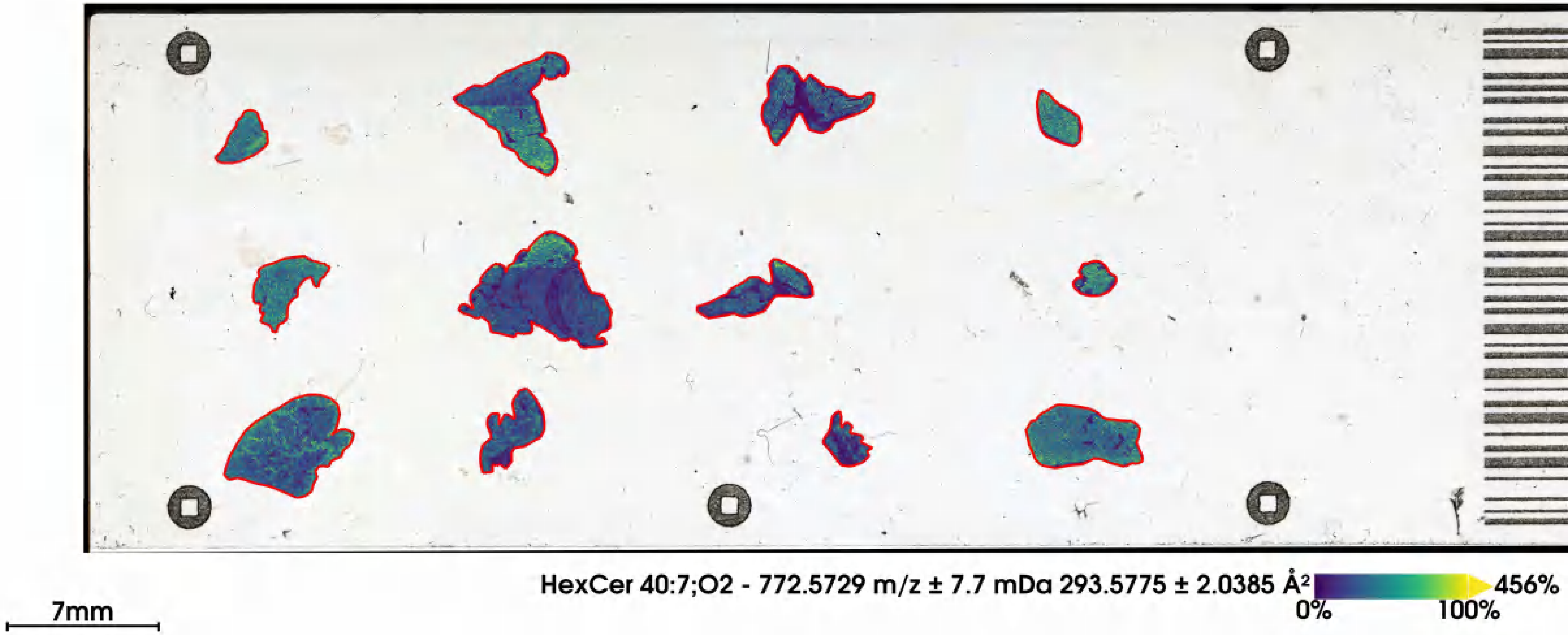

7mm
HexCer 40:7;O2 - 772.5729 m/z ± 7.7 mDa 293.5775 ± 2.0385 Å²
0%
100%
456%

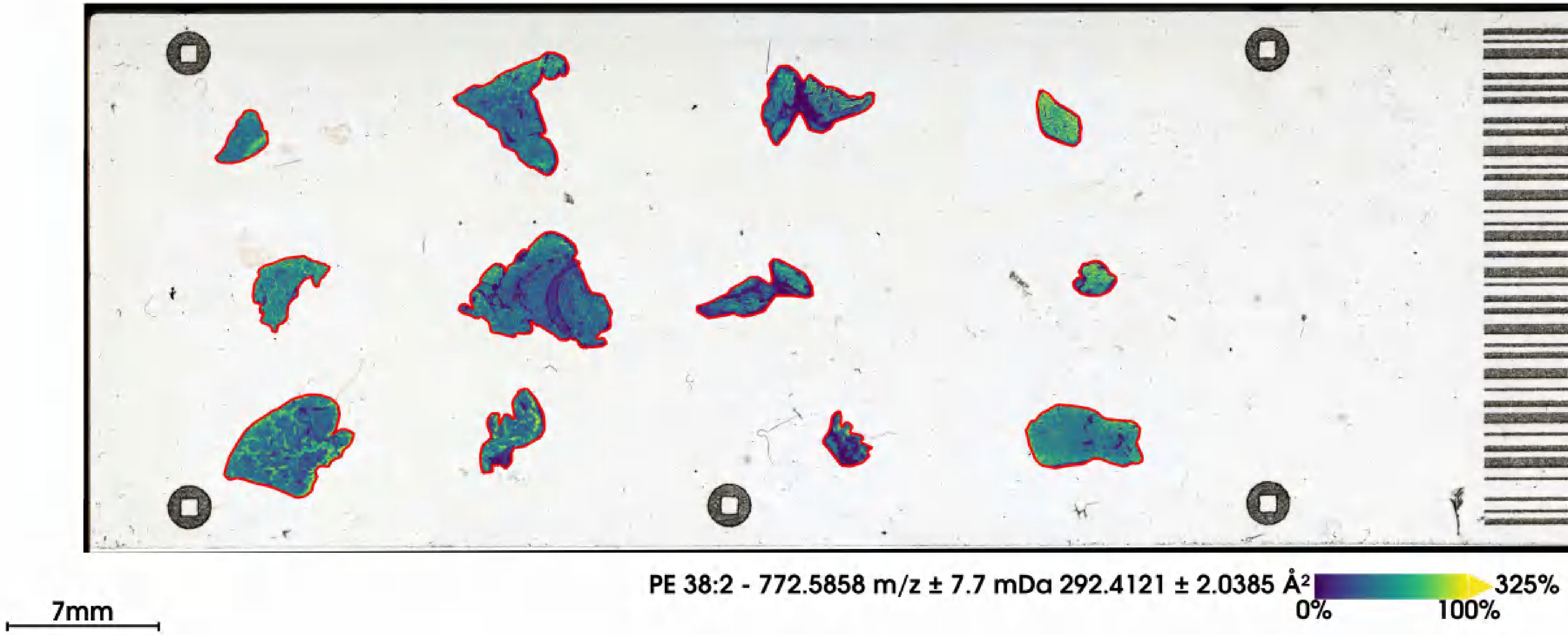

PE 38:2 - 772.5858 m/z ± 7.7 mDa 292.4121 ± 2.0385 Å²
0%
100%
325%
7mm

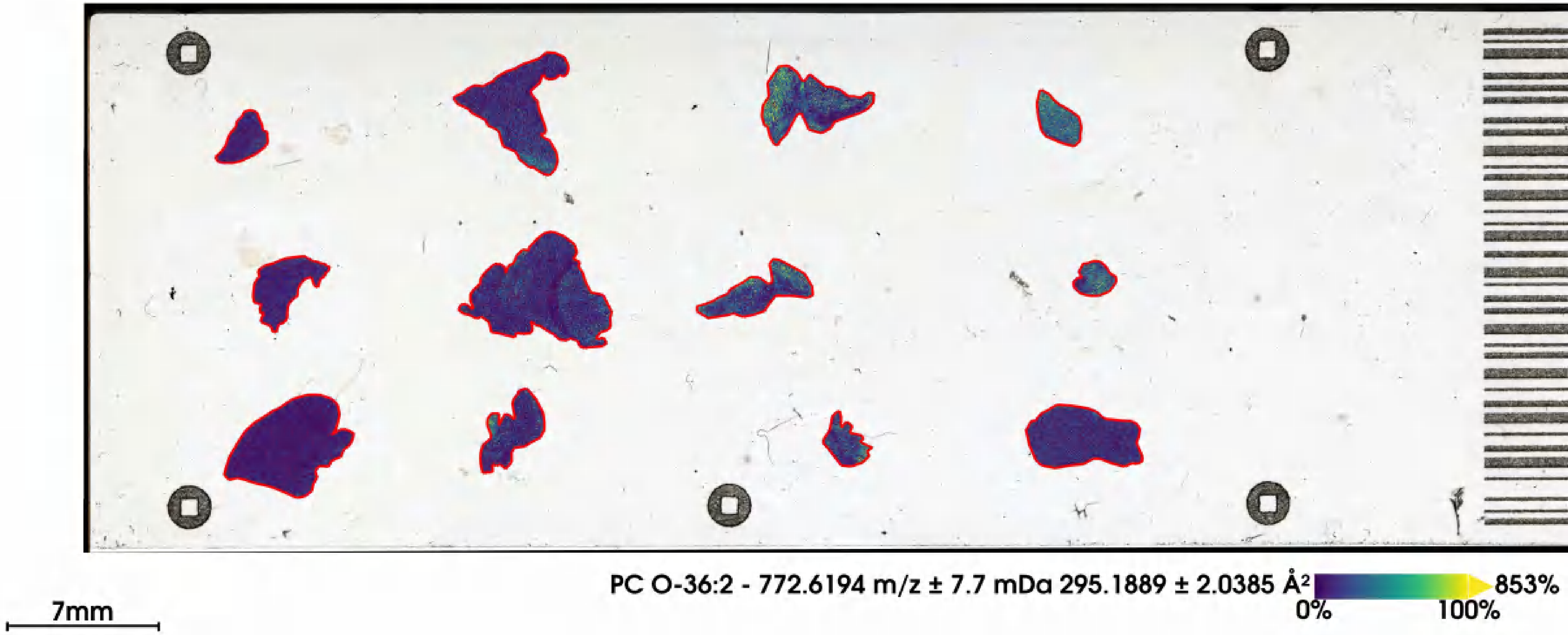
PC O-36:2 - 772.6194 m/z ± 7.7 mDa 295.1889 ± 2.0385 Å²
0%
100%
853%
7mm

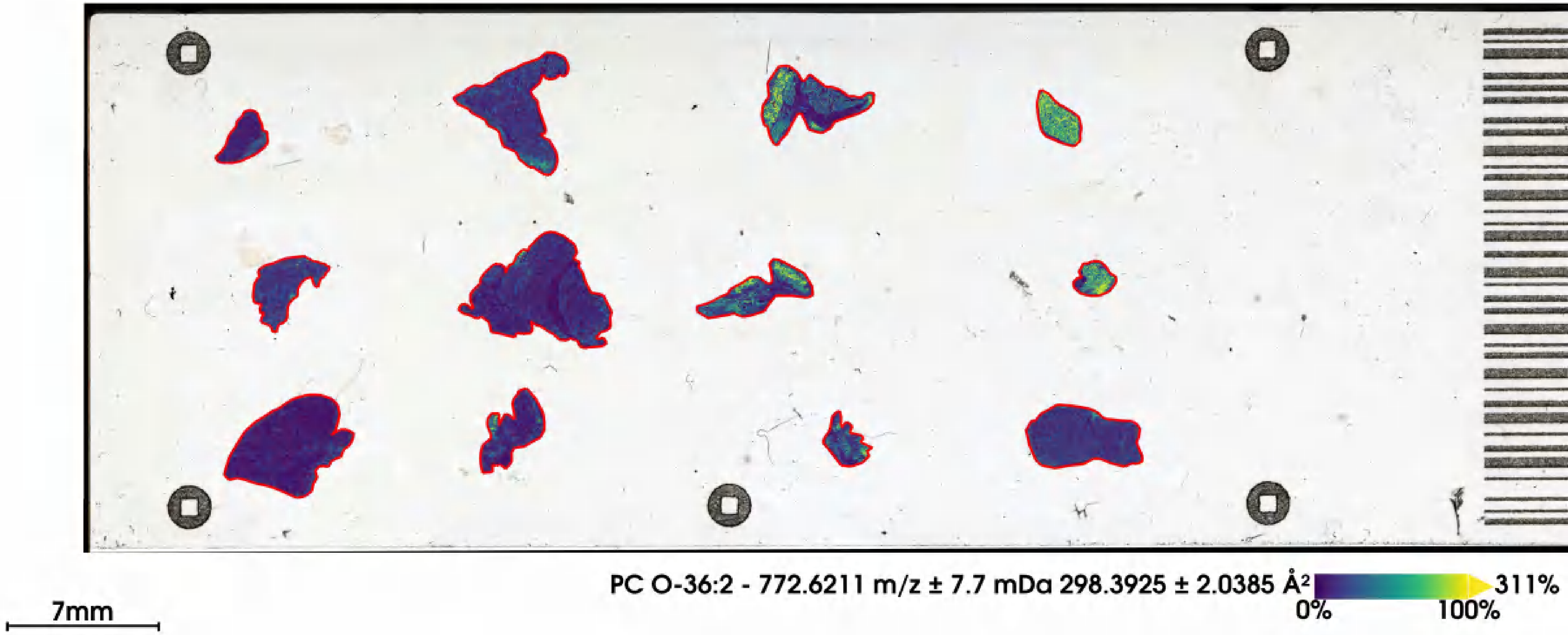

PC O-36:2 - 772.6211 m/z ± 7.7 mDa 298.3925 ± 2.0385 Å²
0%
100%
311%
7mm

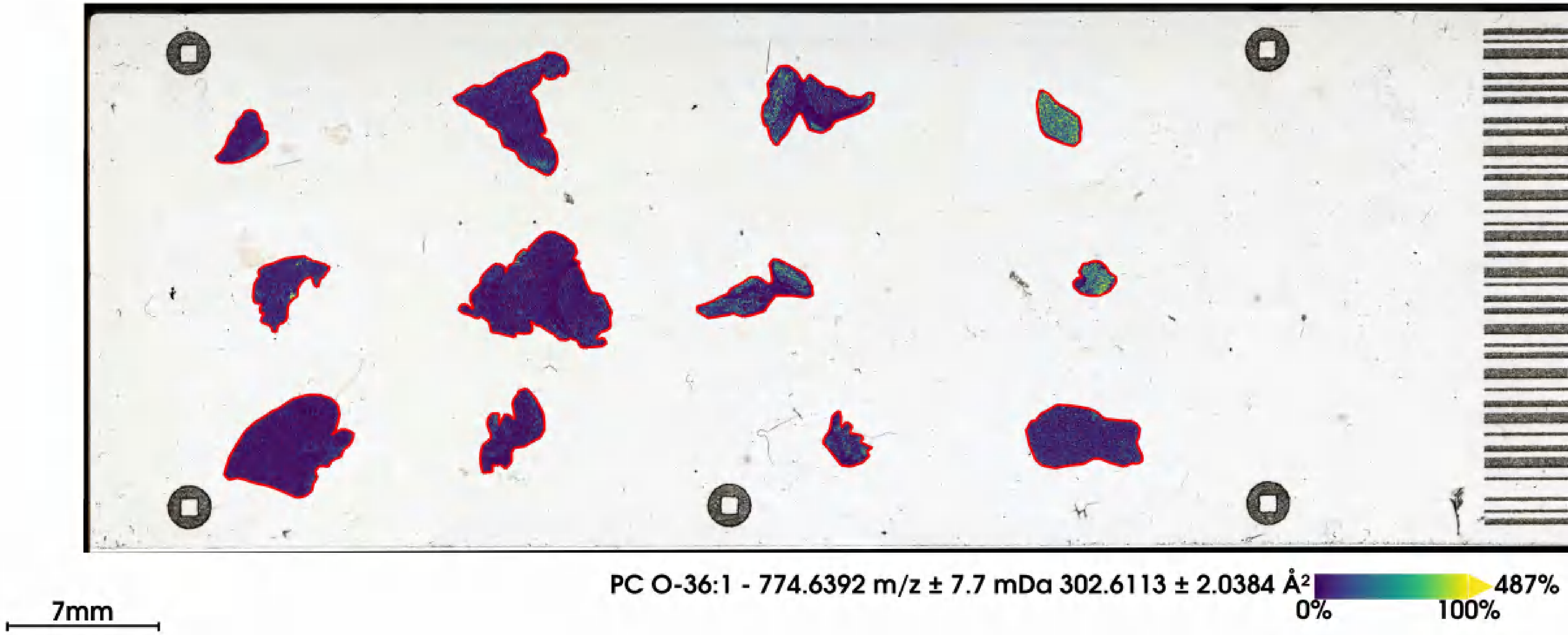

PC O-36:1 - 774.6392 m/z ± 7.7 mDa 302.6113 ± 2.0384 Å²
0%
100%
487%
7mm

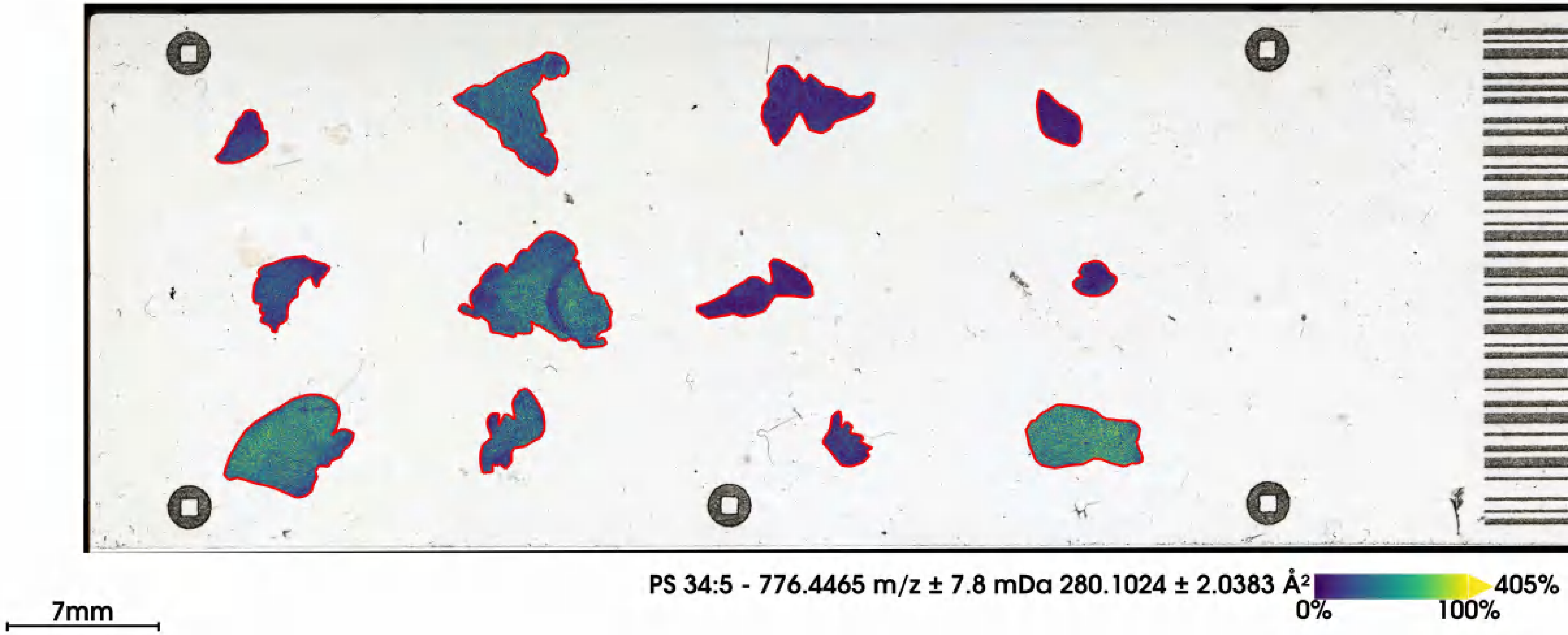
PS 34:5 - 776.4465 m/z ± 7.8 mDa 280.1024 ± 2.0383 Å²
0%
100%
405%
7mm

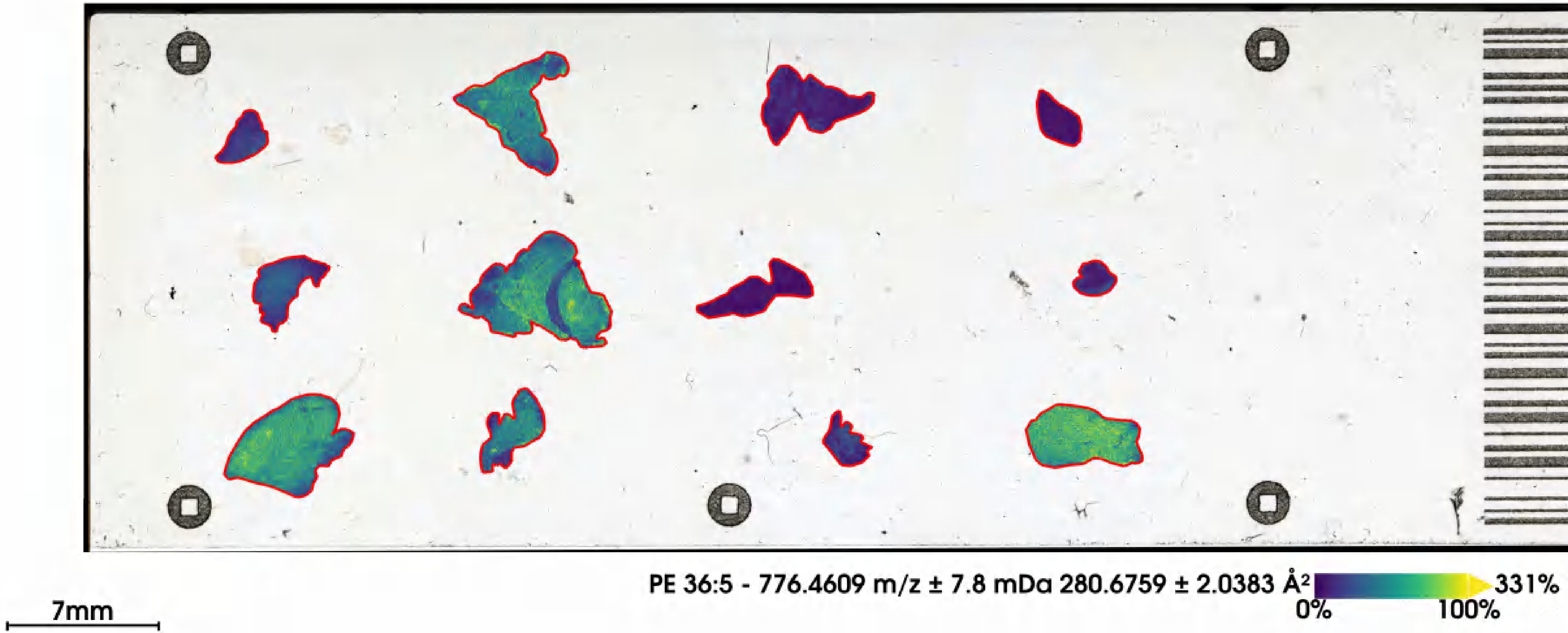

PE 36:5 - 776.4609 m/z ± 7.8 mDa 280.6759 ± 2.0383 Å²
0%
100%
331%
7mm

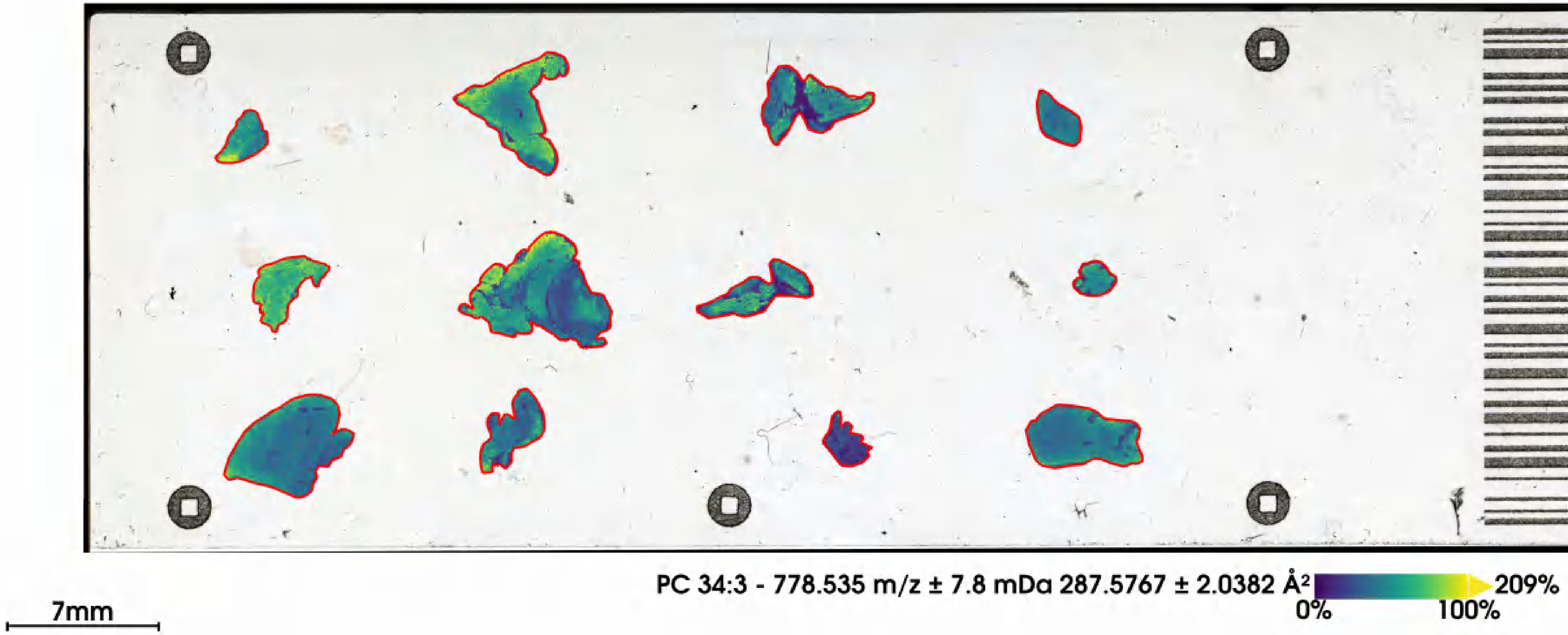

PC 34:3 - 778.535 m/z ± 7.8 mDa 287.5767 ± 2.0382 Å²
0%
100%
209%
7mm

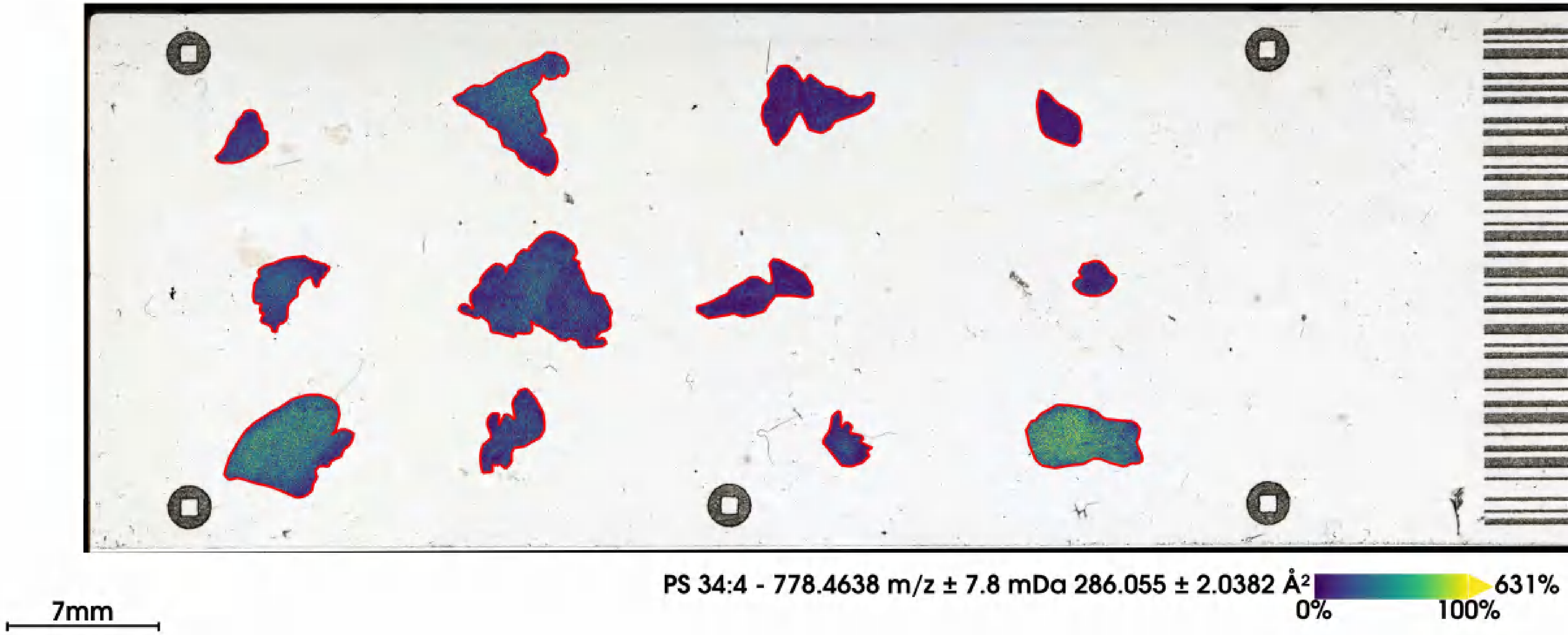
PS 34:4 - 778.4638 m/z ± 7.8 mDa 286.055 ± 2.0382 Å²
0%
100%
631%
7mm

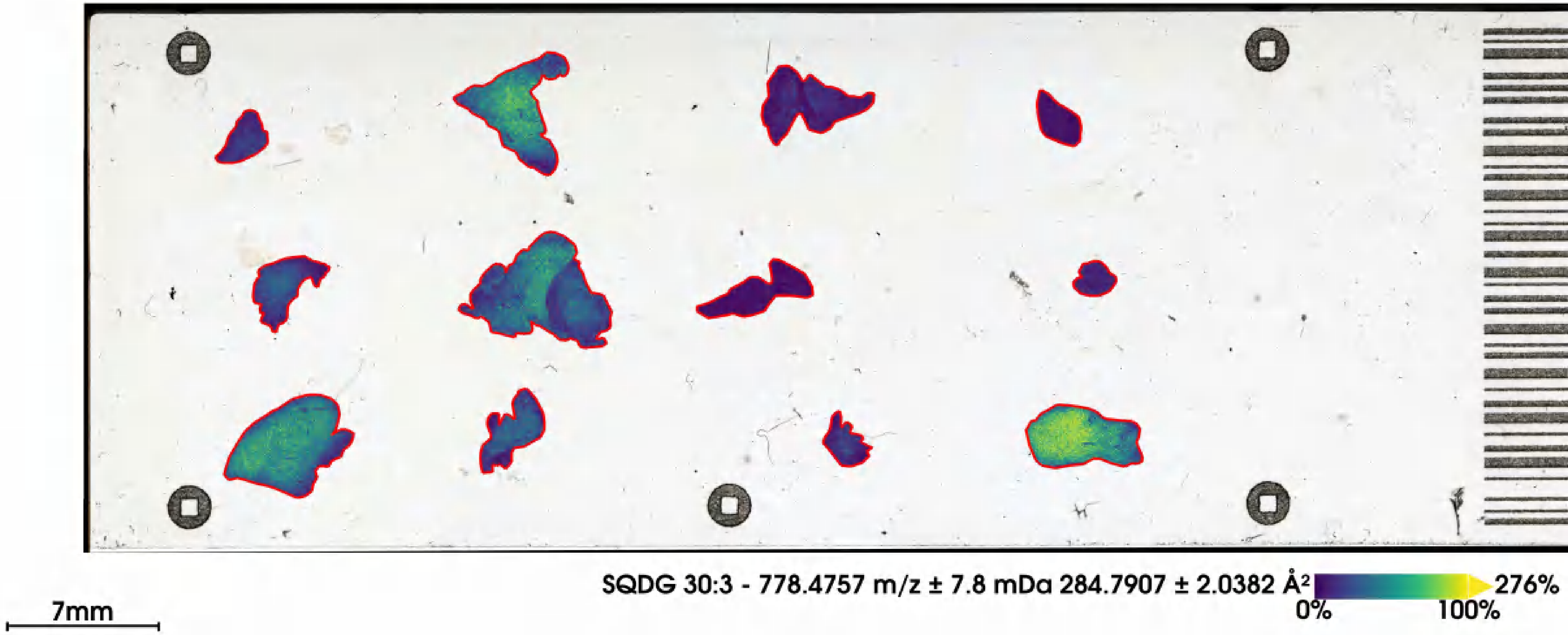
SQDG 30:3 - 778.4757 m/z ± 7.8 mDa 284.7907 ± 2.0382 Å²
0%
100%
276%
7mm

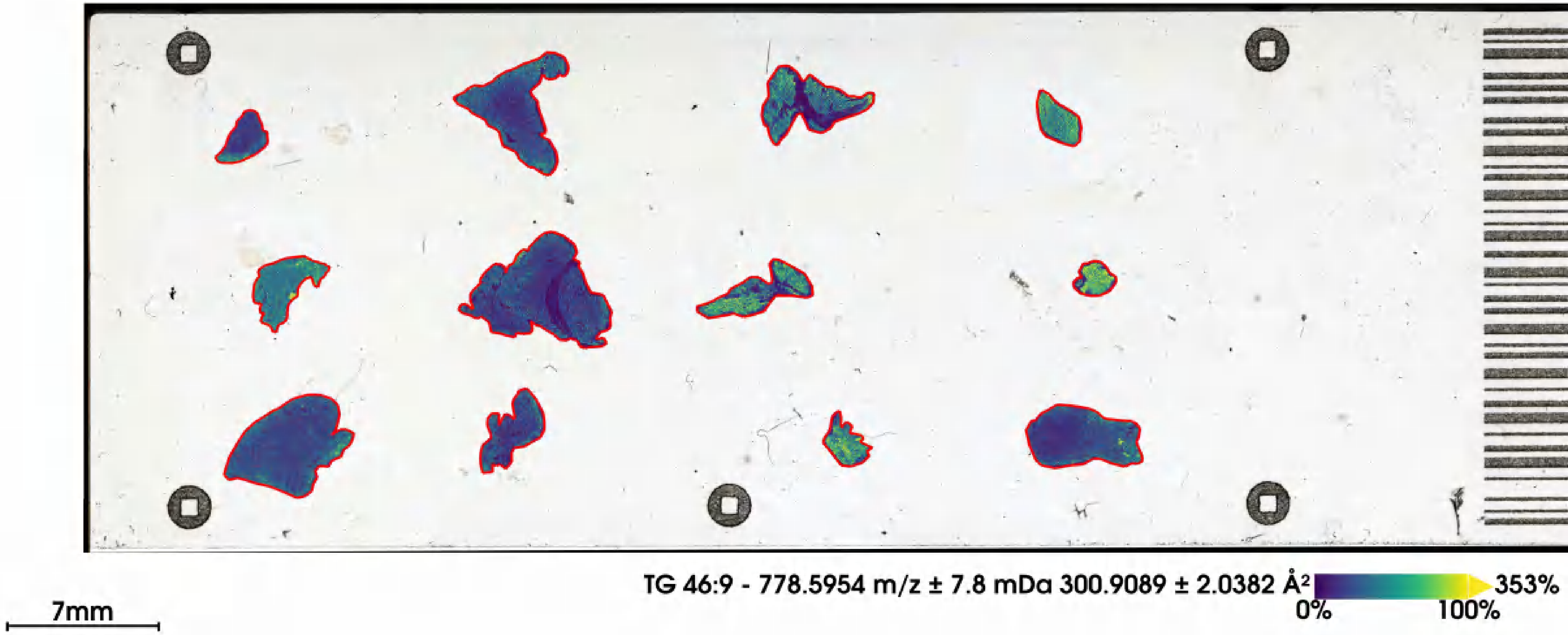
TG 46:9 - 778.5954 m/z ± 7.8 mDa 300.9089 ± 2.0382 Å²
0%
100%
353%
7mm

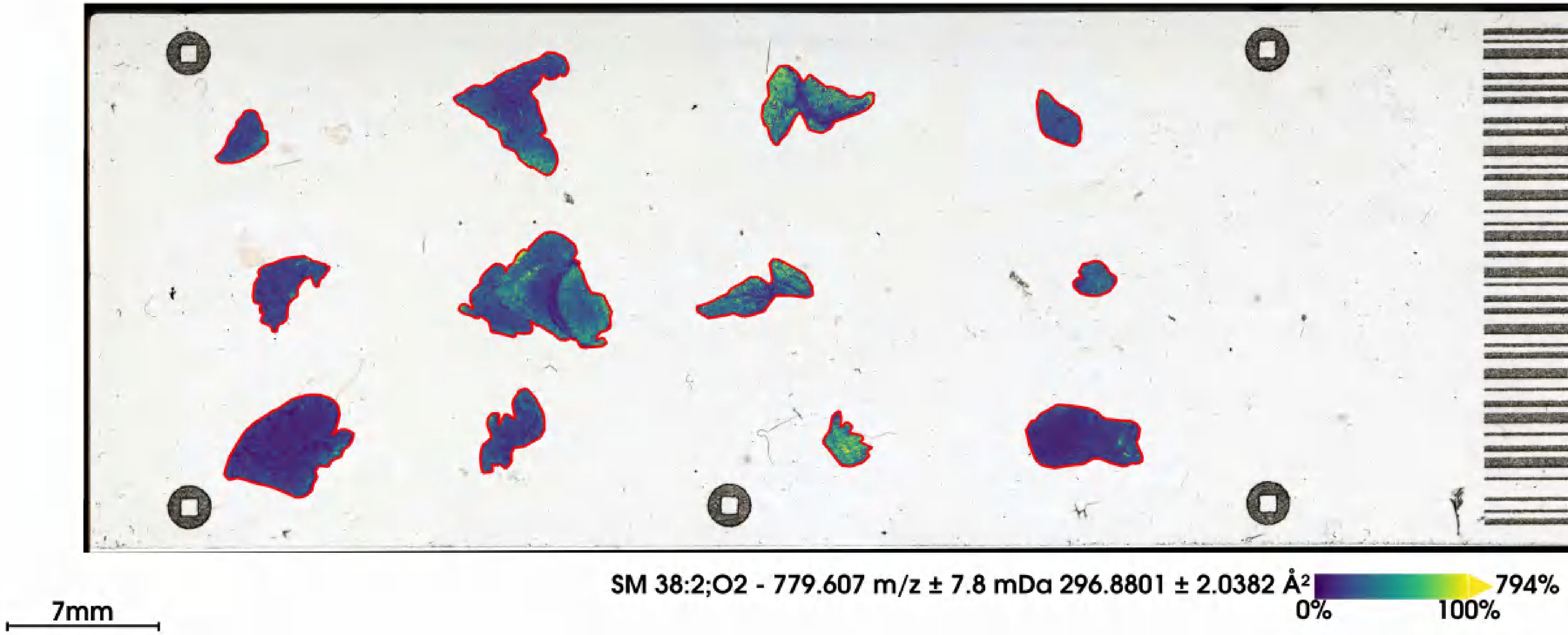

SM 38:2;O2 - 779.607 m/z ± 7.8 mDa 296.8801 ± 2.0382 Å²
794%
0%
100%
7mm

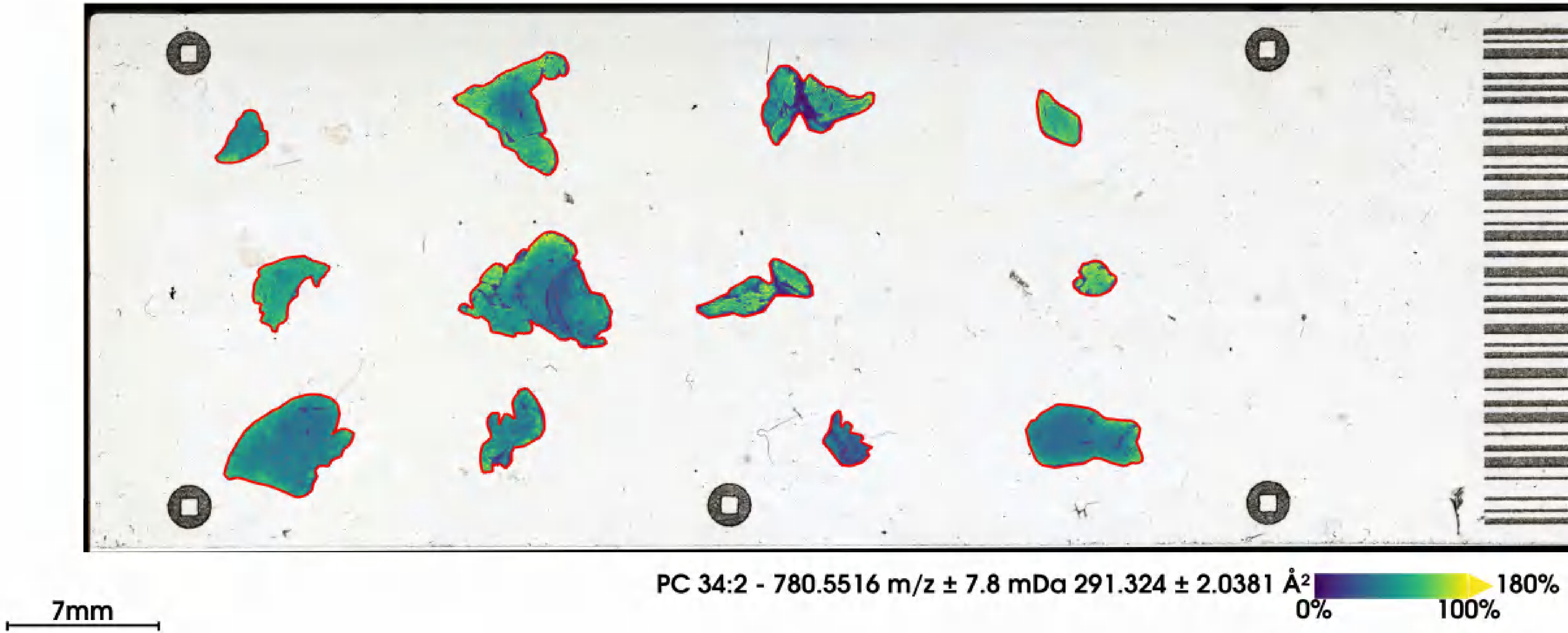

PC 34:2 - 780.5516 m/z ± 7.8 mDa 291.324 ± 2.0381 Å²
0%
100%
180%
7mm

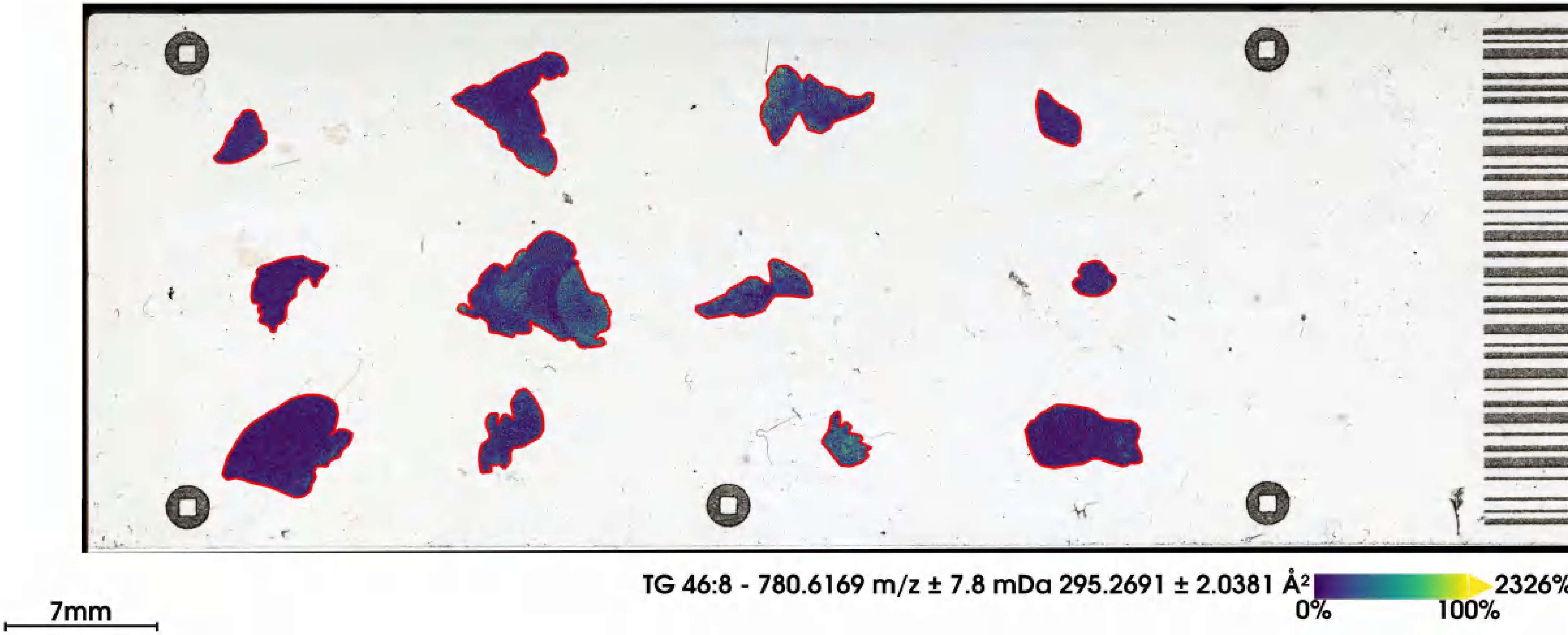
7mm
TG 46:8 - 780.6169 m/z ± 7.8 mDa 295.2691 ± 2.0381 Å²
0%
100%
2326%

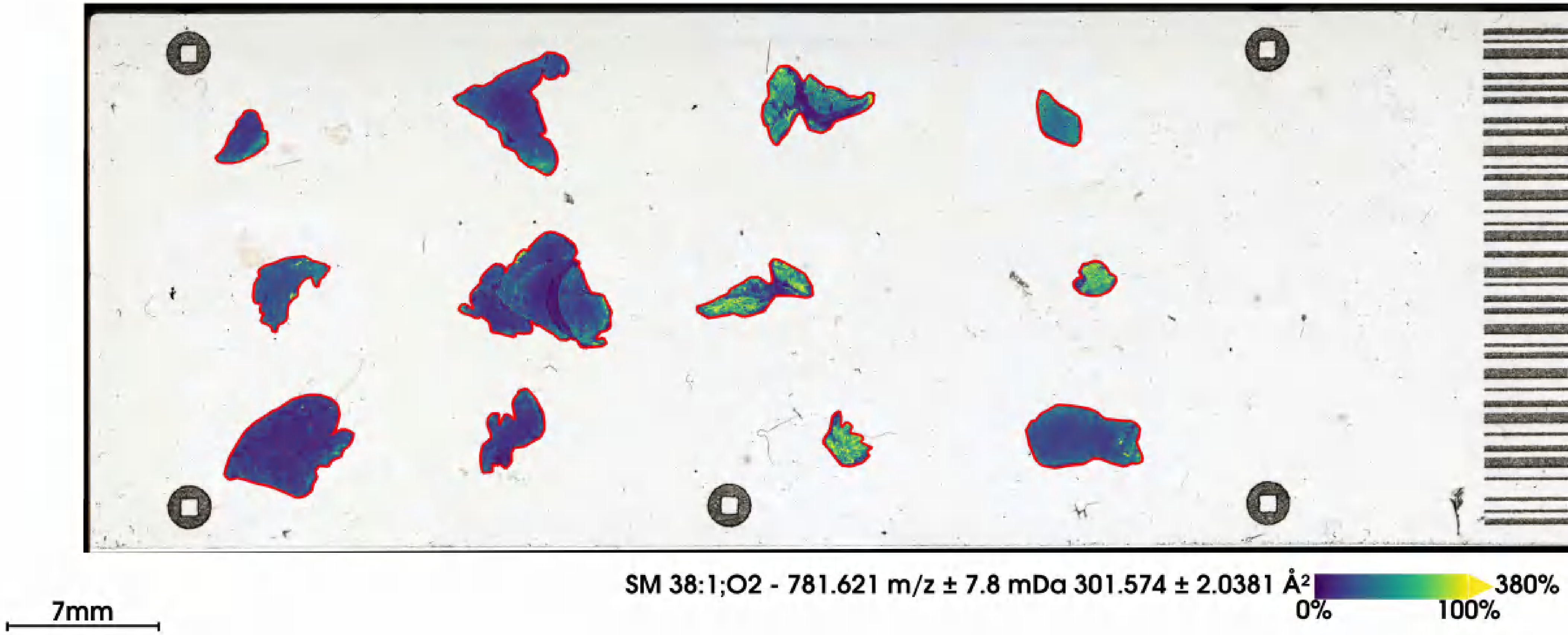
SM 38:1;O2 - 781.621 m/z ± 7.8 mDa 301.574 ± 2.0381 Å²
0%
100%
380%
7mm

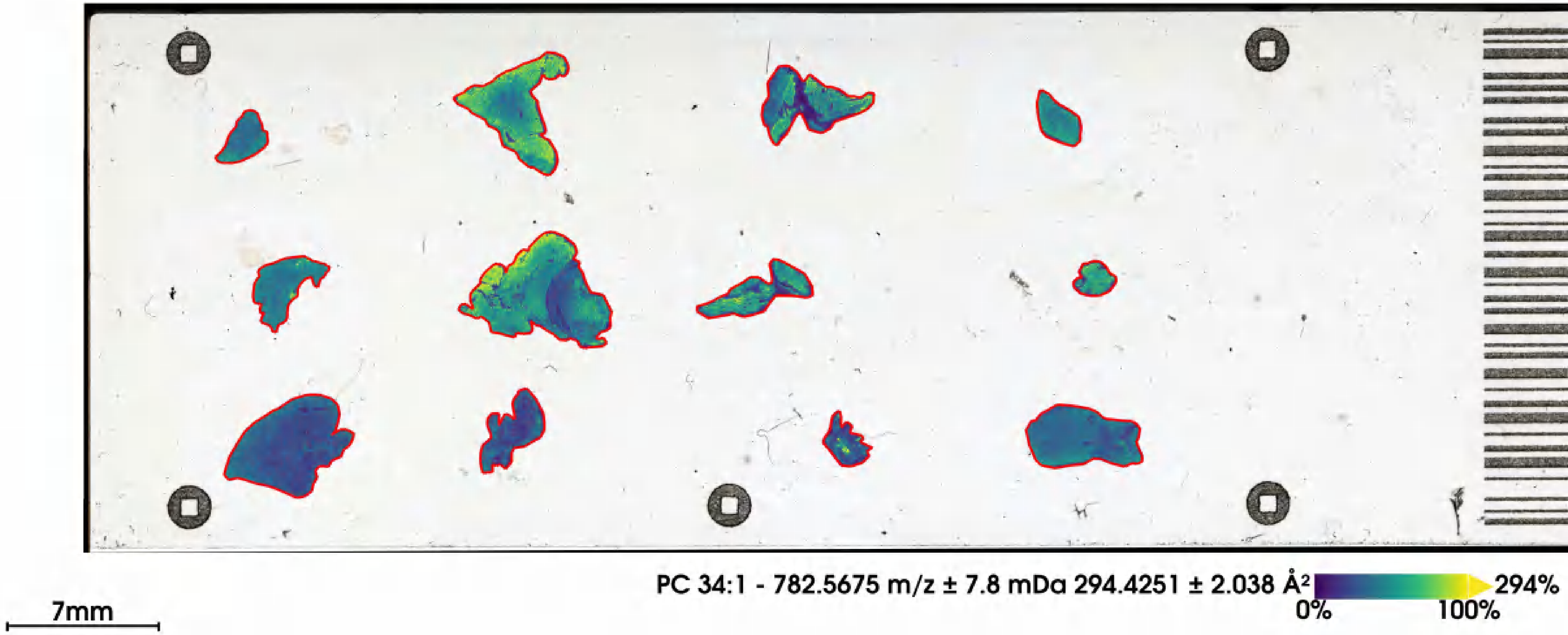

PC 34:1 - 782.5675 m/z ± 7.8 mDa 294.4251 ± 2.038 Å²
0%
100%
294%
7mm

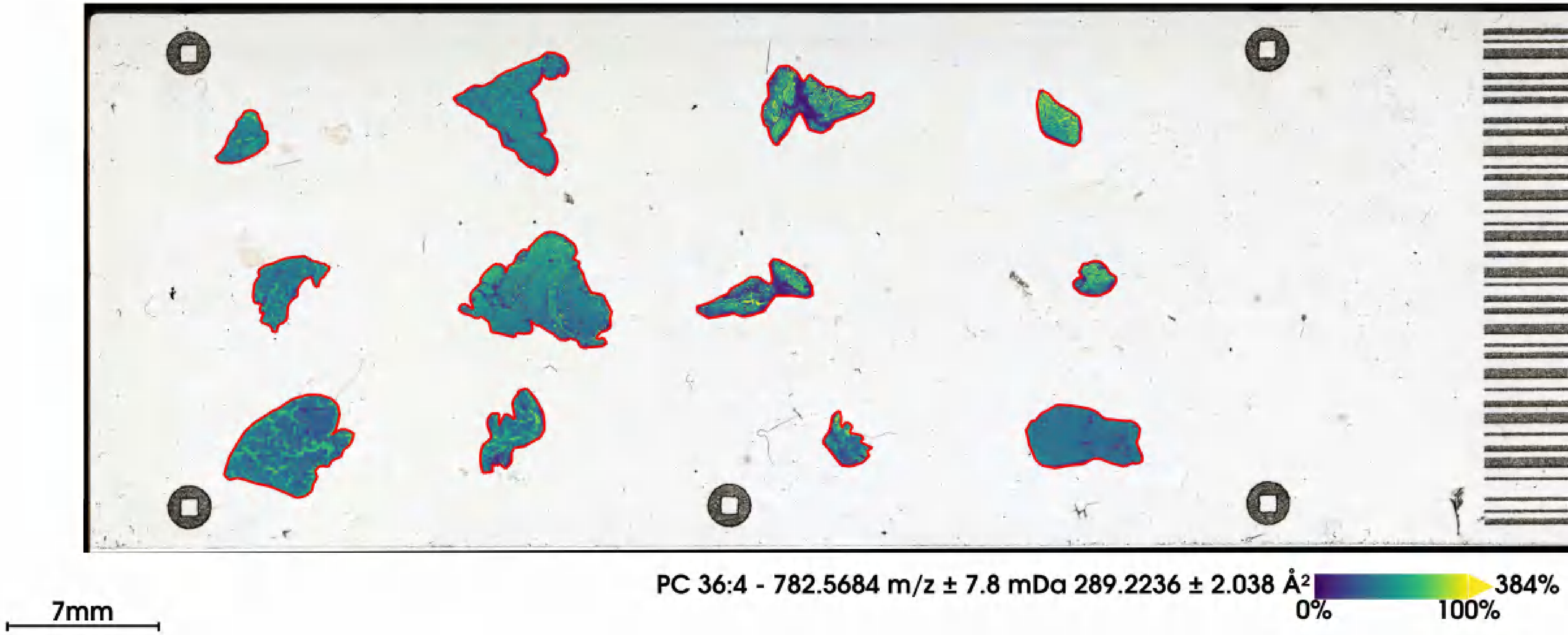
PC 36:4 - 782.5684 m/z ± 7.8 mDa 289.2236 ± 2.038 Å²
384%
0%
100%
7mm

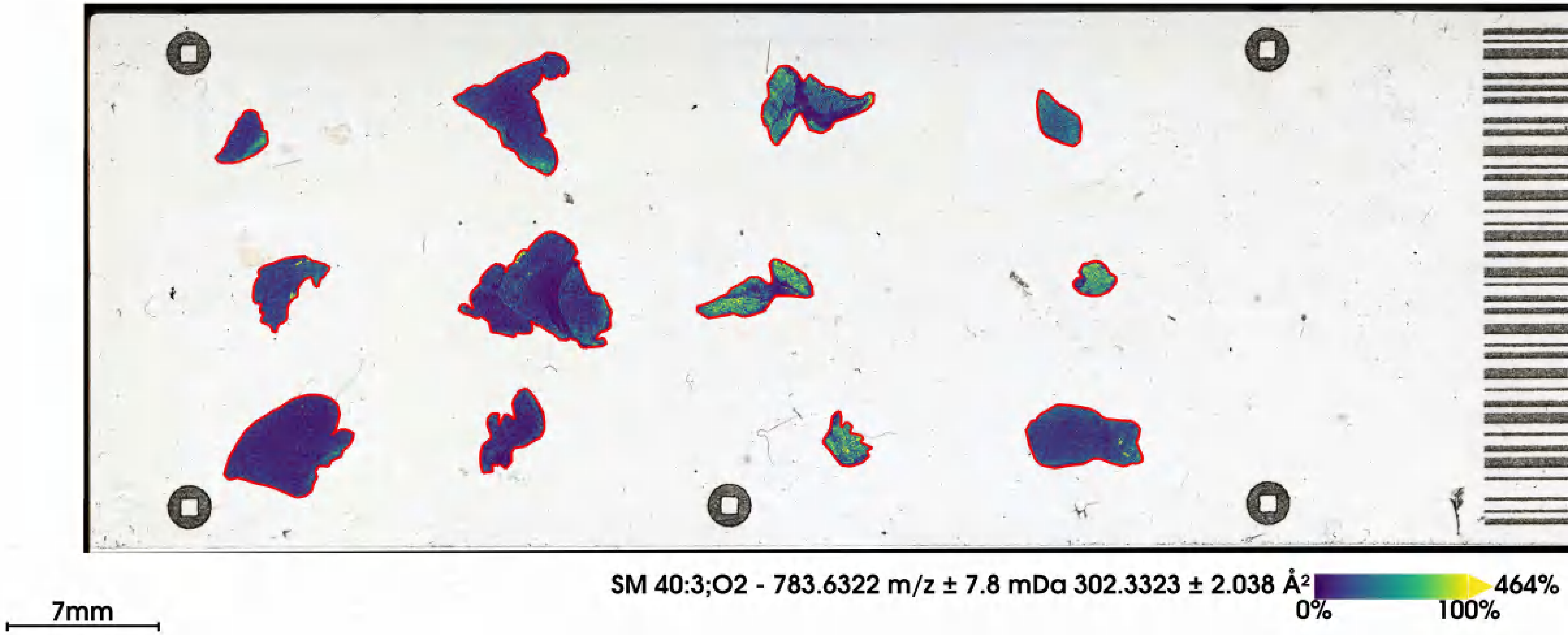

SM 40:3;O2 - 783.6322 m/z ± 7.8 mDa 302.3323 ± 2.038 Å²
464%
0%
100%
7mm

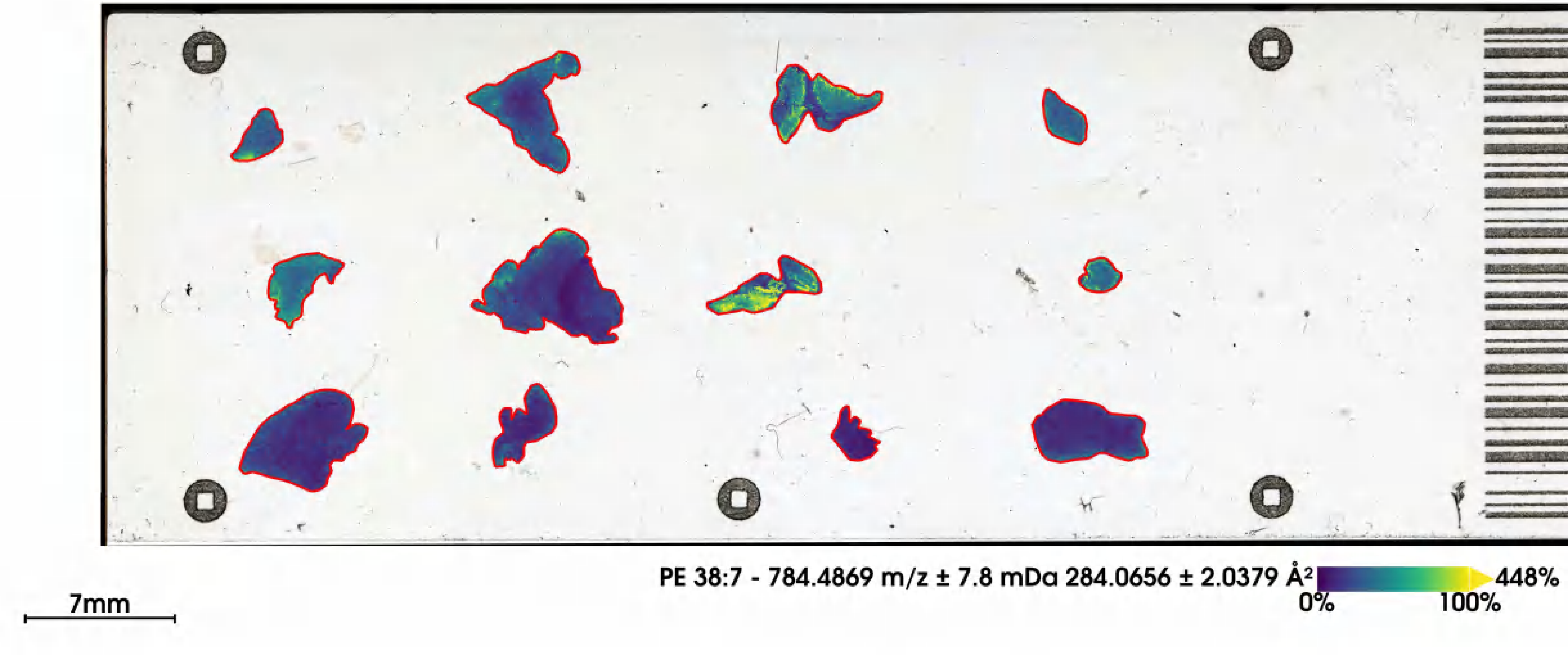
PE 38:7 - 784.4869 m/z ± 7.8 mDa 284.0656 ± 2.0379 Å²
0%
100%
448%
7mm

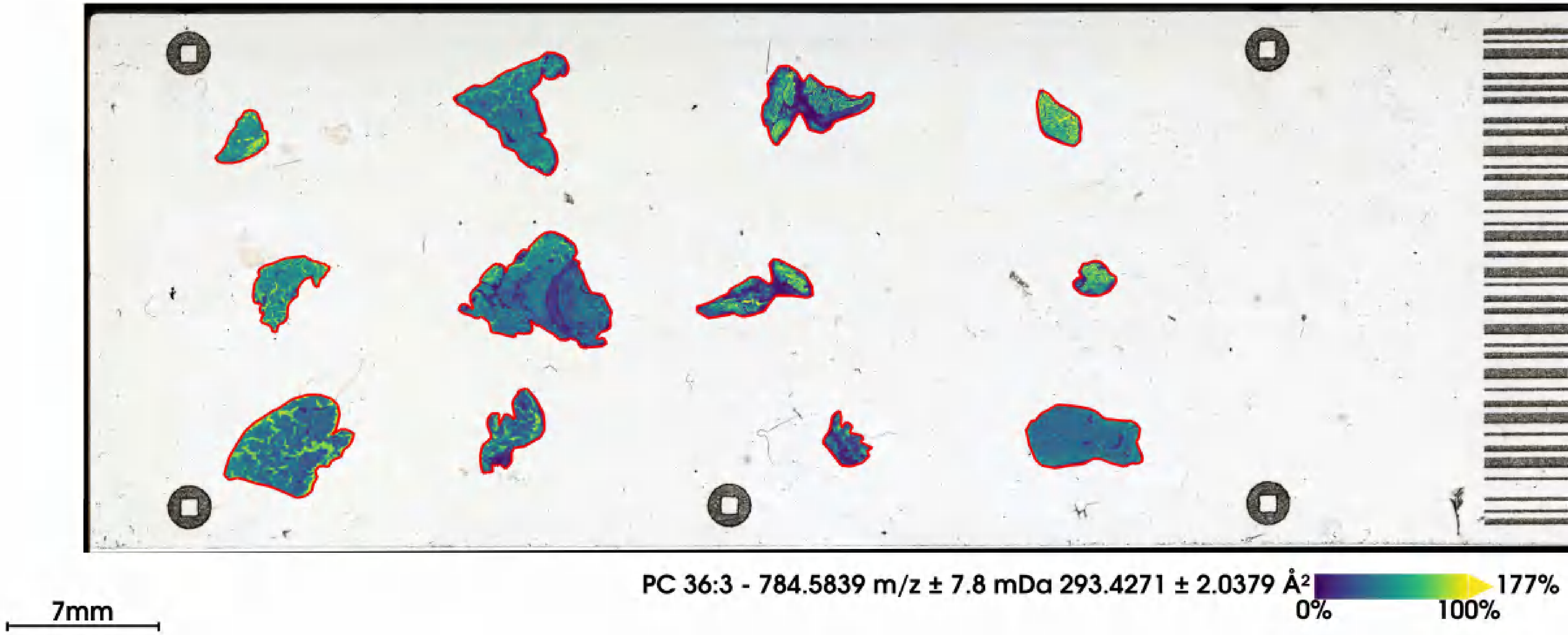
PC 36:3 - 784.5839 m/z ± 7.8 mDa 293.4271 ± 2.0379 Å²
0%
100%
177%
7mm

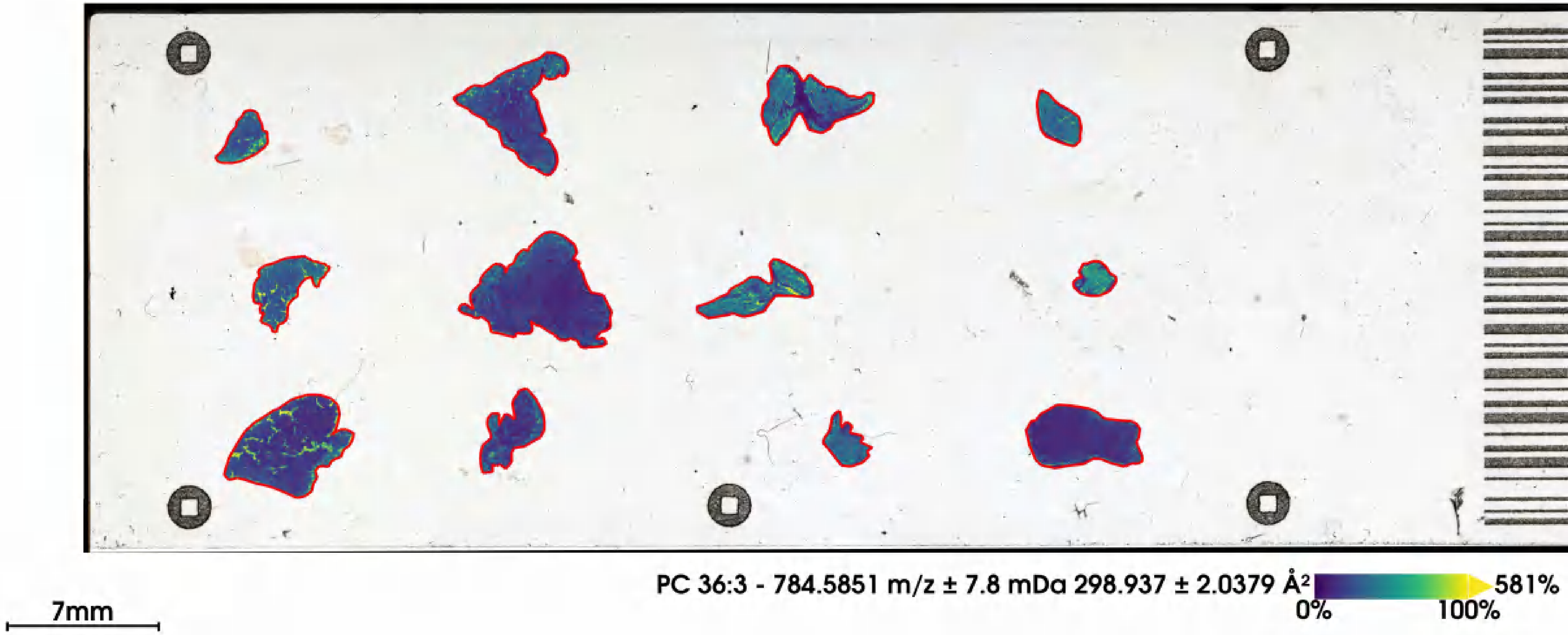

7mm
PC 36:3 - 784.5851 m/z ± 7.8 mDa 298.937 ± 2.0379 Å²
581%
0%
100%

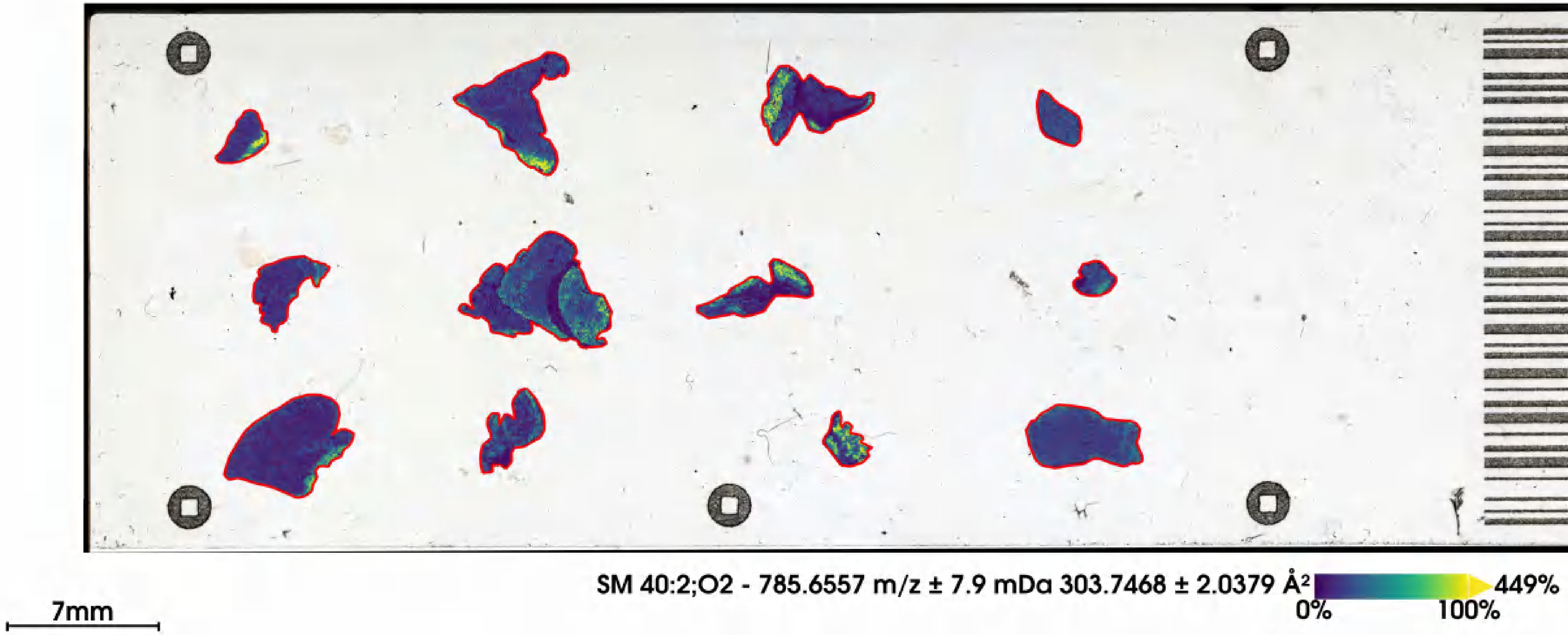

SM 40:2;O2 - 785.6557 m/z ± 7.9 mDa 303.7468 ± 2.0379 Å²
0%
100%
449%
7mm

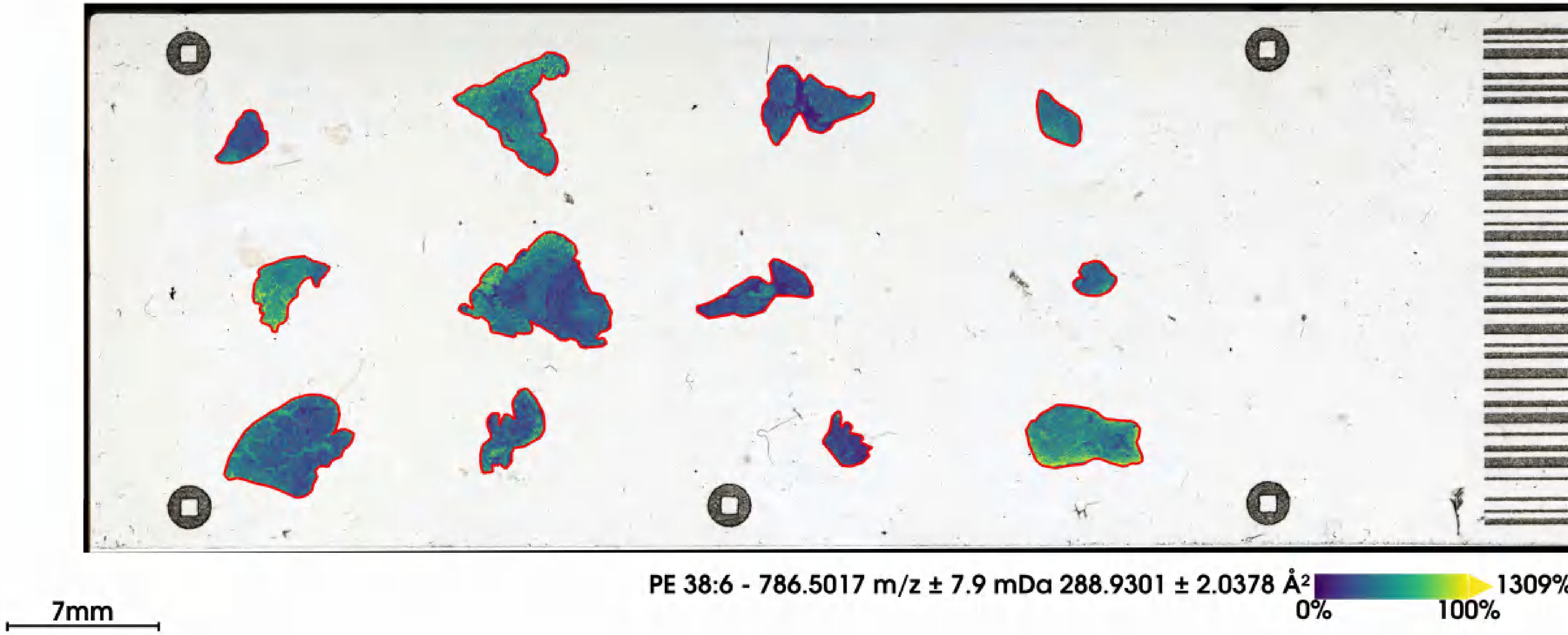
PE 38:6 - 786.5017 m/z ± 7.9 mDa 288.9301 ± 2.0378 Å²
0%
100%
1309%
7mm

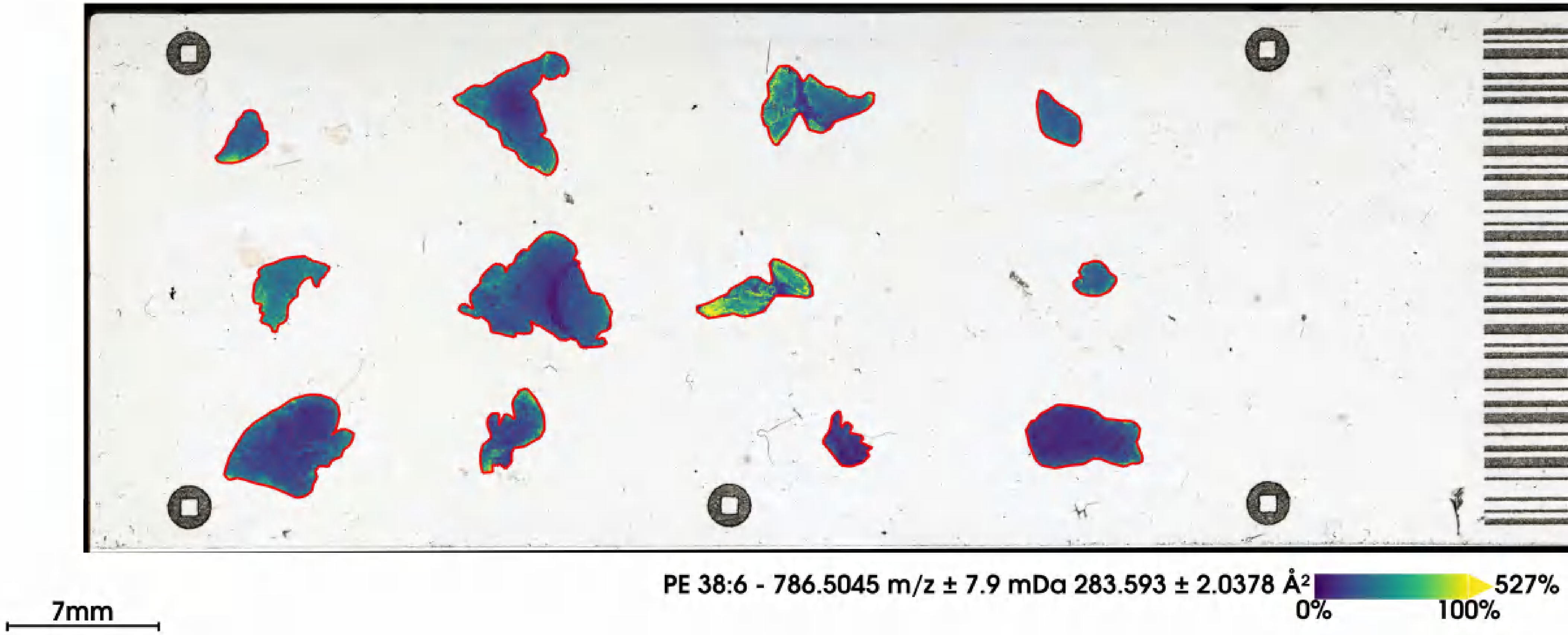
7mm
PE 38:6 - 786.5045 m/z ± 7.9 mDa 283.593 ± 2.0378 Å²
0%
100%
527%

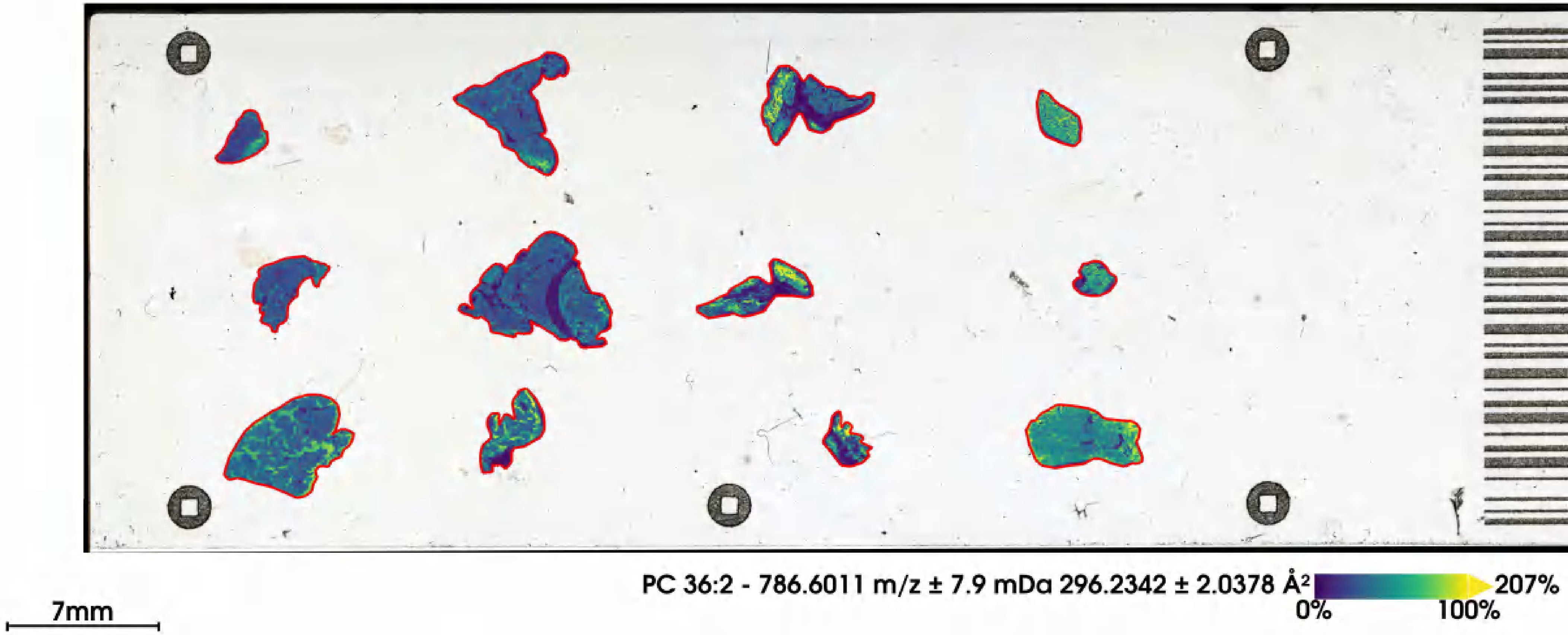
PC 36:2 - 786.6011 m/z ± 7.9 mDa 296.2342 ± 2.0378 Å²
0%
100%
207%
7mm

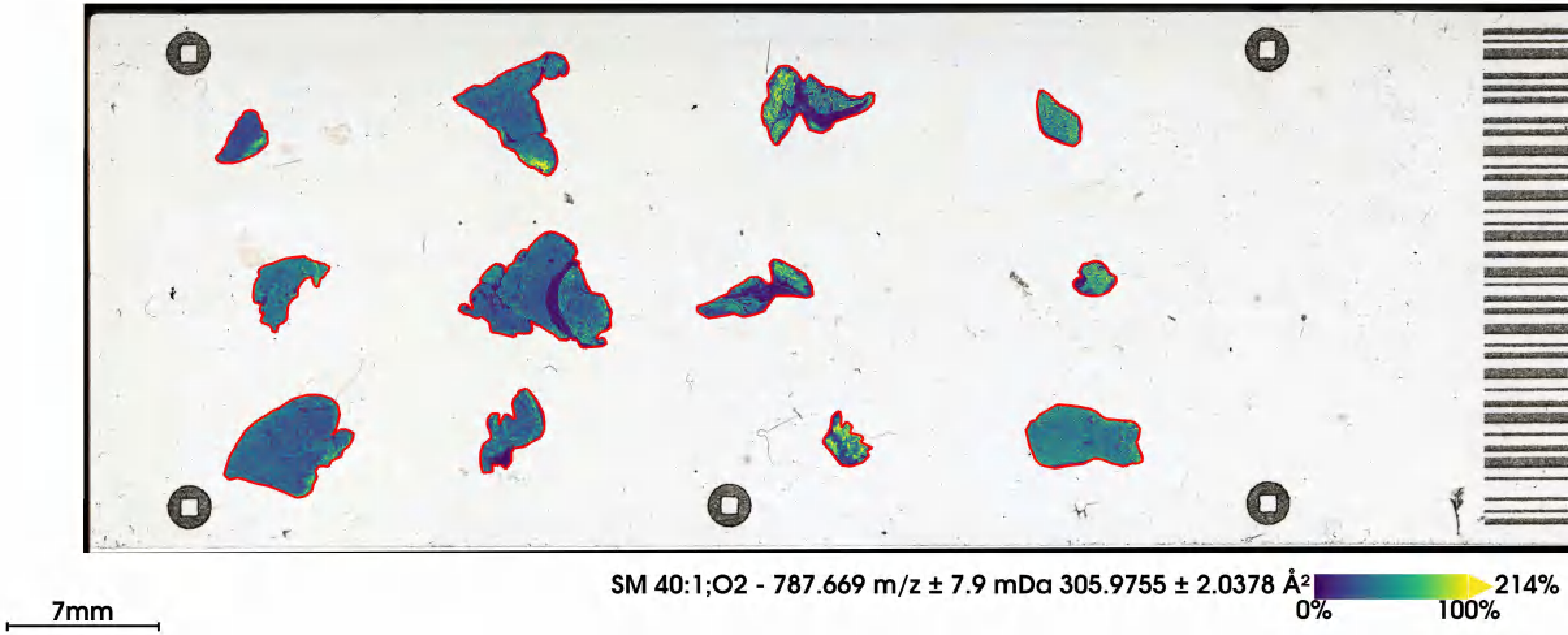

SM 40:1;O2 - 787.669 m/z ± 7.9 mDa 305.9755 ± 2.0378 Å²
0%
100%
214%
7mm

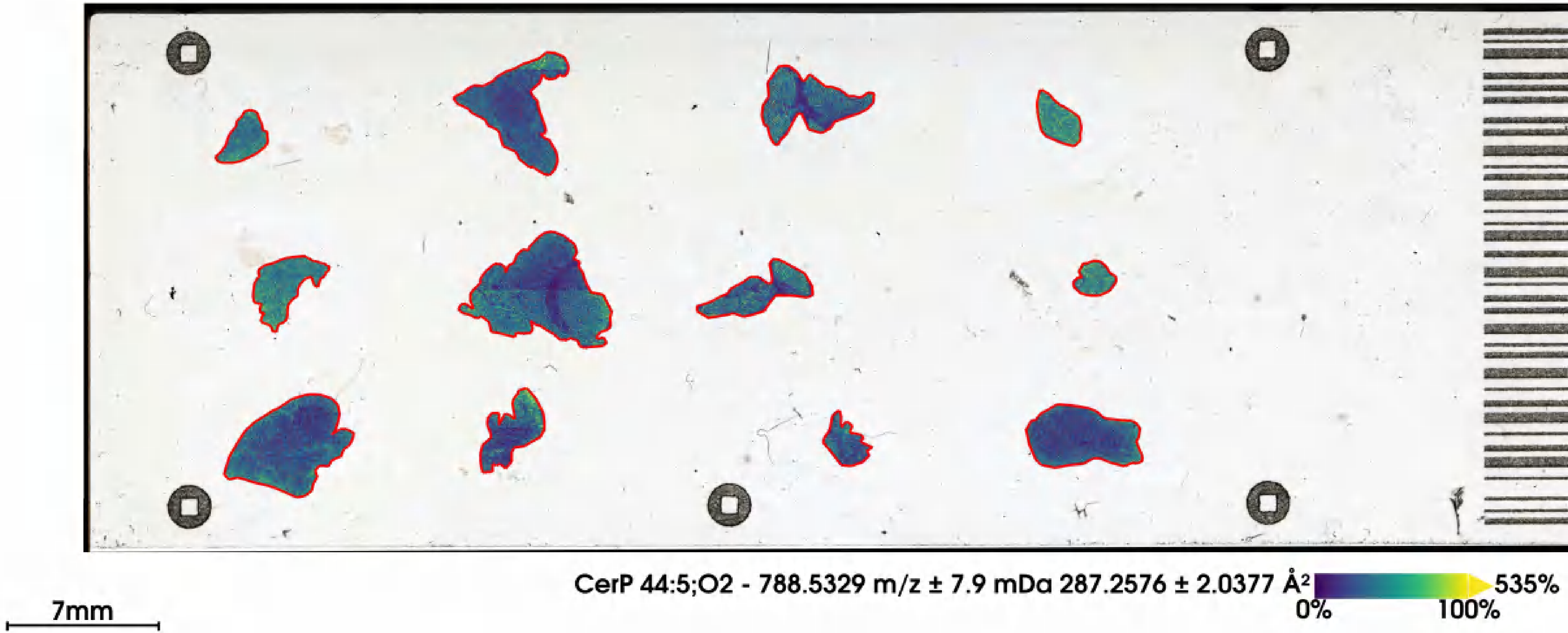
CerP 44:5;O2 - 788.5329 m/z ± 7.9 mDa 287.2576 ± 2.0377 Å²
0%
100%
535%
7mm

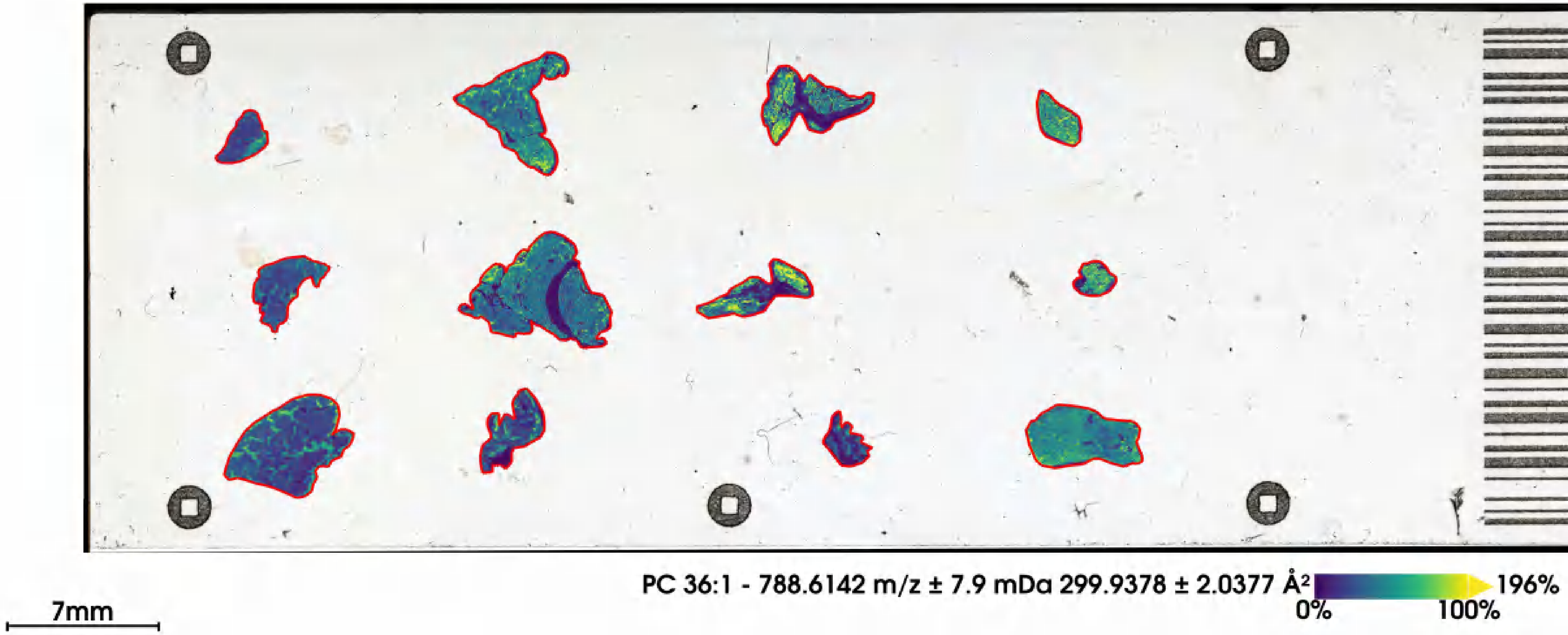
PC 36:1 - 788.6142 m/z ± 7.9 mDa 299.9378 ± 2.0377 Å²
0%
100%
196%
7mm

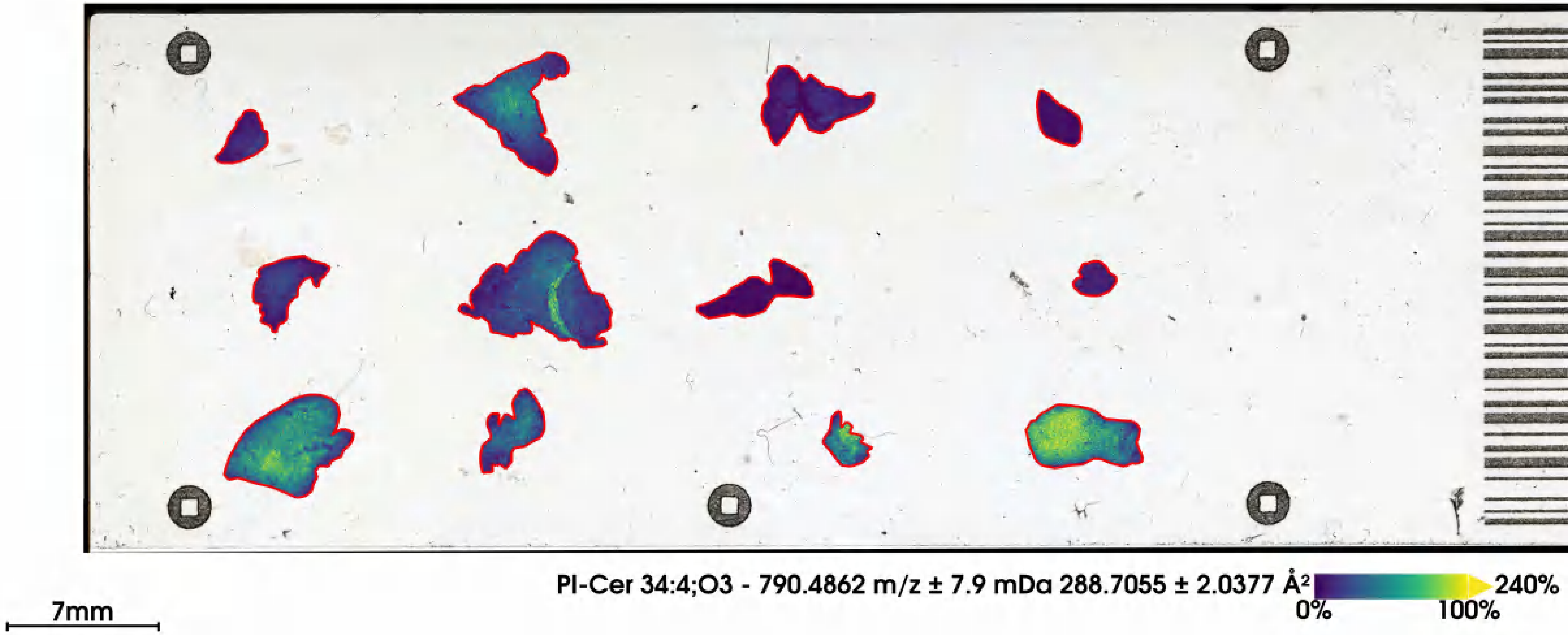

PI-Cer 34:4;O3 - 790.4862 m/z ± 7.9 mDa 288.7055 ± 2.0377 Å²
0%
100%
240%
7mm

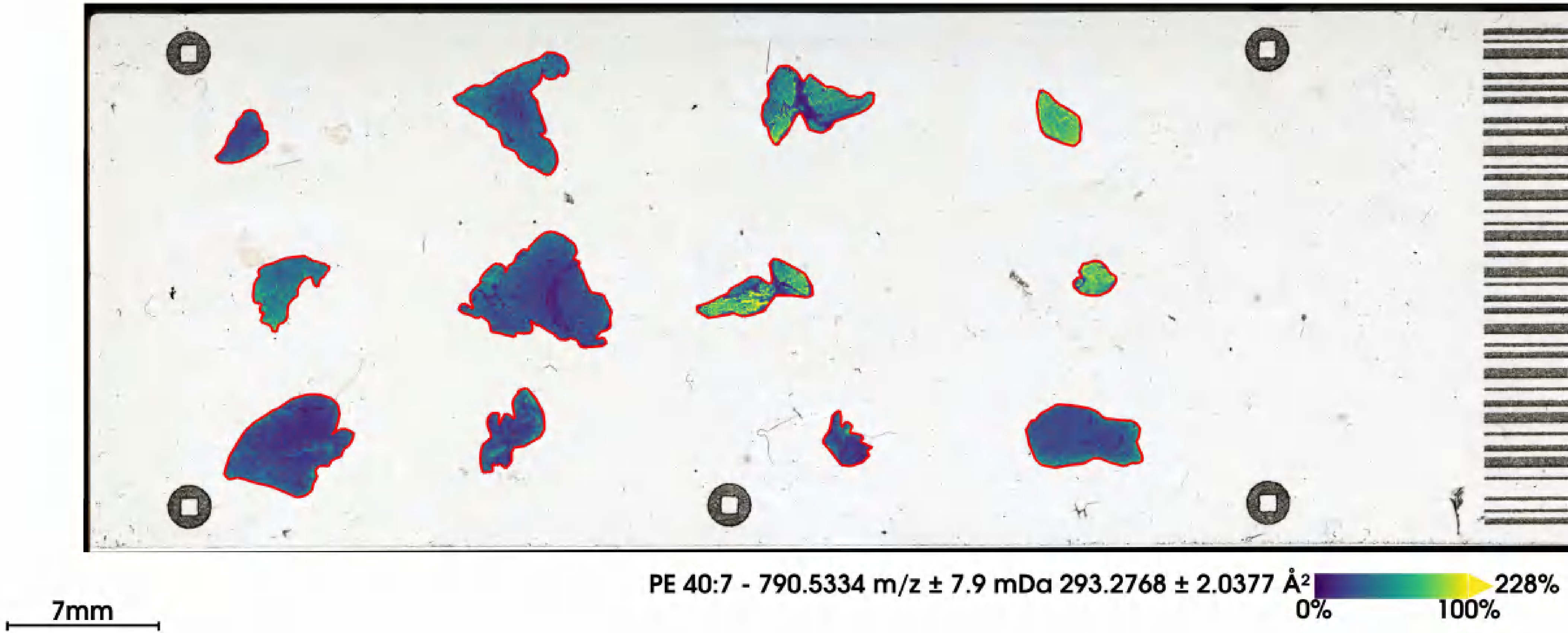

PE 40:7 - 790.5334 m/z ± 7.9 mDa 293.2768 ± 2.0377 Å²
0%
100%
228%
7mm

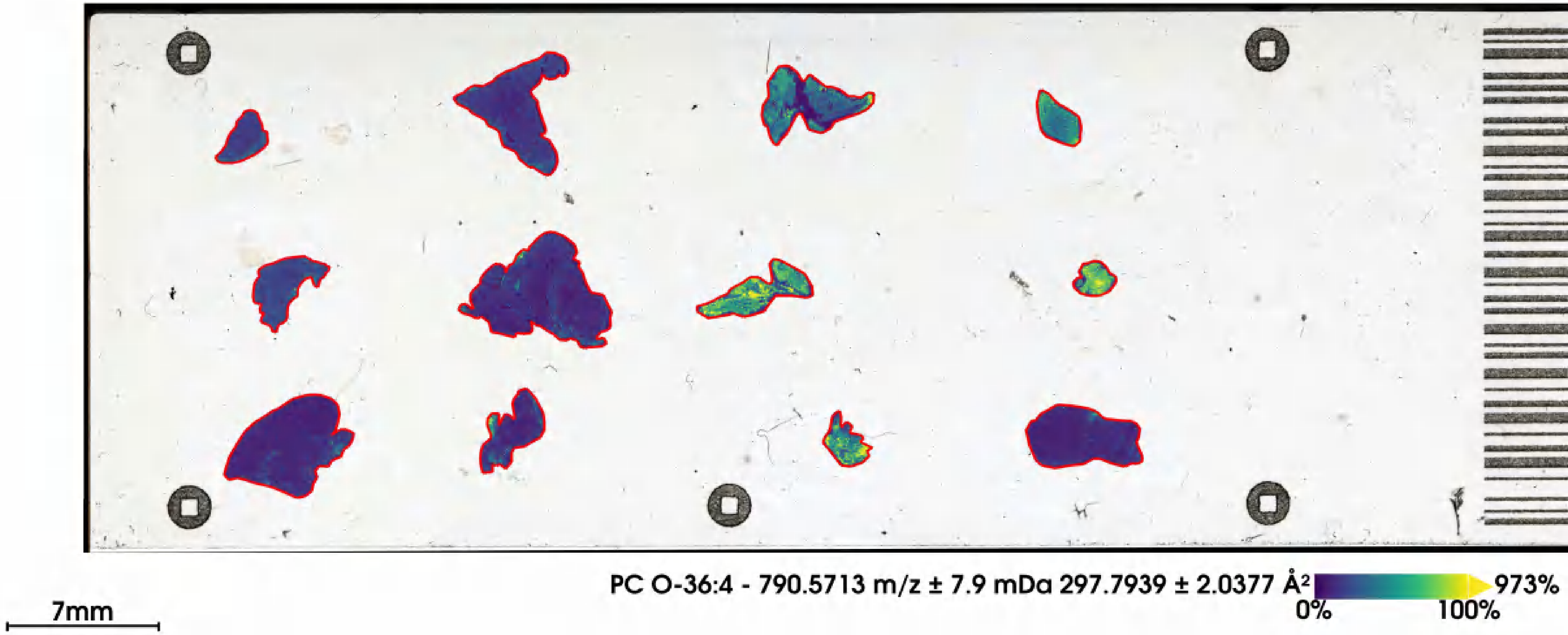
PC O-36:4 - 790.5713 m/z ± 7.9 mDa 297.7939 ± 2.0377 Å²
973%
0%
100%
7mm

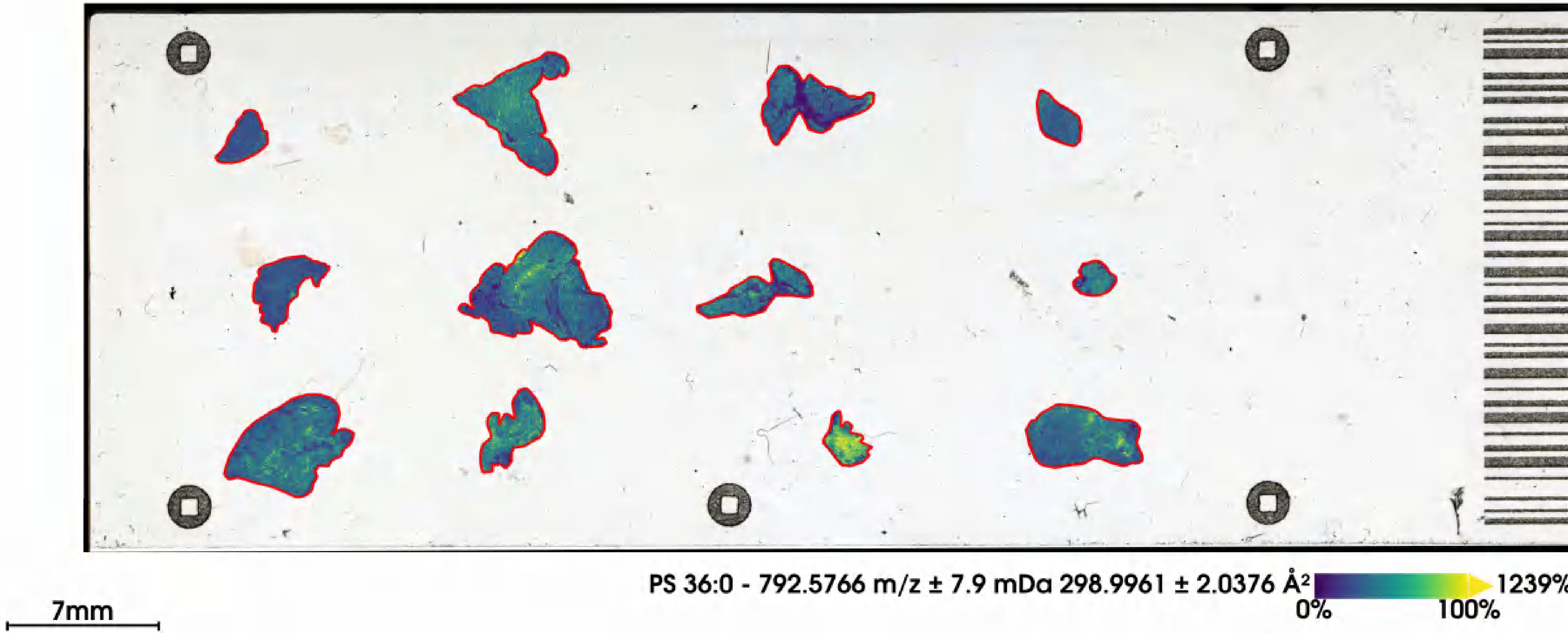
PS 36:0 - 792.5766 m/z ± 7.9 mDa 298.9961 ± 2.0376 Å²
0%
100%
1239%
7mm

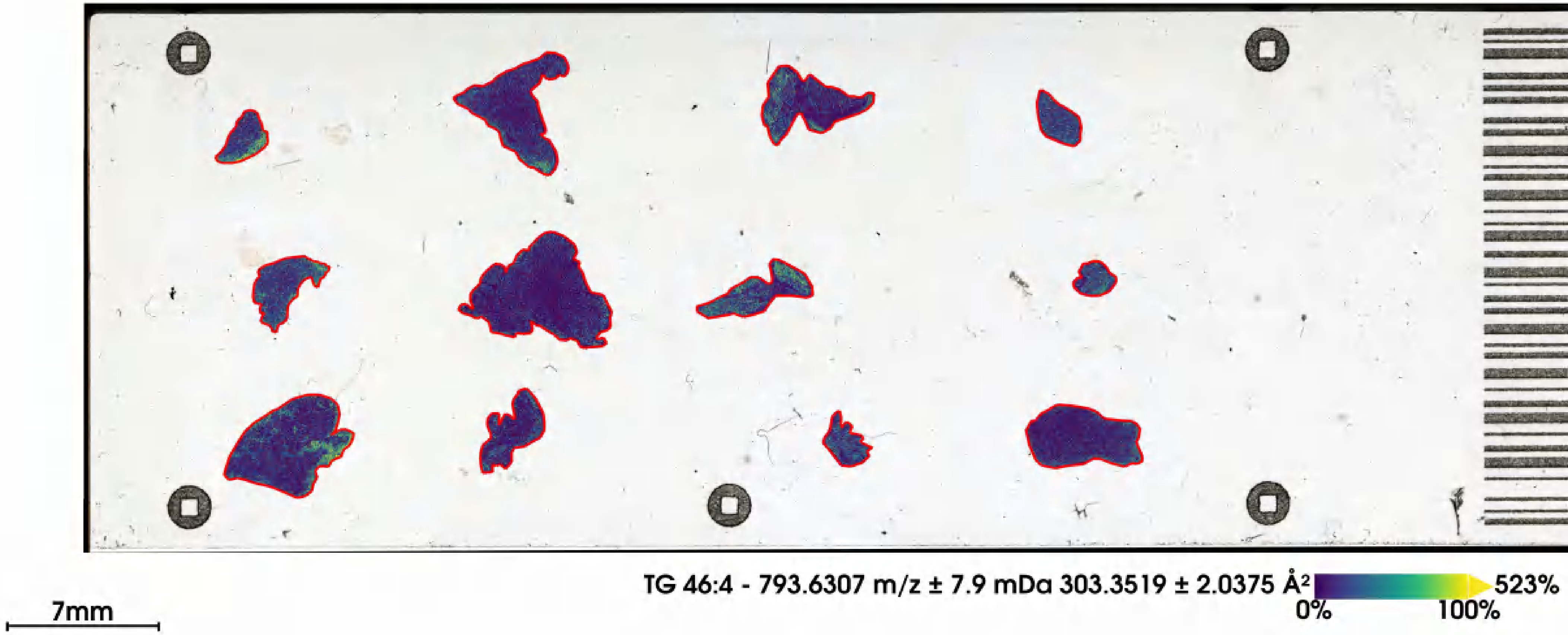
TG 46:4 - 793.6307 m/z ± 7.9 mDa 303.3519 ± 2.0375 Å²
0%
100%
523%
7mm

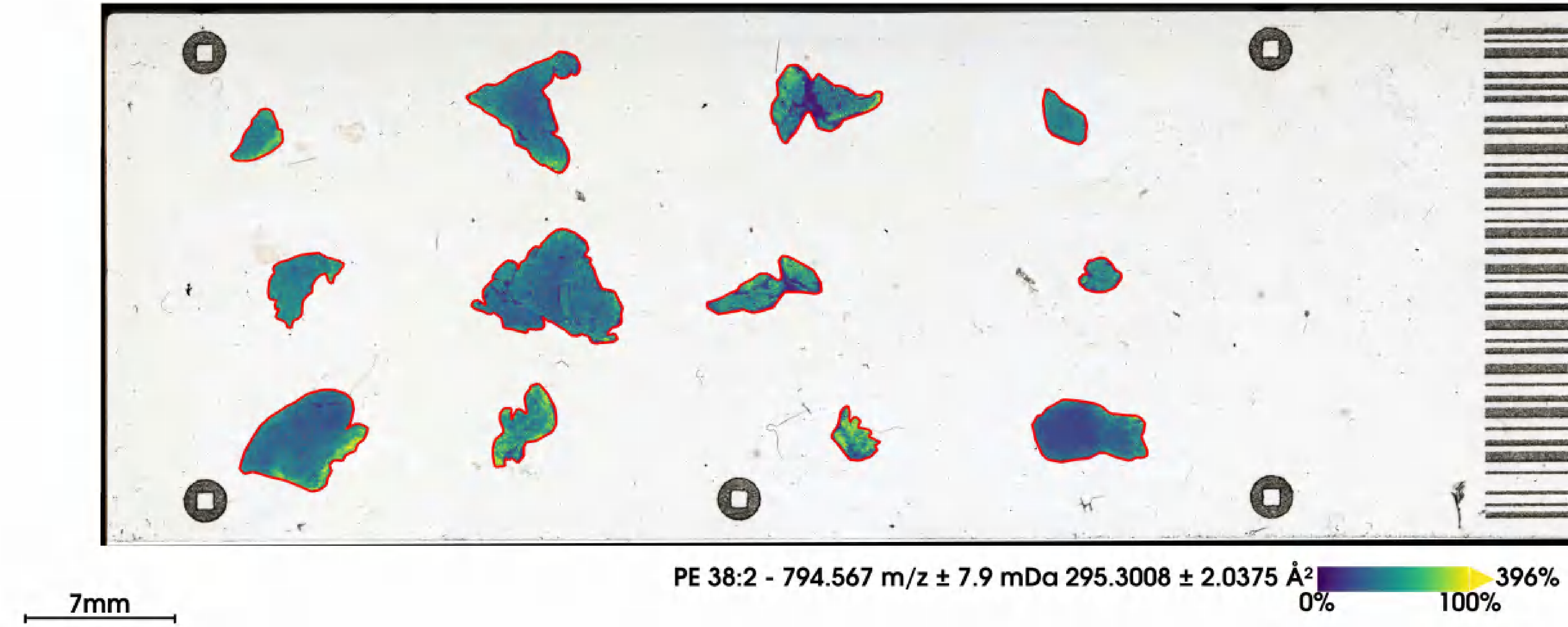

PE 38:2 - 794.567 m/z ± 7.9 mDa 295.3008 ± 2.0375 Å²
0%
100%
396%
7mm

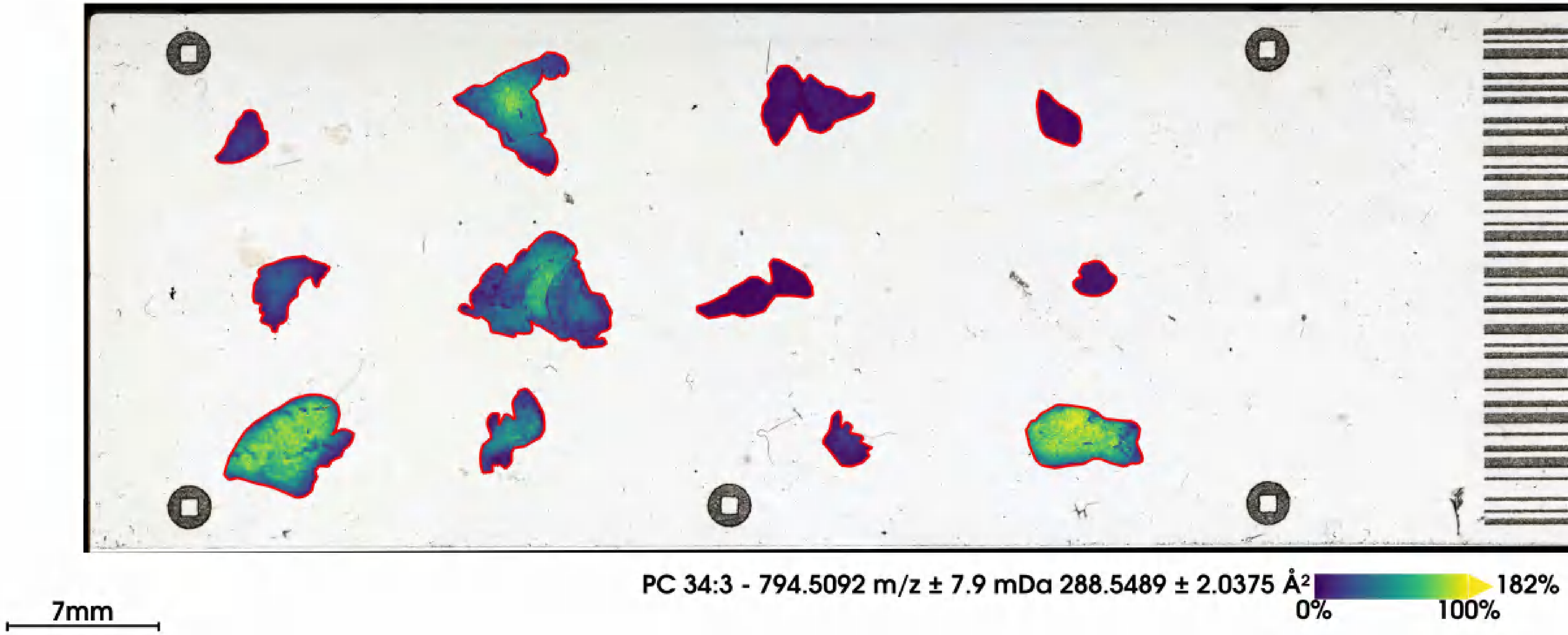
PC 34:3 - 794.5092 m/z ± 7.9 mDa 288.5489 ± 2.0375 Å²
0%
100%
182%
7mm

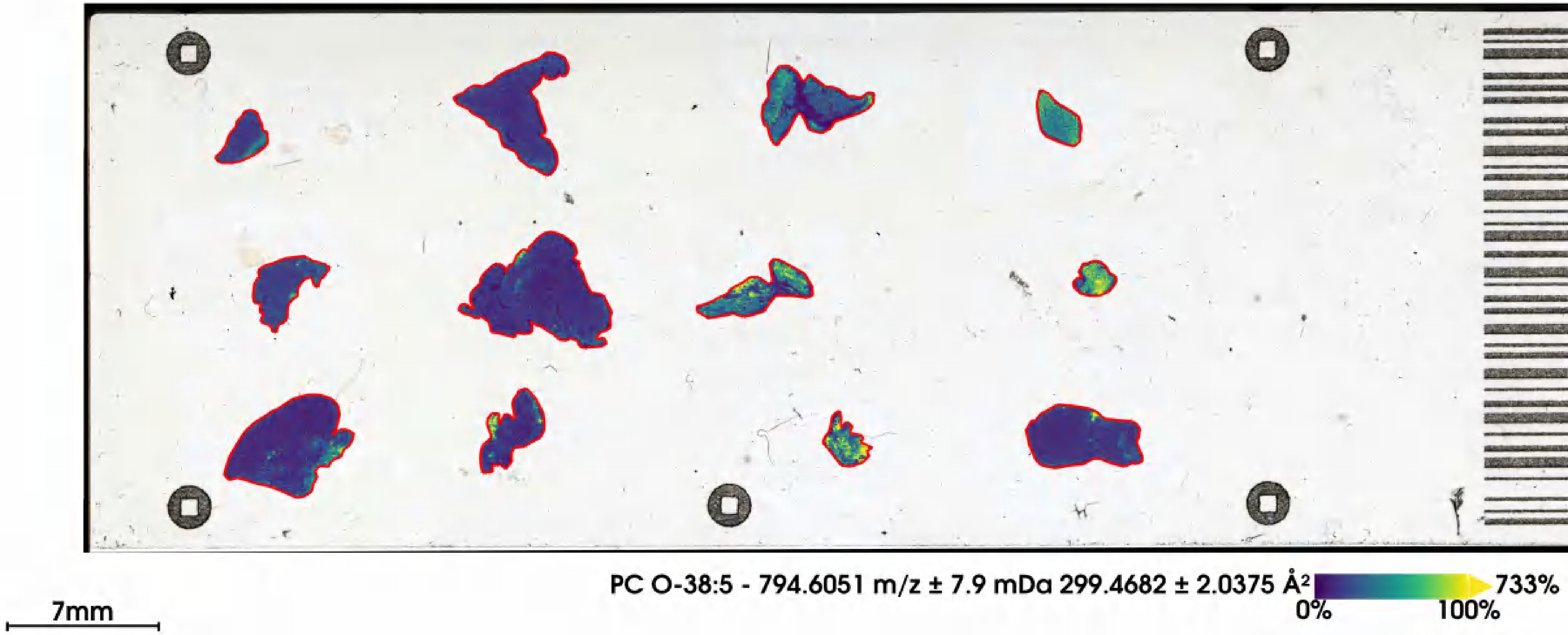
PC O-38:5 - 794.6051 m/z ± 7.9 mDa 299.4682 ± 2.0375 Å²
733%
0%
100%
7mm

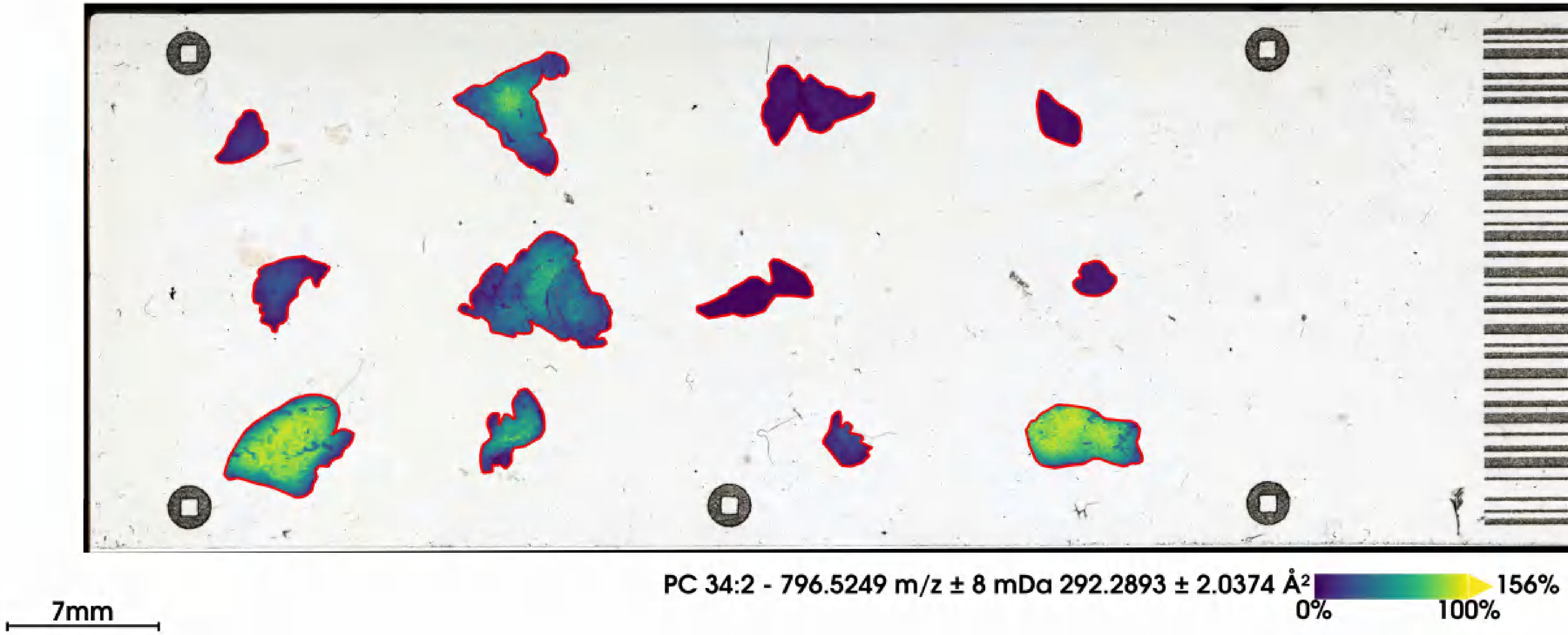

PC 34:2 - 796.5249 m/z ± 8 mDa 292.2893 ± 2.0374 Å²
156%
0%
100%
7mm

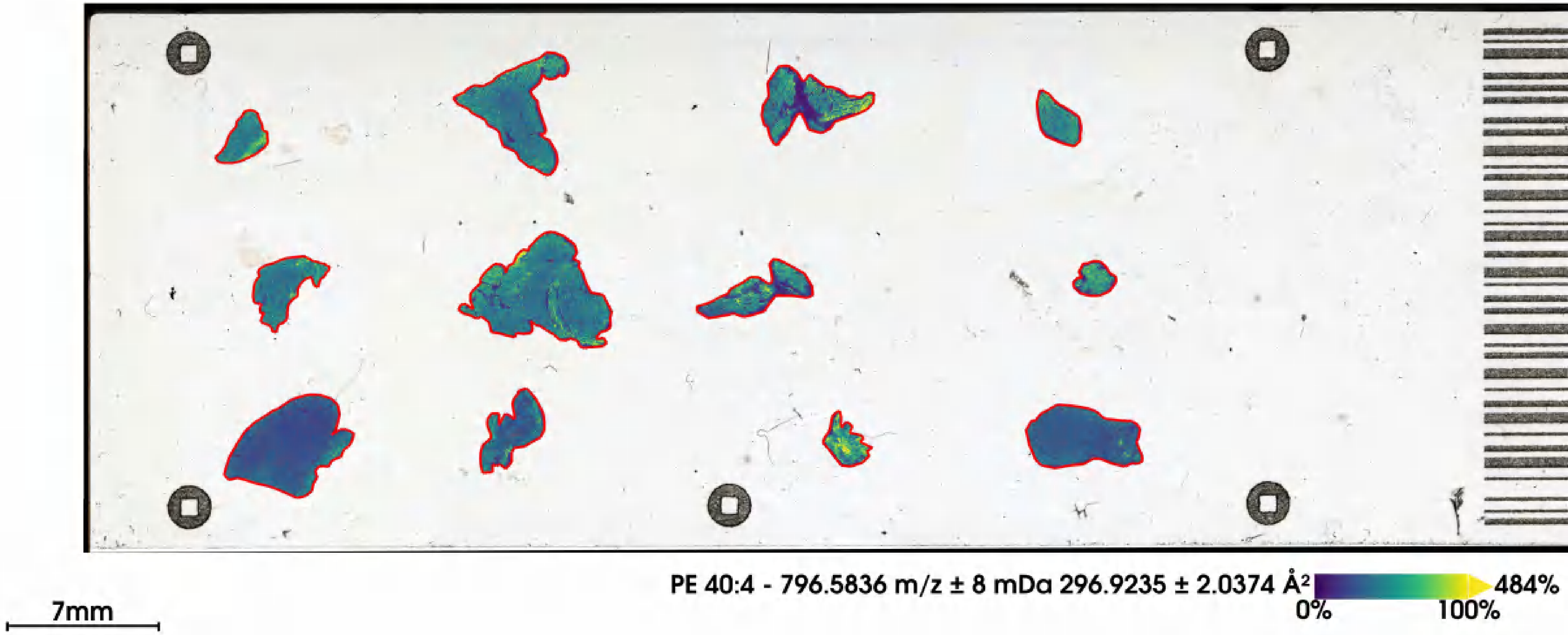
PE 40:4 - 796.5836 m/z ± 8 mDa 296.9235 ± 2.0374 Å²
0%
100%
484%
7mm

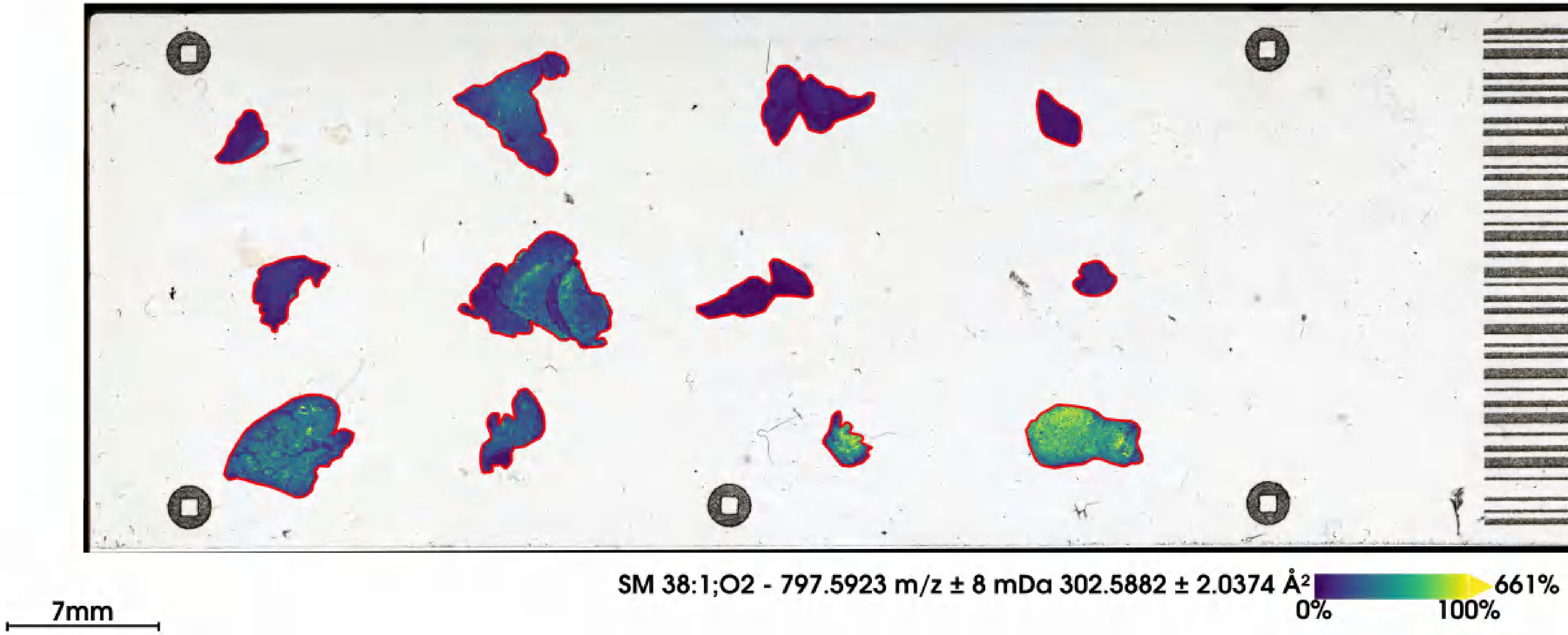
7mm
SM 38:1;O2 - 797.5923 m/z ± 8 mDa 302.5882 ± 2.0374 Å²
0%
100%
661%

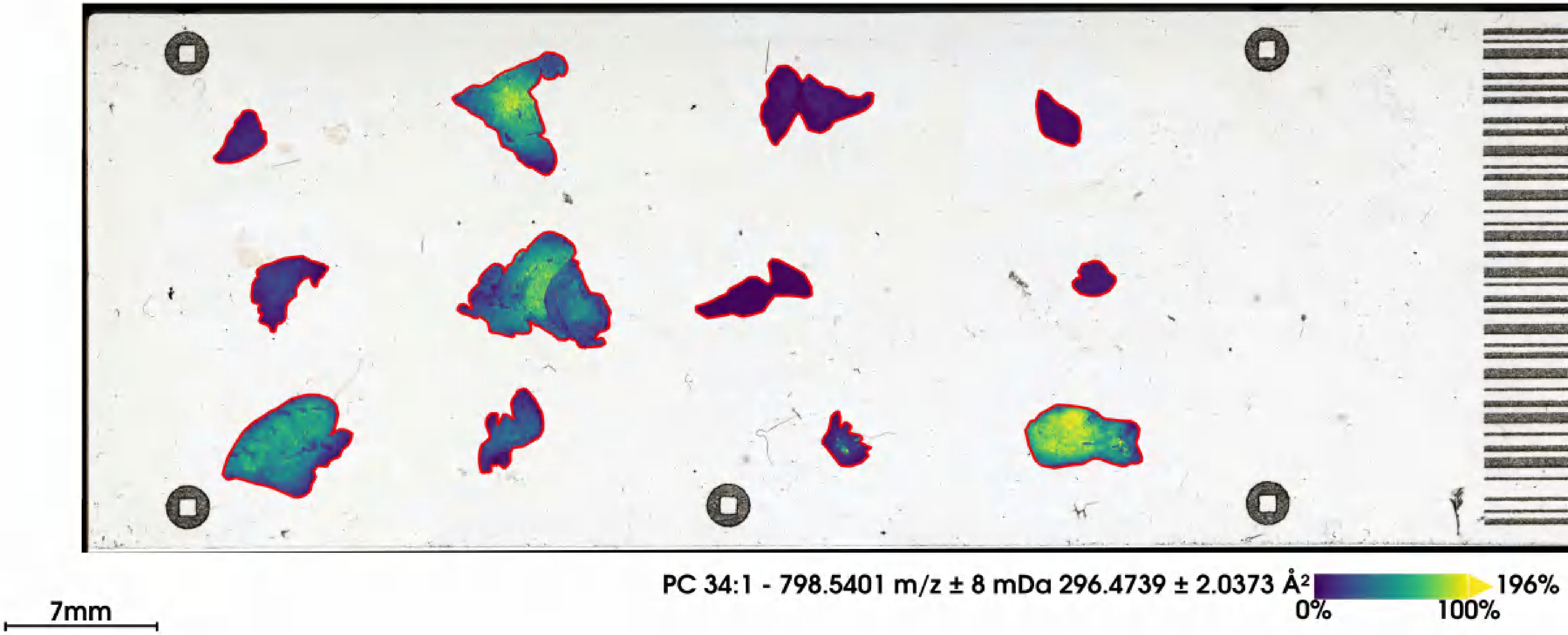
PC 34:1 - 798.5401 m/z ± 8 mDa 296.4739 ± 2.0373 Å²
0%
100%
196%
7mm

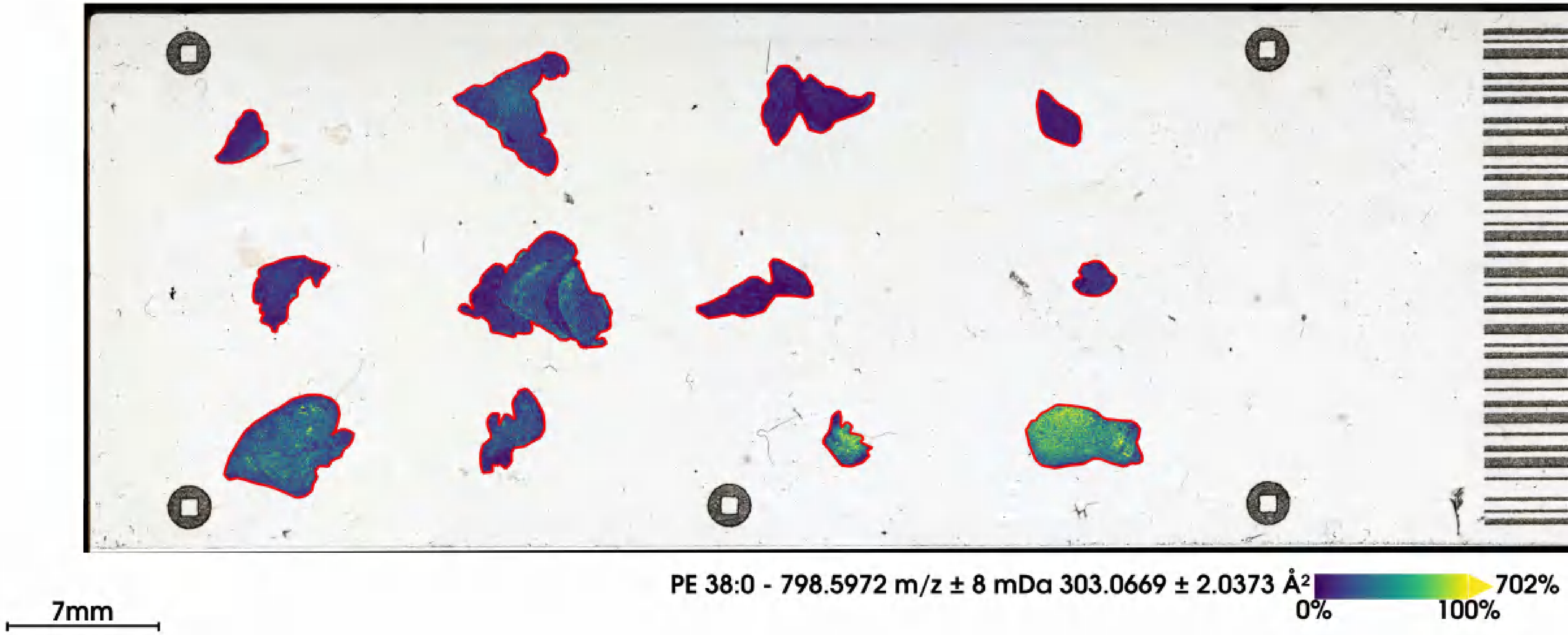
PE 38:0 - 798.5972 m/z ± 8 mDa 303.0669 ± 2.0373 Å²
702%
0%
100%
7mm

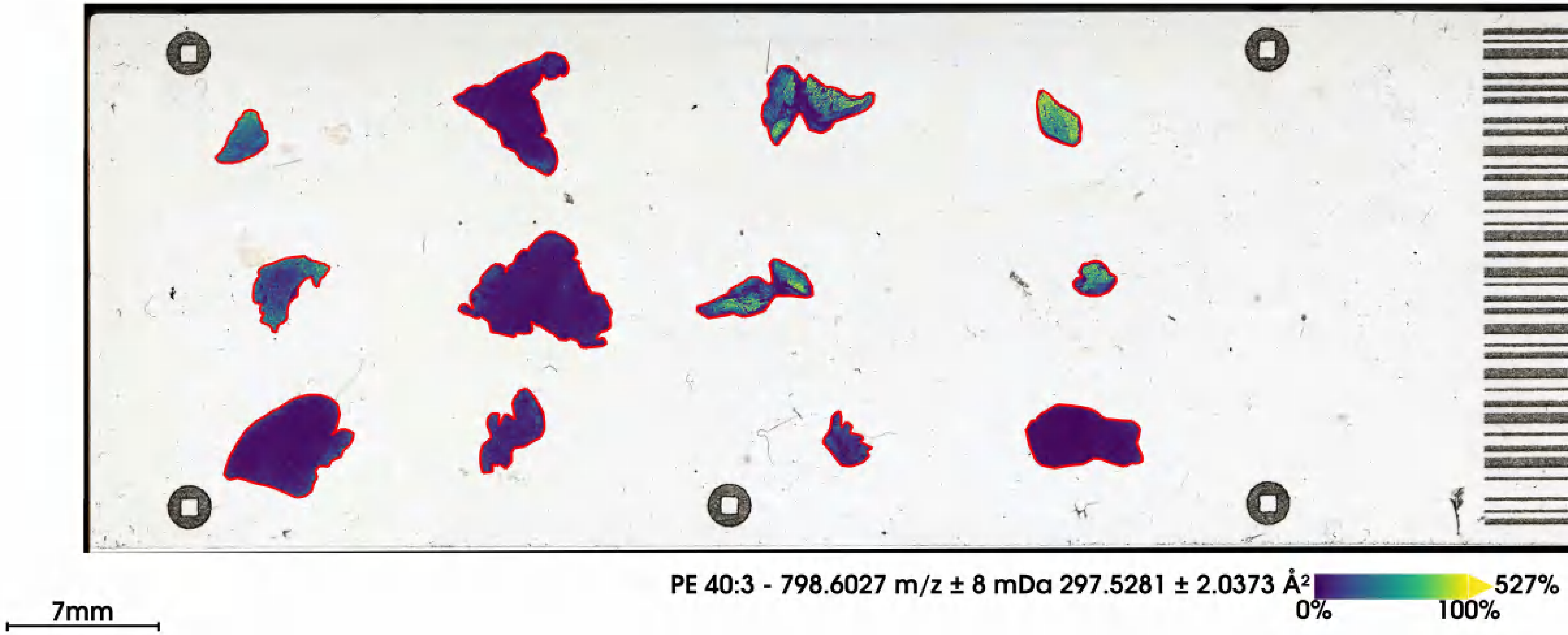
PE 40:3 - 798.6027 m/z ± 8 mDa 297.5281 ± 2.0373 Å²
527%
0%
100%
7mm

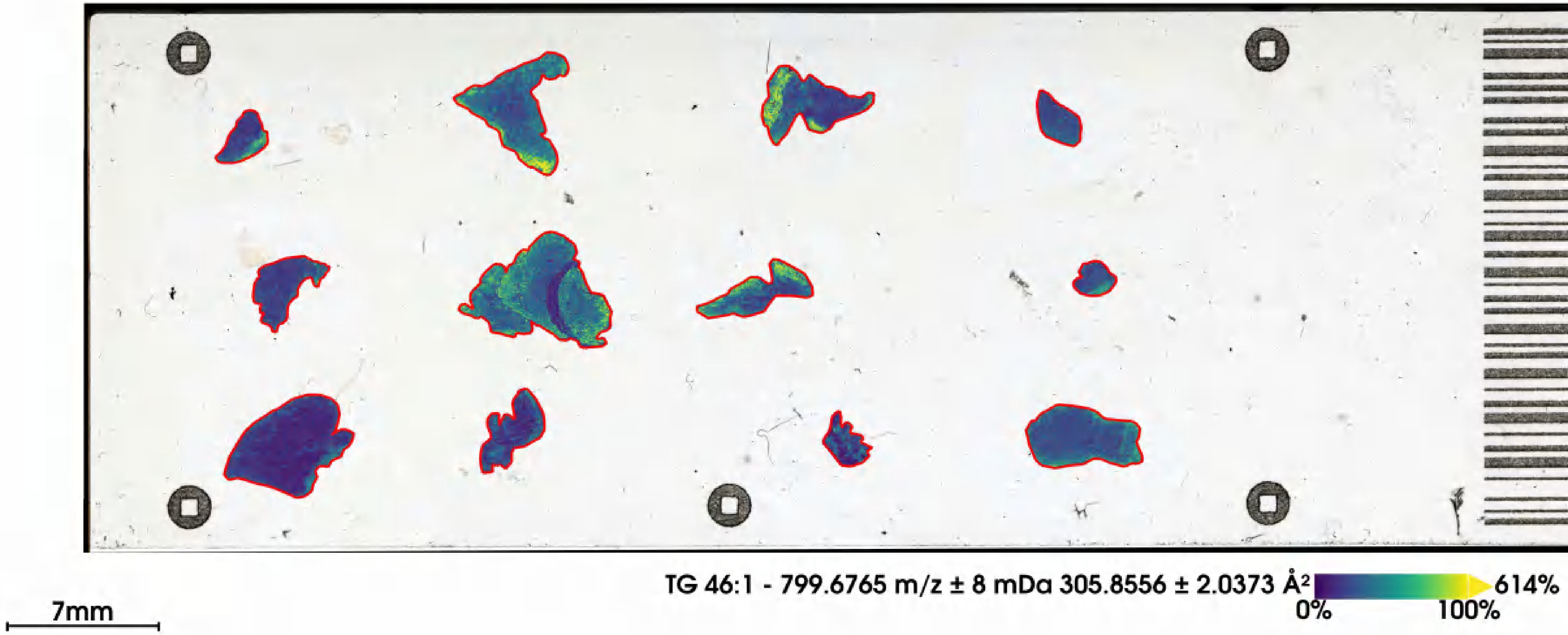
7mm
TG 46:1 - 799.6765 m/z ± 8 mDa 305.8556 ± 2.0373 Å²
0%
100%
614%

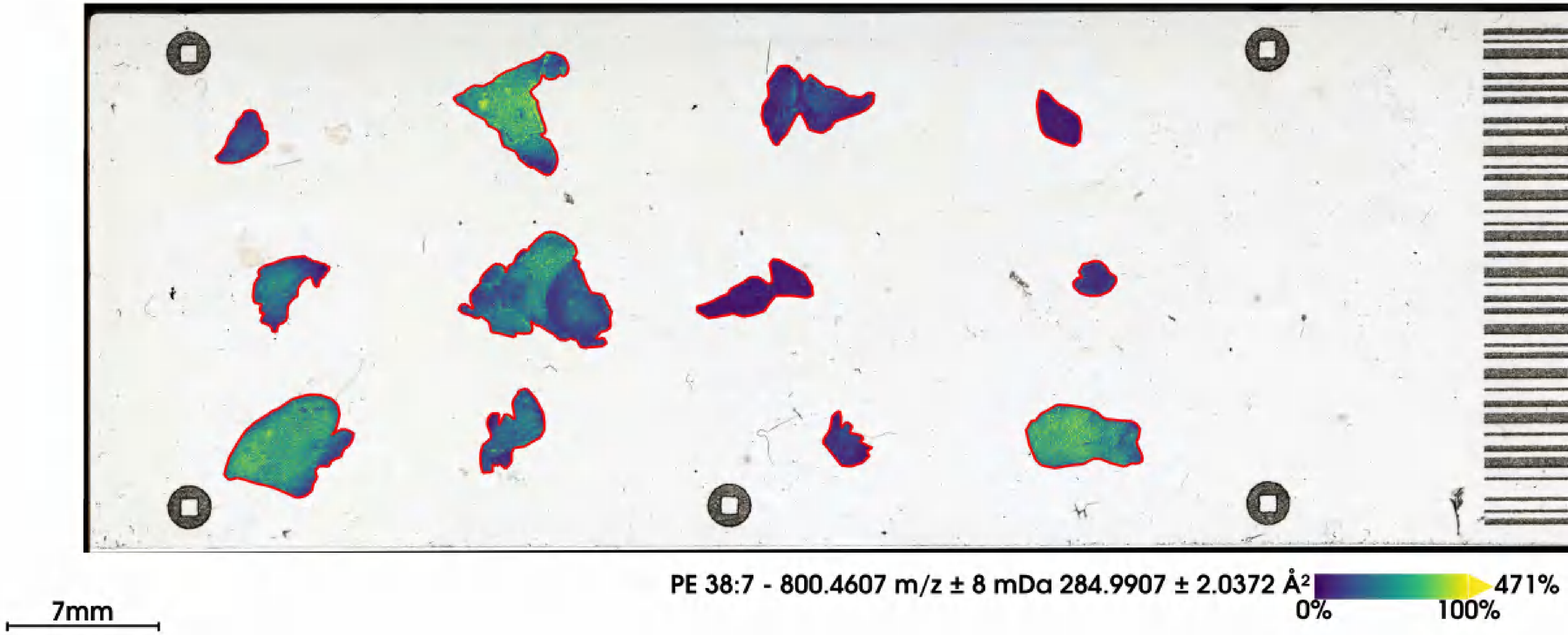
PE 38:7 - 800.4607 m/z ± 8 mDa 284.9907 ± 2.0372 Å²
471%
0%
100%
7mm

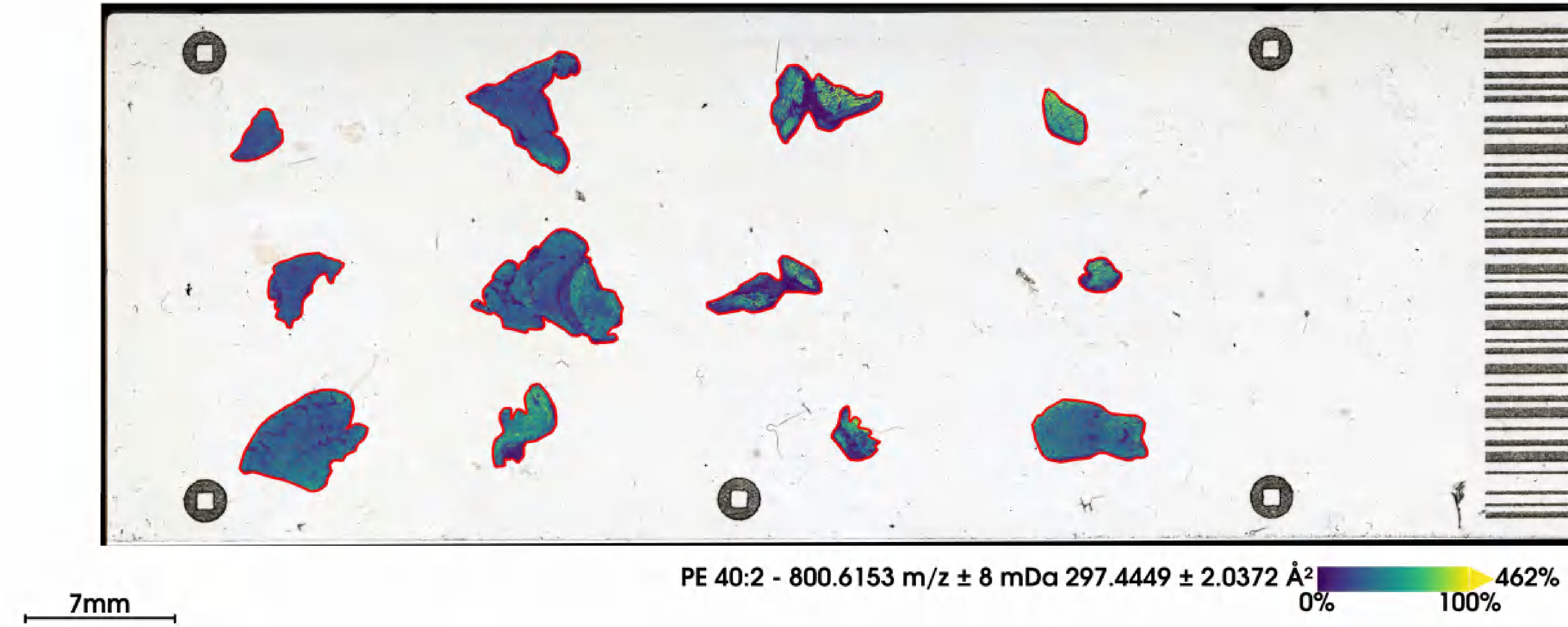

PE 40:2 - 800.6153 m/z ± 8 mDa 297.4449 ± 2.0372 Å²
0%
100%
462%
7mm

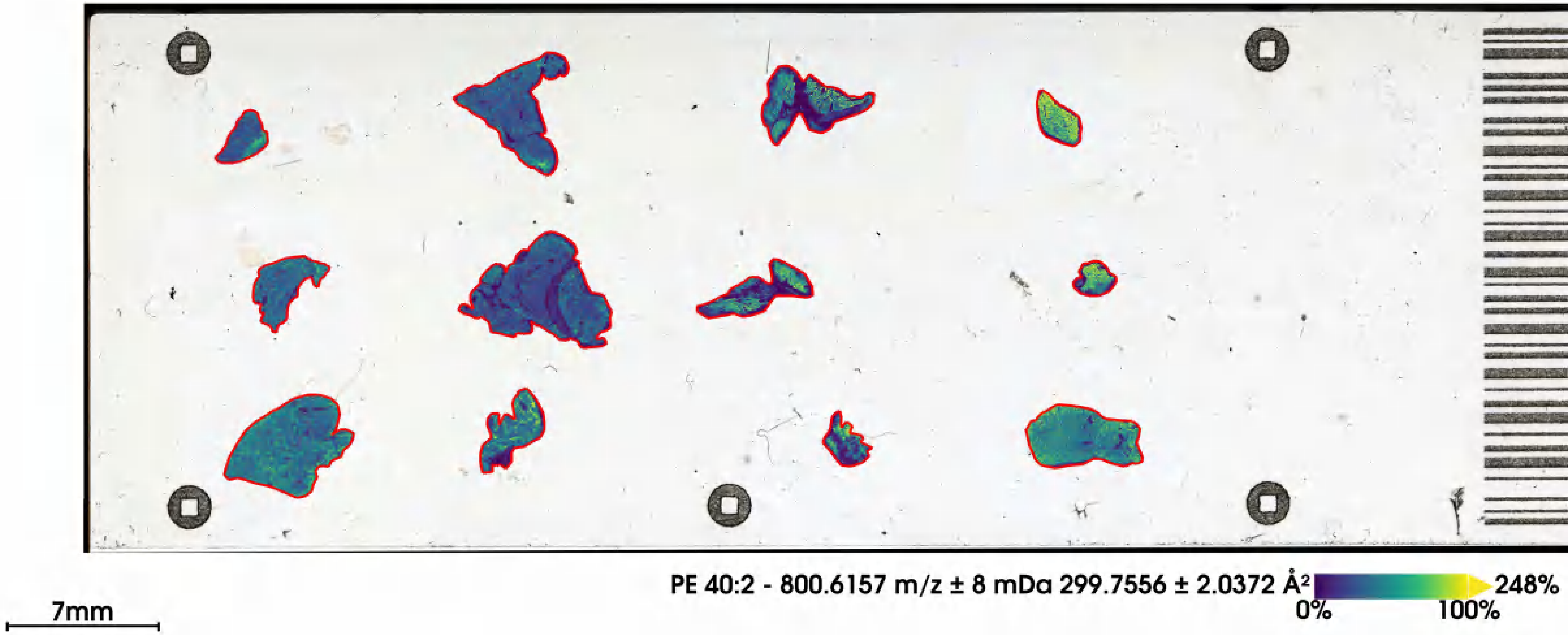

PE 40:2 - 800.6157 m/z ± 8 mDa 299.7556 ± 2.0372 Å²
248%
0%
100%
7mm

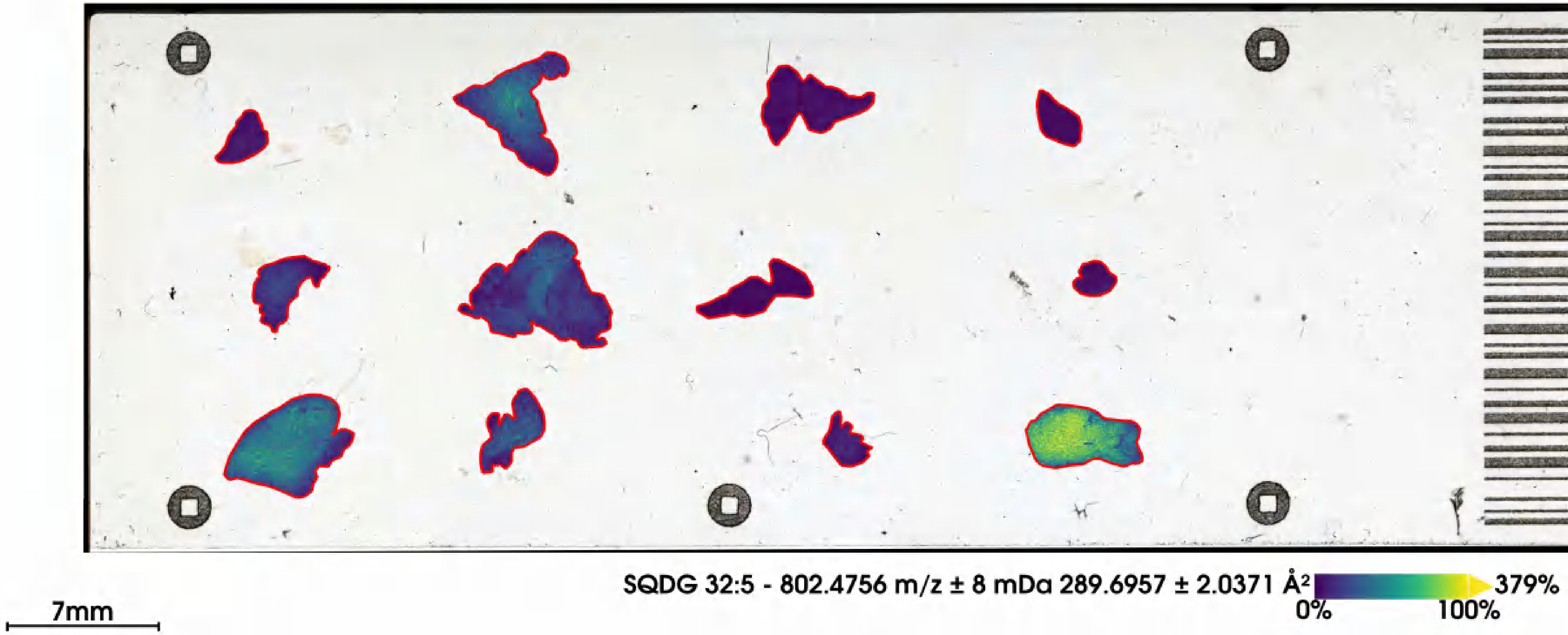
SQDG 32:5 - 802.4756 m/z ± 8 mDa 289.6957 ± 2.0371 Å²
0%
100%
379%
7mm

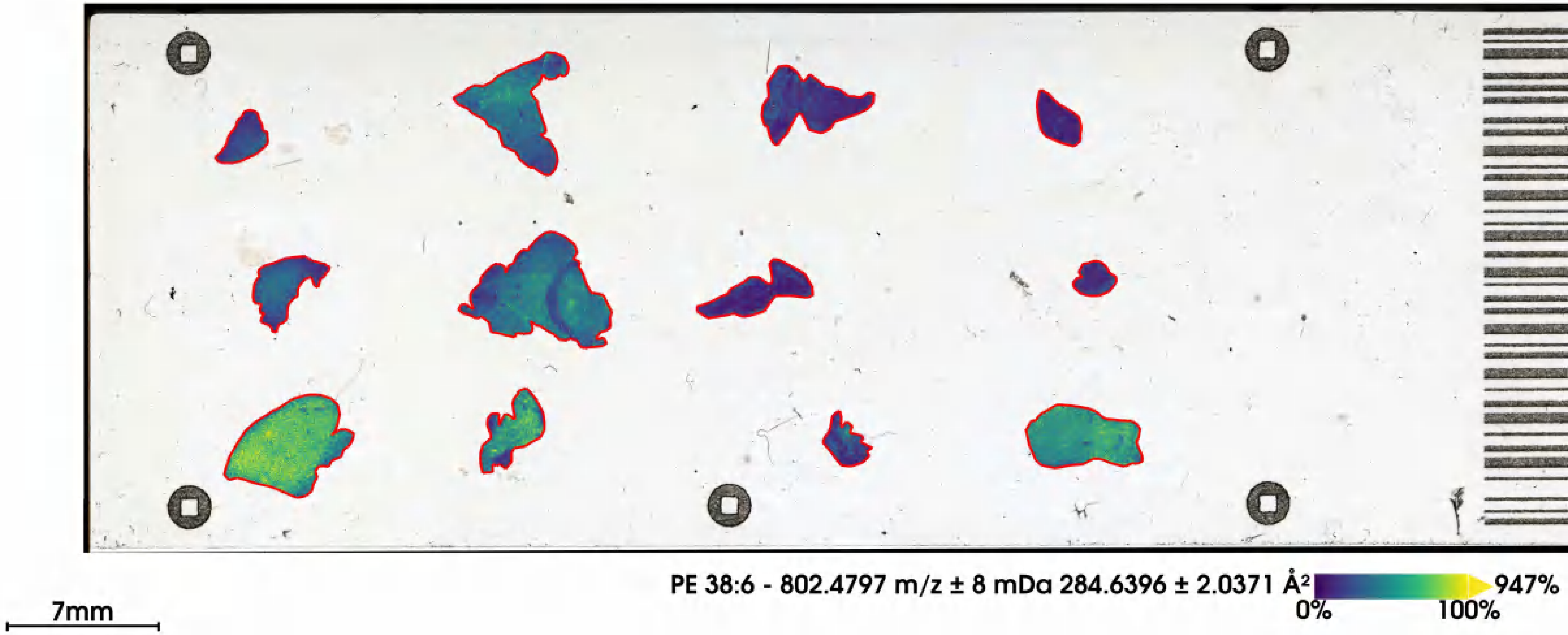
PE 38:6 - 802.4797 m/z ± 8 mDa 284.6396 ± 2.0371 Å²
947%
0%
100%
7mm

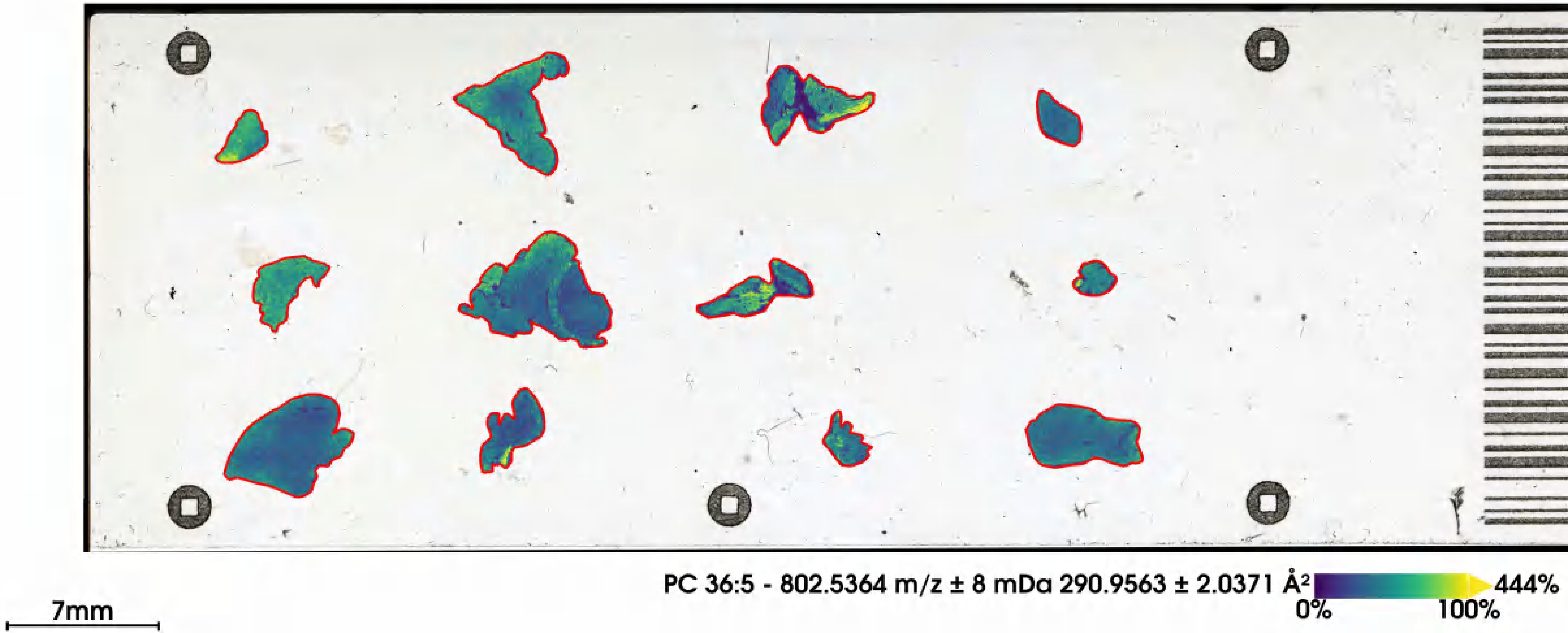
PC 36:5 - 802.5364 m/z ± 8 mDa 290.9563 ± 2.0371 Å²
0%
100%
444%
7mm

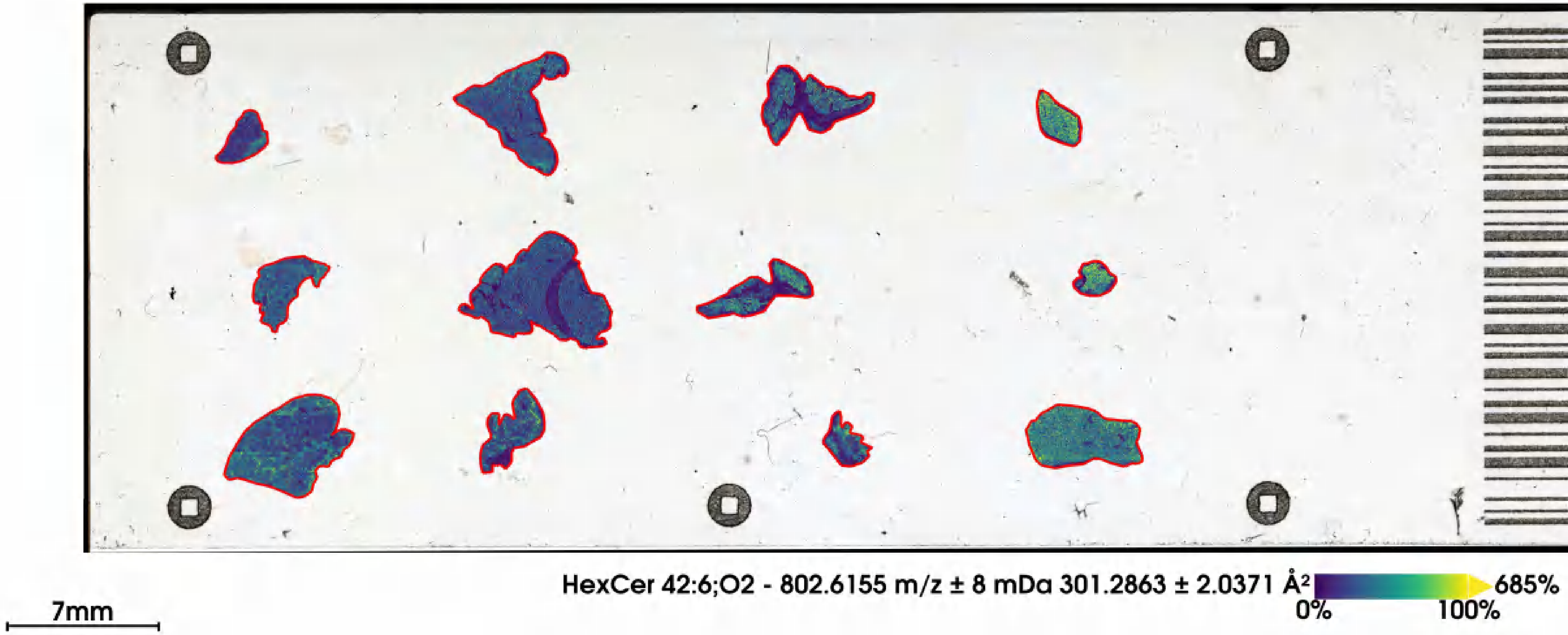
HexCer 42:6;O2 - 802.6155 m/z ± 8 mDa 301.2863 ± 2.0371 Å²
685%
0%
100%
7mm

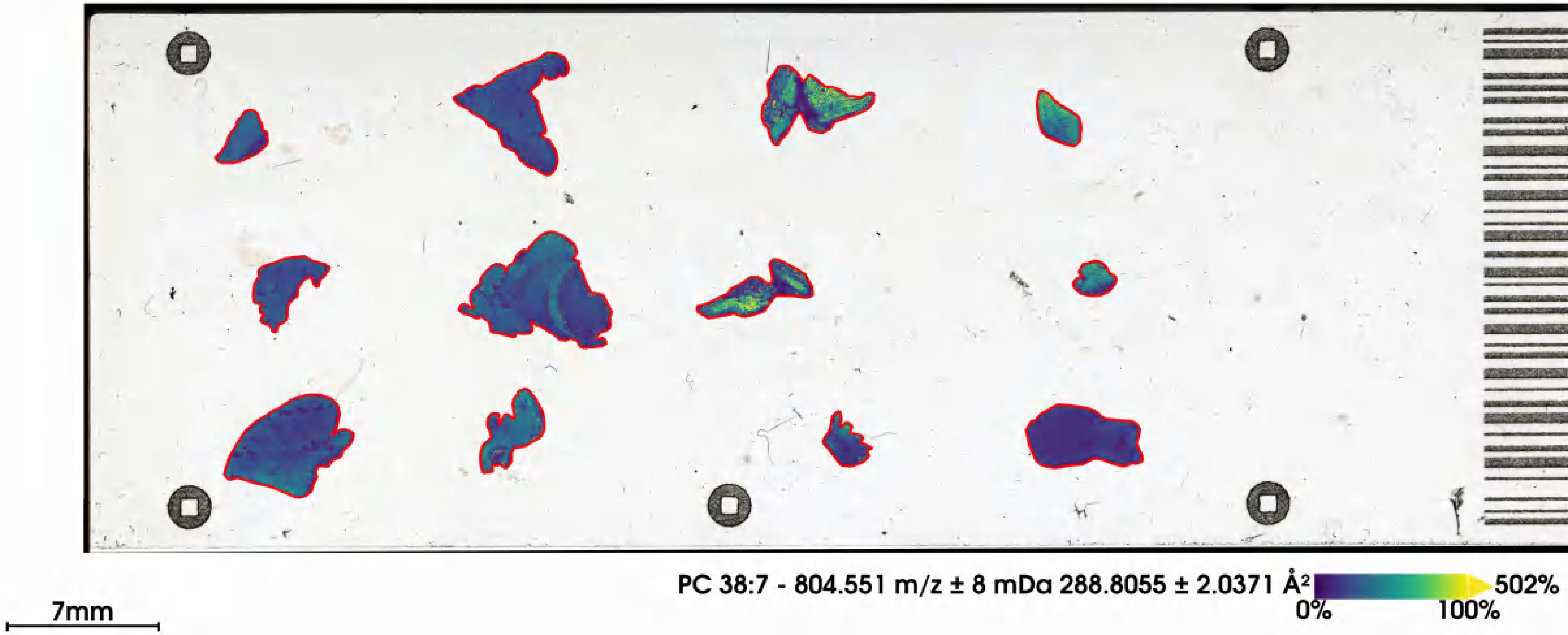
PC 38:7 - 804.551 m/z ± 8 mDa 288.8055 ± 2.0371 Å²
0%
100%
502%
7mm

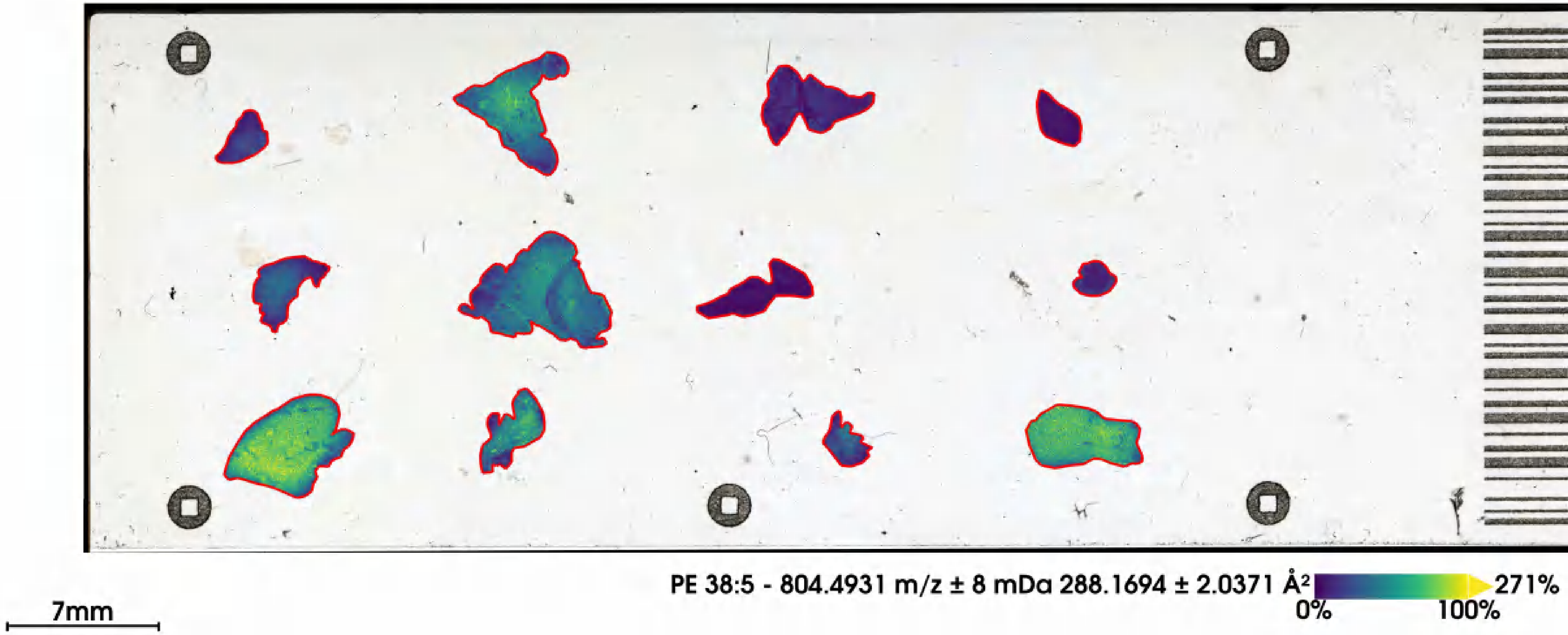
PE 38:5 - 804.4931 m/z ± 8 mDa 288.1694 ± 2.0371 Å²
0%
100%
271%
7mm

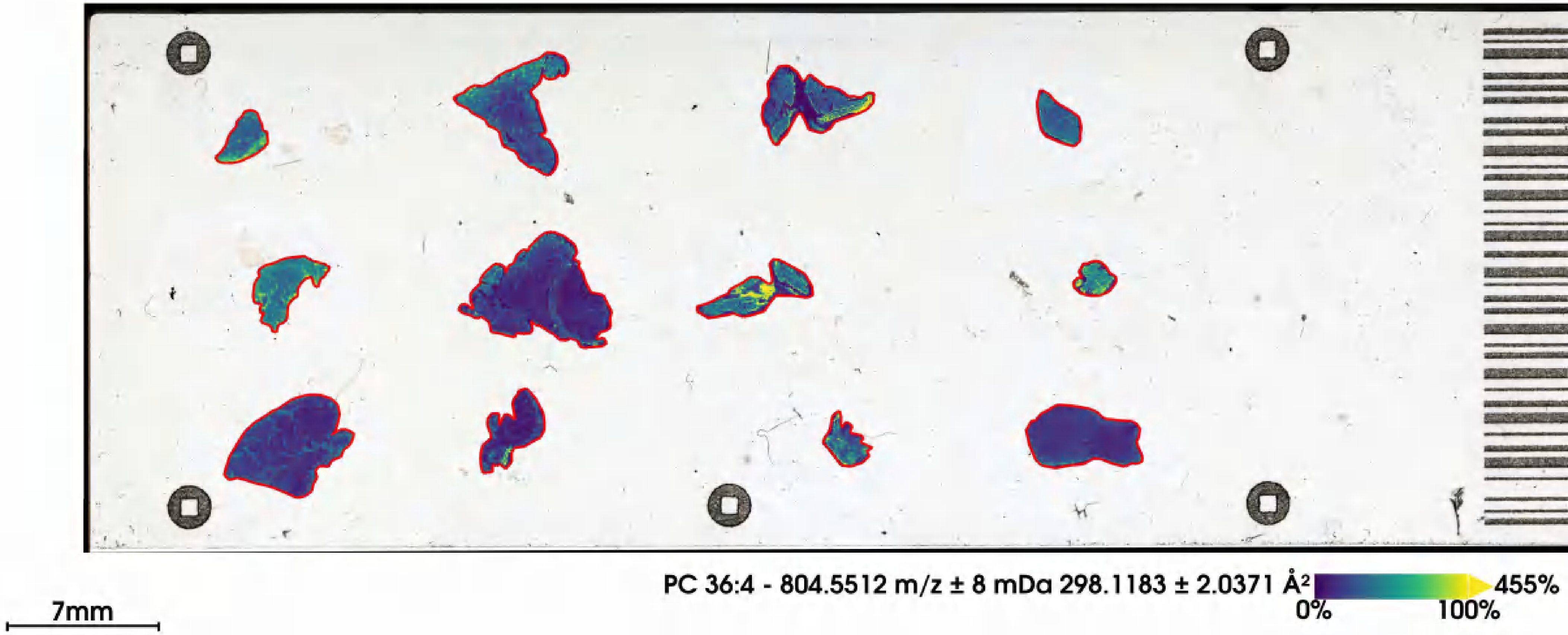

PC 36:4 - 804.5512 m/z ± 8 mDa 298.1183 ± 2.0371 Å²
0%
100%
455%
7mm

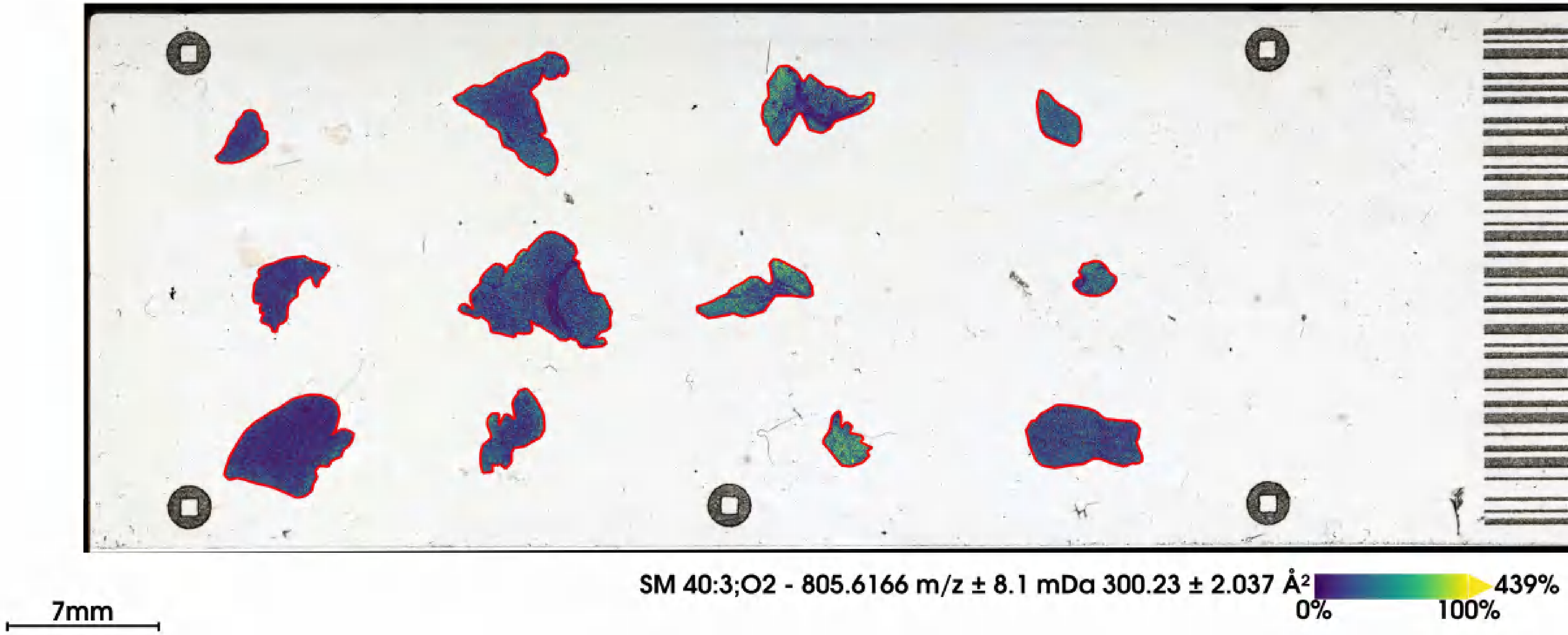
SM 40:3;O2 - 805.6166 m/z ± 8.1 mDa 300.23 ± 2.037 Å²
439%
0%
100%
7mm

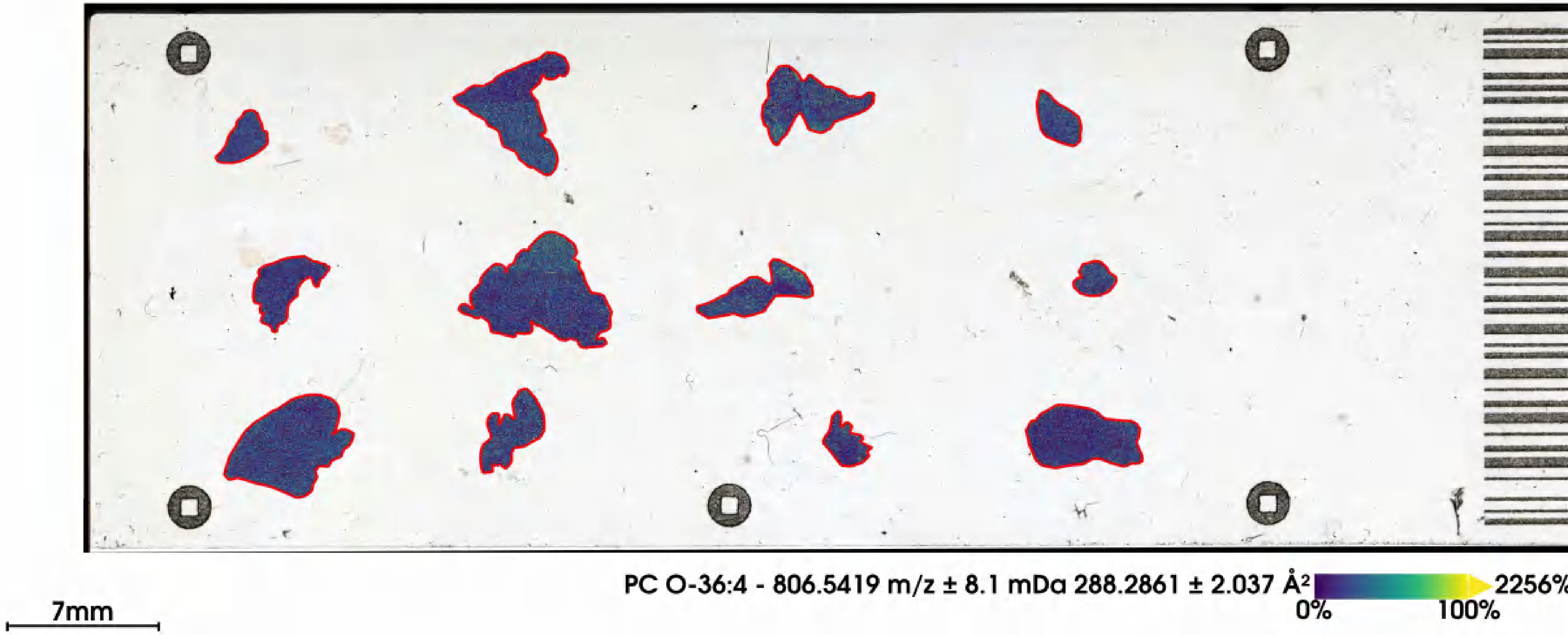
PC O-36:4 - 806.5419 m/z ± 8.1 mDa 288.2861 ± 2.037 Å²
0%
100%
2256%
7mm

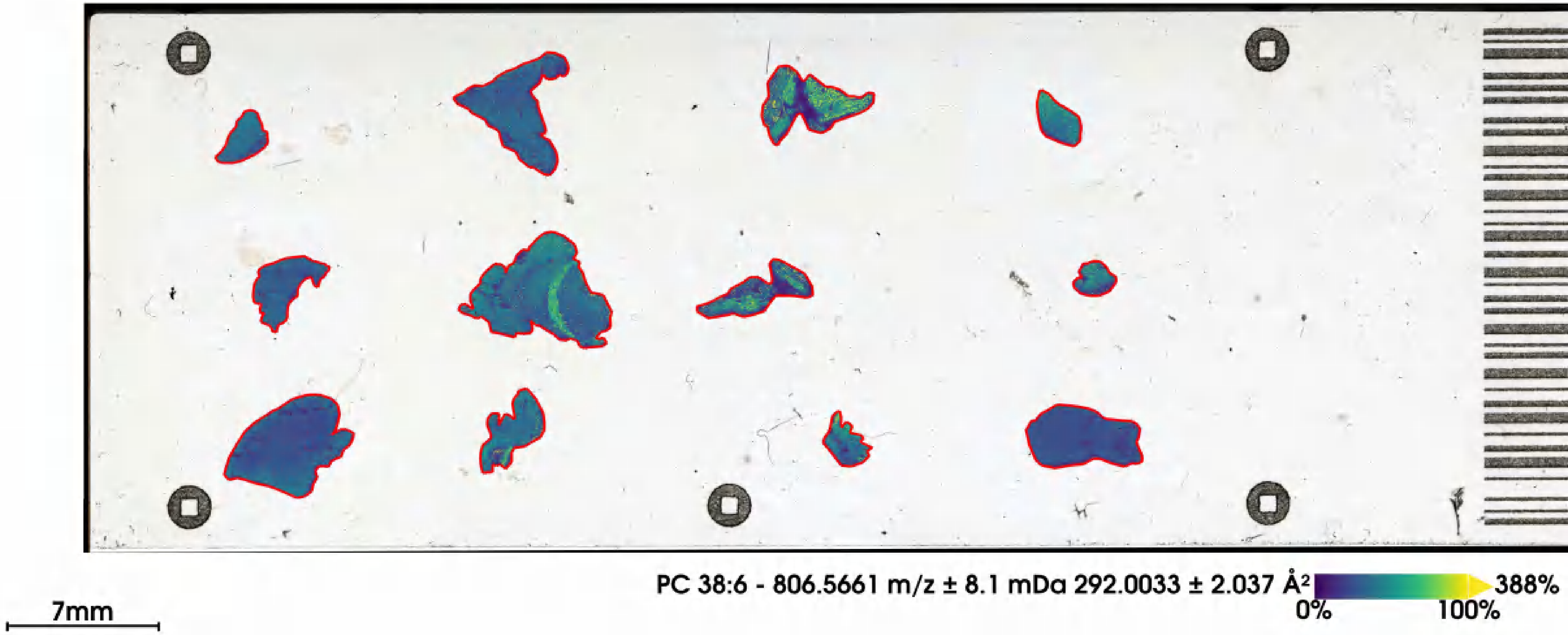

PC 38:6 - 806.5661 m/z ± 8.1 mDa 292.0033 ± 2.037 Å²
0%
100%
388%
7mm

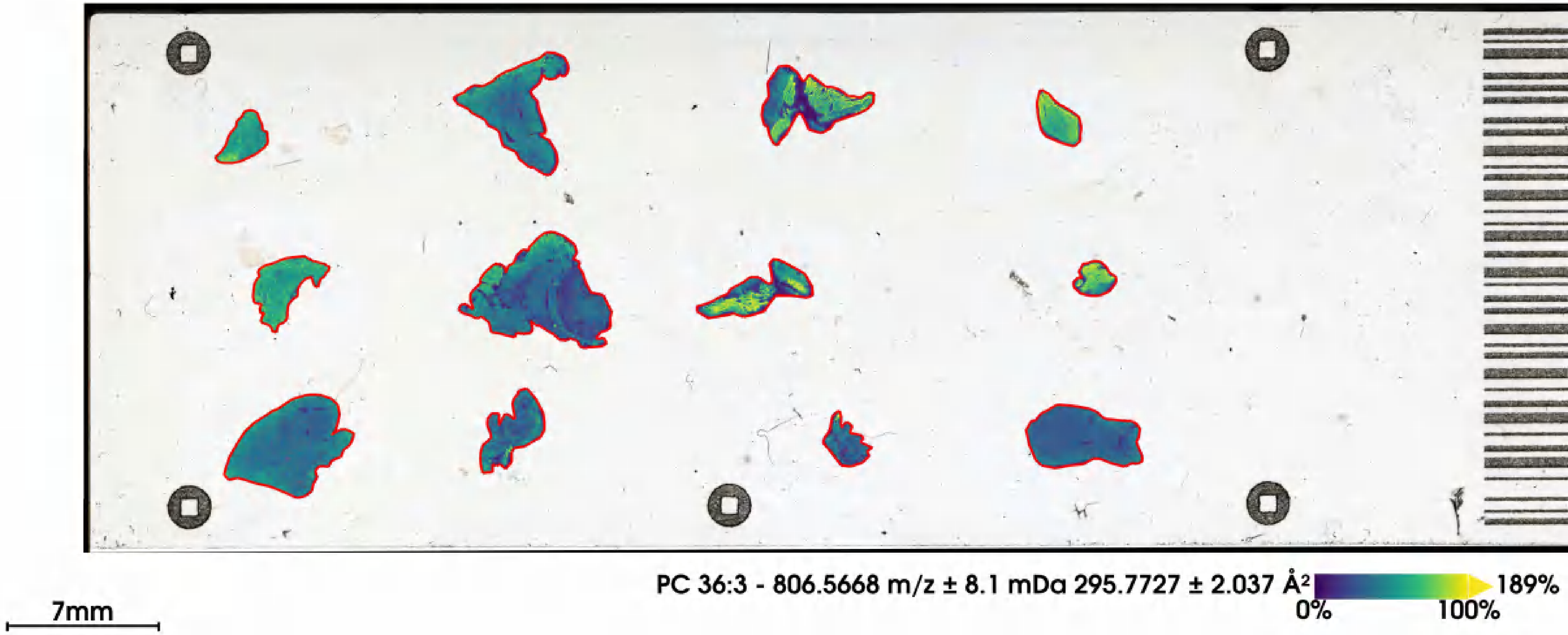
PC 36:3 - 806.5668 m/z ± 8.1 mDa 295.7727 ± 2.037 Å²
0%
100%
189%
7mm

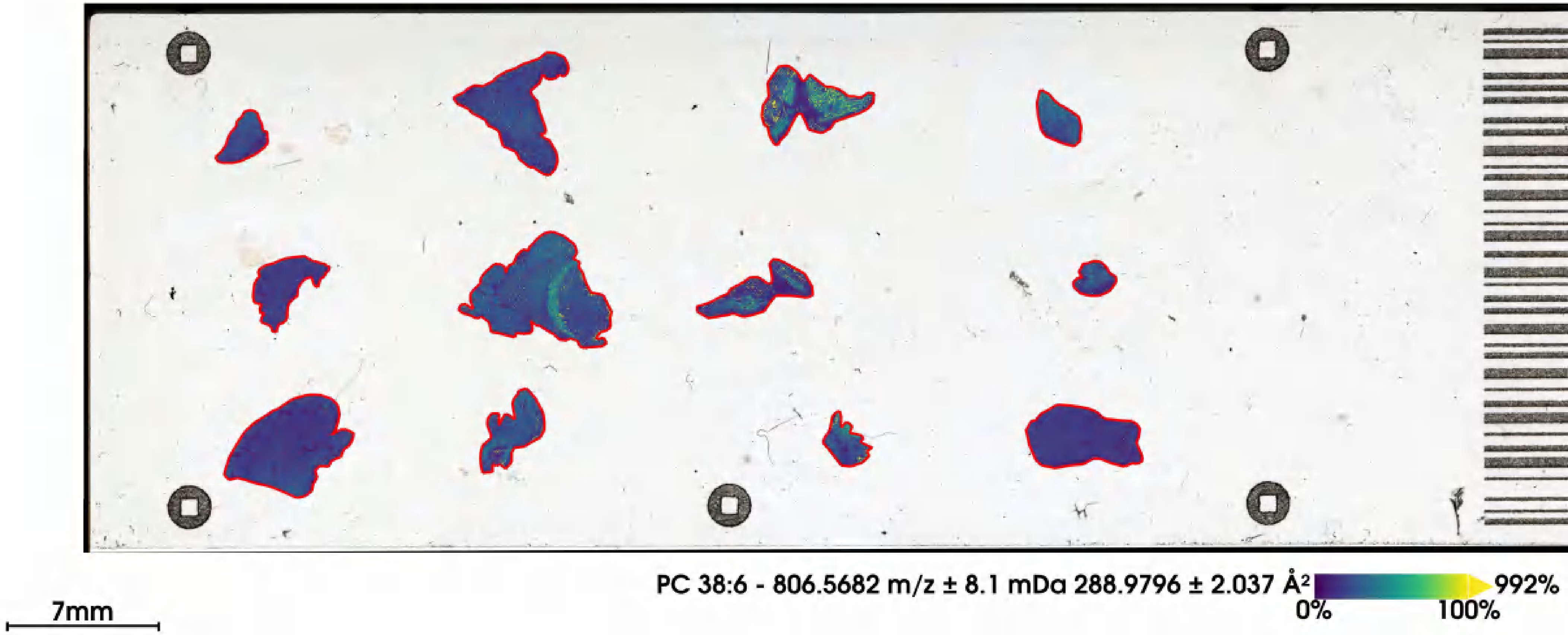

7mm
PC 38:6 - 806.5682 m/z ± 8.1 mDa 288.9796 ± 2.037 Å²
0%
100%
992%

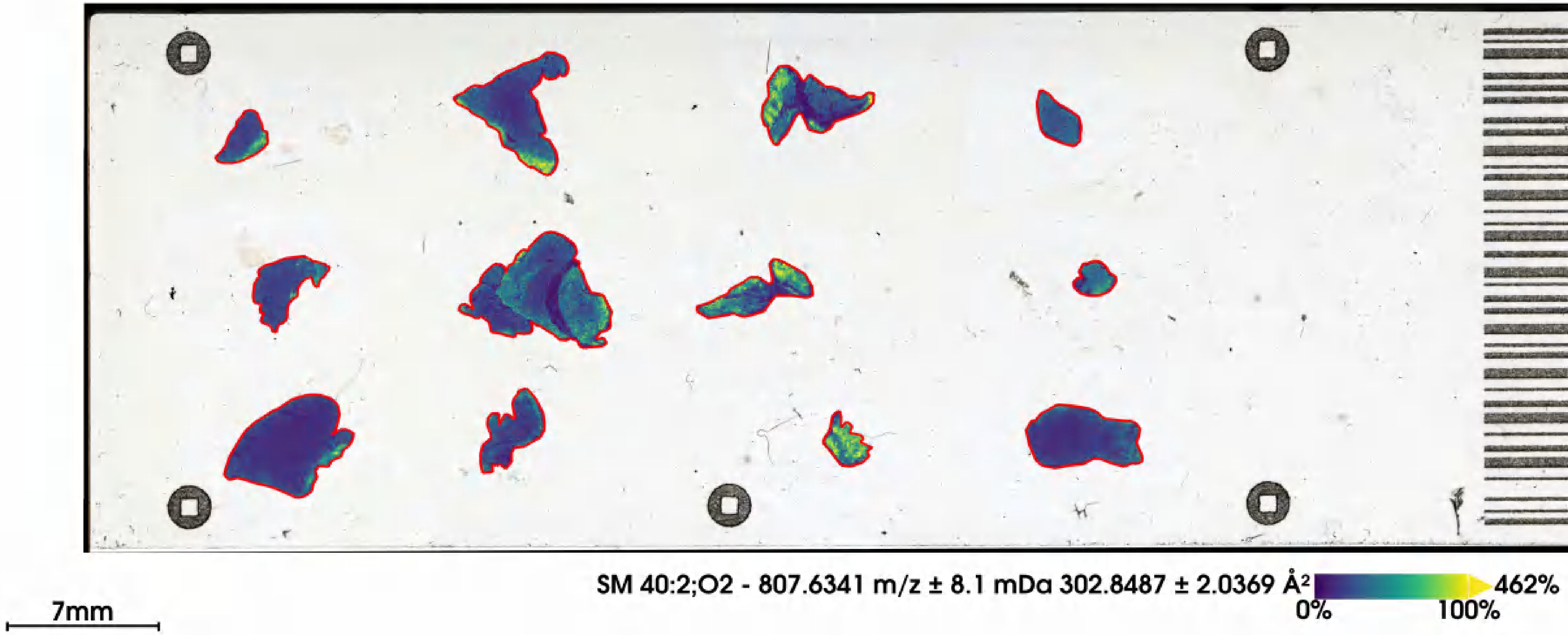
SM 40:2;O2 - 807.6341 m/z ± 8.1 mDa 302.8487 ± 2.0369 Å²
0%
100%
462%
7mm

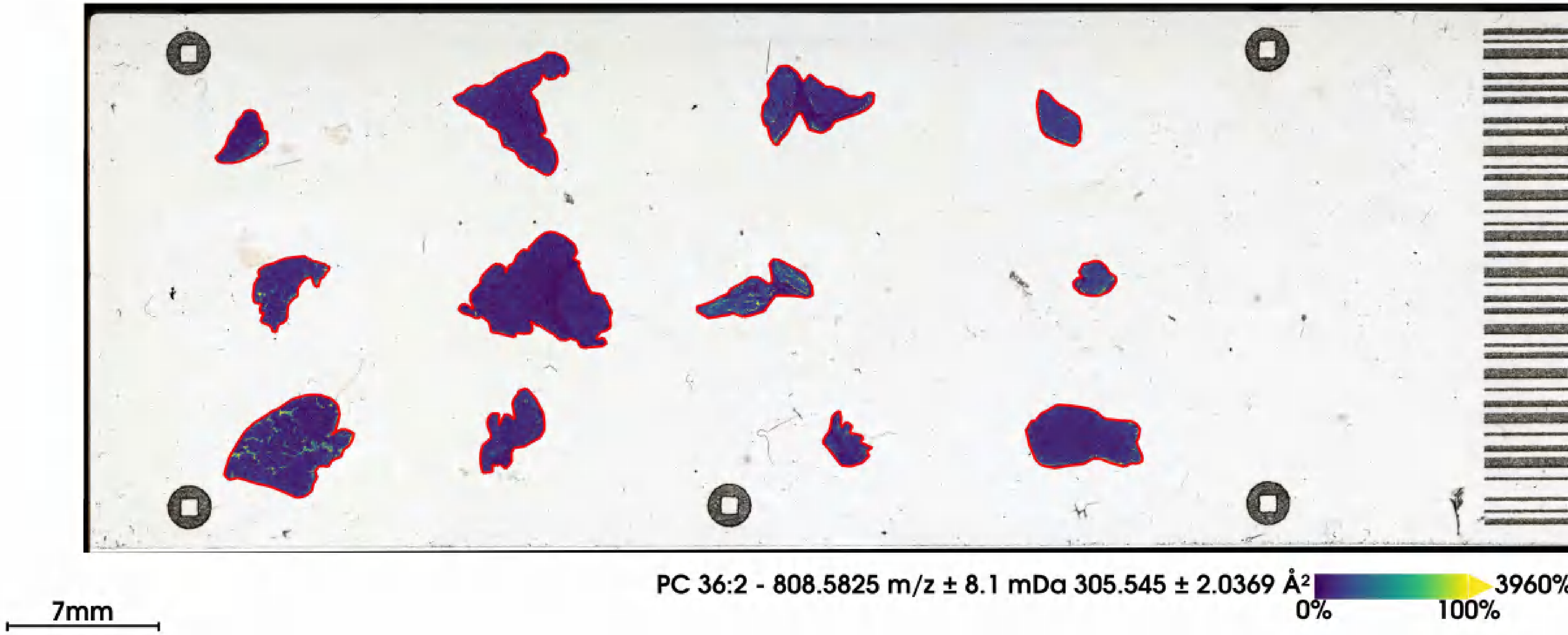
PC 36:2 - 808.5825 m/z ± 8.1 mDa 305.545 ± 2.0369 Å²
3960%
0%
100%
7mm

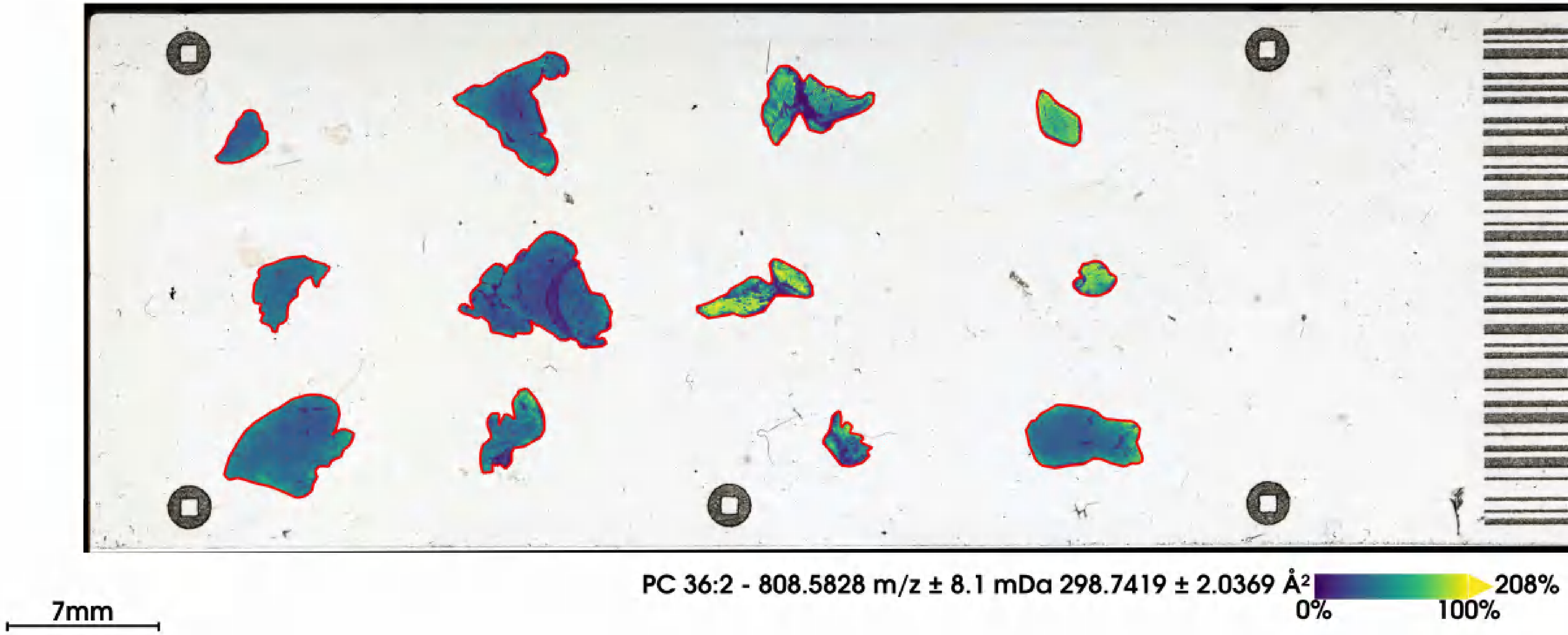
PC 36:2 - 808.5828 m/z ± 8.1 mDa 298.7419 ± 2.0369 Å²
0%
100%
208%
7mm

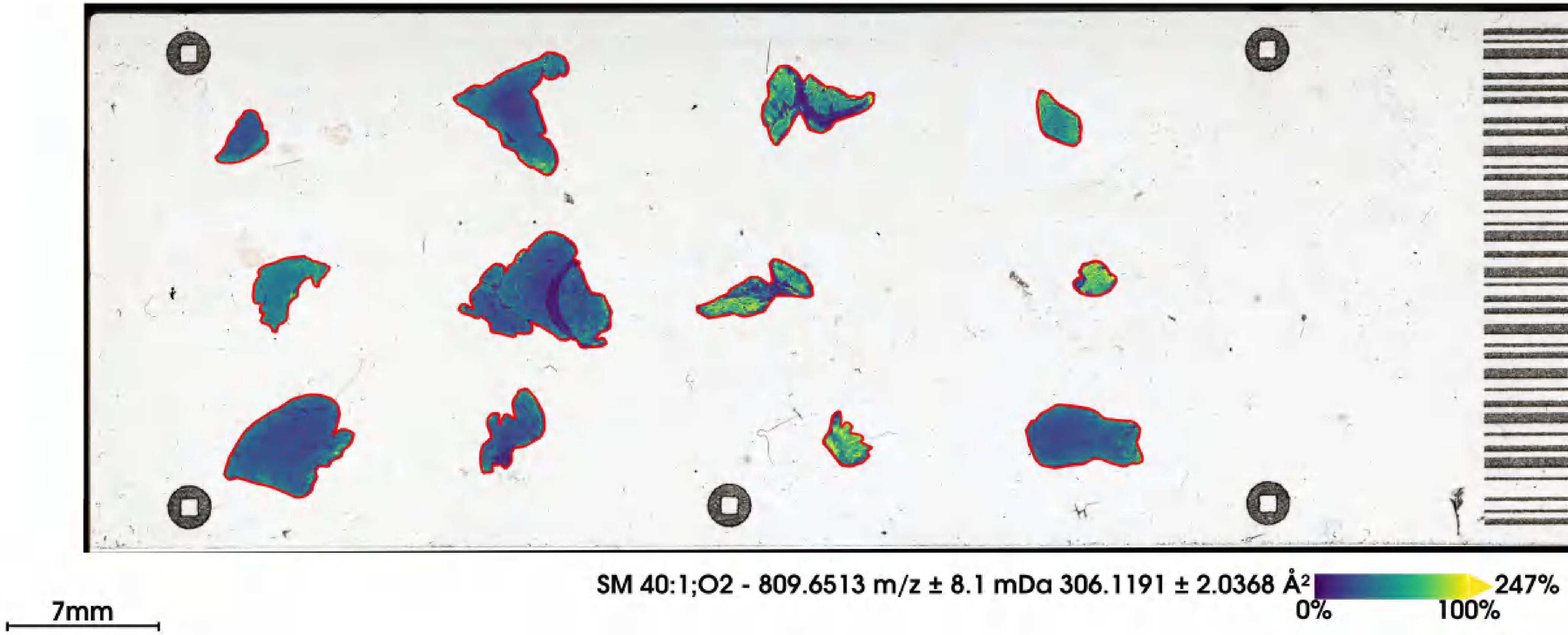

SM 40:1;O2 - 809.6513 m/z ± 8.1 mDa 306.1191 ± 2.0368 Å²
0%
100%
247%
7mm

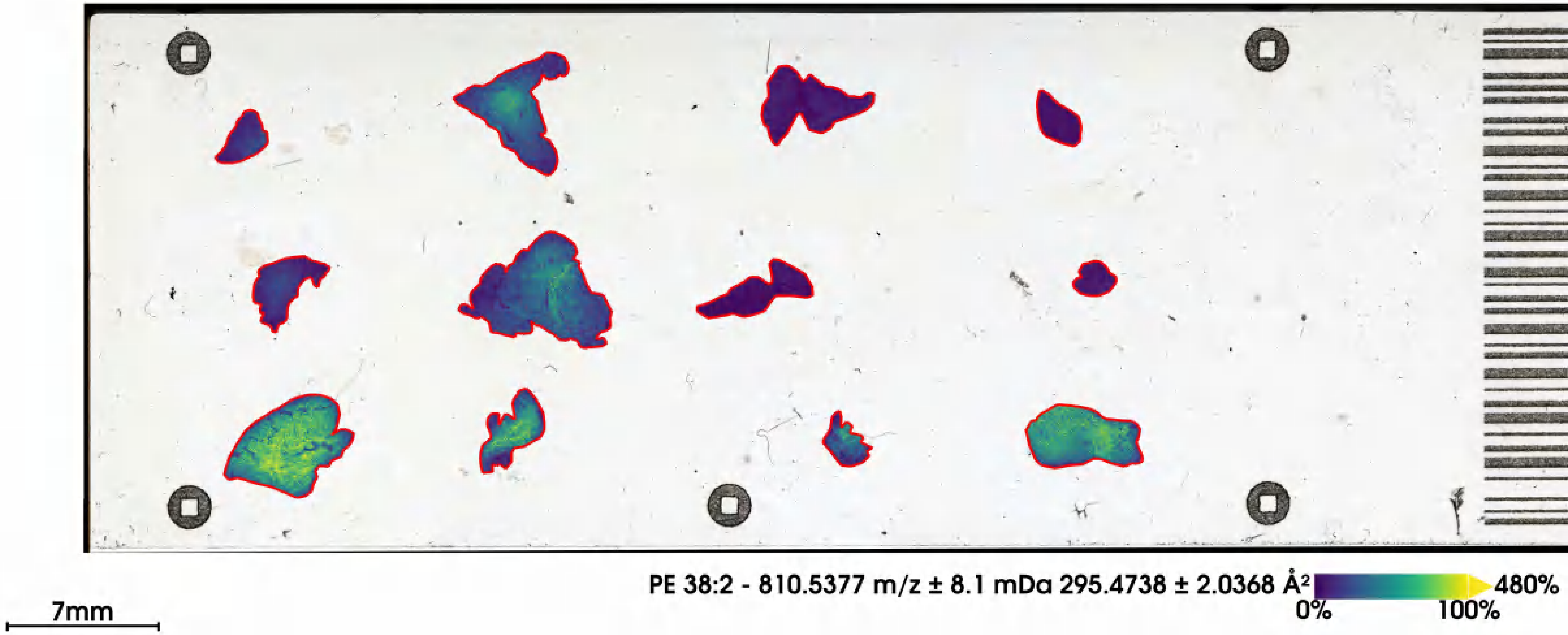
PE 38:2 - 810.5377 m/z ± 8.1 mDa 295.4738 ± 2.0368 Å²
0%
100%
480%
7mm

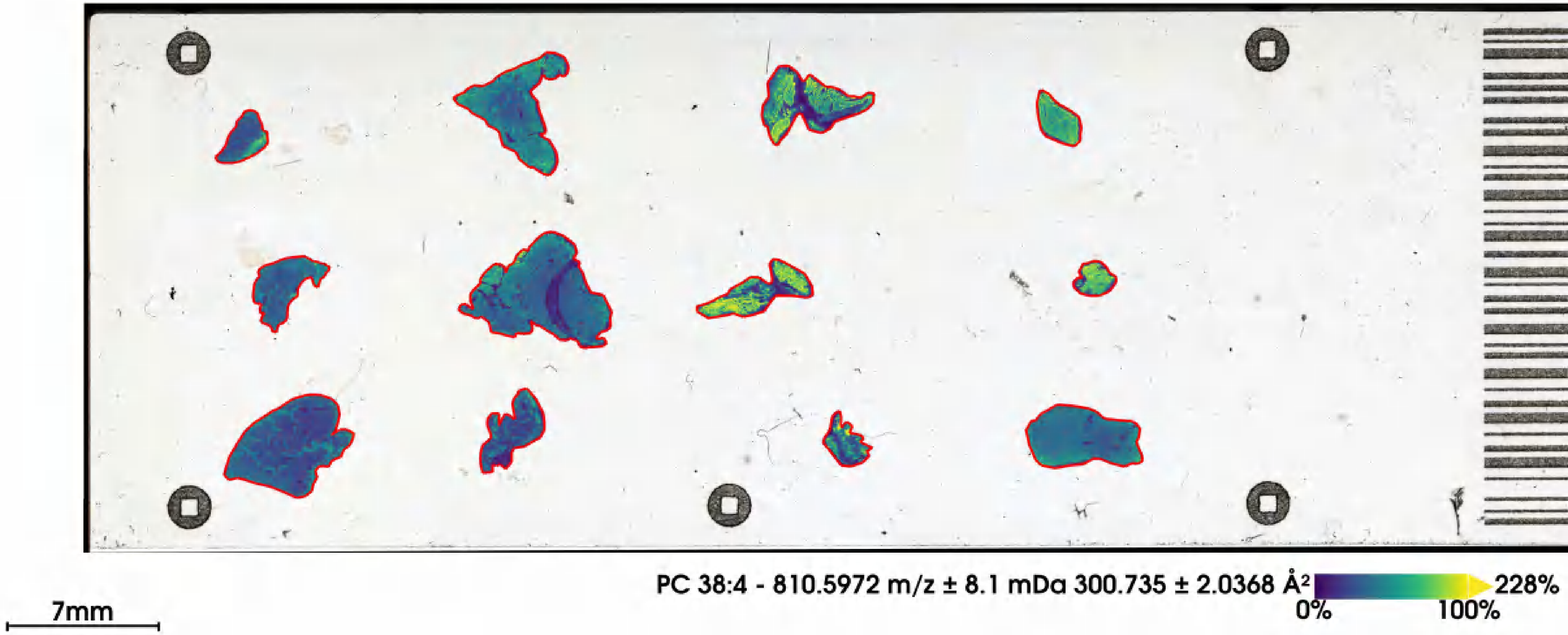

7mm
PC 38:4 - 810.5972 m/z ± 8.1 mDa 300.735 ± 2.0368 Å²
0%
100%
228%

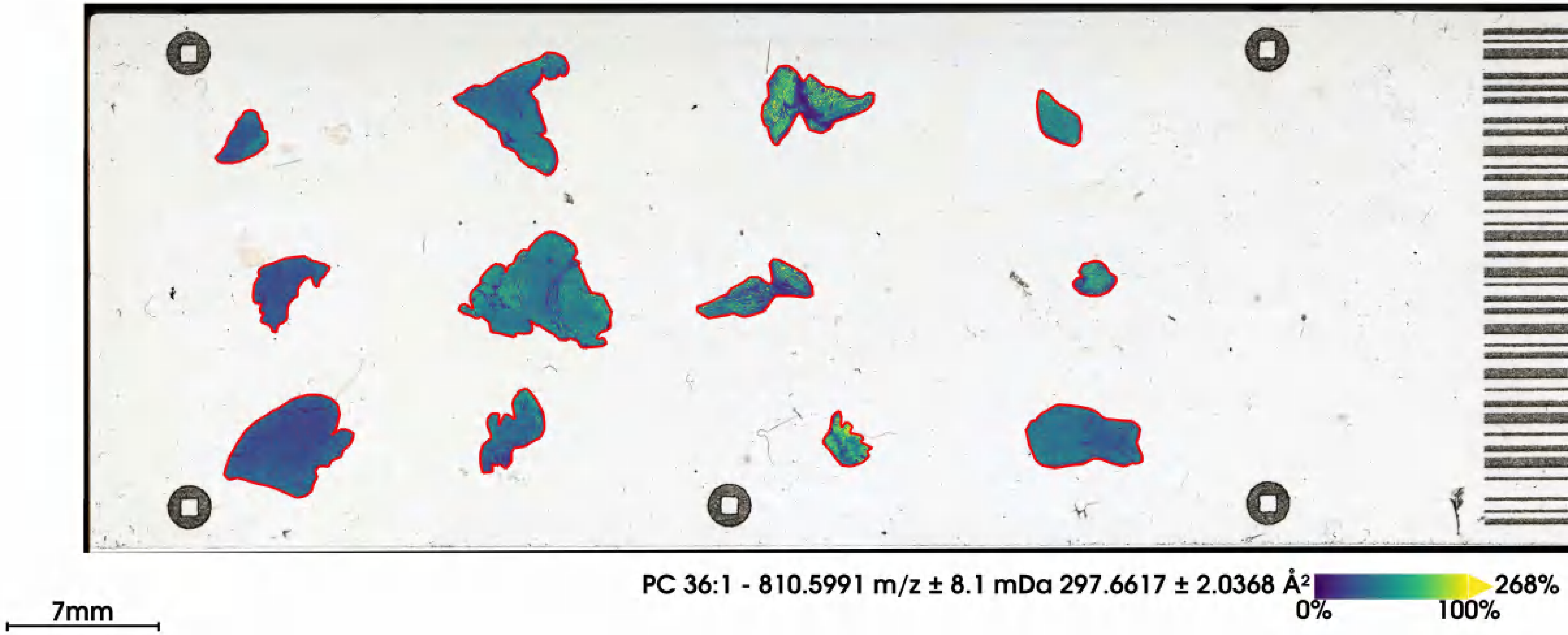

7mm
PC 36:1 - 810.5991 m/z ± 8.1 mDa 297.6617 ± 2.0368 Å²
0%
100%
268%

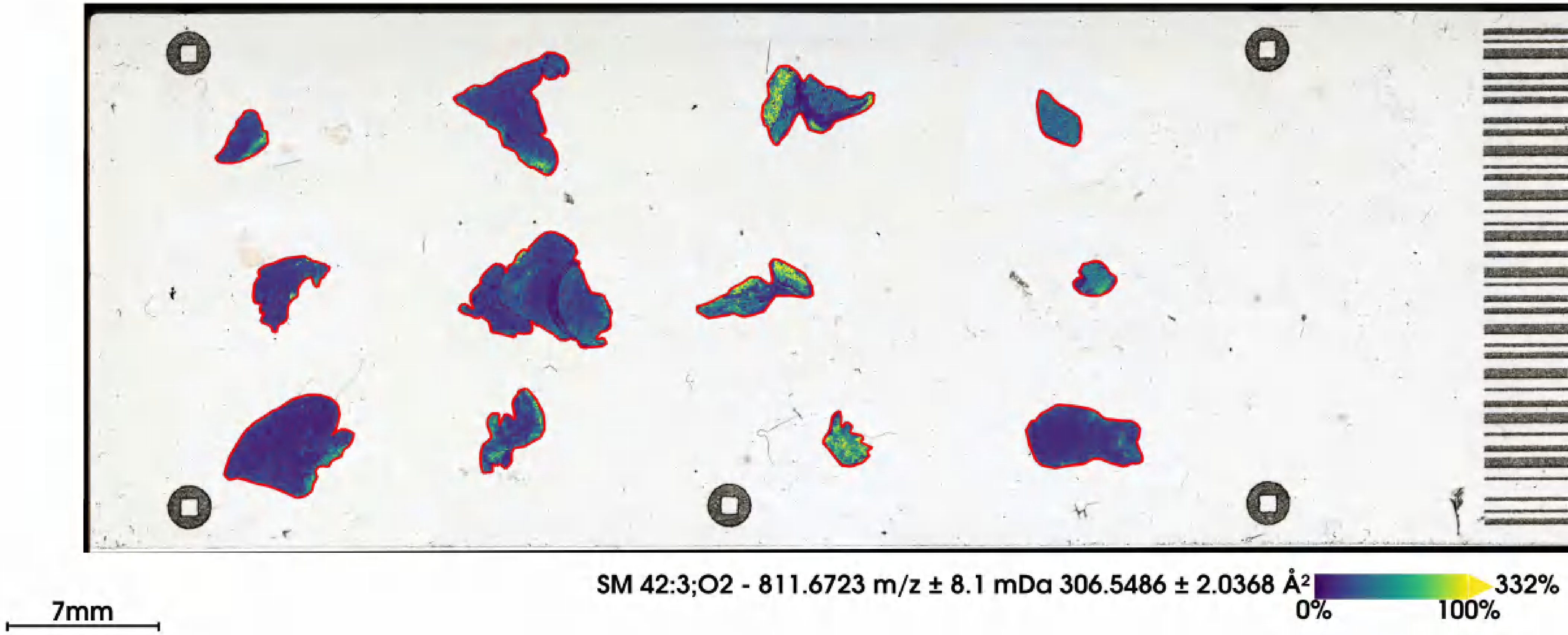
SM 42:3;O2 - 811.6723 m/z ± 8.1 mDa 306.5486 ± 2.0368 Å²
0%
100%
332%
7mm

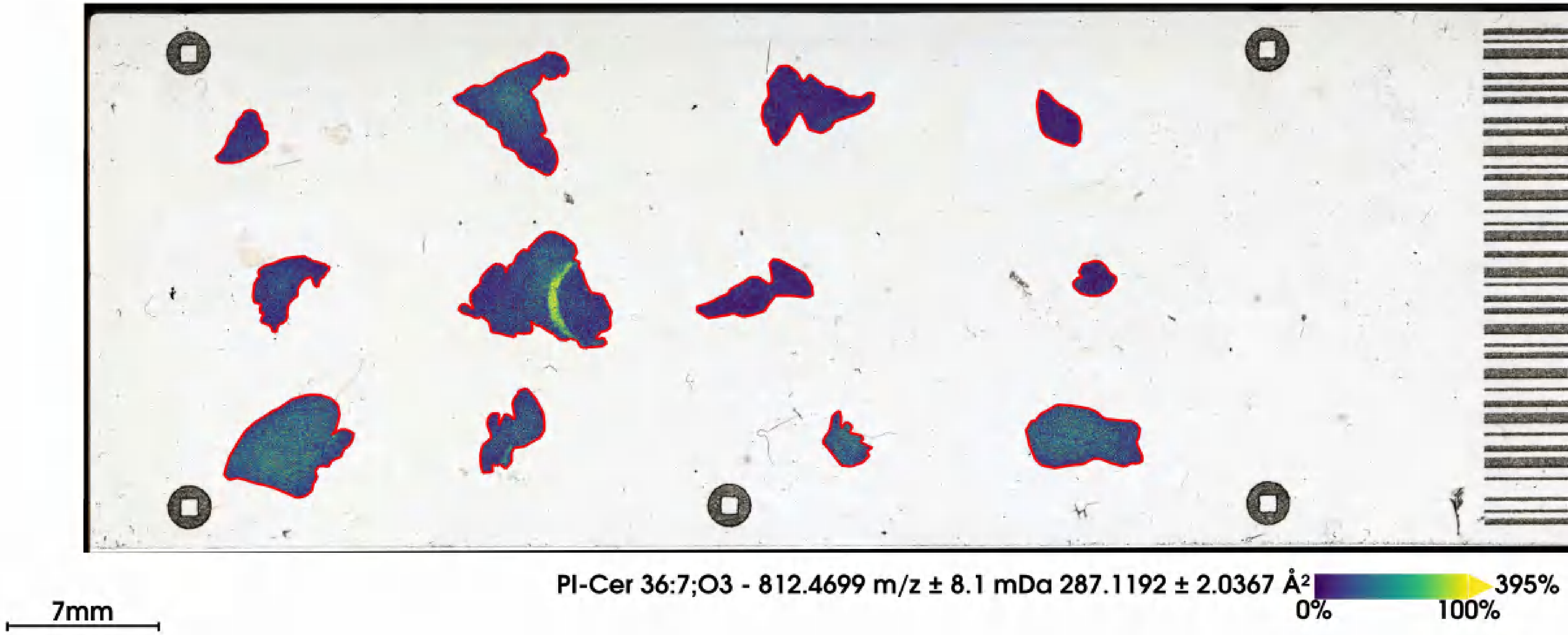
PI-Cer 36:7;O3 - 812.4699 m/z ± 8.1 mDa 287.1192 ± 2.0367 Å²
0%
100%
395%
7mm

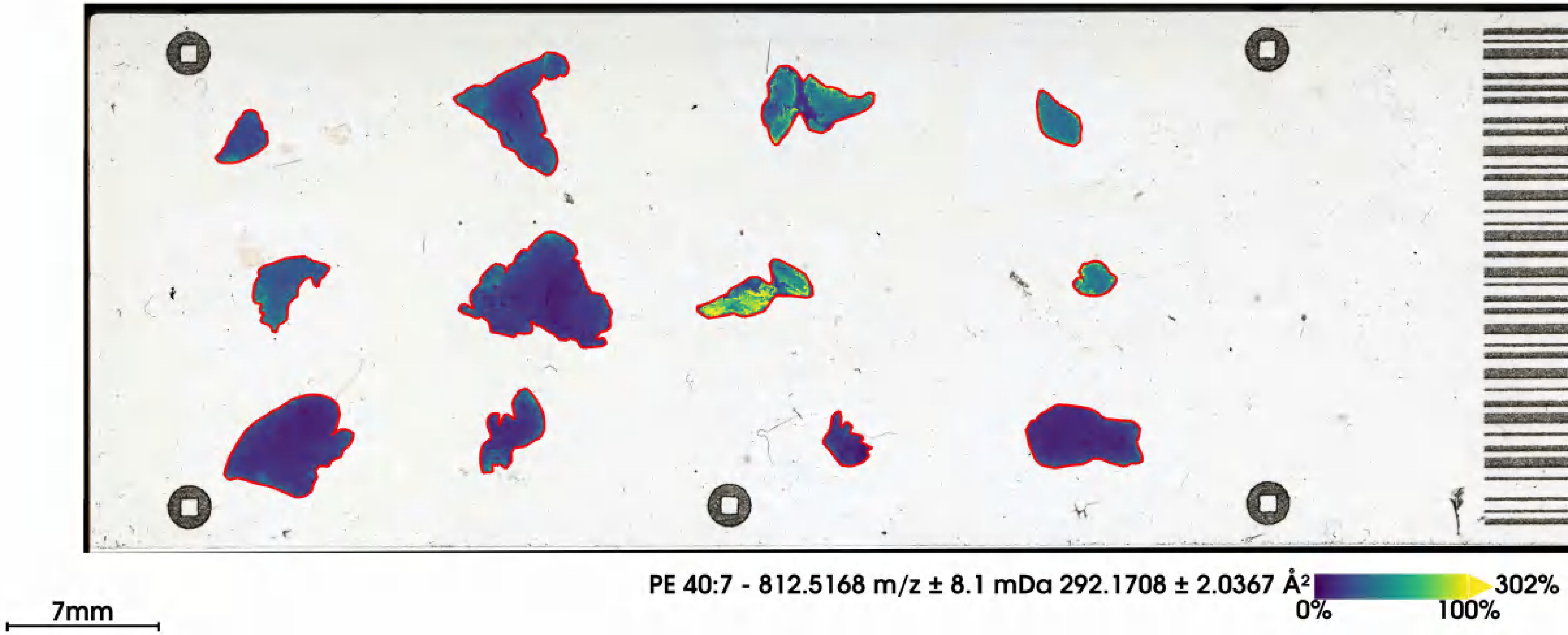
PE 40:7 - 812.5168 m/z ± 8.1 mDa 292.1708 ± 2.0367 Å²
0%
100%
302%
7mm

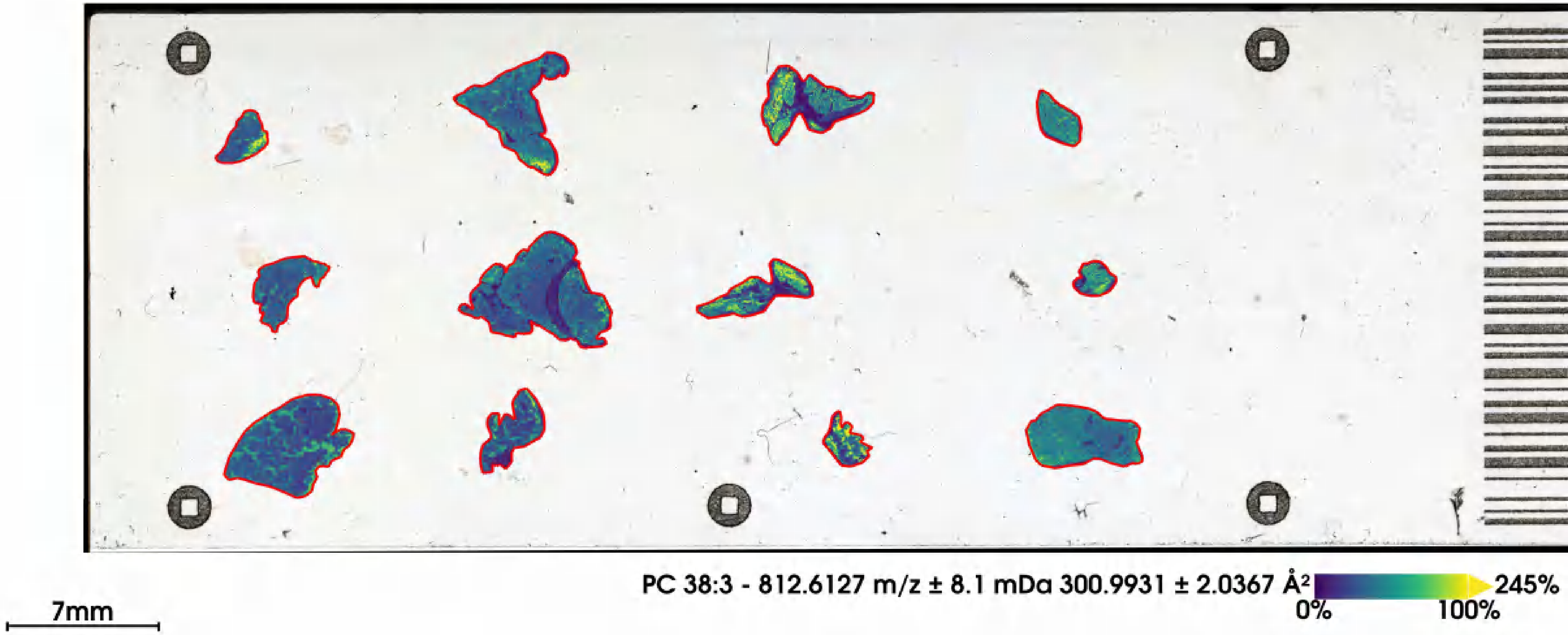
PC 38:3 - 812.6127 m/z ± 8.1 mDa 300.9931 ± 2.0367 Å²
0%
100%
245%
7mm

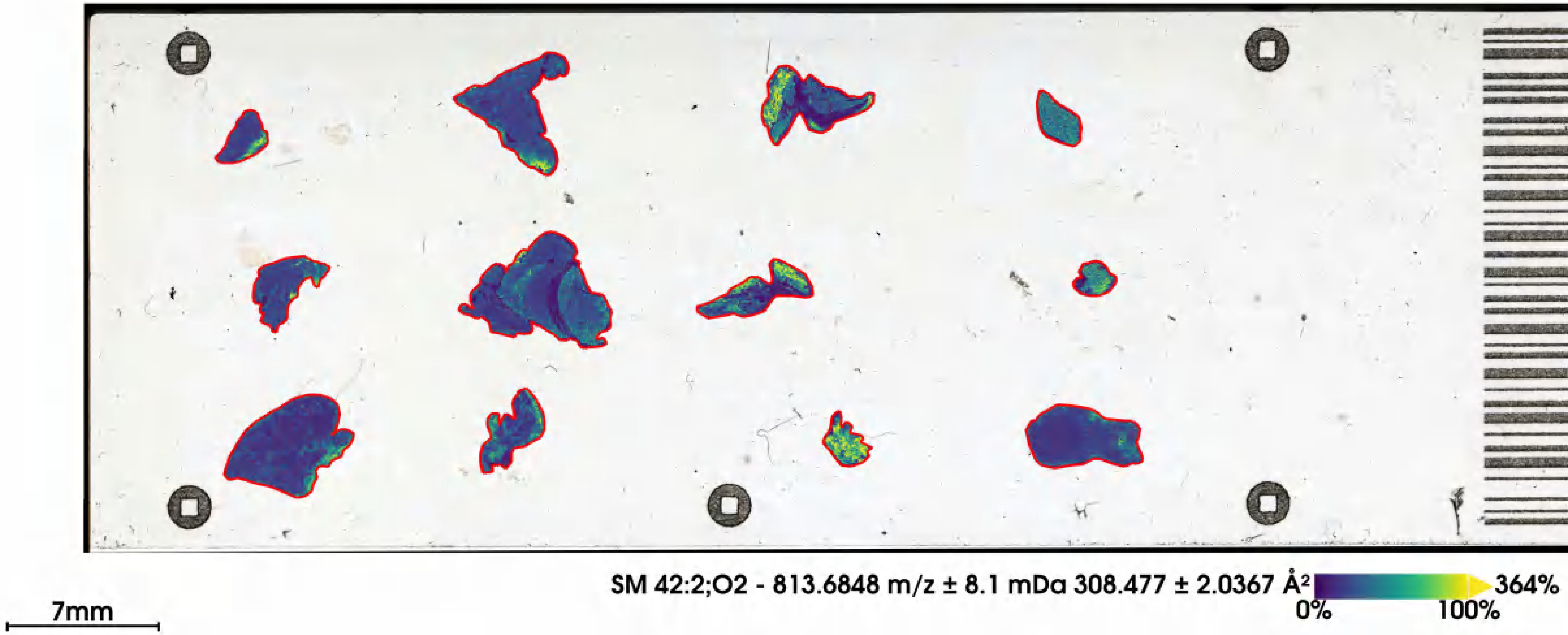

SM 42:2;O2 - 813.6848 m/z ± 8.1 mDa 308.477 ± 2.0367 Å²
0%
100%
364%
7mm

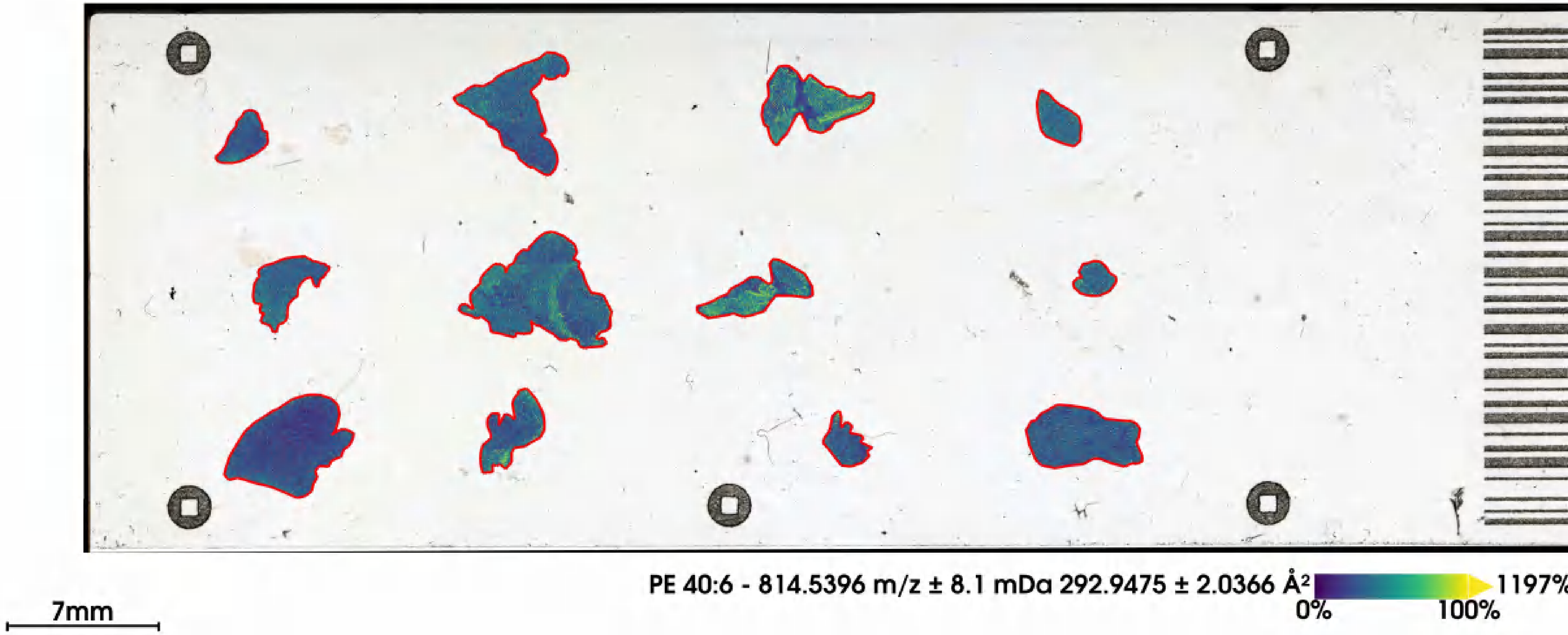
7mm
PE 40:6 - 814.5396 m/z ± 8.1 mDa 292.9475 ± 2.0366 Å²
0%
100%
1197%

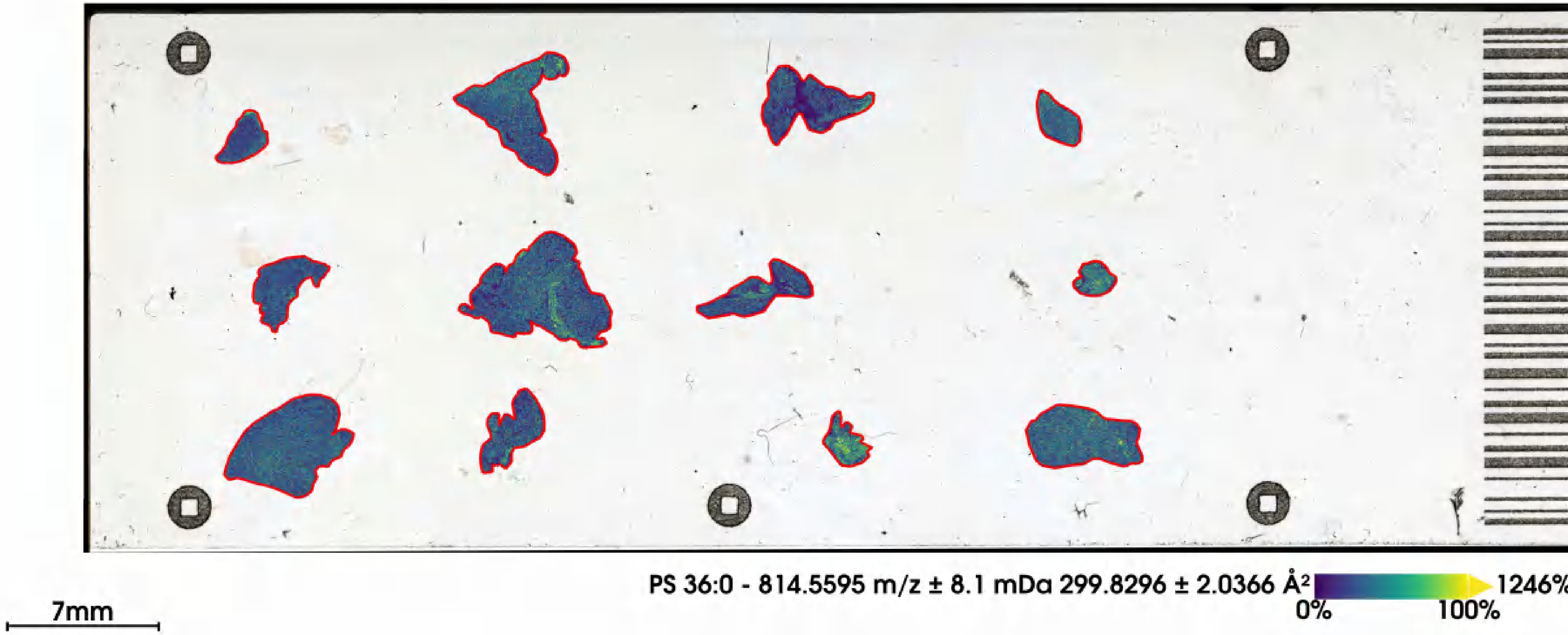
PS 36:0 - 814.5595 m/z ± 8.1 mDa 299.8296 ± 2.0366 Å²
0%
100%
1246%
7mm

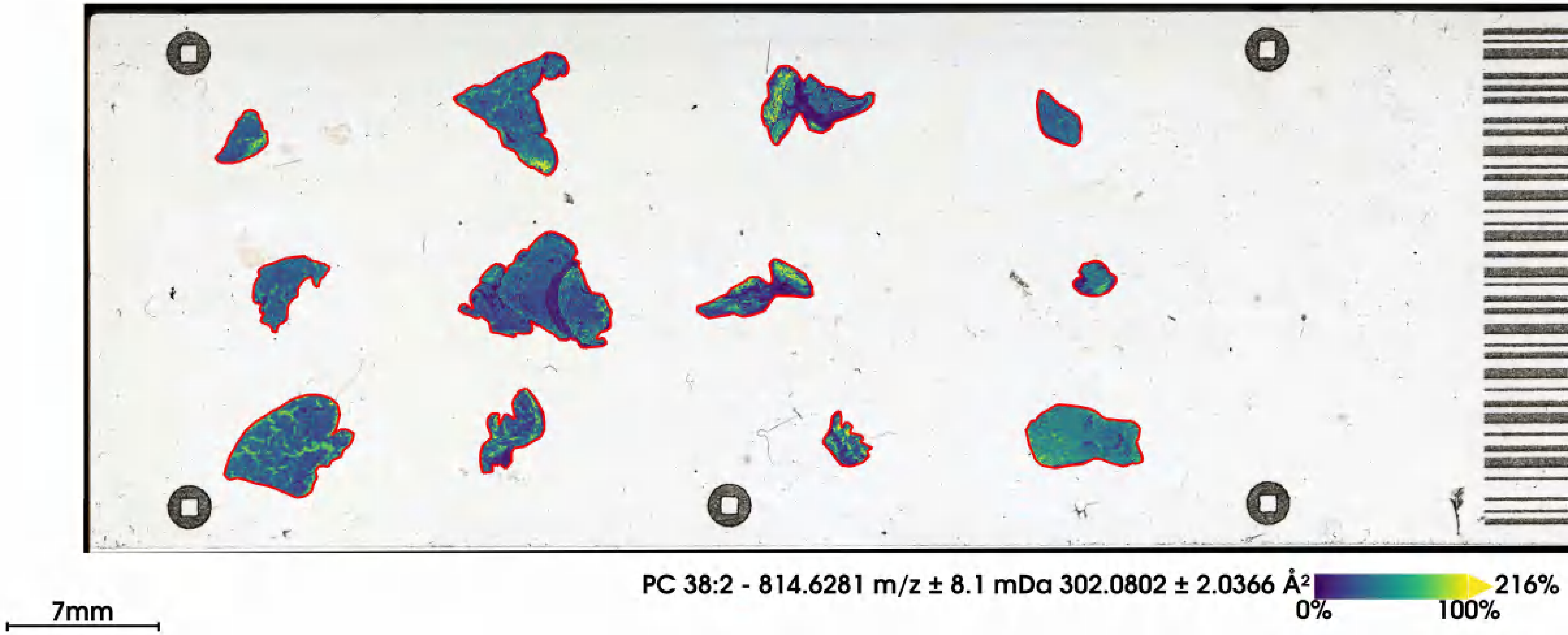
PC 38:2 - 814.6281 m/z ± 8.1 mDa 302.0802 ± 2.0366 Å²
0%
100%
216%
7mm

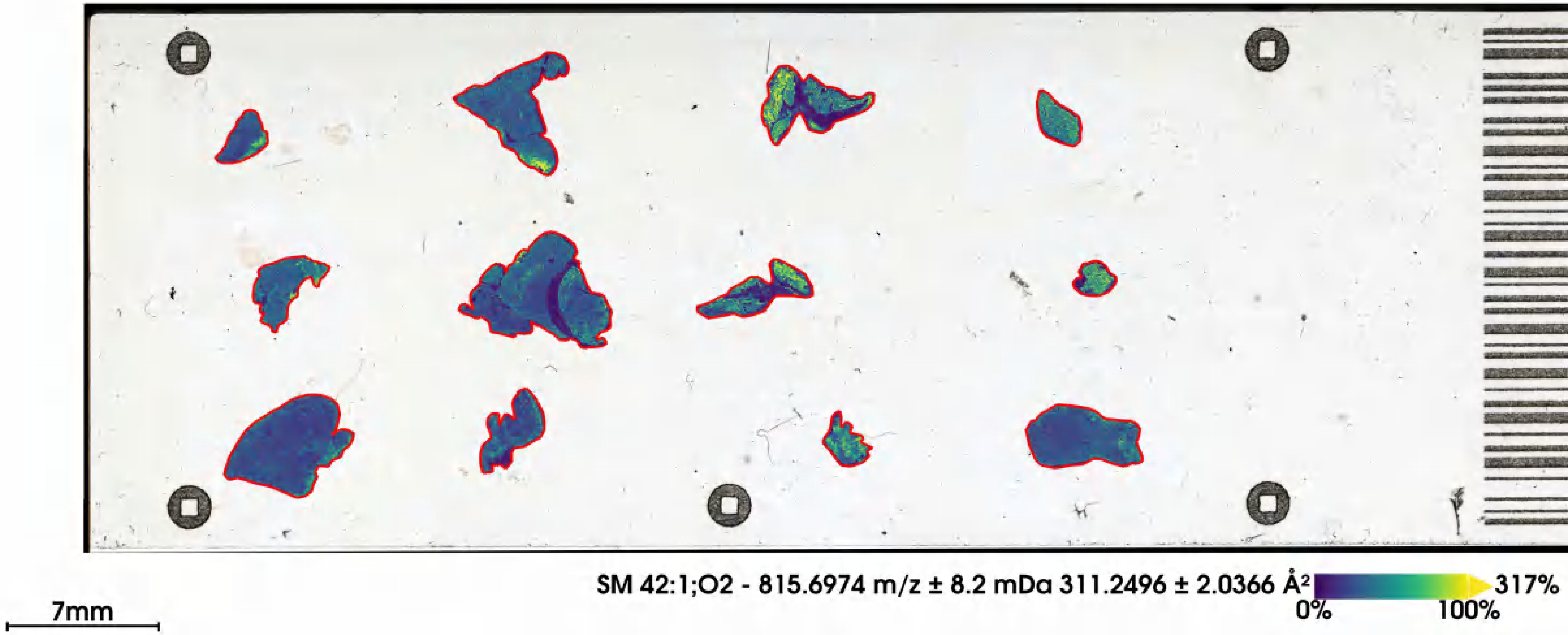

SM 42:1;O2 - 815.6974 m/z ± 8.2 mDa 311.2496 ± 2.0366 Å²
0%
100%
317%
7mm

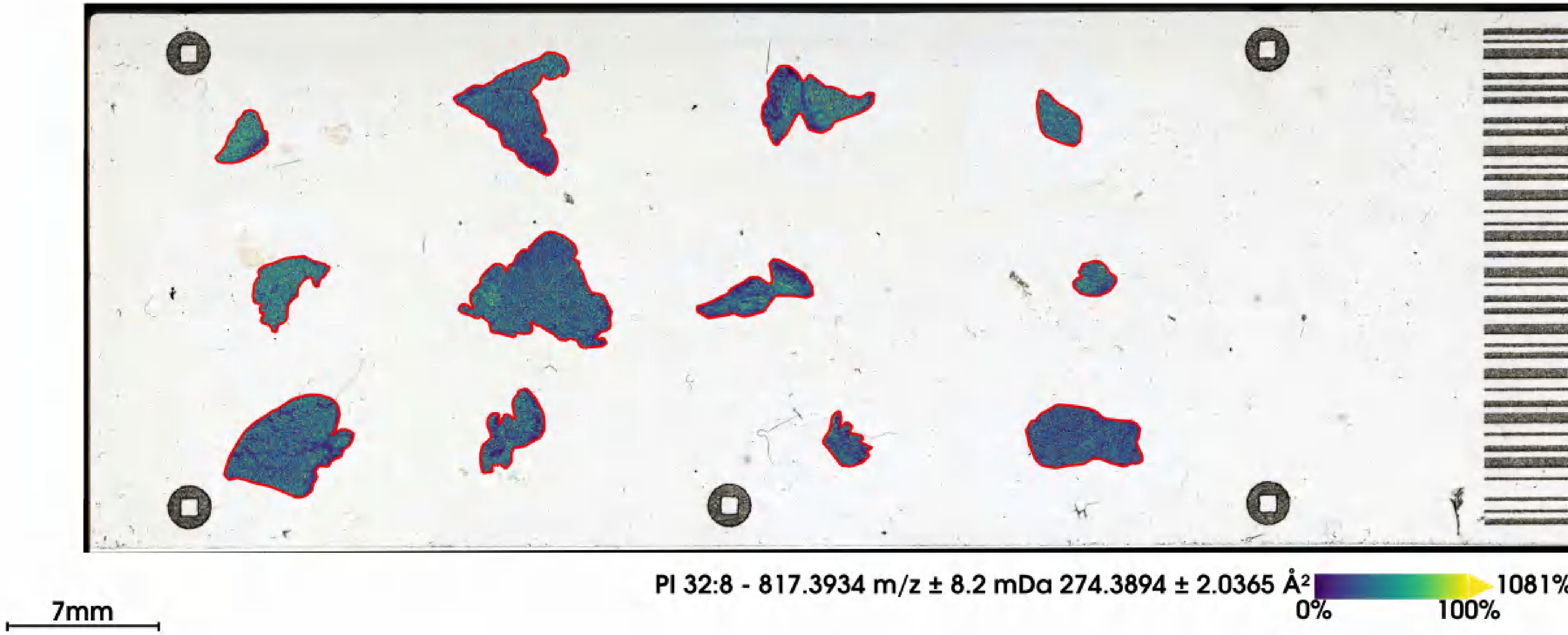
PI 32:8 - 817.3934 m/z ± 8.2 mDa 274.3894 ± 2.0365 Å²
0%
100%
1081%
7mm

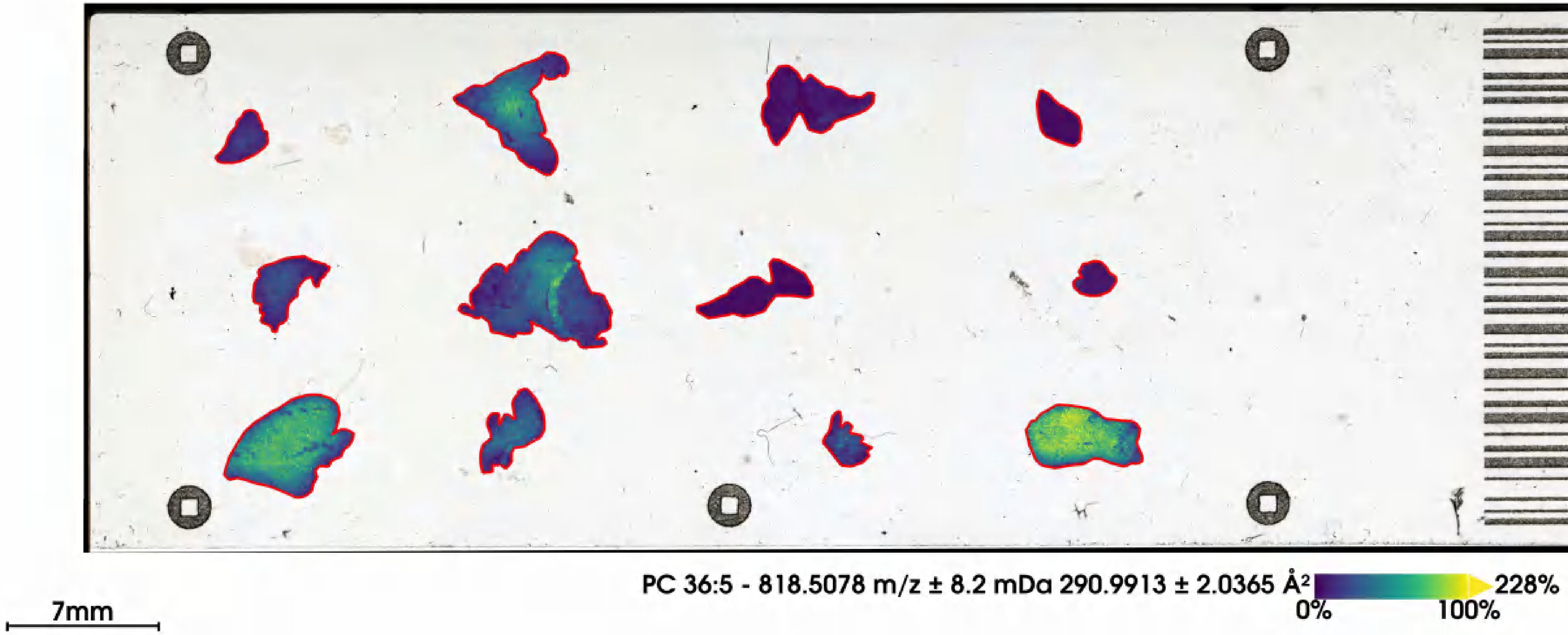

7mm
PC 36:5 - 818.5078 m/z ± 8.2 mDa 290.9913 ± 2.0365 Å²
0%
100%
228%

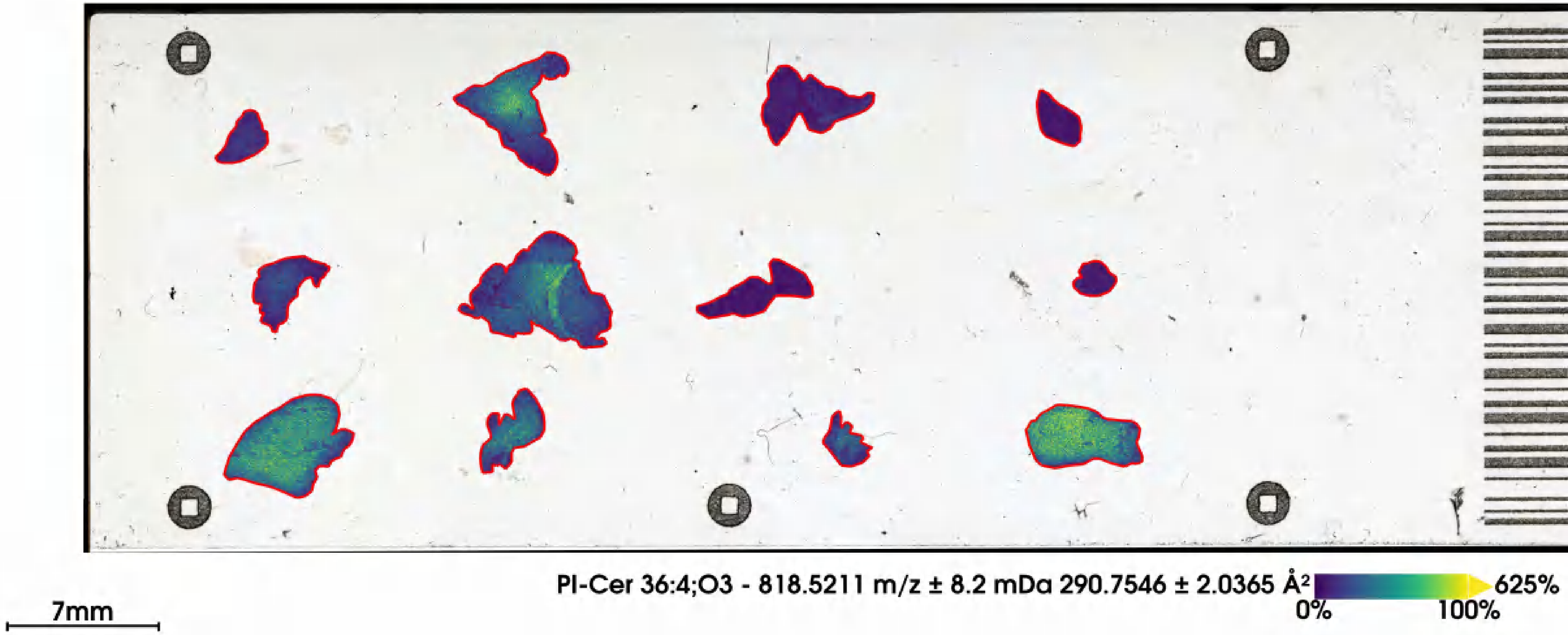

PI-Cer 36:4;O3 - 818.5211 m/z ± 8.2 mDa 290.7546 ± 2.0365 Å²
0%
100%
625%
7mm

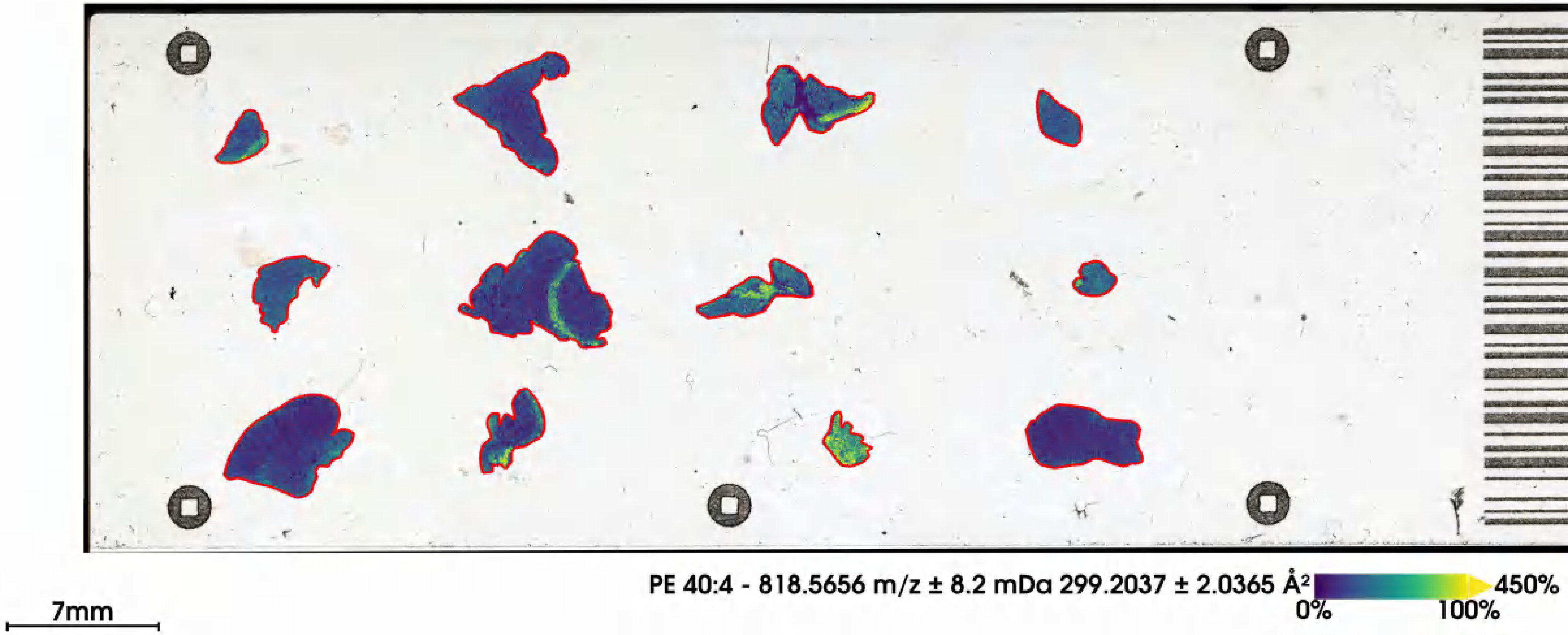
PE 40:4 - 818.5656 m/z ± 8.2 mDa 299.2037 ± 2.0365 Å²
0%
100%
450%
7mm

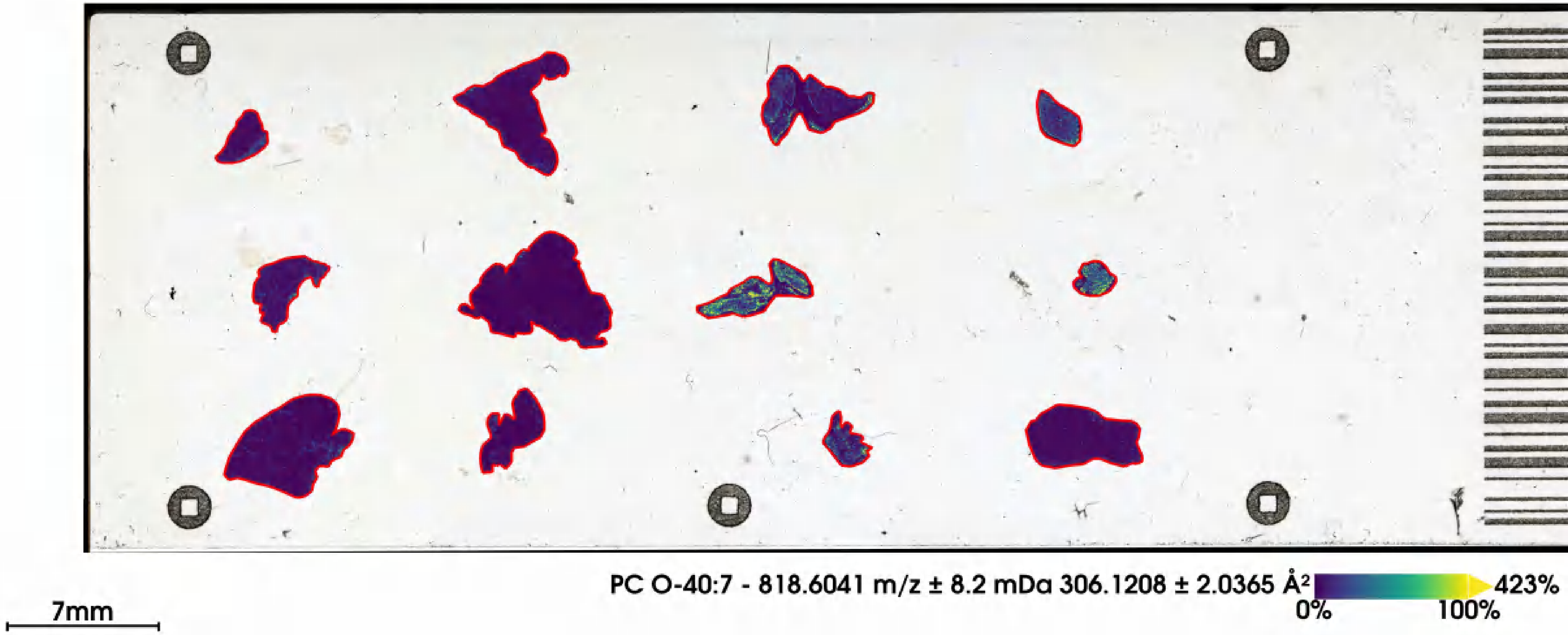
PC O-40:7 - 818.6041 m/z ± 8.2 mDa 306.1208 ± 2.0365 Å²
423%
0%
100%
7mm

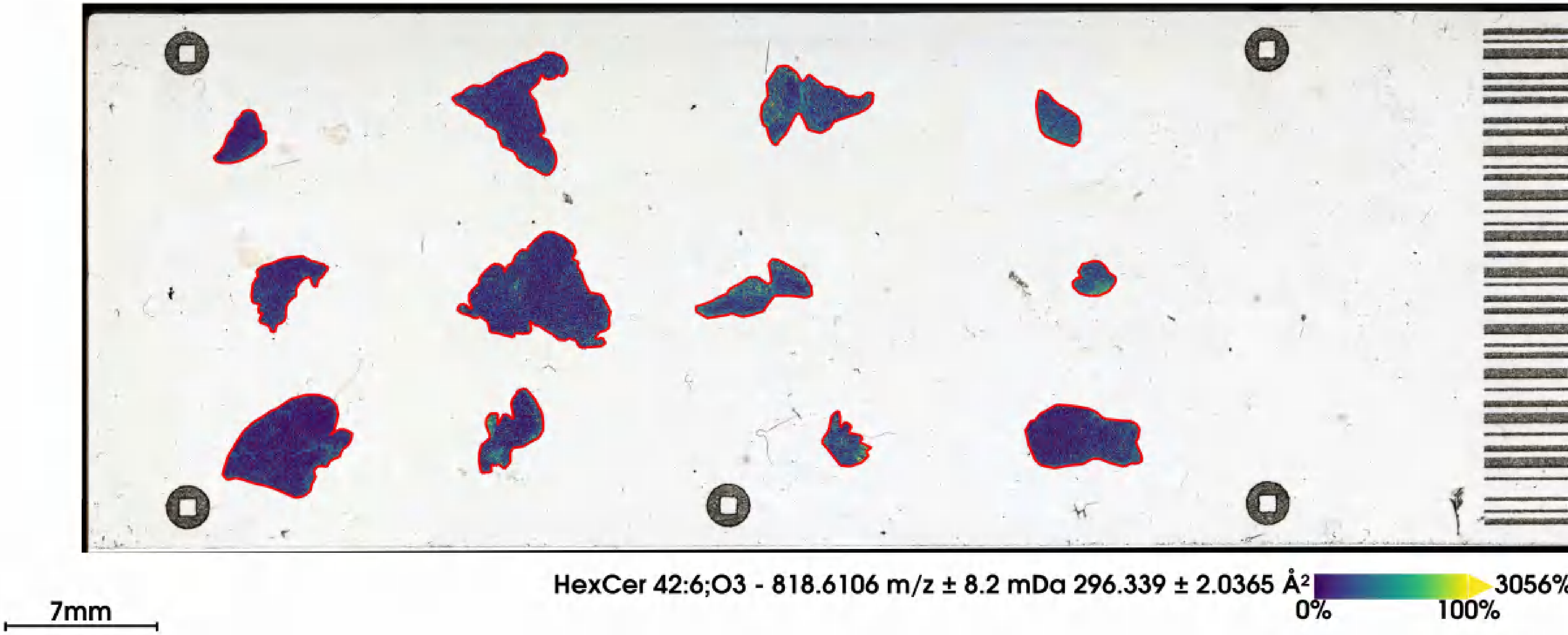

HexCer 42:6;O3 - 818.6106 m/z ± 8.2 mDa 296.339 ± 2.0365 Å²
0%
100%
3056%
7mm

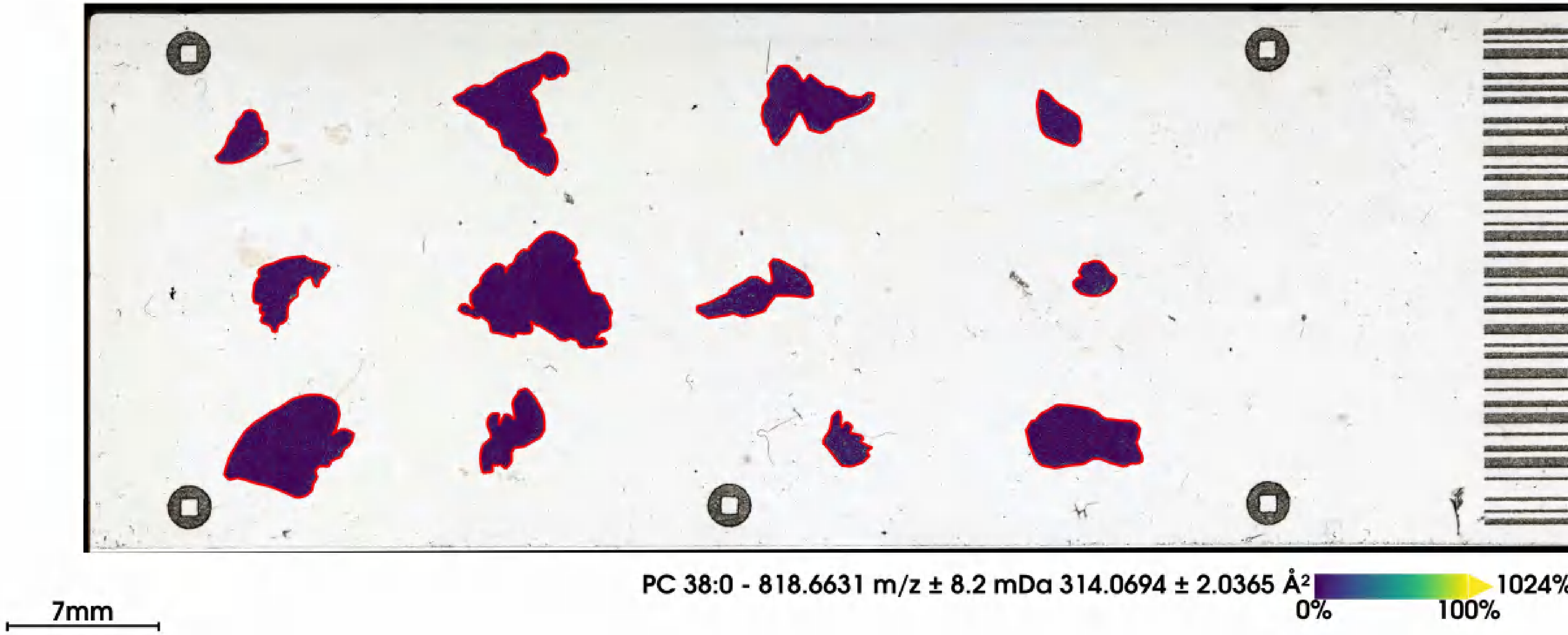
PC 38:0 - 818.6631 m/z ± 8.2 mDa 314.0694 ± 2.0365 Å²
0%
100%
1024%
7mm

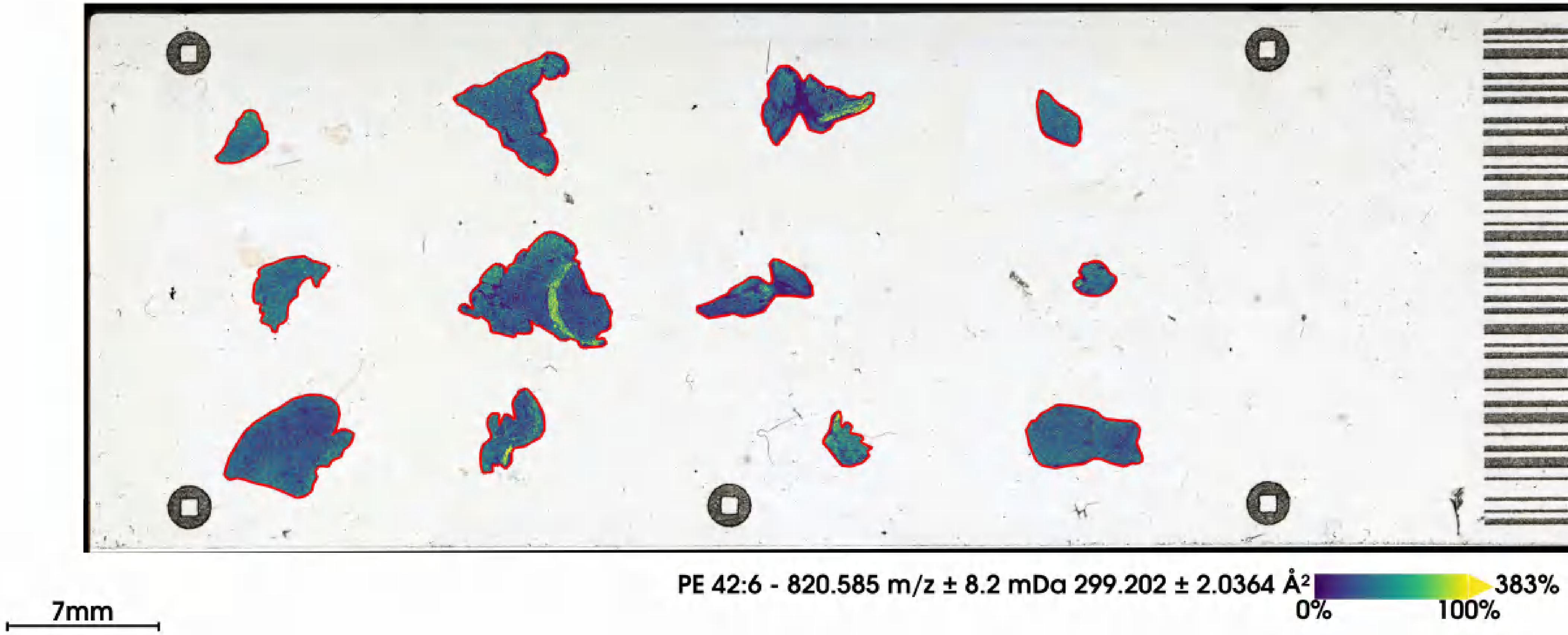

PE 42:6 - 820.585 m/z ± 8.2 mDa 299.202 ± 2.0364 Å²
0%
100%
383%
7mm

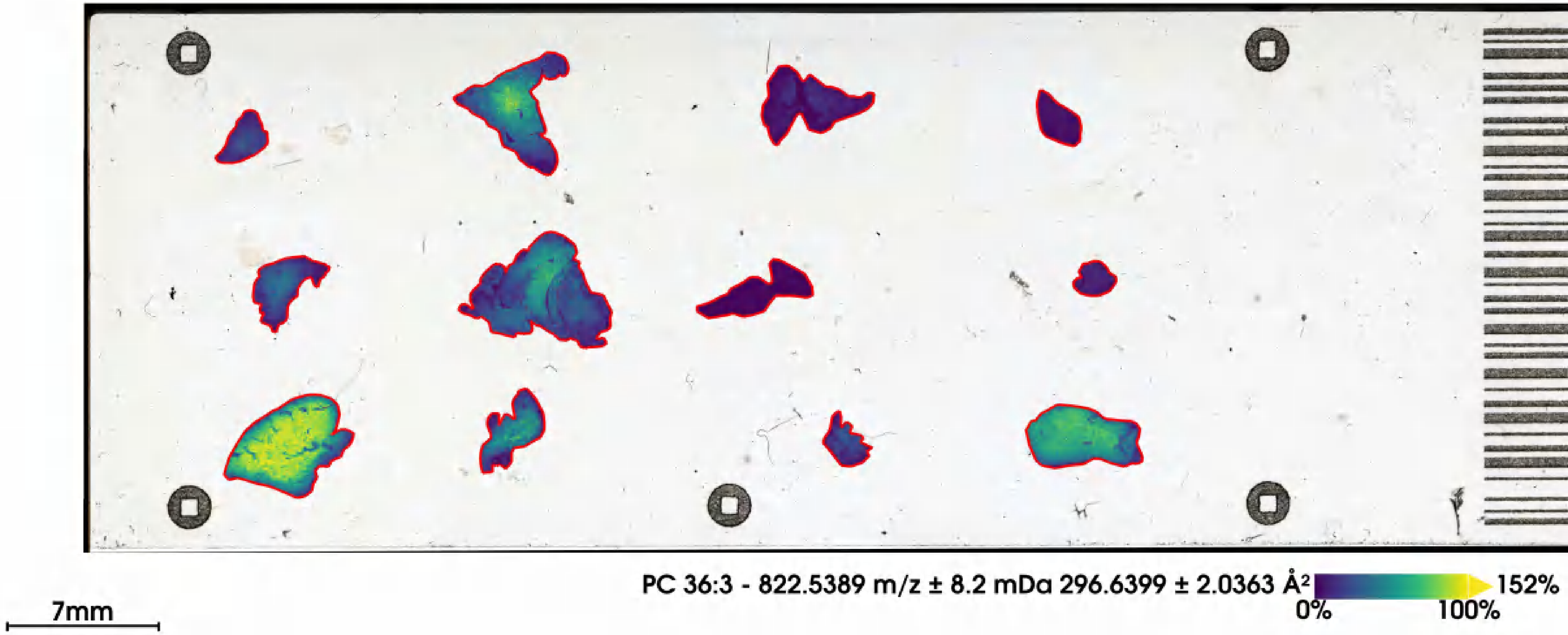
7mm
PC 36:3 - 822.5389 m/z ± 8.2 mDa 296.6399 ± 2.0363 Å²
152%
0%
100%

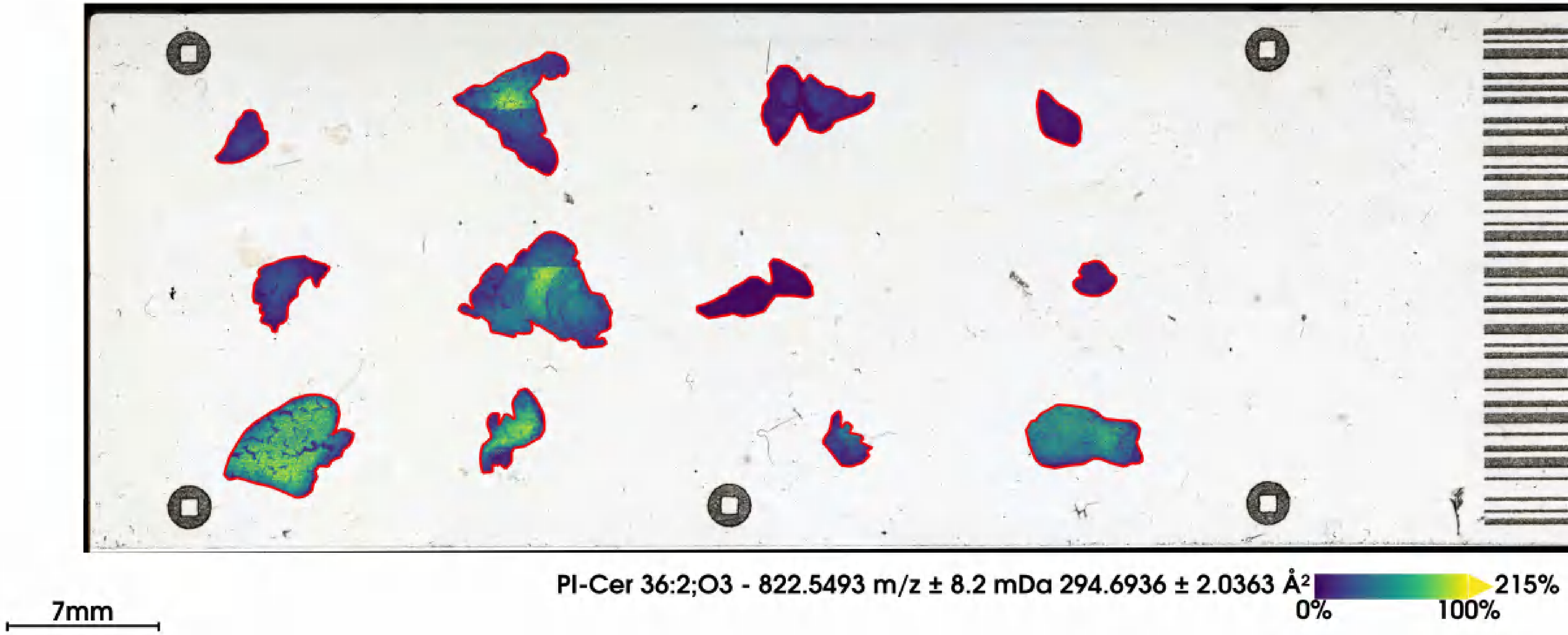

PI-Cer 36:2;O3 - 822.5493 m/z ± 8.2 mDa 294.6936 ± 2.0363 Å²
0%
100%
215%
7mm

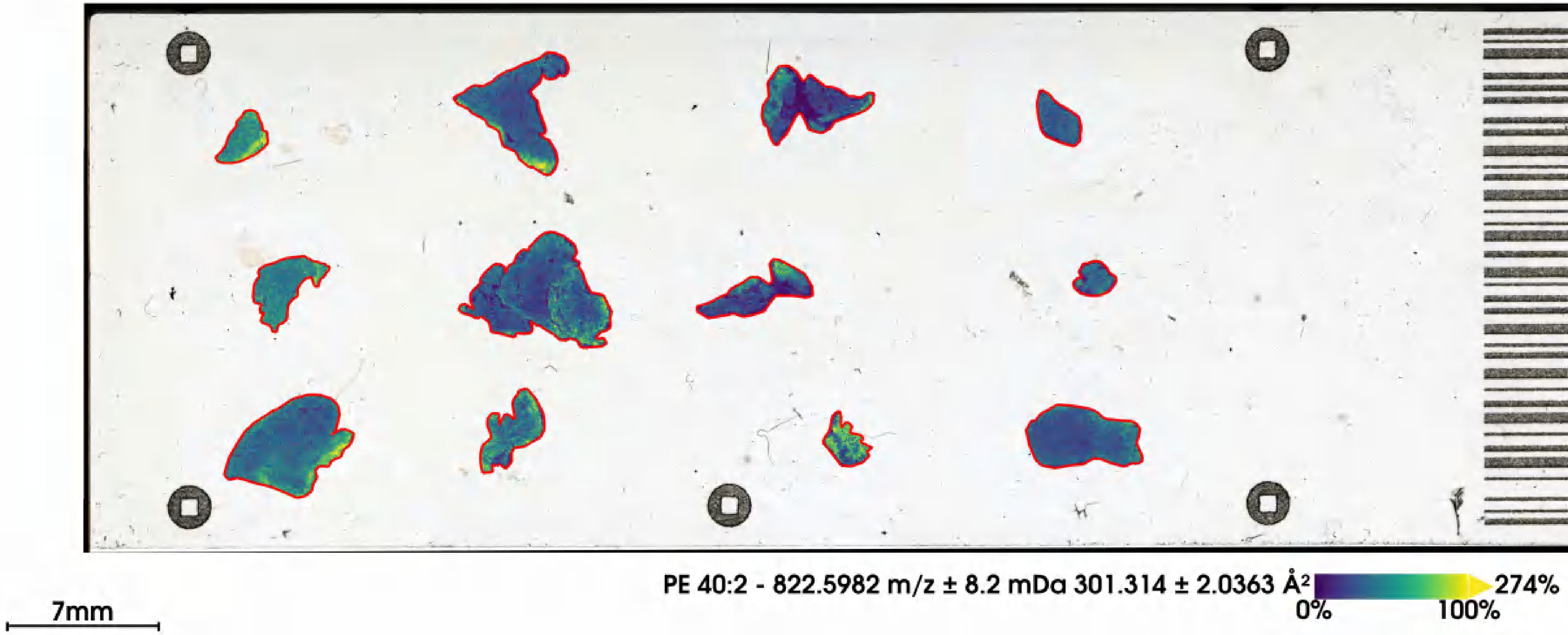
PE 40:2 - 822.5982 m/z ± 8.2 mDa 301.314 ± 2.0363 Å²
274%
0%
100%
7mm

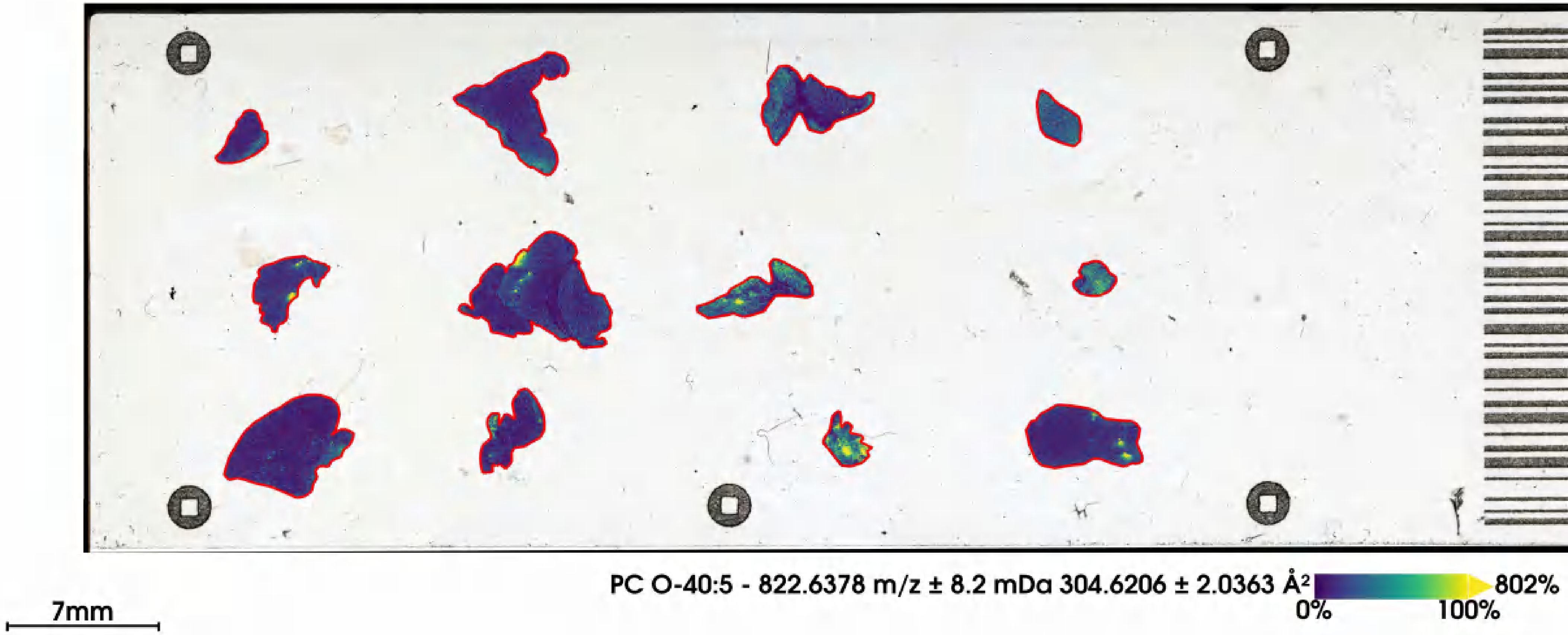
PC O-40:5 - 822.6378 m/z ± 8.2 mDa 304.6206 ± 2.0363 Å²
0%
100%
802%
7mm

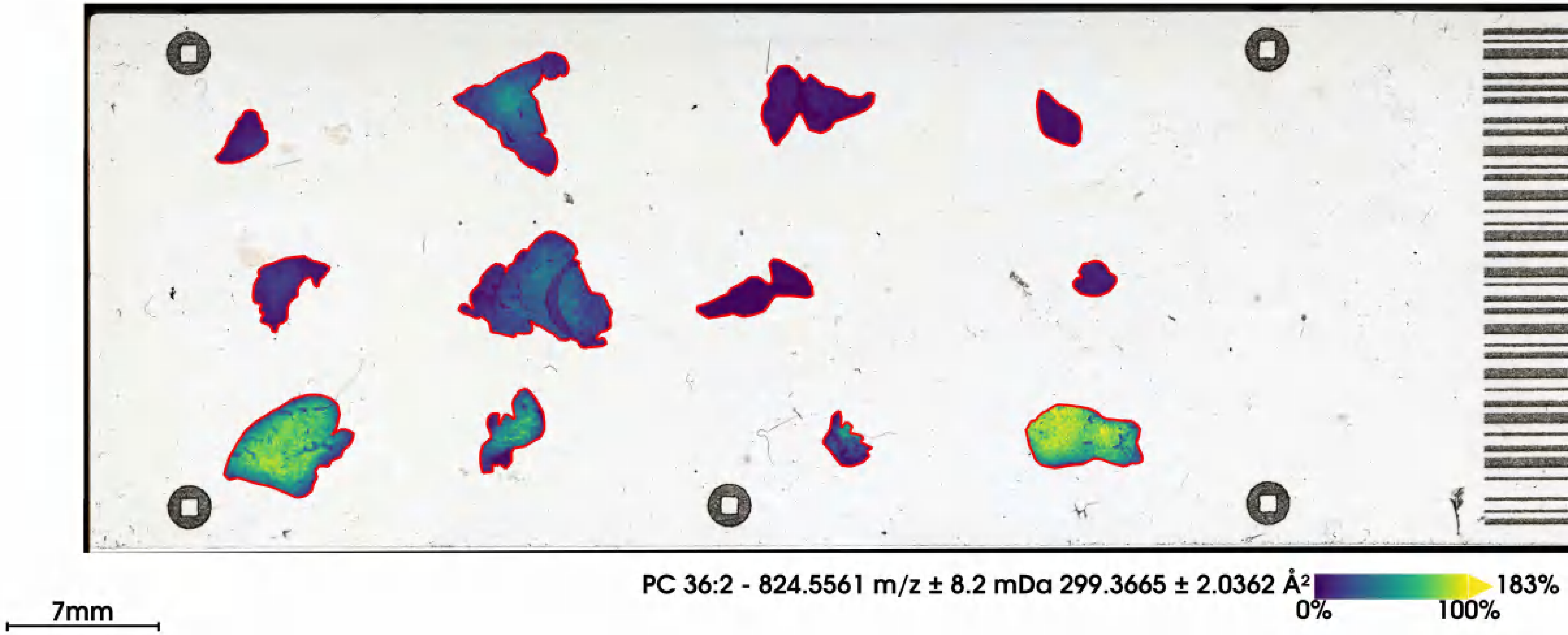
PC 36:2 - 824.5561 m/z ± 8.2 mDa 299.3665 ± 2.0362 Å²
0%
100%
183%
7mm

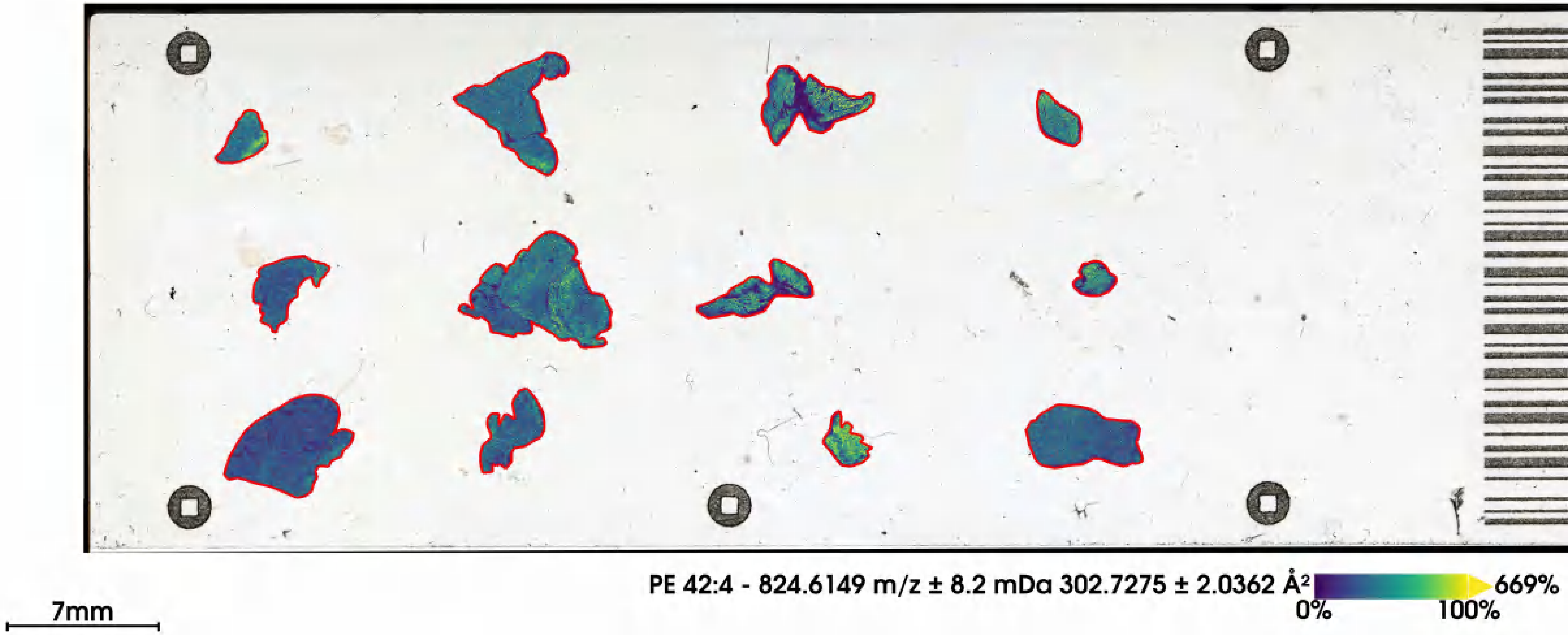
PE 42:4 - 824.6149 m/z ± 8.2 mDa 302.7275 ± 2.0362 Å²
0%
100%
669%
7mm

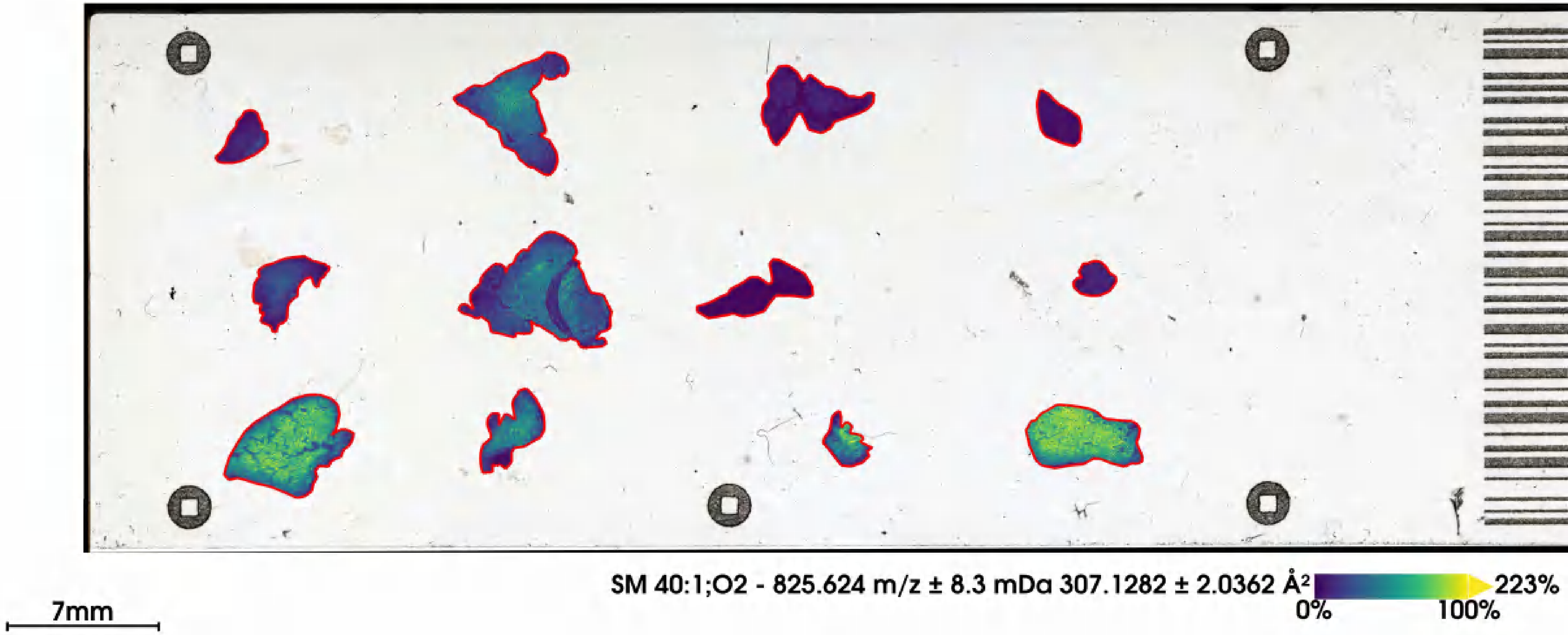

SM 40:1;O2 - 825.624 m/z ± 8.3 mDa 307.1282 ± 2.0362 Å²
0%
100%
223%
7mm

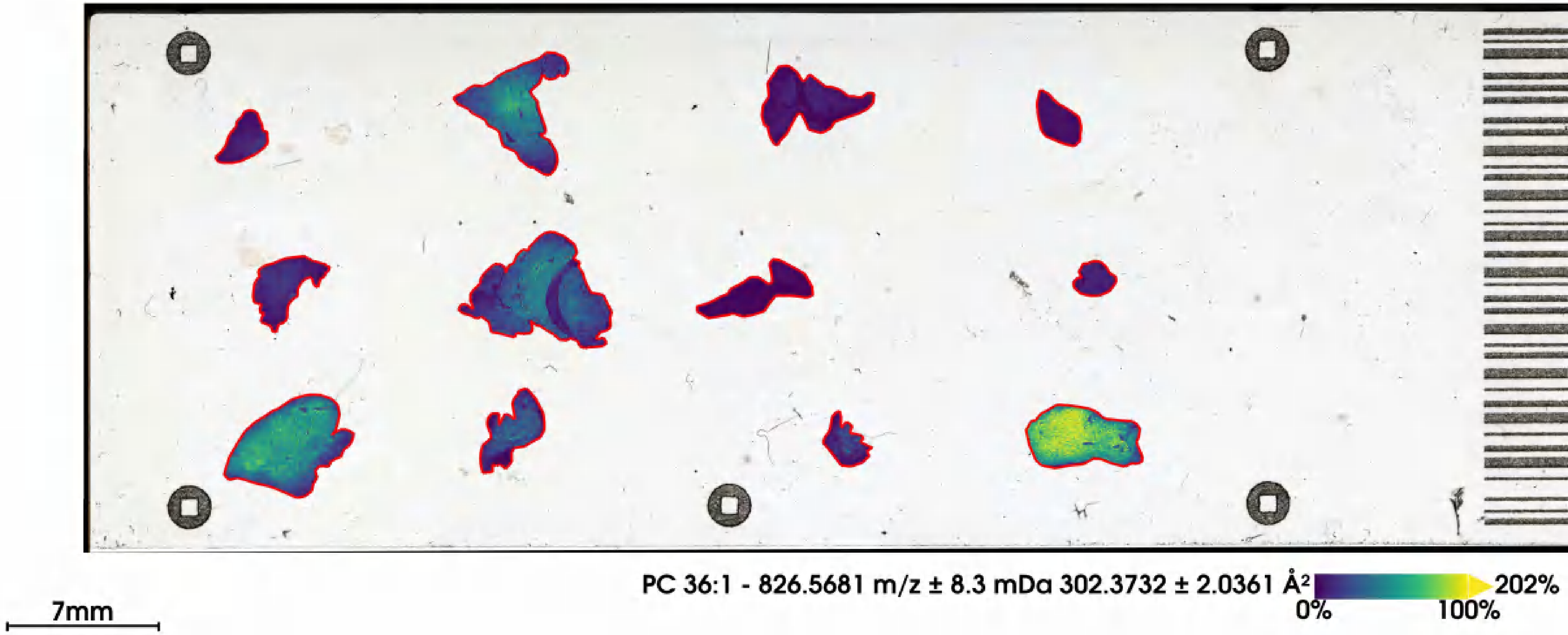
PC 36:1 - 826.5681 m/z ± 8.3 mDa 302.3732 ± 2.0361 Å²
0%
100%
202%
7mm

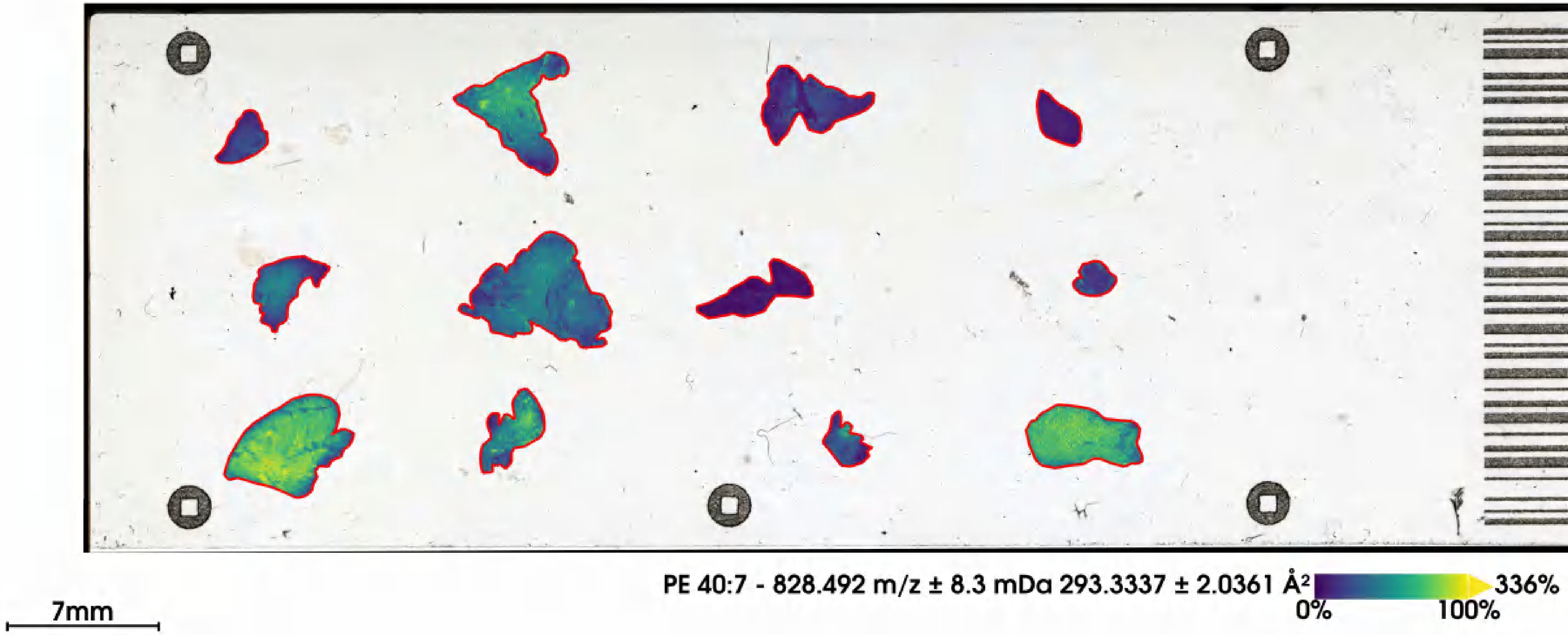
PE 40:7 - 828.492 m/z ± 8.3 mDa 293.3337 ± 2.0361 Å²
336%
0%
100%
7mm

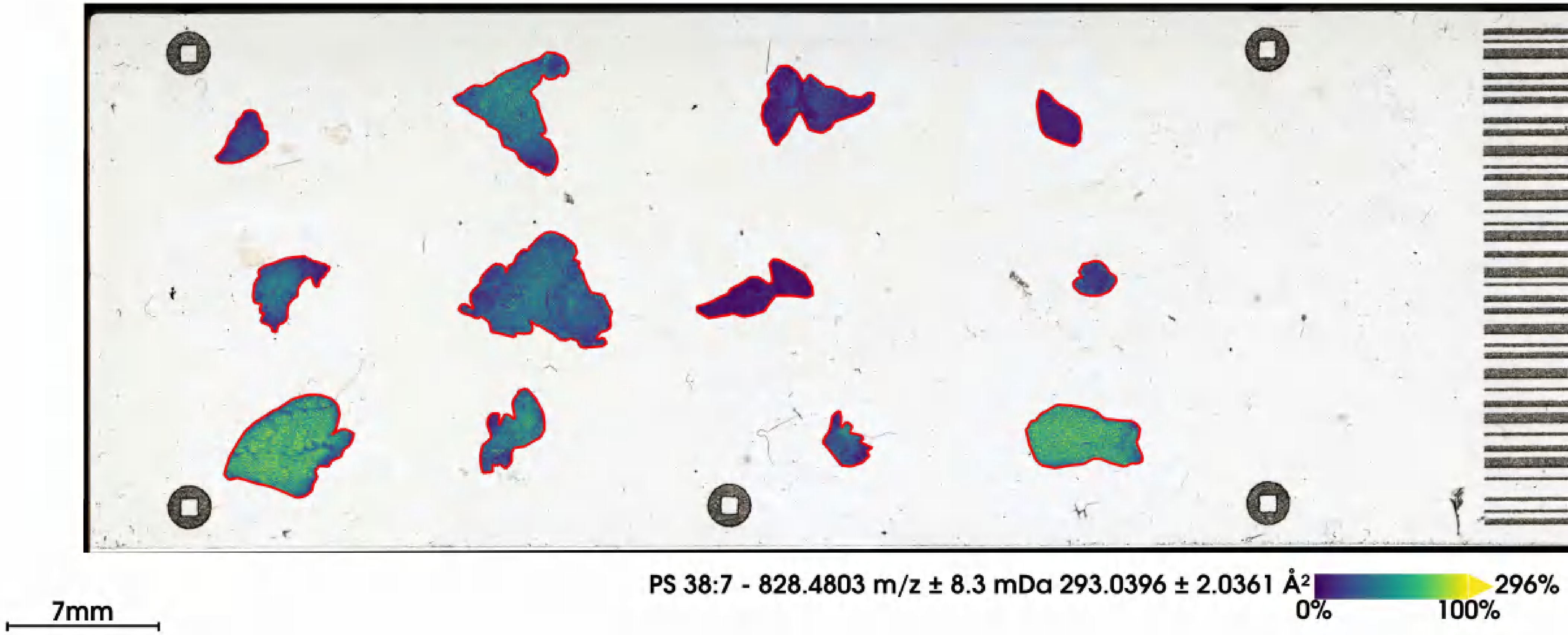
PS 38:7 - 828.4803 m/z ± 8.3 mDa 293.0396 ± 2.0361 Å²
296%
0%
100%
7mm

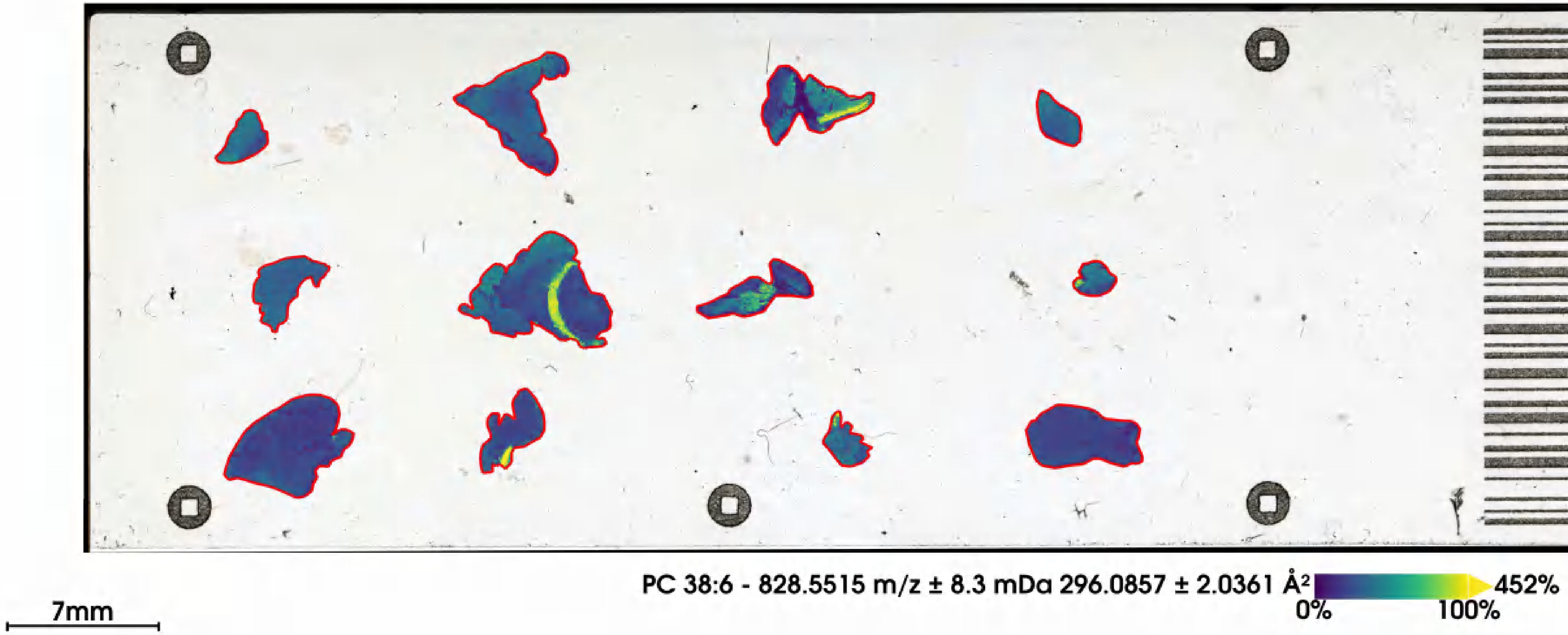
PC 38:6 - 828.5515 m/z ± 8.3 mDa 296.0857 ± 2.0361 Å²
0%
100%
452%
7mm

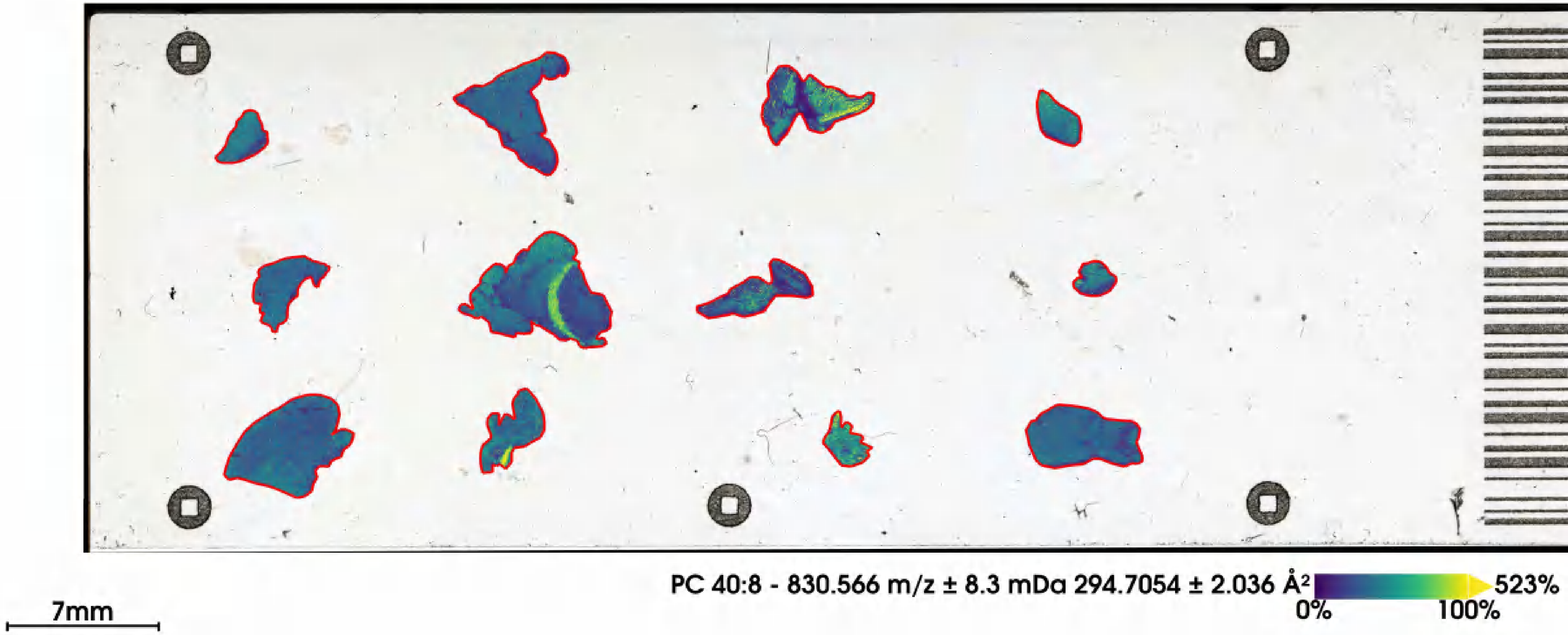
PC 40:8 - 830.566 m/z ± 8.3 mDa 294.7054 ± 2.036 Å²
0%
100%
523%
7mm

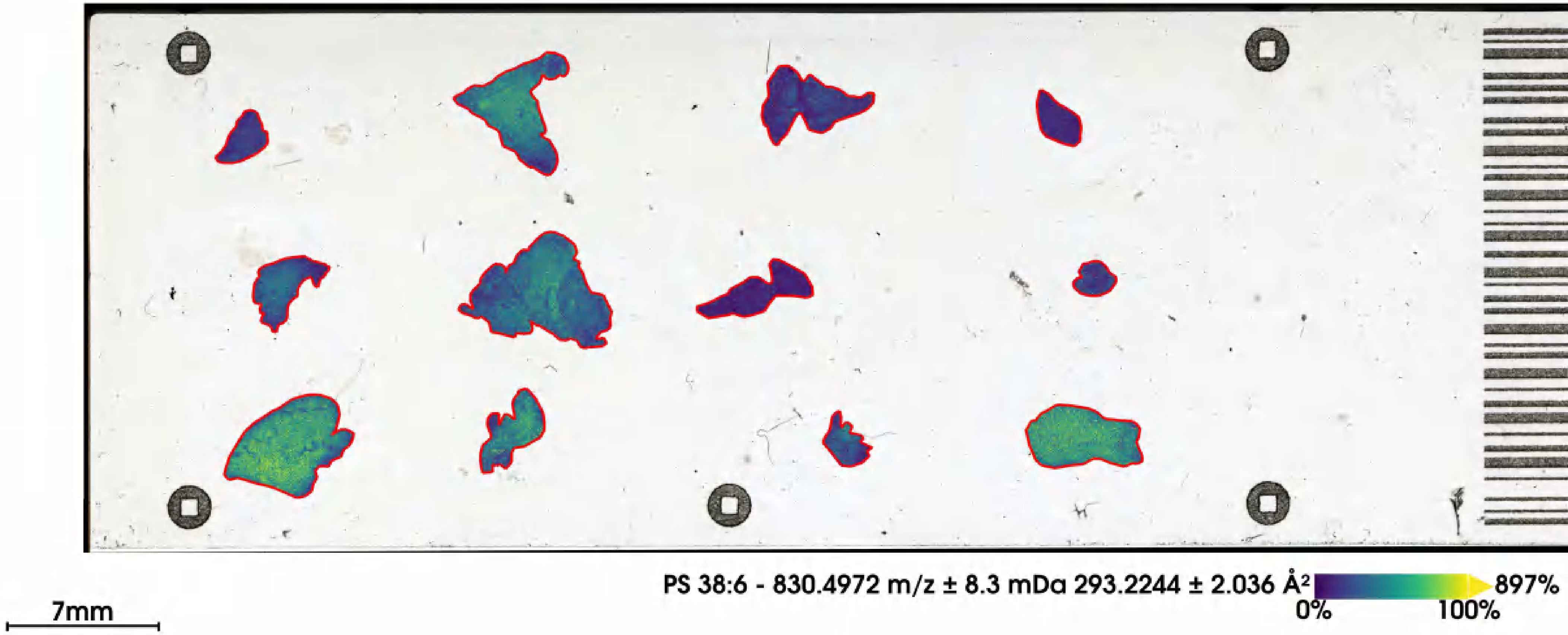

PS 38:6 - 830.4972 m/z ± 8.3 mDa 293.2244 ± 2.036 Å²
897%
0%
100%
7mm

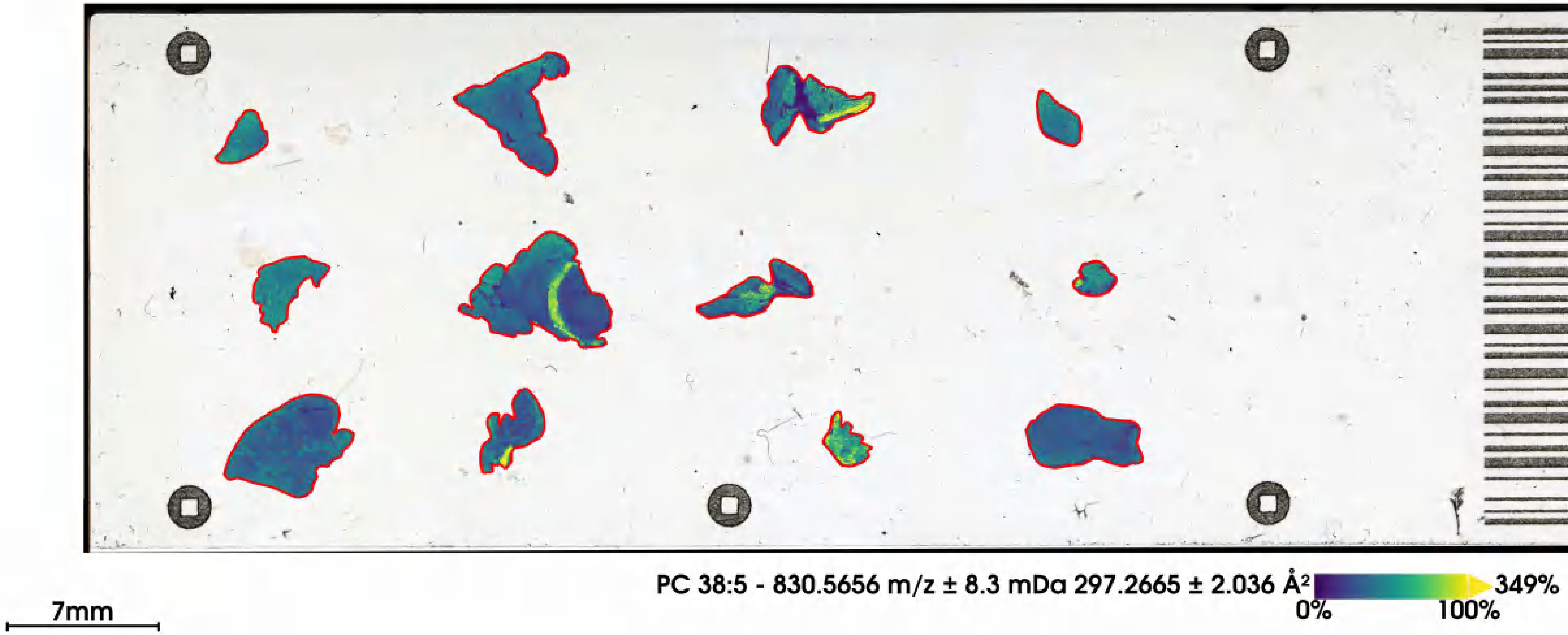
PC 38:5 - 830.5656 m/z ± 8.3 mDa 297.2665 ± 2.036 Å²
0%
100%
349%
7mm

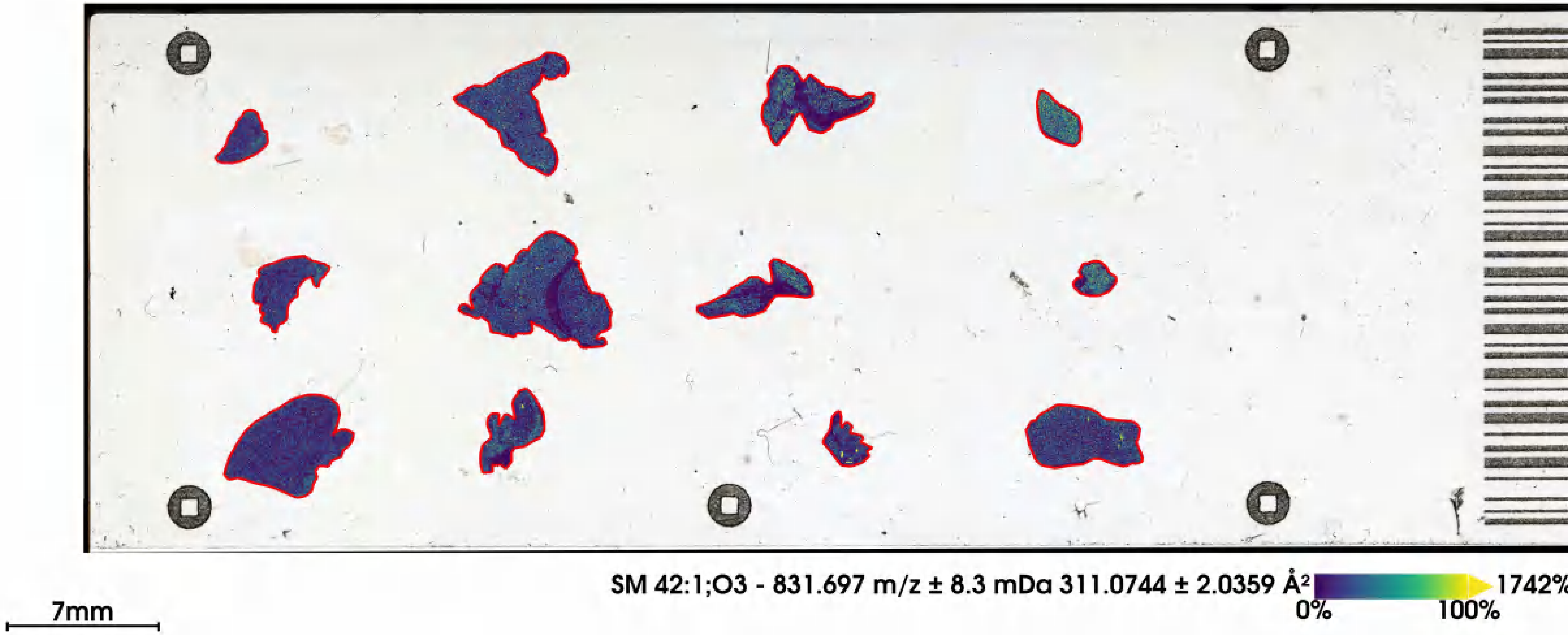
SM 42:1;O3 - 831.697 m/z ± 8.3 mDa 311.0744 ± 2.0359 Å²
0%
100%
1742%
7mm

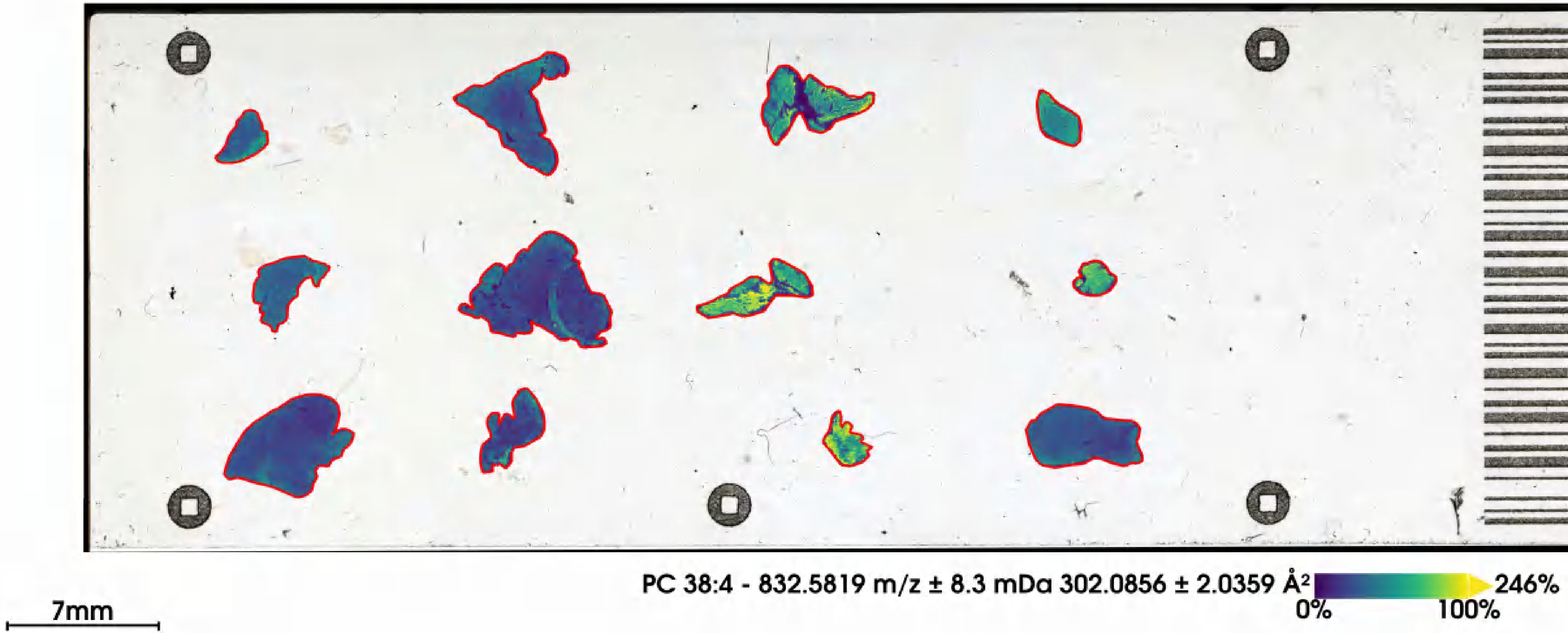
PC 38:4 - 832.5819 m/z ± 8.3 mDa 302.0856 ± 2.0359 Å²
0%
100%
246%
7mm

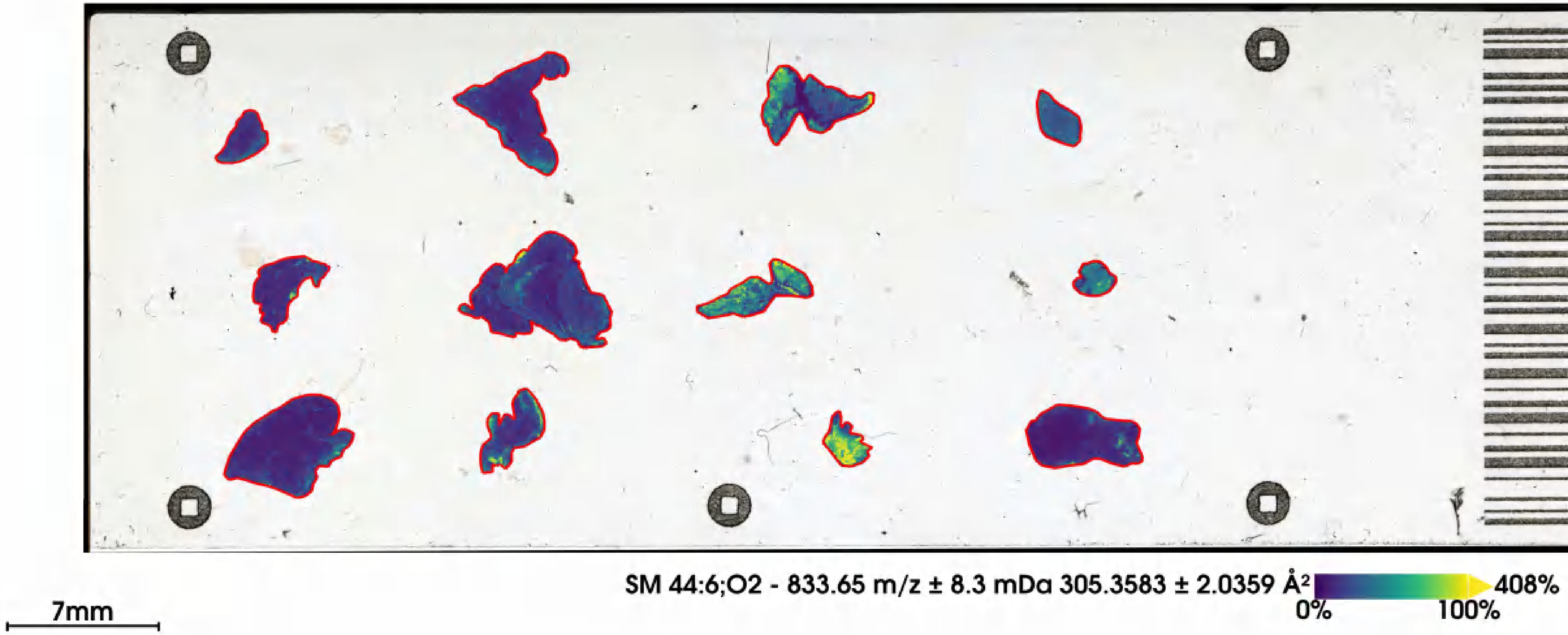

SM 44:6;O2 - 833.65 m/z ± 8.3 mDa 305.3583 ± 2.0359 Å²
0%
100%
408%
7mm

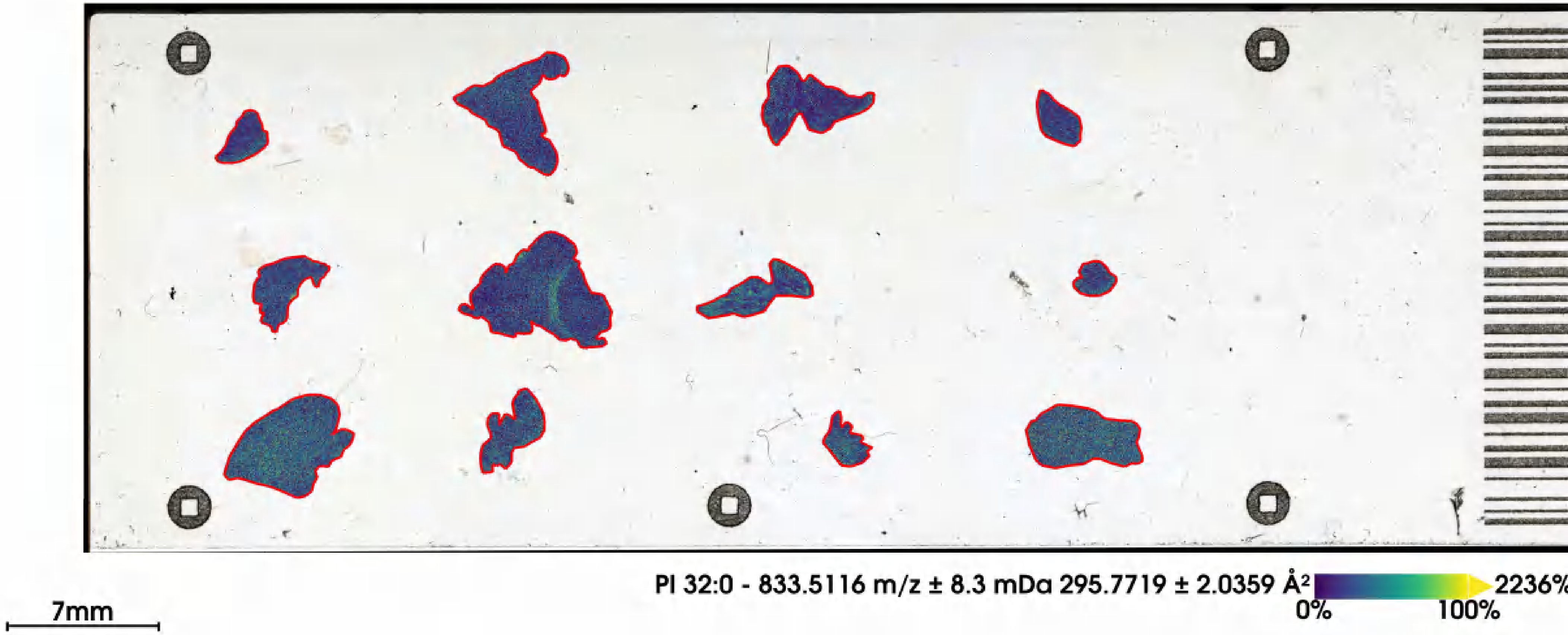
PI 32:0 - 833.5116 m/z ± 8.3 mDa 295.7719 ± 2.0359 Å²
2236%
0%
100%
7mm

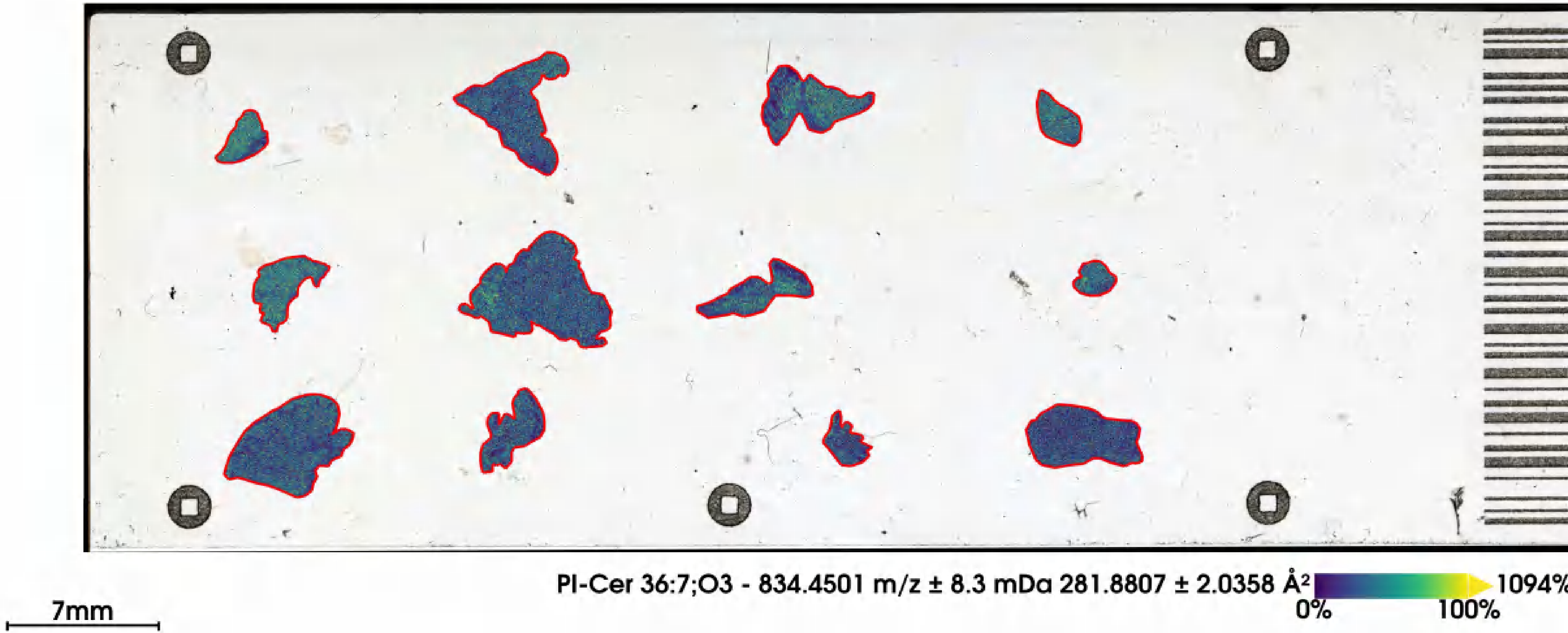
PI-Cer 36:7;O3 - 834.4501 m/z ± 8.3 mDa 281.8807 ± 2.0358 Å²
0%
100%
1094%
7mm

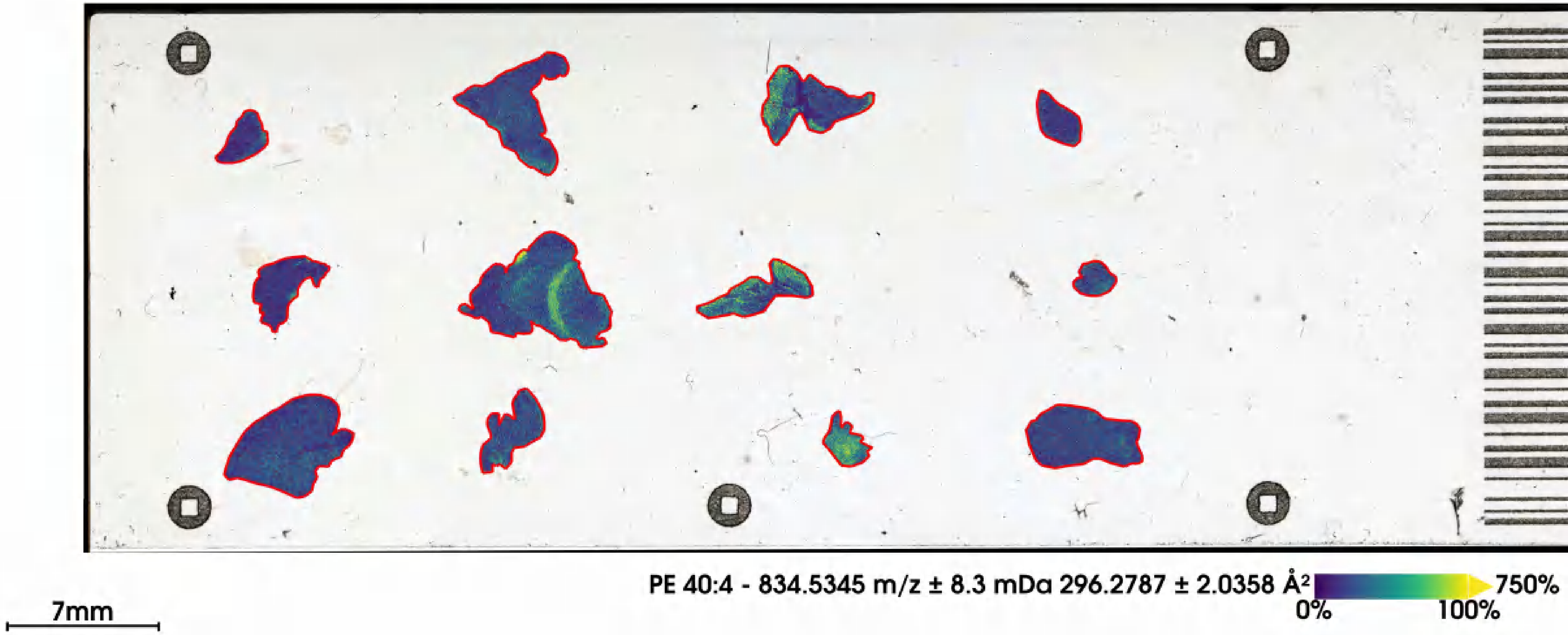
PE 40:4 - 834.5345 m/z ± 8.3 mDa 296.2787 ± 2.0358 Å²
750%
0%
100%
7mm

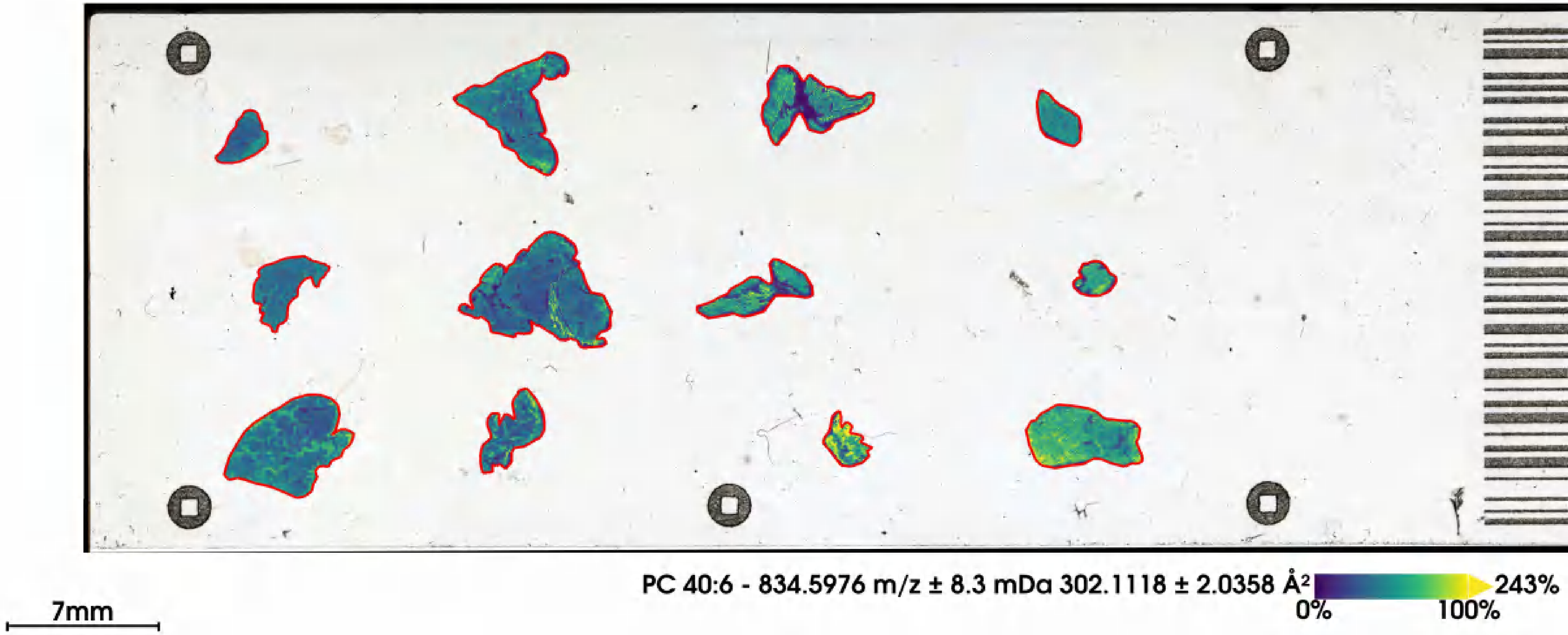
PC 40:6 - 834.5976 m/z ± 8.3 mDa 302.1118 ± 2.0358 Å²
0%
100%
243%
7mm

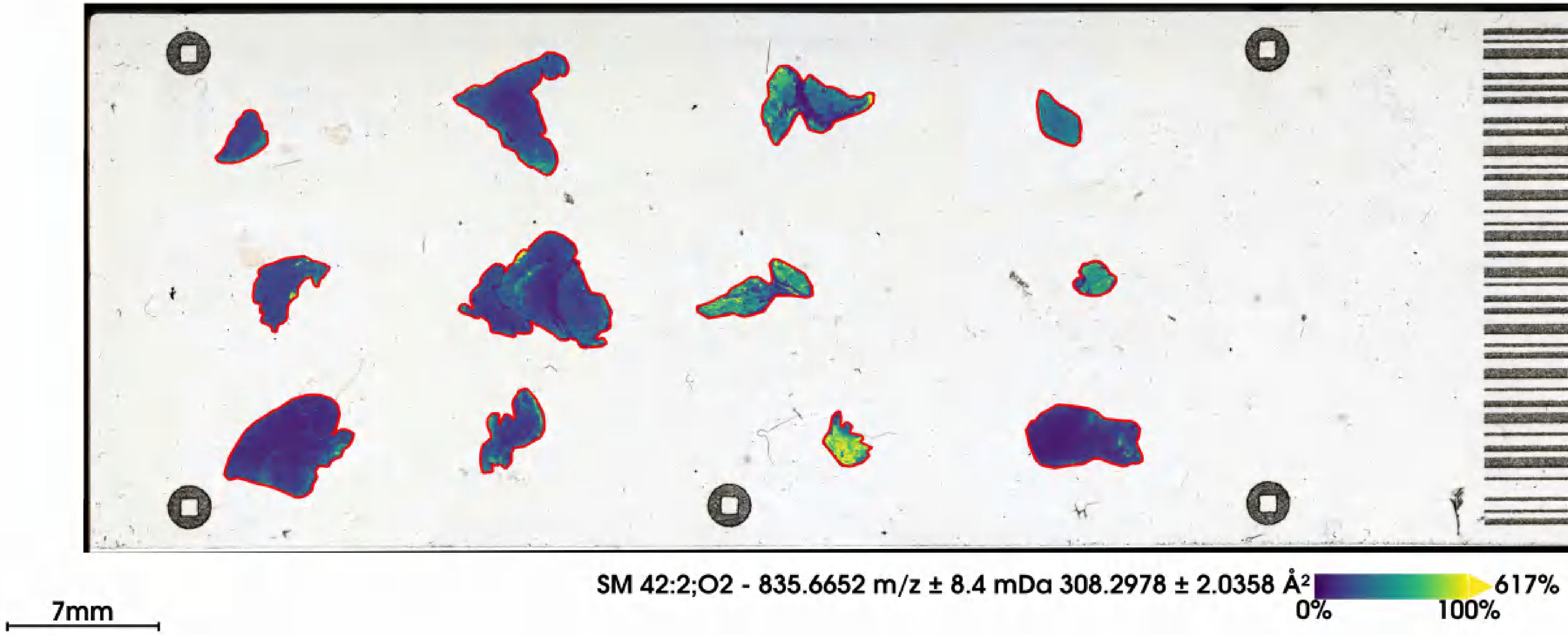
SM 42:2;O2 - 835.6652 m/z ± 8.4 mDa 308.2978 ± 2.0358 Å²
0%
100%
617%
7mm

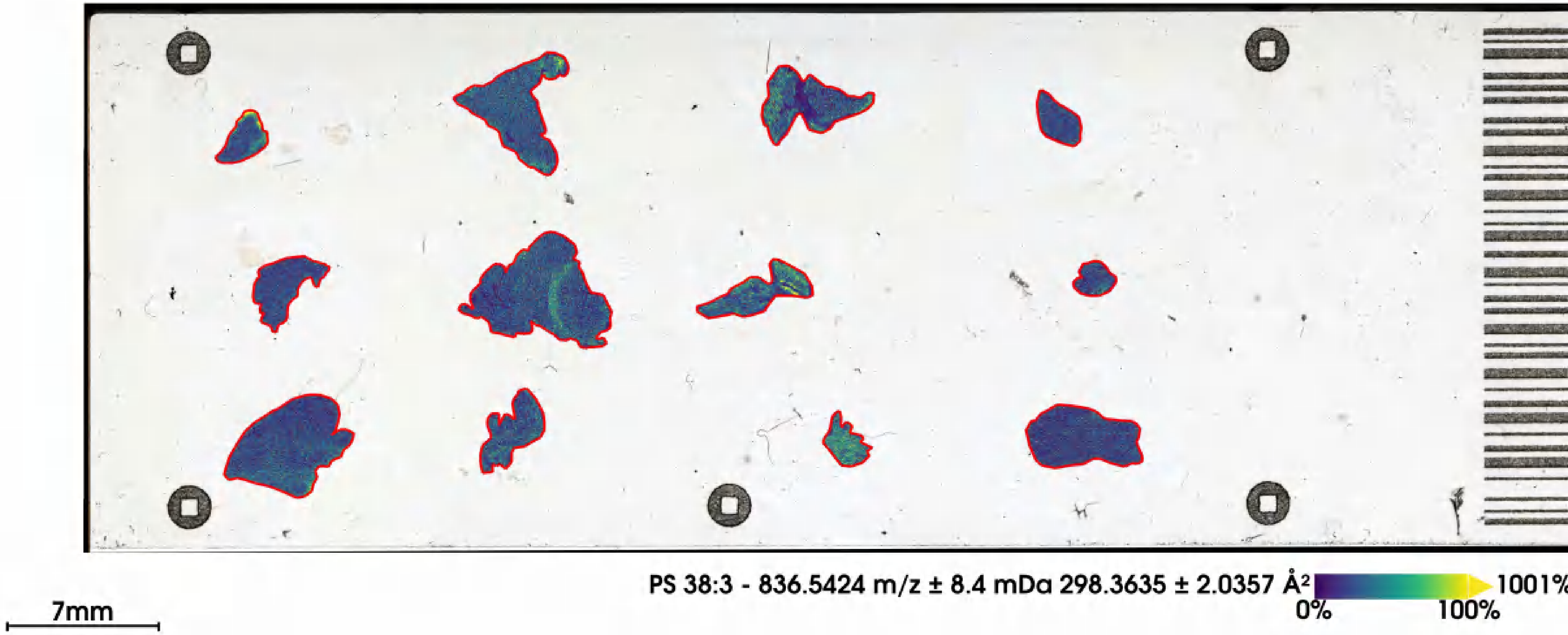
PS 38:3 - 836.5424 m/z ± 8.4 mDa 298.3635 ± 2.0357 Å²
0%
100%
1001%
7mm

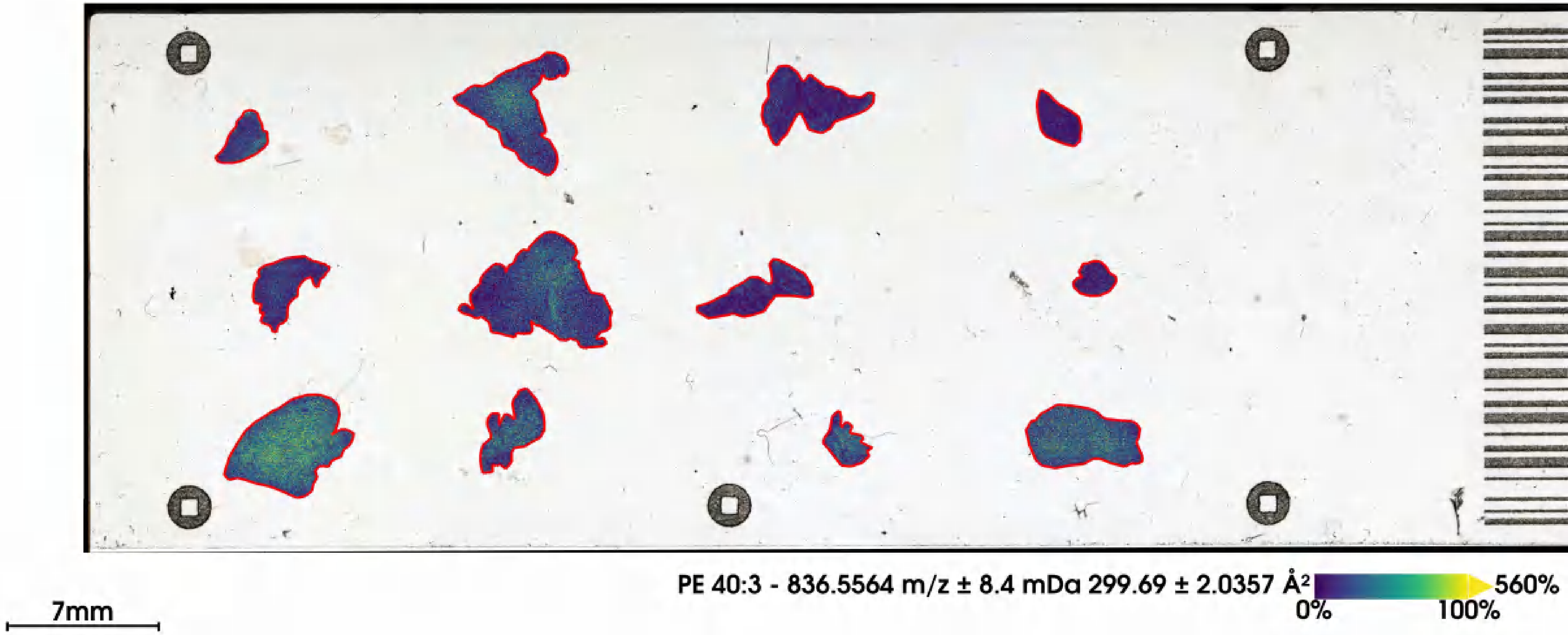
PE 40:3 - 836.5564 m/z ± 8.4 mDa 299.69 ± 2.0357 Å²
0%
100%
560%
7mm

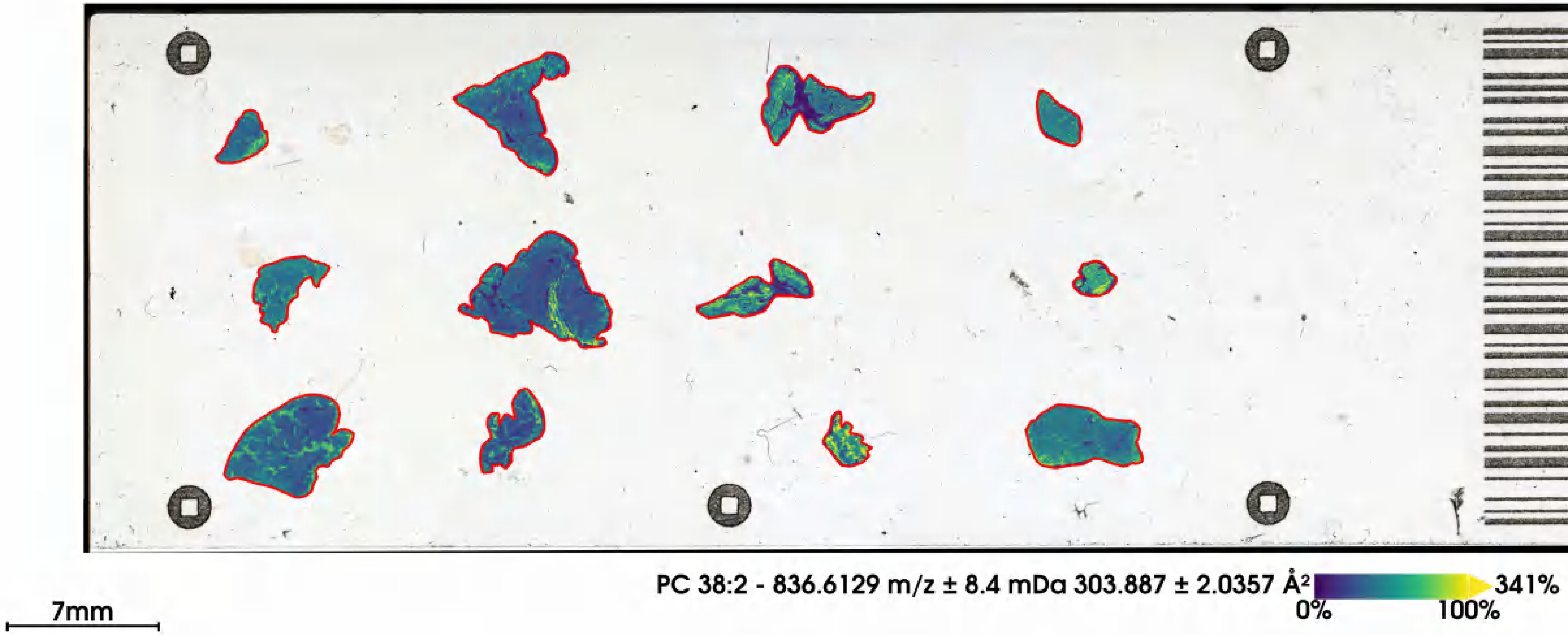
PC 38:2 - 836.6129 m/z ± 8.4 mDa 303.887 ± 2.0357 Å²
0%
100%
341%
7mm

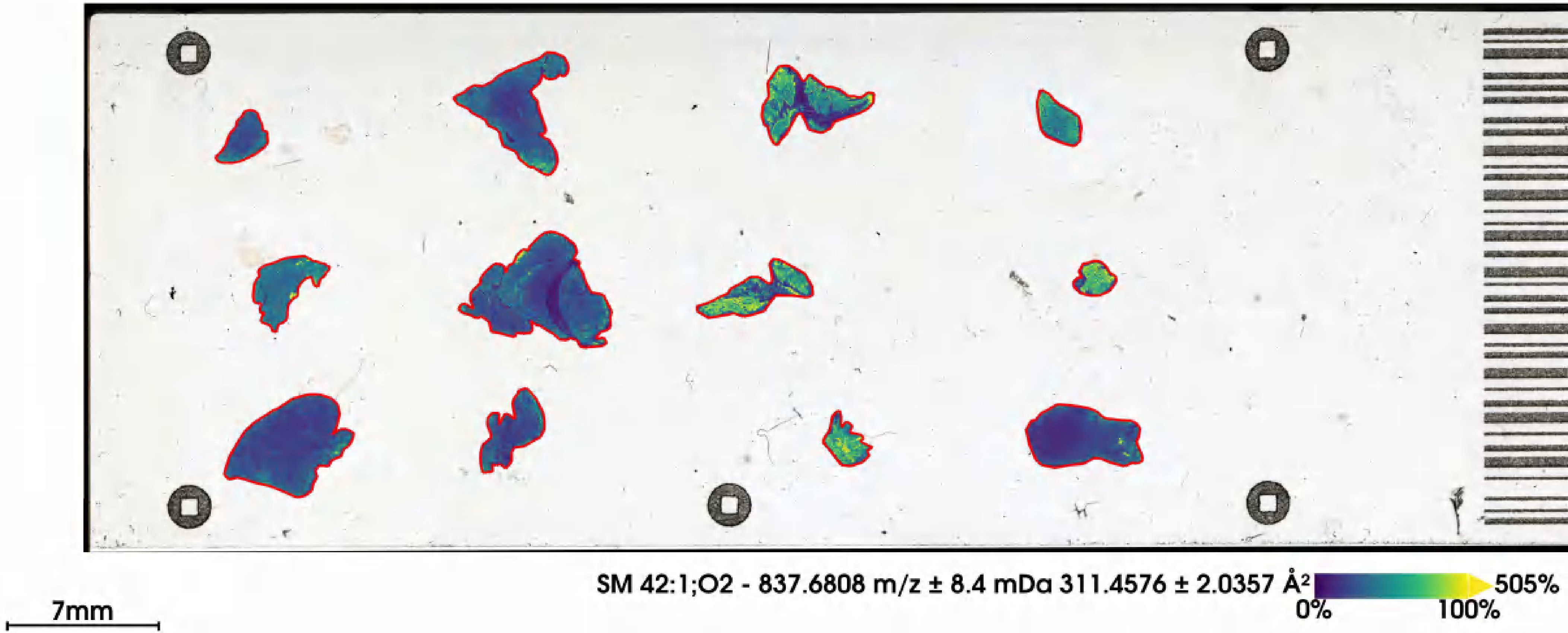

7mm
SM 42:1;O2 - 837.6808 m/z ± 8.4 mDa 311.4576 ± 2.0357 Å²
0%
100%
505%

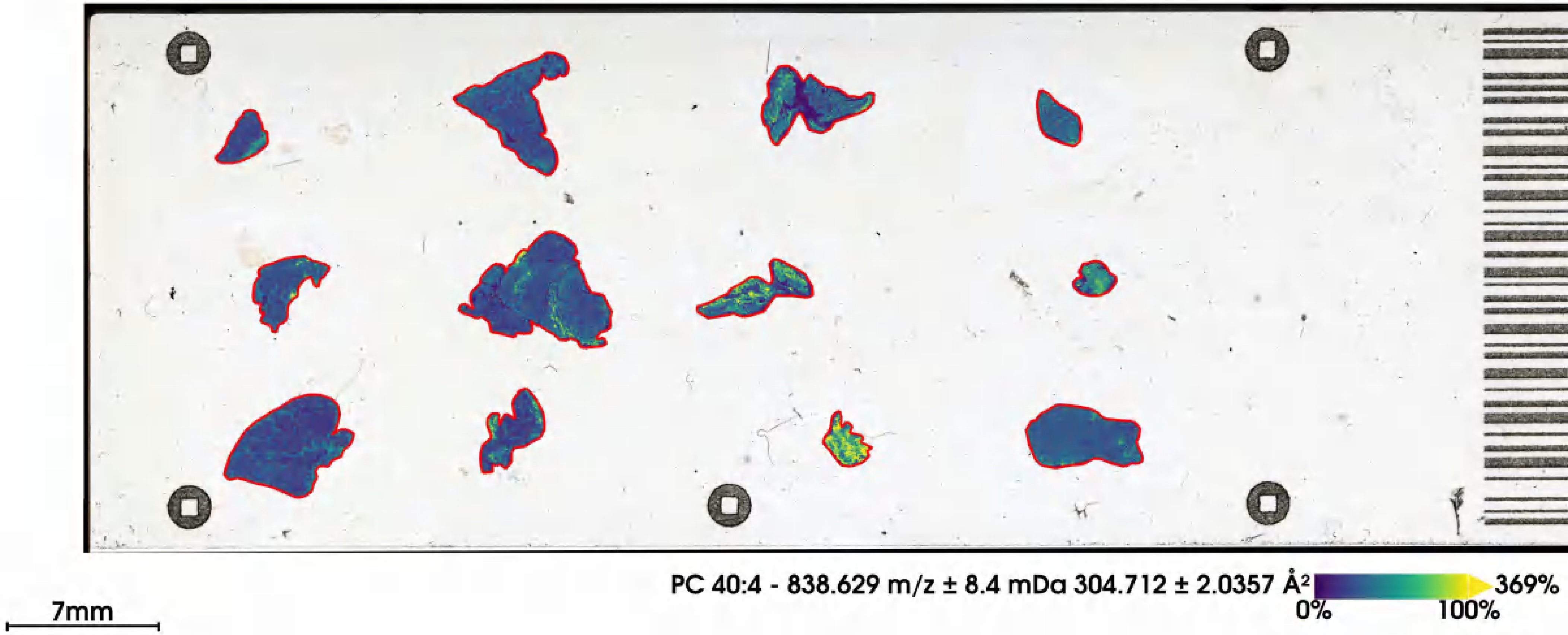
PC 40:4 - 838.629 m/z ± 8.4 mDa 304.712 ± 2.0357 Å²
369%
0%
100%
7mm

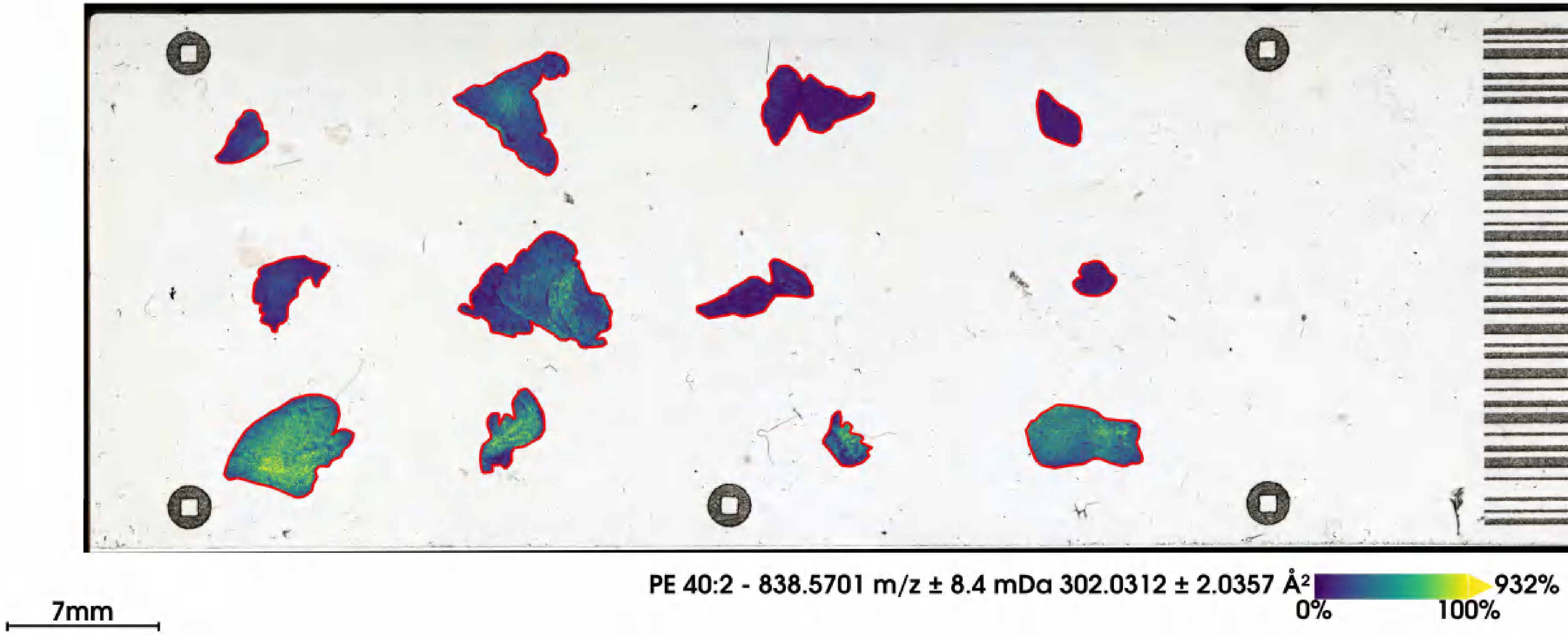

7mm
PE 40:2 - 838.5701 m/z ± 8.4 mDa 302.0312 ± 2.0357 Å²
0%
100%
932%

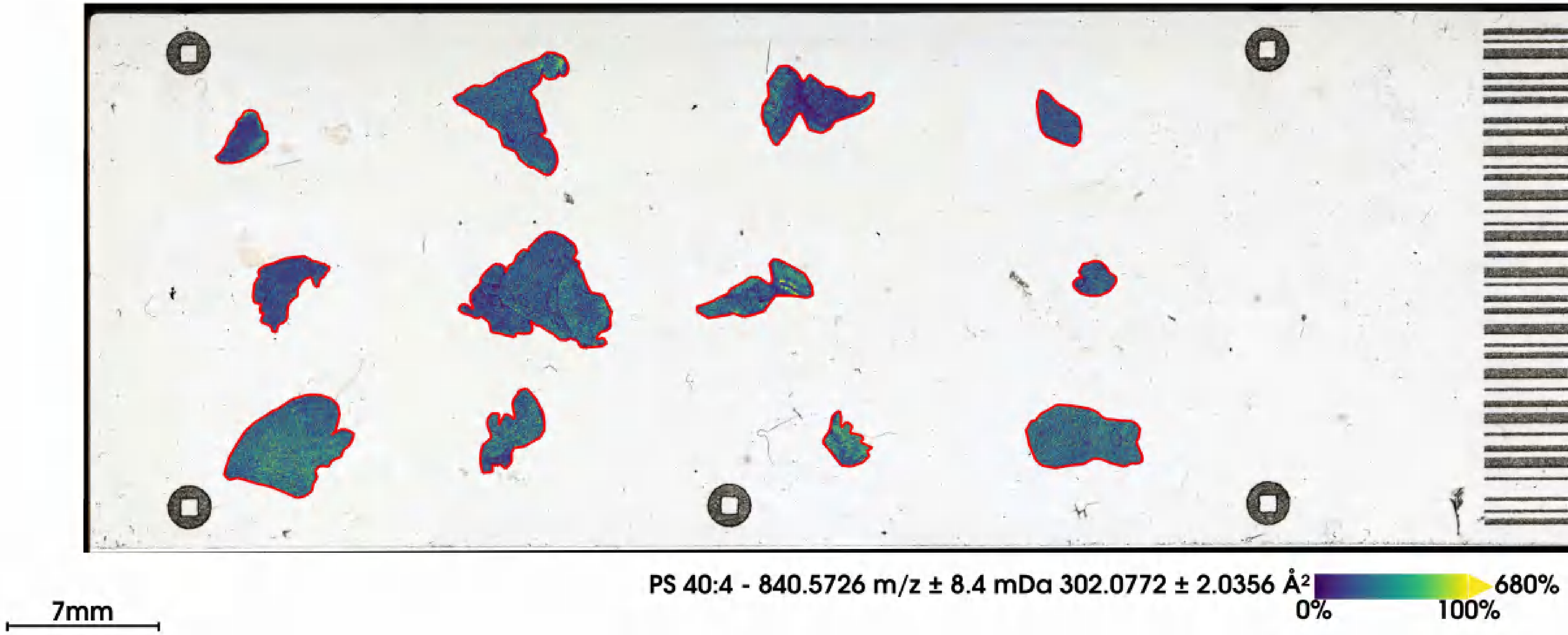
PS 40:4 - 840.5726 m/z ± 8.4 mDa 302.0772 ± 2.0356 Å²
0%
100%
680%
7mm

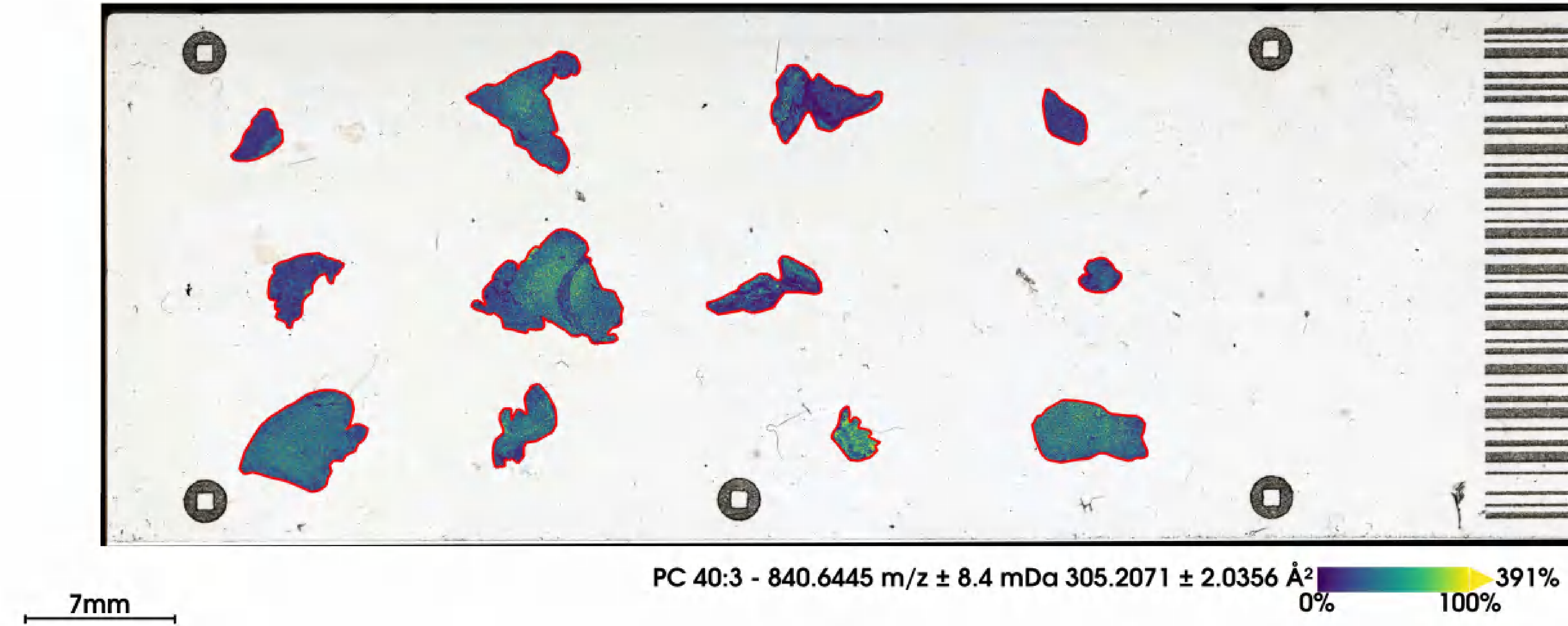
PC 40:3 - 840.6445 m/z ± 8.4 mDa 305.2071 ± 2.0356 Å²
0%
100%
391%
7mm

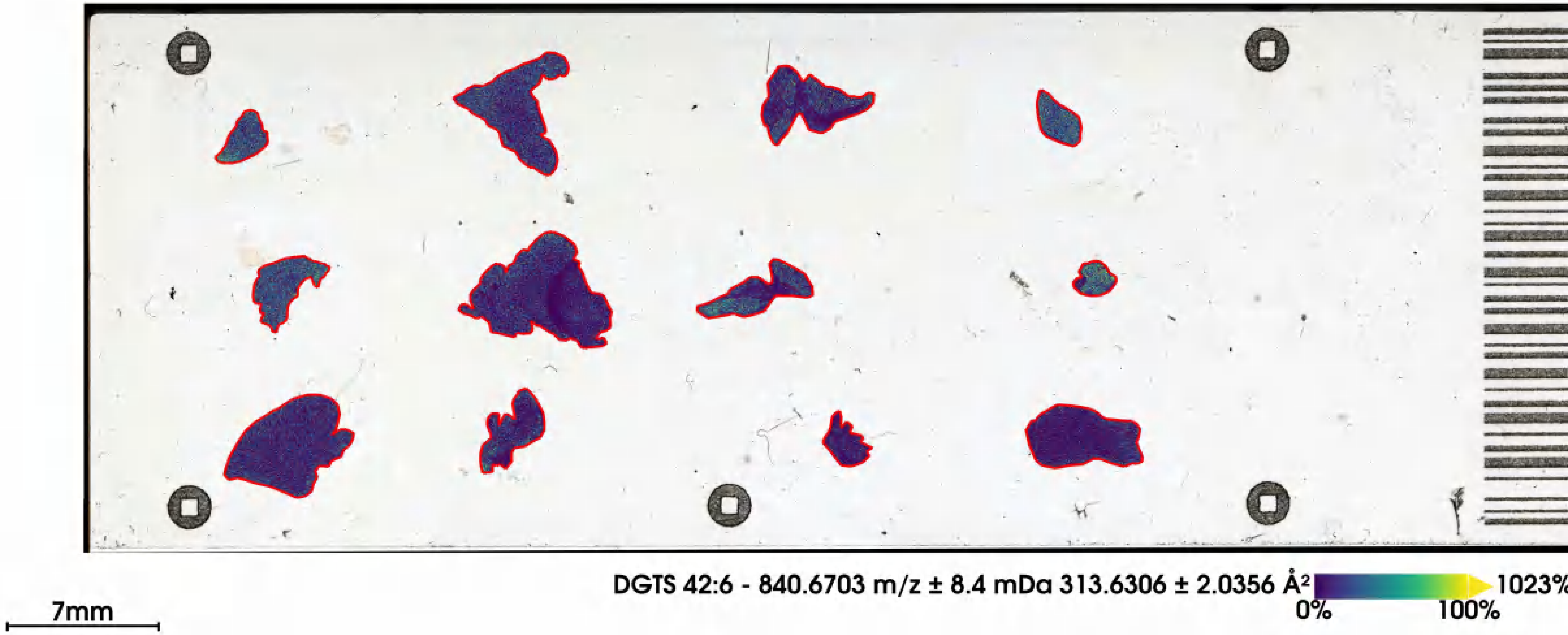

DGTS 42:6 - 840.6703 m/z ± 8.4 mDa 313.6306 ± 2.0356 Å²
1023%
0%
100%
7mm

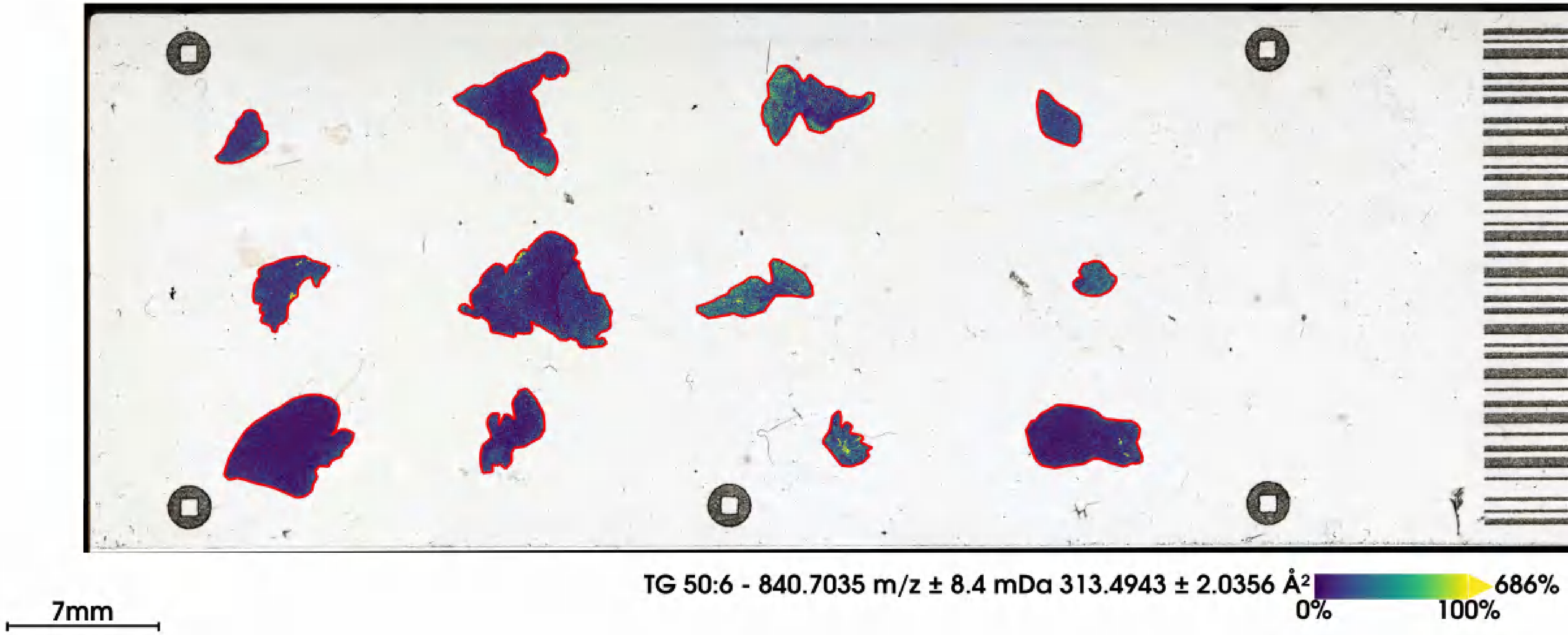
TG 50:6 - 840.7035 m/z ± 8.4 mDa 313.4943 ± 2.0356 Å²
0%
100%
686%
7mm

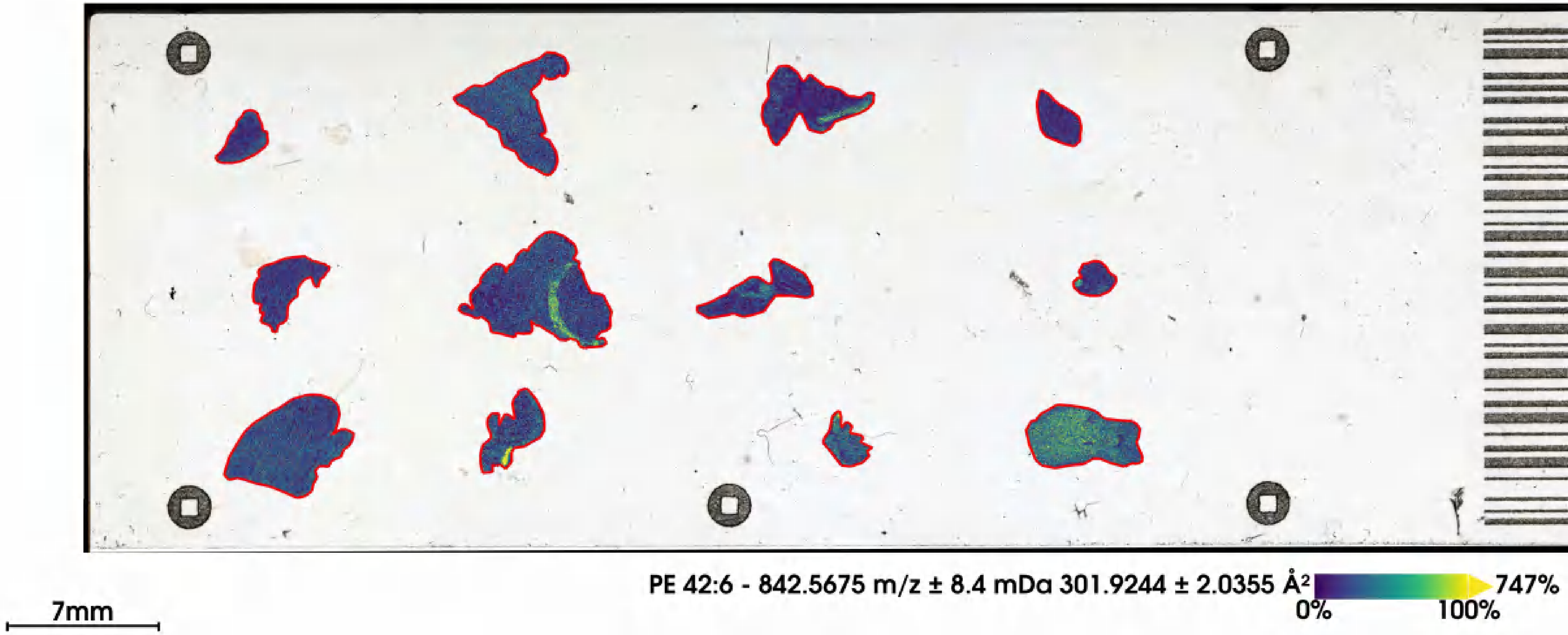
PE 42:6 - 842.5675 m/z ± 8.4 mDa 301.9244 ± 2.0355 Å²
0%
100%
747%
7mm

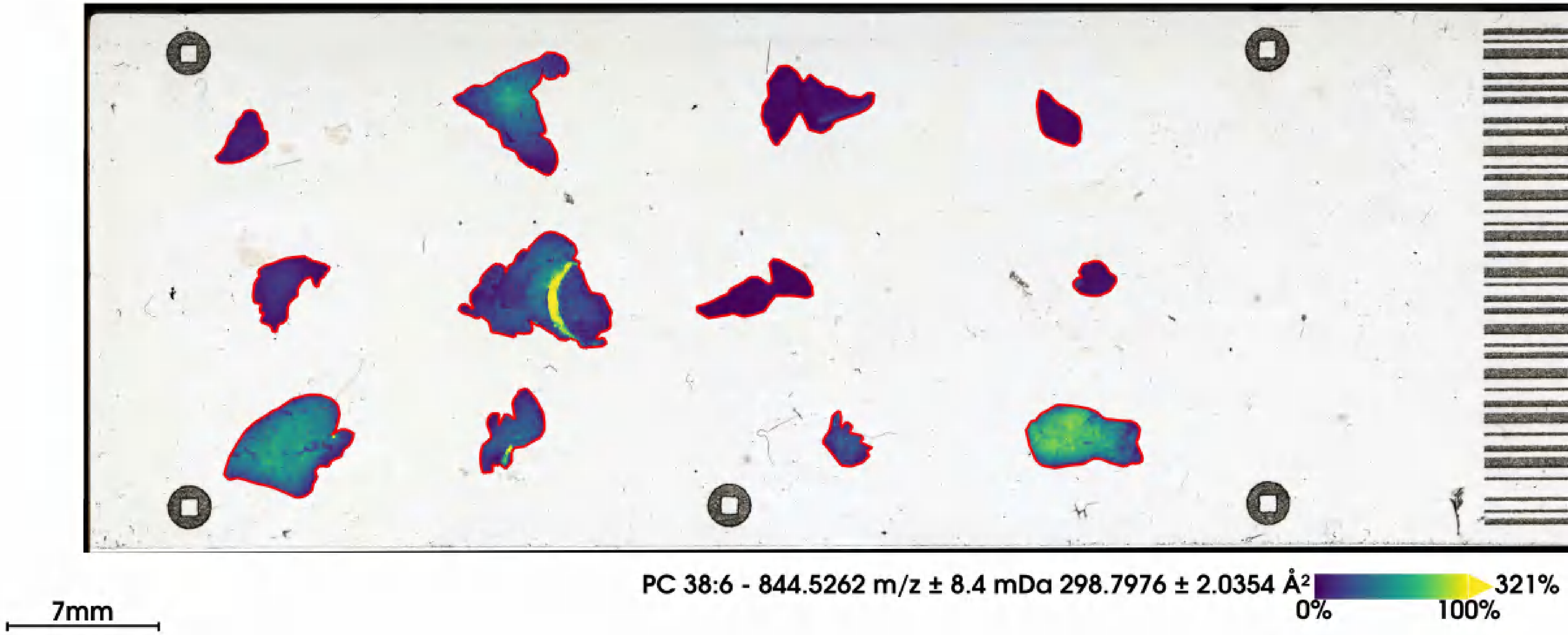

PC 38:6 - 844.5262 m/z ± 8.4 mDa 298.7976 ± 2.0354 Å²
0%
100%
321%
7mm

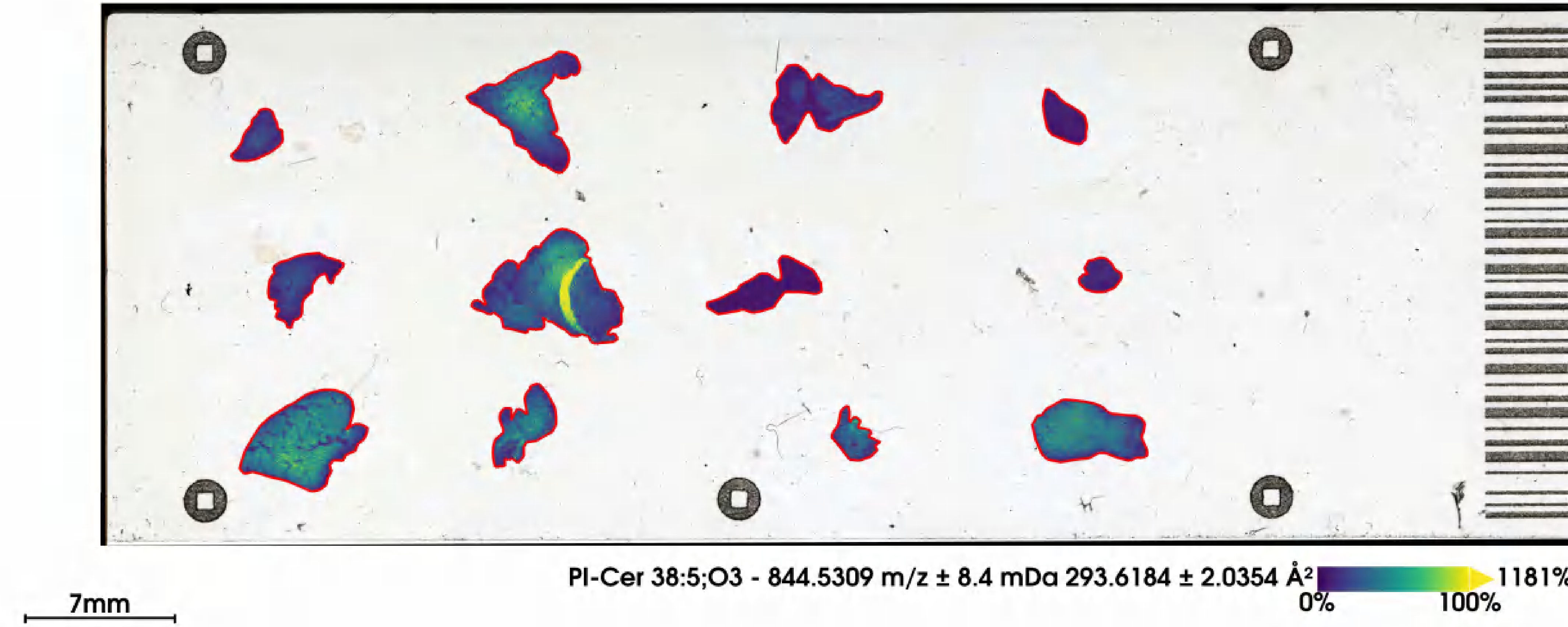
PI-Cer 38:5;O3 - 844.5309 m/z ± 8.4 mDa 293.6184 ± 2.0354 Å²
1181%
0%
100%
7mm

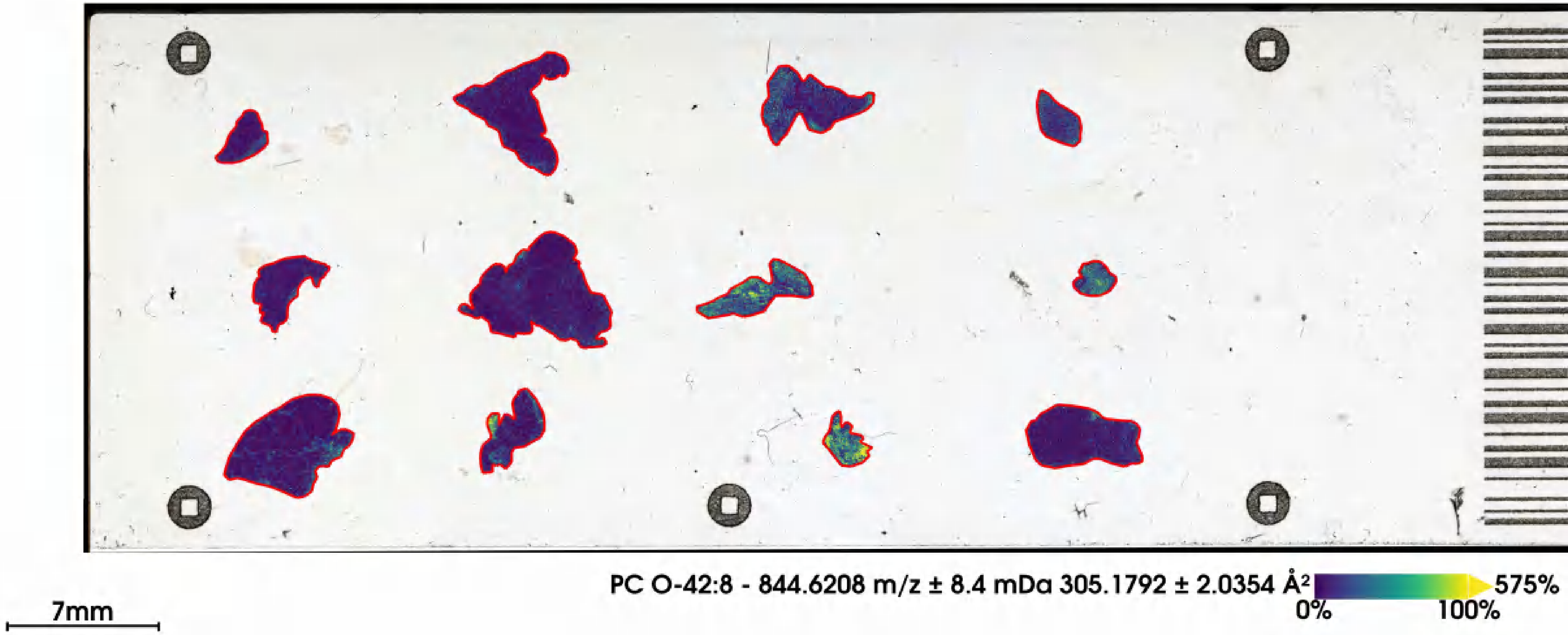
PC O-42:8 - 844.6208 m/z ± 8.4 mDa 305.1792 ± 2.0354 Å²
0%
100%
575%
7mm

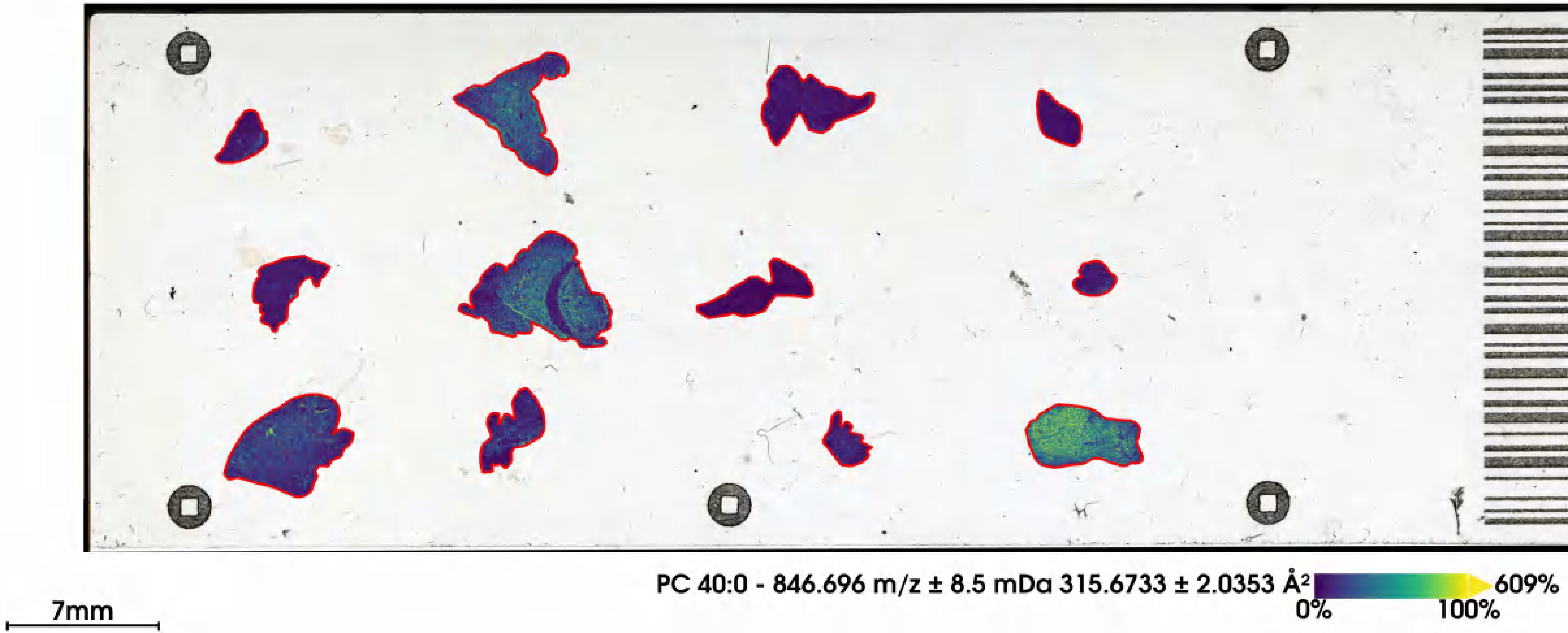
PC 40:0 - 846.696 m/z ± 8.5 mDa 315.6733 ± 2.0353 Å²
0%
100%
609%
7mm

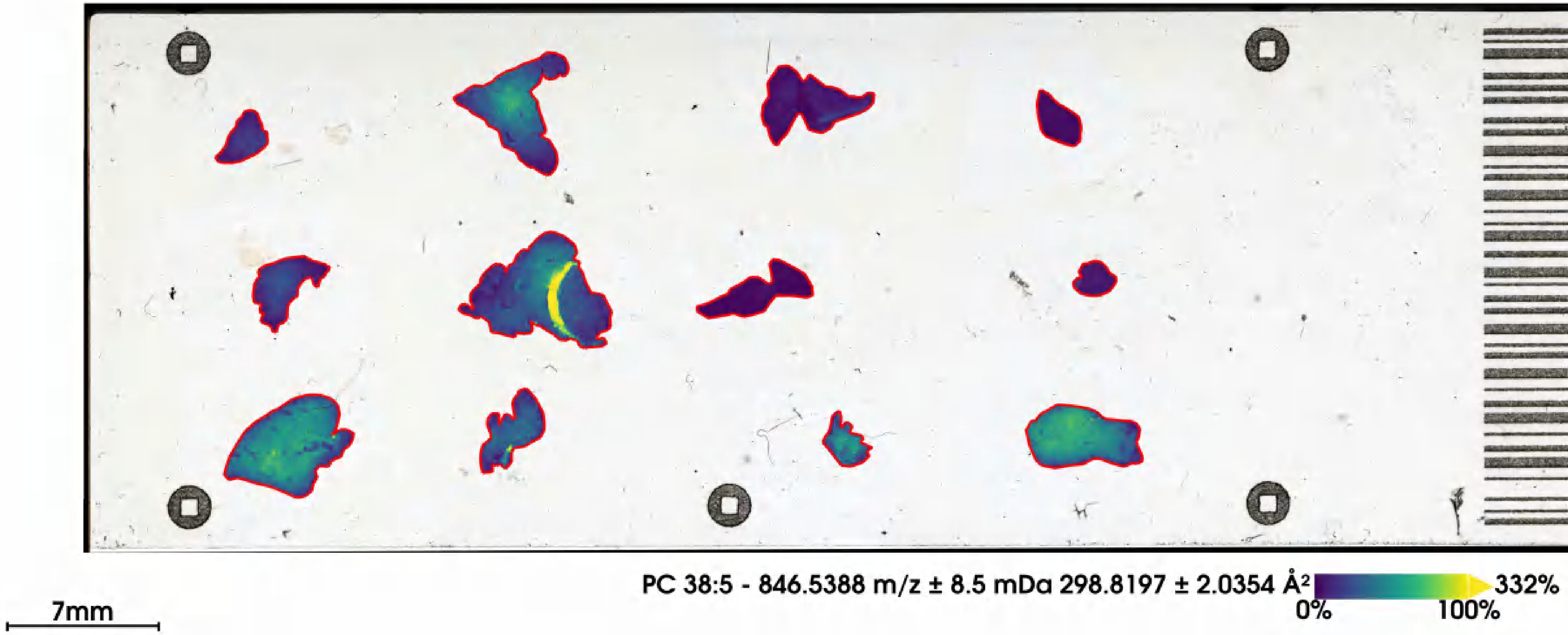
PC 38:5 - 846.5388 m/z ± 8.5 mDa 298.8197 ± 2.0354 Å²
0%
100%
332%
7mm

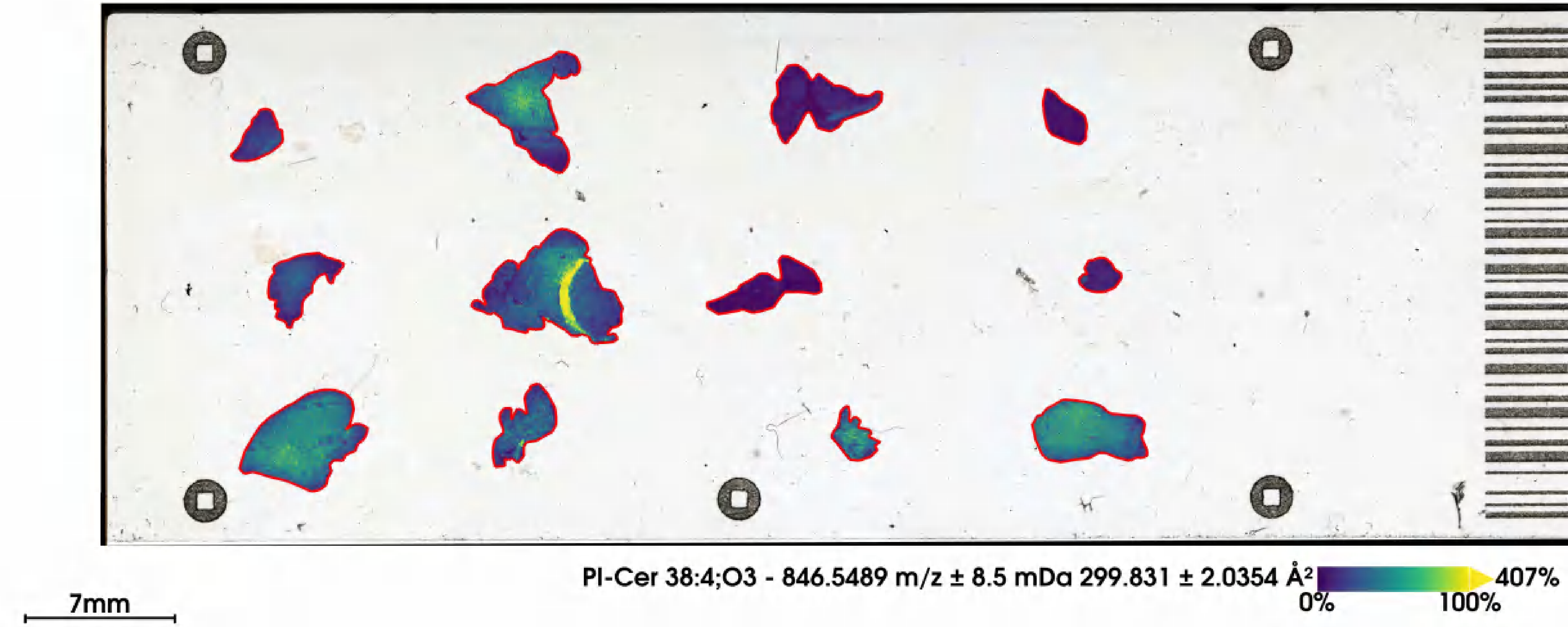
PI-Cer 38:4;O3 - 846.5489 m/z ± 8.5 mDa 299.831 ± 2.0354 Å²
407%
0%
100%
7mm

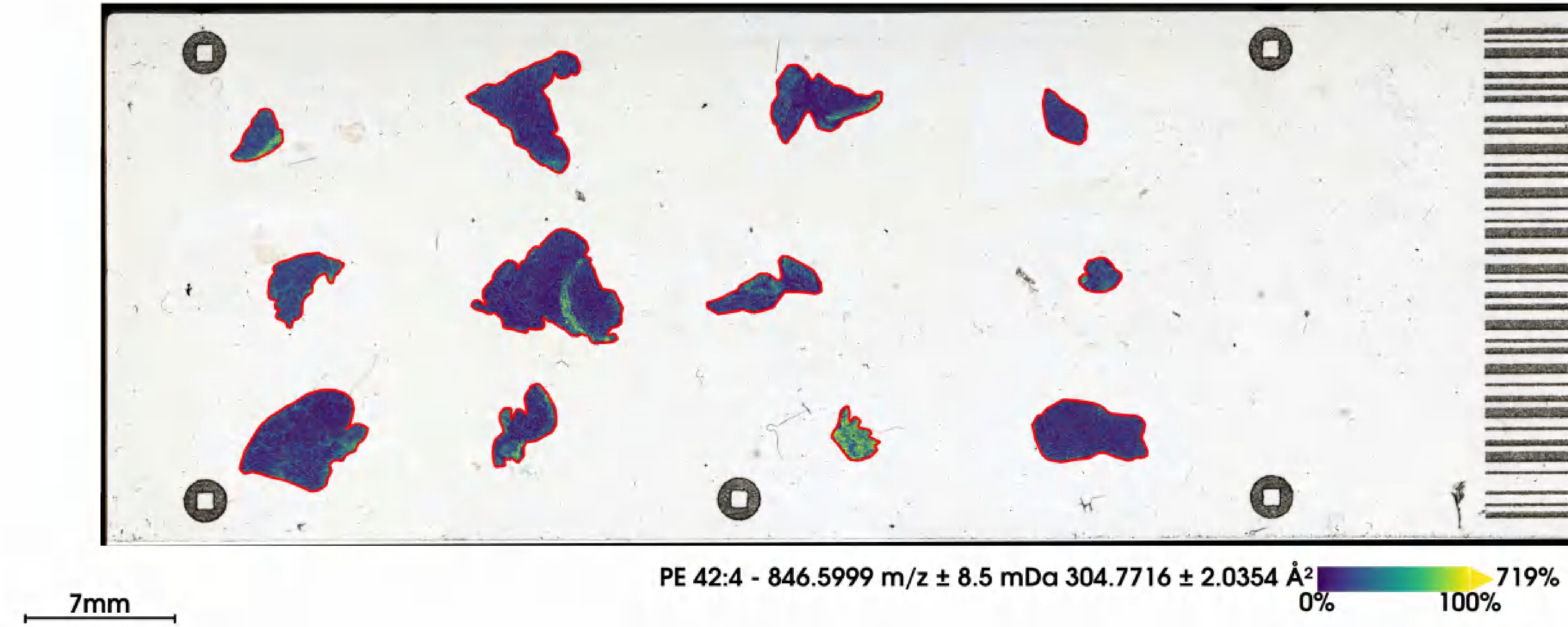

PE 42:4 - 846.5999 m/z ± 8.5 mDa 304.7716 ± 2.0354 Å²
0%
100%
719%
7mm

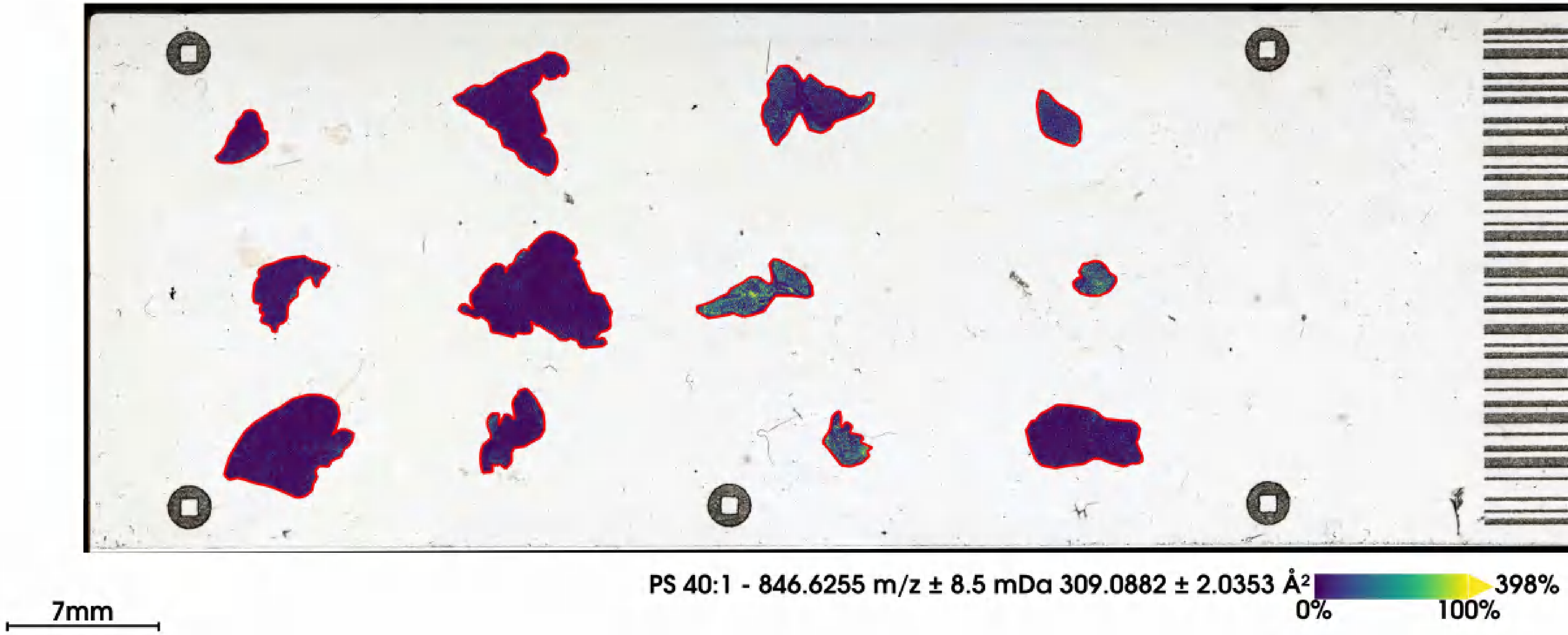
PS 40:1 - 846.6255 m/z ± 8.5 mDa 309.0882 ± 2.0353 Å²
398%
0%
100%
7mm

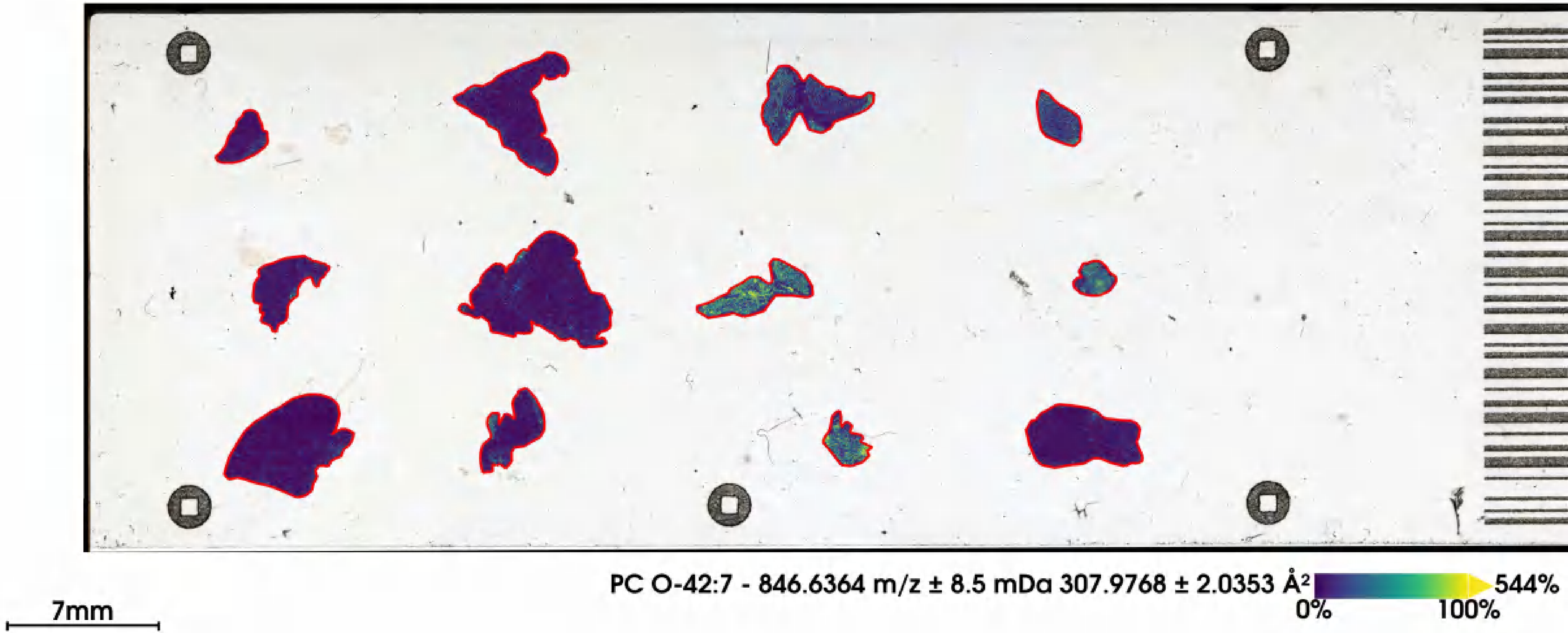
PC O-42:7 - 846.6364 m/z ± 8.5 mDa 307.9768 ± 2.0353 Å²
0%
100%
544%
7mm

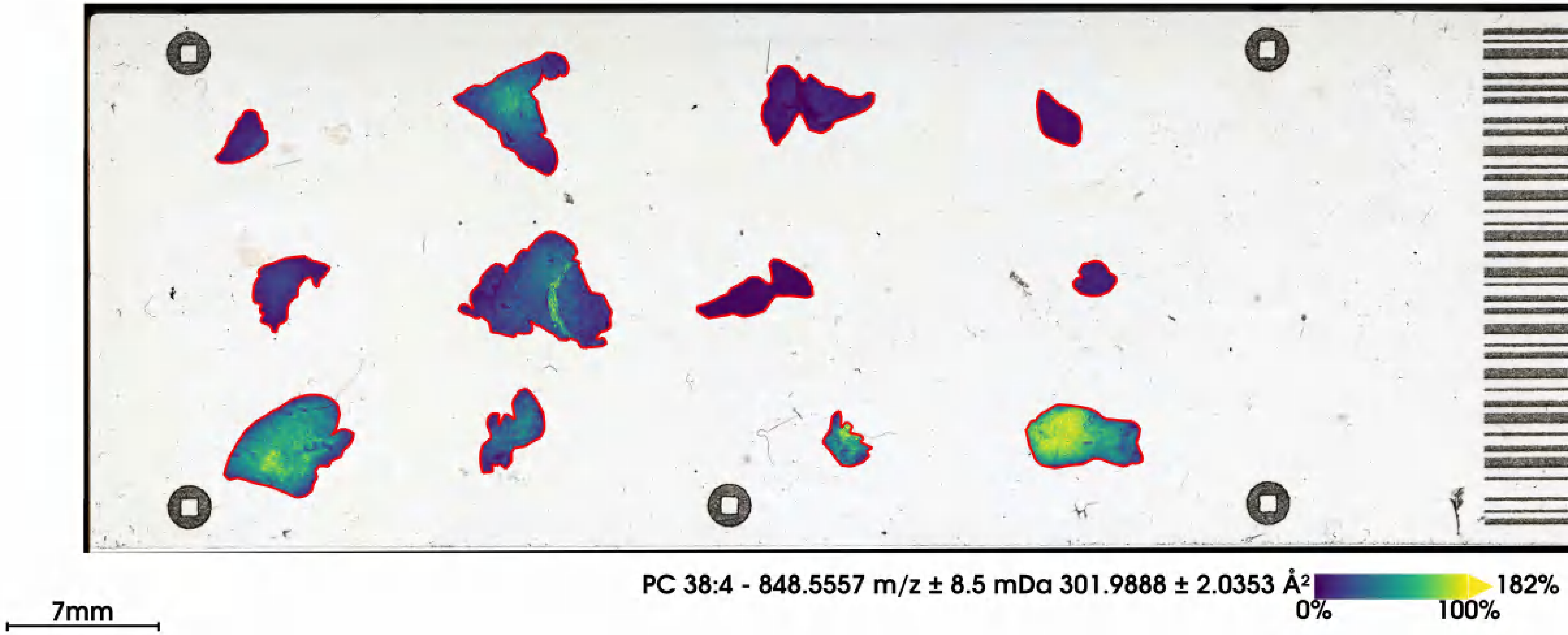

PC 38:4 - 848.5557 m/z ± 8.5 mDa 301.9888 ± 2.0353 Å²
0%
100%
182%
7mm

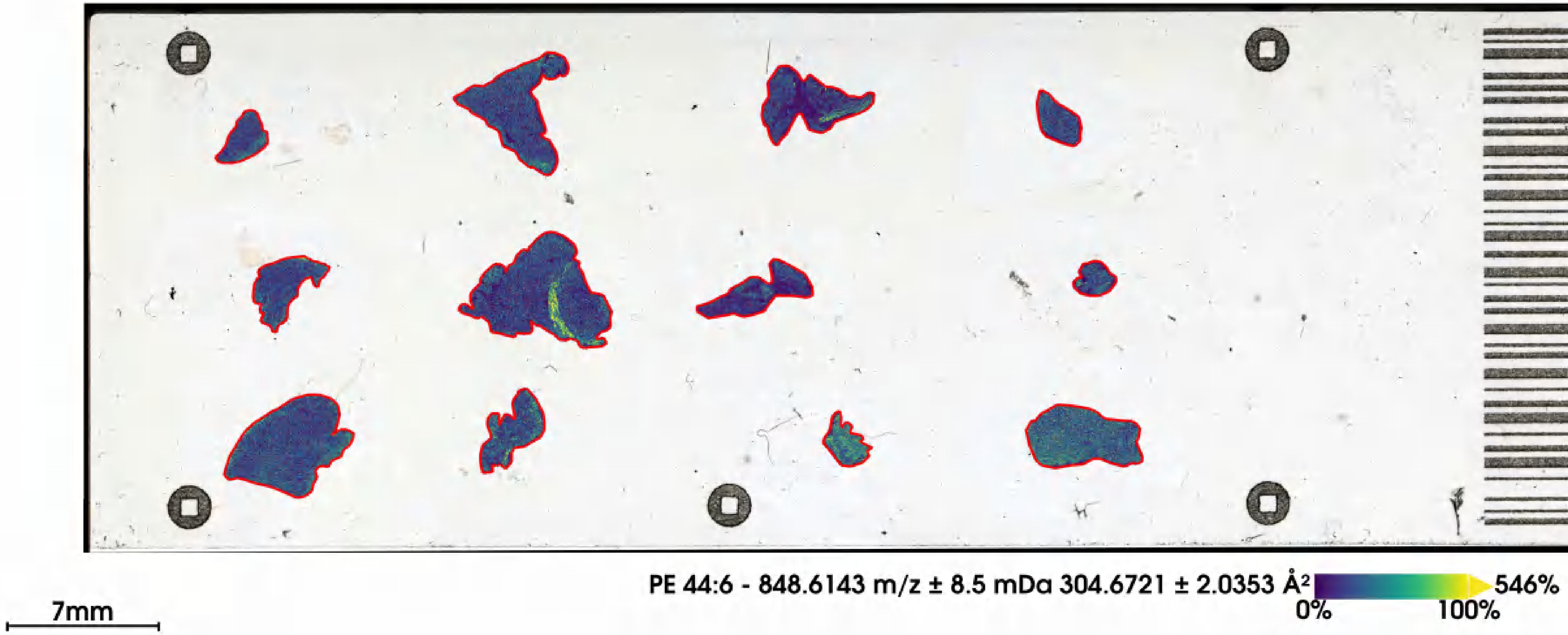
PE 44:6 - 848.6143 m/z ± 8.5 mDa 304.6721 ± 2.0353 Å²
546%
0%
100%
7mm

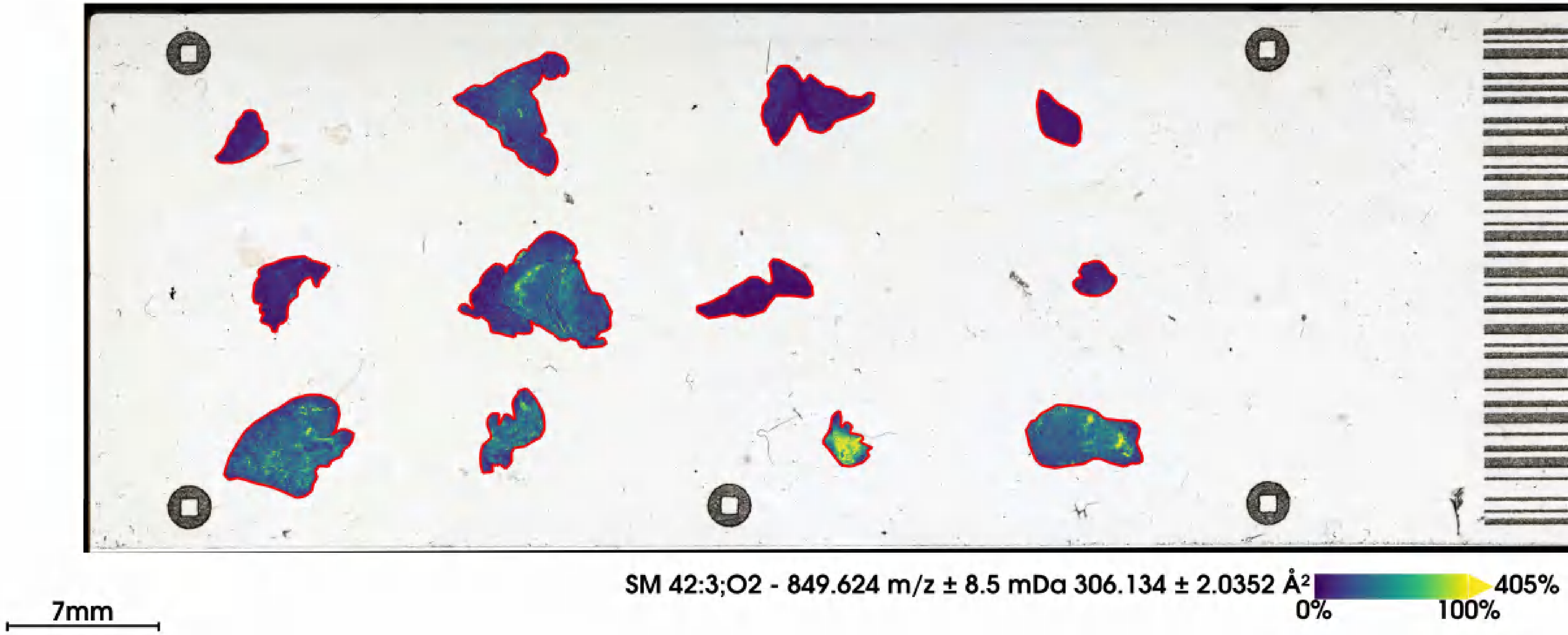
SM 42:3;O2 - 849.624 m/z ± 8.5 mDa 306.134 ± 2.0352 Å²
405%
0%
100%
7mm

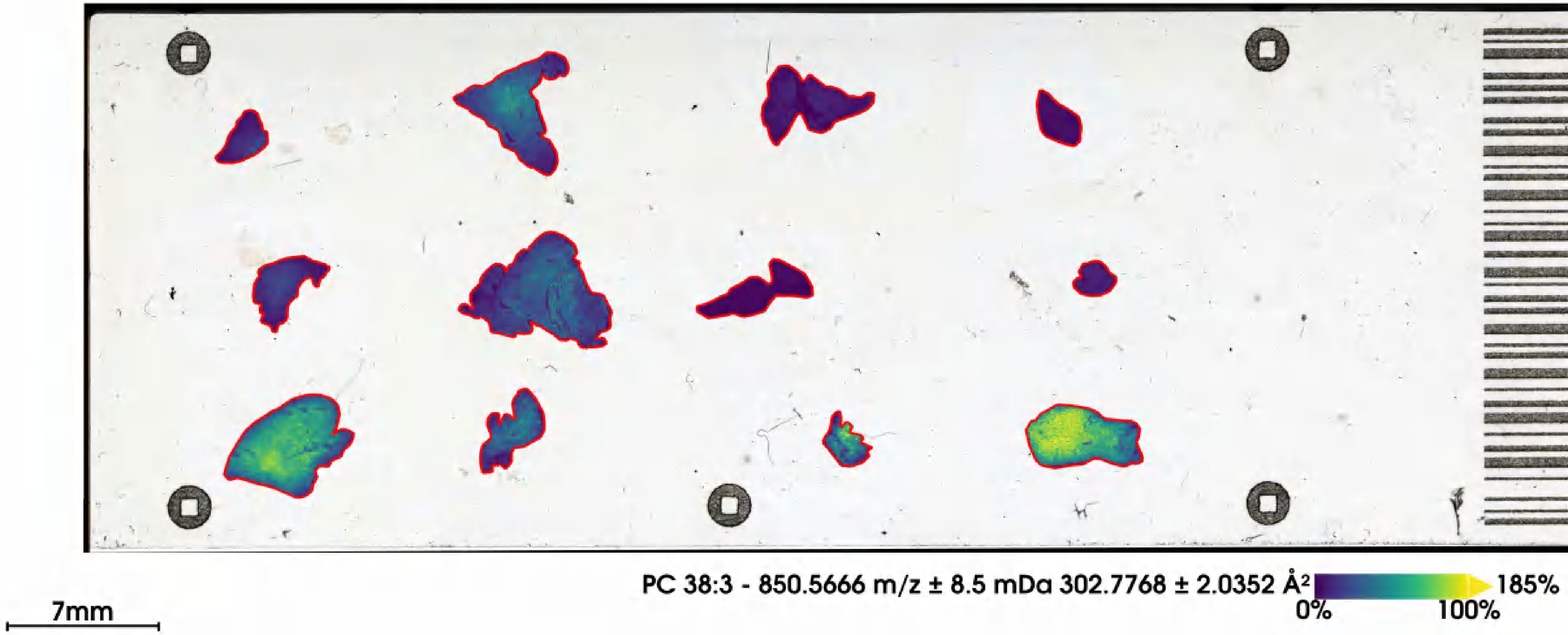
PC 38:3 - 850.5666 m/z ± 8.5 mDa 302.7768 ± 2.0352 Å²
0%
100%
185%
7mm

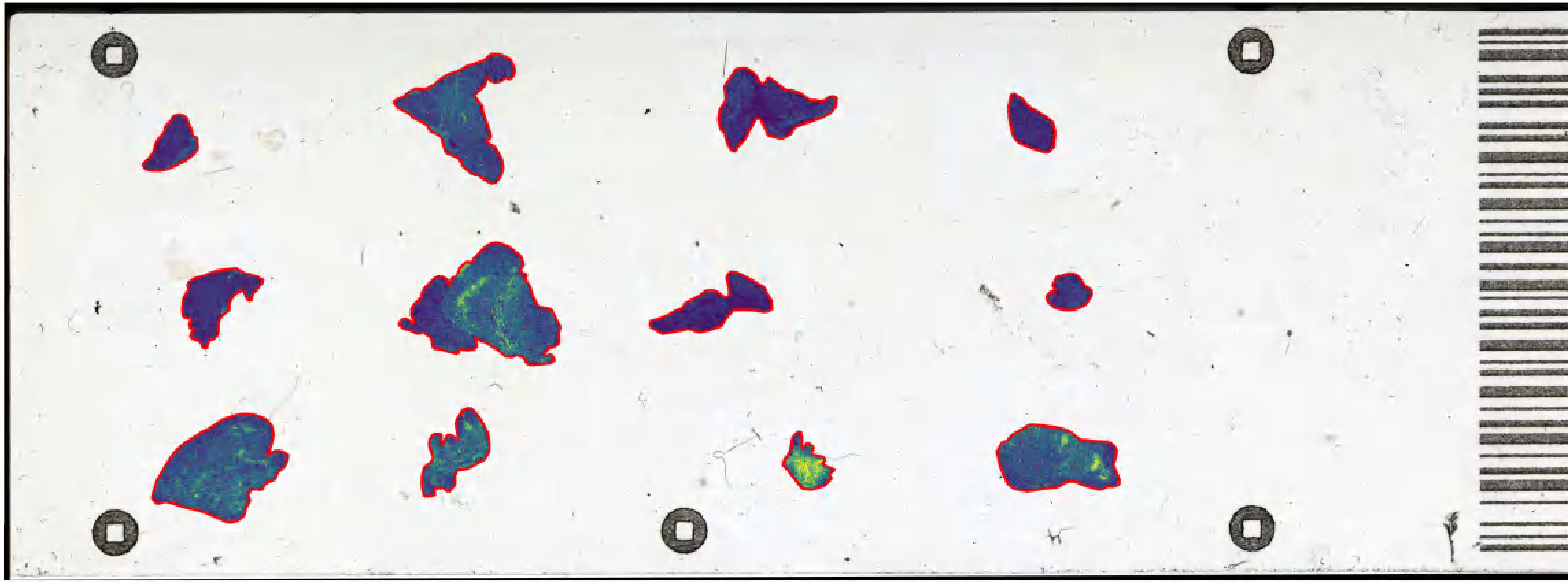

PE 44:5 - 850.6354 m/z ± 8.5 mDa 306.0128 ± 2.0352 Å² 0% 100% 1148%

7mm

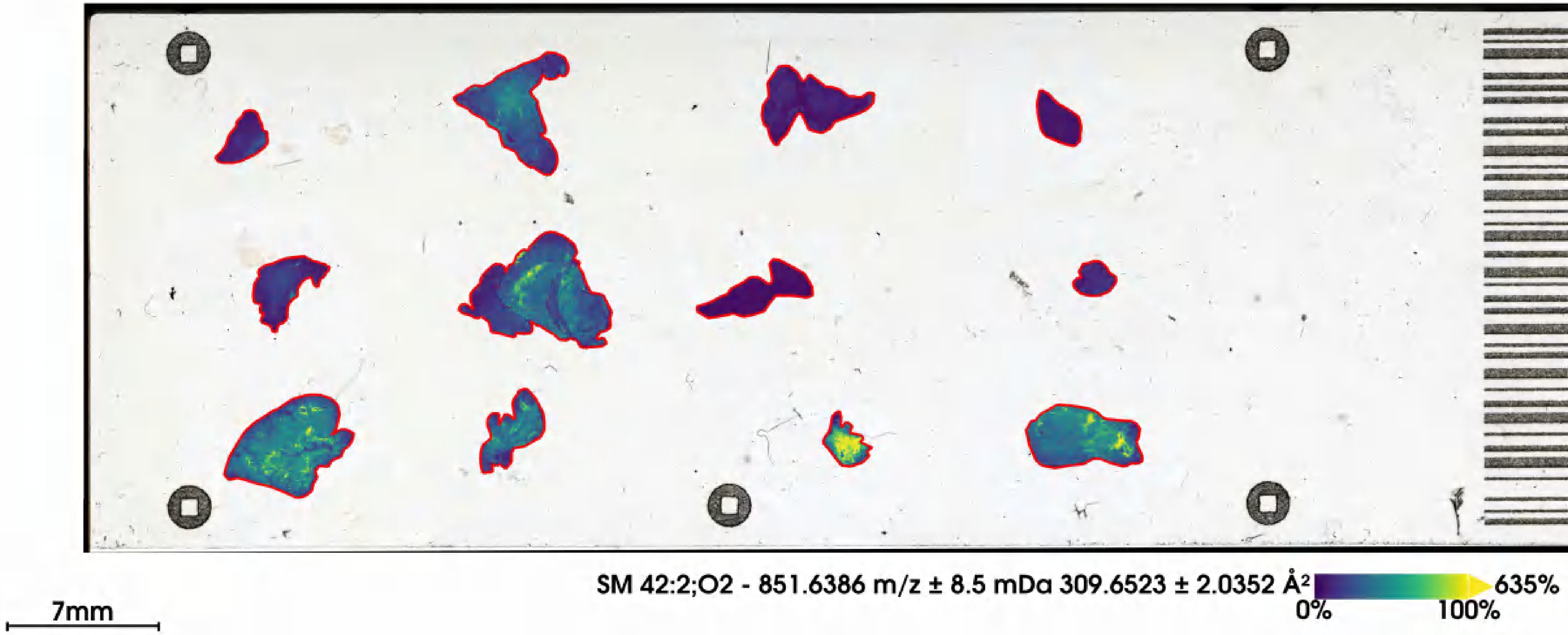
SM 42:2;O2 - 851.6386 m/z ± 8.5 mDa 309.6523 ± 2.0352 Å²
0%
100%
635%
7mm

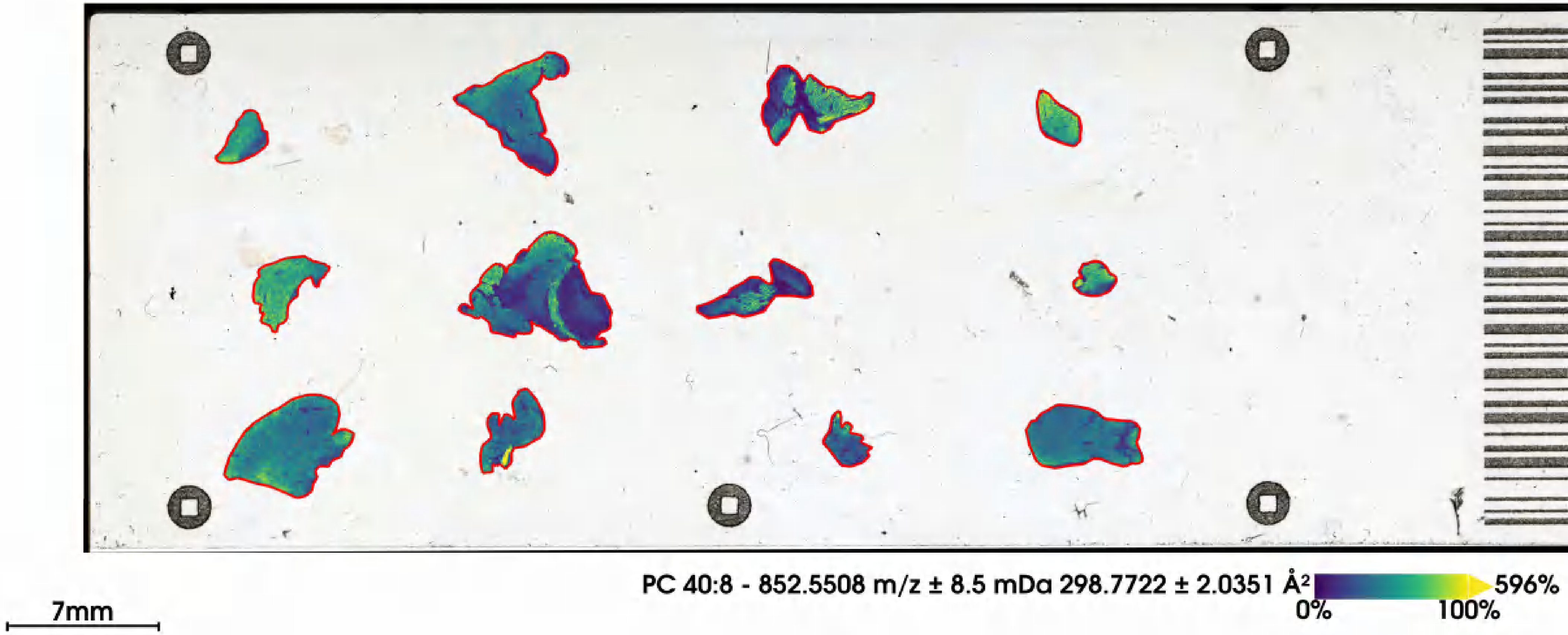
PC 40:8 - 852.5508 m/z ± 8.5 mDa 298.7722 ± 2.0351 Å²
0%
100%
596%
7mm

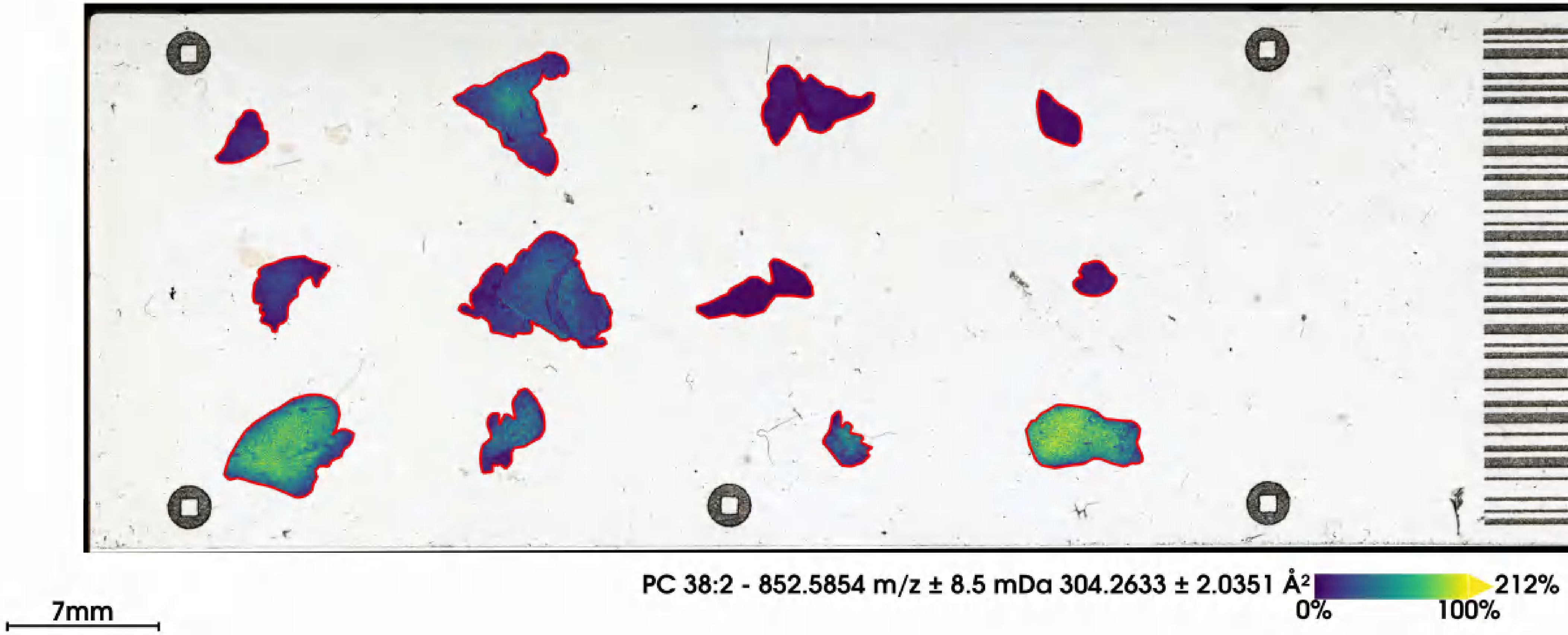

PC 38:2 - 852.5854 m/z ± 8.5 mDa 304.2633 ± 2.0351 Å²
212%
0%
100%
7mm

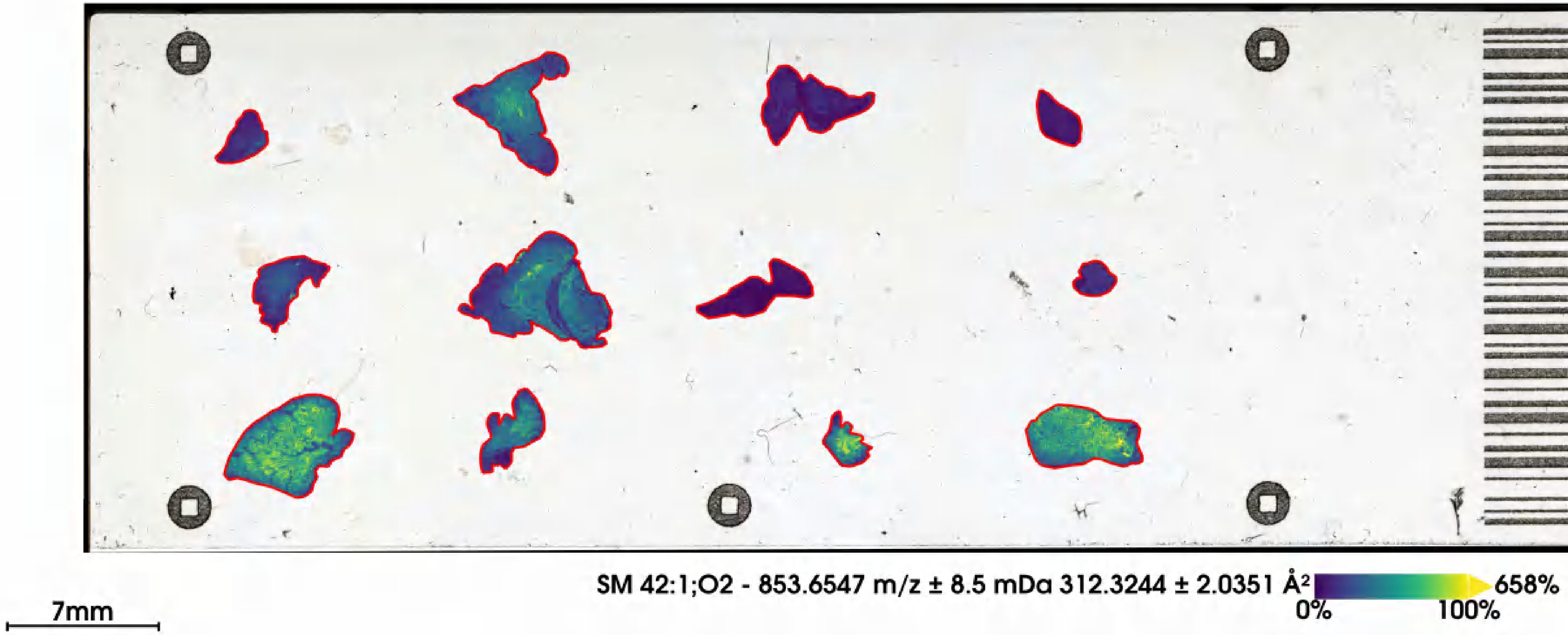

SM 42:1;O2 - 853.6547 m/z ± 8.5 mDa 312.3244 ± 2.0351 Å²
0%
100%
658%
7mm

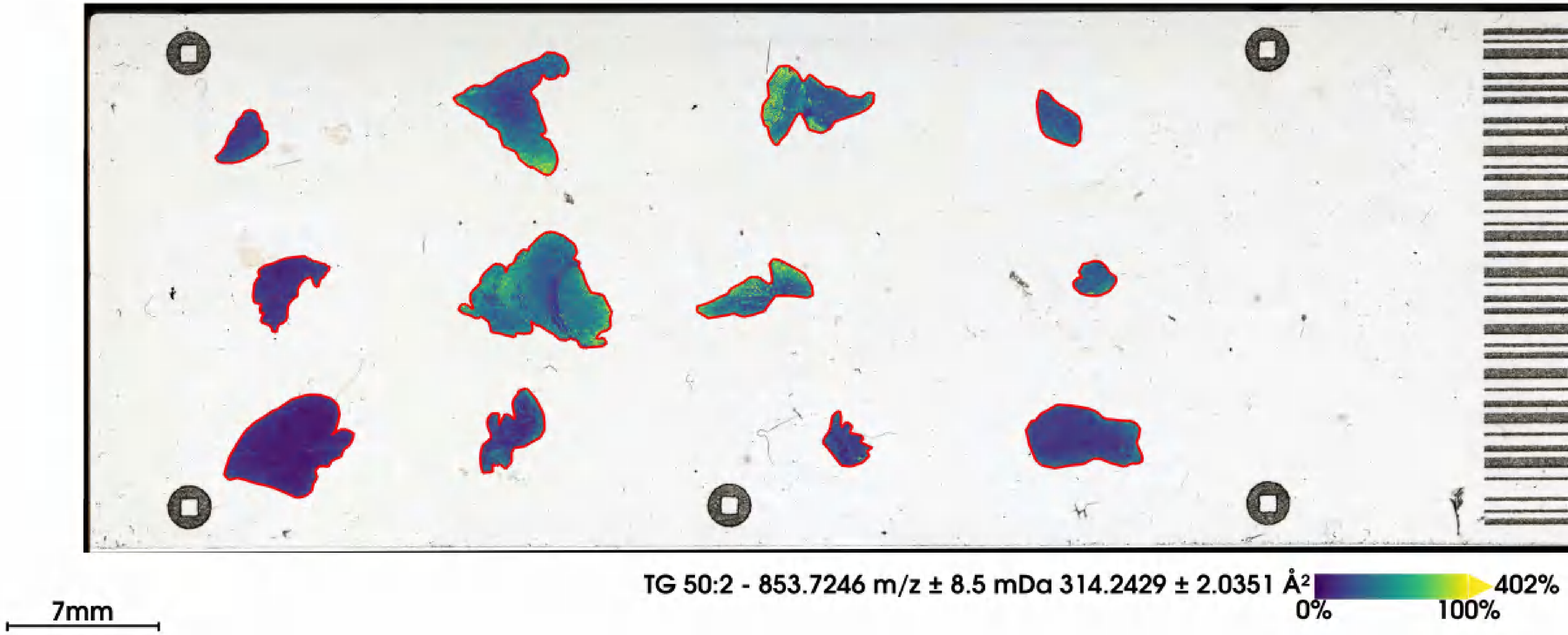
TG 50:2 - 853.7246 m/z ± 8.5 mDa 314.2429 ± 2.0351 Å²
0%
100%
402%
7mm

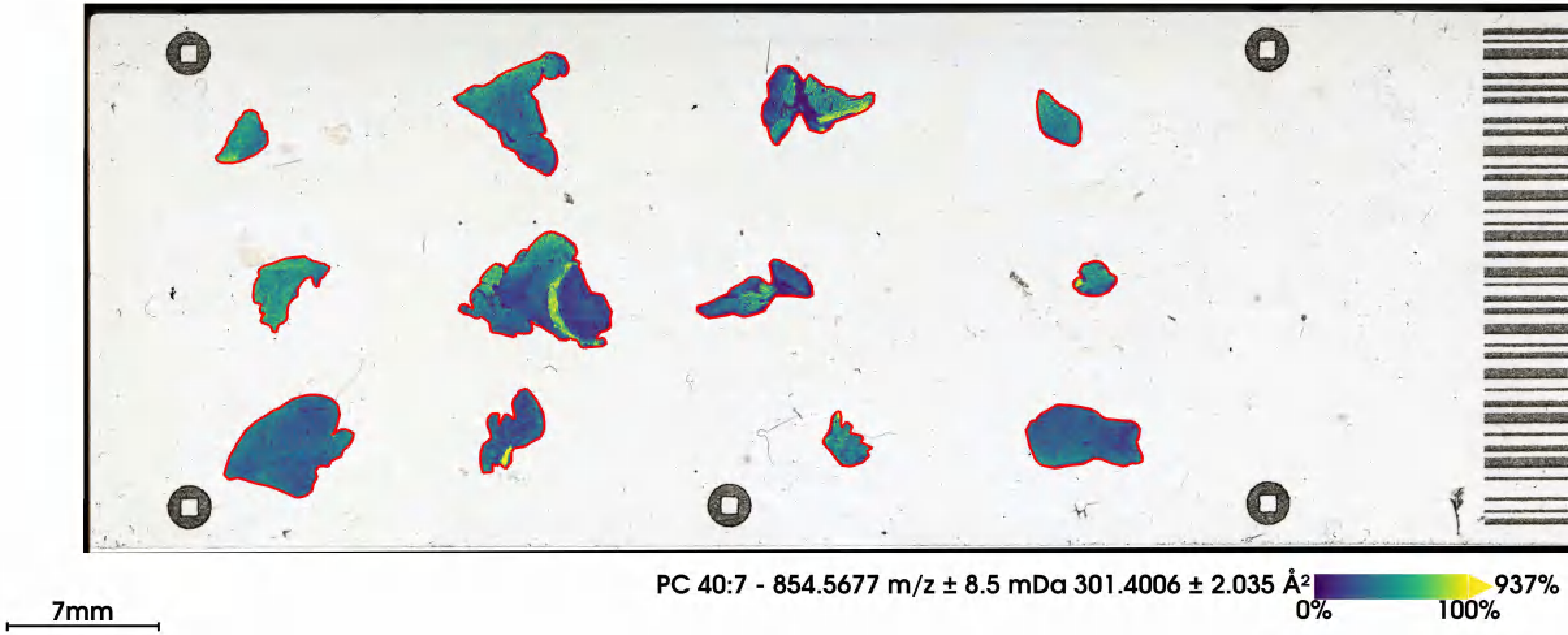
PC 40:7 - 854.5677 m/z ± 8.5 mDa 301.4006 ± 2.035 Å²
0%
100%
937%
7mm

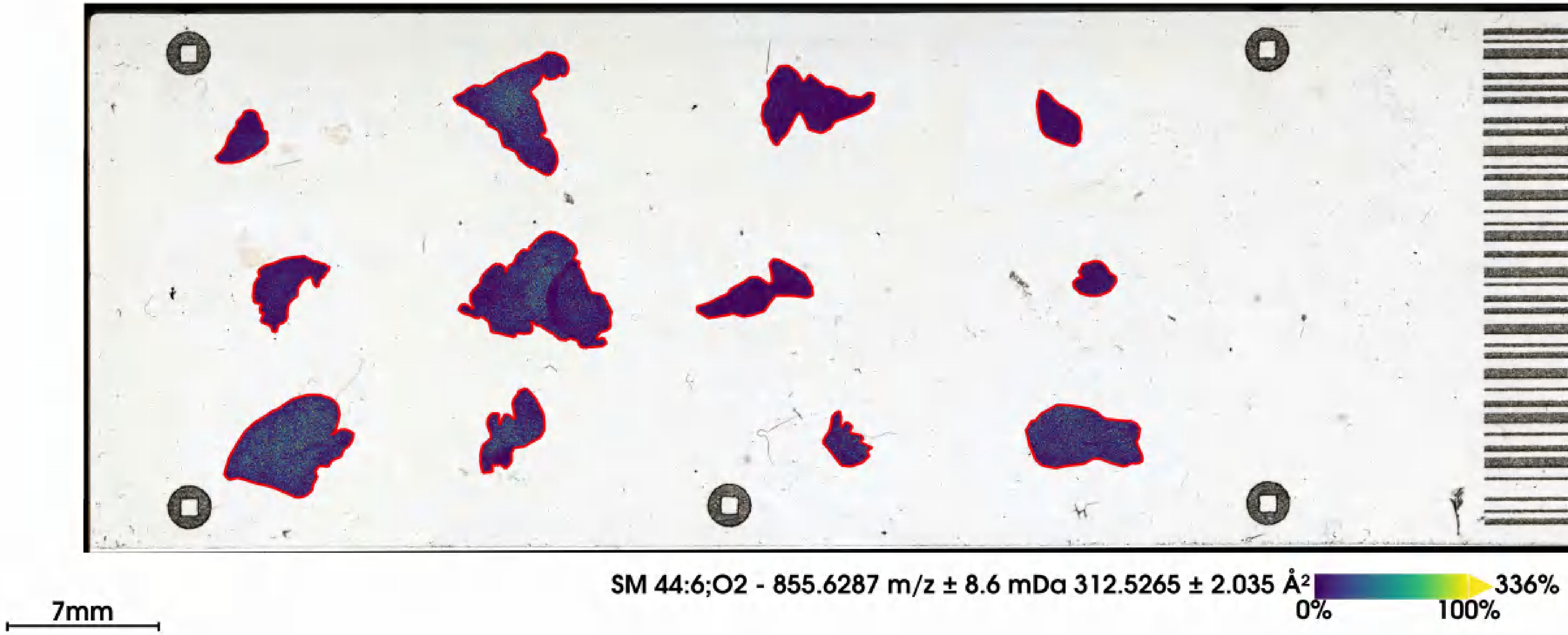

7mm
SM 44:6;O2 - 855.6287 m/z ± 8.6 mDa 312.5265 ± 2.035 Å²
0%
100%
336%

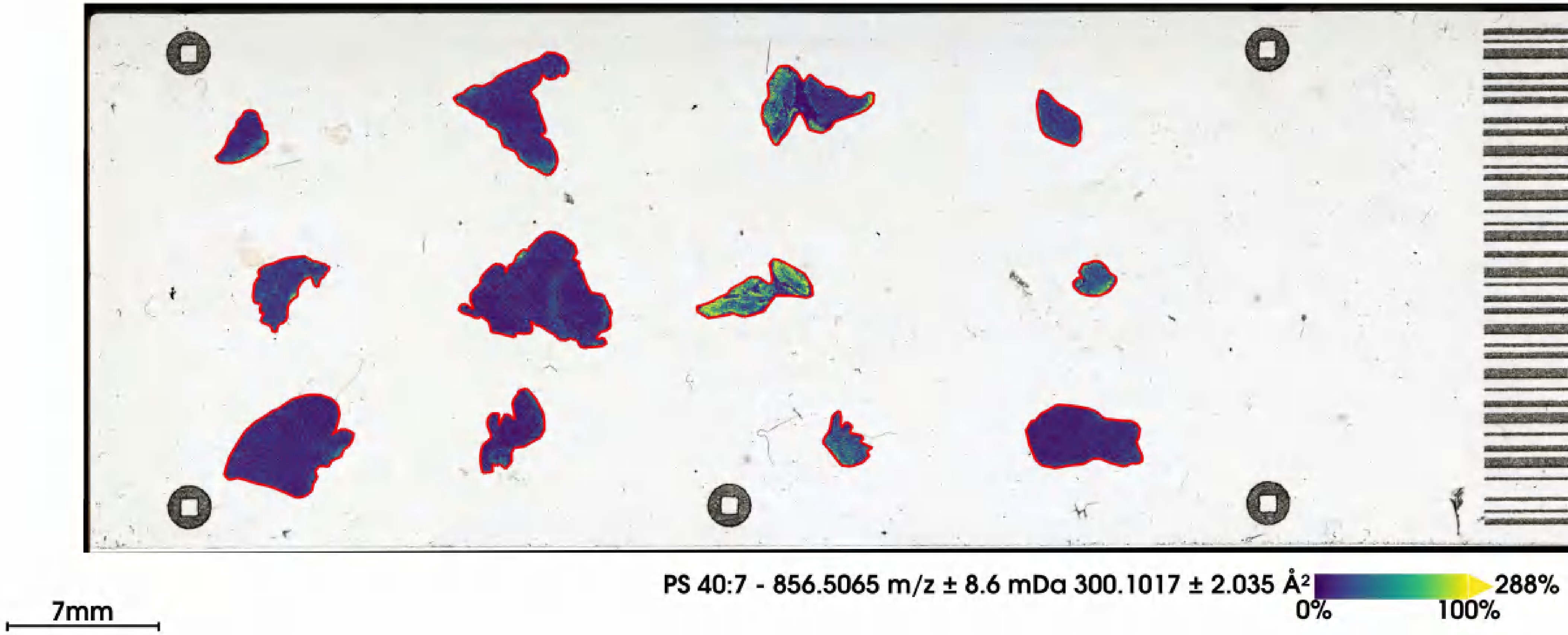

PS 40:7 - 856.5065 m/z ± 8.6 mDa 300.1017 ± 2.035 Å²
288%
0%
100%
7mm

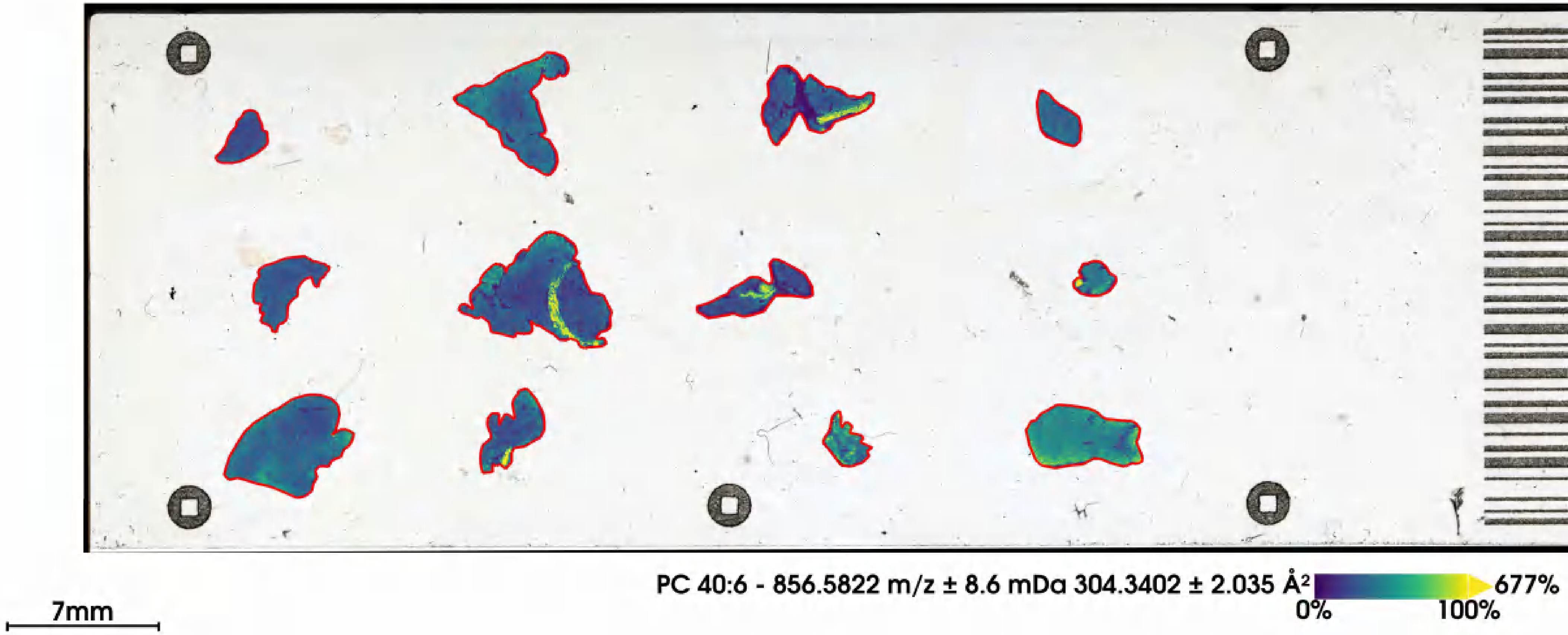

PC 40:6 - 856.5822 m/z ± 8.6 mDa 304.3402 ± 2.035 Å²
0%
100%
677%
7mm

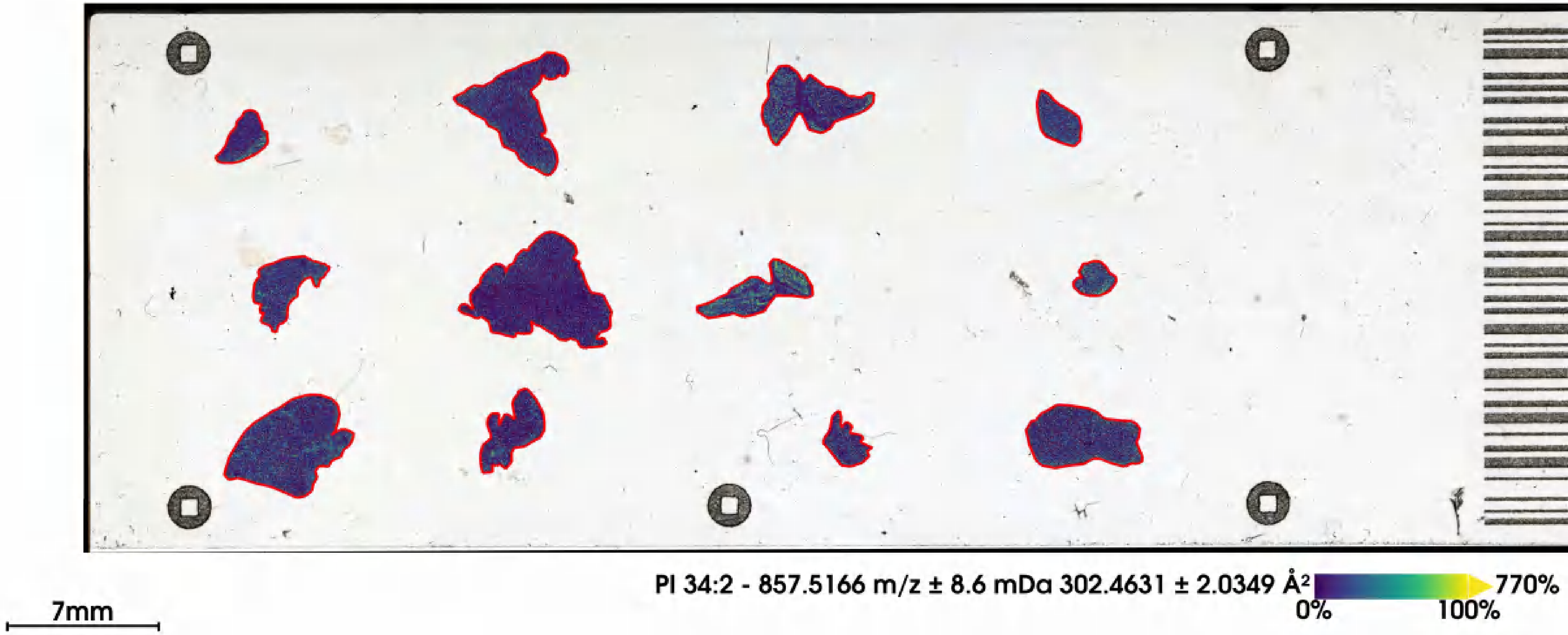

PI 34:2 - 857.5166 m/z ± 8.6 mDa 302.4631 ± 2.0349 Å²
0%
100%
770%
7mm

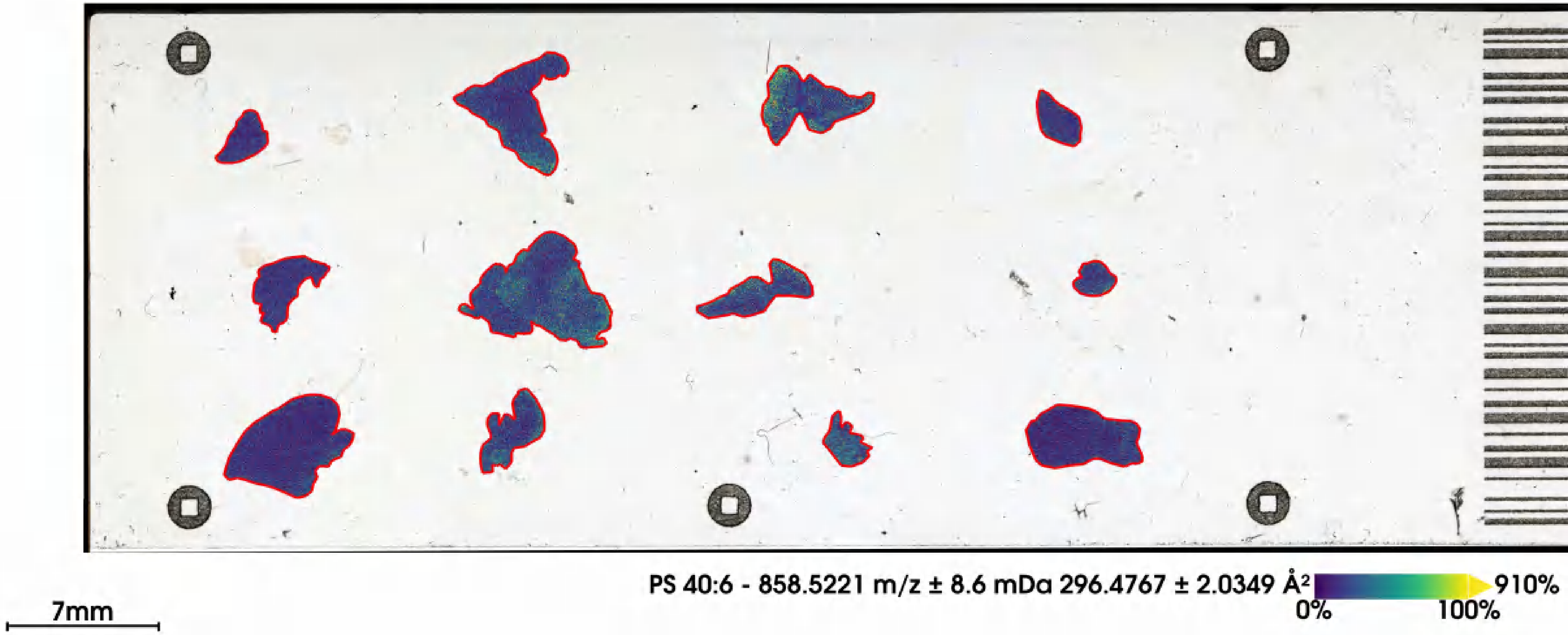
PS 40:6 - 858.5221 m/z ± 8.6 mDa 296.4767 ± 2.0349 Å²
0%
100%
910%
7mm

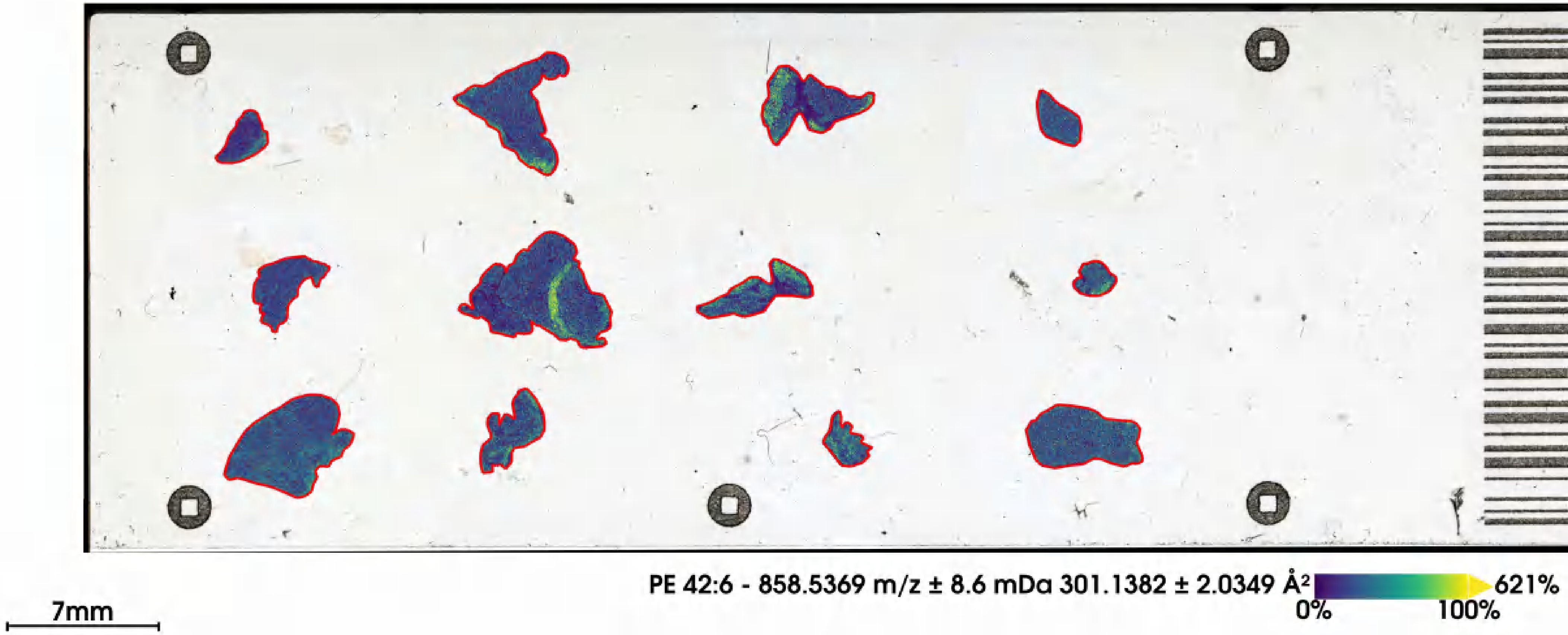
PE 42:6 - 858.5369 m/z ± 8.6 mDa 301.1382 ± 2.0349 Å²
0%
100%
621%
7mm

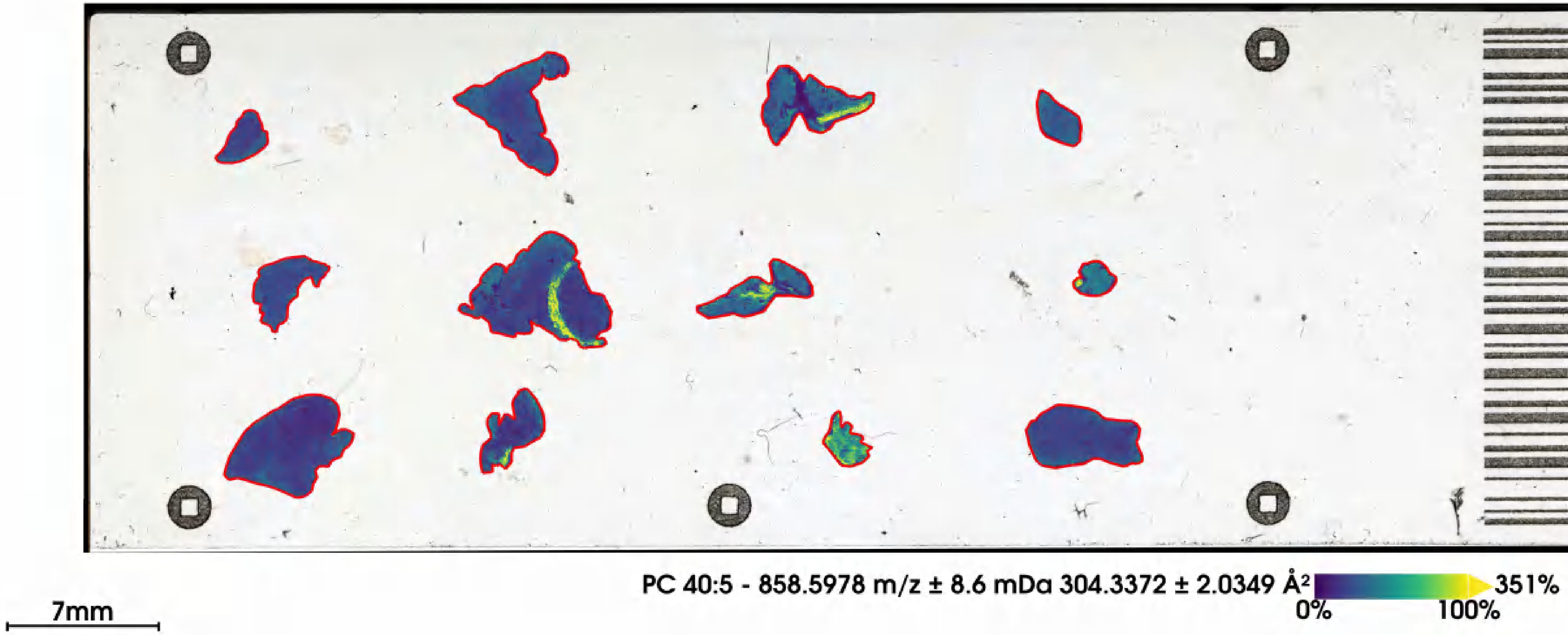

7mm
PC 40:5 - 858.5978 m/z ± 8.6 mDa 304.3372 ± 2.0349 Å²
0%
100%
351%

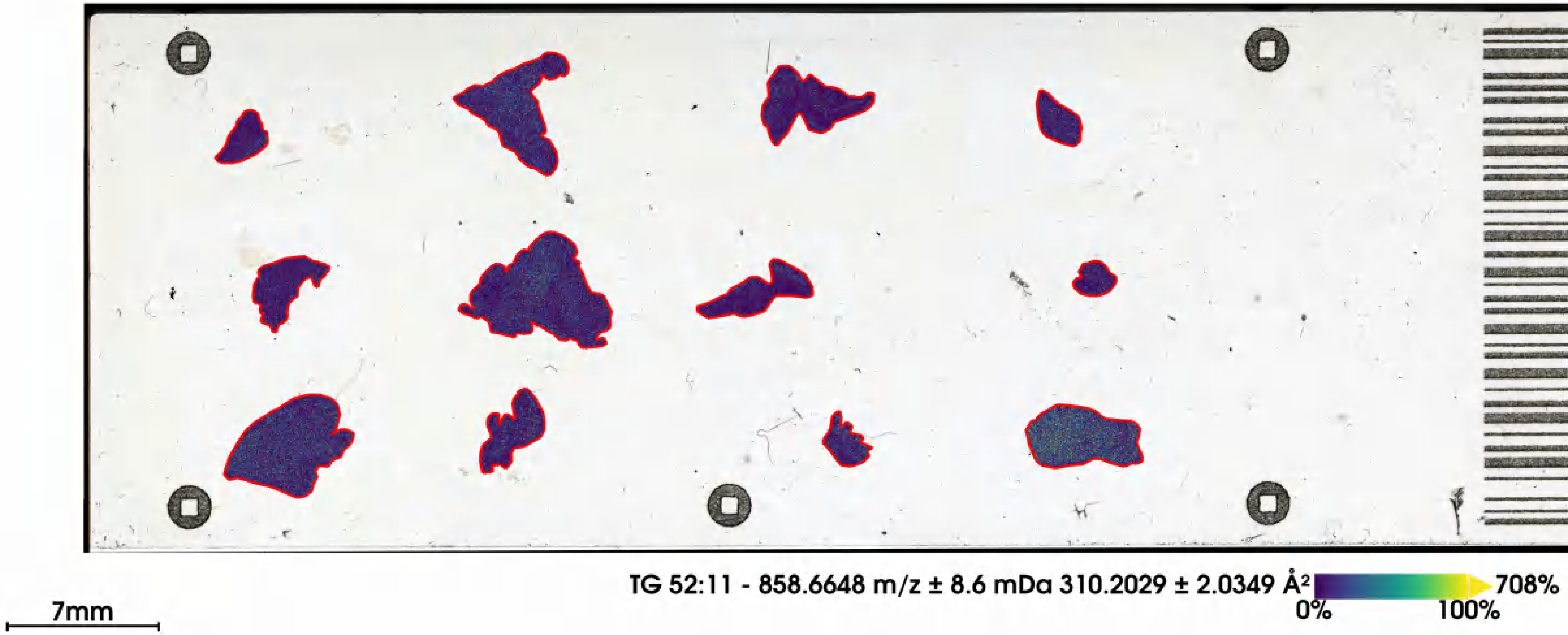
TG 52:11 - 858.6648 m/z ± 8.6 mDa 310.2029 ± 2.0349 Å²
708%
0%
100%
7mm

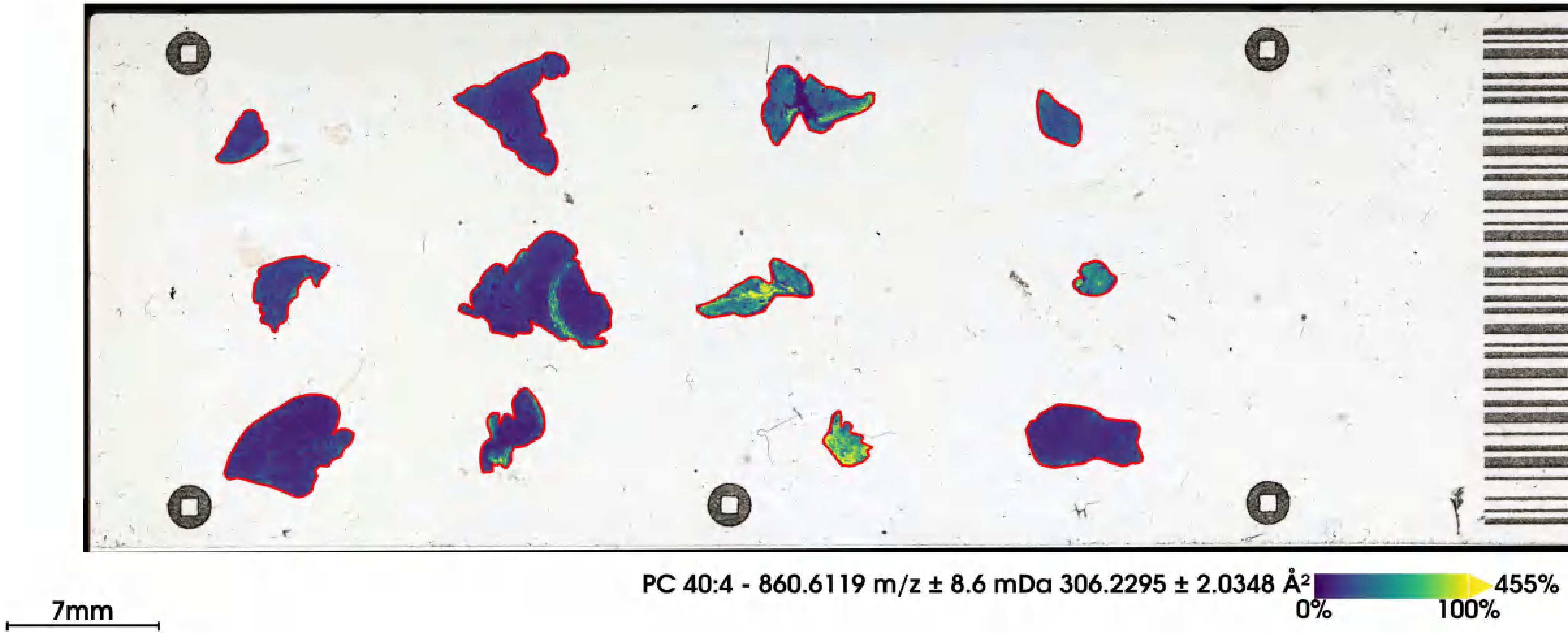

PC 40:4 - 860.6119 m/z ± 8.6 mDa 306.2295 ± 2.0348 Å²
455%
0%
100%
7mm

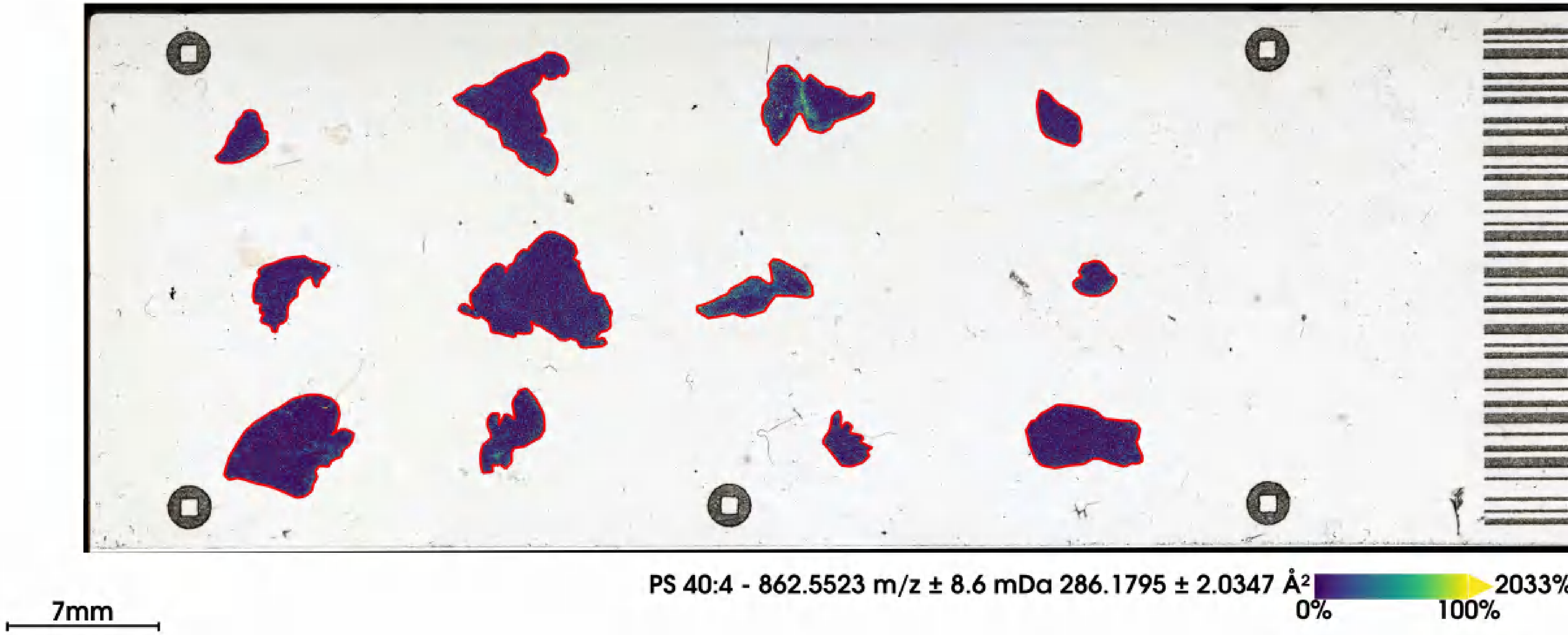
PS 40:4 - 862.5523 m/z ± 8.6 mDa 286.1795 ± 2.0347 Å²
0%
100%
2033%
7mm

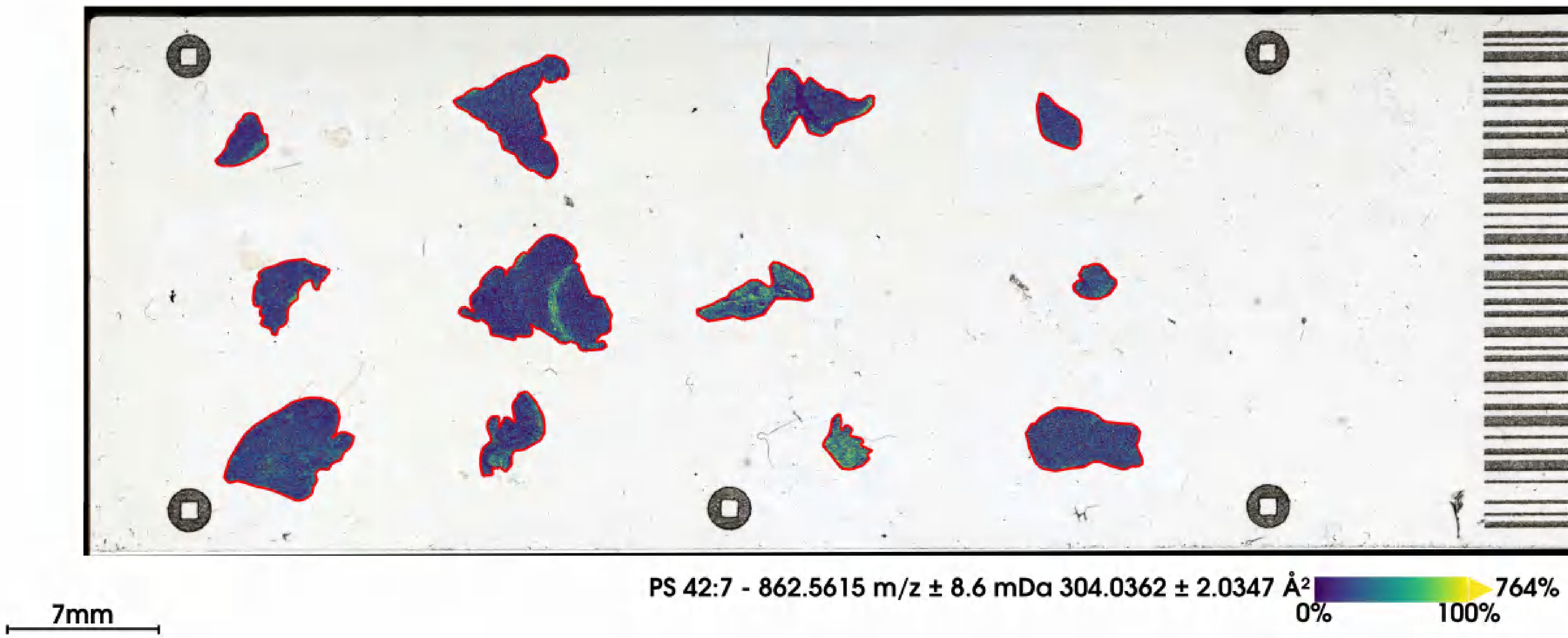
PS 42:7 - 862.5615 m/z ± 8.6 mDa 304.0362 ± 2.0347 Å²
764%
0%
100%
7mm

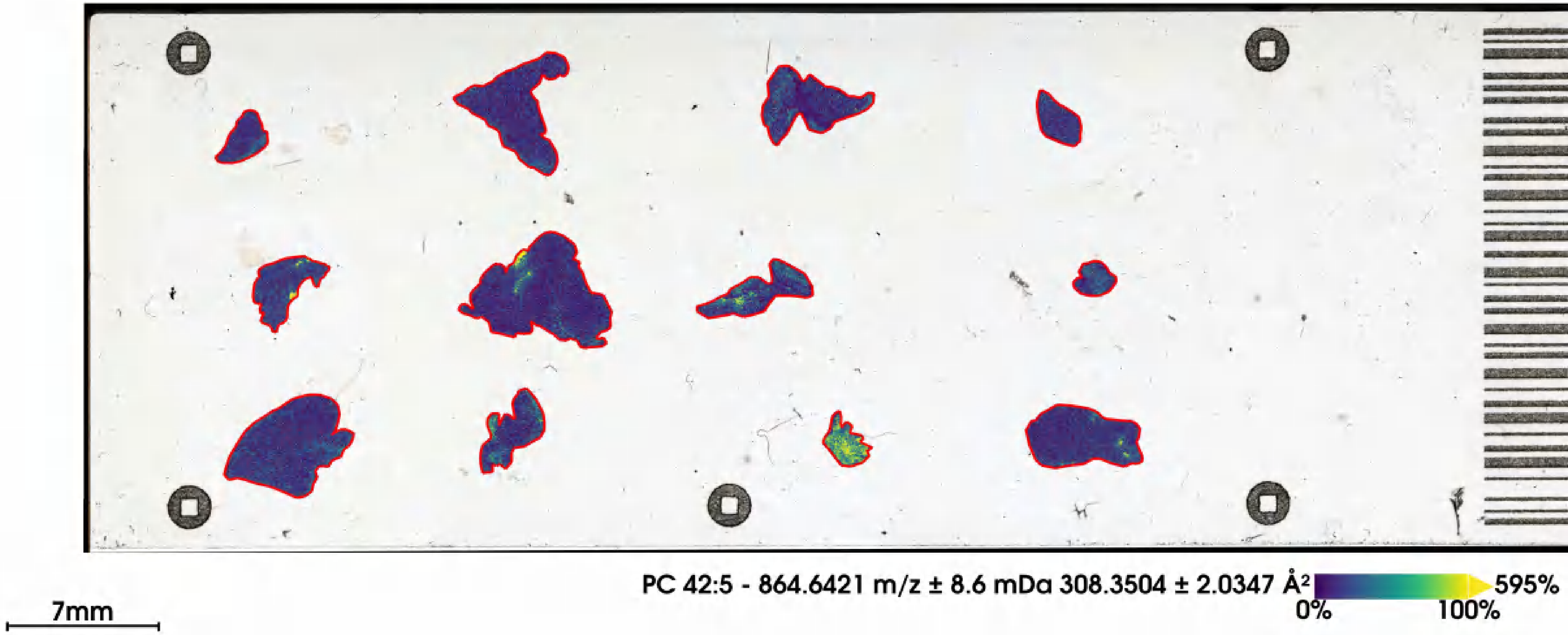
PC 42:5 - 864.6421 m/z ± 8.6 mDa 308.3504 ± 2.0347 Å²
0%
100%
595%
7mm

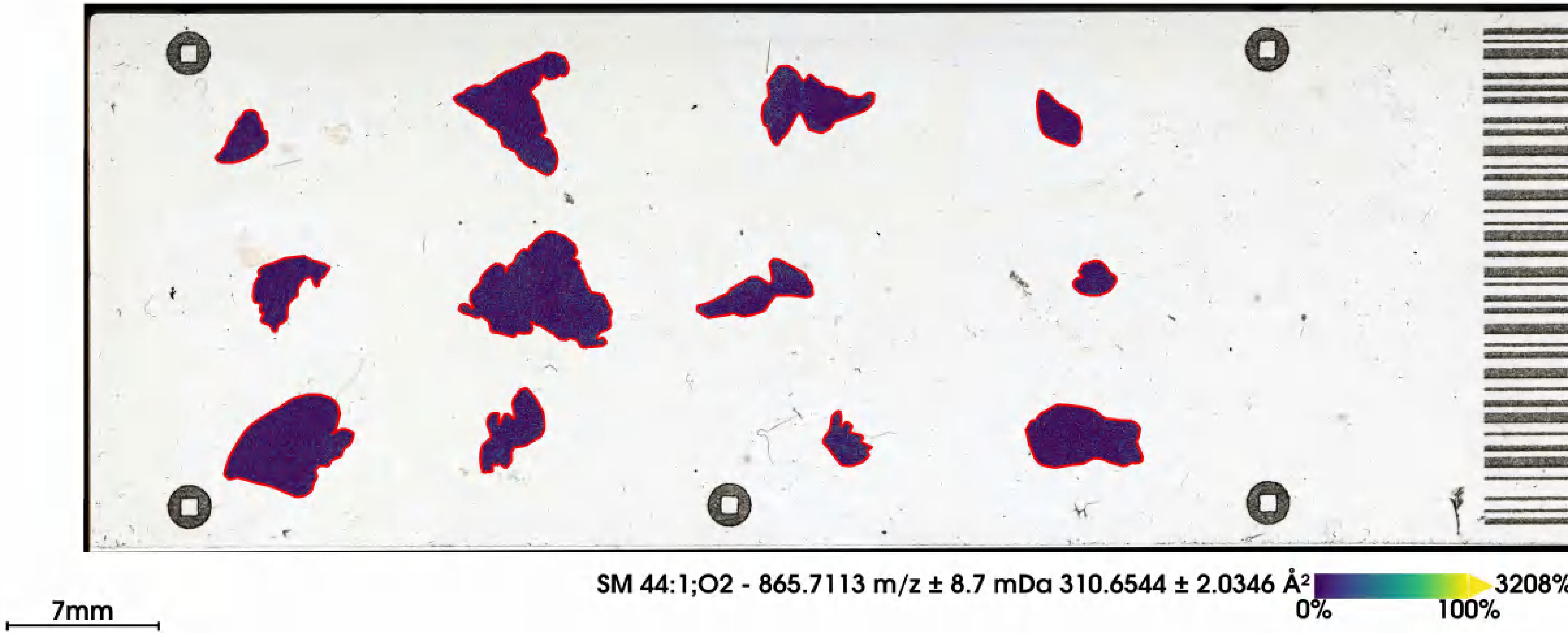

7mm
SM 44:1;O2 - 865.7113 m/z ± 8.7 mDa 310.6544 ± 2.0346 Å²
0%
100%
3208%

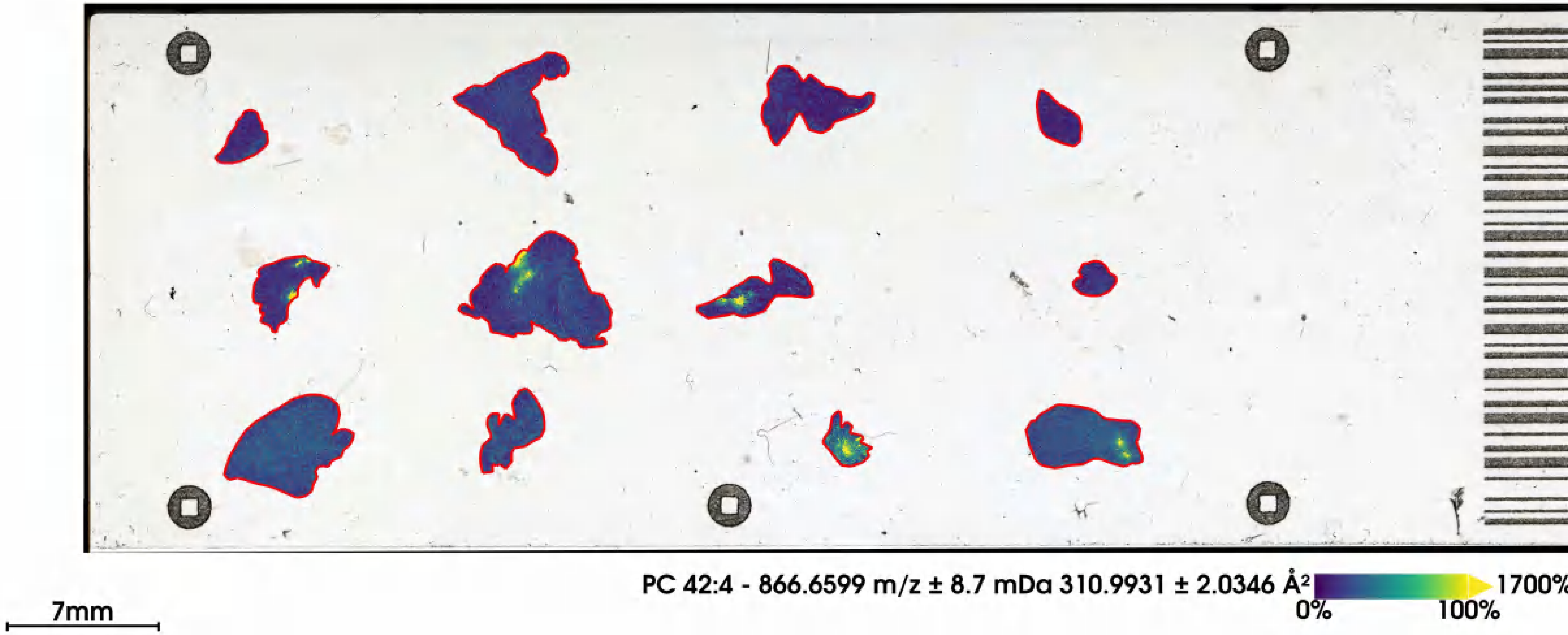
PC 42:4 - 866.6599 m/z ± 8.7 mDa 310.9931 ± 2.0346 Å²
0%
100%
1700%
7mm

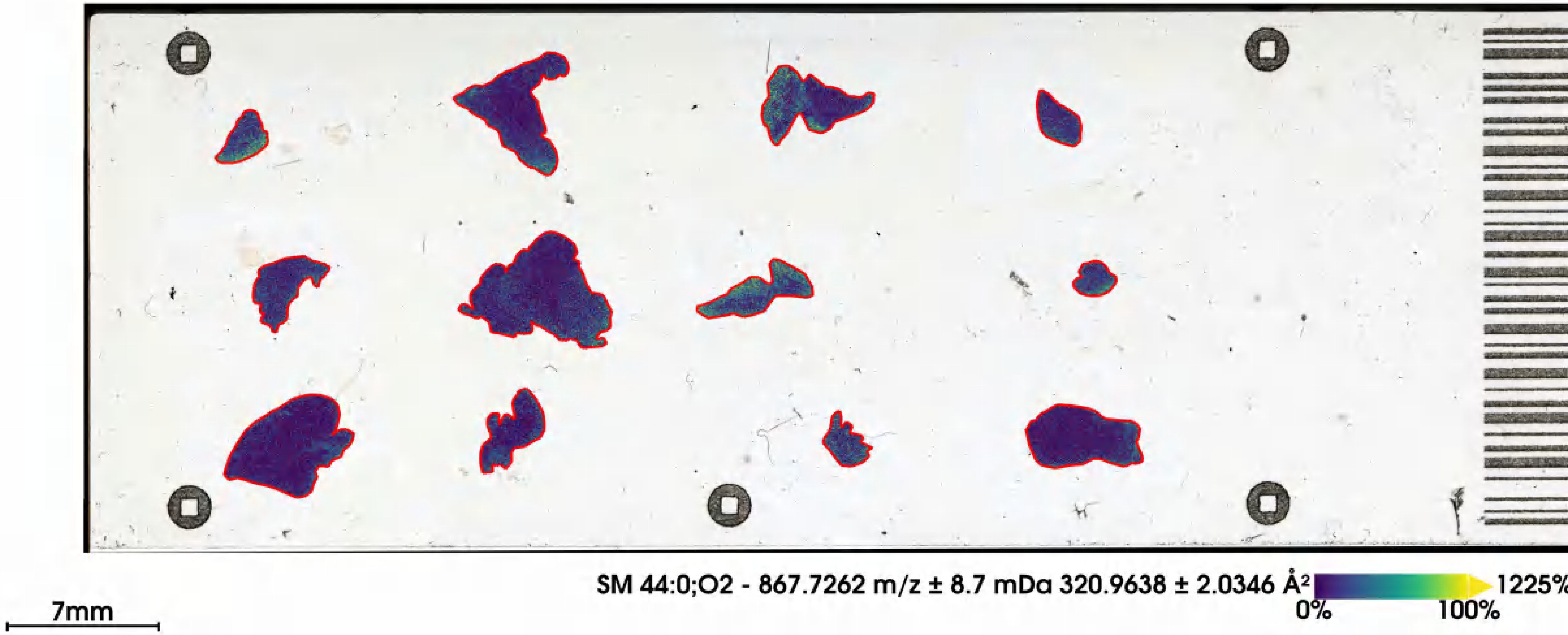

SM 44:0;O2 - 867.7262 m/z ± 8.7 mDa 320.9638 ± 2.0346 Å²
1225%
0%
100%
7mm

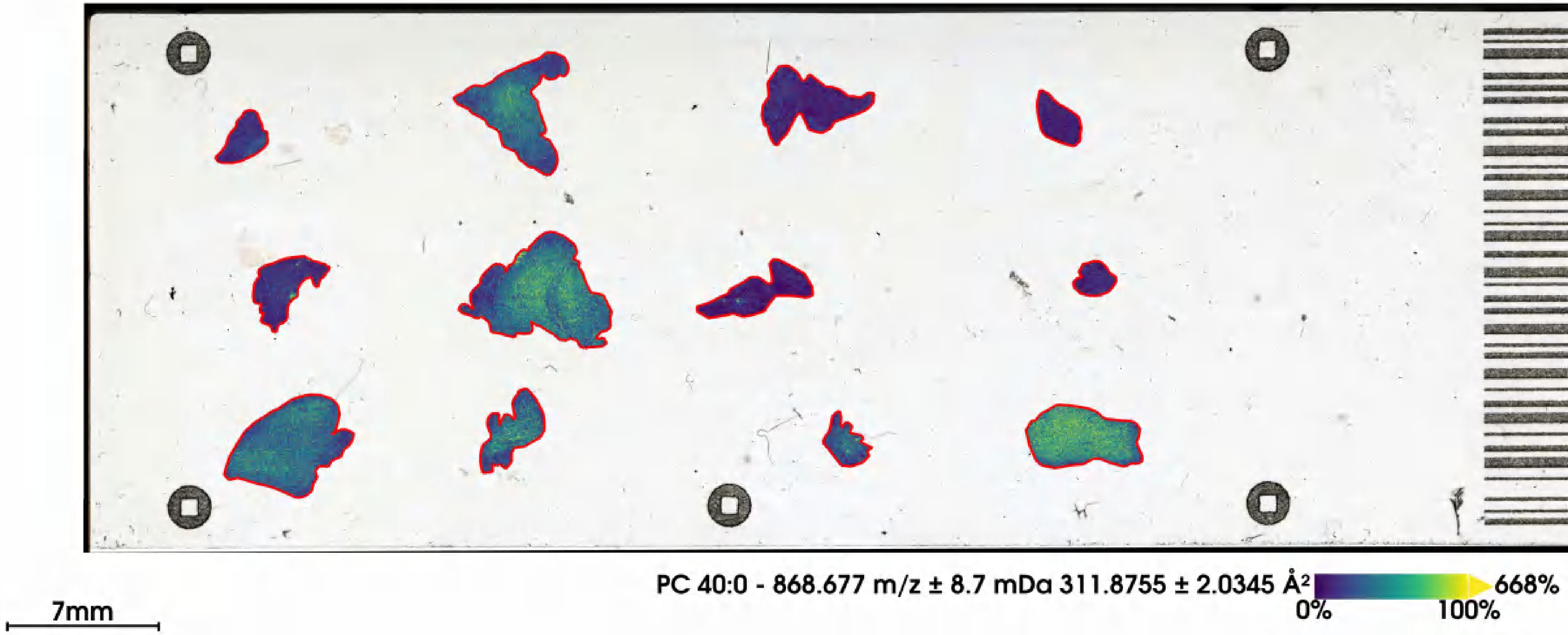
PC 40:0 - 868.677 m/z ± 8.7 mDa 311.8755 ± 2.0345 Å²
0%
100%
668%
7mm

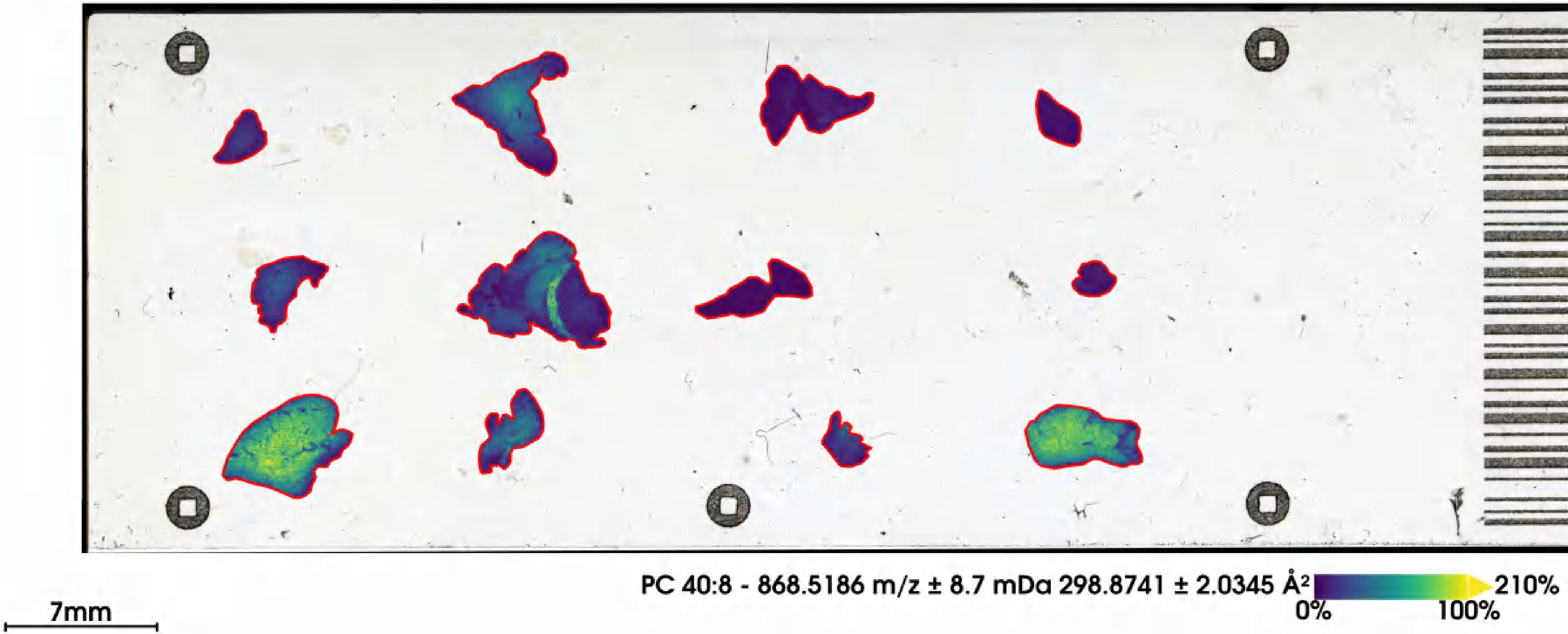

PC 40:8 - 868.5186 m/z ± 8.7 mDa 298.8741 ± 2.0345 Å²
0%
100%
210%
7mm

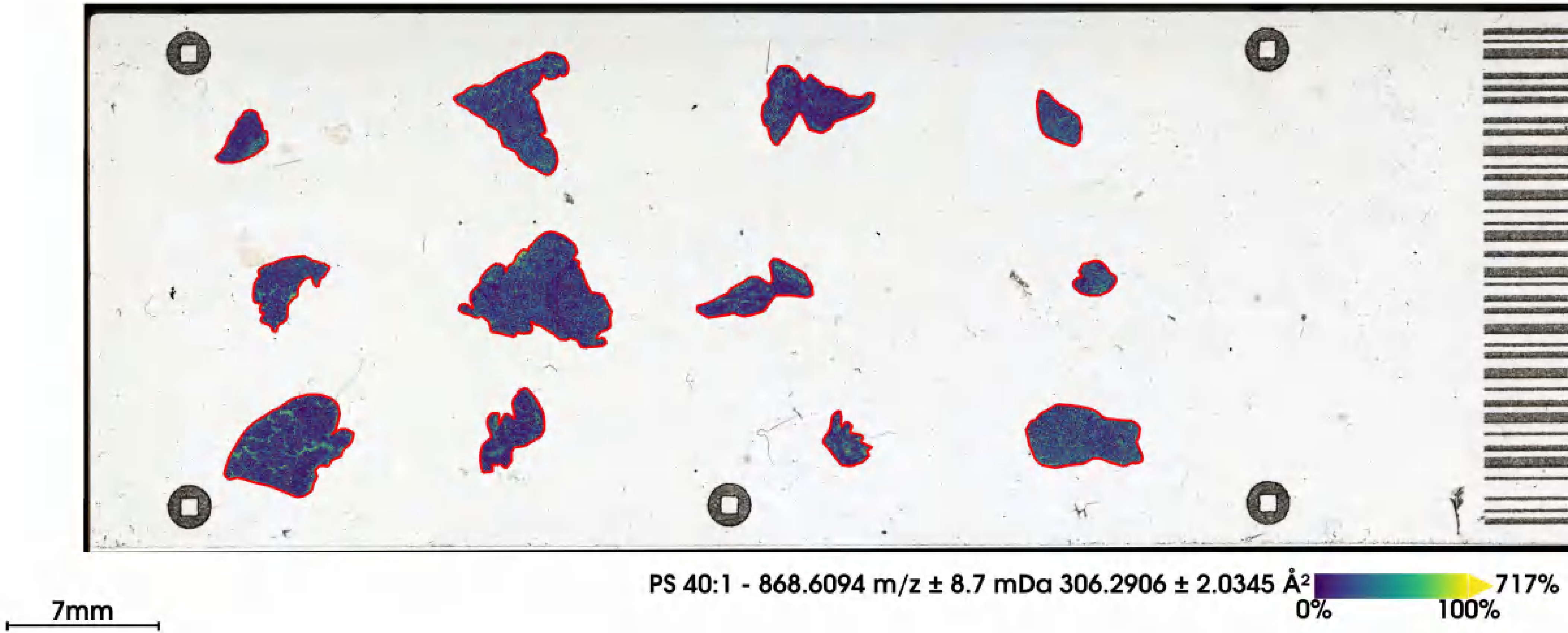
PS 40:1 - 868.6094 m/z ± 8.7 mDa 306.2906 ± 2.0345 Å²
0%
100%
717%
7mm

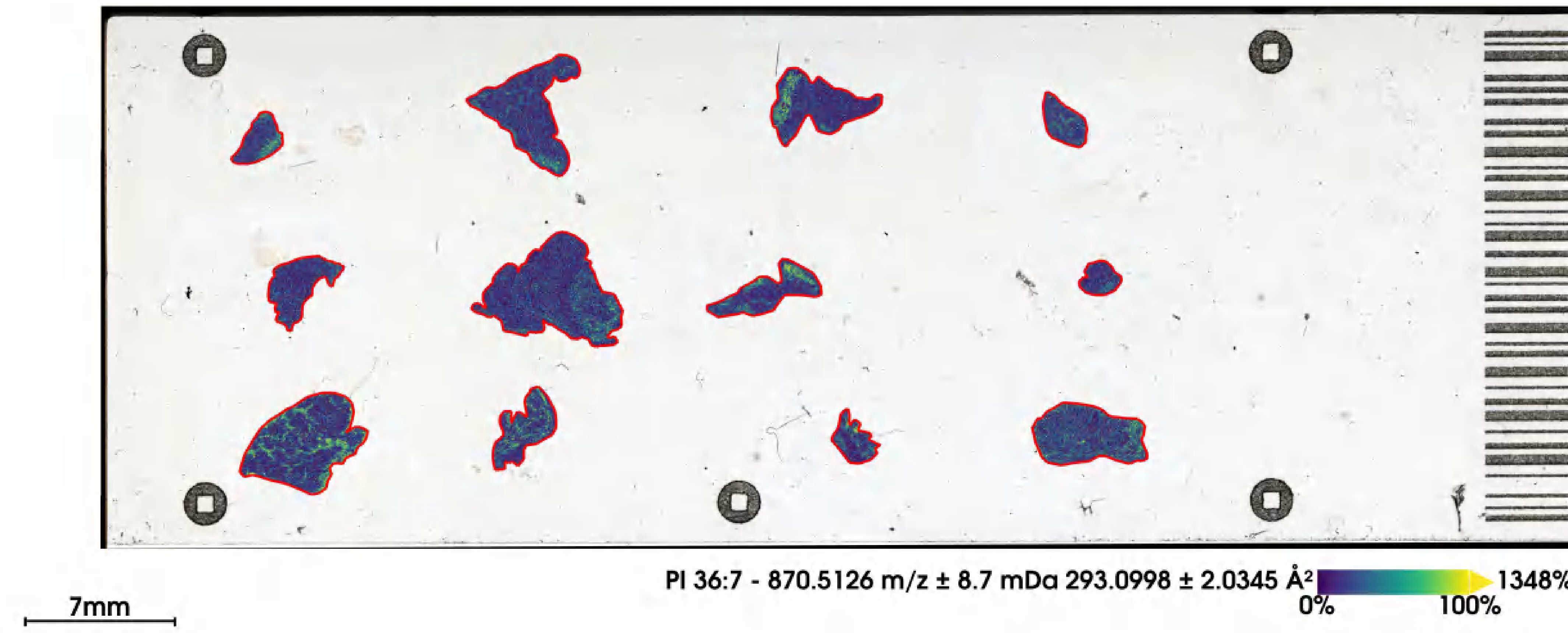
PI 36:7 - 870.5126 m/z ± 8.7 mDa 293.0998 ± 2.0345 Å²
1348%
0%
100%
7mm

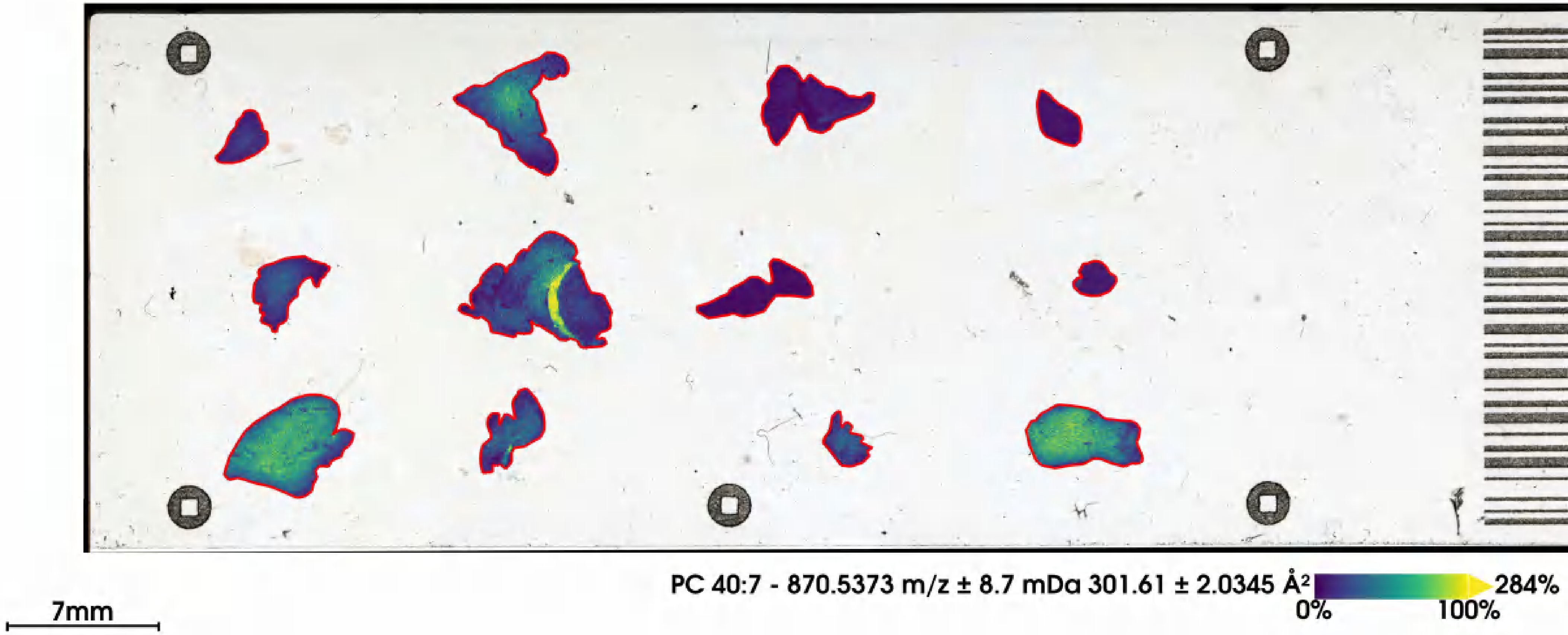
7mm
PC 40:7 - 870.5373 m/z ± 8.7 mDa 301.61 ± 2.0345 Å²
0%
100%
284%

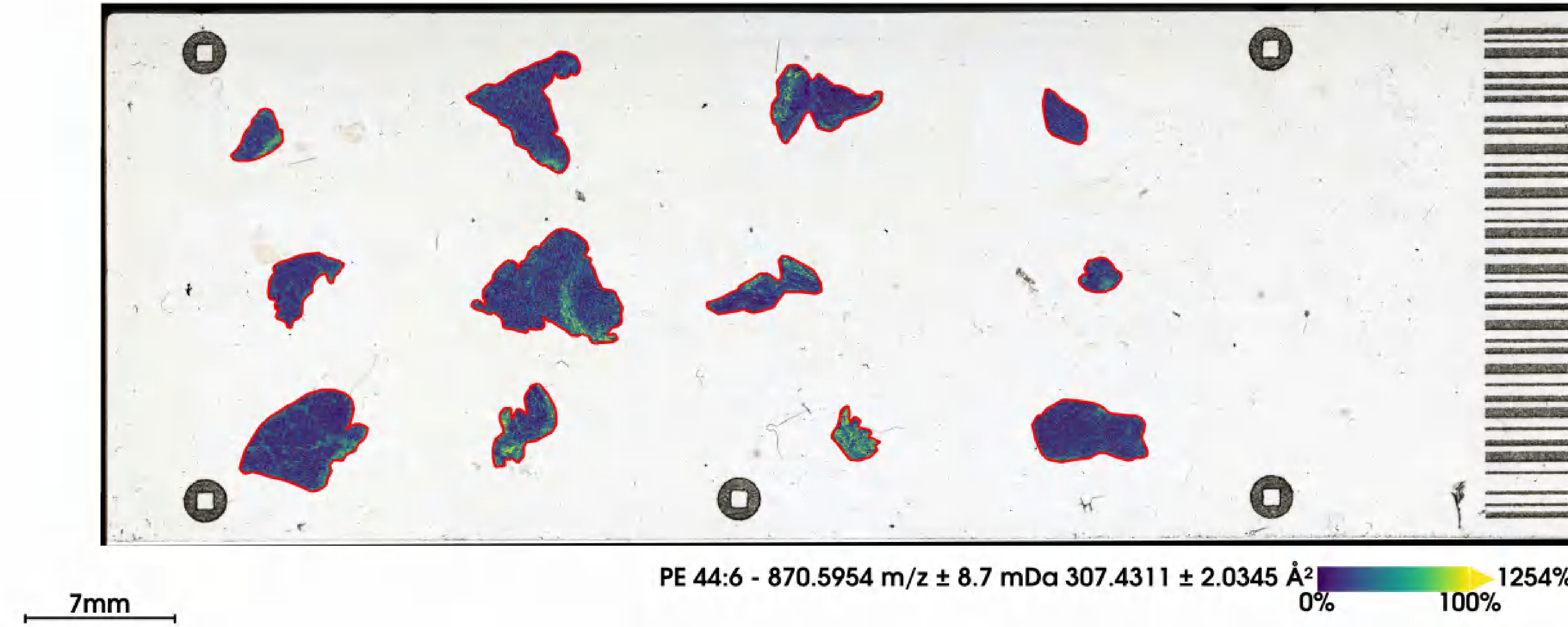
PE 44:6 - 870.5954 m/z ± 8.7 mDa 307.4311 ± 2.0345 Å²
1254%
0%
100%
7mm

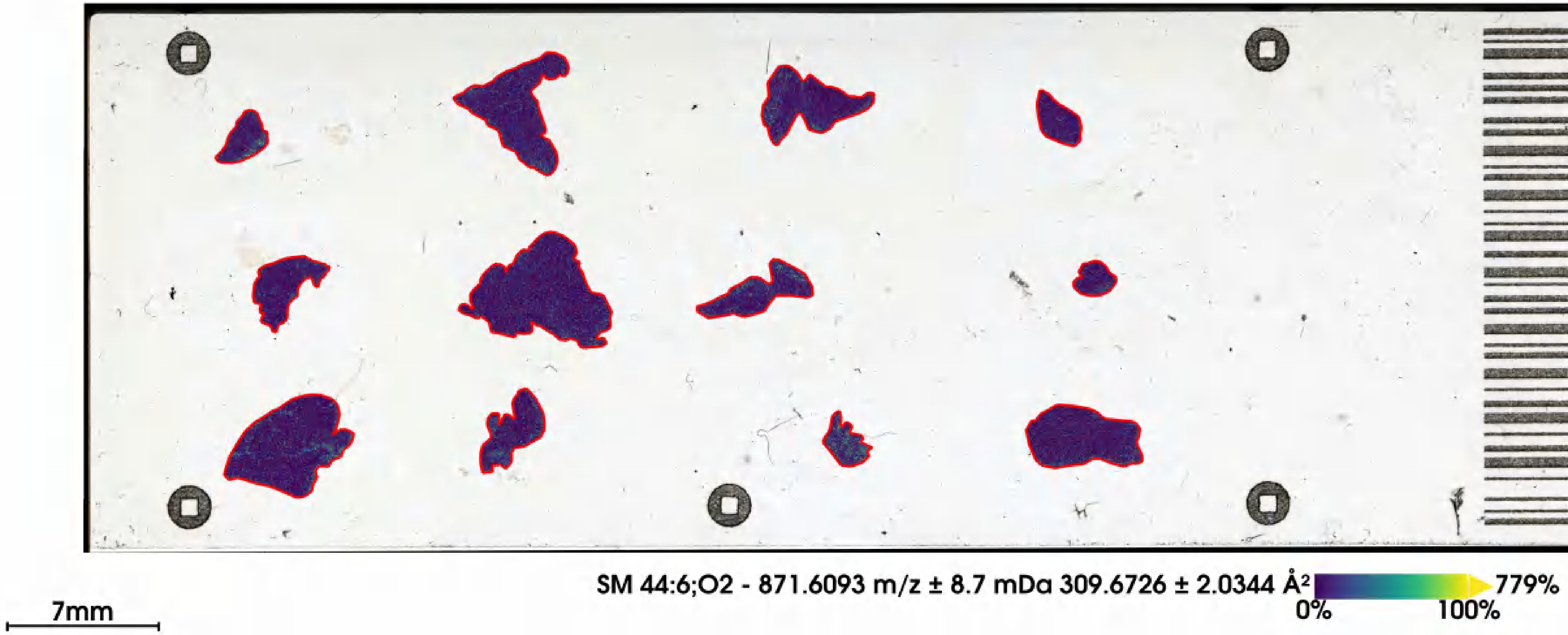

SM 44:6;O2 - 871.6093 m/z ± 8.7 mDa 309.6726 ± 2.0344 Å²
0%
100%
779%
7mm

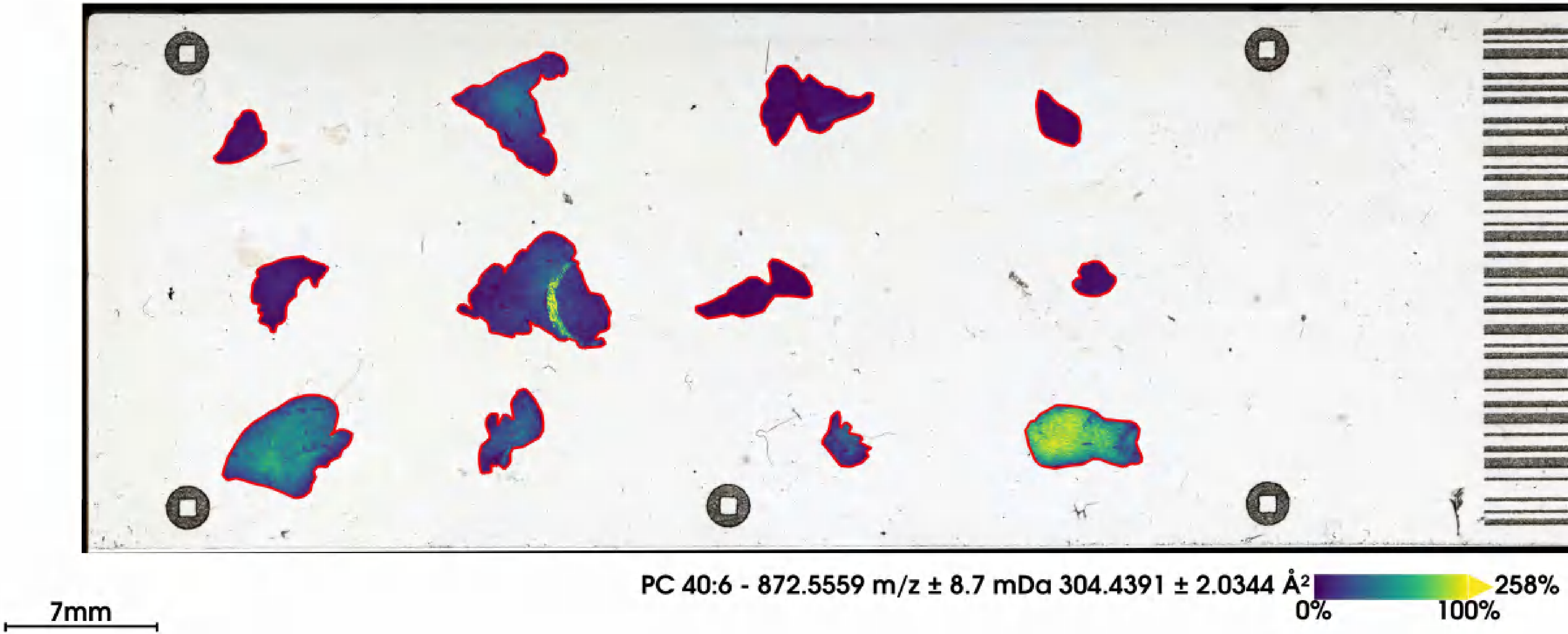
PC 40:6 - 872.5559 m/z ± 8.7 mDa 304.4391 ± 2.0344 Å²
0%
100%
258%
7mm

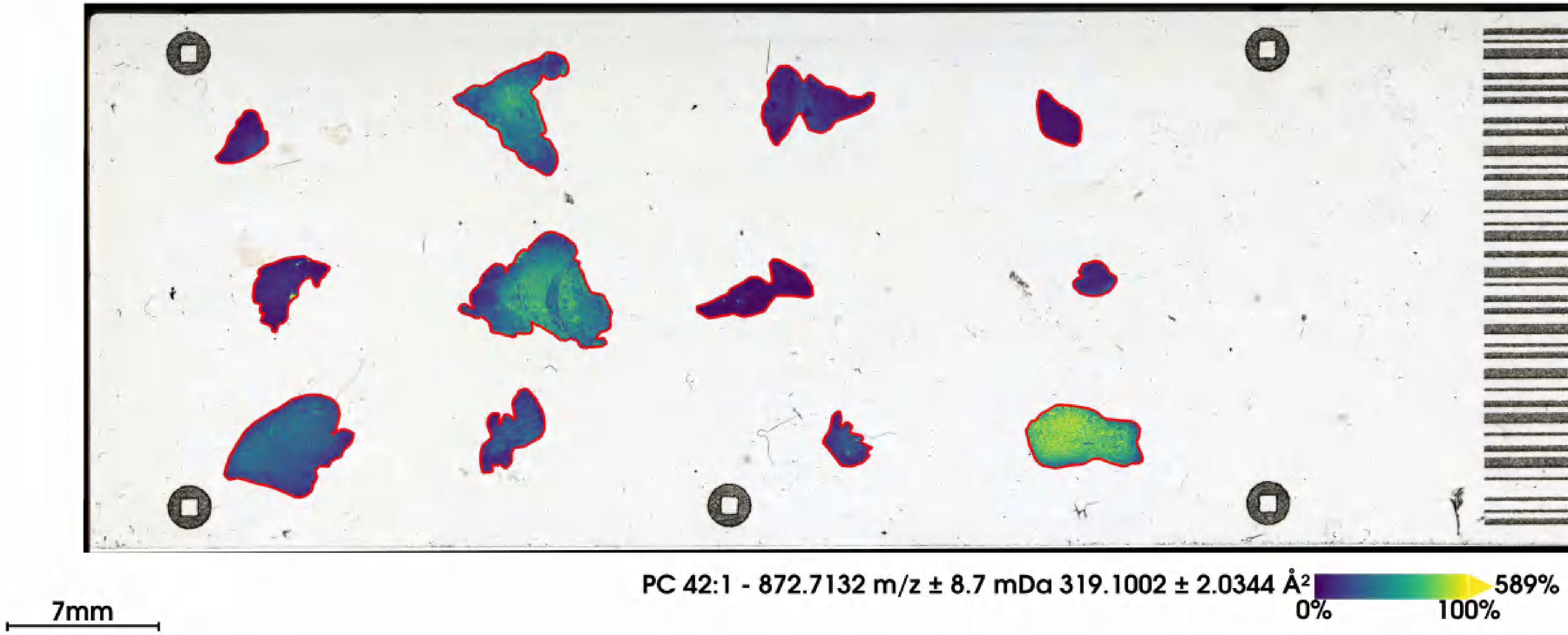
PC 42:1 - 872.7132 m/z ± 8.7 mDa 319.1002 ± 2.0344 Å²
0%
100%
589%
7mm

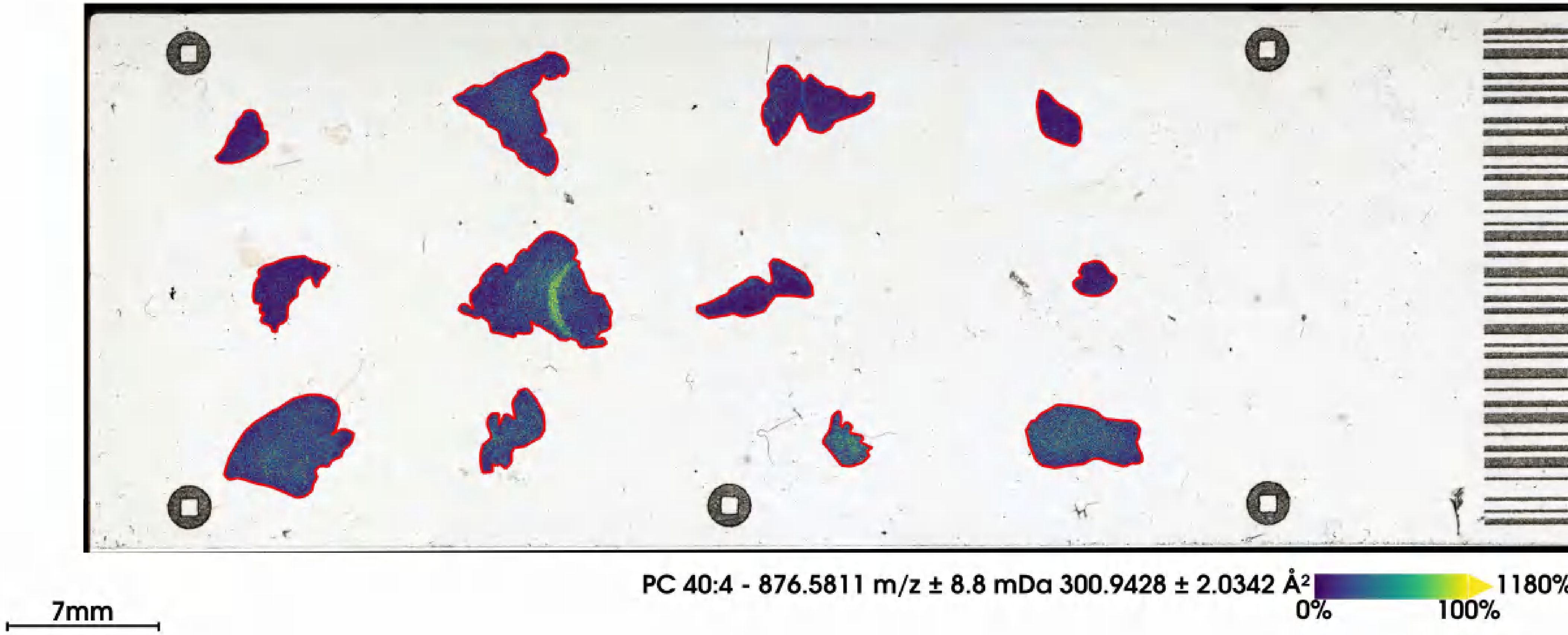
PC 40:4 - 876.5811 m/z ± 8.8 mDa 300.9428 ± 2.0342 Å²
0%
100%
1180%
7mm

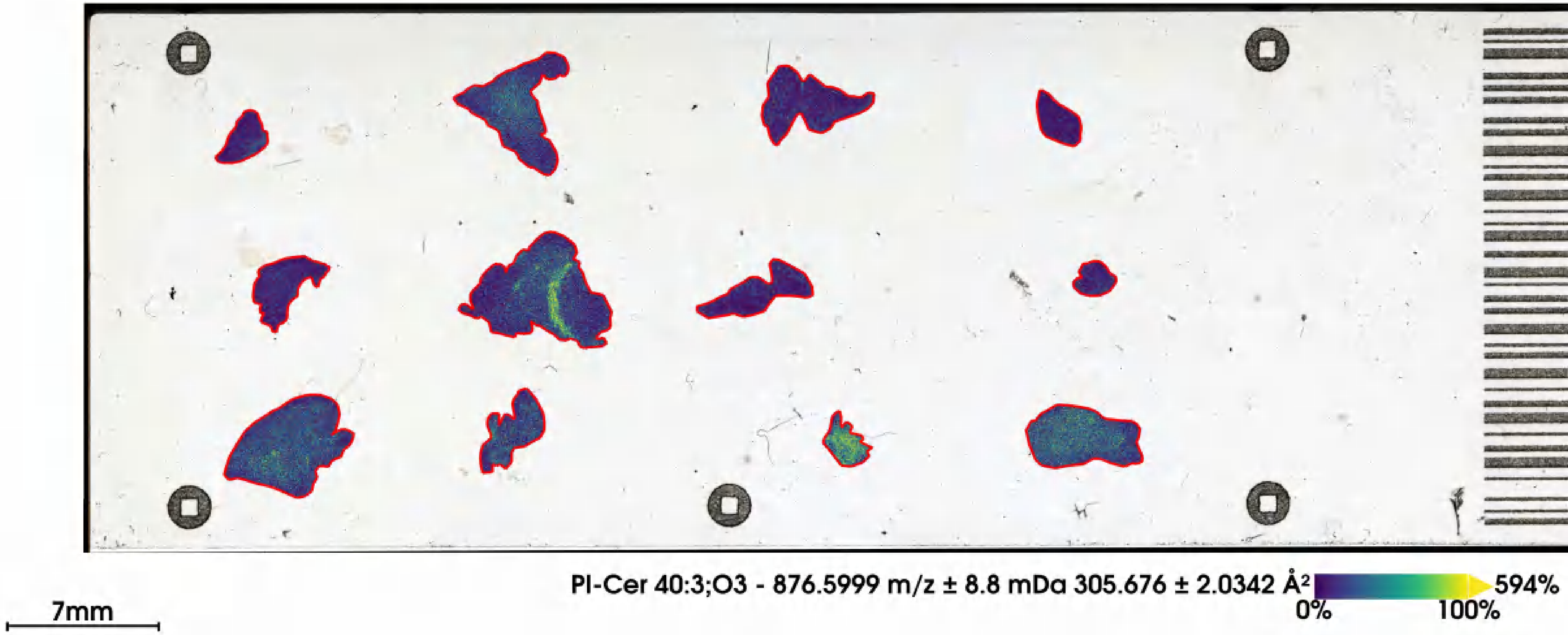
PI-Cer 40:3;O3 - 876.5999 m/z ± 8.8 mDa 305.676 ± 2.0342 Å²
0%
100%
594%
7mm

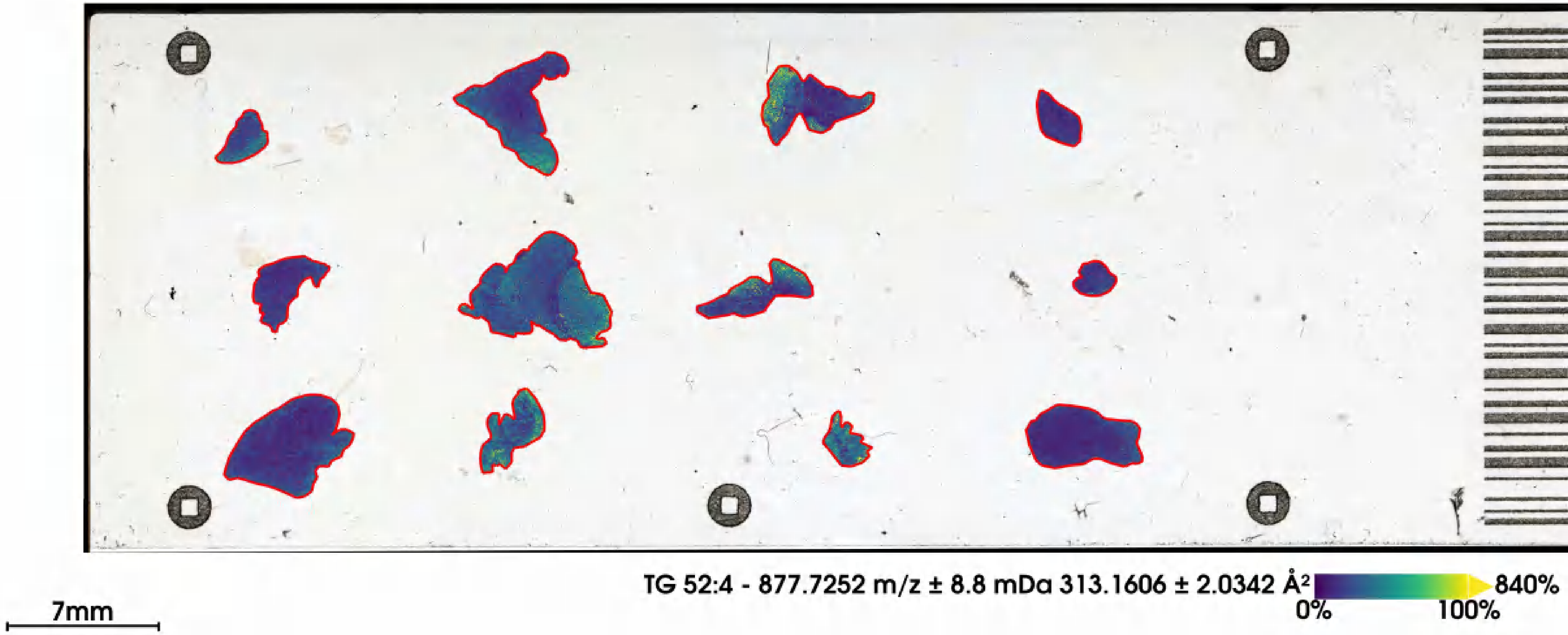

TG 52:4 - 877.7252 m/z ± 8.8 mDa 313.1606 ± 2.0342 Å²
0%
100%
840%
7mm

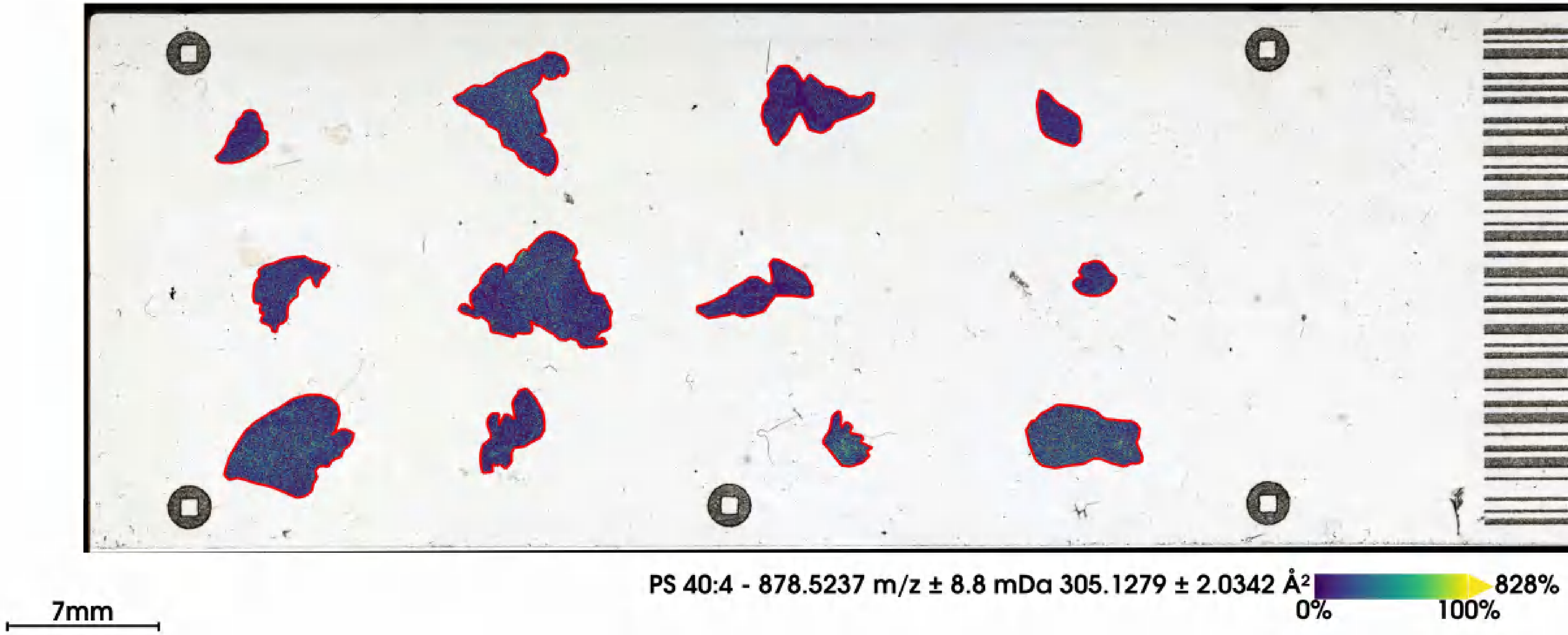

PS 40:4 - 878.5237 m/z ± 8.8 mDa 305.1279 ± 2.0342 Å²
0%
100%
828%
7mm

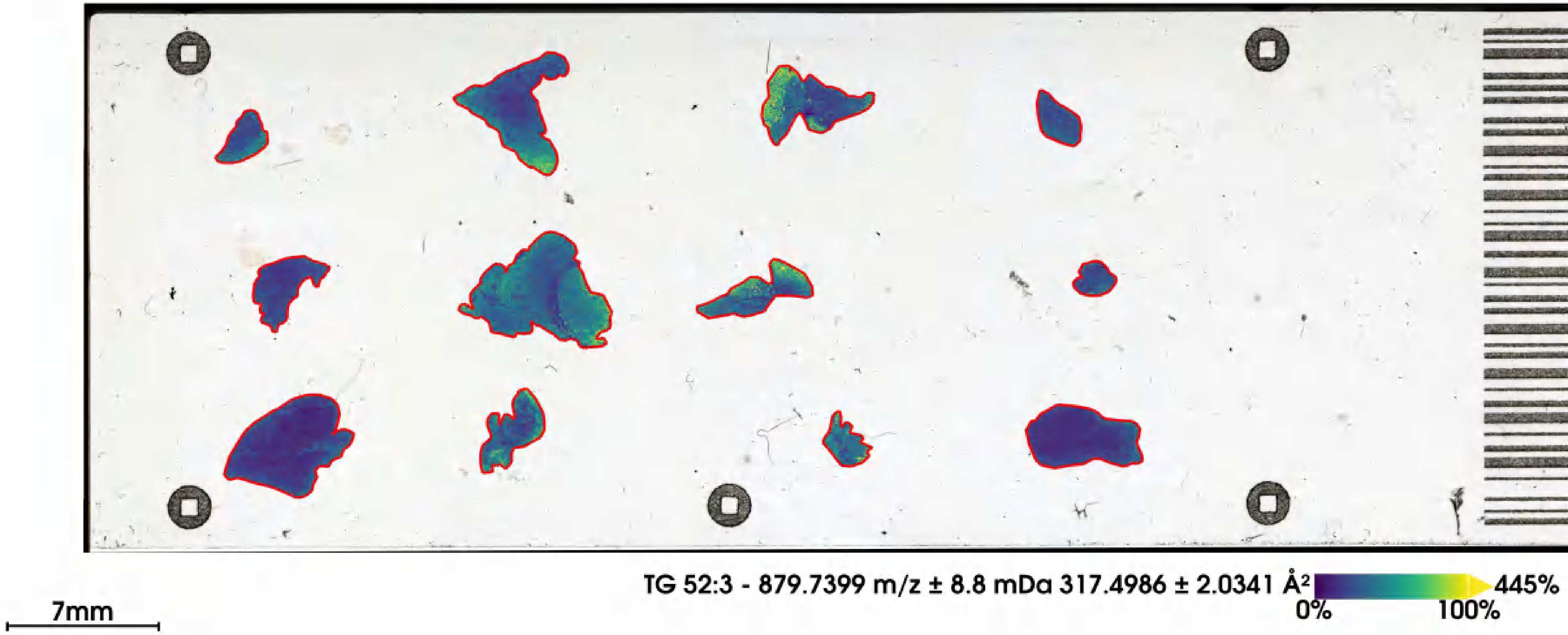

TG 52:3 - 879.7399 m/z ± 8.8 mDa 317.4986 ± 2.0341 Å²
0%
100%
445%
7mm

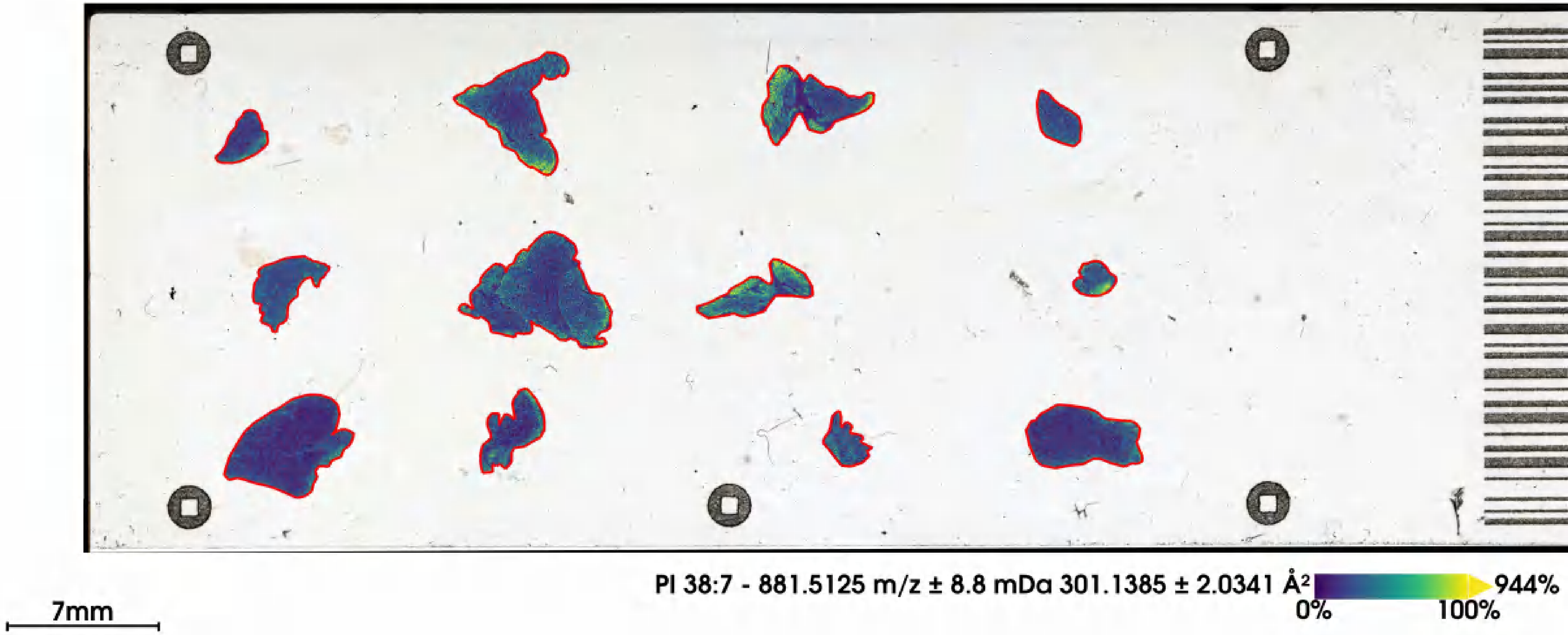
PI 38:7 - 881.5125 m/z ± 8.8 mDa 301.1385 ± 2.0341 Å²
0%
100%
944%
7mm

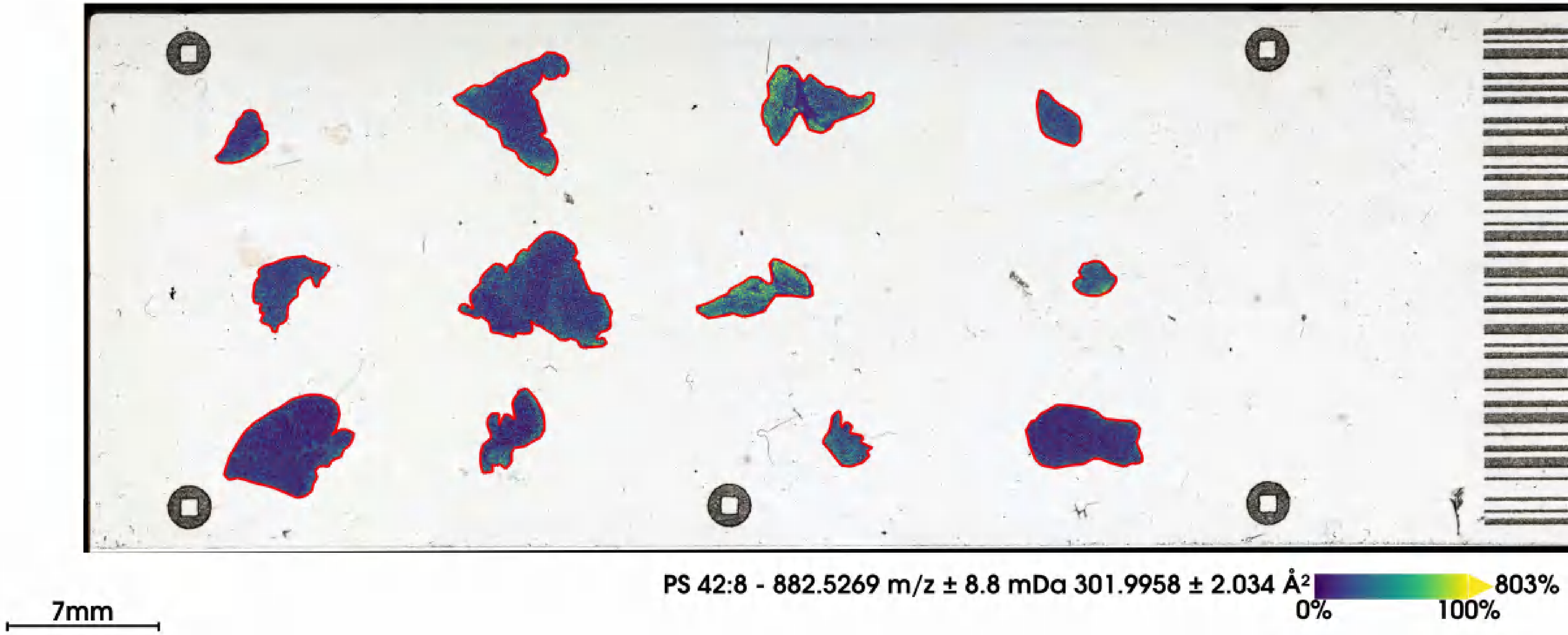

PS 42:8 - 882.5269 m/z ± 8.8 mDa 301.9958 ± 2.034 Å²
0%
100%
803%
7mm

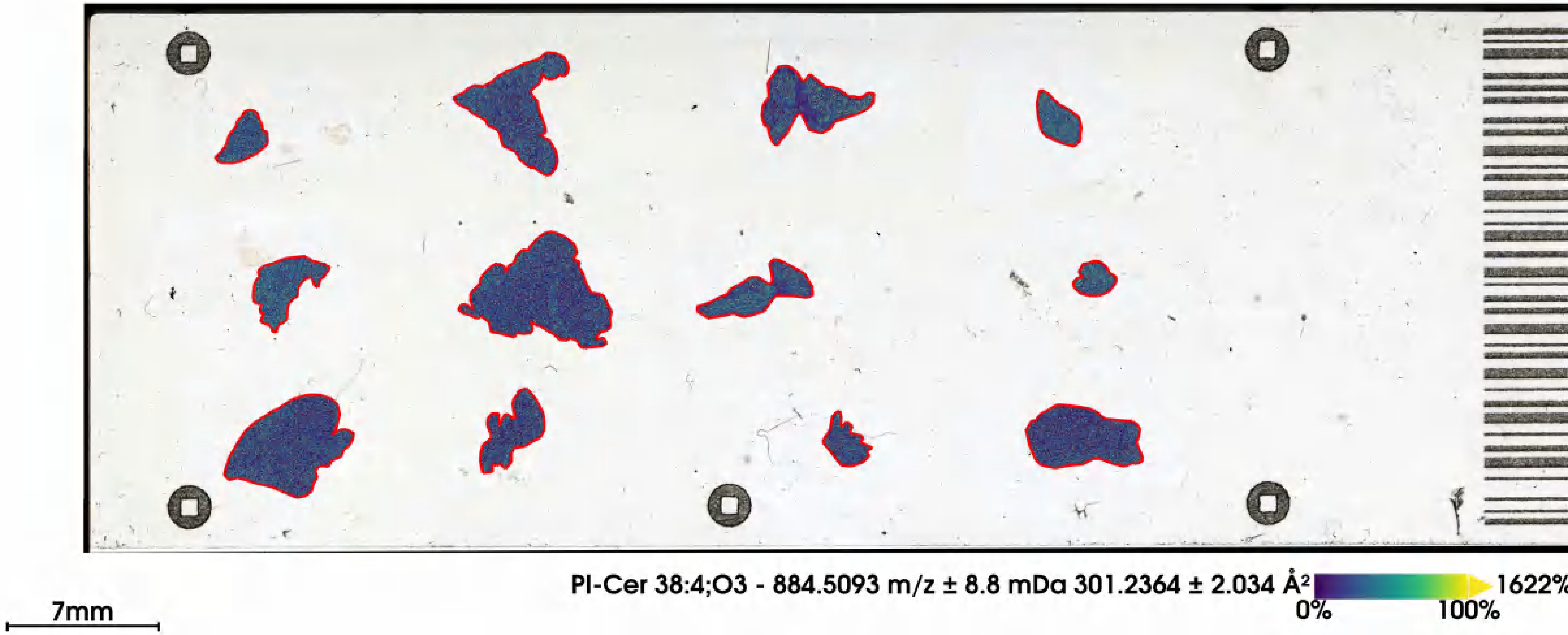
PI-Cer 38:4;O3 - 884.5093 m/z ± 8.8 mDa 301.2364 ± 2.034 Å²
1622%
0%
100%
7mm

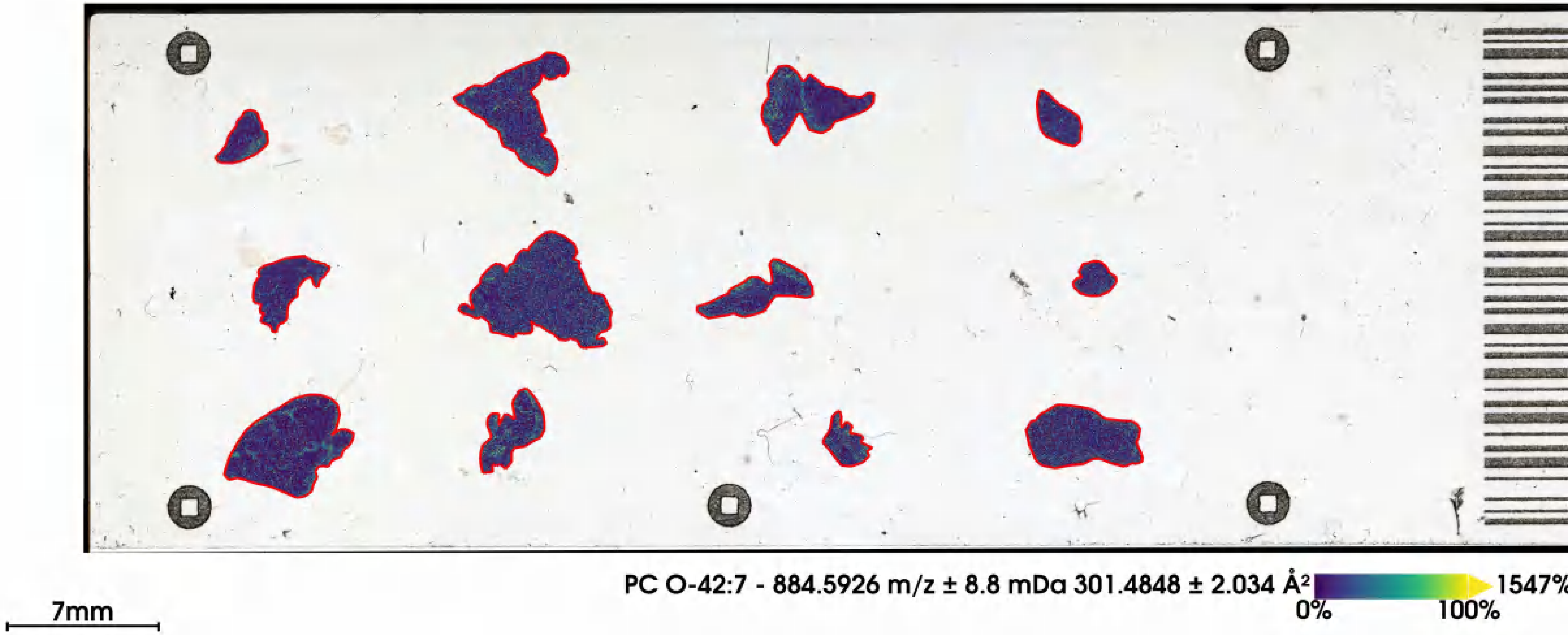
PC O-42:7 - 884.5926 m/z ± 8.8 mDa 301.4848 ± 2.034 Å²
0%
100%
1547%
7mm

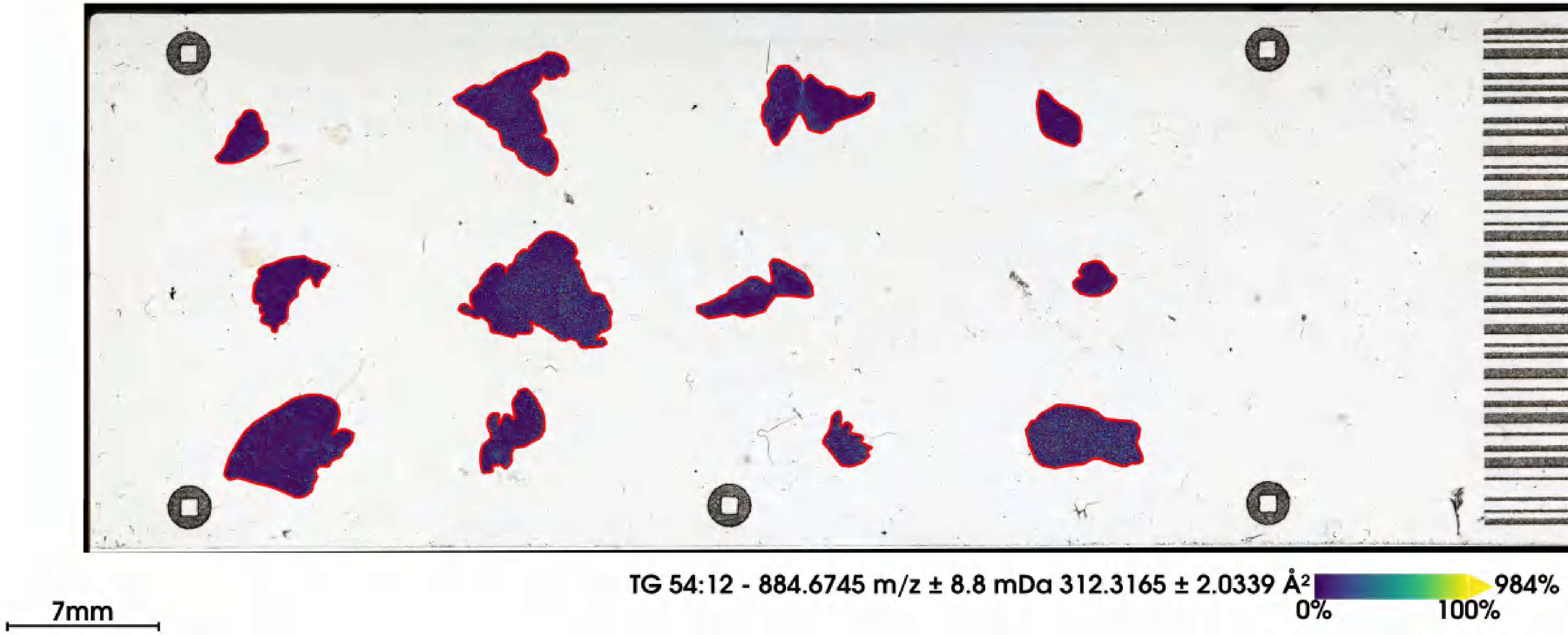

TG 54:12 - 884.6745 m/z ± 8.8 mDa 312.3165 ± 2.0339 Å²
0%
100%
984%
7mm

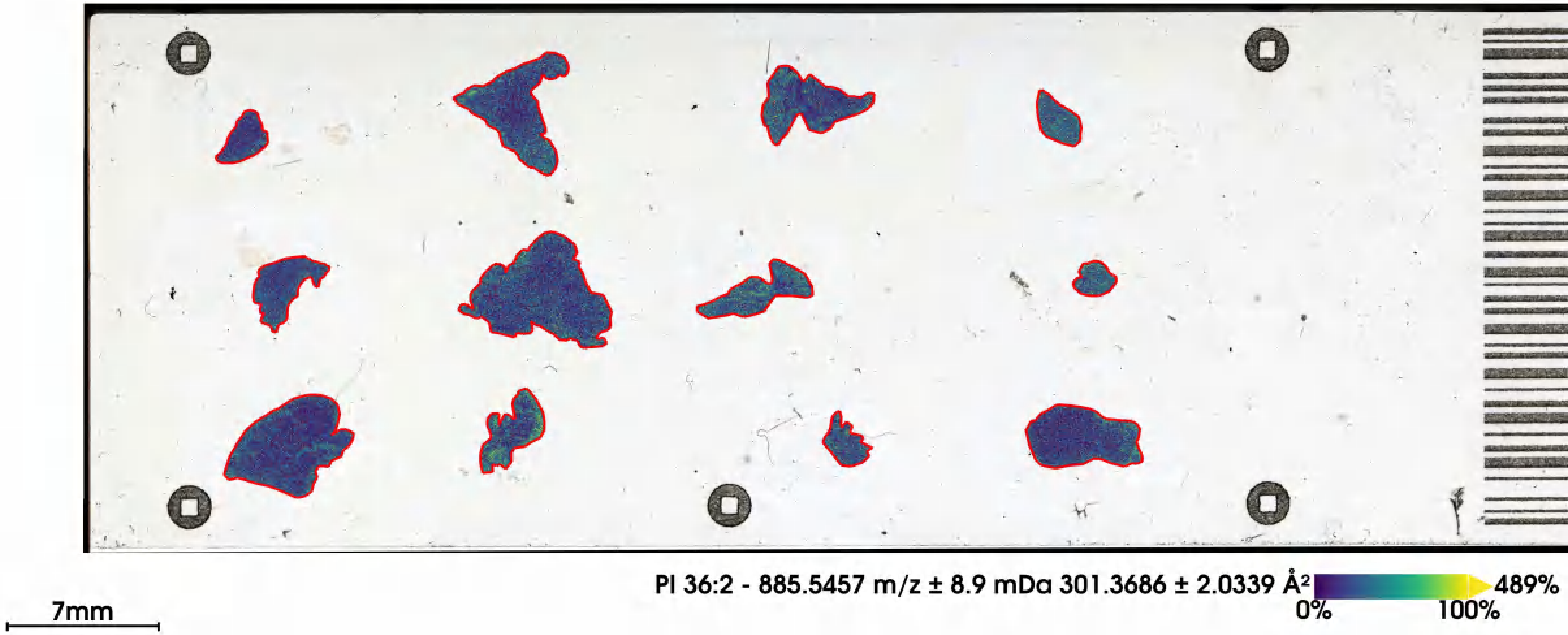
PI 36:2 - 885.5457 m/z ± 8.9 mDa 301.3686 ± 2.0339 Å²
0%
100%
489%
7mm

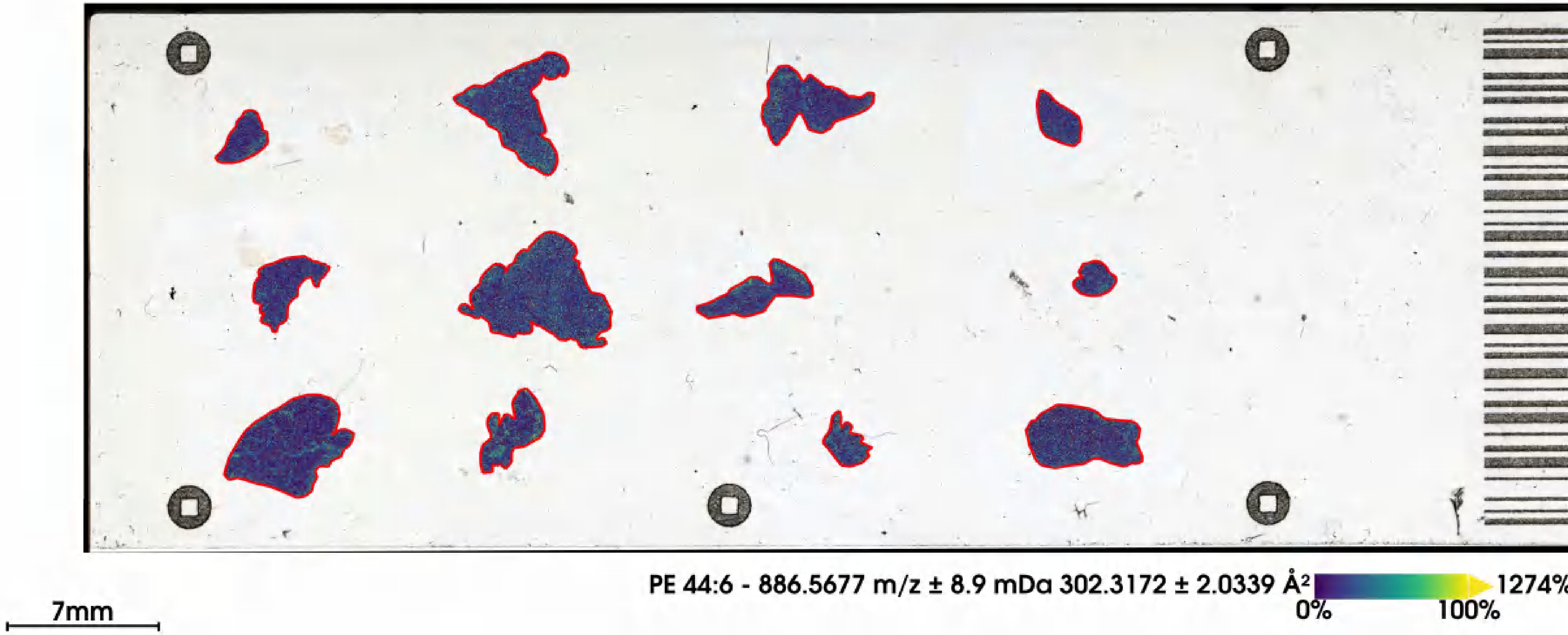
PE 44:6 - 886.5677 m/z ± 8.9 mDa 302.3172 ± 2.0339 Å²
0%
100%
1274%
7mm

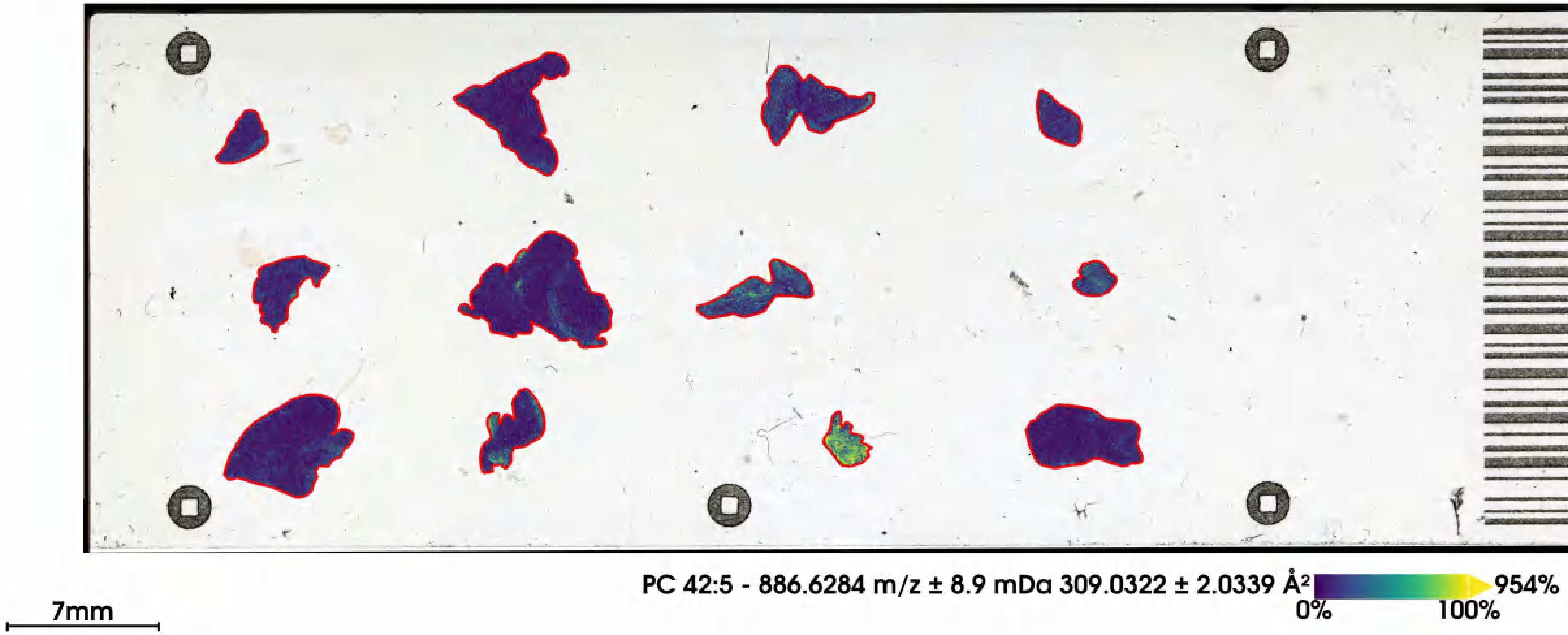

PC 42:5 - 886.6284 m/z ± 8.9 mDa 309.0322 ± 2.0339 Å²
0%
100%
954%
7mm

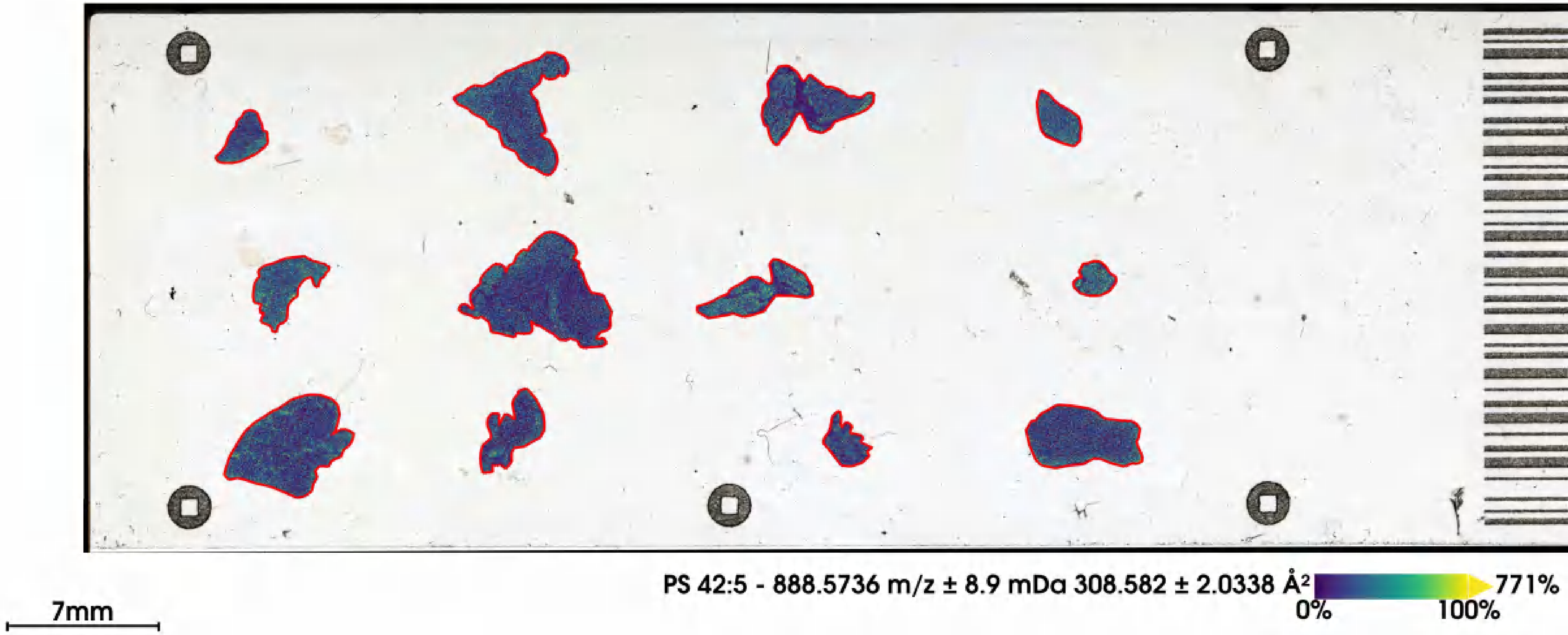

PS 42:5 - 888.5736 m/z ± 8.9 mDa 308.582 ± 2.0338 Å²
771%
0%
100%
7mm

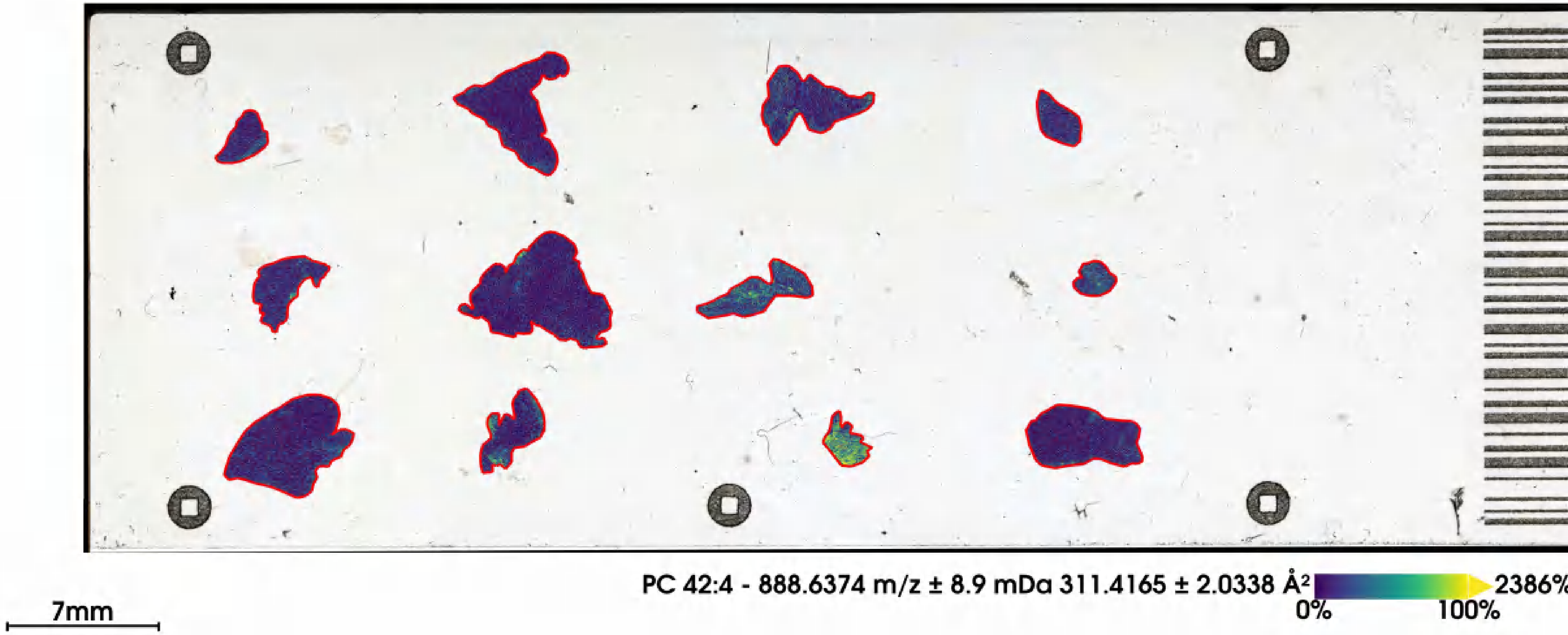

PC 42:4 - 888.6374 m/z ± 8.9 mDa 311.4165 ± 2.0338 Å²
0%
100%
2386%
7mm

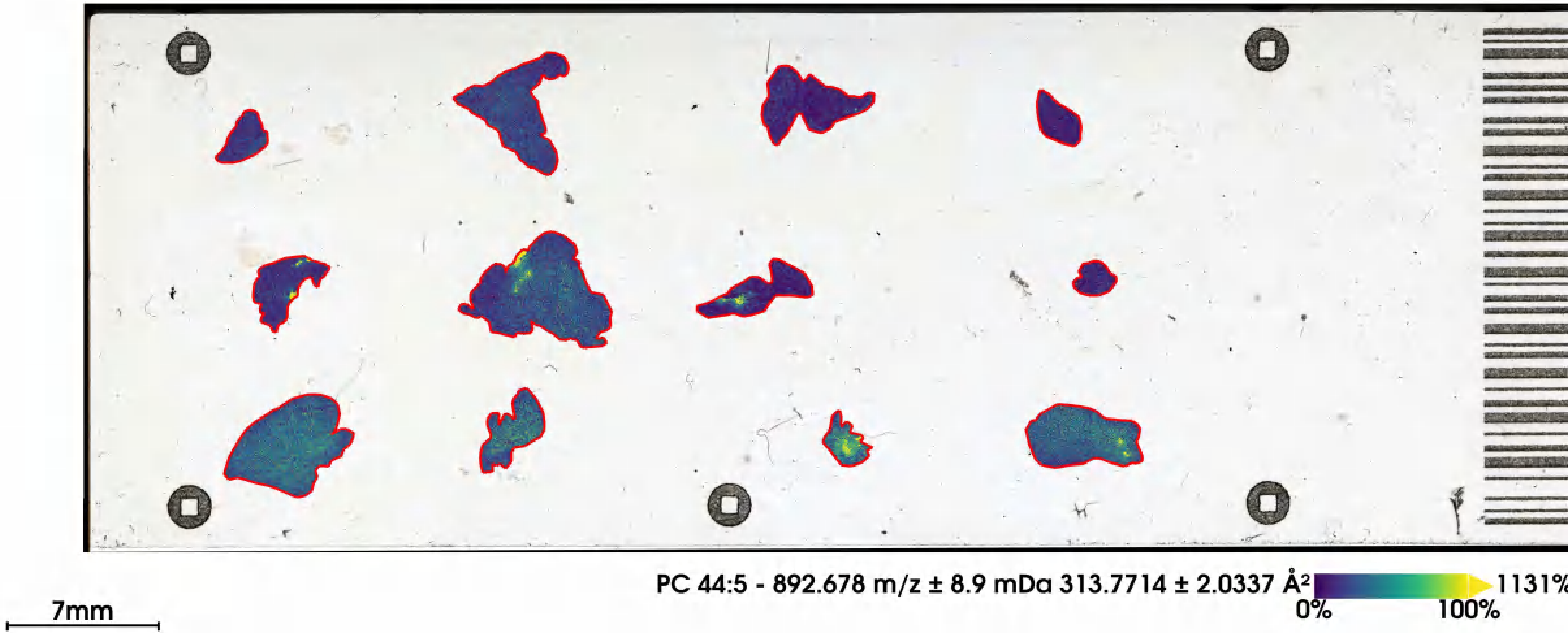

PC 44:5 - 892.678 m/z ± 8.9 mDa 313.7714 ± 2.0337 Å²
0%
100%
1131%
7mm

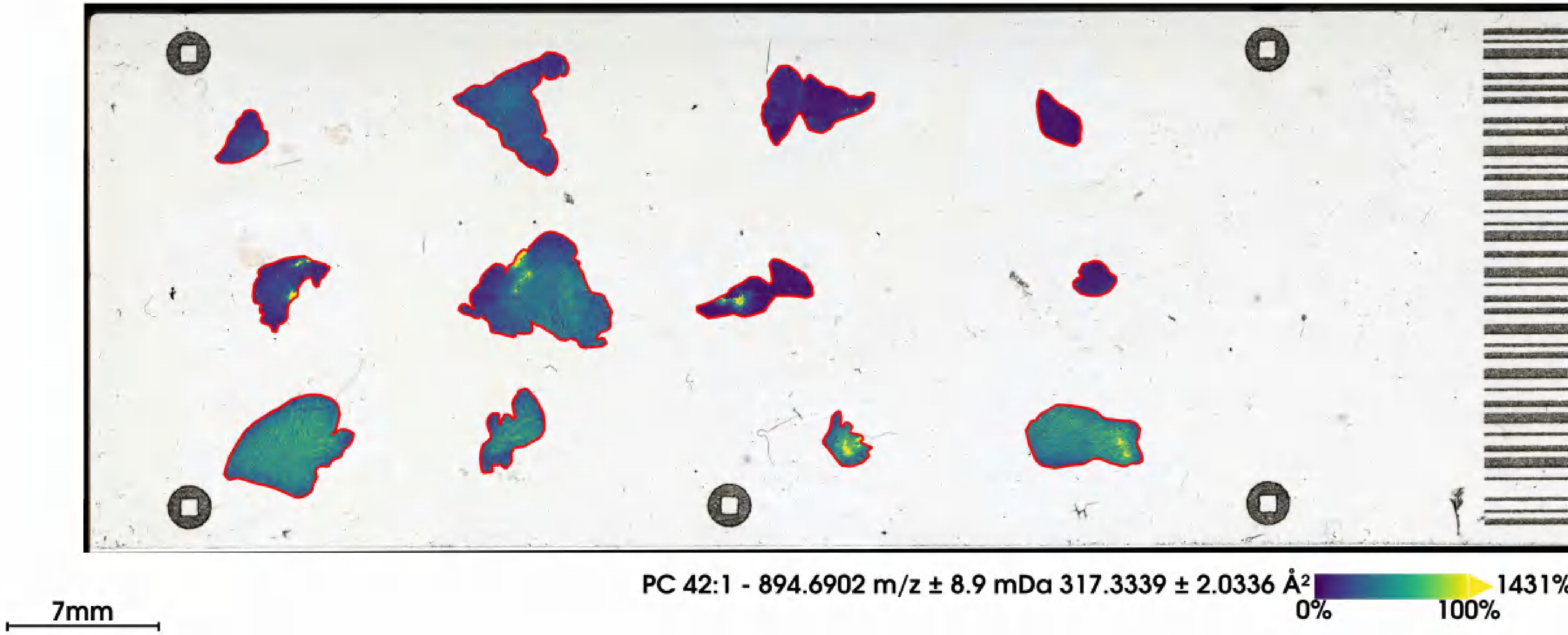
PC 42:1 - 894.6902 m/z ± 8.9 mDa 317.3339 ± 2.0336 Å²
0%
100%
1431%
7mm

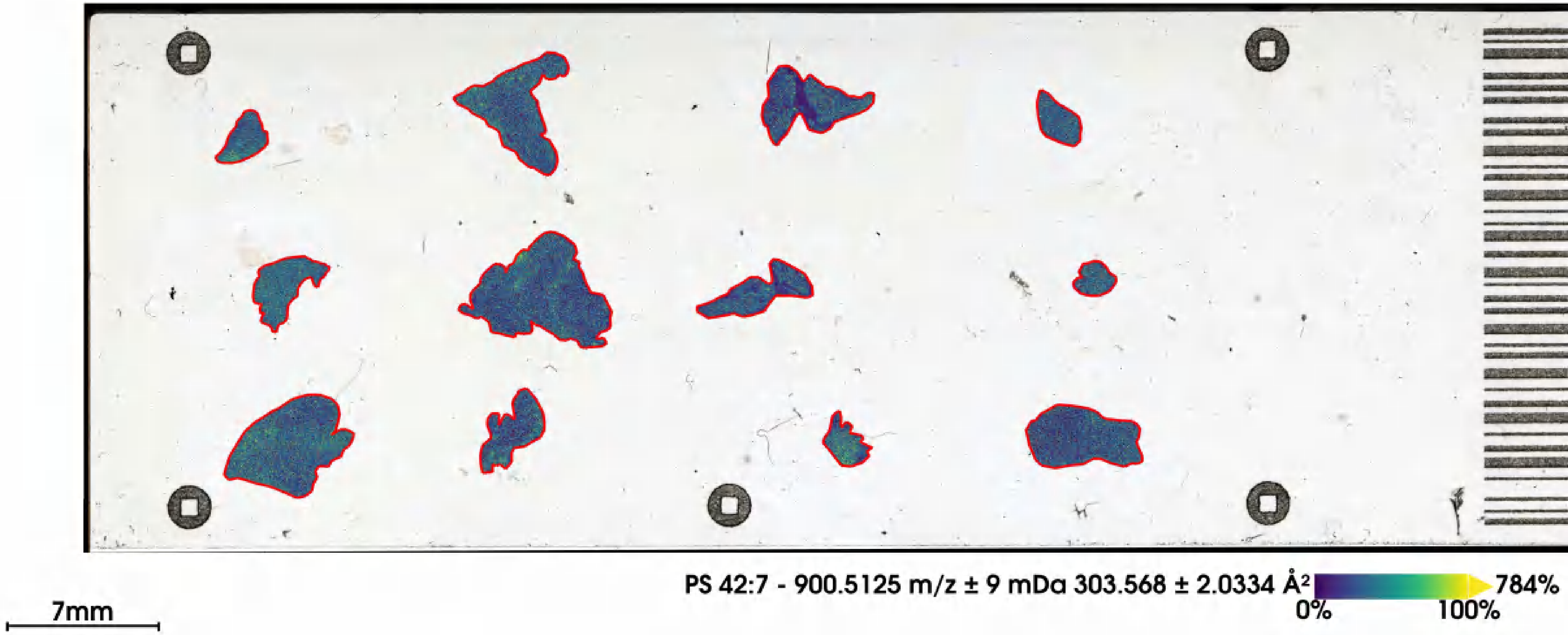
PS 42:7 - 900.5125 m/z ± 9 mDa 303.568 ± 2.0334 Å²
784%
0%
100%
7mm

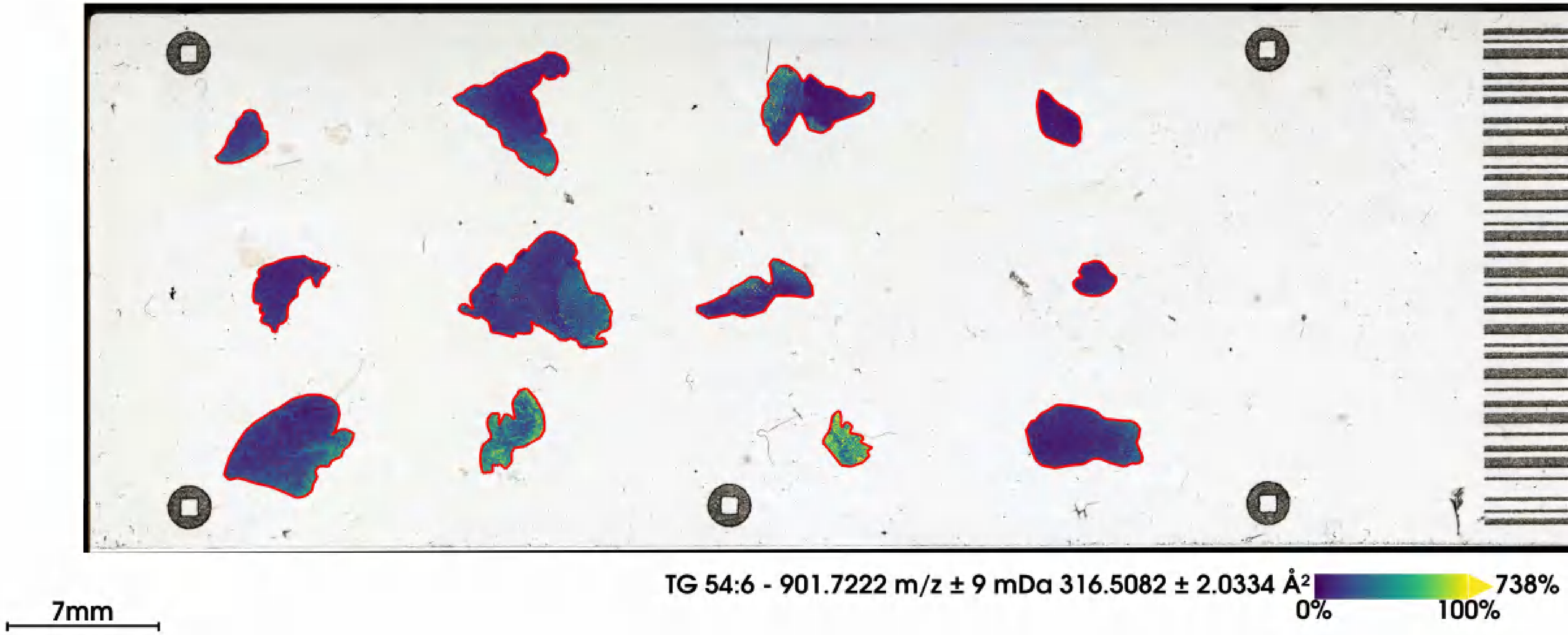
7mm
TG 54:6 - 901.7222 m/z ± 9 mDa 316.5082 ± 2.0334 Å²
0%
100%
738%

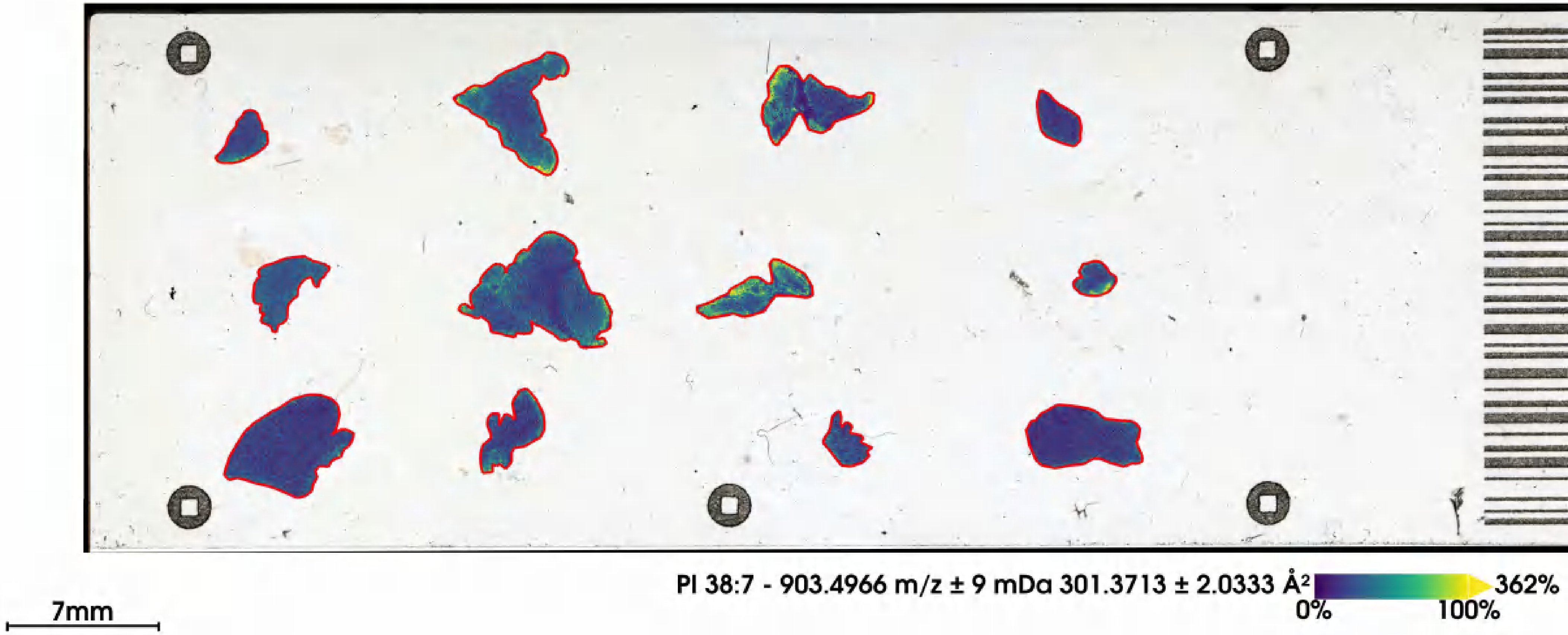
PI 38:7 - 903.4966 m/z ± 9 mDa 301.3713 ± 2.0333 Å²
0%
100%
362%
7mm

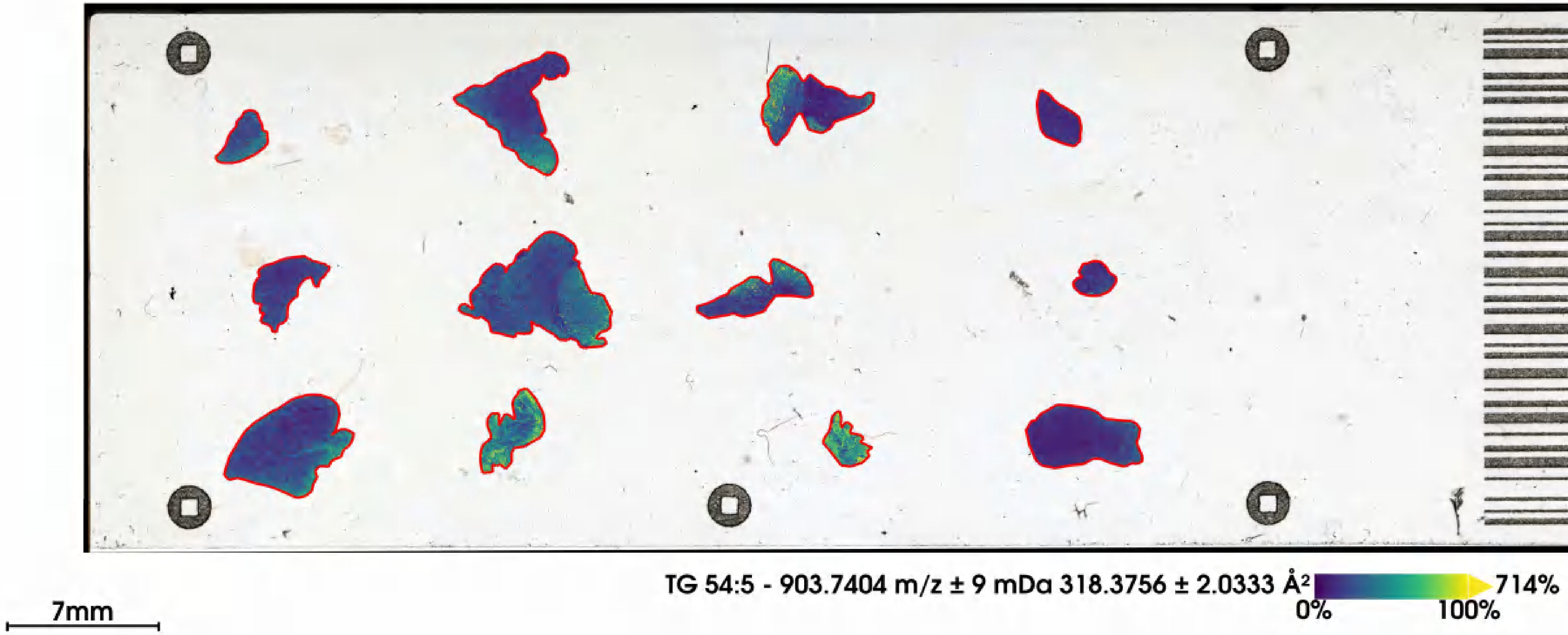

TG 54:5 - 903.7404 m/z ± 9 mDa 318.3756 ± 2.0333 Å²
0%
100%
714%
7mm

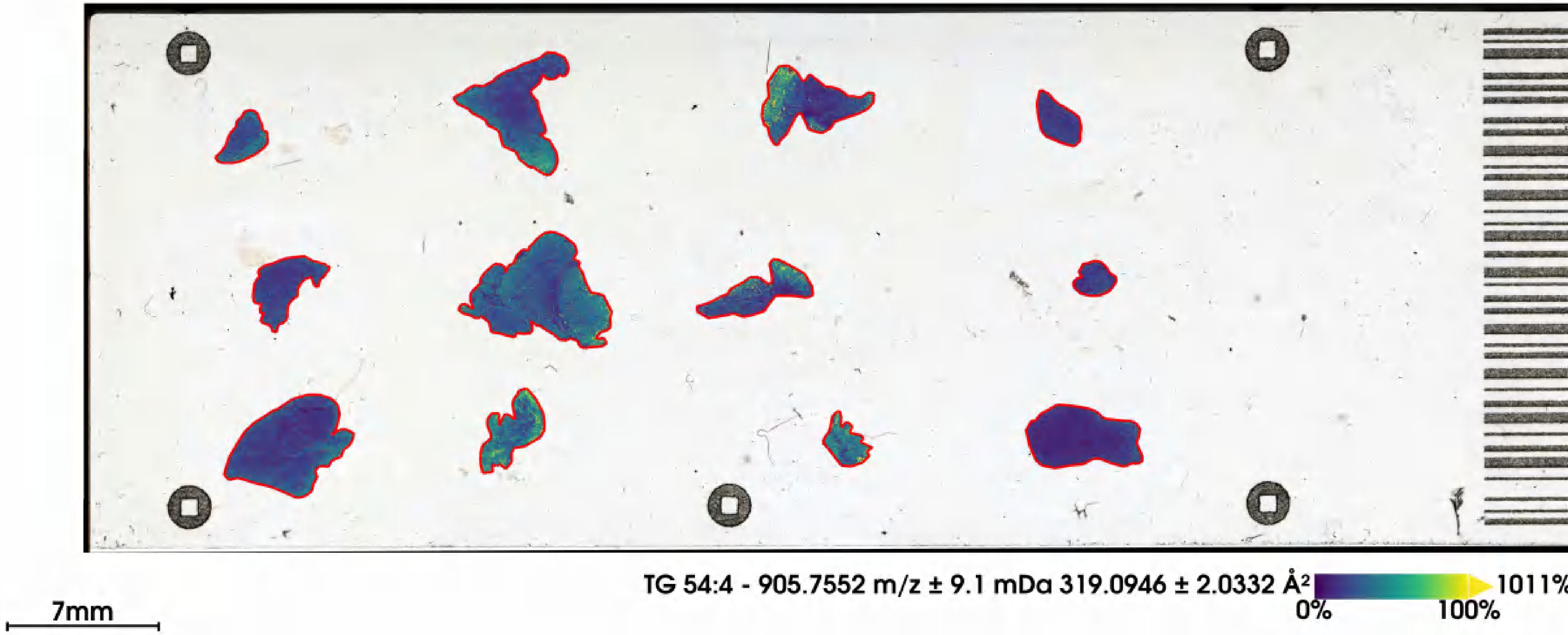
TG 54:4 - 905.7552 m/z ± 9.1 mDa 319.0946 ± 2.0332 Å²
0%
100%
1011%
7mm

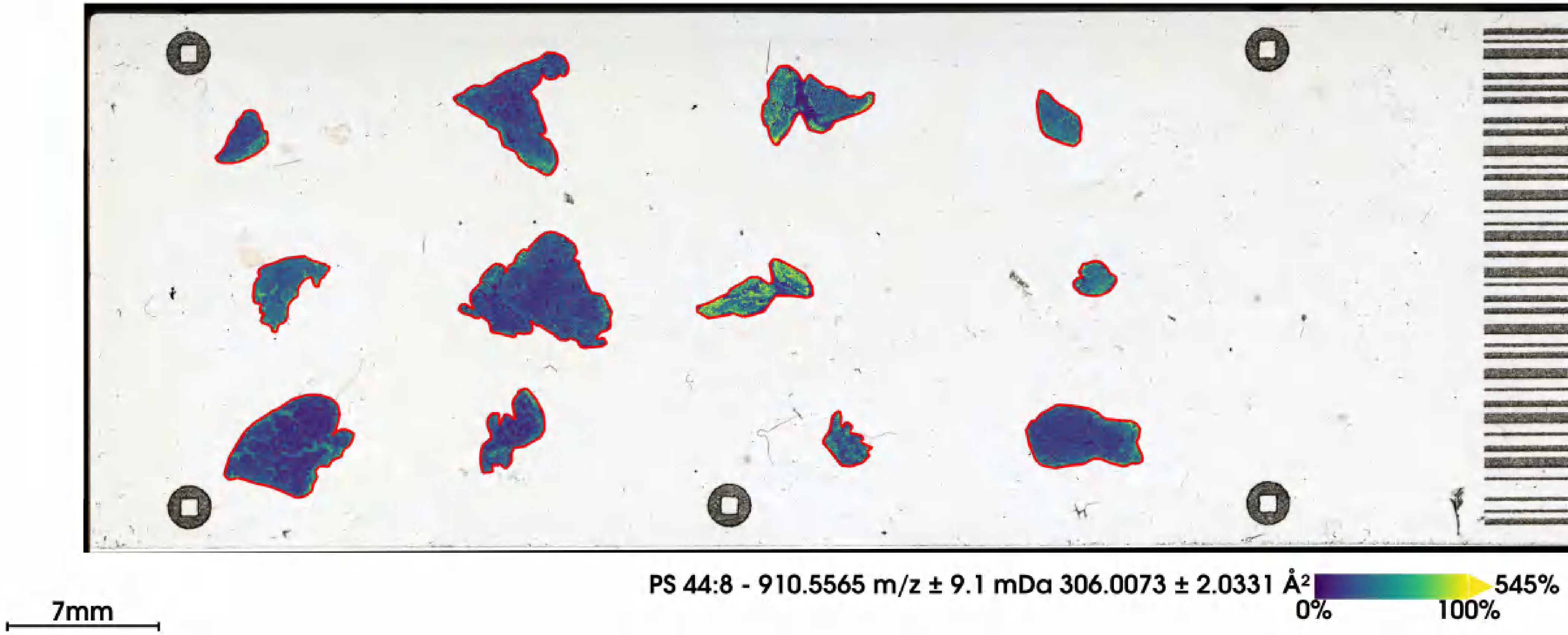

PS 44:8 - 910.5565 m/z ± 9.1 mDa 306.0073 ± 2.0331 Å²
0%
100%
545%
7mm

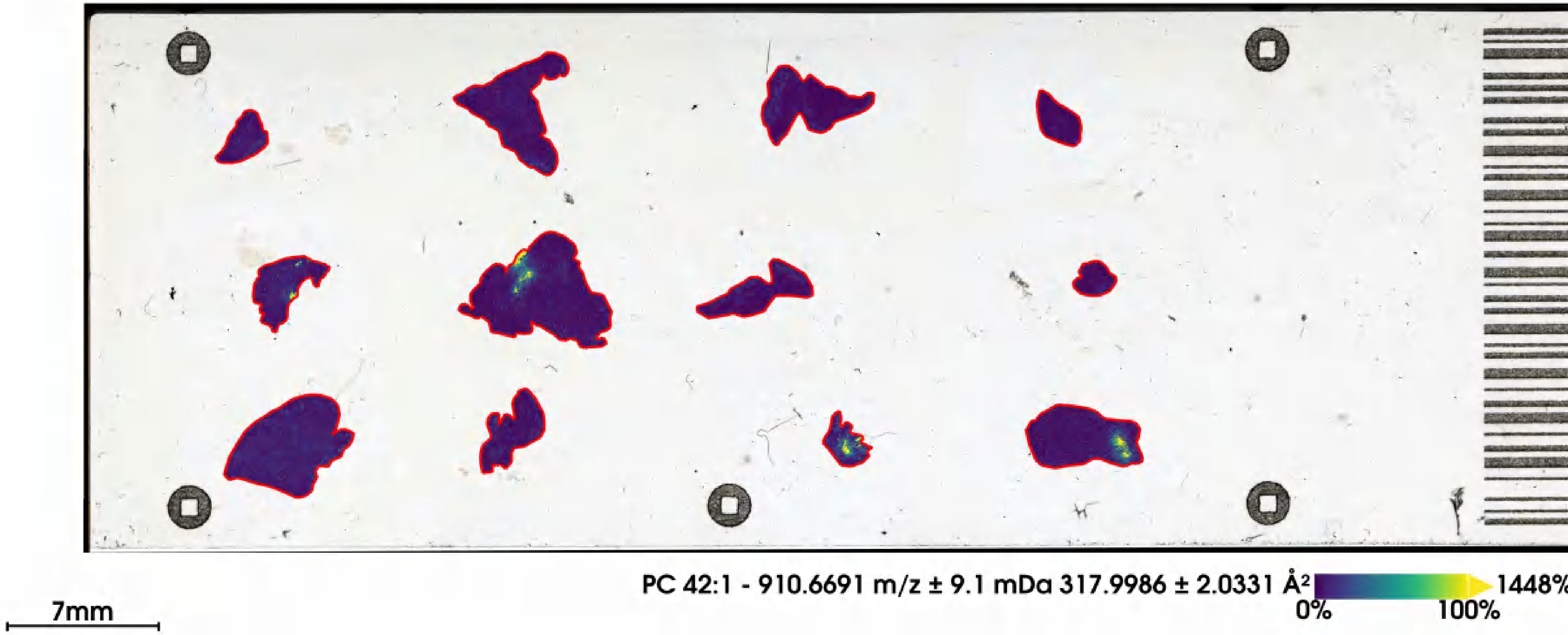

PC 42:1 - 910.6691 m/z ± 9.1 mDa 317.9986 ± 2.0331 Å²
0%
100%
1448%
7mm

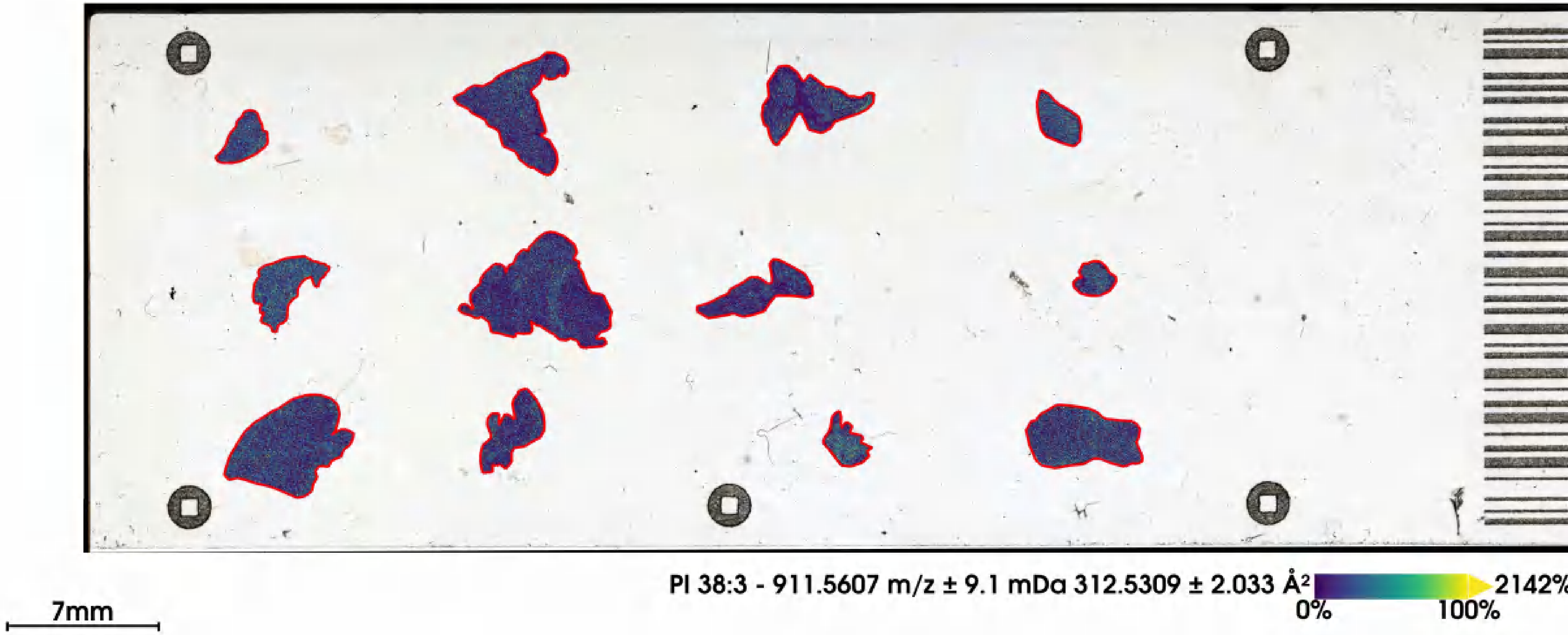

PI 38:3 - 911.5607 m/z ± 9.1 mDa 312.5309 ± 2.033 Å²
0%
100%
2142%
7mm

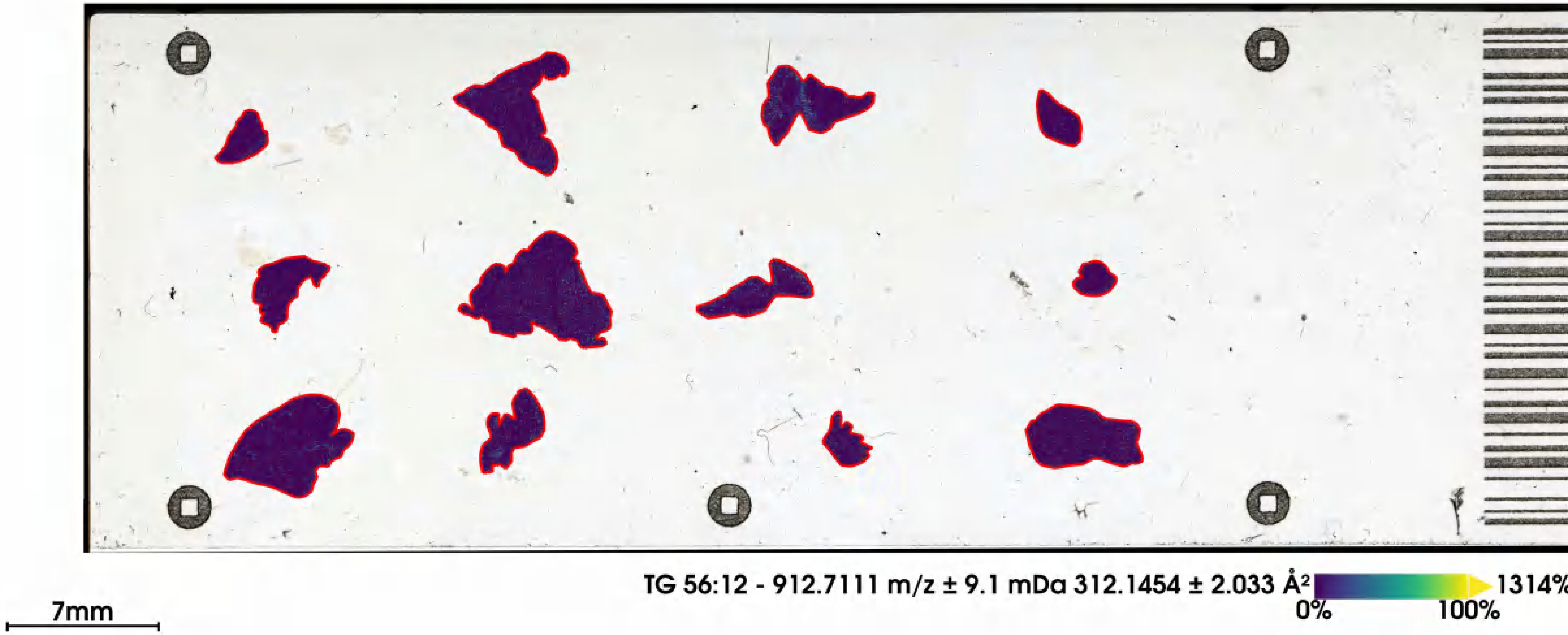
TG 56:12 - 912.7111 m/z ± 9.1 mDa 312.1454 ± 2.033 Å²
0%
100%
1314%
7mm

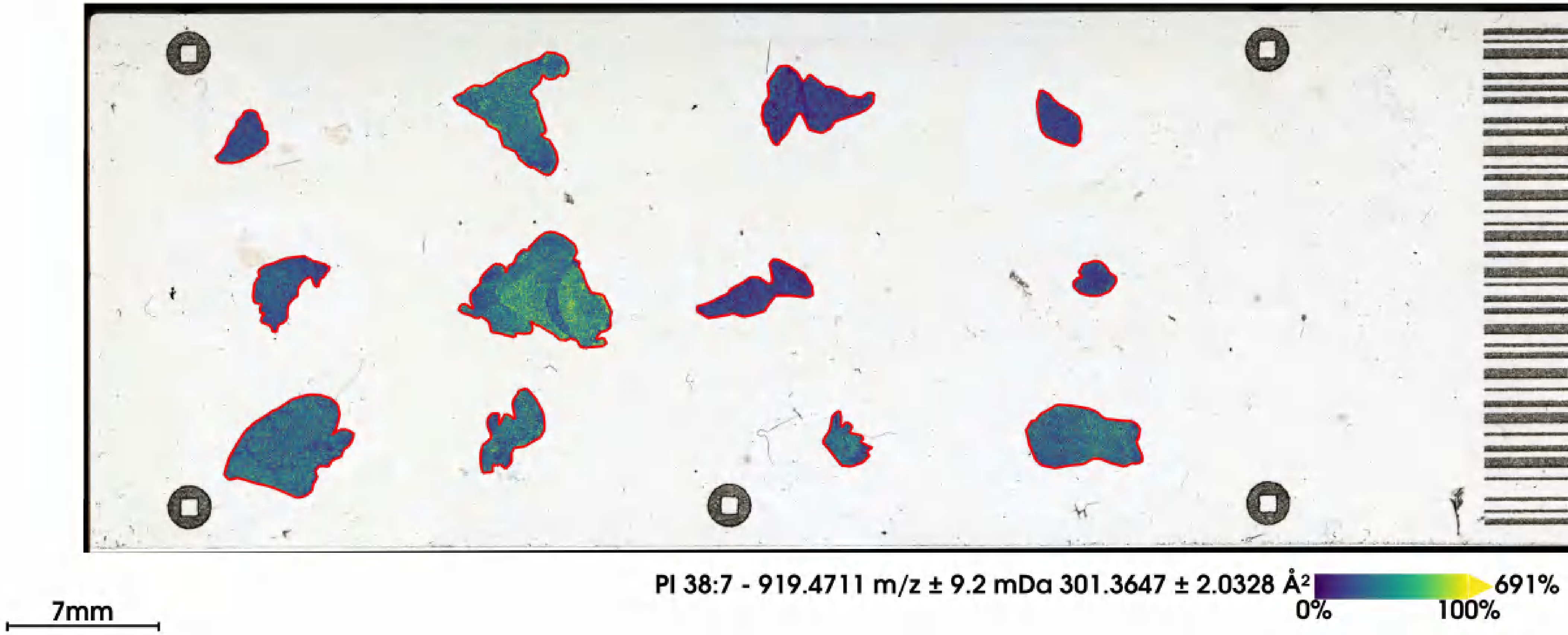

PI 38:7 - 919.4711 m/z ± 9.2 mDa 301.3647 ± 2.0328 Å²
691%
0%
100%
7mm

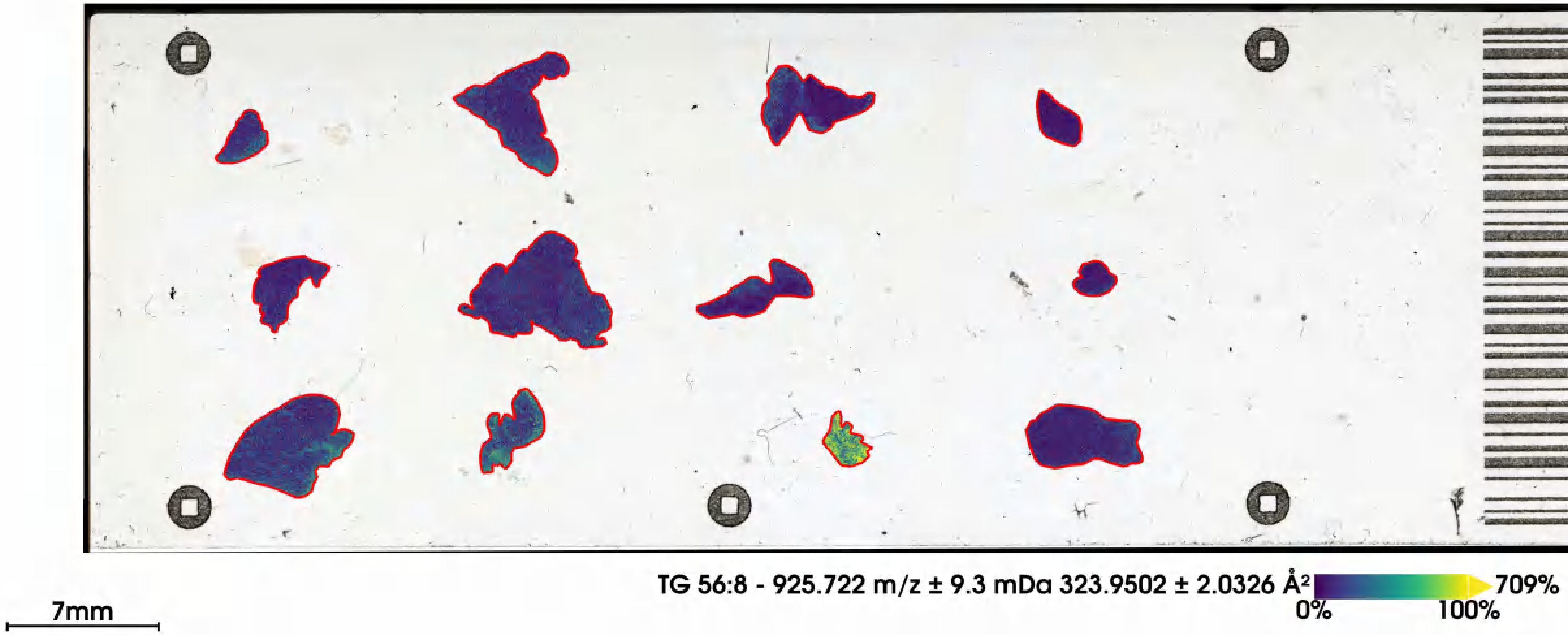
TG 56:8 - 925.722 m/z ± 9.3 mDa 323.9502 ± 2.0326 Å²
709%
0%
100%
7mm

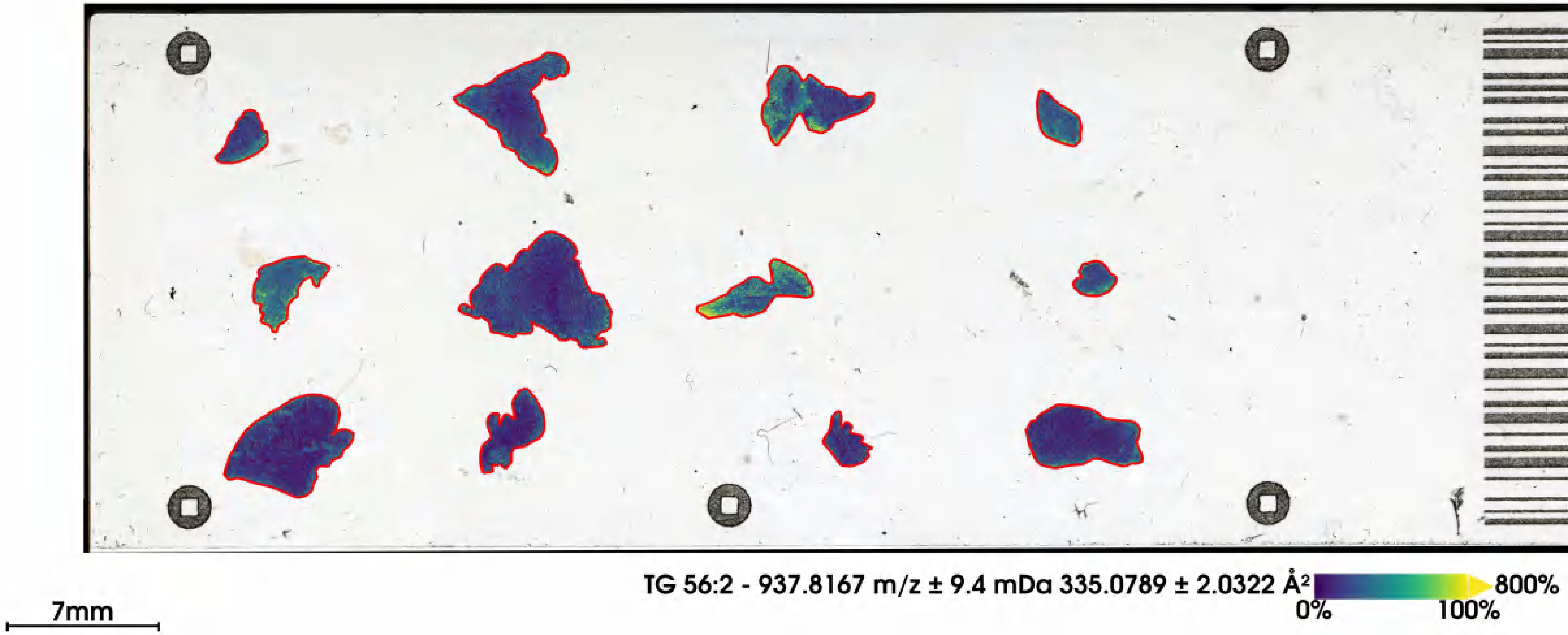
TG 56:2 - 937.8167 m/z ± 9.4 mDa 335.0789 ± 2.0322 Å²
0%
100%
800%
7mm

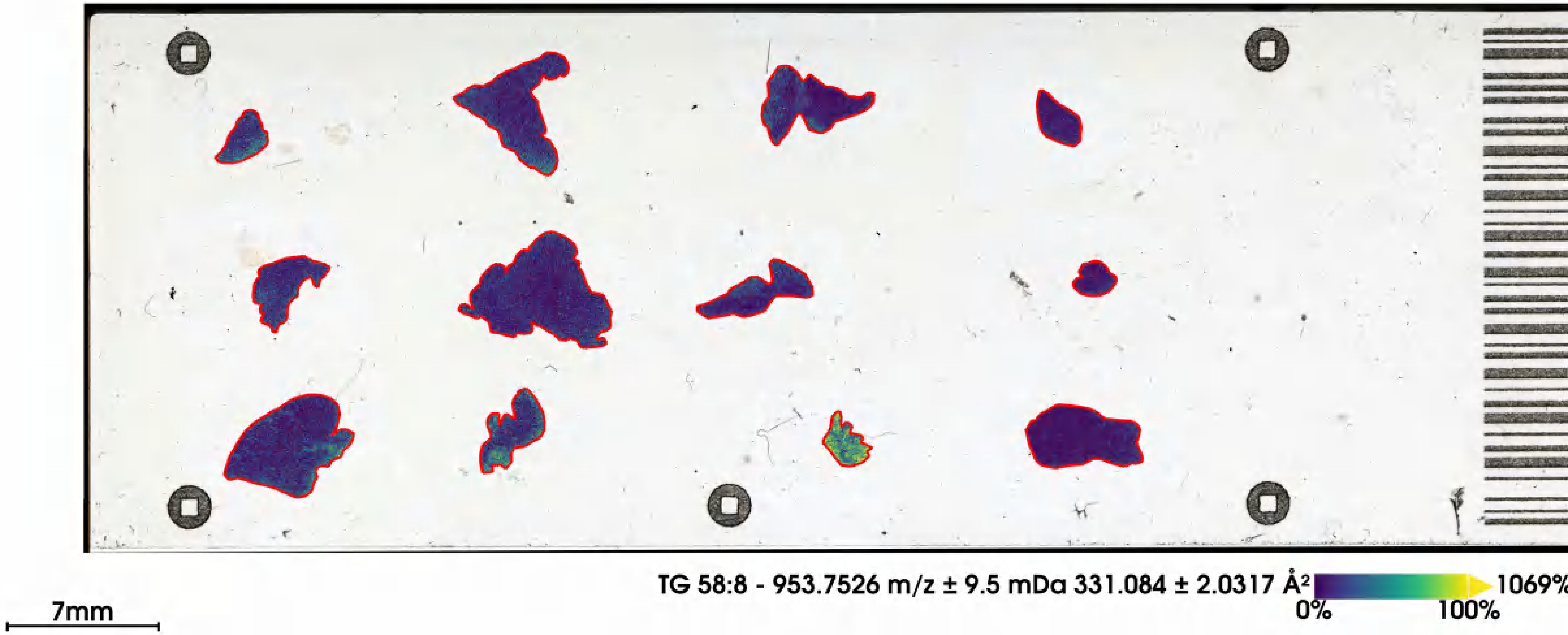

TG 58:8 - 953.7526 m/z ± 9.5 mDa 331.084 ± 2.0317 Å²
0%
100%
1069%
7mm

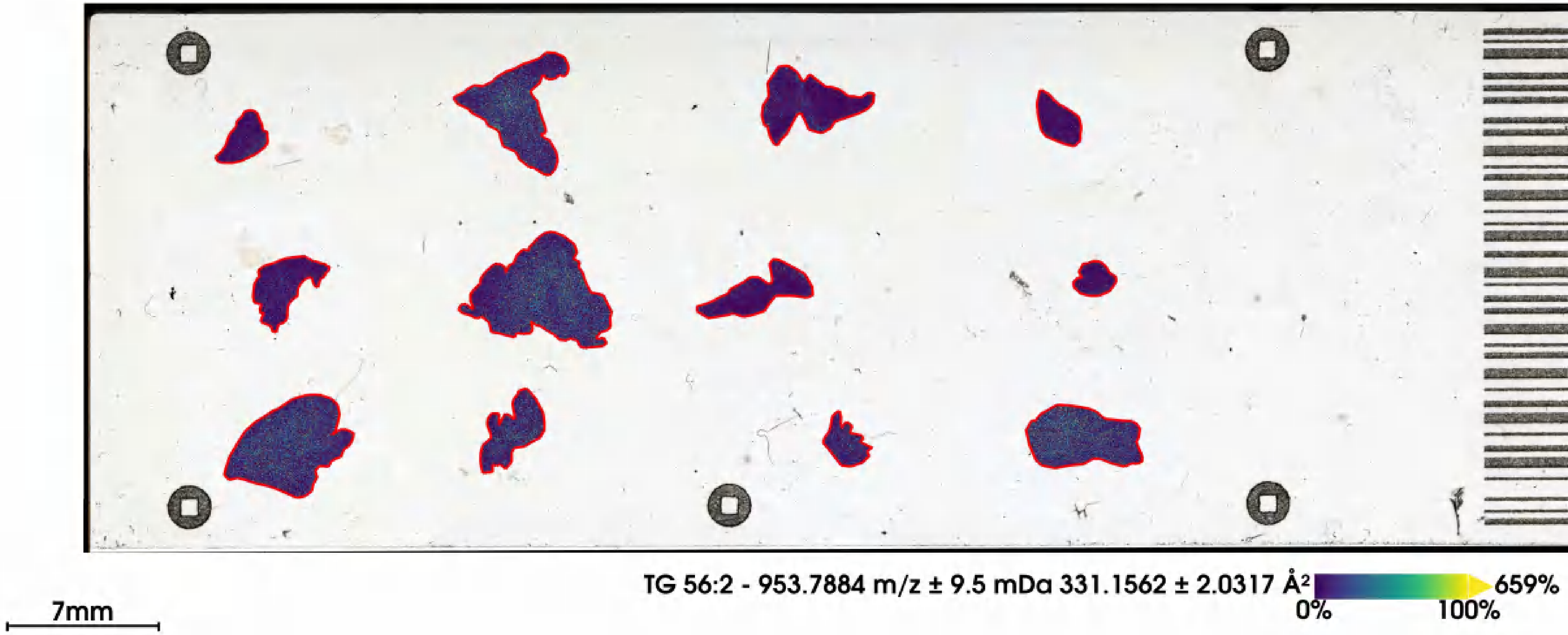

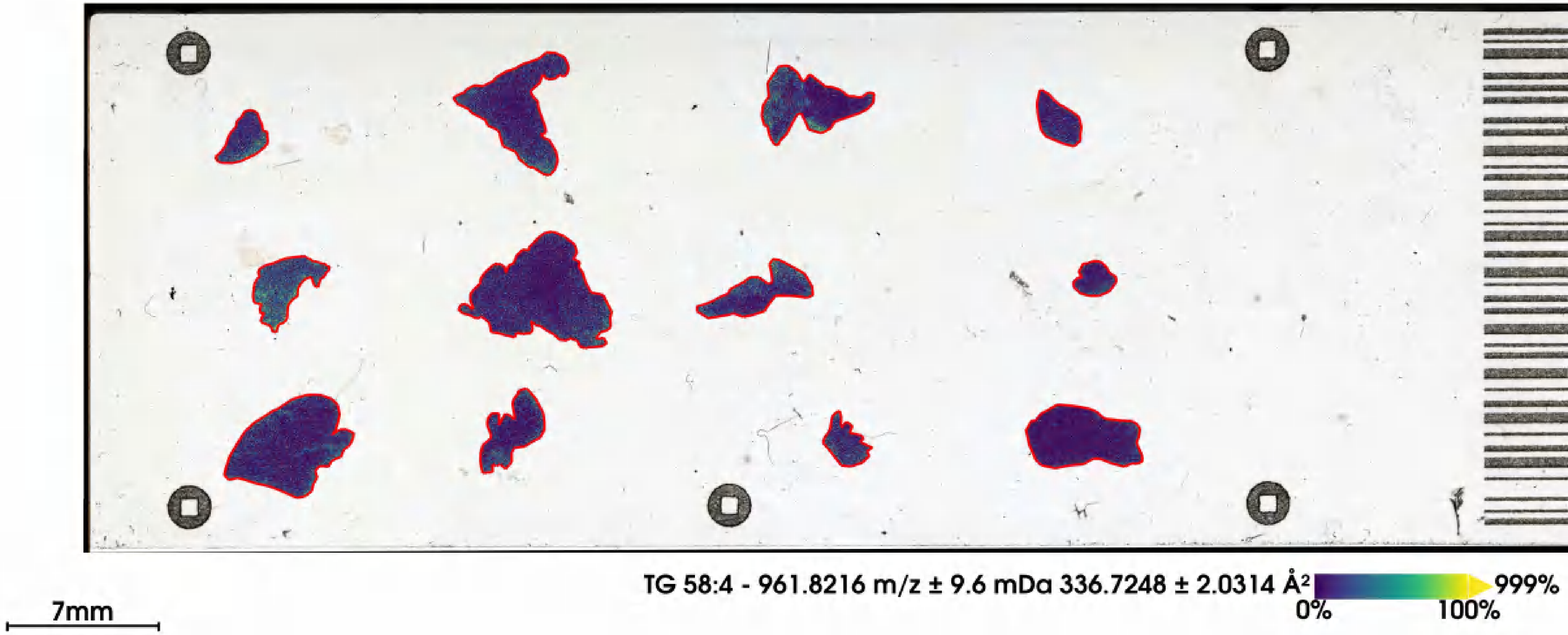
TG 58:4 - 961.8216 m/z ± 9.6 mDa 336.7248 ± 2.0314 Å²
0%
100%
999%
7mm

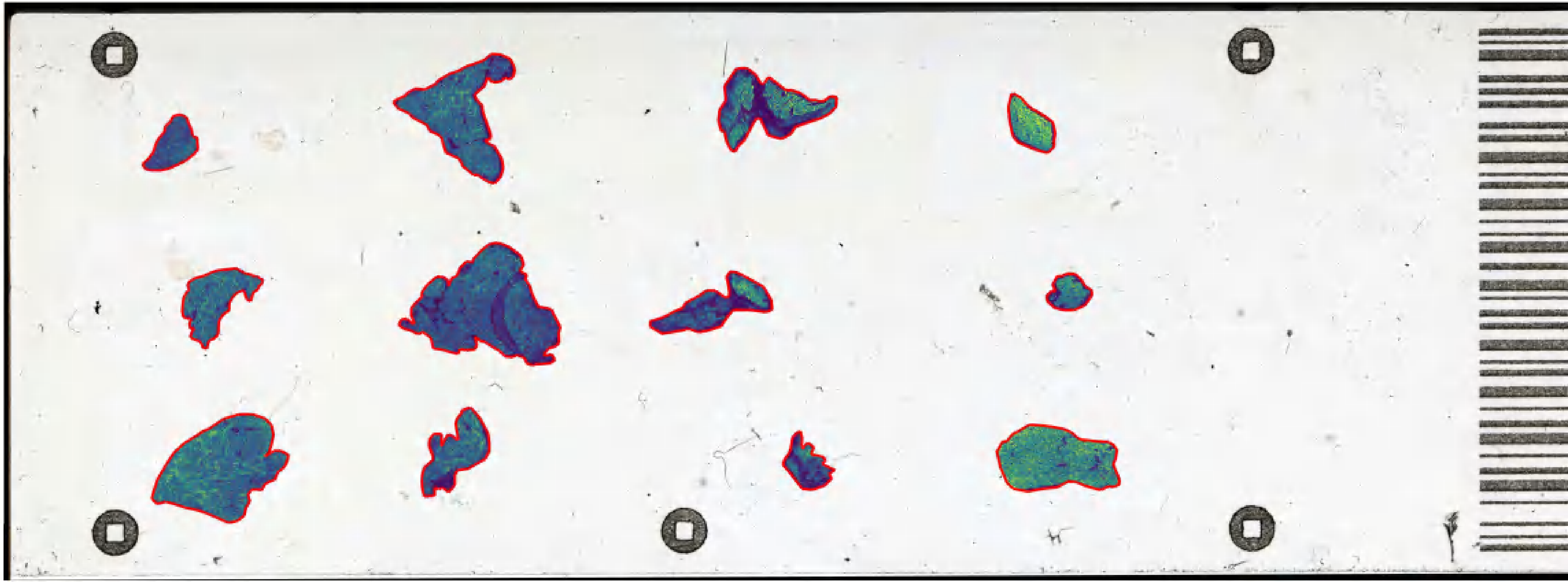

PI 44:8 - 963.602 m/z ± 9.6 mDa 319.1944 ± 2.0314 Å² 0% 100% 276%

7mm

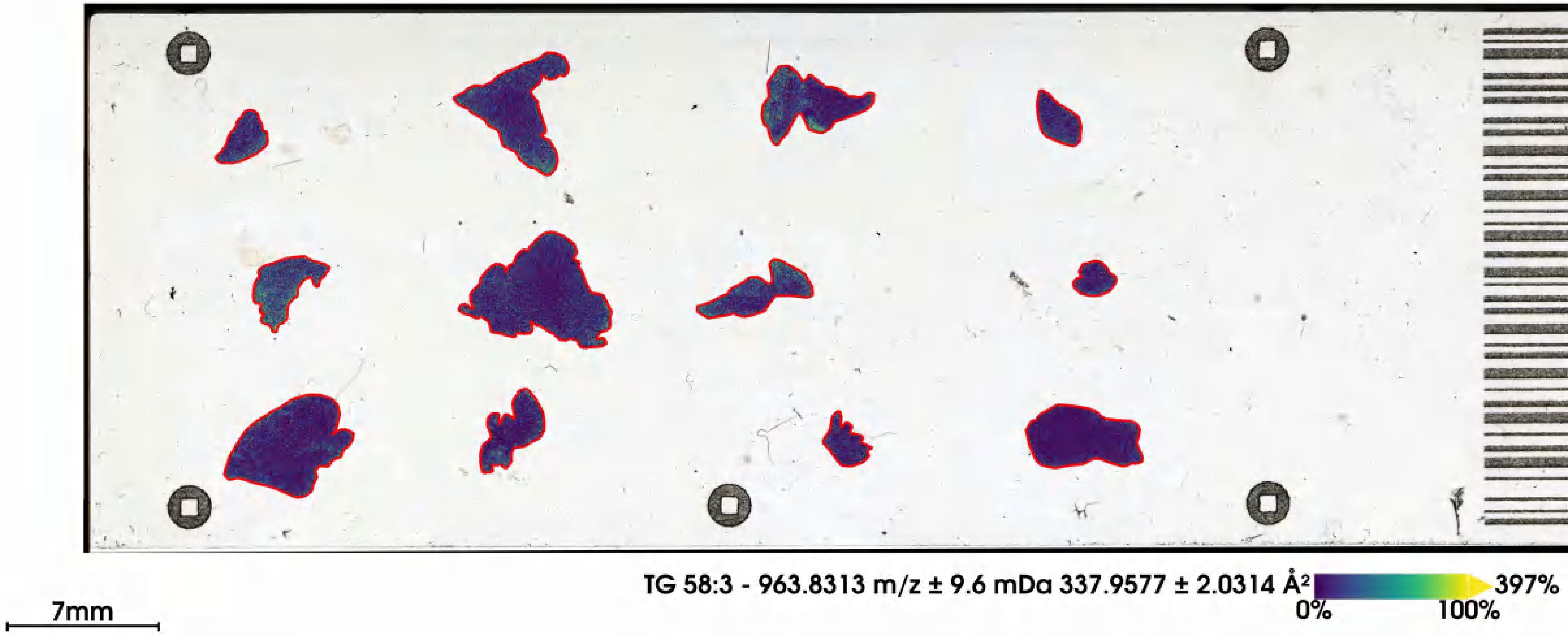

TG 58:3 - 963.8313 m/z ± 9.6 mDa 337.9577 ± 2.0314 Å²
0%
100%
397%
7mm

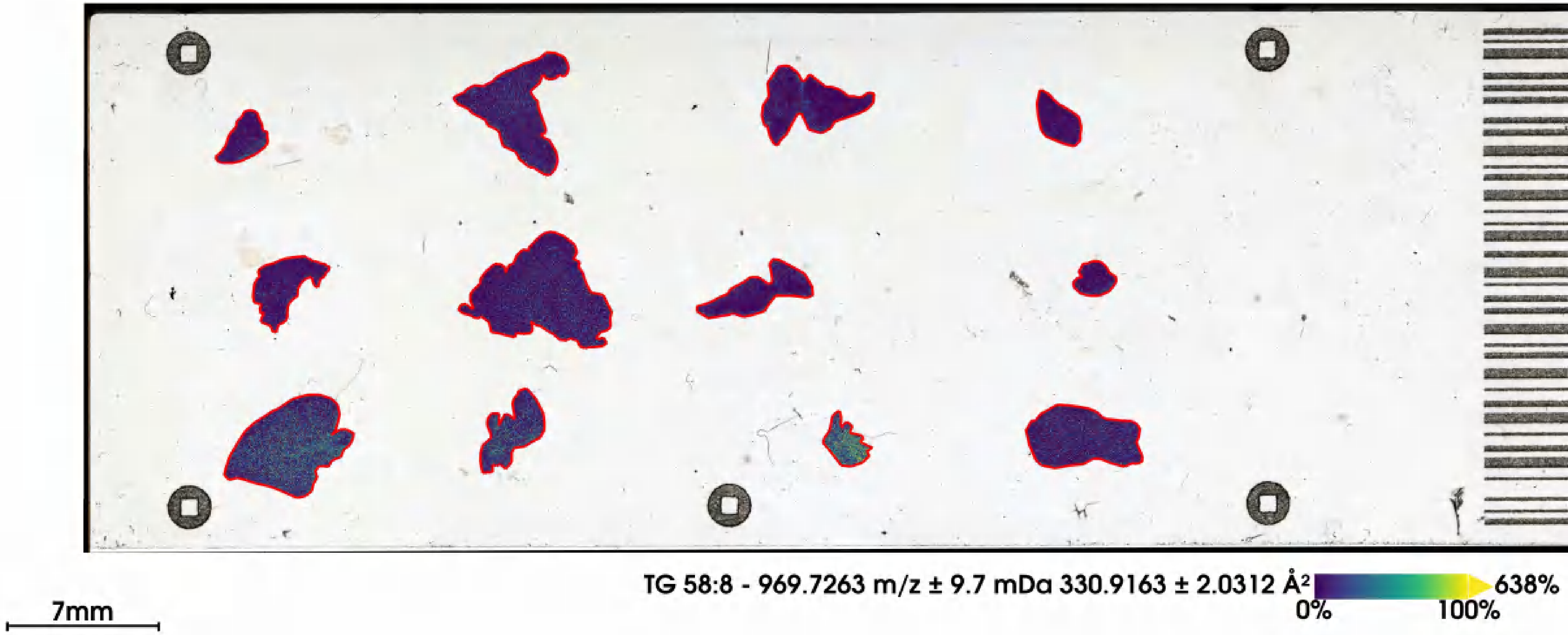
7mm
TG 58:8 - 969.7263 m/z ± 9.7 mDa 330.9163 ± 2.0312 Å²
0%
100%
638%

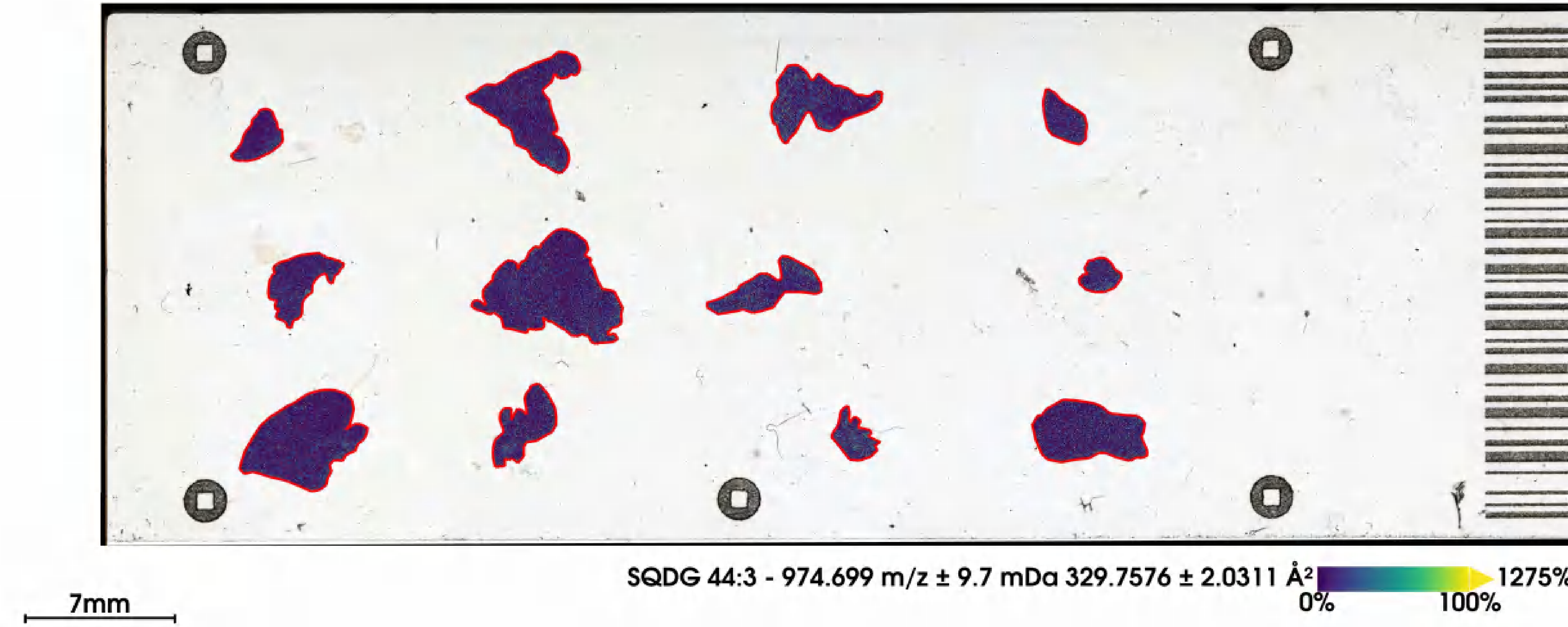
SQDG 44:3 - 974.699 m/z ± 9.7 mDa 329.7576 ± 2.0311 Å²
0%
100%
1275%
7mm

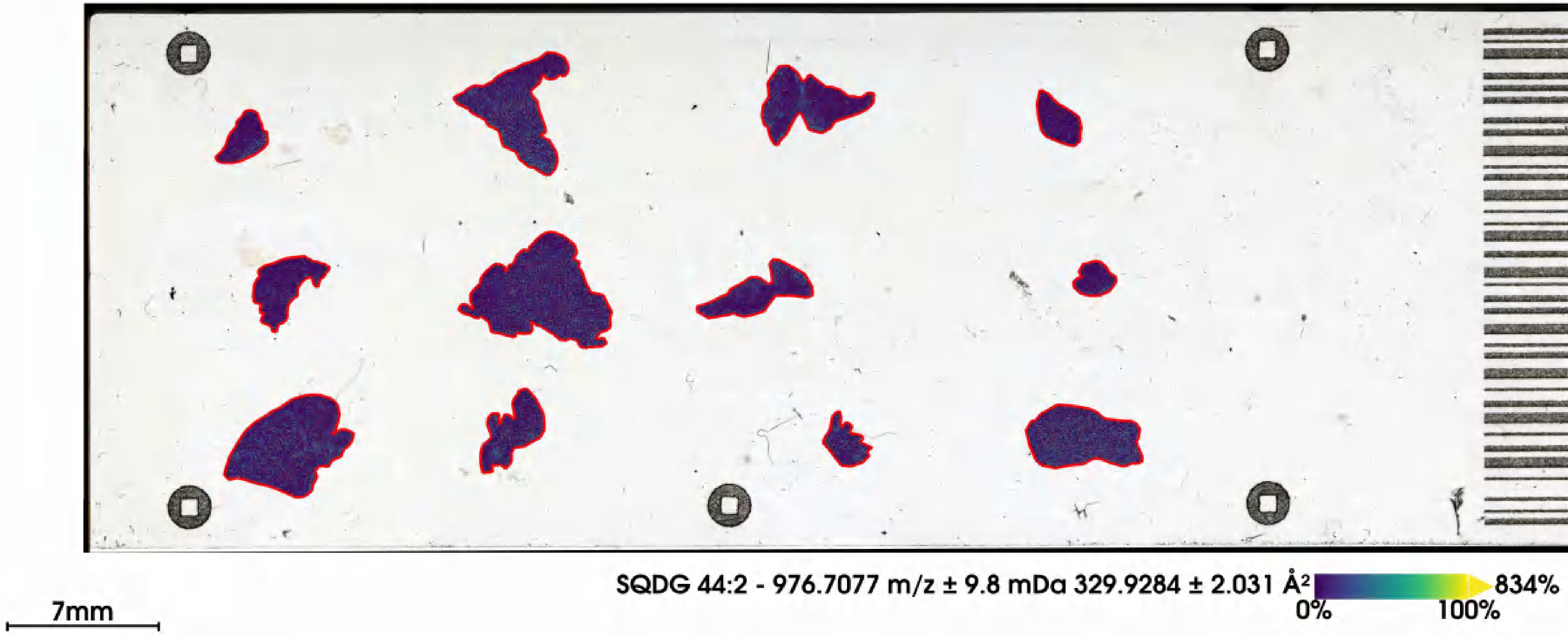

SQDG 44:2 - 976.7077 m/z ± 9.8 mDa 329.9284 ± 2.031 Å²
834%
0%
100%
7mm

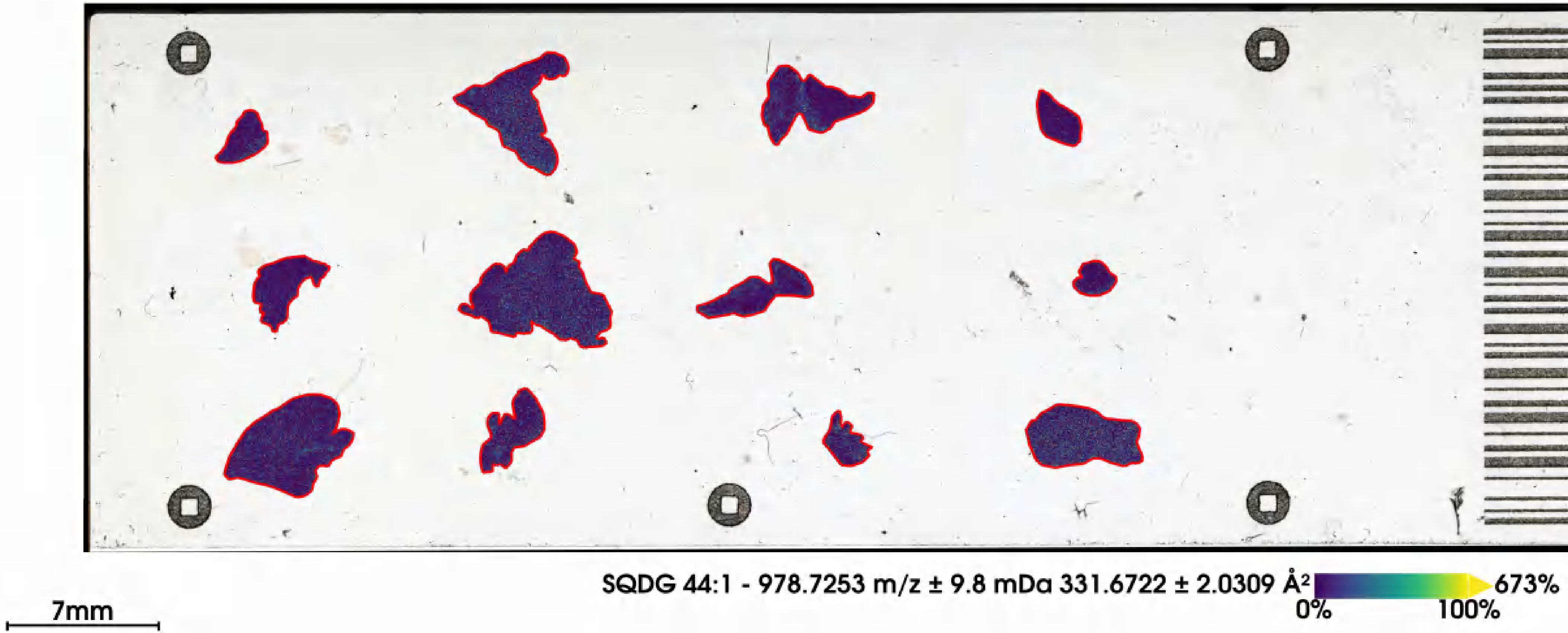

SQDG 44:1 - 978.7253 m/z ± 9.8 mDa 331.6722 ± 2.0309 Å²
0%
100%
673%
7mm

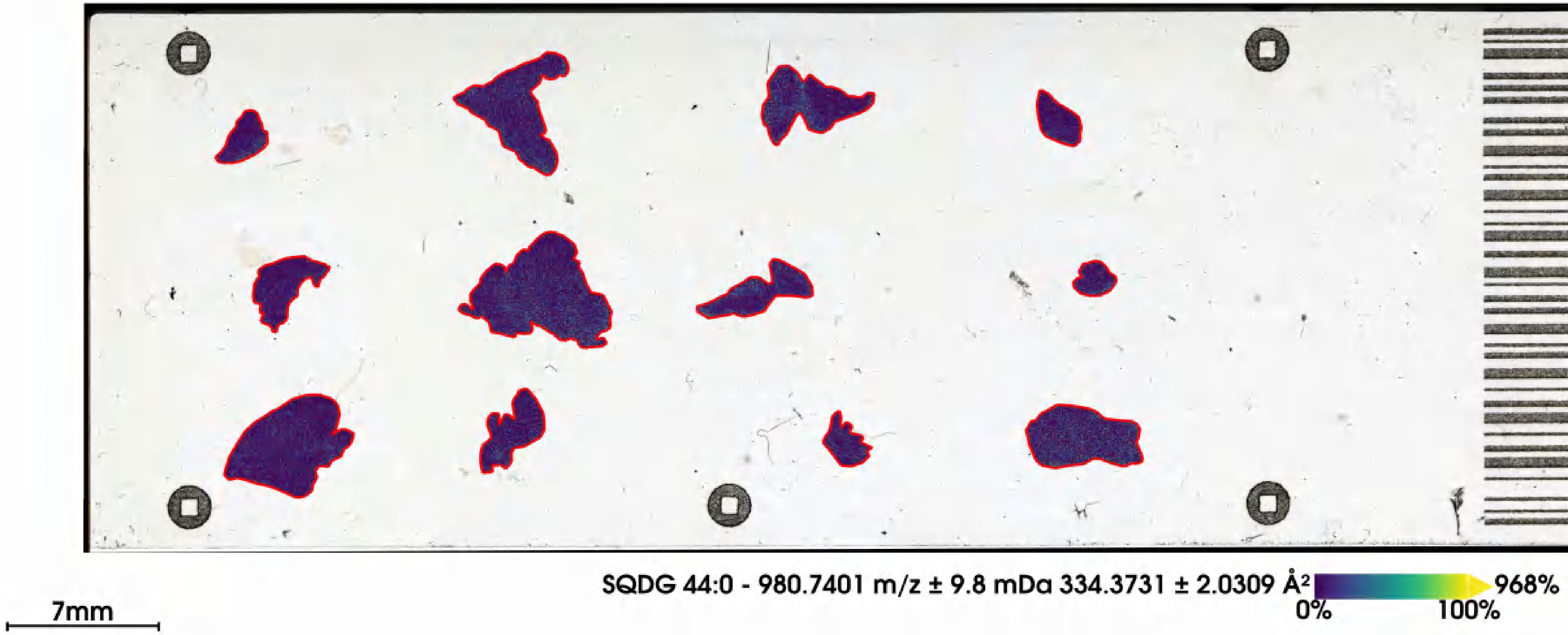

SQDG 44:0 - 980.7401 m/z ± 9.8 mDa 334.3731 ± 2.0309 Å²
0%
100%
968%
7mm

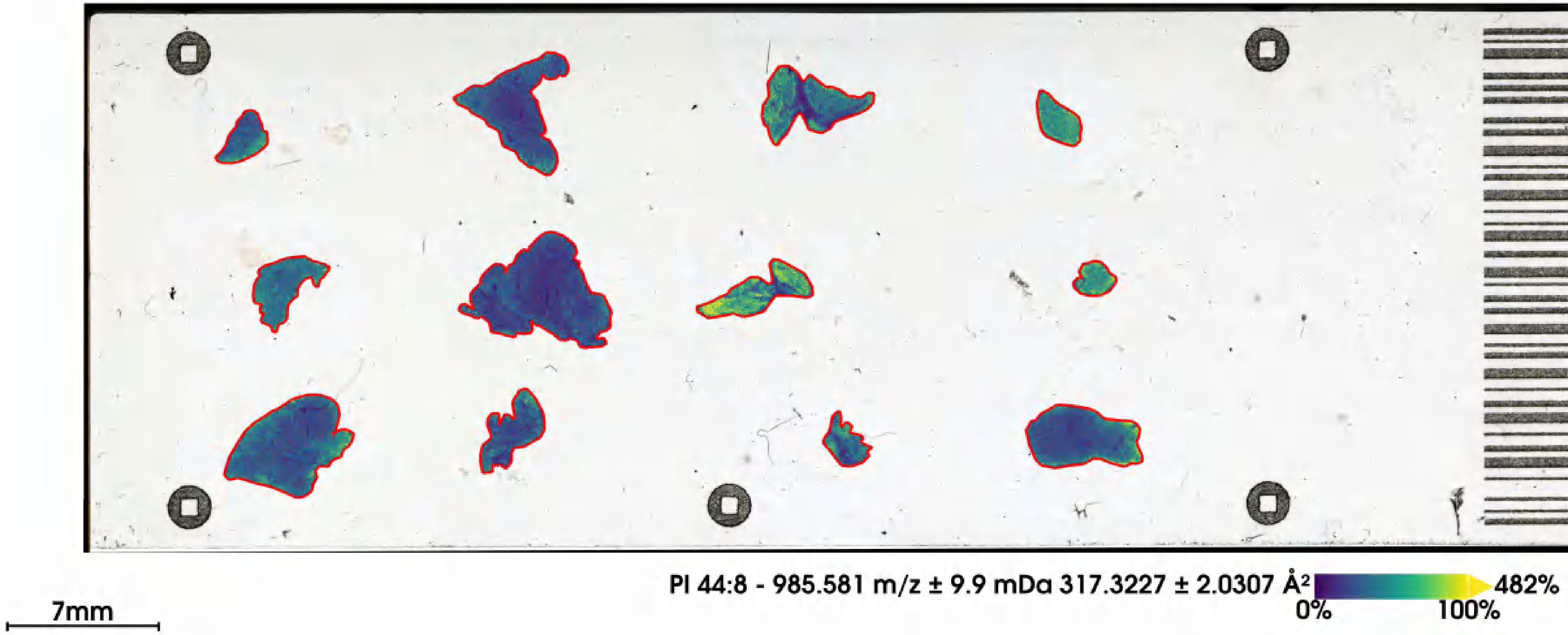

PI 44:8 - 985.581 m/z ± 9.9 mDa 317.3227 ± 2.0307 Å²
0%
100%
482%
7mm

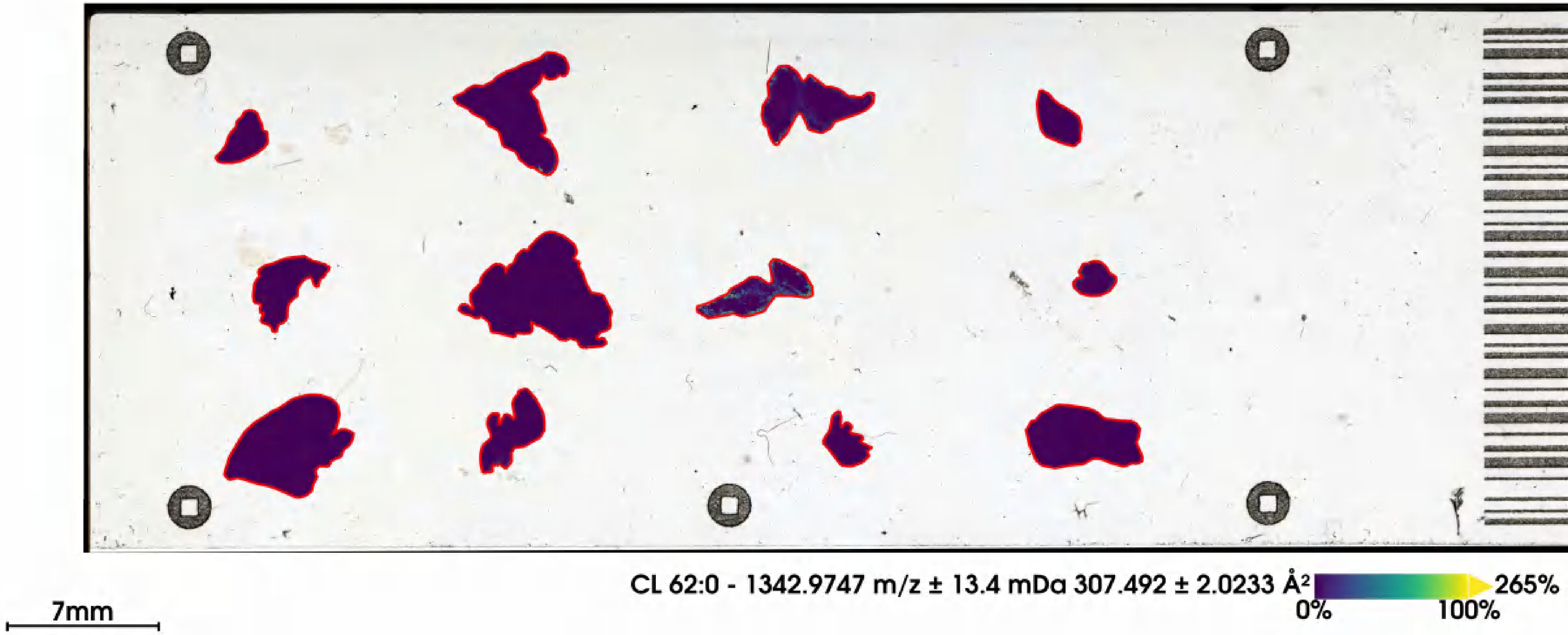
7mm
CL 62:0 - 1342.9747 m/z ± 13.4 mDa 307.492 ± 2.0233 Å²
0%
100%
265%
