# Supplementary material for: Periodic fasting and refeeding re-shapes lipid saturation, storage, and distribution in brown adipose tissue
Source: PLoS Biol. 2026 Jan 12;24(1):e3003593. doi: 10.1371/journal.pbio.3003593 (PMC12795461; doi:10.1371/journal.pbio.3003593)
Supplement: Attachment — Submitted filename: Rebuttal Letter (2)(1).pdf [file pbio.3003593.s022.pdf]

SCHOOL OF MEDICINE
DEPARTMENT OF BIOCHEMISTRY & MOLECULAR BIOLOGY

Editors
PLOS Biology

October 29, 2025

Dear editors,

We were very glad to hear that the reviewers and editors considered our work potentially interesting, technically rich, and timely. According to reviewer's comments and editorial feedback, we have conducted a few new studies, revised our manuscript, and performed proof reading. We hope the revision has adequately addressed the reviewer's concerns. Below are our point-by-point responses.

Comments from the editor

We think the revision will need to respond to all reviewer comments point-by-point, and will particularly need to provide

(1) *validation the mTORC1 regulation of lipid phenotypes*;

Response: We agree with the weakness of the original submission in which the data addressing mTORC1 regulation of lipid phenotypes in Figure 7 were not quantified and statistically analyzed. To address this, we conducted a new study with an increased sample size and combined the resulting data for the validation and quantification. As presented in the revised manuscript, the mediatory effect of mTORC1 on refeeding-elevated relatively saturated glycerolipids was confirmed, and the corresponding graph has been included as Figure 7F. However, the inhibition of the mTORC1 pathway had no significant effects on high-degree unsaturated lipids such as PS 38:8 (Figure 7F). In addition, we observed the induction of fatty acid transport protein SLC27A1 (FATP1) by refeeding (Figs. S4D-S4E), and this induction was attenuated when mTORC1 was inhibited in BAT (Figs. 7G and S7C). These results suggest that mTORC1 mediates BAT lipid remodeling during intermittent fasting, potentially by facilitating the fatty acid esterification process.

(2) *an assessment of the biological importance of lipid remodeling*;

Response: We thank the editors for their insightful feedback regarding the biological significance of the phenomenon we observed. Understanding how lipid remodeling contributes to metabolic adaptation during periodic fasting and refeeding is indeed a compelling and important question. In this study, we observed that brown adipose tissue (BAT) exhibits a distinct response to acute alternate-day fasting (ADF) compared to white adipose tissue (WAT). During fasting, WAT releases substantial amounts of free fatty acids to meet systemic energy demands and subsequently replenishes its lipid stores during refeeding, an expected metabolic response. In contrast, BAT does not appear to contribute significantly to systemic energy supply during fasting. Instead, it likely takes up lipids released from WAT to sustain thermogenesis under moderate cold conditions, as supported by recent studies (PMID: 28988821; PMID: 28988822). Consequently, fasting alone does not markedly alter the lipid profile of BAT. However, refeeding induces a pronounced influx of fatty acids into BAT, which are rapidly esterified into various glycolipids, leading to the expansion of lipid droplets. Although these incoming fatty acids may be relatively low in saturation, their accumulation results in an overall increase in lipid saturation within BAT, contributing to its whitening. This adaptive response in BAT may serve to buffer the liver from lipid overload, functioning as a form of 'metabolic memory' that enhances nutrient uptake during refeeding in response to prior energy deficits.

Refeeding-driven lipid remodeling is also partially interpreted by unrestricted access to food during

refeeding; animals exhibited a 79.6% increase in food intake during refeeding compared to the AD group (Fig. S1B). This led to enhanced uptake of dietary fatty acids by BAT, which were then stored in lipid droplets, contributing to BAT whitening (Figs. 1A, 1B, and S1D). Supporting this, glyceroneogenesis was markedly active during refeeding (Fig. 6), producing high levels of glycerol to rapidly esterify free fatty acids into triglycerides. Furthermore, protein levels of fatty acid-binding protein SLC27A1 (FATP-1) were significantly elevated, indicating a heightened need to manage intracellular free fatty acids (Figs. S4D–S4E). The surge in BAT lipid influx and triglyceride synthesis likely underlies the observed remodeling of lipids, characterized by an increase in saturated fatty acid content during refeeding.

Consistent with thermogenic activation, oxygen consumption increased during refeeding following three cycles of alternate day fasting (Figs. S4B-S4G). This was accompanied by elevated respiratory exchange ratios (Figs. S4H-S4I), suggesting a metabolic switch in BAT from fatty acid oxidation to glucose utilization during the transition from fasting to the refeeding state. Together, these findings link refeeding-induced lipid remodeling in BAT to increased esterification of newly acquired, relatively saturated fatty acids, and a preferential shift toward glucose utilization. We have included newly generated data and expanded the discussion (page 12, paragraph 2).

(3) *validation of key findings under thermoneutral conditions*;

Response: We thank the editors for this important suggestion. We agree that validating our findings under thermoneutral conditions (typically around 30°C for mice) provides valuable insight into whether the observed metabolic and lipid remodeling responses are influenced by ambient temperature and thermogenic demand. In the revised study, we conducted experiments under thermoneutral conditions to assess whether the metabolic adaptations to alternate-day fasting (ADF) are preserved in the absence of cold-induced thermogenesis. Under these conditions, we observed a marked reduction of BAT's response to refeeding. Although changes in body weight and food intake during periodic fasting and refeeding were comparable between 22°C and 30°C, fat mass and adipocyte size across various depots were only modestly affected (Figs. S5A–S5E), suggesting reduced whitening of brown adipose tissue (BAT) under these conditions. A similar trend was also observed in gWAT and iWAT. Despite this, liver's response became more pronounced under thermoneutral conditions compared to mild cold stress (22°C) (Figs. S5B and S5E). Consistently, MALDI analysis indicated that most lipid species remained largely unchanged during fasting and refeeding, although modest shifts were observed in certain highly unsaturated fatty acids, such as PC36:4, PC36:6, and PC38:4 (Fig. S5F). It is important to note that the original experiments were conducted under standard housing conditions (22°C), which are known to induce mild cold stress and activate BAT. These findings highlight the role of environmental temperature and thermogenic programing in shaping metabolic responses specific to fasting regimen. Therefore, some of the observed effects, particularly those related to lipid metabolism and thermogenesis, may have been amplified by this environmental factor.

(4) *supporting western blot and IHC data.*

Response: As suggested by the editors, we performed additional Western blot and immunohistochemistry (IHC) analyses to strengthen the mechanistic aspects of our study. we assessed the expression of important genes involved in fatty acid metabolism, including *Fatp1*/*Slc27a1*, *Cd36*, *Chrebpα*, *Scd1*, *Scd5*, and *Elovl6*. The protein levels of the fatty acid transport protein FATP1/SLC27A1 were elevated during refeeding, while CD36 was not significantly affected (Figs. S4D–S4E). Concurrently, glycerol production was stimulated via glyceroneogenesis, enabling BAT to esterify the rapidly elevated fatty acids (Fig. 6). Although a slight induction of ChREBPα, a glucose-responsive transcription factor that promotes de novo lipogenesis independently of insulin, was observed during refeeding, it was not statistically significant (Figs. S4D–S4E). The expression levels of SCD1, SCD5, and Elovl6 were not significantly affected by fasting or refeeding (Figs. S4D-S4E). These results suggest that FATP1/SLC27A1 may play a critical role in BAT lipid remodeling during the alternate day fasting, handling newly uptake relatively saturated fatty acids. Supporting this, CD31 immunostaining revealed lipid accumulation near vasculature (Figs. S3I–S3J),

indicating potential neutralization of incoming fatty acids and a shift toward more saturated glycolipids such as LPC, MG, PS, PE, PC, and TG.

On the other hand, we examined the expression levels of key thermogenic markers, including UCP1, C/EBPβ, PGC1α, and PRDM16, following 3 cycles periodic fasting and refeeding. The results showed that *Ucp1* and *Pgc1α* were activated during refeeding, with less pronounced induction during fasting (Fig. S4B-S4C), suggesting that thermogenesis may be driven by the post-refeeding surge of various nutrients such as glucose, amino acids, and fatty acids. This is also supported by the increase in respiratory exchange ratios and energy expenditure during the 3rd refeeding (Figs. S4F-S4I), suggesting the metabolic shift in BAT from fatty acid oxidation to glucose utilization. These additional experiments have been incorporated into the revised manuscript to provide a more comprehensive mechanistic understanding of the observed lipid remodeling.

In addition, the co-localization studies using immunostaining for markers such as UCP1 (thermogenic adipocytes) and CD31 (endothelial cells) were performed. Although CD31 expression was highest during fasting phase, the expression levels of UCP1 were higher during refeeding (Figs. S3M and S3N). We then zoomed in and explored the alignment between lipid sparkle zones and vasculature and thermogenic activation zones using LPC16:0 as an example. As indicated by the images, the lipid "sparkles" are related to CD31 enriched region but not UCP1^{high}-expressed zone during refeeding (Figs. S3I-S3K), suggesting that these lipid-enriched zones correspond to fatty acid flux within BAT but not necessary to thermogenic activity. These studies not only characterized the spatial context of these lipid hotspots but also highlighted the contribution of circulating (or dietary) lipids to the formation of sparkle zones.

Comments from the reviewers

Reviewer #1

Major comments

1. While the link between mTORC1 and the lipid phenotypes has been suggested by the AAV experiment, there are several confounding factors that complicate its interpretation such as the viral leakage and liver effects. The authors should use a different methodology or in vitro system to provide data that supports their conclusions.

Response: We appreciate the reviewer's insightful comment regarding potential confounding factors in the AAV experiment. As correctly noted, we observed downregulation of Raptor not only in BAT but also in the liver of the AAV-Cre group (Figs. 7B and S7B), suggesting that mTORC1 inhibition in the liver could contribute to the observed phenotype. We acknowledge the inherent challenges in studying the development-independent function of mTORC1 in adipose tissue, largely due to the lack of an optimal in vivo model, as described in our previous studies (PMID: 30232001; PMID: 31840569). To address this, we also generated inducible KO models using Adiponectin-CreERT and UCP1-CreERT drivers to bypass developmental effects. Unfortunately, these models exhibited leaky Cre activity, leading to recombination and phenotypes resembling the conditional KO mice even before tamoxifen administration. This unintended recombination limited their utility for studying post-developmental mTORC1 function in adipose tissue. As an alternative, we employed AAV-mediated Cre delivery directly into Raptor-floxed BAT, enabling localized and timely knockdown. This approach specifically targeted BAT without affecting other fat depots and avoided developmental confounding factors. Notably, we observed a significant reduction in BAT fat mass under refeeding conditions following mTORC1 inhibition, whereas gWAT remained unaffected (Figure 7). Looking ahead, we plan to adopt a doxycycline-inducible, adipose-specific Cre-LoxP system to achieve temporal control of Raptor deletion in BAT. This strategy will help minimize developmental compensation and off-target effects, including those in the liver, thereby providing a more precise tool for dissecting mTORC1 function in mature adipose tissue. The related discussion has been included in the revision (Page 13, paragraph 3).

2. The authors state in the abstract "Mechanistically, periodic fasting and refeeding activated mTORC1, and genetic inactivation of mTORC1 in BAT diminished ADF induced lipid saturation, storage, and redistribution". I'm not convinced that changes in lipid saturation have been demonstrated by the data shown. Additional experiments/data are required to confirm this point.

Response: To validate the results, we have conducted a new study by increasing the size of groups followed by quantification and statistical analysis. As presented in the revised manuscript, the attenuating effect of mTORC1 inhibition on refeeding-induced lipids with lower degrees of unsaturation was confirmed, and the corresponding graph has been included as Figure 7F. We observed that inhibition of the mTORC1 pathway diminished the increase in fed-induced more saturated lipids, including TG50:1, TG50:2, PE 34:0 and PC36:1, with little effects on highly unsaturated lipids such as PS 38:8 (Figure 7F). In addition, we observed the induction of fatty acid transport protein SLC27A1 (FATP1) by refeeding (Figs. S4D-S4E), and this induction was attenuated when mTORC1 was inhibited in BAT (Figs. 7G and S7C). These results suggest that mTORC1 partially mediates BAT lipid remodeling during intermittent fasting.

3. The data in every panel of Figure 7 needs to be quantified and plots generated with stats testing. The phospho western blots need to be normalised relative to each total protein blot. The raptor westerns normalised to tubulin.

Response: As suggested, another run of MALDI study was performed. Combined with the lipid profile data in original Figure 7E, all the data have been quantified and statistically analyzed in Figure 7F. In Figure 7A, the Western blot results have been quantified and normalized for statistical analysis. P-S6K and P-AKT have been normalized with S6K and AKT, respectively, and Raptor has been normalized by Tubulin, as shown in the new graph (Fig. S7A). The blot quantification and analysis for Figure 7B and 7G have been included in the revision (Fig. S7B and S7C).

4. BAT can be difficult to dissect from the WAT that lies adjacent to it. How did the authors remove contaminating WAT from the BAT depot?

Response: In order to minimize the contamination, three approaches were applied: 1) the interscapular BAT (iBAT) depot was carefully dissected with particular attention to removing surrounding white adipose tissue; 2) The half of total BAT tissue (one side of butterfly) were used for MALDI analysis (see the image below), and 3) The center region of BAT tissue was screened using MALDI as shown below. All tissues were kept on ice throughout the procedure to preserve morphology and facilitate accurate identification. To further confirm the purity of the dissected BAT, we evaluated adipose tissue morphology using H&E and UCP1 staining (Figs. S3I-S3N). The BAT samples consistently exhibited characteristic multilocular lipid droplets and uniform tissue structure, along with high and consistent expression of UCP1, indicating minimal contamination from WAT (Figs. S1D, 7D, and S3N). We have now added a detailed description of the dissection protocol and marker gene analysis to the Methods section of the revised manuscript (page 4, last

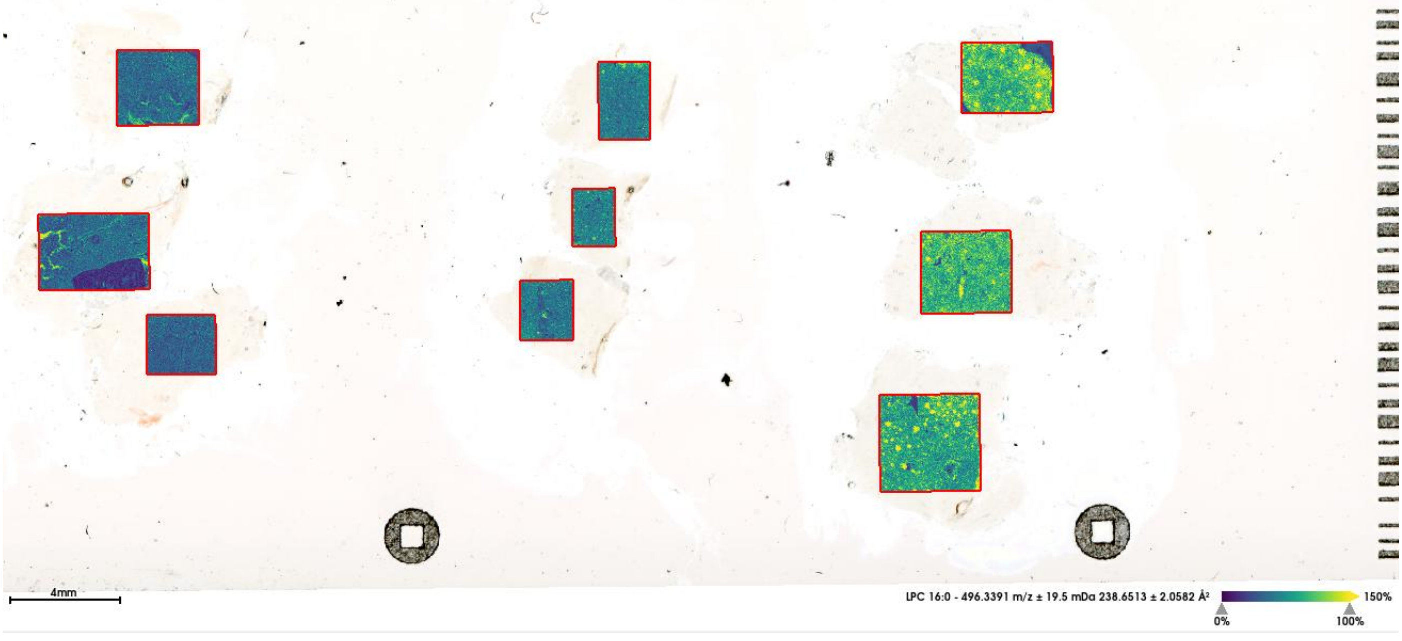

paragraph).

5. Why did the authors choose this particular ADF intervention length of only 6 days? Other studies use 2 or more weeks of ADF intervention.

Response: We thank the reviewer for raising this important point regarding the duration of the ADF intervention. We selected a 6-day ADF protocol based on several considerations. Our primary objective was to capture the early metabolic and molecular responses to ADF, particularly during the transition from fasting to refeeding. A shorter intervention allowed us to focus on these acute regulatory events while minimizing the confounding effects of long-term adaptation. Each ADF cycle includes two distinct phases, fasting and post-fast refeeding. While the fasting state has been extensively studied, the physiological impact of the refeeding phase remains less understood. Emerging evidence suggests that refeeding not only reverses fasting-induced catabolism but may also trigger a nutrient-augmented anabolic rebound. By focusing on this early window, our study aimed to provide new insights into the underexplored physiology of refeeding. Moreover, the 6-day duration helped reduce systemic compensatory changes and enabled us to isolate tissue-specific responses, particularly in BAT. Although longer ADF protocols are valuable for studying chronic adaptations, our goal was to dissect the initial metabolic adaptation and mechanistic links in adipose tissues.

6. The authors say that the "activity" of certain pathways have increased or decreased throughout the manuscript, but they are only providing static measurement at a single point in time, please restate this as altered abundance instead.

Response: We thank the reviewer for pointing out that our current data represents static measurements at a single time point and do not directly assess dynamic pathway activity. To more accurately reflect the nature of our findings, we have revised the manuscript to describe these changes as alterations in the abundance of pathway components or markers, rather than inferring changes in activity. This adjustment ensures that our interpretations remain consistent with the data presented. For instance, "Furthermore, over activation of mTORC1 signaling results in the development of adiposity and obesity" has been rephrased into "Furthermore, excessive mTORC1 signaling results in the development of adiposity and obesity" (Last paragraph, page 2).

7. The authors propose that the mRNA measurement in Figure 6L is an accurate surrogate for the measurement of the enzymes themselves, but I disagree. The authors should measure the protein levels of these enzymes using westerns/proteomics. A proteomic analysis of these samples would also add significant insight into how each metabolic pathway is being regulated.

Response: We agree with the reviewer that mRNA expression levels do not always directly correlate with protein abundance or enzymatic activity. In our study, the mRNA measurements in Figure 6L were intended to provide an initial indication of transcriptional regulation within the relevant metabolic pathways. To strengthen our conclusions, we conducted follow-up experiments to assess the protein levels of these enzymes using western blotting. In the revised manuscript, we have clarified the interpretation of the mRNA data and now refer to them as indicators of transcriptional changes rather than direct measures of their protein levels. Please refer to our response to the editor's comment 4.

8. The manuscript lacks consistency and clarity throughout, for example, referring to "feast BAT" at many points, is that the refed group or the AD group? Please rewrite this throughout in a consistent manner.

Response: To improve clarity and consistency throughout the manuscript, we have revised the text to clearly distinguish between experimental groups. Specifically, we now consistently refer to the groups as "refed BAT" (for tissues collected after the refeeding phase of ADF) and "fasting BAT" (for tissues collected during the alternate day fasting protocol), as appropriate. These changes have been implemented throughout the manuscript, including figure legends and labels, to ensure that readers can easily follow the experimental

design and interpret the results accurately.

9. The manuscript has issues with grammar and style throughout: "became shrank"
Response: As reviewer suggested, we have performed proof reading and corrected those typos and grammatic errors throughout the manuscript. The “became shrank” has been replaced with “became shrunken” or “underwent shrinkage”.

Minor comments
1. Why did this study only analyse male mice?
Response: Given the known protection of female mice against diet-induced obesity, we conducted the study using female mice at the initial stage. However, we acknowledge this as a limitation of the study, and future work should incorporate both sexes to address potential gender differences (page 13, paragraph 3).

2. Please add an additional tab in Supplementary Table 1 that contains a detailed legend describing all abbreviations used in the table for animal tissues/groups. Also add details of the statistical tests performed, any p-value adjustment, and the number of animals per group in stats tests.
Response: As reviewer suggested, we have updated tissues/groups and animal numbers in the table and added table legend in the supplementary information.

3. Please provide results of statistical tests for each row in Supplementary Table 2. Also, please add an additional tab that contains a detailed legend describing all abbreviations used in the table for animal tissues/groups. Also add details of the statistical tests performed, any p-value adjustment, and the number of animals per group in stats tests.
Response: As reviewer suggested, we have performed two-tailed Student’s T-test and updated P value in the table and added table legend in the supplementary information.

4. Colour legends need to be placed on all supplementary figures/plots as per figure 1b.
Response: Thanks for the suggestion. As the reviewer suggested, we have added all the color legends in the figures as per figure 1b.

5. The following sentence doesn't make sense: "When lipolytic enzymes are deficient in brown adipocytes, the utilization of fatty acids in circulation is induced and substitutes for BAT TG to sustain cold-induced thermogenesis". What is "the utilization of fatty acids in circulation"?
Response: We thank the reviewer for pointing out the ambiguity in this sentence. Our intention was to convey that, in the absence of intracellular triglyceride (TG) stores due to lipolytic enzyme deficiency in brown adipocytes, BAT compensates by increasing its uptake and utilization of circulating fatty acids to sustain thermogenesis during cold exposure. The sentence has been edited as follows: *When lipolytic enzymes are deficient in brown adipocytes, BAT compensates by increasing the uptake and oxidation of circulating fatty acids, which substitute for intracellular triglyceride stores to support cold-induced thermogenesis* (page 2, paragraph 3).

Reviewer #2
Major Points:
1. While the study describes shifts in lipid saturation and composition during fasting/refeeding, it does not establish whether these changes influence thermogenesis, energy expenditure, or systemic metabolism. Are these compositional changes functionally adaptive, or merely reflective of nutrient flux? No data are provided on oxygen consumption, thermogenic response to cold or β3-adrenergic stimulation. These assessments may be essential to validate the biological importance of the observed lipid remodeling.
Response: We thank the reviewer for the insightful comments. To address the biological significance of lipid

remodeling, we have conducted additional studies in the revised manuscript, including metabolic phenotyping, thermogenic gene expression analysis, thermoneutral adaptation, and imaging to assess the spatial alignment of lipid accumulation with vasculature (Figs.7F-7G, S3I-S3M, S4D-S4K, and S5A-S5F). First, we assessed the expression of important genes involved in fatty acid metabolism, including *Fatp1*/*Slc27a1*, *Cd36*, *Chrebpα*, *Scd1*, *Scd5*, and *Elovl6*. The protein levels of the fatty acid transport protein FATP1/SLC27A1 were elevated during refeeding, while CD36 was not significantly affected (Figs. S4D–S4E). Concurrently, glycerol production was stimulated via glyceroneogenesis, enabling BAT to esterify the rapidly elevated fatty acids (Fig. 6). Although a slight induction of ChREBPα, a glucose-responsive transcription factor that promotes de novo lipogenesis independently of insulin, was observed during refeeding, it was not statistically significant (Figs. S4D–S4E). The expression levels of SCD1, SCD5, and Elovl6 were not significantly affected by fasting or refeeding (Figs. S4D-S4E). These results suggest that FATP1/SLC27A1 may play a critical role in BAT lipid remodeling during the alternate day fasting, handling newly uptake relatively saturated fatty acids. Additionally, CD31 immunostaining revealed lipid accumulation near vasculature (Figs. S3I–S3J), indicating potential neutralization of incoming fatty acids and a shift toward more saturated glycolipids such as LPC, MG, PS, PE, PC, and TG.

To investigate its physiological relevance, we examined the expression levels of key thermogenic markers, including UCP1, C/EBPβ, PGC1α, and PRDM16, following 3 cycles periodic fasting and refeeding. The results showed that Ucp1 and Pgc1α were activated during refeeding, with less pronounced induction during fasting (Fig. S4B-S4C). In line with this, oxygen consumption was significantly elevated during the light cycle of refeeding phase following three cycles of alternate-day fasting (Figs. S4F-S4G), accompanied by an increase in respiratory exchange ratio (Figs. S4H-S4I). These results suggest that thermogenesis may be driven by the post-refeeding surge of glucose rather than fatty acids, with a metabolic shift in BAT from fatty acid oxidation to glucose utilization during anabolic rebound. BAT lipid remodeling is associated with thermogenic activation at room temperature, an effect that is significantly attenuated under thermoneutral conditions (please see our response to editor's comment 3). These additional experiments have been incorporated into the revised manuscript to provide a more comprehensive mechanistic understanding of the observed lipid remodeling. We have also expanded the discussion accordingly (page 12, paragraph 2).

2. The manuscript posits that lipid saturation increases upon refeeding and contributes to BAT whitening and TG accumulation. However, whether this shift causal for the phenotypes is not tested. Could a pharmacological or genetic manipulation that blocks desaturation (e.g., SCD1 inhibition or Elovl3 knockdown) prevent the whitening or suppress TG accumulation in refeeding conditions? Conversely, does increasing PUFA availability protect against whitening?

Response: We agree with the reviewer that, while our data suggests a correlation between increased lipid saturation during refeeding and BAT whitening/triglyceride accumulation, causality was not directly tested in the original submission. In the revised study, we examined the expression of SCD1, SCD5, and Elovl6—enzymes highly expressed in BAT and involved in fatty acid desaturation and elongation (PMID: 26628376). However, none of these enzymes showed significant changes in expression in response to alternate-day fasting, regardless of the fasting or refeeding phase (Fig. S4D–S4E). Despite this, we observed that refeeding-induced glycolipid accumulation, visualized as "sparkles", was spatially aligned with vasculature, as confirmed by H&E and CD31 staining (Figs. S3I–S3J). This suggests that these glycolipids are newly synthesized from influx fatty acids, which may be relatively saturated. These findings support the notion that BAT whitening and triglyceride accumulation during refeeding are driven by the uptake and esterification of dietary fatty acids (please see our response to your comment 1).

3. The MALDI-MSI results show spatial "sparkle" zones enriched in saturated lipids during refeeding. However, these regions are not linked to any known BAT microanatomy. Are they aligned with blood vessels, multilocular zones, or regions of sympathetic innervation? Some validation using co-staining or marker overlays (e.g., UCP1, CD31, perilipin) may help to determine whether these lipid hotspots correspond to

functionally distinct areas.

Response: We thank the reviewer for this thoughtful observation. The "sparkle" zones enriched in saturated lipids observed in our MALDI-MSI data indeed represent intriguing spatial features. In the revision, the co-localization studies using immunostaining for markers such as UCP1 (thermogenic adipocytes), and CD31 (endothelial cells) were performed. Although CD31 expression was highest during fasting phase, the expression levels of UCP1 were higher during refeeding (Figs. S3M and S3N). We then zoomed in and explored the alignment between lipid sparkle zones and vasculature and thermogenic activation zones using LPC16:0 as an example. As indicated by the images, the lipid "sparkles" are related to CD31 enriched region but not $UCP1^{high}$-expressed zone during refeeding (Figs. S3I-S3K), suggesting that these lipid-enriched zones correspond to fatty acid flux within BAT but not necessary to thermogenic activity. These studies not only characterized the spatial context of these lipid hotspots but also highlighted the contribution of circulating (or dietary) lipids to the formation of sparkle zones. We have included the image data in the revised manuscript.

4. The authors observe reduced thiamine levels in BAT upon refeeding and speculate that this limits pyruvate oxidation via PDH, redirecting carbon flux to glyceroneogenesis. However, this idea is not directly tested. Are acetyl-CoA or citrate levels affected? Does thiamine repletion restore PDH flux or attenuate TG accumulation? Including even one tracer experiment would substantially strengthen this part of the model.

Response: We thank the reviewer for this insightful comment. As noted, our observation of reduced thiamine levels in BAT upon refeeding led us to hypothesize that thiamine deficiency may impair PDH activity, thereby limiting pyruvate oxidation and redirecting carbon flux toward glyceroneogenesis. Supporting this hypothesis, we observed elevated levels of fumaric acid and malic acid, along with upregulation of associated enzymes as determined by qPCR. In line with reduced PDH flux, citric acid levels were significantly decreased, while acetyl-CoA levels remained unchanged (Figs. S8G-S8H and S8N). Furthermore, the correlation between decreased thiamine and the accumulation of related metabolites suggests a potential role for thiamine in regulating glycerol production, glyceroneogenesis, and triglyceride accumulation. This is further supported by increased PAS staining and BAT whitening (Fig. S6G and 1B). We agree that tracer experiments would provide valuable insights into the potential connection between thiamine metabolism and lipid remodeling in the current study. Given that the central focus of this work is to elucidate lipid remodeling and the role of mTOR signaling, we will incorporate isotope tracing in future studies to further validate this model. Such a limitation has been discussed in the revised manuscript (page 13, paragraph 3).

Minor Points

* *Fig. 1D: PCA results are somewhat difficult to interpret without access to the loading plots. Which metabolites or lipid species drive the separation among conditions?*

Response: We thank the reviewer for this point. The contribution values have now been incorporated into Supplementary Table 1. The statistical analysis was carried out using proprietary software from HMT, Inc. (Tsuruoka, Japan). As the software is a proprietary product, we have accordingly acknowledged HMT, Inc. for their service in the revised version of the manuscript.

* *Fig. 2-3: Please clarify if these are FFAs or FAs derived from all lipid classes?*

Response: In our analysis, the term "FAs" in Figures 2 and 3 specifically refers to free fatty acids (FFAs). The nomenclature used for these FFAs is based on carbon chain length and the number of double bonds. For example, FA (24:5) denotes a free fatty acid with 24 carbon atoms and 5 double bonds. To avoid confusion, we have revised the manuscript to clearly state that these measurements pertain to FFAs and not to fatty acids derived from complex lipid classes. This clarification has been added to both figure legends and the relevant sections of the results.

* *Fig. 4: Are MSI ion intensities normalized to internal standards?*
Response: The laser energy and scan setting have been included in the method section "Imaging was performed at a spatial resolution of 20 µm with beam scan on (16 µm), using 1 burst of 200 shots per pixel, at 70% laser power, and a 10 kHz laser repetition rate." And the pixel numbers are below, which are related to the section size (scan area). The related information has been included in revised manuscript (page 5, line 5).

* *Fig. 6M: The pathway schematic is useful, but the connection to mTORC1 or the proposed glyceroneogenesis mechanism could be clearer.*
Response: In the revision, the schematic summary of metabolic pathways has been revised by adding the connection to refeeding and glyceroneogenesis, highlighting the mechanistic advancement. As mTORC1 is stimulated under these conditions, allowing us to propose its potential role in mediating refeeding-increased glycerol production (Fig. S8F).

* *Methods: Please include more technical details for MALDI-MSI acquisition (e.g., laser energy, scan settings, number of pixels/ROI). These are essential for reproducibility.*
Response: As suggested, the laser energy and scan setting have been included in the method section " Imaging was performed at a spatial resolution of 20 µm with beam scan on (16 µm), using 1 burst of 200 shots per pixel, at 70% laser power, and a 10 kHz laser repetition rate." And the pixel numbers are also included, which are related to the section size (scan area). The related information has been added in the revision (page 5, line 5-11).

Reviewer #3:
1. One of the key physiological changes during fasting is a reduction in core body temperature. Therefore, while the observed lipid remodeling in BAT may be driven by nutritional cues, it is also possible that these changes are secondary to fasting-induced hypothermia. To disentangle the effects of nutrient status from those of body temperature, it would be important to reproduce the observed lipid phenotypes under thermoneutral conditions. Demonstrating that these effects persist independent of cold-induced thermogenesis would significantly strengthen the conclusions of the manuscript.
Response: We agree that validating our findings under thermoneutral conditions (typically around 30°C for mice) would provide valuable insight into whether the observed metabolic and lipid remodeling responses are influenced by ambient temperature and thermogenic demand. To determine whether the metabolic adaptations to alternate-day fasting (ADF) are preserved in the absence of cold-induced thermogenesis, we have conducted the studies under thermoneutral conditions in the revision. The results obtained suggest that the dramatic lipid remodeling in BAT during periodic fasting and refeeding is dependent on thermogenic demand. Please see our response to editor's comment 3.

2. The authors show that fasting and refeeding reshape the BAT lipidome by reducing polyunsaturated lipid species and enriching saturated lipids, with significant spatial heterogeneity and dynamic redistribution. The observed shift from polyunsaturated to saturated/monounsaturated lipids in specific BAT regions is intriguing. However, it is unclear whether these changes result from de novo lipogenesis and fatty acid elongation/desaturation within BAT or from the uptake of circulating lipids. The authors should consider including expression data for key enzymes and transporters such as SCD1, Elovl6, and fatty acid transporters (e.g., CD36, FATP) to help clarify the underlying mechanism.
Response: We thank the reviewer for the insightful comments. We agree that the observed shift from polyunsaturated to saturated and monounsaturated lipids in BAT during refeeding raises important questions about the underlying mechanisms, specifically, whether these changes result from intrinsic lipid remodeling

(involving de novo lipogenesis, elongation, and desaturation) or from the uptake of circulating lipids. To address this, we conducted a series of studies, including metabolic phenotyping, analysis of thermogenic gene expression, and protein-level assessment of key enzymes and transporters involved in fatty acid metabolism and uptake, such as CD36, FATP1 (SLC27A1), SCD1, SCD5, Elovl6, and MLXIPL (ChREBPα) (Figs. 7G, S4D-S4E, and S7C). Selectively, refeeding significantly upregulated the expression levels of FATP1 but not other proteins. Our results suggest that fatty acid influx is a major contributor to the synthesis of glycolipids, which are relatively saturated and contribute to the overall increase in BAT lipid saturation. Consistent with this, refeeding after three cycles of alternate day fasting led to activation of thermogenic genes Ucp1 and Pgc1α, enhanced glucose ultilization, and increased energy expenditure. These findings support a model in which refeeding drives lipid remodeling in BAT primarily through the uptake and processing of circulating nutrients such as glucose and fatty acids. Please find more detailed response to Editor's Comment 4.

3. Also, the functional consequences of the observed lipid shift are not addressed. What are the potential implications of increased lipid saturation in BAT thermogenesis or metabolic flexibility?

Response: We thank the reviewer for this excellent point. To further investigate the physiological relevance of lipid shifts in BAT, we conducted additional studies including assessments of thermogenesis, energy expenditure, immunostaining, Western blot analysis, and responses under thermoneutral conditions. These results highlight a unique adaptive mechanism in BAT in response to periodic fasting and refeeding (Figs. 7F-7G, S3I-S3N, S4B-S4K, S5A-S5F, and S7A-S7C). Unlike WAT, which mobilizes its own lipids to meet systemic energy demands during fasting and replenishes them upon refeeding, BAT appears to rely on lipids derived from WAT during fasting and stores excess lipids during refeeding. This may serve as a "metabolic memory" mechanism to compensate for prior energy deficits. Such adaptation likely underlies the observed lipid remodeling during refeeding, characterized by a surge in fatty acid influx and increased glycolipid synthesis. These incoming fatty acids are relatively saturated, contributing to elevated lipid saturation in BAT and the formation of lipid "sparkles" around the vasculature.

During refeeding, glucose, rather than fatty acids, is preferentially utilized for thermogenesis, leading to enhanced thermogenic activity, increased respiratory exchange ratio, and elevated energy expenditure after acute periodic fasting and refeeding. However, this response is associated with mild cold adaptation. Notably, when animals were housed under thermoneutral conditions, lipid remodeling in BAT was markedly attenuated. The related discussion has been included in the revised manuscript (page 12, paragraph 2). Please refer to our detailed responses to Editor's Comments 2 and 3 for further discussion.

4. Could the authors speculate on the enzymatic or hormonal signals driving this selective remodeling upon refeeding? Are insulin signaling or nutrient-sensing pathways (e.g., ChREBP, SREBP1c) potentially involved?

Response: As the reviewer commented, we have performed Western blots to examine the involvement of enzymes and transcriptional factors in lipid metabolism. Please see our response to Editor's comment 4.

5. The authors report that refeeding selectively activates upper glycolysis, glyceroneogenesis, triglyceride synthesis, and glycolytic shunts in BAT—indicating a distinct metabolic reprogramming from white adipose tissue (WAT) that supports both lipid storage and thermogenic readiness. However, it remains unclear why lower glycolysis is not equivalently upregulated. Does this reflect a bottleneck in glycolytic flux, or is it due to a redirection of intermediates toward anabolic processes like triglyceride synthesis? Additional metabolite profiling or enzymatic activity assays may clarify this point.

Response: Our data indeed showed selective upregulation of upper glycolysis, glyceroneogenesis, and glycolytic shunts in BAT during refeeding, while lower glycolysis appears less prominently activated. We agree that this raises important mechanistic questions about glycolytic flux and metabolic routing. One possible explanation is that glycolytic intermediates are being diverted from lower glycolysis toward

anabolic pathways such as glyceroneogenesis, triglyceride synthesis, glycogenesis, and the pentose phosphate pathway, rather than proceeding through to pyruvate and full oxidation. This redirection would support lipid accumulation and biosynthesis, consistent with the observed BAT whitening phenotype. To address this, we have now included mRNA expression data for key enzymes GDP1 and TPI involved in glyceroneogenesis, as well as upper and lower glycolysis, including HK, GAPDH, PGK1, and PKM. These data suggest a metabolic rerouting rather than a uniform upregulation of glycolysis. We agree that additional metabolite profiling and enzymatic activity assays would provide more definitive insight into pathway flux and are planning such experiments in future work, considering the focus on lipid remodeling in the present study.

6. The authors demonstrate that mTORC1 activation in BAT is essential for refeeding-induced metabolic reprogramming, including increased triglyceride synthesis and a shift to less unsaturated lipids. In this regard, 1) what were the rationales that make the author assume the involvement of mTORC1 in this shift? 2) also, the mechanistic role of mTORC1 in regulating lipid saturation remains insufficiently explored. 3) How does mTORC1 control key enzymatic steps in lipid remodeling, such as elongation and desaturation? Expression or activity data for downstream effectors (e.g., Elovl family members, SCD1) would help clarify the pathway.

Response: We thank the reviewer for these thoughtful and important questions regarding the role of mTORC1 in lipid remodeling during refeeding. Our rationale for examining mTORC1 stemmed from its well-established role as a nutrient-sensing kinase that integrates signals from feeding, insulin, and amino acids to regulate anabolic processes, including lipid biosynthesis. Given the activation of mTORC1 observed in BAT upon refeeding and its role in intracellular fatty acid transport and lipid droplet restoring in fat tissues (Fig. 7), we hypothesized that mTORC1 might contribute to the observed lipid remodeling in BAT through enhancing fatty acid influx, transport, and esterification upon anabolic stimulation. To further explore the mechanistic link between mTORC1 signaling and lipid saturation remains incompletely explored. Our current data show that mTORC1 inactivation in BAT blunts the refeeding-induced increase in saturated and monounsaturated lipids, suggesting a regulatory role. In the revision, we further investigated the precise downstream effectors mediating this effect by examining the protein expression for key enzymes involved in lipid metabolism, including CD36, FATP1, SCD1, SCD5, and Elovl6. Notably, FATP1 was upregulated by refeeding despite no significant changes on other proteins (Figs. S4D and S4E). Furthermore, mTORC1 partially mediates refeeding-induced FATP1 expression and lipid remodeling (Figs. 7E-7G and S7C). We also examined ChREBPα (MLXIPL) expressions and found that mTORC1 is critical for activation of *Chrebpα* gene, although its expression was not significantly upregulated by refeeding (Figs. 7G and S7C). These results suggest that mTORC1 is a key contributor of lipid remodeling during post-fast refeeding.

7. Also, the potential viral leakage into the liver complicates the interpretation of BAT-specific mTORC1 effects. Ex vivo or in vitro studies using isolated brown adipocytes or BAT explants could provide more definitive insight into tissue-autonomous mechanisms.

Response: Please see our response to reviewer 1's comment 1.

8. Could the refeeding-induced lipid and fatty acid changes in BAT originate from other tissues, such as the liver? Specifically, are the unsaturated fatty acids enriched during refeeding synthesized de novo in the liver or mobilized from white adipose tissue (WAT)? How can the authors distinguish whether the observed phenotypes are intrinsic to BAT or secondarily influenced by systemic lipid flux from other organs? Demonstrating tissue-autonomous effects will be essential to strengthen the conclusions.

Response: Please see our response to reviewer 2's comment 3.

9. Have the authors considered using stable isotope tracing (e.g., 13C- or 2H-labeled fatty acids or

glycerol) to directly track the source of lipids incorporated into BAT during refeeding? Such experiments could help distinguish contributions from liver-derived triglyceride-rich lipoproteins versus WAT-released free fatty acids, providing mechanistic insight into inter-organ metabolic crosstalk. Perhaps this is not necessary for the current study, it would strengthen the manuscript.

Response: We agree that further validation at the protein level and functional assays such as isotope tracing or transporter inhibition would provide deeper mechanistic insight, and we are actively pursuing these directions in ongoing studies.

10. Given the spatial heterogeneity observed in lipid remodeling, is there evidence that specific cell populations within BAT (e.g., mature brown adipocytes, progenitors, or immune cells) differentially contribute to these metabolic adaptations? If needed, the use of single-cell or spatial transcriptomics could clarify cell-type-specific metabolic responses and regulatory networks during fasting-refeeding cycles.

Response: Please see our response to reviewer 2‘s comment 3.

11. How robust and reproducible are the observed spatial lipid changes in BAT? Could technical factors such as tissue sampling orientation, sectioning depth, or inherent depot heterogeneity account for the spatial differences? Additional details on how spatial resolution was standardized and validated would help assess the reliability of the imaging-based lipidomic analysis.

Response: We thank the reviewer for raising this important point regarding the robustness and spatial reliability of our MALDI-MSI data. We took several steps to ensure that the observed spatial lipid patterns in BAT were biologically meaningful and not artifacts of technical variability. First, half of total BAT tissue (one side of butterfly) was used for MALDI analysis, and the center region of BAT tissue was screened using MALDI, as mentioned in our response to Reviewer 1's comment 4. Therefore, sections were collected from comparable anatomical regions across biological replicates to reduce sampling bias. Second, to minimize variability due to tissue orientation and sectioning depth, all BAT samples were embedded and sectioned using a standardized protocol, with consistent anatomical orientation and thickness (10 µm). Third, to address spatial reproducibility, we analyzed multiple biological replicates (n = 3-5 per group), and the spatial enrichment of saturated lipids in specific zones was consistently observed across samples. These patterns were not random or sporadic but showed reproducible localization, suggesting biological relevance rather than technical noise. To validate these spatial features against known BAT microanatomy, we performed co-registration with histological markers of vascular endothelial cells and adipocytes and showed that refeeding-stimulated lipid sparkles are correlated with vasculature zone indicated by CD31 staining and H&E staining (Figs. S3I-S3N), indicating that these lipid-enriched zones correspond to specific functional or structural domains.

In summary, we want to thank the editors and reviewers for all the constructive comments and further consideration. We believe the revised manuscript has been improved by addressing those concerns experimentally.

Sincerely,

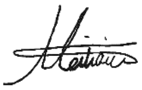

Meilian Liu, Ph.D.
Professor of Biochemistry and Molecular Biology
University of New Mexico Health Sciences Center
Albuquerque, NM 87131
